# Supplementary figures and images for: Genomewide association study of ionomic traits on diverse soybean populations from germplasm collections
Source: Plant Direct. 2018 Jan 15;2(1):e00033. doi: 10.1002/pld3.33 (PMC6508489; doi:10.1002/pld3.33)

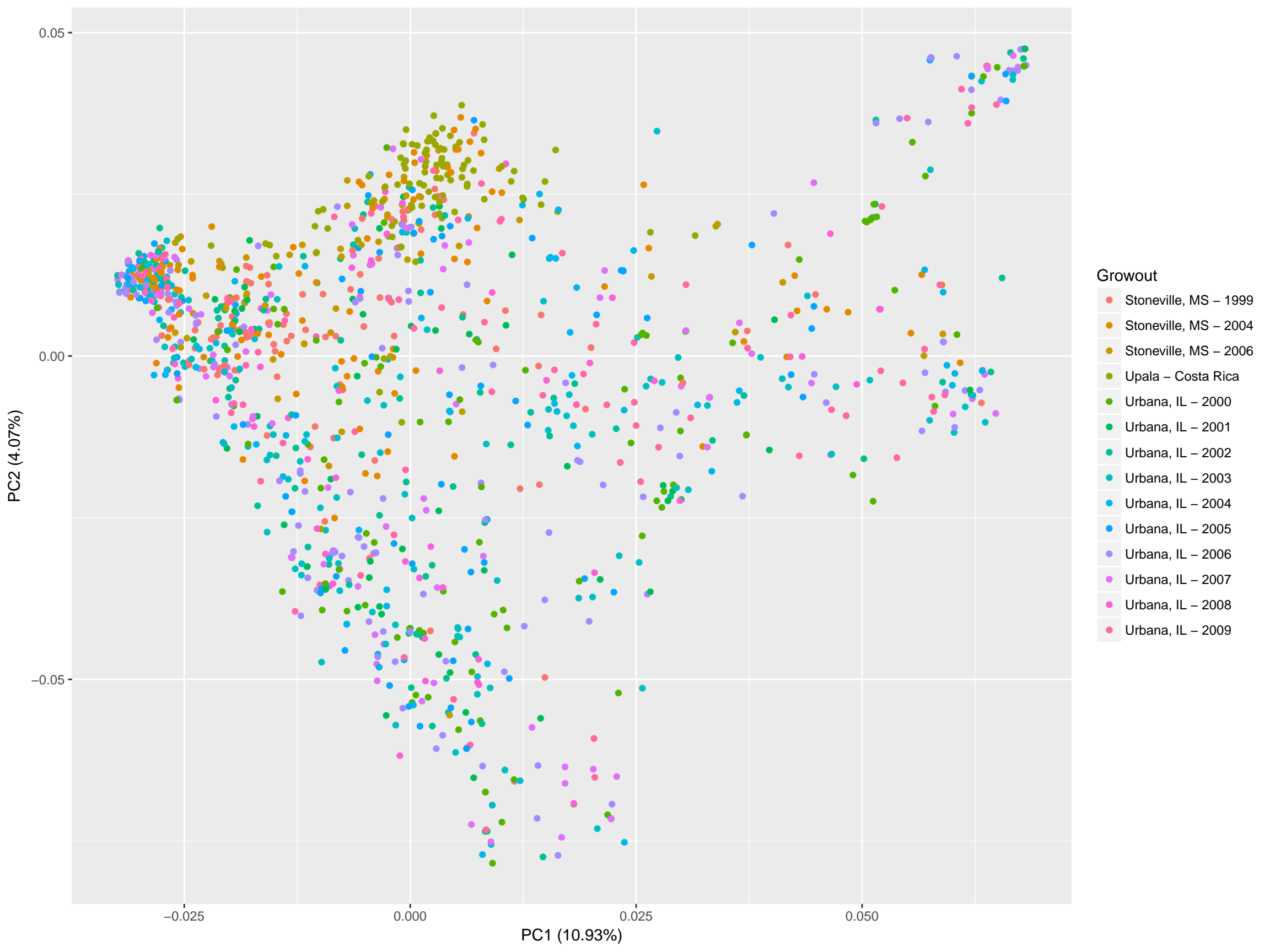

Supplement: Supplementary file 1 [file PLD3-2-e00033-s001.pdf]

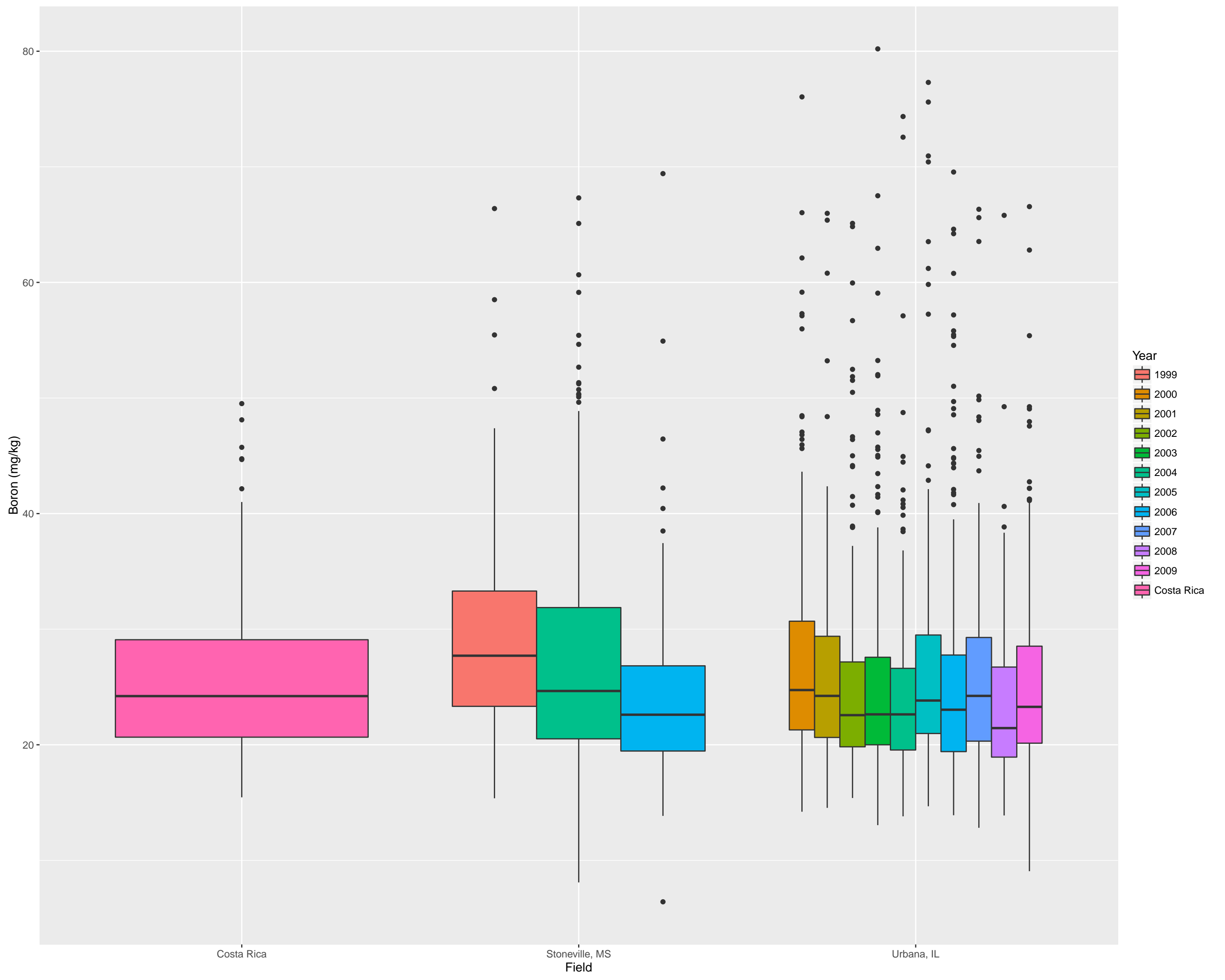

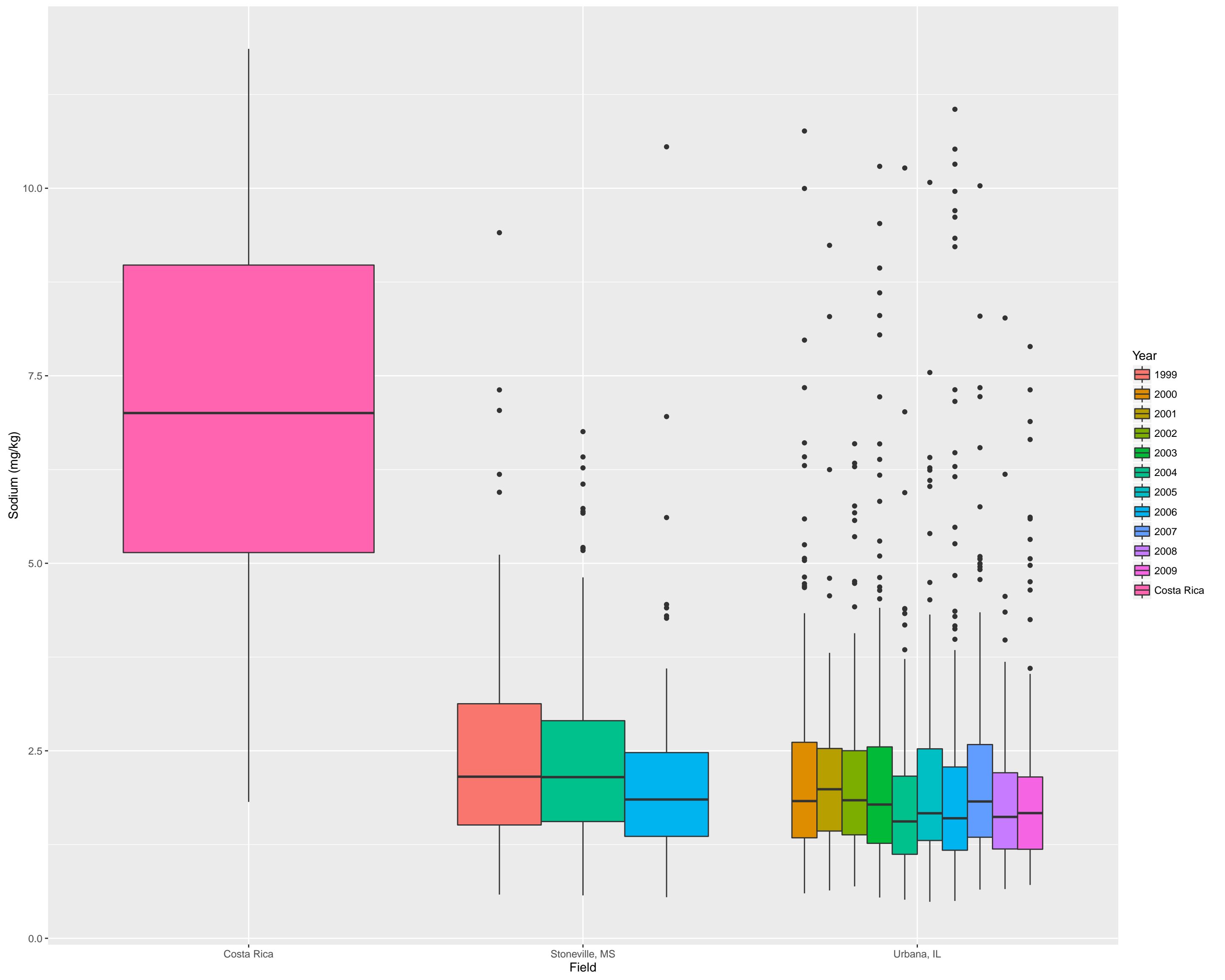

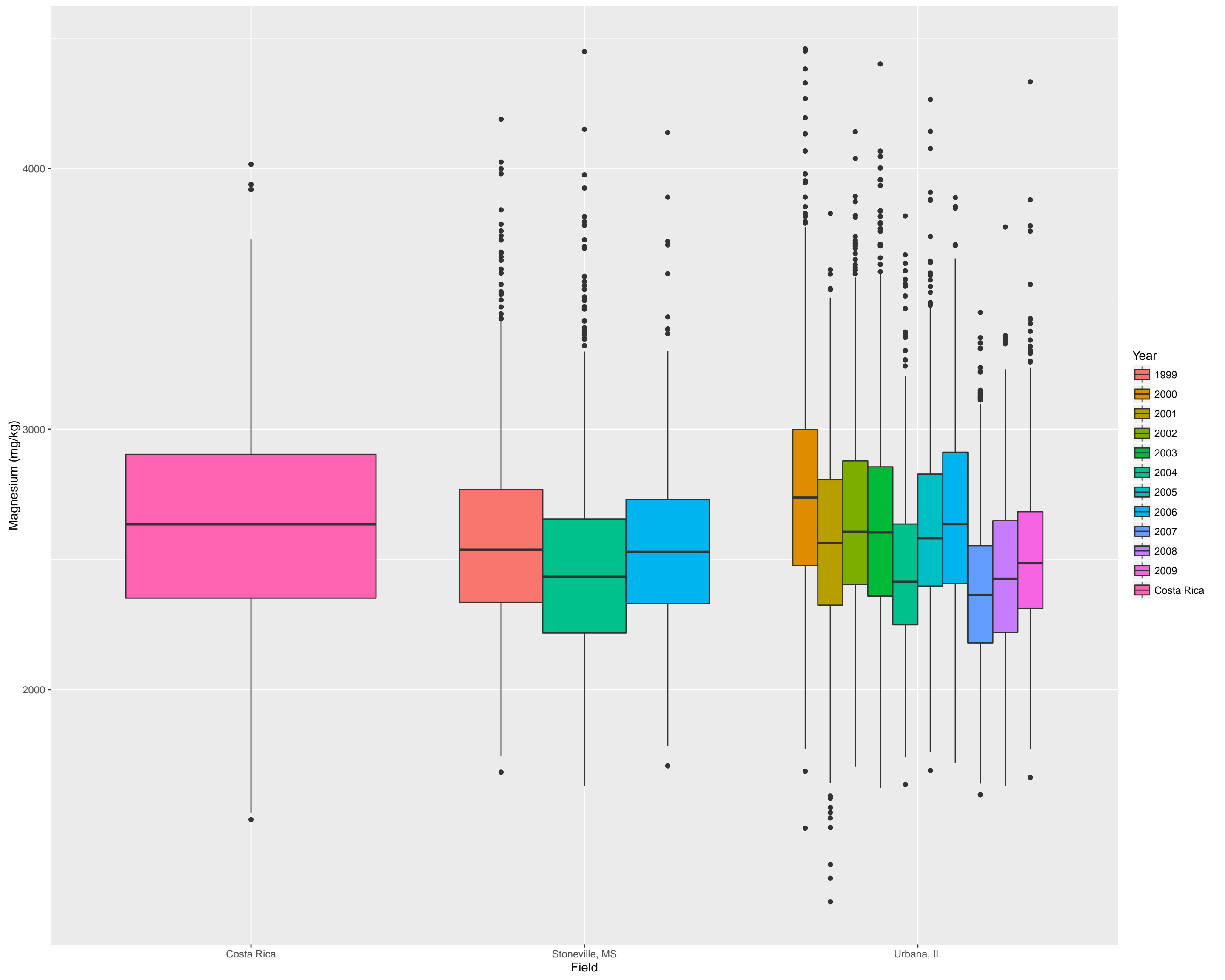

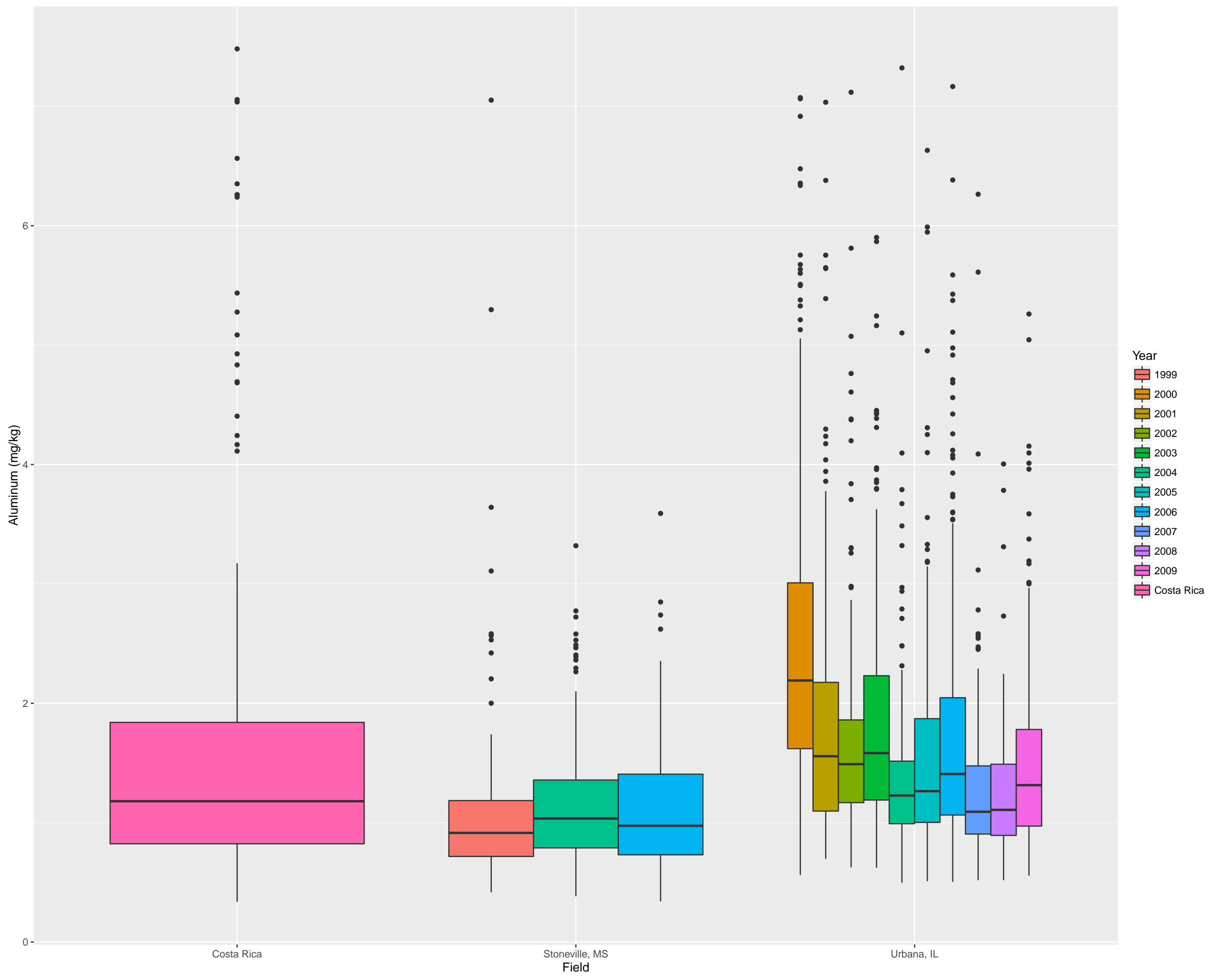

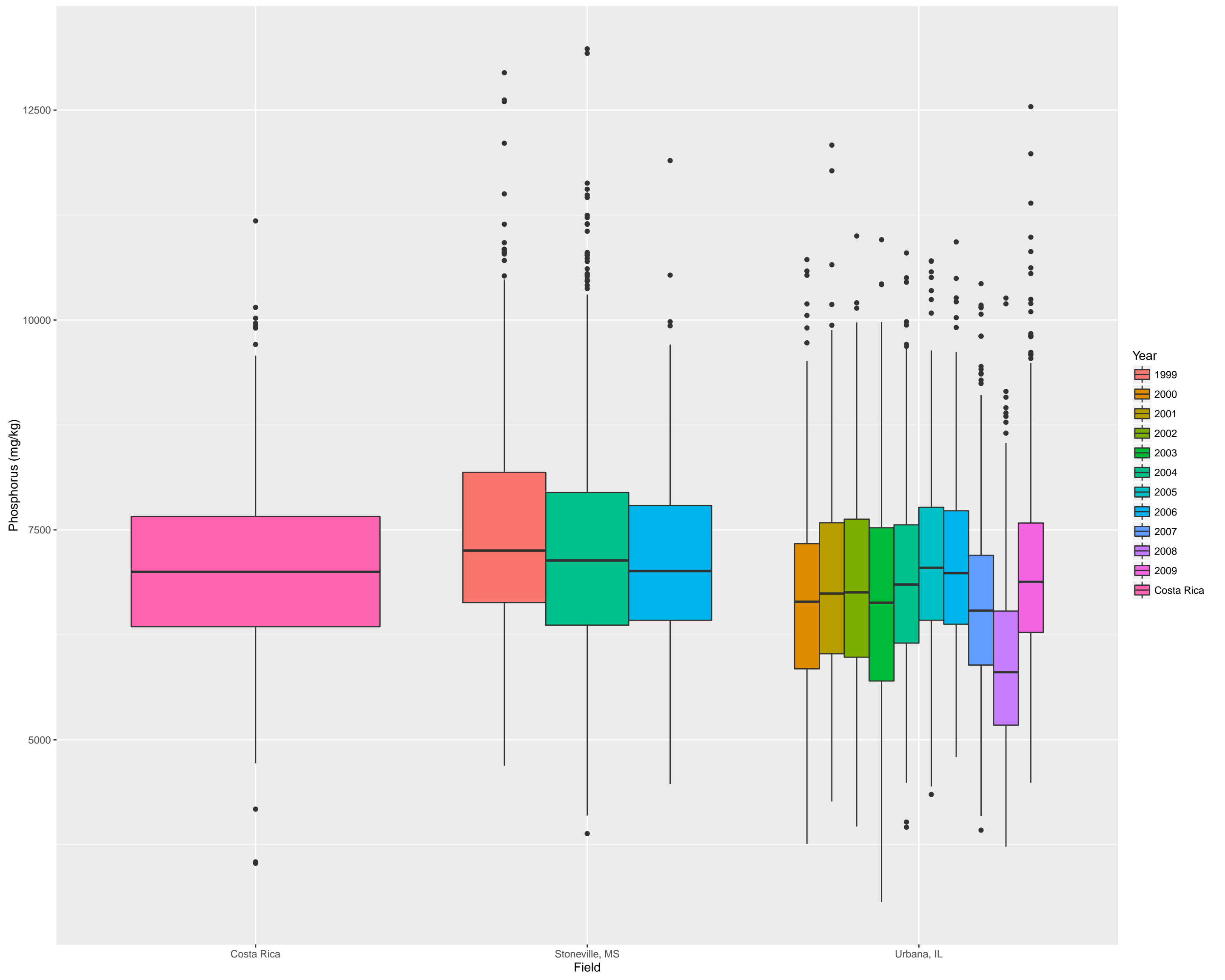

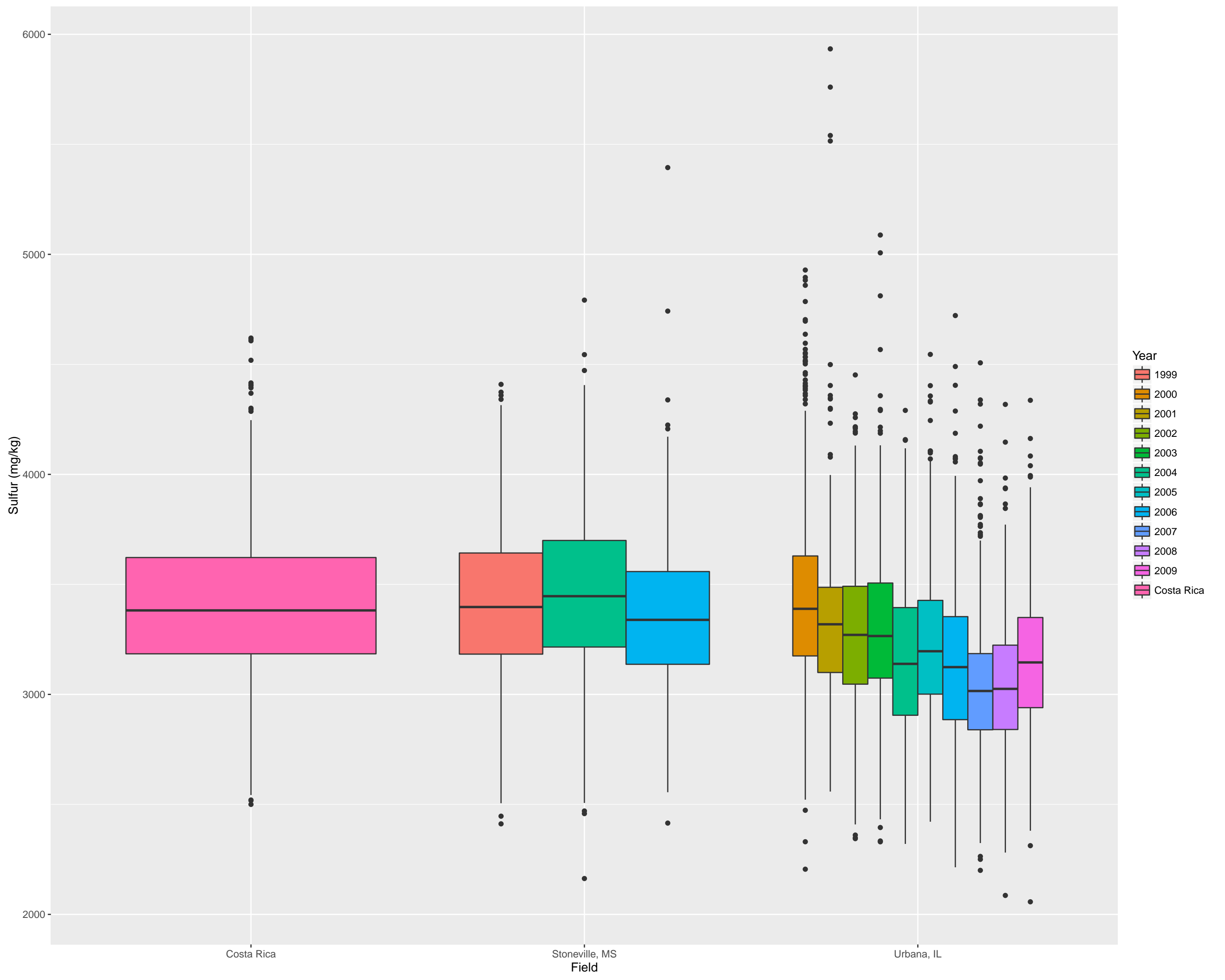

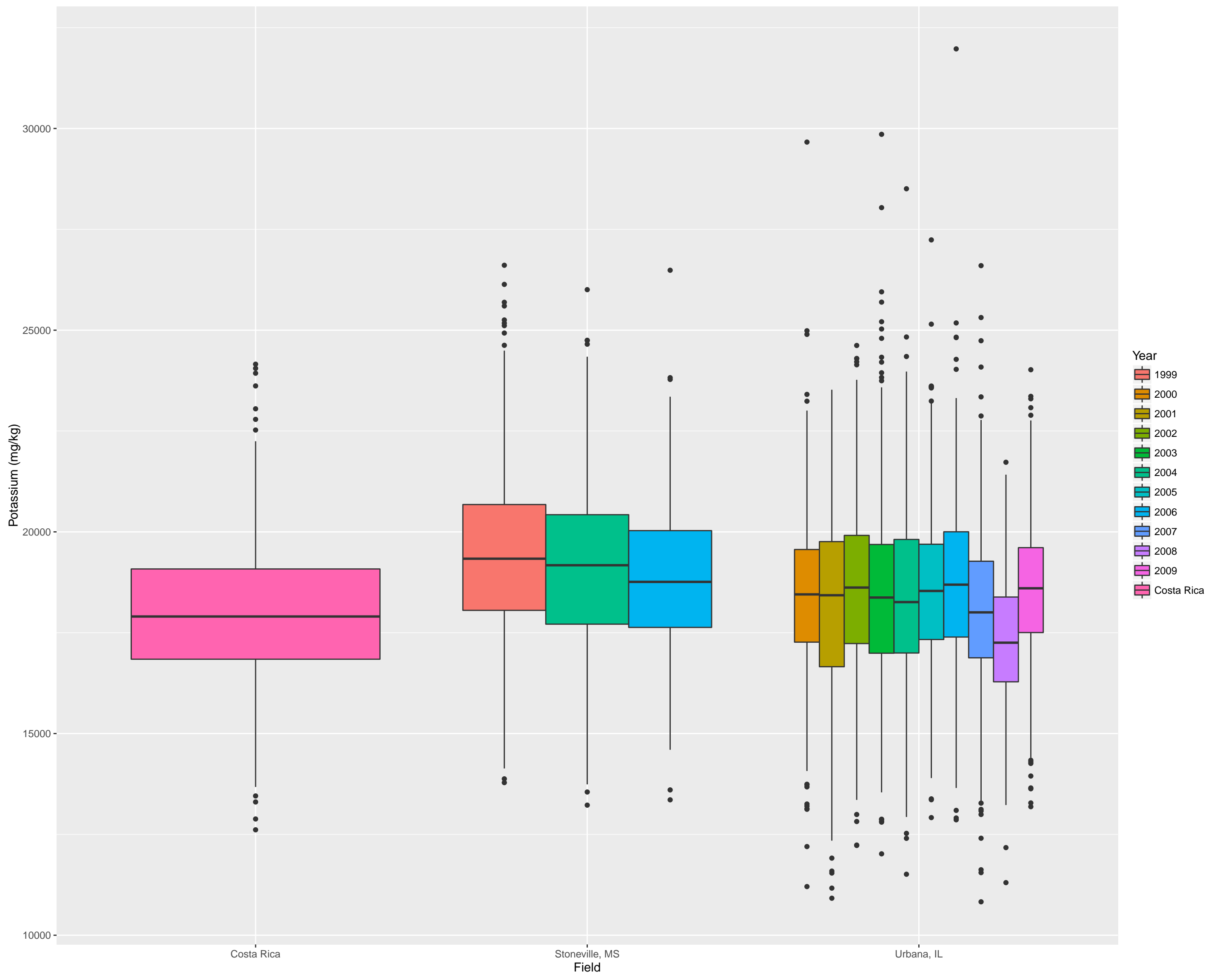

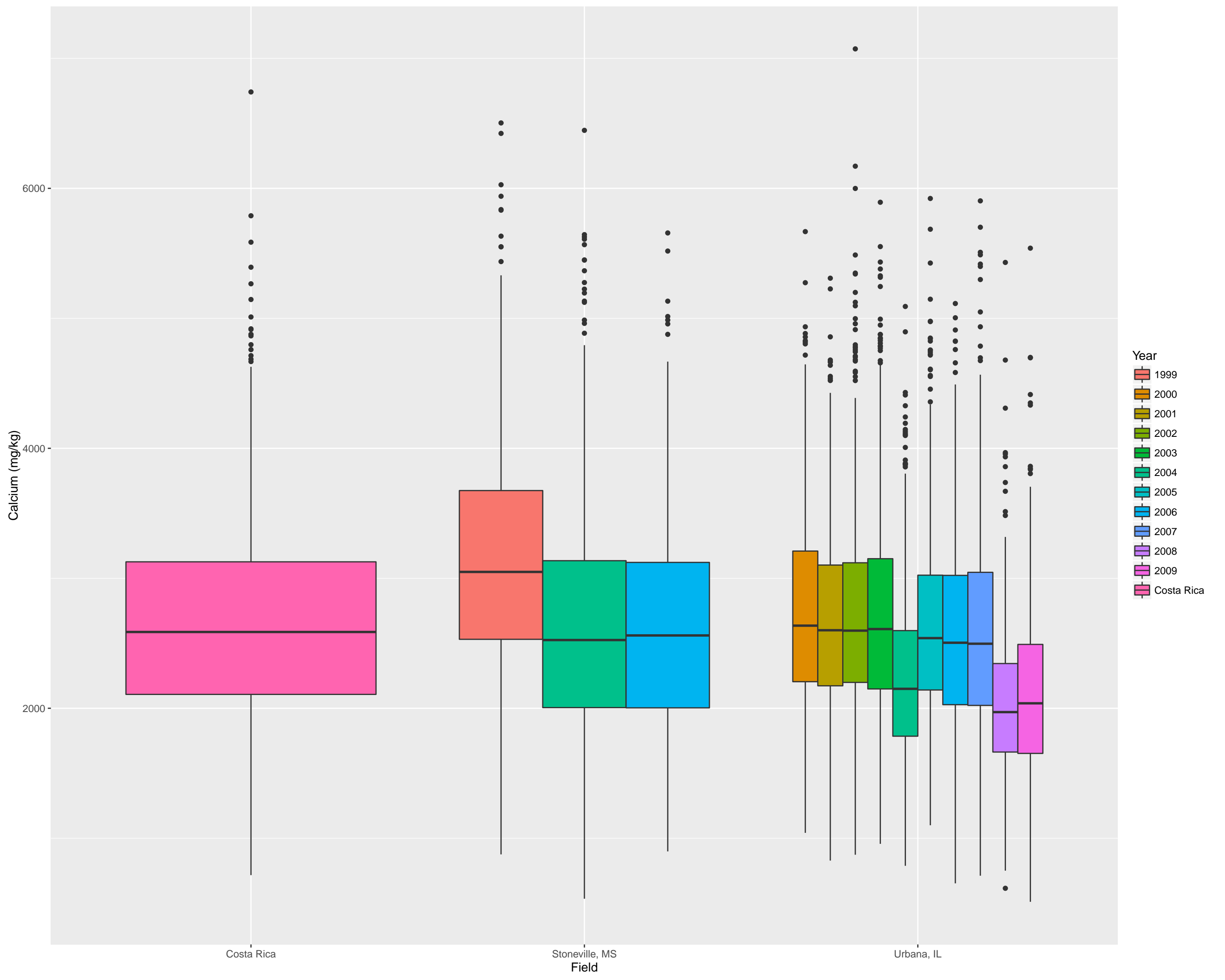

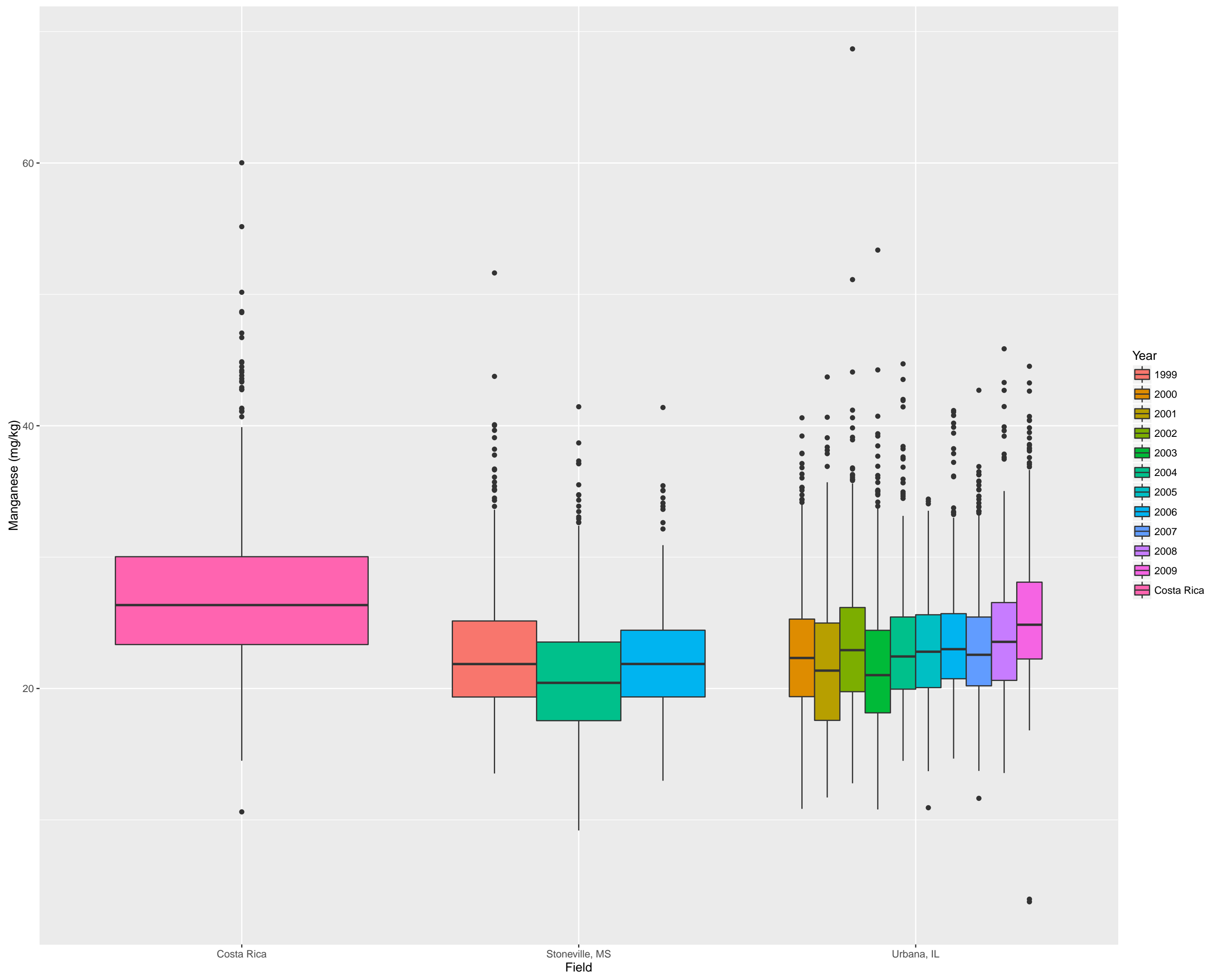

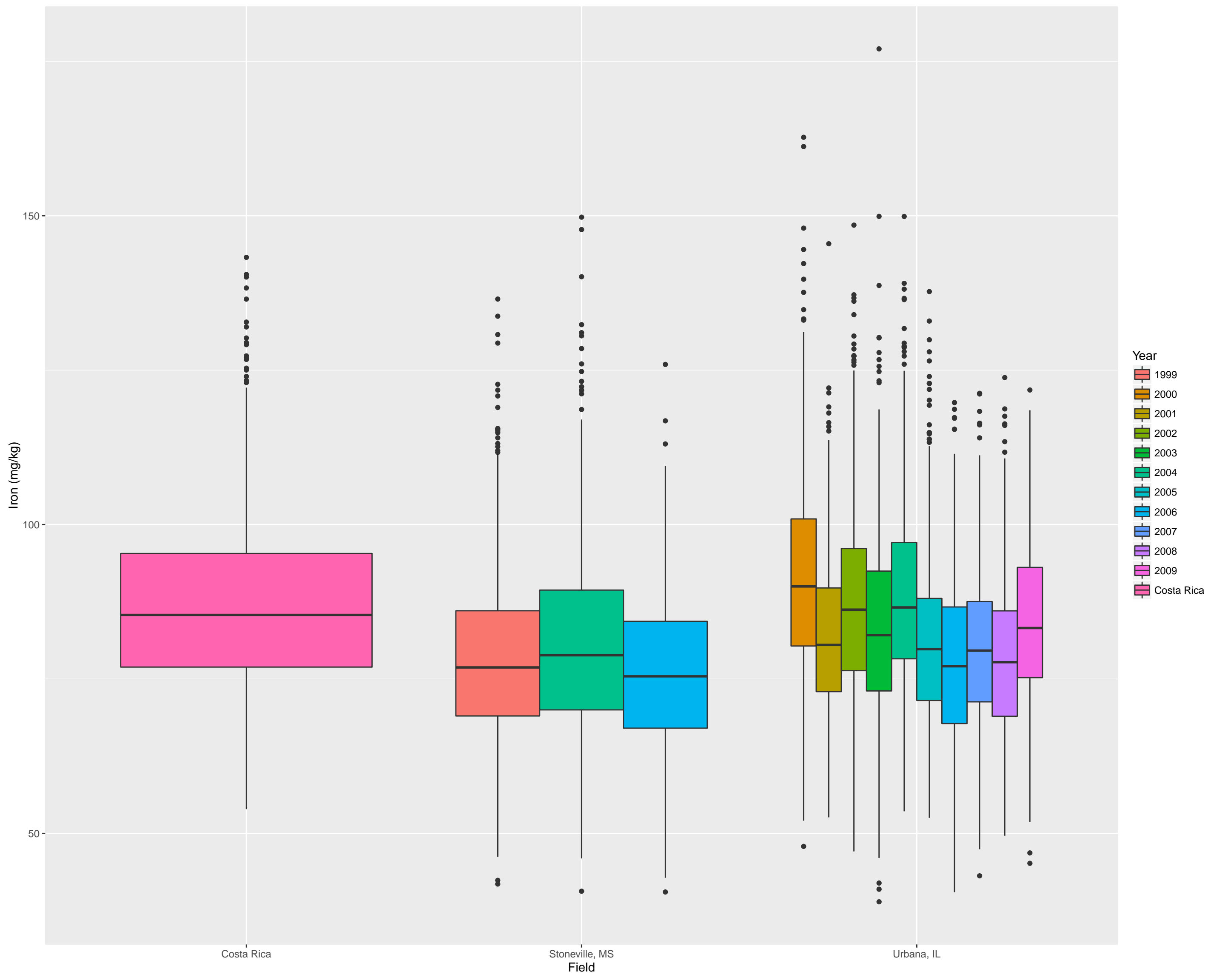

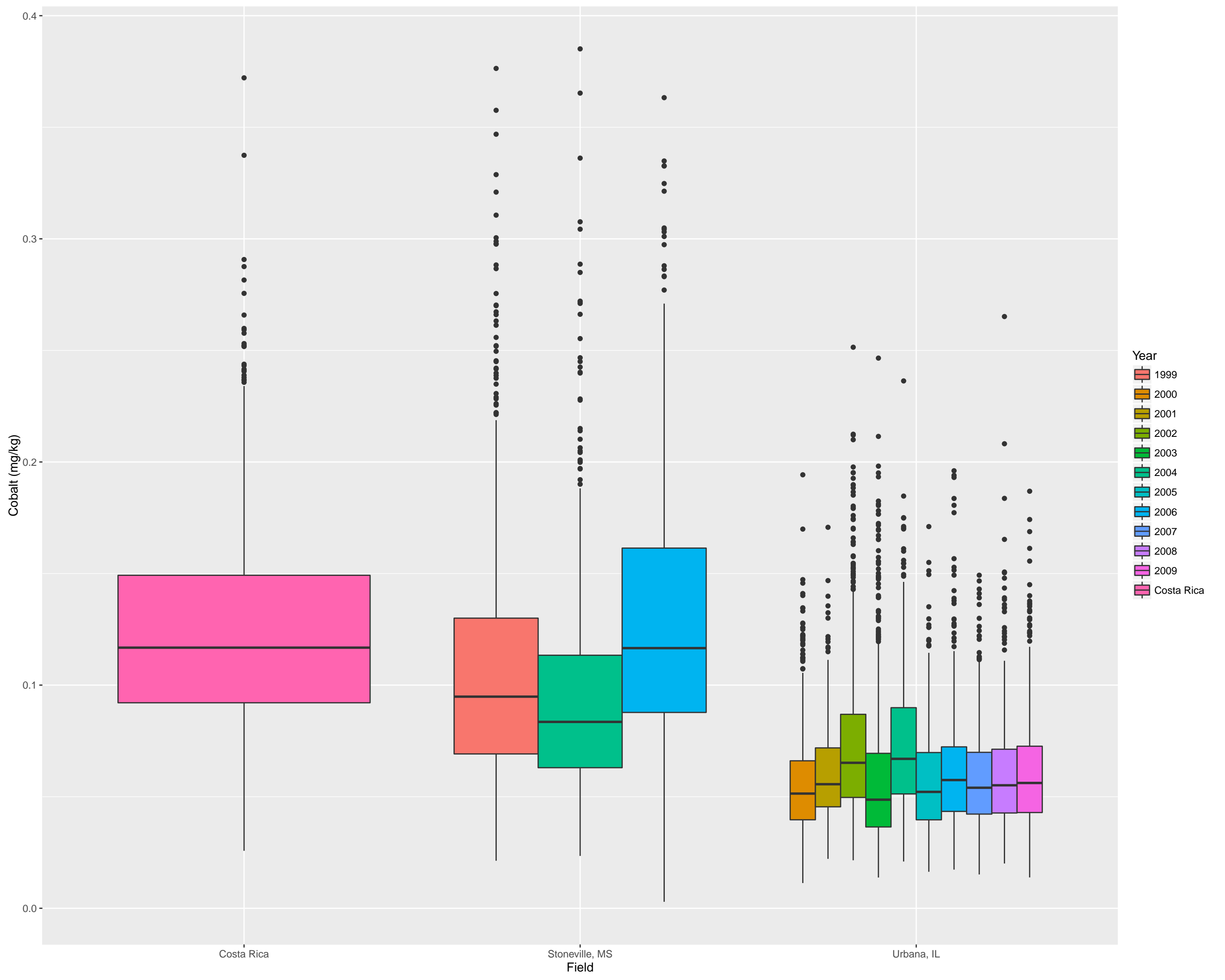

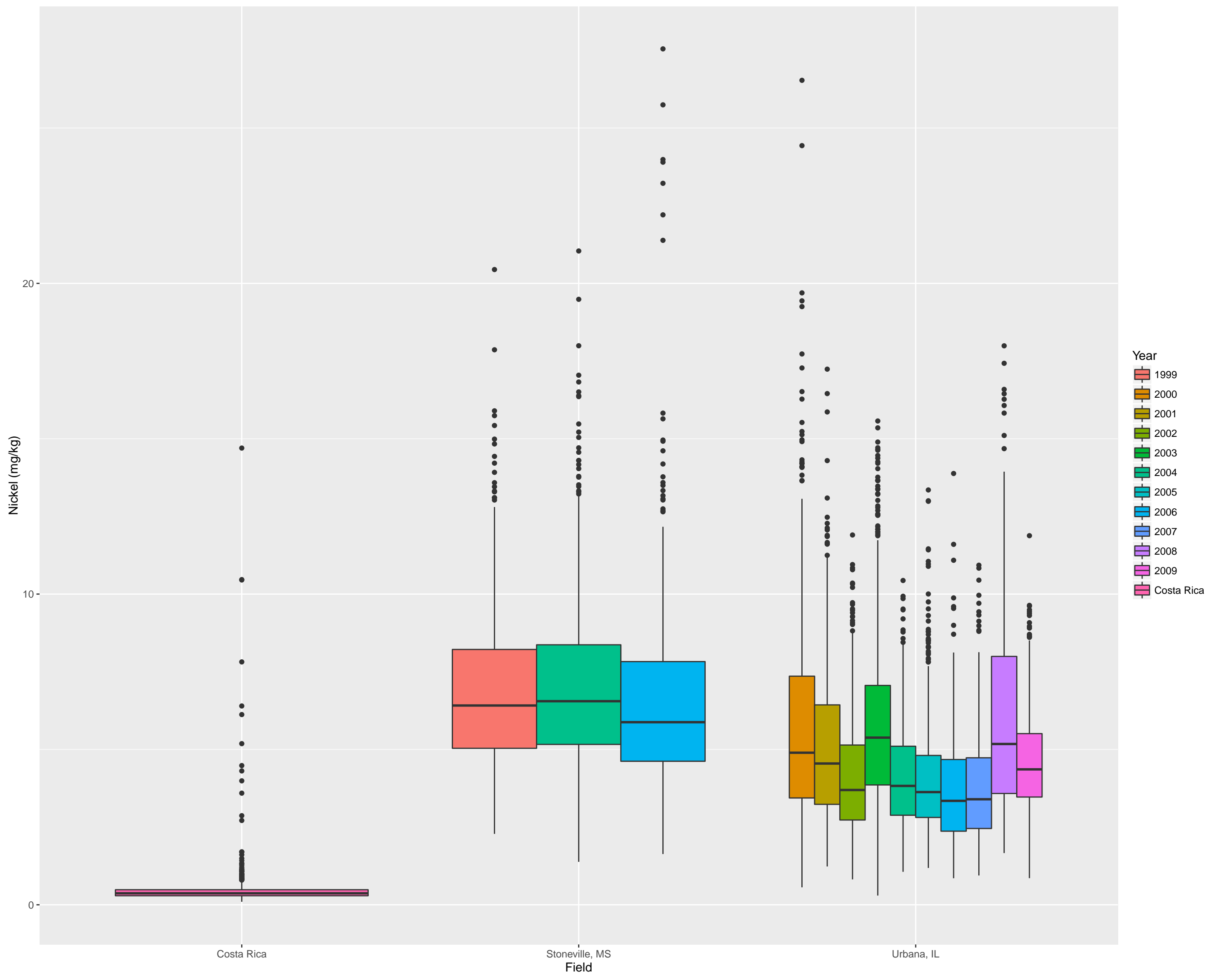

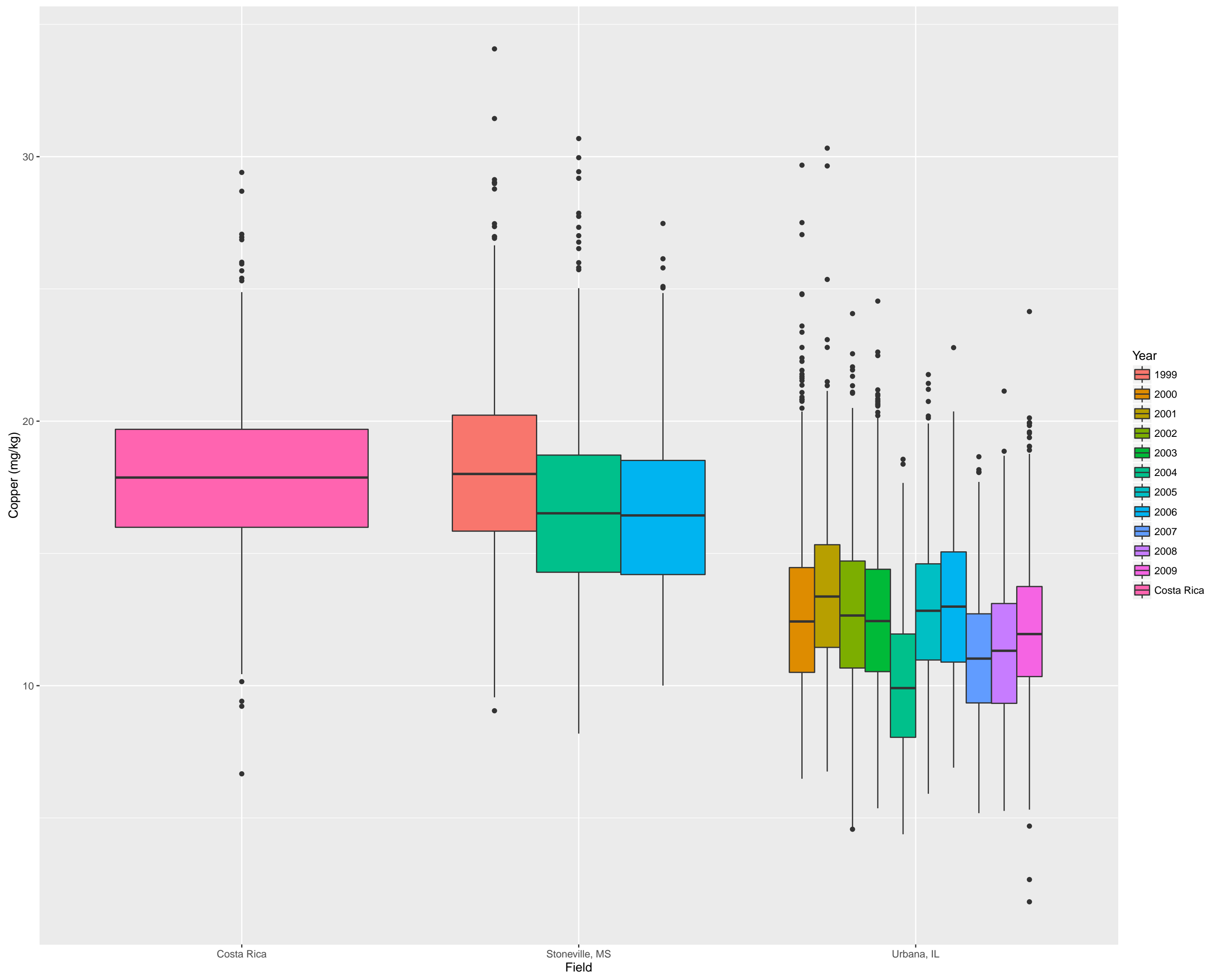

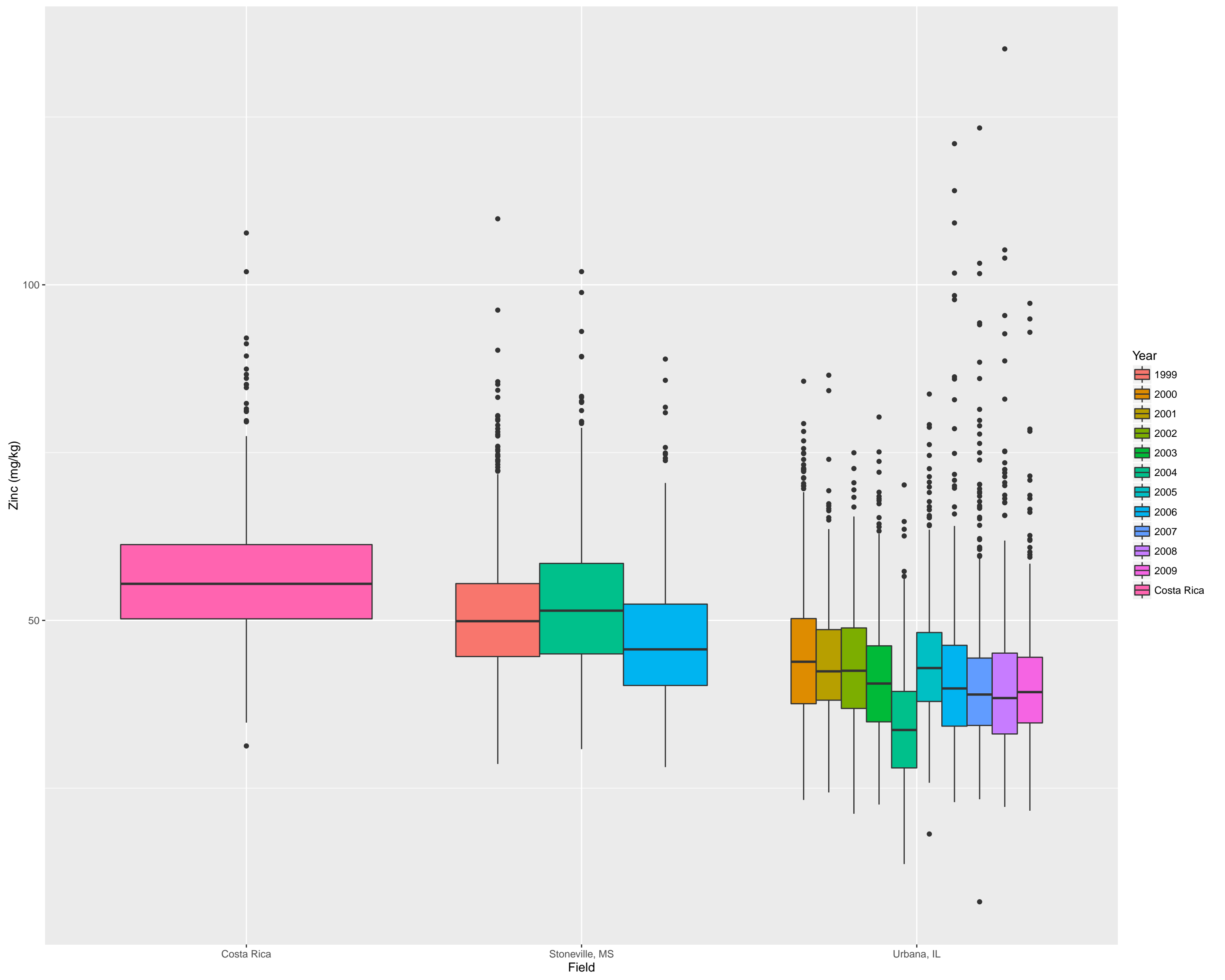

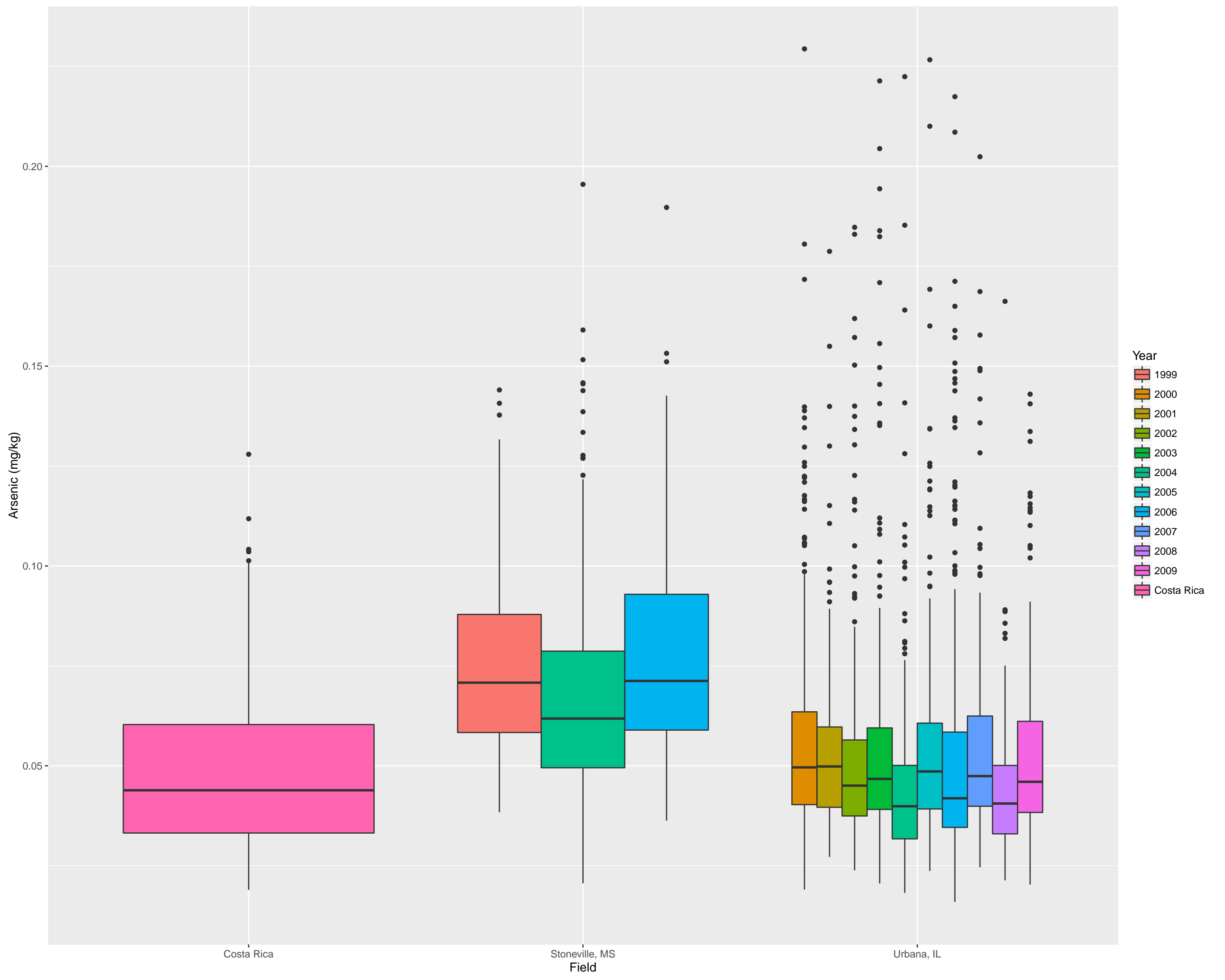

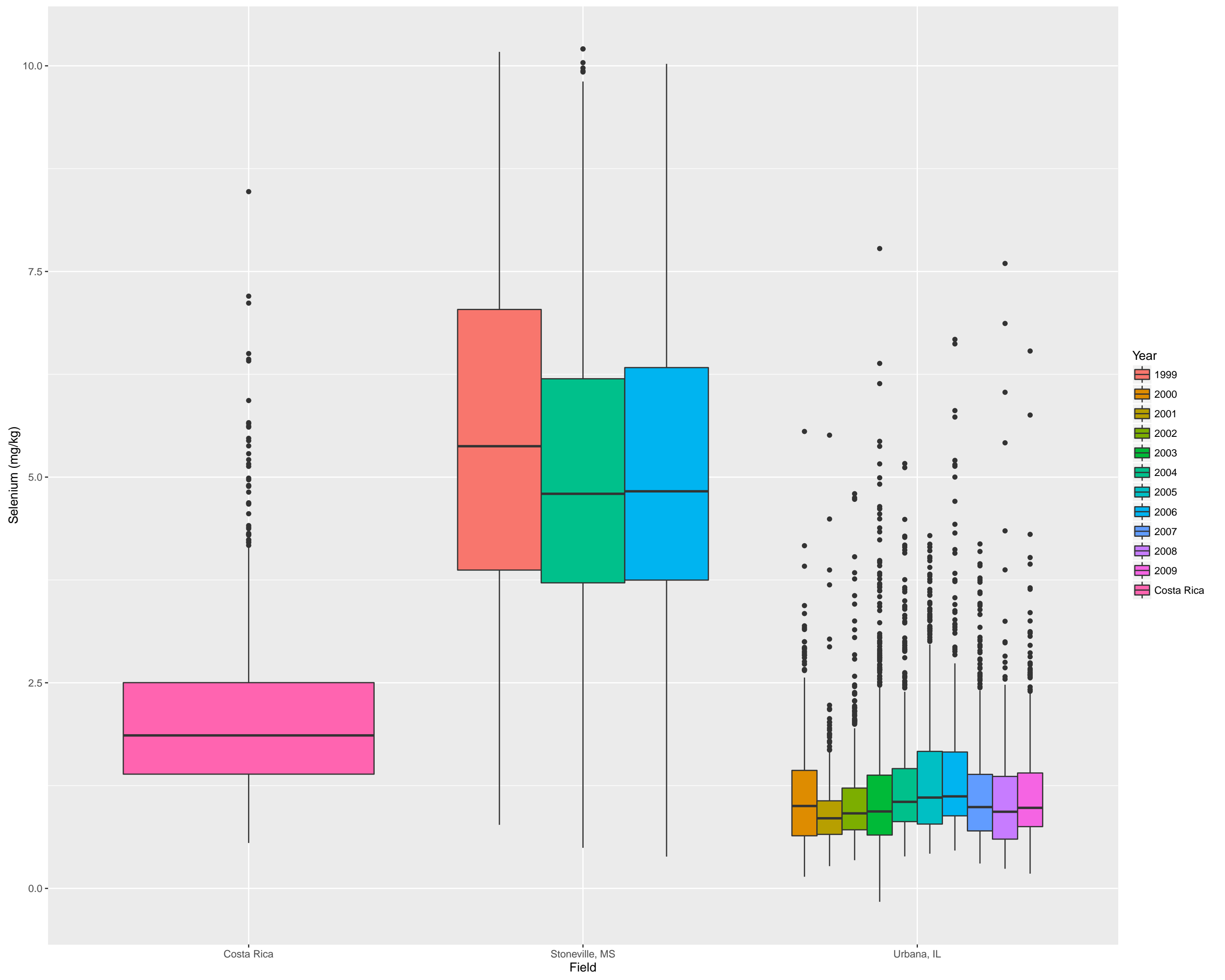

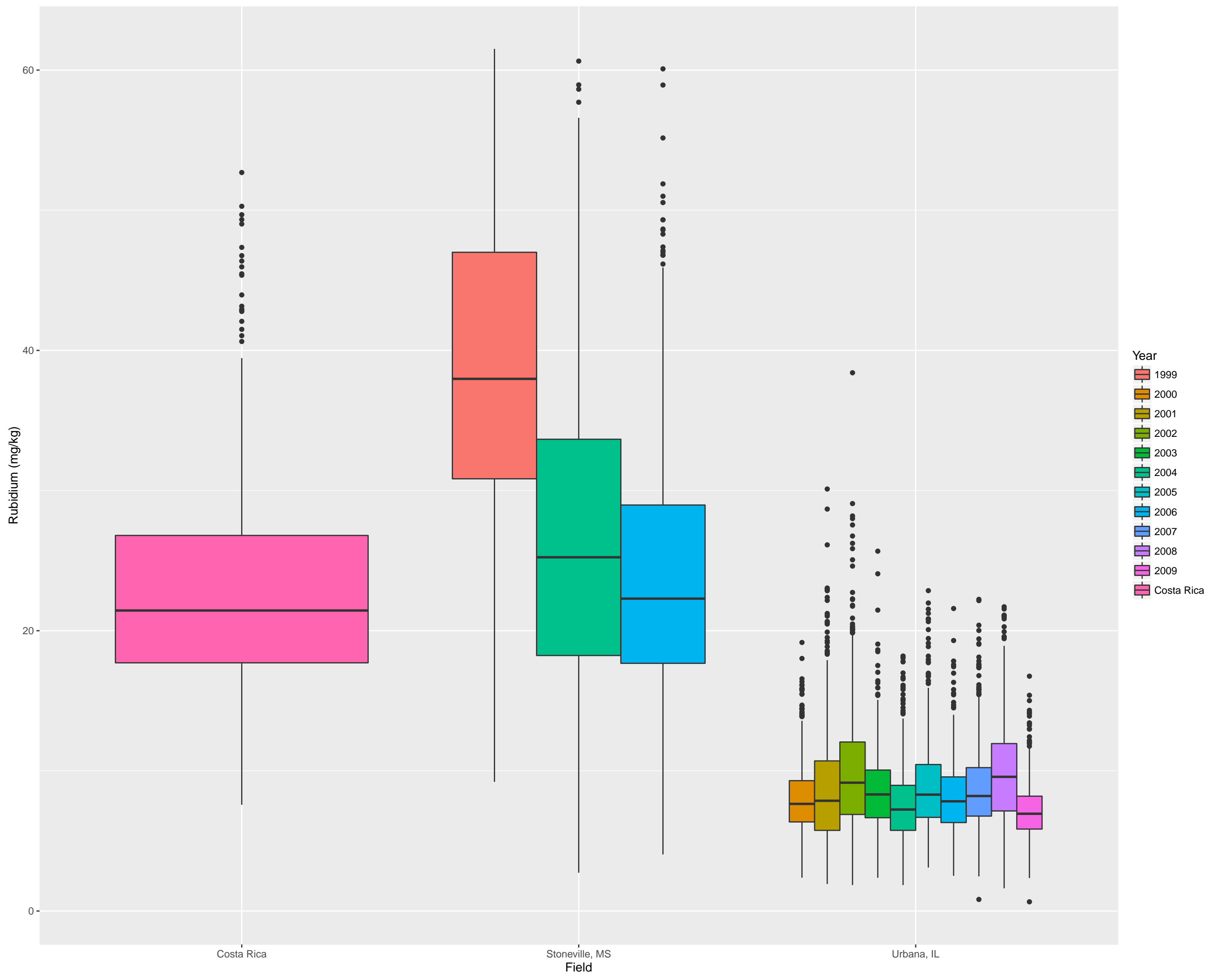

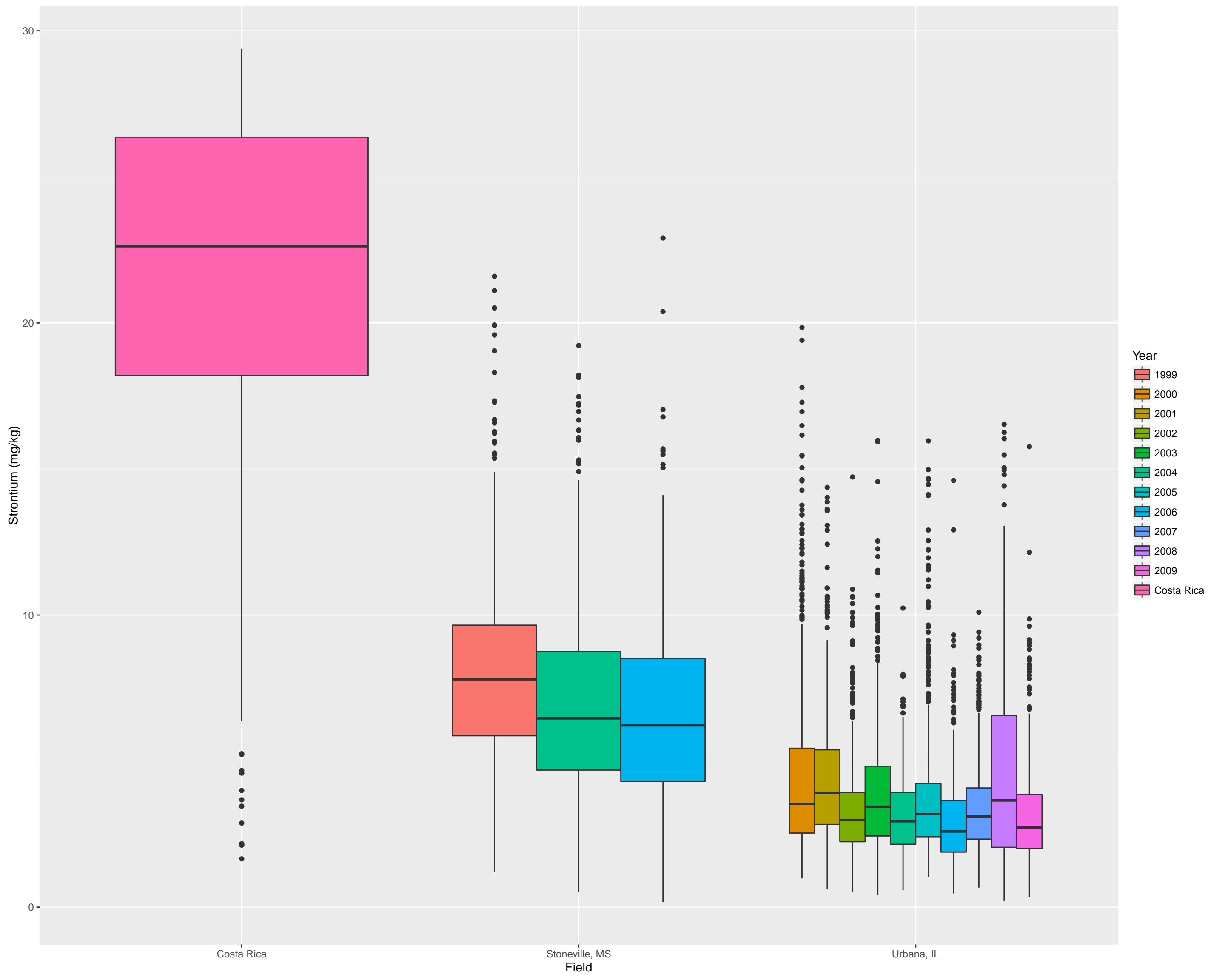

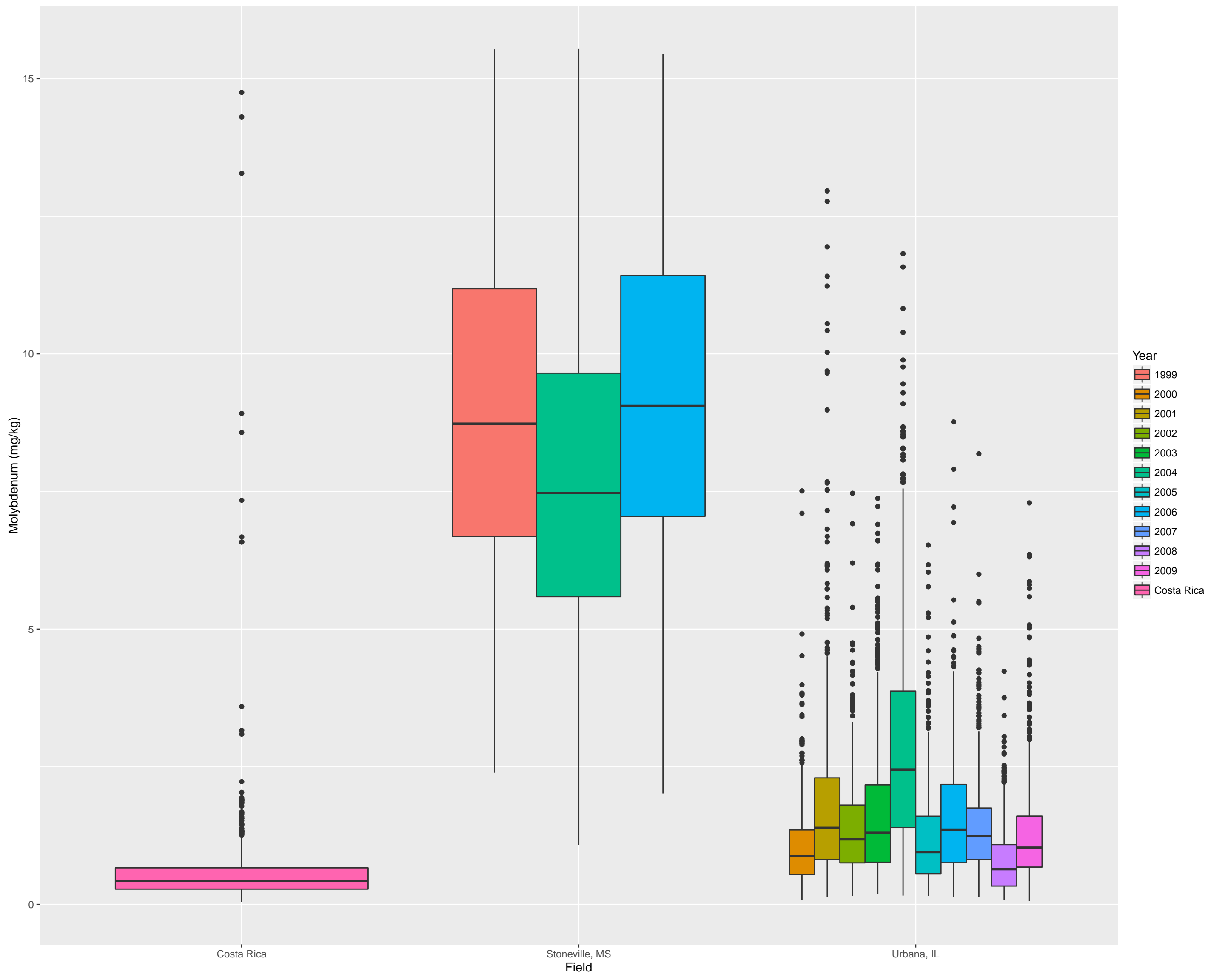

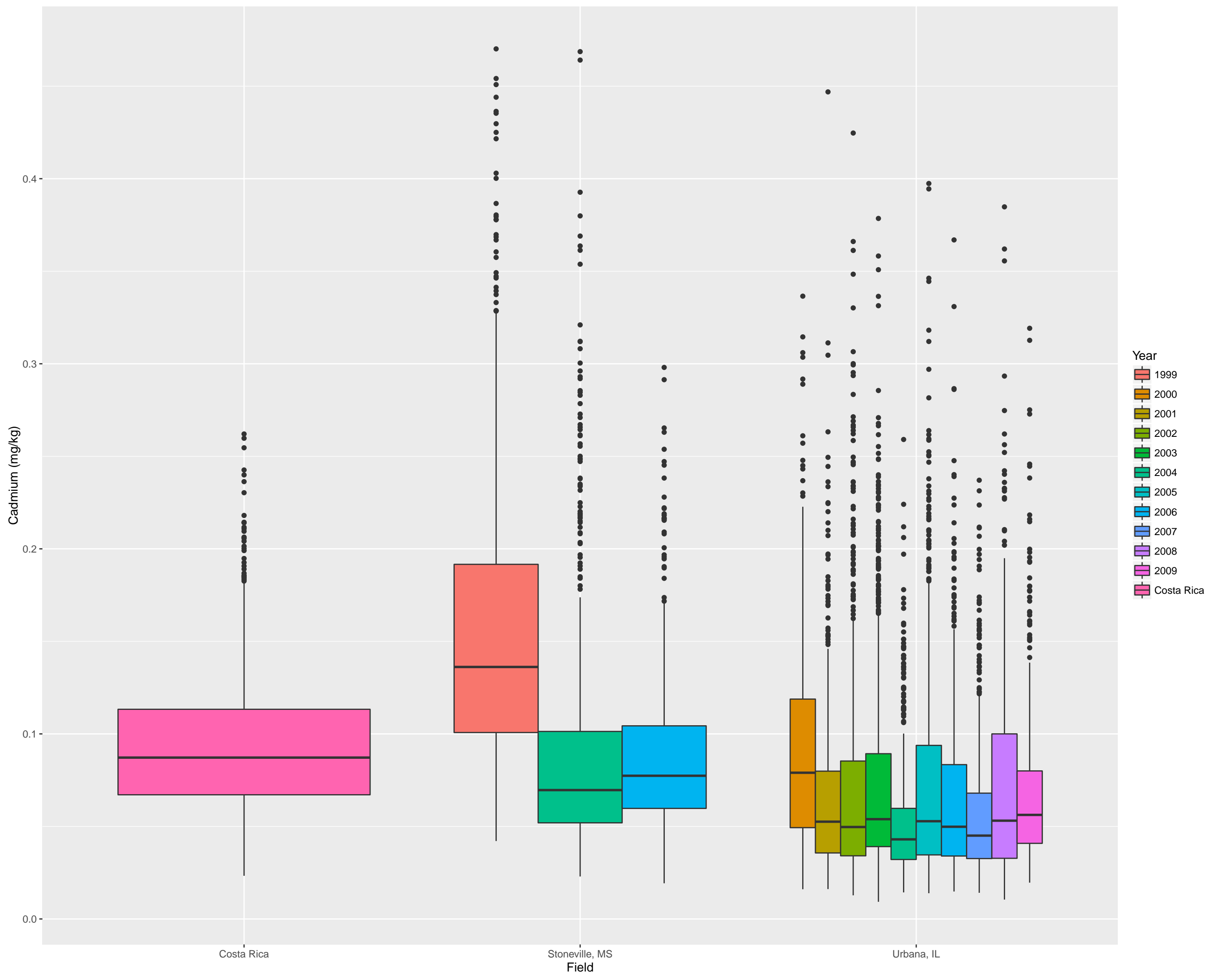

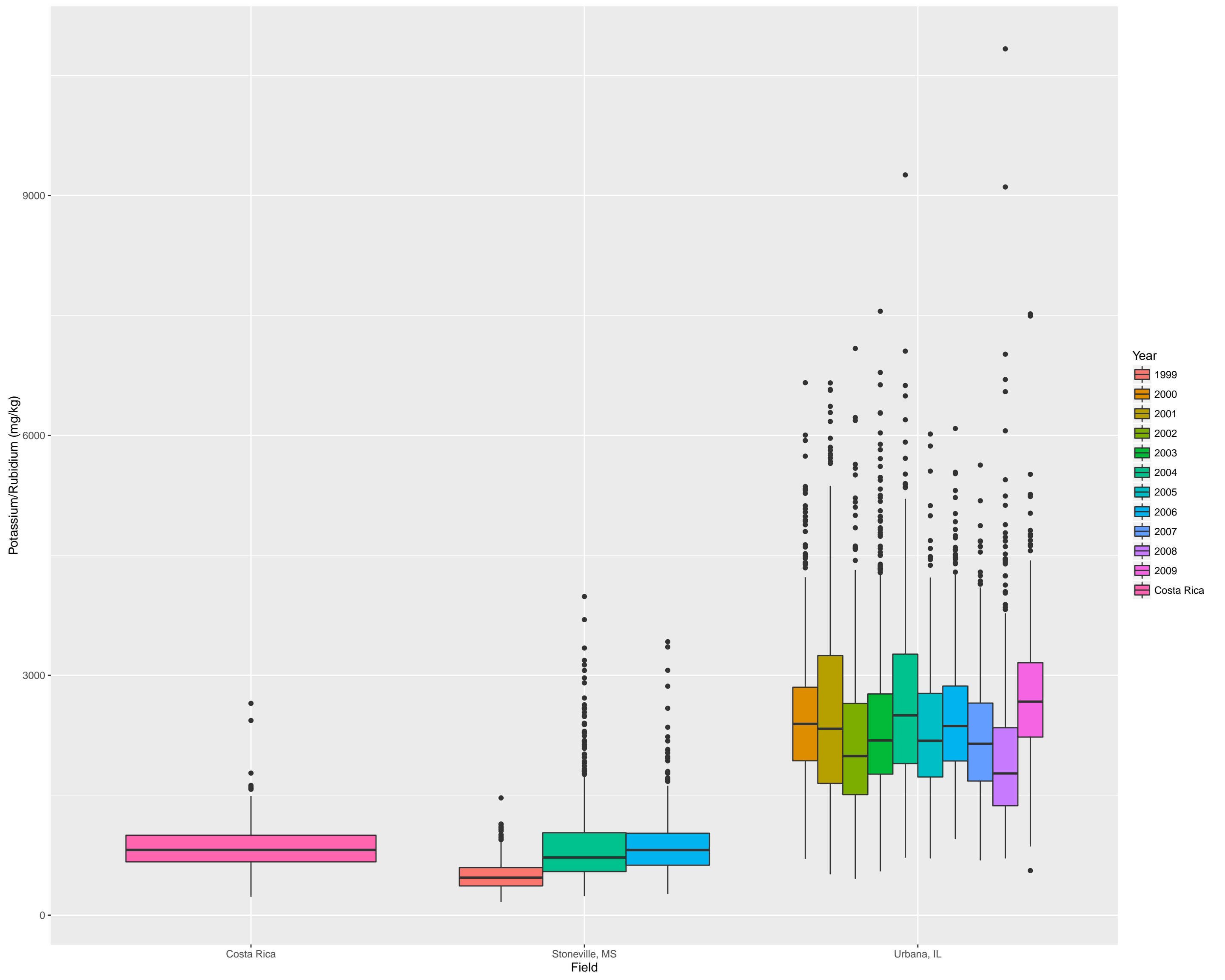

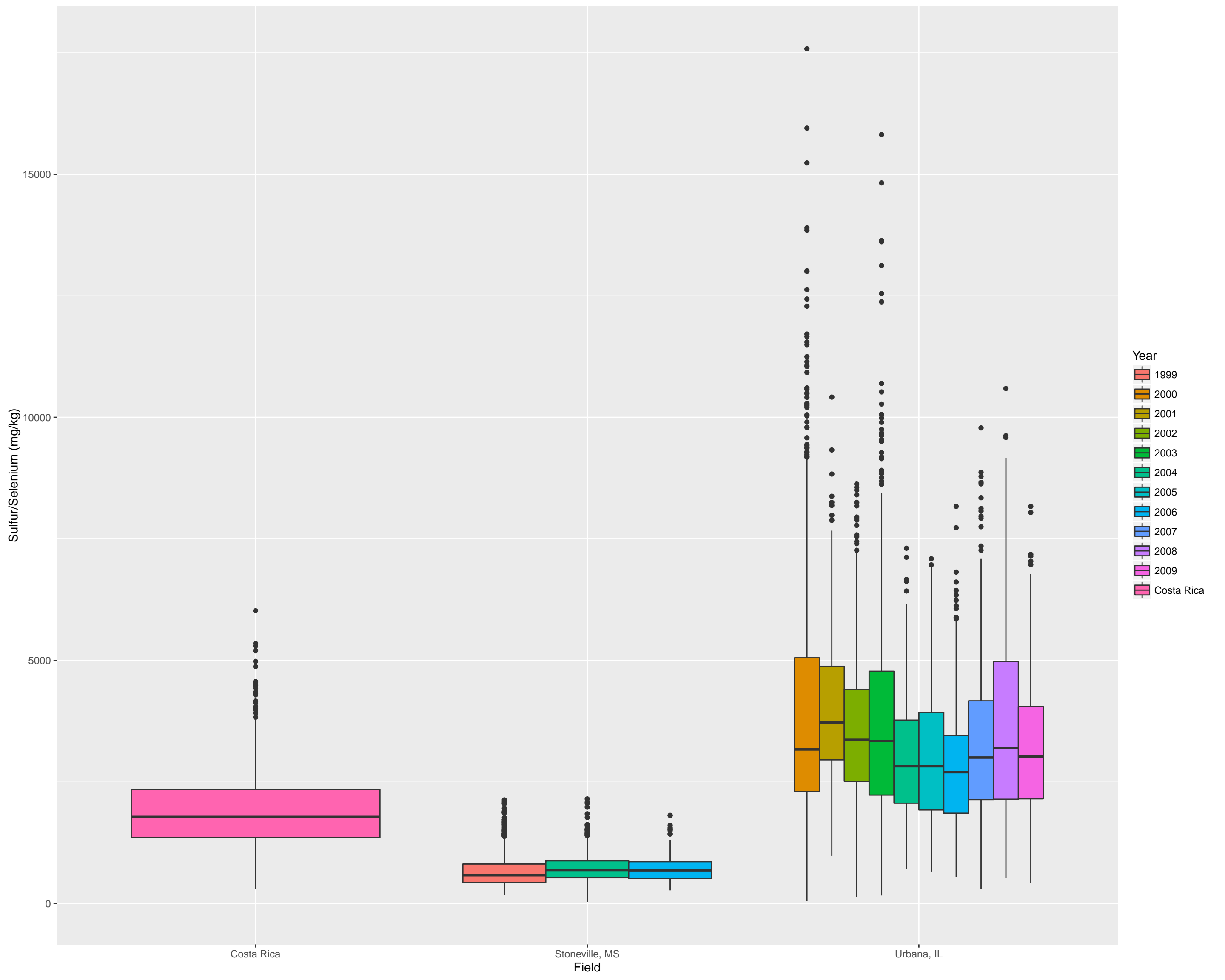

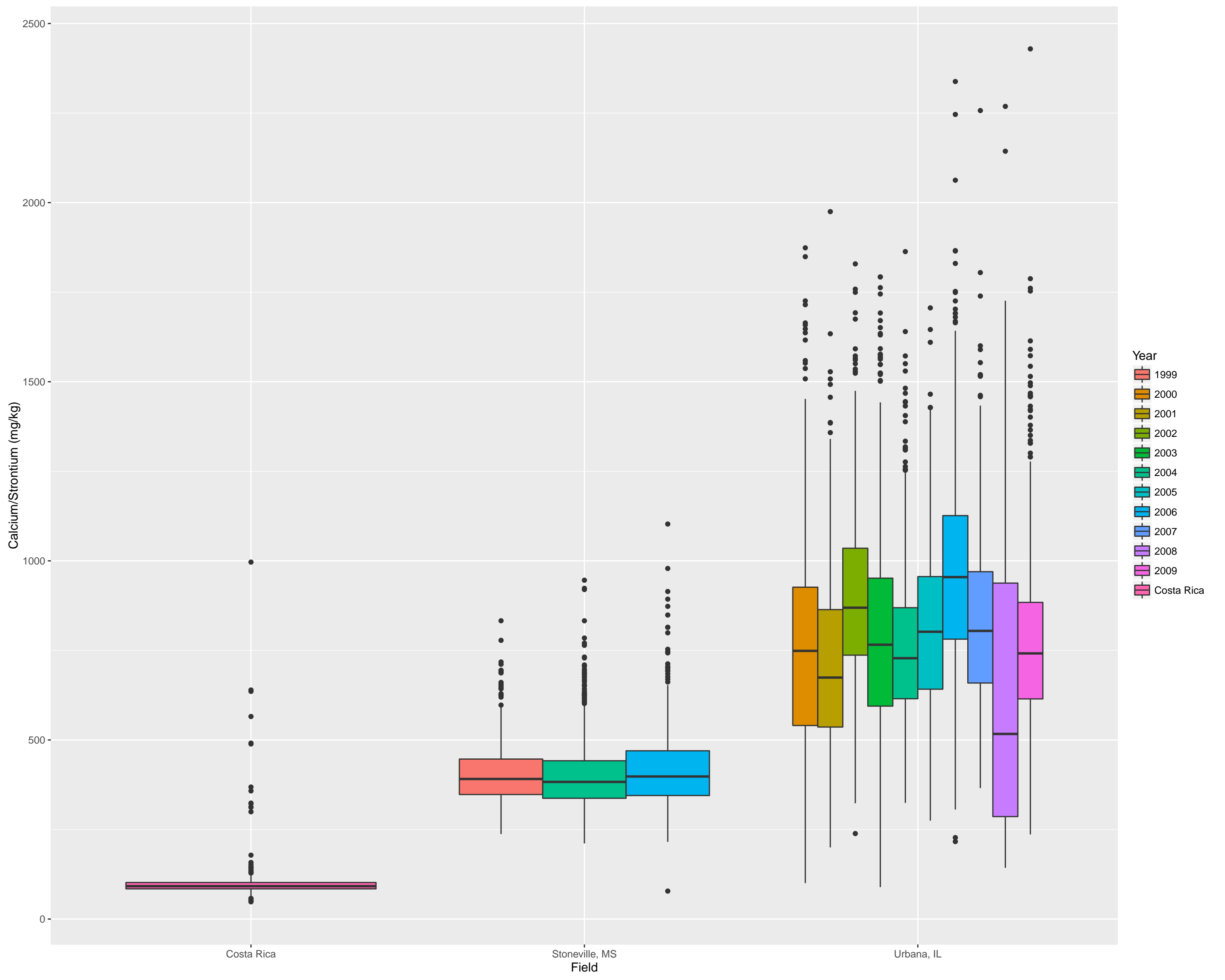

Supplement: Supplementary file 2 [file PLD3-2-e00033-s002.pdf]
