# Supplementary material for: Genomewide association study of ionomic traits on diverse soybean populations from germplasm collections
Source: Plant Direct. 2018 Jan 15;2(1):e00033. doi: 10.1002/pld3.33 (PMC6508489; doi:10.1002/pld3.33)
Supplement: Supplementary file 3 [file PLD3-2-e00033-s003.pdf]

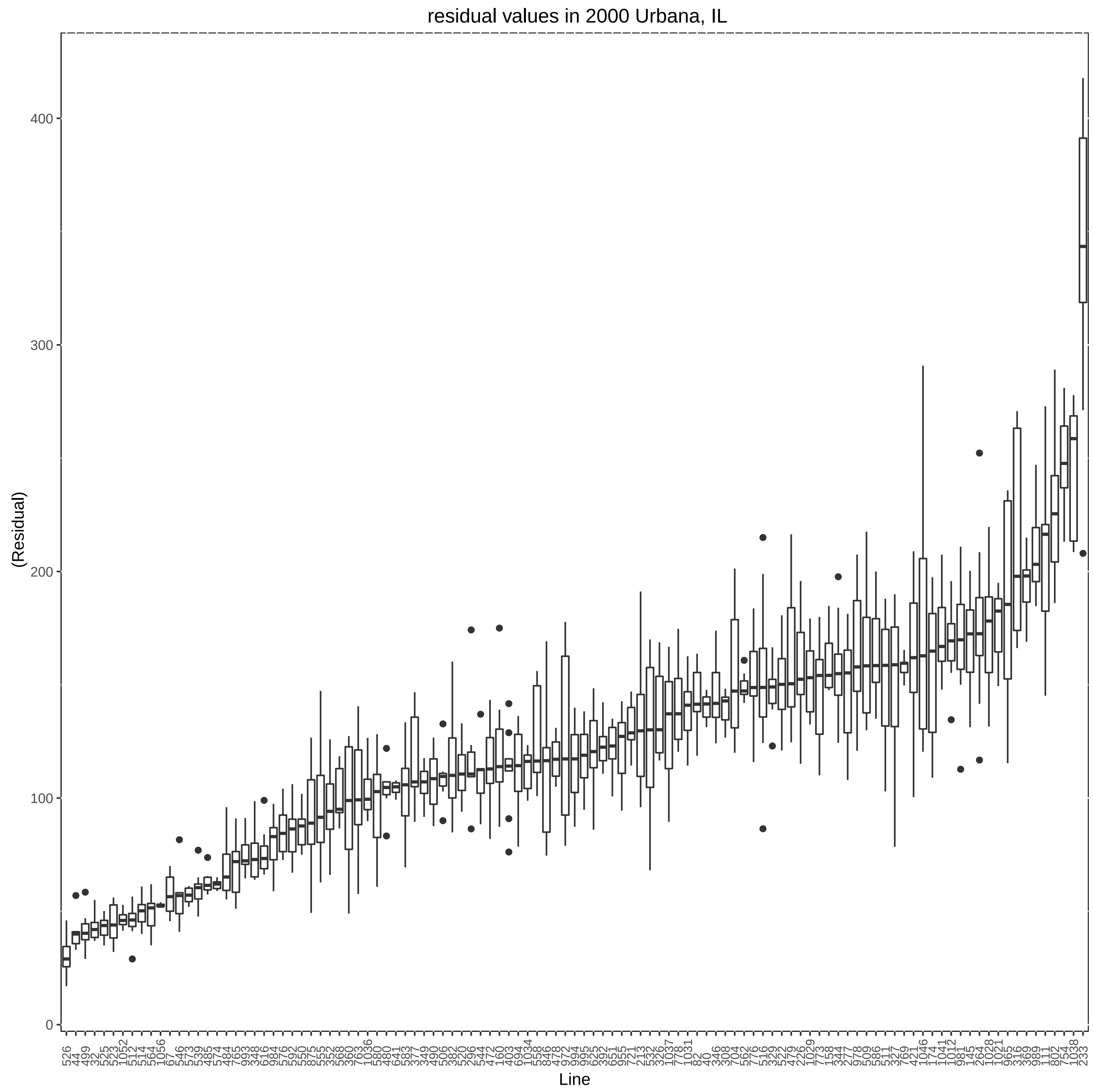
residual values in 2000 Urbana, IL
(Residual)
Line
0
100
200
300
400

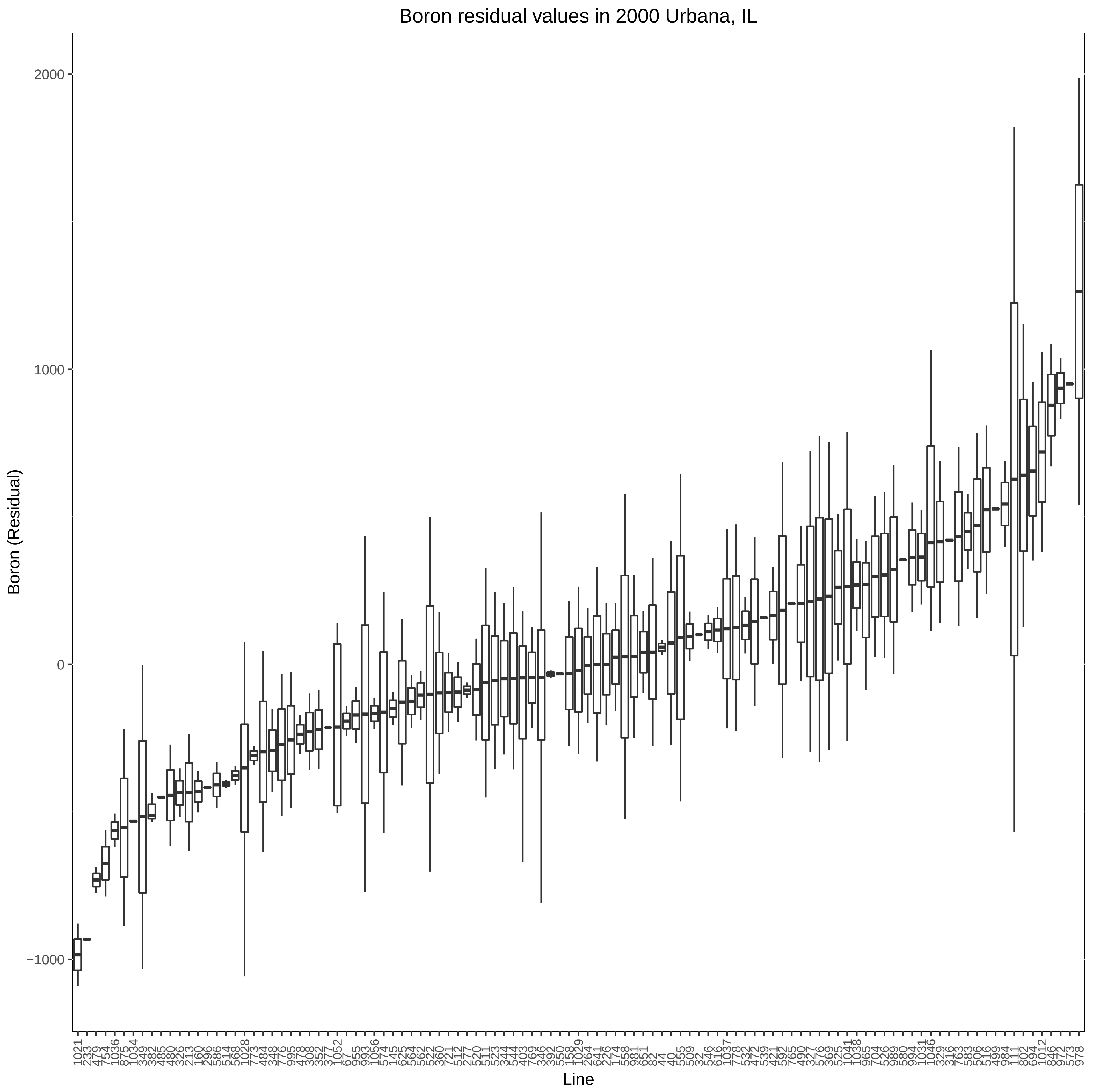
Boron residual values in 2000 Urbana, IL
Boron (Residual)
Line
−1000
0
1000
2000

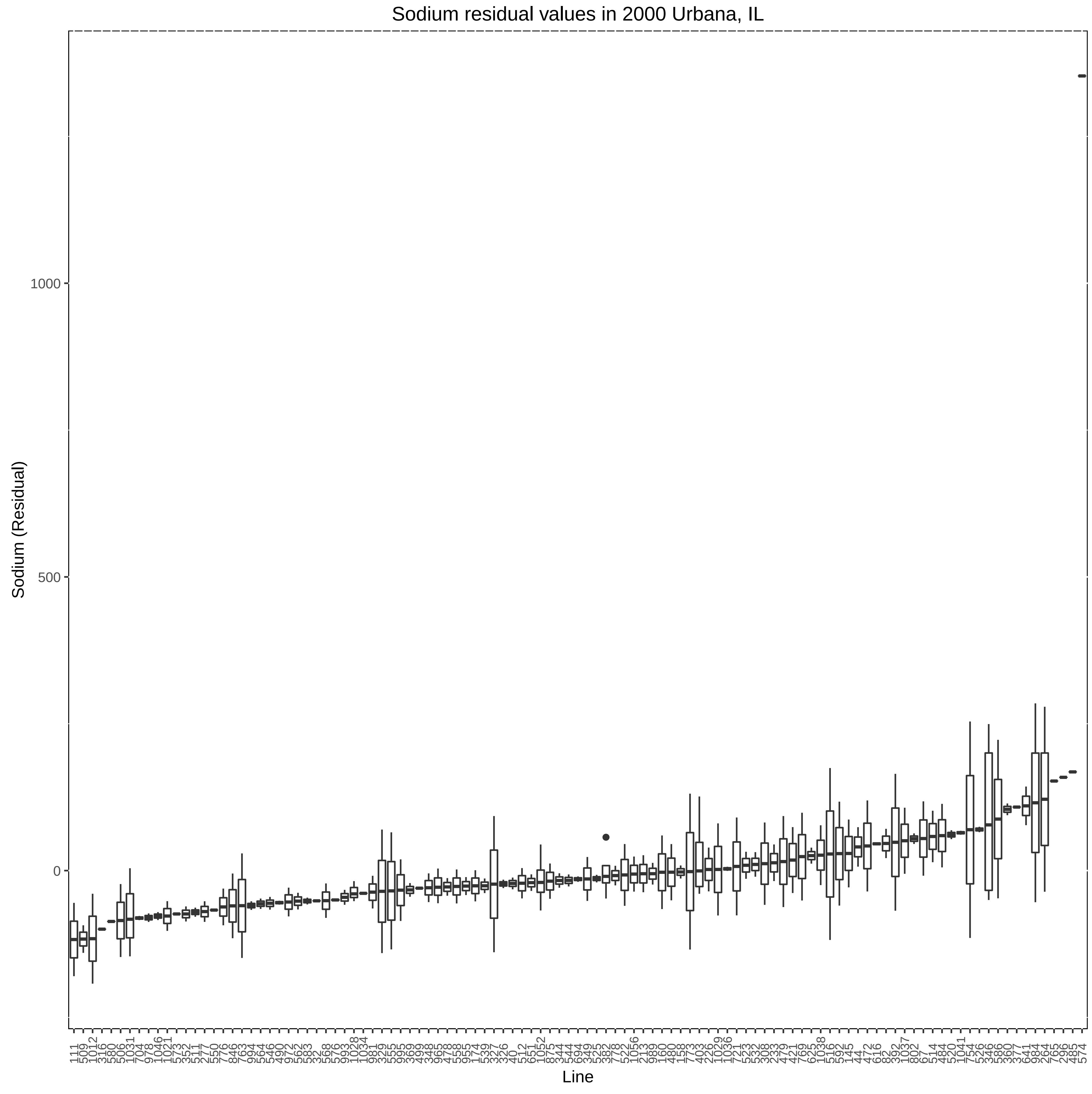
Sodium residual values in 2000 Urbana, IL
Sodium (Residual)
Line
0
500
1000

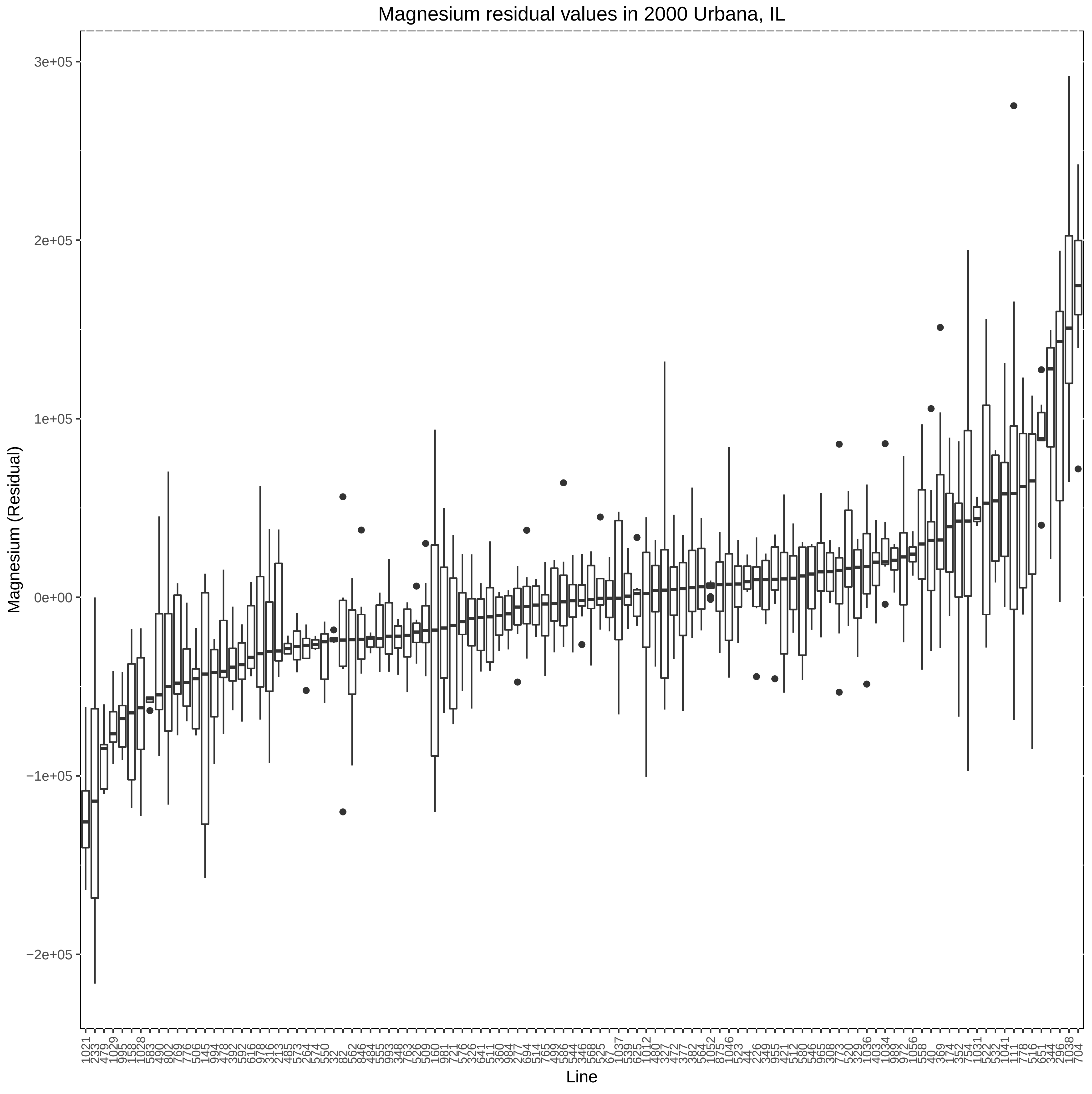
Magnesium residual values in 2000 Urbana, IL
Magnesium (Residual)
Line
−2e+05
−1e+05
0e+00
1e+05
2e+05
3e+05

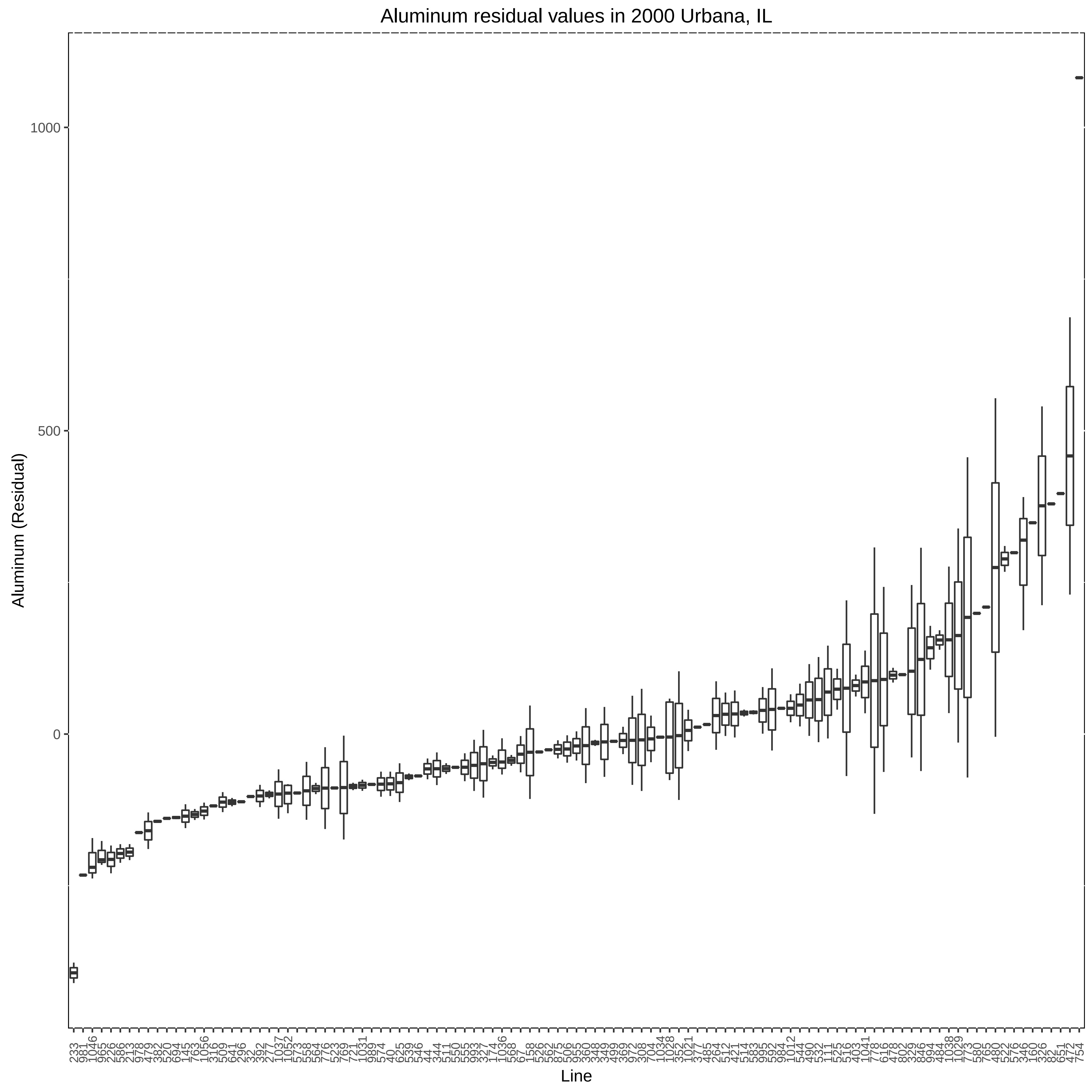
Aluminum residual values in 2000 Urbana, IL
Aluminum (Residual)
1000
500
0
Line

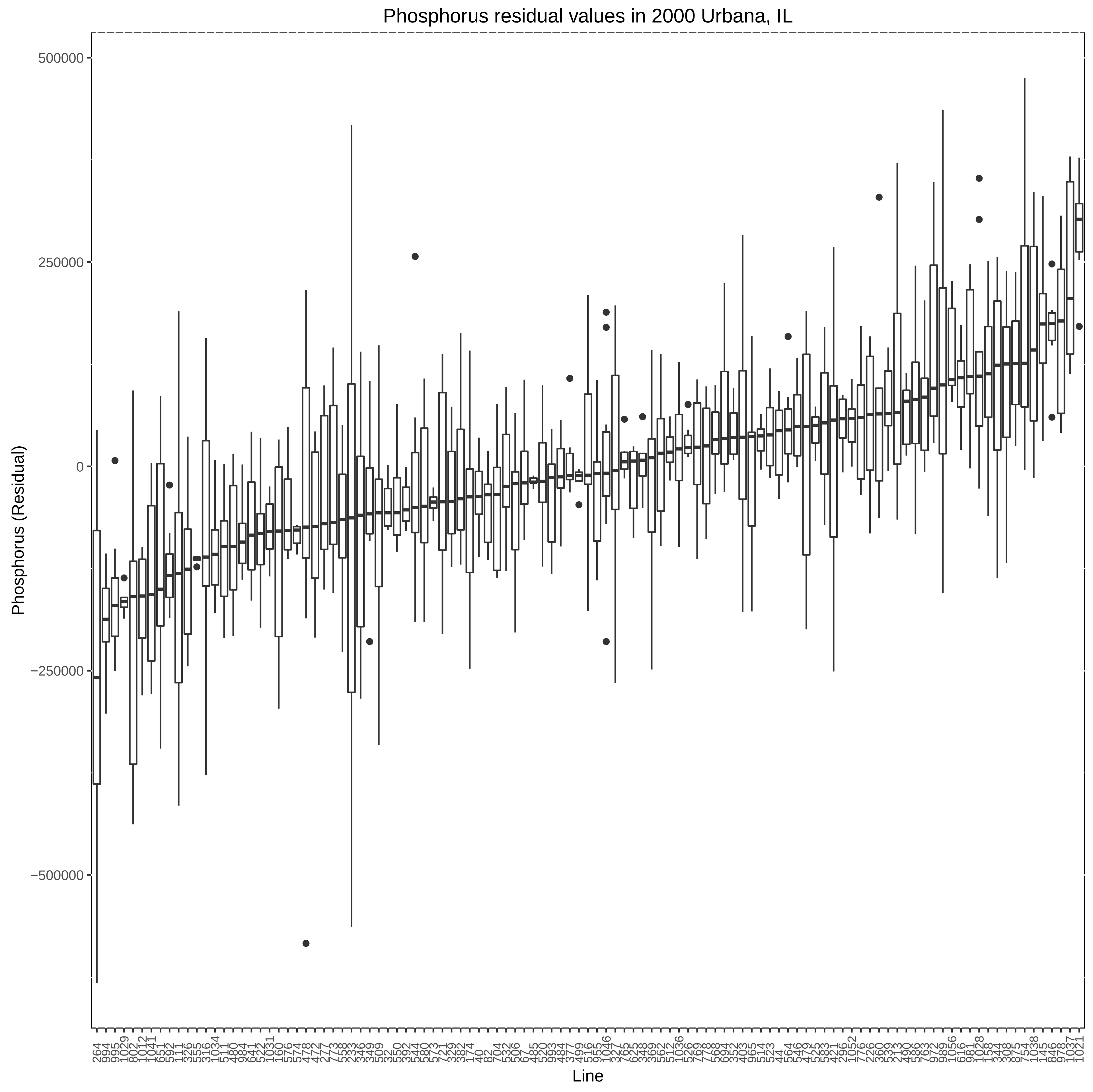
Phosphorus residual values in 2000 Urbana, IL
Phosphorus (Residual)
500000
250000
0
−250000
−500000
Line

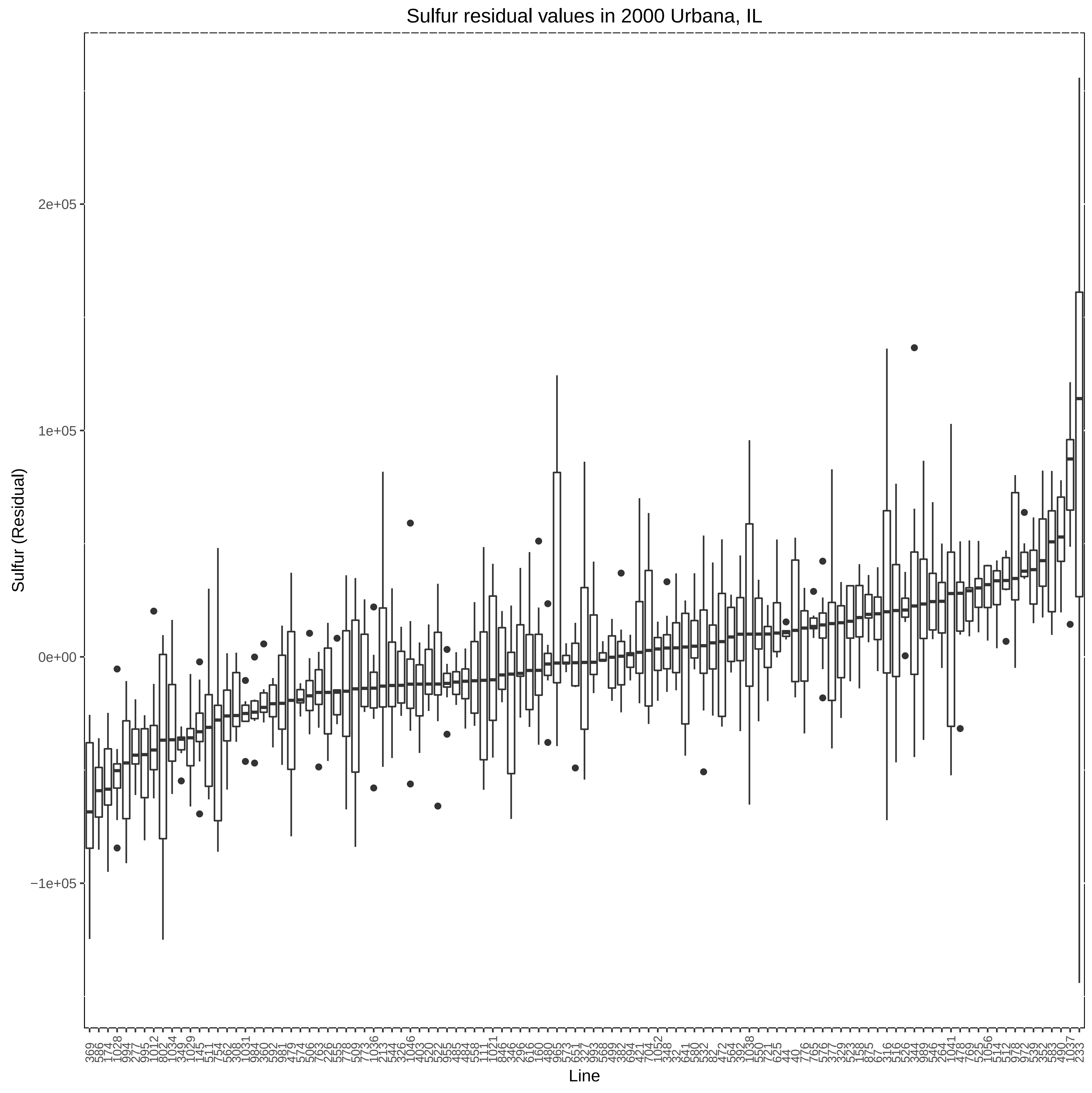
Sulfur residual values in 2000 Urbana, IL
Sulfur (Residual)
2e+05
1e+05
0e+00
−1e+05
Line

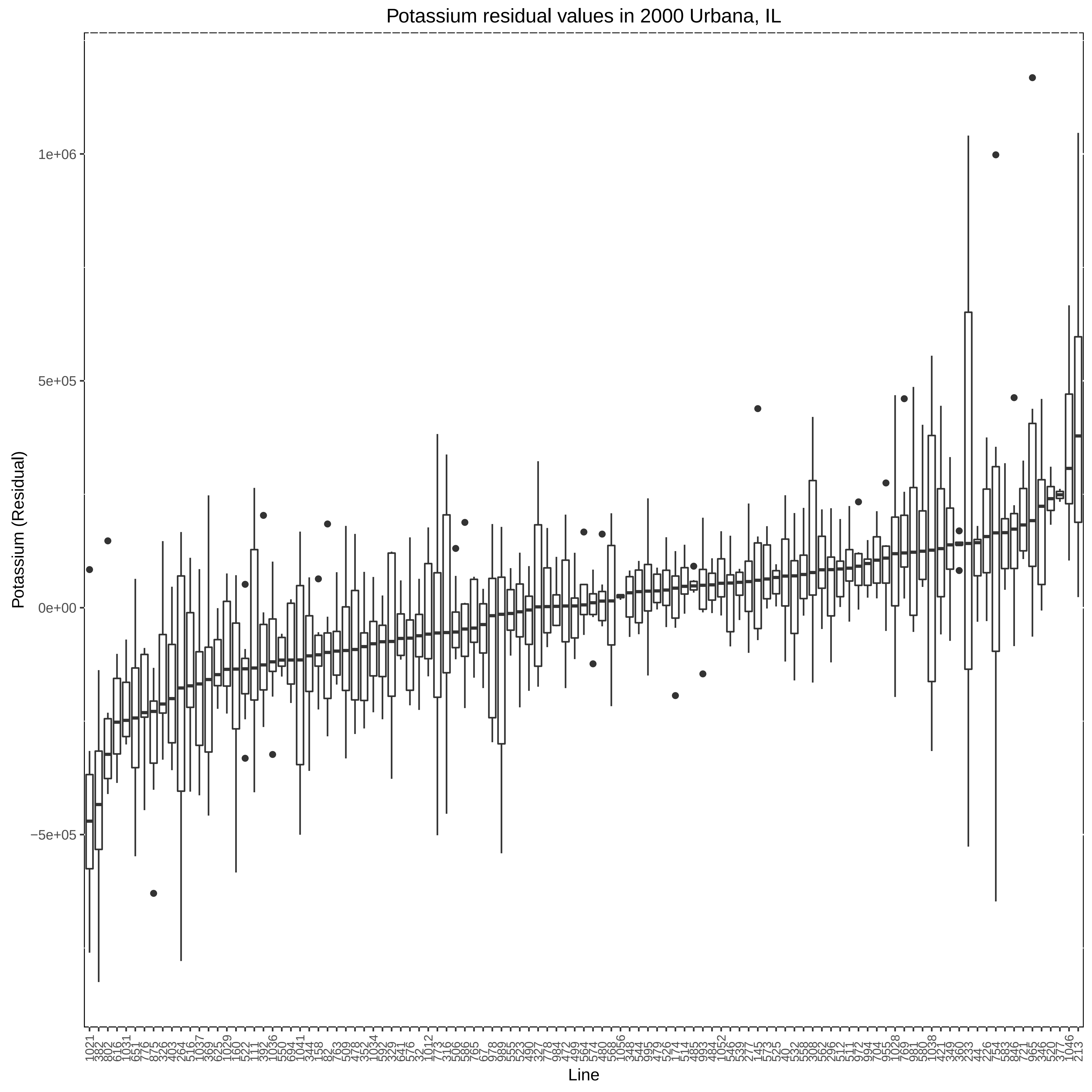
Potassium residual values in 2000 Urbana, IL
Potassium (Residual)
1e+06
5e+05
0e+00
−5e+05
Line

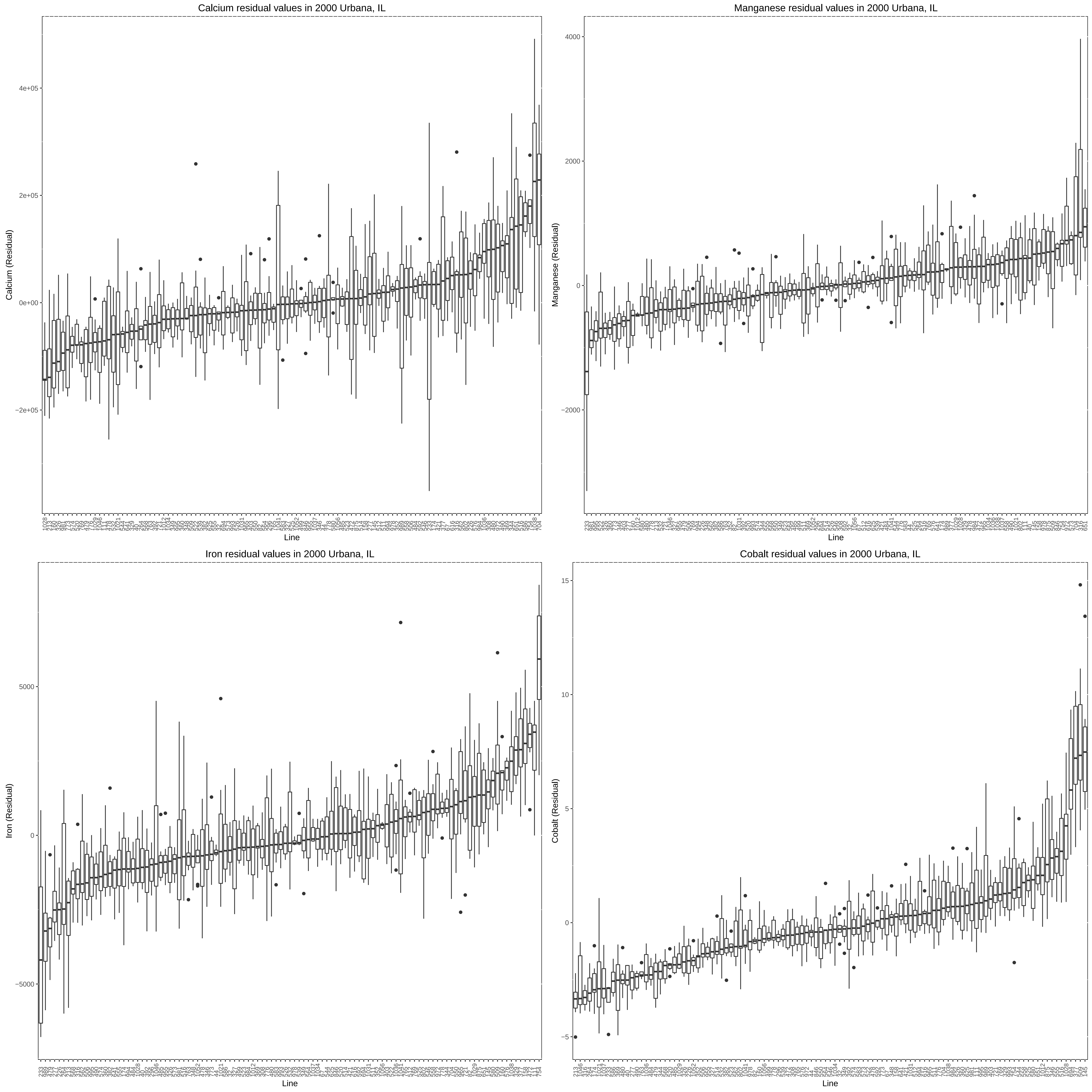
Calcium residual values in 2000 Urbana, IL
Calcium (Residual)
4e+05
2e+05
0e+00
-2e+05
Line
Manganese residual values in 2000 Urbana, IL
Manganese (Residual)
4000
2000
0
-2000
Line
Iron residual values in 2000 Urbana, IL
Iron (Residual)
5000
0
-5000
Line
Cobalt residual values in 2000 Urbana, IL
Cobalt (Residual)
15
10
5
0
-5
Line

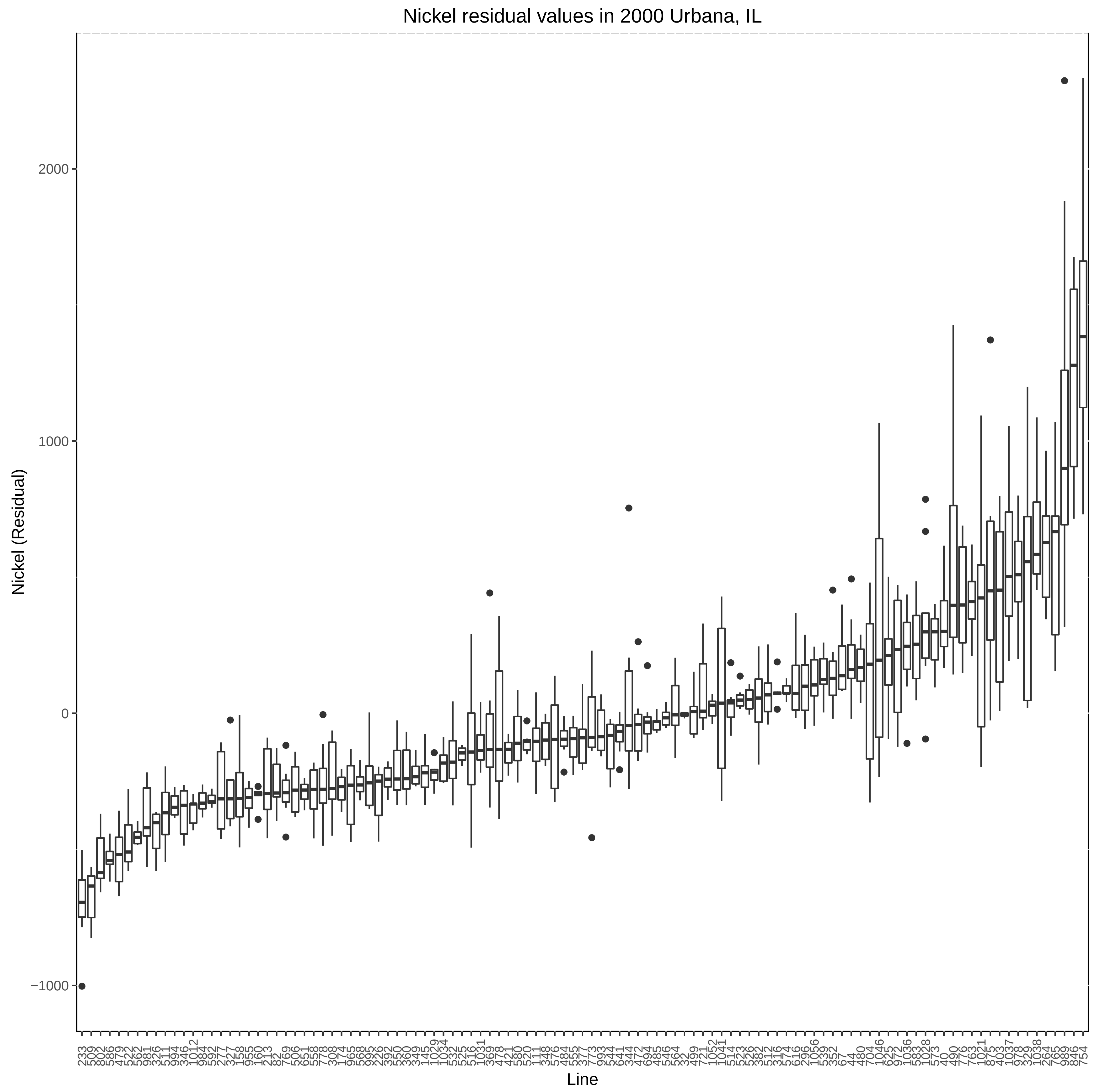
Nickel residual values in 2000 Urbana, IL
Nickel (Residual)
2000
1000
0
−1000
Line

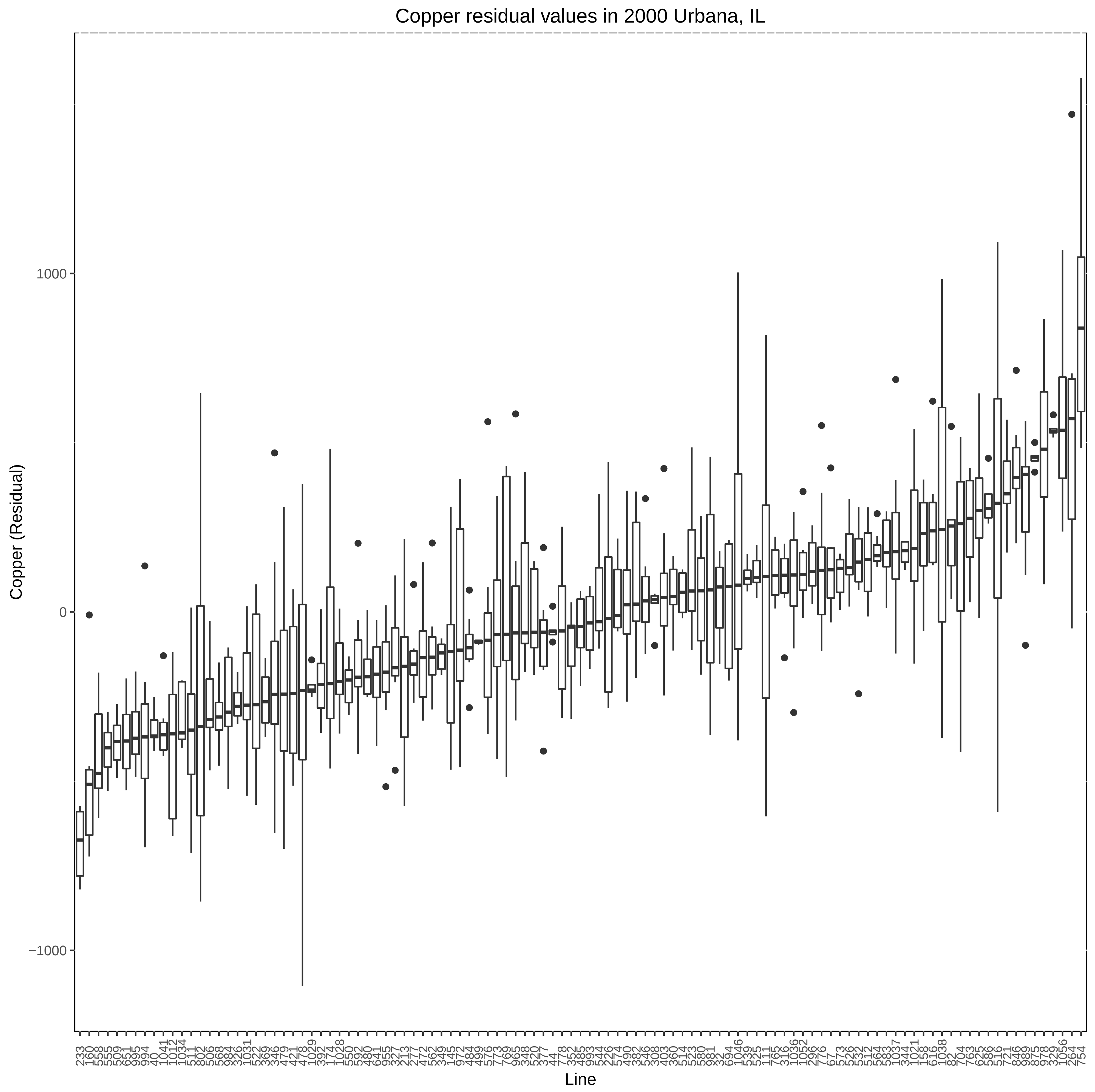
Copper residual values in 2000 Urbana, IL
Copper (Residual)
1000
0
−1000
Line

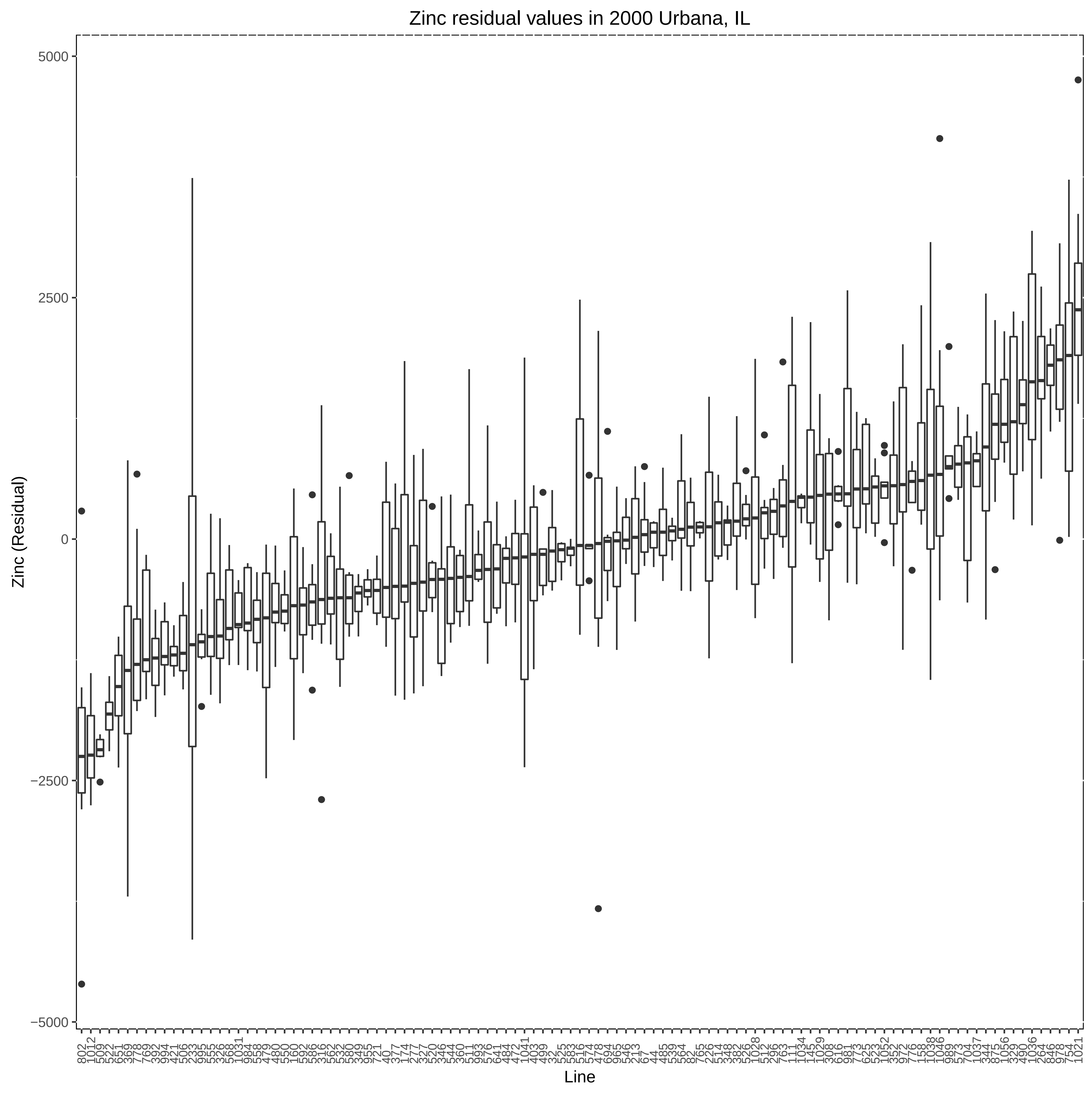
Zinc residual values in 2000 Urbana, IL
Zinc (Residual)
5000
2500
0
−2500
−5000
Line

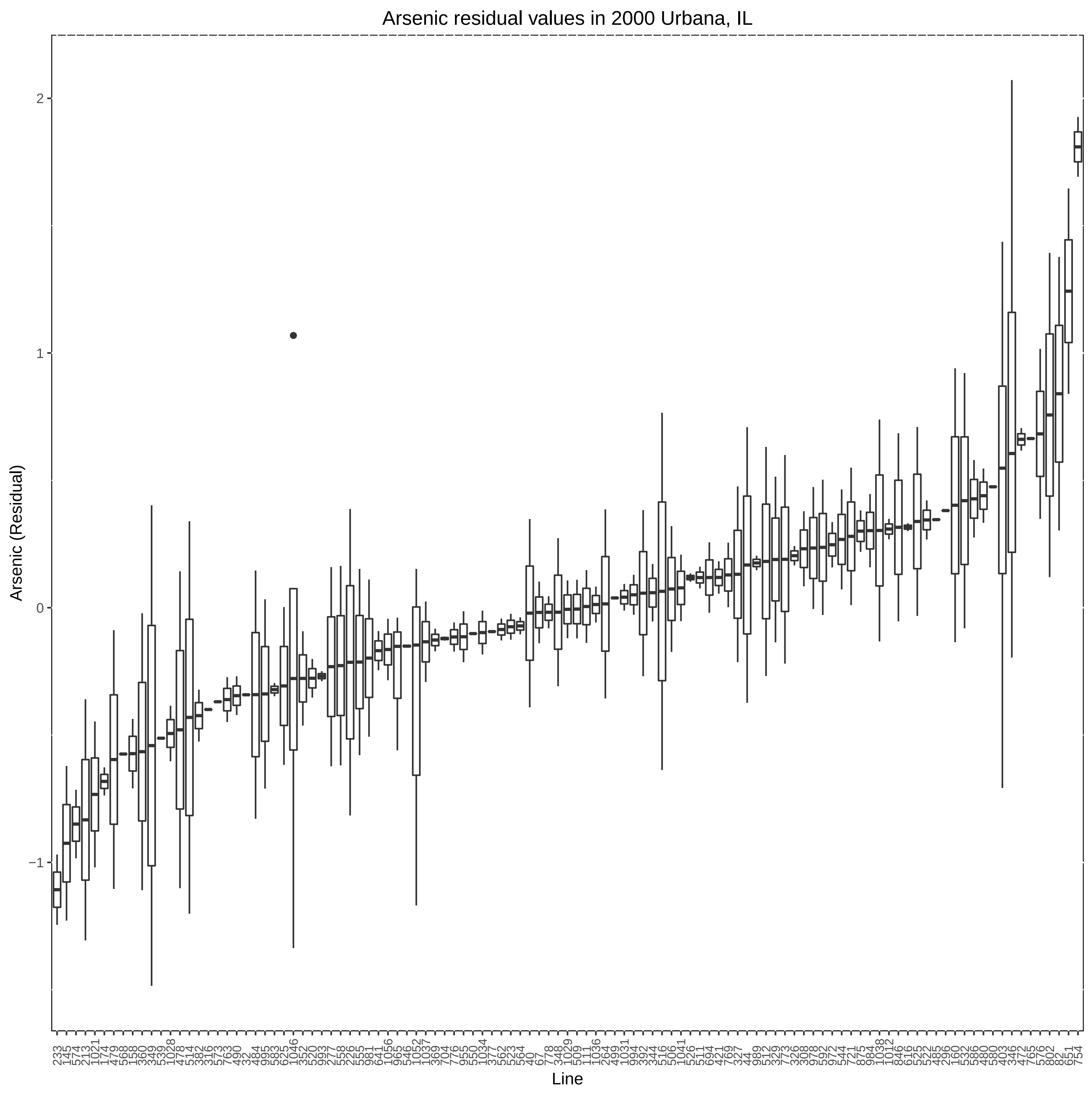
Arsenic residual values in 2000 Urbana, IL
Arsenic (Residual)
2
1
0
−1
Line

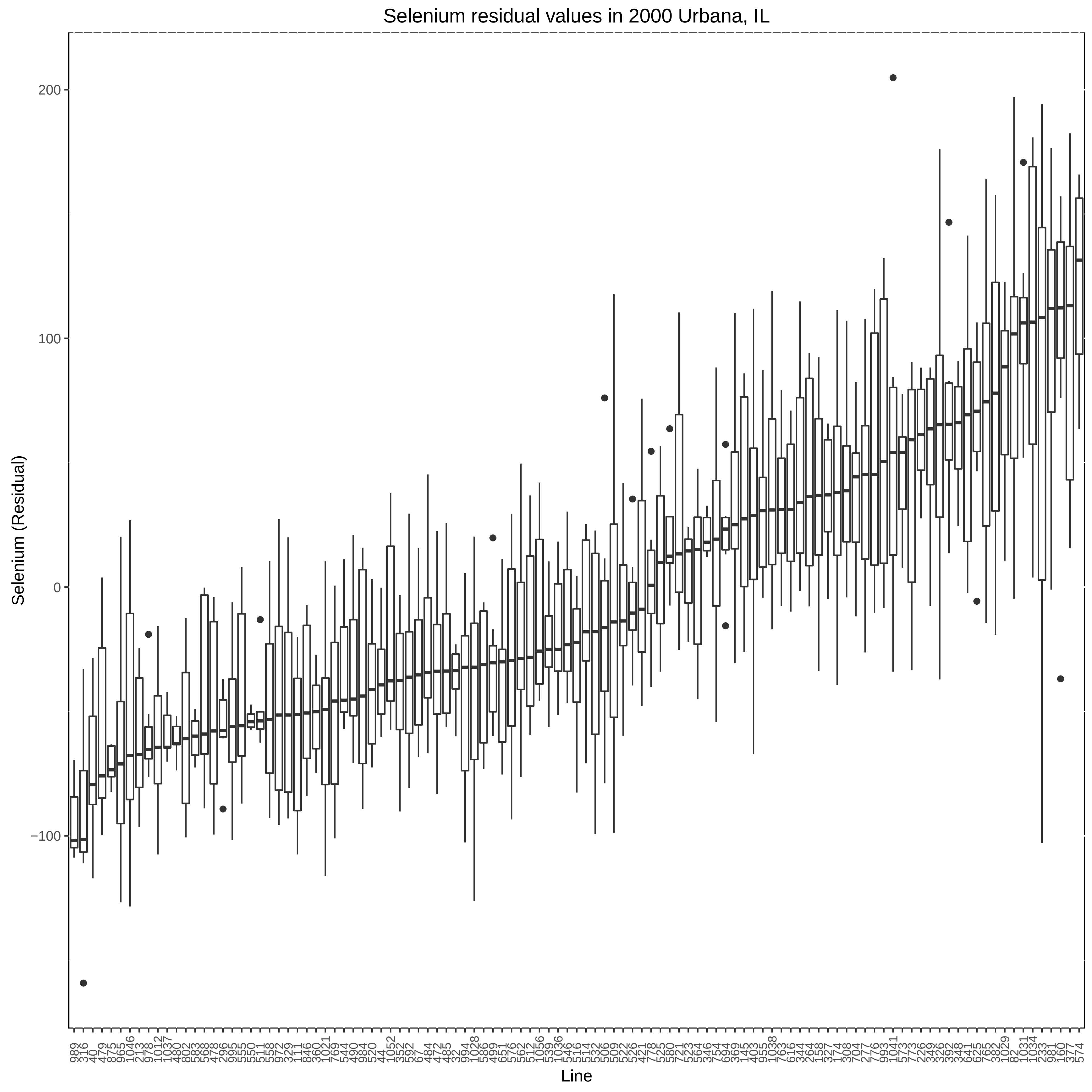
Selenium residual values in 2000 Urbana, IL
Selenium (Residual)
Line
200
100
0
-100

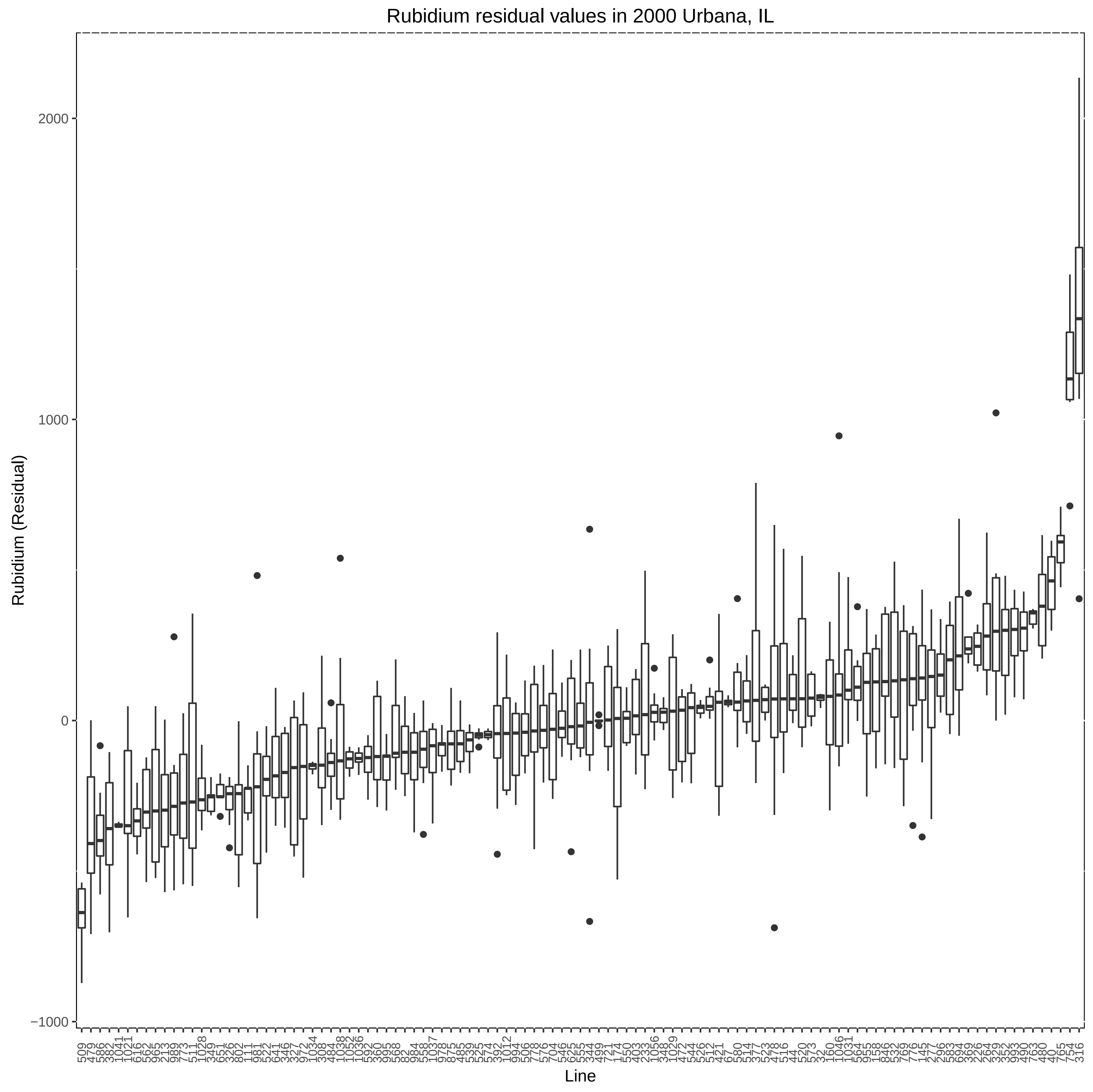
Rubidium residual values in 2000 Urbana, IL
Rubidium (Residual)
Line
2000
1000
0
-1000

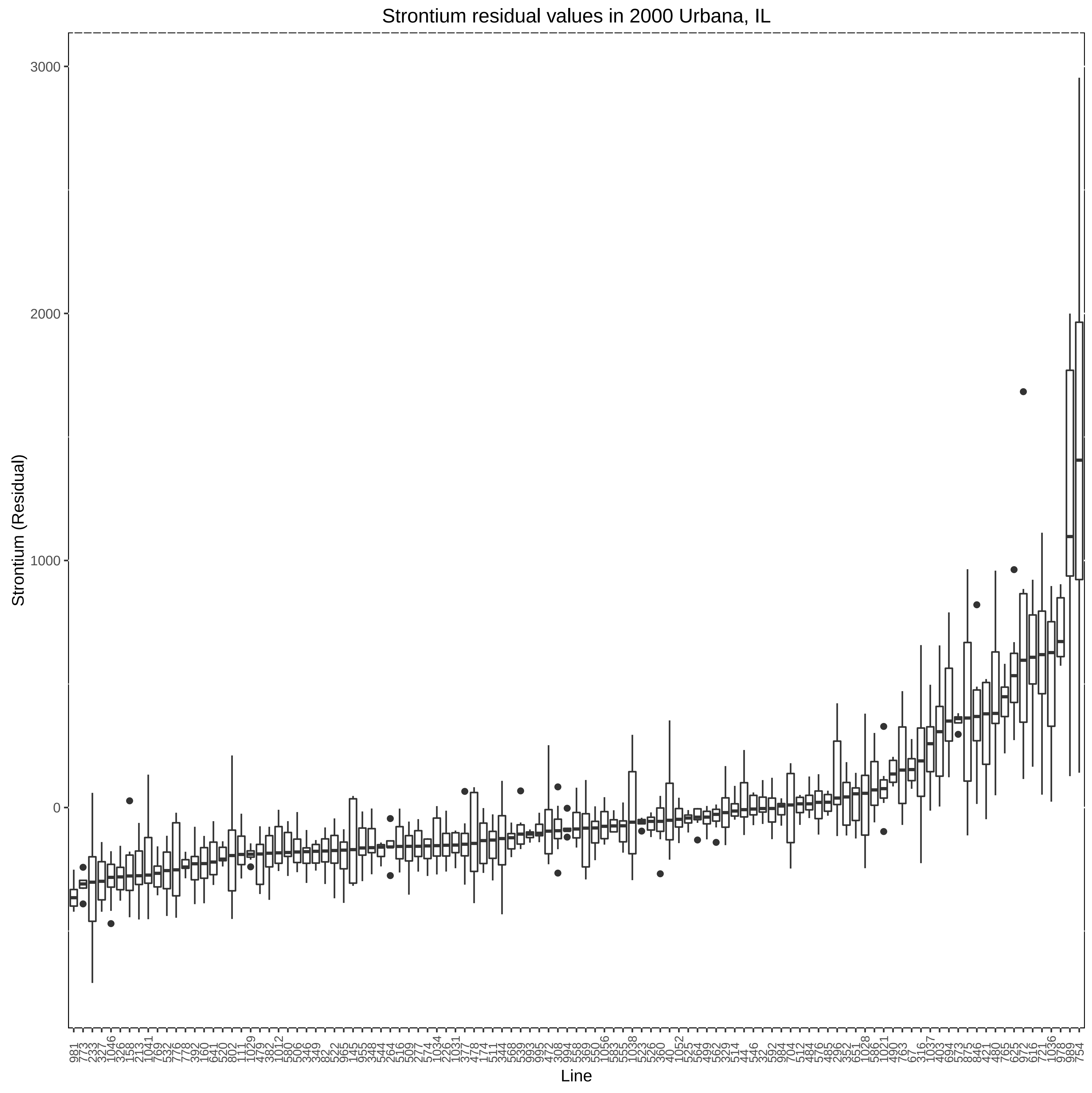
Strontium residual values in 2000 Urbana, IL
Strontium (Residual)
Line
3000
2000
1000
0

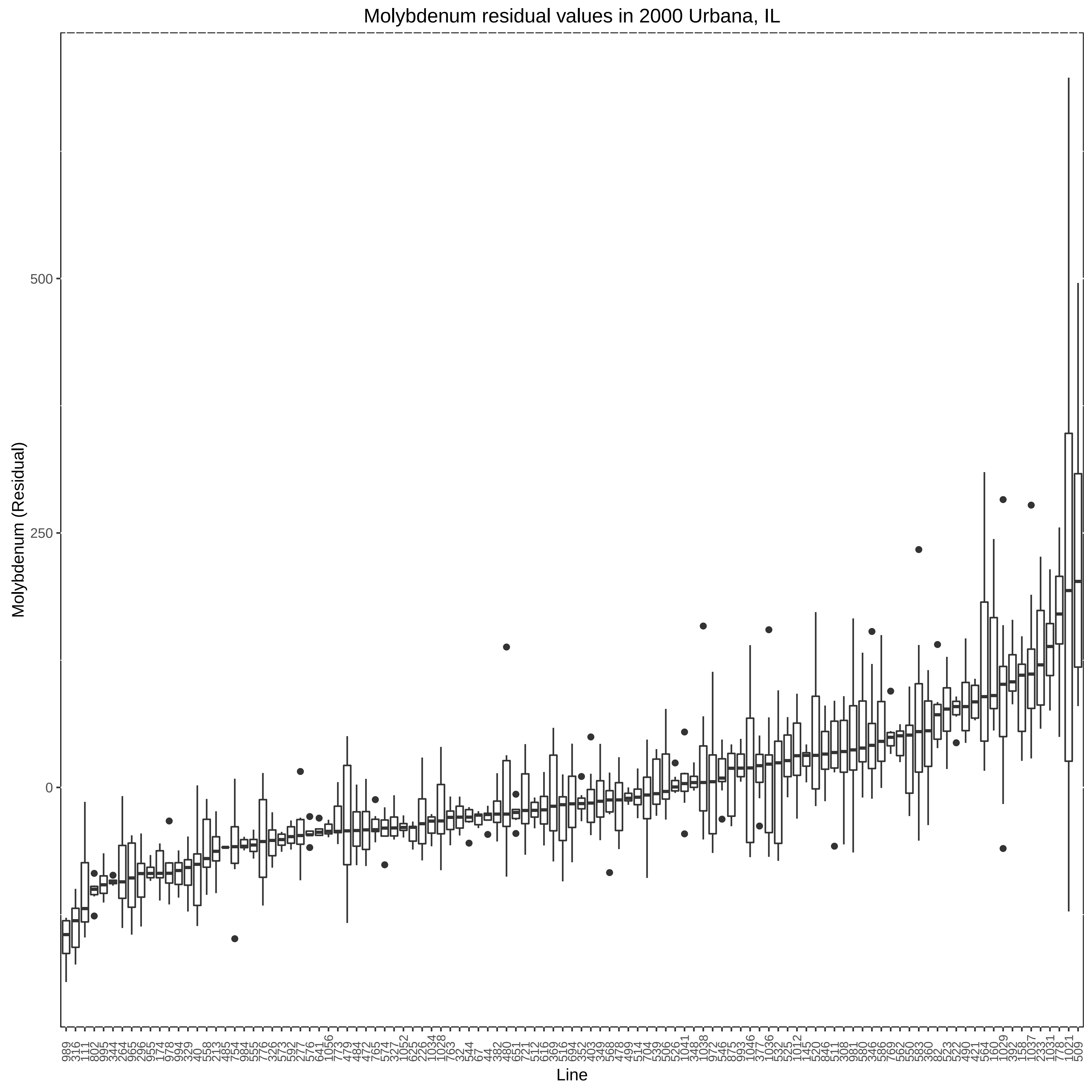
Molybdenum residual values in 2000 Urbana, IL
Molybdenum (Residual)
Line
500
250
0

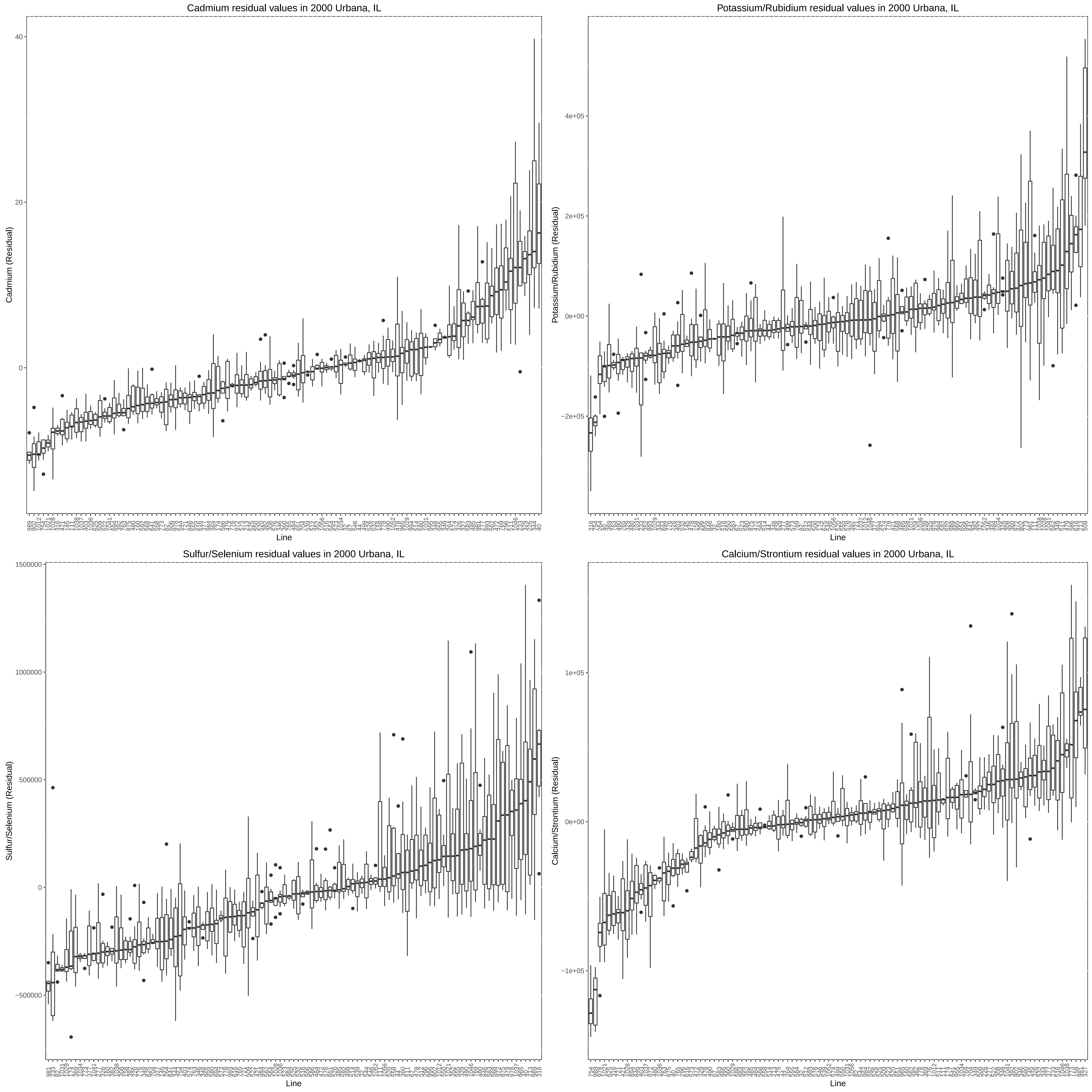
Cadmium residual values in 2000 Urbana, IL
Cadmium (Residual)
40
20
0
Line
Potassium/Rubidium residual values in 2000 Urbana, IL
Potassium/Rubidium (Residual)
4e+05
2e+05
0e+00
-2e+05
Line
Sulfur/Selenium residual values in 2000 Urbana, IL
Sulfur/Selenium (Residual)
1500000
1000000
500000
0
-500000
Line
Calcium/Strontium residual values in 2000 Urbana, IL
Calcium/Strontium (Residual)
1e+05
0e+00
-1e+05
Line

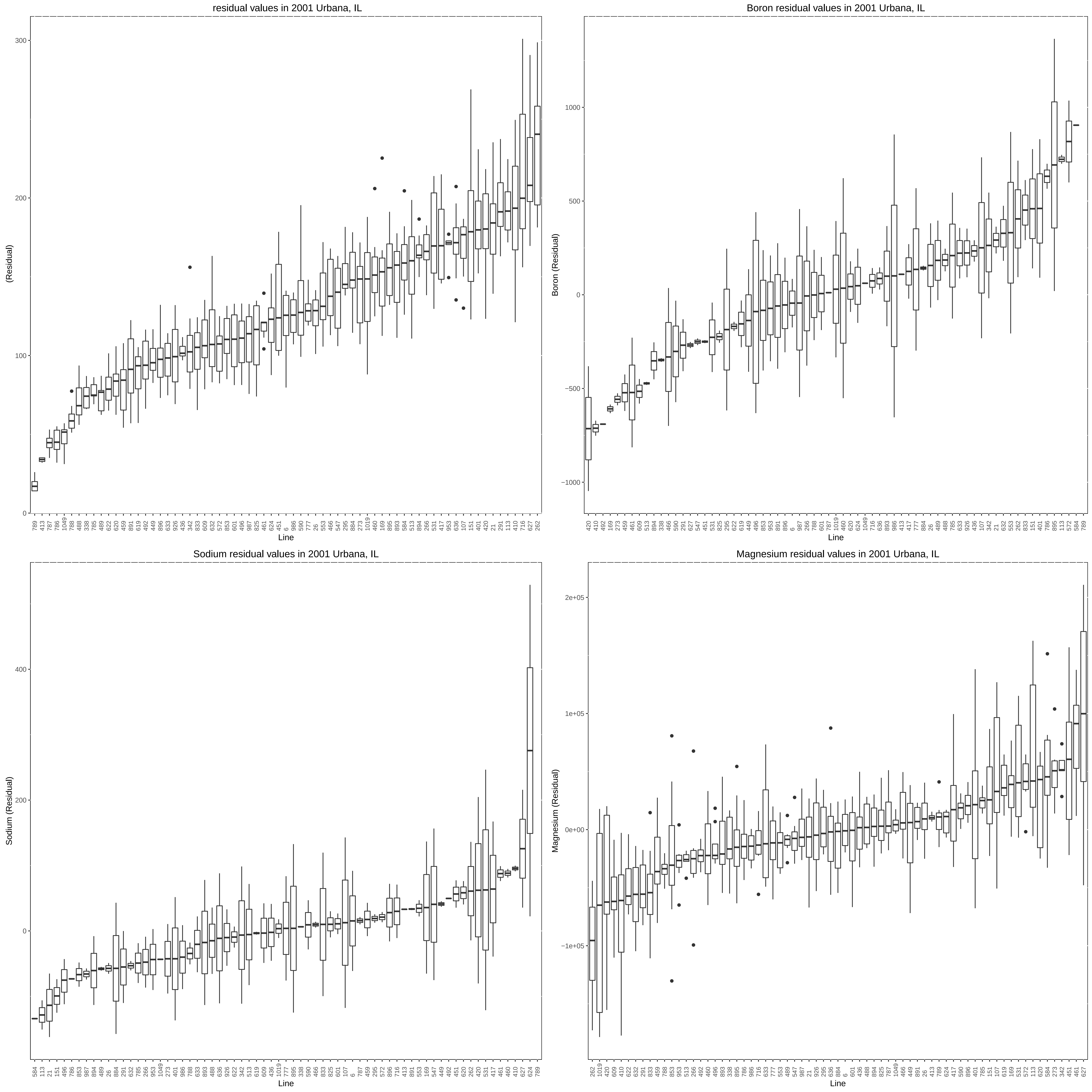

residual values in 2001 Urbana, IL
(Residual)
Line
300
200
100
0
Boron residual values in 2001 Urbana, IL
Boron (Residual)
Line
1000
500
0
−500
−1000
Sodium residual values in 2001 Urbana, IL
Sodium (Residual)
Line
400
200
0
Magnesium residual values in 2001 Urbana, IL
Magnesium (Residual)
Line
2e+05
1e+05
0e+00
−1e+05

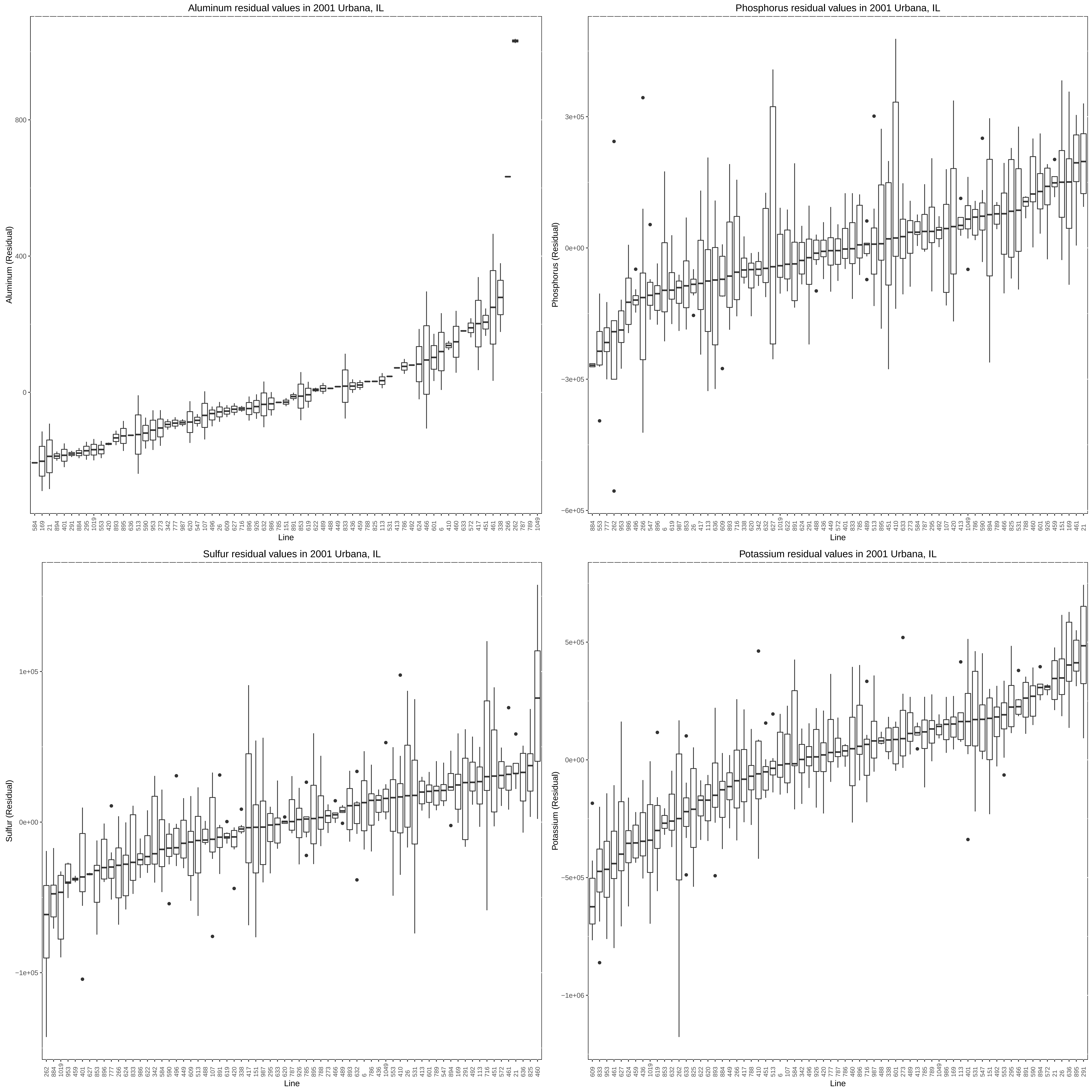
Aluminum residual values in 2001 Urbana, IL
Aluminum (Residual)
800
400
0
Line
584 169 21 894 401 291 884 295 1019 553 420 893 895 636 513 590 953 273 342 777 987 620 547 107 496 26 609 627 716 896 926 632 986 785 151 891 853 619 622 489 488 449 833 436 459 788 825 113 531 413 786 492 624 466 601 6 410 460 633 572 417 451 461 338 266 262 787 789 1049
Phosphorus residual values in 2001 Urbana, IL
Phosphorus (Residual)
3e+05
0e+00
-3e+05
-6e+05
Line
884 553 777 262 953 986 496 266 547 896 6 619 987 853 26 417 113 636 609 893 716 338 620 342 632 627 1019 622 891 624 291 488 436 449 572 401 833 785 489 513 895 451 410 633 273 584 787 295 492 107 420 413 1049 786 590 894 789 466 825 531 788 460 601 926 459 151 169 461 21
Sulfur residual values in 2001 Urbana, IL
Sulfur (Residual)
1e+05
0e+00
-1e+05
Line
262 884 1019 953 459 401 627 853 896 777 266 624 833 986 622 342 584 590 496 449 609 513 488 107 891 619 420 338 417 151 987 295 633 620 787 926 785 895 788 273 466 489 893 632 6 786 436 1049 553 410 26 531 413 601 789 547 894 169 291 492 113 716 451 572 461 21 636 825 460
Potassium residual values in 2001 Urbana, IL
Potassium (Residual)
5e+05
0e+00
-5e+05
-1e+06
Line
609 833 953 461 627 624 459 436 1019 619 853 632 262 633 825 622 620 893 884 449 266 417 788 410 451 513 6 107 584 342 496 926 420 777 787 786 460 896 716 987 488 338 601 273 489 413 785 789 1049 986 169 113 401 531 547 151 492 553 295 466 891 590 894 572 21 26 636 895 291

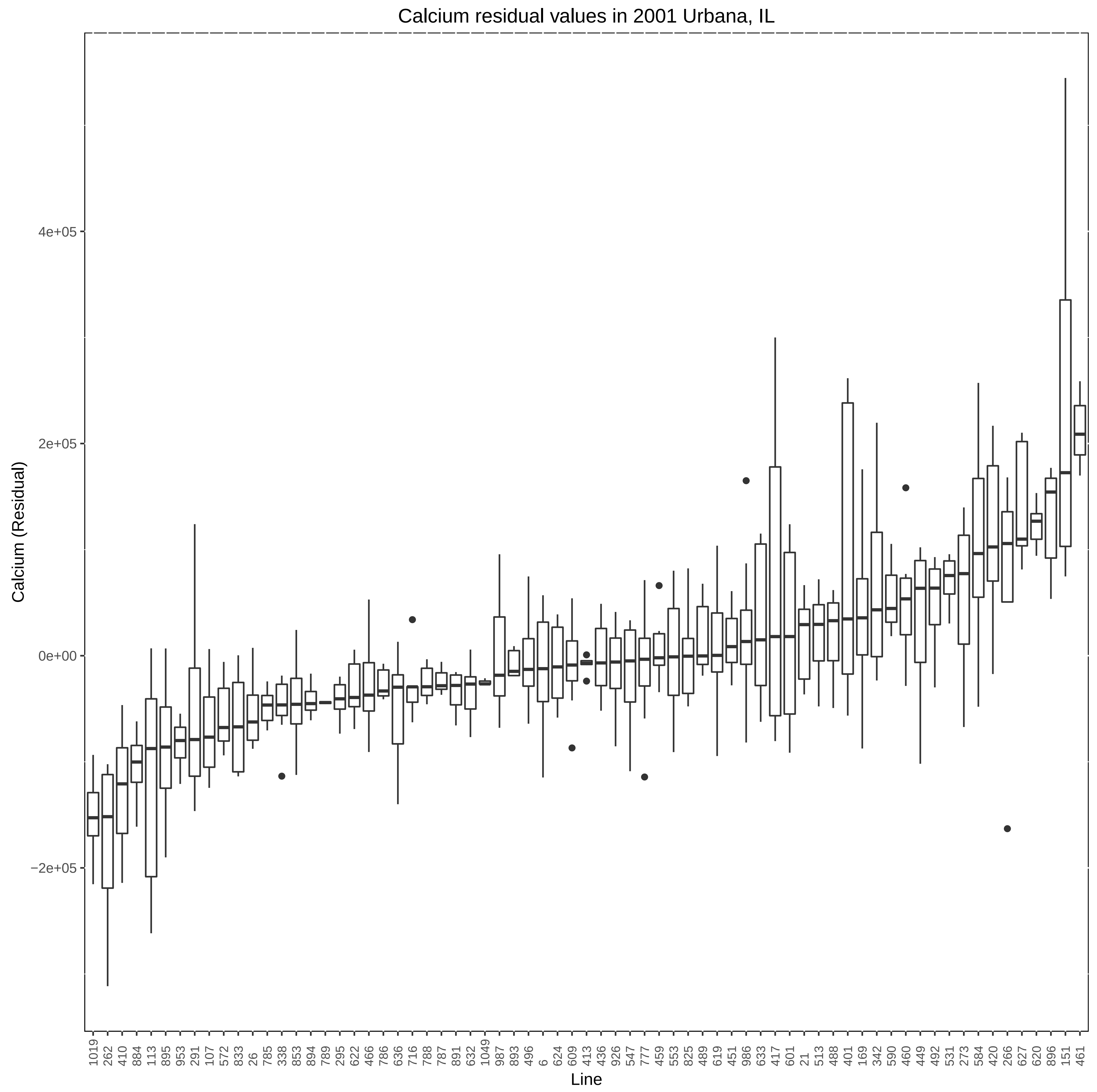
Calcium residual values in 2001 Urbana, IL
Calcium (Residual)
4e+05
2e+05
0e+00
-2e+05
Line
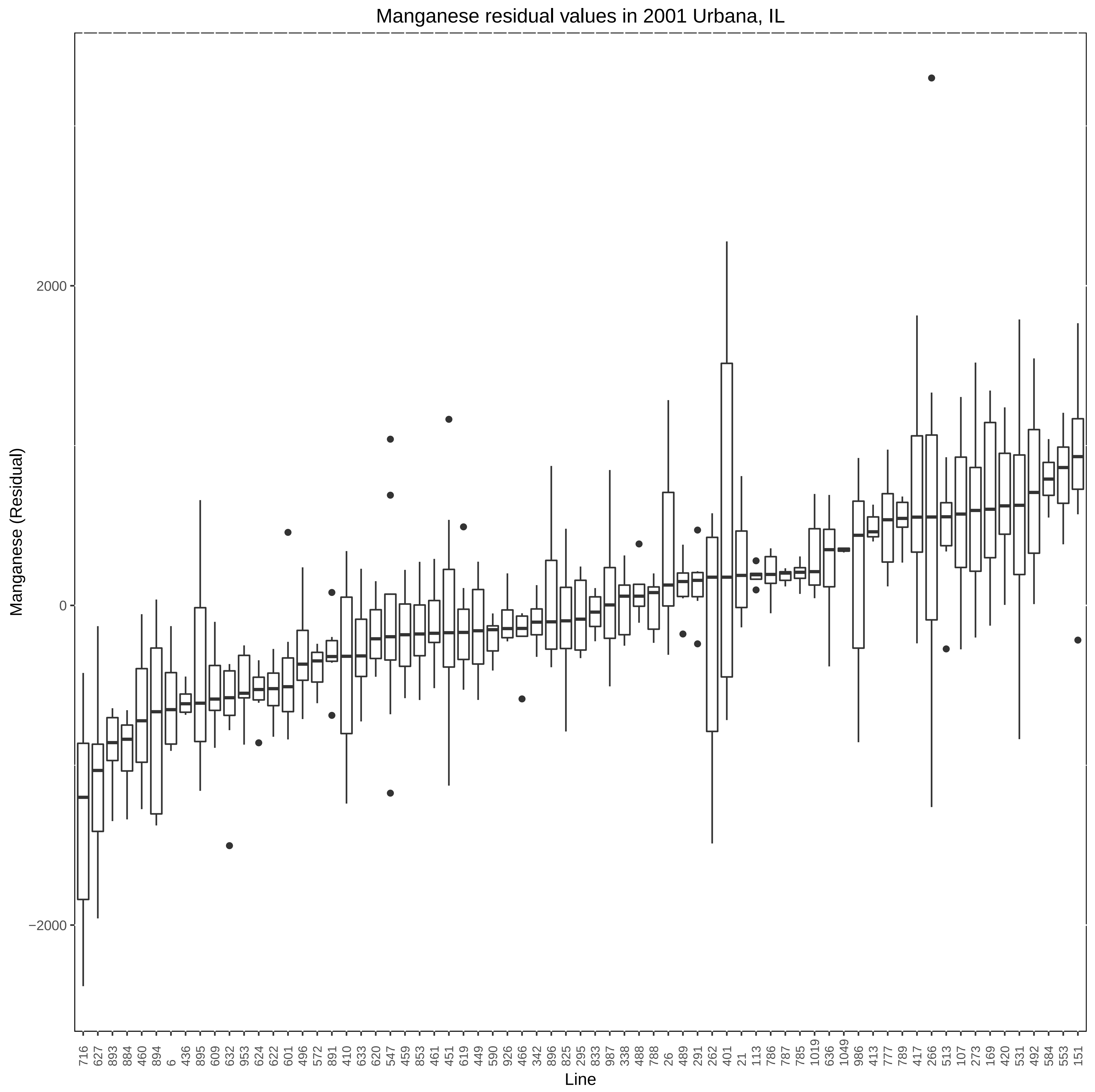
Manganese residual values in 2001 Urbana, IL
Manganese (Residual)
2000
0
-2000
Line
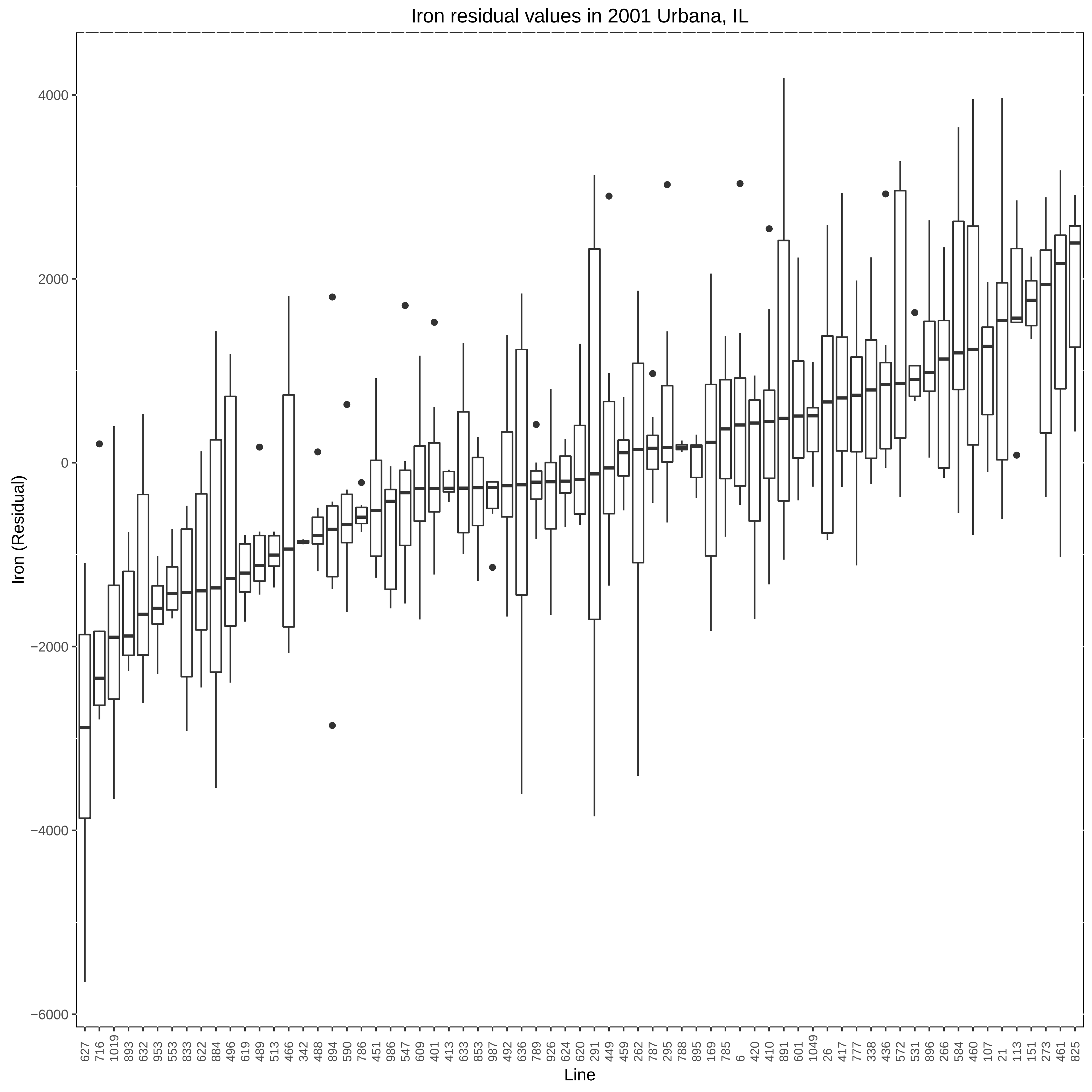
Iron residual values in 2001 Urbana, IL
Iron (Residual)
4000
2000
0
-2000
-4000
-6000
Line
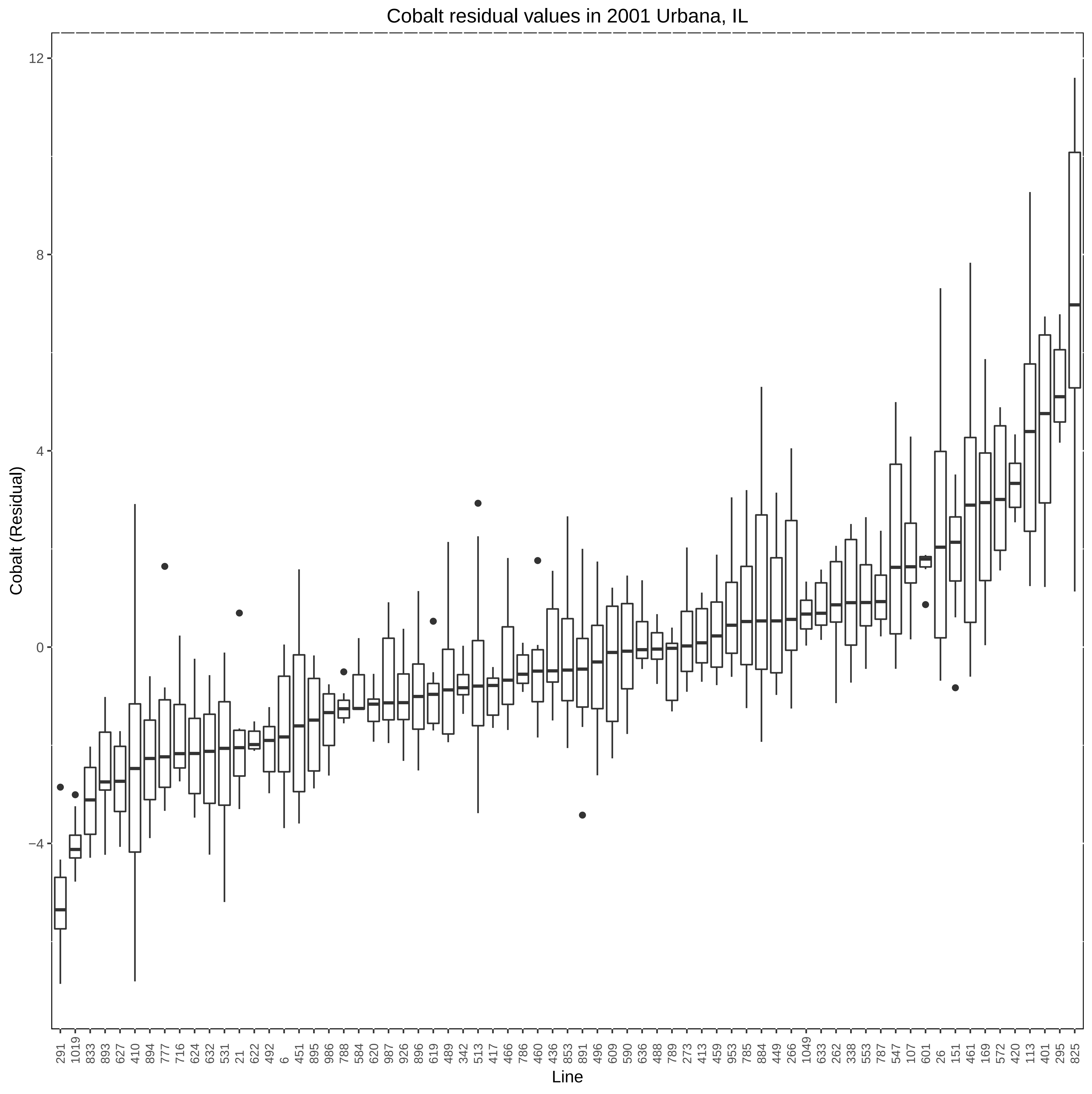
Cobalt residual values in 2001 Urbana, IL
Cobalt (Residual)
12
8
4
0
-4
Line

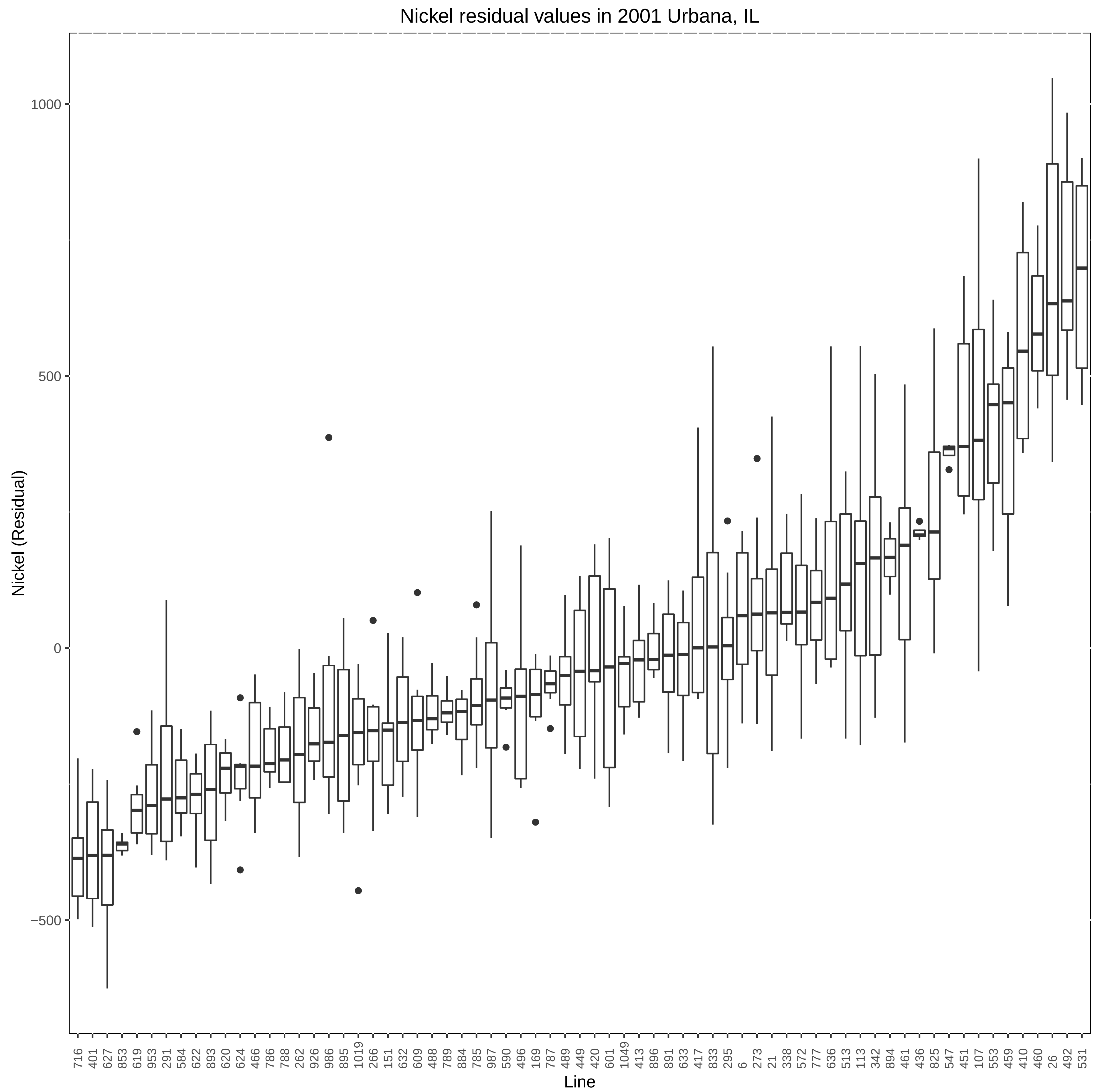
Nickel residual values in 2001 Urbana, IL
Nickel (Residual)
1000
500
0
-500
Line

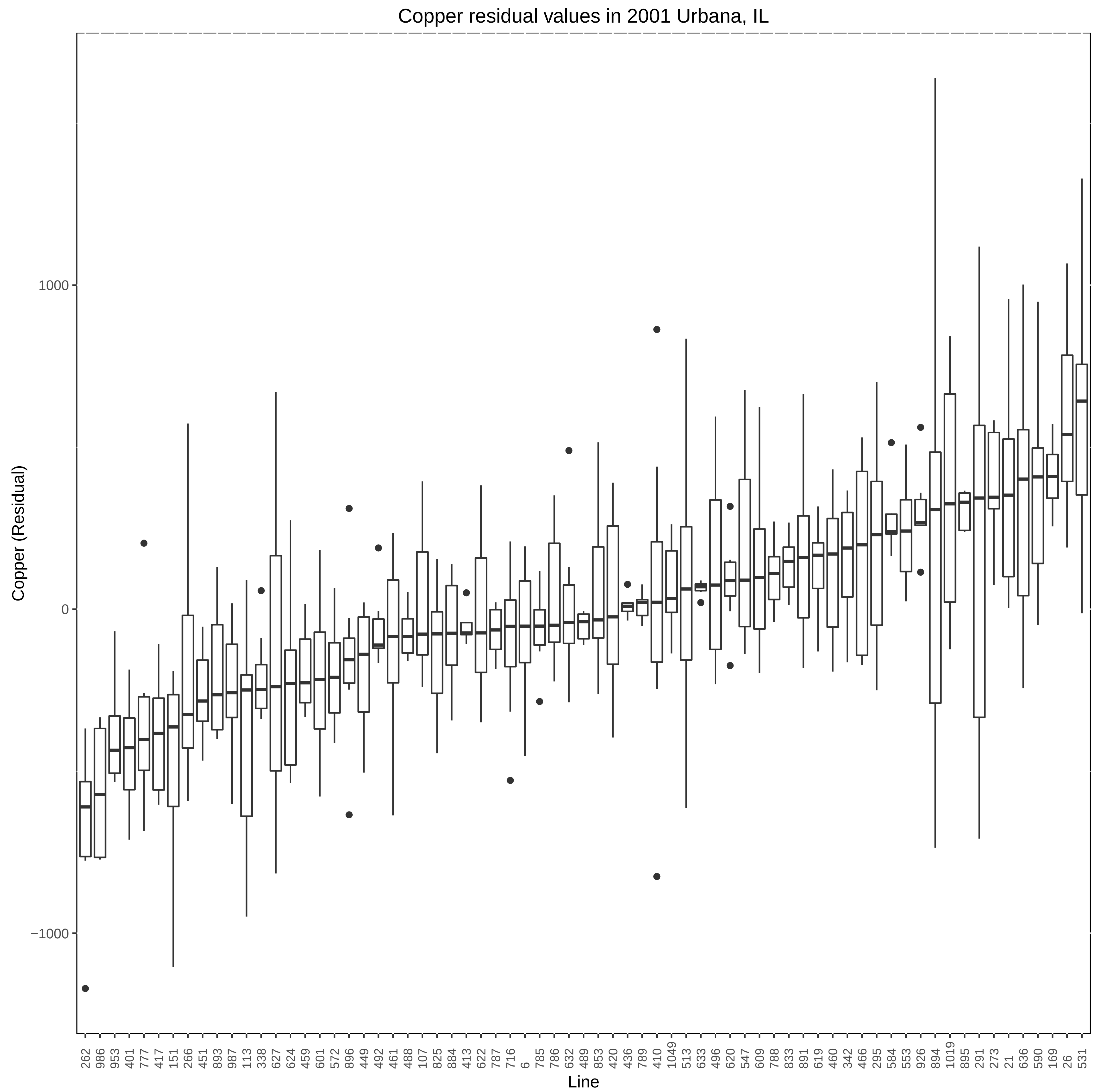
Copper residual values in 2001 Urbana, IL
Copper (Residual)
1000
0
-1000
Line

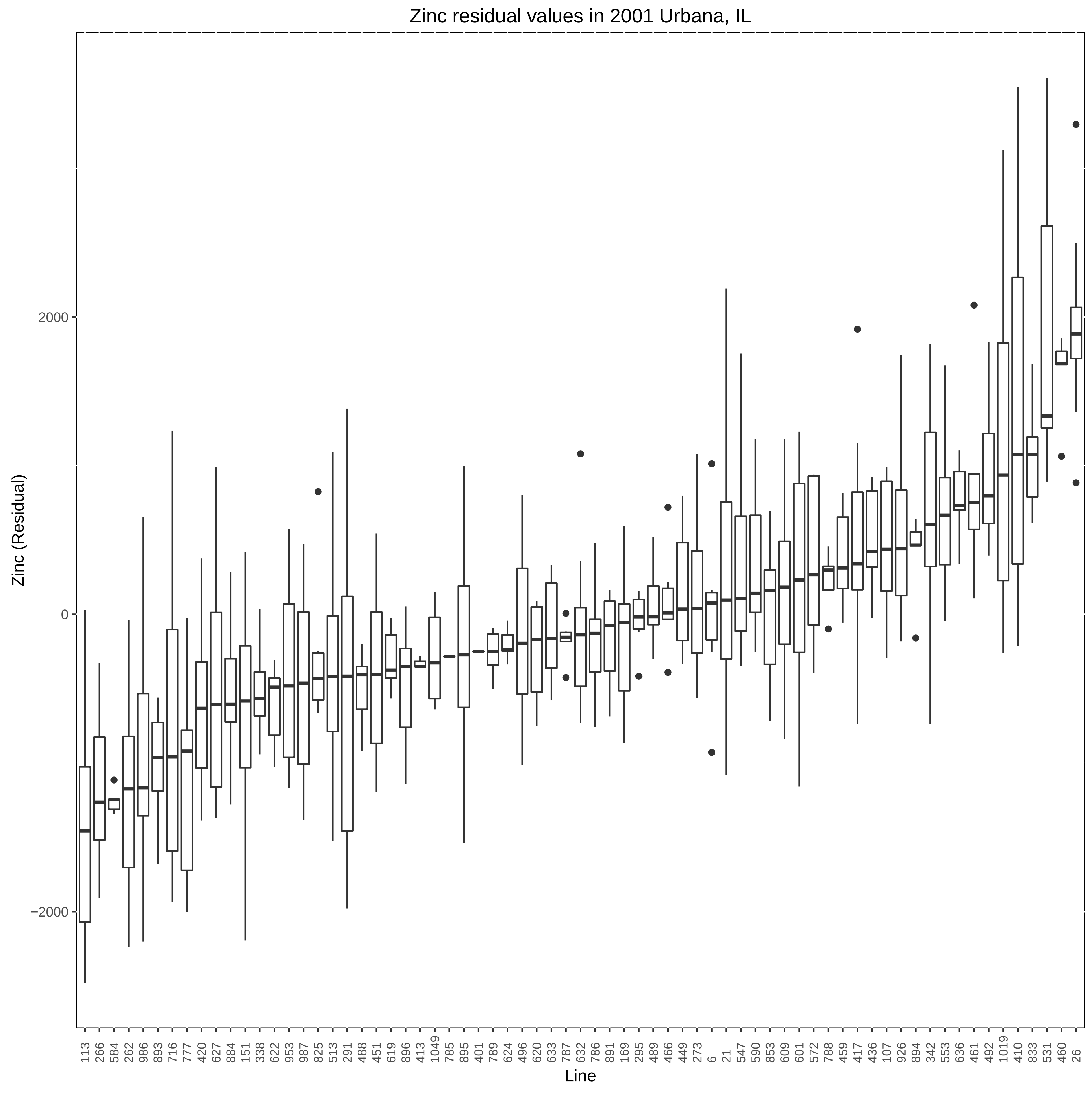
Zinc residual values in 2001 Urbana, IL
Zinc (Residual)
2000
0
-2000
Line

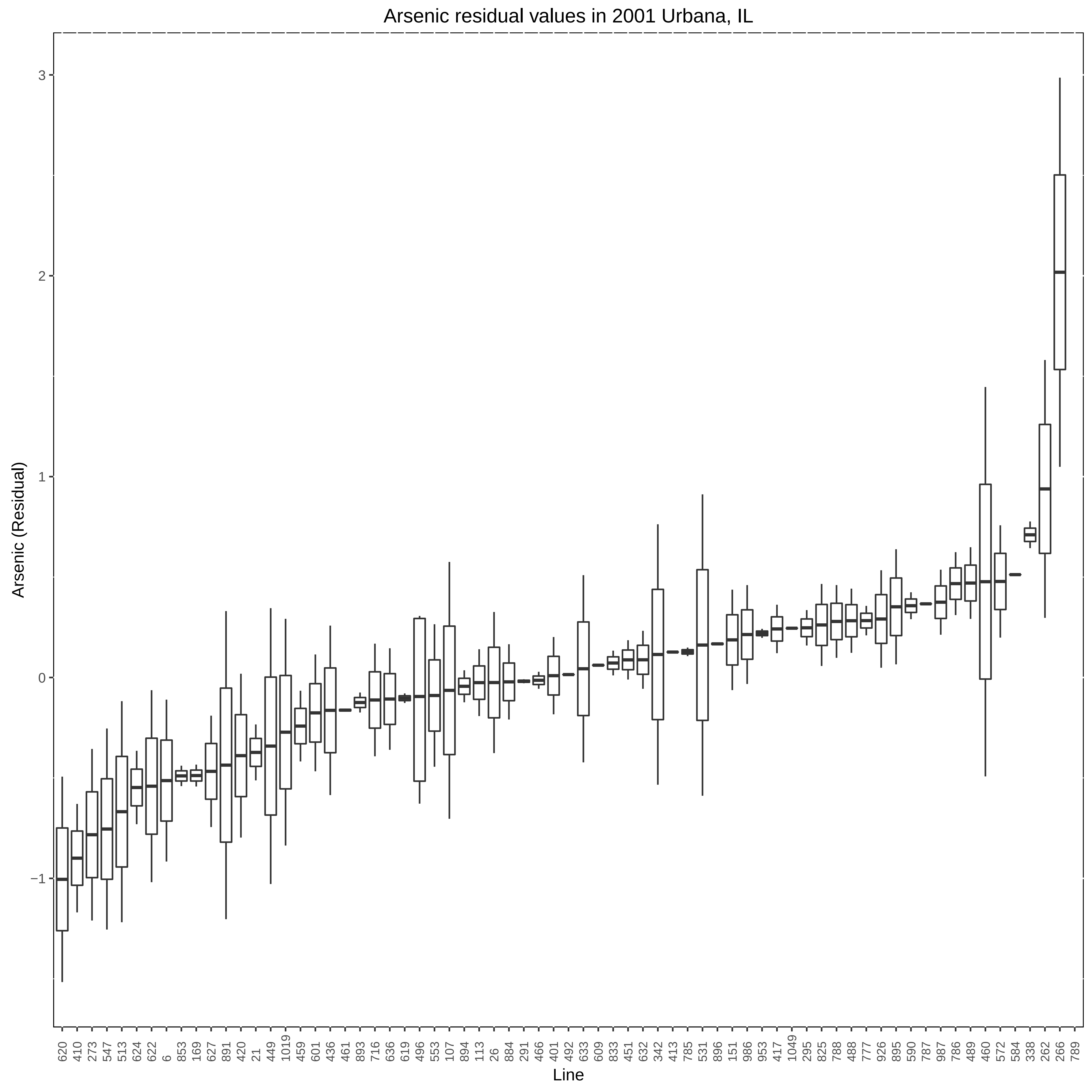
Arsenic residual values in 2001 Urbana, IL
Arsenic (Residual)
3
2
1
0
-1
Line

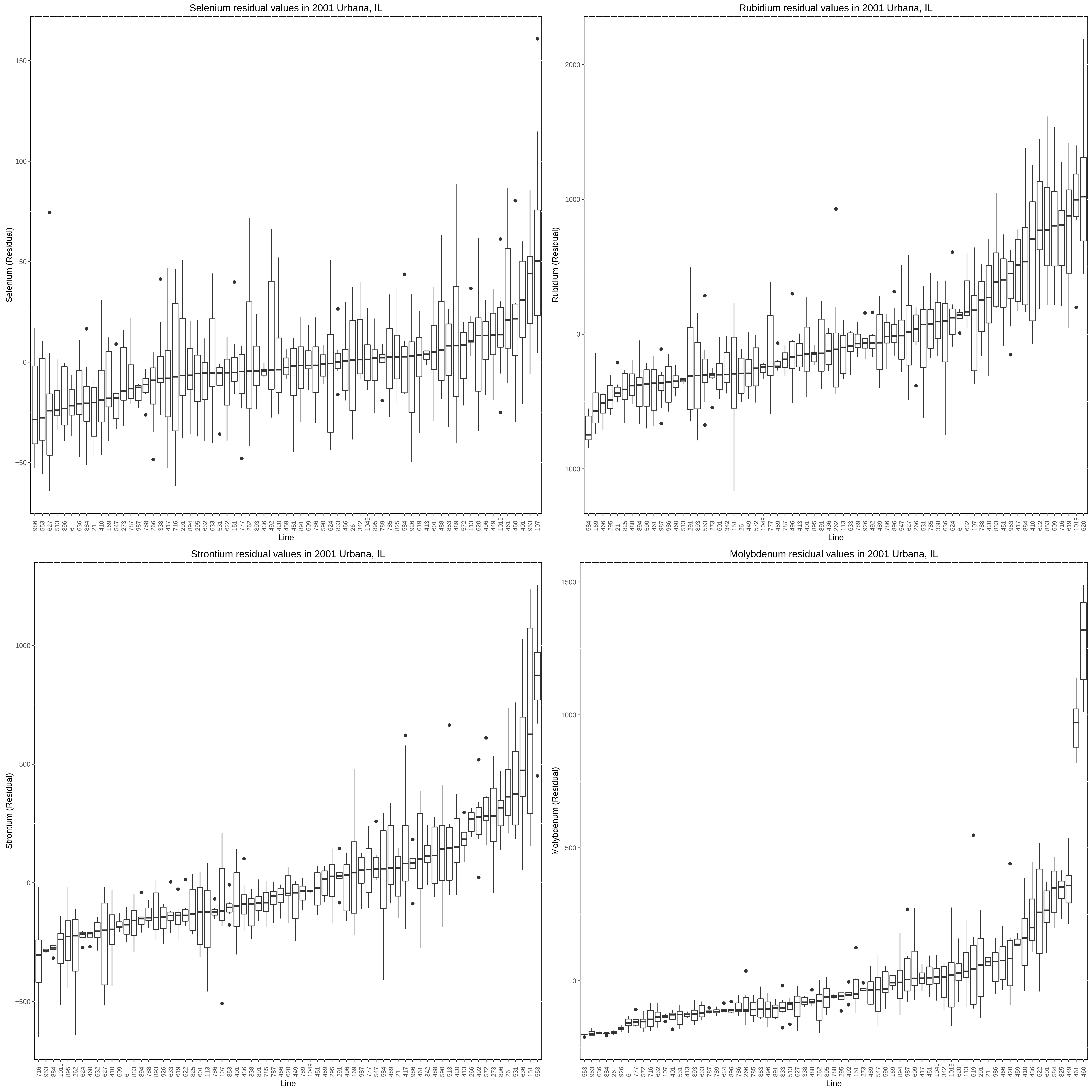
Selenium residual values in 2001 Urbana, IL
Selenium (Residual)
150
100
50
0
-50
Line
Rubidium residual values in 2001 Urbana, IL
Rubidium (Residual)
2000
1000
0
-1000
Line
Strontium residual values in 2001 Urbana, IL
Strontium (Residual)
1000
500
0
-500
Line
Molybdenum residual values in 2001 Urbana, IL
Molybdenum (Residual)
1500
1000
500
0
Line

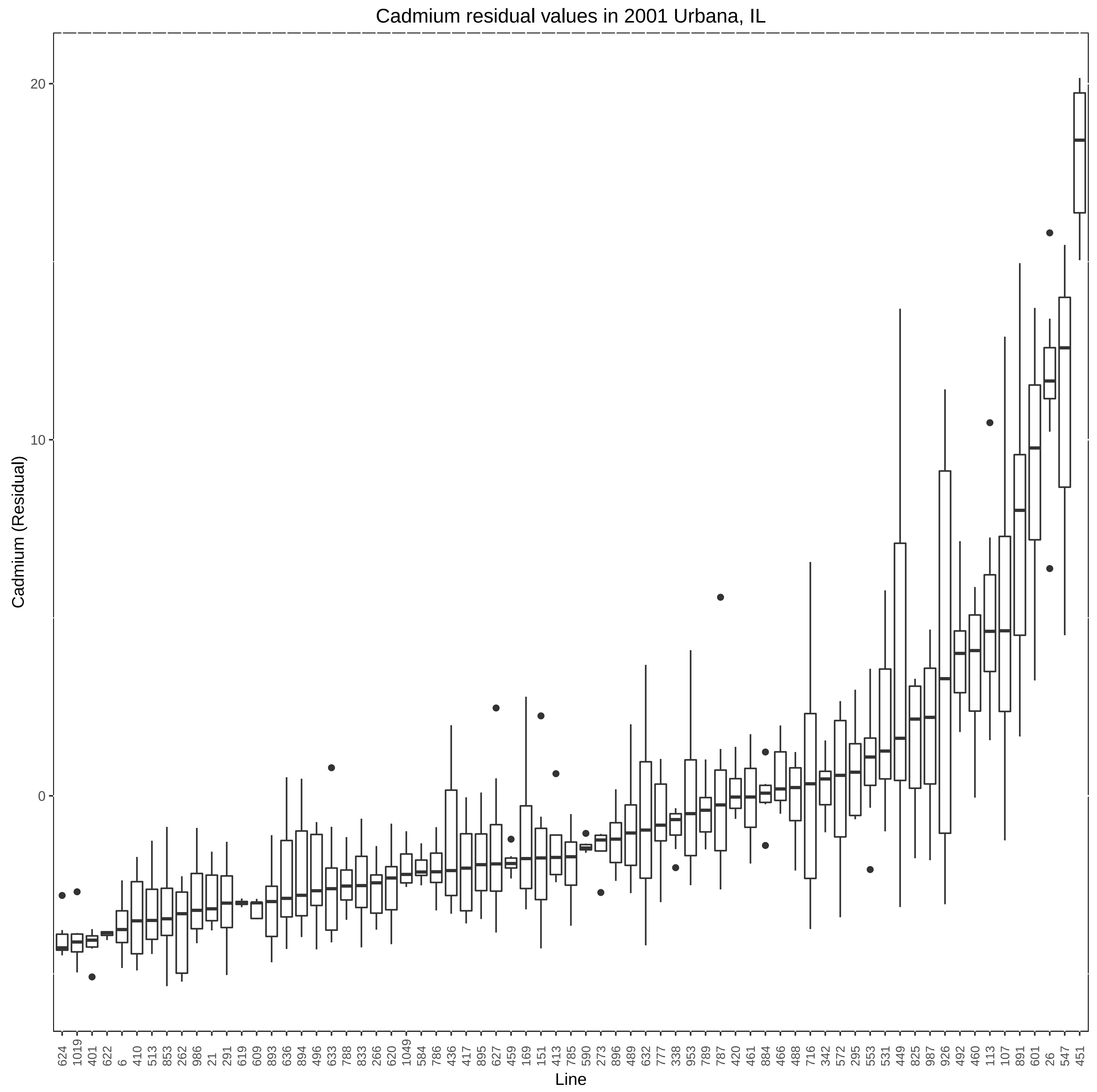
Cadmium residual values in 2001 Urbana, IL
Cadmium (Residual)
20
10
0
Line
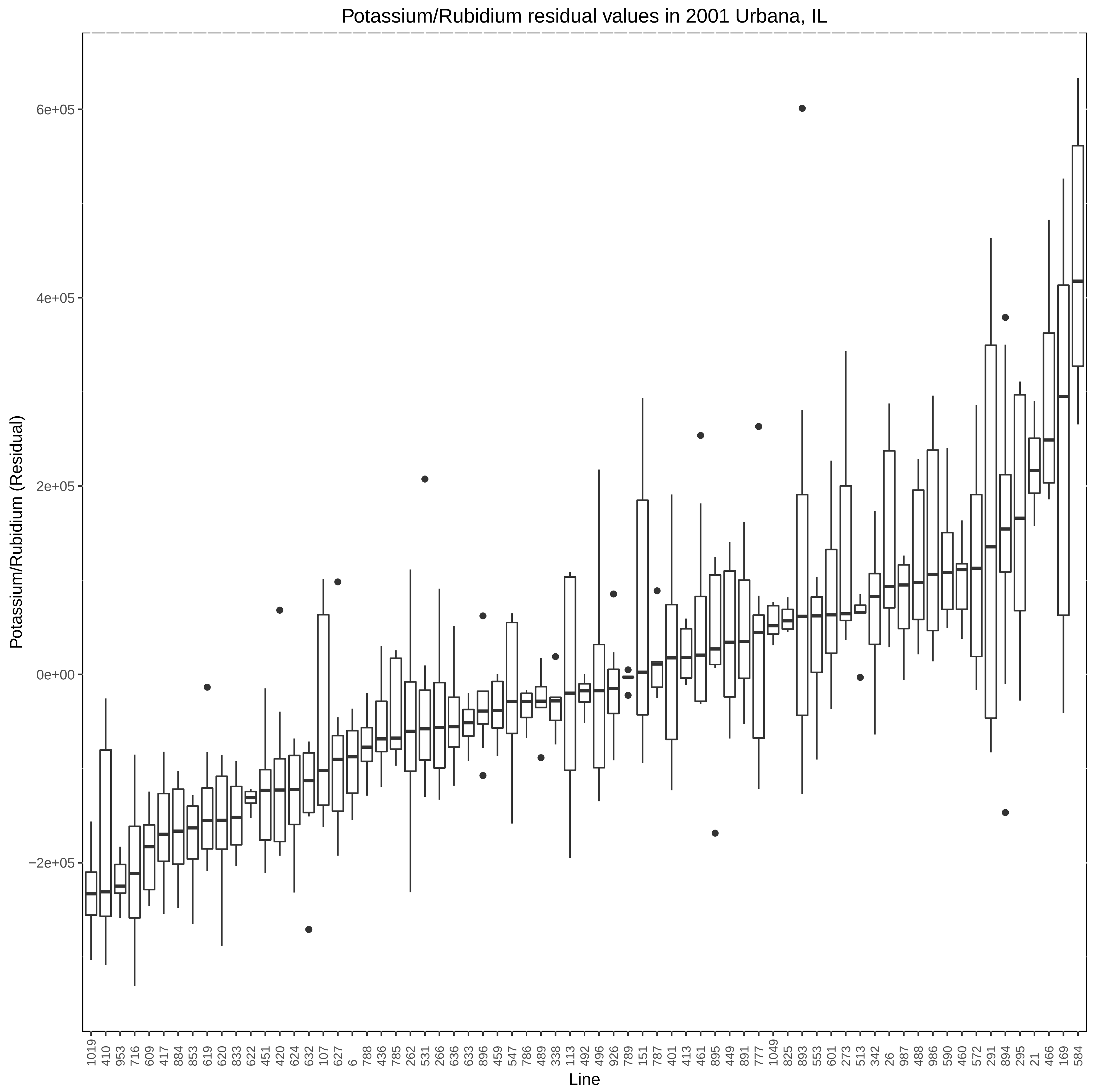
Potassium/Rubidium residual values in 2001 Urbana, IL
Potassium/Rubidium (Residual)
6e+05
4e+05
2e+05
0e+00
−2e+05
Line
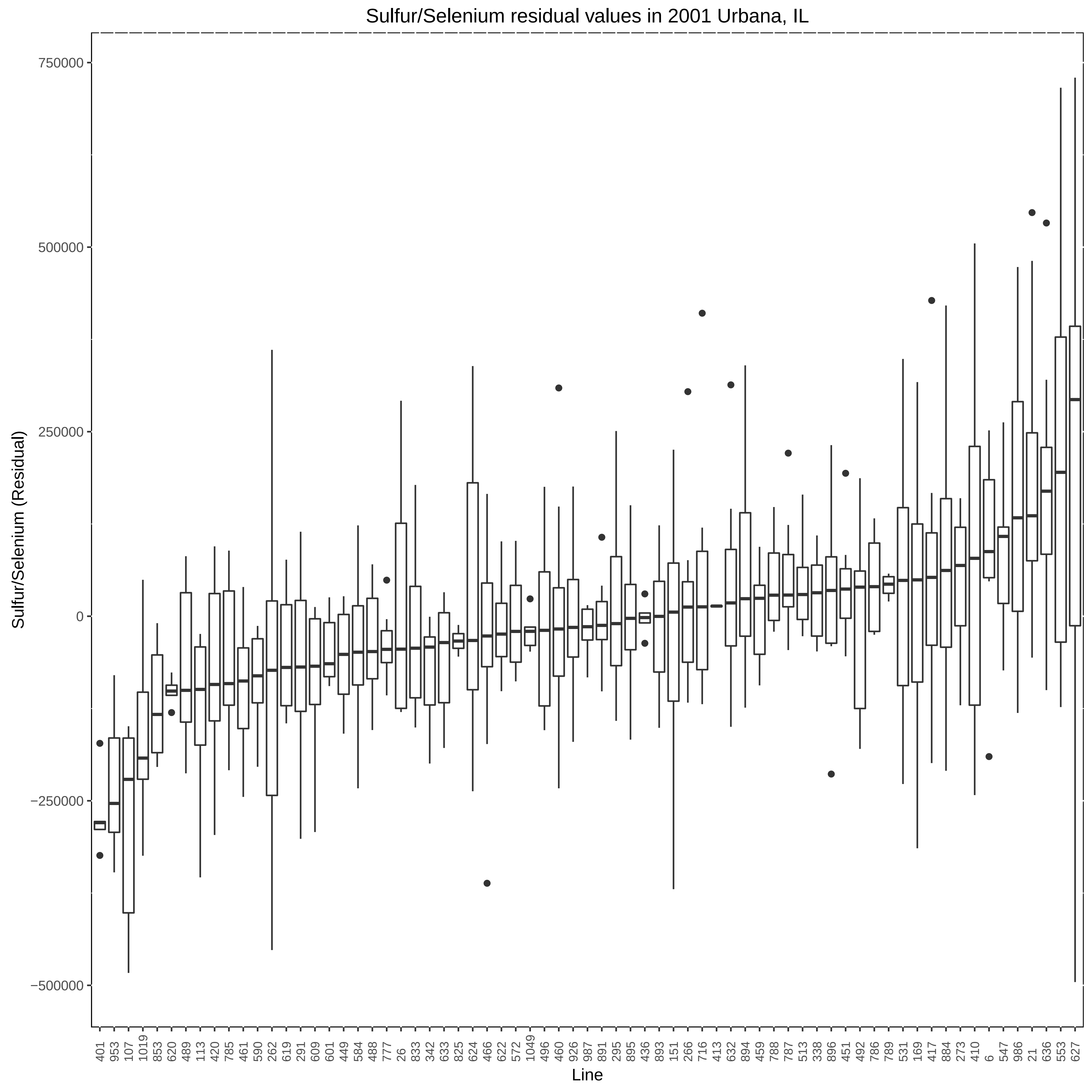
Sulfur/Selenium residual values in 2001 Urbana, IL
Sulfur/Selenium (Residual)
750000
500000
250000
0
−250000
−500000
Line
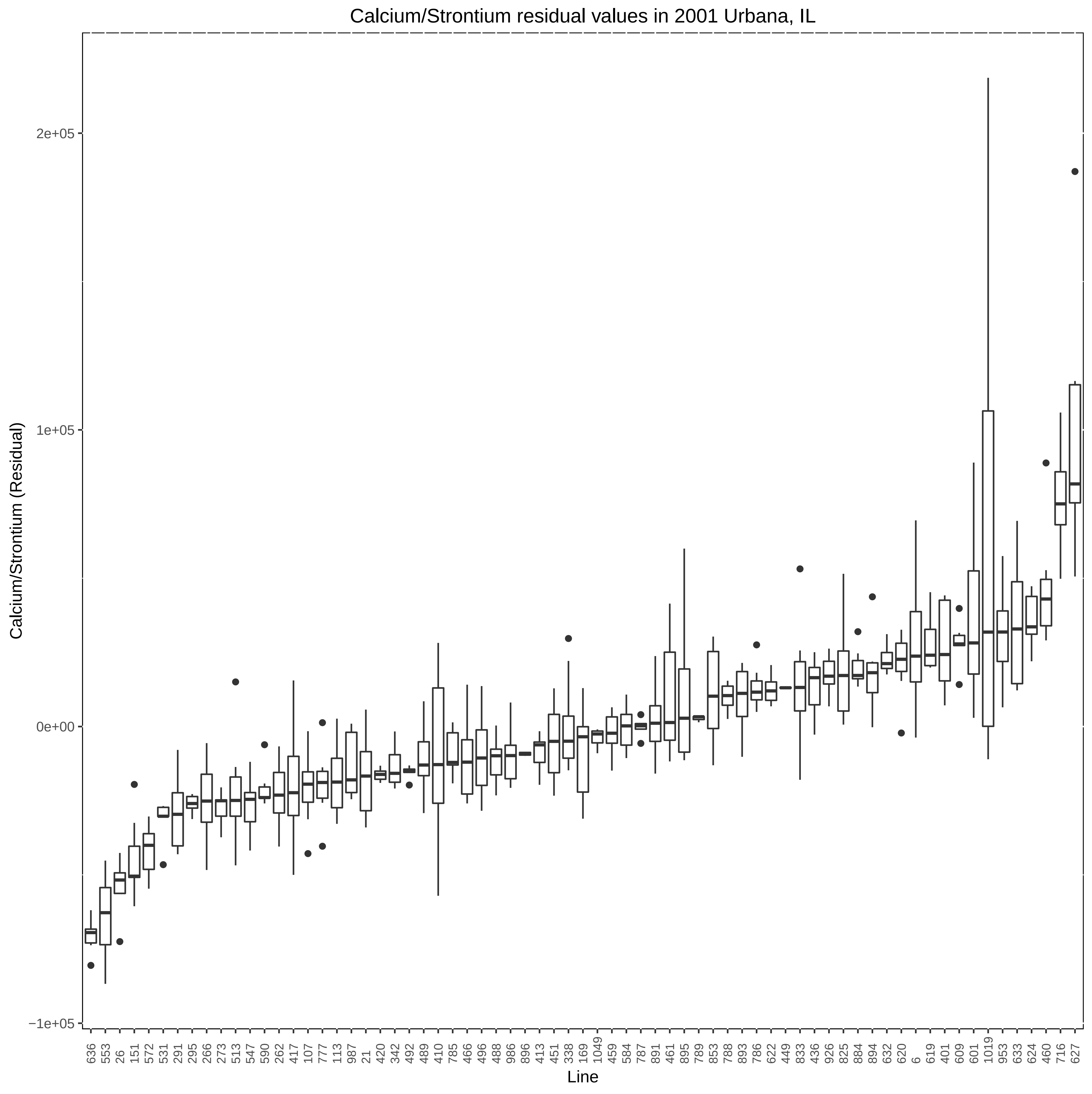
Calcium/Strontium residual values in 2001 Urbana, IL
Calcium/Strontium (Residual)
2e+05
1e+05
0e+00
−1e+05
Line

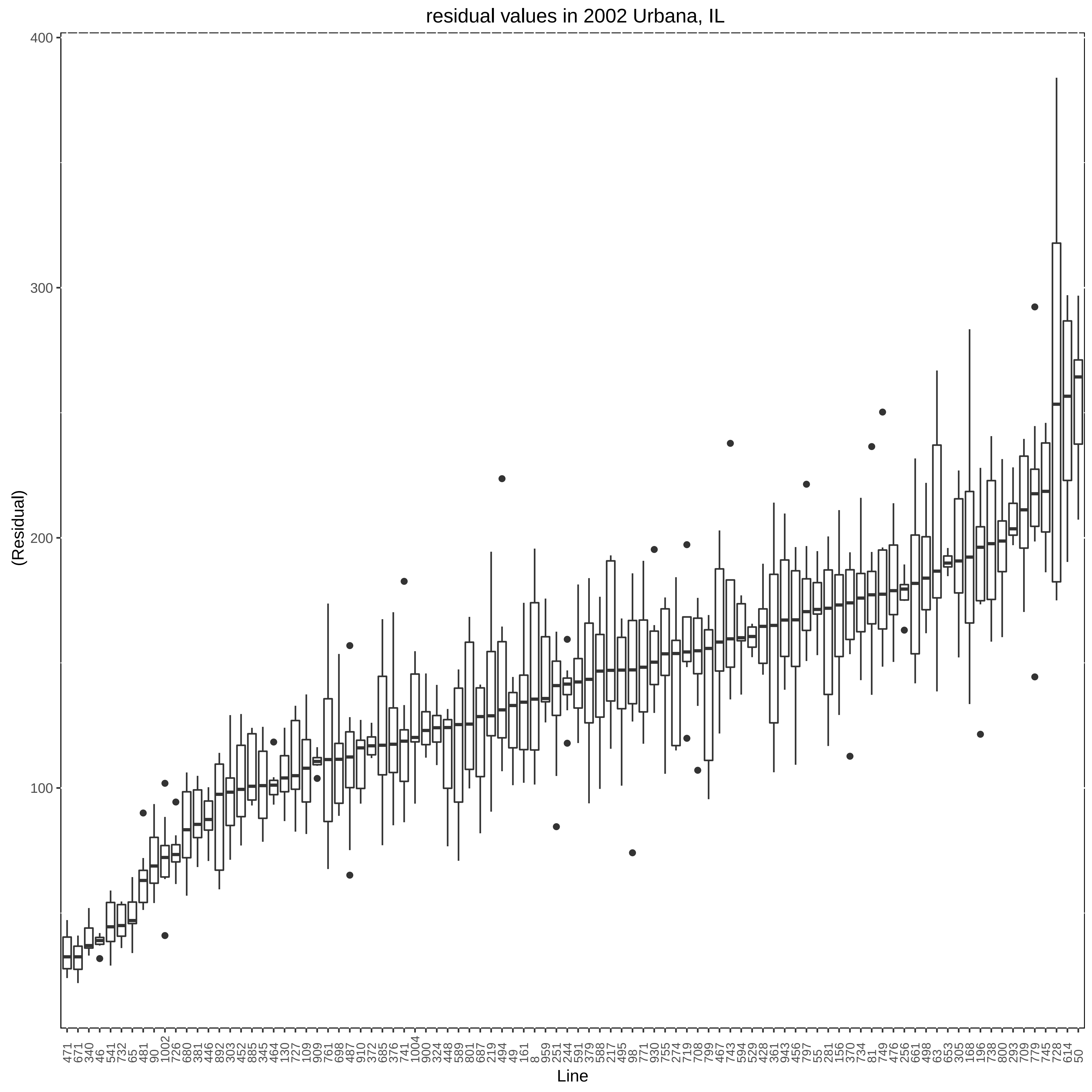

residual values in 2002 Urbana, IL
(Residual)
Line
400
300
200
100

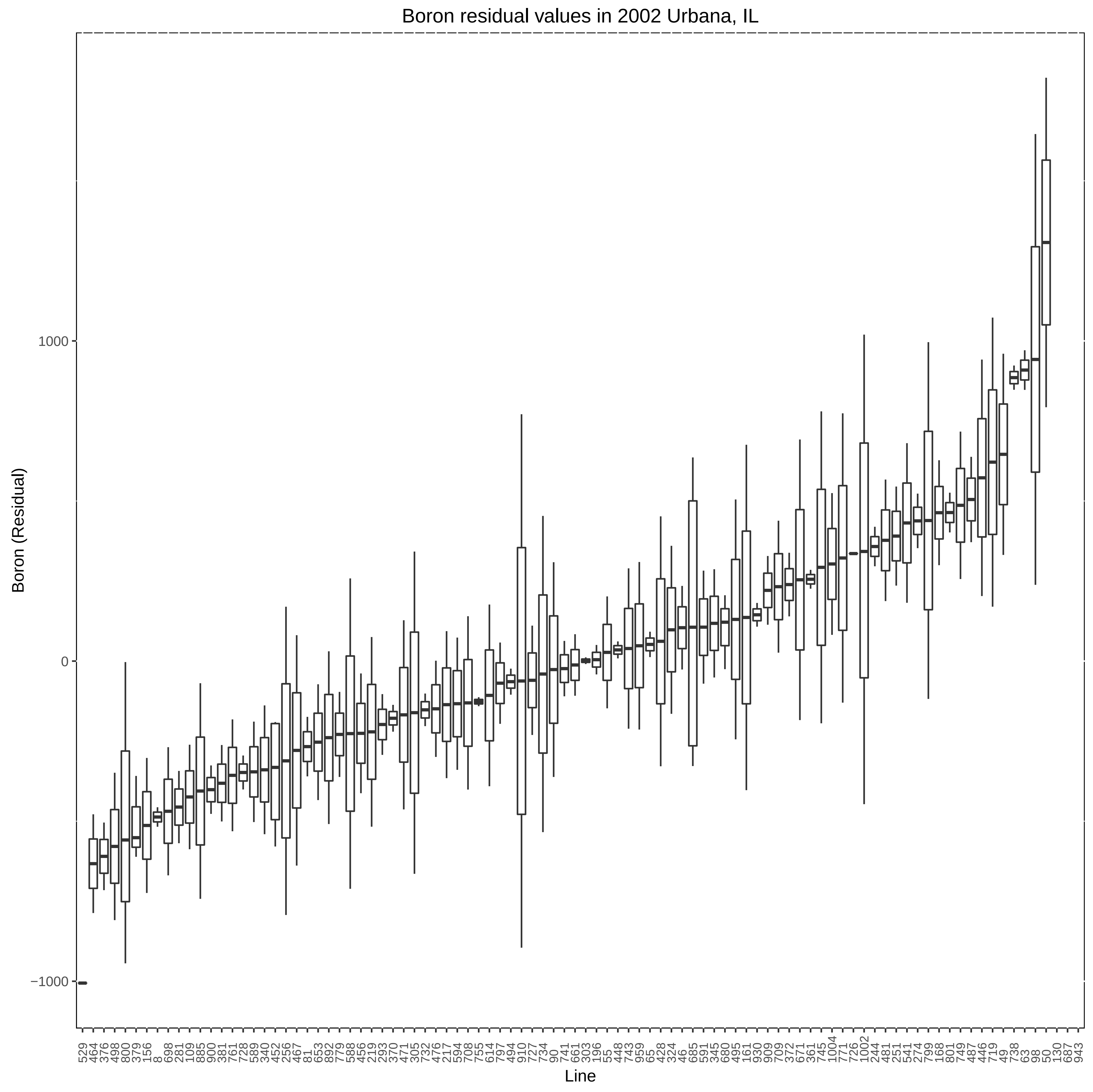

Boron residual values in 2002 Urbana, IL
Boron (Residual)
Line
1000
0
-1000

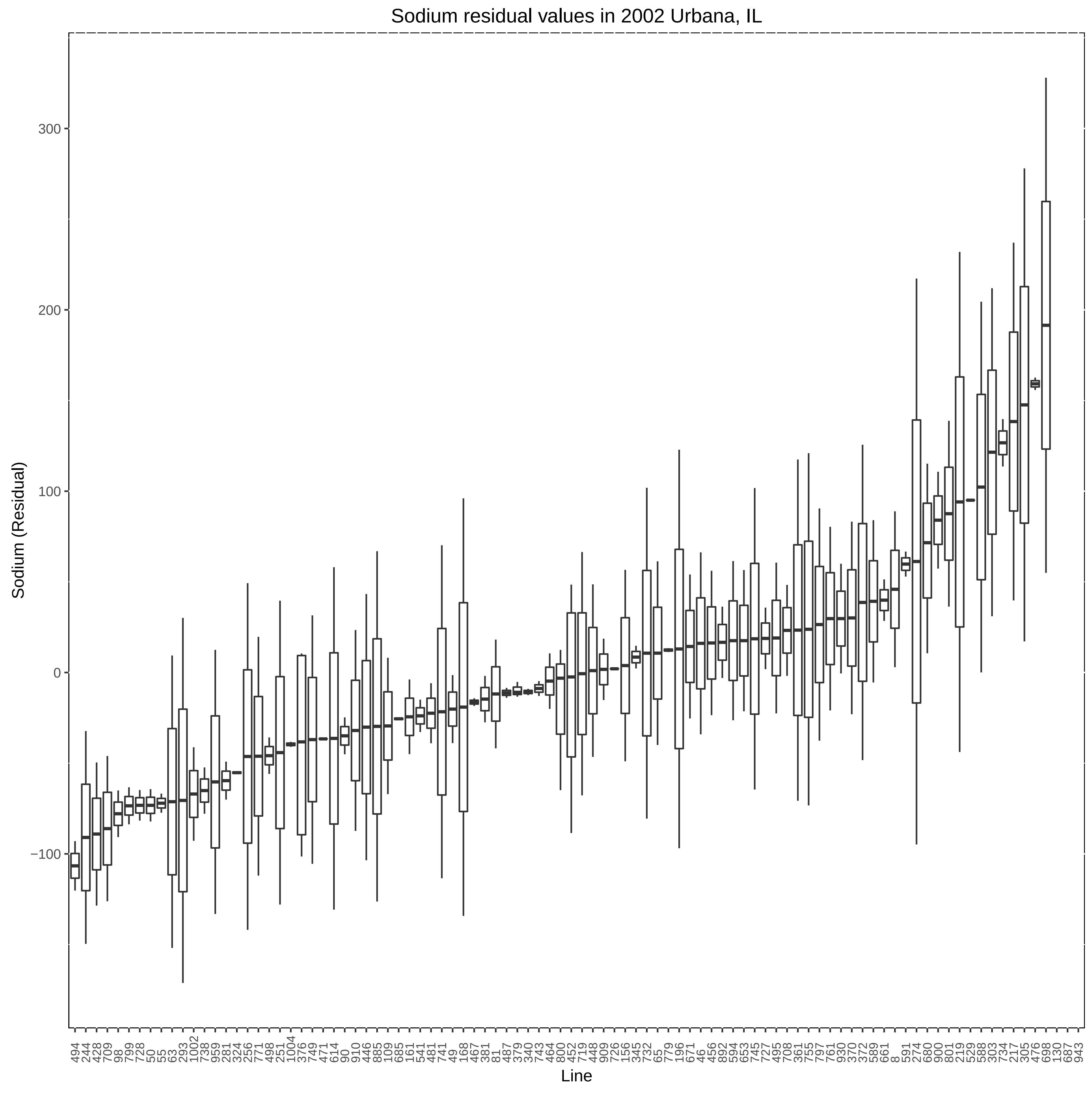

Sodium residual values in 2002 Urbana, IL
Sodium (Residual)
Line
300
200
100
0
-100

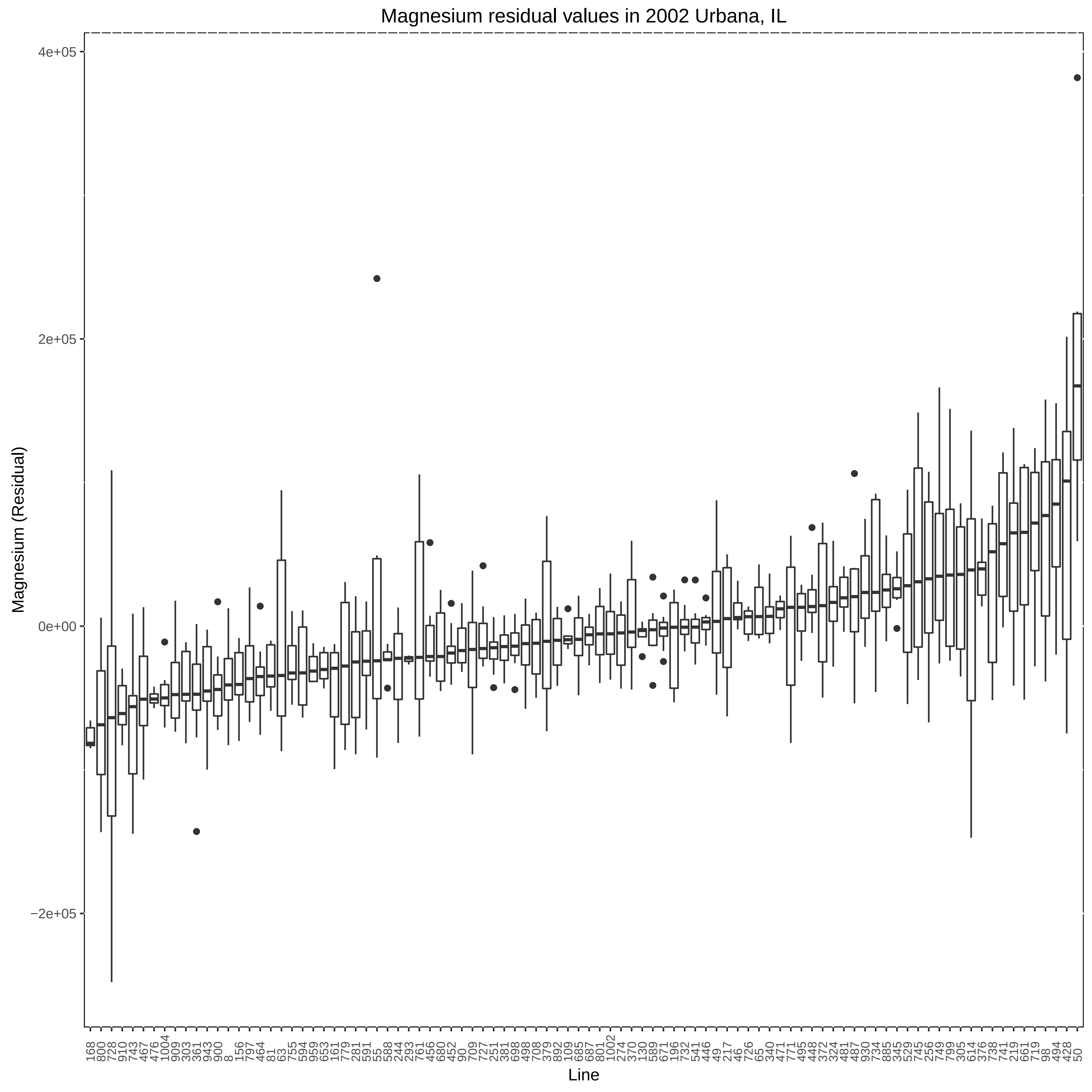

Magnesium residual values in 2002 Urbana, IL
Magnesium (Residual)
Line
4e+05
2e+05
0e+00
-2e+05

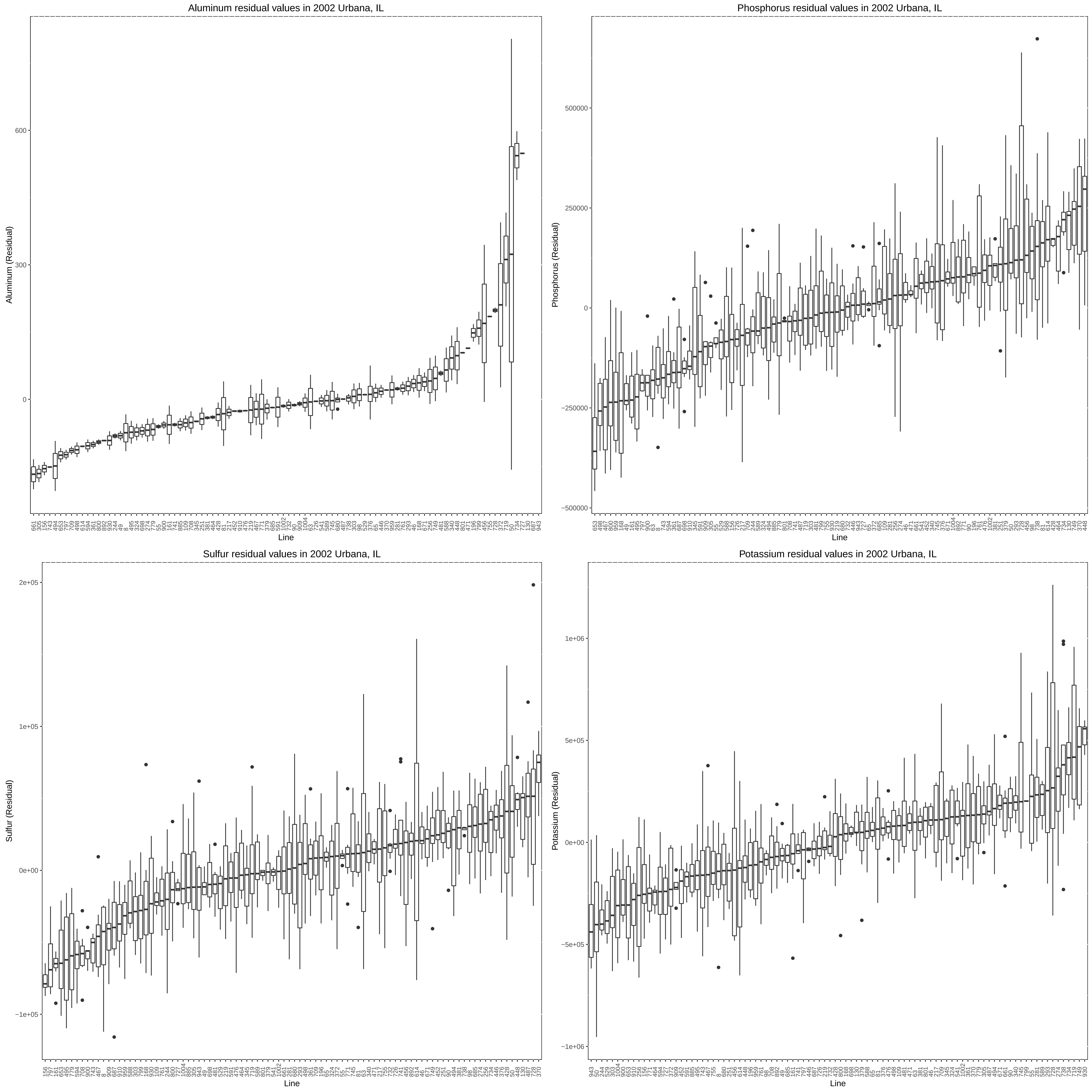

Aluminum residual values in 2002 Urbana, IL
Aluminum (Residual)
600
300
0
Line
Phosphorus residual values in 2002 Urbana, IL
Phosphorus (Residual)
500000
250000
0
-250000
-500000
Line
Sulfur residual values in 2002 Urbana, IL
Sulfur (Residual)
2e+05
1e+05
0e+00
-1e+05
Line
Potassium residual values in 2002 Urbana, IL
Potassium (Residual)
1e+06
5e+05
0e+00
-5e+05
-1e+06
Line

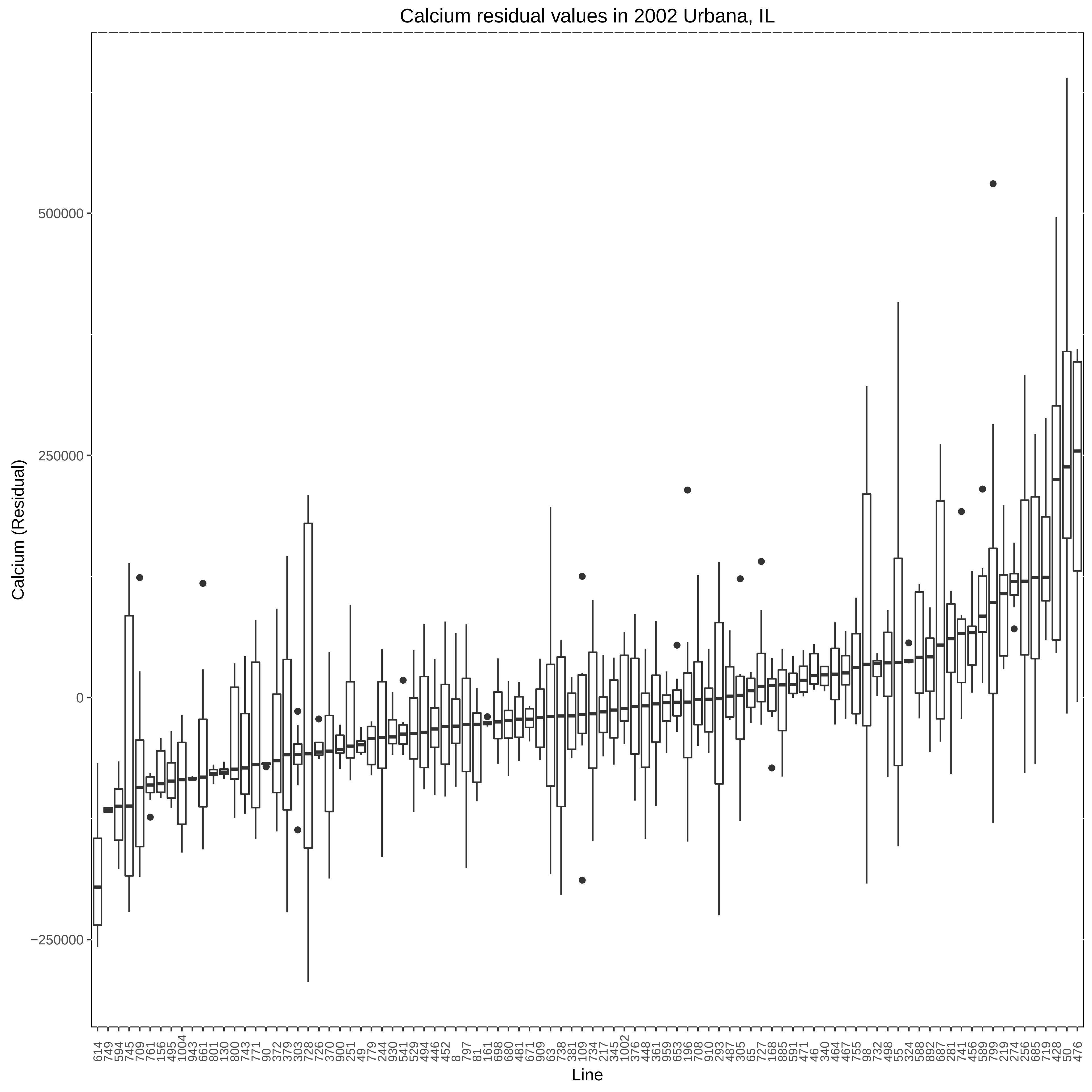
Calcium residual values in 2002 Urbana, IL
Calcium (Residual)
500000
250000
0
-250000
Line

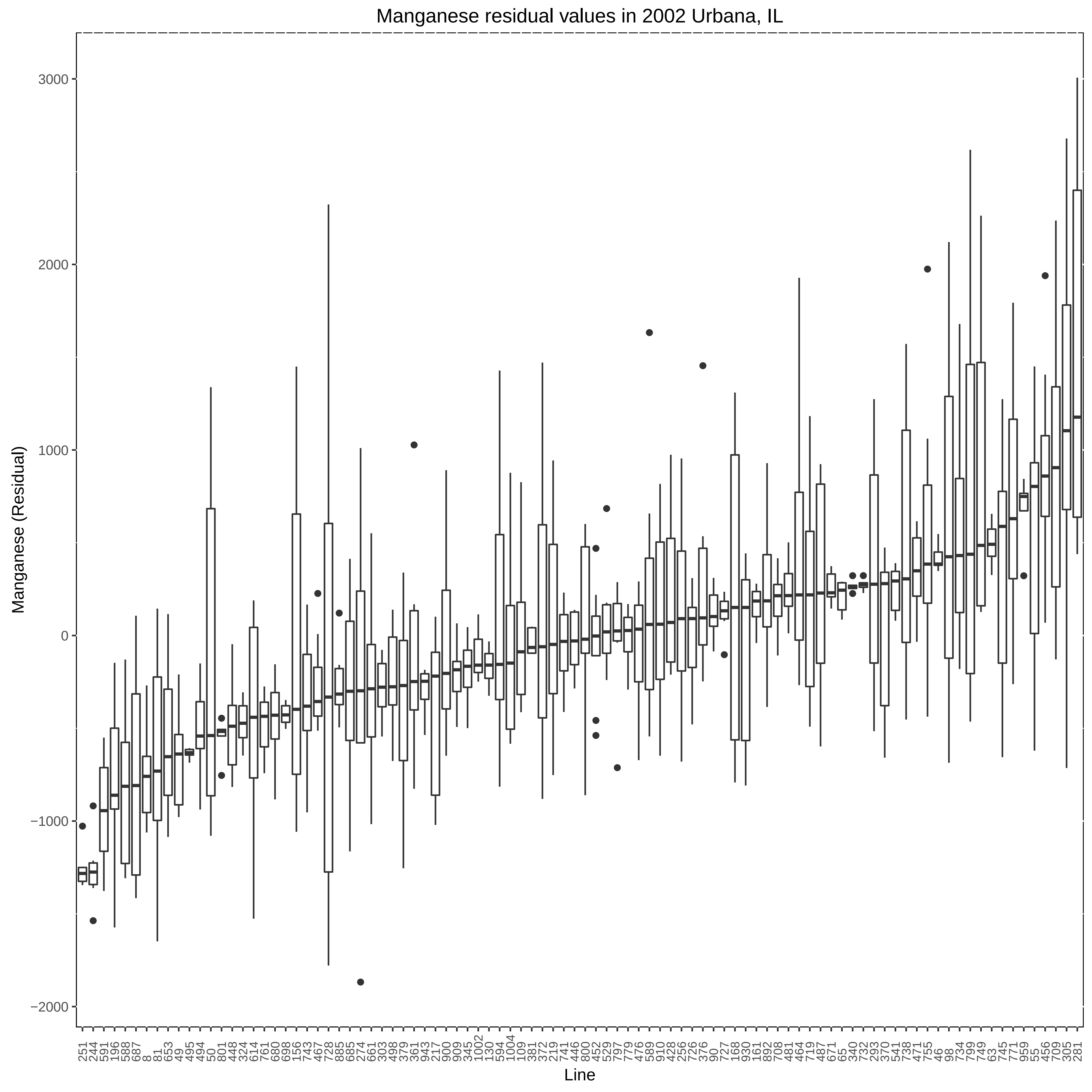
Manganese residual values in 2002 Urbana, IL
Manganese (Residual)
3000
2000
1000
0
-1000
-2000
Line

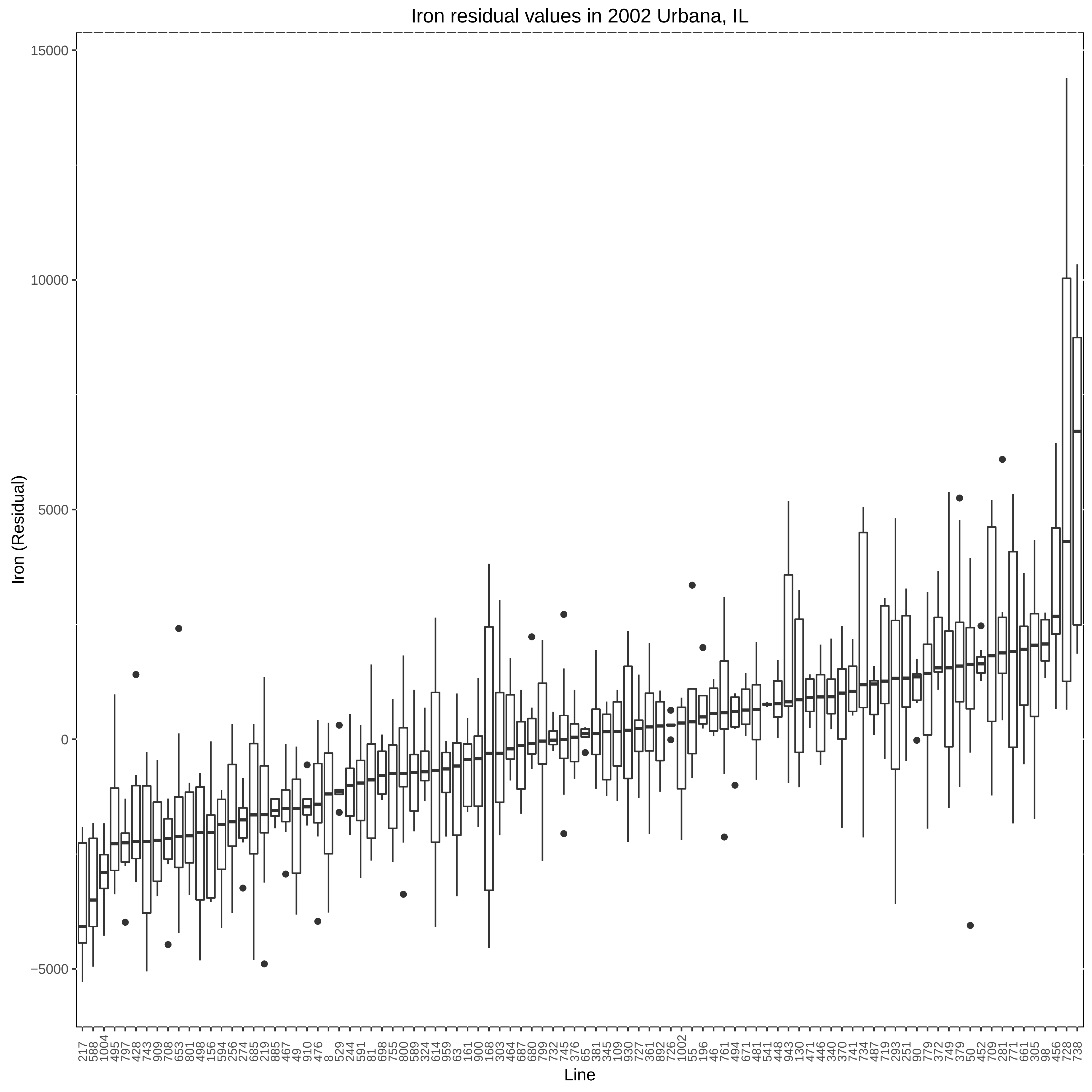
Iron residual values in 2002 Urbana, IL
Iron (Residual)
15000
10000
5000
0
-5000
Line

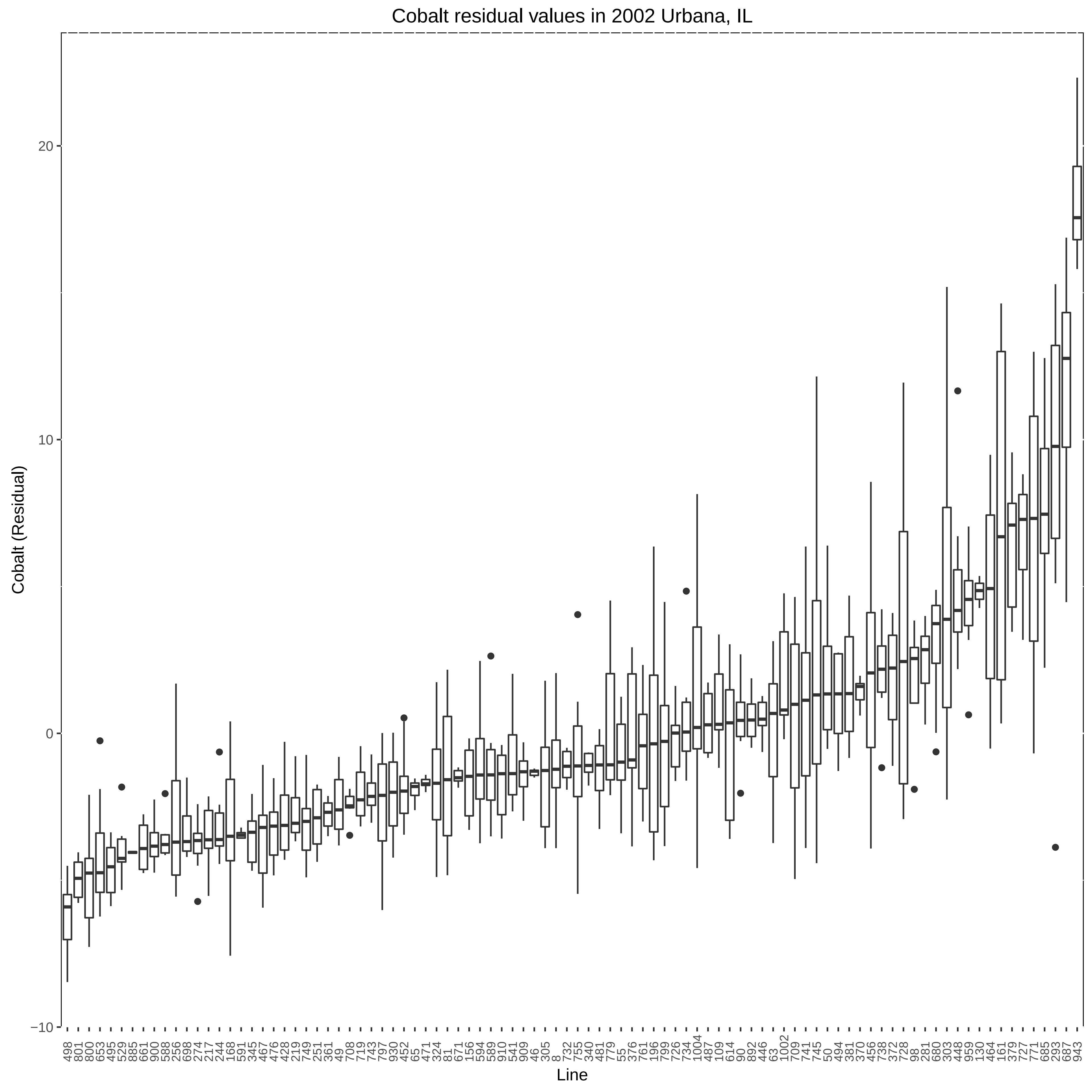
Cobalt residual values in 2002 Urbana, IL
Cobalt (Residual)
20
10
0
-10
Line

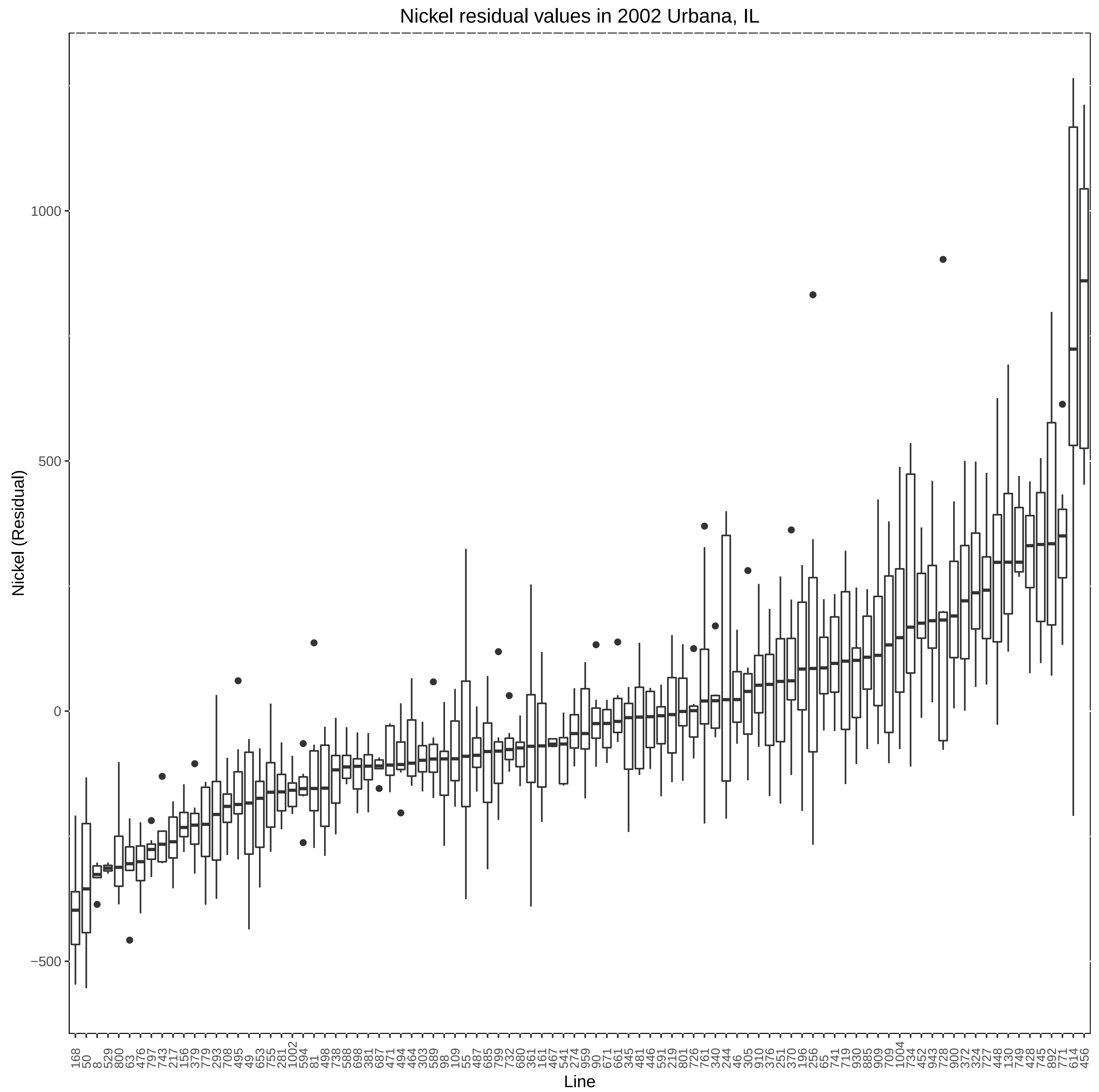
Nickel residual values in 2002 Urbana, IL
Nickel (Residual)
1000
500
0
-500
Line

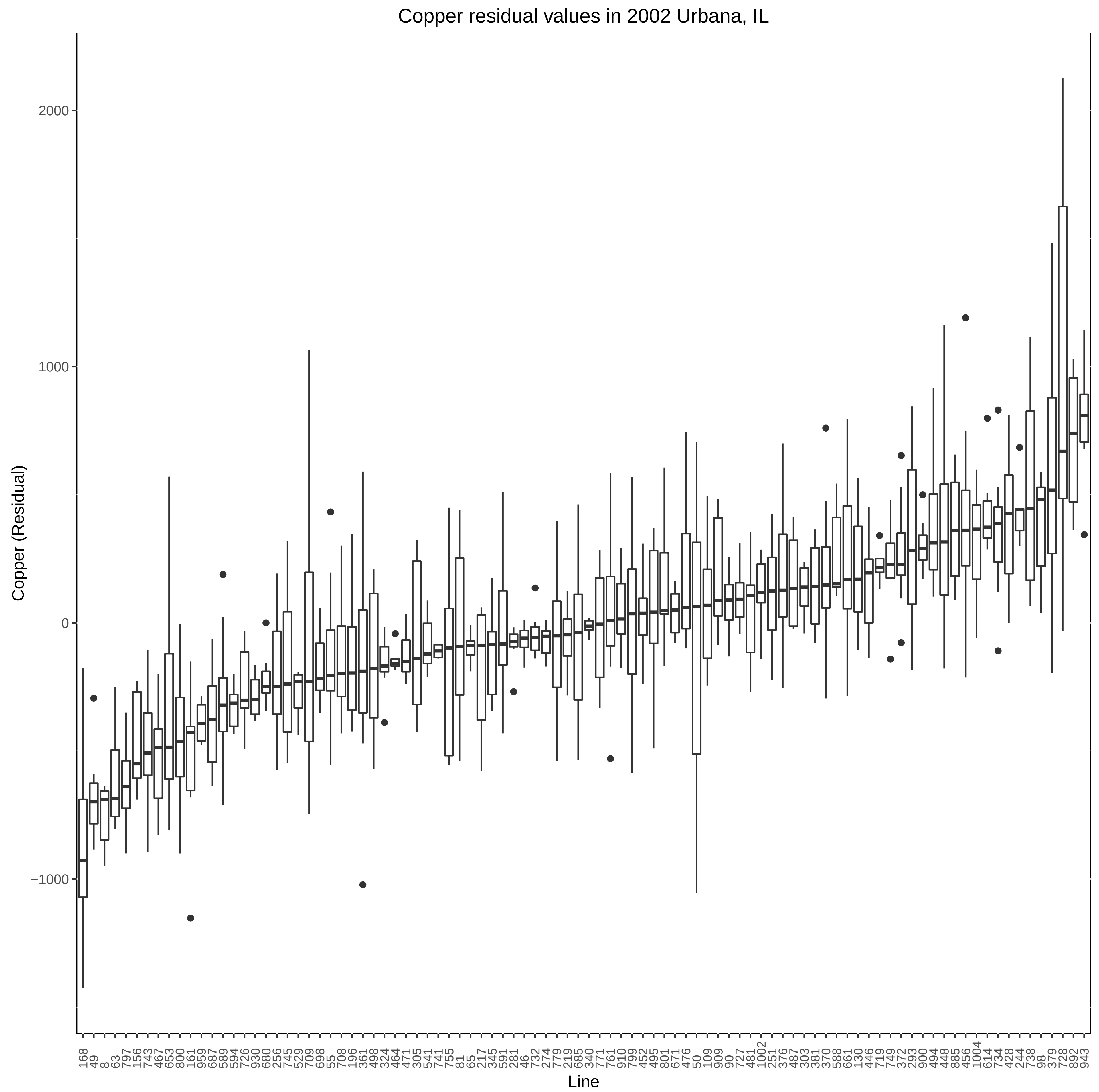
Copper residual values in 2002 Urbana, IL
Copper (Residual)
2000
1000
0
-1000
Line

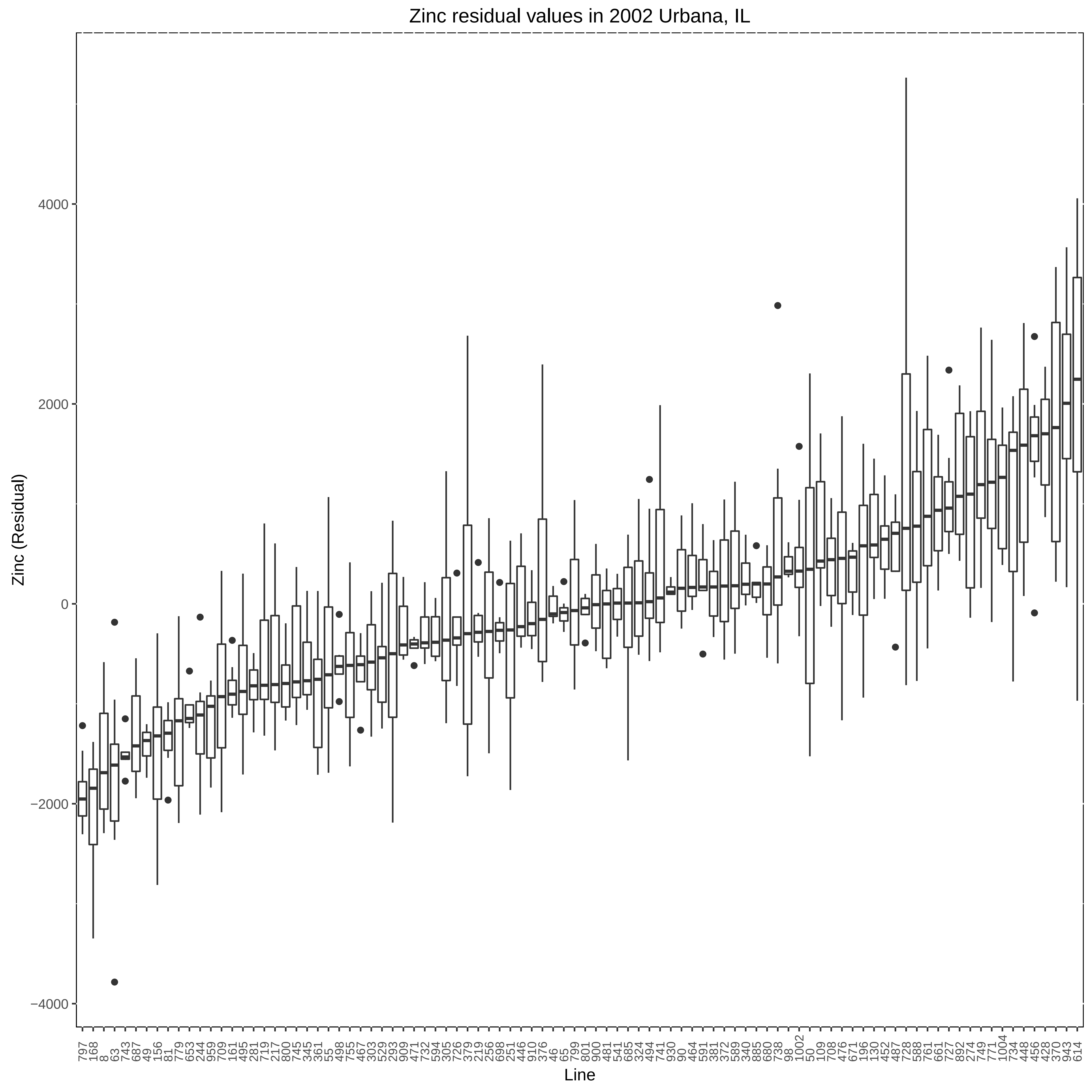
Zinc residual values in 2002 Urbana, IL
Zinc (Residual)
4000
2000
0
-2000
-4000
Line

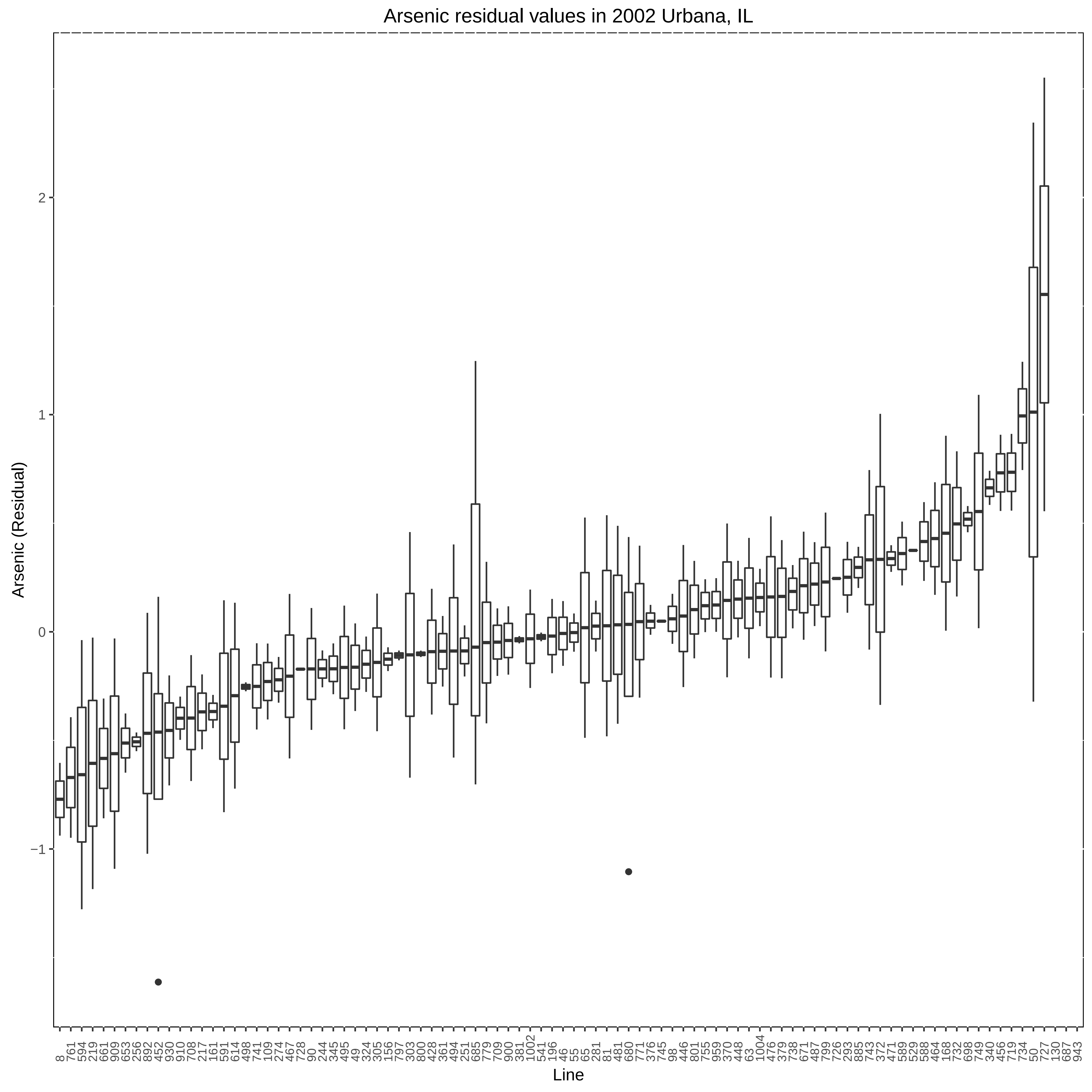
Arsenic residual values in 2002 Urbana, IL
Arsenic (Residual)
2
1
0
-1
Line

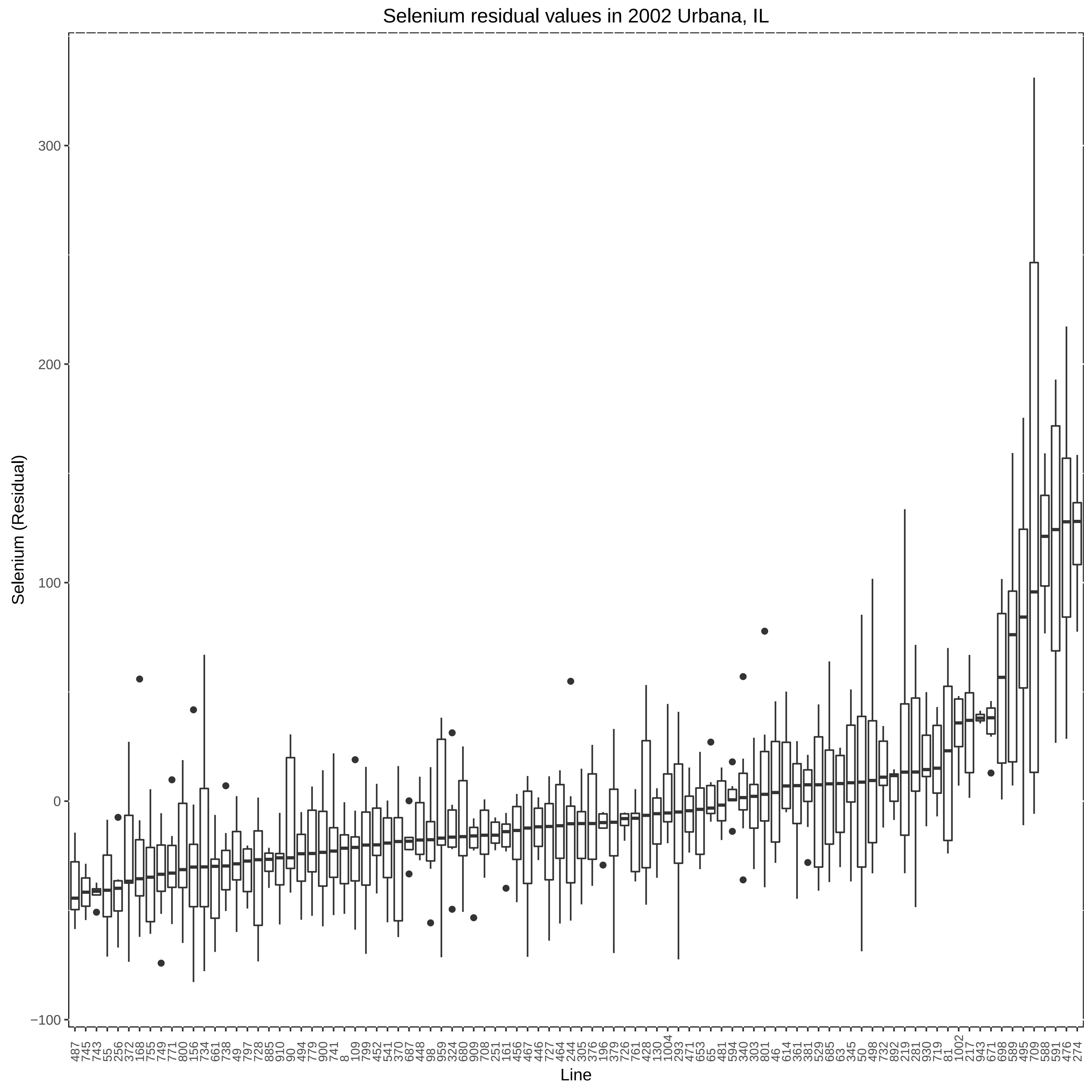
Selenium residual values in 2002 Urbana, IL
Selenium (Residual)
300
200
100
0
-100
Line

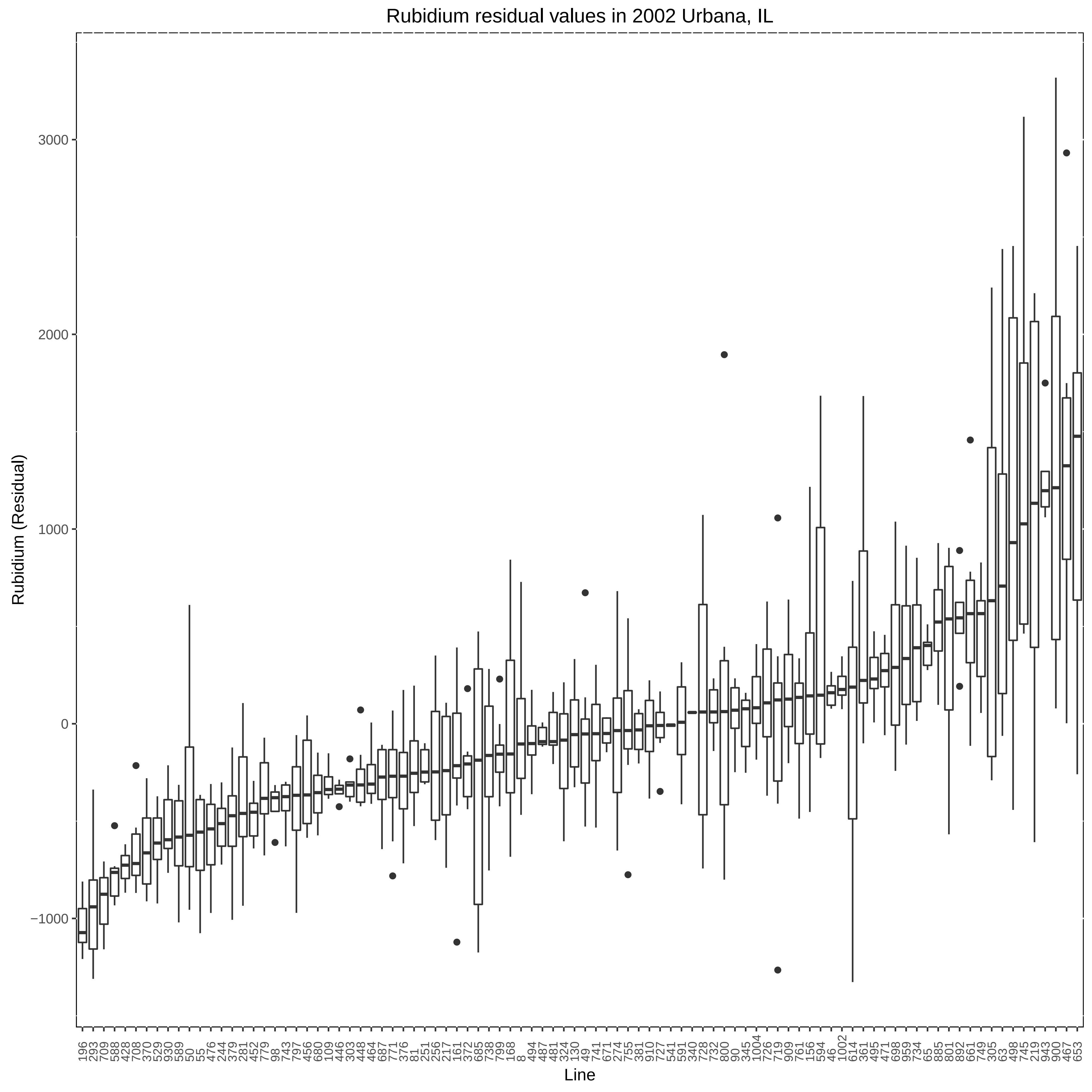
Rubidium residual values in 2002 Urbana, IL
Rubidium (Residual)
3000
2000
1000
0
-1000
Line

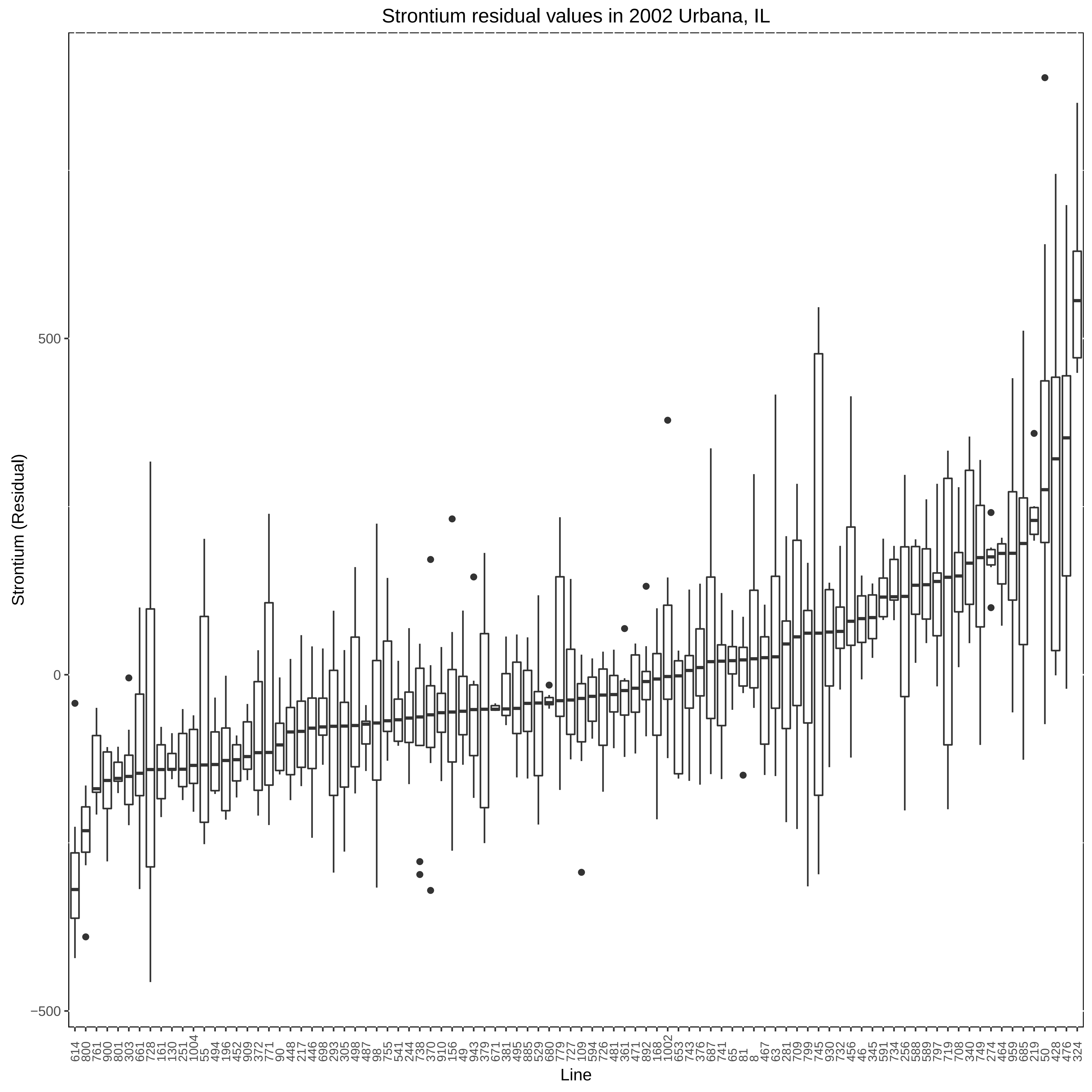
Strontium residual values in 2002 Urbana, IL
Strontium (Residual)
500
0
-500
Line

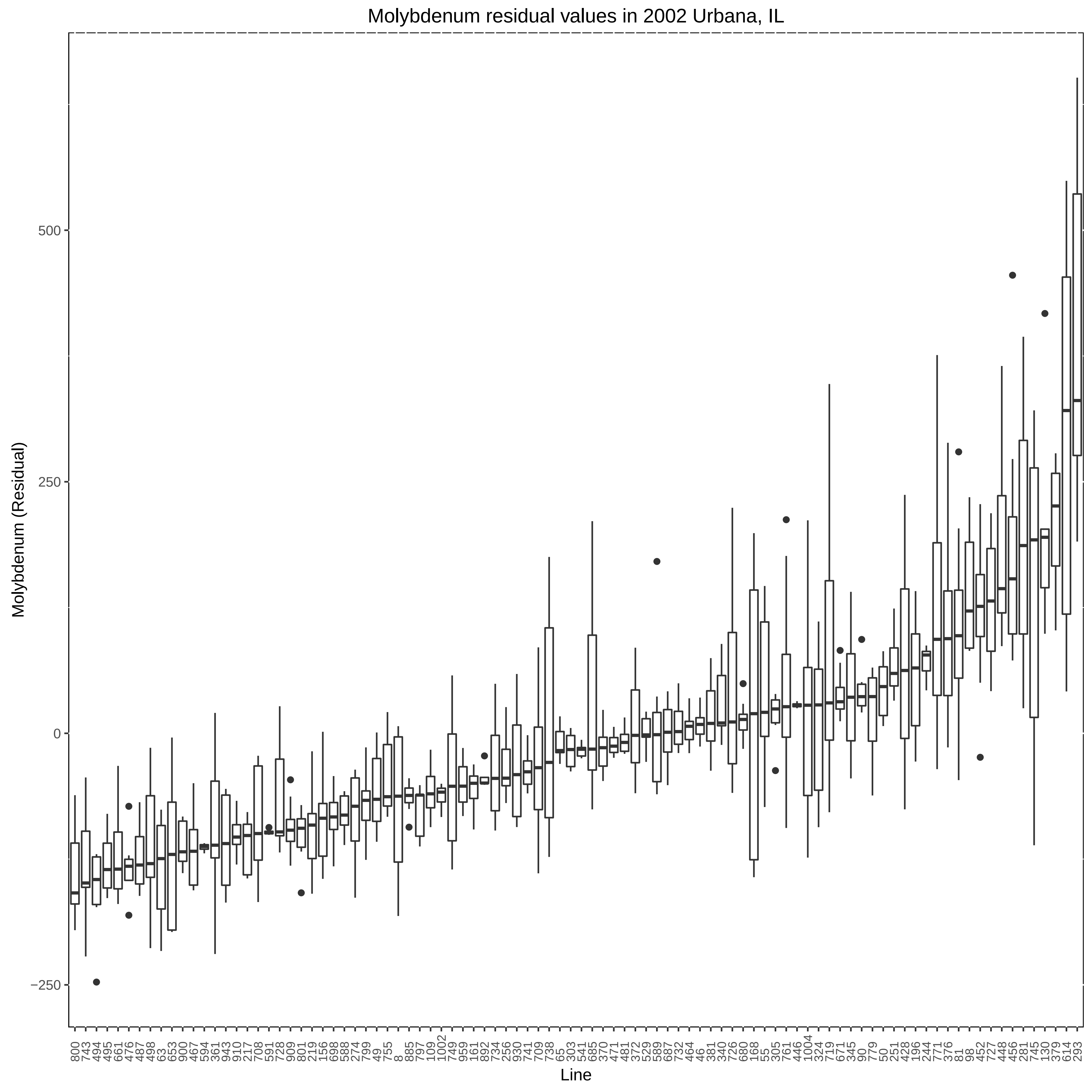
Molybdenum residual values in 2002 Urbana, IL
Molybdenum (Residual)
500
250
0
-250
Line

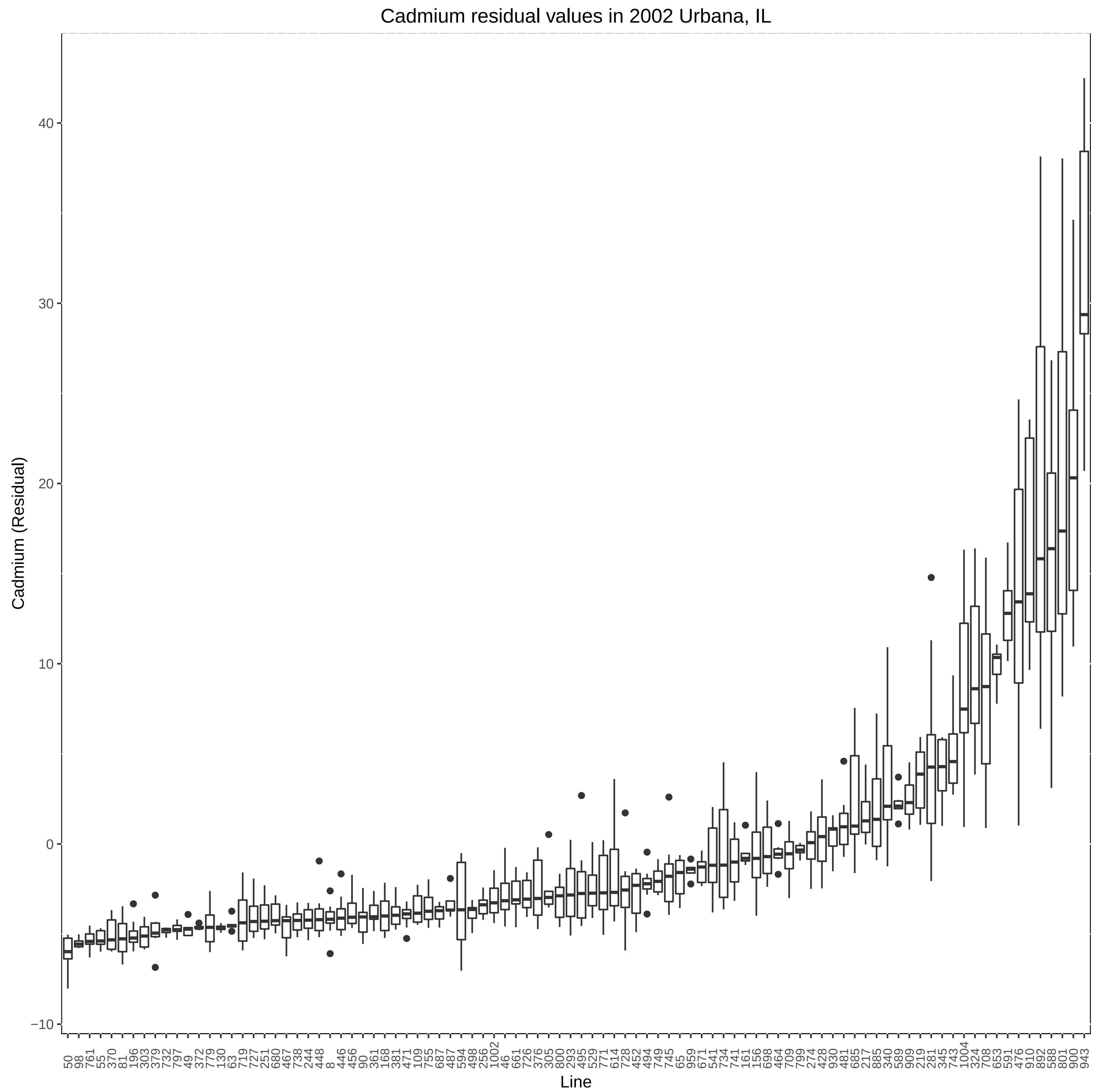
Cadmium residual values in 2002 Urbana, IL
Cadmium (Residual)
40
30
20
10
0
-10
Line

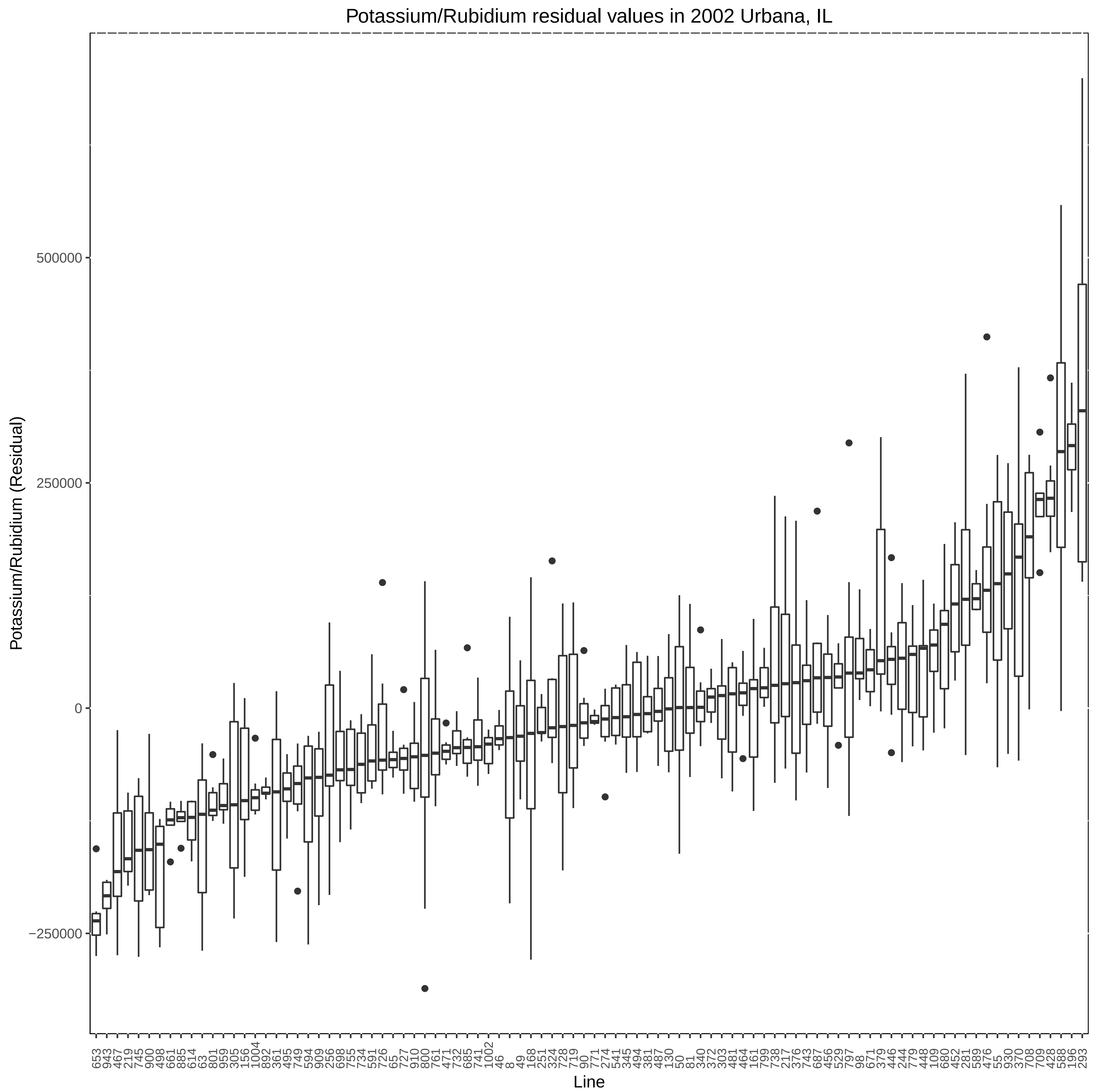
Potassium/Rubidium residual values in 2002 Urbana, IL
Potassium/Rubidium (Residual)
500000
250000
0
-250000
Line

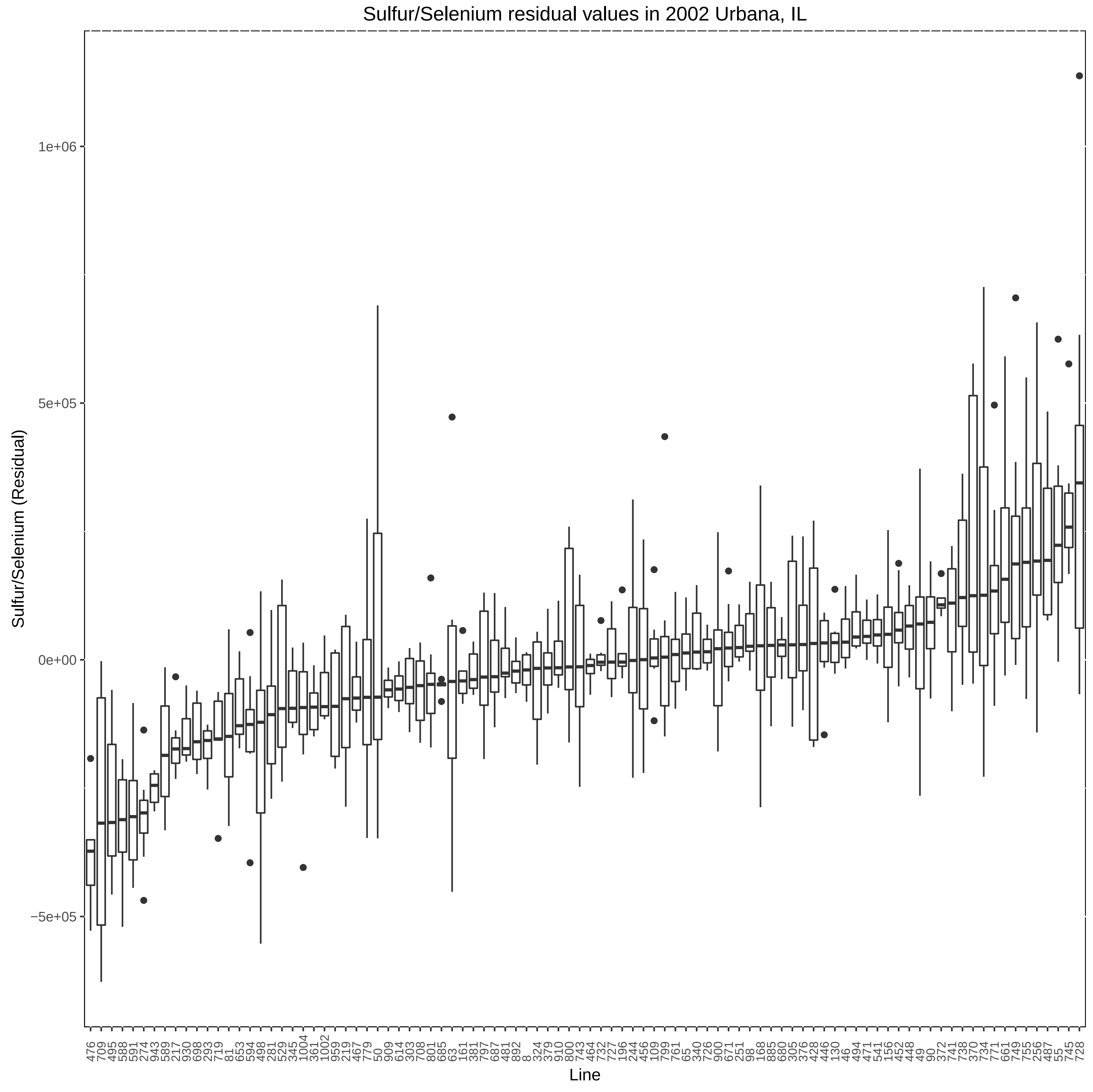
Sulfur/Selenium residual values in 2002 Urbana, IL
Sulfur/Selenium (Residual)
1e+06
5e+05
0e+00
-5e+05
Line

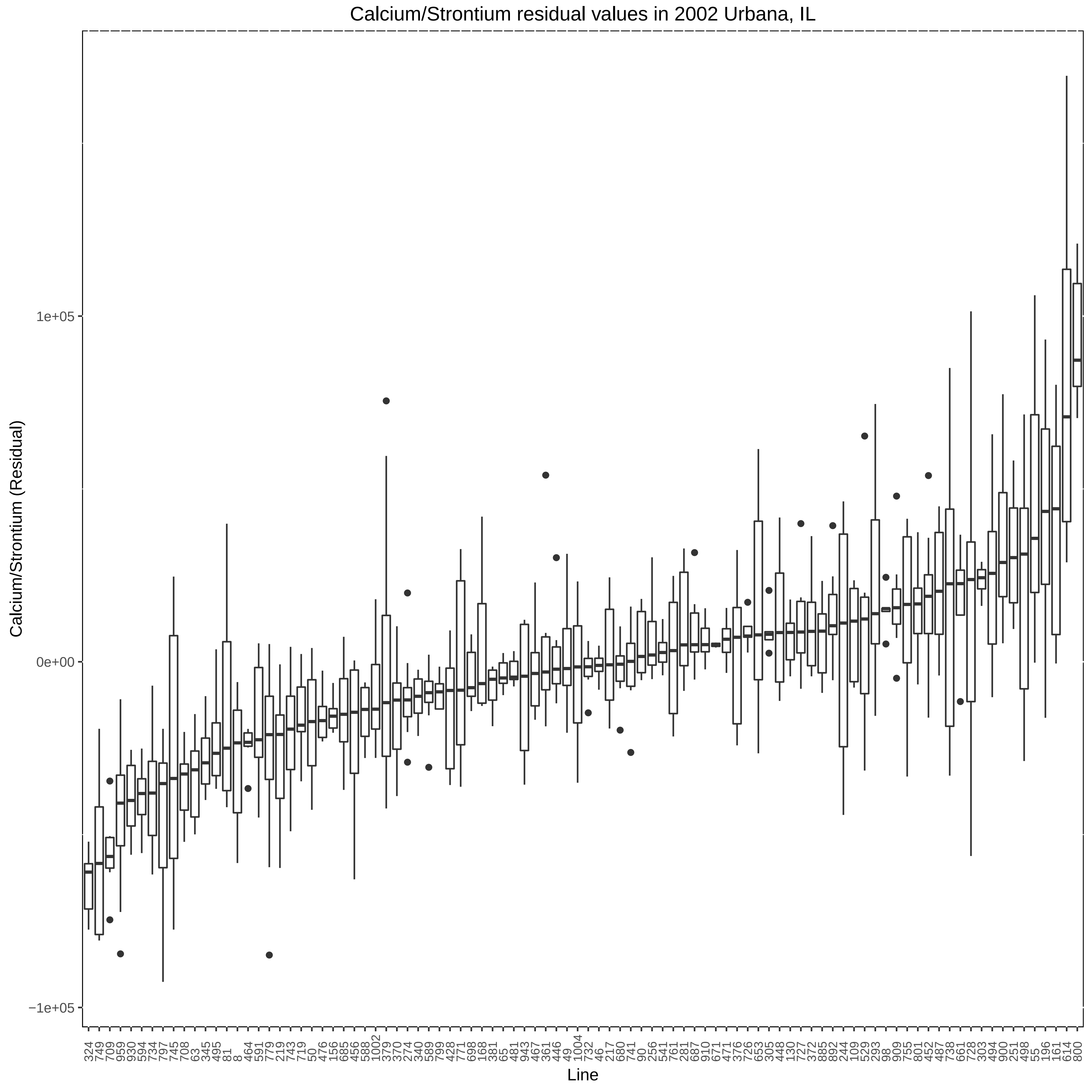
Calcium/Strontium residual values in 2002 Urbana, IL
Calcium/Strontium (Residual)
1e+05
0e+00
-1e+05
Line

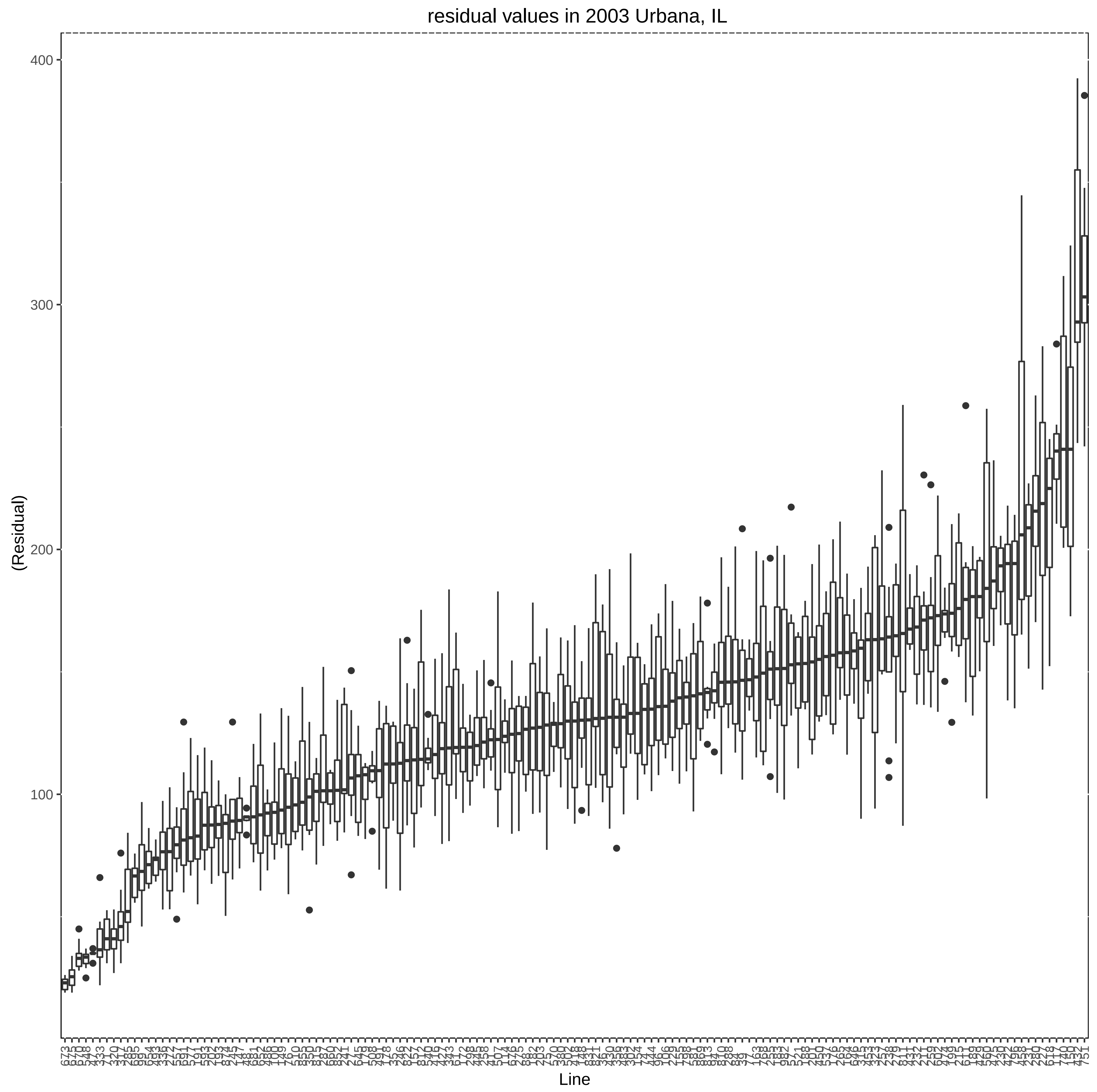
residual values in 2003 Urbana, IL
400
300
200
100
(Residual)
Line

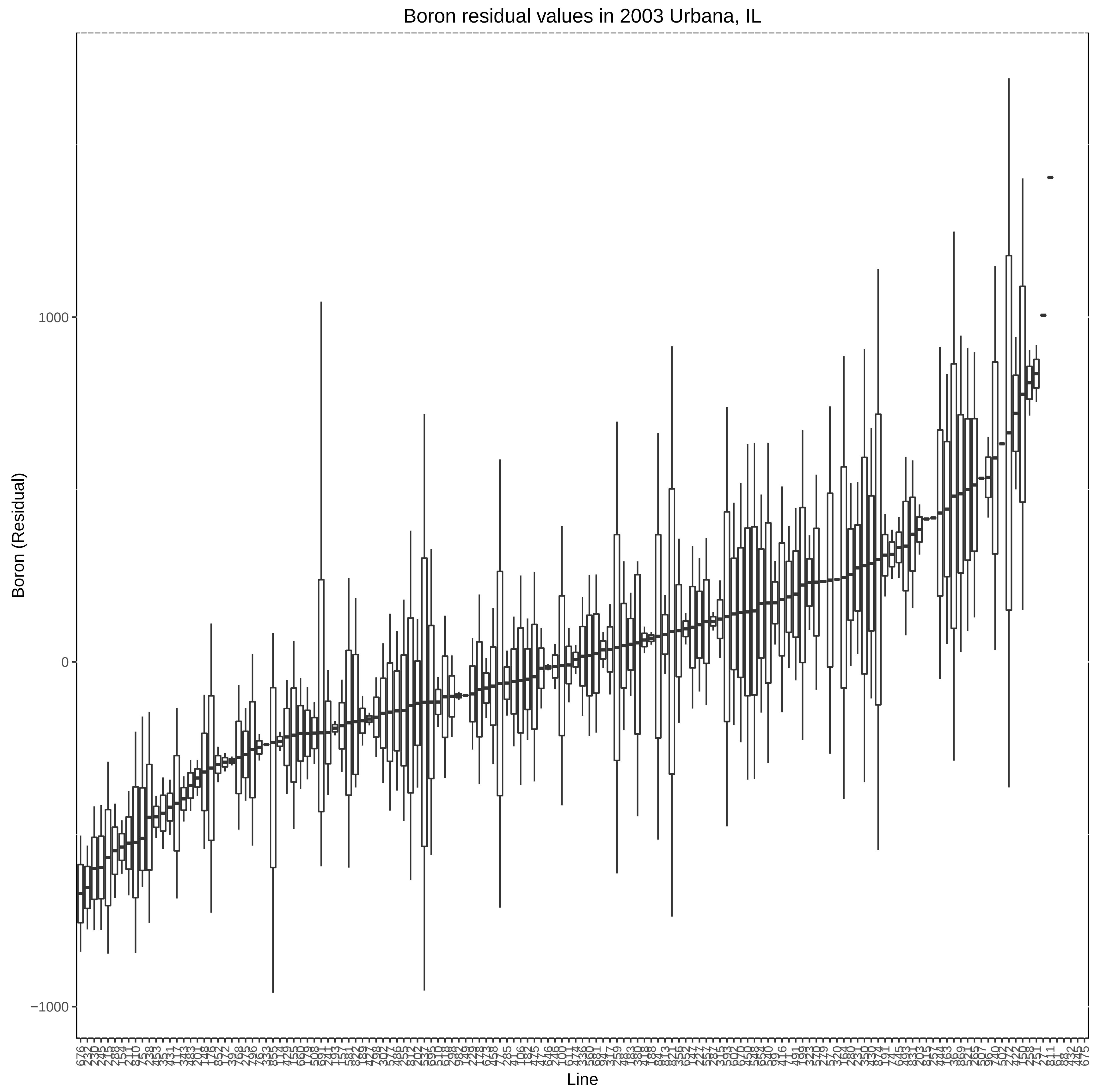
Boron residual values in 2003 Urbana, IL
1000
0
−1000
Boron (Residual)
Line

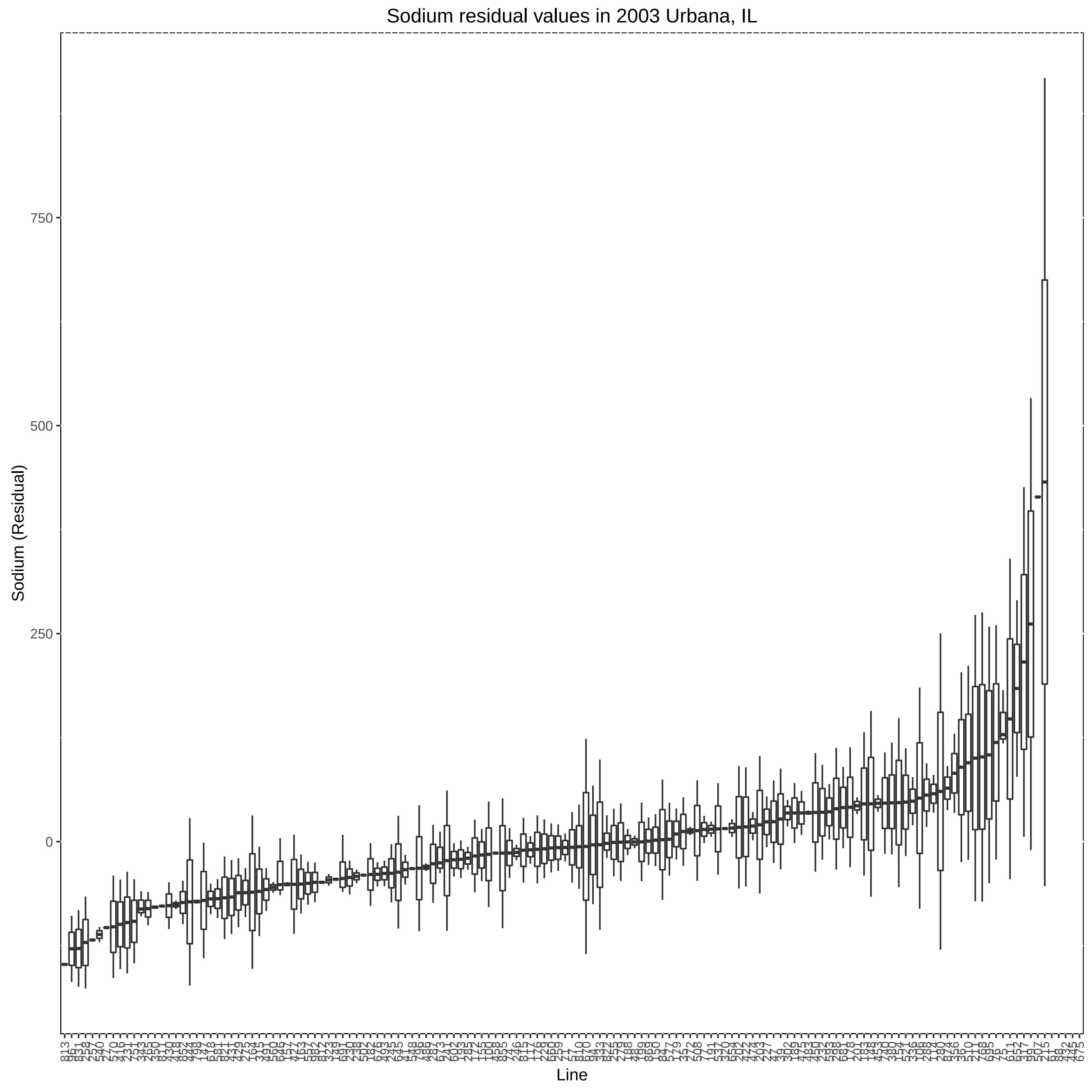
Sodium residual values in 2003 Urbana, IL
750
500
250
0
Sodium (Residual)
Line

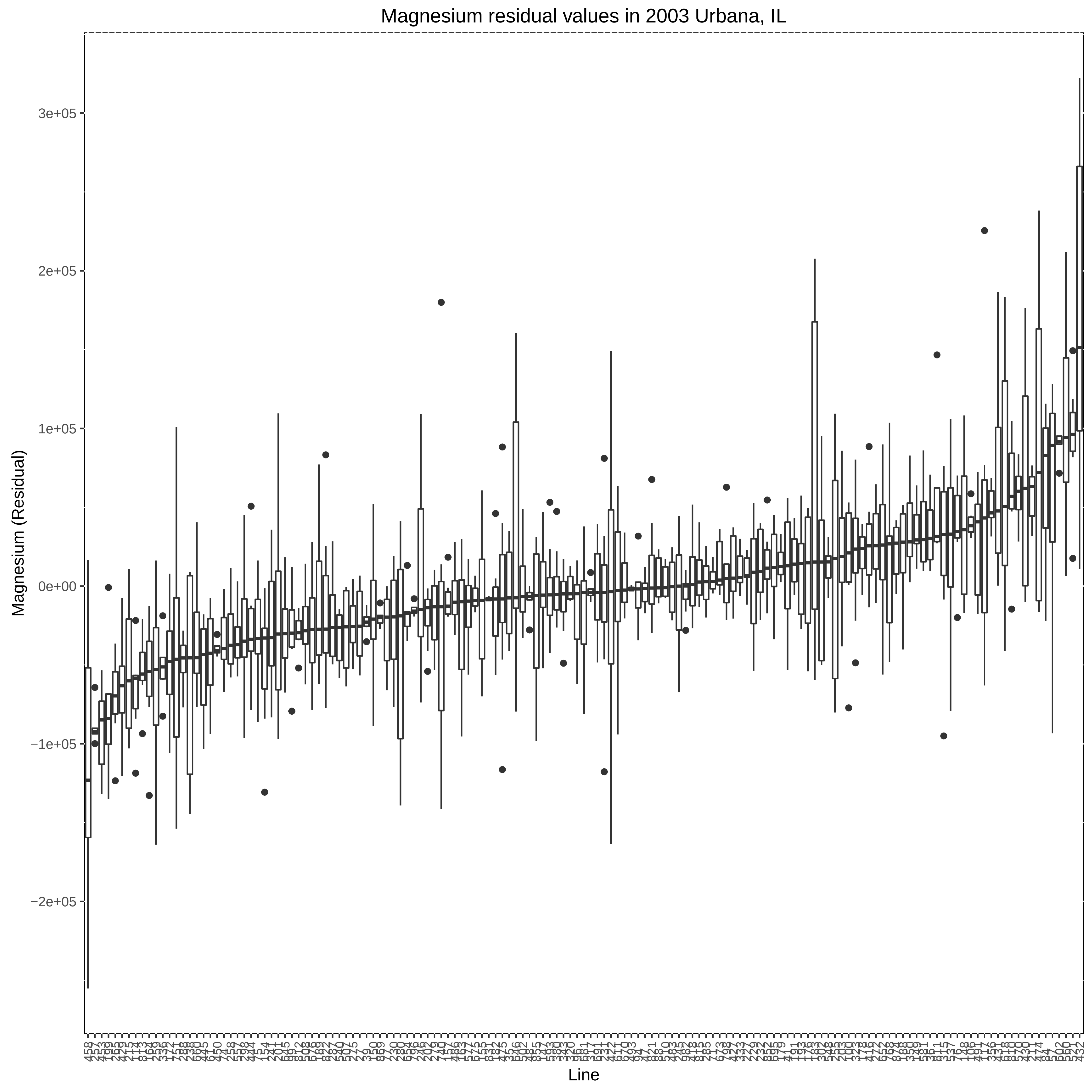
Magnesium residual values in 2003 Urbana, IL
3e+05
2e+05
1e+05
0e+00
−1e+05
−2e+05
Magnesium (Residual)
Line

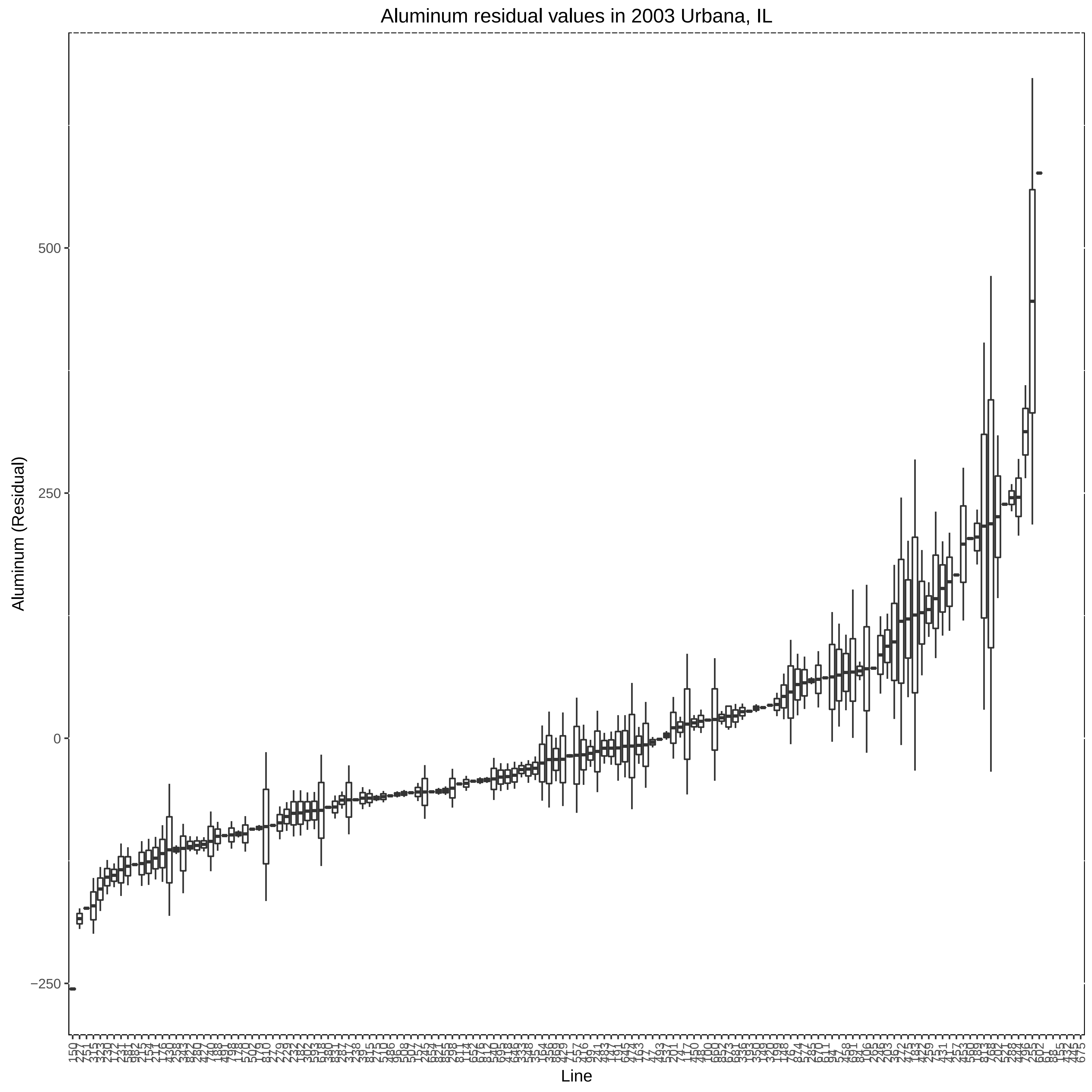
Aluminum residual values in 2003 Urbana, IL
Aluminum (Residual)
500
250
0
−250
Line

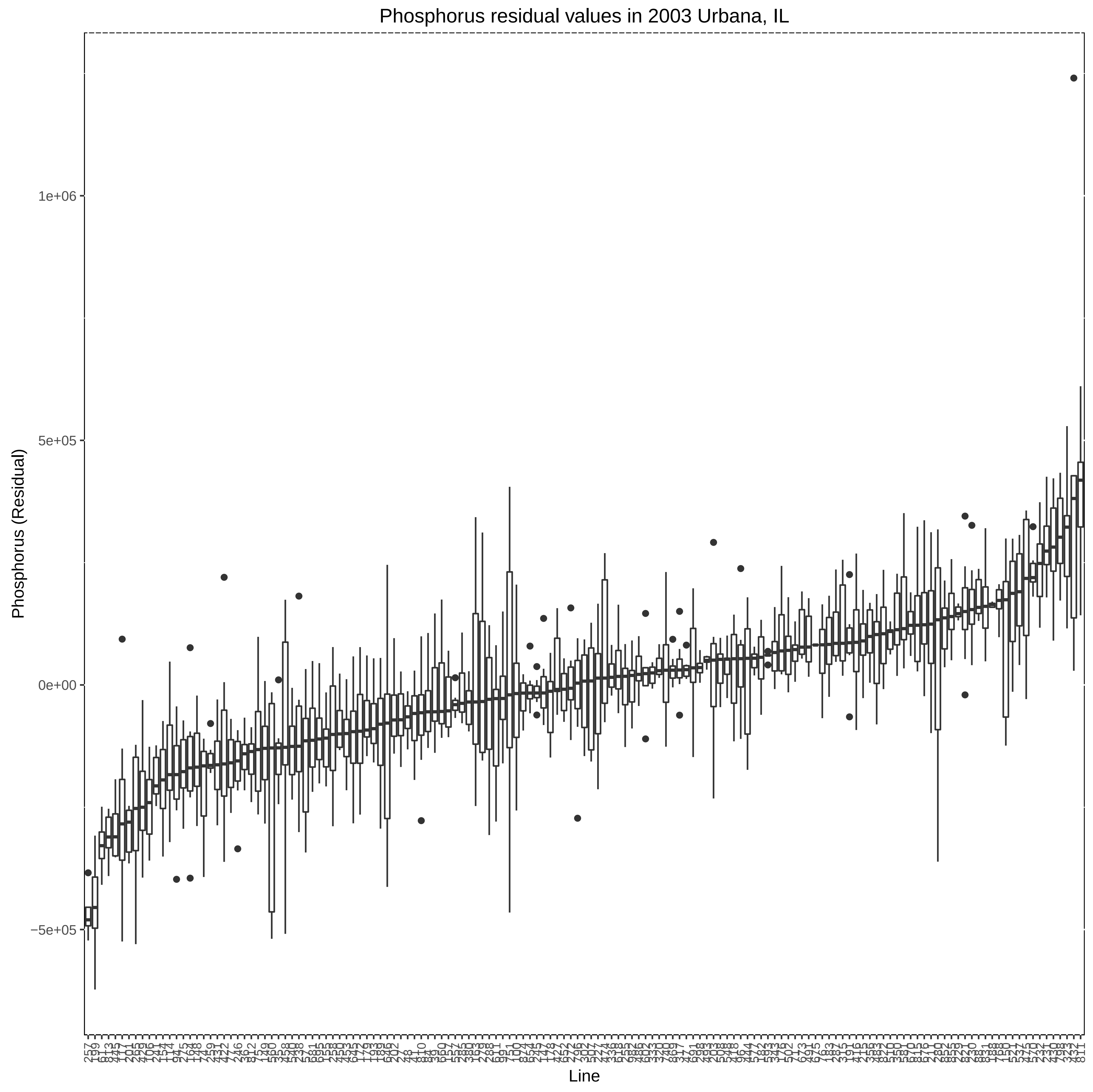
Phosphorus residual values in 2003 Urbana, IL
Phosphorus (Residual)
1e+06
5e+05
0e+00
−5e+05
Line

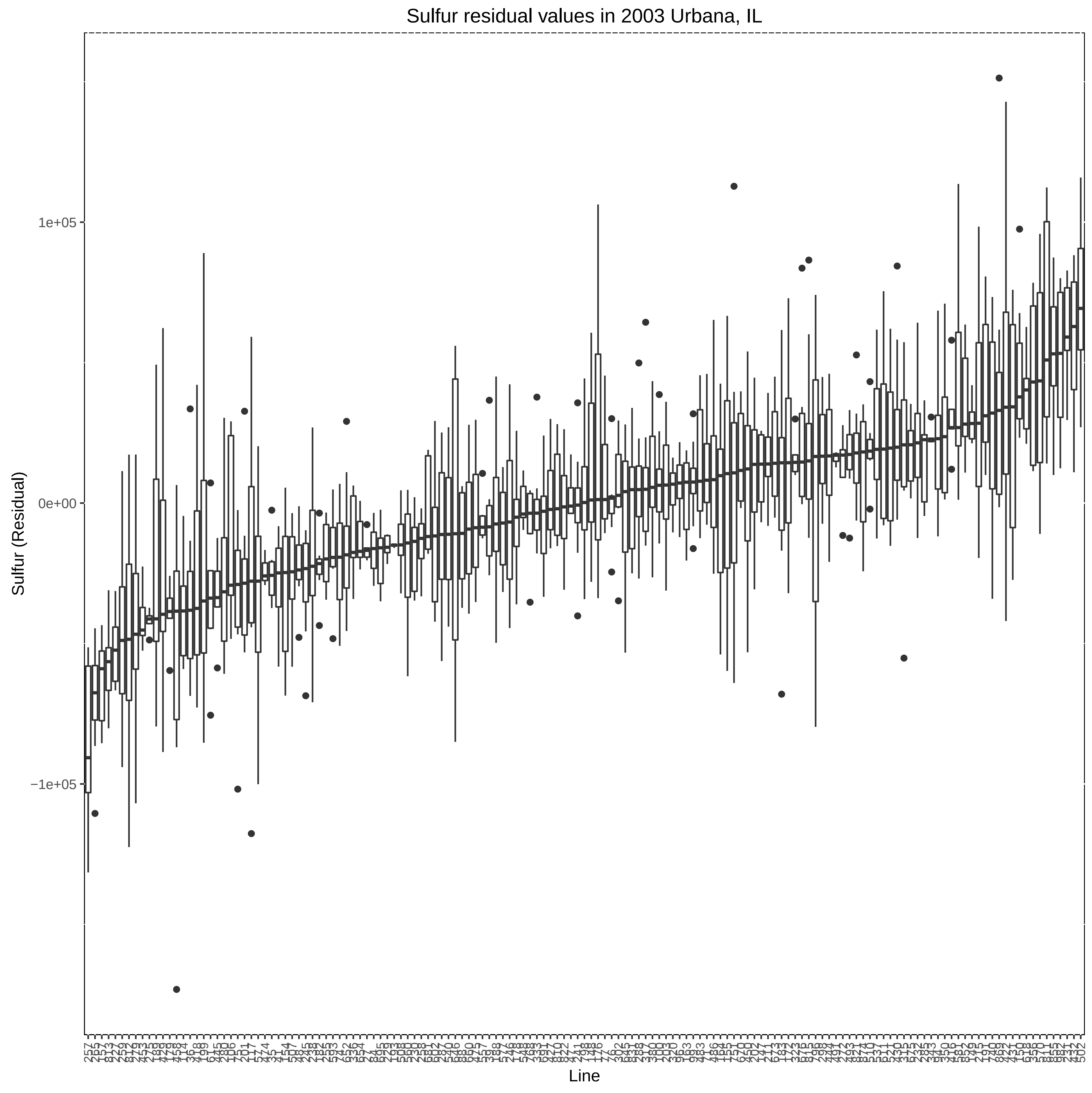
Sulfur residual values in 2003 Urbana, IL
Sulfur (Residual)
1e+05
0e+00
−1e+05
Line

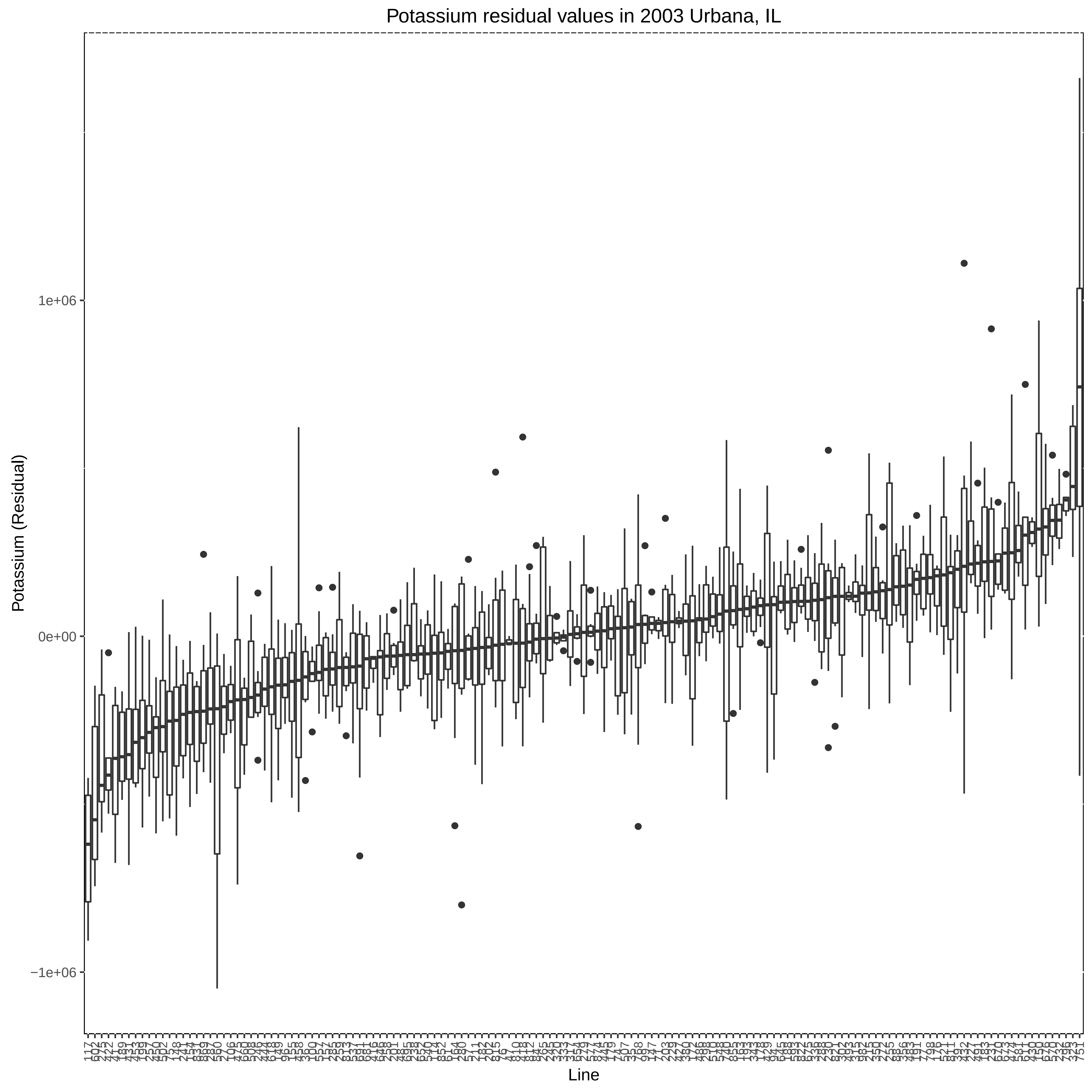
Potassium residual values in 2003 Urbana, IL
Potassium (Residual)
1e+06
0e+00
−1e+06
Line

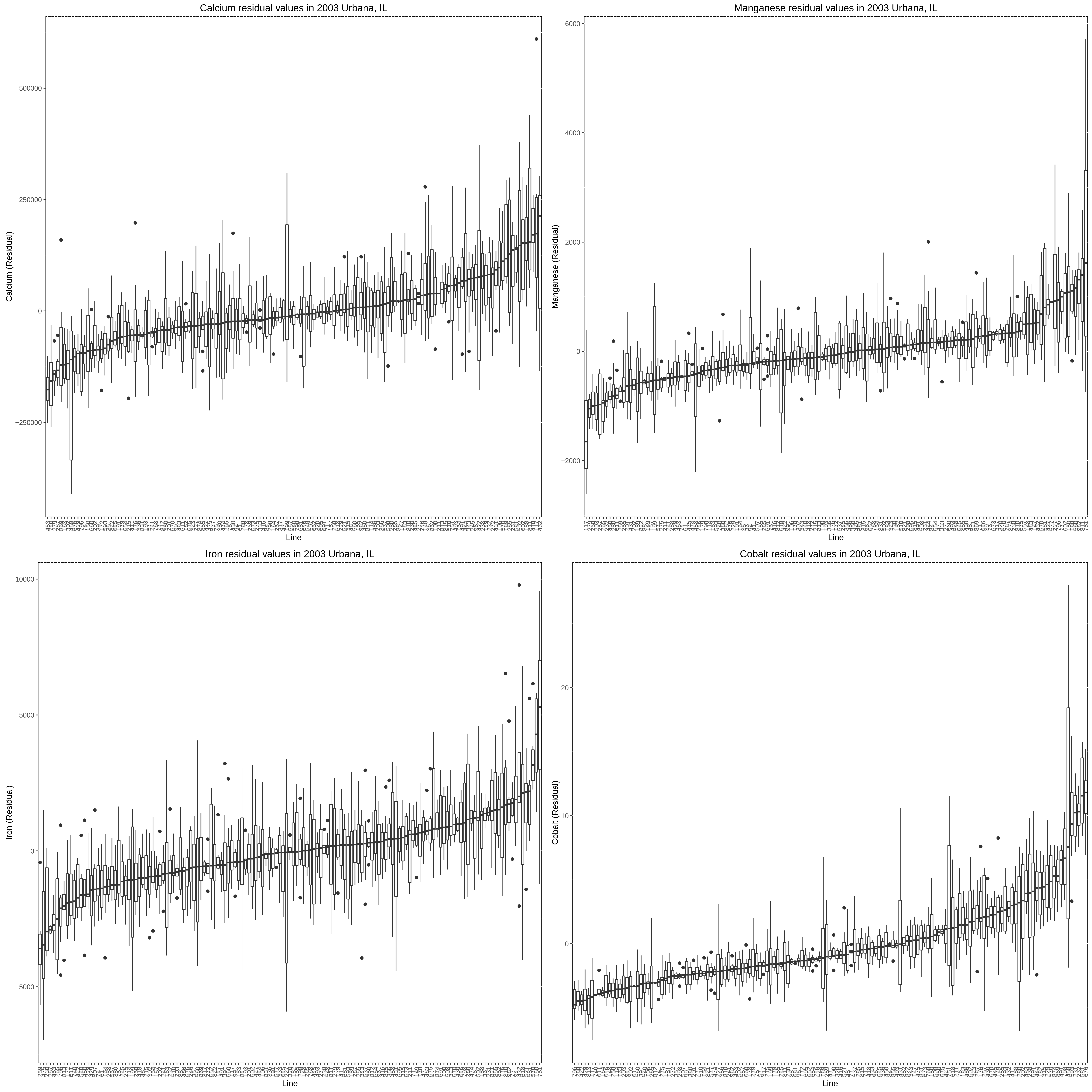

Calcium residual values in 2003 Urbana, IL
Calcium (Residual)
500000
250000
0
-250000
Line
Manganese residual values in 2003 Urbana, IL
Manganese (Residual)
6000
4000
2000
0
-2000
Line
Iron residual values in 2003 Urbana, IL
Iron (Residual)
10000
5000
0
-5000
Line
Cobalt residual values in 2003 Urbana, IL
Cobalt (Residual)
20
10
0
Line

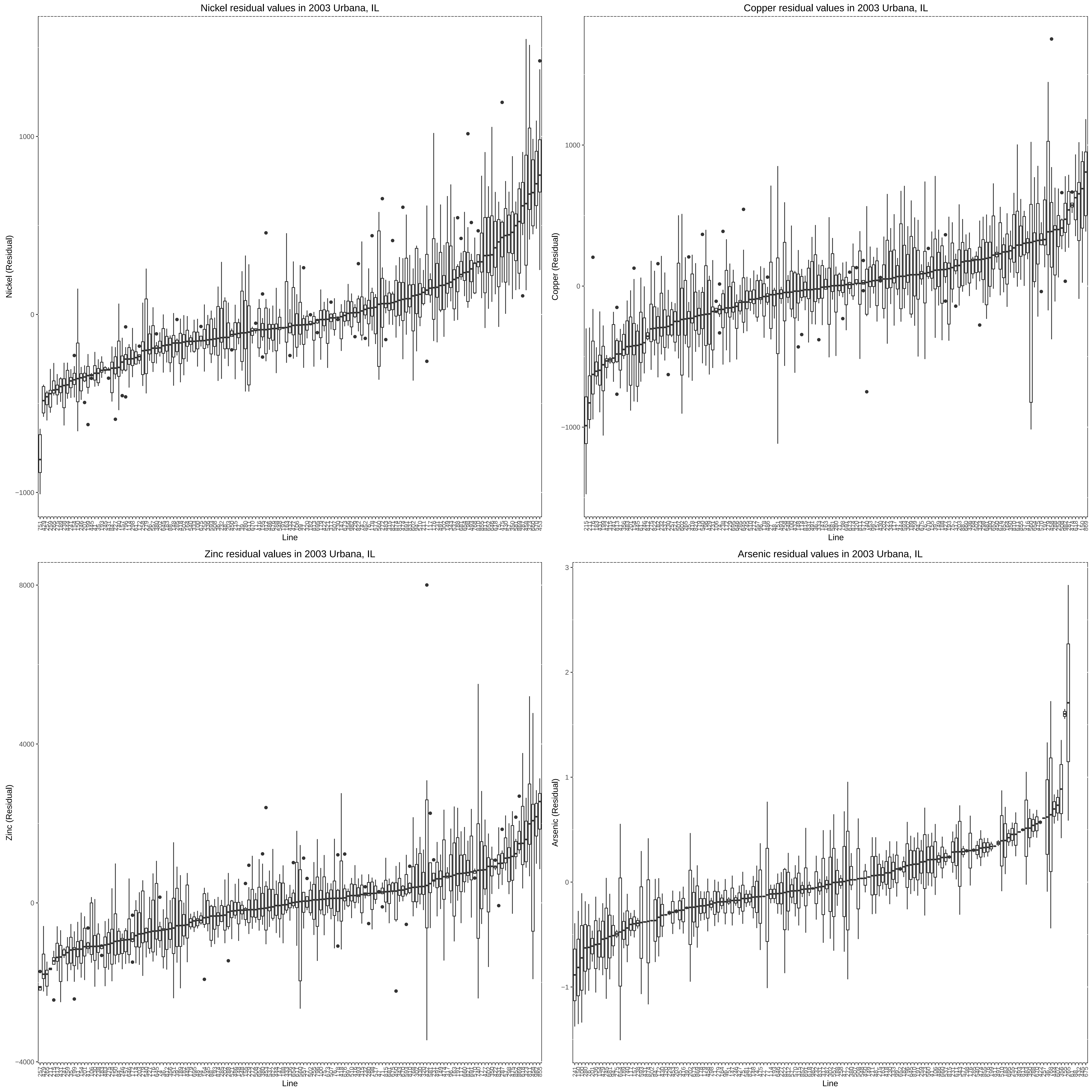
Nickel residual values in 2003 Urbana, IL
Nickel (Residual)
1000
0
−1000
Line
Copper residual values in 2003 Urbana, IL
Copper (Residual)
1000
0
−1000
Line
Zinc residual values in 2003 Urbana, IL
Zinc (Residual)
8000
4000
0
−4000
Line
Arsenic residual values in 2003 Urbana, IL
Arsenic (Residual)
3
2
1
0
−1
Line

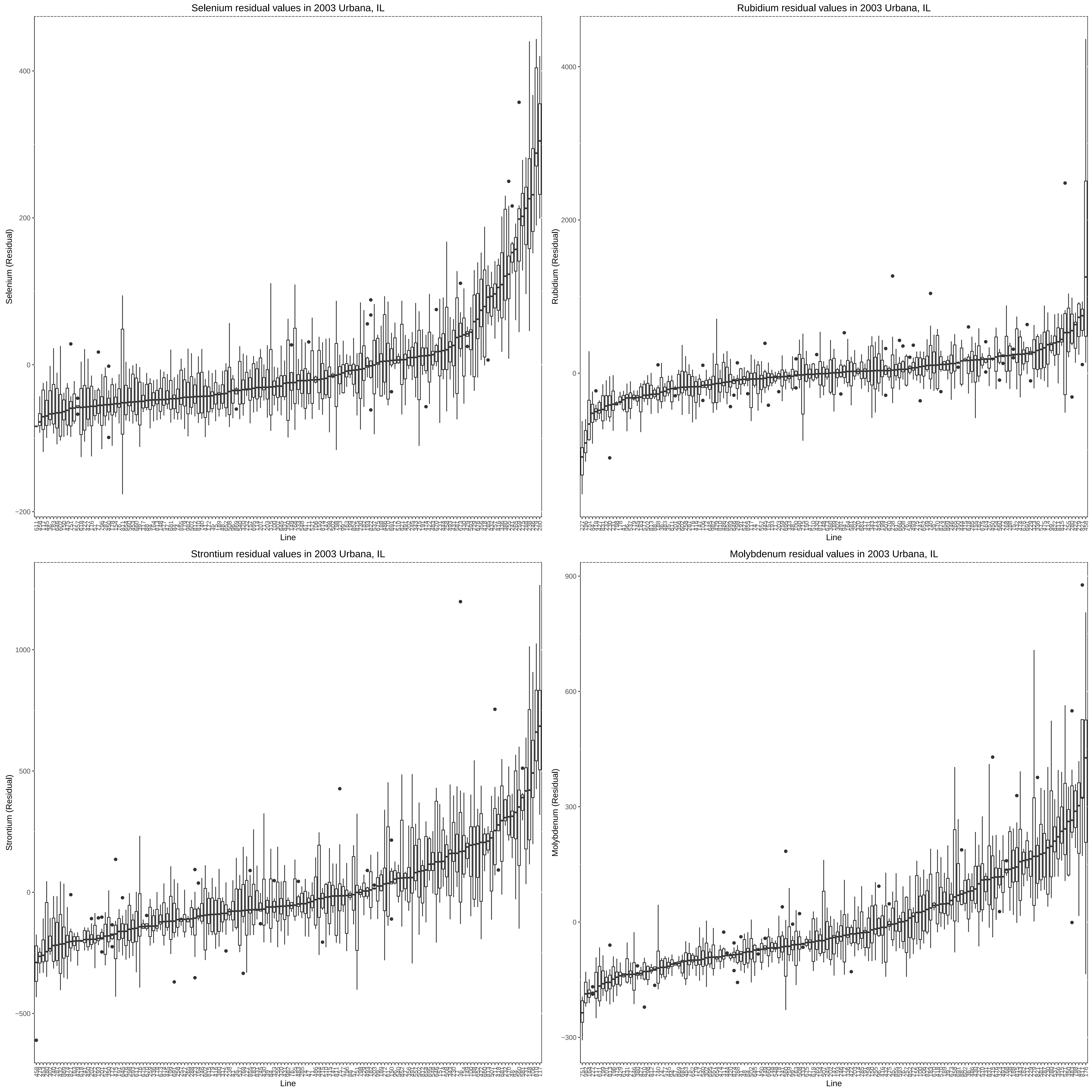
Selenium residual values in 2003 Urbana, IL
Selenium (Residual)
400
200
0
-200
Line
Rubidium residual values in 2003 Urbana, IL
Rubidium (Residual)
4000
2000
0
Line
Strontium residual values in 2003 Urbana, IL
Strontium (Residual)
1000
500
0
-500
Line
Molybdenum residual values in 2003 Urbana, IL
Molybdenum (Residual)
900
600
300
0
-300
Line

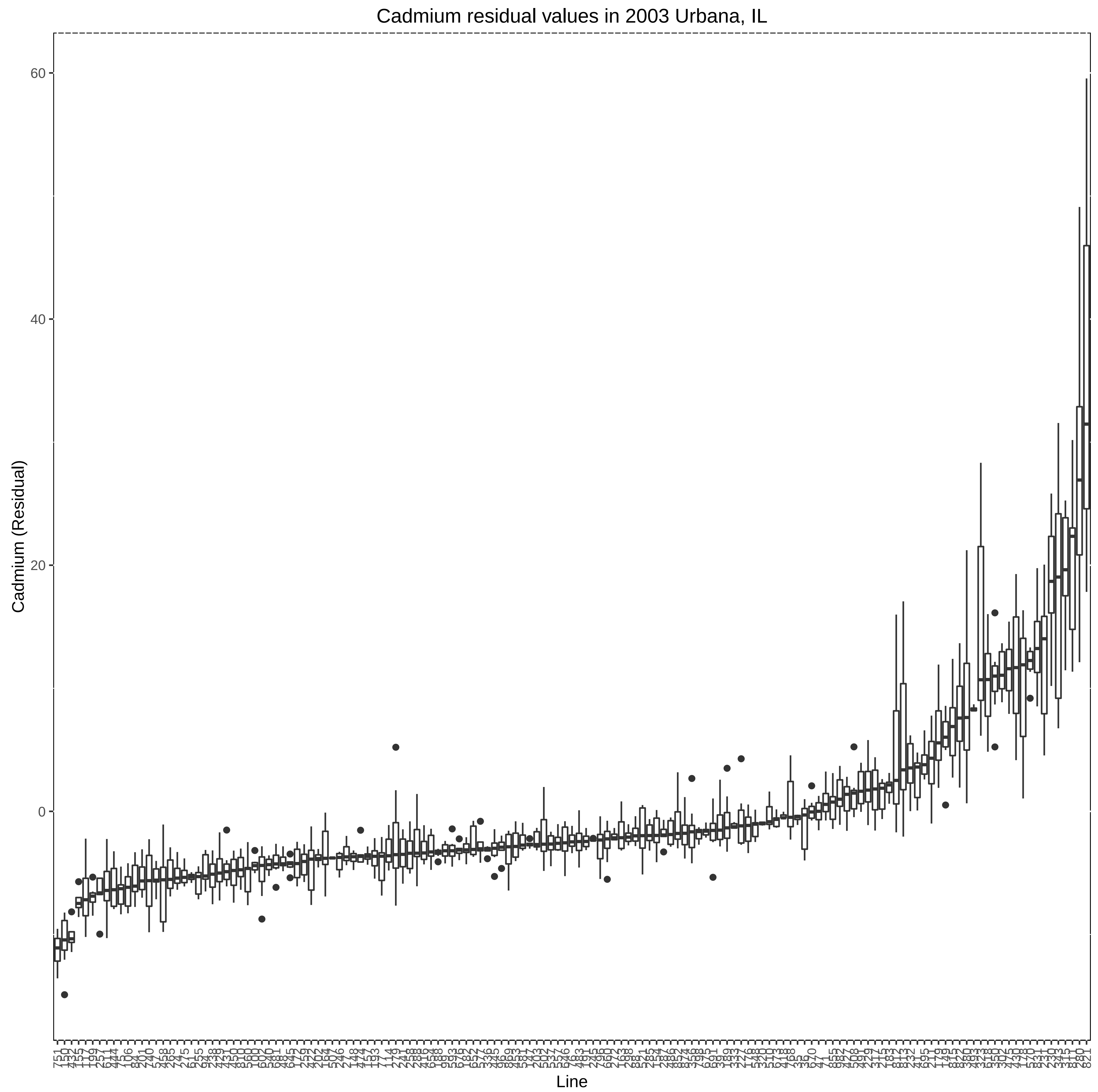
Cadmium residual values in 2003 Urbana, IL
Cadmium (Residual)
60
40
20
0
Line

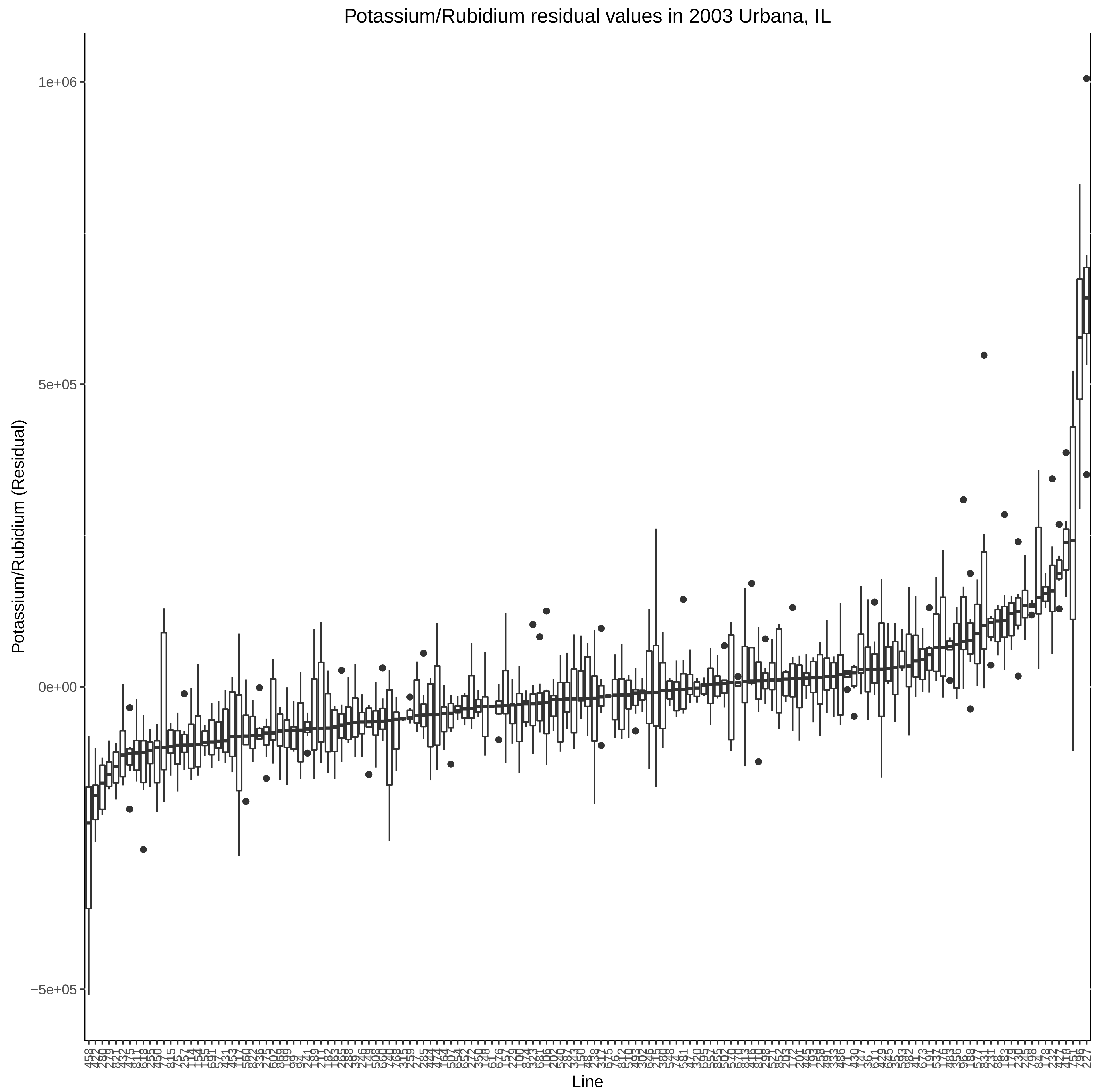
Potassium/Rubidium residual values in 2003 Urbana, IL
Potassium/Rubidium (Residual)
1e+06
5e+05
0e+00
-5e+05
Line

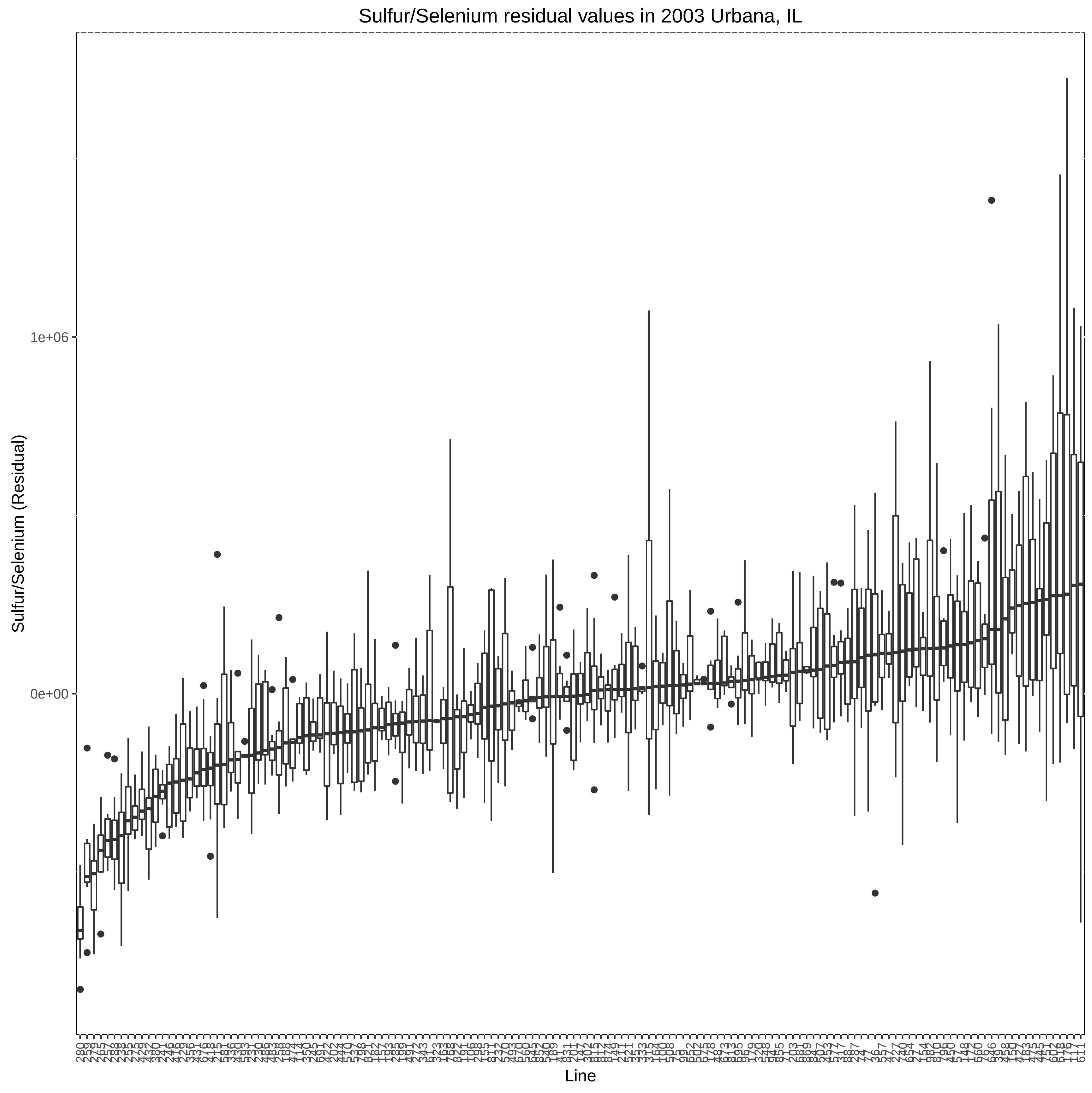
Sulfur/Selenium residual values in 2003 Urbana, IL
Sulfur/Selenium (Residual)
1e+06
0e+00
Line

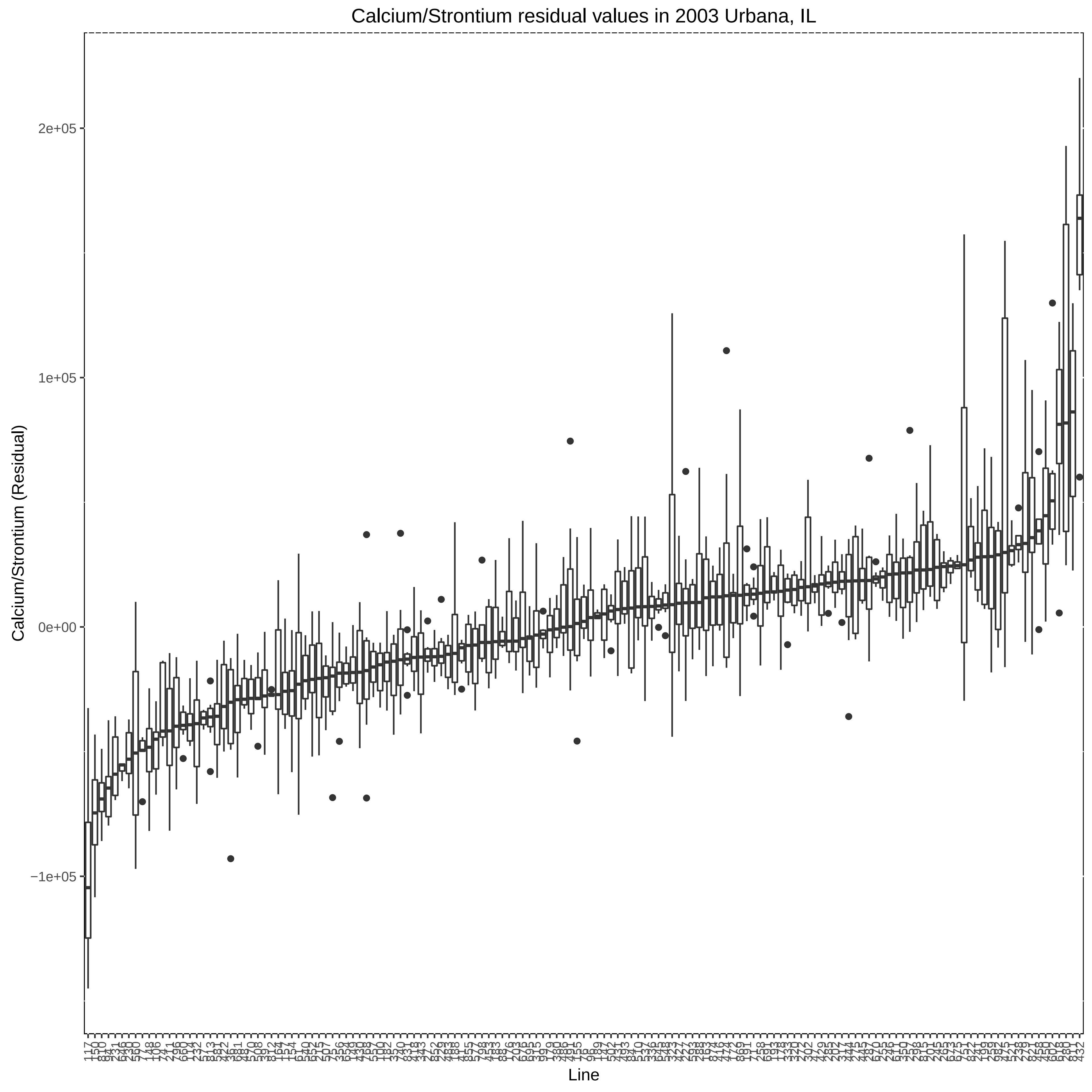
Calcium/Strontium residual values in 2003 Urbana, IL
Calcium/Strontium (Residual)
2e+05
1e+05
0e+00
-1e+05
Line

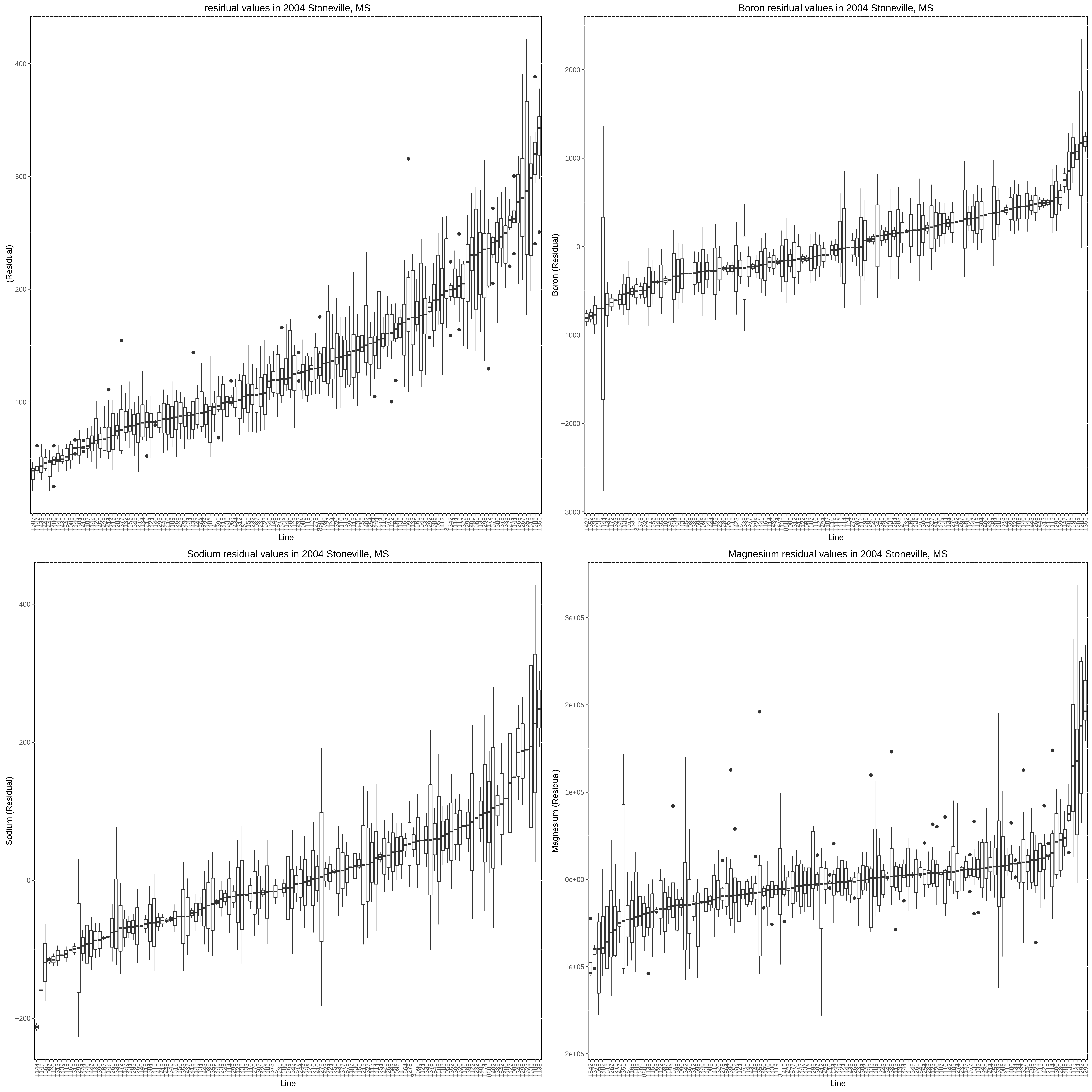

residual values in 2004 Stoneville, MS
(Residual)
Line
Boron residual values in 2004 Stoneville, MS
Boron (Residual)
Line
Sodium residual values in 2004 Stoneville, MS
Sodium (Residual)
Line
Magnesium residual values in 2004 Stoneville, MS
Magnesium (Residual)
Line

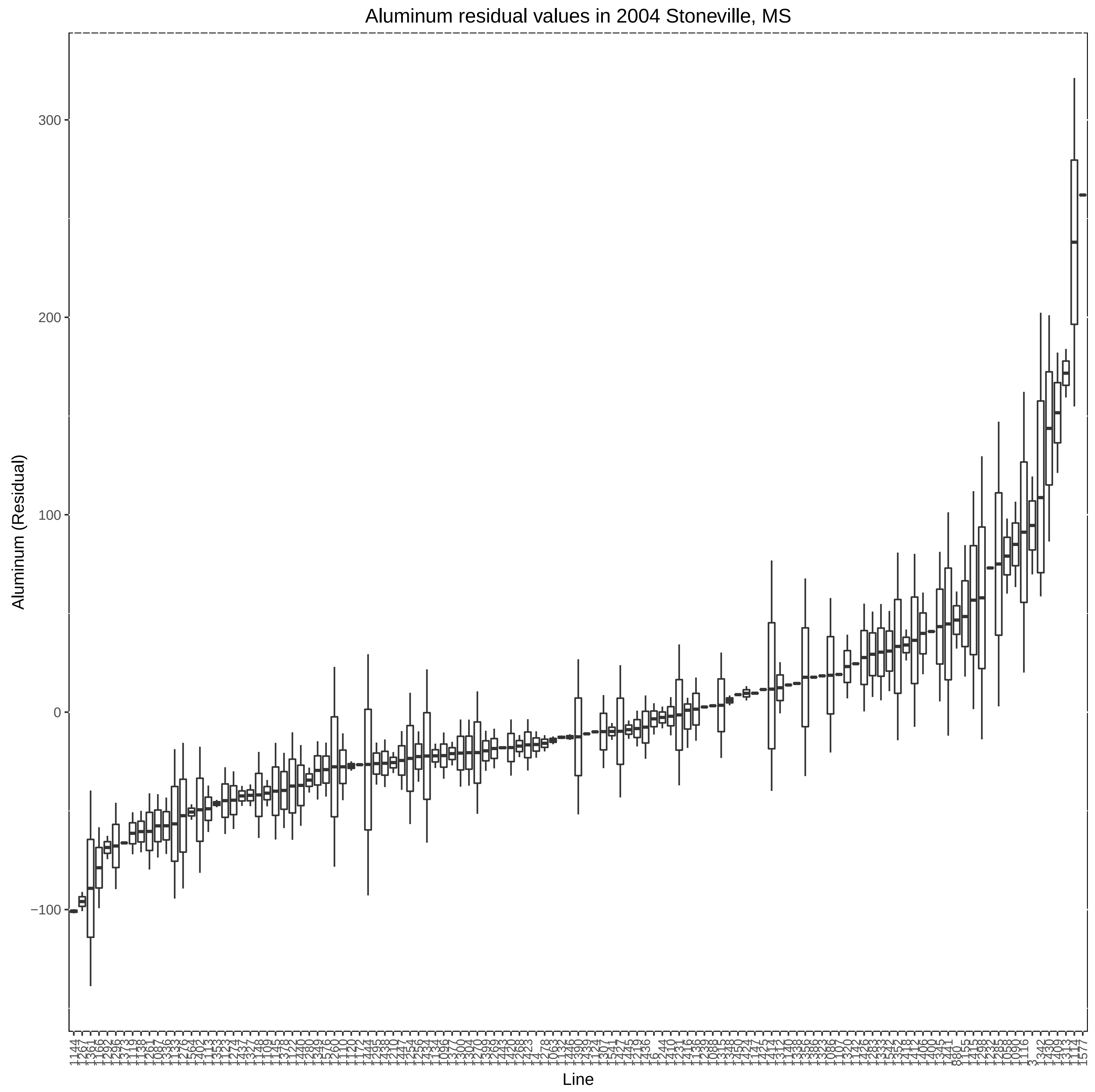
Aluminum residual values in 2004 Stoneville, MS
Aluminum (Residual)
Line
300
200
100
0
-100
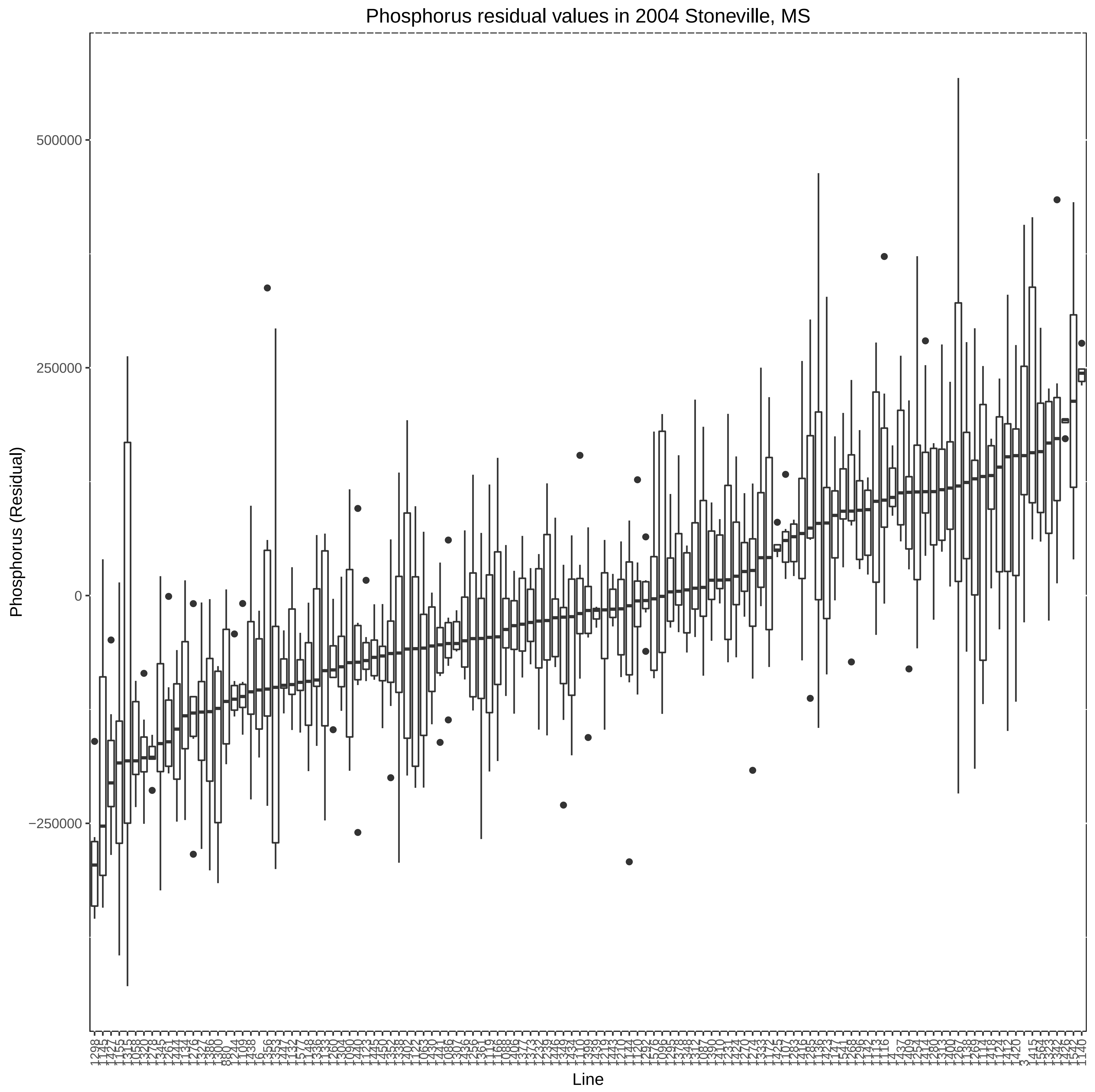
Phosphorus residual values in 2004 Stoneville, MS
Phosphorus (Residual)
Line
500000
250000
0
-250000
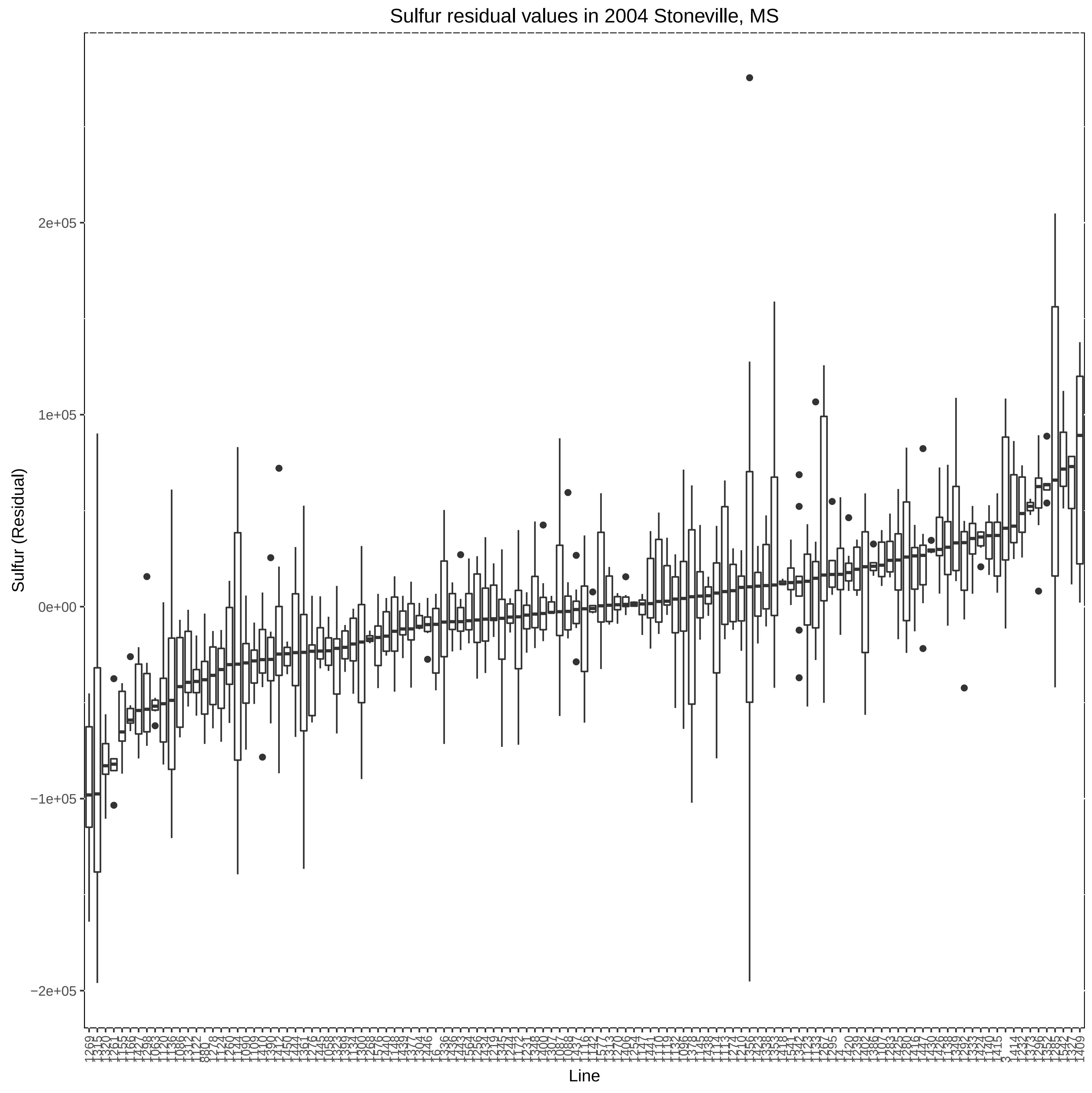
Sulfur residual values in 2004 Stoneville, MS
Sulfur (Residual)
Line
2e+05
1e+05
0e+00
-1e+05
-2e+05
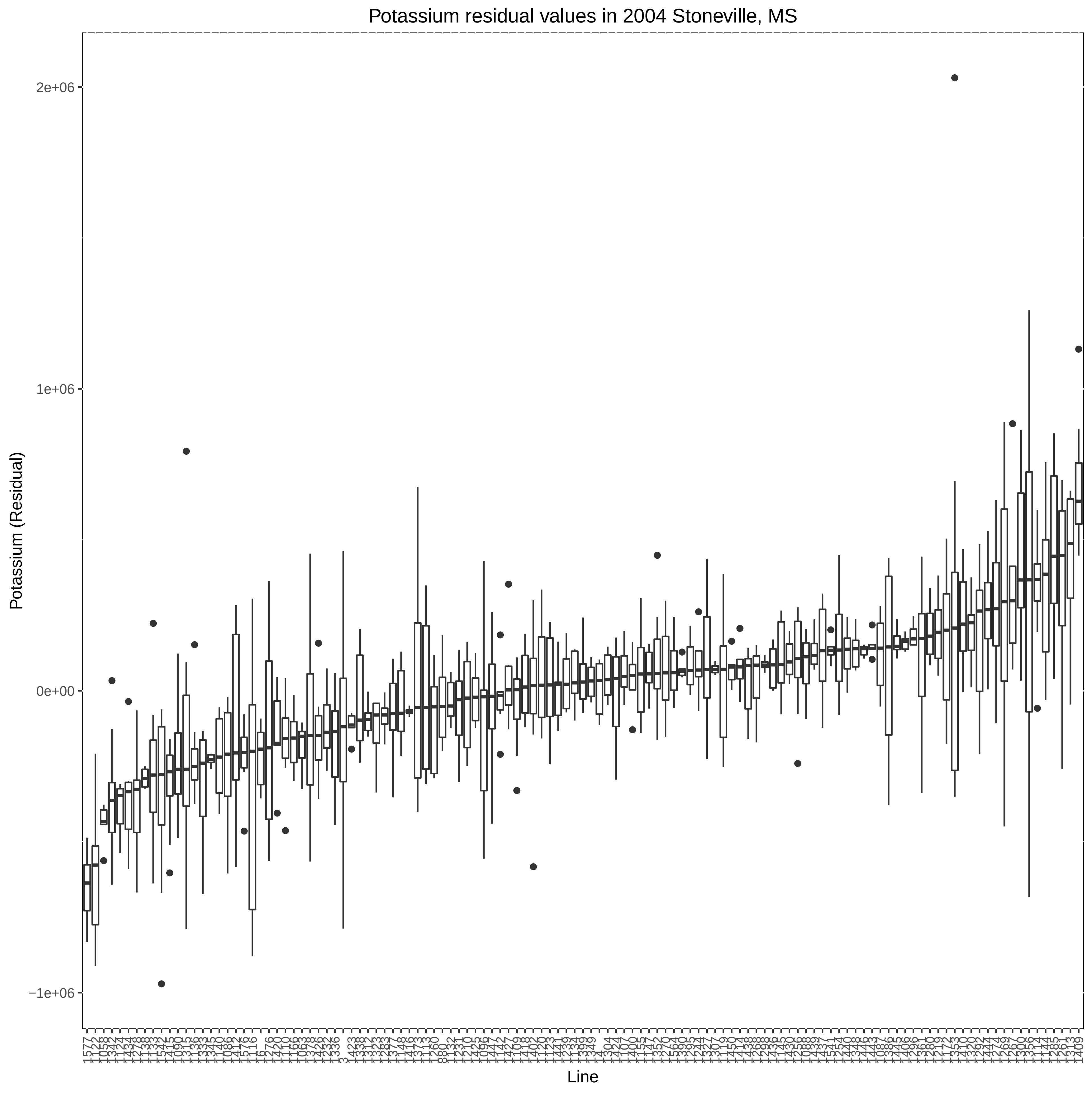
Potassium residual values in 2004 Stoneville, MS
Potassium (Residual)
Line
2e+06
1e+06
0e+00
-1e+06

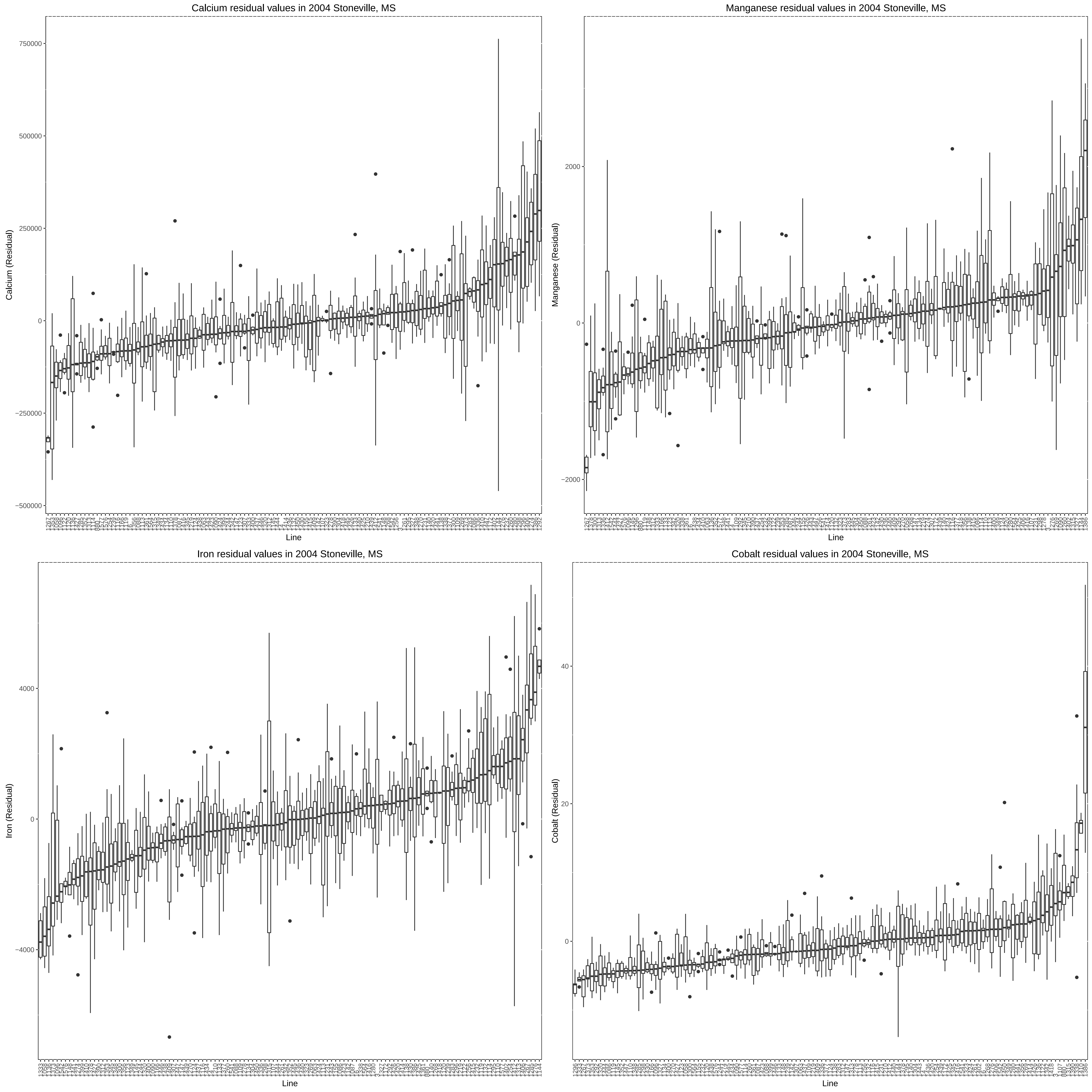

Calcium residual values in 2004 Stoneville, MS
Calcium (Residual)
750000
500000
250000
0
−250000
−500000
Line
Manganese residual values in 2004 Stoneville, MS
Manganese (Residual)
2000
0
−2000
Line
Iron residual values in 2004 Stoneville, MS
Iron (Residual)
4000
0
−4000
Line
Cobalt residual values in 2004 Stoneville, MS
Cobalt (Residual)
40
20
0
Line

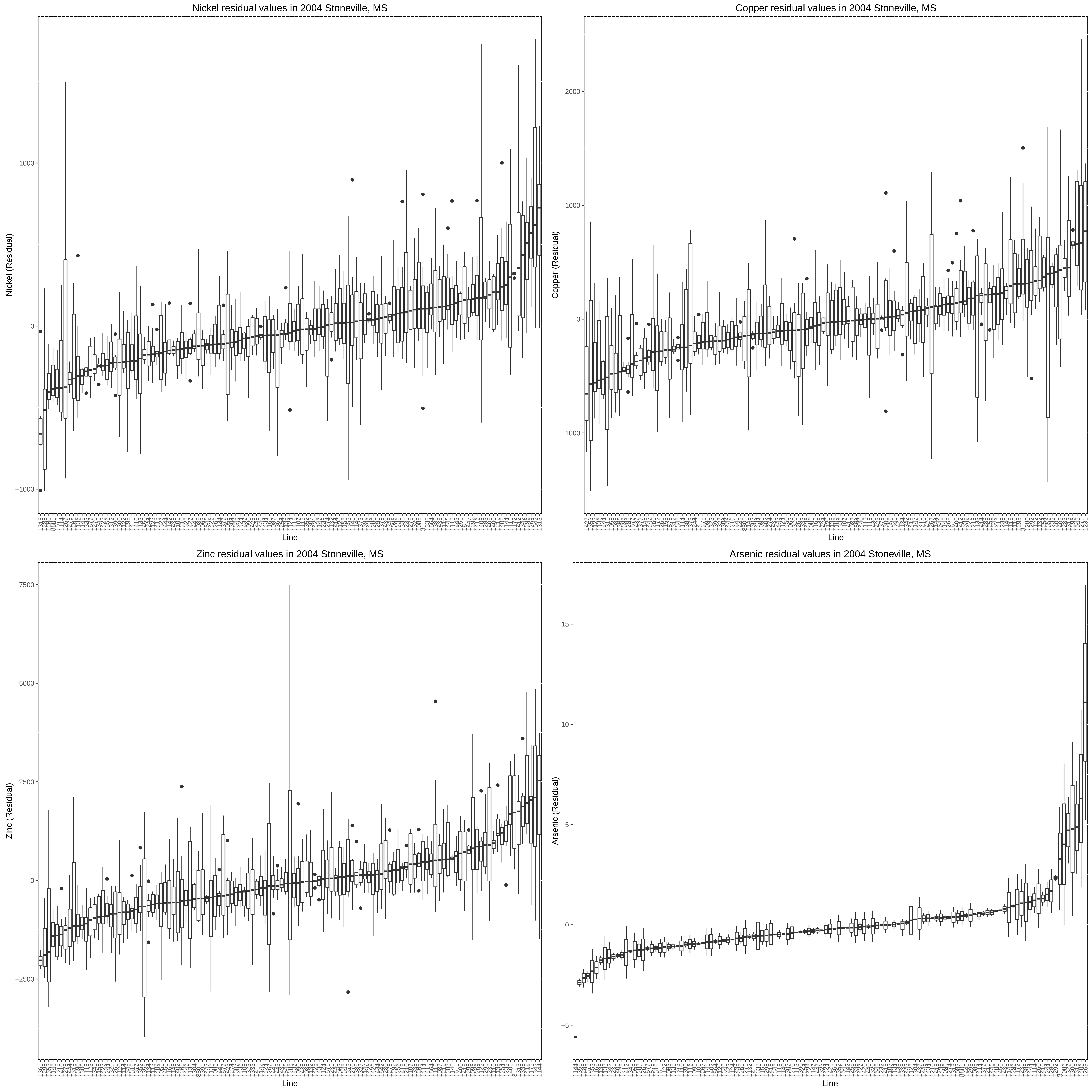

Nickel residual values in 2004 Stoneville, MS
Nickel (Residual)
Line
Copper residual values in 2004 Stoneville, MS
Copper (Residual)
Line
Zinc residual values in 2004 Stoneville, MS
Zinc (Residual)
Line
Arsenic residual values in 2004 Stoneville, MS
Arsenic (Residual)
Line

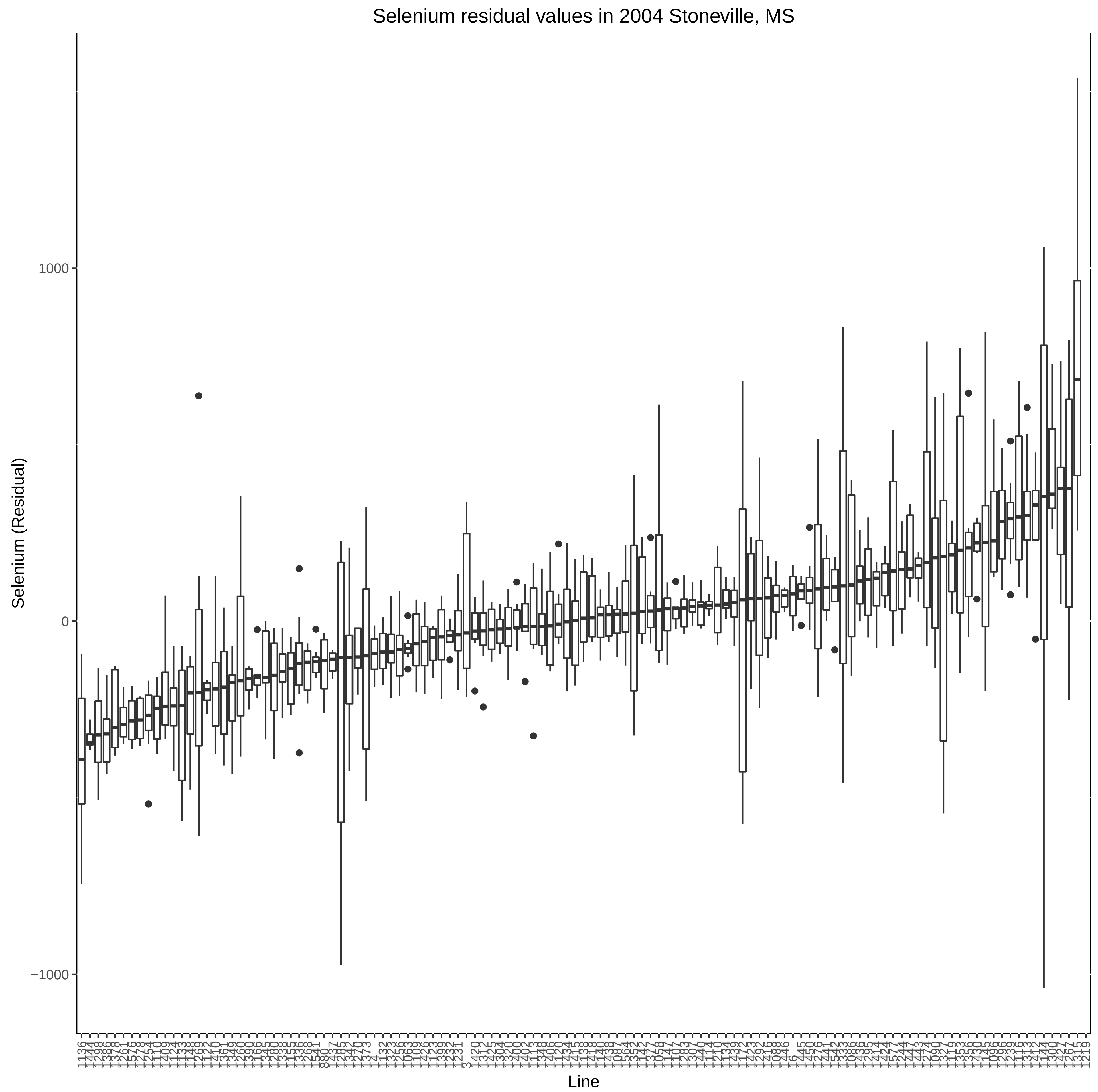
Selenium residual values in 2004 Stoneville, MS
Selenium (Residual)
1000
0
−1000
Line

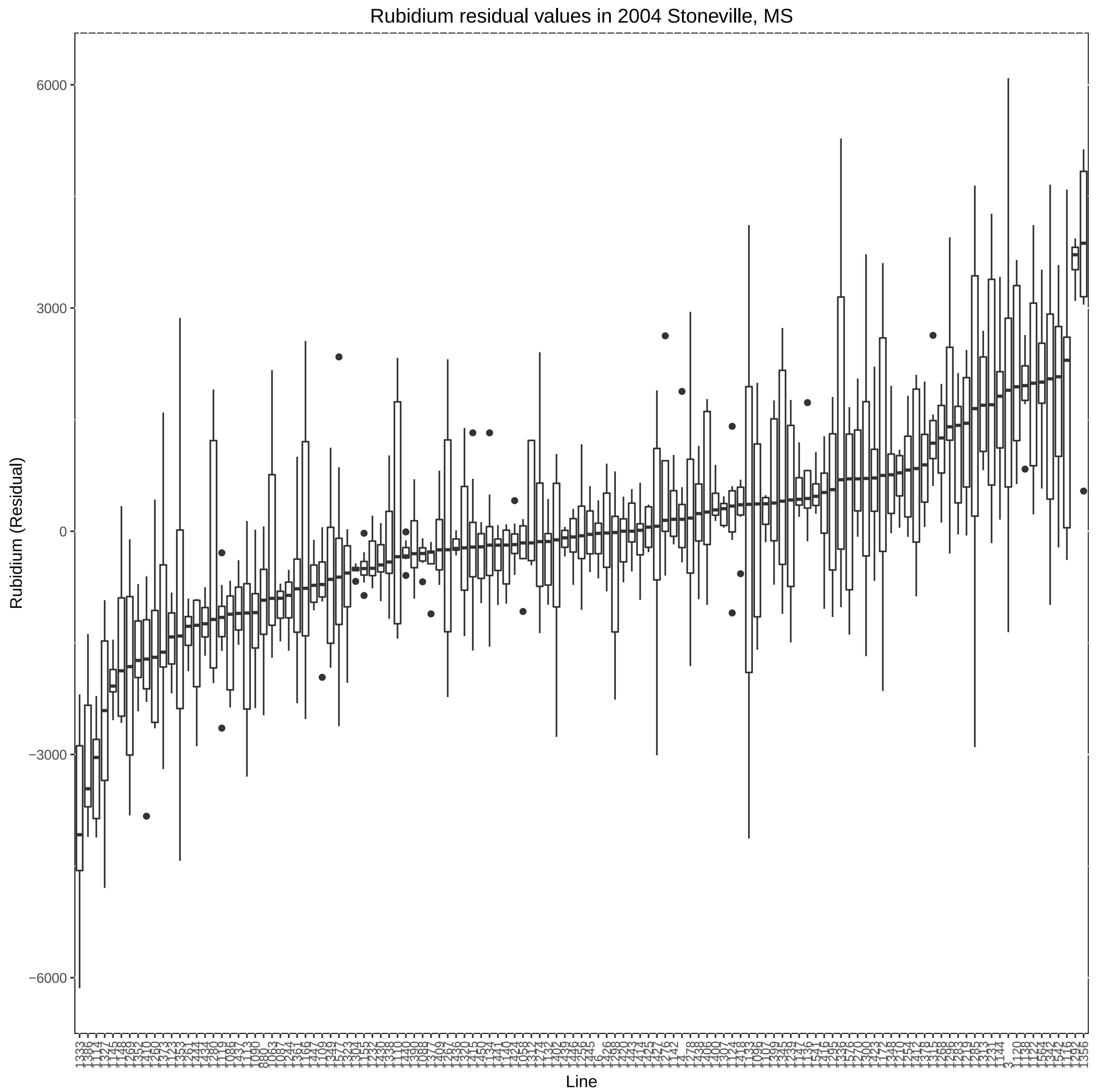
Rubidium residual values in 2004 Stoneville, MS
Rubidium (Residual)
6000
3000
0
−3000
−6000
Line

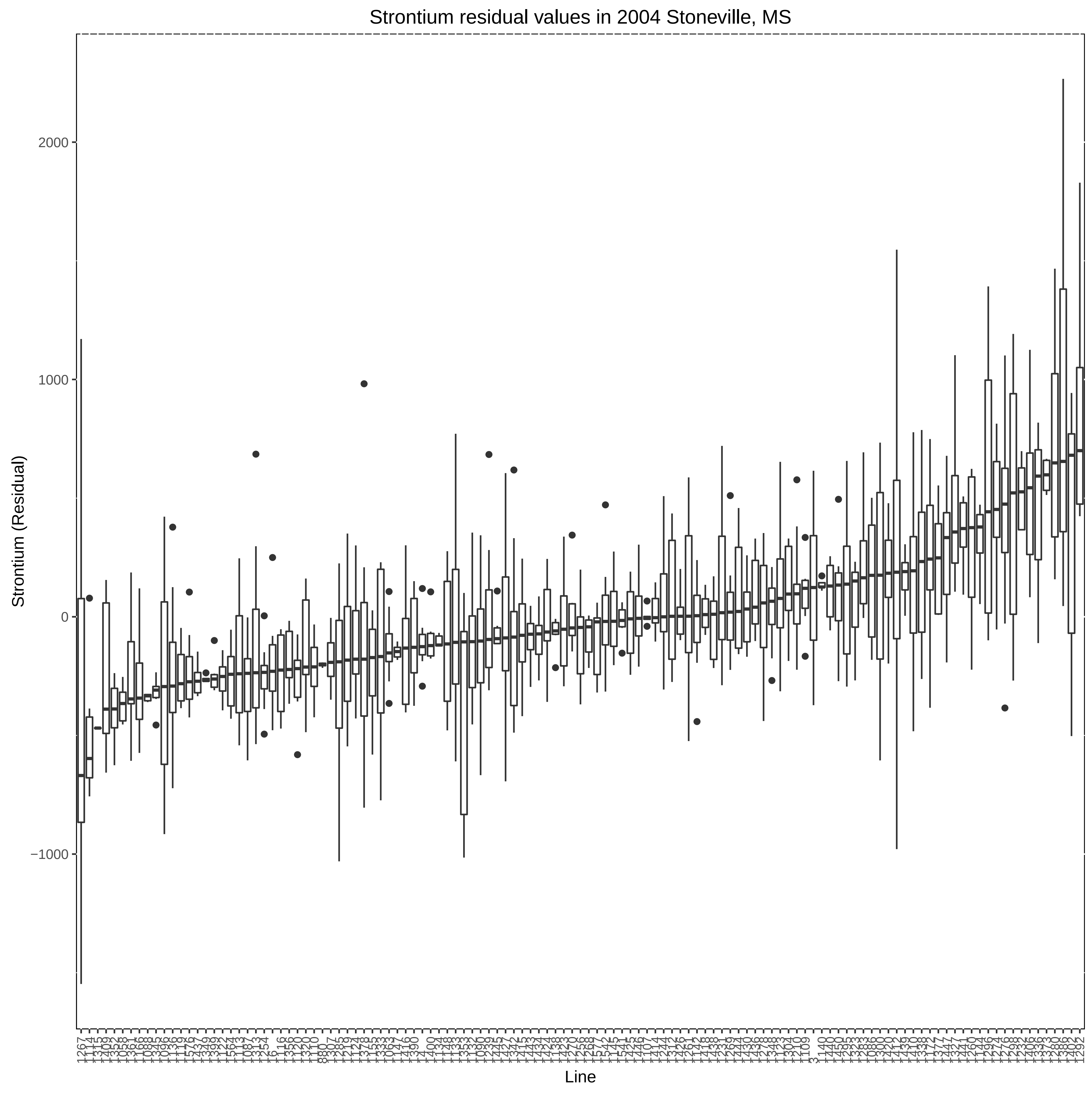
Strontium residual values in 2004 Stoneville, MS
Strontium (Residual)
2000
1000
0
−1000
Line

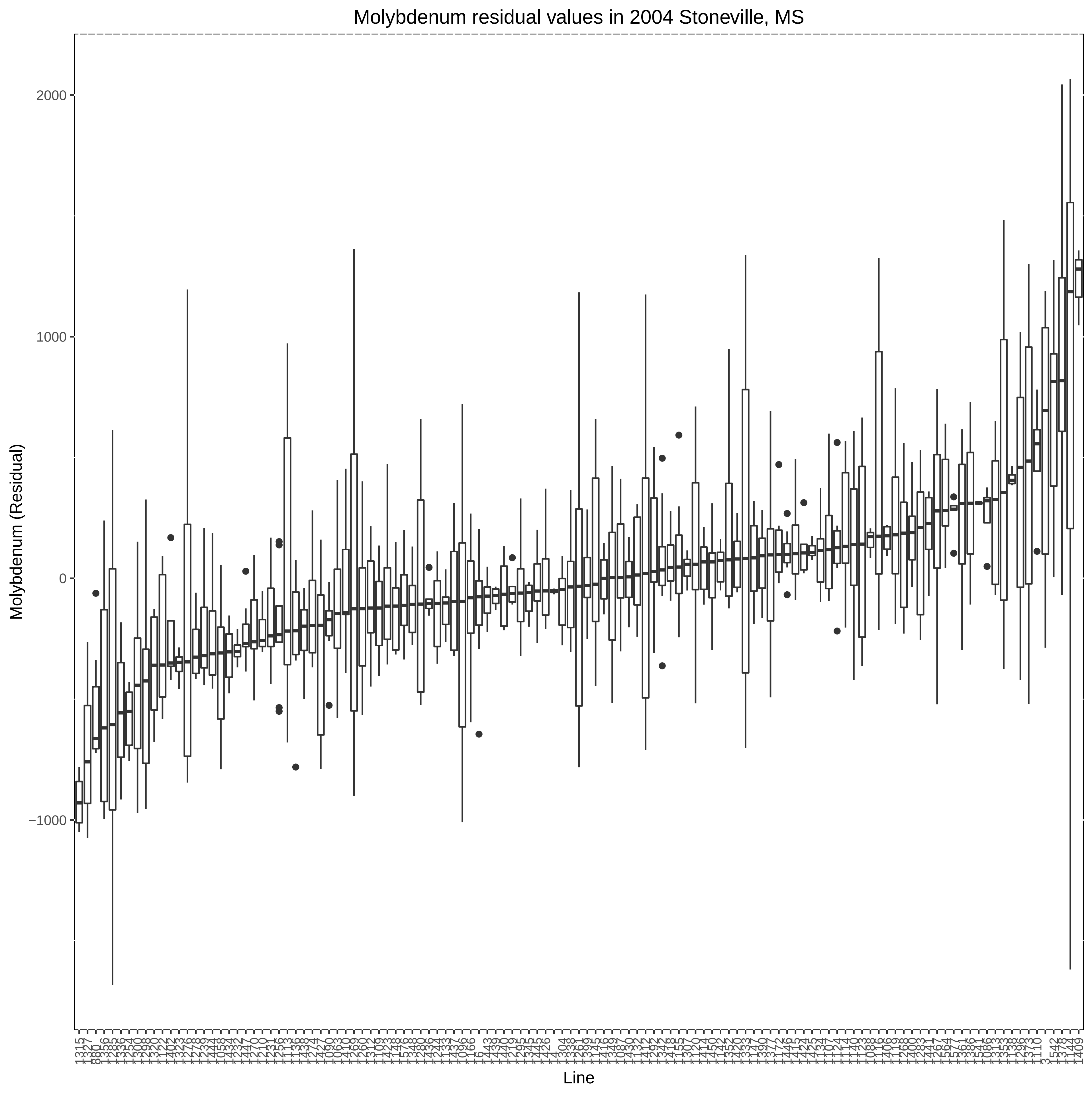
Molybdenum residual values in 2004 Stoneville, MS
Molybdenum (Residual)
2000
1000
0
−1000
Line

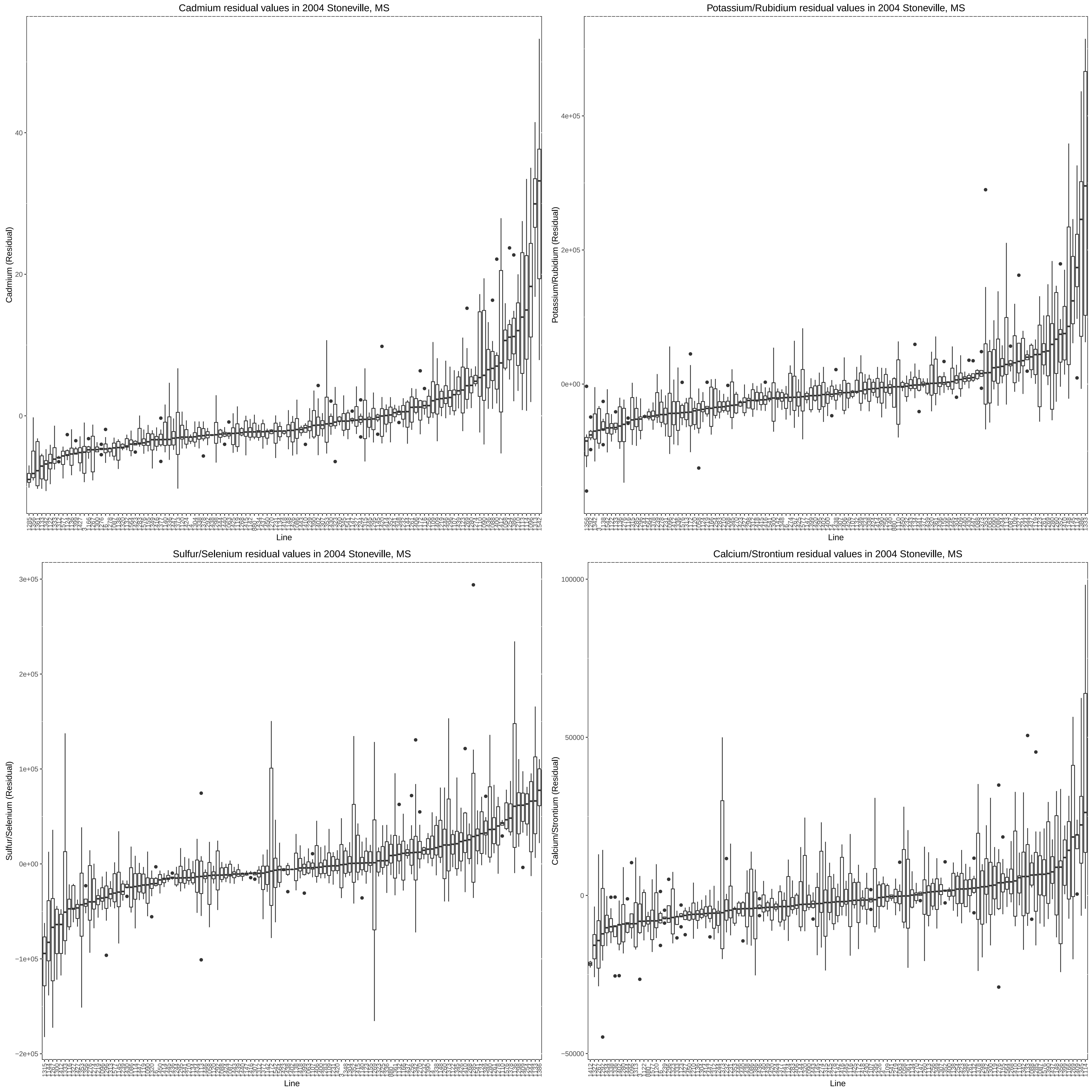
Cadmium residual values in 2004 Stoneville, MS
Cadmium (Residual)
Line
40
20
0
Potassium/Rubidium residual values in 2004 Stoneville, MS
Potassium/Rubidium (Residual)
Line
4e+05
2e+05
0e+00
Sulfur/Selenium residual values in 2004 Stoneville, MS
Sulfur/Selenium (Residual)
Line
3e+05
2e+05
1e+05
0e+00
-1e+05
-2e+05
Calcium/Strontium residual values in 2004 Stoneville, MS
Calcium/Strontium (Residual)
Line
100000
50000
0
-50000

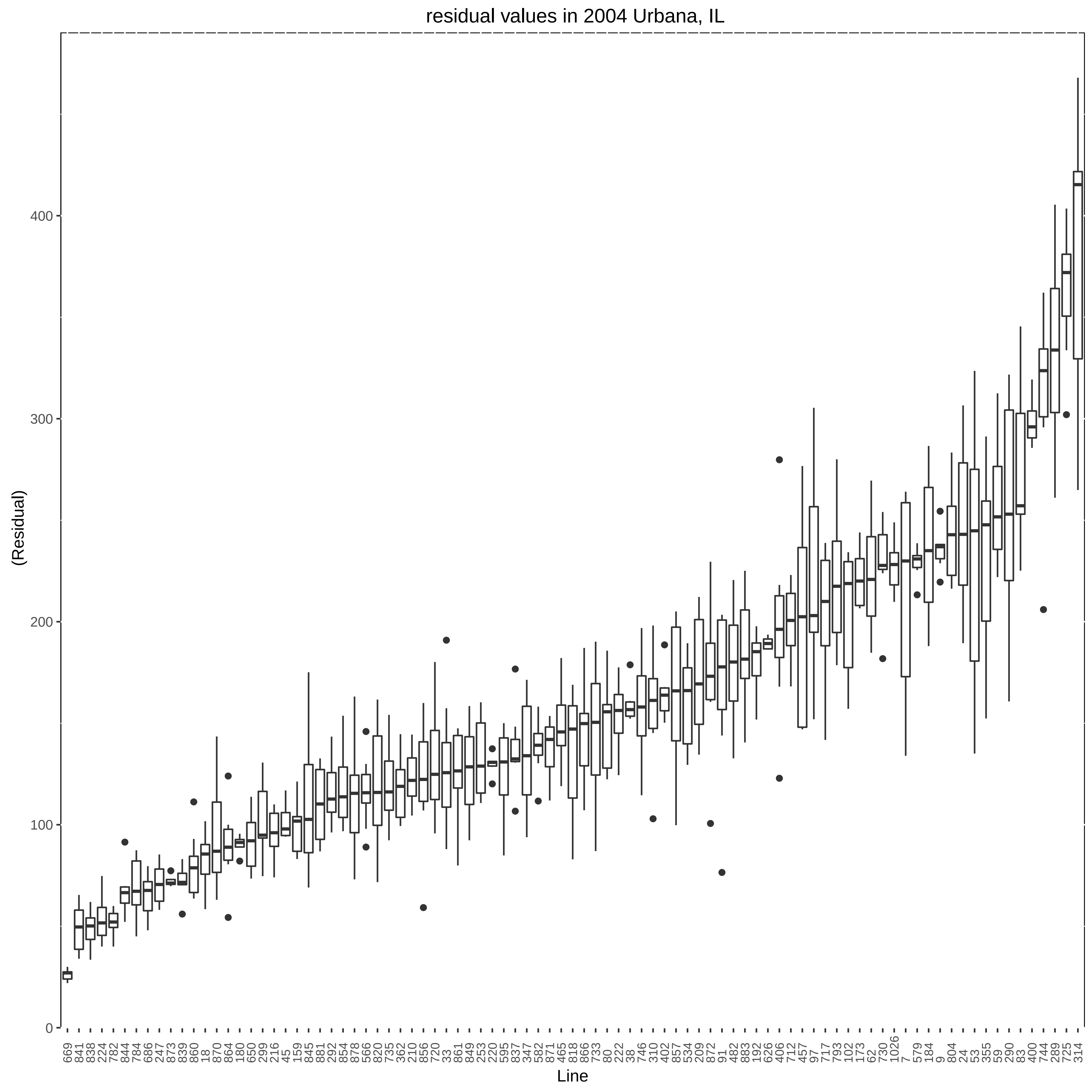
residual values in 2004 Urbana, IL
(Residual)
Line
0
100
200
300
400

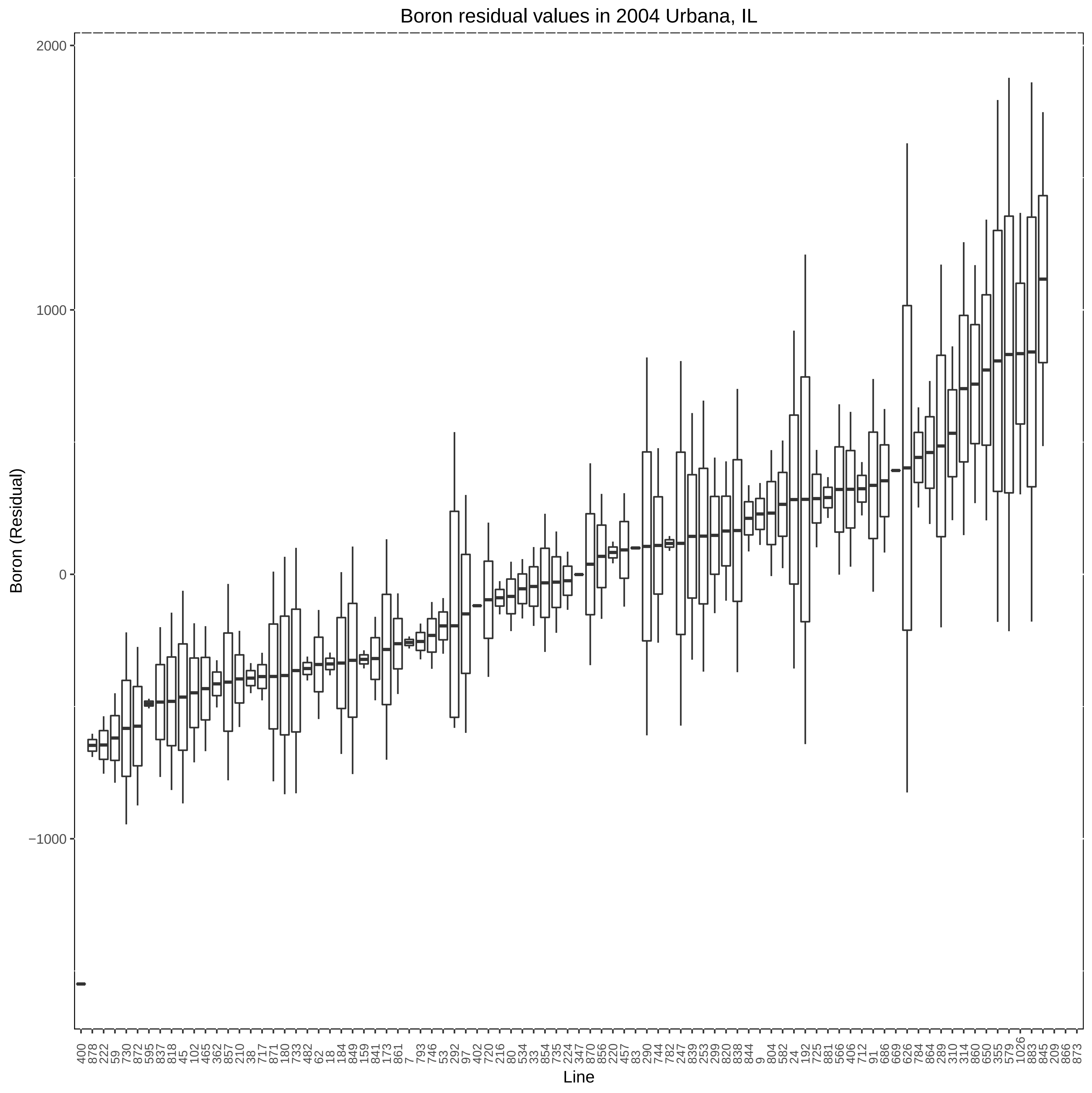
Boron residual values in 2004 Urbana, IL
Boron (Residual)
Line
2000
1000
0
-1000

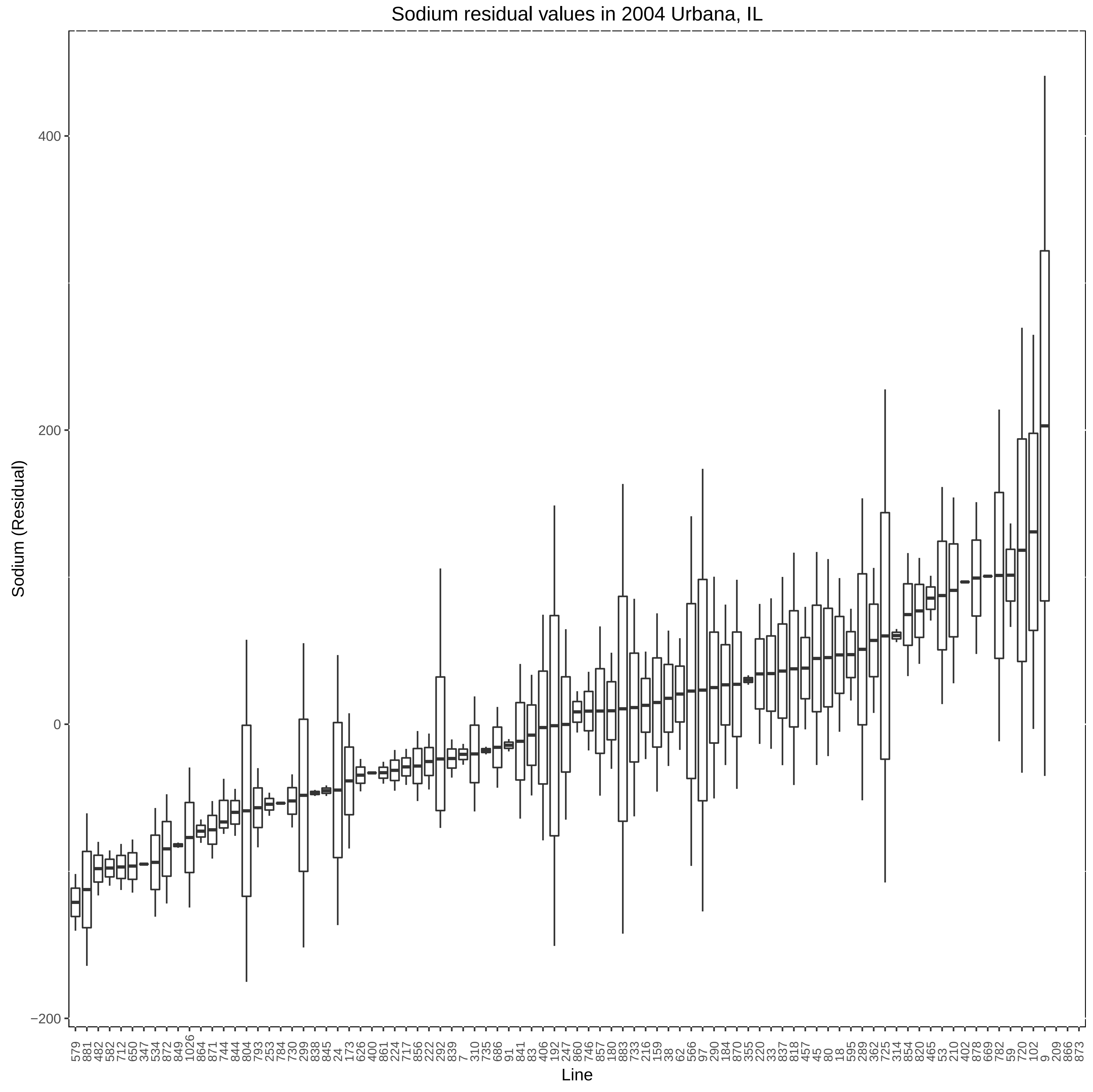
Sodium residual values in 2004 Urbana, IL
Sodium (Residual)
Line
400
200
0
-200

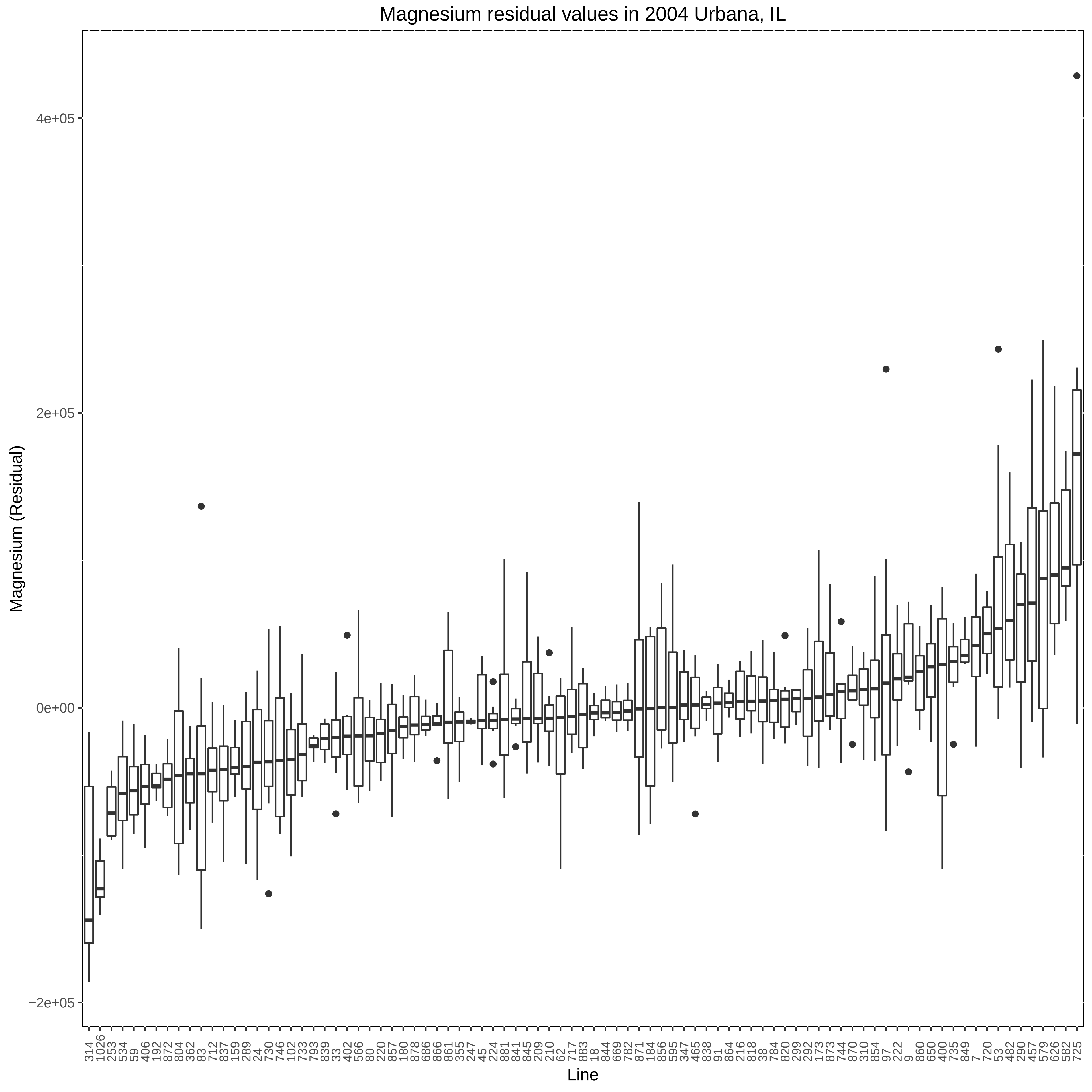
Magnesium residual values in 2004 Urbana, IL
Magnesium (Residual)
Line
4e+05
2e+05
0e+00
-2e+05

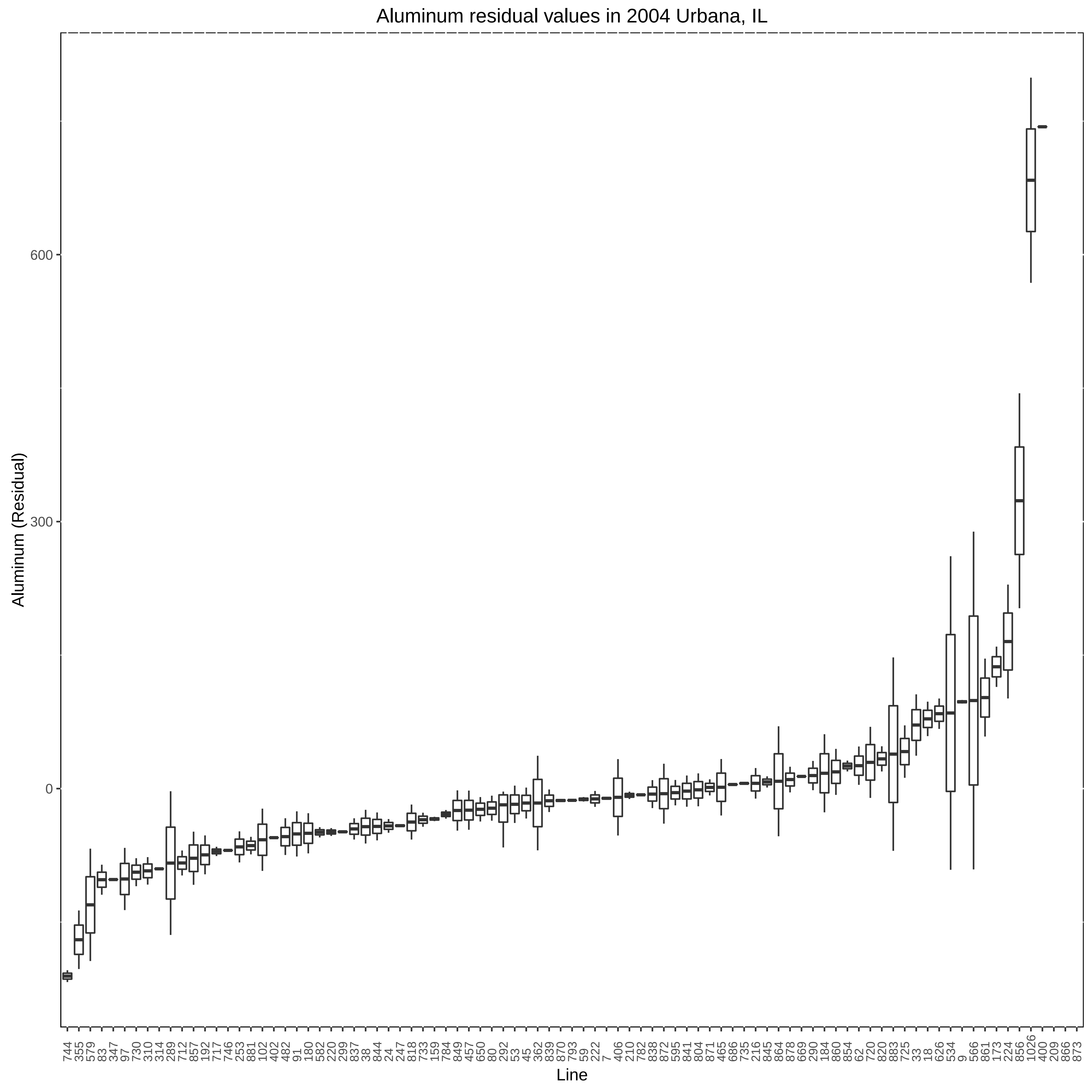
Aluminum residual values in 2004 Urbana, IL
Aluminum (Residual)
600
300
0
Line

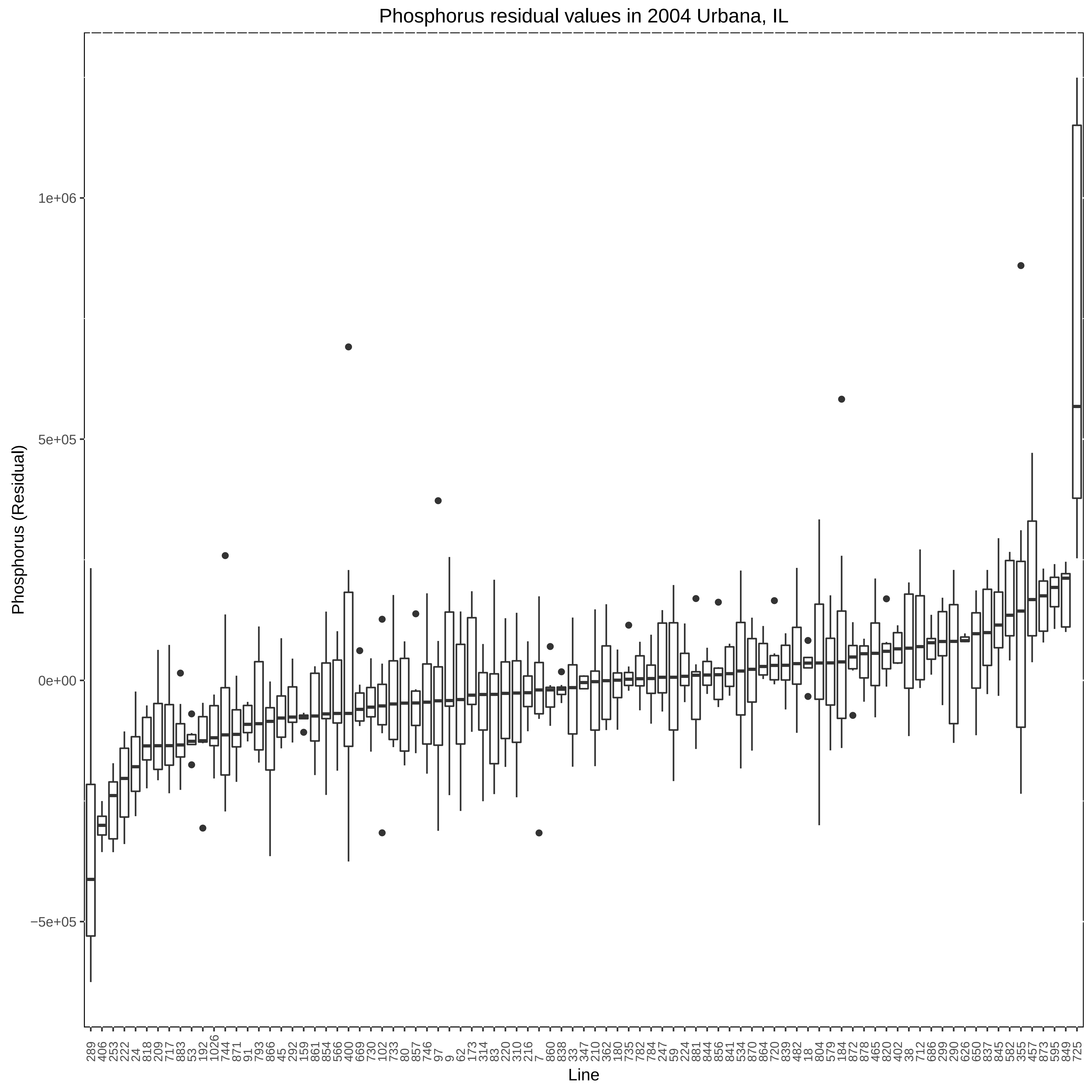
Phosphorus residual values in 2004 Urbana, IL
Phosphorus (Residual)
1e+06
5e+05
0e+00
-5e+05
Line

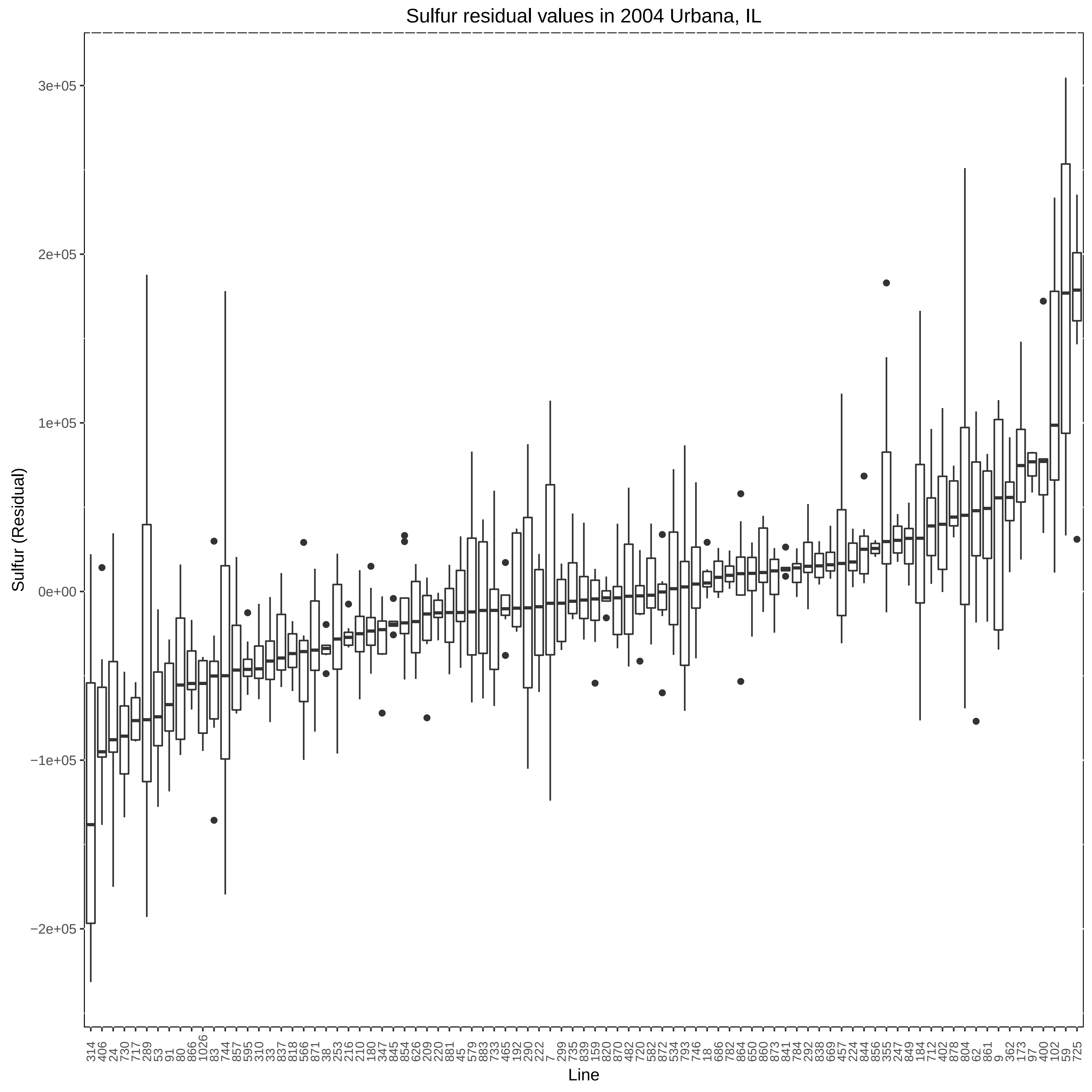
Sulfur residual values in 2004 Urbana, IL
Sulfur (Residual)
3e+05
2e+05
1e+05
0e+00
-1e+05
-2e+05
Line

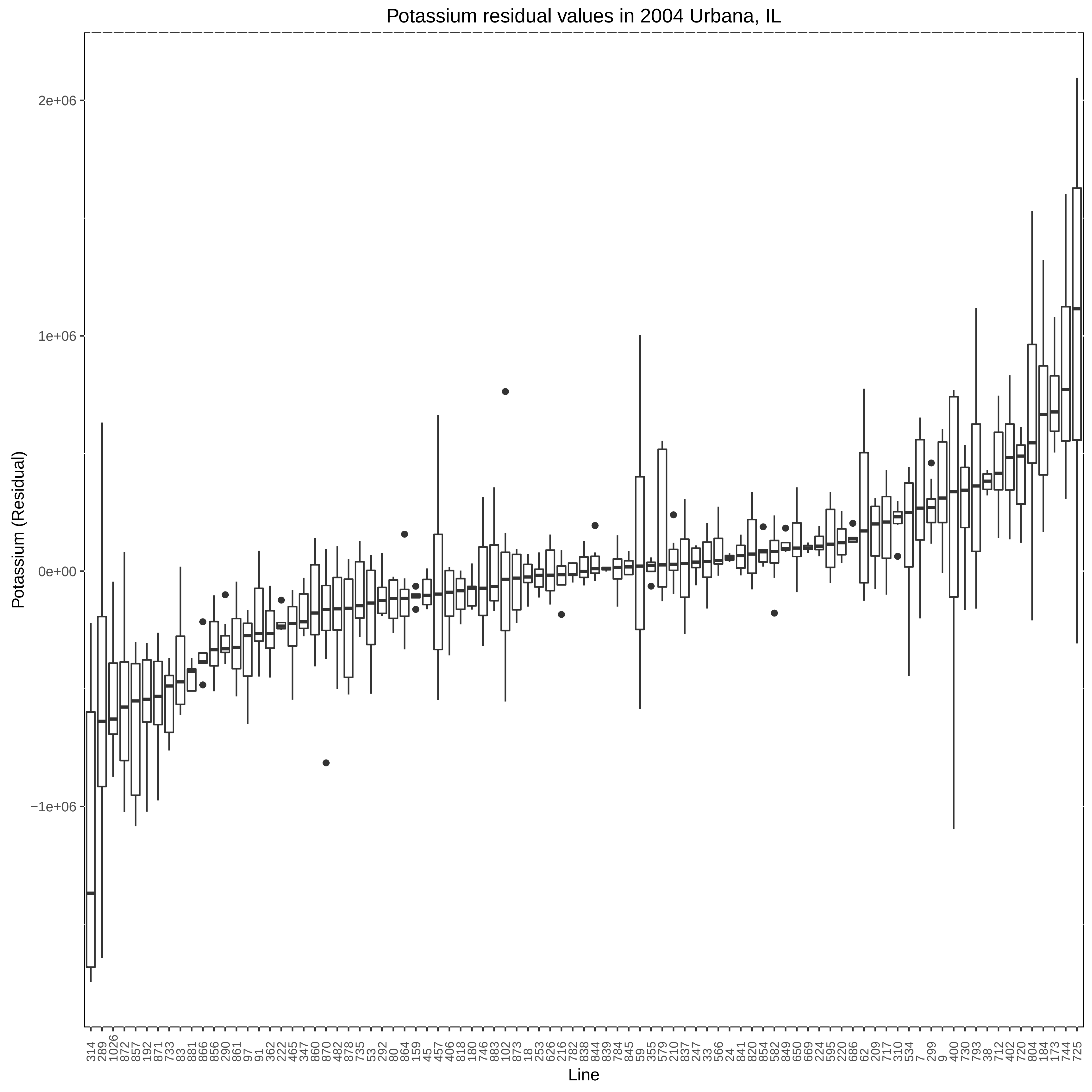
Potassium residual values in 2004 Urbana, IL
Potassium (Residual)
2e+06
1e+06
0e+00
-1e+06
Line

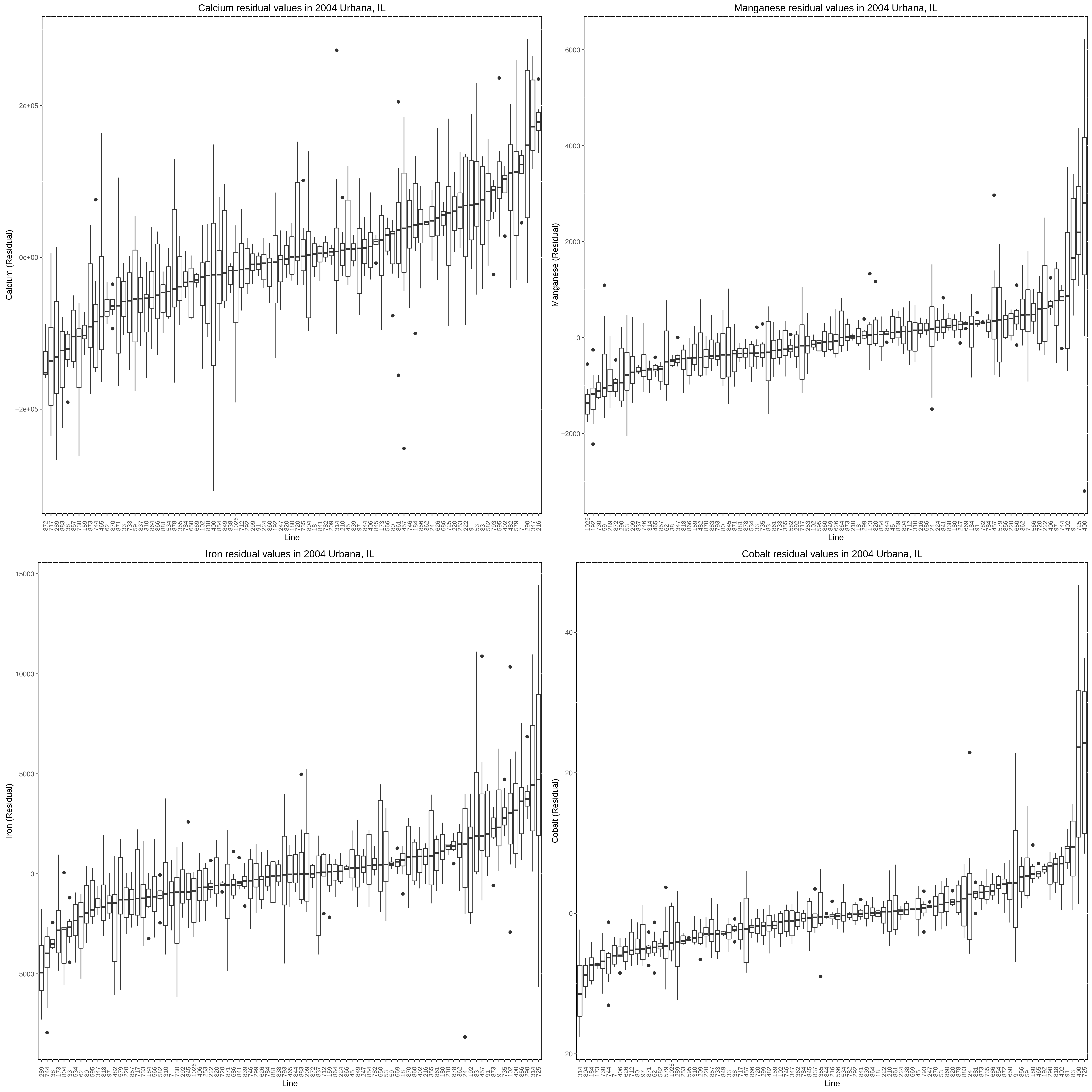
Calcium residual values in 2004 Urbana, IL
Calcium (Residual)
2e+05
0e+00
-2e+05
Line
Manganese residual values in 2004 Urbana, IL
Manganese (Residual)
6000
4000
2000
0
-2000
Line
Iron residual values in 2004 Urbana, IL
Iron (Residual)
15000
10000
5000
0
-5000
Line
Cobalt residual values in 2004 Urbana, IL
Cobalt (Residual)
40
20
0
-20
Line

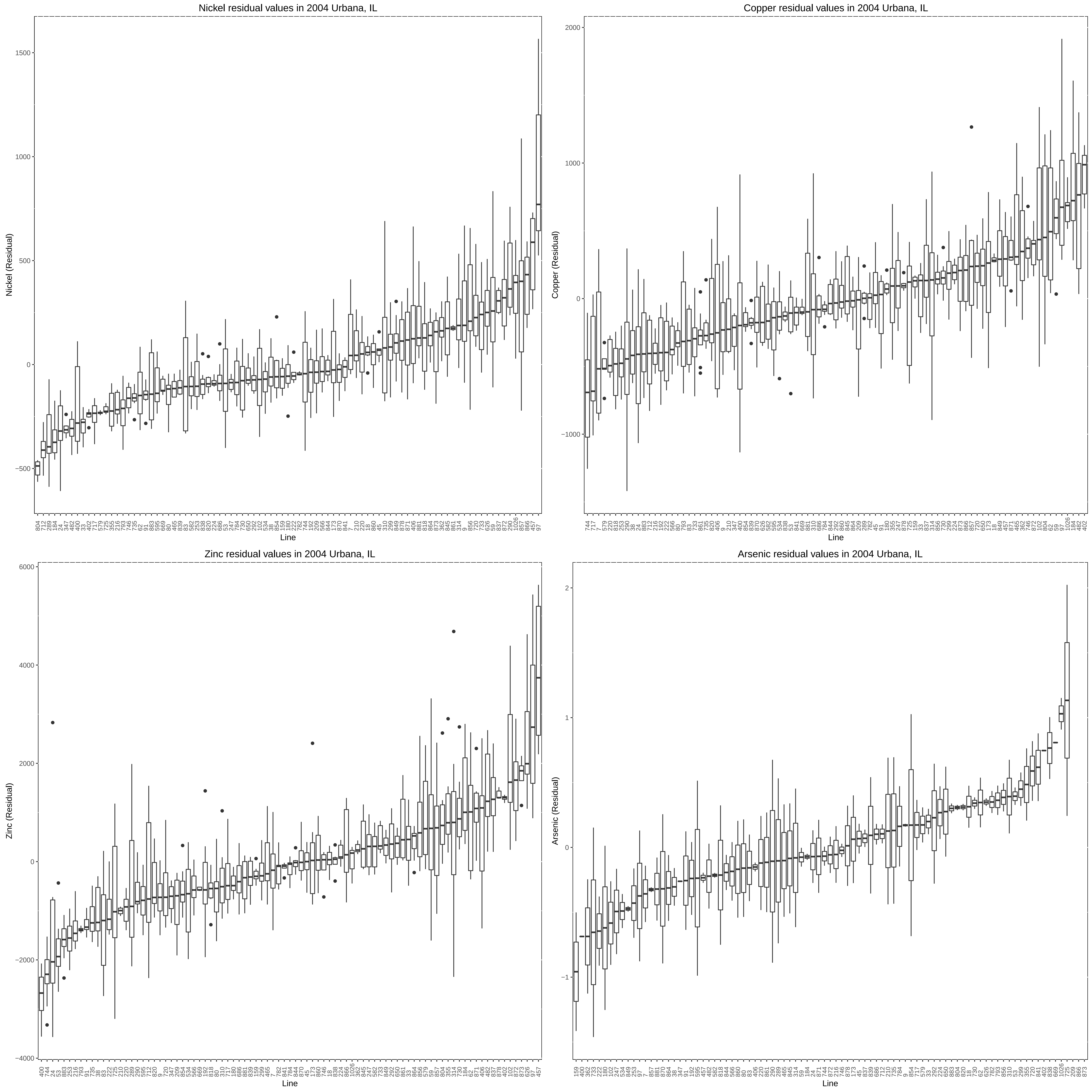
Nickel residual values in 2004 Urbana, IL
Nickel (Residual)
1500
1000
500
0
-500
Line
Copper residual values in 2004 Urbana, IL
Copper (Residual)
2000
1000
0
-1000
Line
Zinc residual values in 2004 Urbana, IL
Zinc (Residual)
6000
4000
2000
0
-2000
-4000
Line
Arsenic residual values in 2004 Urbana, IL
Arsenic (Residual)
2
1
0
-1
Line

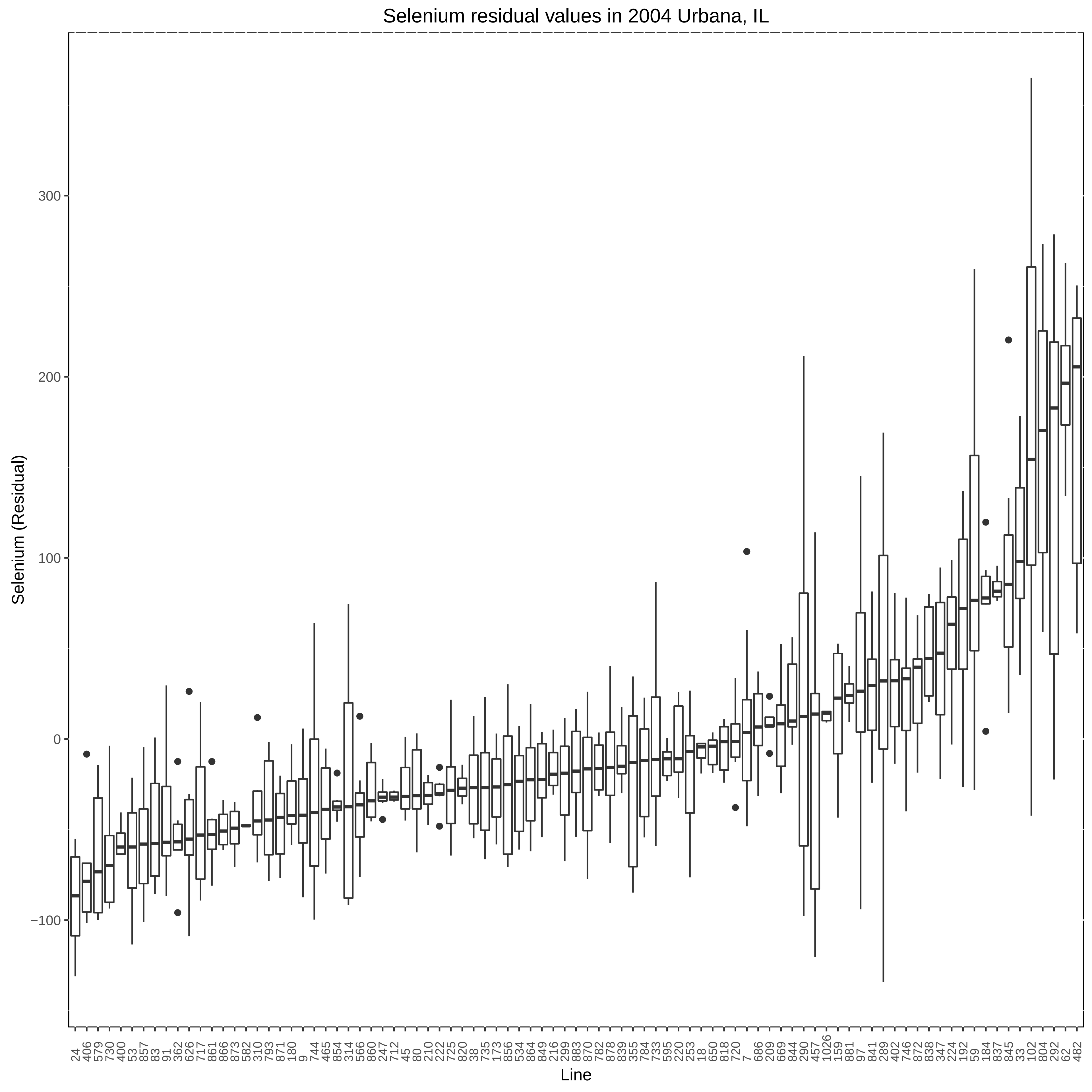
Selenium residual values in 2004 Urbana, IL
Selenium (Residual)
300
200
100
0
−100
Line

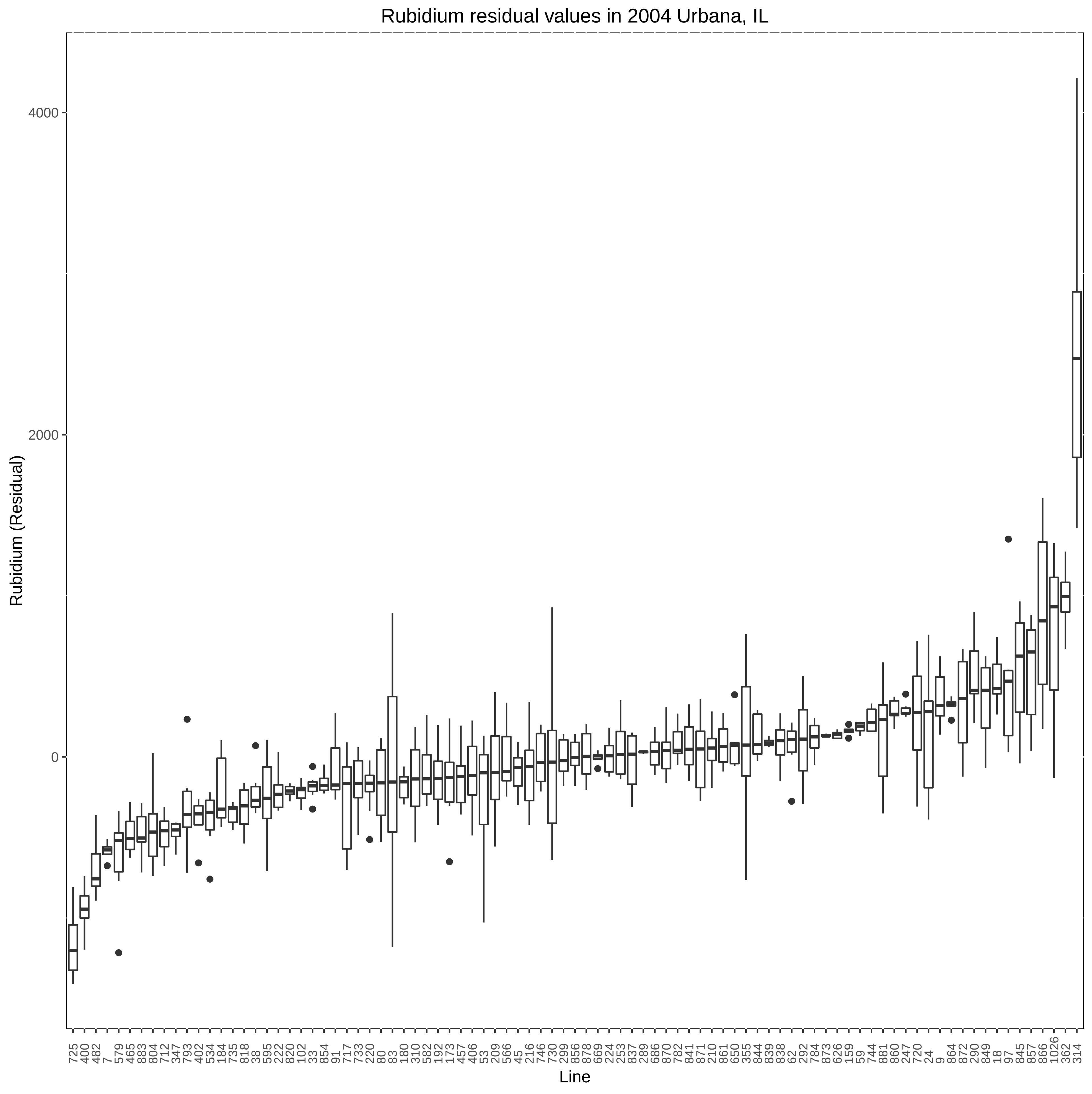
Rubidium residual values in 2004 Urbana, IL
Rubidium (Residual)
4000
2000
0
Line

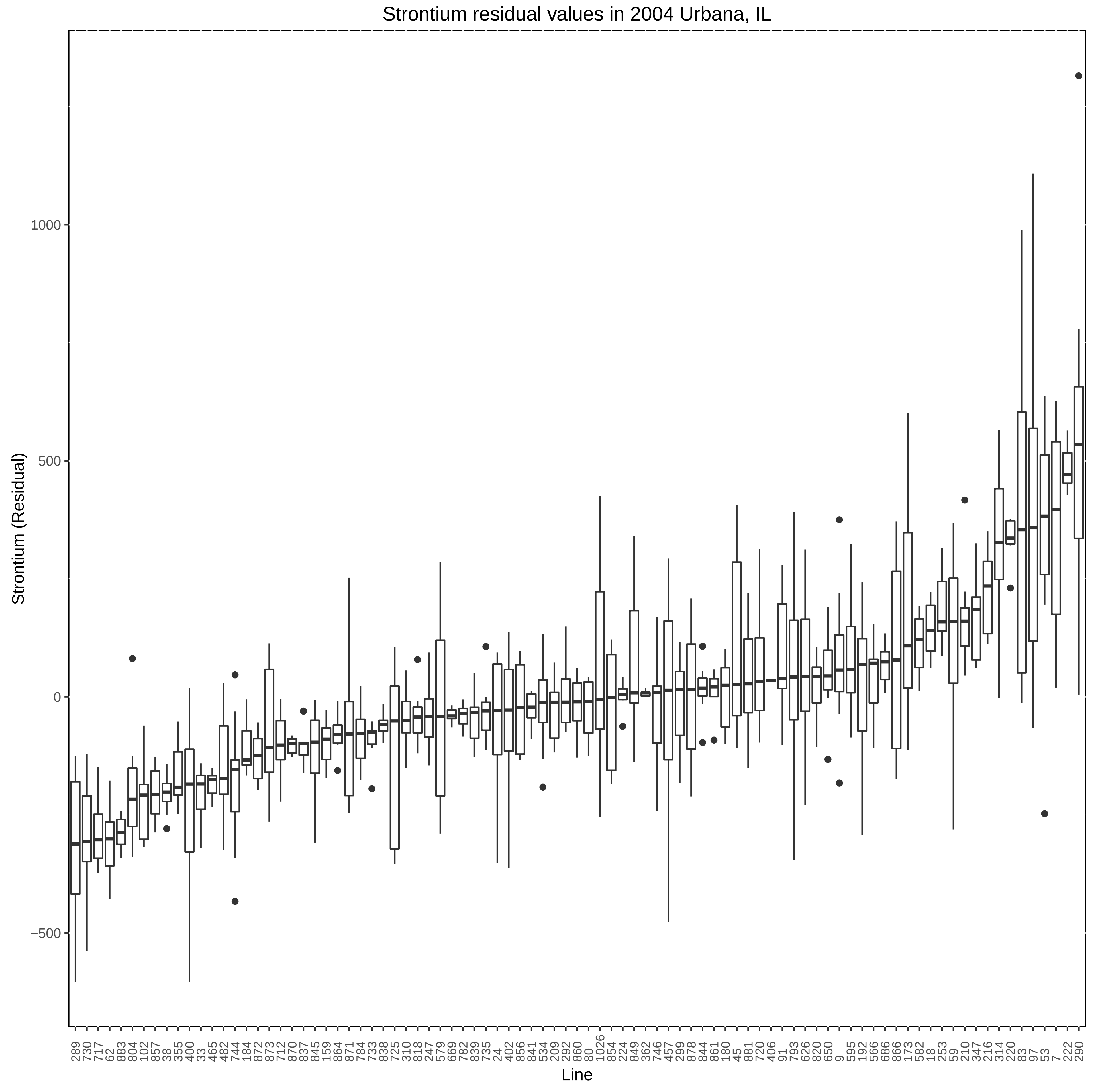
Strontium residual values in 2004 Urbana, IL
Strontium (Residual)
1000
500
0
−500
Line

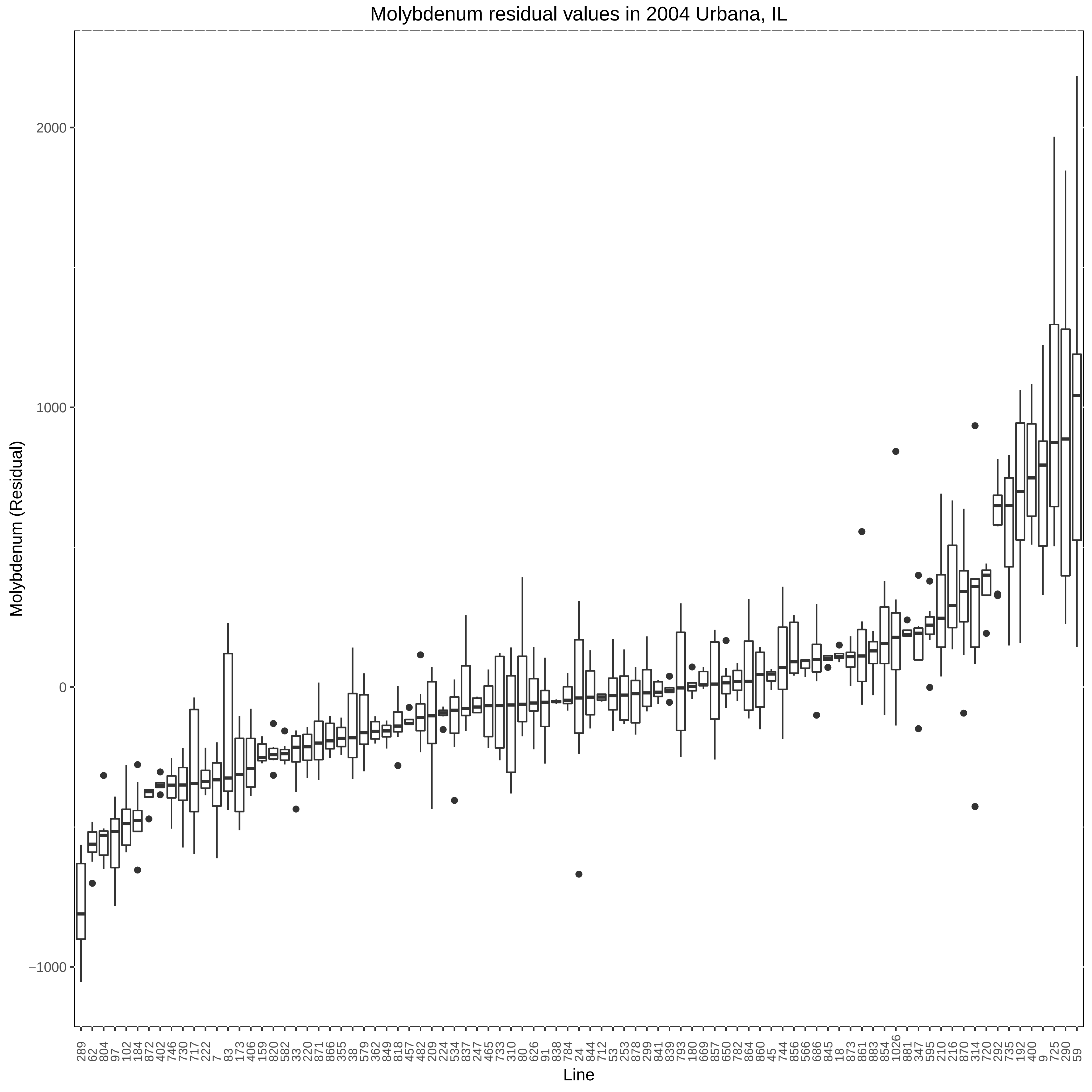
Molybdenum residual values in 2004 Urbana, IL
Molybdenum (Residual)
2000
1000
0
−1000
Line

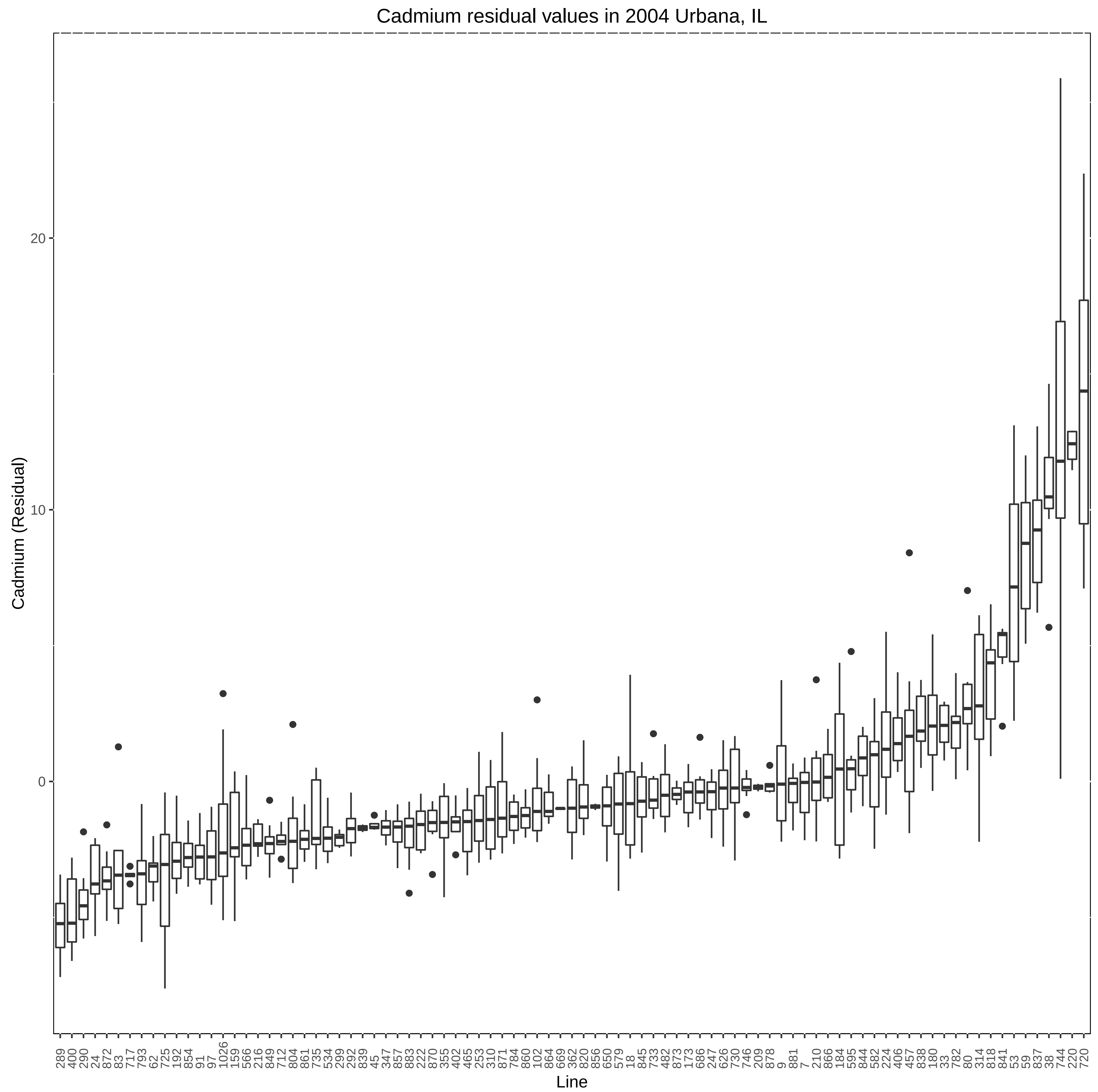
Cadmium residual values in 2004 Urbana, IL
Cadmium (Residual)
Line
20
10
0
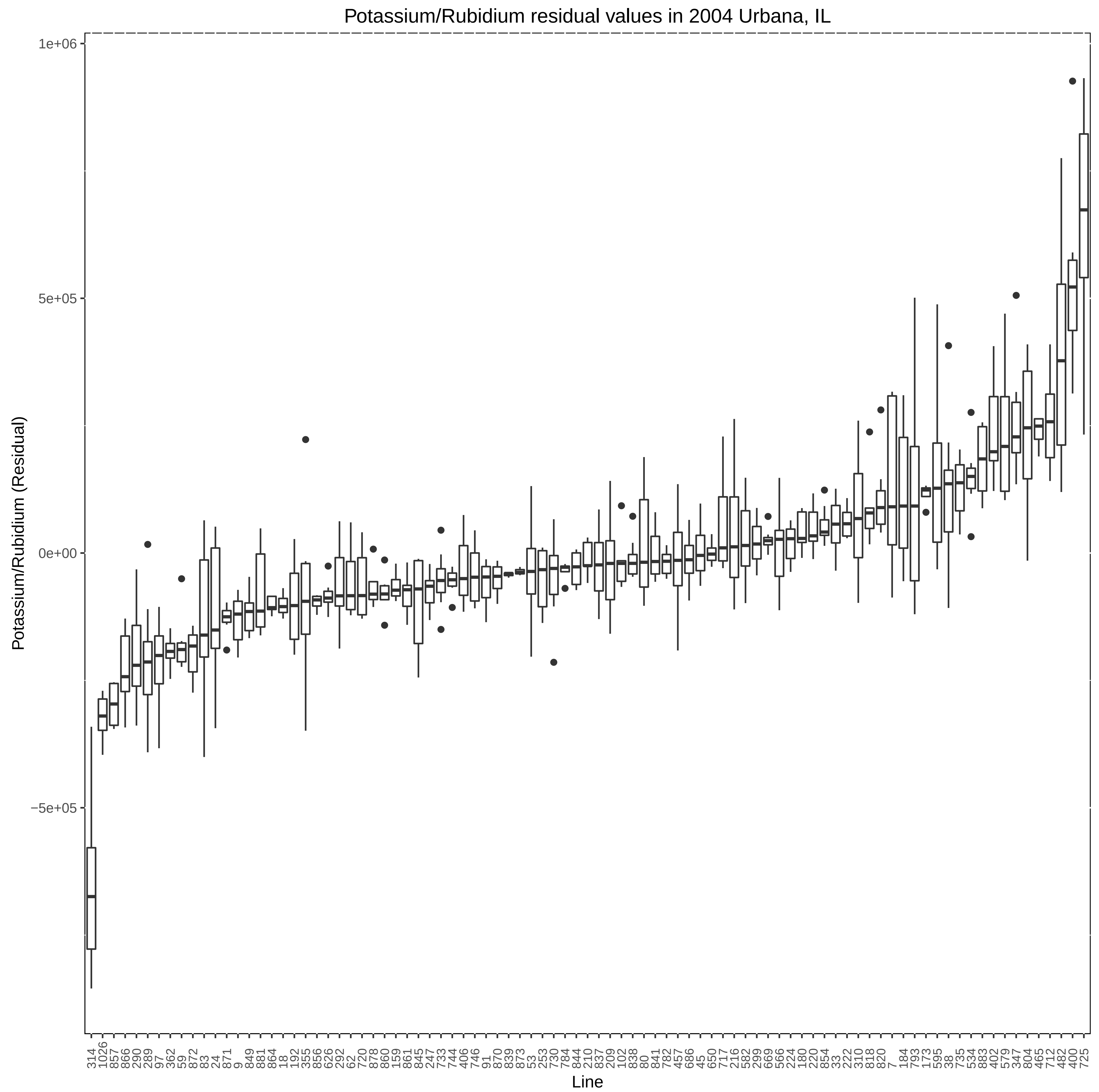
Potassium/Rubidium residual values in 2004 Urbana, IL
Potassium/Rubidium (Residual)
Line
1e+06
5e+05
0e+00
-5e+05
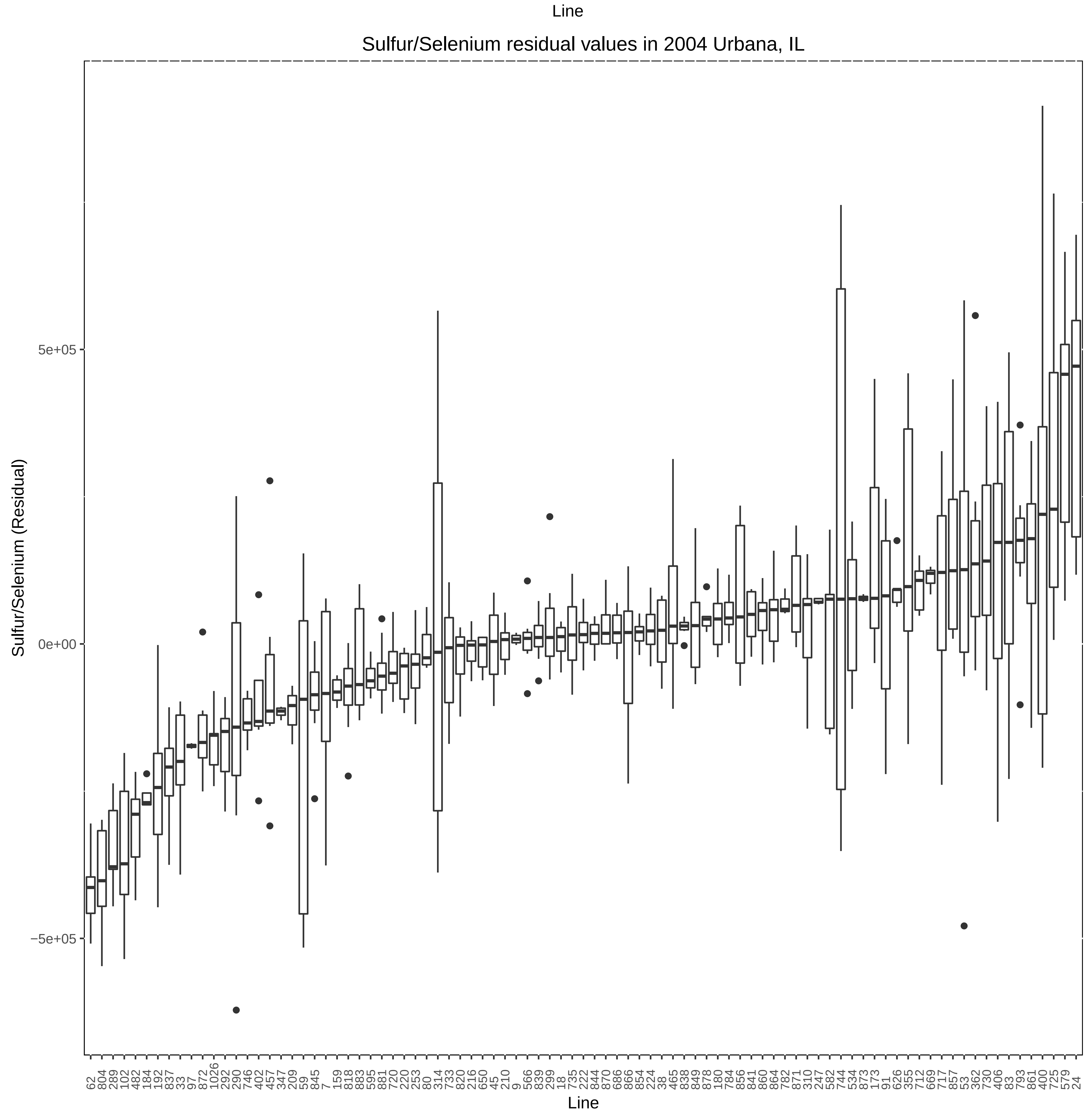
Sulfur/Selenium residual values in 2004 Urbana, IL
Sulfur/Selenium (Residual)
Line
5e+05
0e+00
-5e+05
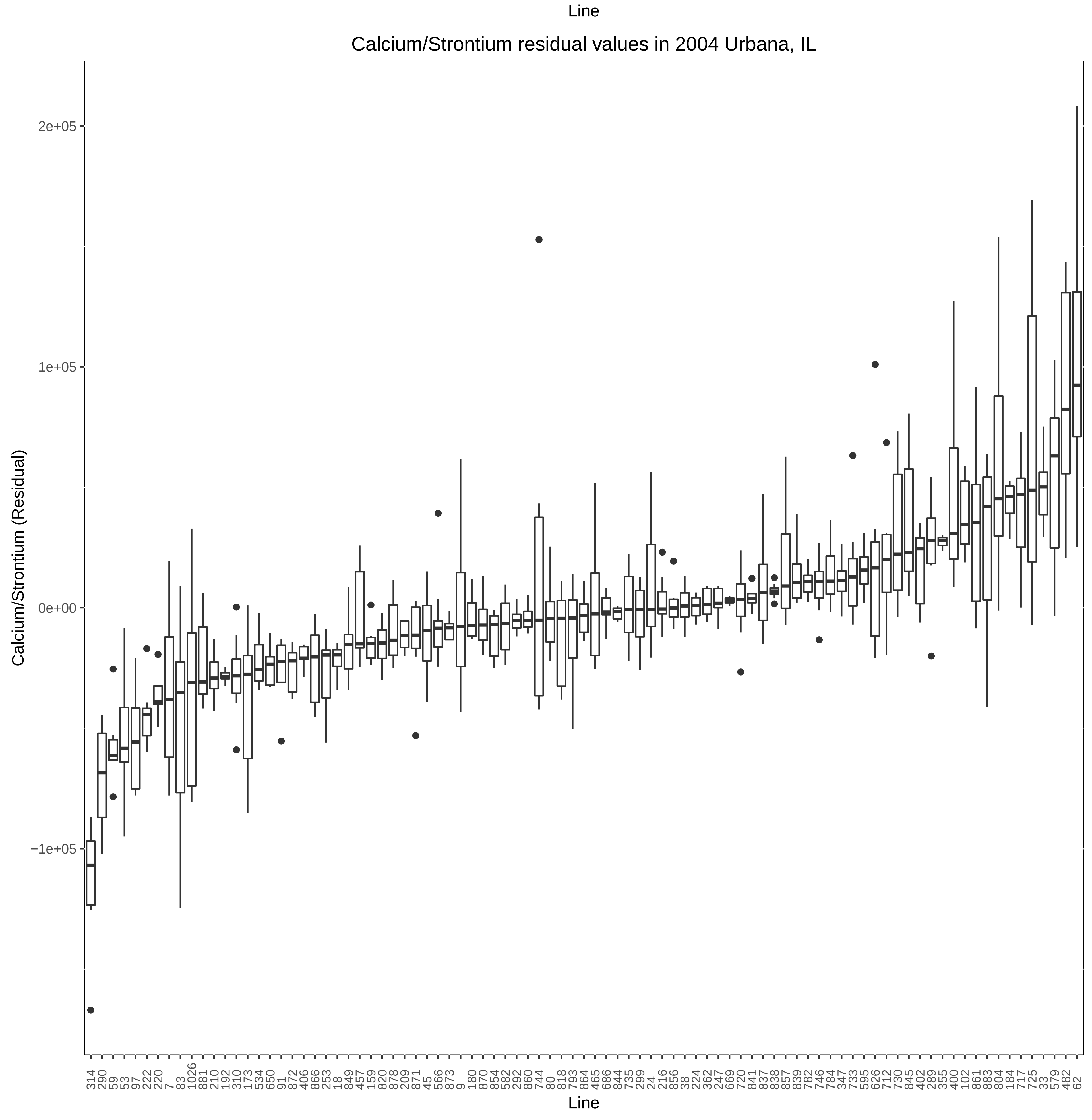
Calcium/Strontium residual values in 2004 Urbana, IL
Calcium/Strontium (Residual)
Line
2e+05
1e+05
0e+00
-1e+05

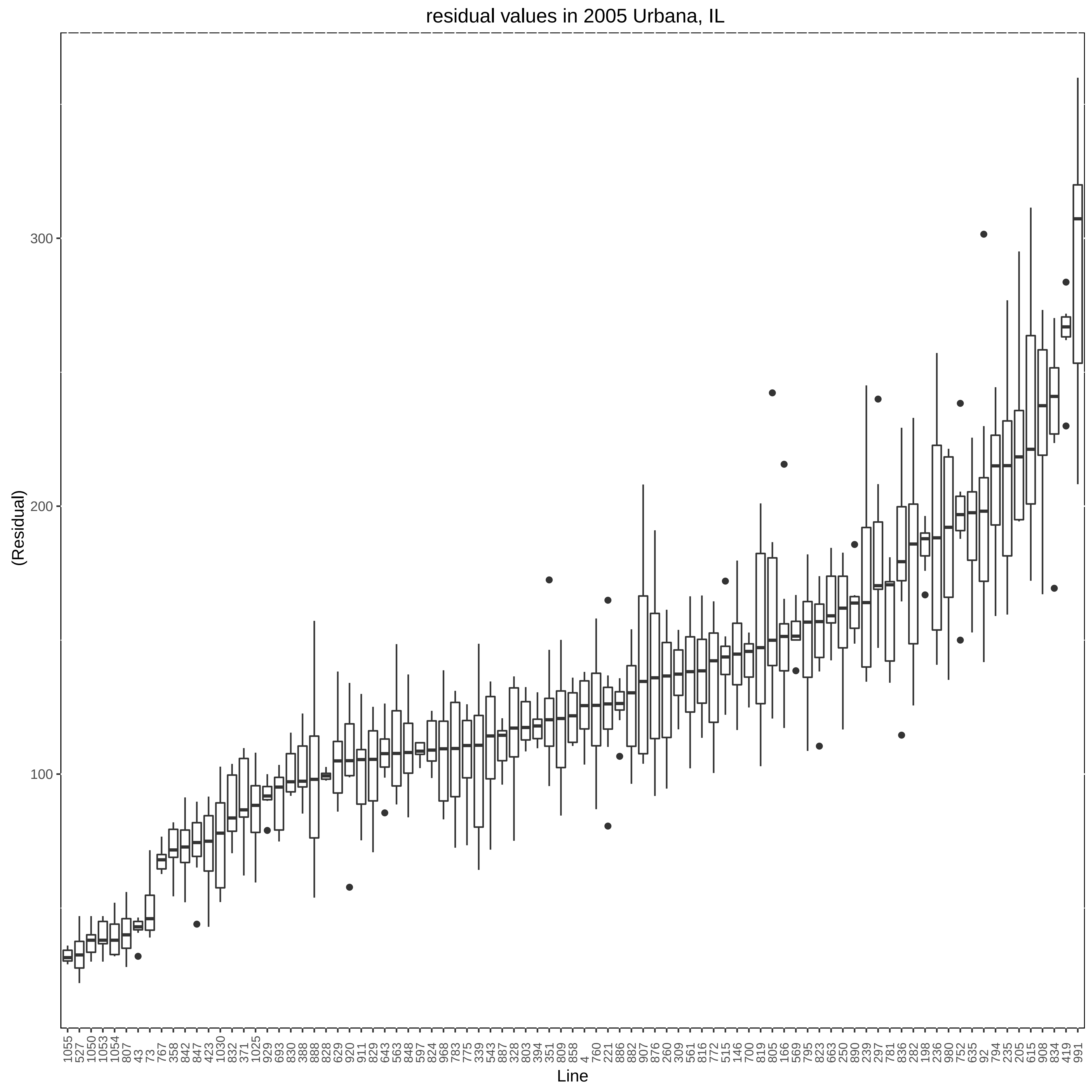
residual values in 2005 Urbana, IL
(Residual)
300
200
100
Line

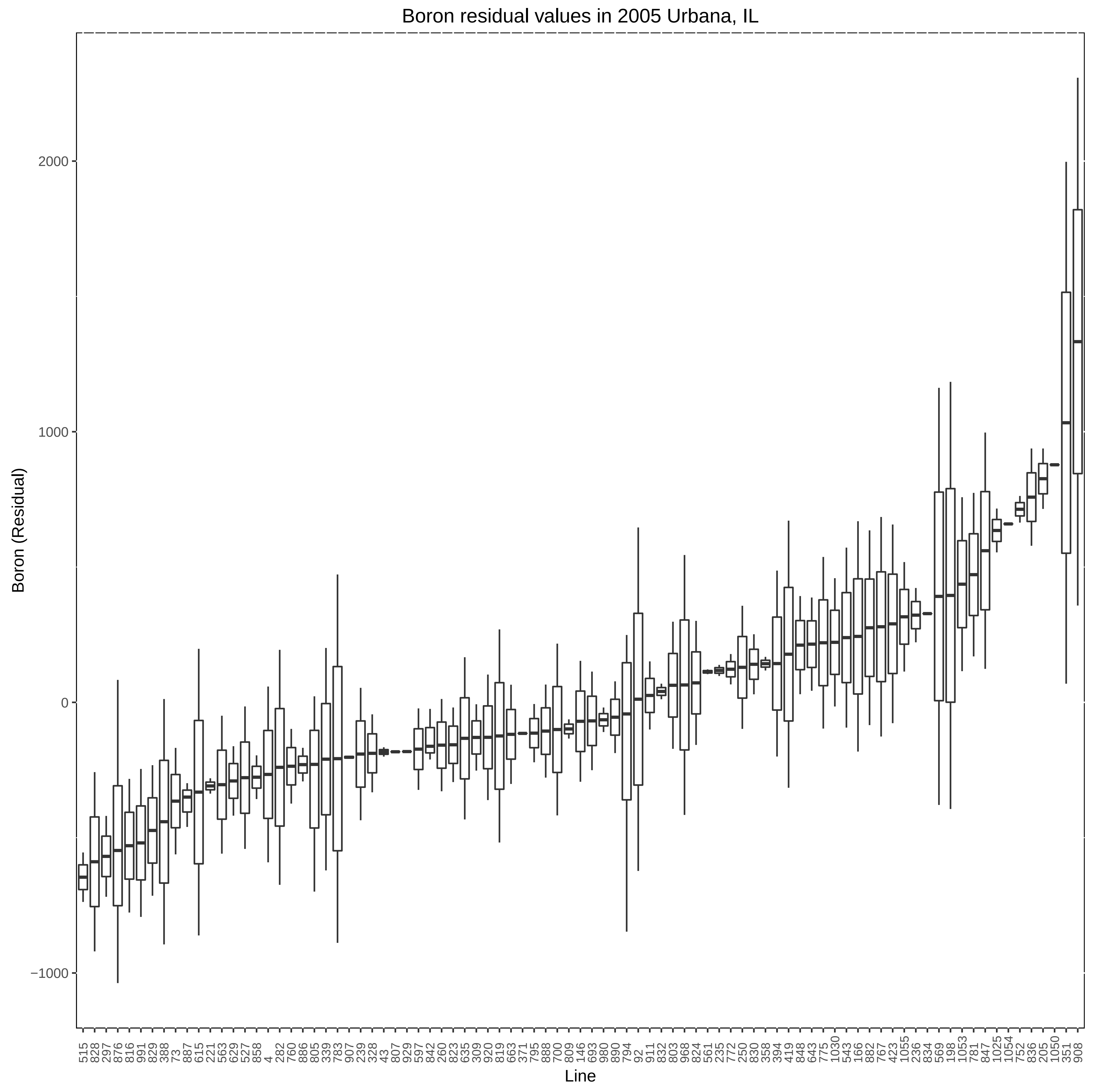
Boron residual values in 2005 Urbana, IL
Boron (Residual)
2000
1000
0
−1000
Line

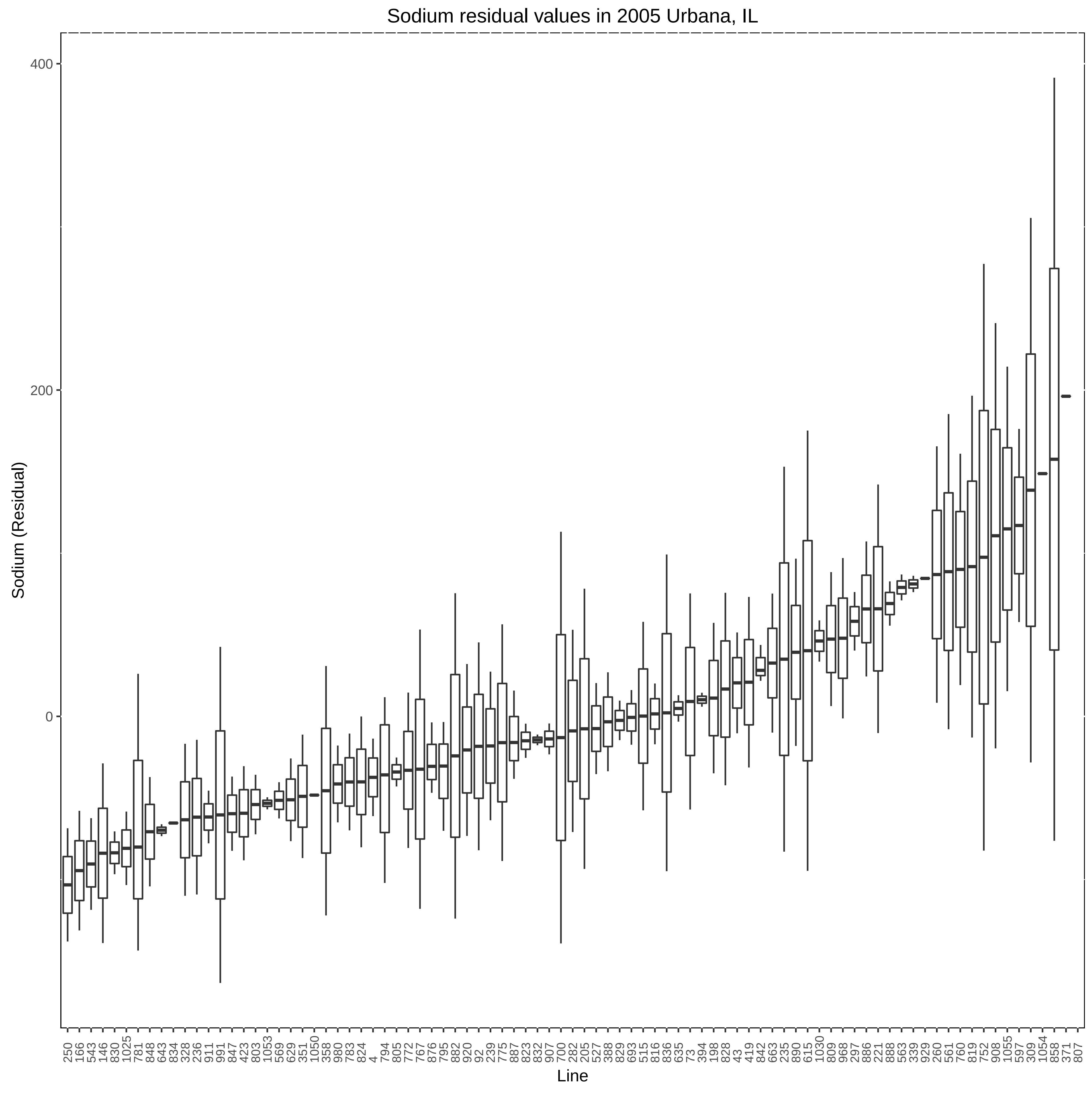
Sodium residual values in 2005 Urbana, IL
Sodium (Residual)
400
200
0
Line

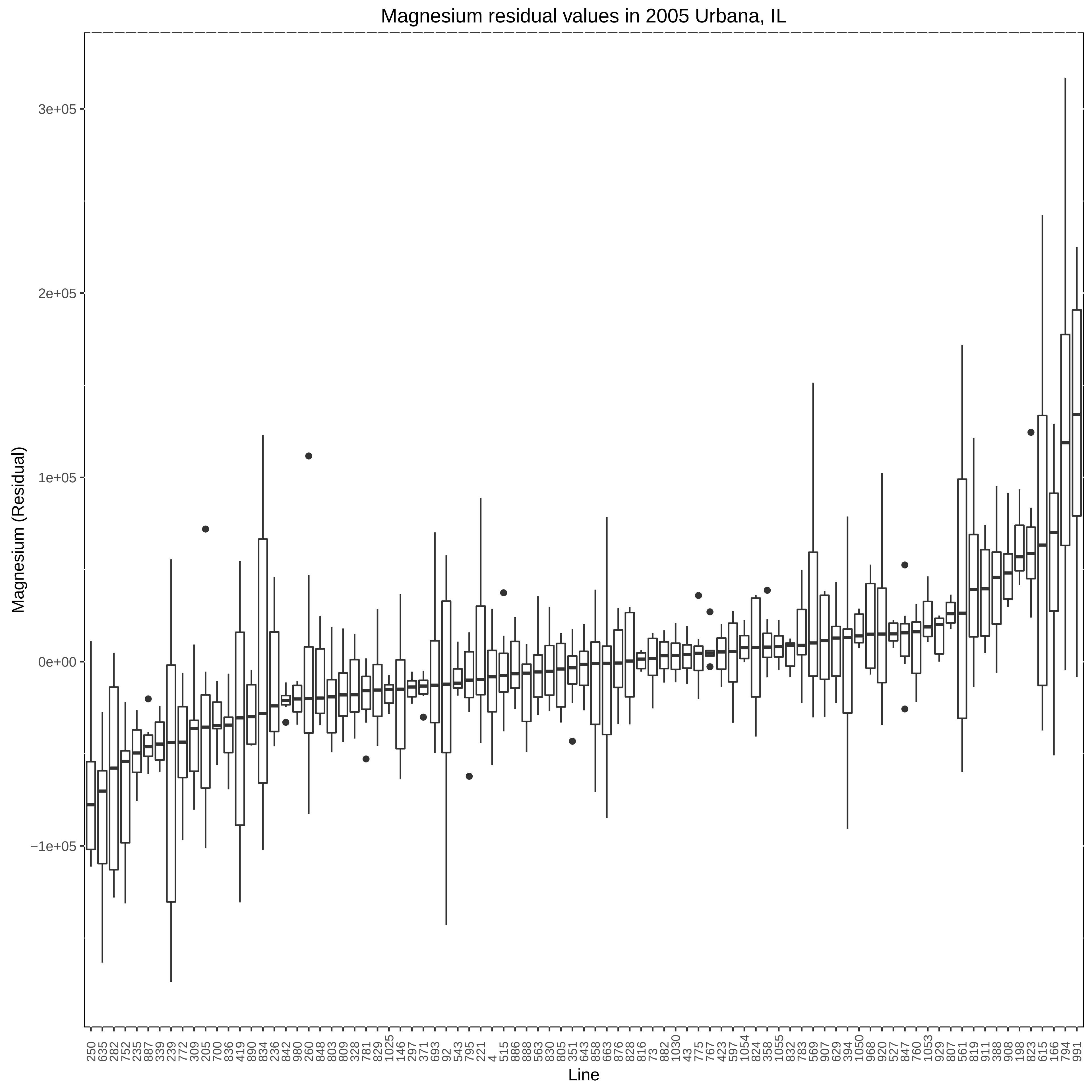
Magnesium residual values in 2005 Urbana, IL
Magnesium (Residual)
3e+05
2e+05
1e+05
0e+00
−1e+05
Line

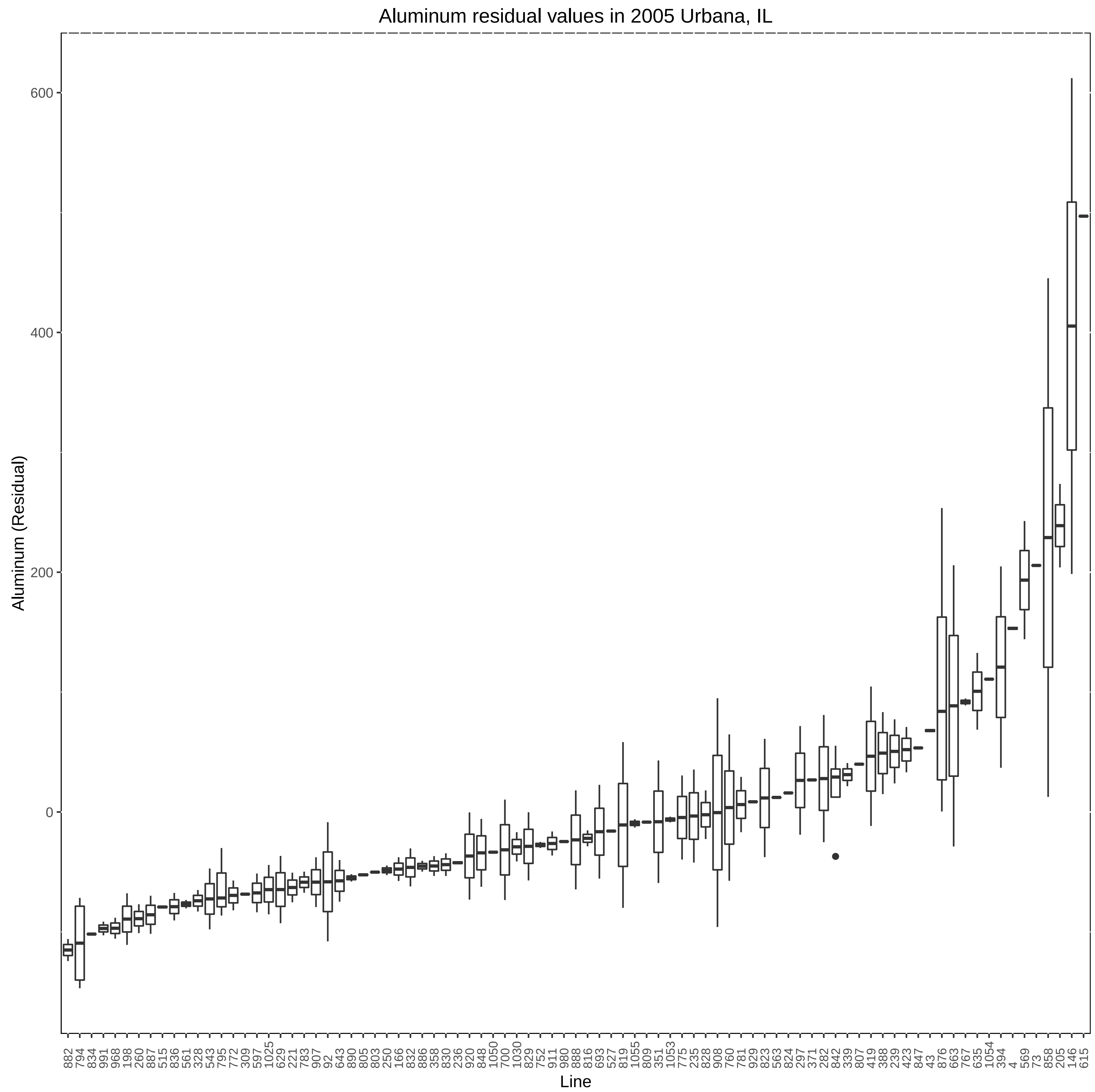
Aluminum residual values in 2005 Urbana, IL
Aluminum (Residual)
600
400
200
0
Line

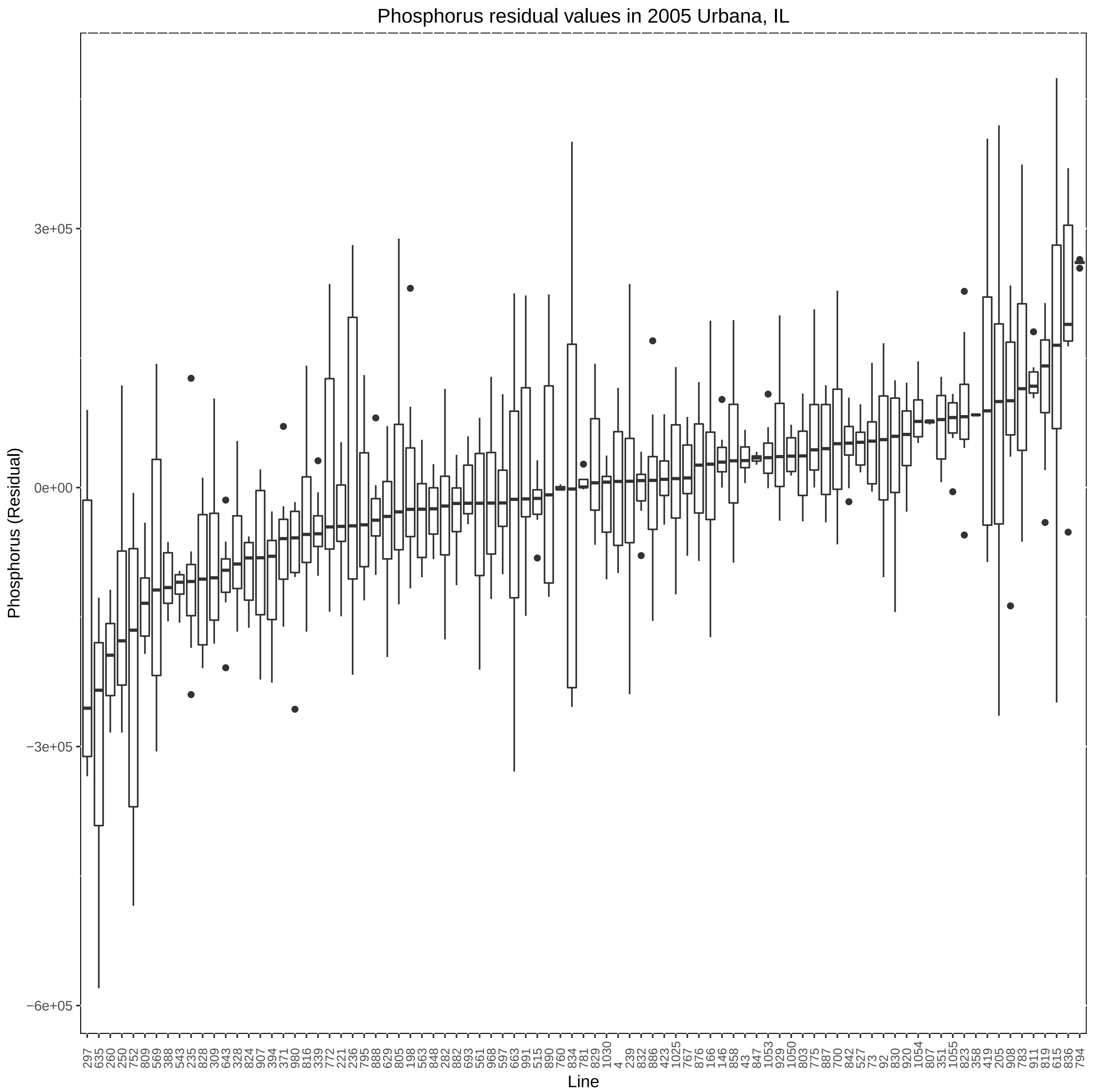
Phosphorus residual values in 2005 Urbana, IL
Phosphorus (Residual)
3e+05
0e+00
-3e+05
-6e+05
Line

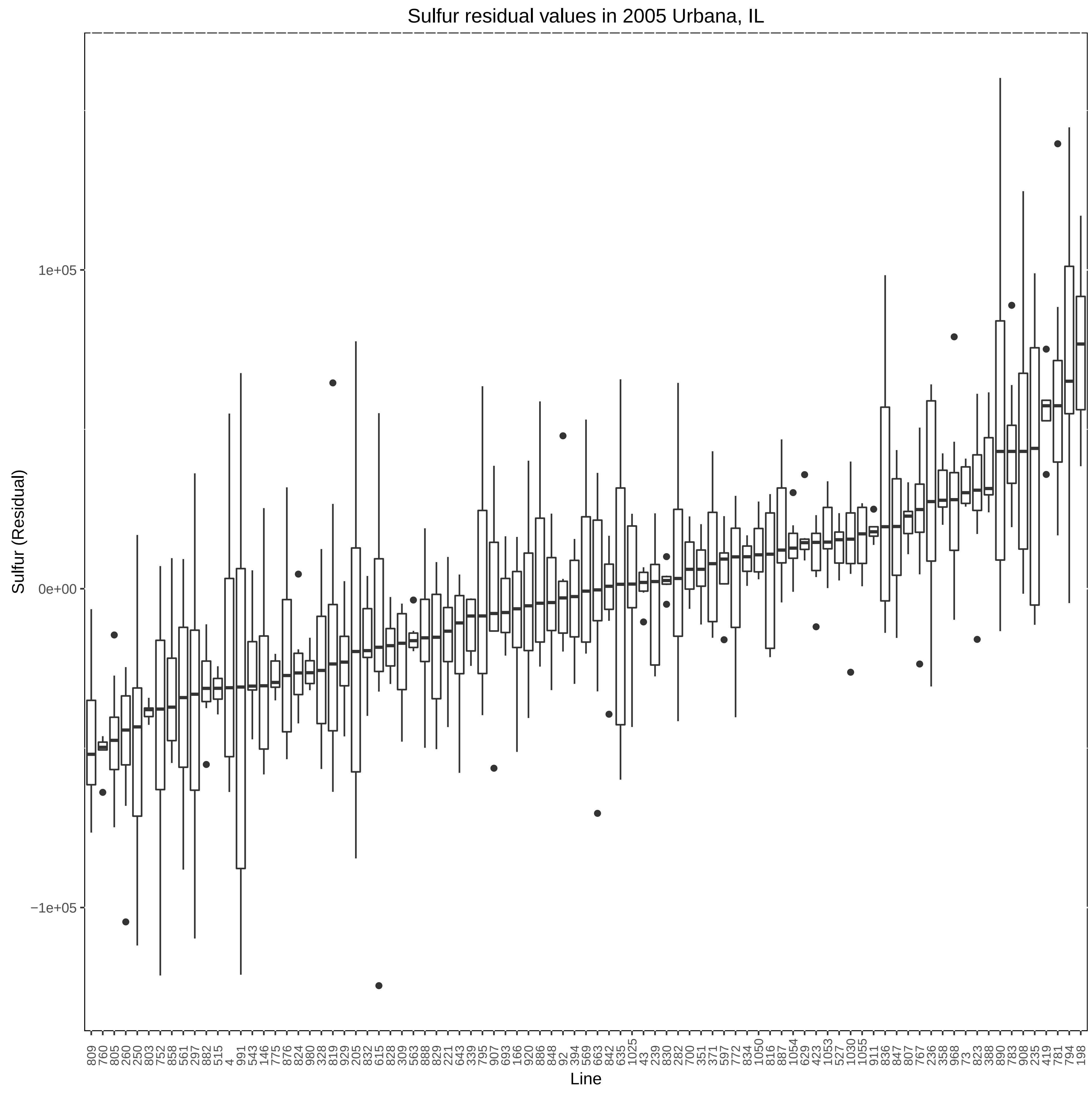
Sulfur residual values in 2005 Urbana, IL
Sulfur (Residual)
1e+05
0e+00
-1e+05
Line

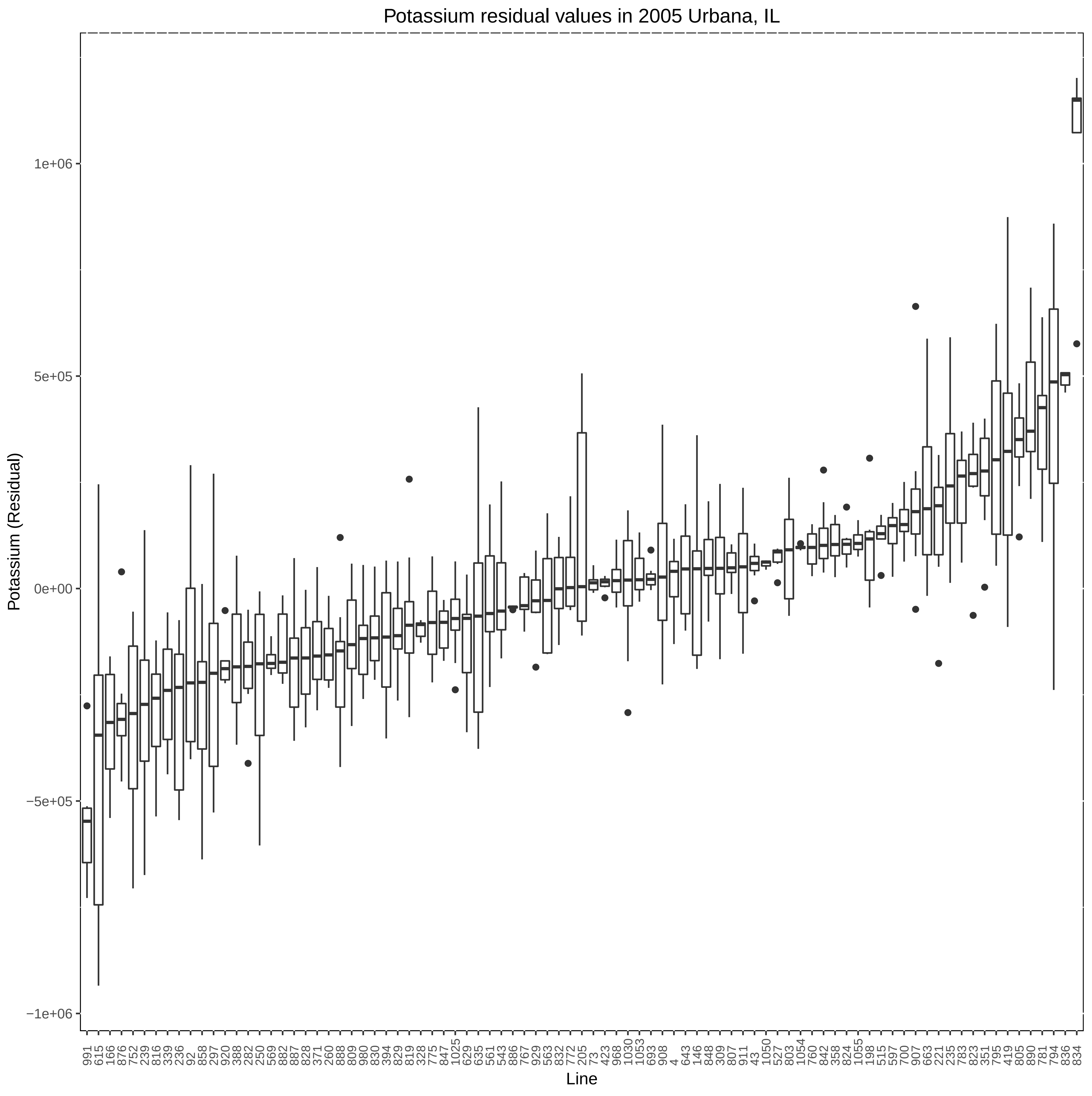
Potassium residual values in 2005 Urbana, IL
Potassium (Residual)
1e+06
5e+05
0e+00
-5e+05
-1e+06
Line

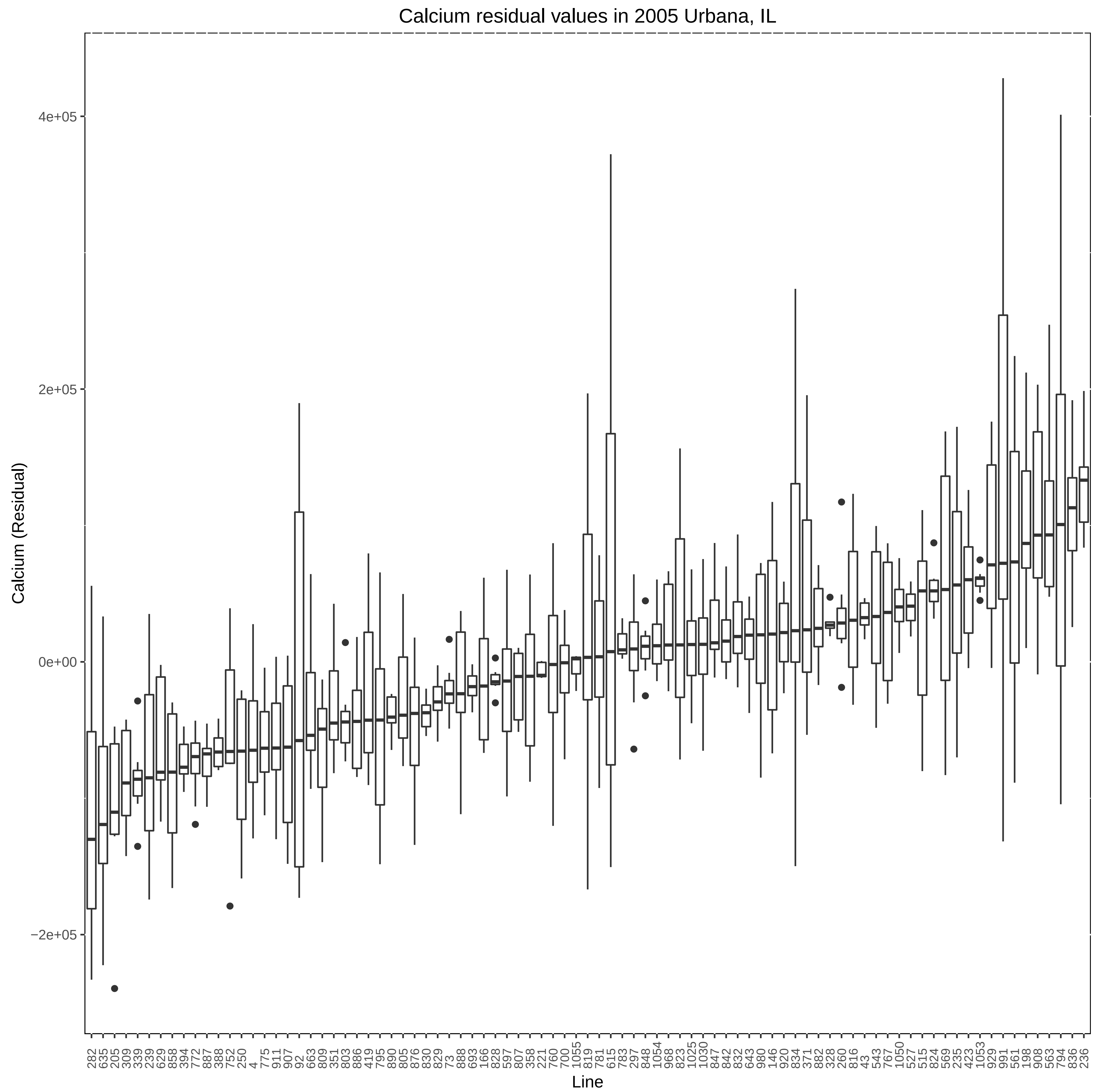
Calcium residual values in 2005 Urbana, IL
Calcium (Residual)
4e+05
2e+05
0e+00
-2e+05
Line

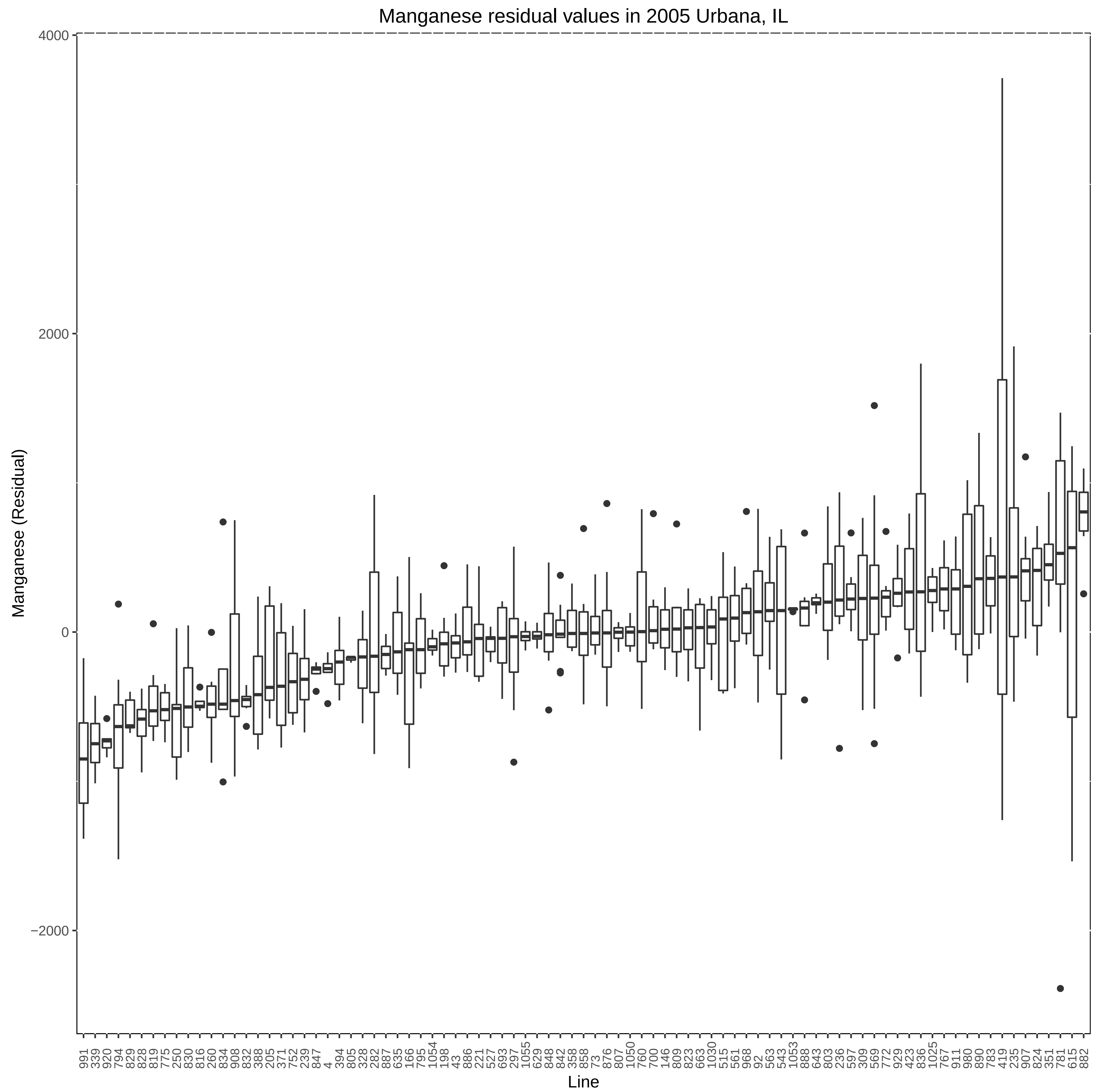
Manganese residual values in 2005 Urbana, IL
Manganese (Residual)
4000
2000
0
-2000
Line

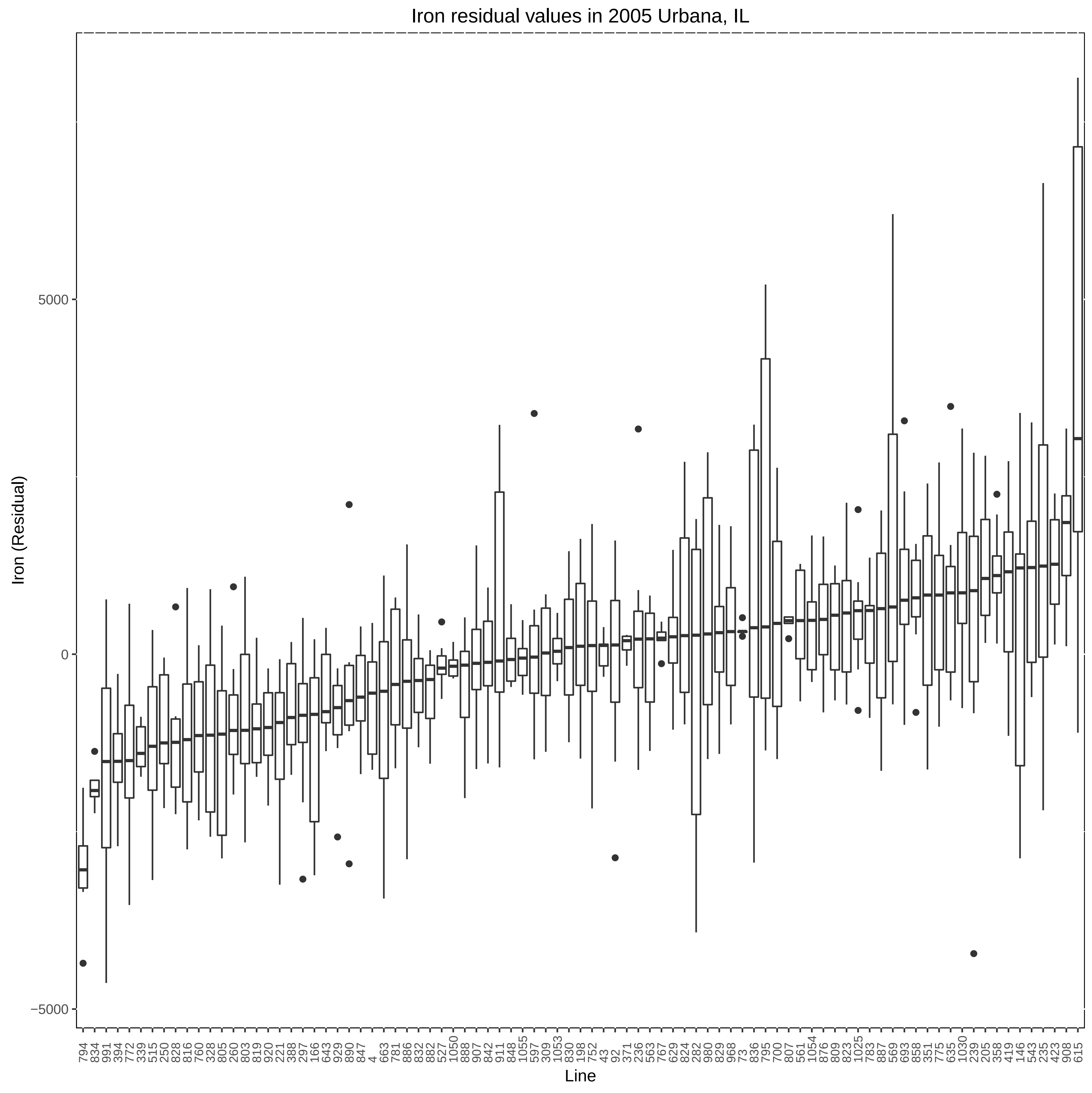
Iron residual values in 2005 Urbana, IL
Iron (Residual)
5000
0
-5000
Line

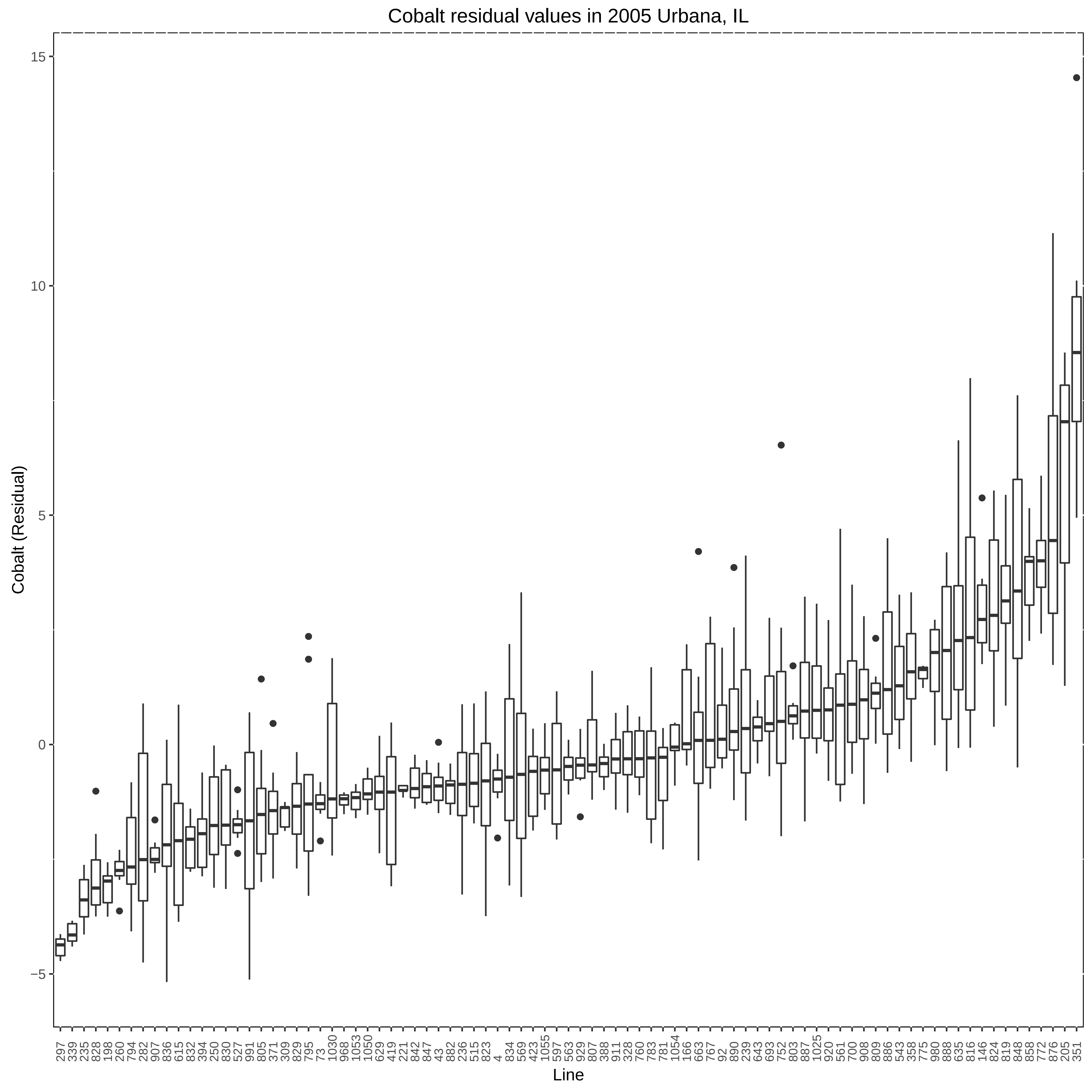
Cobalt residual values in 2005 Urbana, IL
Cobalt (Residual)
15
10
5
0
-5
Line

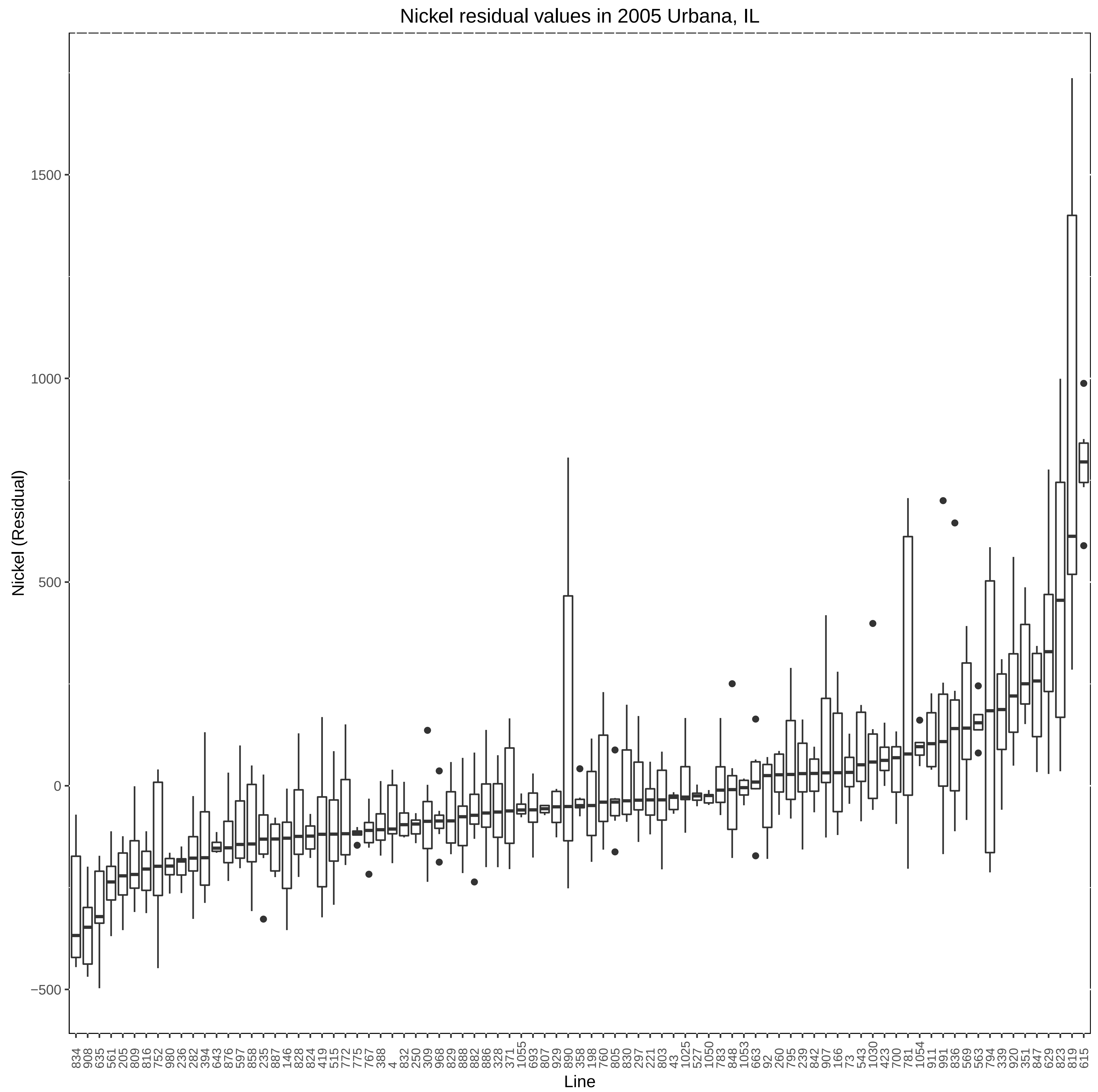
Nickel residual values in 2005 Urbana, IL
Nickel (Residual)
1500
1000
500
0
-500
Line

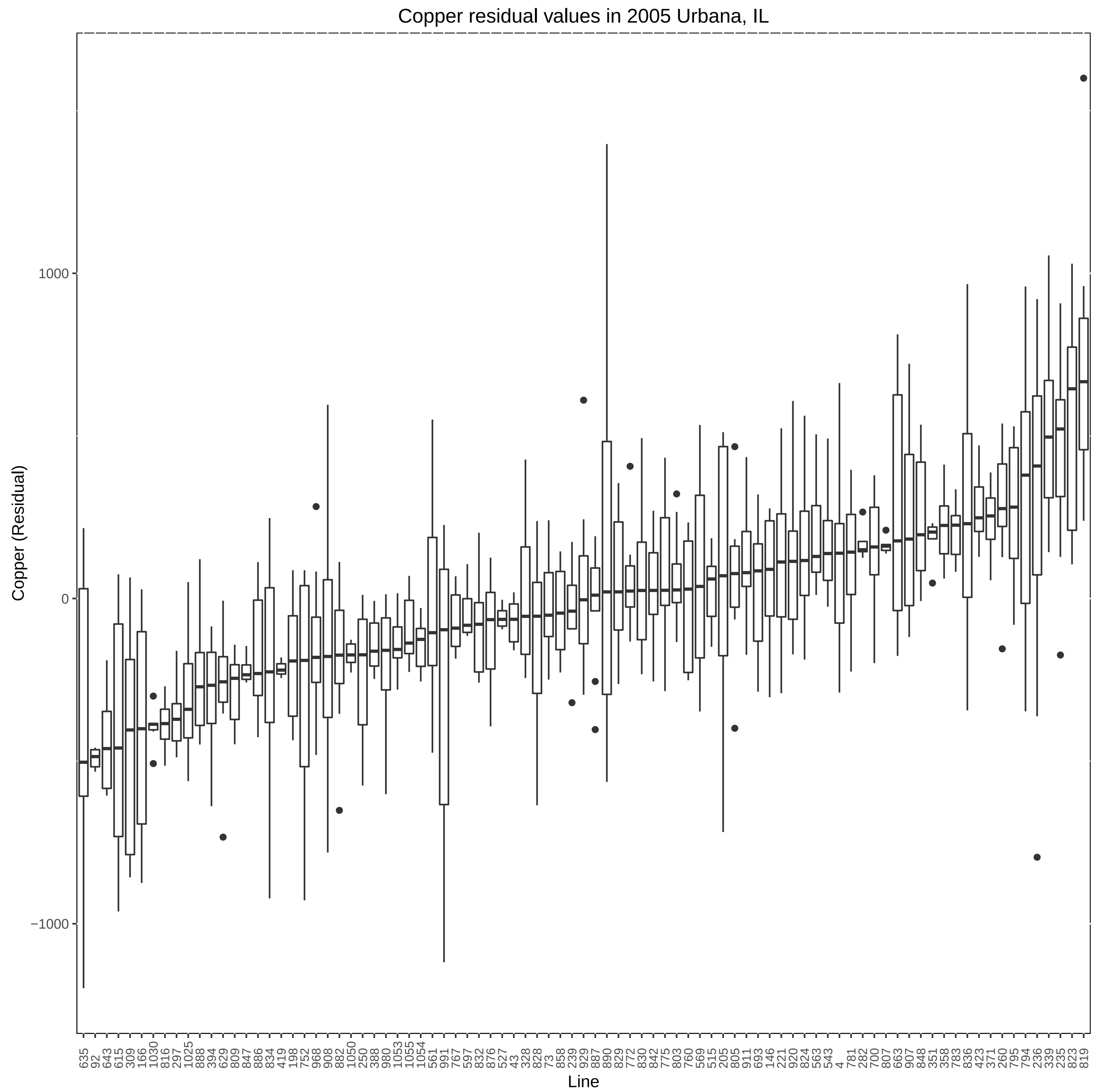
Copper residual values in 2005 Urbana, IL
Copper (Residual)
1000
0
−1000
Line

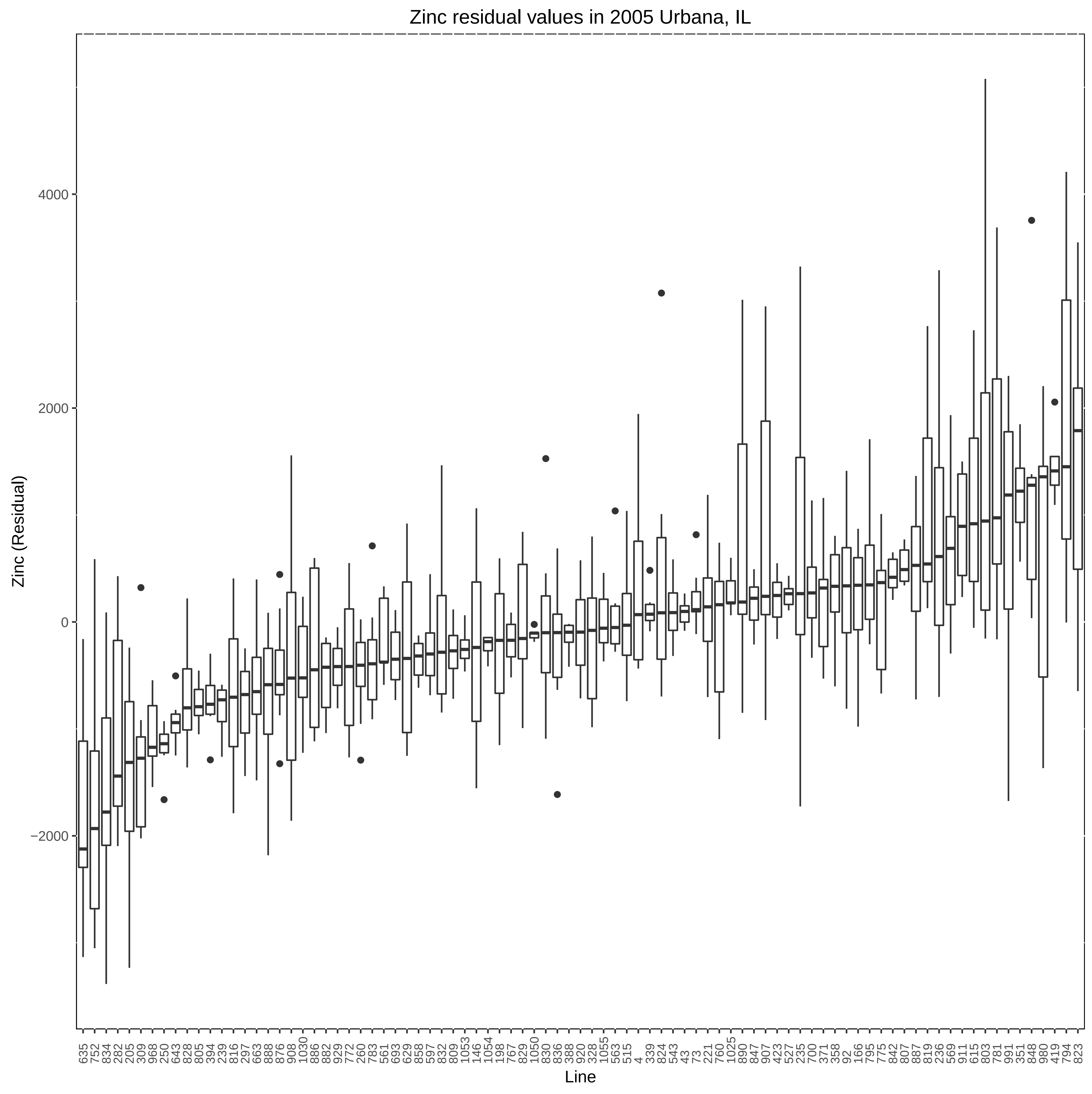
Zinc residual values in 2005 Urbana, IL
Zinc (Residual)
4000
2000
0
−2000
Line

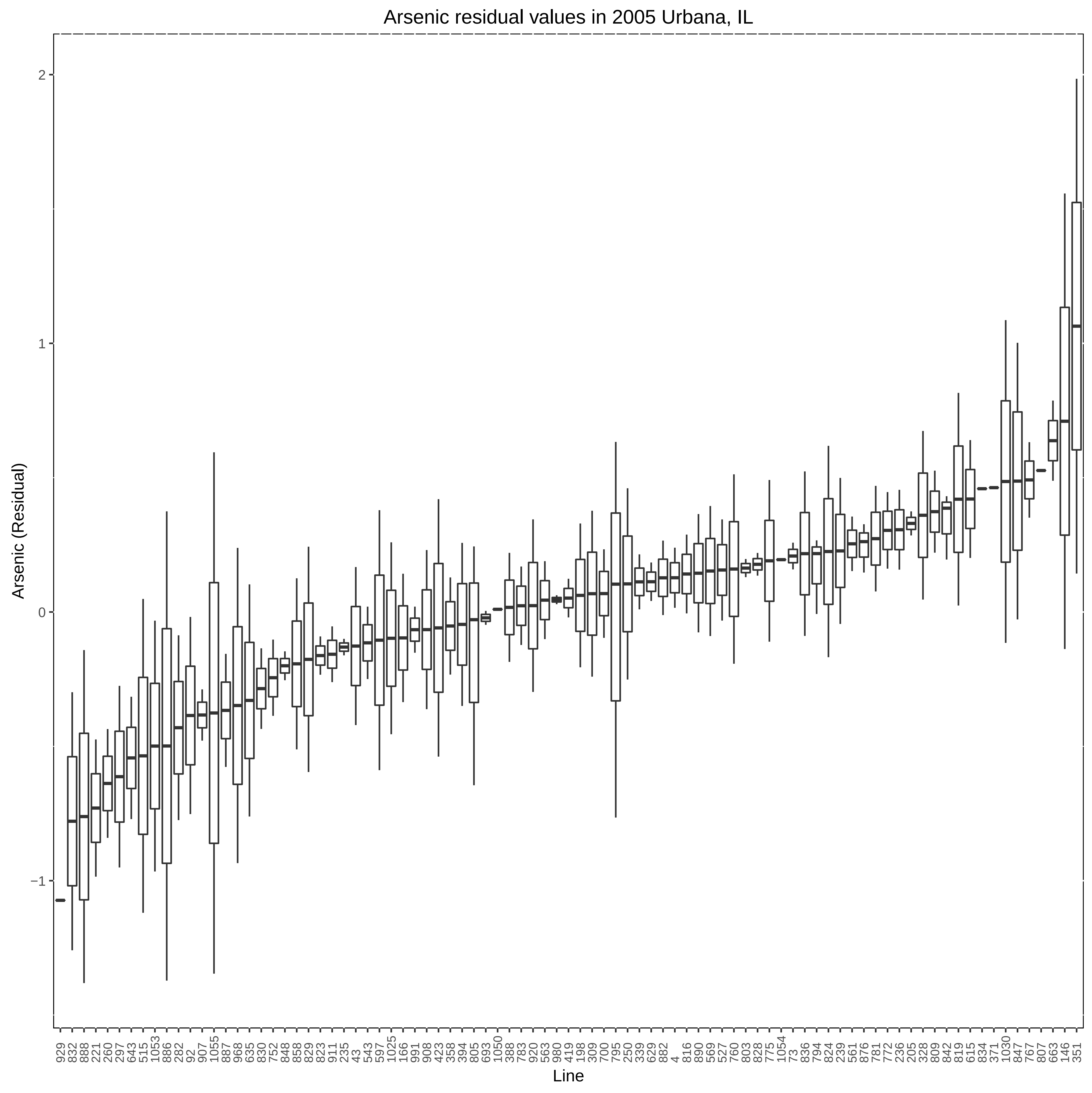
Arsenic residual values in 2005 Urbana, IL
Arsenic (Residual)
2
1
0
−1
Line

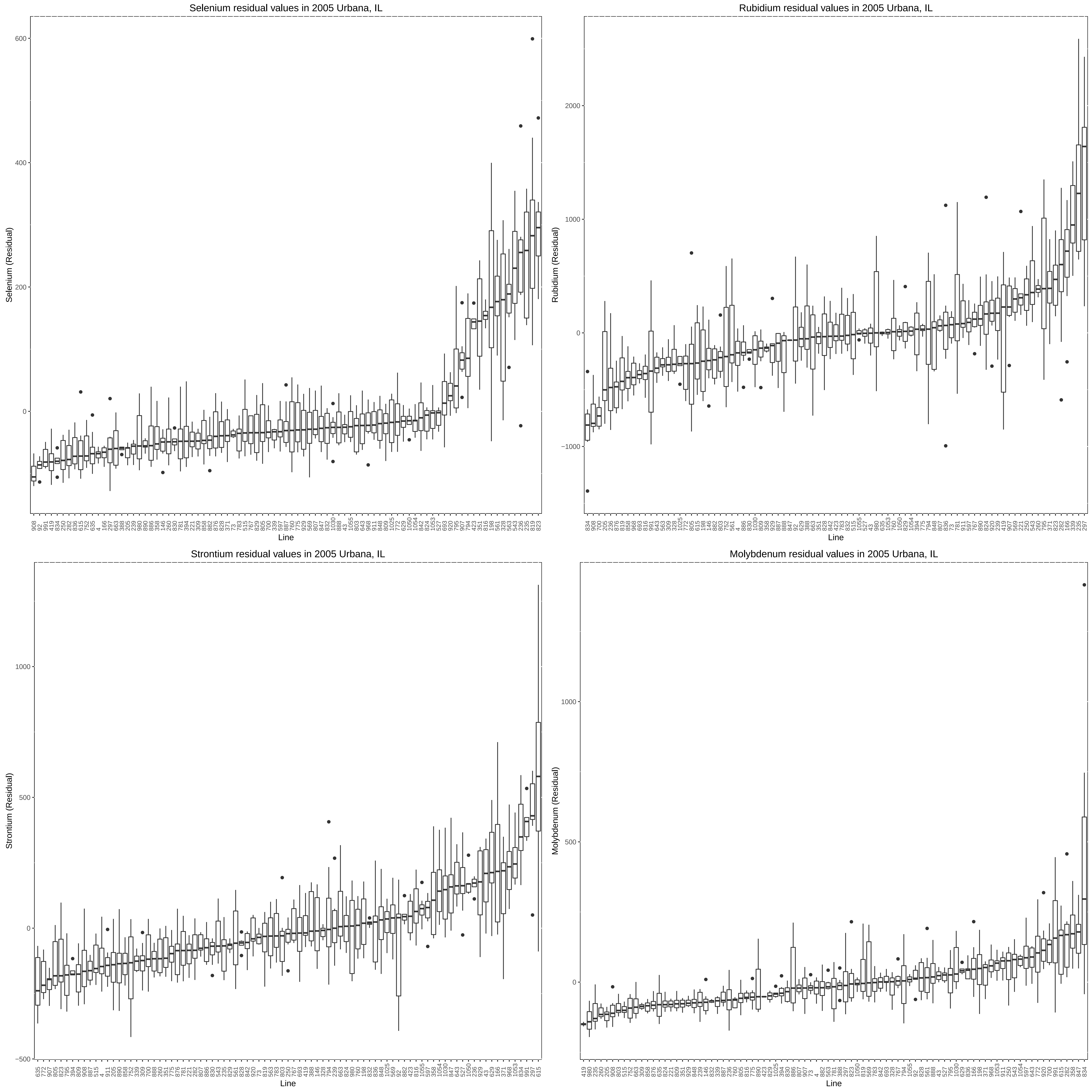

Selenium residual values in 2005 Urbana, IL
Selenium (Residual)
Line
Rubidium residual values in 2005 Urbana, IL
Rubidium (Residual)
Line
Strontium residual values in 2005 Urbana, IL
Strontium (Residual)
Line
Molybdenum residual values in 2005 Urbana, IL
Molybdenum (Residual)
Line

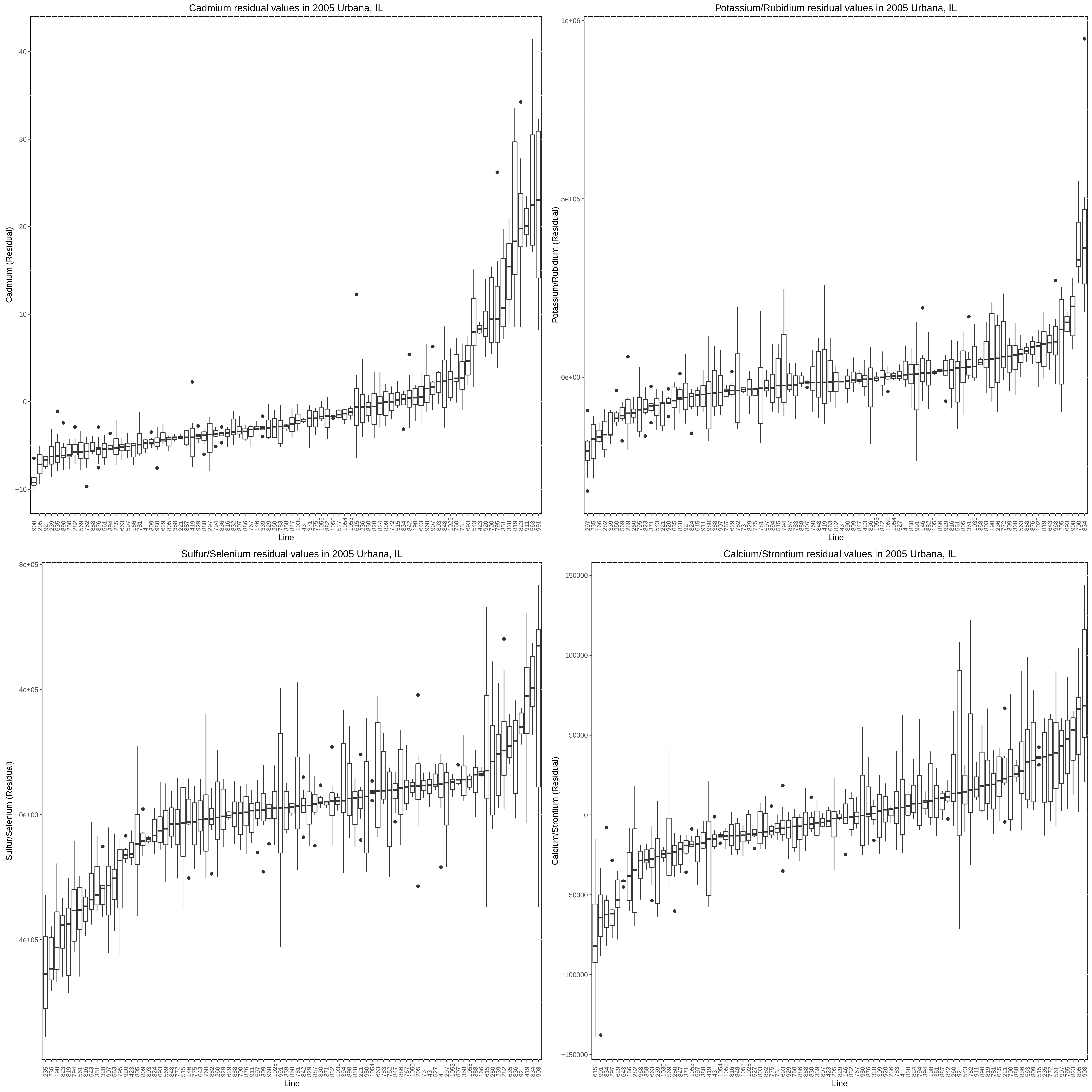
Cadmium residual values in 2005 Urbana, IL
Cadmium (Residual)
40
30
20
10
0
-10
Line
Potassium/Rubidium residual values in 2005 Urbana, IL
Potassium/Rubidium (Residual)
1e+06
5e+05
0e+00
Line
Sulfur/Selenium residual values in 2005 Urbana, IL
Sulfur/Selenium (Residual)
8e+05
4e+05
0e+00
-4e+05
Line
Calcium/Strontium residual values in 2005 Urbana, IL
Calcium/Strontium (Residual)
150000
100000
50000
0
-50000
-100000
-150000
Line

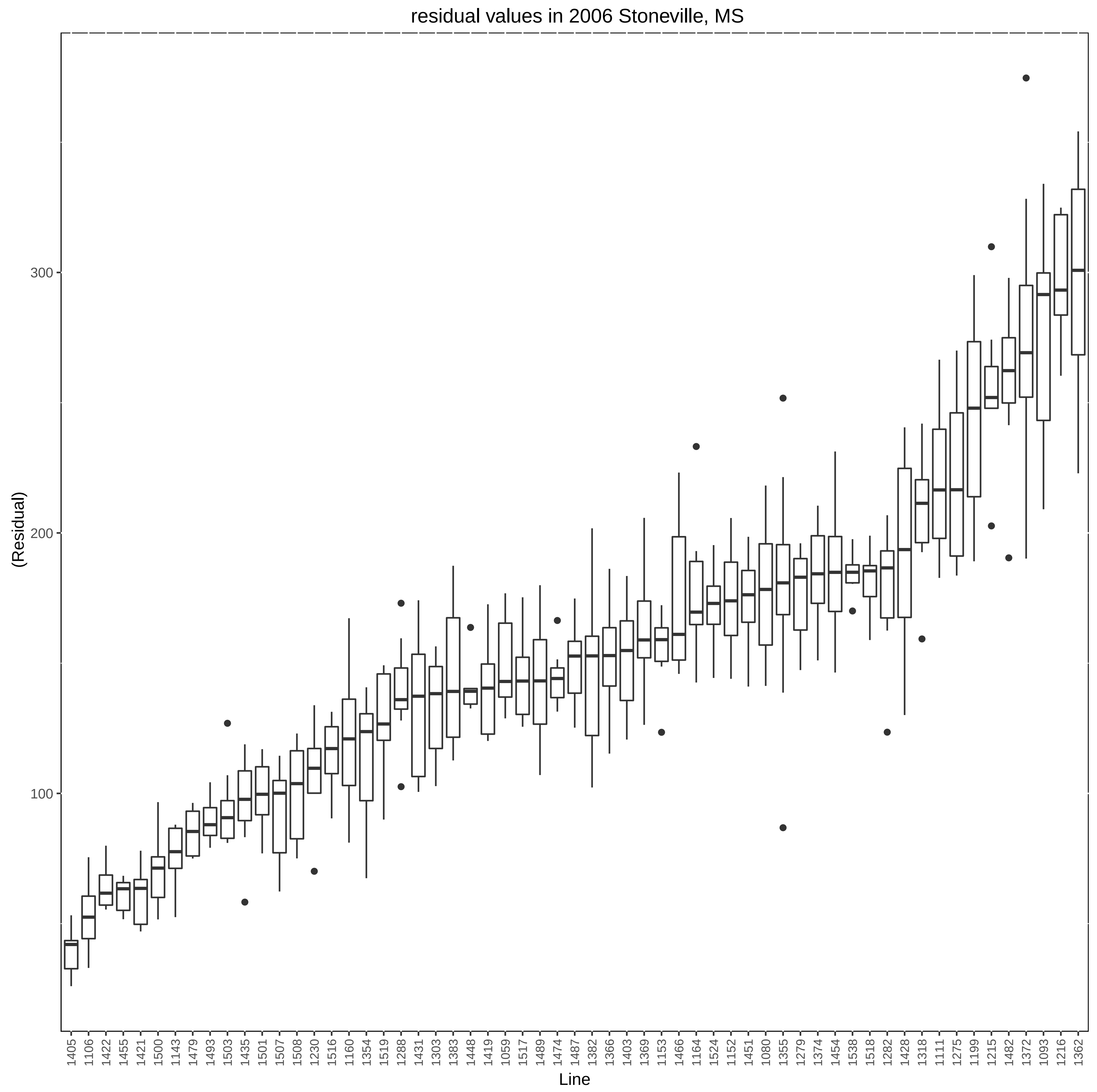
residual values in 2006 Stoneville, MS
(Residual)
300
200
100
Line

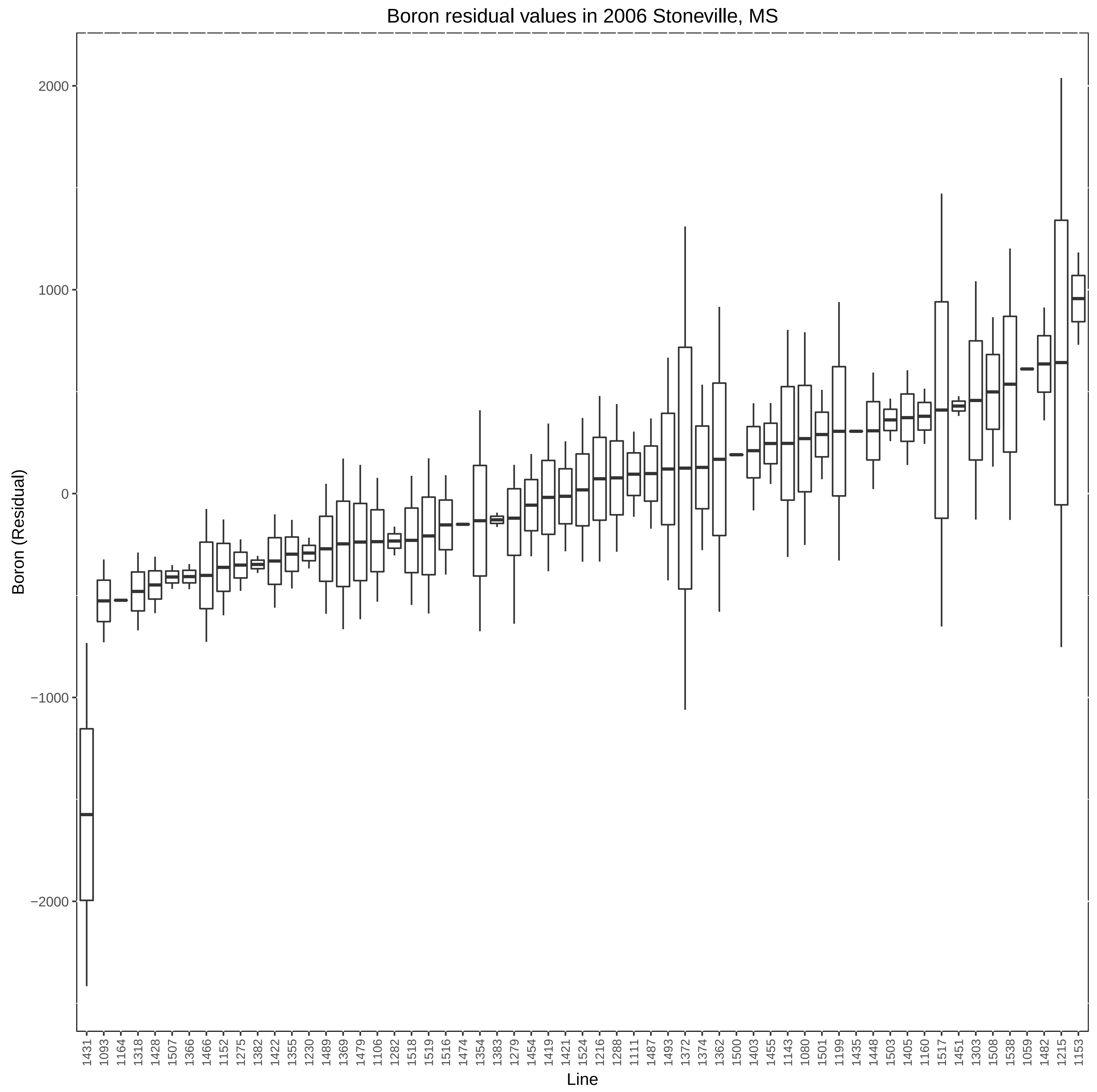
Boron residual values in 2006 Stoneville, MS
Boron (Residual)
2000
1000
0
−1000
−2000
Line

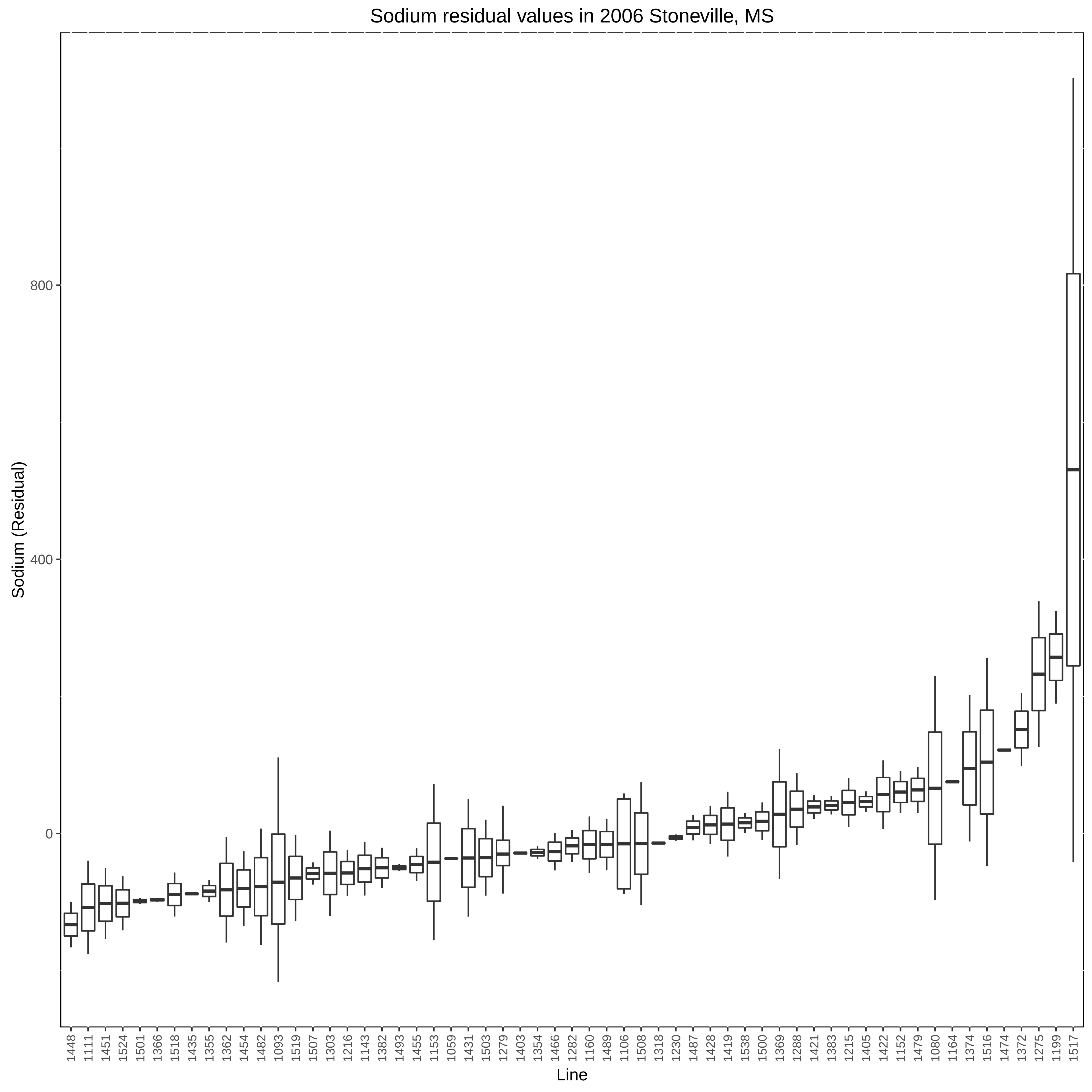
Sodium residual values in 2006 Stoneville, MS
Sodium (Residual)
800
400
0
Line

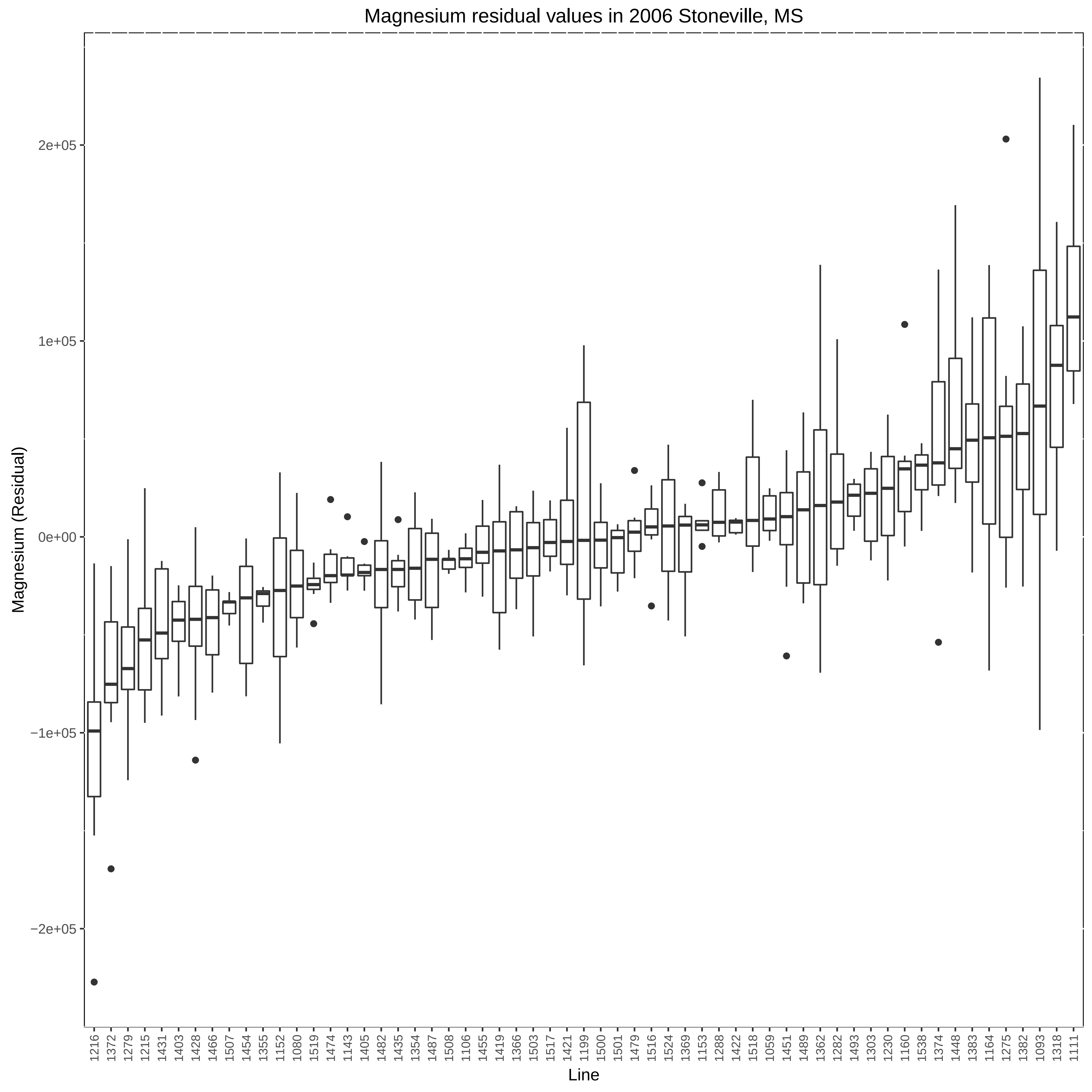
Magnesium residual values in 2006 Stoneville, MS
Magnesium (Residual)
2e+05
1e+05
0e+00
−1e+05
−2e+05
Line

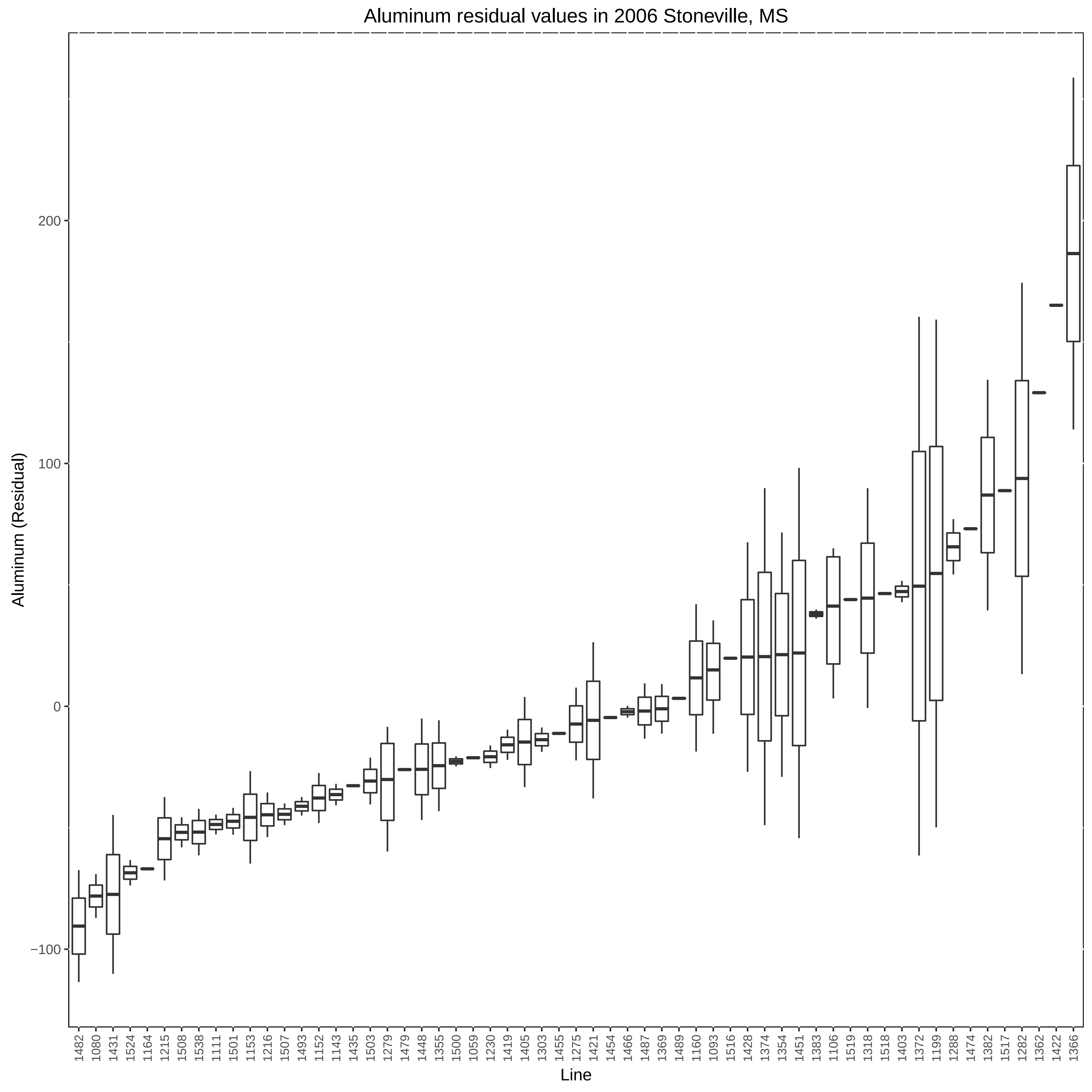
Aluminum residual values in 2006 Stoneville, MS
Aluminum (Residual)
200
100
0
-100
Line
1482 1080 1431 1524 1164 1215 1508 1538 1111 1501 1153 1216 1507 1493 1152 1143 1435 1503 1279 1479 1448 1355 1500 1059 1230 1419 1405 1303 1455 1275 1421 1454 1466 1487 1369 1489 1160 1093 1516 1428 1374 1354 1451 1383 1106 1519 1518 1403 1372 1199 1288 1474 1382 1517 1282 1362 1422 1366

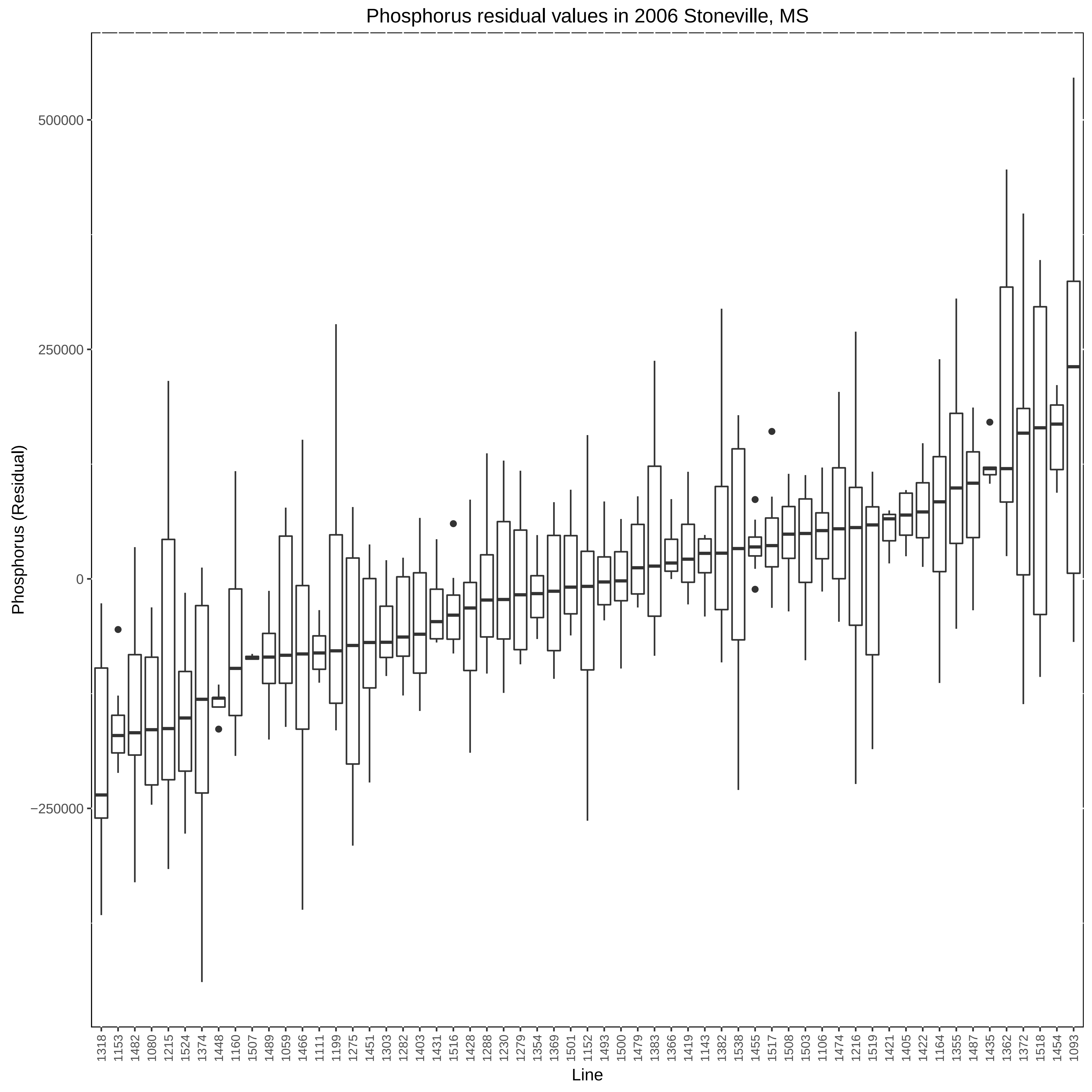
Phosphorus residual values in 2006 Stoneville, MS
Phosphorus (Residual)
500000
250000
0
-250000
Line
1318 1153 1482 1080 1215 1524 1374 1448 1160 1507 1489 1059 1466 1111 1199 1275 1451 1303 1282 1403 1431 1516 1428 1288 1230 1279 1354 1369 1501 1152 1493 1500 1479 1383 1366 1419 1143 1382 1538 1455 1517 1508 1106 1474 1216 1519 1421 1405 1422 1164 1355 1487 1435 1362 1372 1518 1454 1093

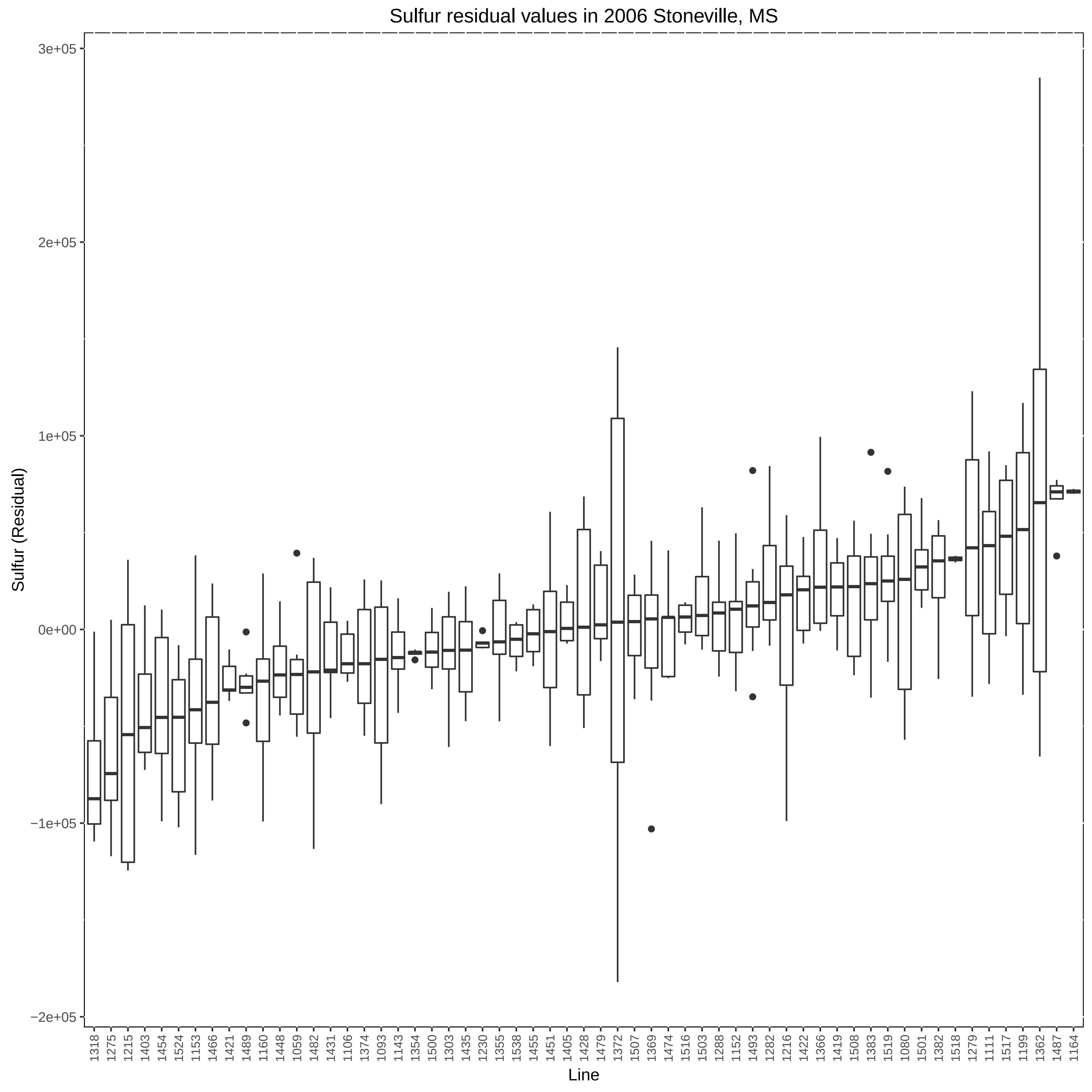
Sulfur residual values in 2006 Stoneville, MS
Sulfur (Residual)
3e+05
2e+05
1e+05
0e+00
-1e+05
-2e+05
Line
1318 1275 1215 1403 1454 1524 1153 1466 1421 1489 1160 1448 1059 1482 1431 1106 1374 1093 1143 1354 1500 1303 1435 1230 1355 1538 1455 1451 1405 1428 1479 1372 1507 1369 1474 1516 1503 1288 1152 1493 1282 1216 1422 1366 1419 1508 1383 1519 1080 1501 1382 1518 1279 1111 1517 1199 1362 1487 1164

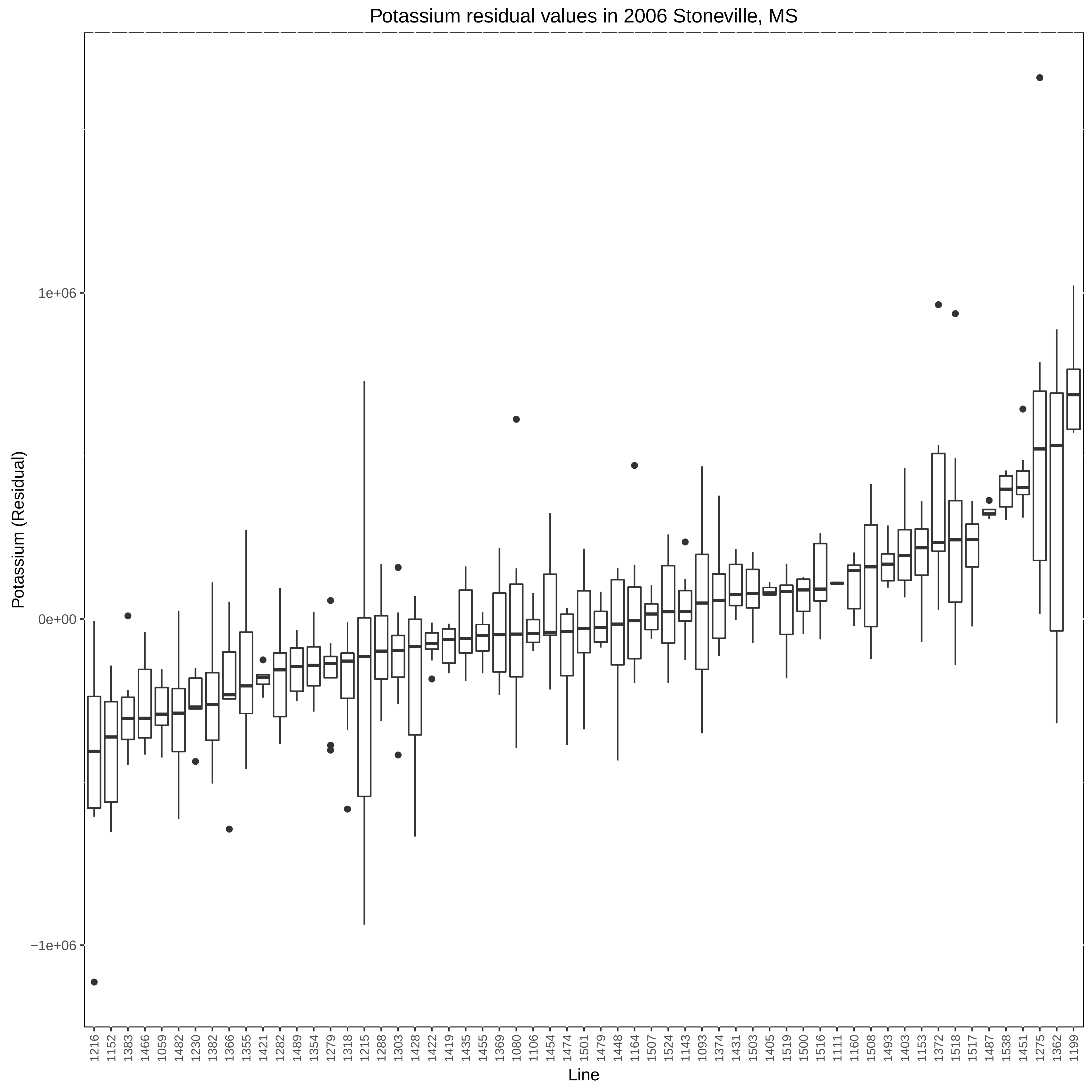
Potassium residual values in 2006 Stoneville, MS
Potassium (Residual)
1e+06
0e+00
-1e+06
Line
1216 1152 1383 1466 1059 1482 1230 1382 1366 1355 1421 1282 1489 1354 1279 1318 1215 1288 1303 1428 1422 1419 1435 1369 1080 1106 1454 1474 1501 1479 1448 1164 1507 1524 1143 1093 1374 1431 1503 1405 1519 1500 1516 1111 1160 1508 1493 1403 1153 1372 1518 1517 1487 1538 1451 1275 1362 1199

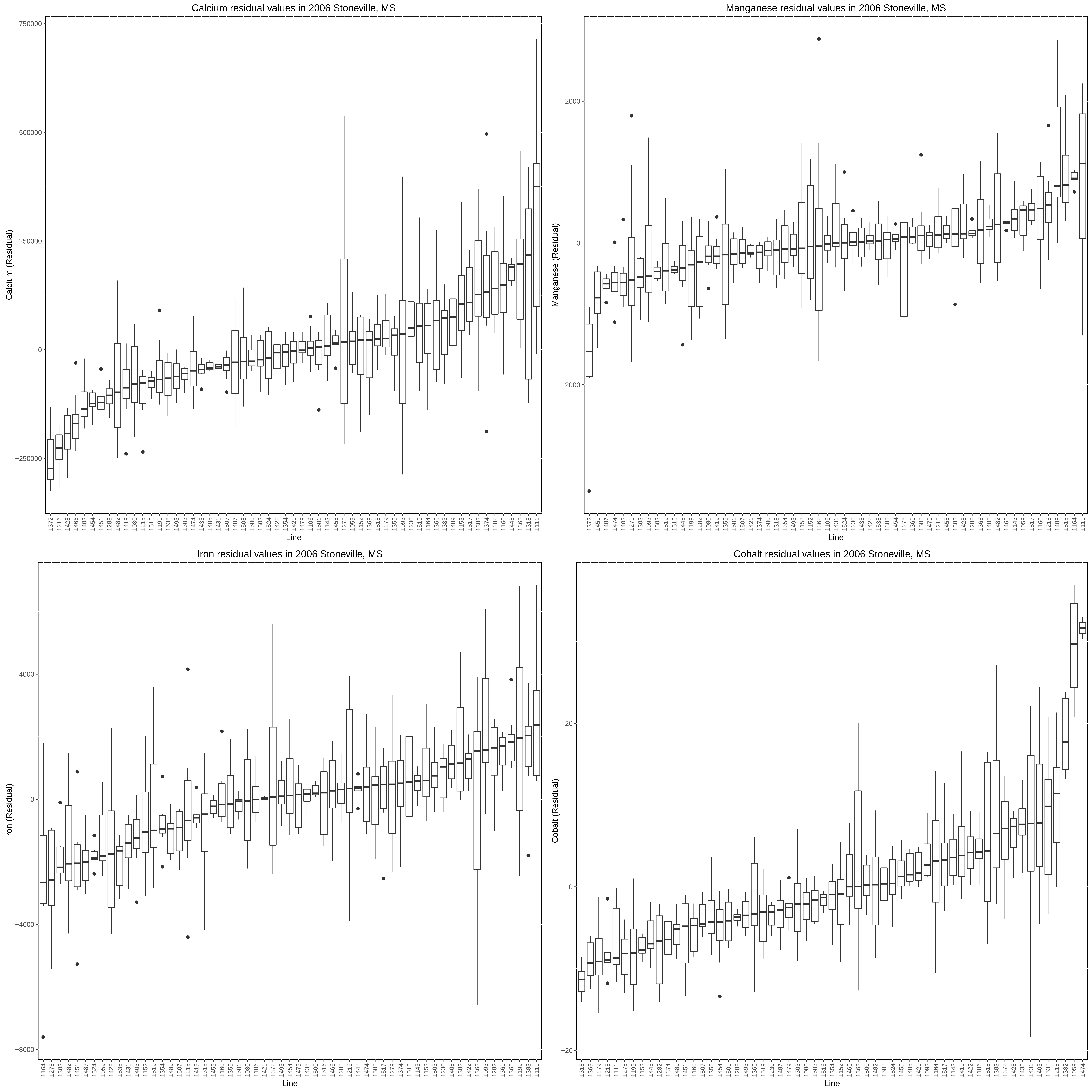

Calcium residual values in 2006 Stoneville, MS
Calcium (Residual)
750000
500000
250000
0
-250000
Line
1372 1216 1428 1466 1403 1454 1451 1288 1482 1419 1080 1215 1516 1199 1538 1493 1303 1474 1435 1405 1431 1507 1487 1508 1500 1503 1524 1422 1354 1421 1479 1106 1501 1143 1455 1275 1059 1152 1369 1518 1279 1355 1093 1230 1519 1164 1366 1383 1489 1153 1517 1382 1374 1282 1160 1448 1362 1318 1111
Manganese residual values in 2006 Stoneville, MS
Manganese (Residual)
2000
0
-2000
Line
1372 1451 1487 1474 1403 1279 1303 1093 1503 1519 1516 1448 1199 1282 1080 1419 1355 1501 1507 1421 1374 1500 1318 1354 1493 1153 1152 1362 1106 1431 1524 1230 1435 1422 1538 1382 1454 1275 1369 1508 1479 1215 1455 1428 1383 1288 1366 1405 1482 1466 1143 1059 1517 1160 1216 1489 1518 1164 1111
Iron residual values in 2006 Stoneville, MS
Iron (Residual)
4000
0
-4000
-8000
Line
1164 1275 1303 1482 1451 1487 1524 1059 1428 1538 1431 1403 1152 1519 1354 1489 1507 1215 1419 1318 1455 1160 1355 1501 1080 1106 1421 1372 1493 1454 1479 1435 1500 1516 1466 1288 1216 1448 1474 1508 1517 1279 1374 1518 1143 1153 1503 1230 1405 1382 1422 1362 1093 1282 1369 1366 1199 1383 1111
Cobalt residual values in 2006 Stoneville, MS
Cobalt (Residual)
20
0
-20
Line
1318 1369 1279 1215 1111 1275 1199 1153 1448 1282 1374 1489 1451 1160 1507 1355 1454 1501 1288 1493 1366 1519 1230 1487 1479 1303 1080 1503 1516 1354 1152 1466 1362 1500 1482 1508 1524 1455 1405 1421 1093 1164 1517 1143 1419 1422 1106 1518 1383 1372 1428 1435 1431 1538 1216 1382 1059 1474

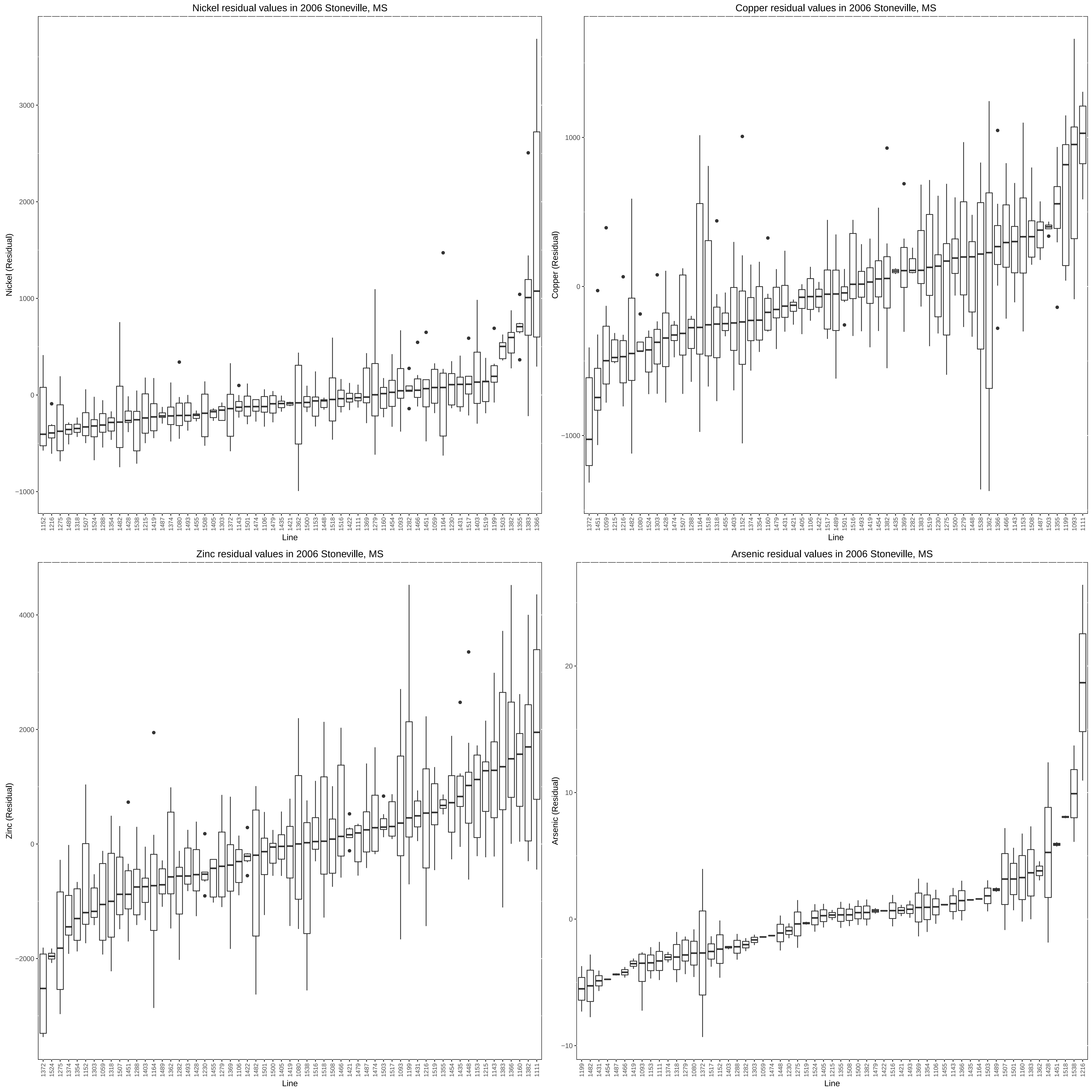

Nickel residual values in 2006 Stoneville, MS
Nickel (Residual)
3000
2000
1000
0
−1000
Line
Copper residual values in 2006 Stoneville, MS
Copper (Residual)
1000
0
−1000
Line
Zinc residual values in 2006 Stoneville, MS
Zinc (Residual)
4000
2000
0
-2000
Line
Arsenic residual values in 2006 Stoneville, MS
Arsenic (Residual)
20
10
0
−10
Line

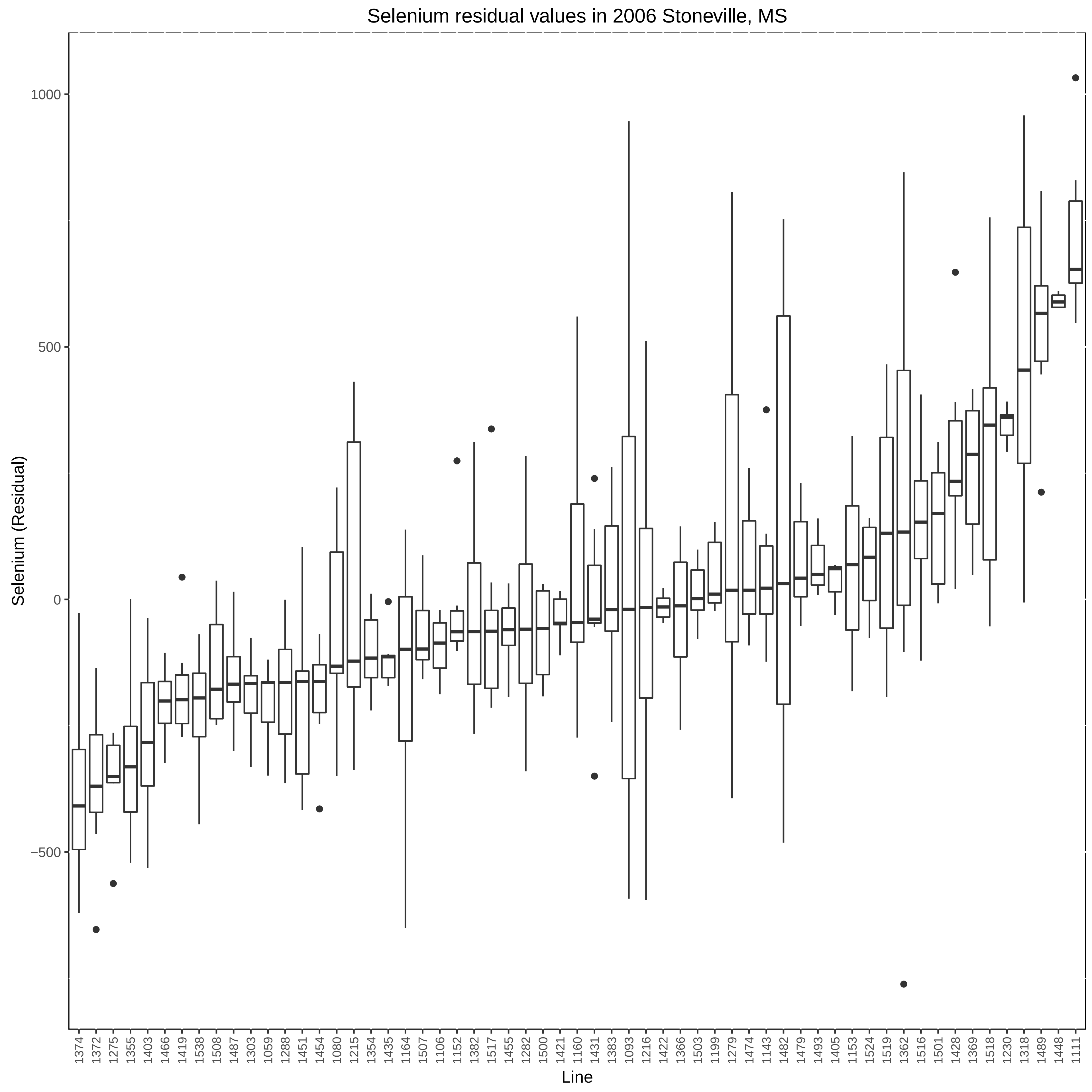
Selenium residual values in 2006 Stoneville, MS
Selenium (Residual)
Line

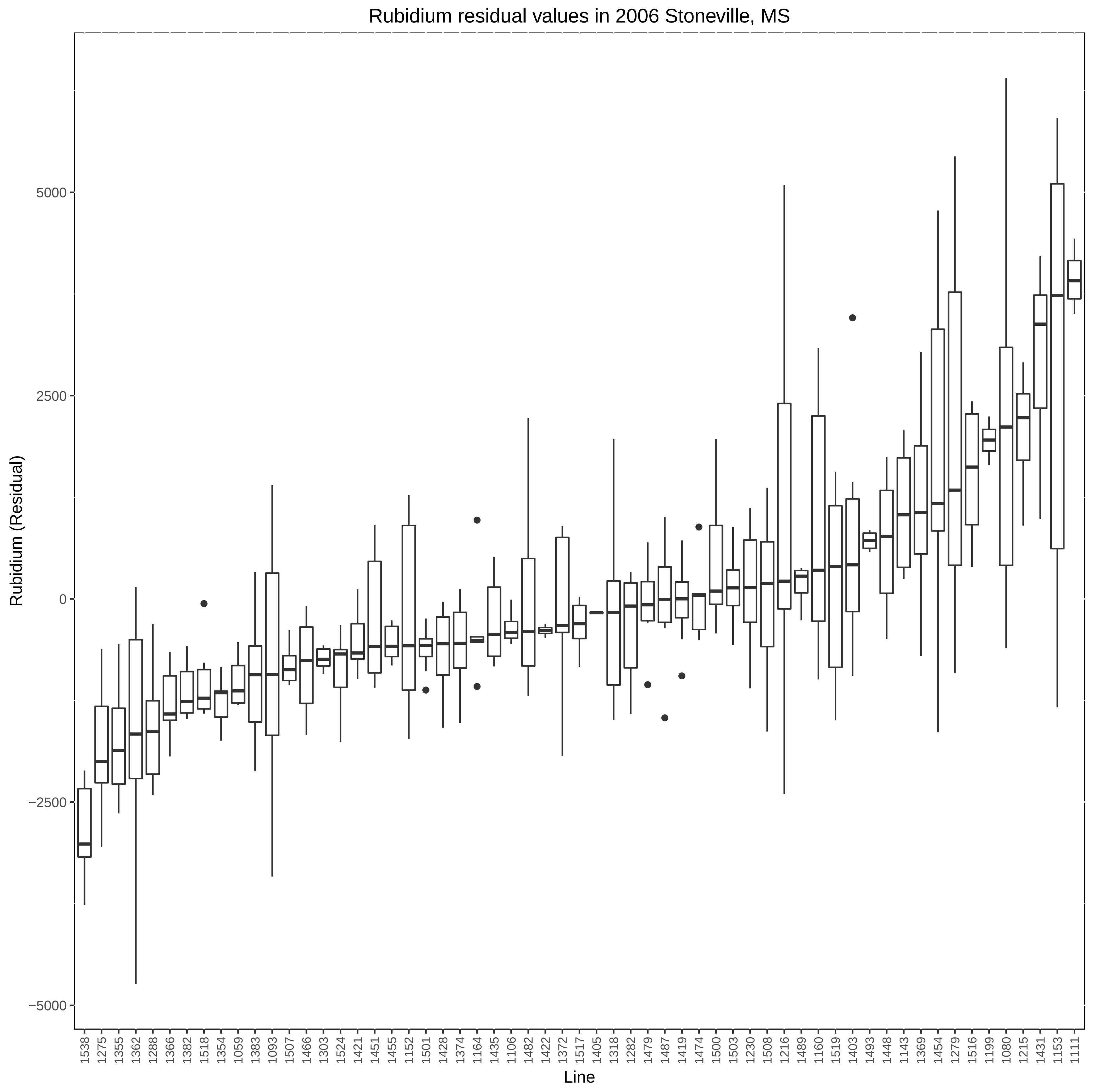
Rubidium residual values in 2006 Stoneville, MS
Rubidium (Residual)
Line

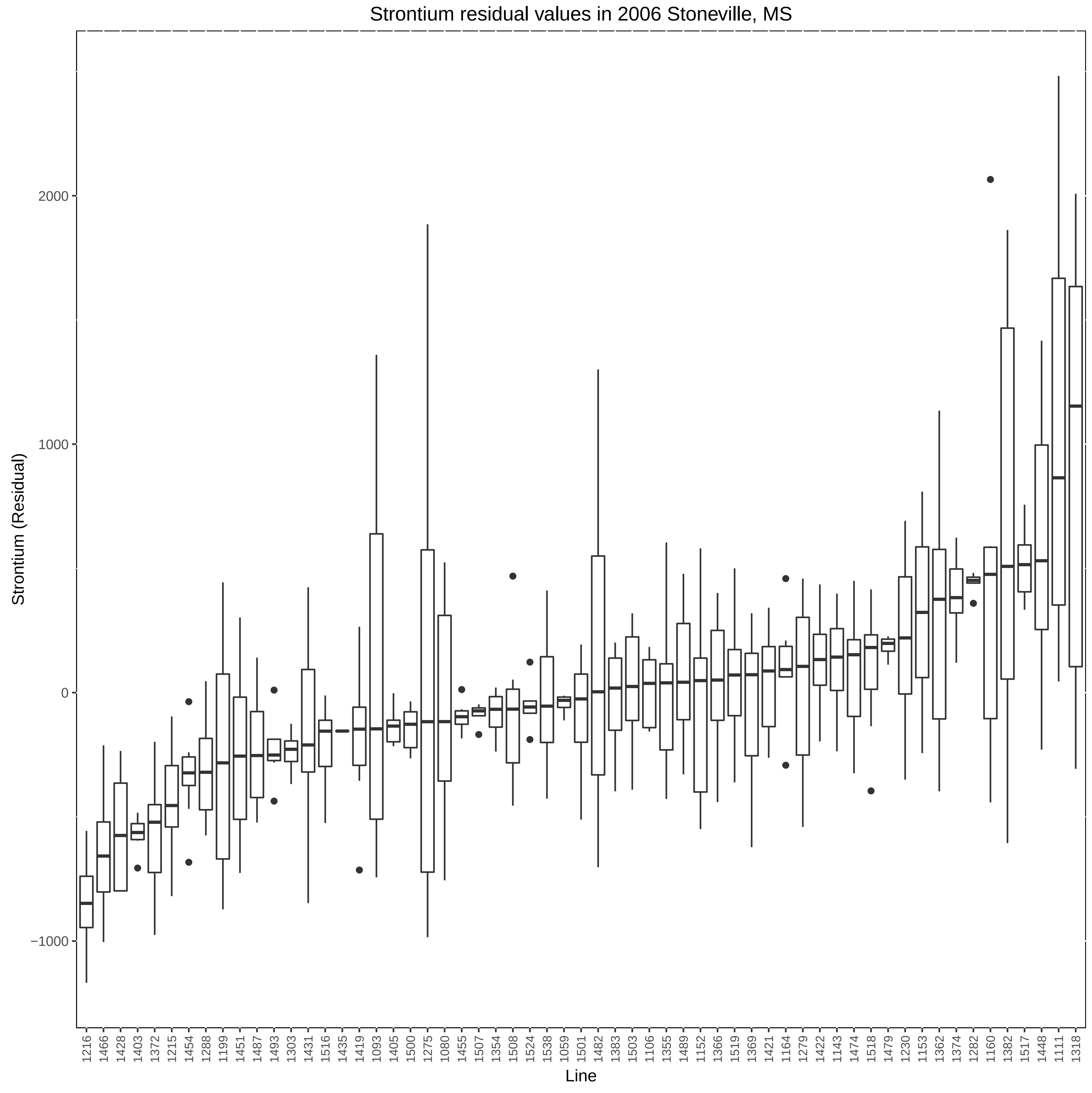
Strontium residual values in 2006 Stoneville, MS
Strontium (Residual)
Line

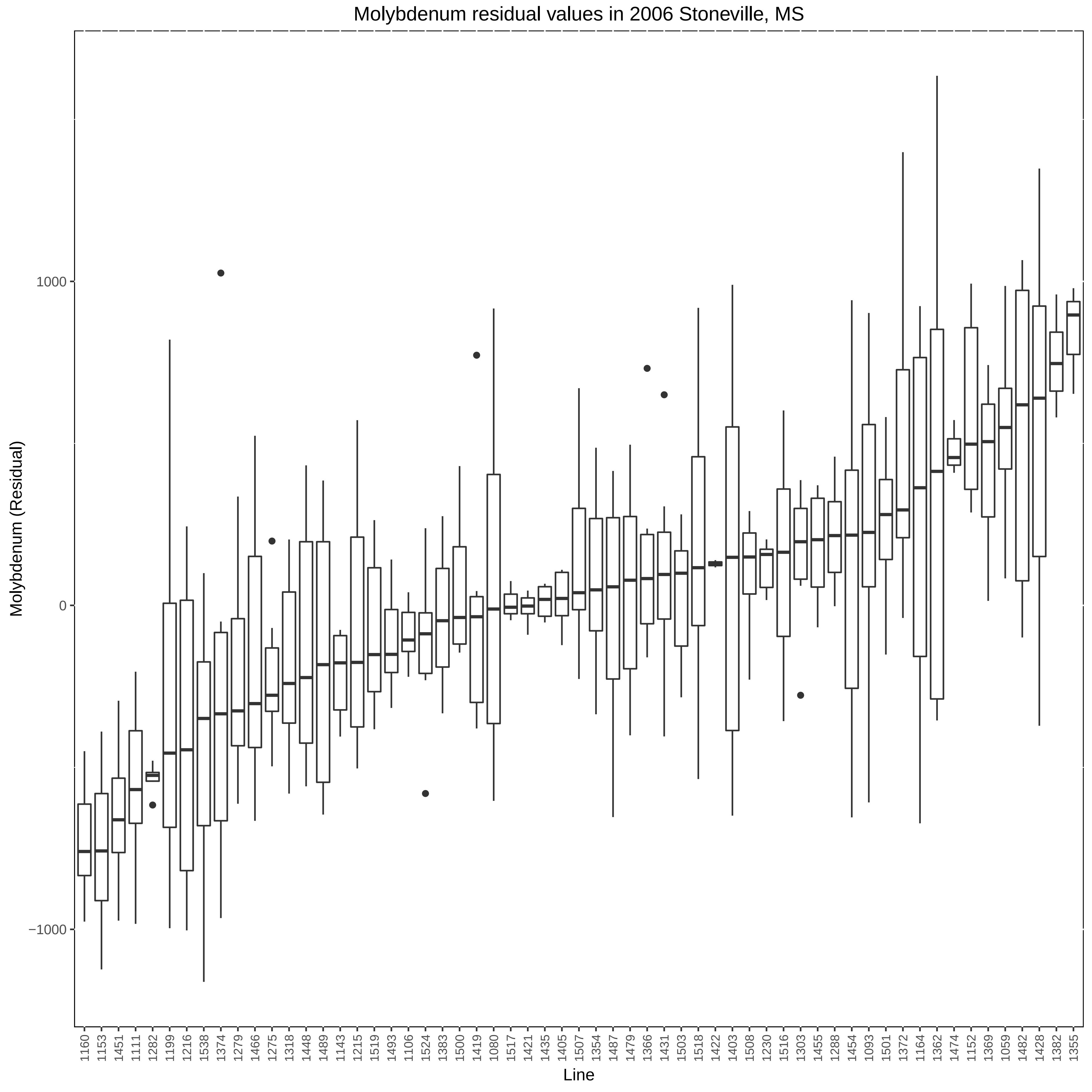
Molybdenum residual values in 2006 Stoneville, MS
Molybdenum (Residual)
Line

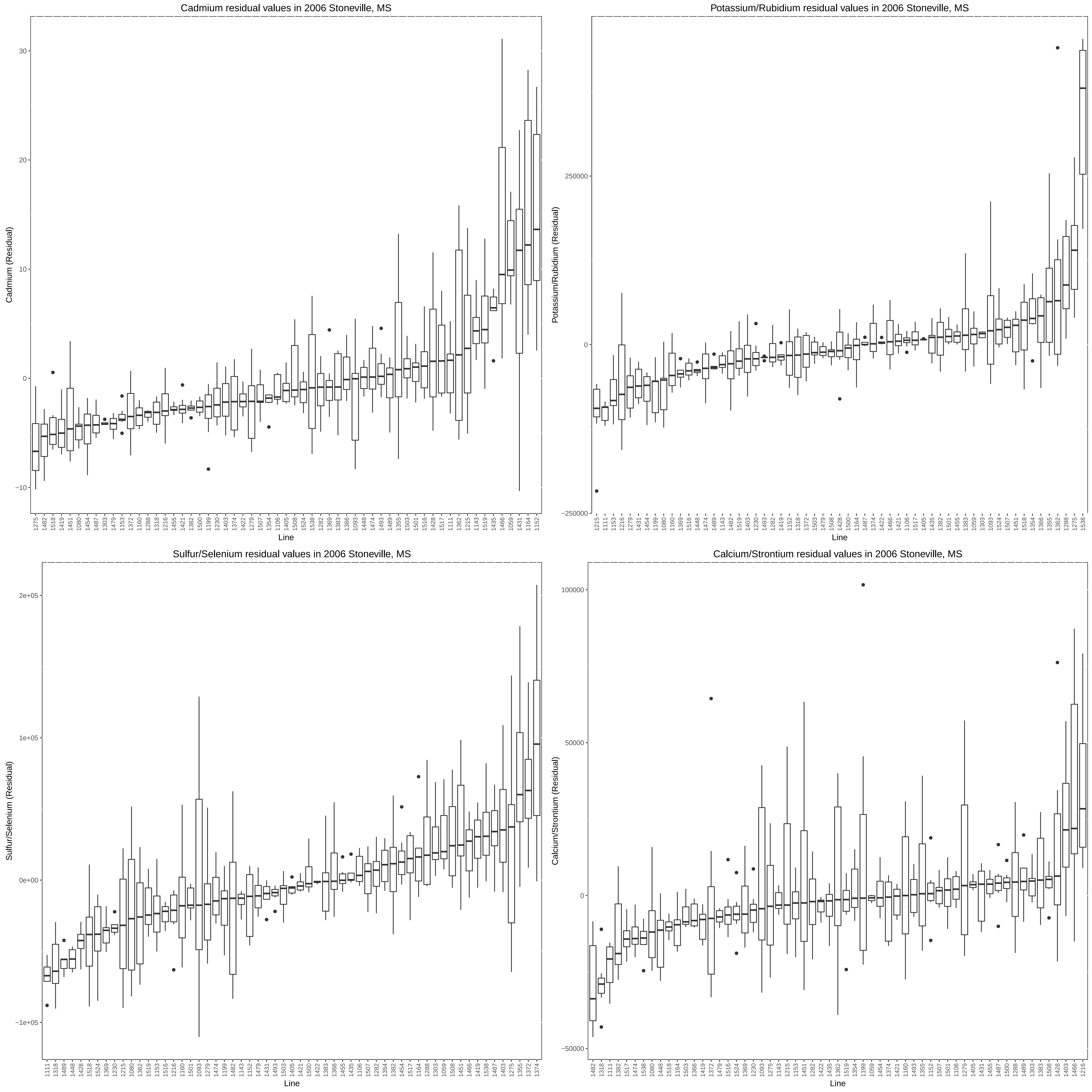
Cadmium residual values in 2006 Stoneville, MS
Cadmium (Residual)
30
20
10
0
-10
1275 1482 1518 1419 1451 1080 1454 1487 1303 1479 1153 1372 1160 1288 1318 1216 1455 1421 1382 1500 1199 1230 1403 1374 1422 1279 1507 1354 1106 1405 1508 1524 1538 1282 1369 1383 1366 1093 1448 1474 1493 1489 1355 1503 1501 1516 1428 1517 1111 1362 1215 1143 1519 1435 1466 1059 1431 1164 1152
Line
Potassium/Rubidium residual values in 2006 Stoneville, MS
Potassium/Rubidium (Residual)
250000
0
-250000
1215 1111 1153 1216 1279 1431 1454 1199 1080 1160 1369 1516 1448 1474 1489 1143 1482 1519 1403 1230 1493 1282 1419 1152 1372 1503 1479 1508 1428 1500 1164 1487 1374 1422 1466 1421 1106 1405 1517 1435 1382 1501 1455 1383 1059 1303 1093 1524 1507 1451 1518 1354 1366 1355 1362 1288 1275 1538
Line
Sulfur/Selenium residual values in 2006 Stoneville, MS
Sulfur/Selenium (Residual)
2e+05
1e+05
0e+00
-1e+05
1111 1318 1489 1448 1428 1518 1524 1369 1230 1215 1080 1362 1519 1153 1516 1216 1160 1501 1093 1279 1474 1199 1482 1143 1152 1479 1431 1493 1503 1405 1421 1500 1422 1383 1366 1455 1435 1106 1507 1282 1354 1382 1454 1517 1164 1288 1303 1059 1508 1451 1466 1419 1538 1487 1403 1275 1355 1372 1374
Line
Calcium/Strontium residual values in 2006 Stoneville, MS
Calcium/Strontium (Residual)
100000
50000
0
-50000
1482 1318 1111 1382 1517 1474 1538 1080 1448 1518 1164 1503 1366 1419 1372 1479 1516 1524 1369 1230 1093 1275 1143 1215 1153 1451 1282 1422 1435 1362 1519 1354 1199 1059 1454 1374 1421 1160 1493 1355 1152 1507 1501 1106 1279 1405 1431 1455 1487 1500 1288 1489 1303 1383 1508 1428 1403 1466 1216
Line

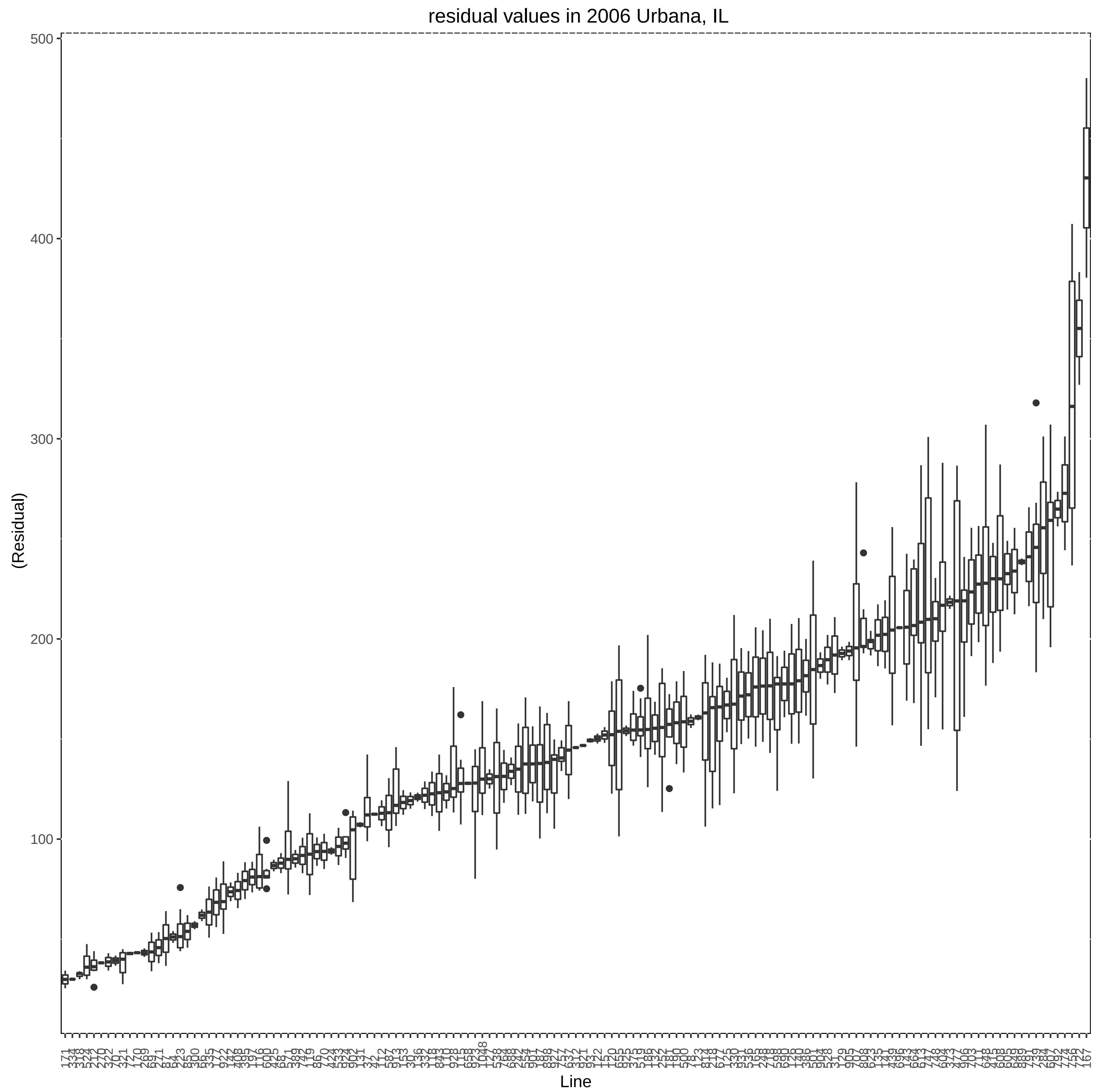
residual values in 2006 Urbana, IL
(Residual)
500
400
300
200
100
Line

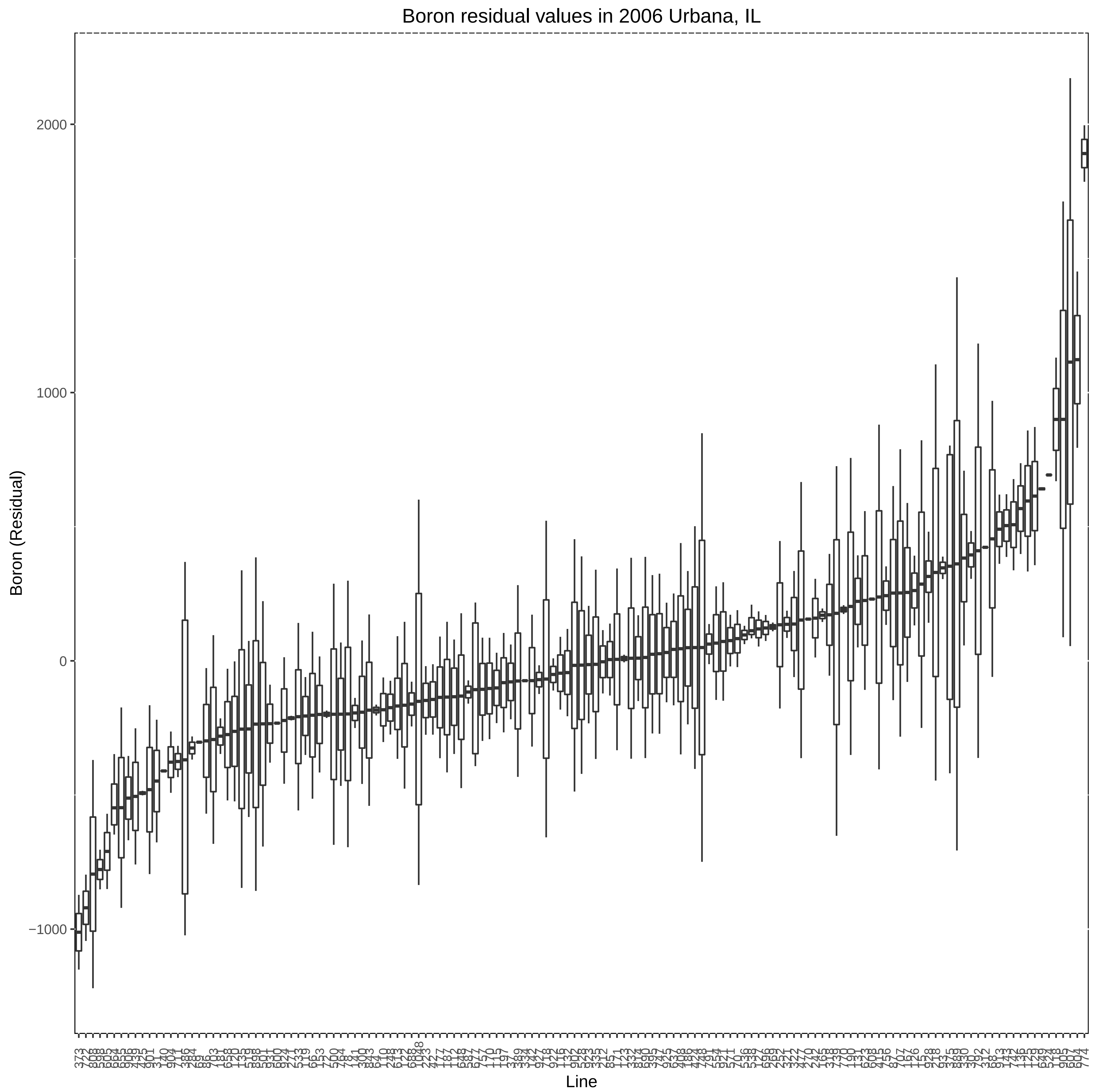
Boron residual values in 2006 Urbana, IL
Boron (Residual)
2000
1000
0
−1000
Line

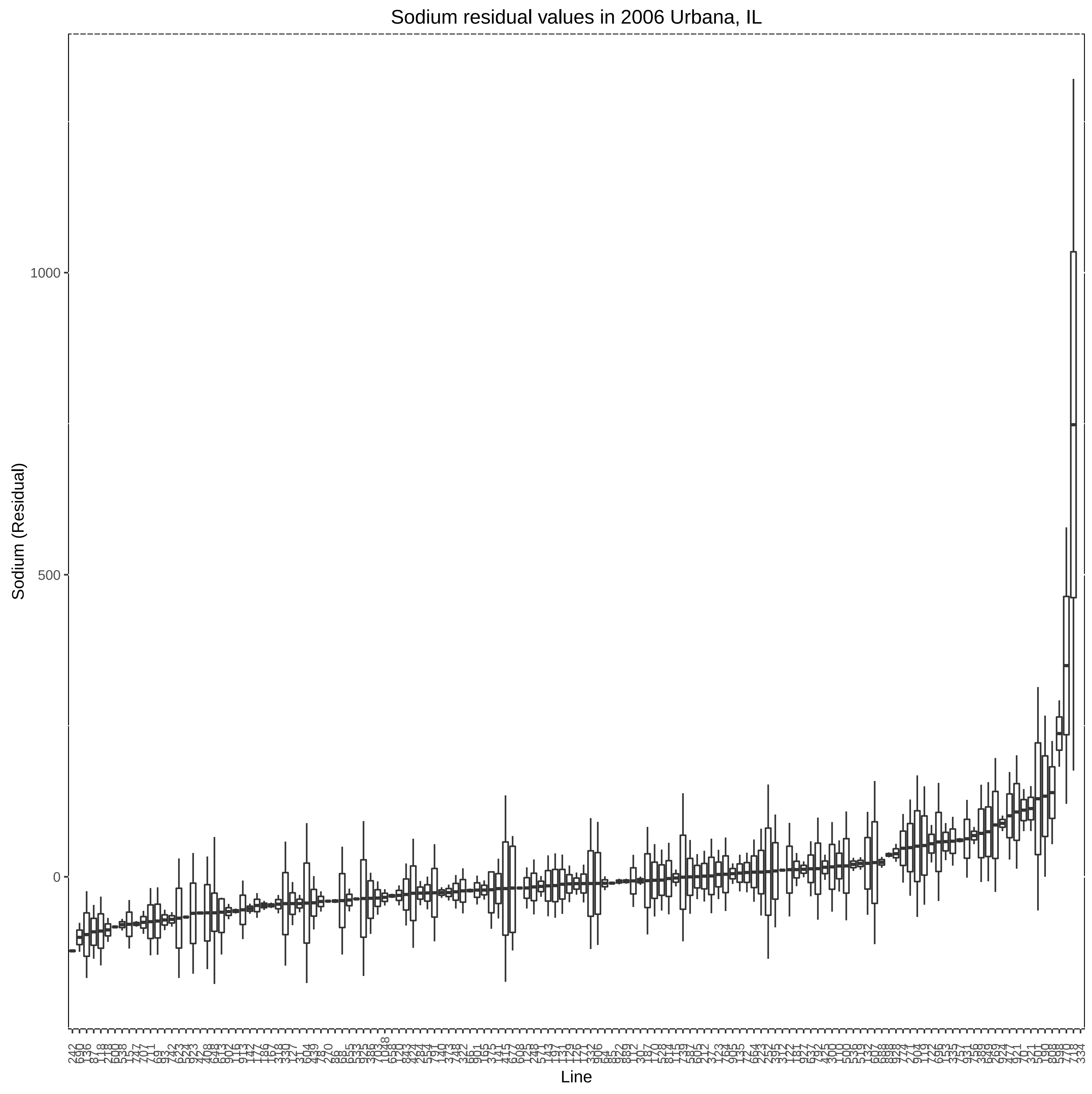
Sodium residual values in 2006 Urbana, IL
Sodium (Residual)
1000
500
0
Line

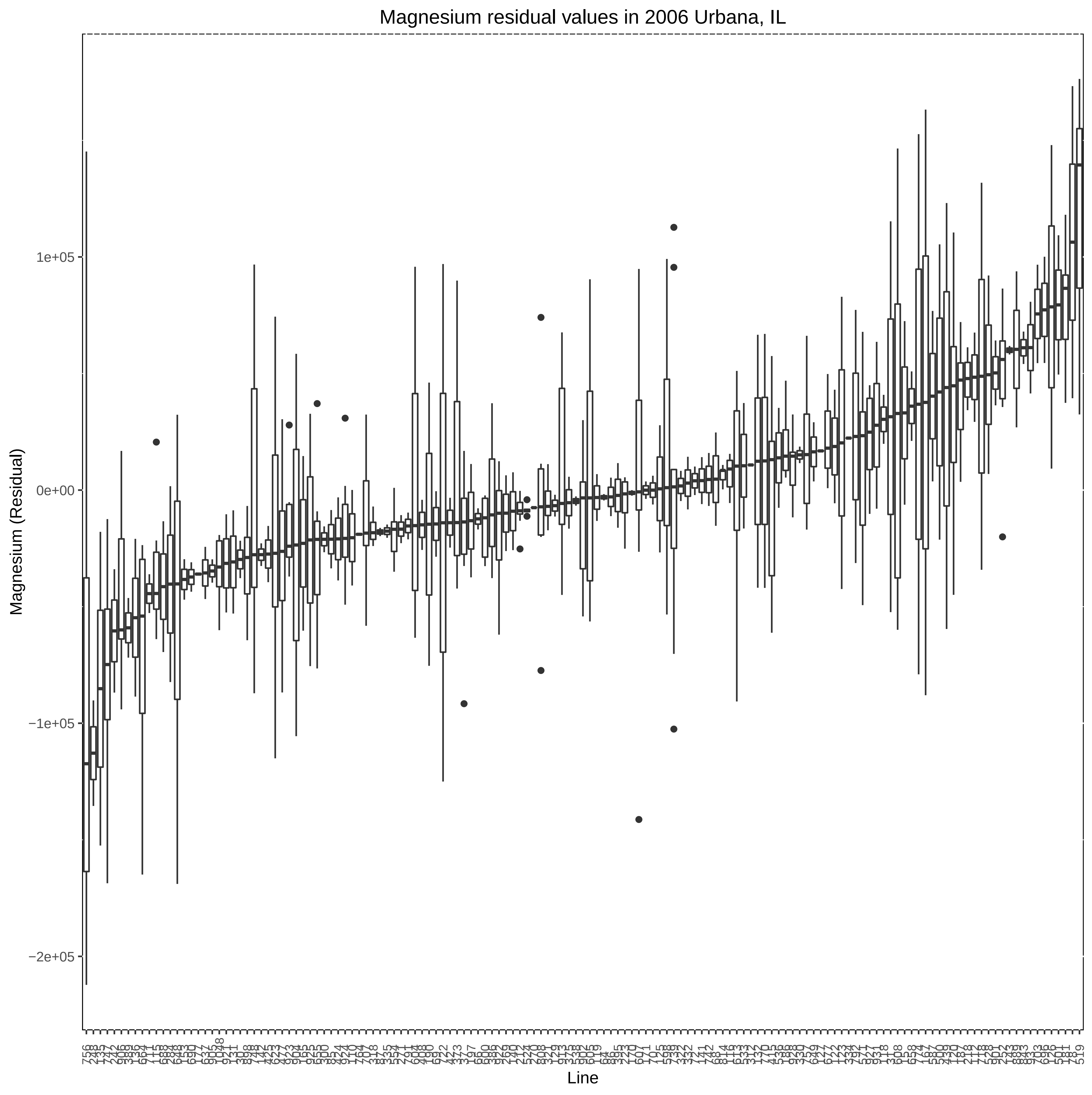
Magnesium residual values in 2006 Urbana, IL
Magnesium (Residual)
1e+05
0e+00
−1e+05
−2e+05
Line

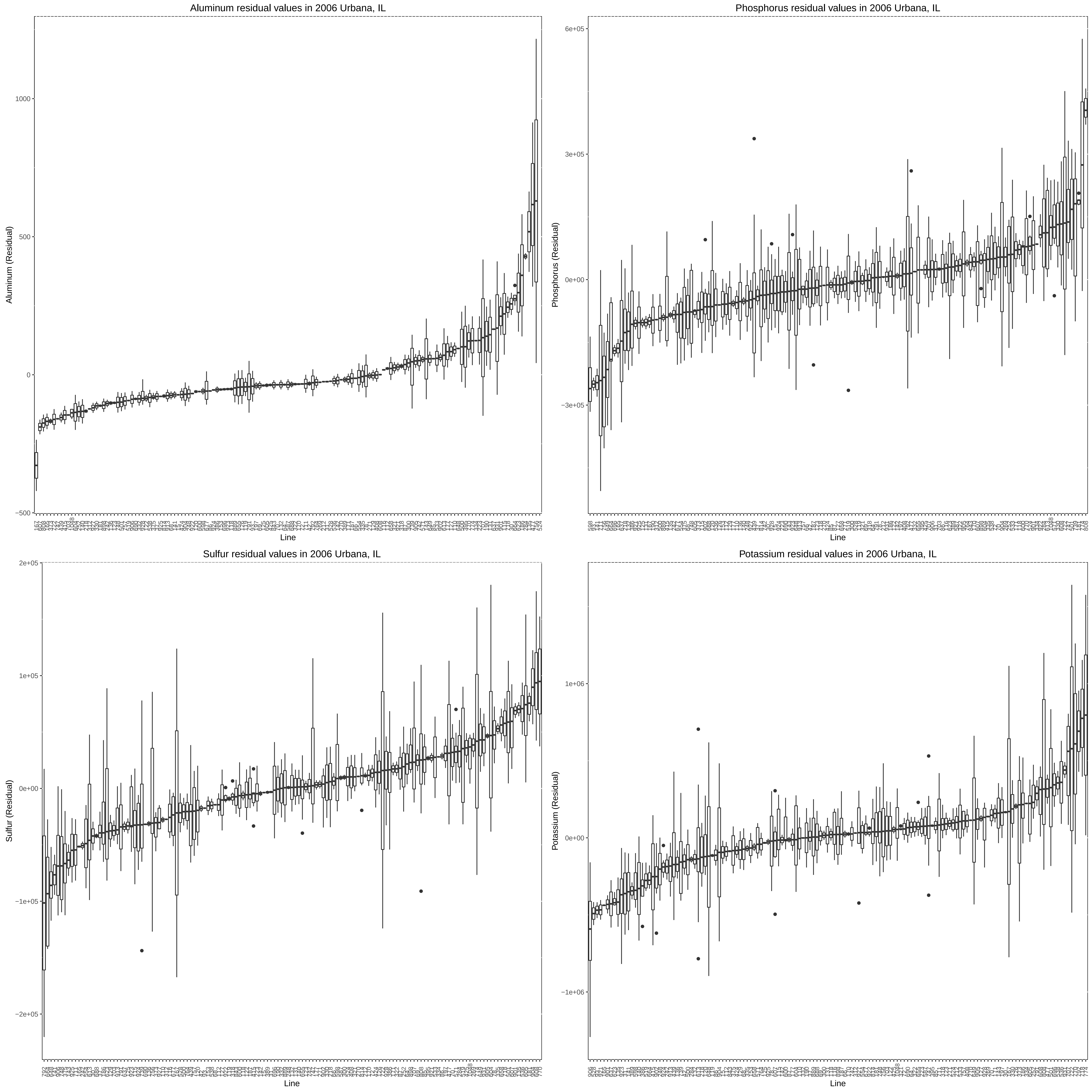
Aluminum residual values in 2006 Urbana, IL
Aluminum (Residual)
1000
500
0
-500
Line
Phosphorus residual values in 2006 Urbana, IL
Phosphorus (Residual)
6e+05
3e+05
0e+00
-3e+05
Line
Sulfur residual values in 2006 Urbana, IL
Sulfur (Residual)
2e+05
1e+05
0e+00
-1e+05
-2e+05
Line
Potassium residual values in 2006 Urbana, IL
Potassium (Residual)
1e+06
0e+00
-1e+06
Line

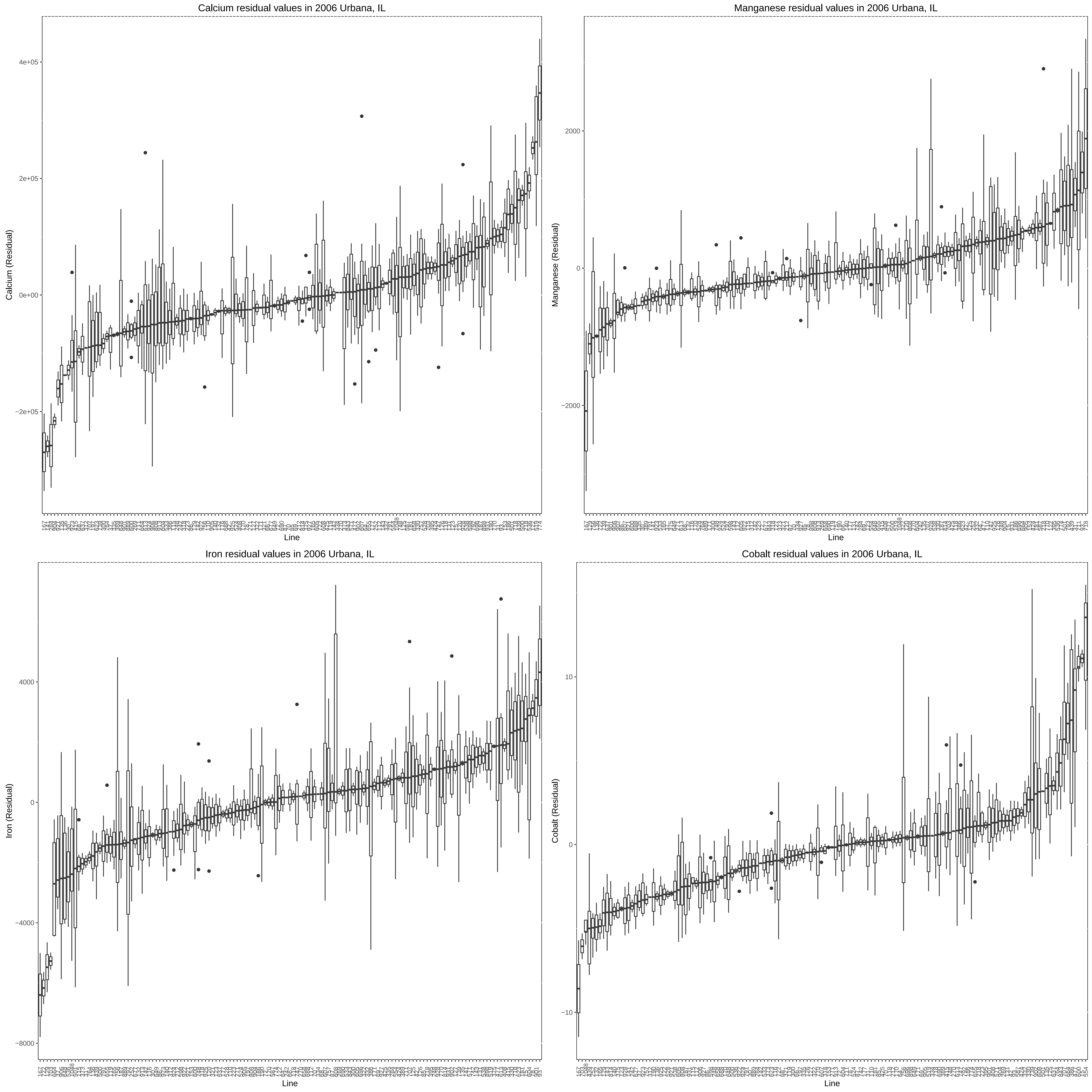
Calcium residual values in 2006 Urbana, IL
Calcium (Residual)
4e+05
2e+05
0e+00
−2e+05
Line
Manganese residual values in 2006 Urbana, IL
Manganese (Residual)
2000
0
−2000
Line
Iron residual values in 2006 Urbana, IL
Iron (Residual)
4000
0
−4000
−8000
Line
Cobalt residual values in 2006 Urbana, IL
Cobalt (Residual)
10
0
−10
Line

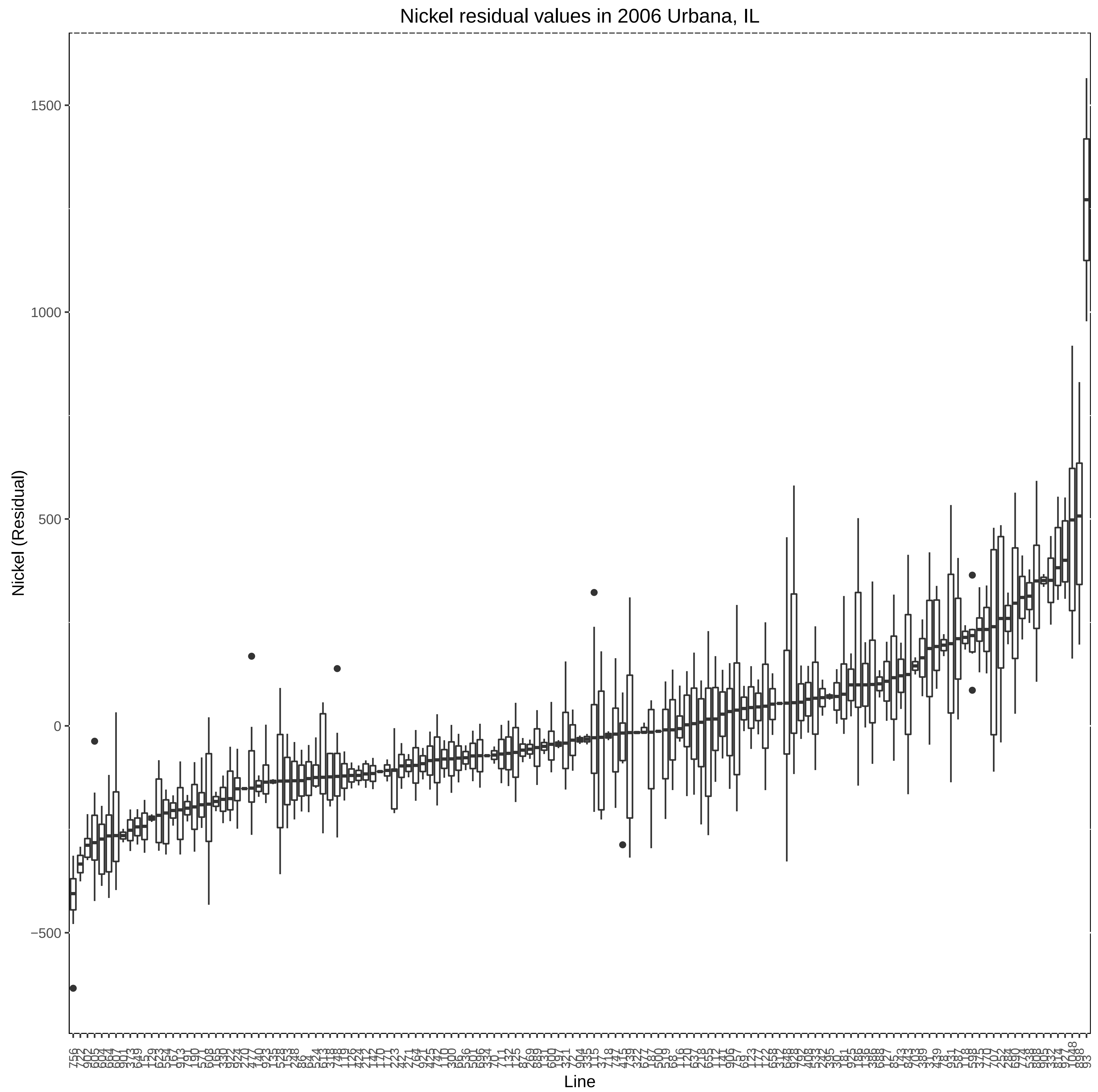
Nickel residual values in 2006 Urbana, IL
Nickel (Residual)
1500
1000
500
0
-500
Line
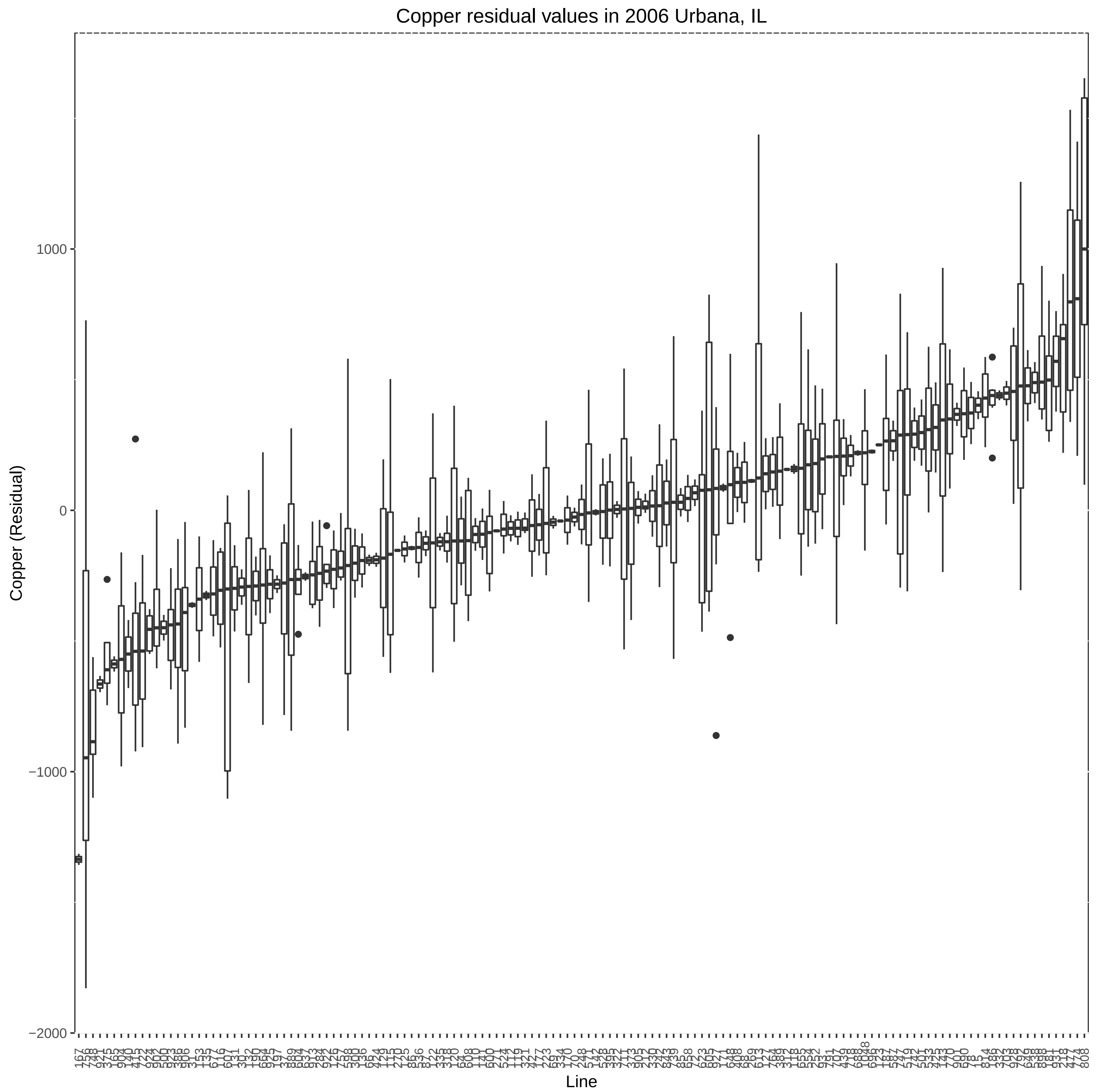
Copper residual values in 2006 Urbana, IL
Copper (Residual)
1000
0
-1000
-2000
Line
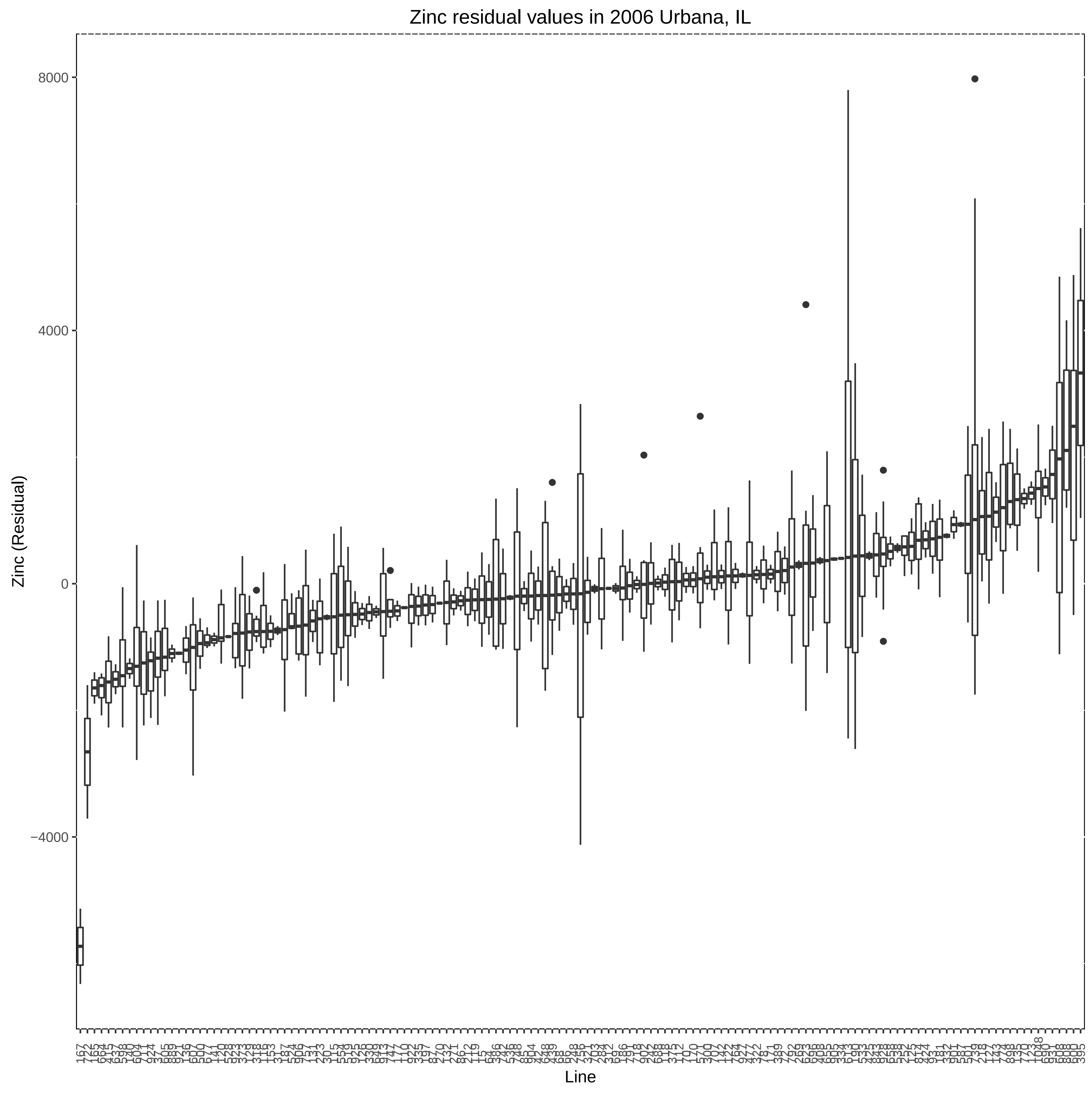
Zinc residual values in 2006 Urbana, IL
Zinc (Residual)
8000
4000
0
-4000
Line
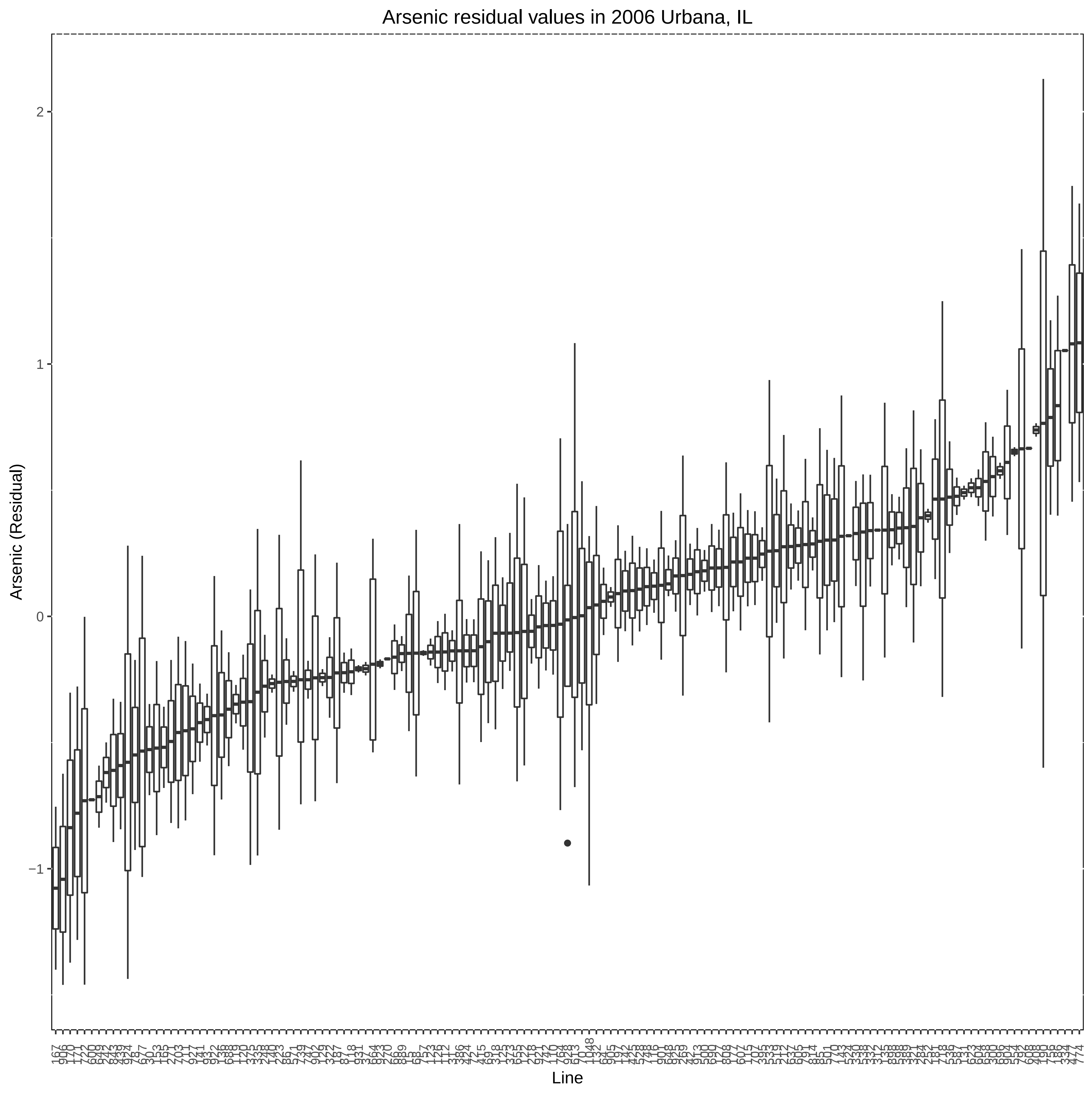
Arsenic residual values in 2006 Urbana, IL
Arsenic (Residual)
2
1
0
-1
Line

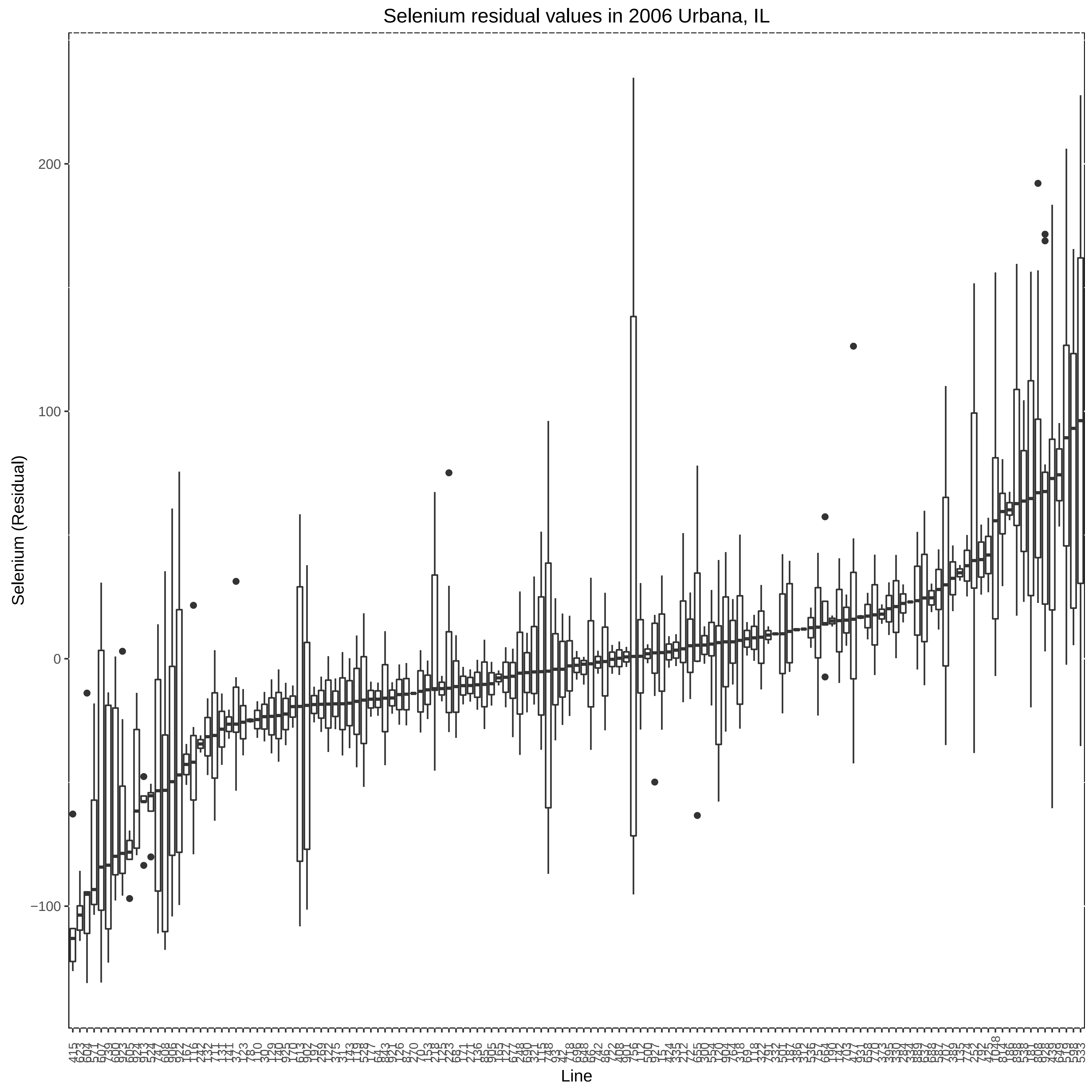
Selenium residual values in 2006 Urbana, IL
Selenium (Residual)
200
100
0
-100
Line

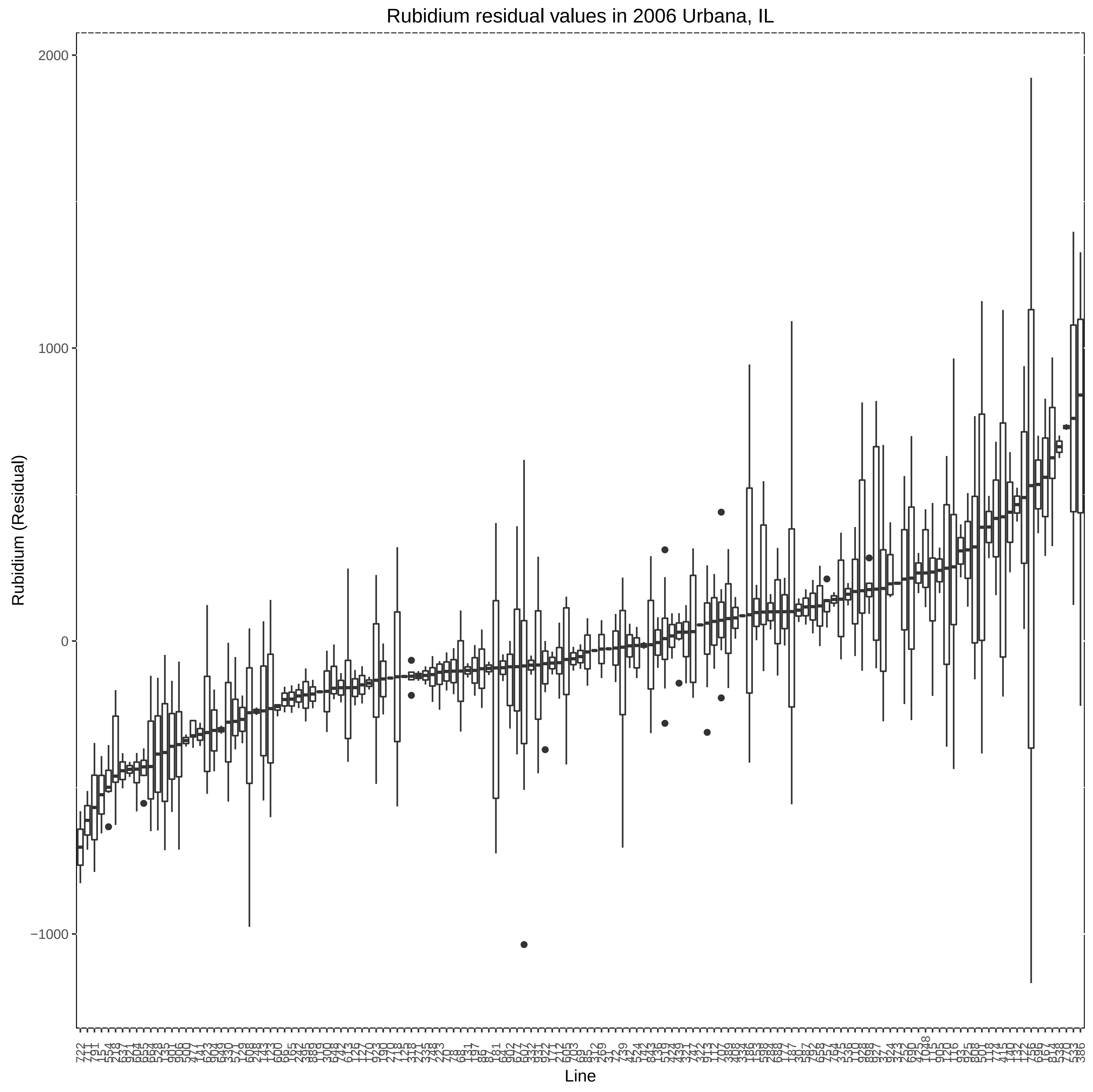
Rubidium residual values in 2006 Urbana, IL
Rubidium (Residual)
2000
1000
0
-1000
Line

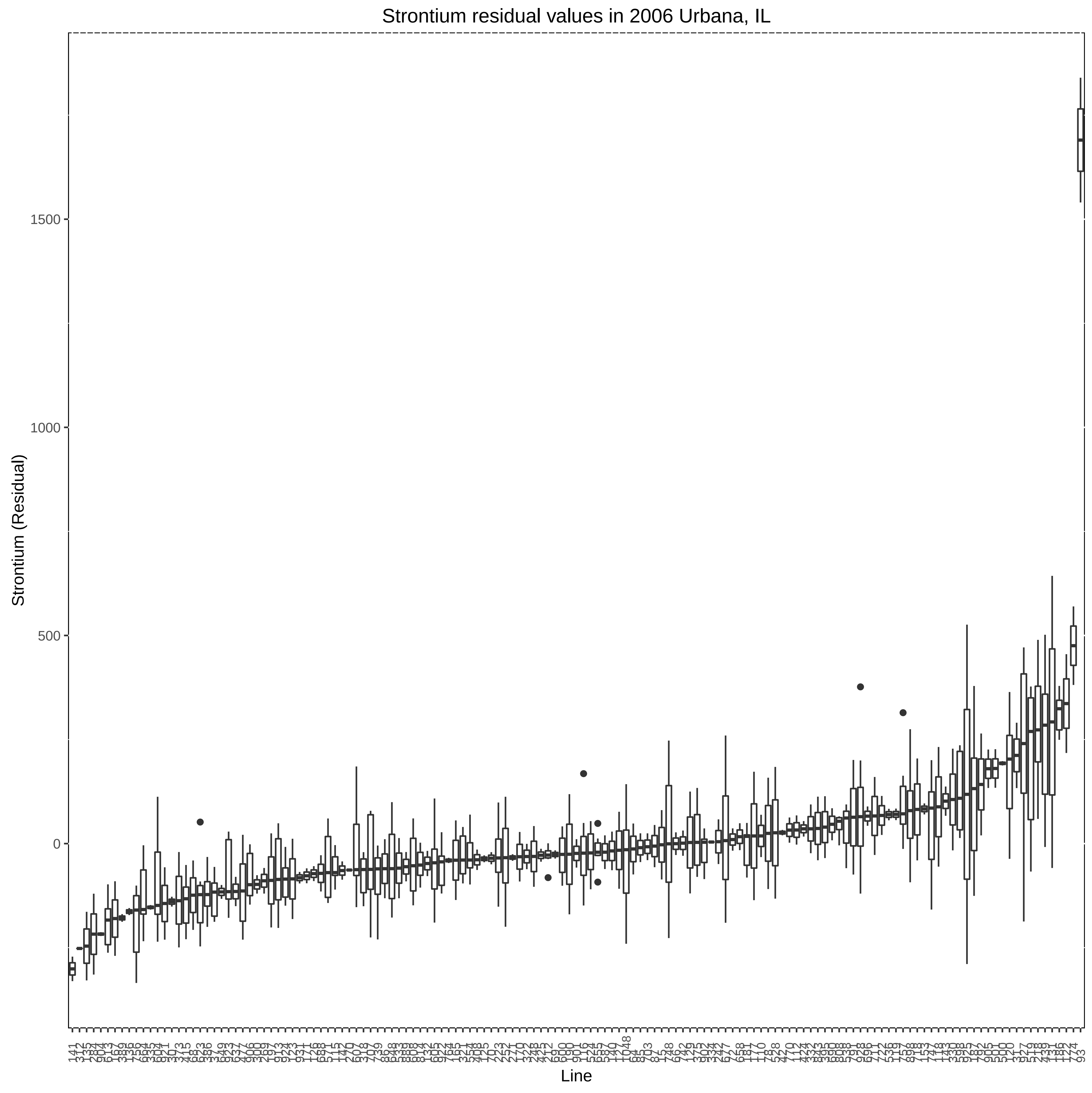
Strontium residual values in 2006 Urbana, IL
Strontium (Residual)
1500
1000
500
0
Line

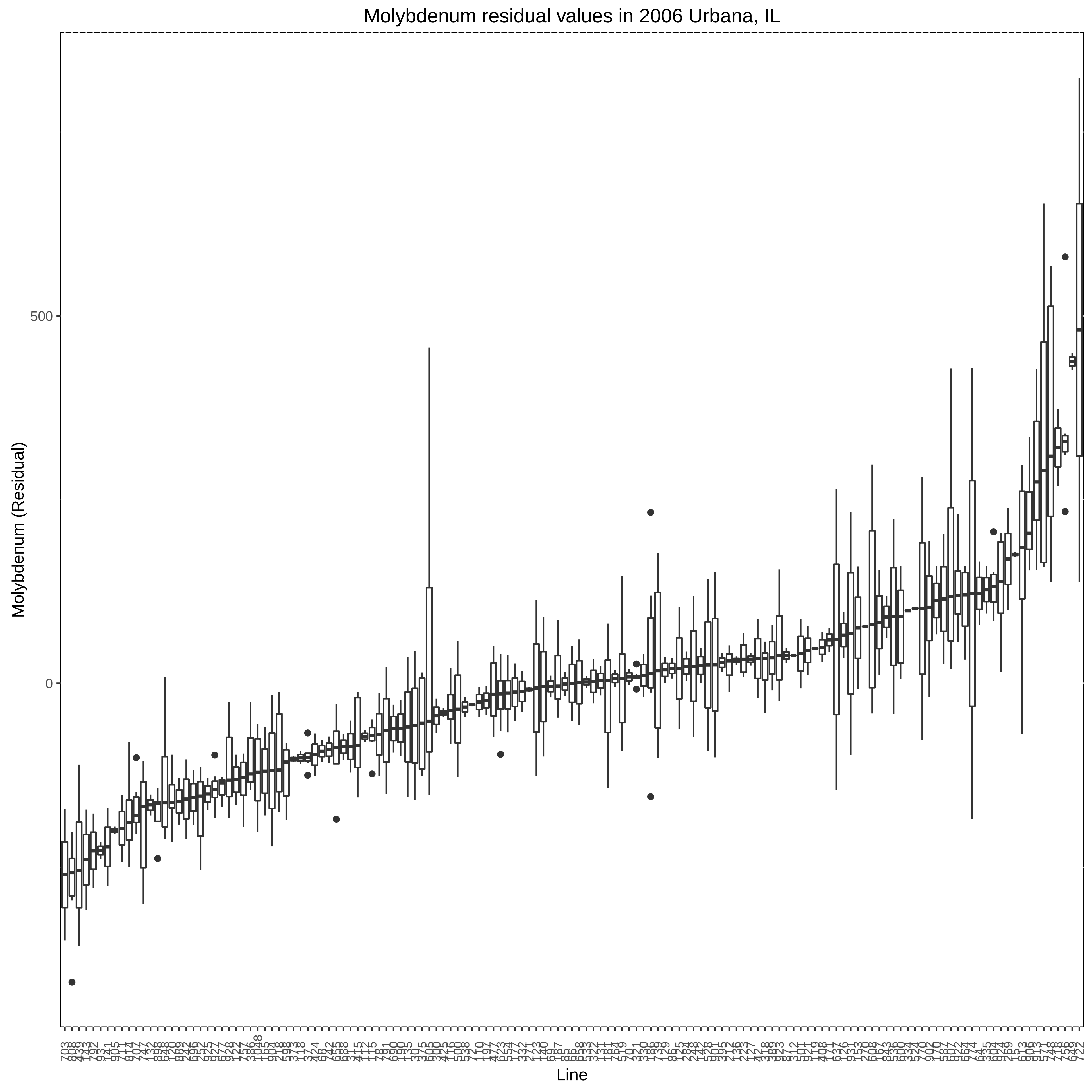
Molybdenum residual values in 2006 Urbana, IL
Molybdenum (Residual)
500
0
Line

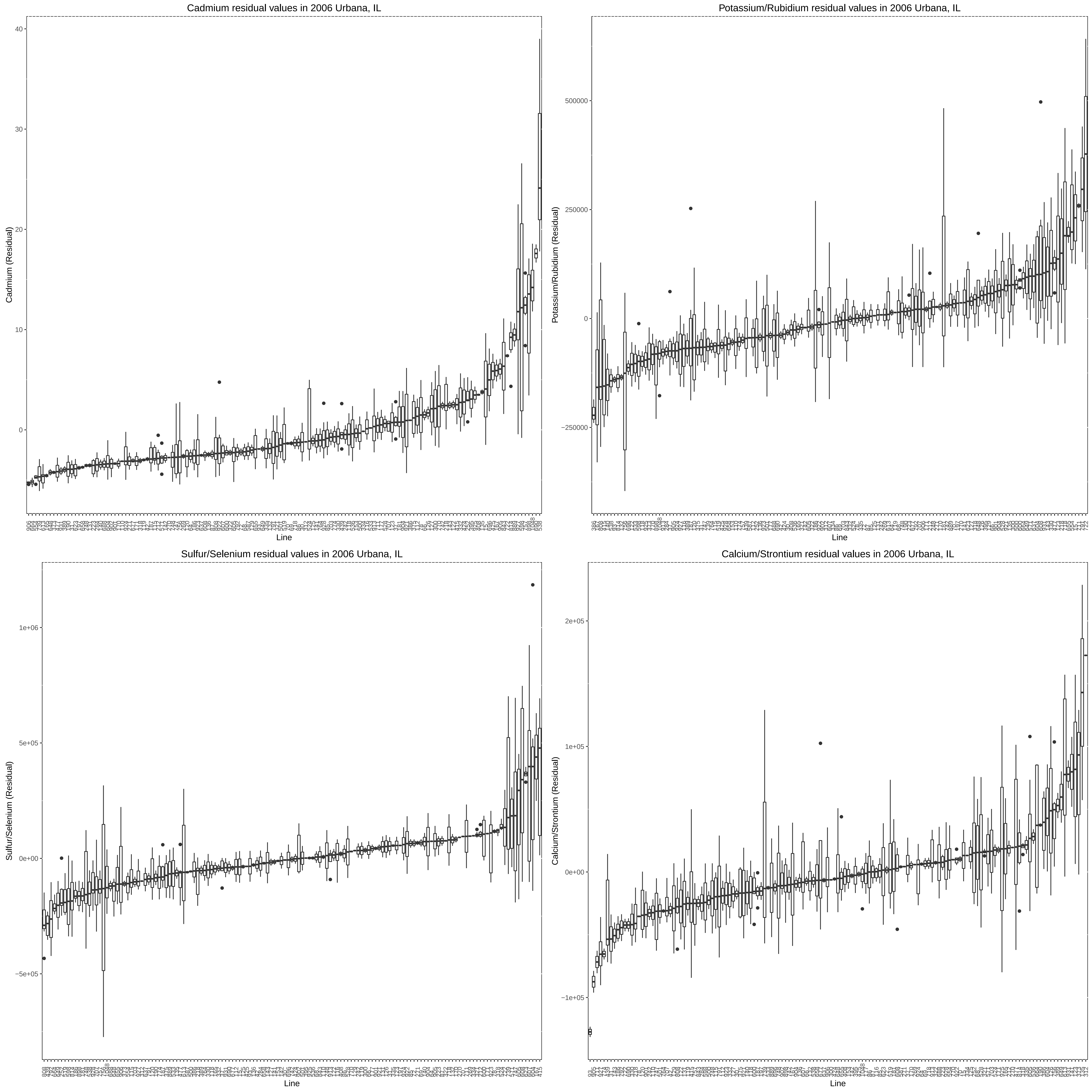

Cadmium residual values in 2006 Urbana, IL
Cadmium (Residual)
0
10
20
30
40
Line
Potassium/Rubidium residual values in 2006 Urbana, IL
Potassium/Rubidium (Residual)
−250000
0
250000
500000
Line
Sulfur/Selenium residual values in 2006 Urbana, IL
Sulfur/Selenium (Residual)
−5e+05
0e+00
5e+05
1e+06
Line
Calcium/Strontium residual values in 2006 Urbana, IL
Calcium/Strontium (Residual)
−1e+05
0e+00
1e+05
2e+05
Line

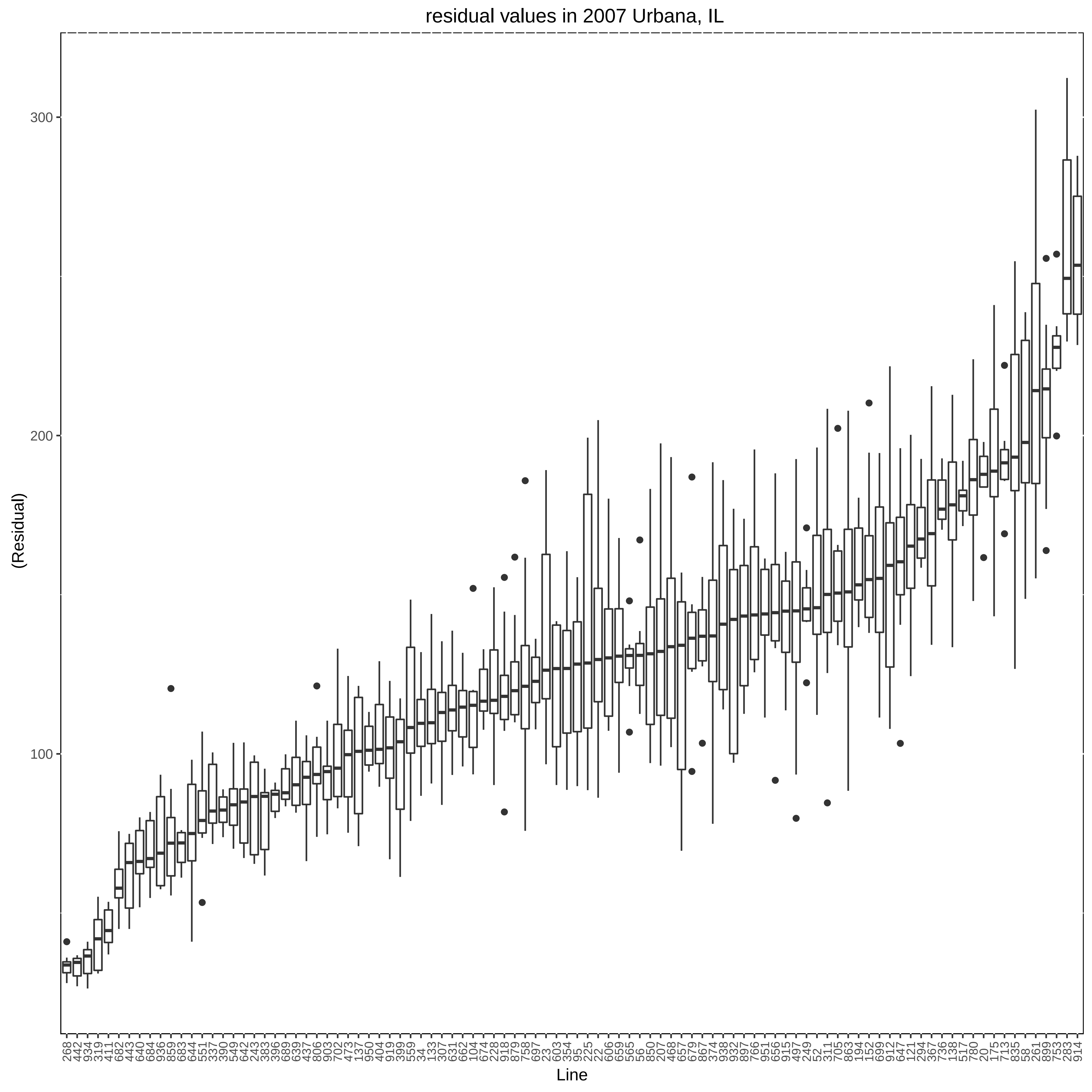
residual values in 2007 Urbana, IL
(Residual)
300
200
100
Line

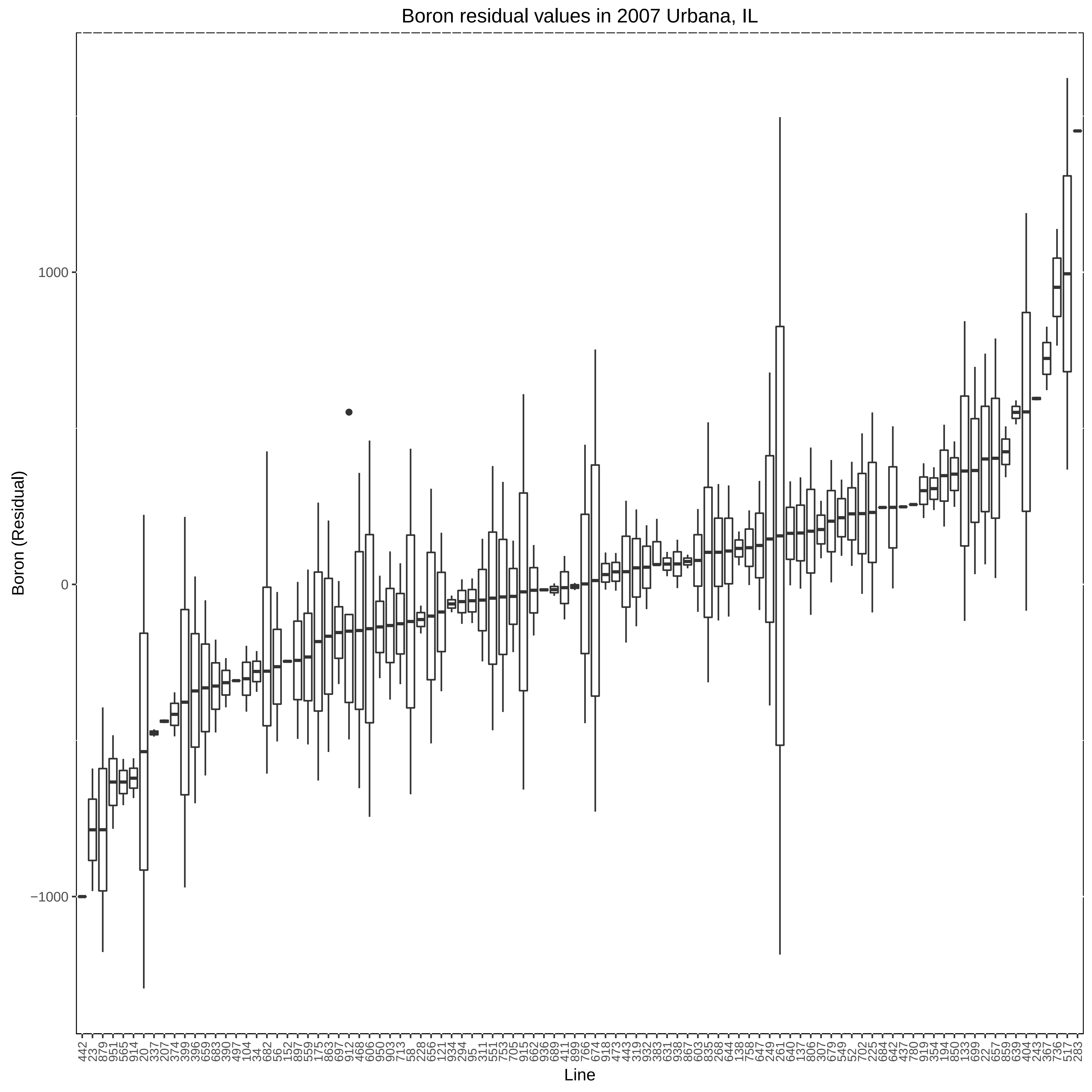
Boron residual values in 2007 Urbana, IL
Boron (Residual)
1000
0
−1000
Line

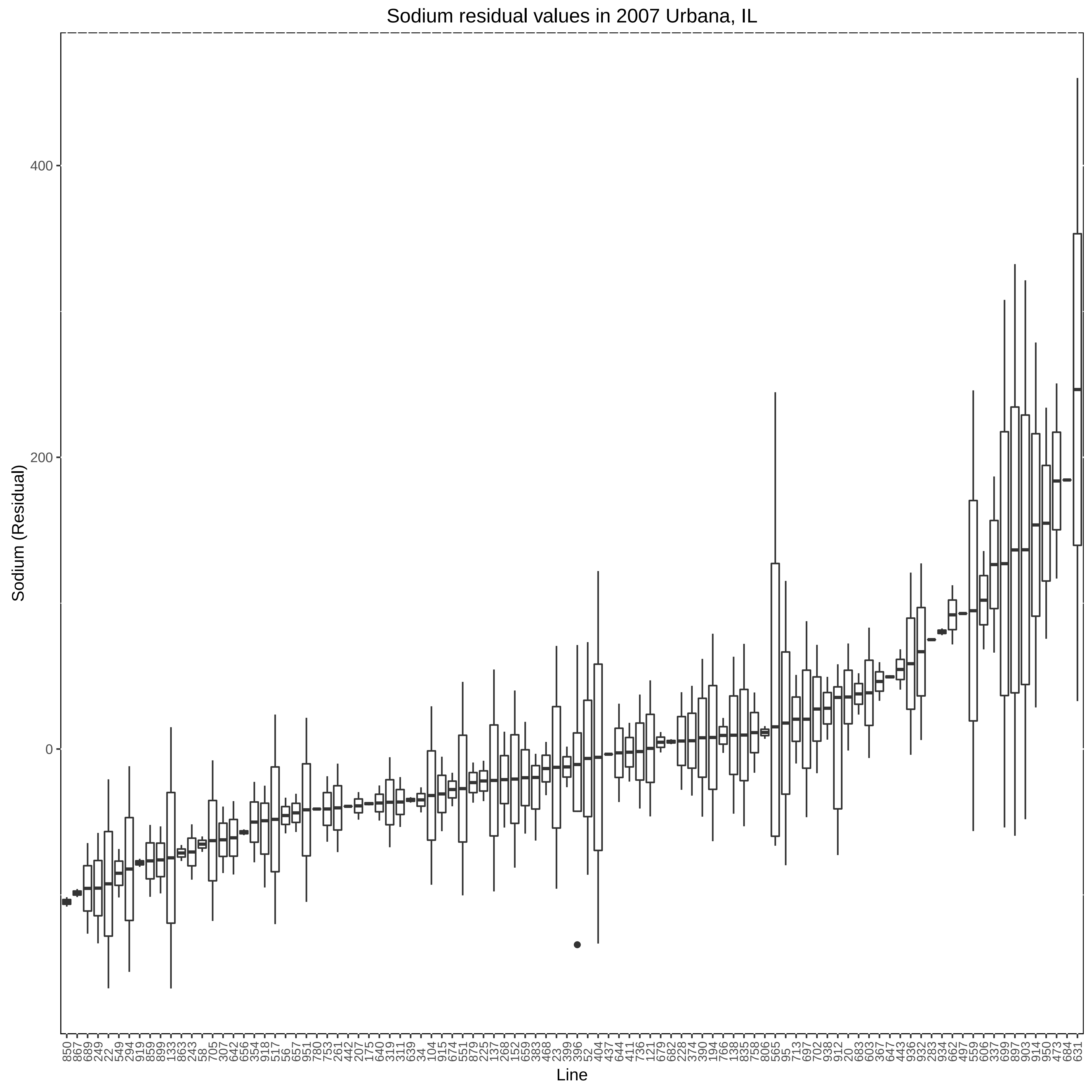
Sodium residual values in 2007 Urbana, IL
Sodium (Residual)
400
200
0
Line

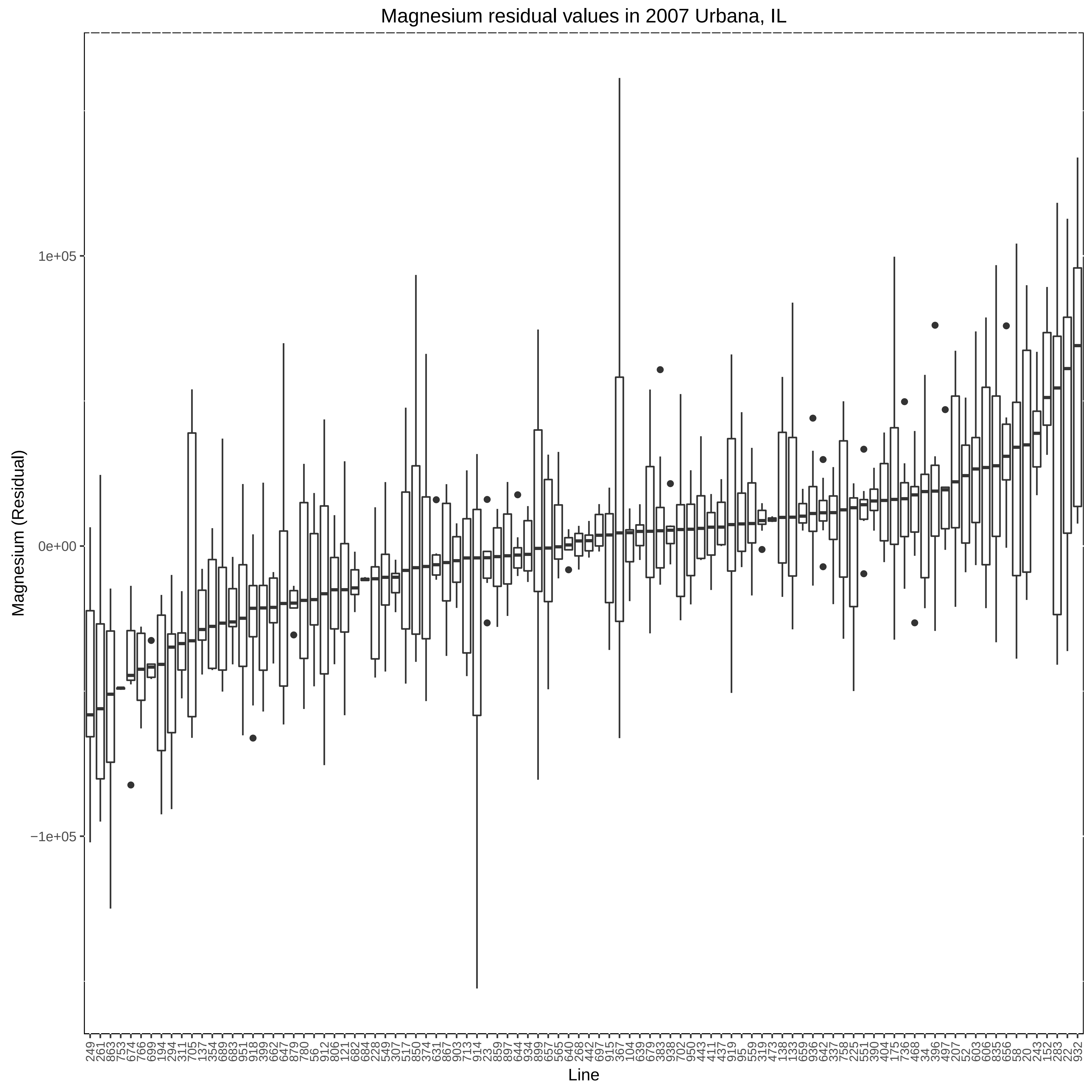
Magnesium residual values in 2007 Urbana, IL
Magnesium (Residual)
1e+05
0e+00
−1e+05
Line

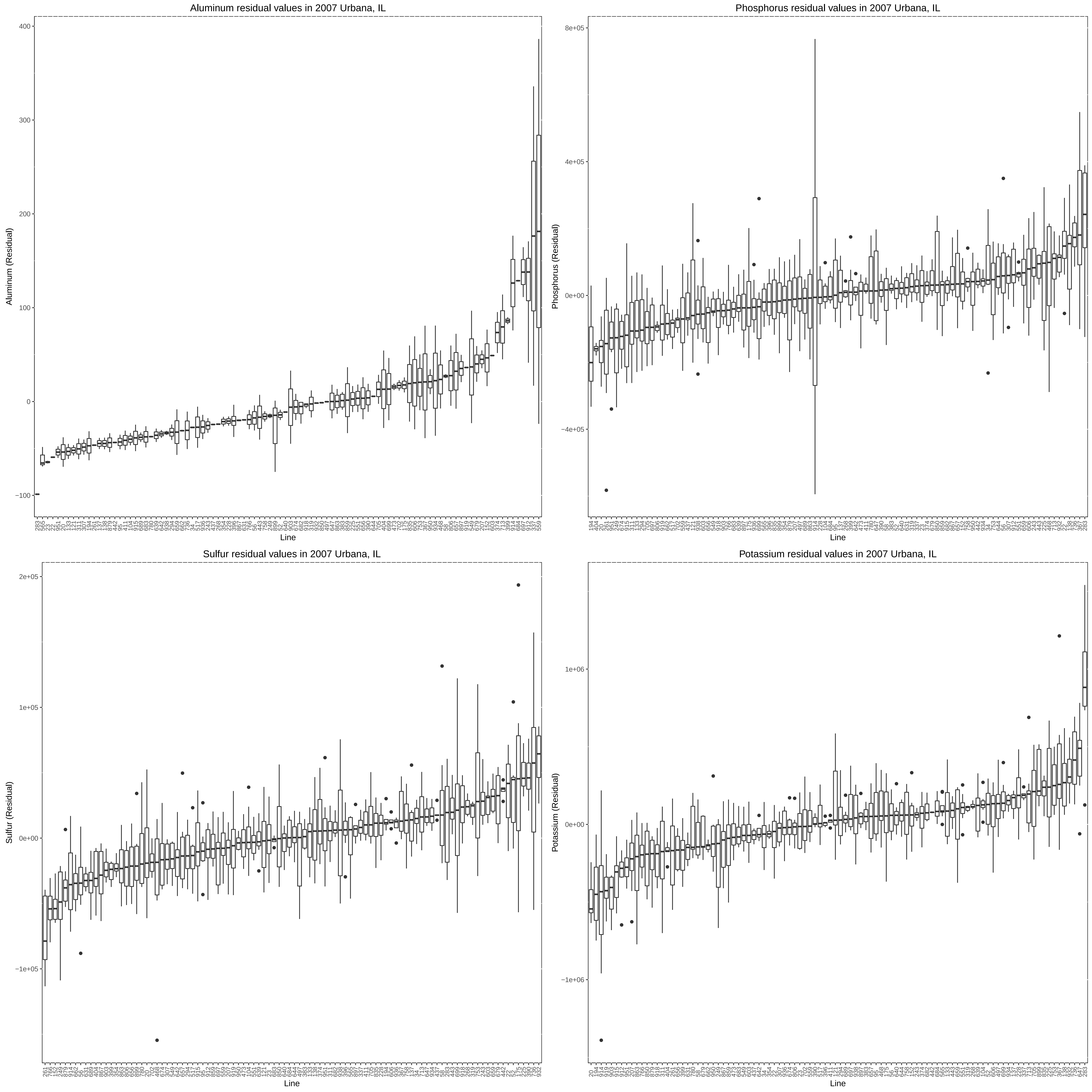
Aluminum residual values in 2007 Urbana, IL
Aluminum (Residual)
Line
400
300
200
100
0
-100
Phosphorus residual values in 2007 Urbana, IL
Phosphorus (Residual)
Line
8e+05
4e+05
0e+00
-4e+05
Sulfur residual values in 2007 Urbana, IL
Sulfur (Residual)
Line
2e+05
1e+05
0e+00
-1e+05
Potassium residual values in 2007 Urbana, IL
Potassium (Residual)
Line
1e+06
0e+00
-1e+06

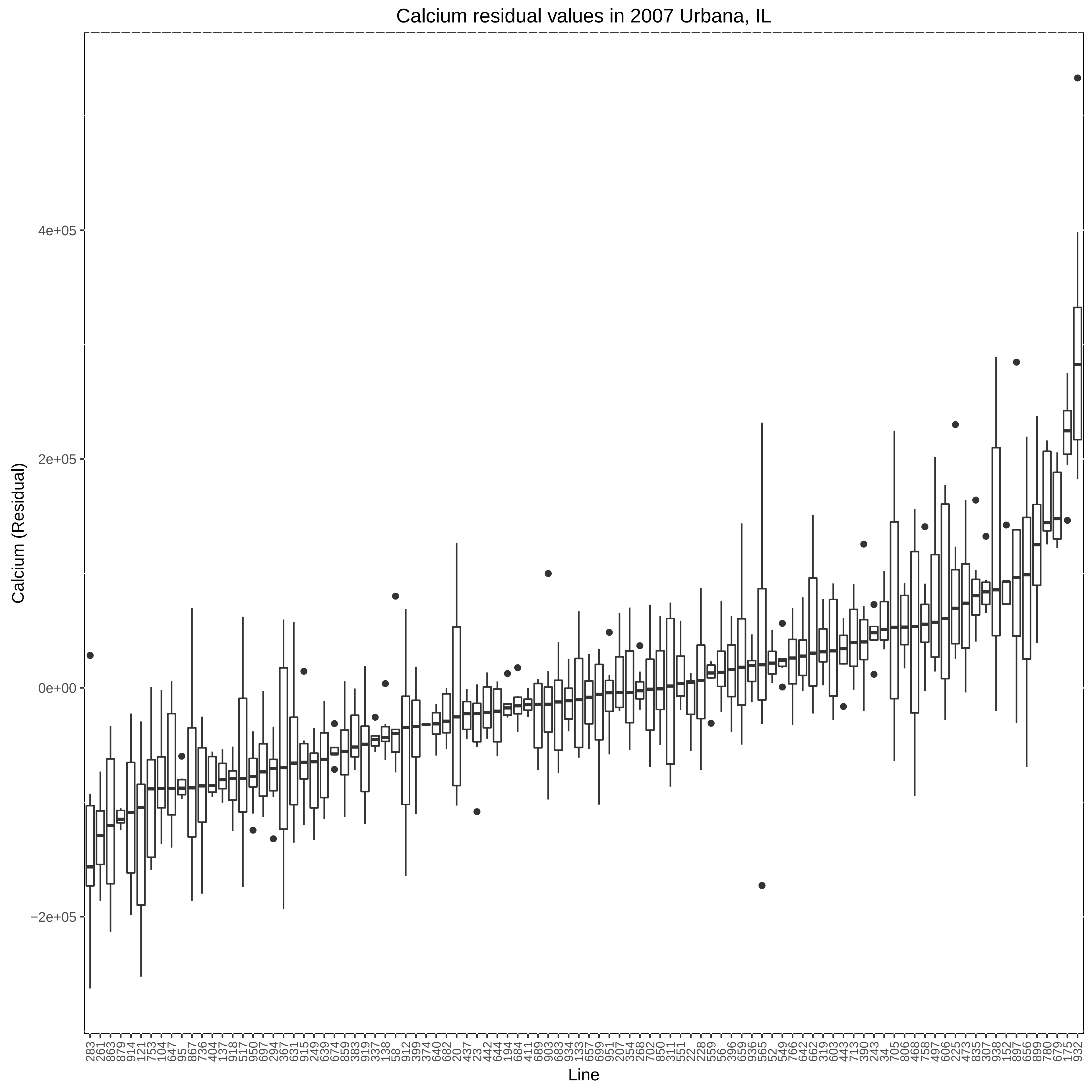
Calcium residual values in 2007 Urbana, IL
Calcium (Residual)
4e+05
2e+05
0e+00
-2e+05
Line

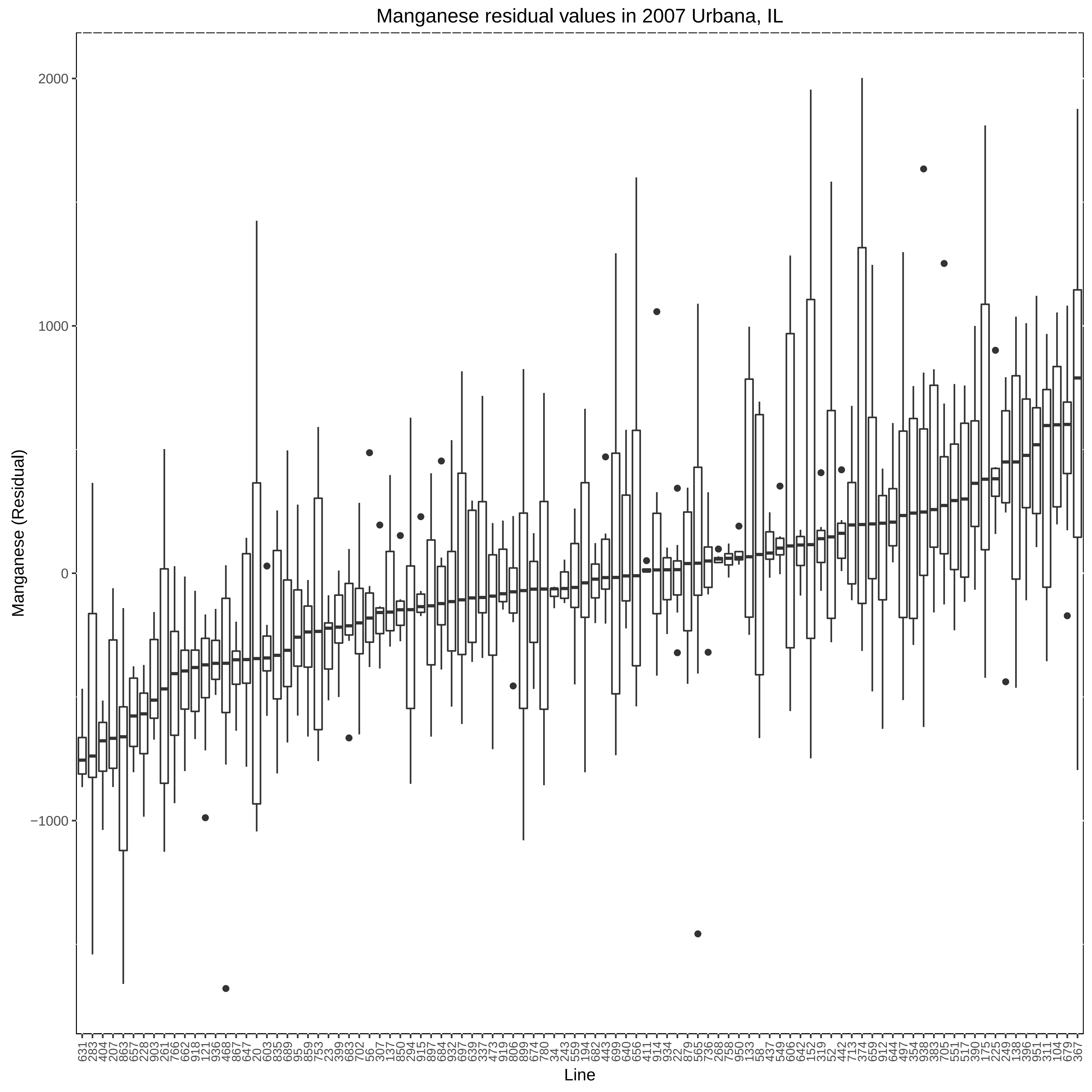
Manganese residual values in 2007 Urbana, IL
Manganese (Residual)
2000
1000
0
-1000
Line

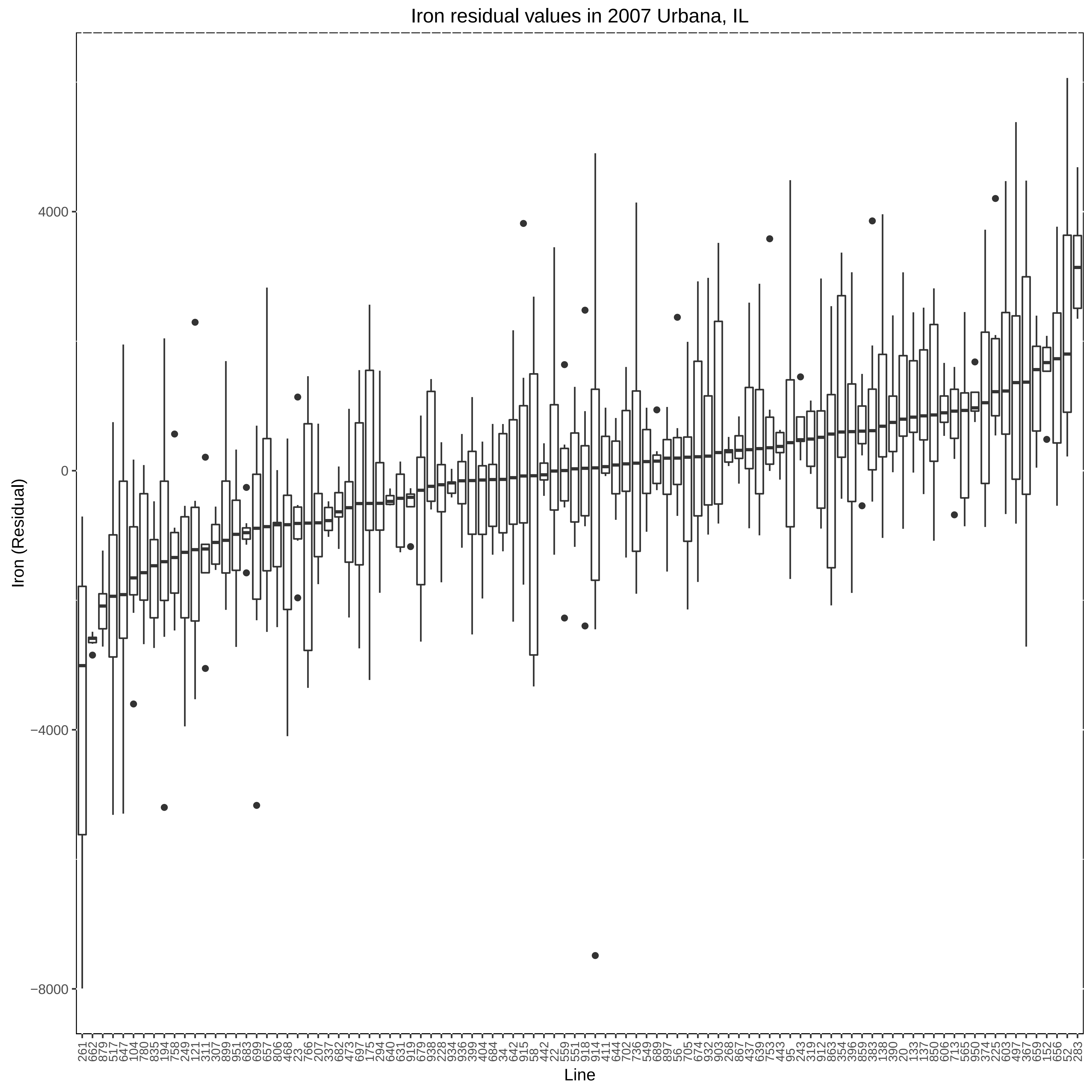
Iron residual values in 2007 Urbana, IL
Iron (Residual)
4000
0
-4000
-8000
Line

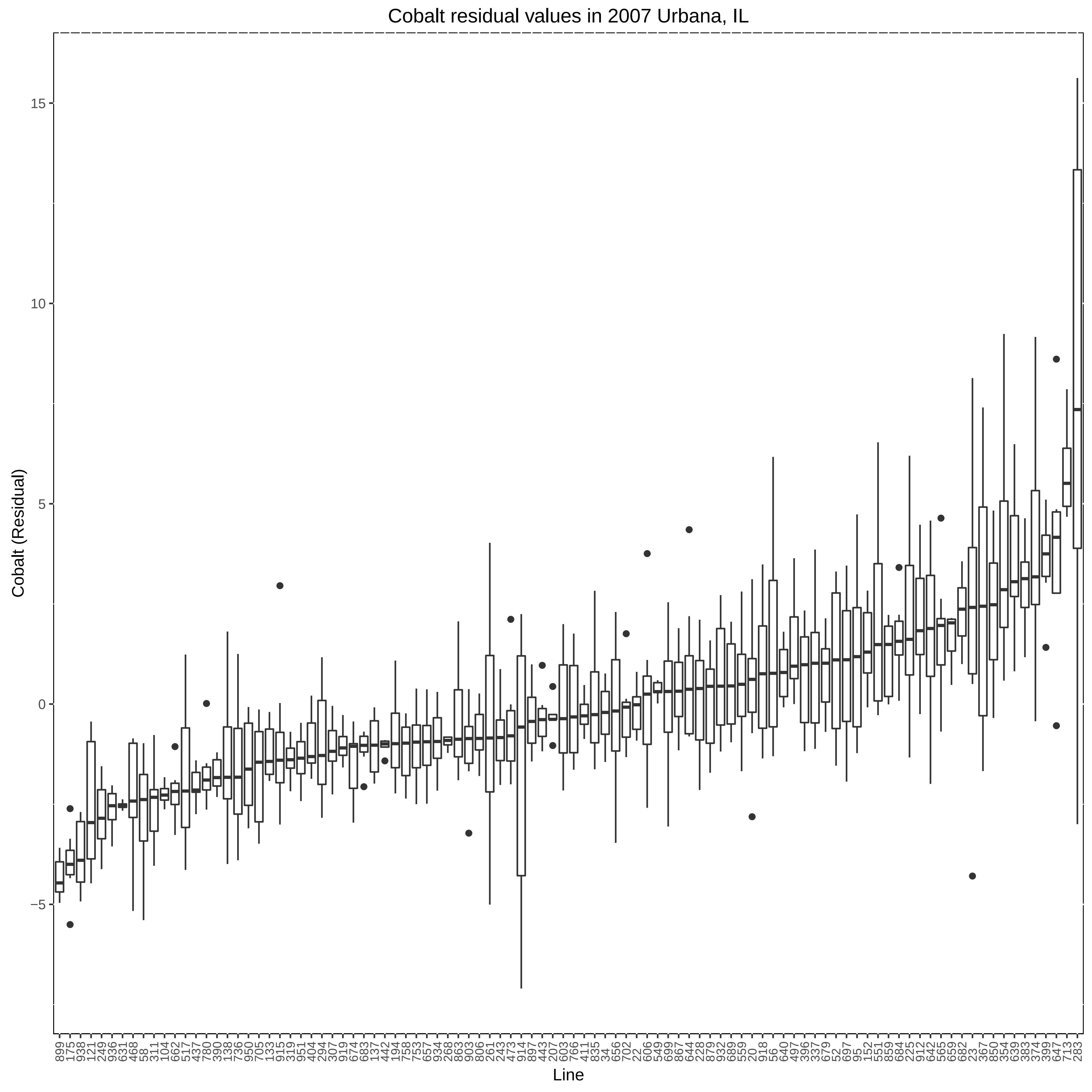
Cobalt residual values in 2007 Urbana, IL
Cobalt (Residual)
15
10
5
0
-5
Line

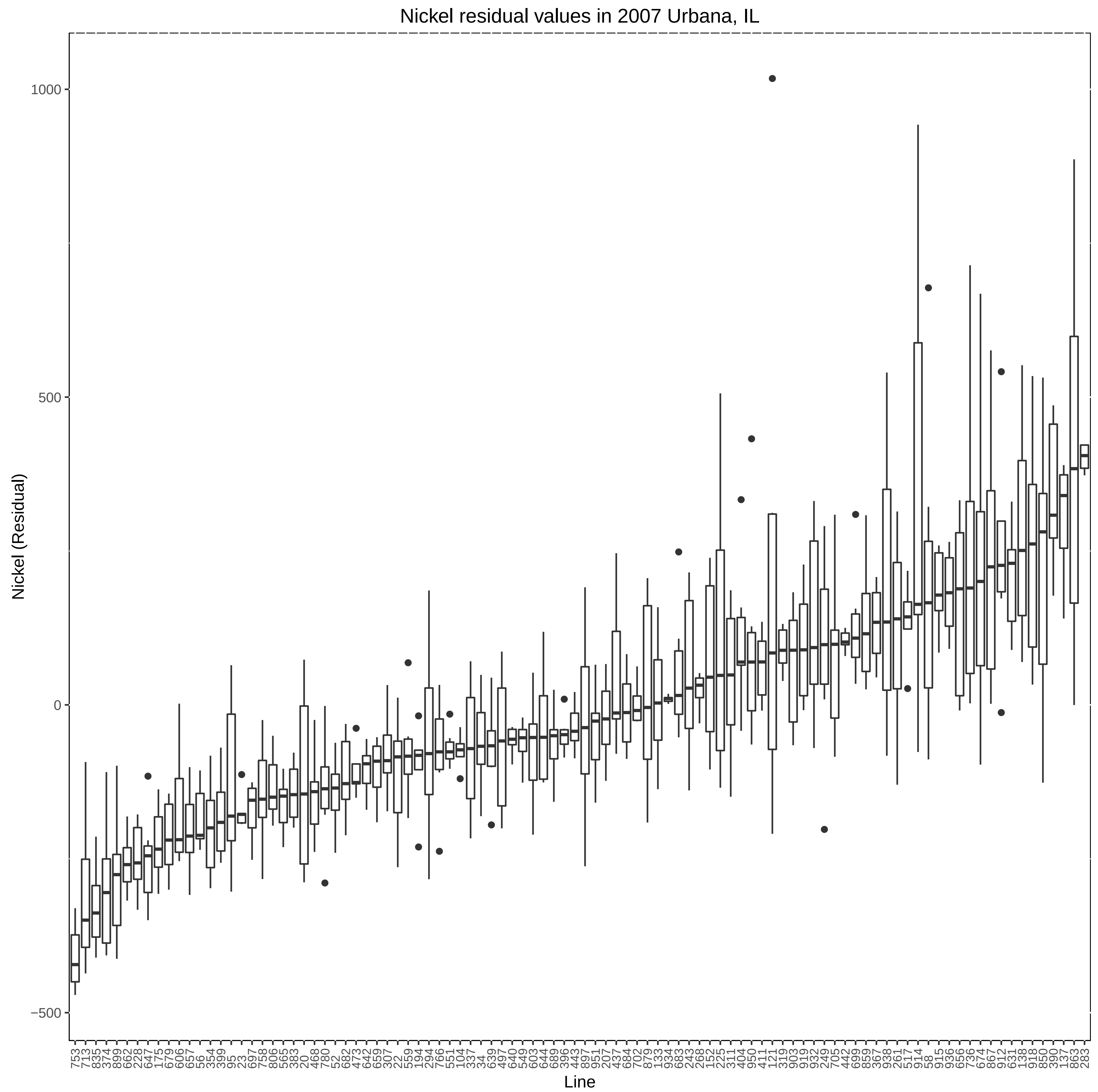
Nickel residual values in 2007 Urbana, IL
Nickel (Residual)
1000
500
0
-500
Line

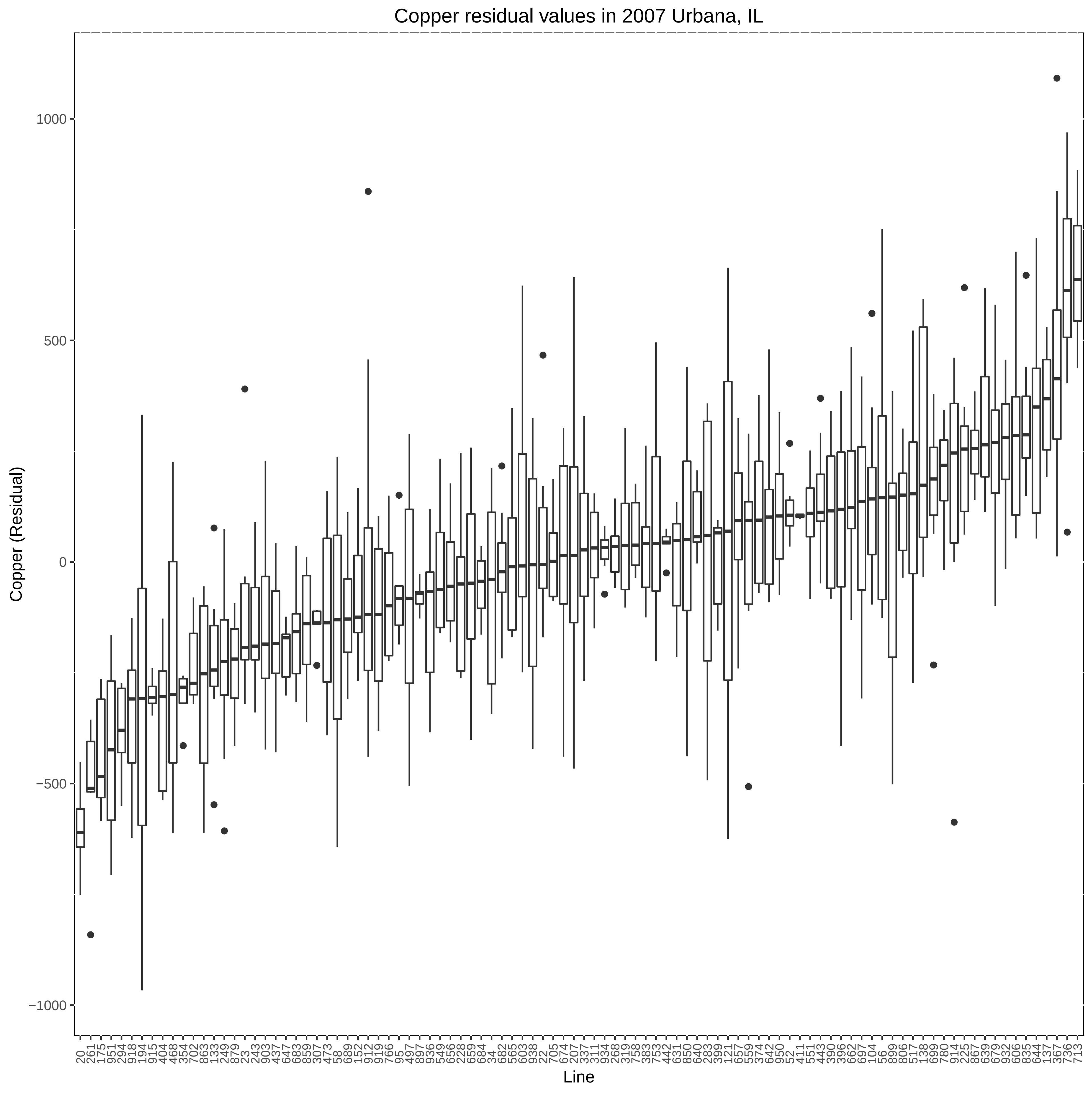
Copper residual values in 2007 Urbana, IL
Copper (Residual)
1000
500
0
-500
-1000
Line

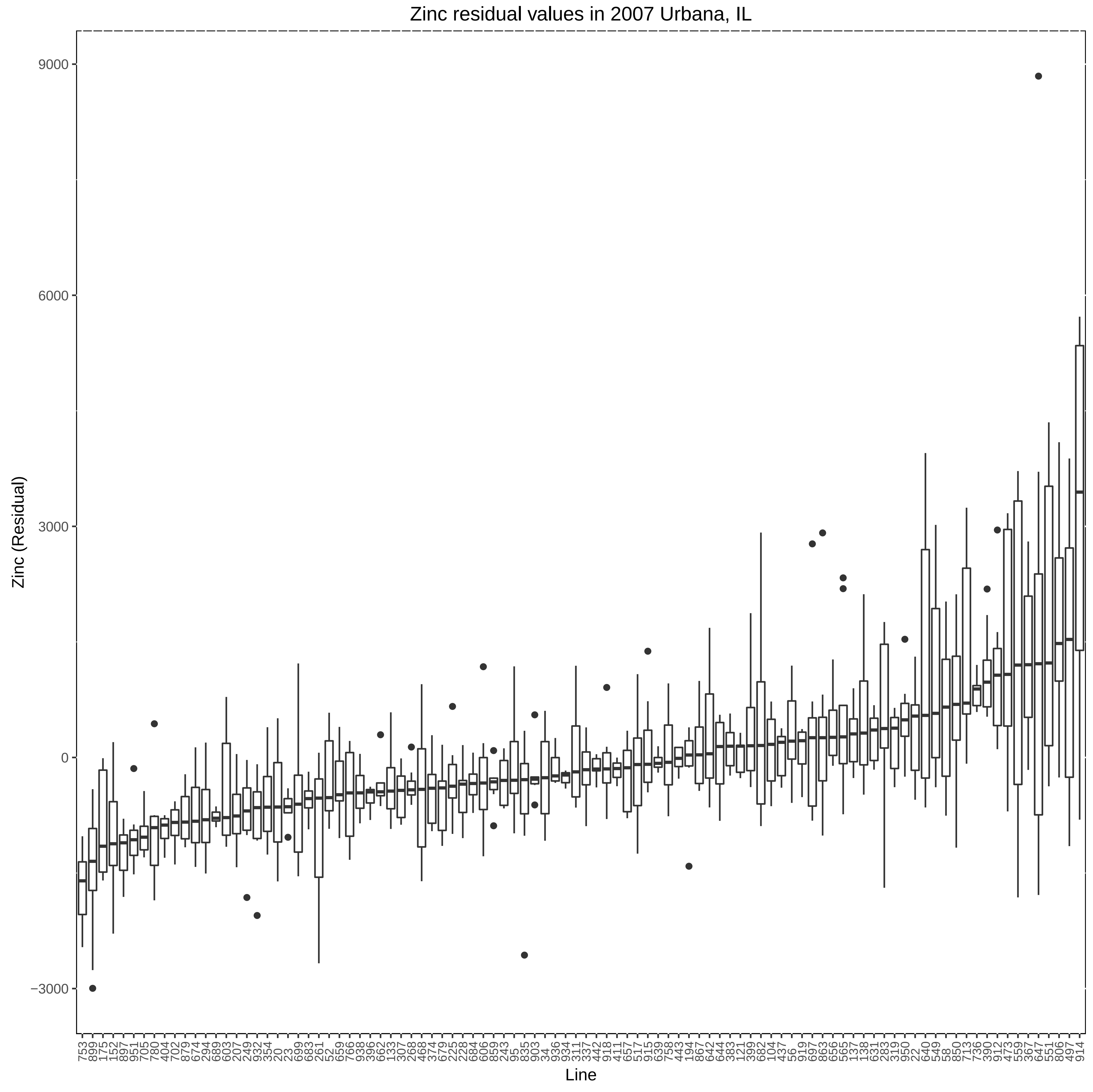
Zinc residual values in 2007 Urbana, IL
Zinc (Residual)
9000
6000
3000
0
-3000
Line

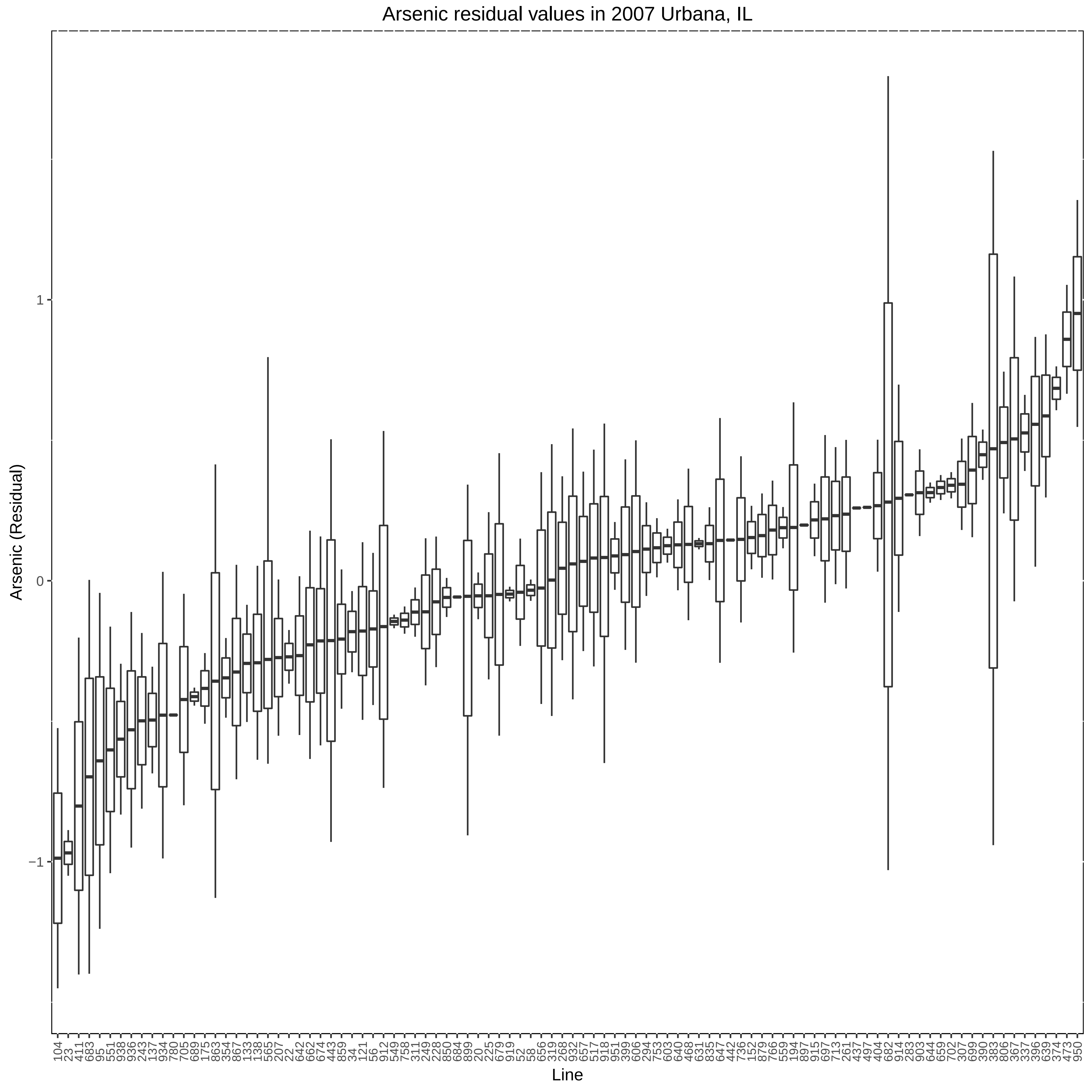
Arsenic residual values in 2007 Urbana, IL
Arsenic (Residual)
1
0
-1
Line

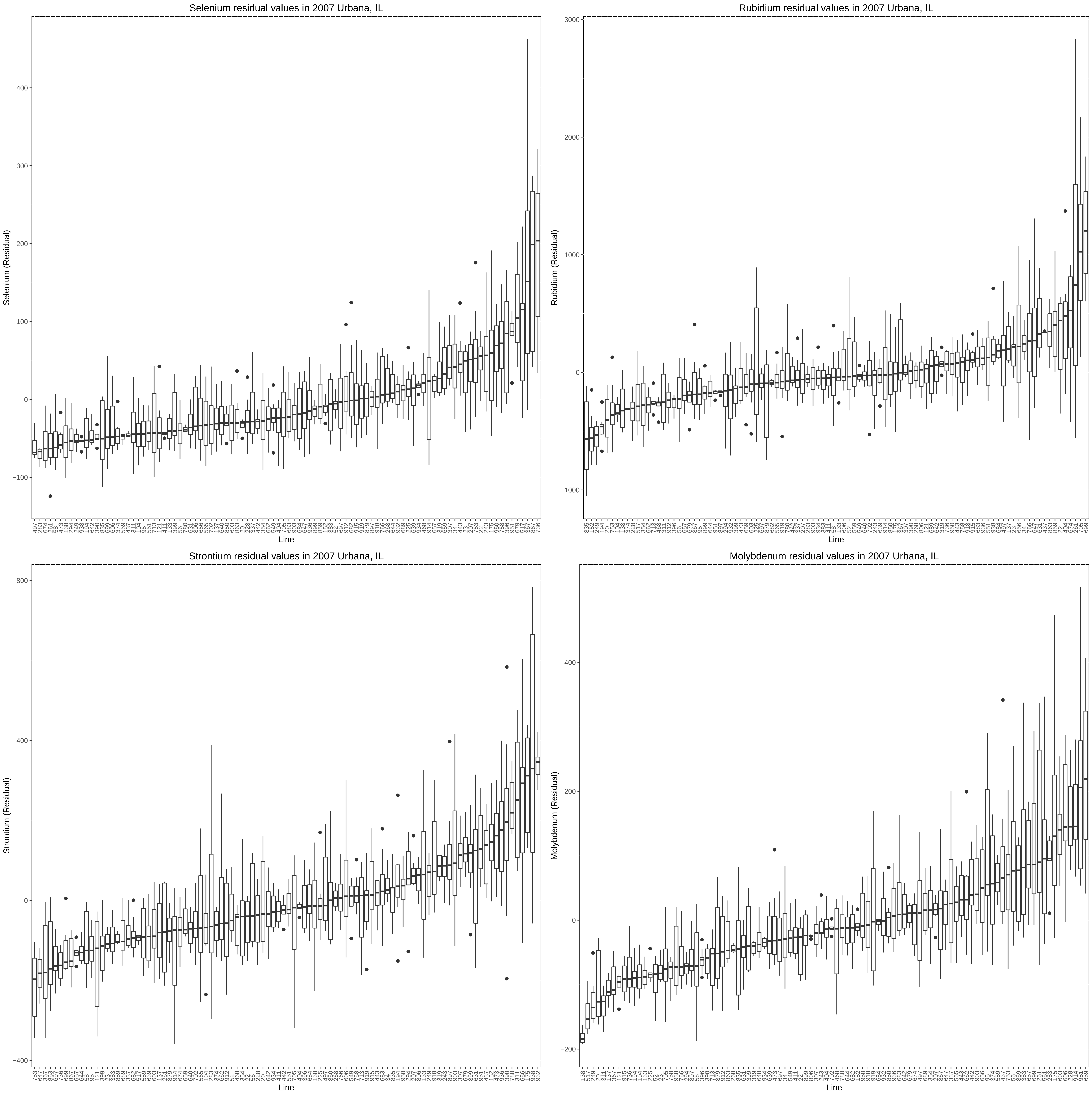
Selenium residual values in 2007 Urbana, IL
Selenium (Residual)
400
300
200
100
0
−100
Line
Rubidium residual values in 2007 Urbana, IL
Rubidium (Residual)
3000
2000
1000
0
−1000
Line
Strontium residual values in 2007 Urbana, IL
Strontium (Residual)
800
400
0
−400
Line
Molybdenum residual values in 2007 Urbana, IL
Molybdenum (Residual)
400
200
0
−200
Line

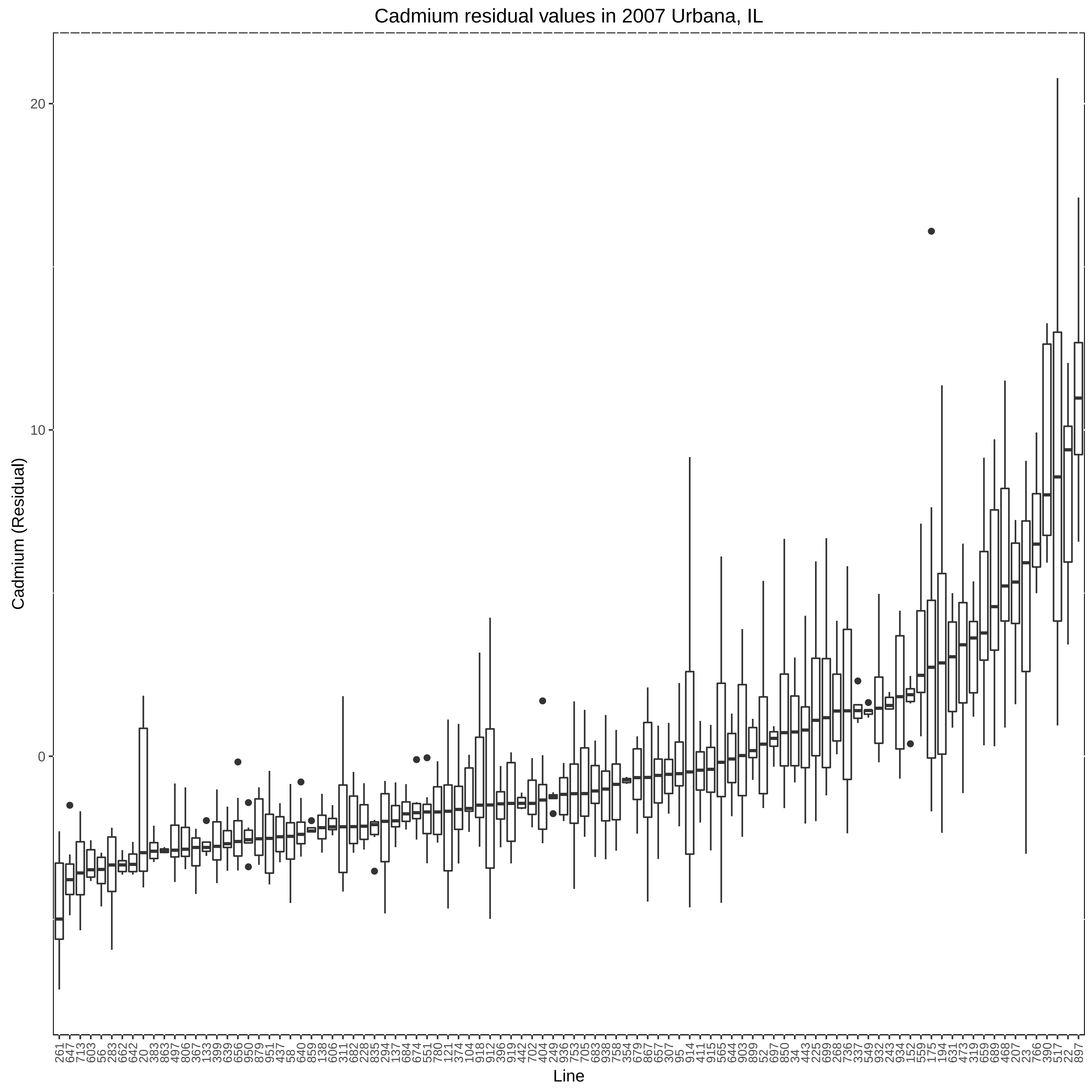
Cadmium residual values in 2007 Urbana, IL
Cadmium (Residual)
20
10
0
Line

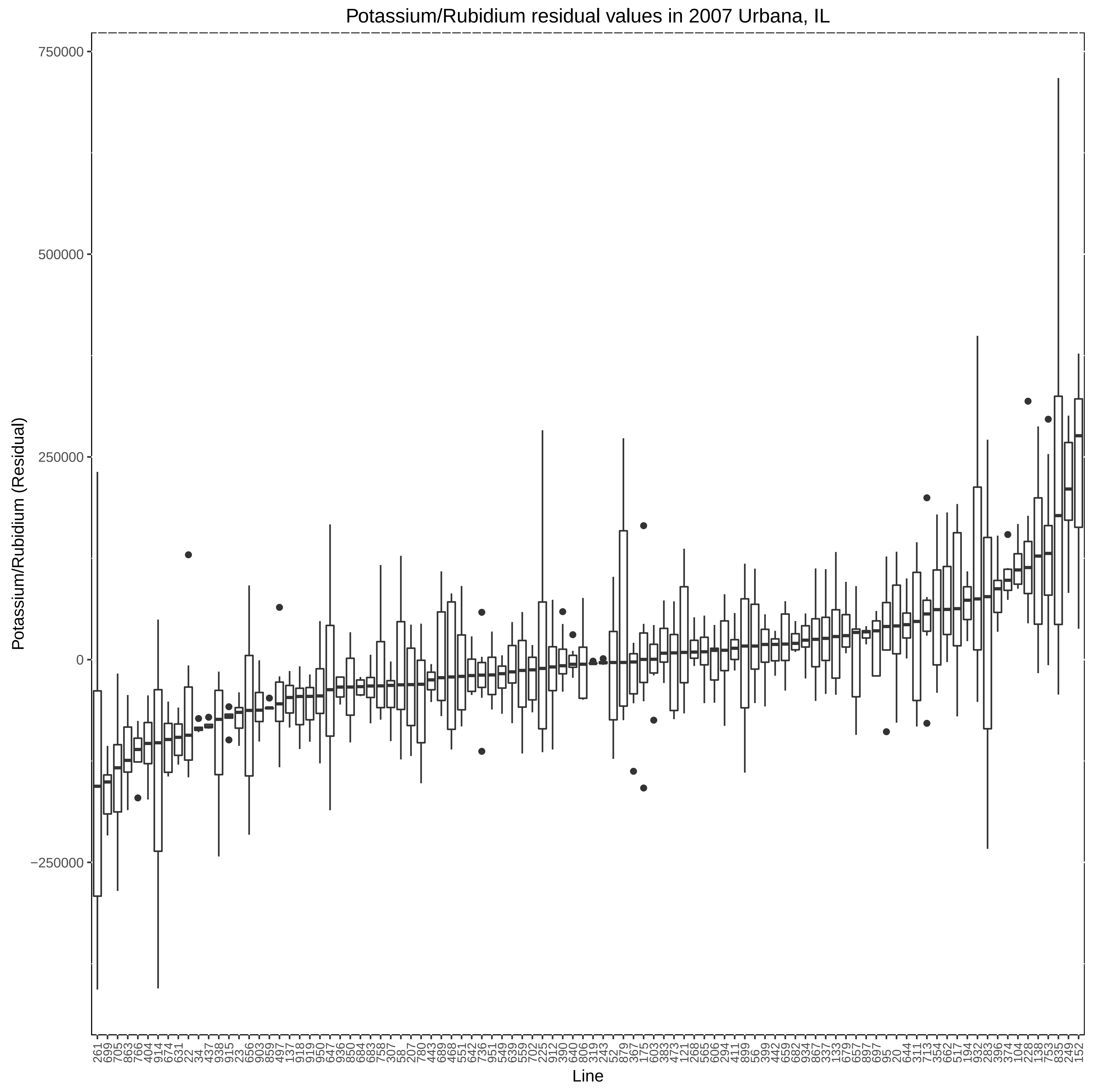
Potassium/Rubidium residual values in 2007 Urbana, IL
Potassium/Rubidium (Residual)
750000
500000
250000
0
-250000
Line

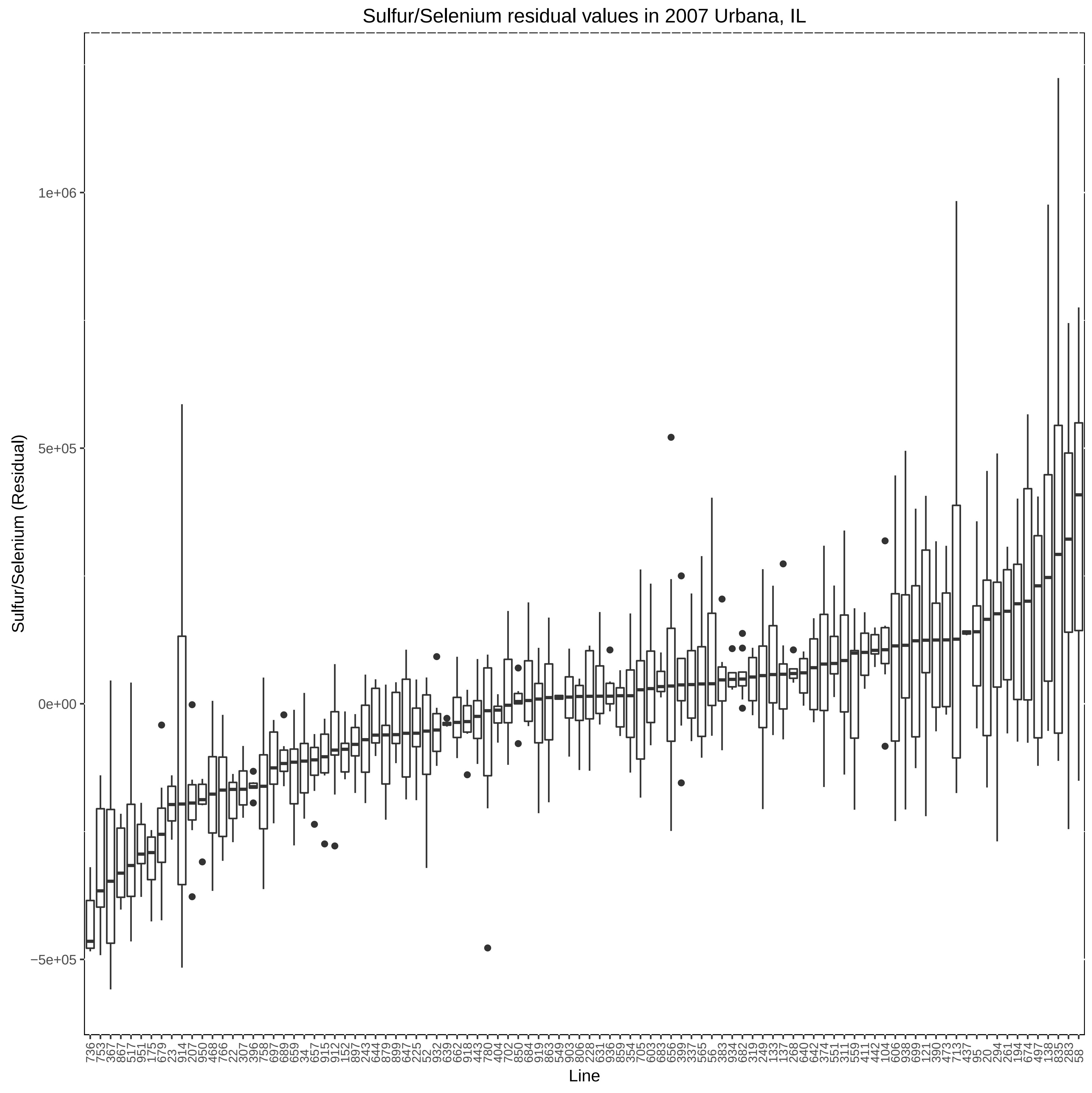
Sulfur/Selenium residual values in 2007 Urbana, IL
Sulfur/Selenium (Residual)
1e+06
5e+05
0e+00
-5e+05
Line

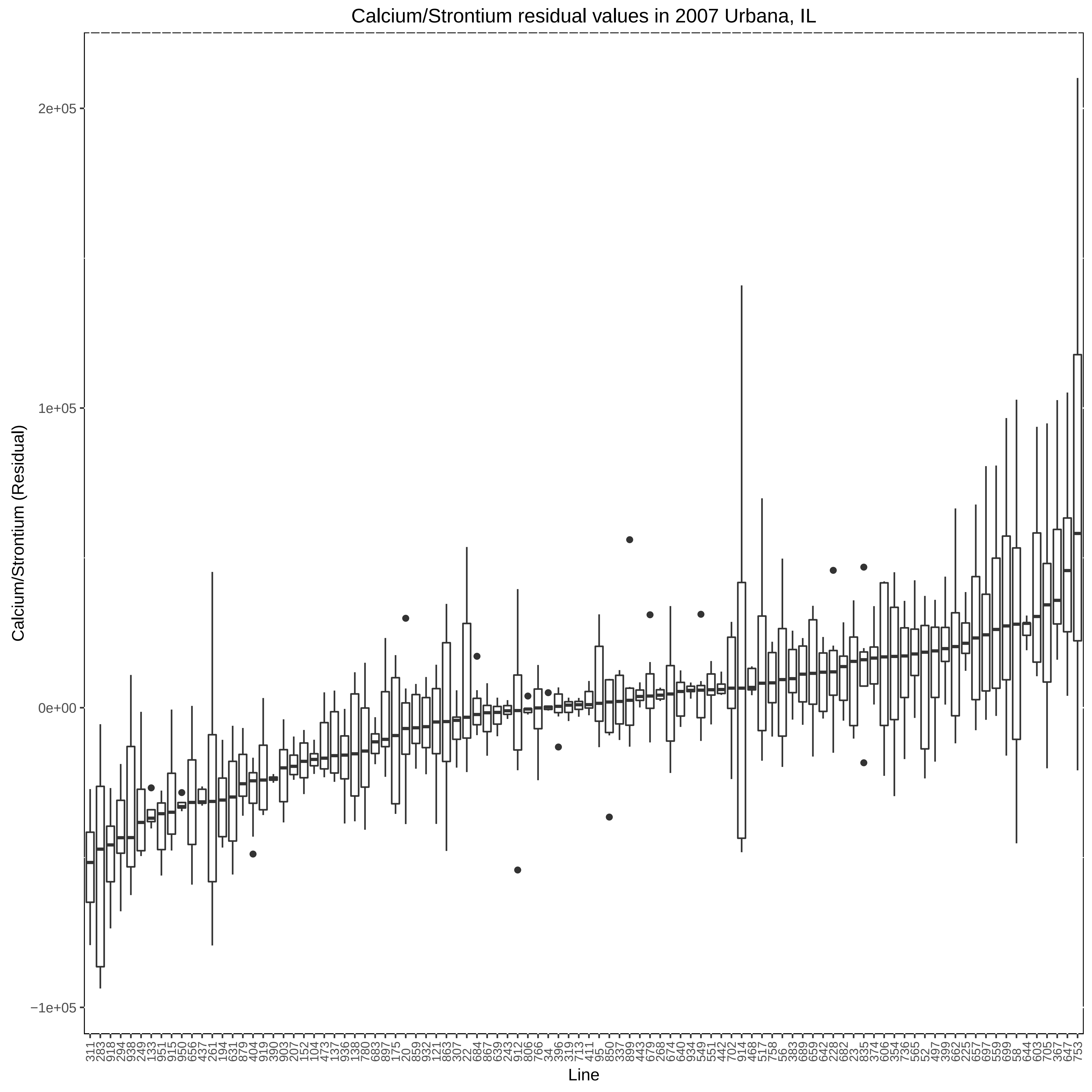
Calcium/Strontium residual values in 2007 Urbana, IL
Calcium/Strontium (Residual)
2e+05
1e+05
0e+00
-1e+05
Line

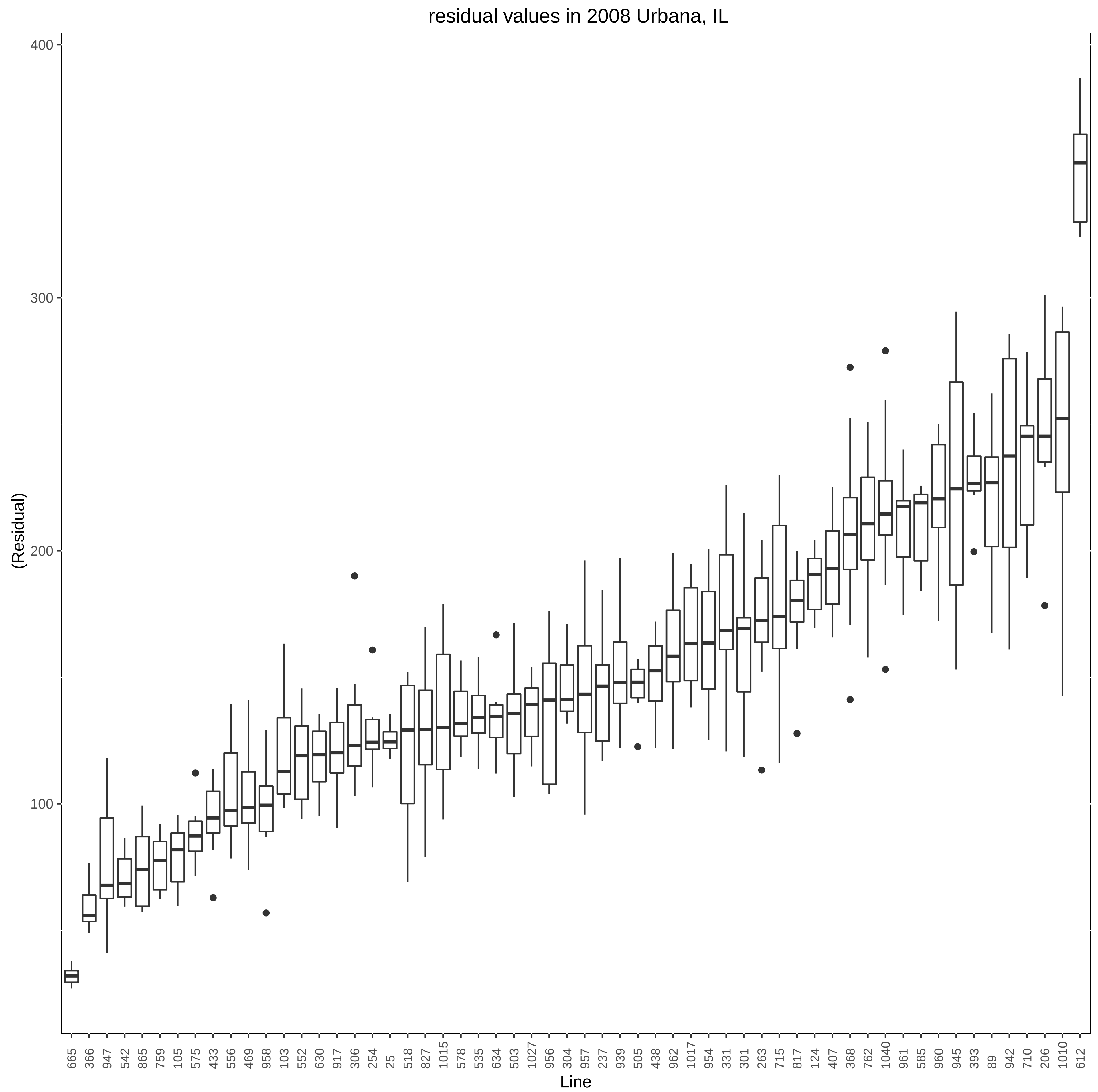
residual values in 2008 Urbana, IL
(Residual)
Line
100
200
300
400

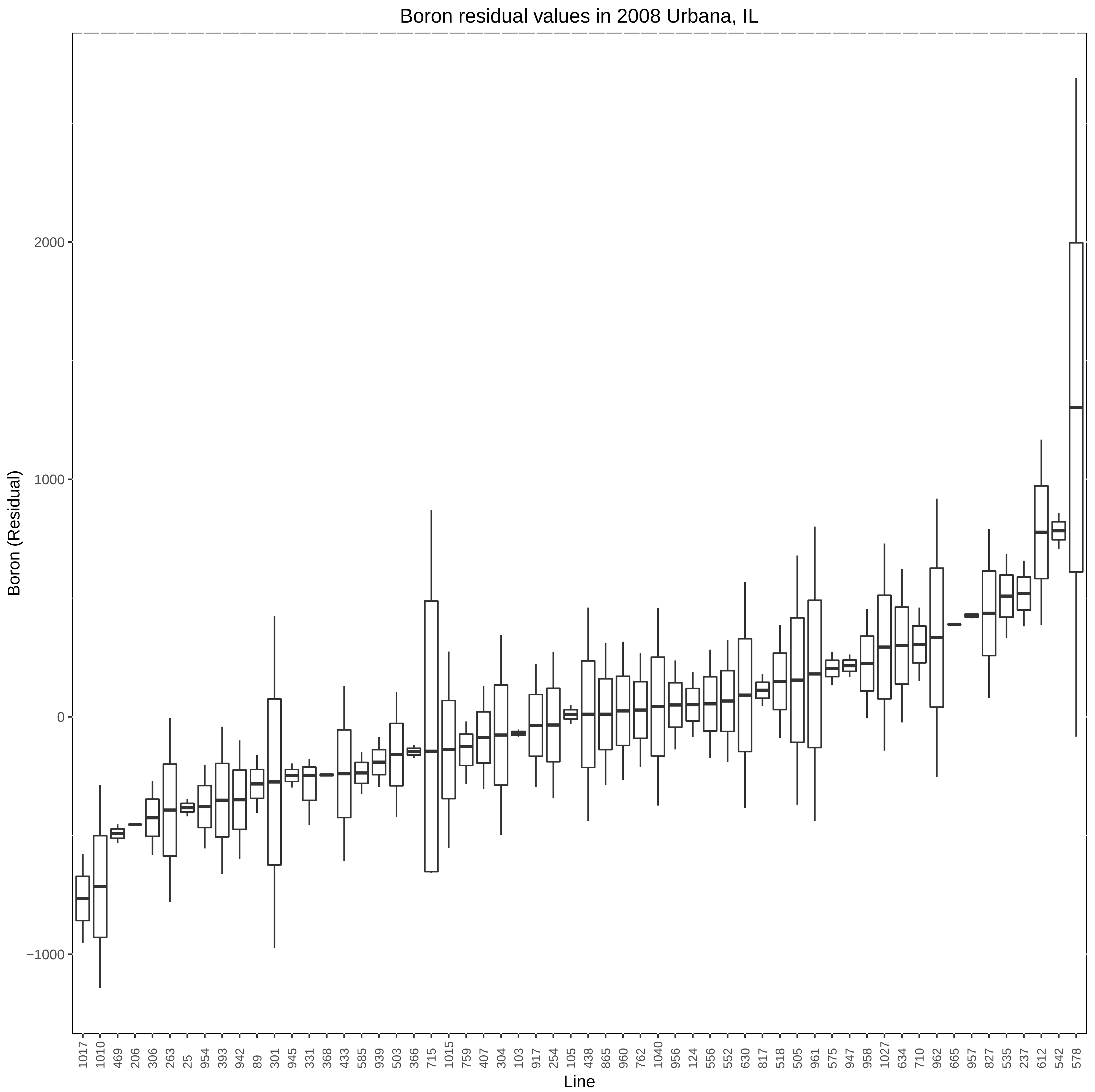
Boron residual values in 2008 Urbana, IL
Boron (Residual)
Line
−1000
0
1000
2000

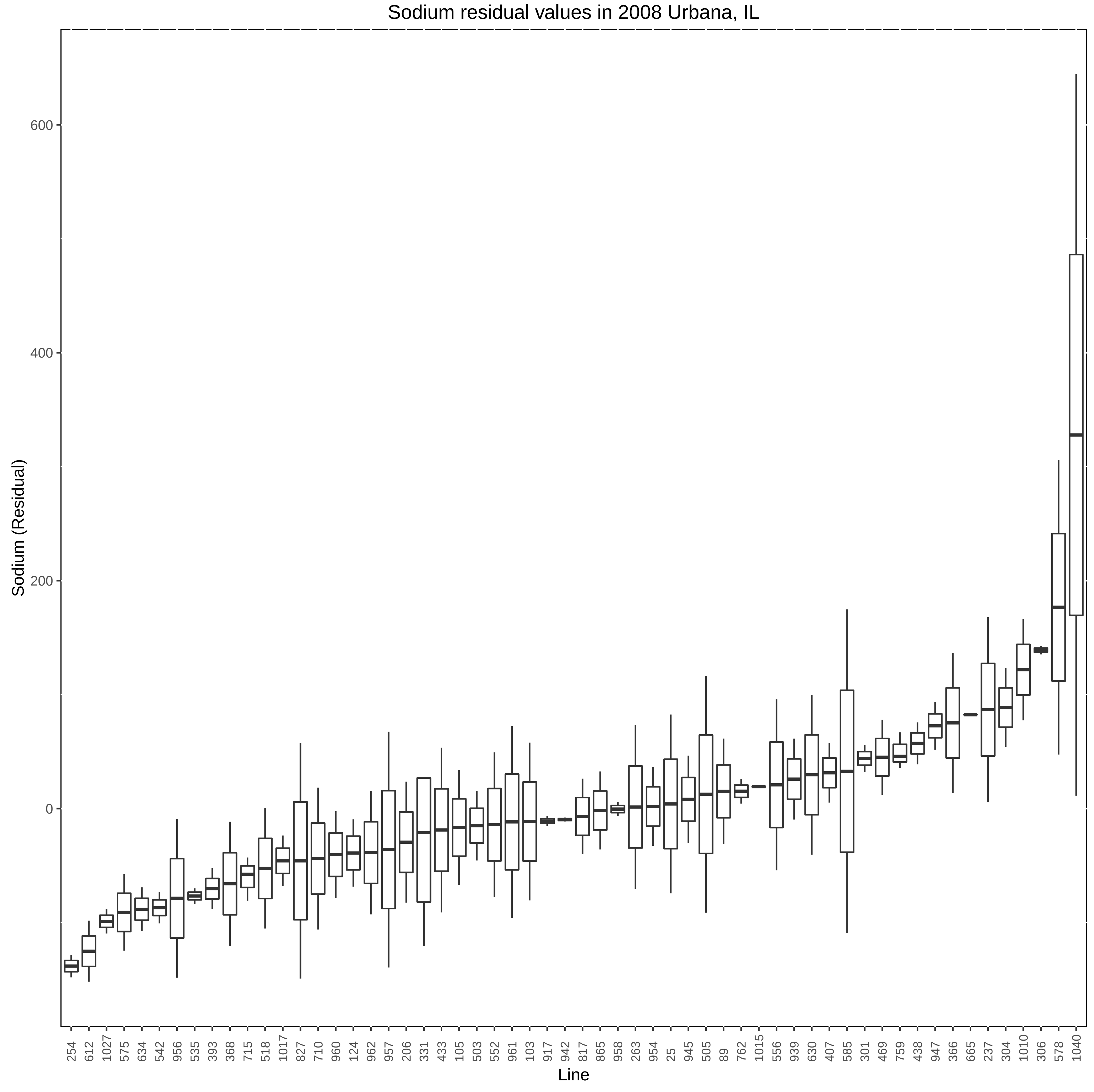
Sodium residual values in 2008 Urbana, IL
Sodium (Residual)
Line
0
200
400
600

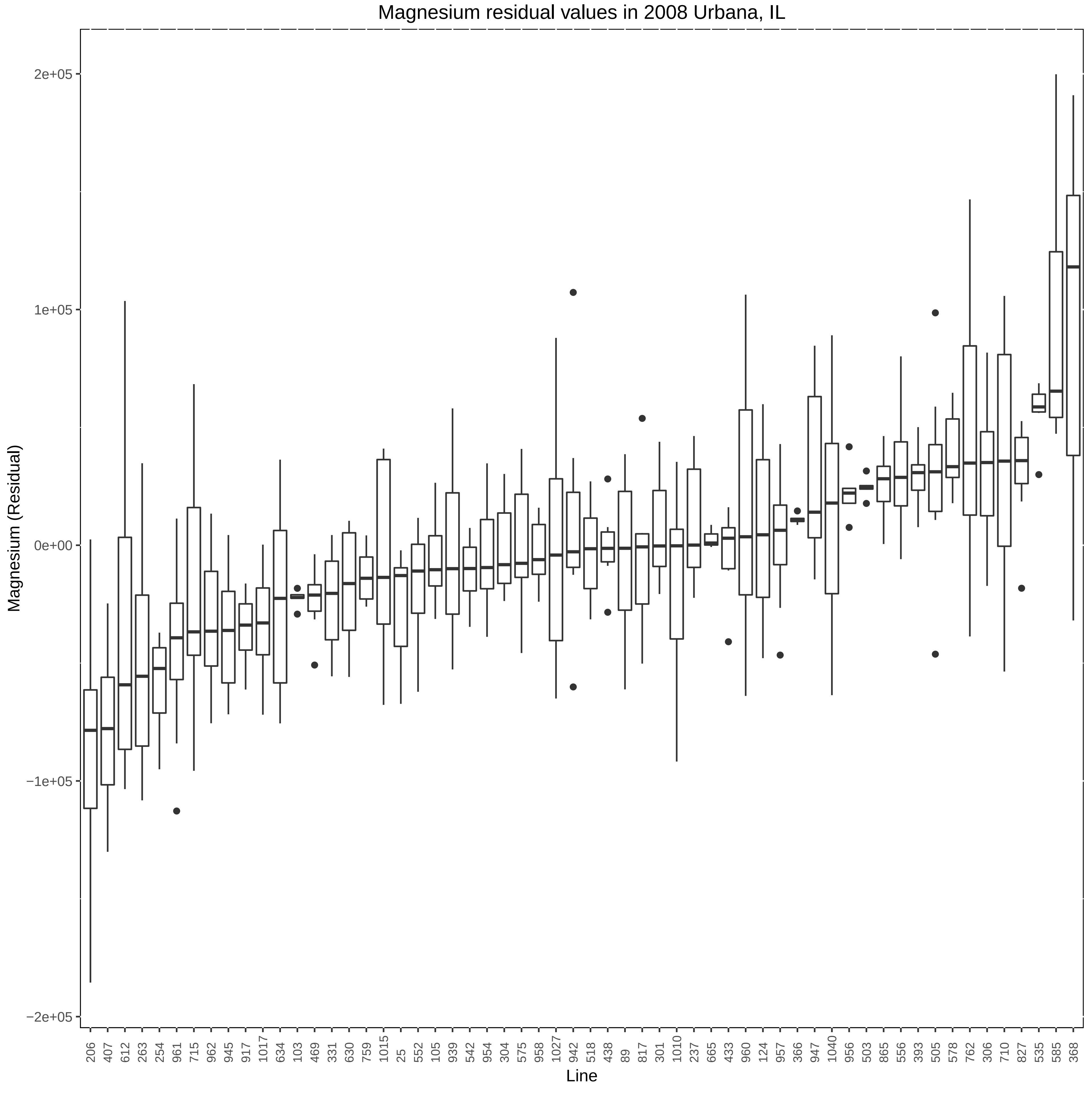
Magnesium residual values in 2008 Urbana, IL
Magnesium (Residual)
Line
−2e+05
−1e+05
0e+00
1e+05
2e+05

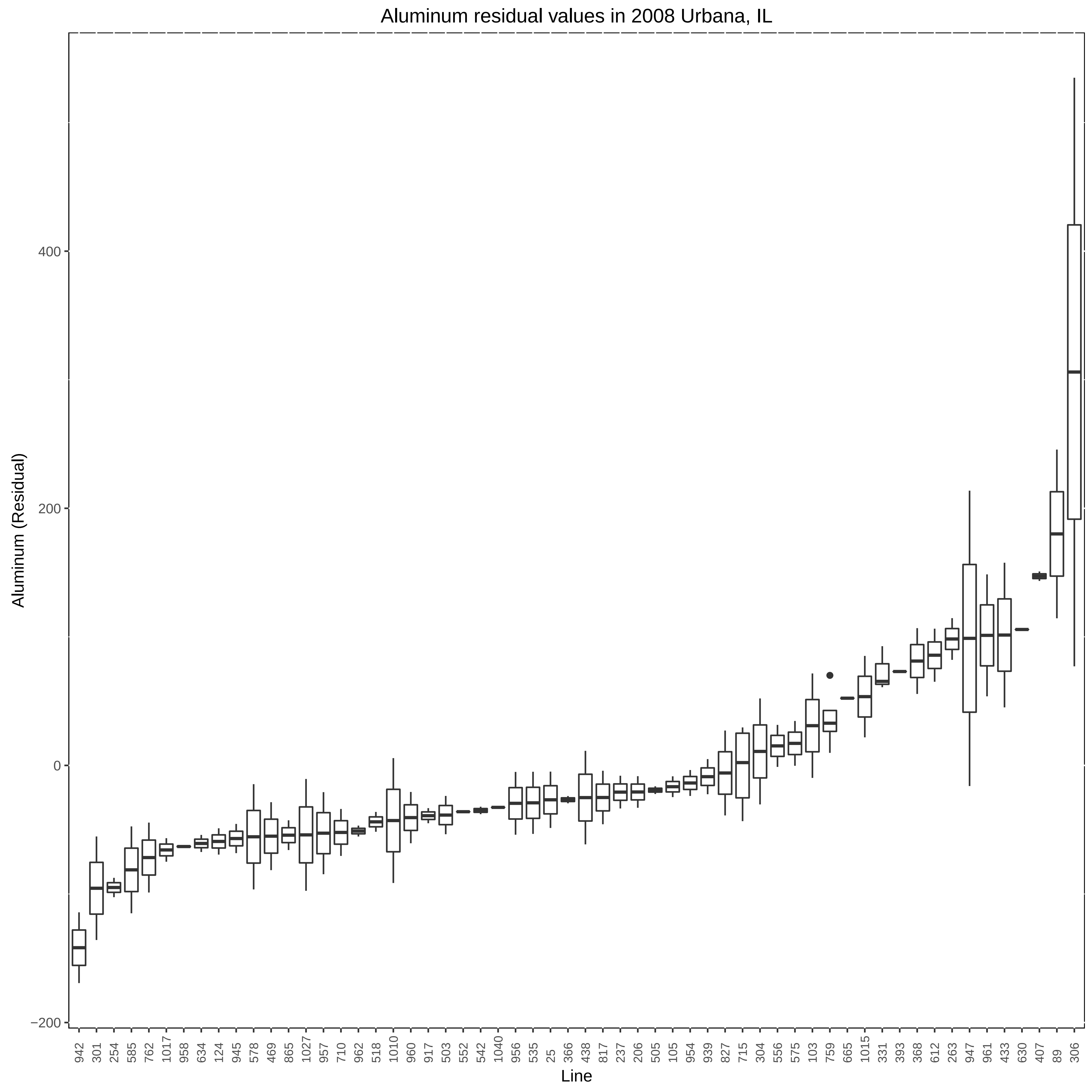
Aluminum residual values in 2008 Urbana, IL
Aluminum (Residual)
Line

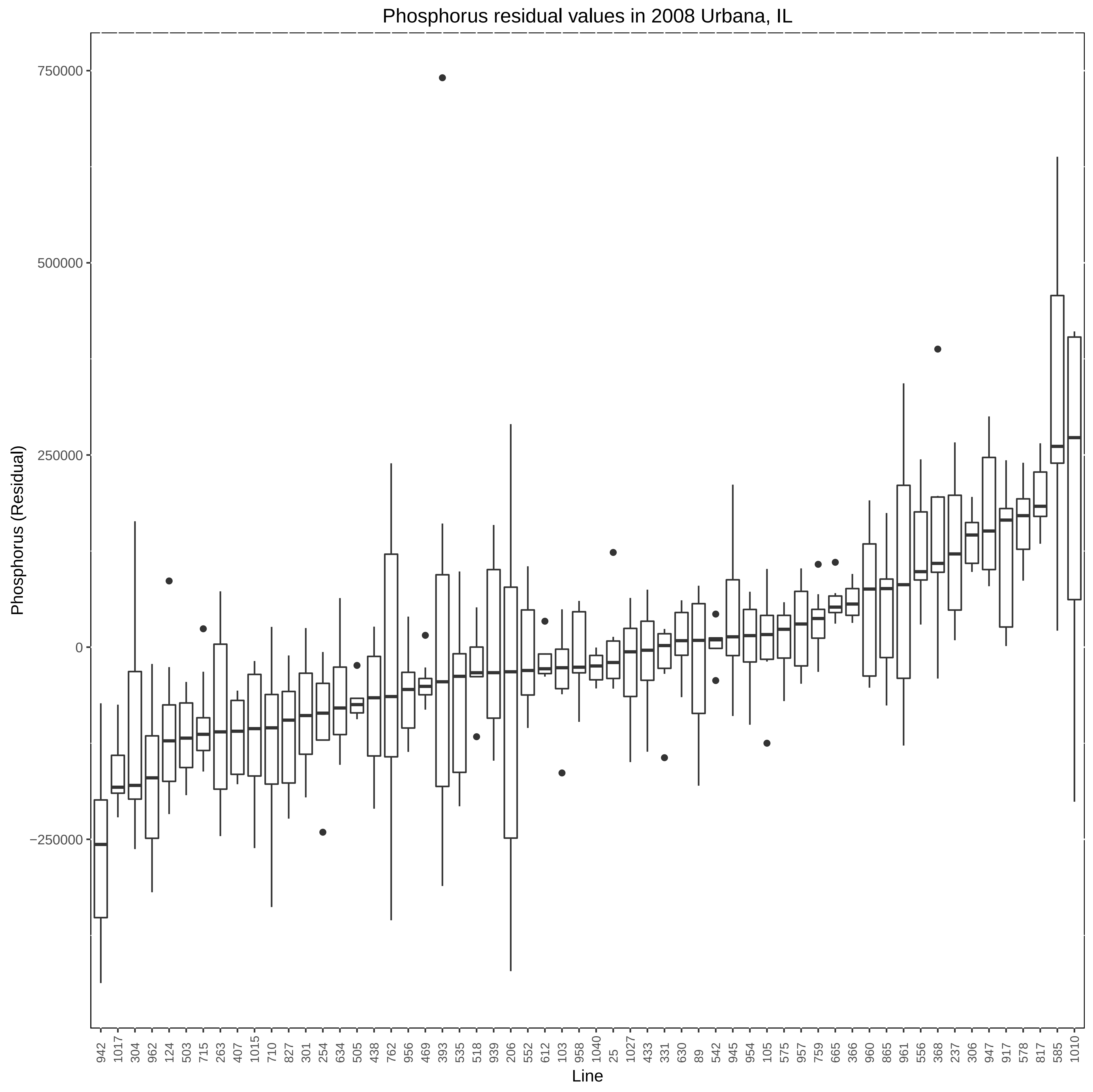
Phosphorus residual values in 2008 Urbana, IL
Phosphorus (Residual)
Line

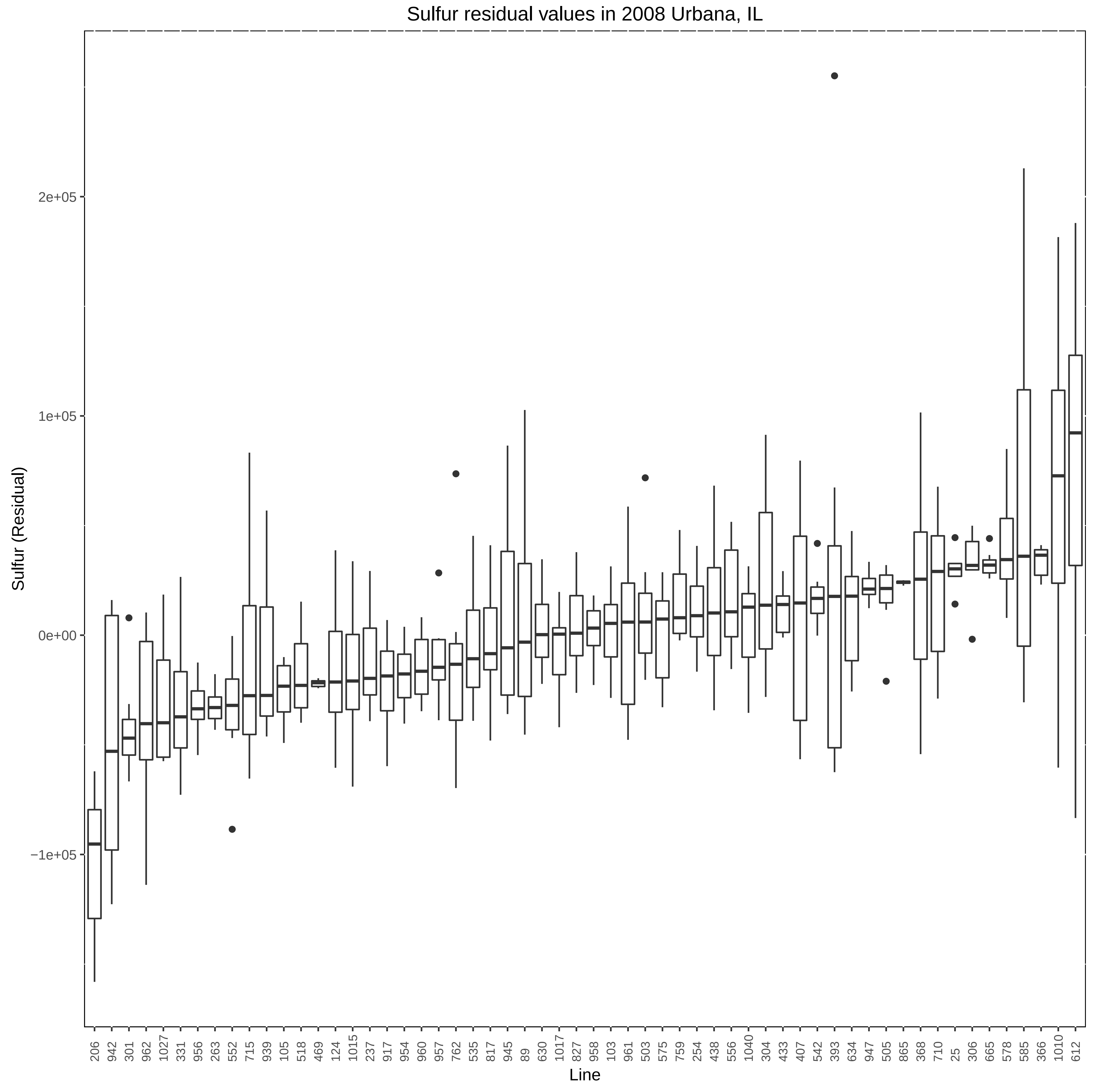
Sulfur residual values in 2008 Urbana, IL
Sulfur (Residual)
Line

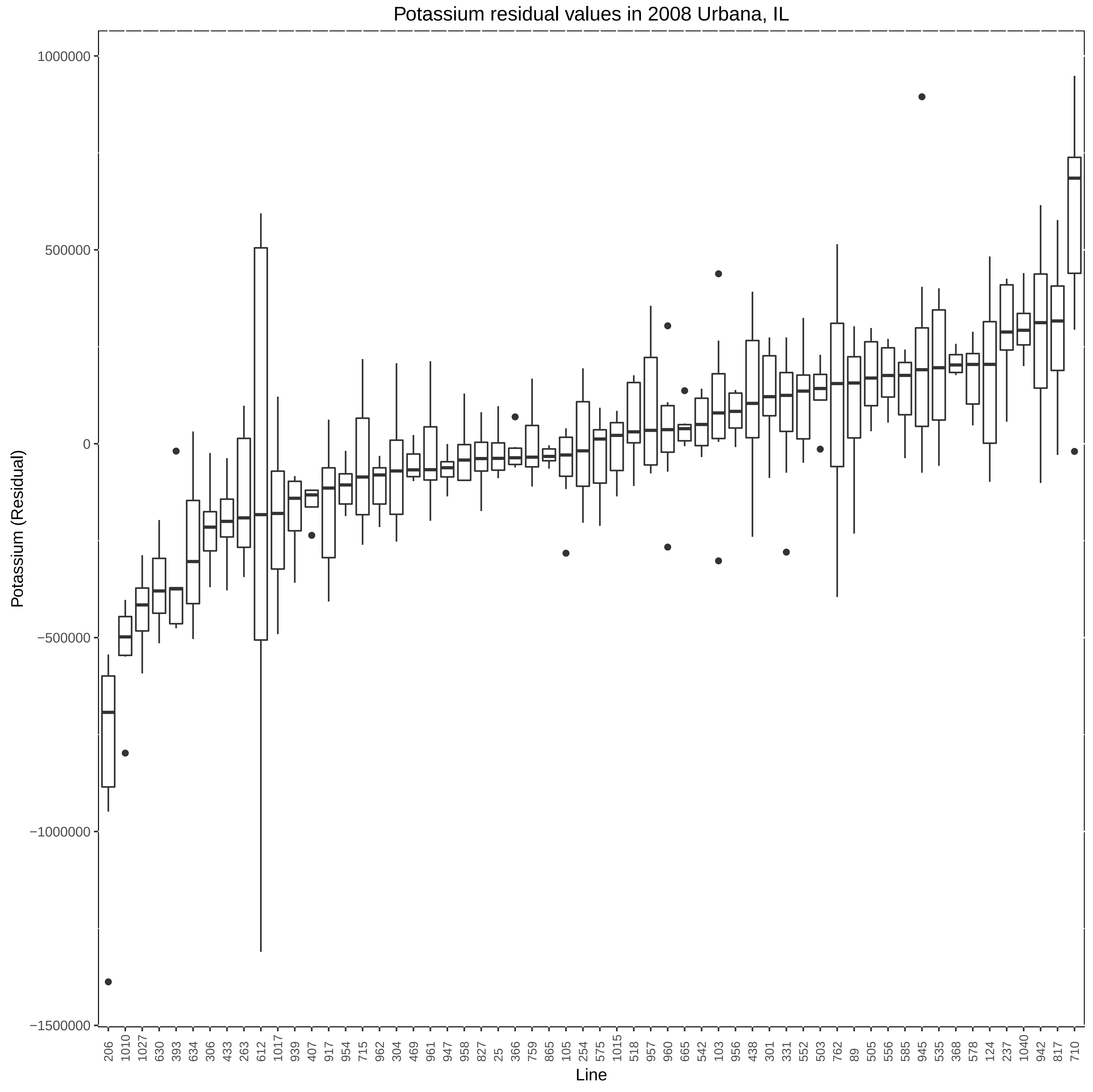
Potassium residual values in 2008 Urbana, IL
Potassium (Residual)
Line

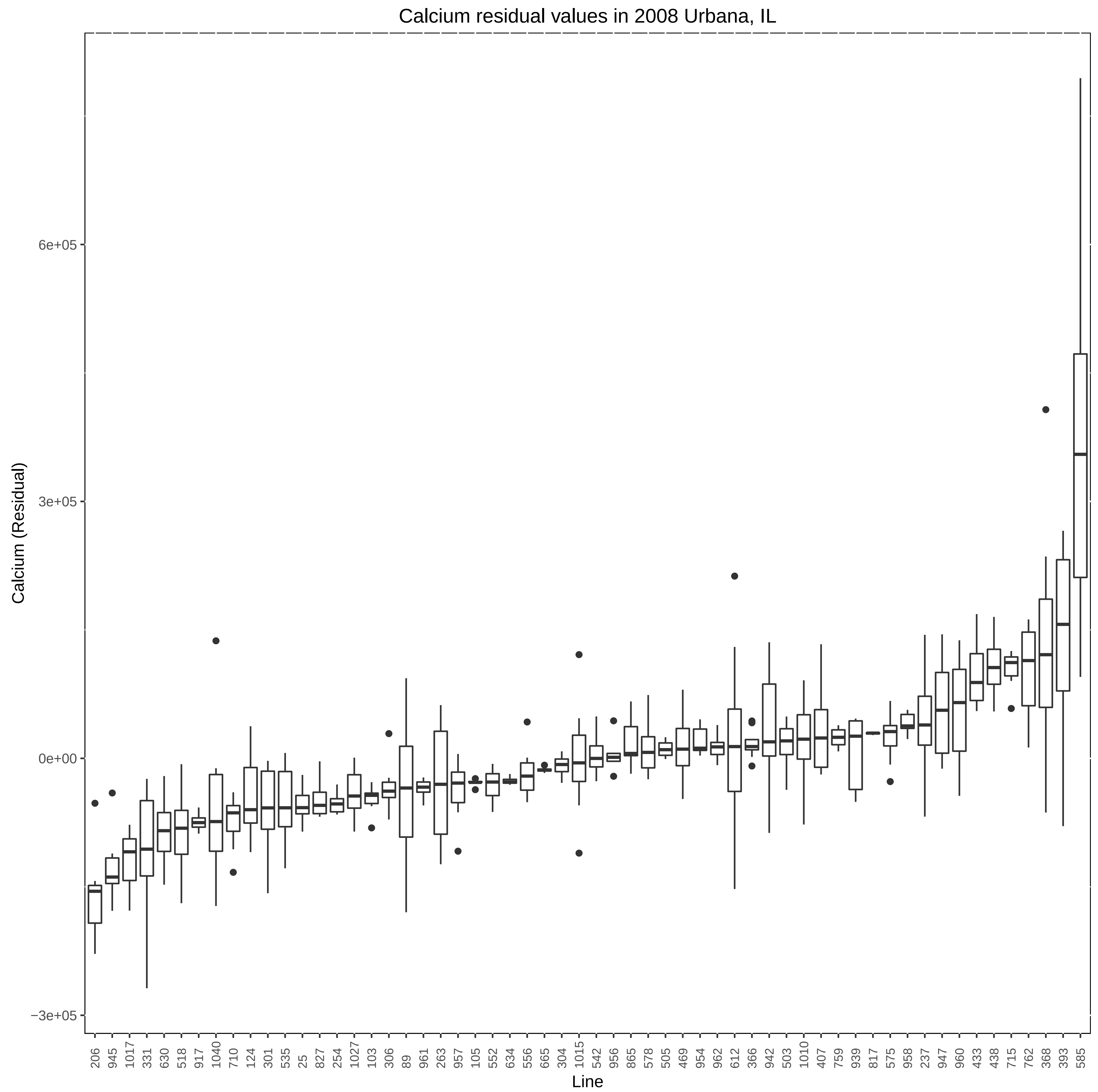
Calcium residual values in 2008 Urbana, IL
Calcium (Residual)
6e+05
3e+05
0e+00
-3e+05
Line
206 945 1017 331 630 518 917 1040 710 124 301 535 25 827 254 1027 103 306 89 961 263 957 105 552 634 556 665 304 1015 542 956 865 578 505 469 954 962 612 366 942 503 1010 407 759 939 817 575 958 237 947 960 433 438 715 762 368 393 585

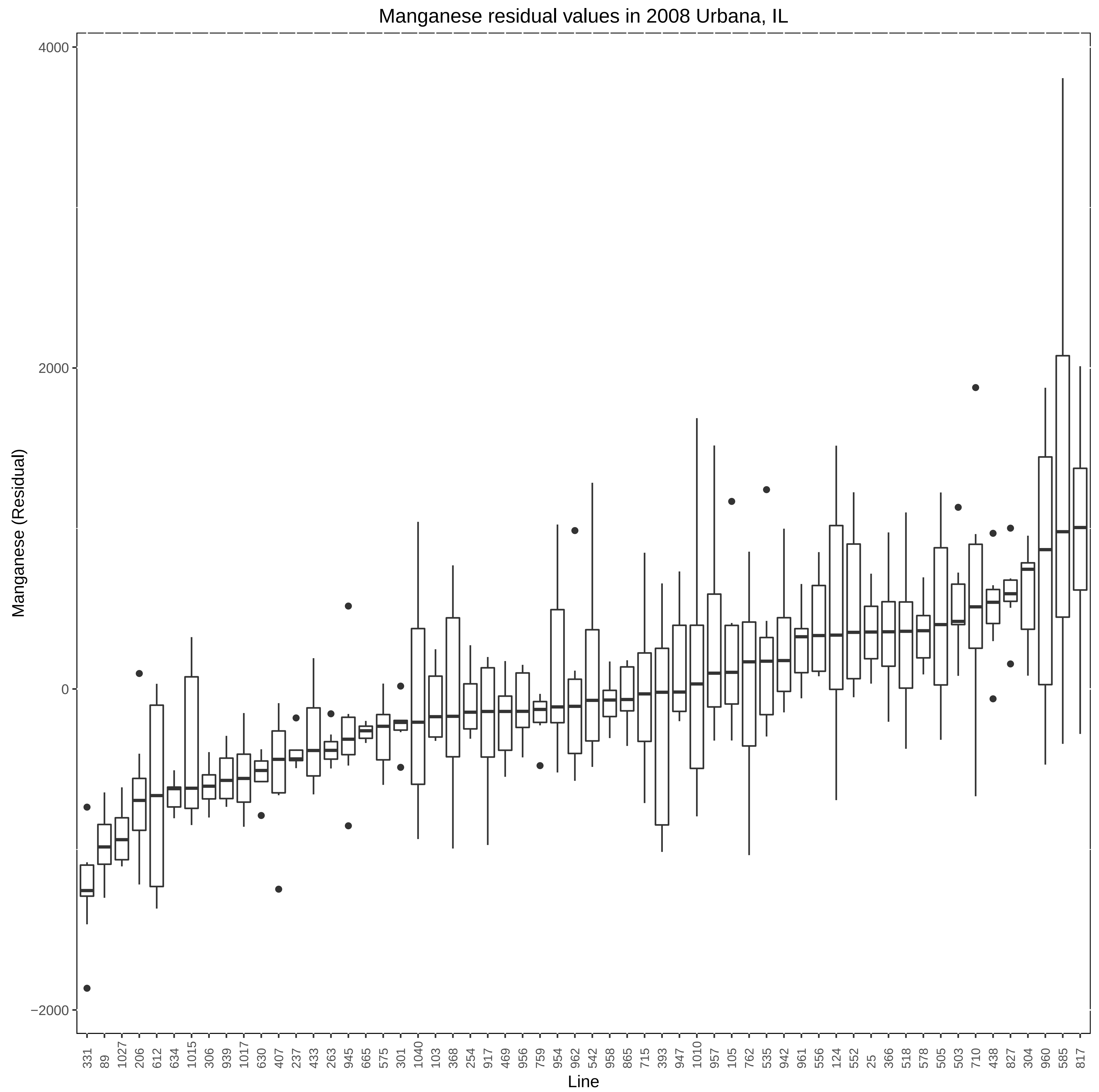
Manganese residual values in 2008 Urbana, IL
Manganese (Residual)
4000
2000
0
-2000
Line
331 89 1027 206 612 634 1015 306 939 1017 630 407 237 433 263 945 665 575 301 1040 103 368 254 917 469 956 759 954 962 542 958 865 715 393 947 1010 957 105 762 535 942 961 556 124 552 25 366 518 578 505 710 438 827 304 960 585 817

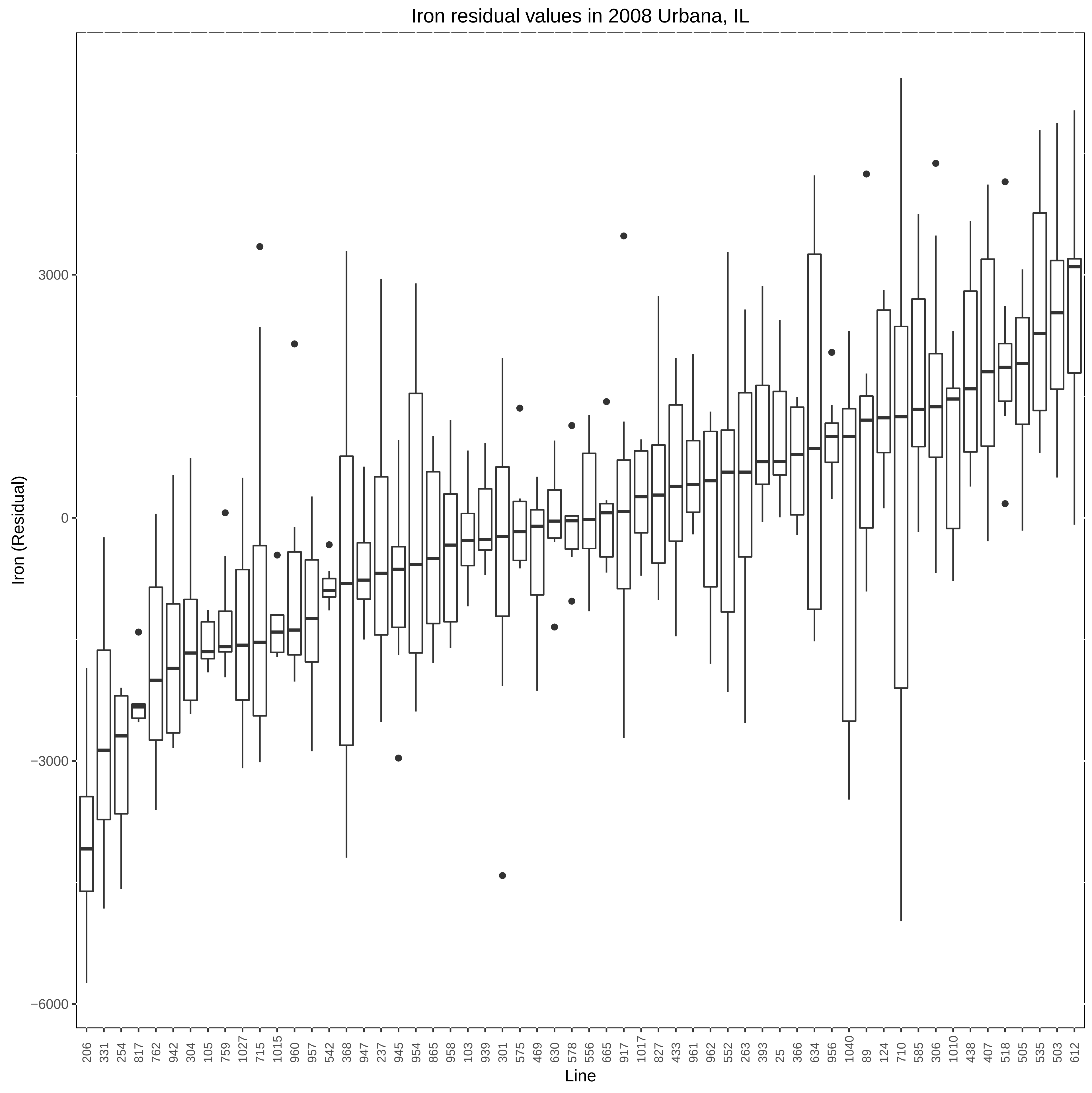
Iron residual values in 2008 Urbana, IL
Iron (Residual)
3000
0
-3000
-6000
Line
206 331 254 817 762 942 304 105 759 1027 715 1015 960 957 542 368 947 237 945 954 865 958 103 939 301 575 469 630 578 556 665 917 1017 827 433 961 962 552 263 393 25 366 634 956 1040 89 124 710 585 306 1010 438 947 518 505 535 503 612

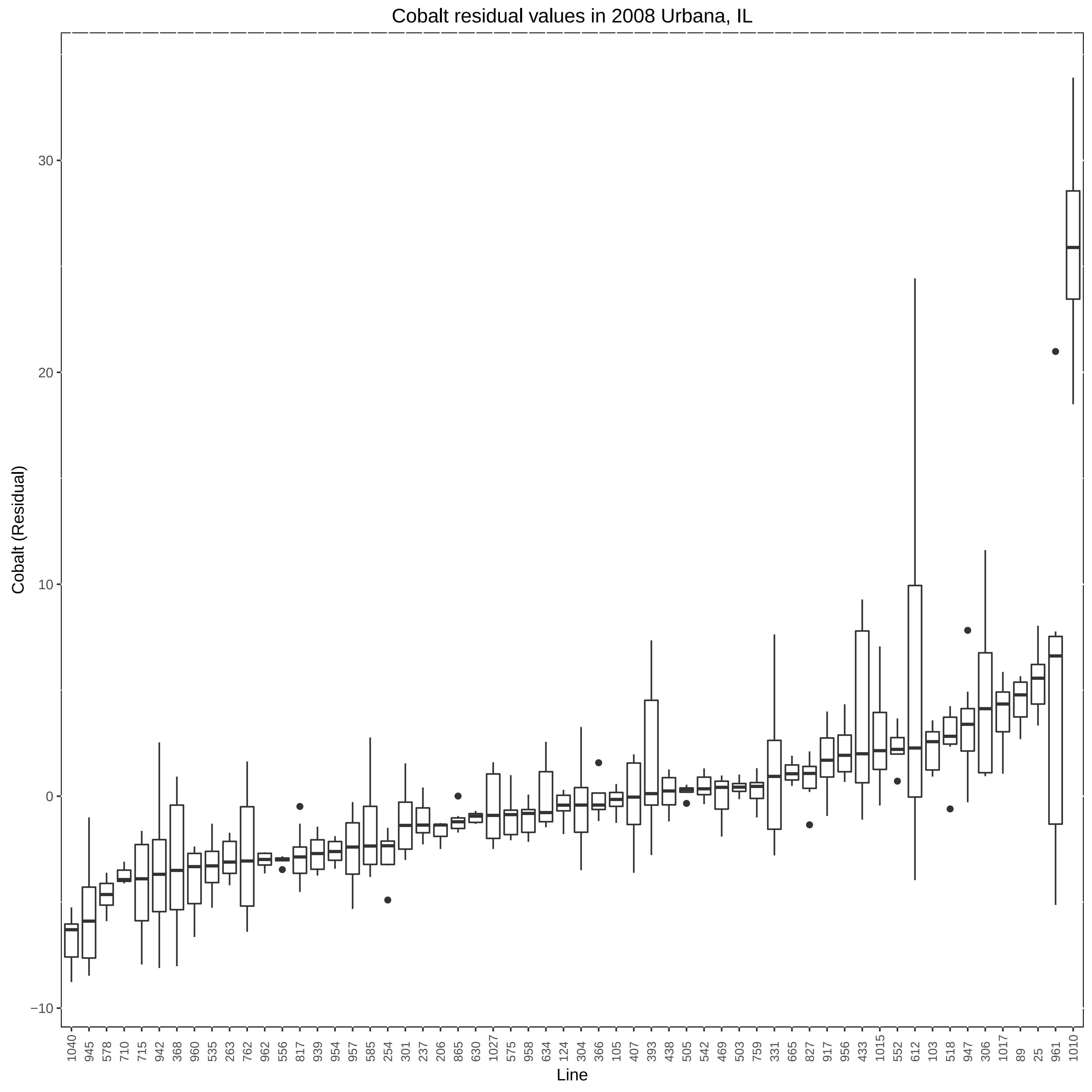
Cobalt residual values in 2008 Urbana, IL
Cobalt (Residual)
30
20
10
0
-10
Line
1040 945 578 710 715 942 368 960 535 263 762 556 817 939 954 957 585 254 301 237 206 865 630 1027 575 958 634 124 304 366 105 407 393 438 505 542 469 503 759 331 665 827 917 956 433 1015 552 612 103 518 947 306 1017 89 25 961 1010

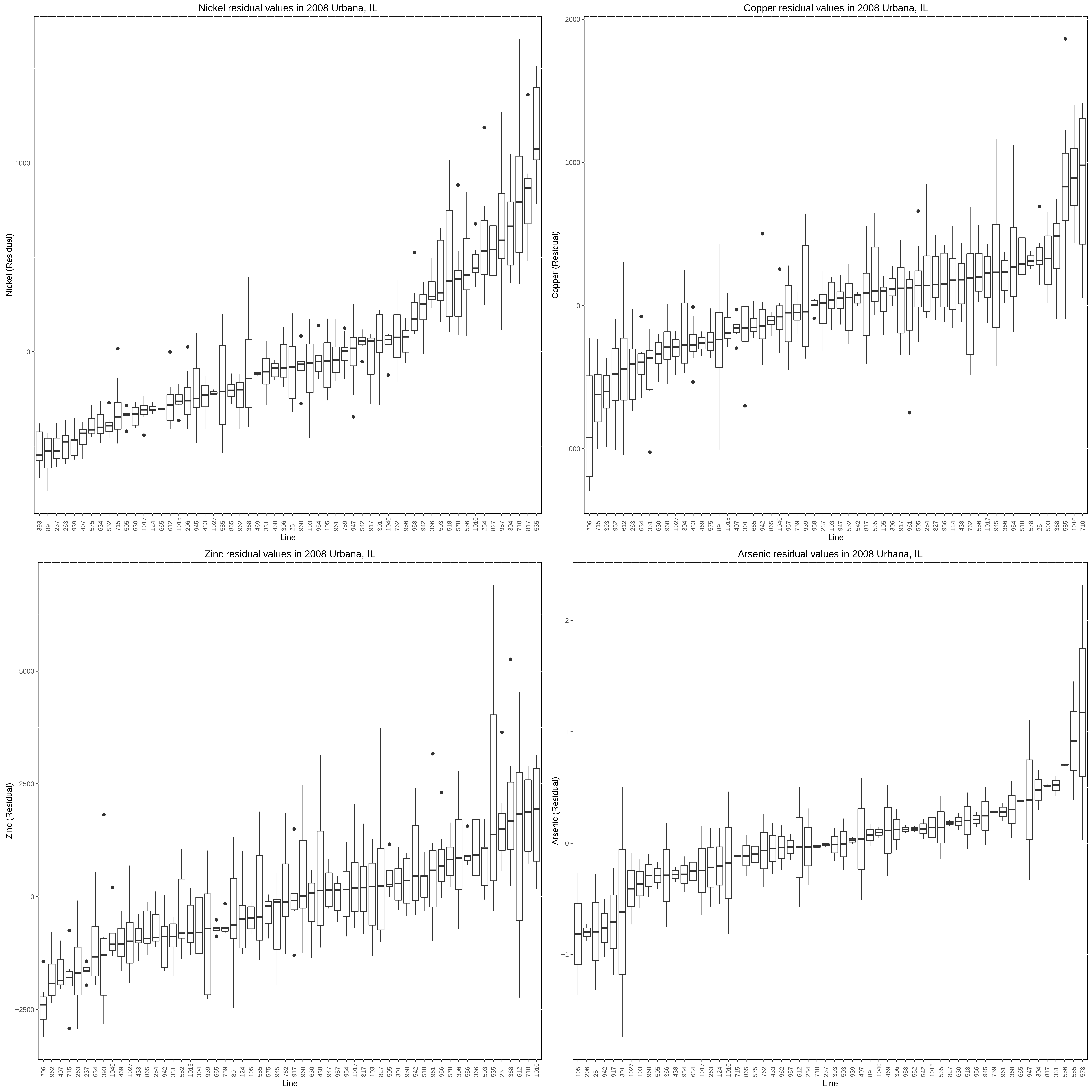
Nickel residual values in 2008 Urbana, IL
Nickel (Residual)
Line
Copper residual values in 2008 Urbana, IL
Copper (Residual)
Line
Zinc residual values in 2008 Urbana, IL
Zinc (Residual)
Line
Arsenic residual values in 2008 Urbana, IL
Arsenic (Residual)
Line

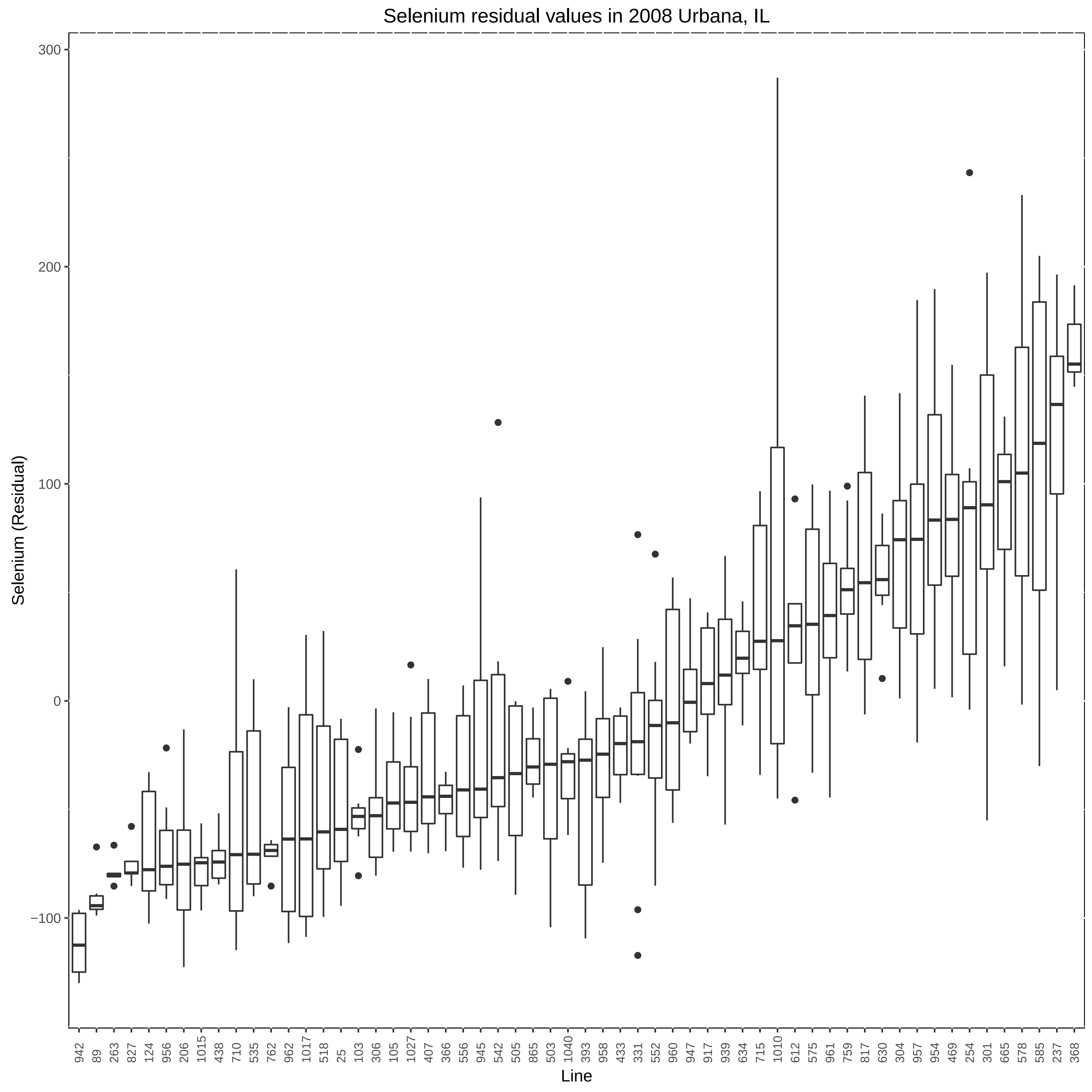
Selenium residual values in 2008 Urbana, IL
Selenium (Residual)
Line
300
200
100
0
−100

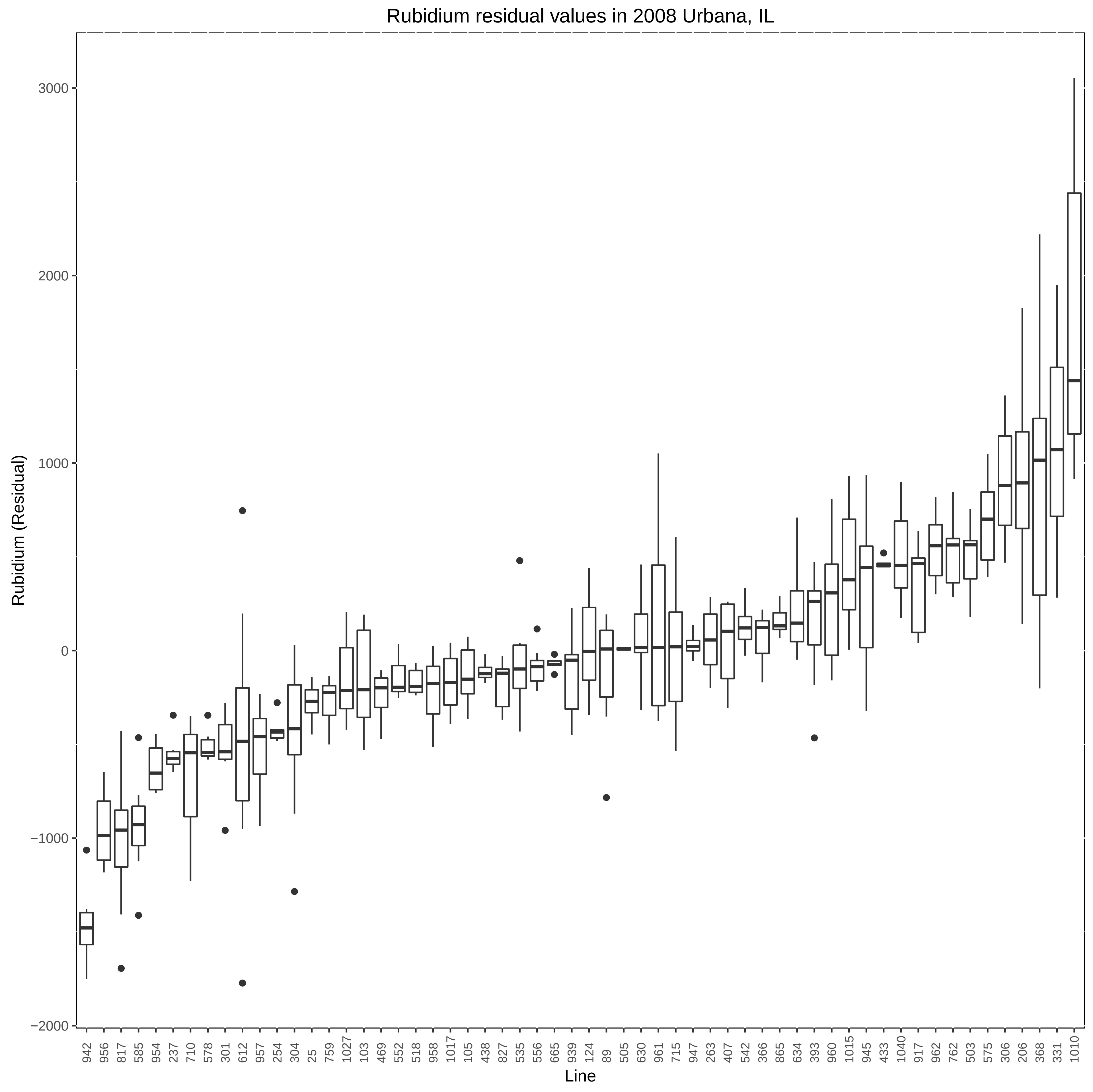
Rubidium residual values in 2008 Urbana, IL
Rubidium (Residual)
Line
3000
2000
1000
0
−1000
−2000

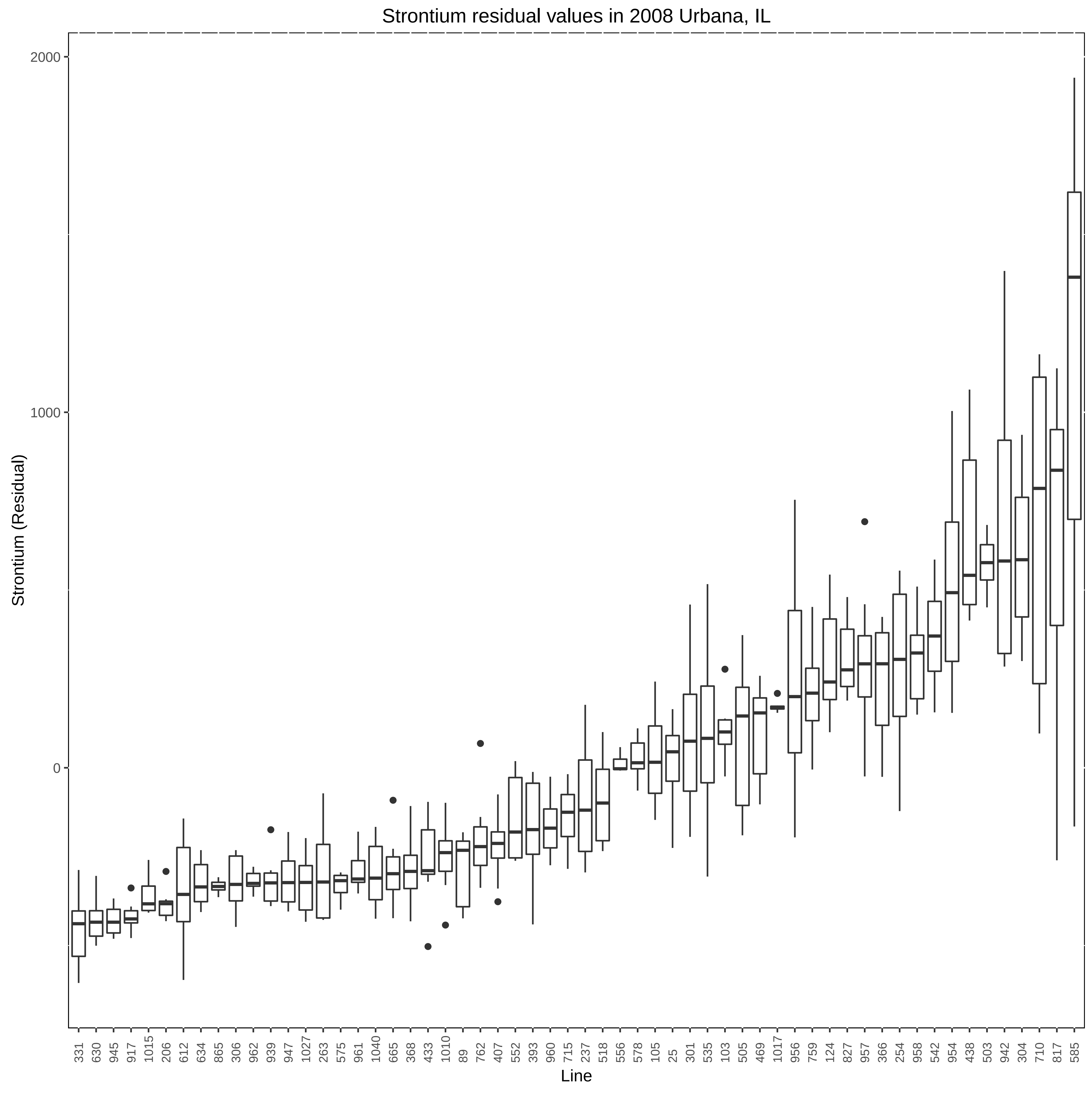
Strontium residual values in 2008 Urbana, IL
Strontium (Residual)
Line
2000
1000
0

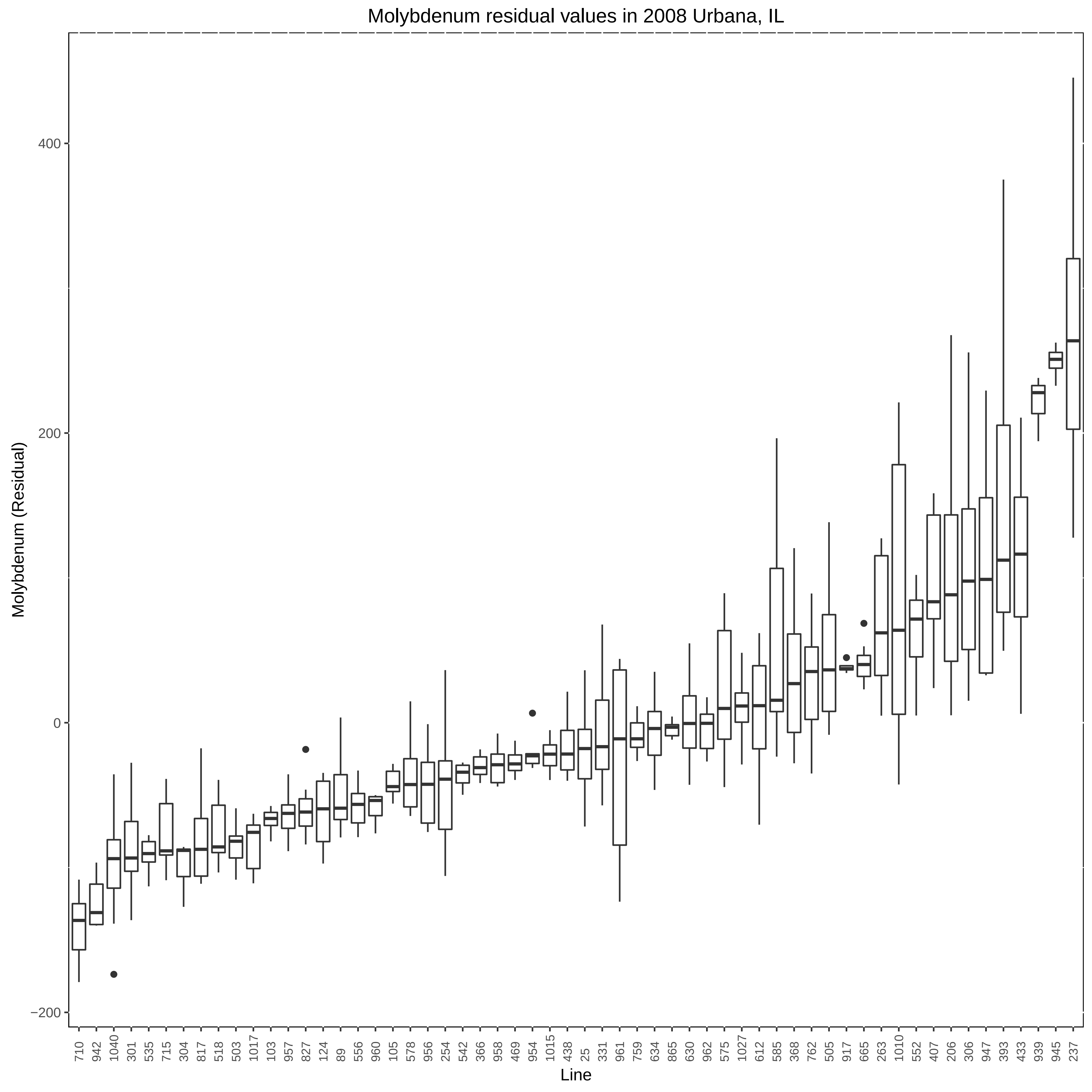
Molybdenum residual values in 2008 Urbana, IL
Molybdenum (Residual)
Line
400
200
0
−200

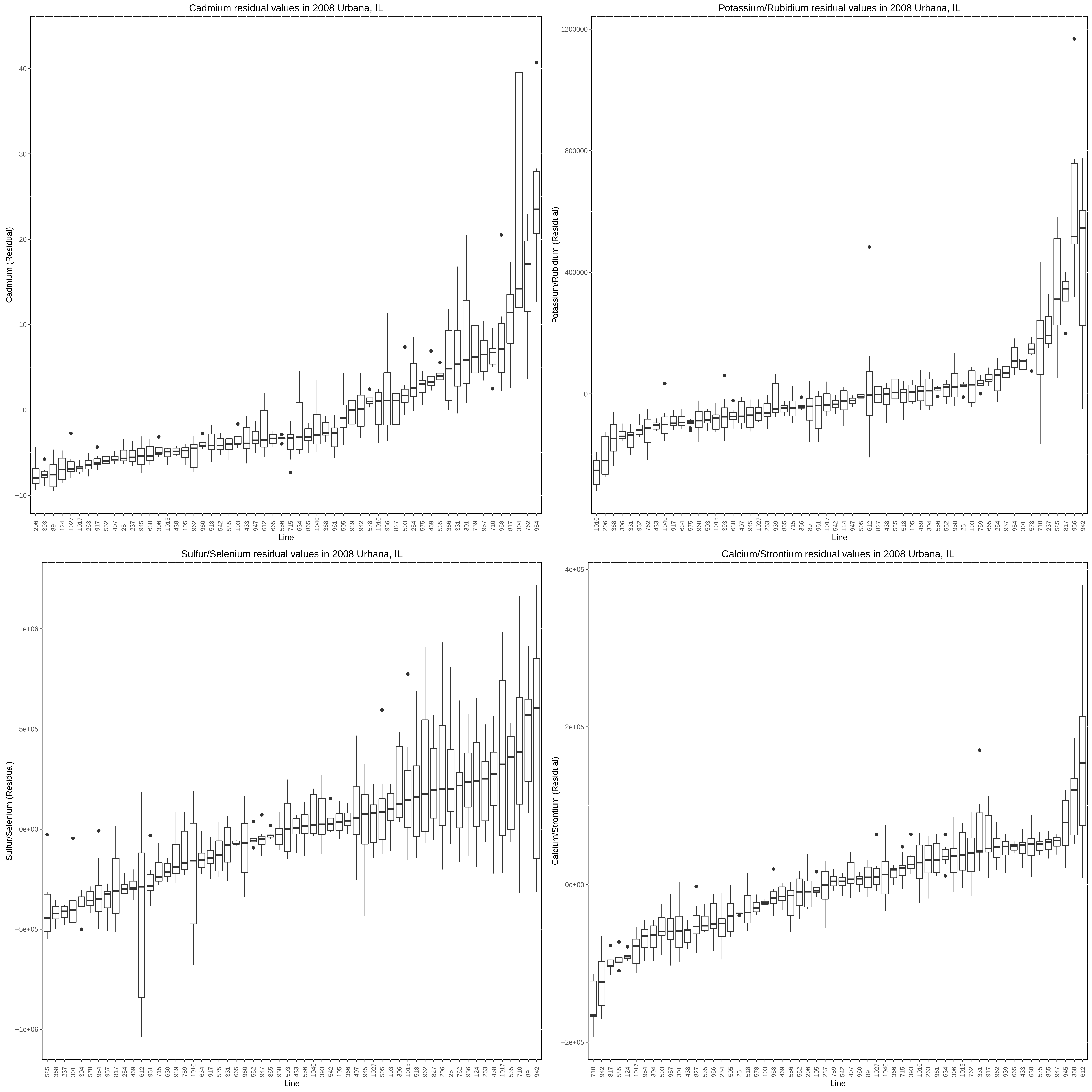

Cadmium residual values in 2008 Urbana, IL
Cadmium (Residual)
-10
0
10
20
30
40
Line
206 393 89 124 1027 1017 263 917 552 407 25 237 945 630 306 1015 438 105 962 960 518 542 585 103 433 947 612 665 556 715 634 865 1040 368 961 505 939 942 578 1010 956 827 503 254 575 469 535 366 331 301 759 957 710 958 817 304 762 954
Potassium/Rubidium residual values in 2008 Urbana, IL
Potassium/Rubidium (Residual)
0
400000
800000
1200000
Line
1010 206 368 306 331 962 762 433 1040 917 634 575 960 503 1015 393 630 407 945 1027 263 939 865 715 366 89 961 1017 542 124 947 505 612 827 438 535 518 105 469 304 556 552 958 25 103 759 665 254 957 954 301 578 710 237 585 817 956 942
Sulfur/Selenium residual values in 2008 Urbana, IL
Sulfur/Selenium (Residual)
-1e+06
-5e+05
0e+00
5e+05
1e+06
Line
585 368 237 301 304 578 954 957 817 254 469 612 961 715 630 939 759 1010 634 917 575 331 665 960 552 947 865 958 503 433 556 1040 393 542 105 366 407 945 1027 505 103 306 1015 518 962 827 206 25 762 956 124 263 438 1017 535 710 89 942
Calcium/Strontium residual values in 2008 Urbana, IL
Calcium/Strontium (Residual)
-2e+05
0e+00
2e+05
4e+05
Line
710 942 817 585 124 1017 954 304 503 957 301 438 827 535 956 254 505 25 518 578 103 958 469 556 552 206 105 237 759 542 407 960 89 1027 1040 366 715 393 1010 263 961 634 306 1015 762 331 917 962 939 665 433 630 575 865 947 945 368 612

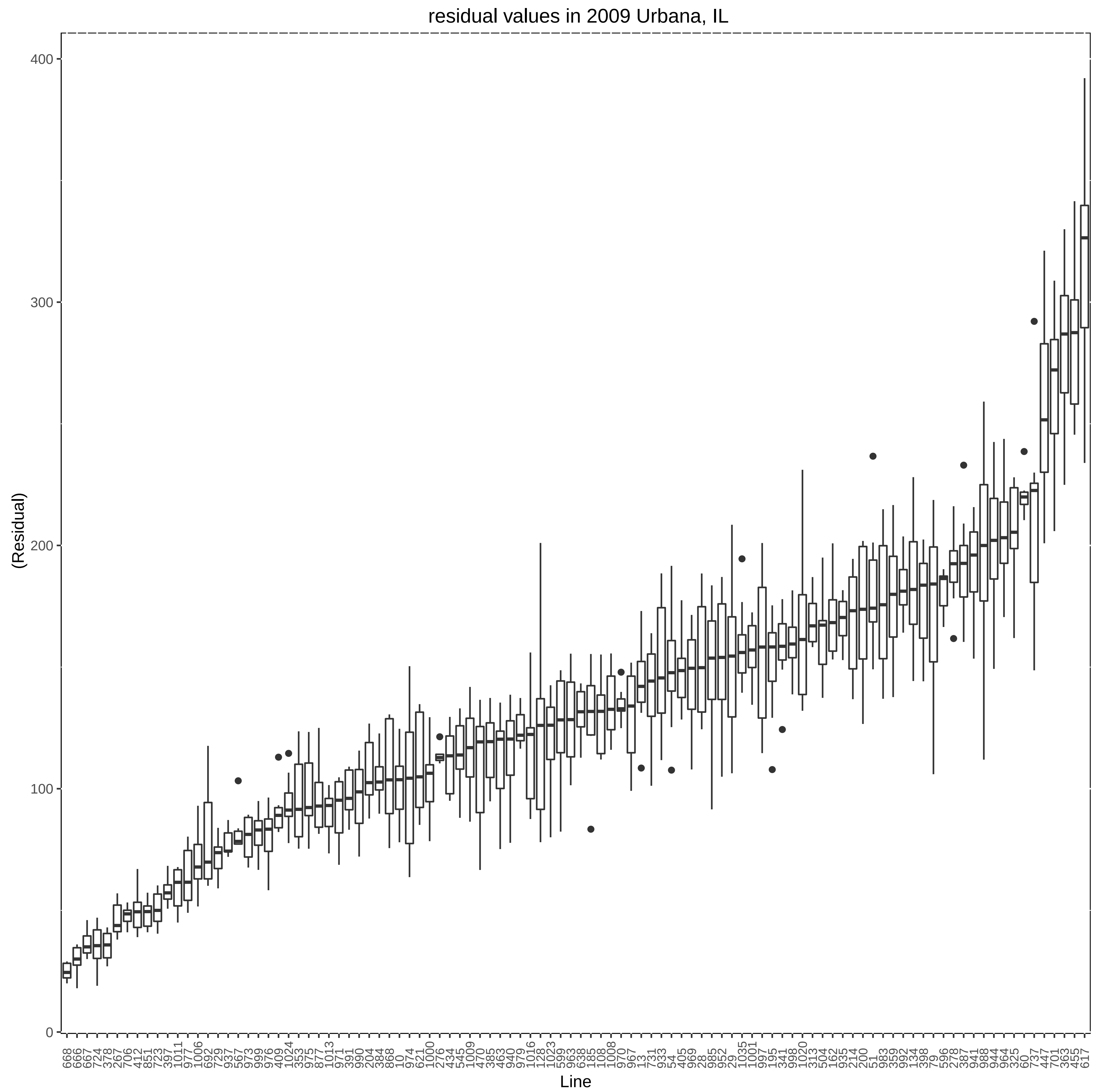
residual values in 2009 Urbana, IL
(Residual)
Line
0
100
200
300
400

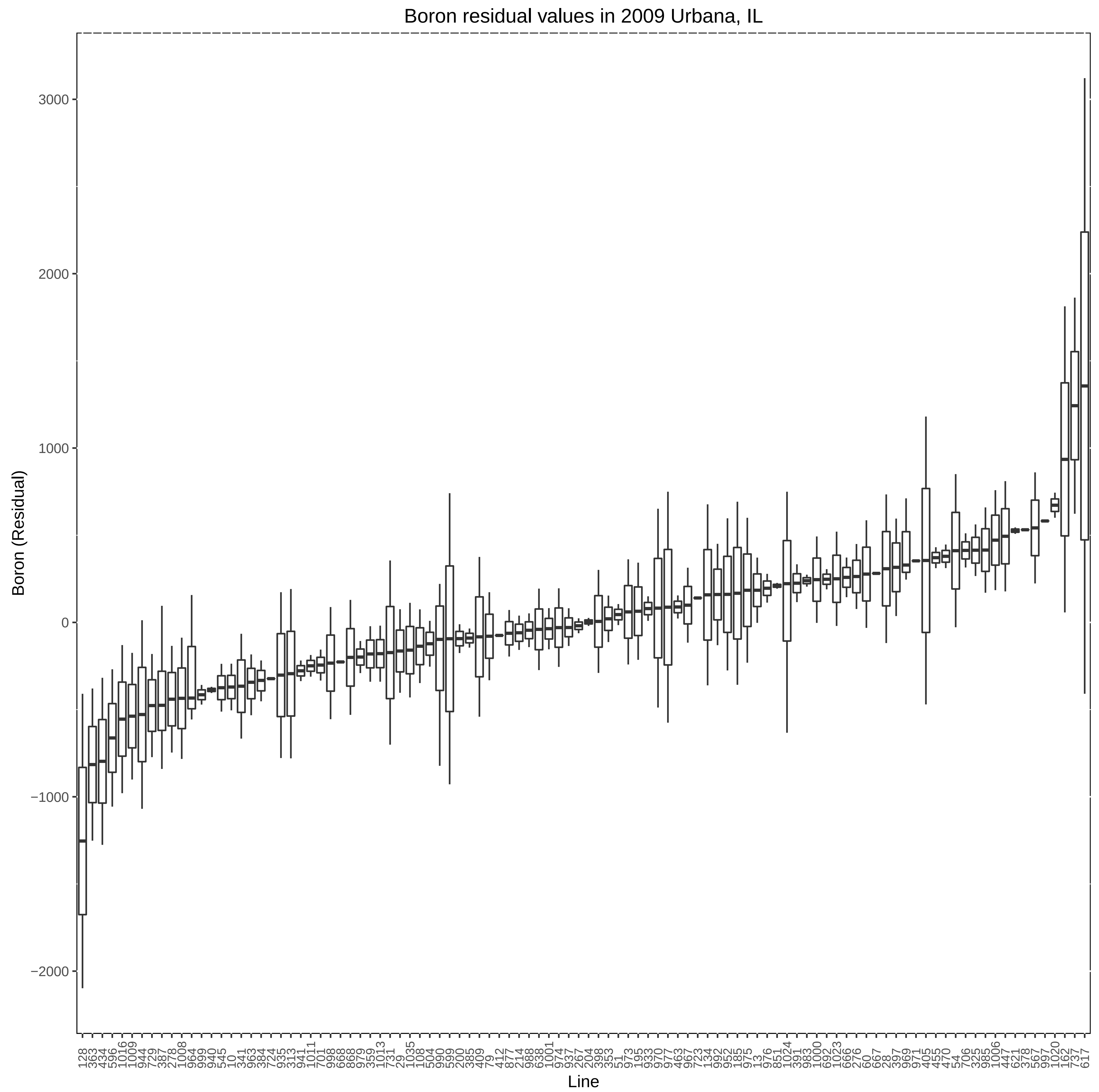
Boron residual values in 2009 Urbana, IL
Boron (Residual)
Line
−2000
−1000
0
1000
2000
3000

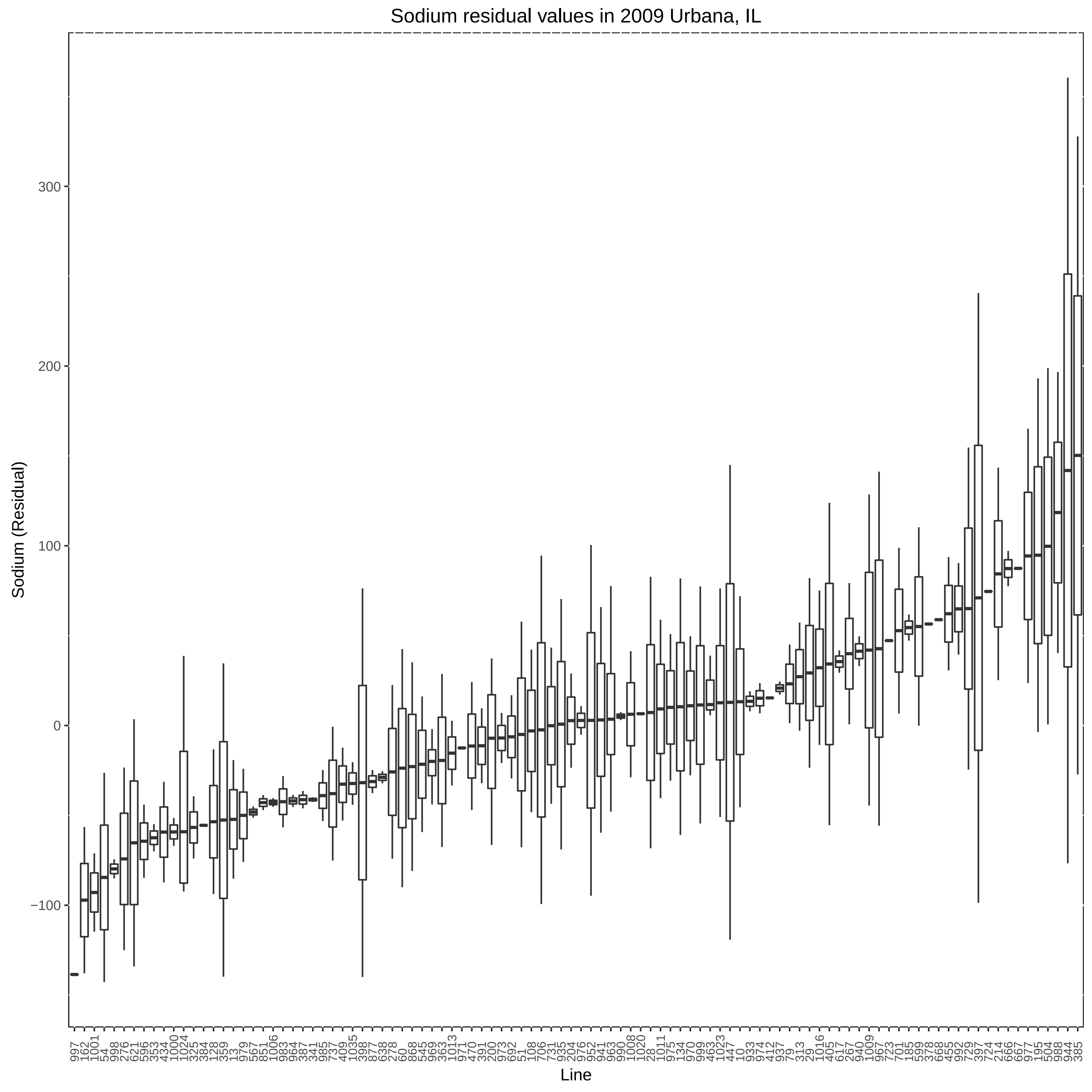
Sodium residual values in 2009 Urbana, IL
Sodium (Residual)
Line
−100
0
100
200
300

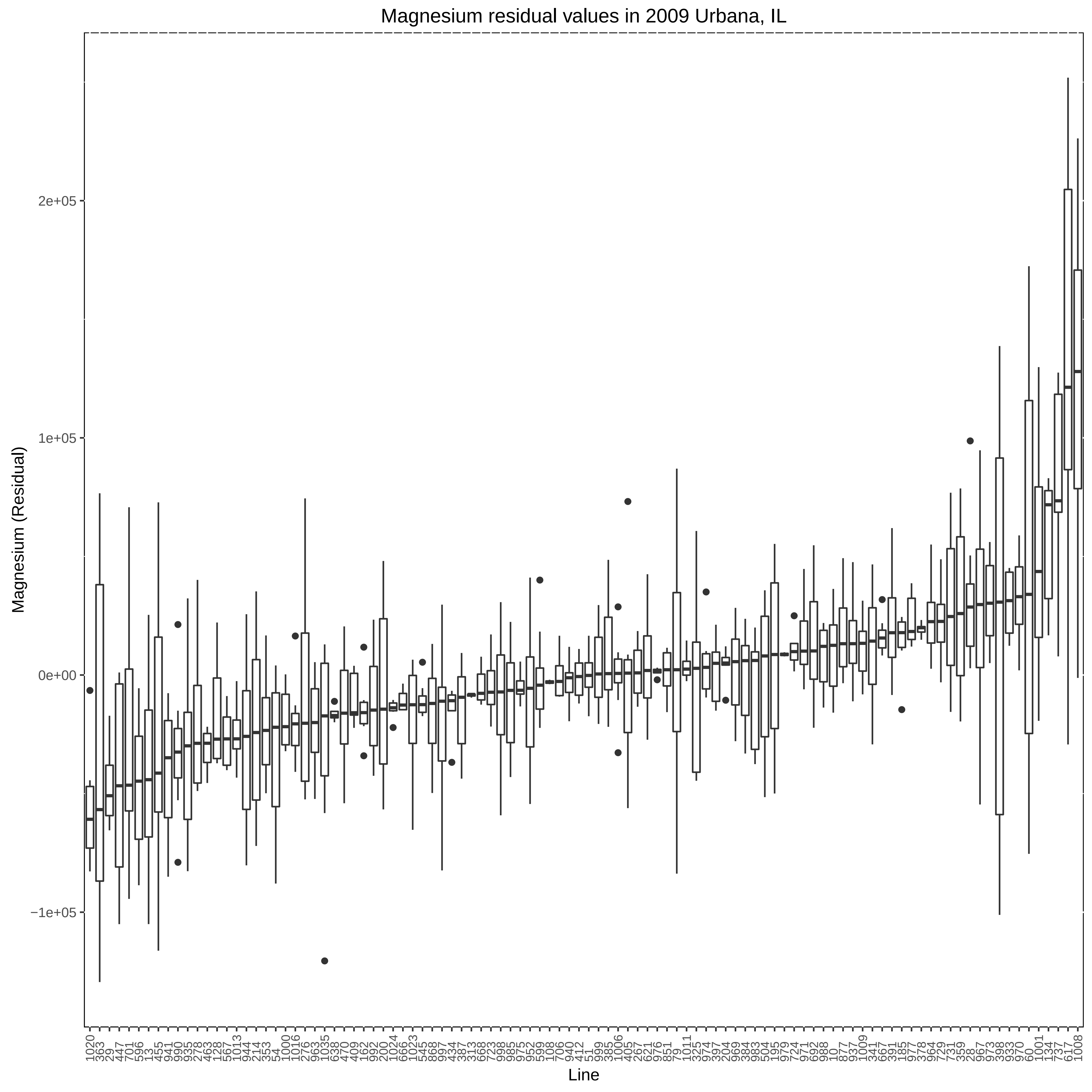
Magnesium residual values in 2009 Urbana, IL
Magnesium (Residual)
Line
−1e+05
0e+00
1e+05
2e+05

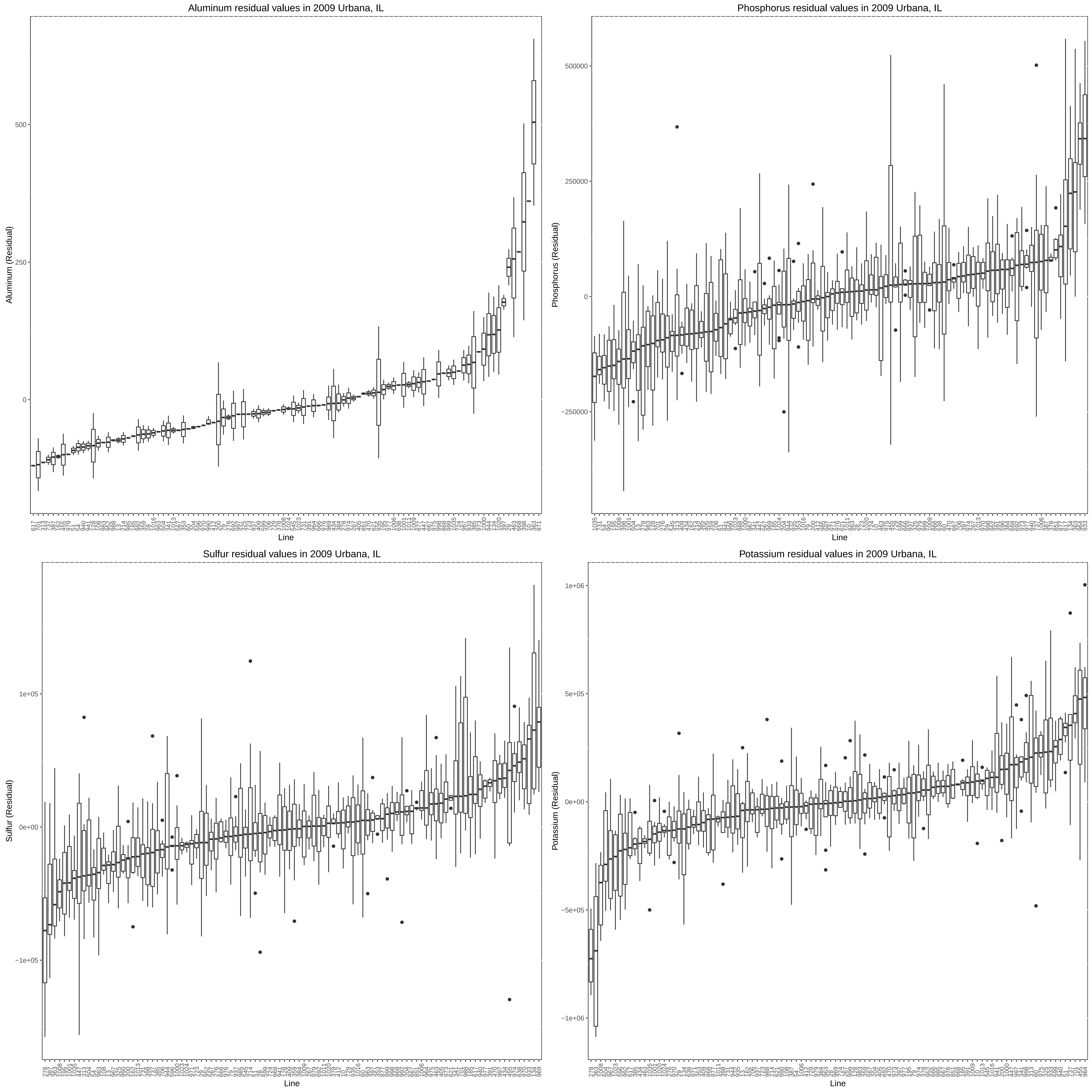
Aluminum residual values in 2009 Urbana, IL
Aluminum (Residual)
500
250
0
Line
Phosphorus residual values in 2009 Urbana, IL
Phosphorus (Residual)
500000
250000
0
−250000
Line
Sulfur residual values in 2009 Urbana, IL
Sulfur (Residual)
1e+05
0e+00
−1e+05
Line
Potassium residual values in 2009 Urbana, IL
Potassium (Residual)
1e+06
5e+05
0e+00
−5e+05
−1e+06
Line

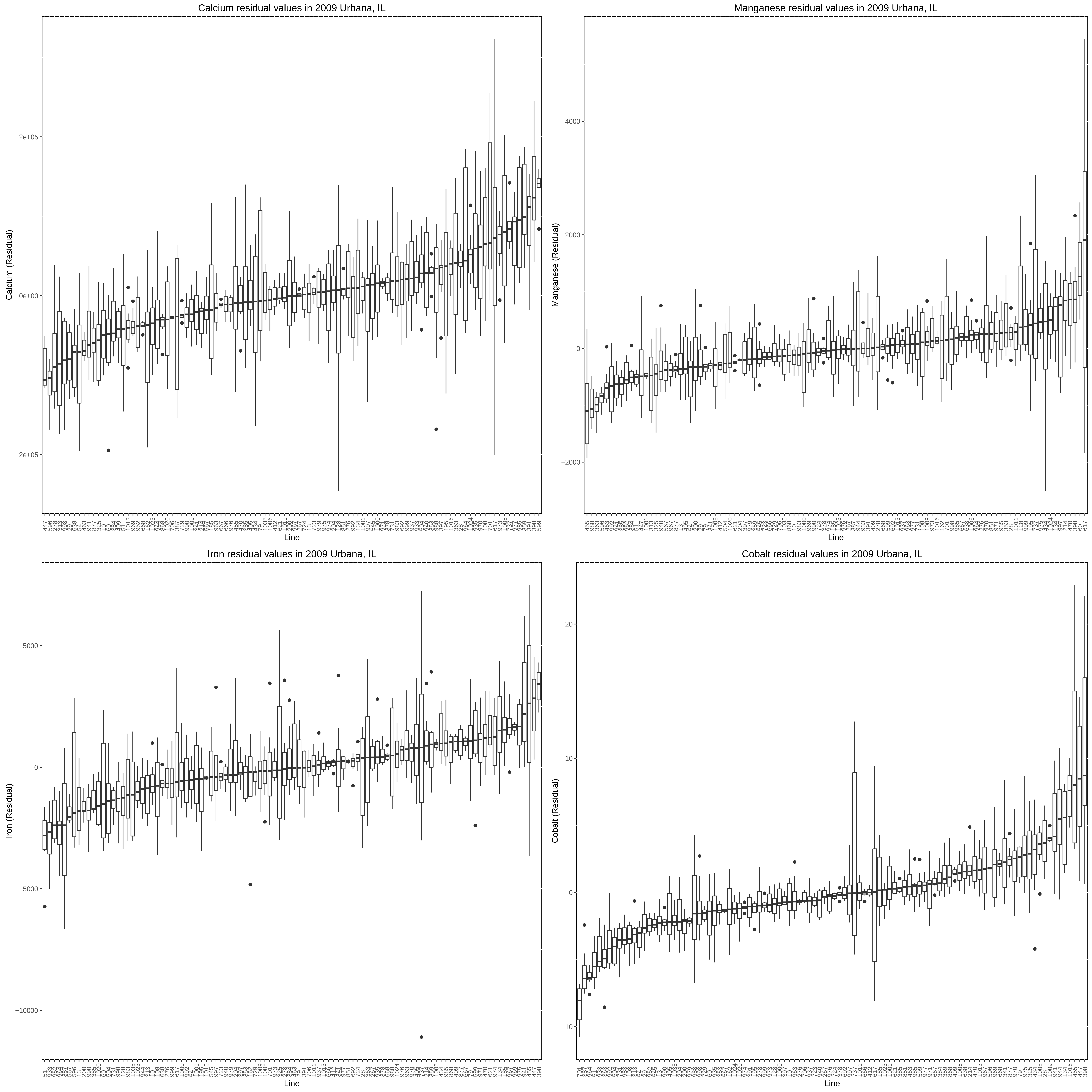

Calcium residual values in 2009 Urbana, IL
Calcium (Residual)
2e+05
0e+00
-2e+05
Line
Manganese residual values in 2009 Urbana, IL
Manganese (Residual)
4000
2000
0
-2000
Line
Iron residual values in 2009 Urbana, IL
Iron (Residual)
5000
0
-5000
-10000
Line
Cobalt residual values in 2009 Urbana, IL
Cobalt (Residual)
20
10
0
-10
Line

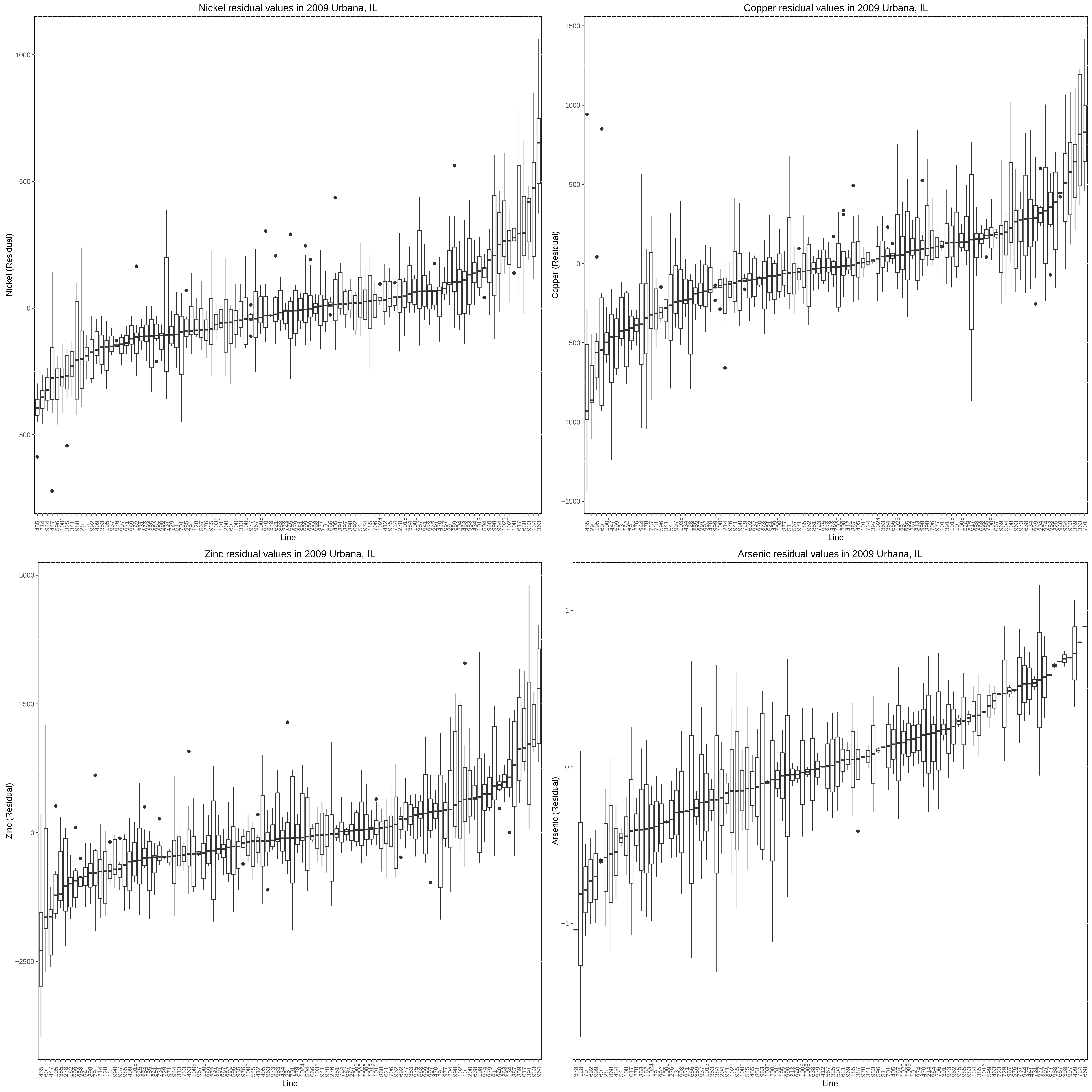

Nickel residual values in 2009 Urbana, IL
Nickel (Residual)
1000
500
0
-500
Line
Copper residual values in 2009 Urbana, IL
Copper (Residual)
1500
1000
500
0
-500
-1000
-1500
Line
Zinc residual values in 2009 Urbana, IL
Zinc (Residual)
5000
2500
0
-2500
Line
Arsenic residual values in 2009 Urbana, IL
Arsenic (Residual)
1
0
-1
Line

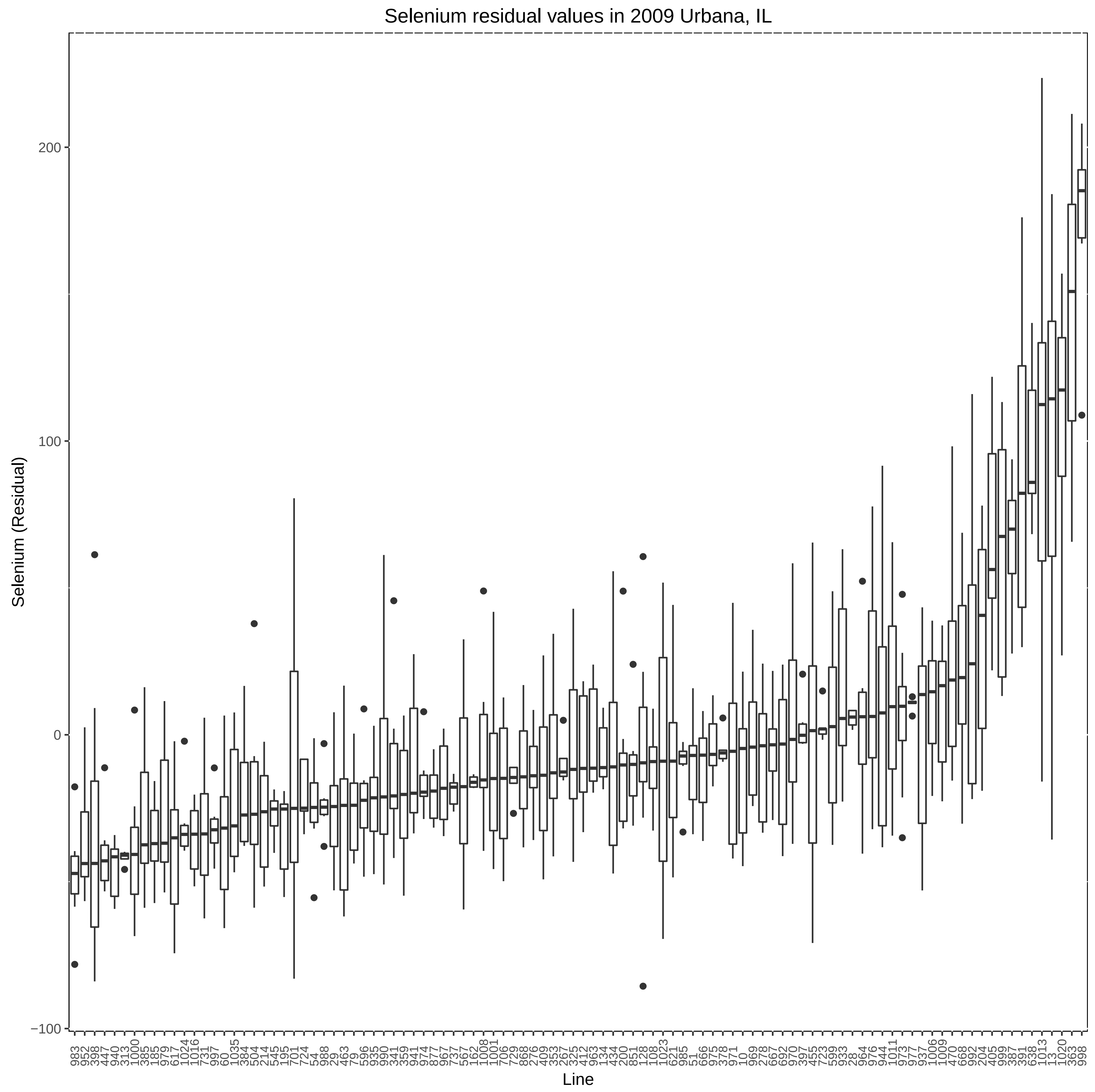
Selenium residual values in 2009 Urbana, IL
Selenium (Residual)
200
100
0
−100
Line

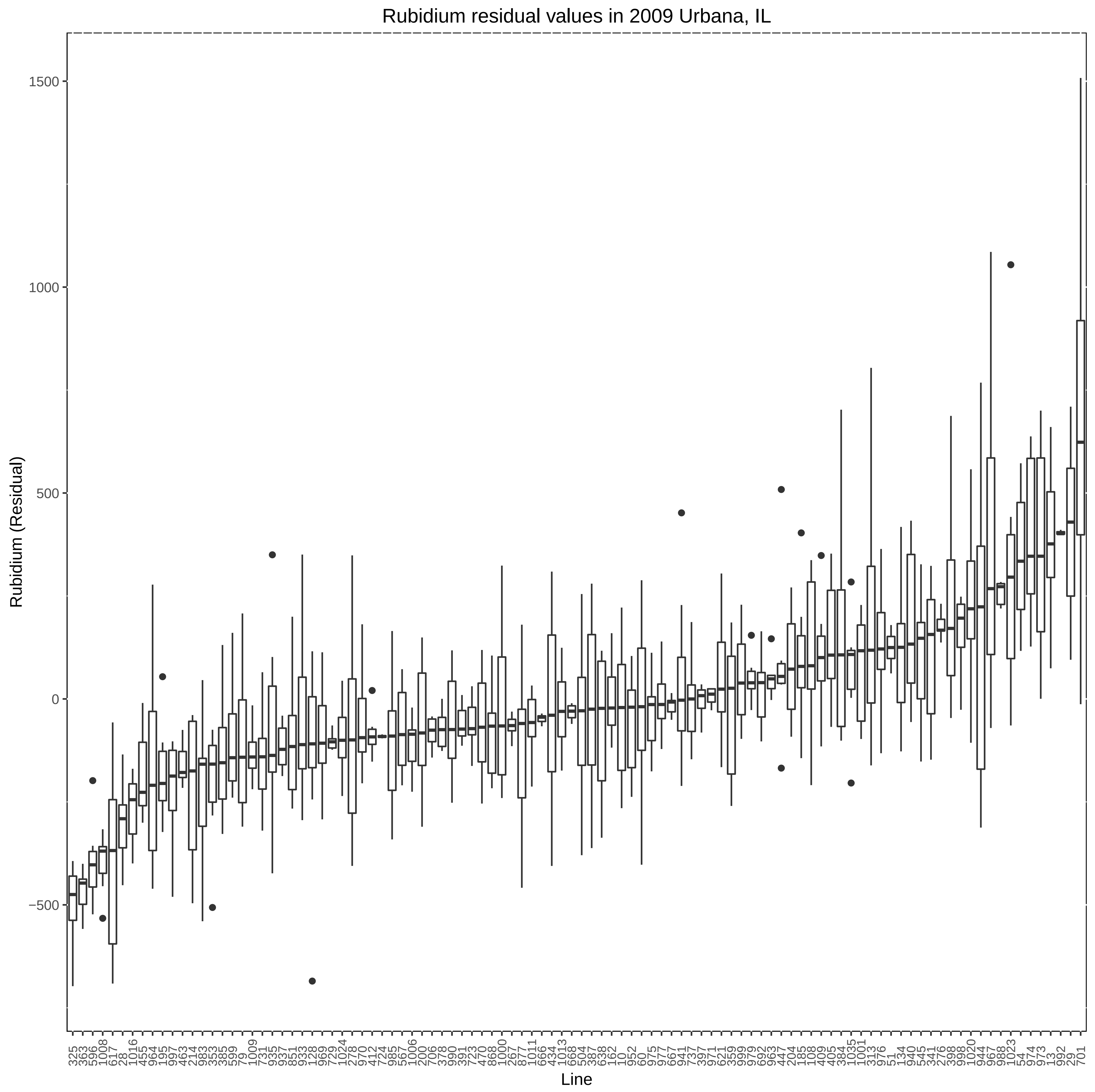
Rubidium residual values in 2009 Urbana, IL
Rubidium (Residual)
1500
1000
500
0
−500
Line

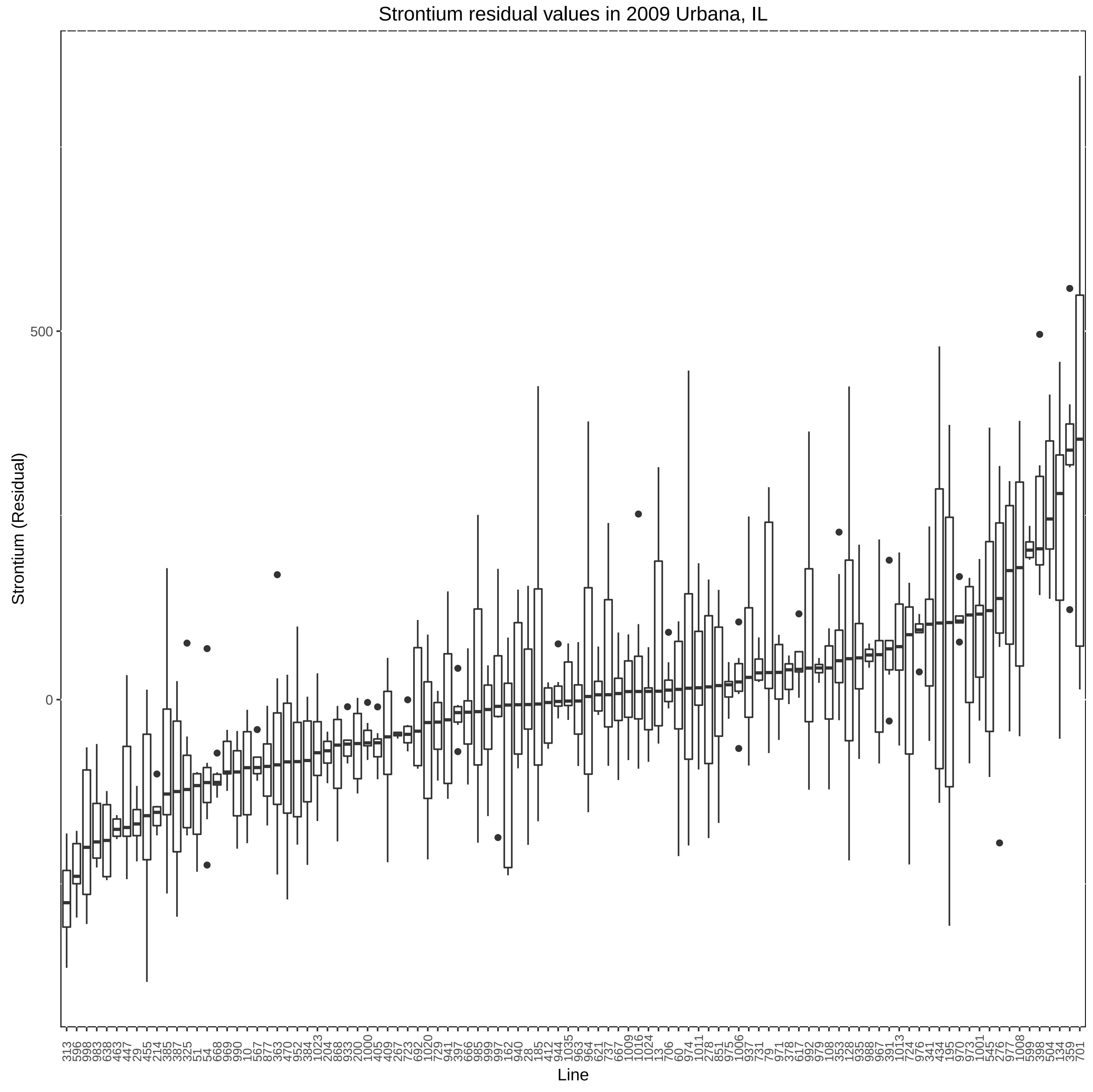
Strontium residual values in 2009 Urbana, IL
Strontium (Residual)
500
0
Line

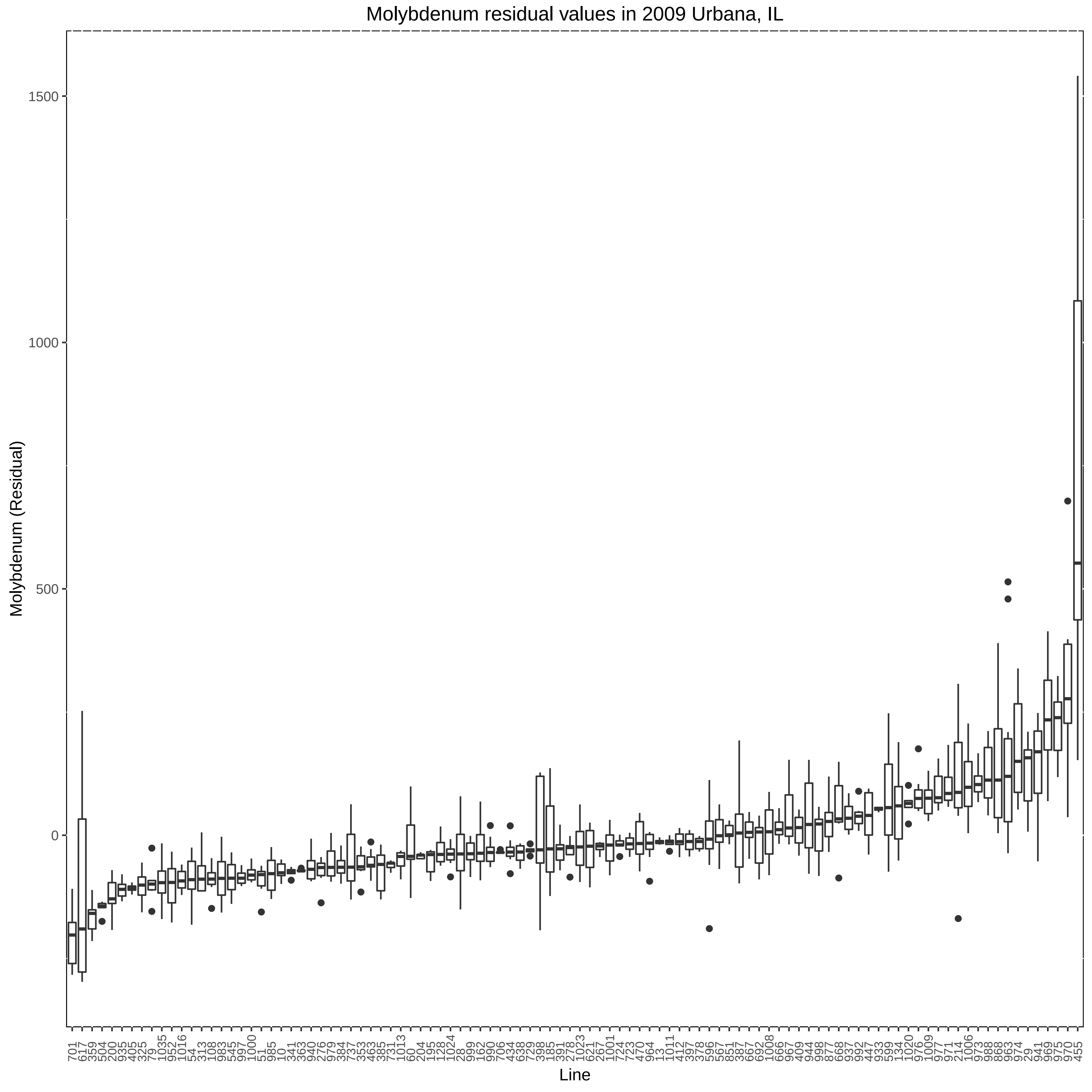
Molybdenum residual values in 2009 Urbana, IL
Molybdenum (Residual)
1500
1000
500
0
Line

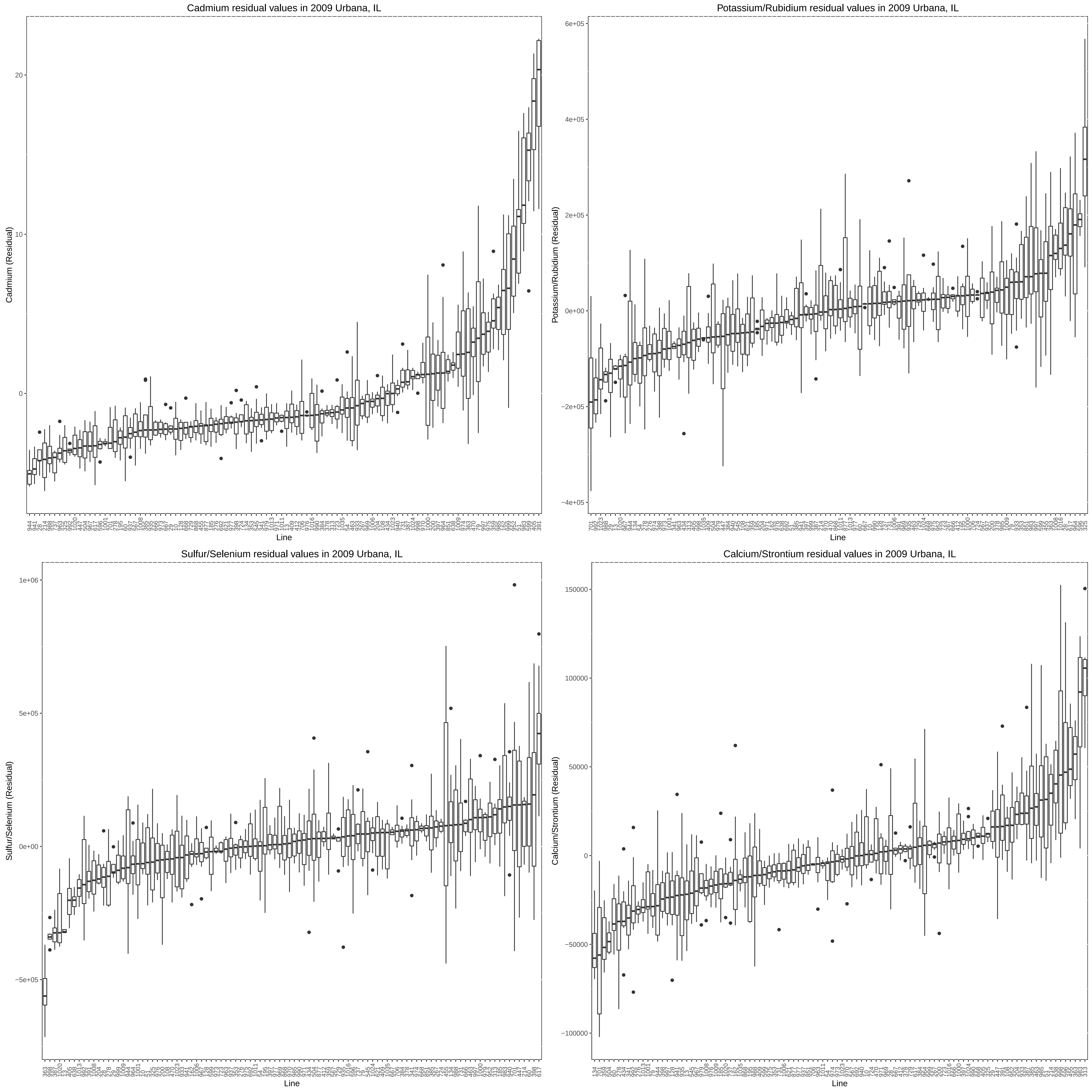
Cadmium residual values in 2009 Urbana, IL
Cadmium (Residual)
20
10
0
Line
Potassium/Rubidium residual values in 2009 Urbana, IL
Potassium/Rubidium (Residual)
6e+05
4e+05
2e+05
0e+00
−2e+05
−4e+05
Line
Sulfur/Selenium residual values in 2009 Urbana, IL
Sulfur/Selenium (Residual)
1e+06
5e+05
0e+00
−5e+05
Line
Calcium/Strontium residual values in 2009 Urbana, IL
Calcium/Strontium (Residual)
150000
100000
50000
0
−50000
−100000
Line

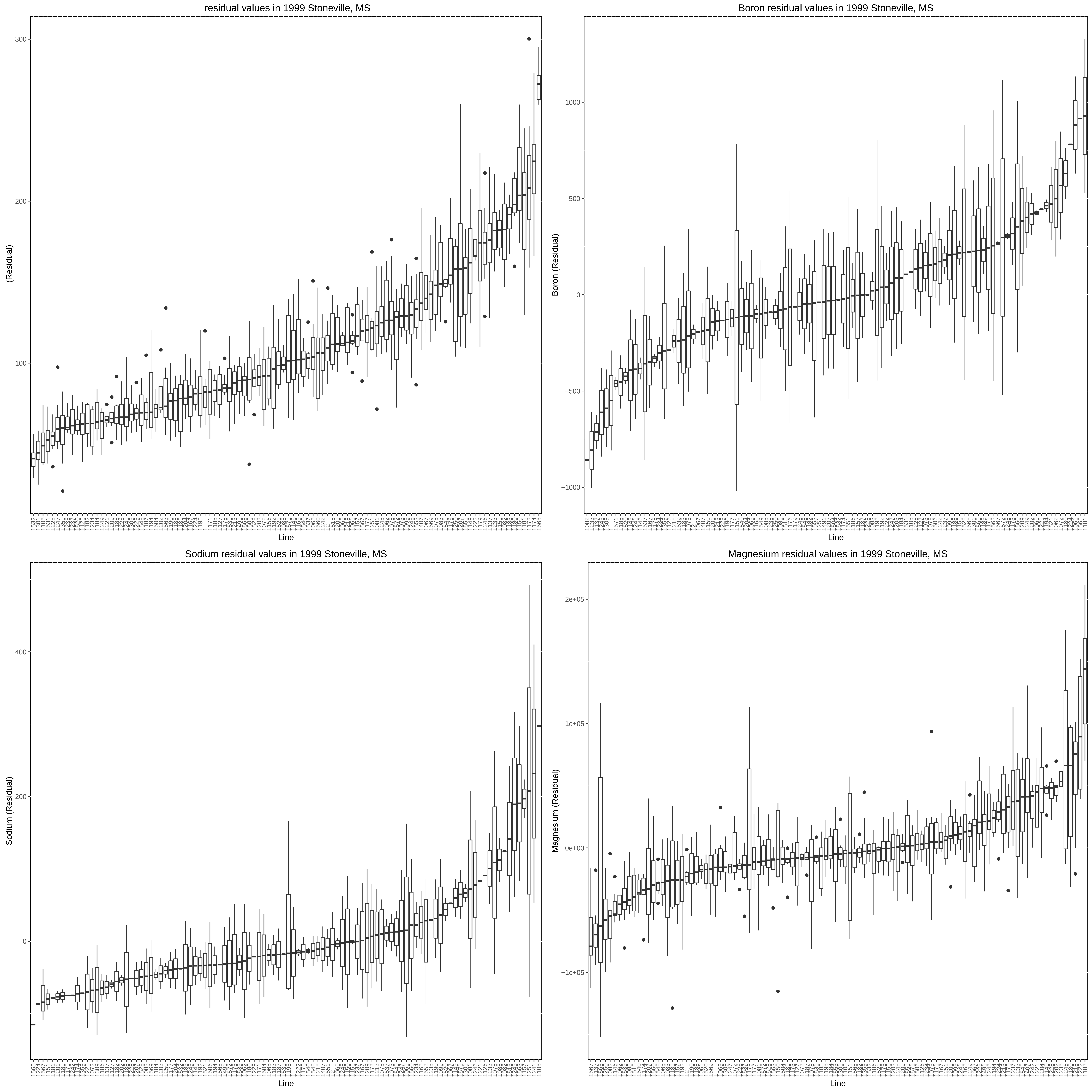
residual values in 1999 Stoneville, MS
(Residual)
Line
Boron residual values in 1999 Stoneville, MS
Boron (Residual)
Line
Sodium residual values in 1999 Stoneville, MS
Sodium (Residual)
Line
Magnesium residual values in 1999 Stoneville, MS
Magnesium (Residual)
Line

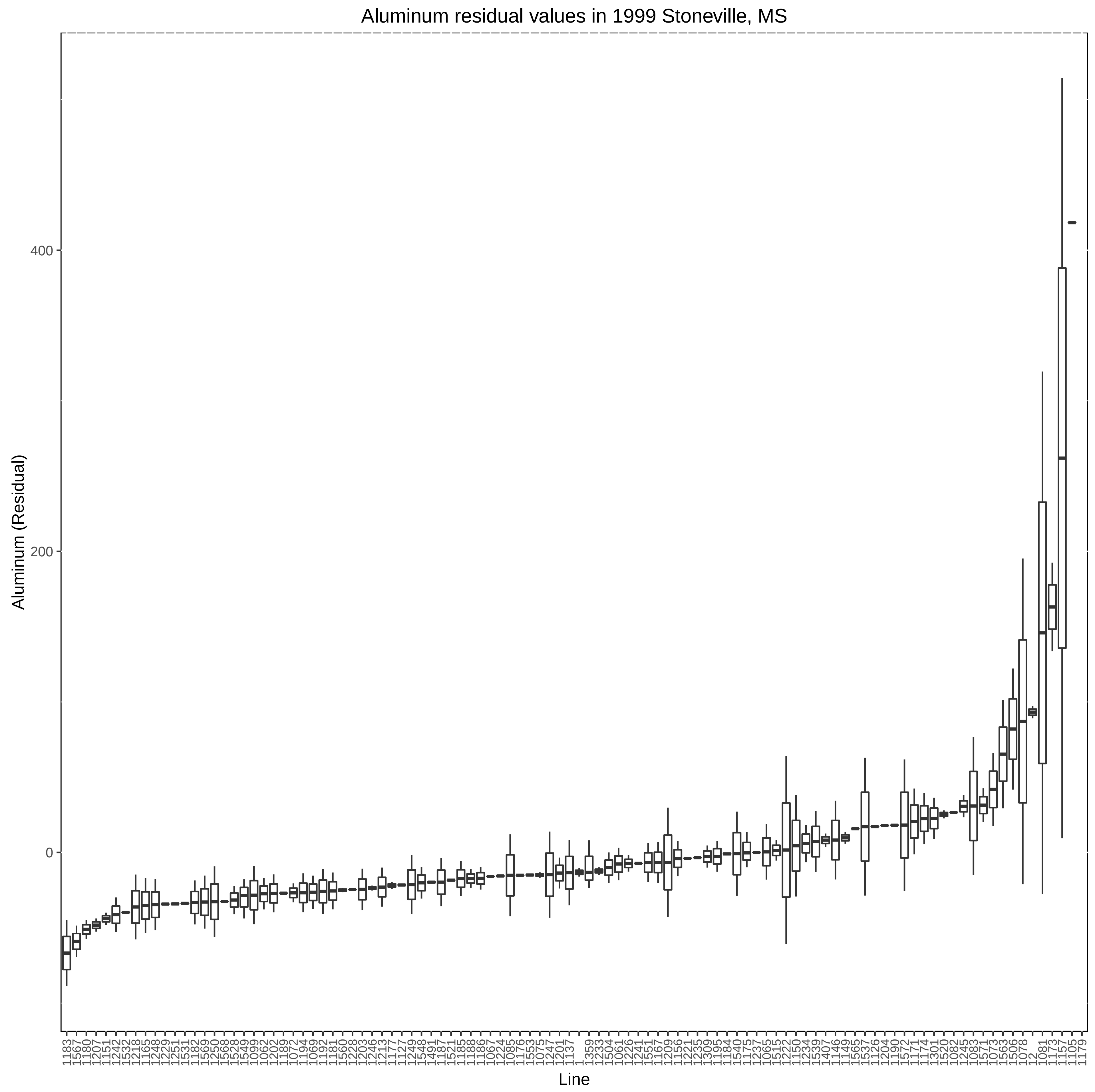
Aluminum residual values in 1999 Stoneville, MS
Aluminum (Residual)
400
200
0
Line

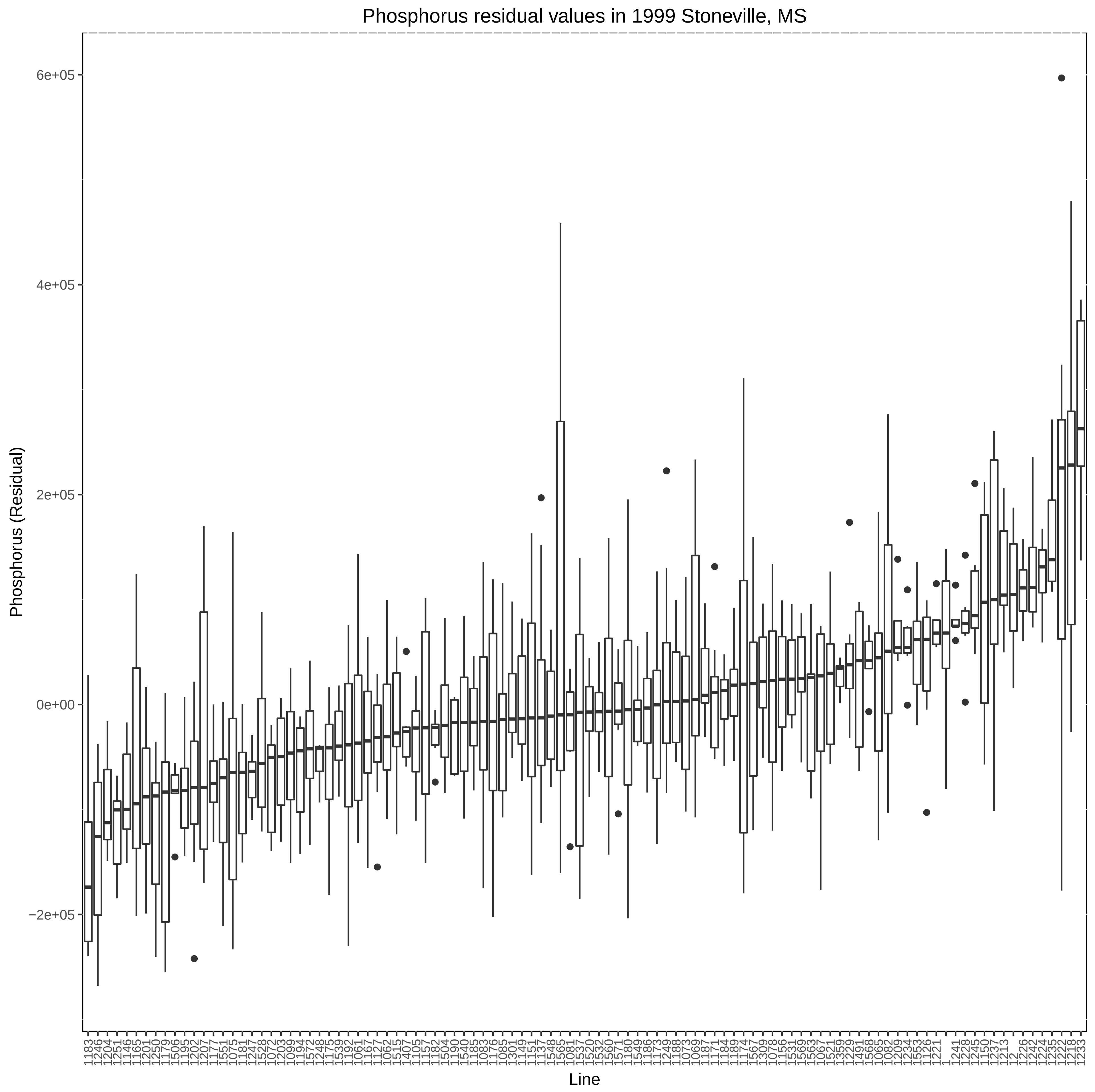
Phosphorus residual values in 1999 Stoneville, MS
Phosphorus (Residual)
6e+05
4e+05
2e+05
0e+00
−2e+05
Line

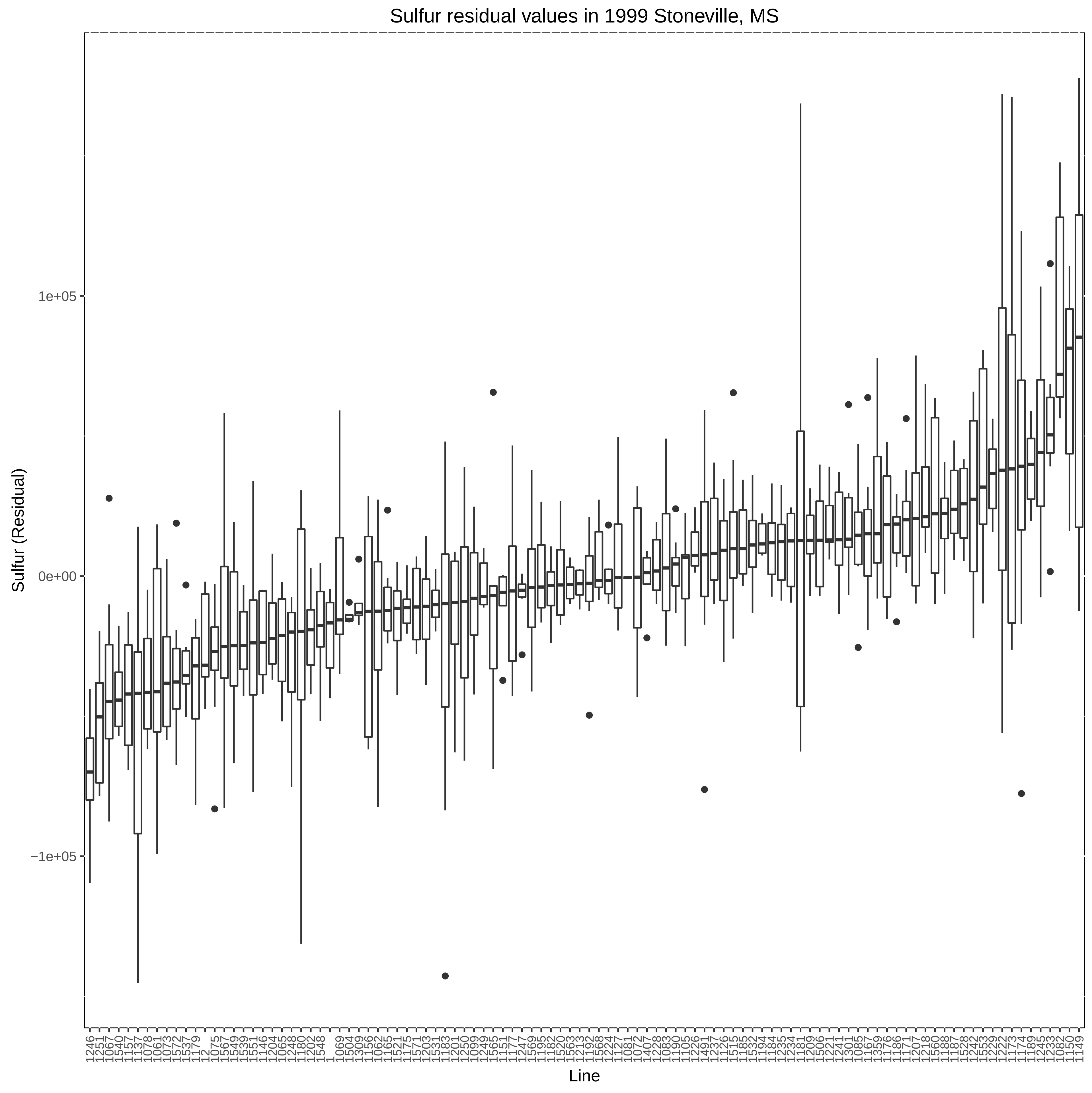
Sulfur residual values in 1999 Stoneville, MS
Sulfur (Residual)
1e+05
0e+00
−1e+05
Line

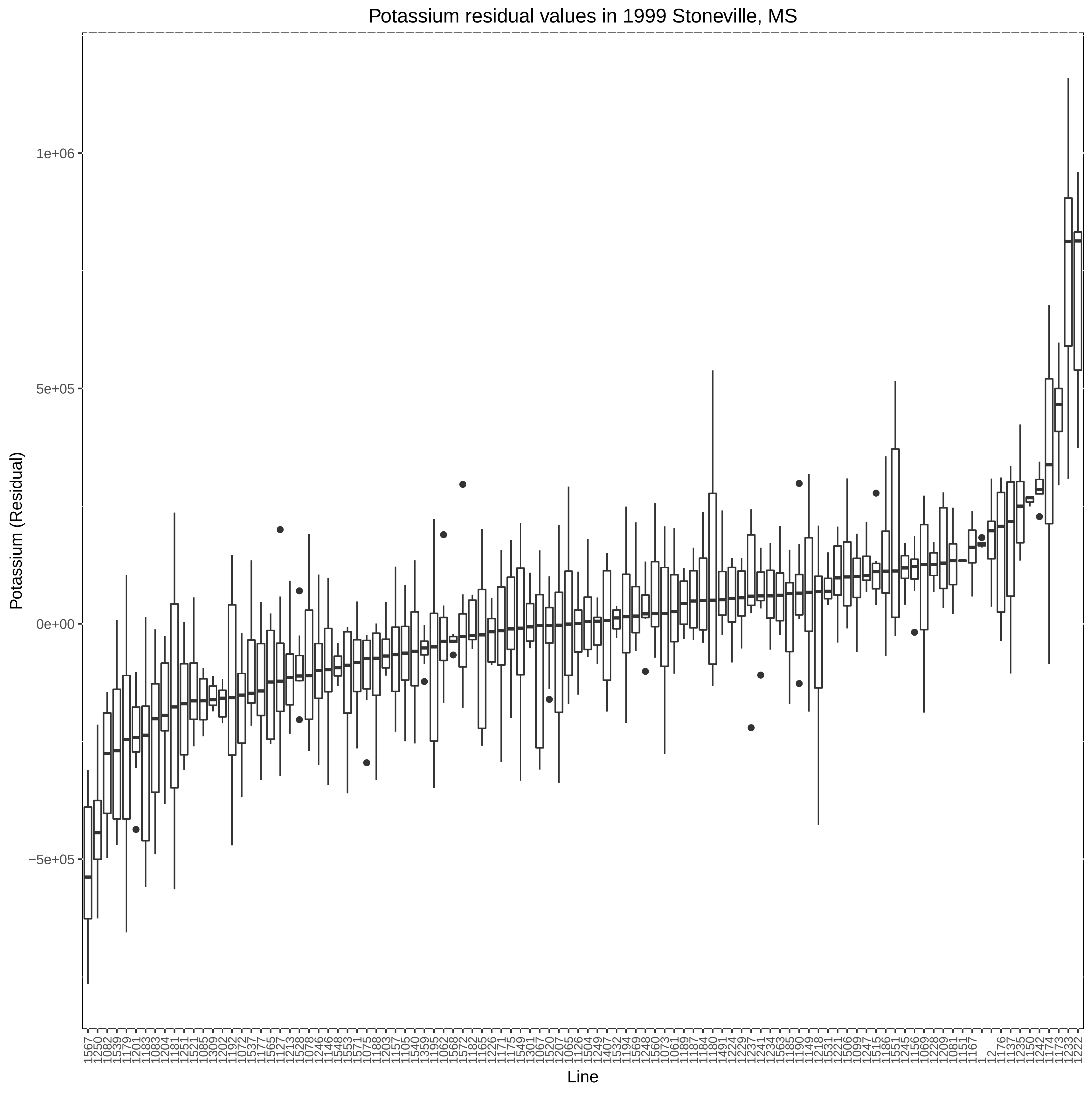
Potassium residual values in 1999 Stoneville, MS
Potassium (Residual)
1e+06
5e+05
0e+00
−5e+05
Line

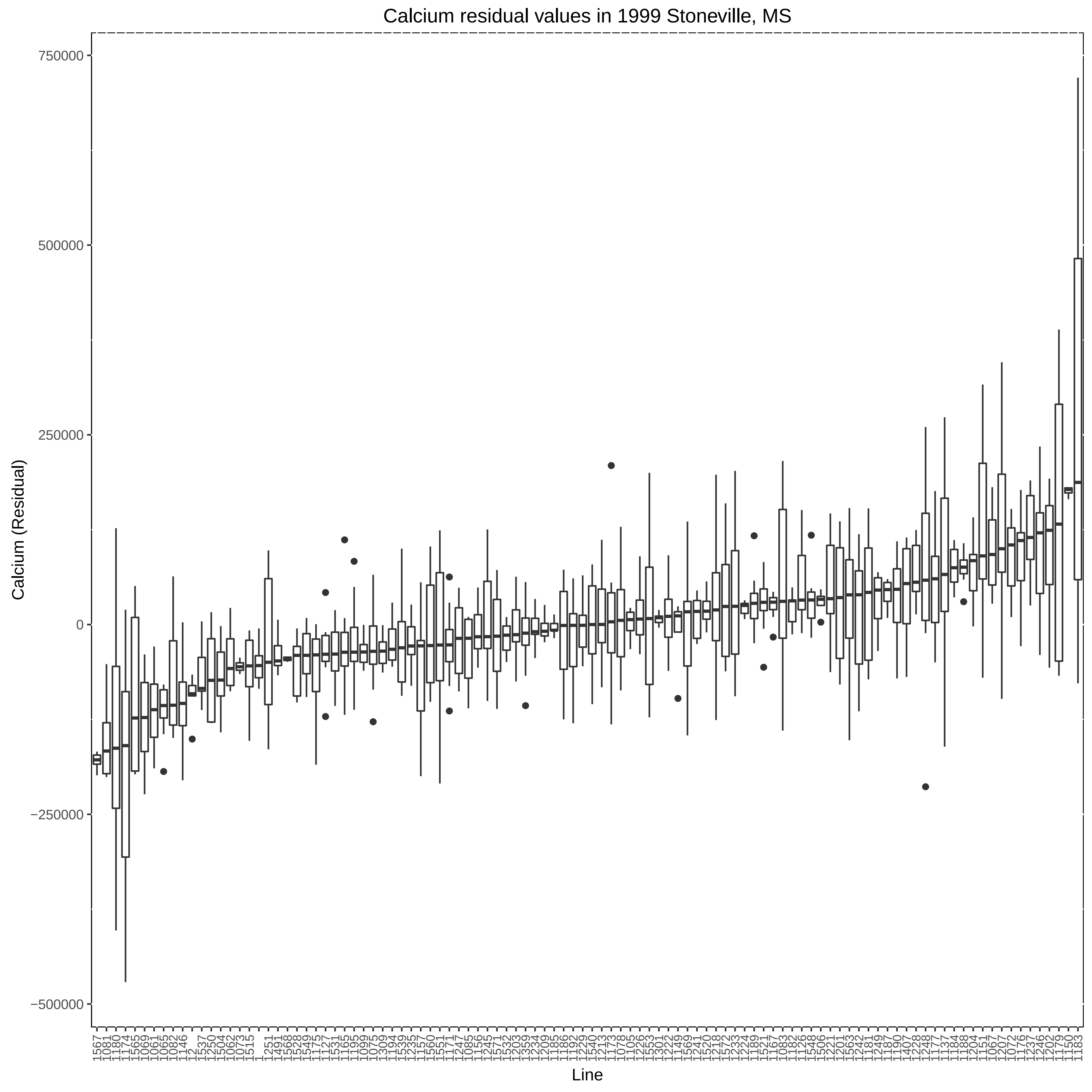
Calcium residual values in 1999 Stoneville, MS
Calcium (Residual)
750000
500000
250000
0
-250000
-500000
Line

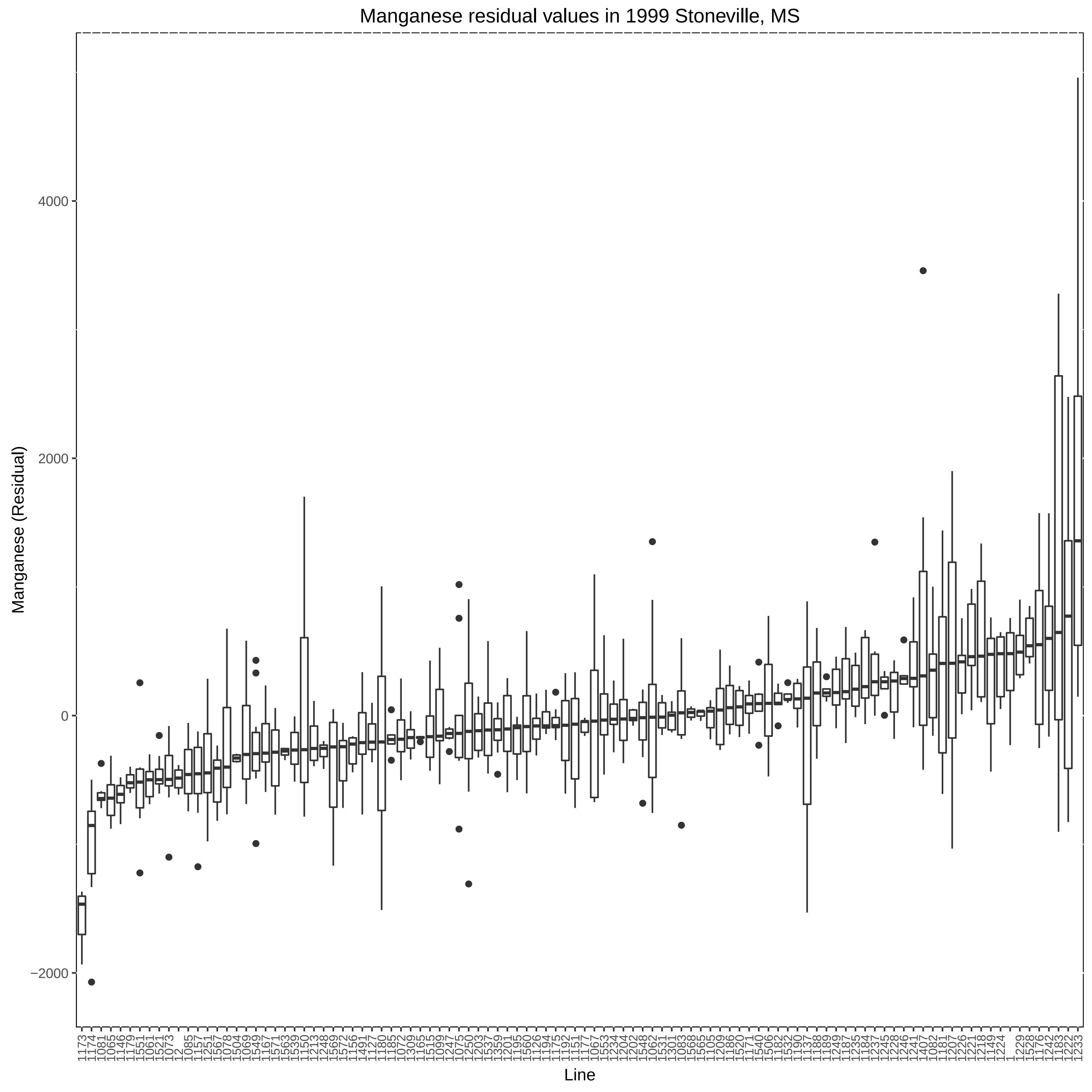
Manganese residual values in 1999 Stoneville, MS
Manganese (Residual)
4000
2000
0
-2000
Line

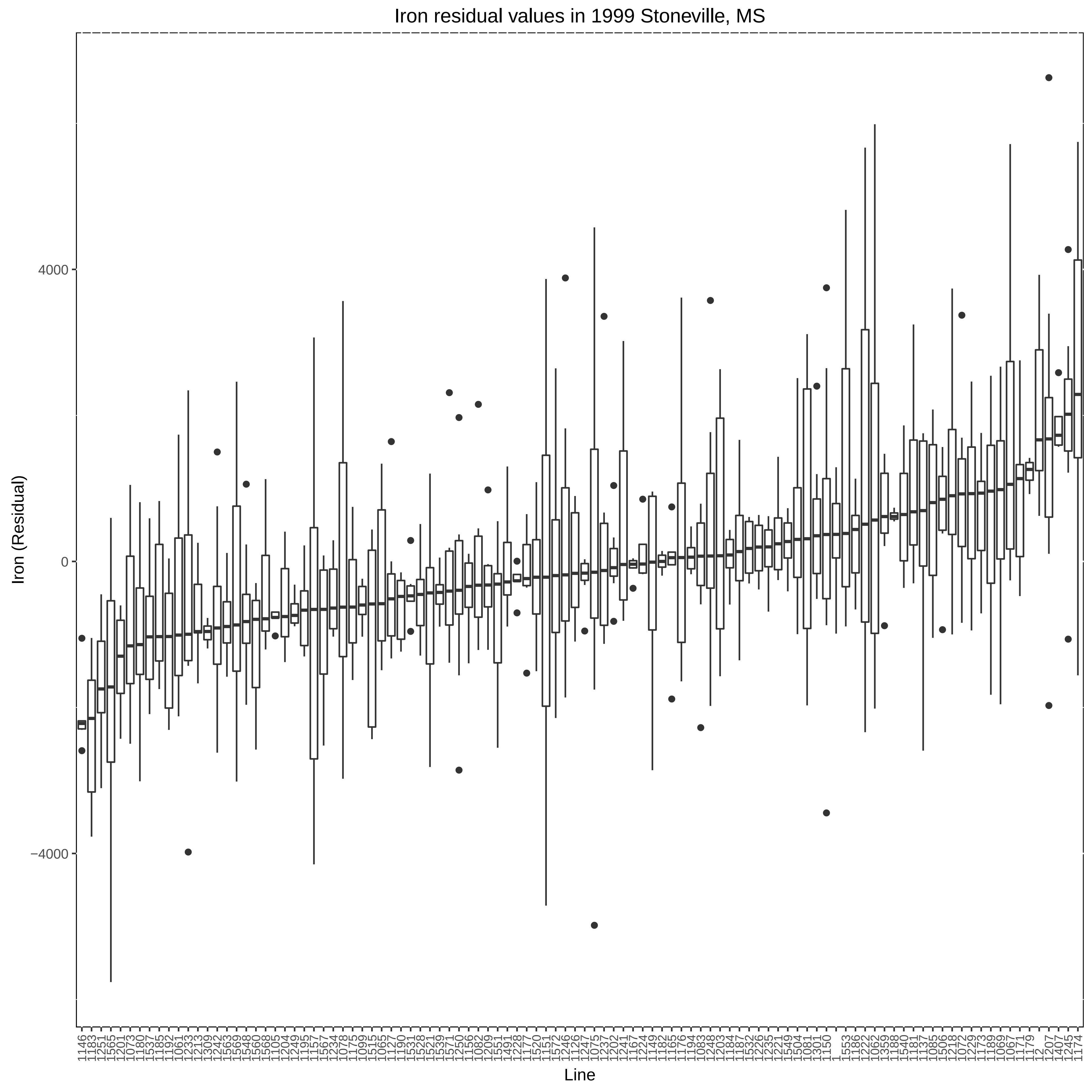
Iron residual values in 1999 Stoneville, MS
Iron (Residual)
4000
0
-4000
Line

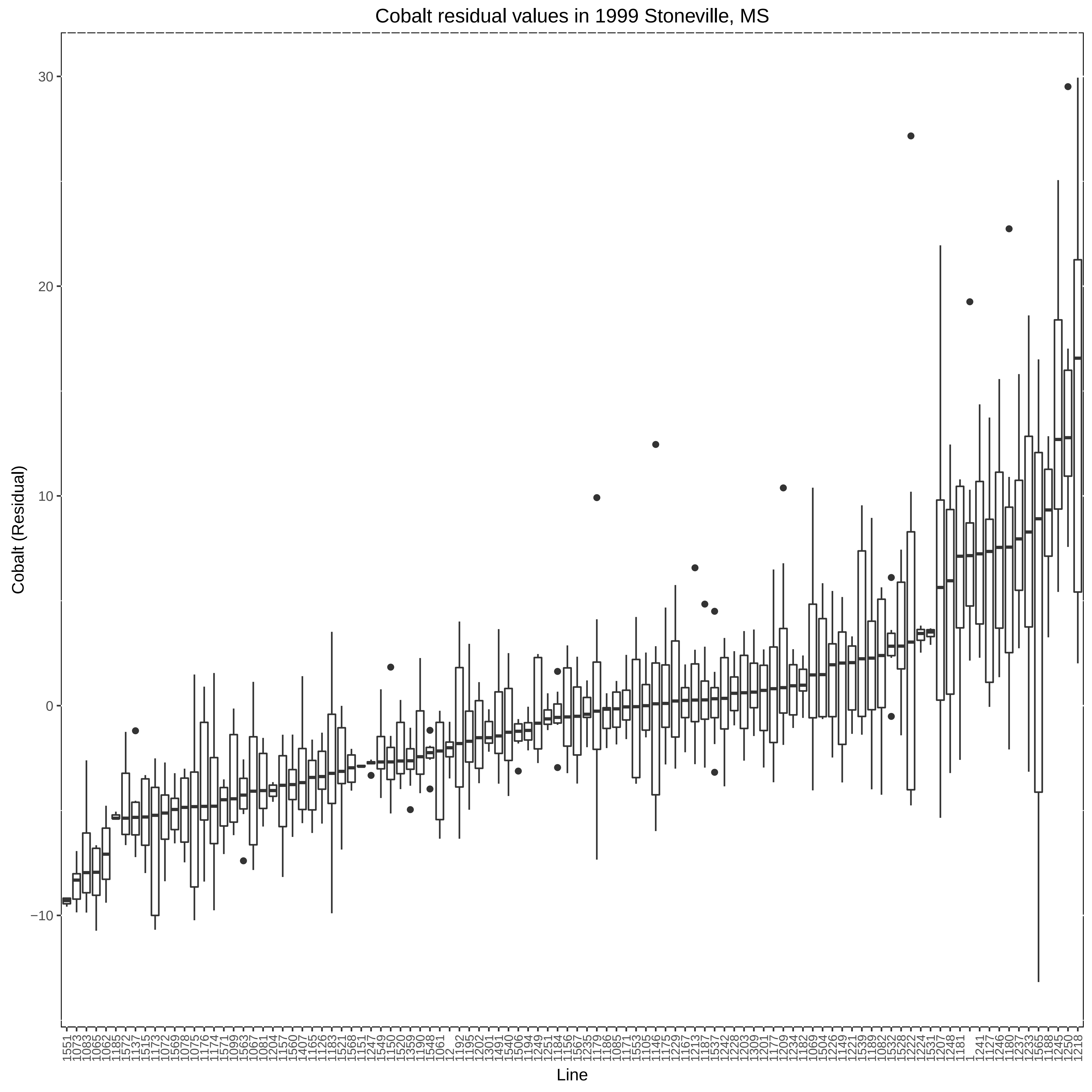
Cobalt residual values in 1999 Stoneville, MS
Cobalt (Residual)
30
20
10
0
-10
Line

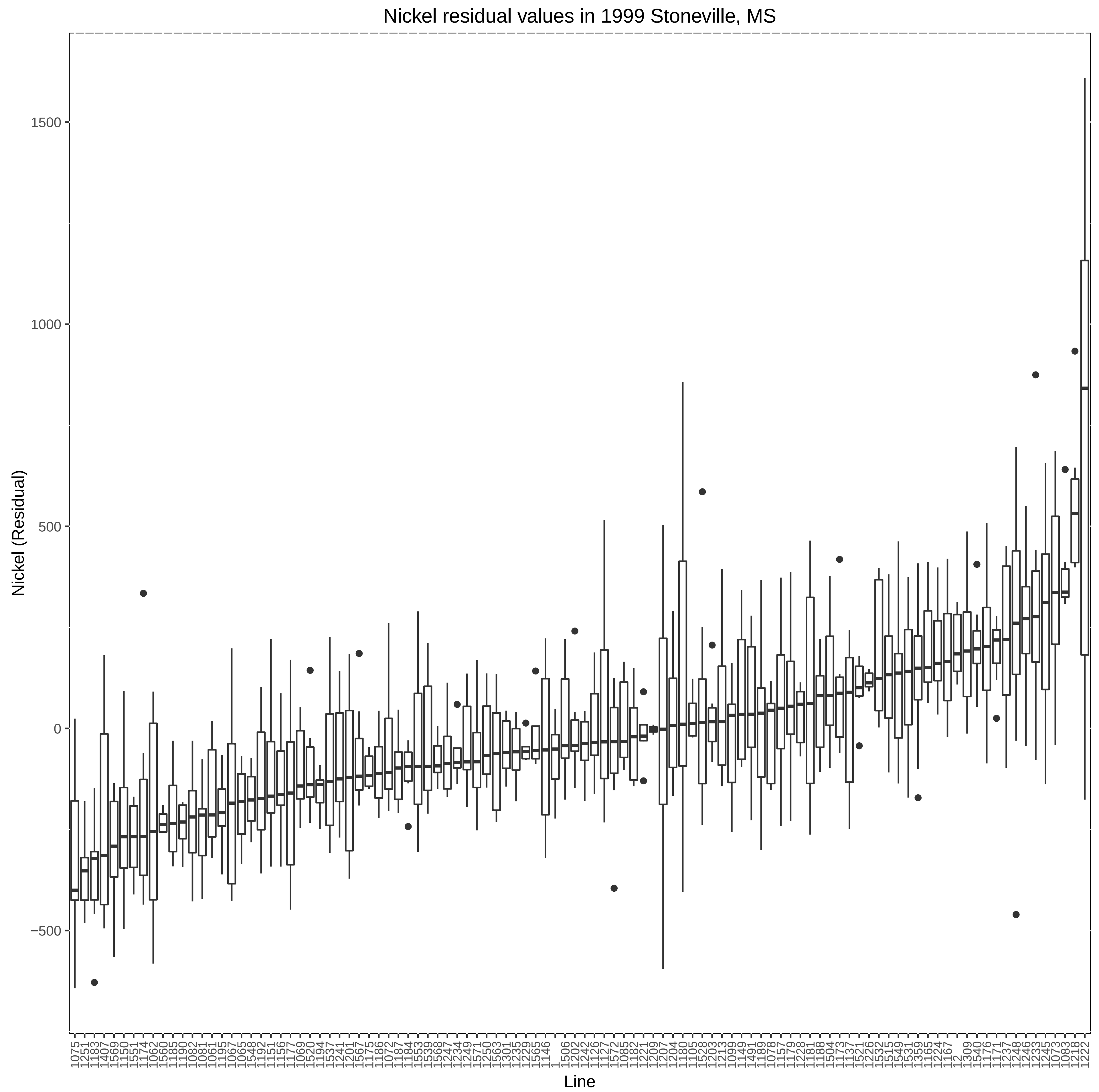
Nickel residual values in 1999 Stoneville, MS
Nickel (Residual)
1500
1000
500
0
-500
Line

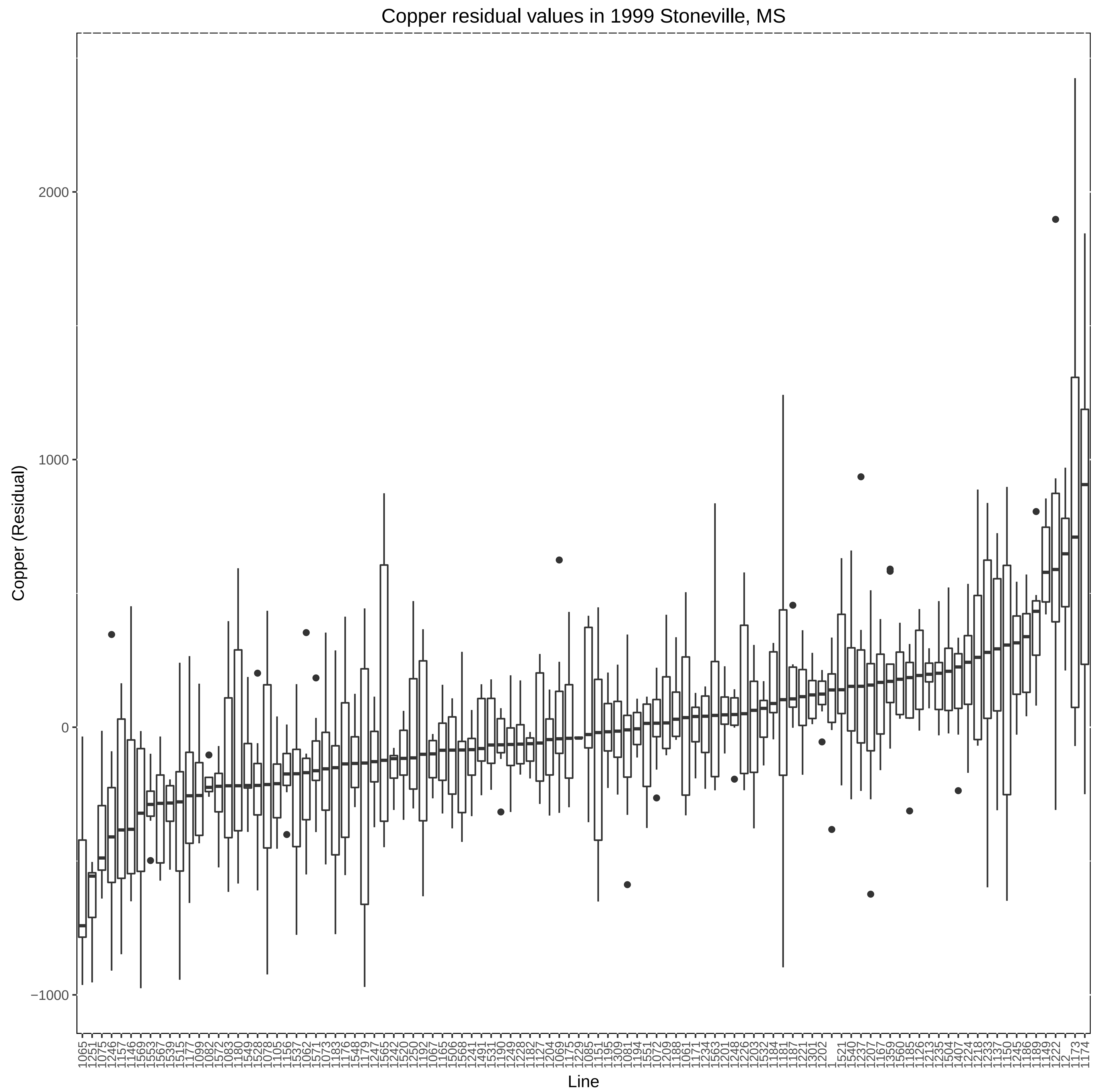
Copper residual values in 1999 Stoneville, MS
Copper (Residual)
2000
1000
0
−1000
Line

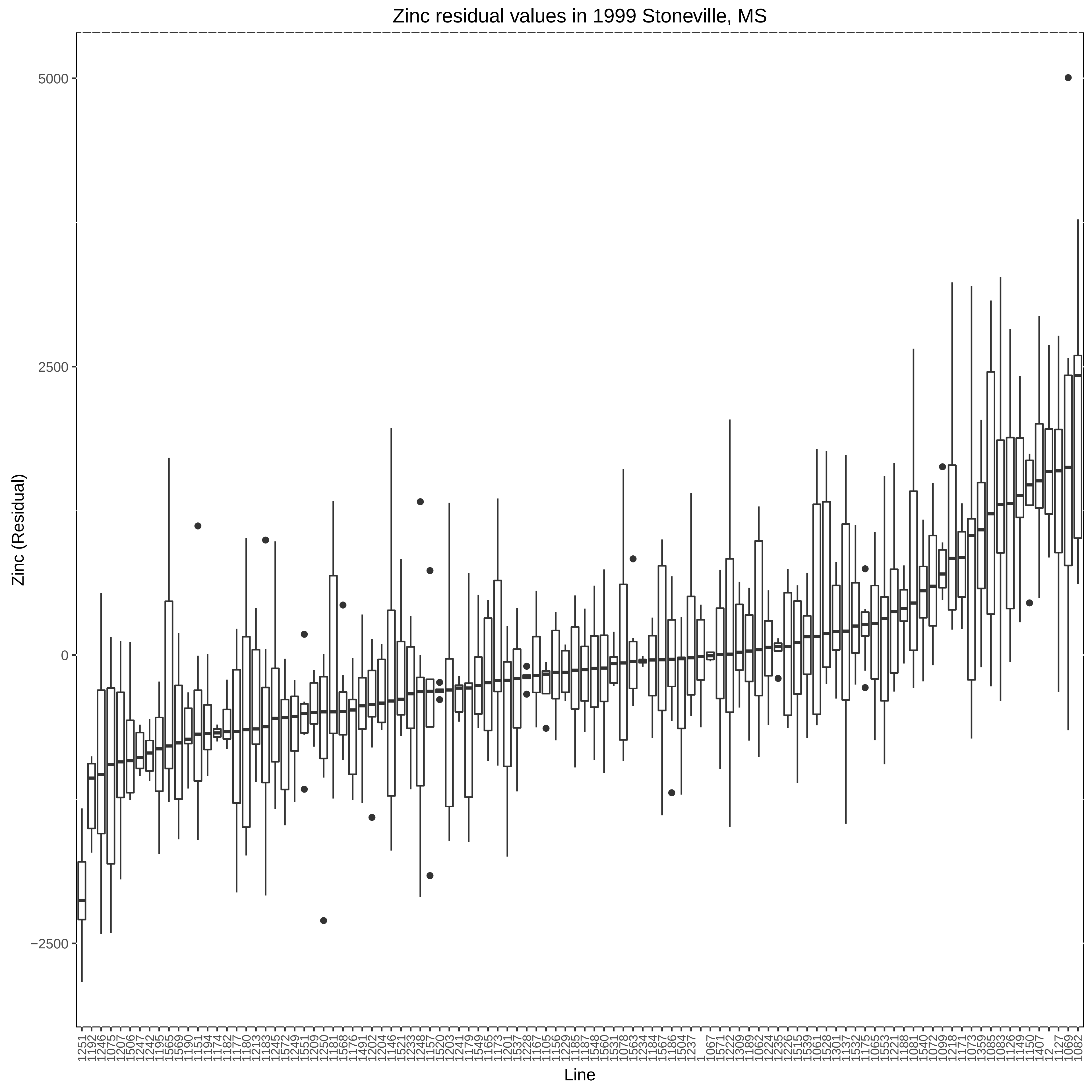
Zinc residual values in 1999 Stoneville, MS
Zinc (Residual)
5000
2500
0
-2500
Line

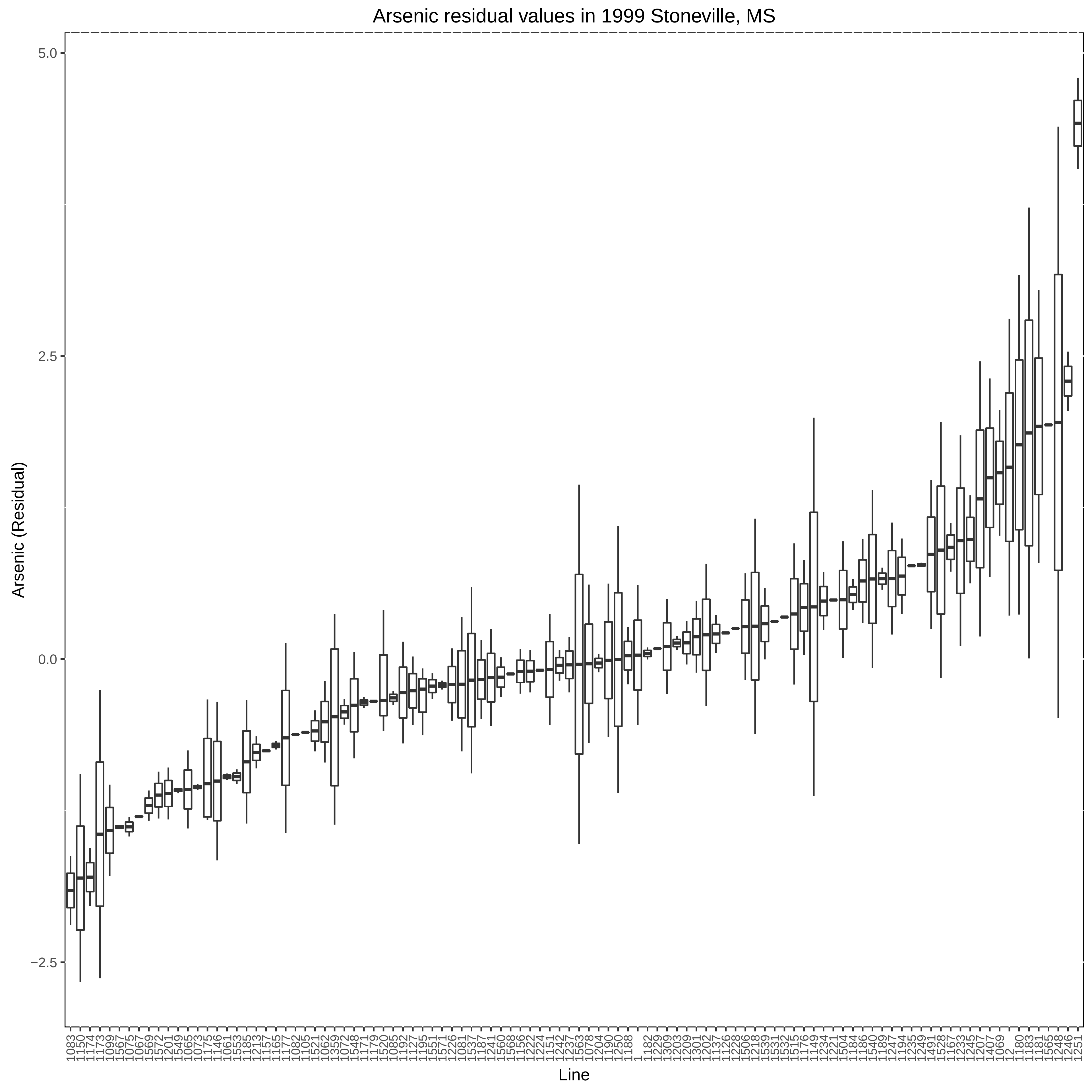
Arsenic residual values in 1999 Stoneville, MS
Arsenic (Residual)
5.0
2.5
0.0
-2.5
Line

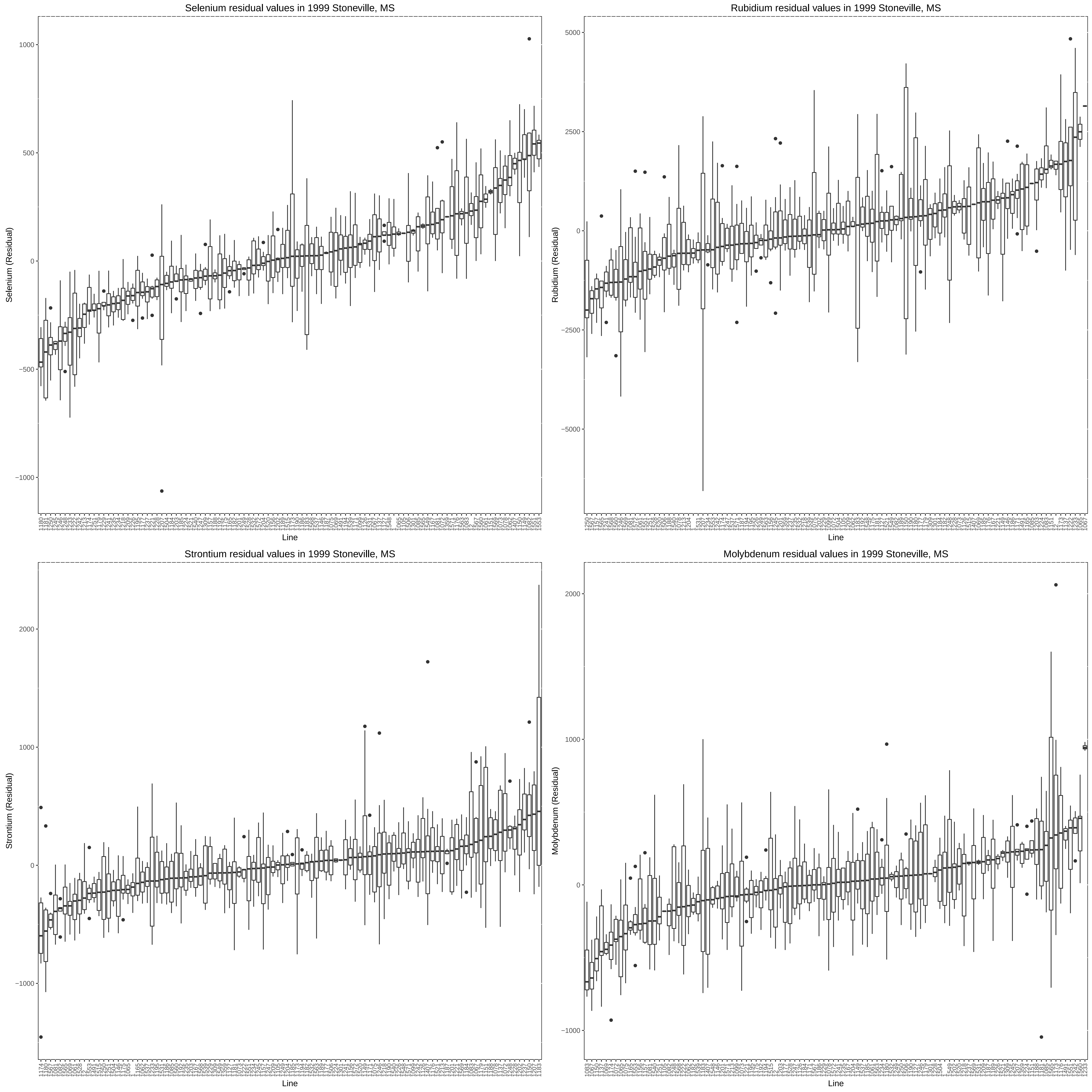

Selenium residual values in 1999 Stoneville, MS
Selenium (Residual)
Line
Rubidium residual values in 1999 Stoneville, MS
Rubidium (Residual)
Line
Strontium residual values in 1999 Stoneville, MS
Strontium (Residual)
Line
Molybdenum residual values in 1999 Stoneville, MS
Molybdenum (Residual)
Line

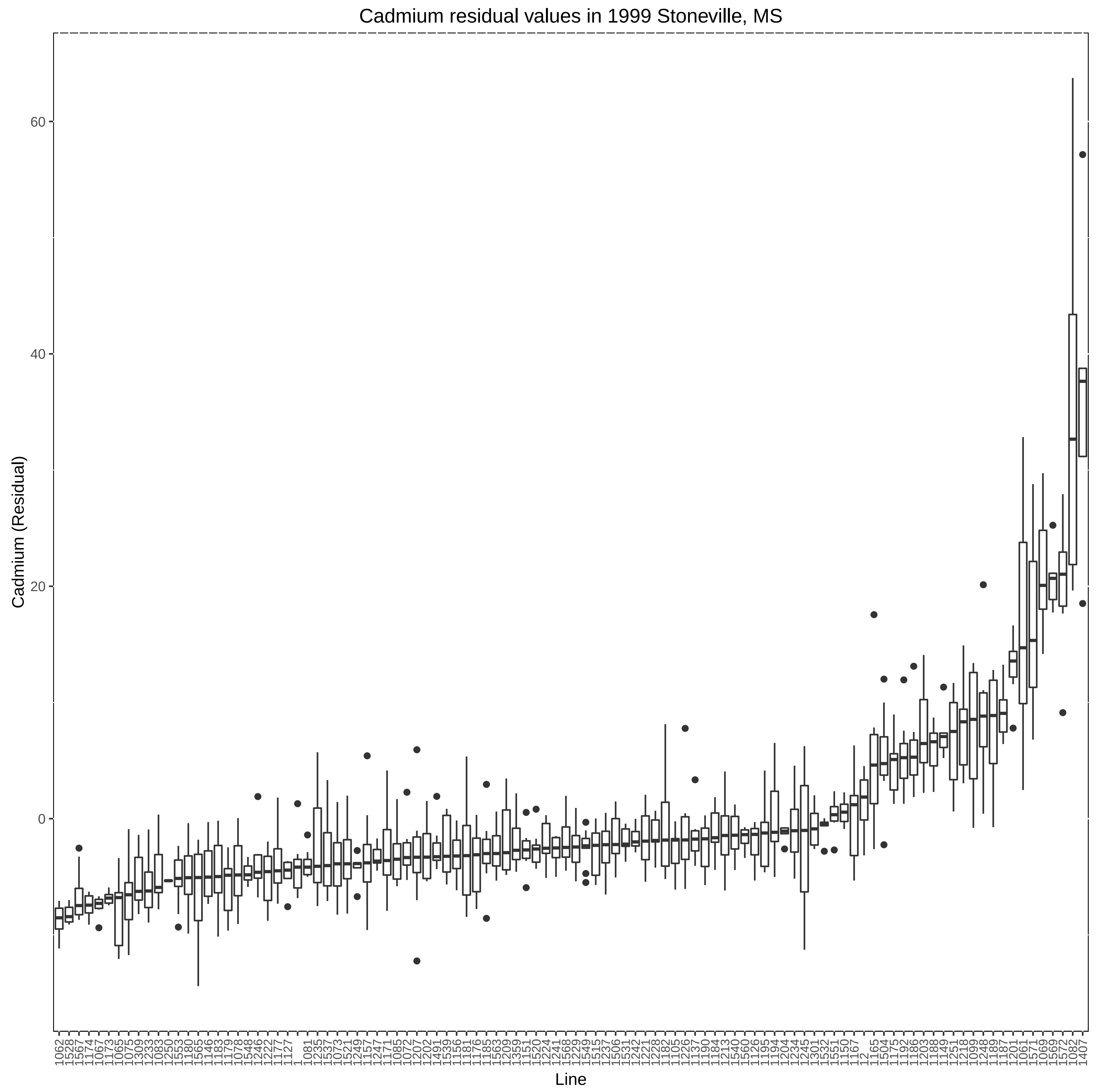
Cadmium residual values in 1999 Stoneville, MS
Cadmium (Residual)
Line

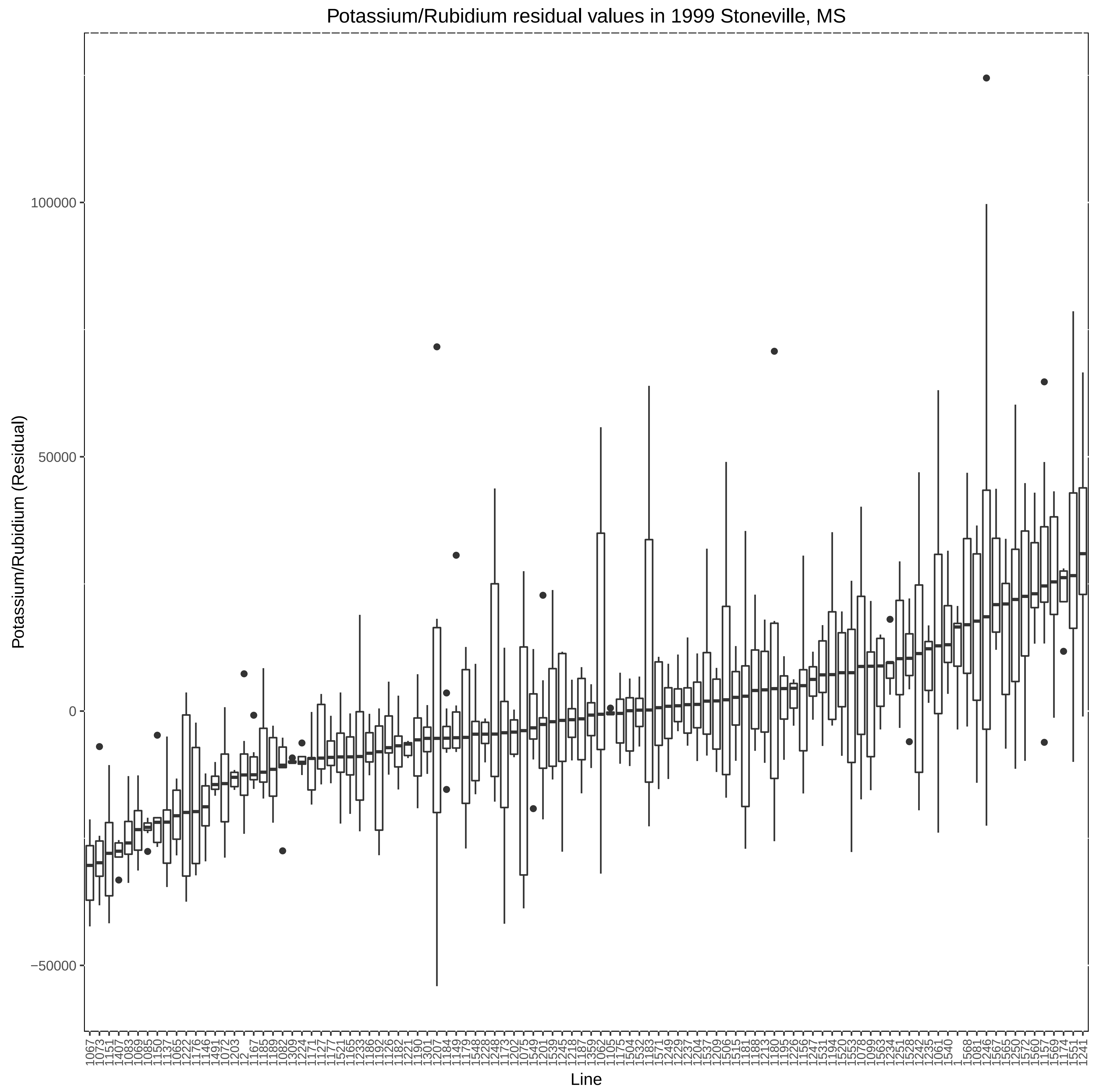
Potassium/Rubidium residual values in 1999 Stoneville, MS
Potassium/Rubidium (Residual)
Line

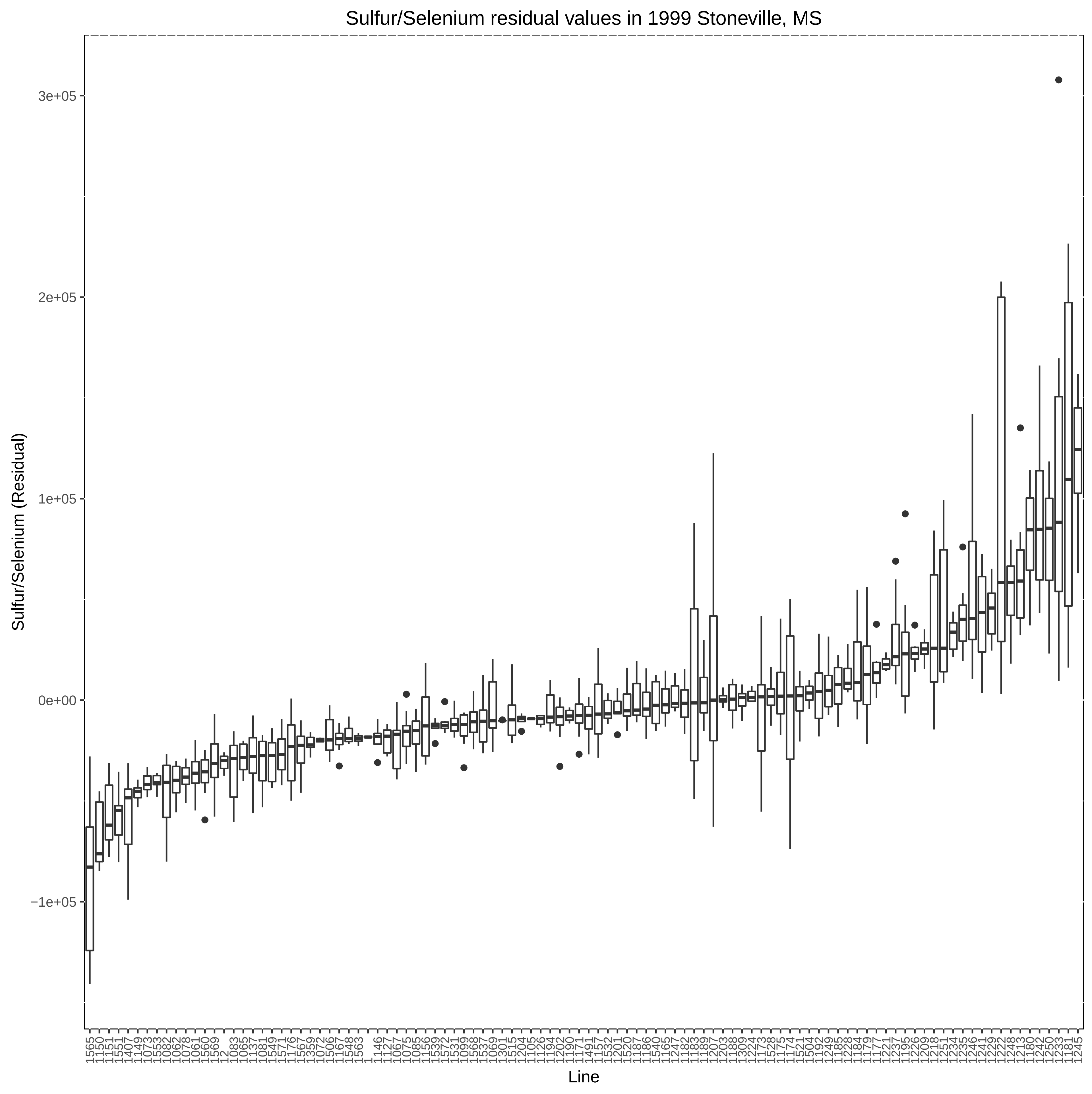
Sulfur/Selenium residual values in 1999 Stoneville, MS
Sulfur/Selenium (Residual)
Line

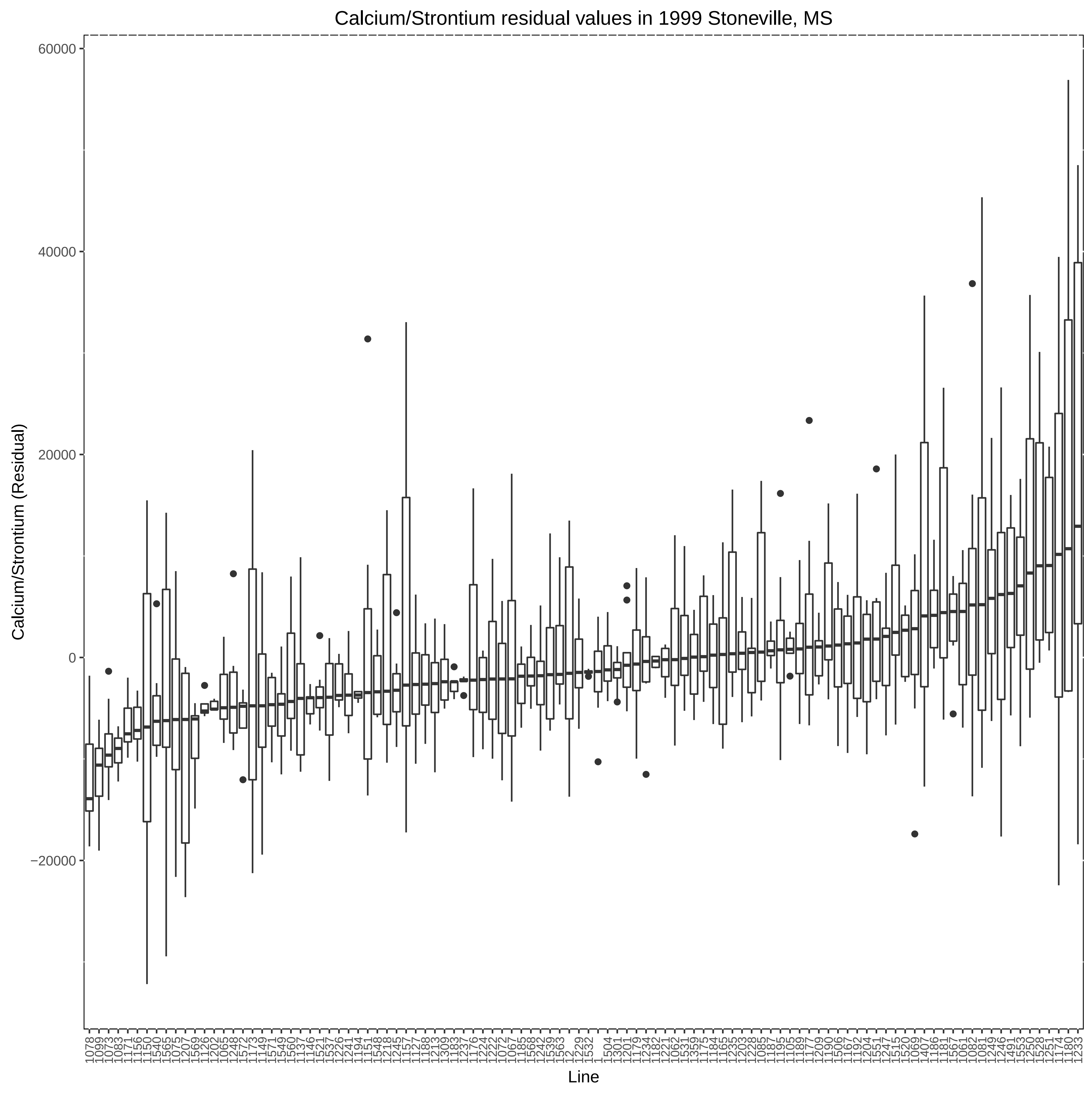
Calcium/Strontium residual values in 1999 Stoneville, MS
Calcium/Strontium (Residual)
Line

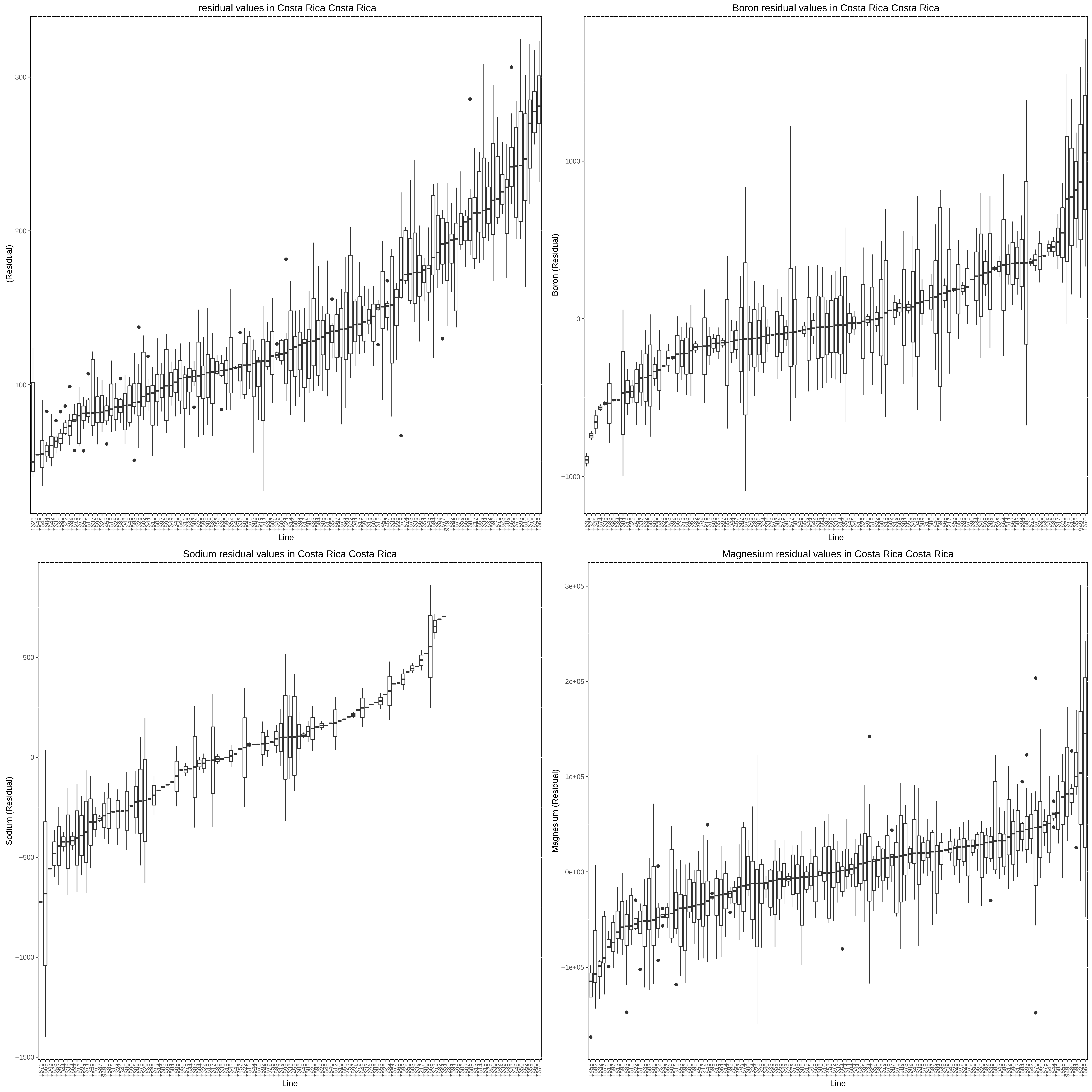
residual values in Costa Rica Costa Rica
(Residual)
300
200
100
Line
Boron residual values in Costa Rica Costa Rica
Boron (Residual)
1000
0
−1000
Line
Sodium residual values in Costa Rica Costa Rica
Sodium (Residual)
500
0
-500
-1000
-1500
Line
Magnesium residual values in Costa Rica Costa Rica
Magnesium (Residual)
3e+05
2e+05
1e+05
0e+00
-1e+05
Line

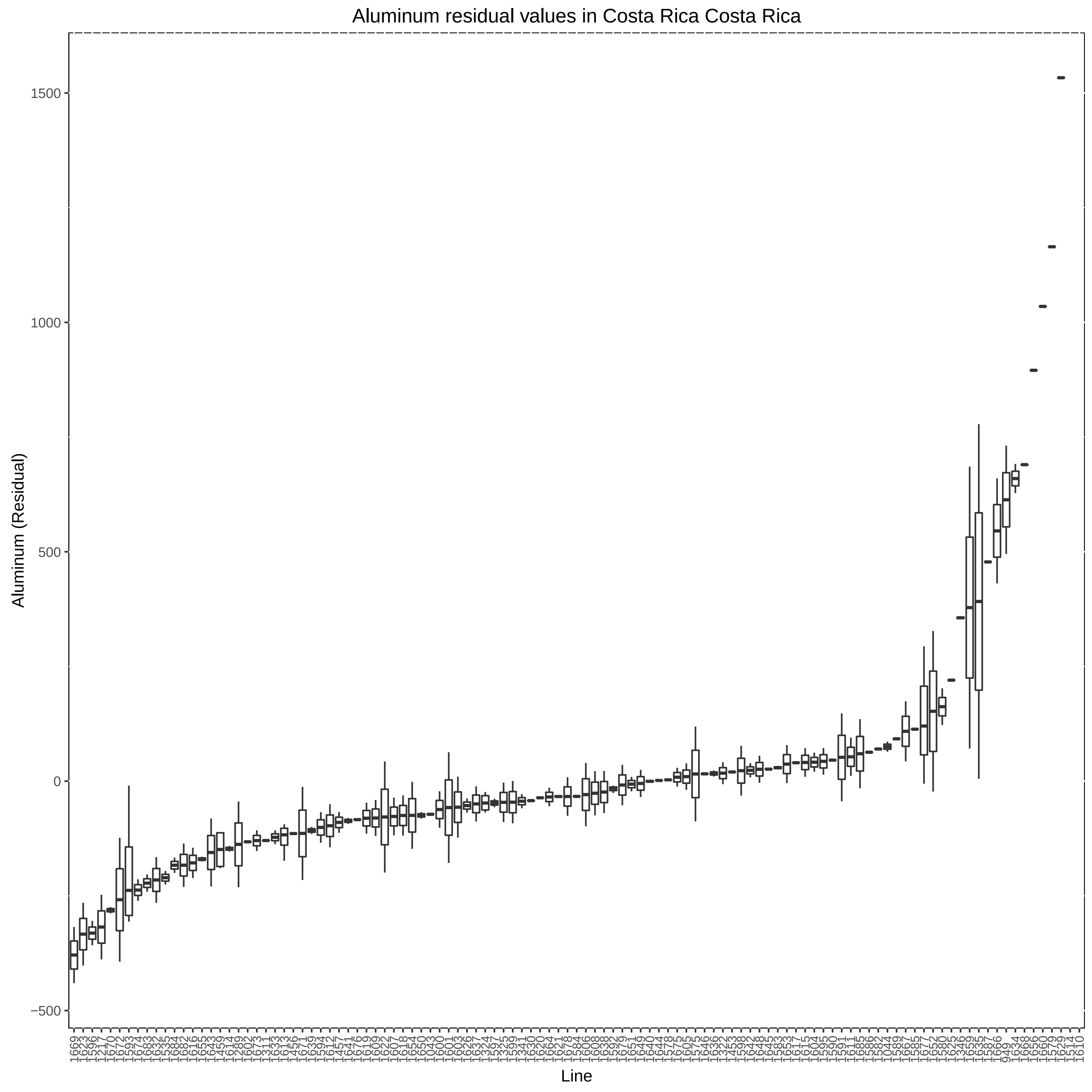
Aluminum residual values in Costa Rica Costa Rica
Aluminum (Residual)
1500
1000
500
0
-500
Line

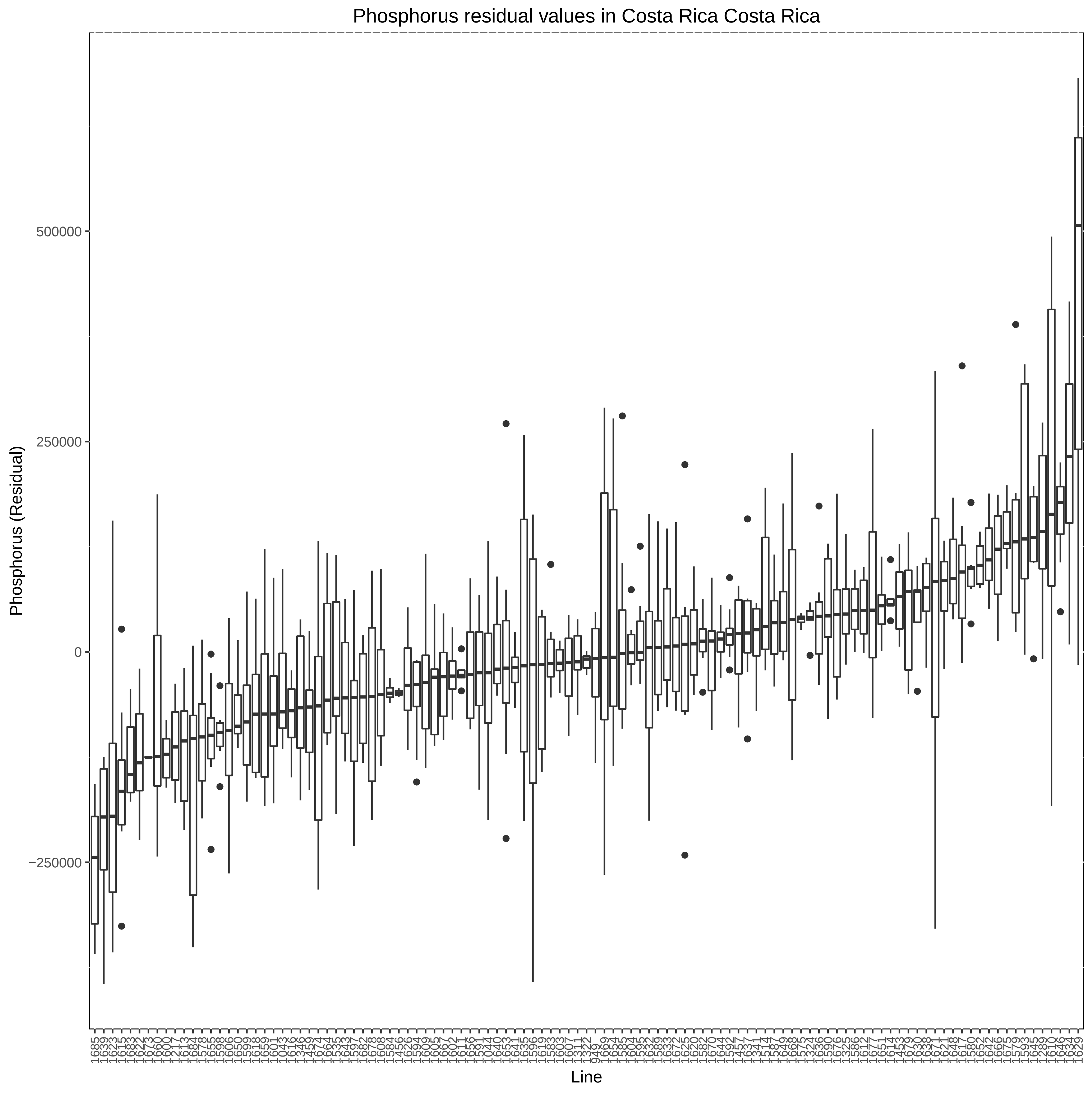
Phosphorus residual values in Costa Rica Costa Rica
Phosphorus (Residual)
500000
250000
0
-250000
Line

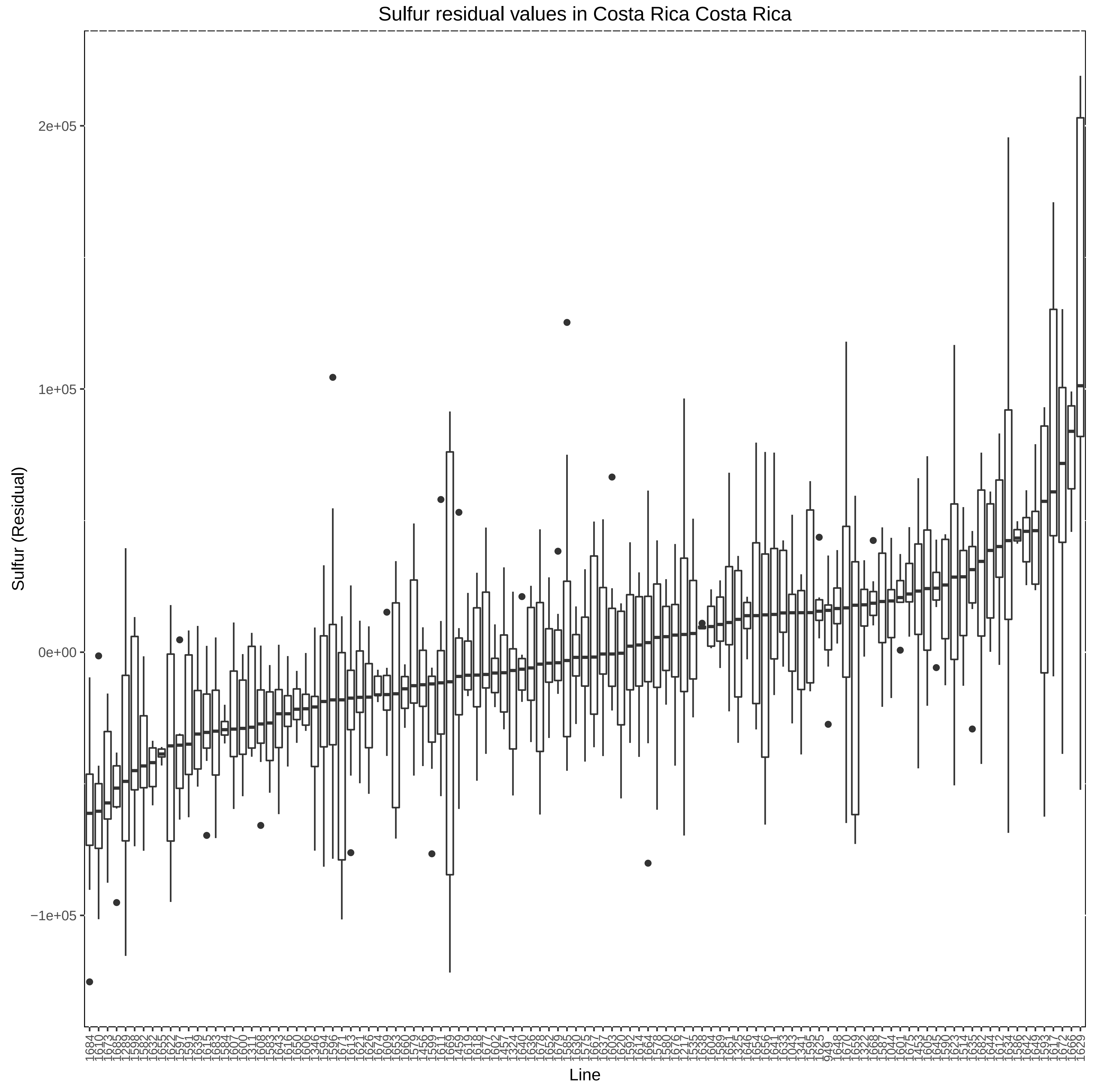
Sulfur residual values in Costa Rica Costa Rica
Sulfur (Residual)
2e+05
1e+05
0e+00
-1e+05
Line

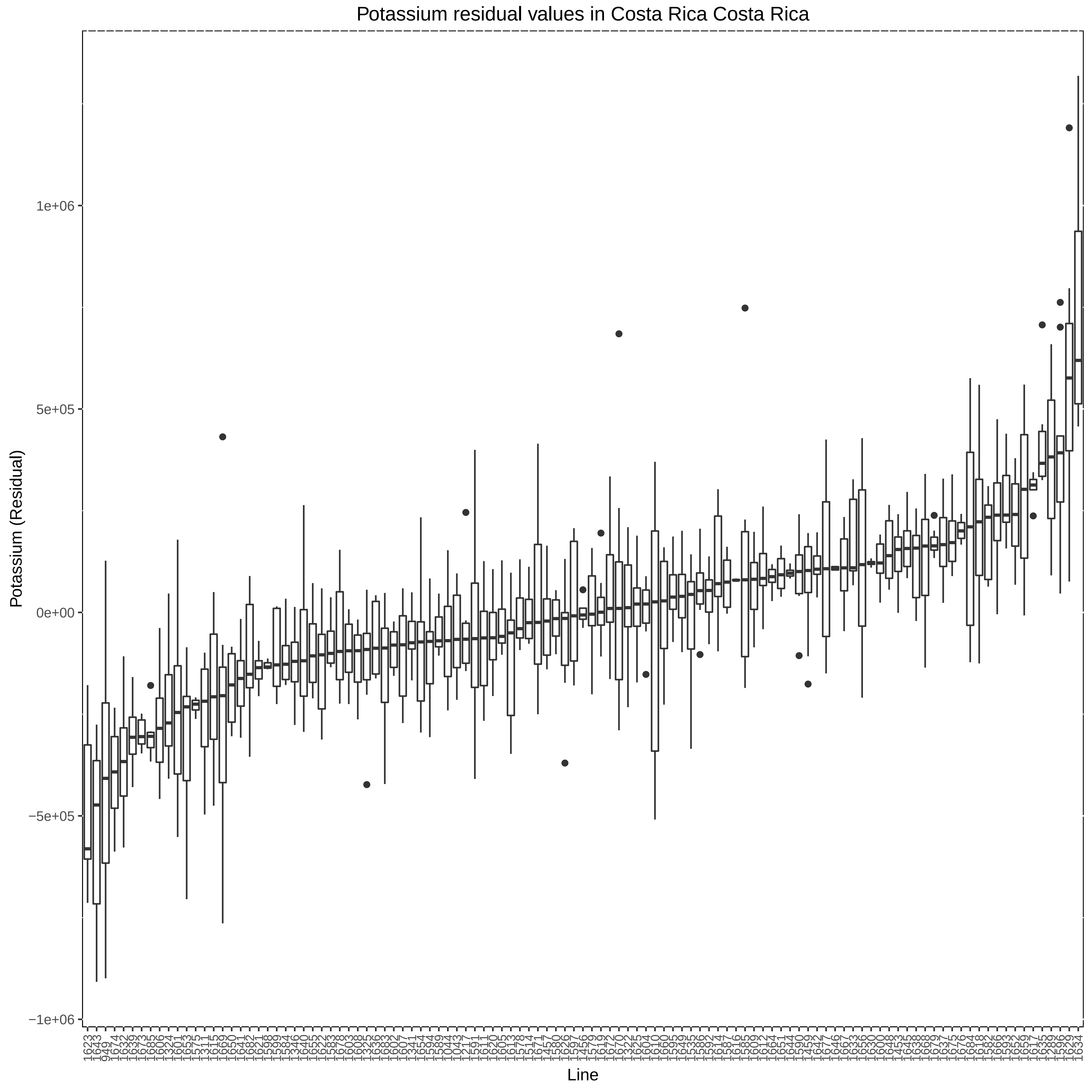
Potassium residual values in Costa Rica Costa Rica
Potassium (Residual)
1e+06
5e+05
0e+00
-5e+05
-1e+06
Line

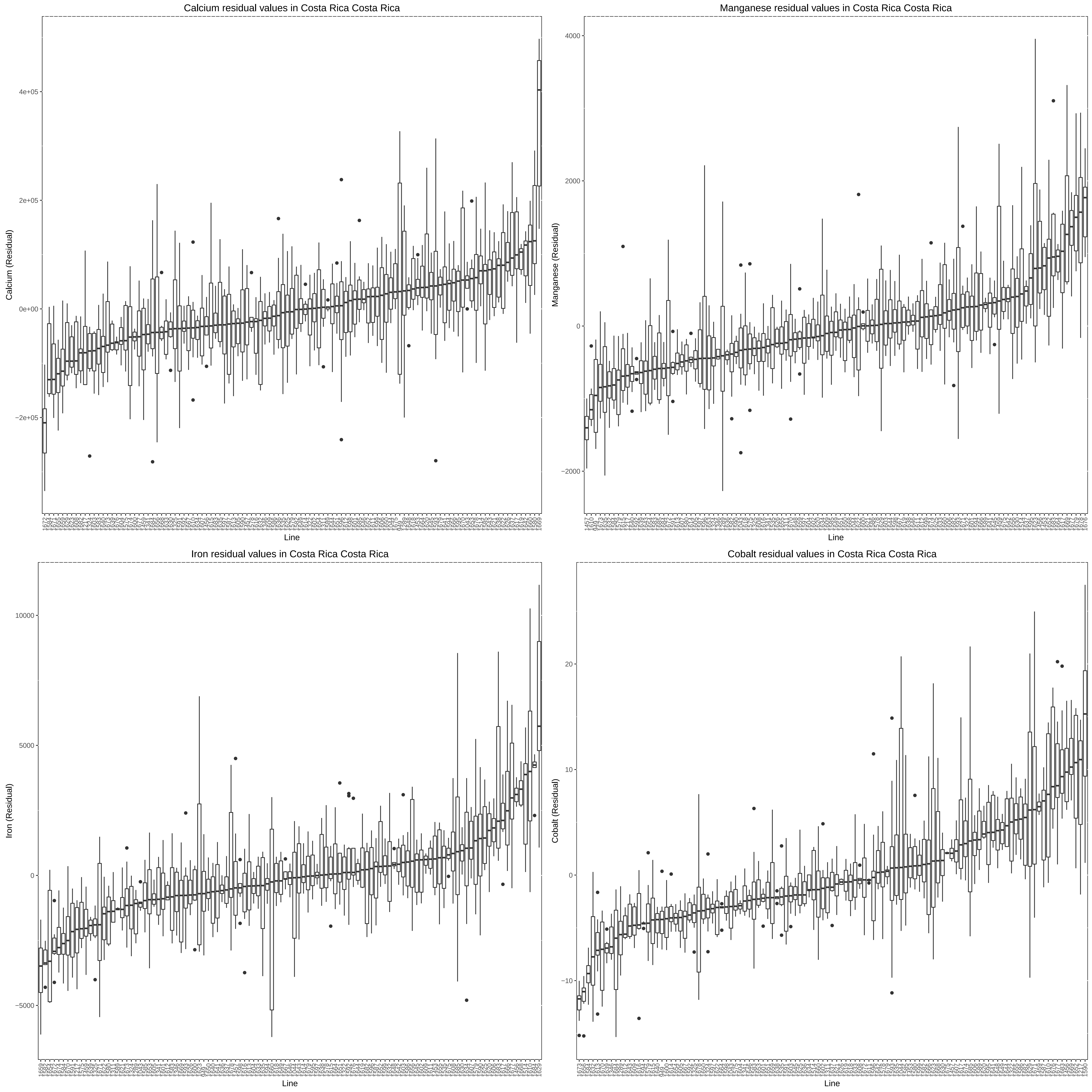

Calcium residual values in Costa Rica Costa Rica
Calcium (Residual)
4e+05
2e+05
0e+00
-2e+05
Line
Manganese residual values in Costa Rica Costa Rica
Manganese (Residual)
4000
2000
0
-2000
Line
Iron residual values in Costa Rica Costa Rica
Iron (Residual)
10000
5000
0
-5000
Line
Cobalt residual values in Costa Rica Costa Rica
Cobalt (Residual)
20
10
0
-10
Line

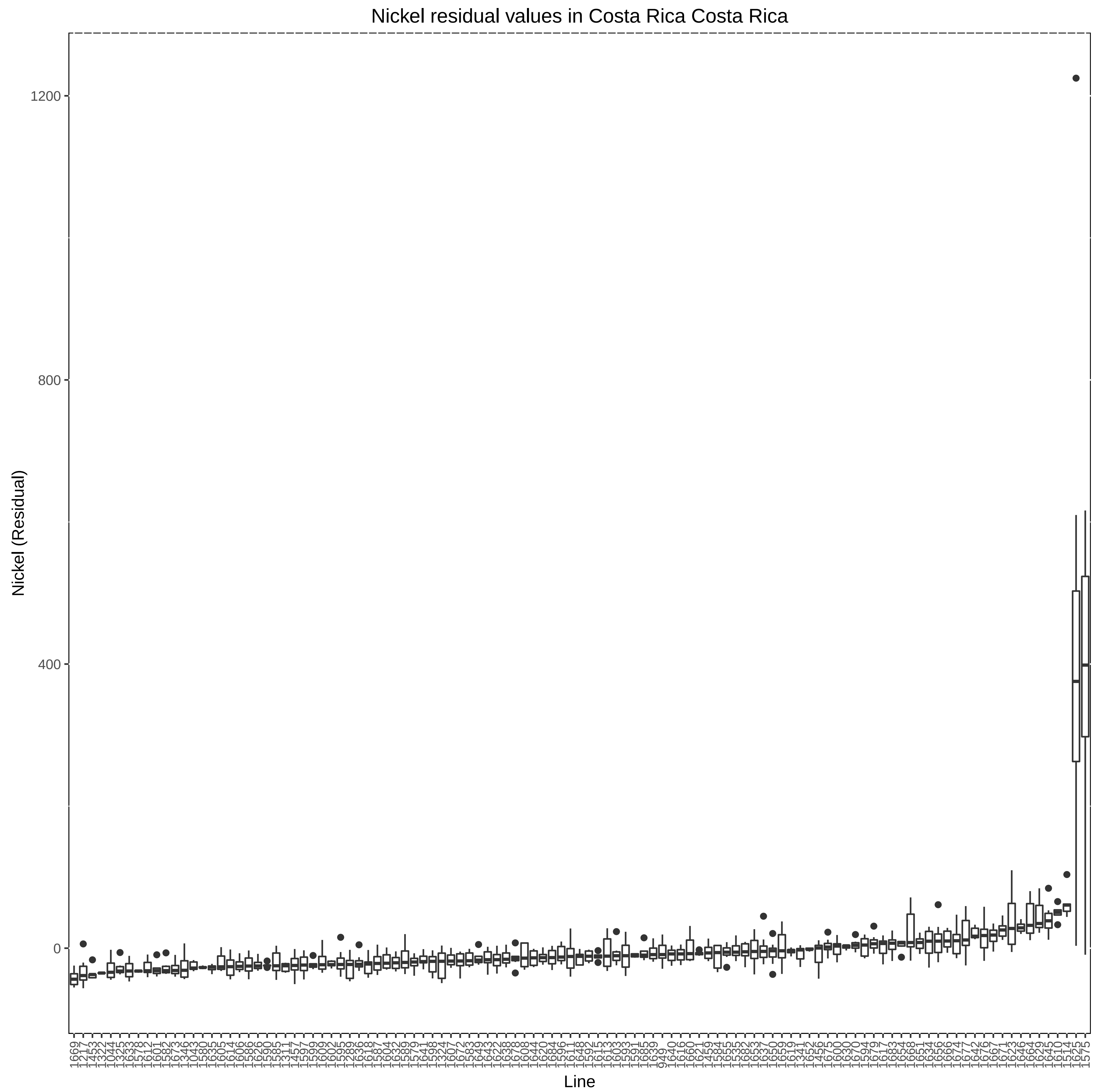
Nickel residual values in Costa Rica Costa Rica
Nickel (Residual)
1200
800
400
0
Line

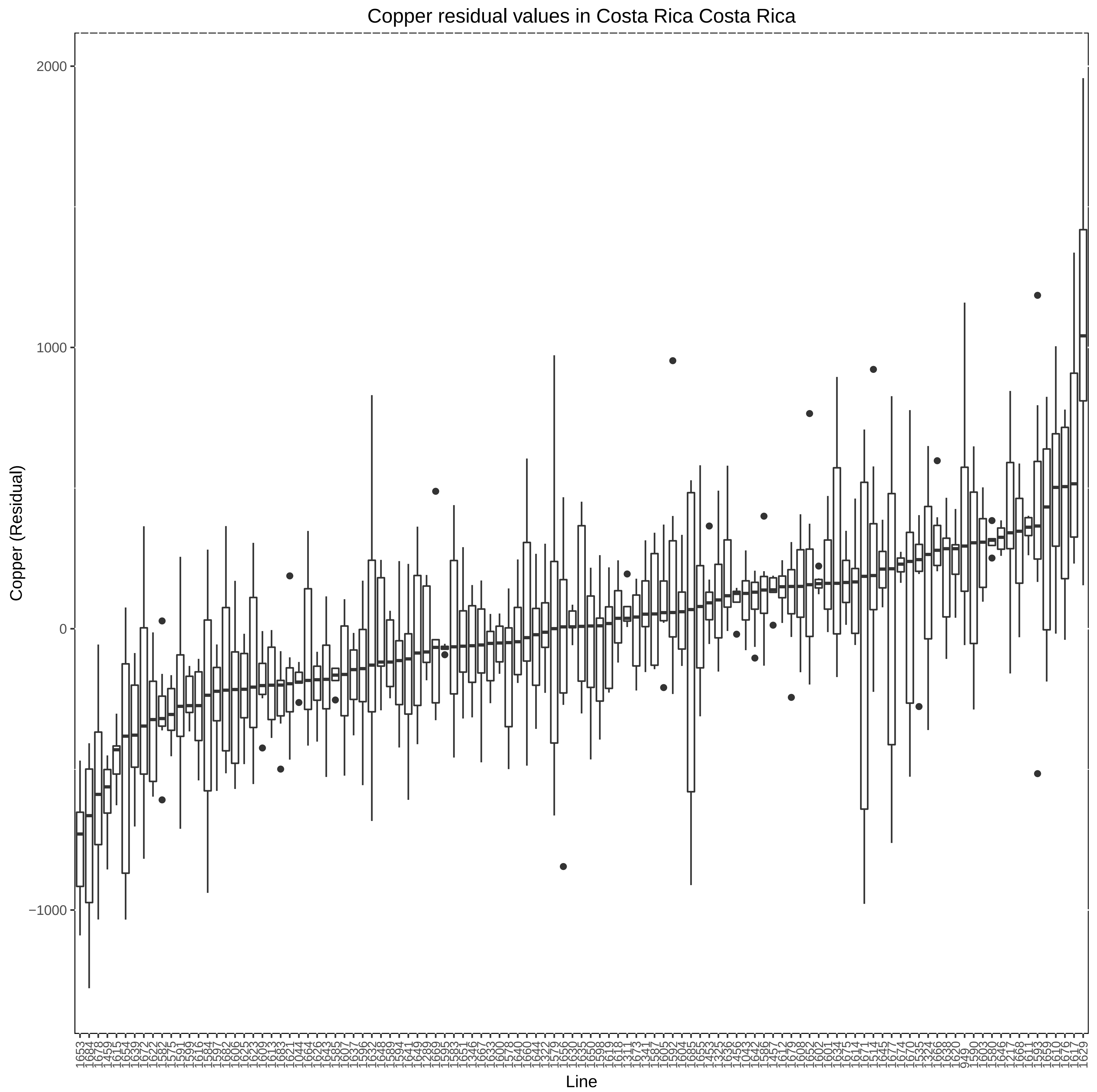
Copper residual values in Costa Rica Costa Rica
Copper (Residual)
2000
1000
0
-1000
Line

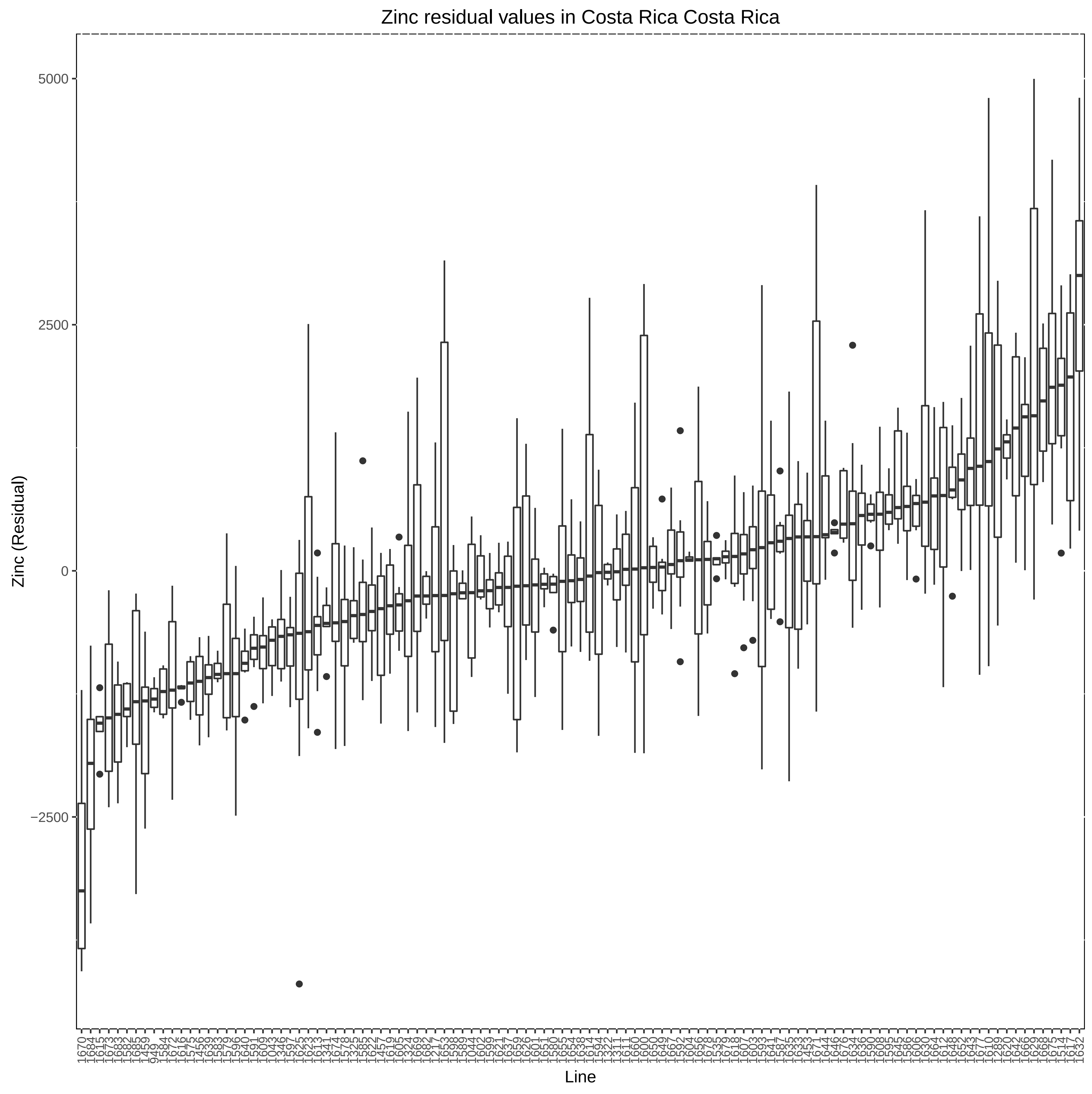
Zinc residual values in Costa Rica Costa Rica
Zinc (Residual)
5000
2500
0
-2500
Line

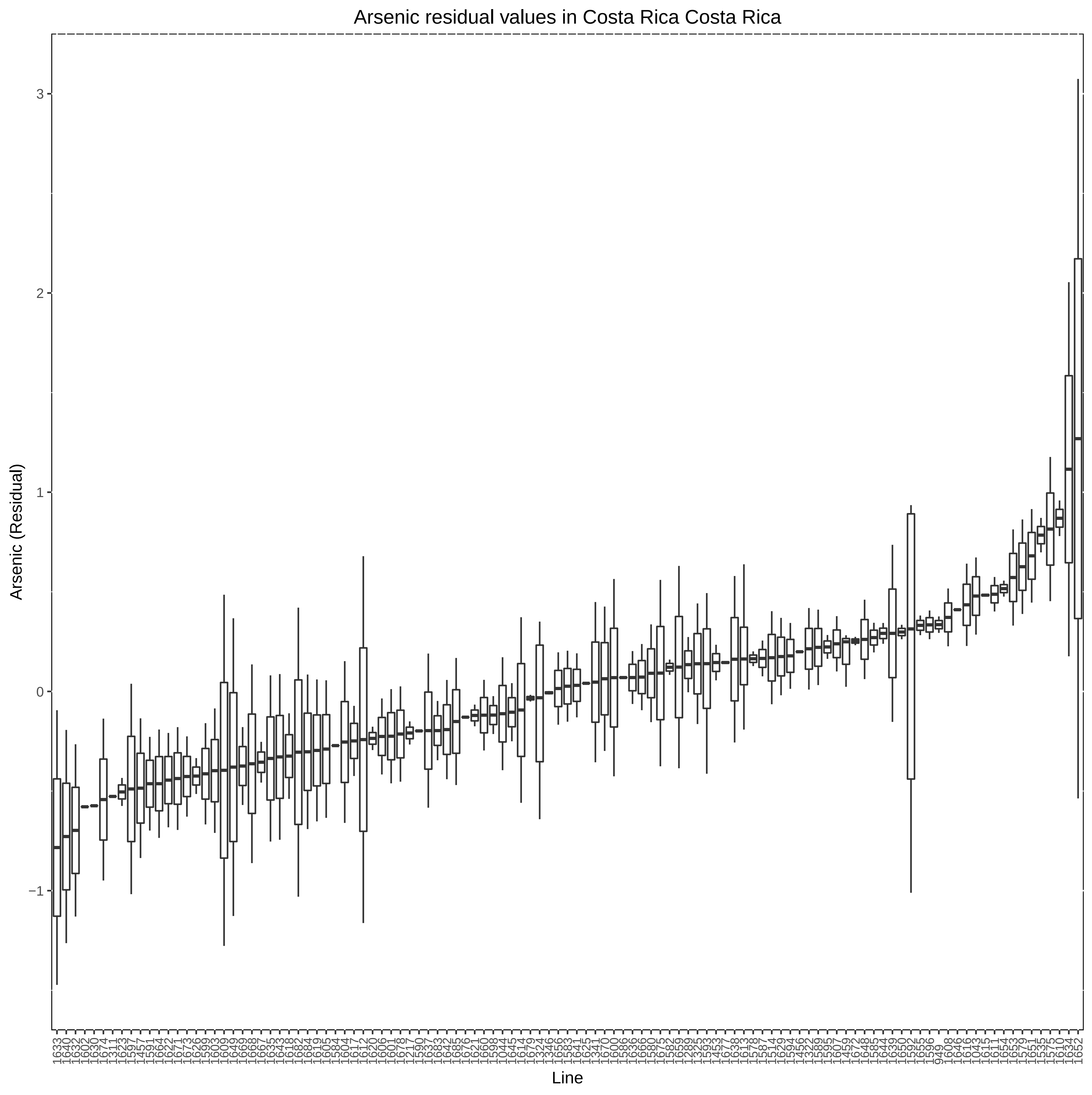
Arsenic residual values in Costa Rica Costa Rica
Arsenic (Residual)
3
2
1
0
-1
Line

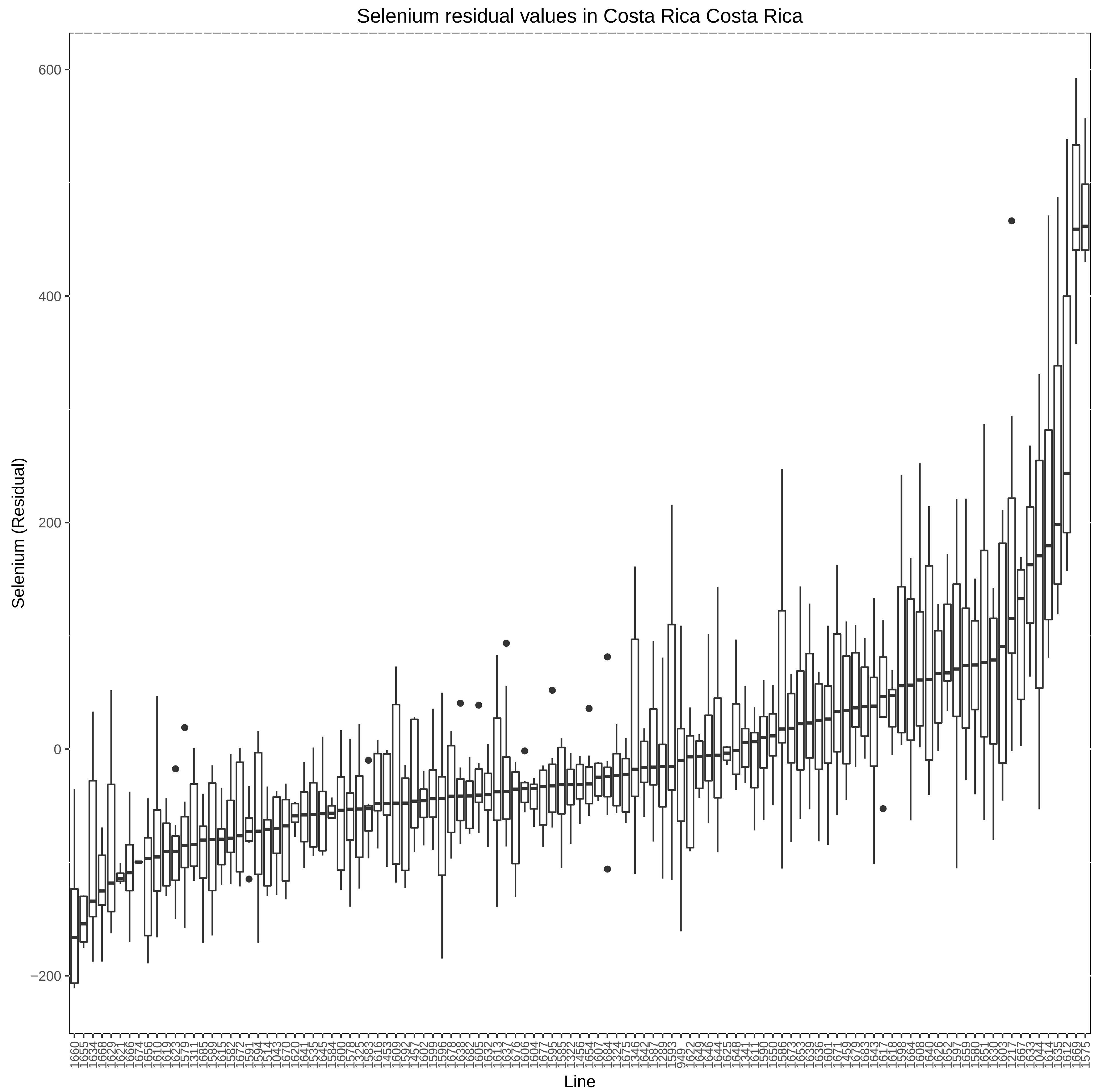

Selenium residual values in Costa Rica Costa Rica
Selenium (Residual)
600
400
200
0
-200
Line

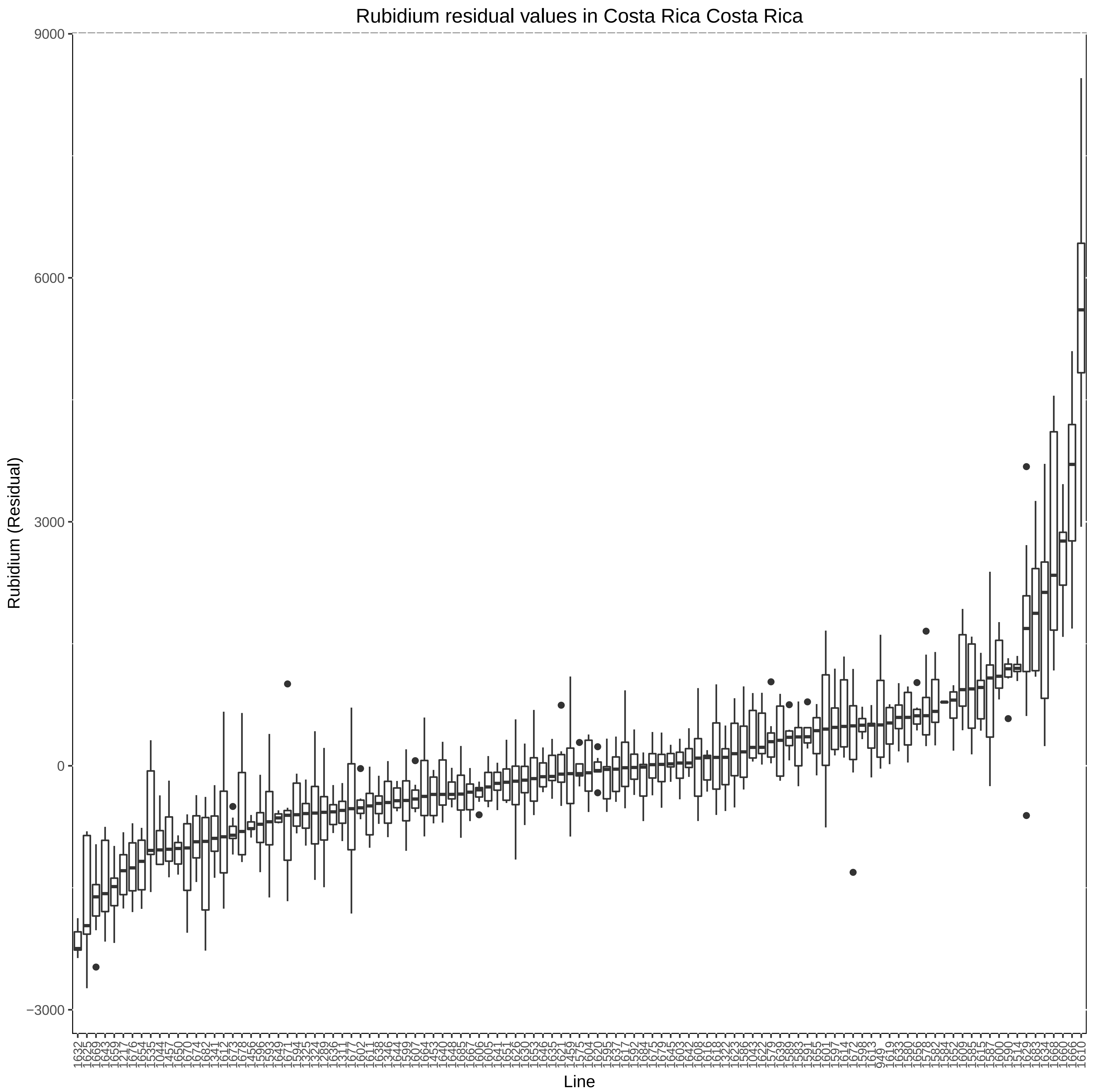

Rubidium residual values in Costa Rica Costa Rica
Rubidium (Residual)
9000
6000
3000
0
-3000
Line

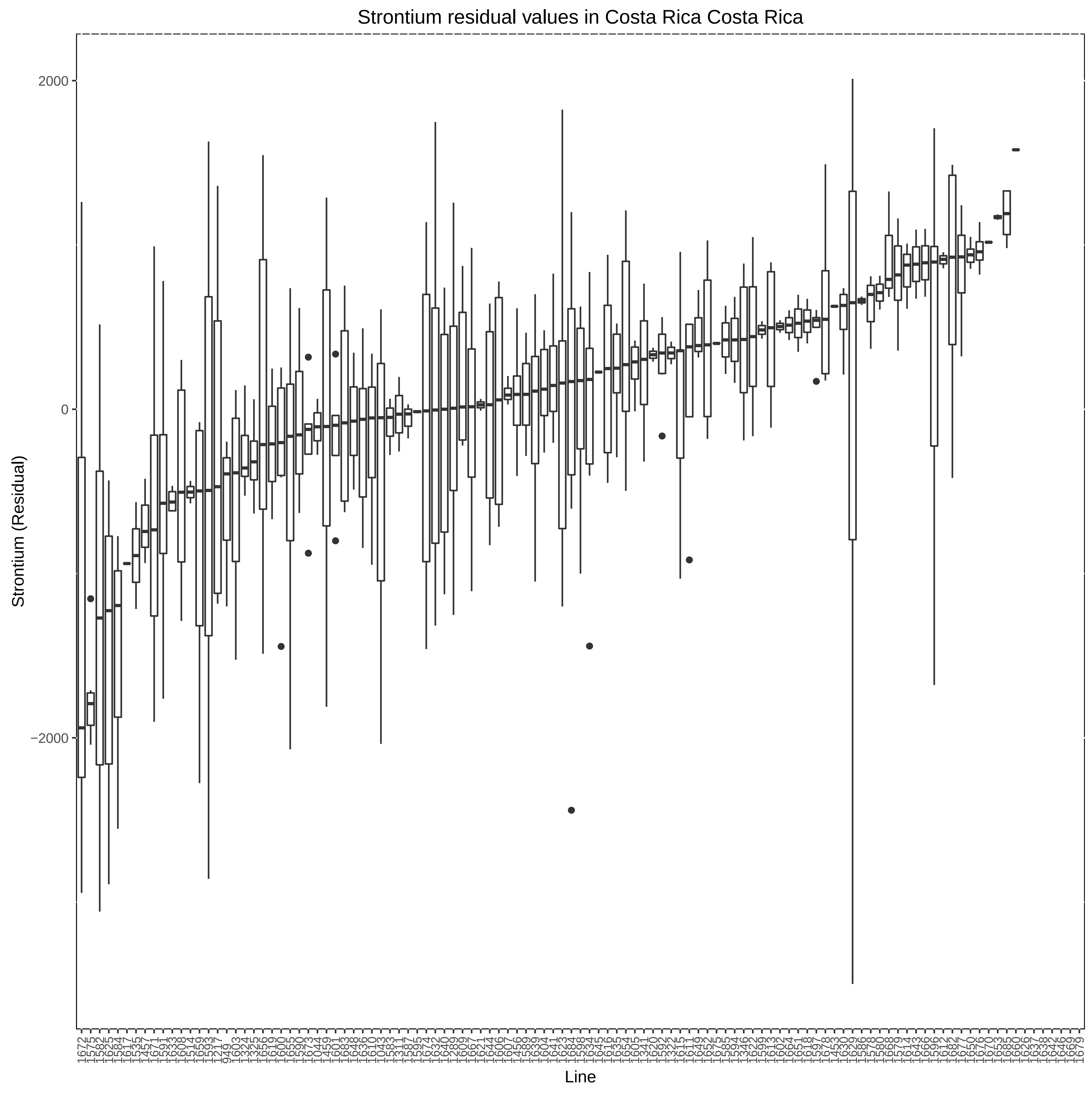

Strontium residual values in Costa Rica Costa Rica
Strontium (Residual)
2000
0
-2000
Line

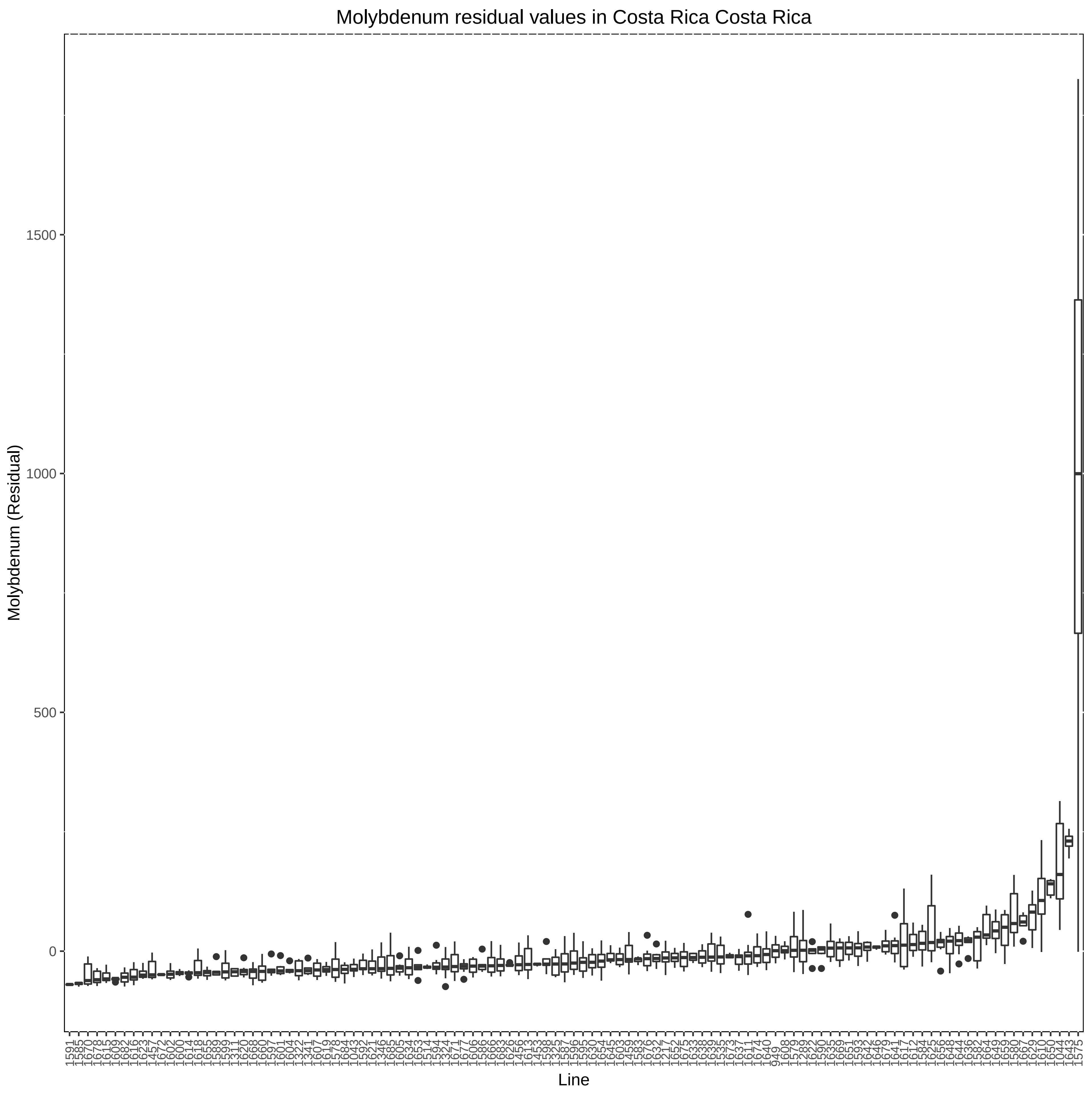

Molybdenum residual values in Costa Rica Costa Rica
Molybdenum (Residual)
1500
1000
500
0
Line

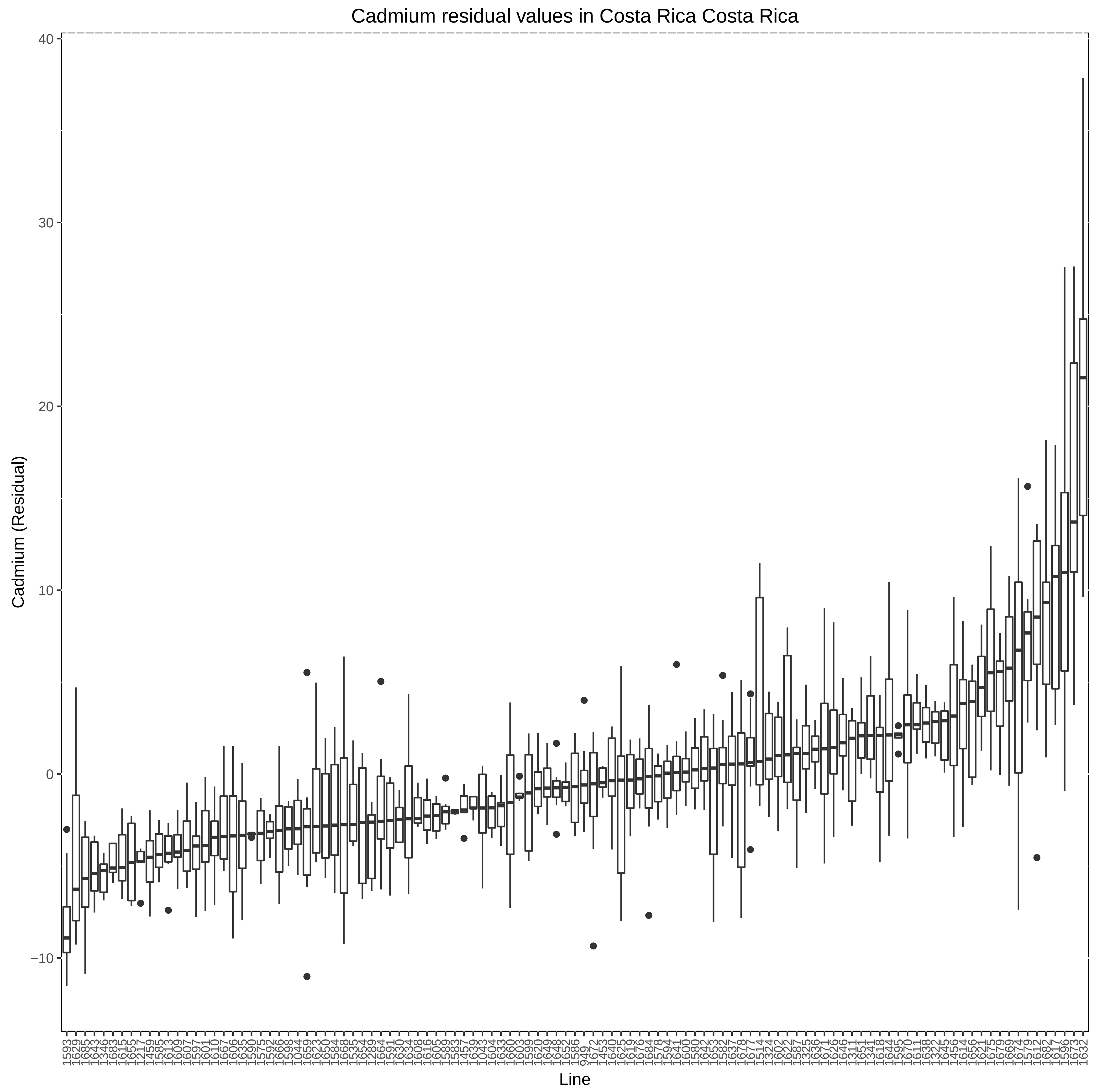
Cadmium residual values in Costa Rica Costa Rica
Cadmium (Residual)
40
30
20
10
0
−10
Line

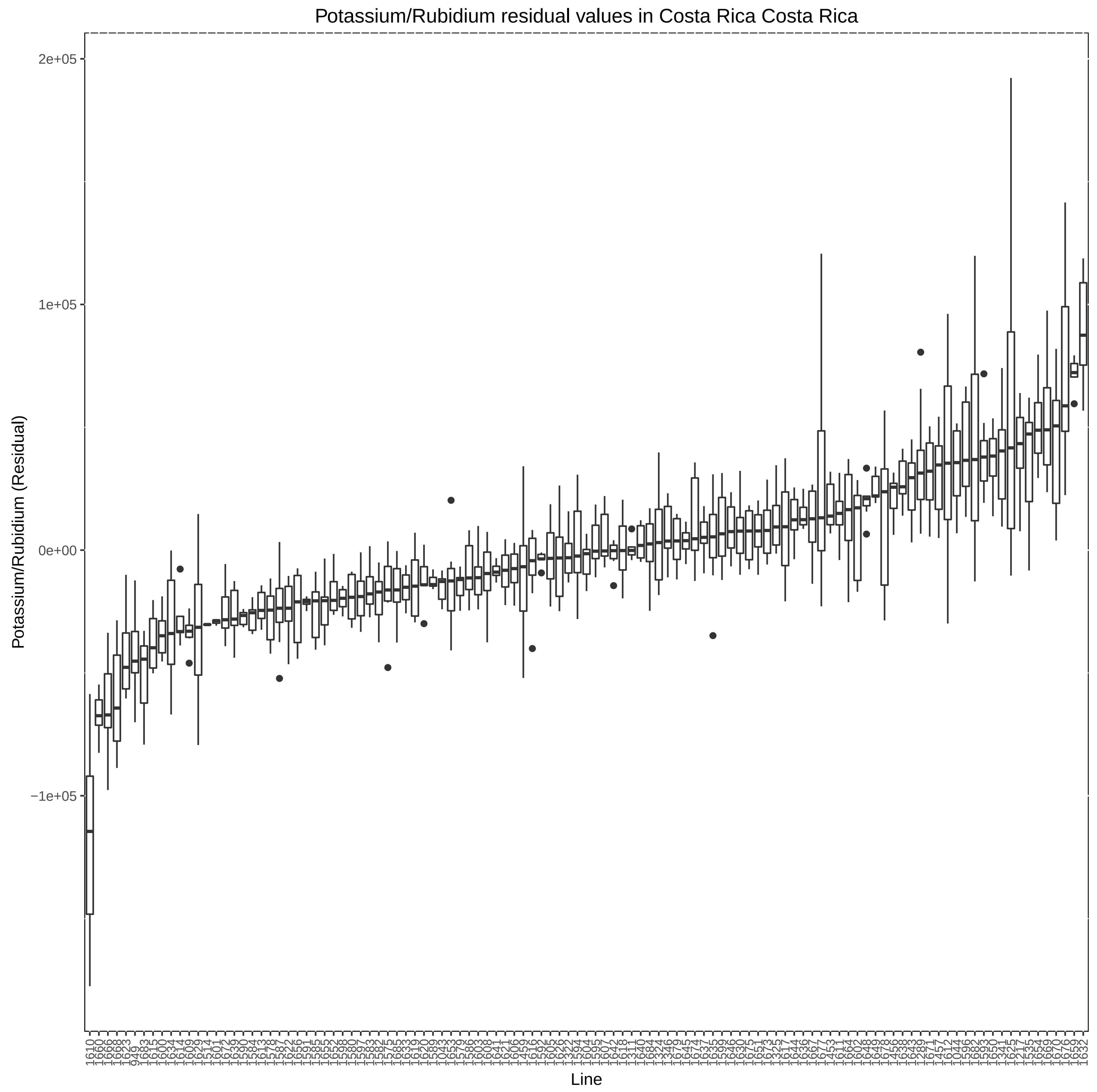
Potassium/Rubidium residual values in Costa Rica Costa Rica
Potassium/Rubidium (Residual)
2e+05
1e+05
0e+00
−1e+05
Line

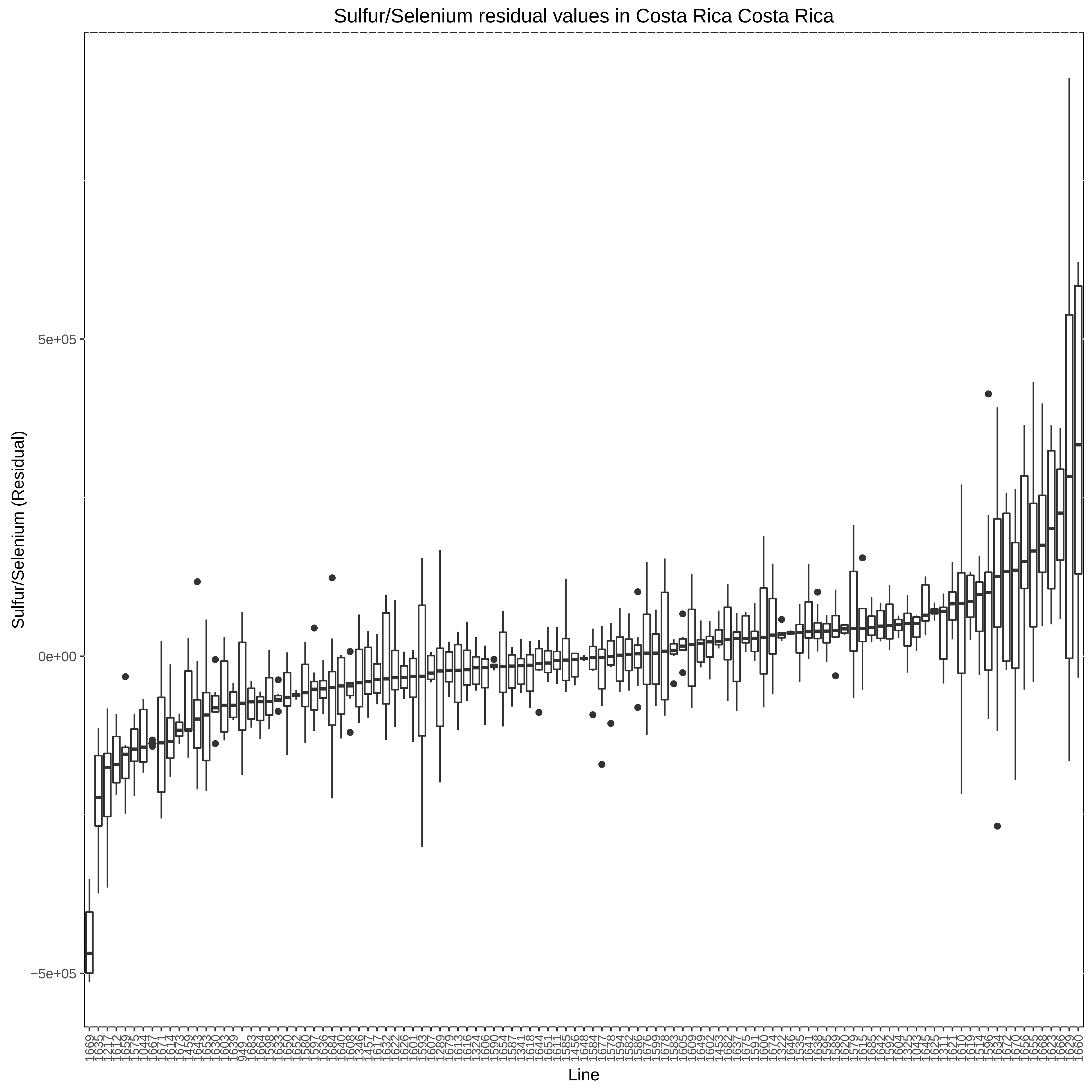
Sulfur/Selenium residual values in Costa Rica Costa Rica
Sulfur/Selenium (Residual)
5e+05
0e+00
−5e+05
Line

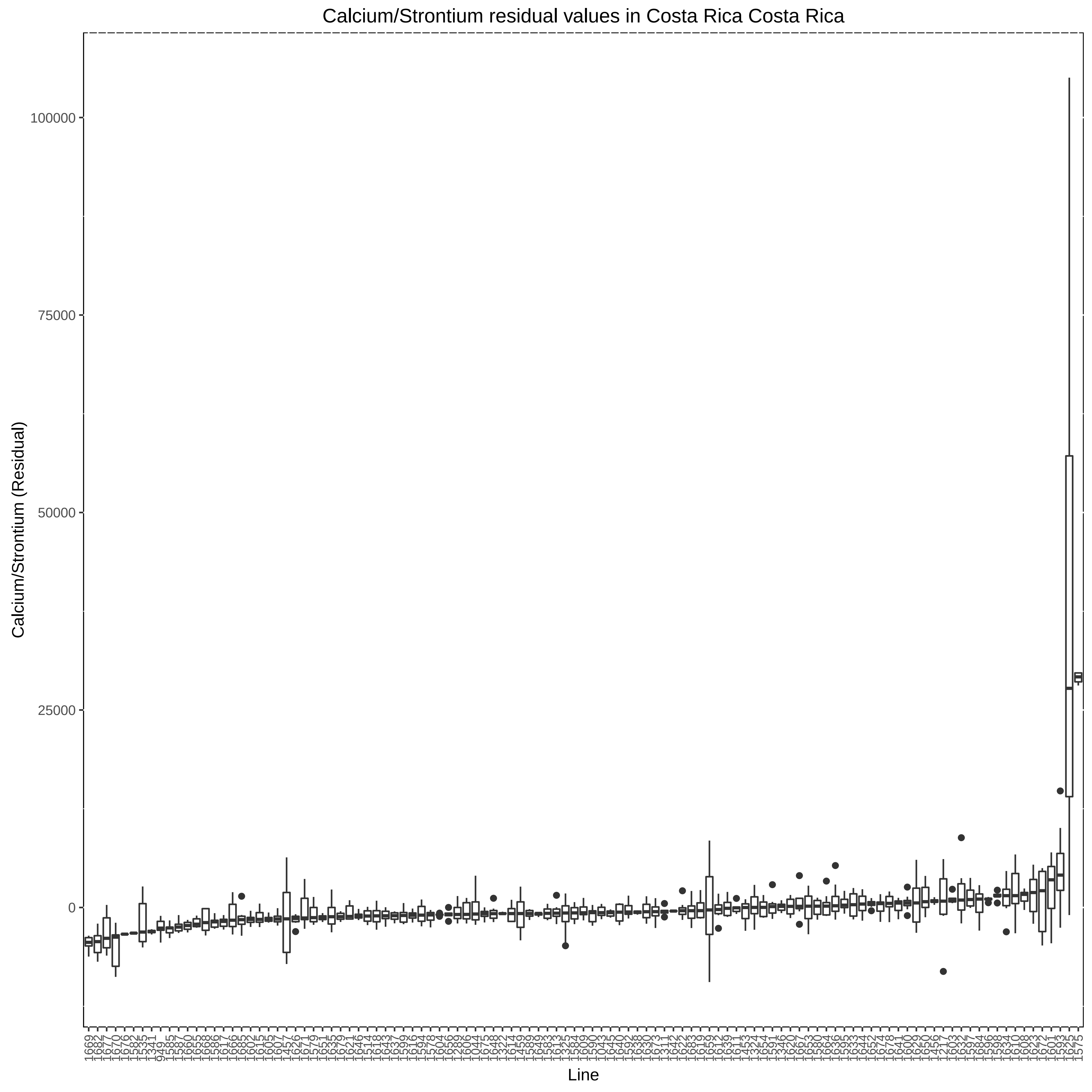
Calcium/Strontium residual values in Costa Rica Costa Rica
Calcium/Strontium (Residual)
100000
75000
50000
25000
0
Line
