# Supplementary material for: Genomewide association study of ionomic traits on diverse soybean populations from germplasm collections
Source: Plant Direct. 2018 Jan 15;2(1):e00033. doi: 10.1002/pld3.33 (PMC6508489; doi:10.1002/pld3.33)
Supplement: Supplementary file 4 [file PLD3-2-e00033-s004.pdf]

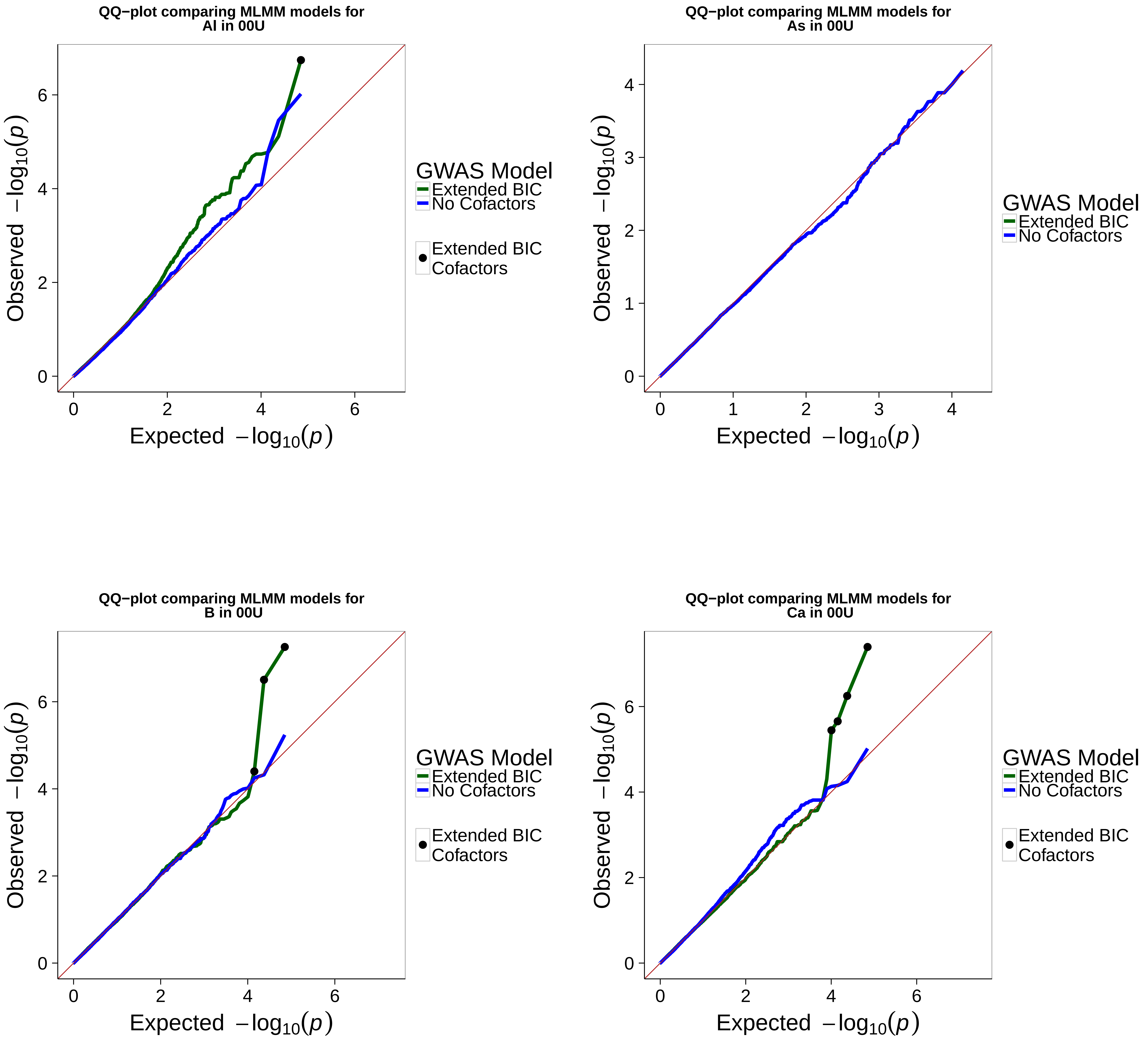
QQ−plot comparing MLMM models for
Al in 00U
Observed −log10(p)
Expected −log10(p)
GWAS Model
Extended BIC
No Cofactors
Extended BIC
Cofactors
QQ−plot comparing MLMM models for
As in 00U
Observed −log10(p)
Expected −log10(p)
GWAS Model
Extended BIC
No Cofactors
QQ−plot comparing MLMM models for
B in 00U
Observed −log10(p)
Expected −log10(p)
GWAS Model
Extended BIC
No Cofactors
Extended BIC
Cofactors
QQ−plot comparing MLMM models for
Ca in 00U
Observed −log10(p)
Expected −log10(p)
GWAS Model
Extended BIC
No Cofactors
Extended BIC
Cofactors

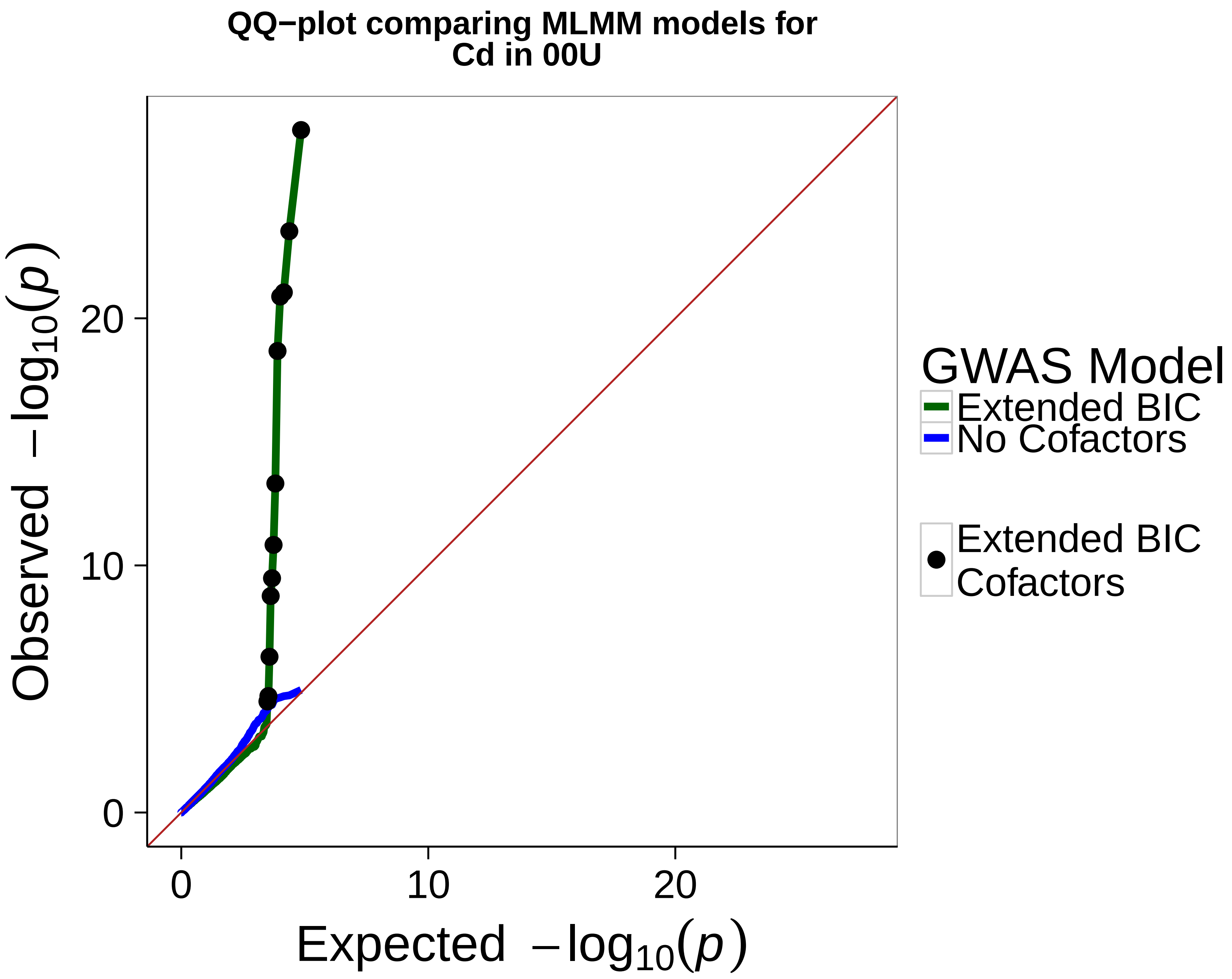
QQ−plot comparing MLMM models for
Cd in 00U
Observed −$\log_{10}(p)$
Expected −$\log_{10}(p)$
0
10
20
GWAS Model
Extended BIC
No Cofactors
Extended BIC
Cofactors

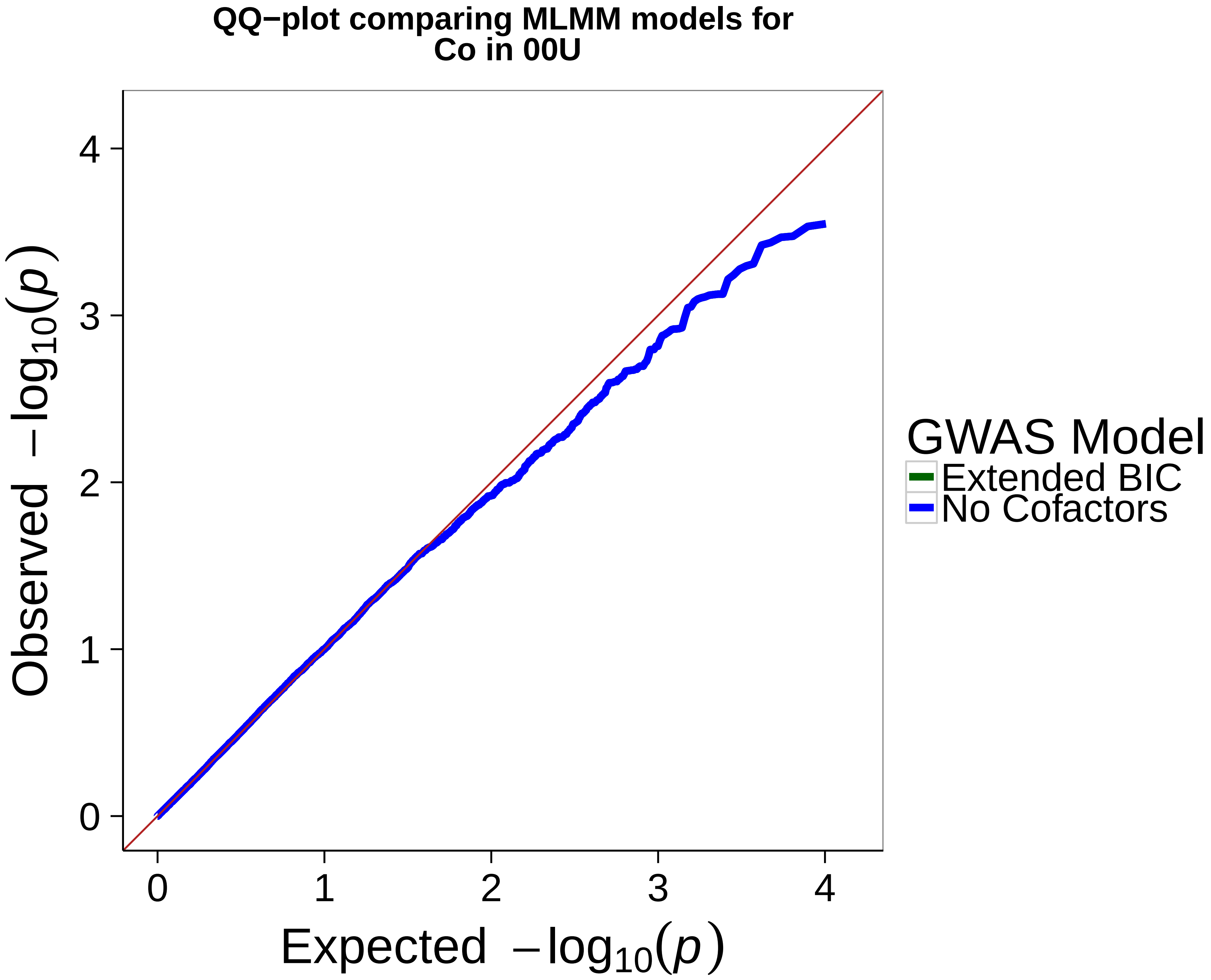
QQ−plot comparing MLMM models for
Co in 00U
Observed −$\log_{10}(p)$
Expected −$\log_{10}(p)$
0
1
2
3
4
GWAS Model
Extended BIC
No Cofactors

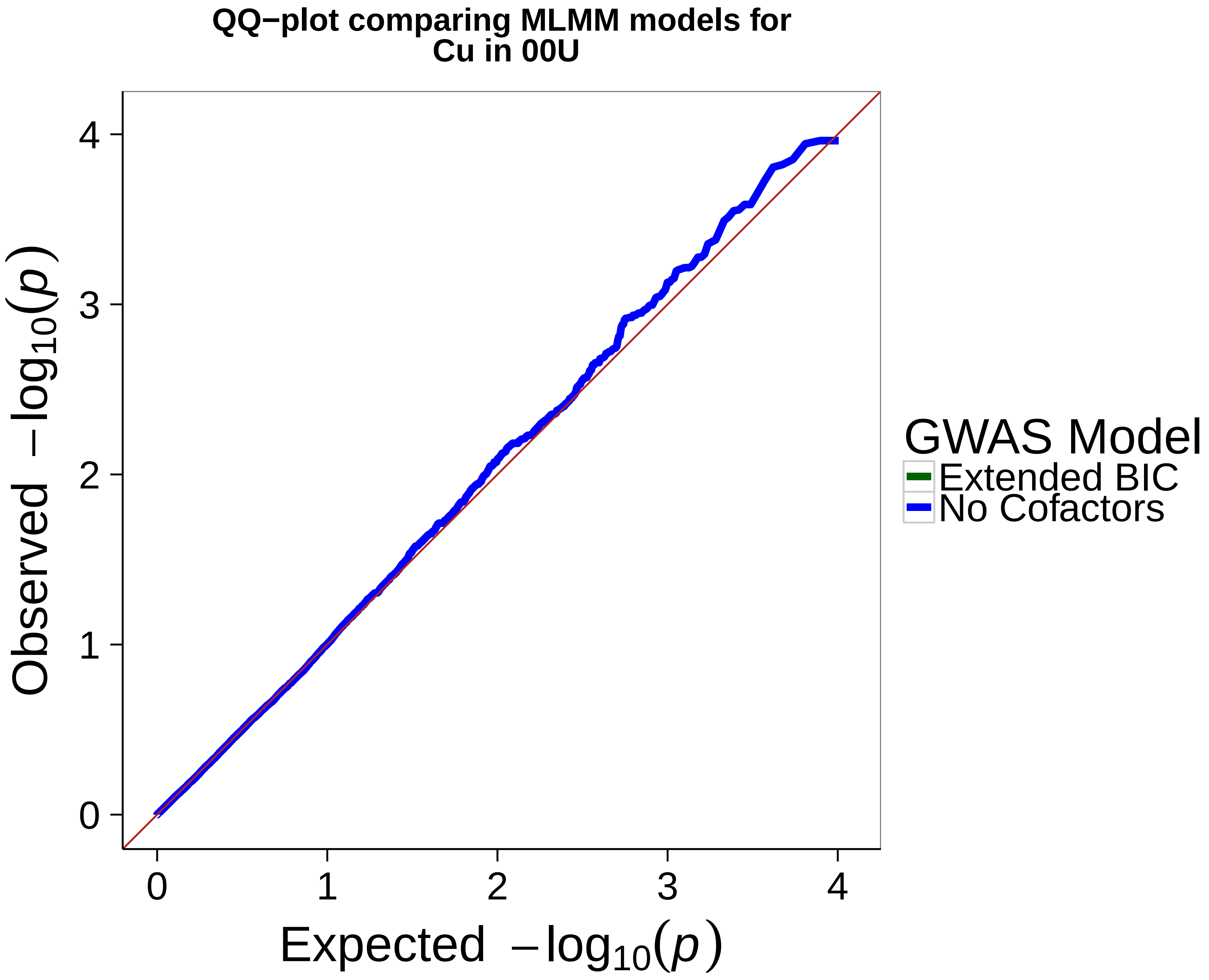
QQ−plot comparing MLMM models for
Cu in 00U
Observed −$\log_{10}(p)$
Expected −$\log_{10}(p)$
0
1
2
3
4
GWAS Model
Extended BIC
No Cofactors

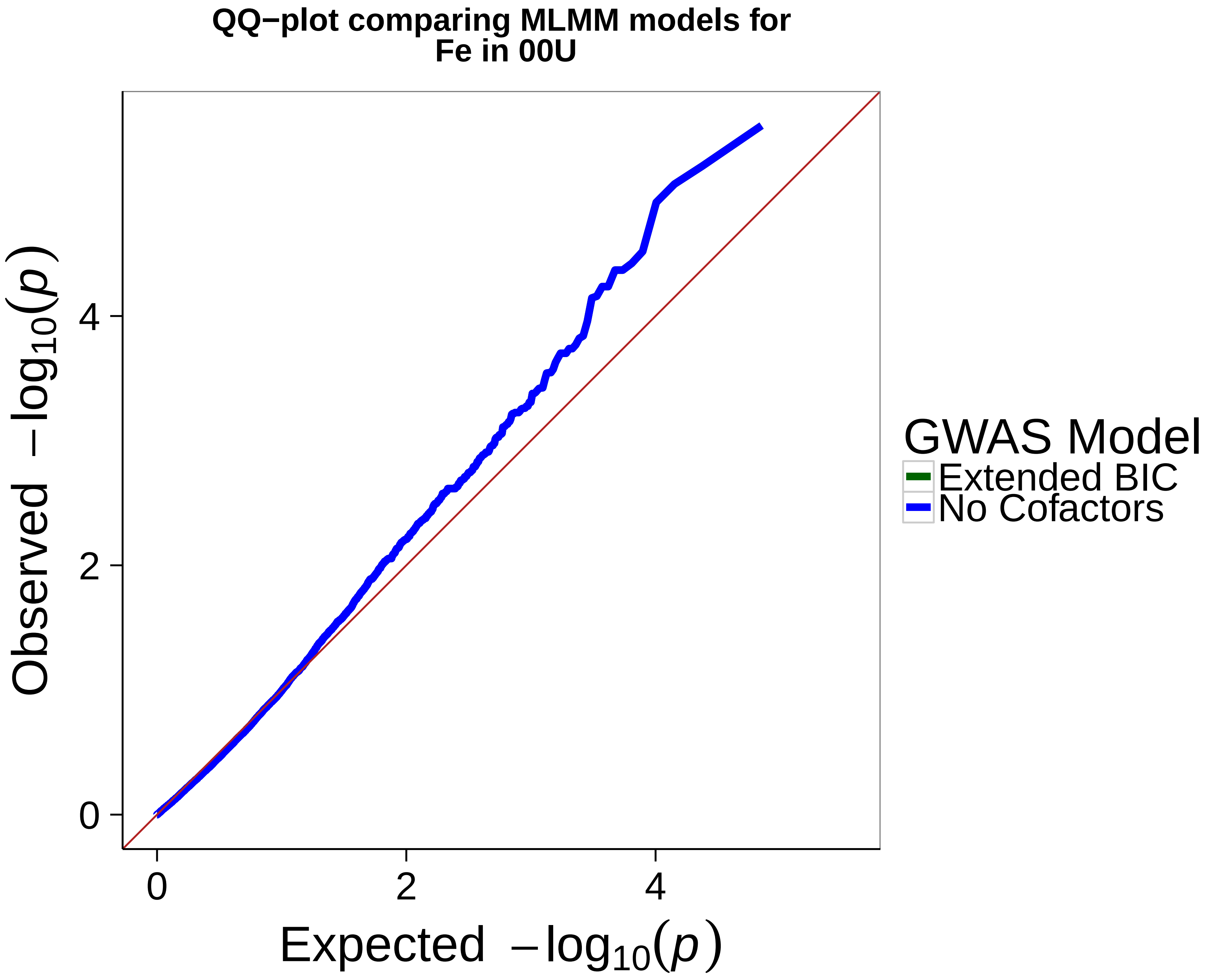
QQ−plot comparing MLMM models for
Fe in 00U
Observed −$\log_{10}(p)$
Expected −$\log_{10}(p)$
0
2
4
GWAS Model
Extended BIC
No Cofactors

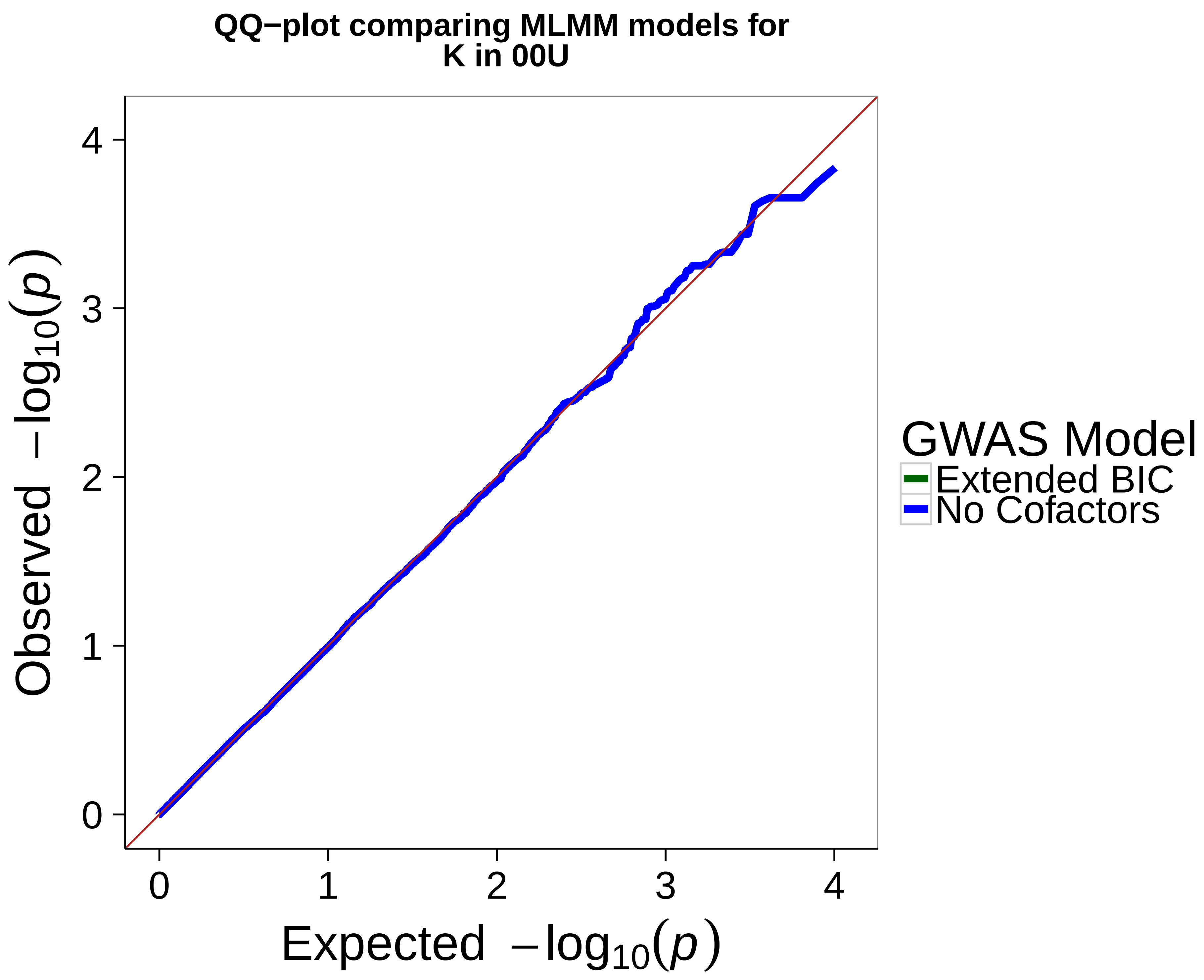
QQ−plot comparing MLMM models for
K in 00U
Observed −log10(p)
Expected −log10(p)
0
1
2
3
4
GWAS Model
Extended BIC
No Cofactors

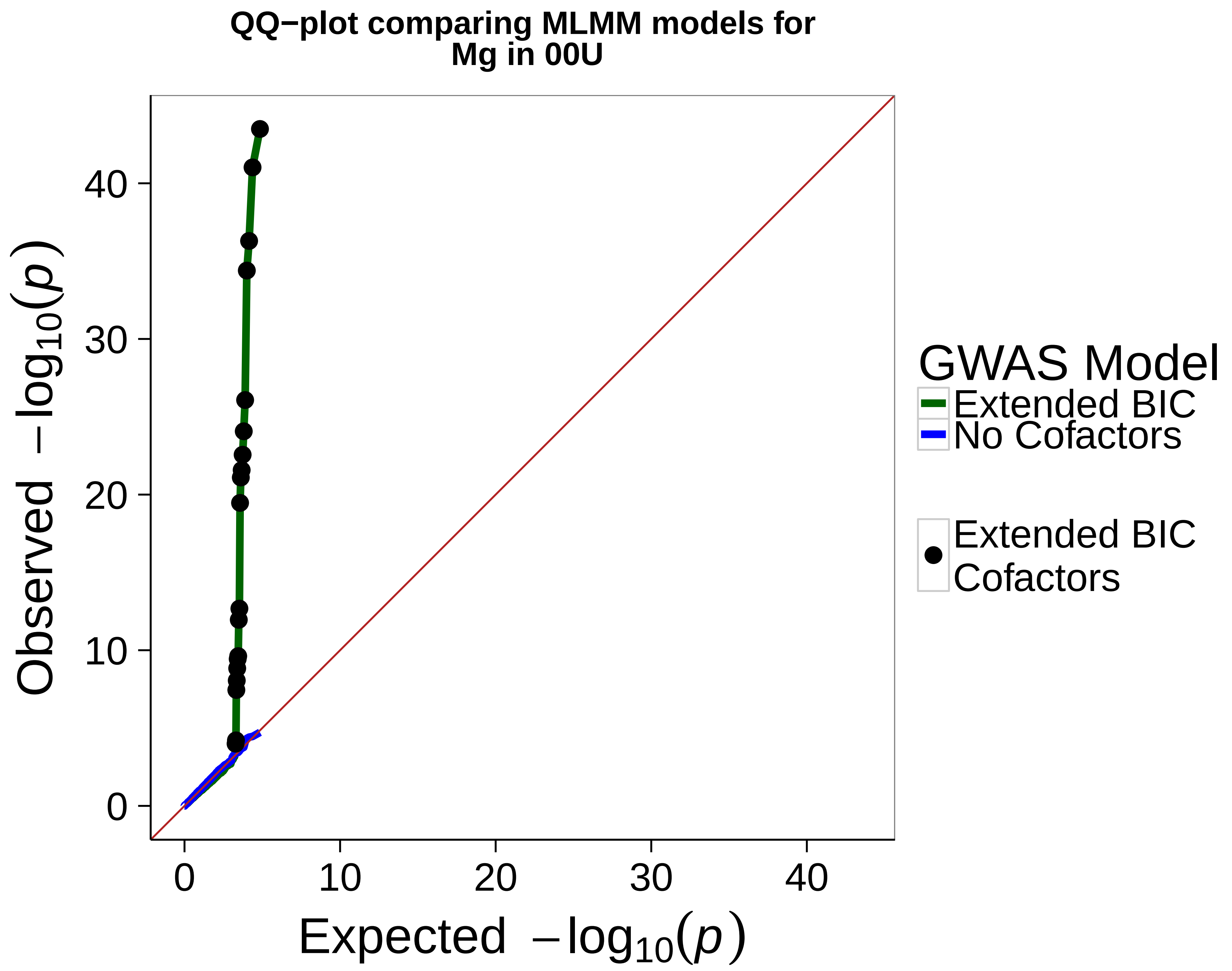
QQ−plot comparing MLMM models for
Mg in 00U
Observed −log10(p)
Expected −log10(p)
0
10
20
30
40
GWAS Model
Extended BIC
No Cofactors
Extended BIC
Cofactors

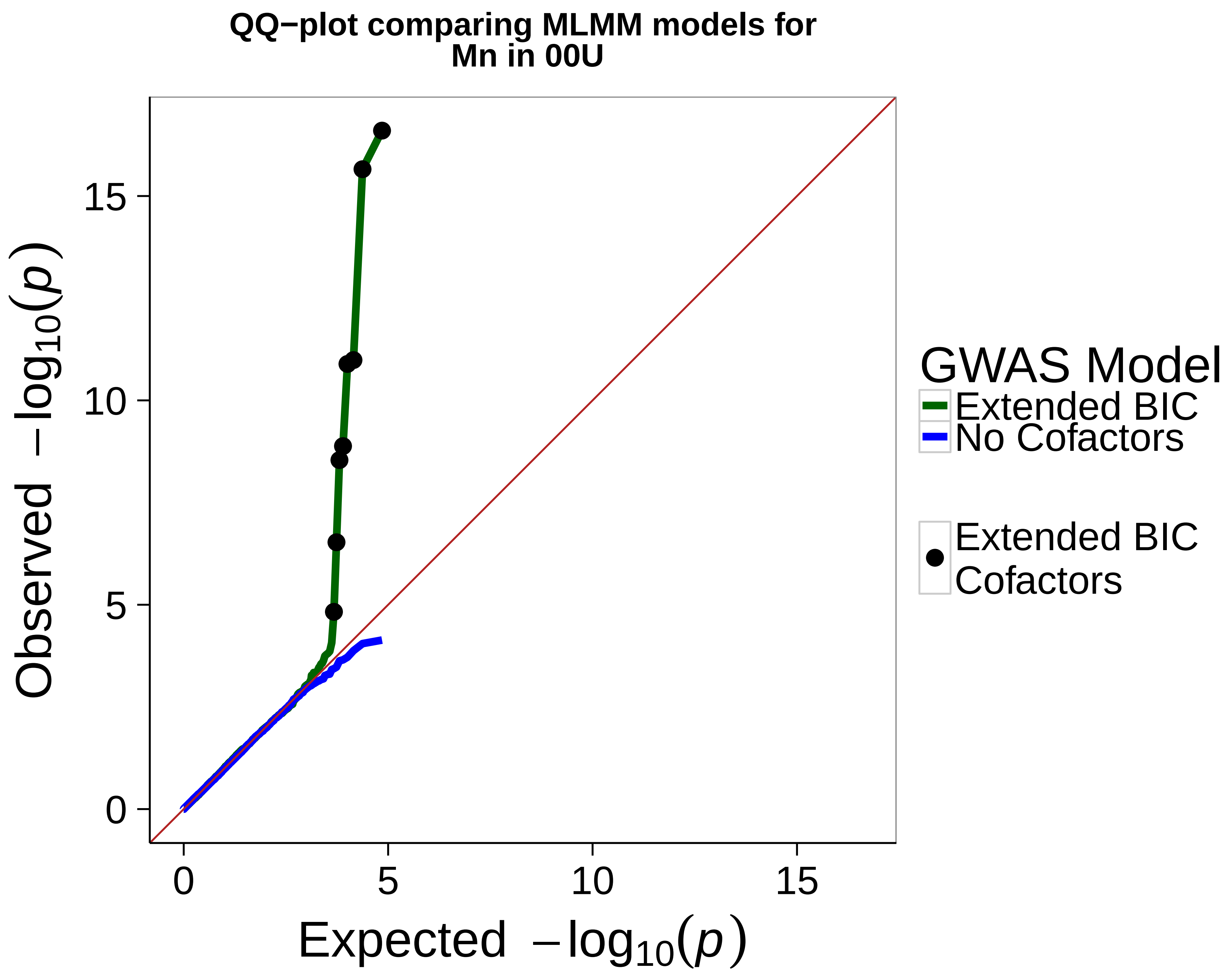
QQ−plot comparing MLMM models for
Mn in 00U
Observed −log10(p)
Expected −log10(p)
0
5
10
15
GWAS Model
Extended BIC
No Cofactors
Extended BIC
Cofactors

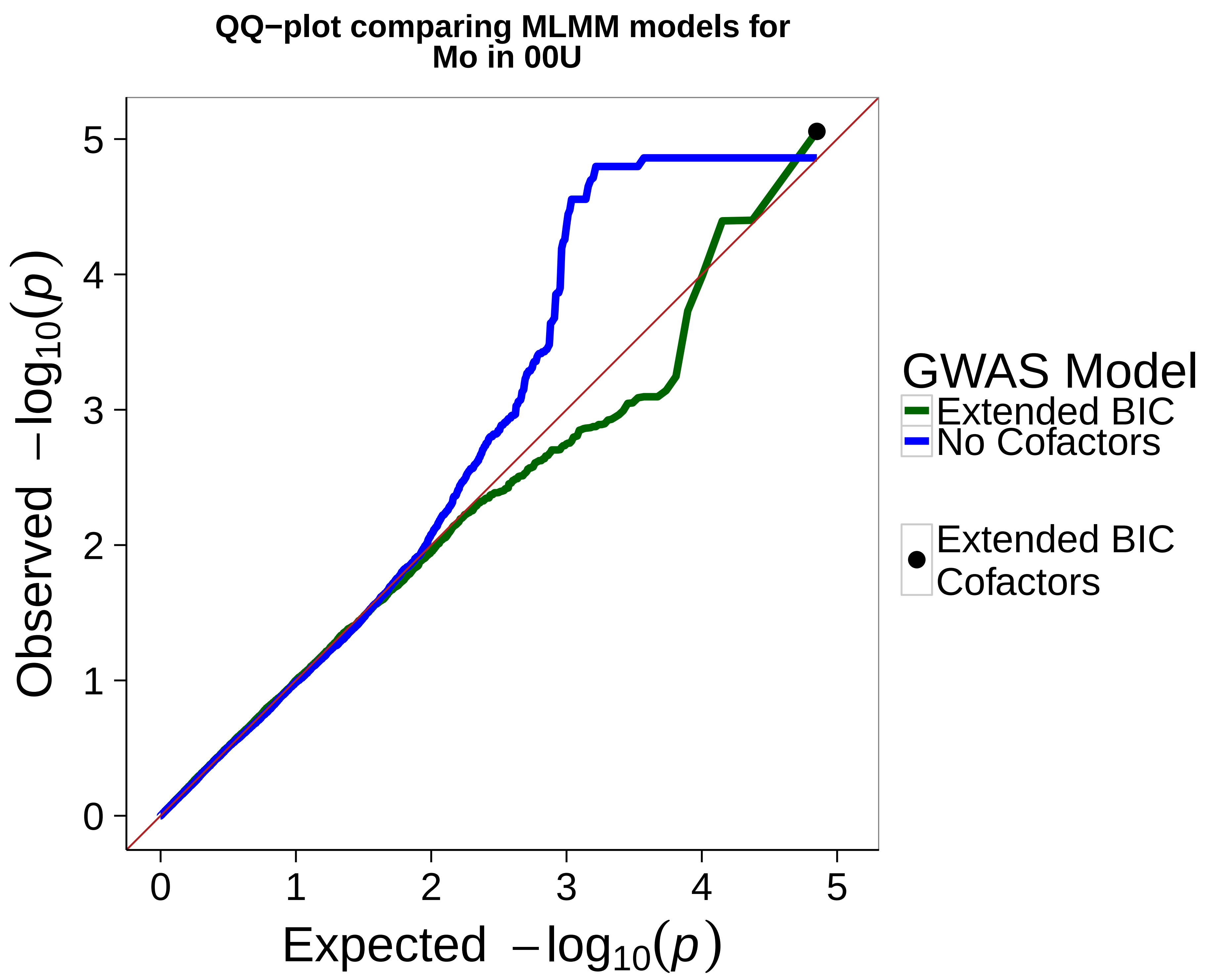
QQ−plot comparing MLMM models for
Mo in 00U
Observed −log10(p)
Expected −log10(p)
0
1
2
3
4
5
GWAS Model
Extended BIC
No Cofactors
Extended BIC
Cofactors

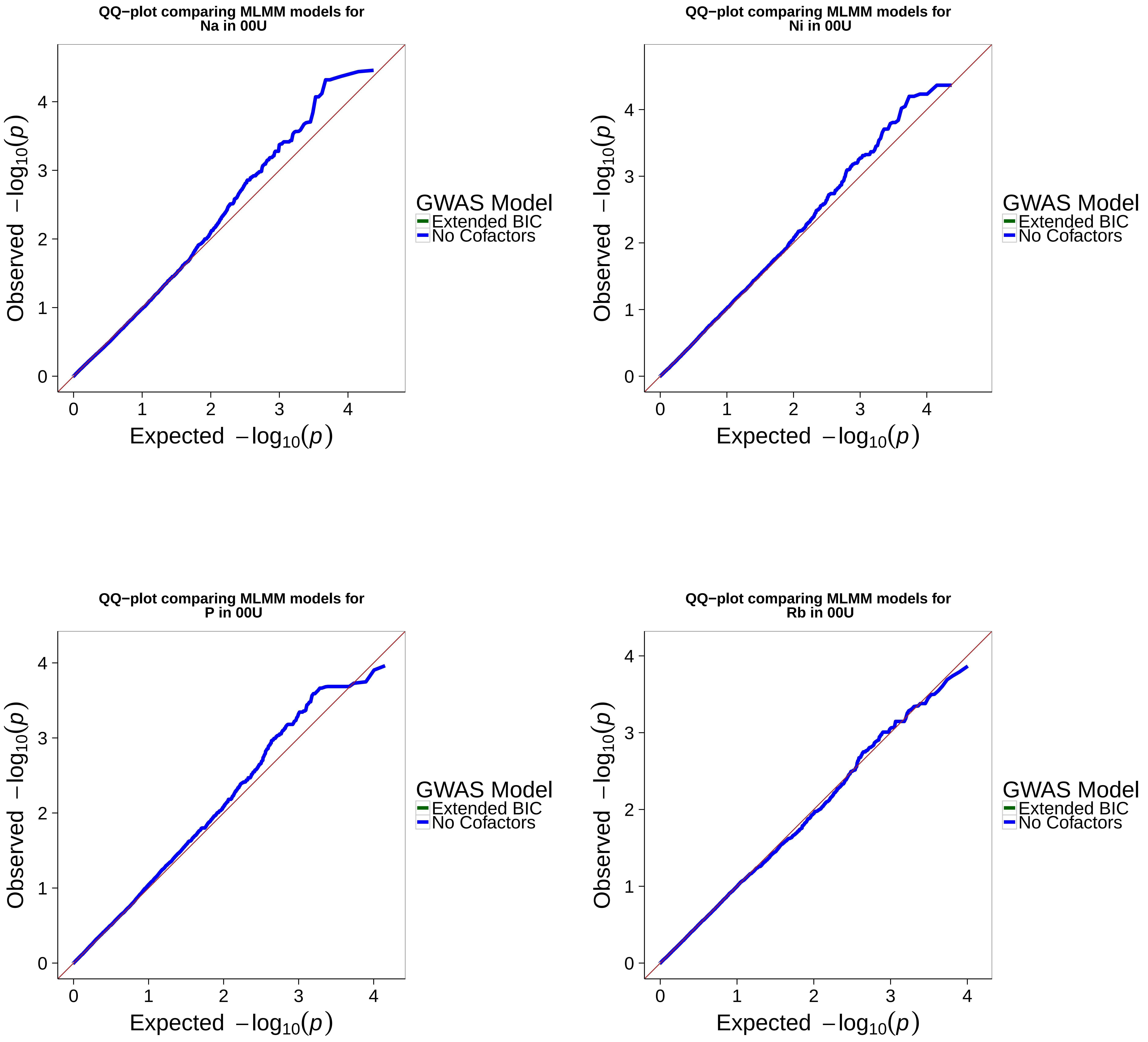
QQ−plot comparing MLMM models for Na in 00U
Observed −log10(p)
Expected −log10(p)
GWAS Model
Extended BIC
No Cofactors
QQ−plot comparing MLMM models for Ni in 00U
Observed −log10(p)
Expected −log10(p)
GWAS Model
Extended BIC
No Cofactors
QQ−plot comparing MLMM models for P in 00U
Observed −log10(p)
Expected −log10(p)
GWAS Model
Extended BIC
No Cofactors
QQ−plot comparing MLMM models for Rb in 00U
Observed −log10(p)
Expected −log10(p)
GWAS Model
Extended BIC
No Cofactors

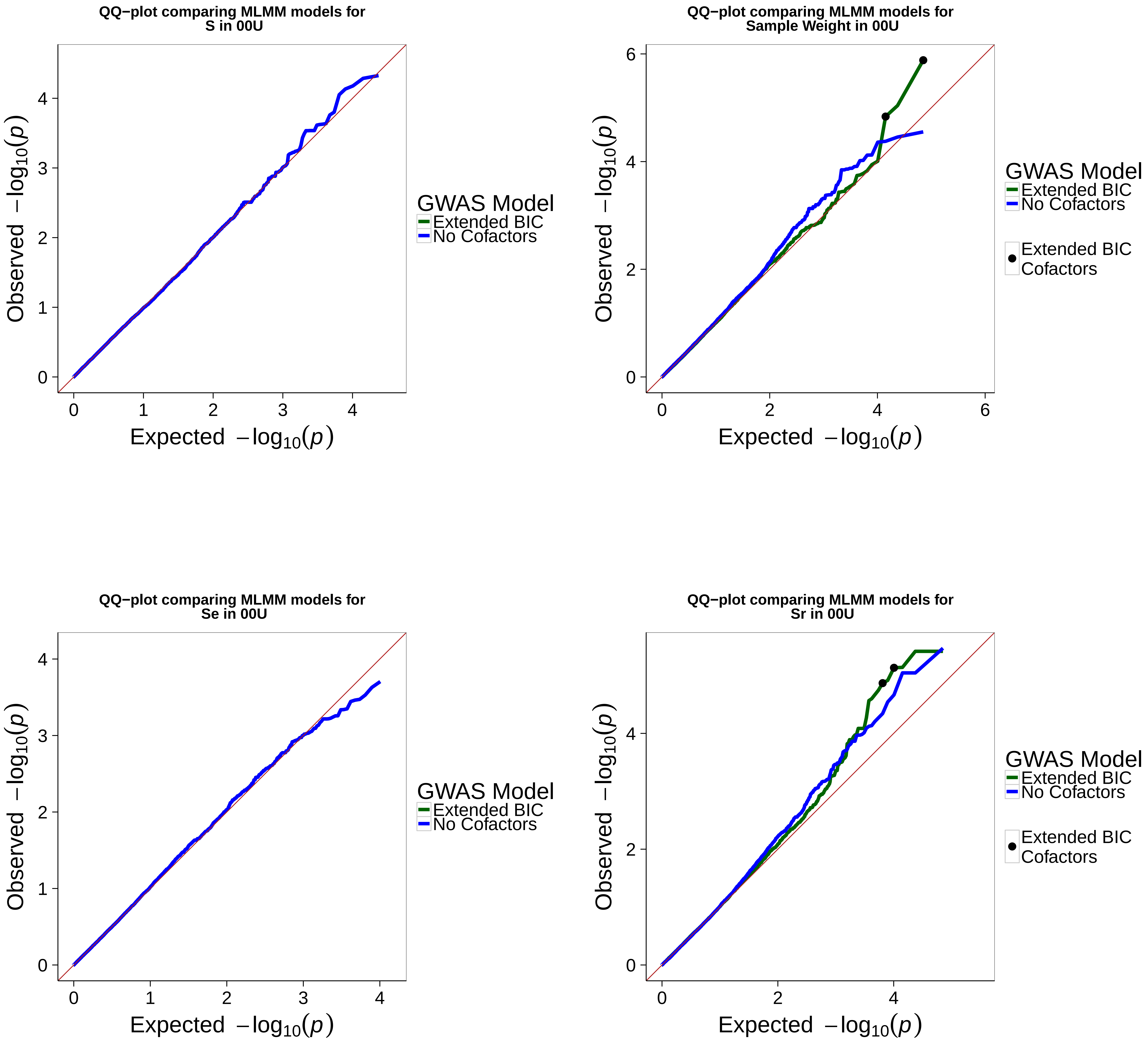
QQ−plot comparing MLMM models for
S in 00U
QQ−plot comparing MLMM models for
Sample Weight in 00U
QQ−plot comparing MLMM models for
Se in 00U
QQ−plot comparing MLMM models for
Sr in 00U
Observed $-\log_{10}(p)$
Expected $-\log_{10}(p)$
GWAS Model
Extended BIC
No Cofactors
Extended BIC
Cofactors
0
1
2
3
4
5
6

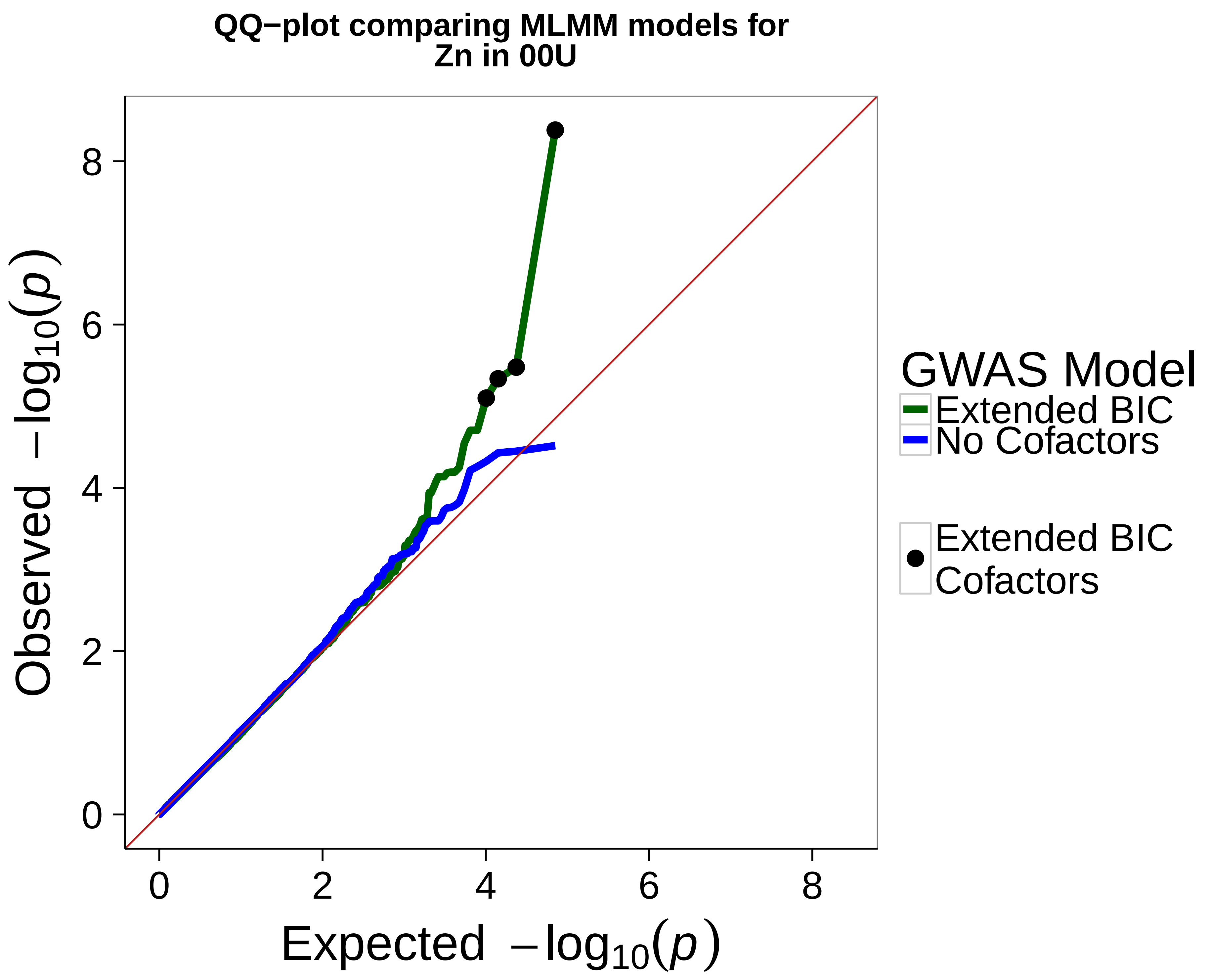
QQ−plot comparing MLMM models for
Zn in 00U
Observed −log10(p)
Expected −log10(p)
GWAS Model
Extended BIC
No Cofactors
Extended BIC
Cofactors

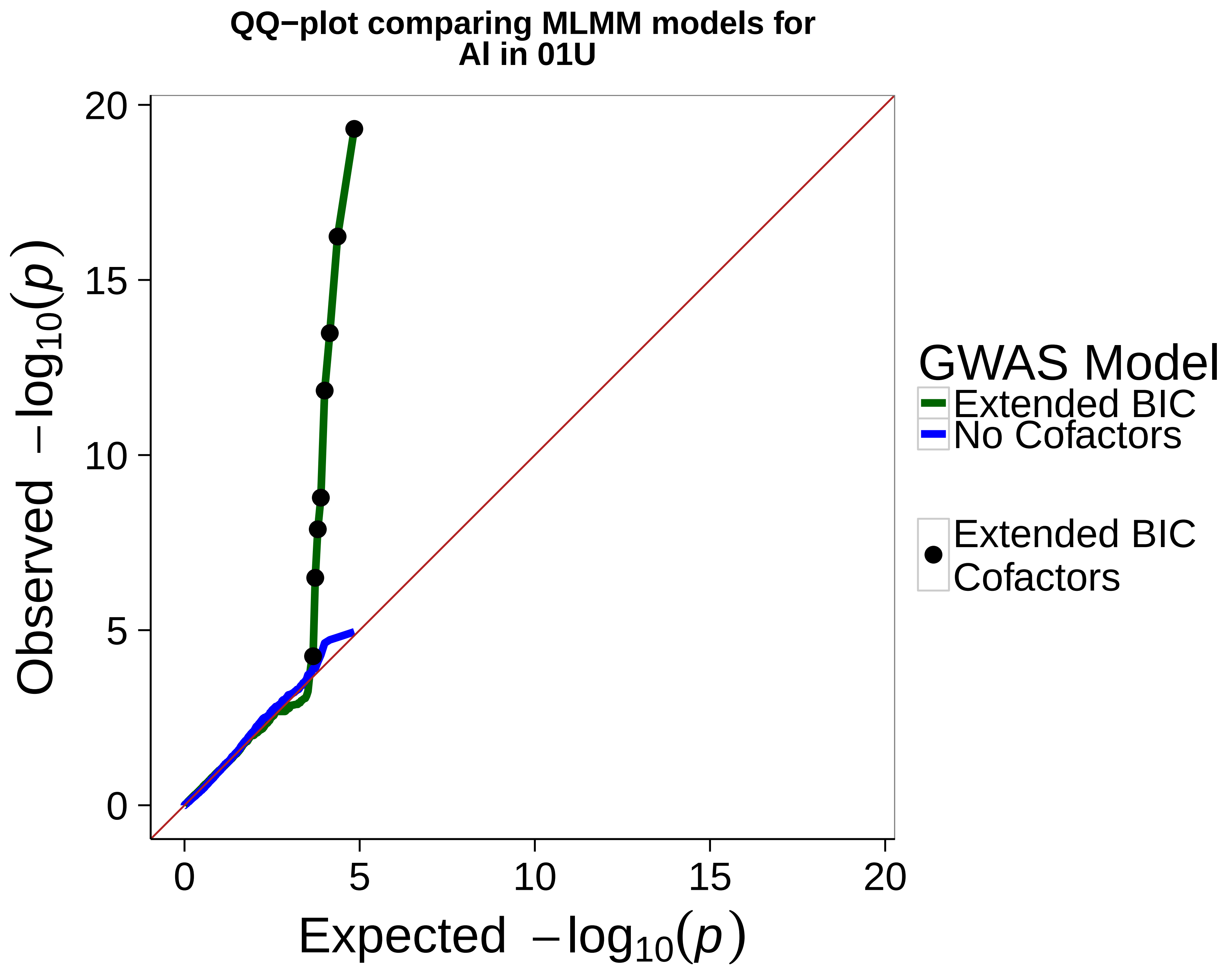
QQ−plot comparing MLMM models for
Al in 01U
Observed −log10(p)
Expected −log10(p)
GWAS Model
Extended BIC
No Cofactors
Extended BIC
Cofactors

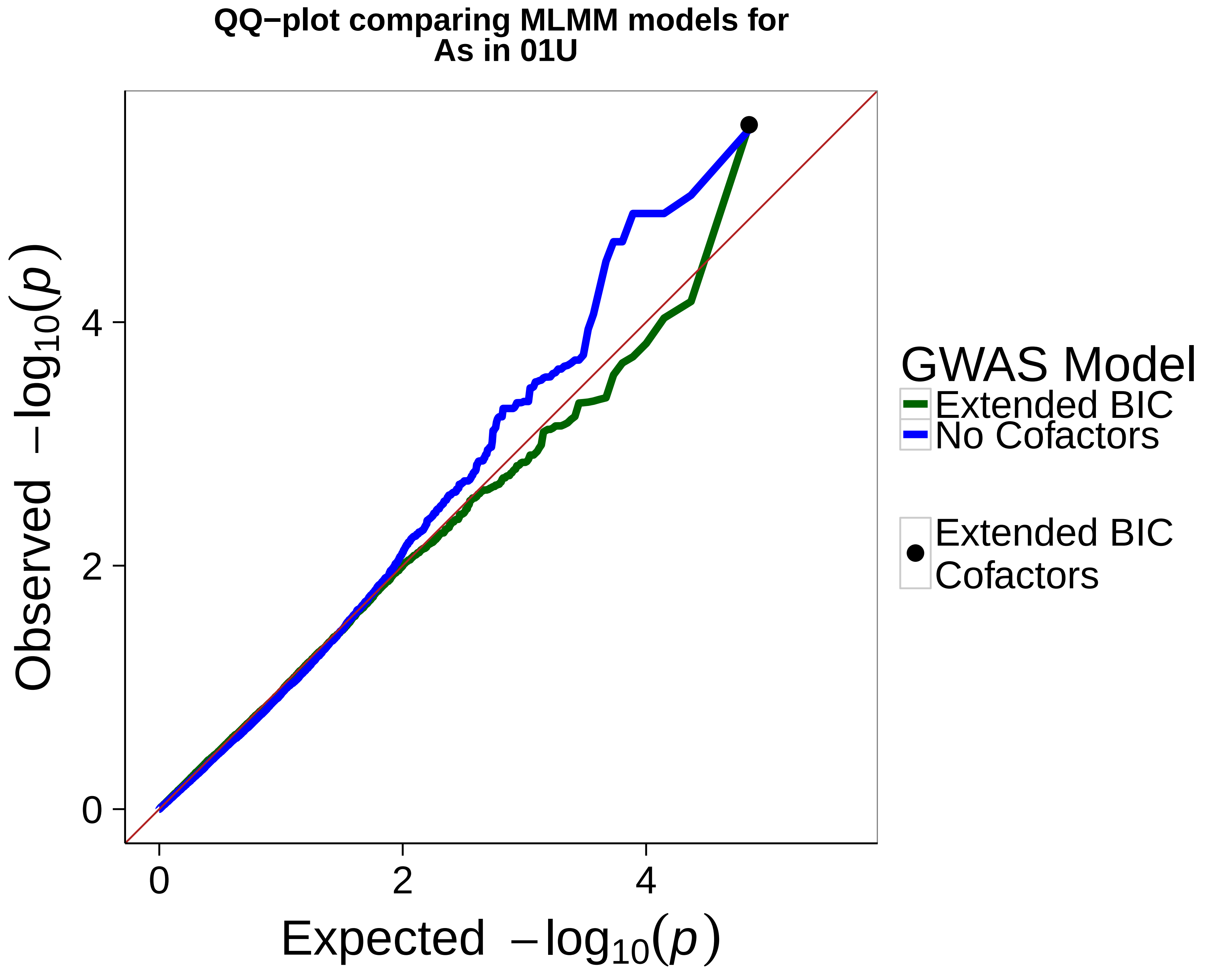
QQ−plot comparing MLMM models for
As in 01U
Observed −log10(p)
Expected −log10(p)
GWAS Model
Extended BIC
No Cofactors
Extended BIC
Cofactors

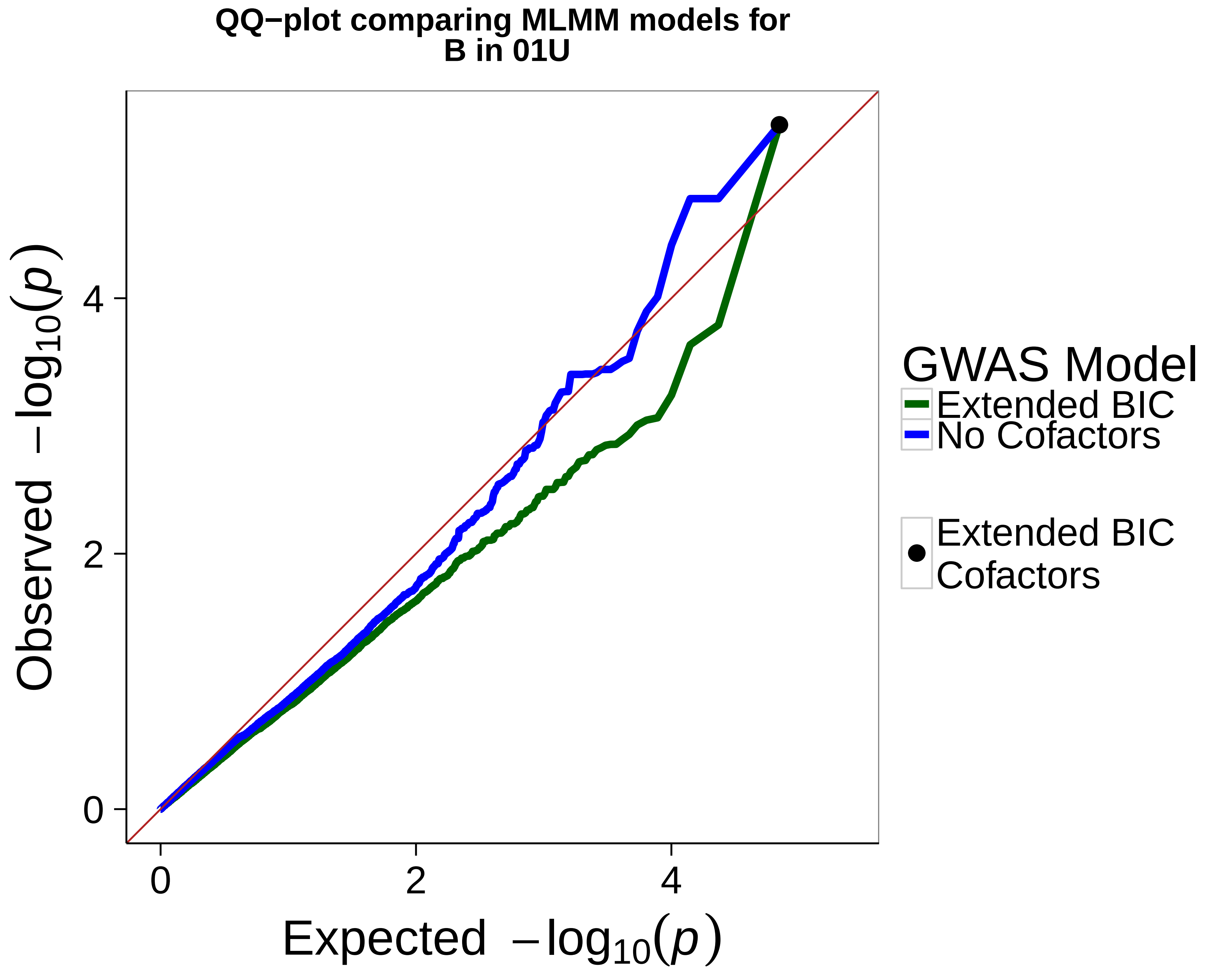
QQ−plot comparing MLMM models for
B in 01U
Observed −log10(p)
Expected −log10(p)
GWAS Model
Extended BIC
No Cofactors
Extended BIC
Cofactors

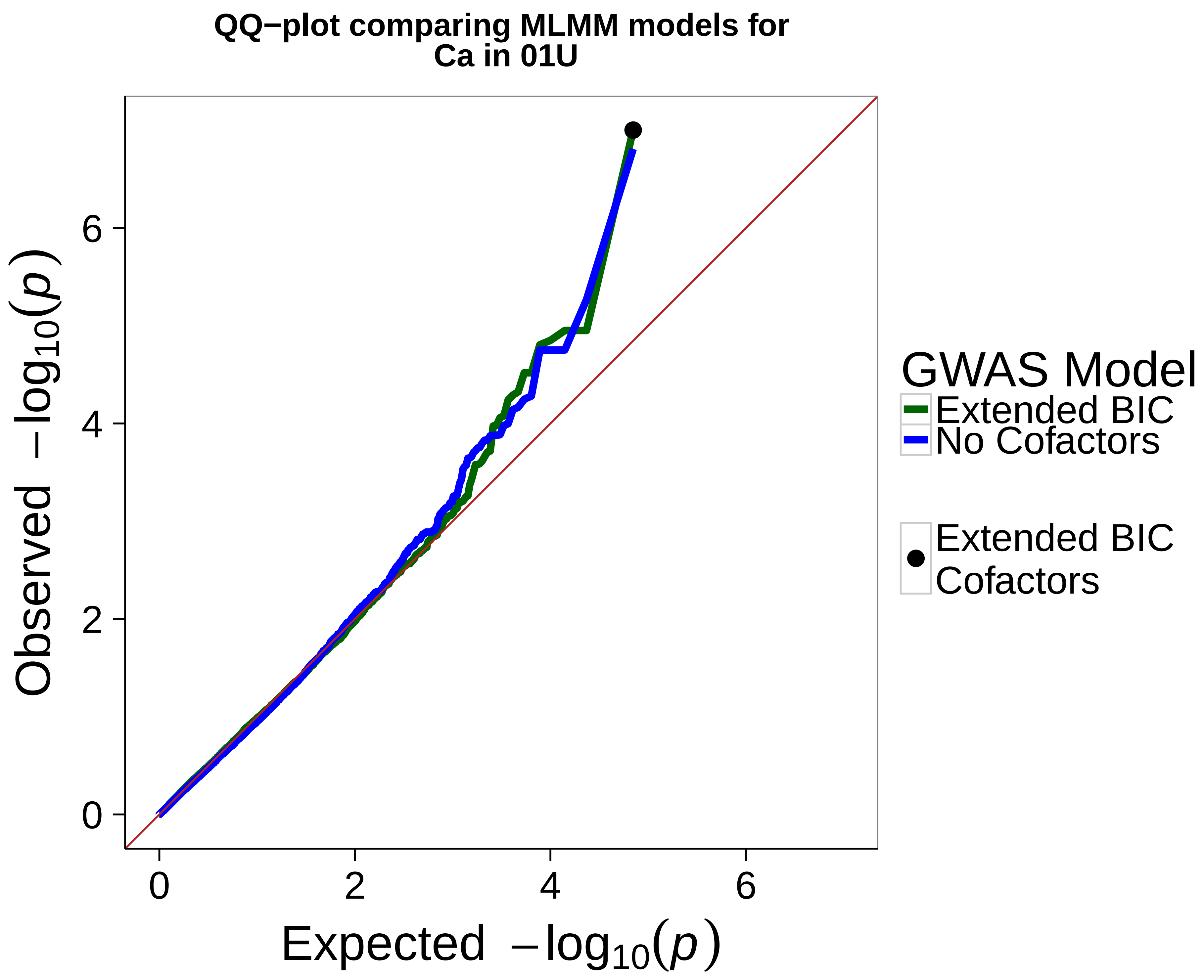
QQ−plot comparing MLMM models for
Ca in 01U
Observed −log10(p)
Expected −log10(p)
GWAS Model
Extended BIC
No Cofactors
Extended BIC
Cofactors

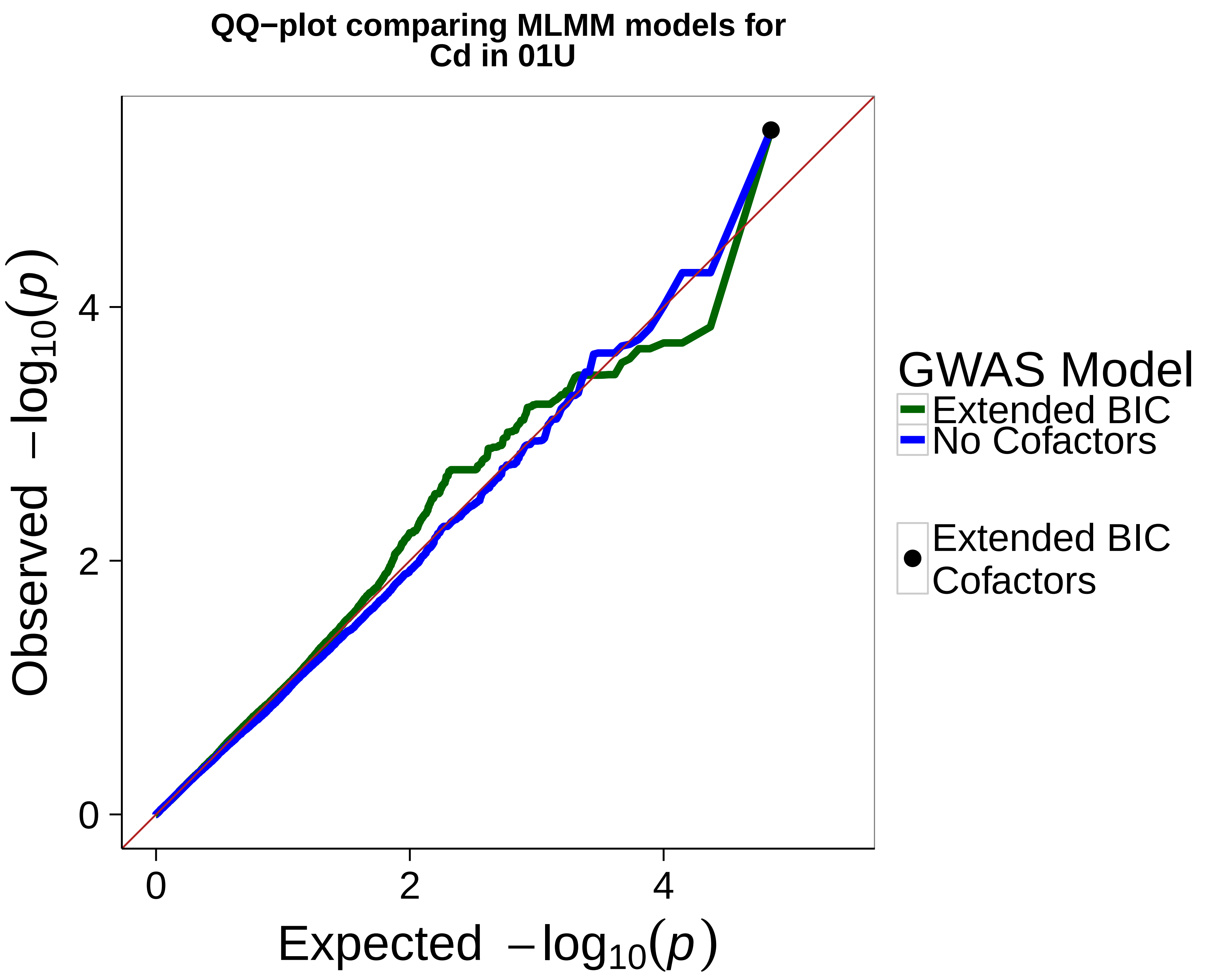
QQ−plot comparing MLMM models for
Cd in 01U
Observed −log10(p)
Expected −log10(p)
GWAS Model
Extended BIC
No Cofactors
Extended BIC
Cofactors

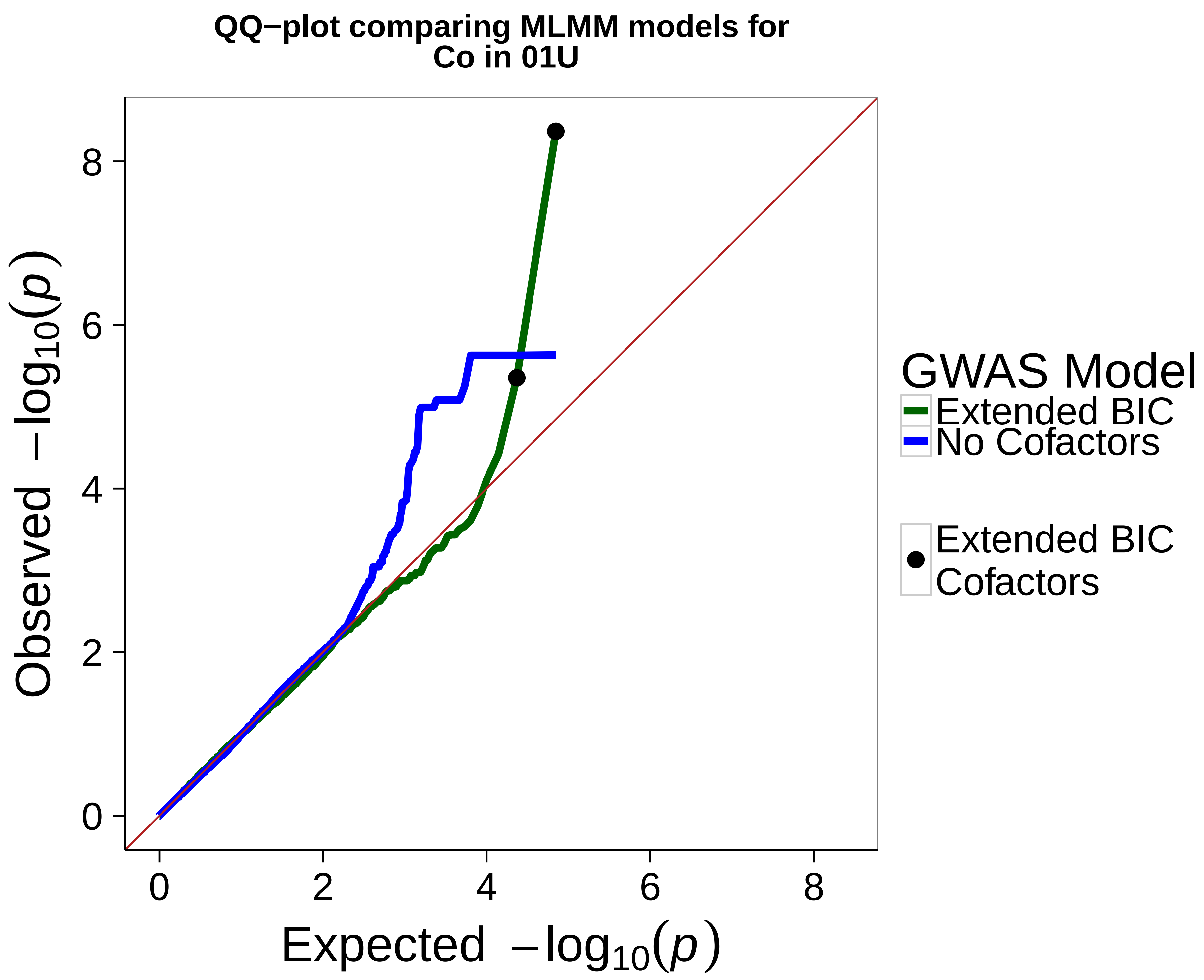
QQ−plot comparing MLMM models for
Co in 01U
Observed −log10(p)
Expected −log10(p)
GWAS Model
Extended BIC
No Cofactors
Extended BIC
Cofactors

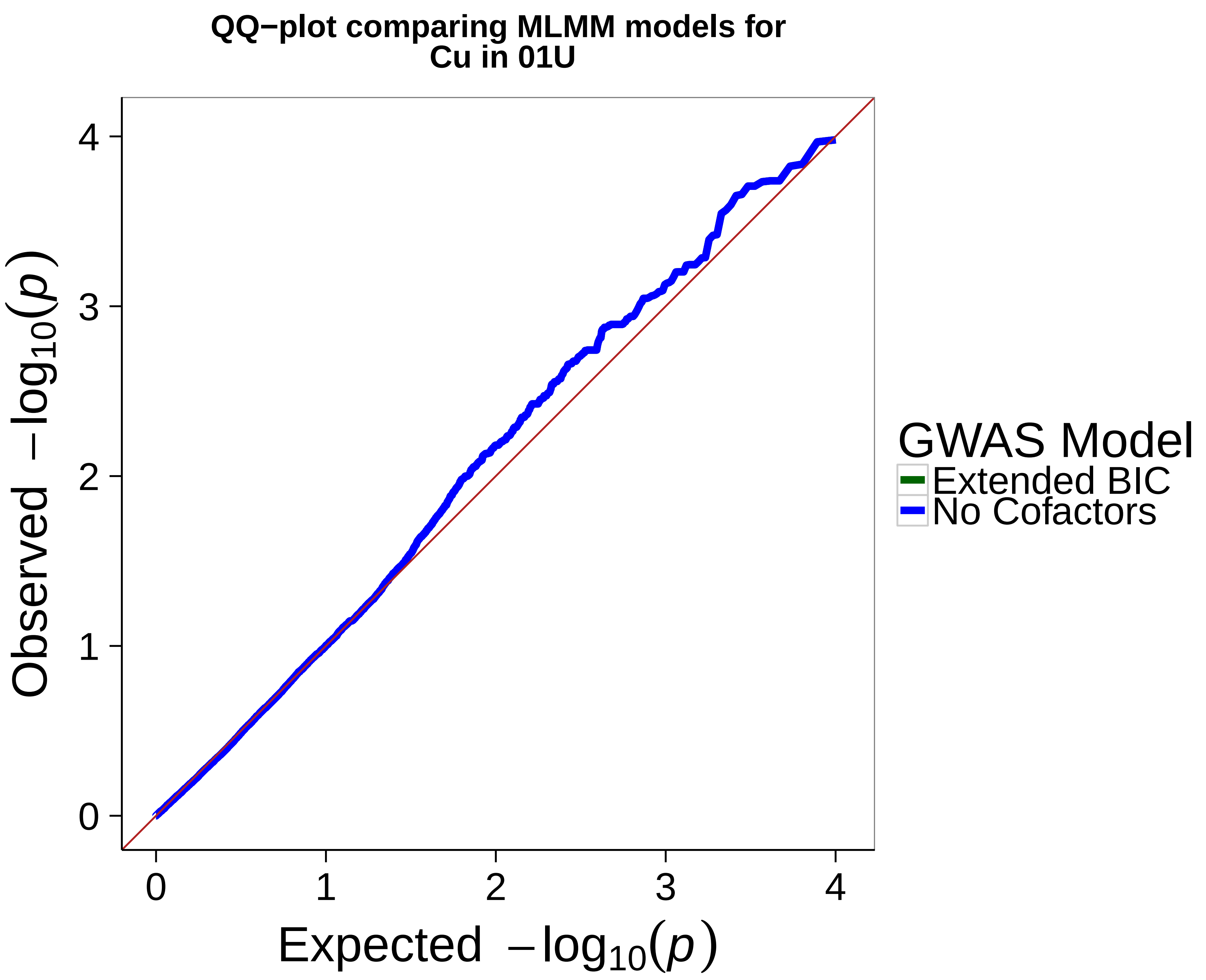
QQ−plot comparing MLMM models for
Cu in 01U
Observed −log10(p)
Expected −log10(p)
GWAS Model
Extended BIC
No Cofactors

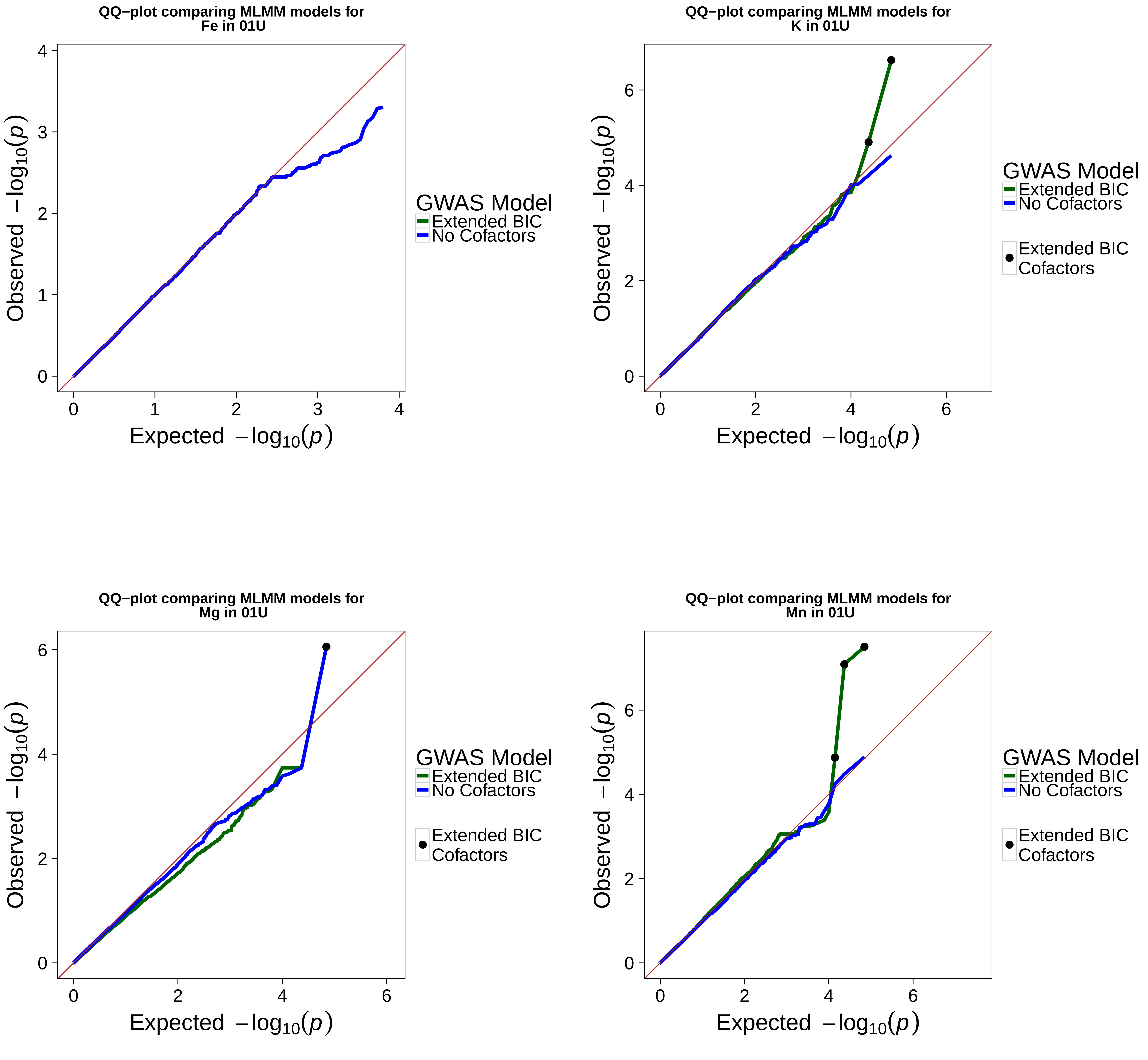

QQ−plot comparing MLMM models for
Fe in 01U
Observed −log10(p)
Expected −log10(p)
GWAS Model
Extended BIC
No Cofactors
QQ−plot comparing MLMM models for
K in 01U
Observed −log10(p)
Expected −log10(p)
GWAS Model
Extended BIC
No Cofactors
Extended BIC
Cofactors
QQ−plot comparing MLMM models for
Mg in 01U
Observed −log10(p)
Expected −log10(p)
GWAS Model
Extended BIC
No Cofactors
Extended BIC
Cofactors
QQ−plot comparing MLMM models for
Mn in 01U
Observed −log10(p)
Expected −log10(p)
GWAS Model
Extended BIC
No Cofactors
Extended BIC
Cofactors

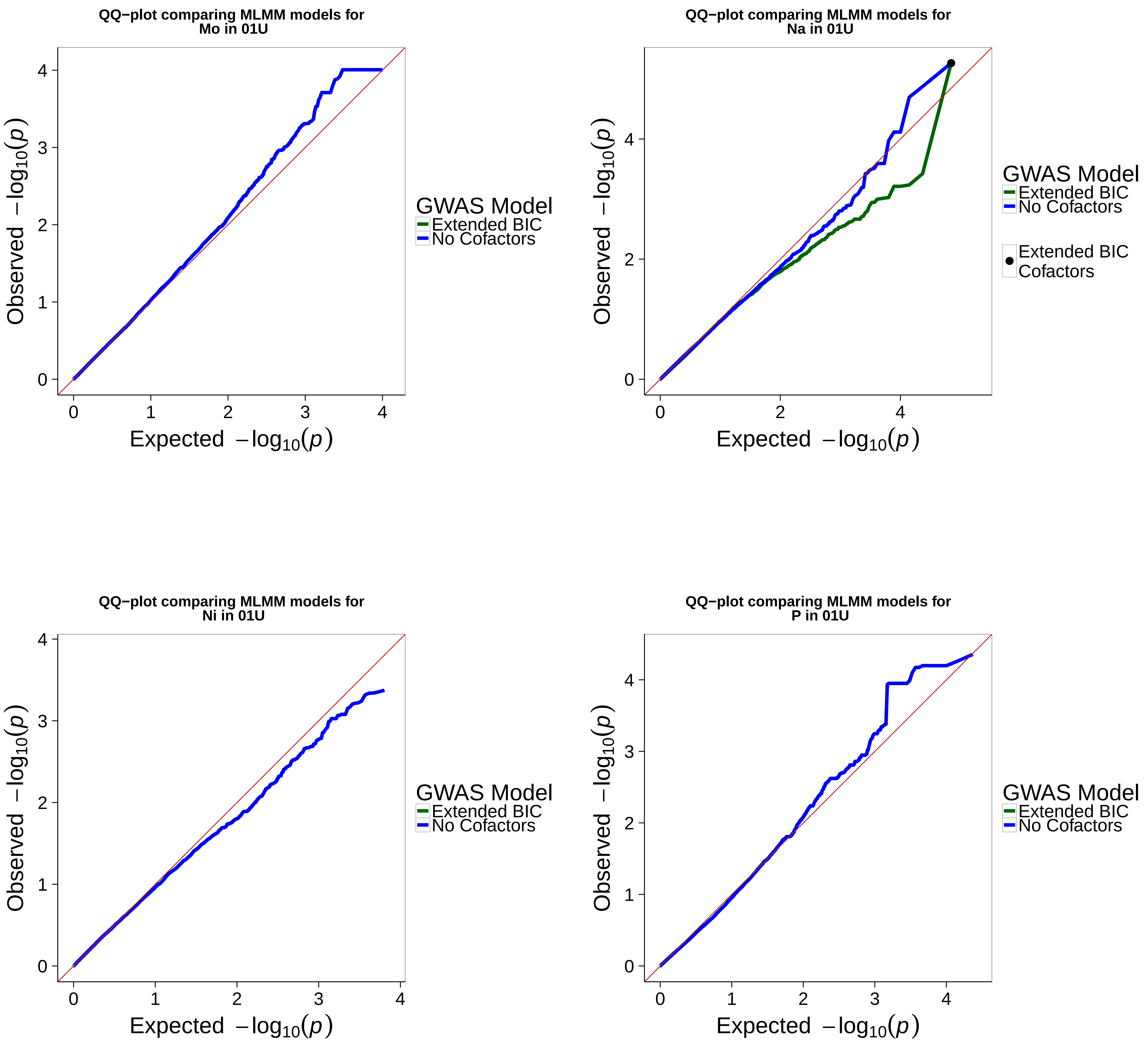
QQ−plot comparing MLMM models for
Mo in 01U
Observed −log10(p)
Expected −log10(p)
GWAS Model
Extended BIC
No Cofactors
QQ−plot comparing MLMM models for
Na in 01U
Observed −log10(p)
Expected −log10(p)
GWAS Model
Extended BIC
No Cofactors
Extended BIC
Cofactors
QQ−plot comparing MLMM models for
Ni in 01U
Observed −log10(p)
Expected −log10(p)
GWAS Model
Extended BIC
No Cofactors
QQ−plot comparing MLMM models for
P in 01U
Observed −log10(p)
Expected −log10(p)
GWAS Model
Extended BIC
No Cofactors

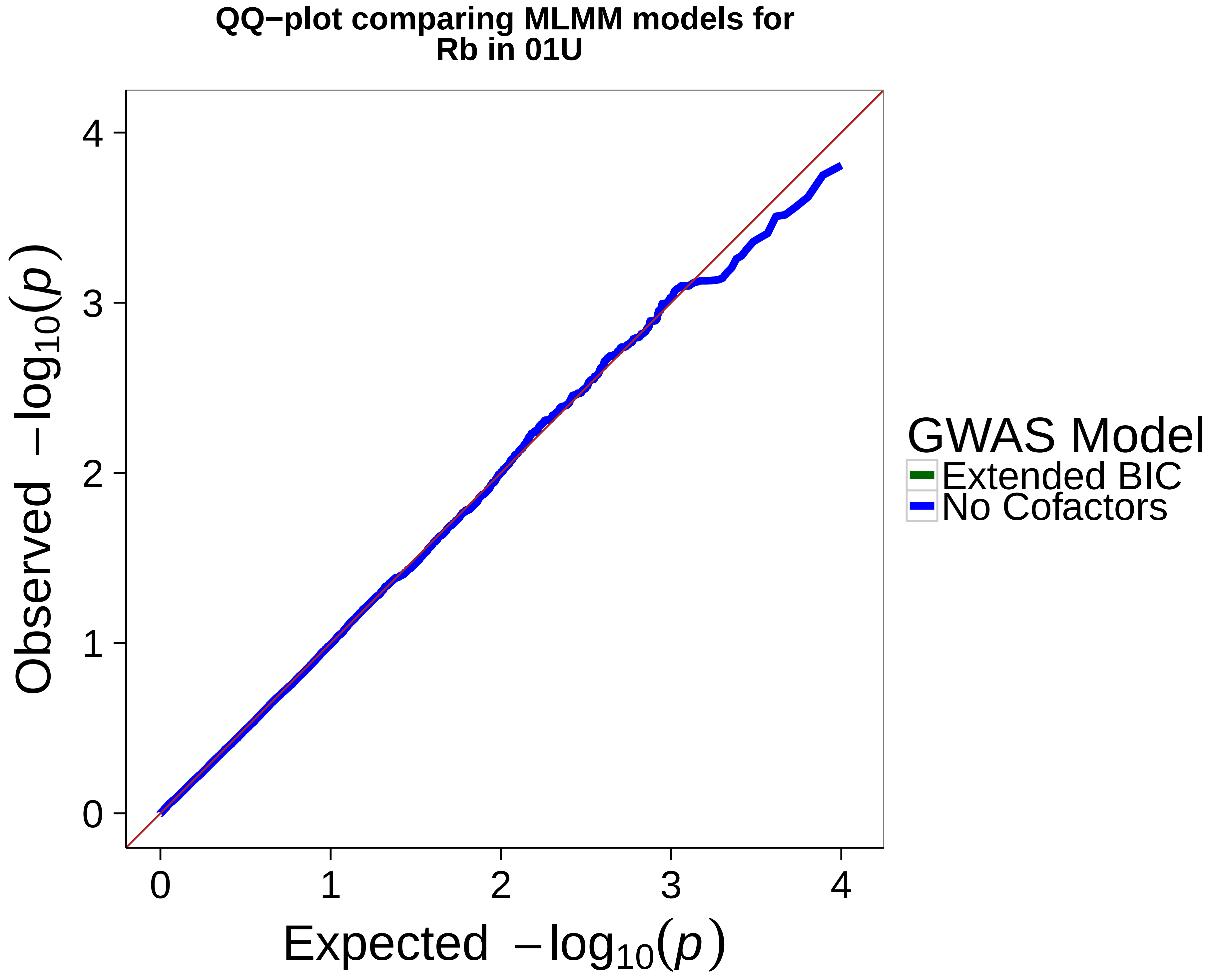
QQ−plot comparing MLMM models for
Rb in 01U
Observed −log10(p)
Expected −log10(p)
0
1
2
3
4
GWAS Model
Extended BIC
No Cofactors

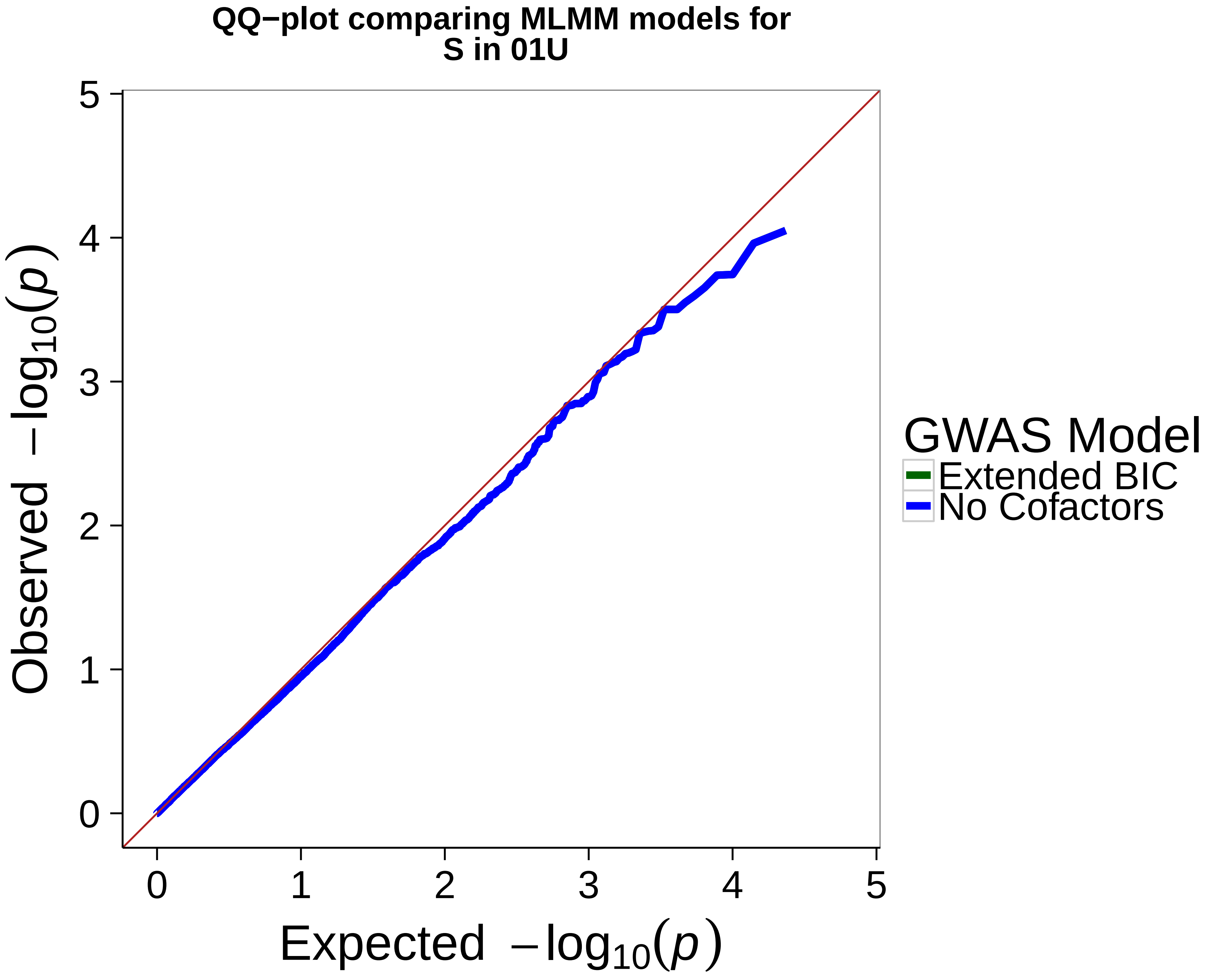
QQ−plot comparing MLMM models for
S in 01U
Observed −log10(p)
Expected −log10(p)
0
1
2
3
4
5
GWAS Model
Extended BIC
No Cofactors

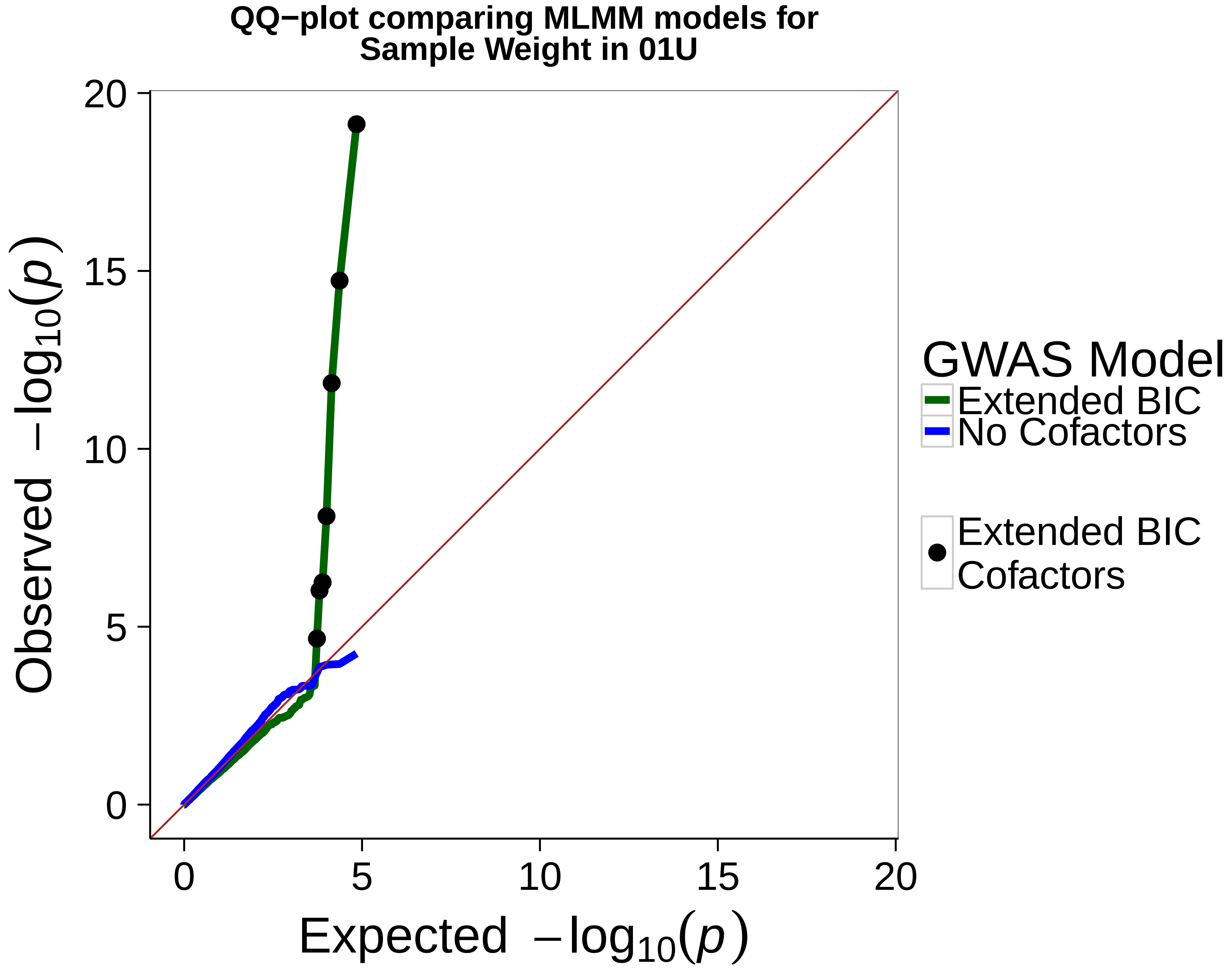
QQ−plot comparing MLMM models for
Sample Weight in 01U
Observed −log10(p)
Expected −log10(p)
0
5
10
15
20
GWAS Model
Extended BIC
No Cofactors
Extended BIC
Cofactors

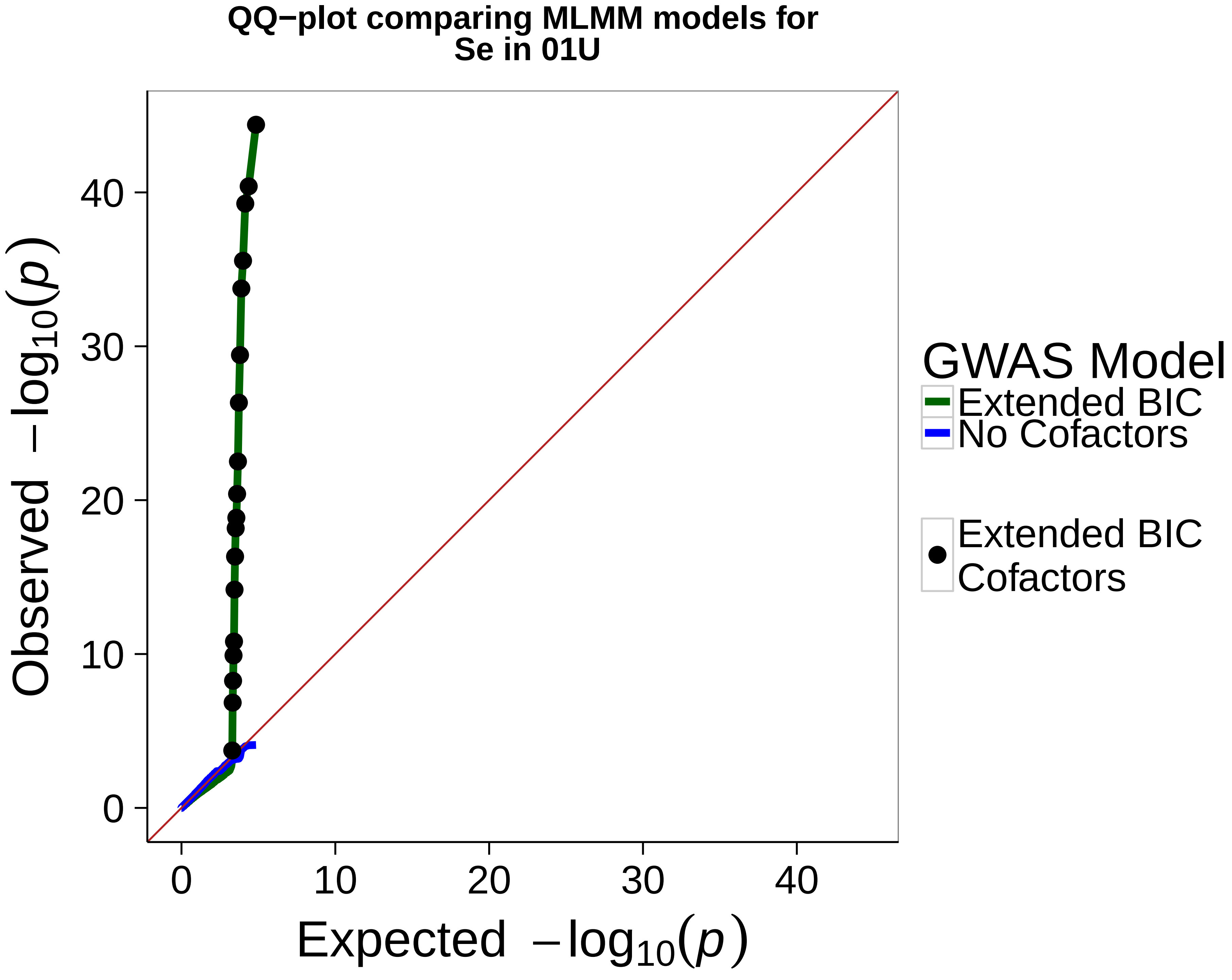
QQ−plot comparing MLMM models for
Se in 01U
Observed −log10(p)
Expected −log10(p)
0
10
20
30
40
GWAS Model
Extended BIC
No Cofactors
Extended BIC
Cofactors

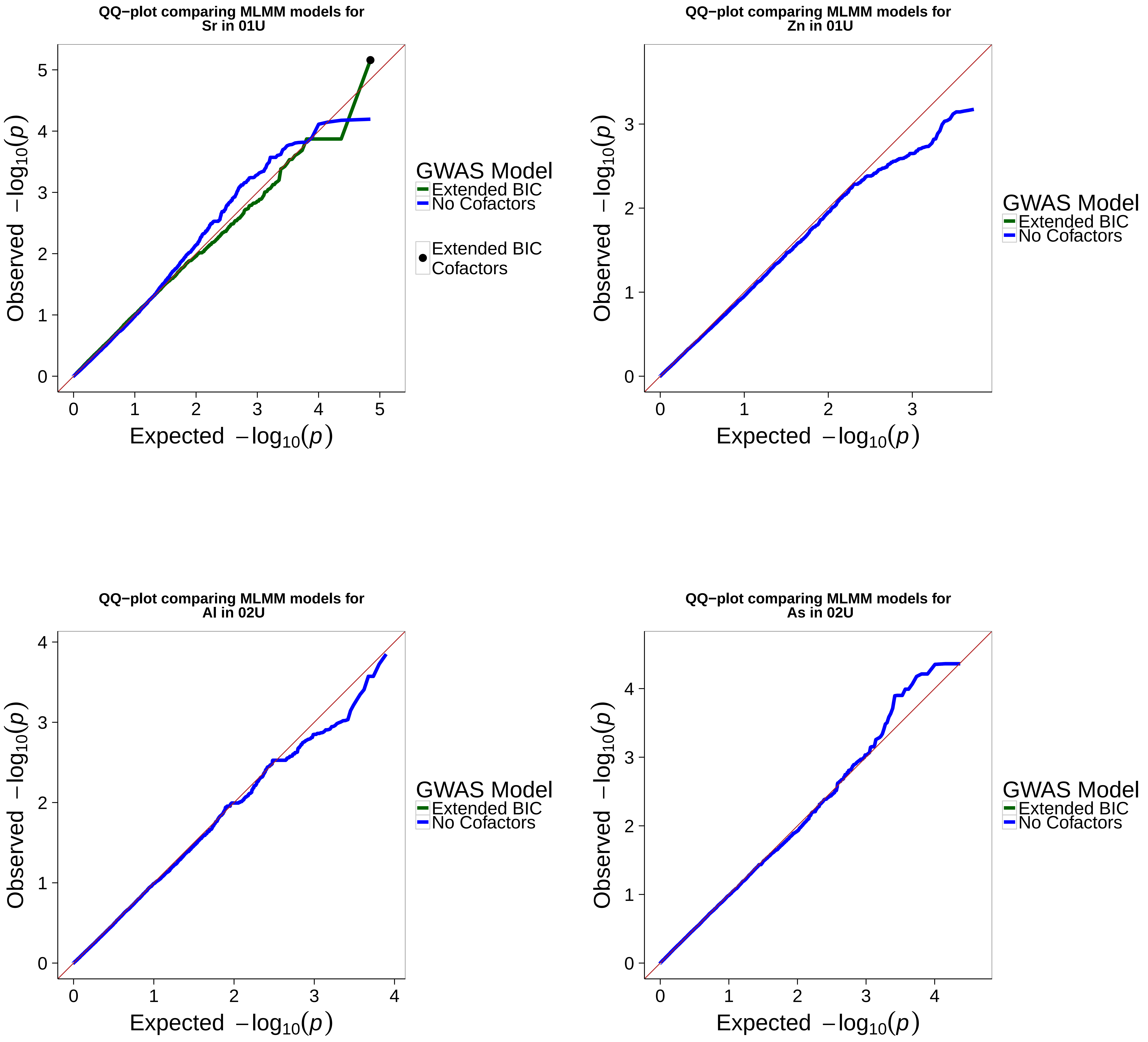
QQ−plot comparing MLMM models for
Sr in 01U
Observed −log10(p)
Expected −log10(p)
GWAS Model
Extended BIC
No Cofactors
Extended BIC
Cofactors
QQ−plot comparing MLMM models for
Zn in 01U
Observed −log10(p)
Expected −log10(p)
GWAS Model
Extended BIC
No Cofactors
QQ−plot comparing MLMM models for
Al in 02U
Observed −log10(p)
Expected −log10(p)
GWAS Model
Extended BIC
No Cofactors
QQ−plot comparing MLMM models for
As in 02U
Observed −log10(p)
Expected −log10(p)
GWAS Model
Extended BIC
No Cofactors

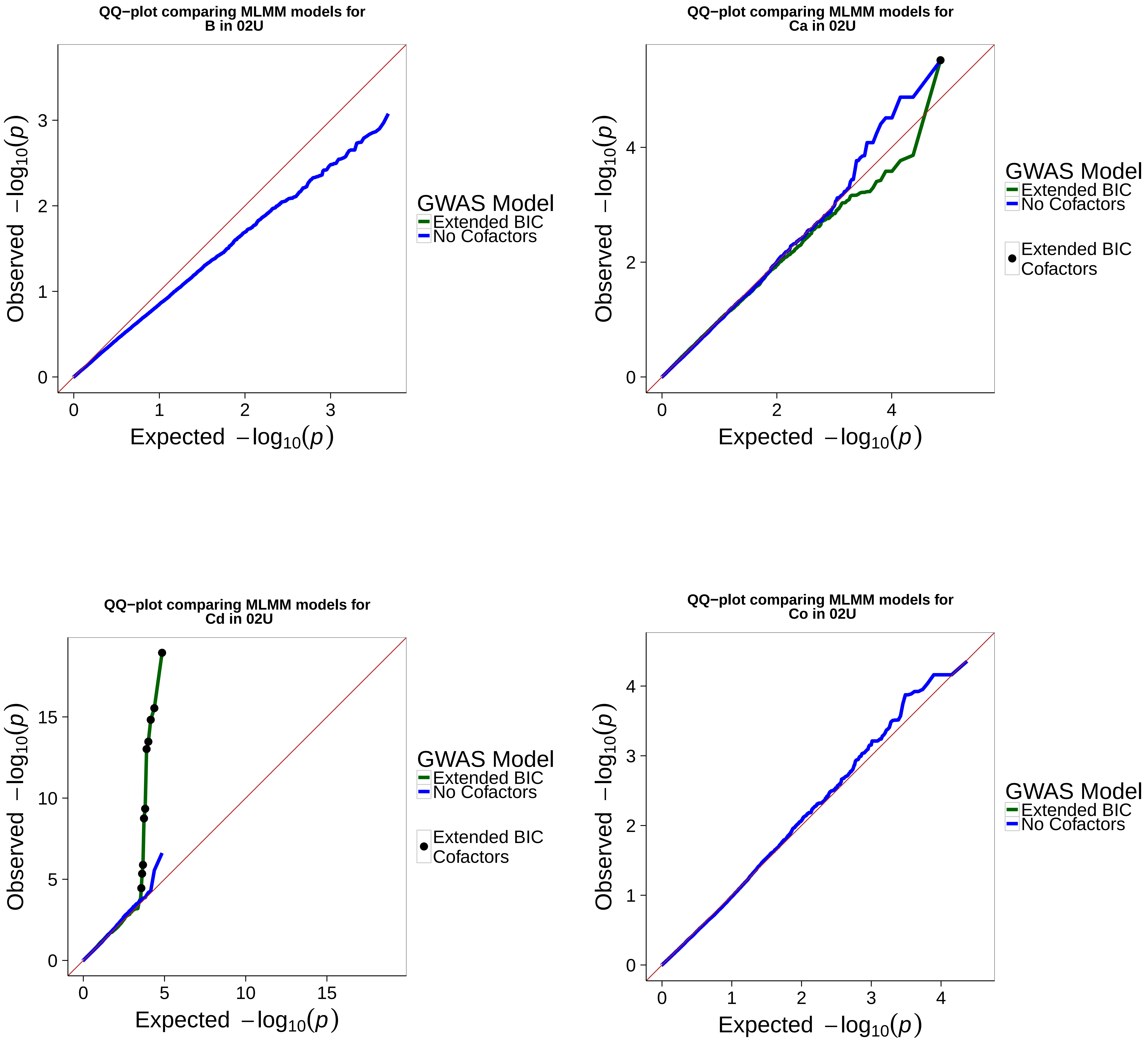
QQ−plot comparing MLMM models for
B in 02U
QQ−plot comparing MLMM models for
Ca in 02U
QQ−plot comparing MLMM models for
Cd in 02U
QQ−plot comparing MLMM models for
Co in 02U
Observed −log10(p)
Expected −log10(p)
GWAS Model
Extended BIC
No Cofactors
Extended BIC
Cofactors

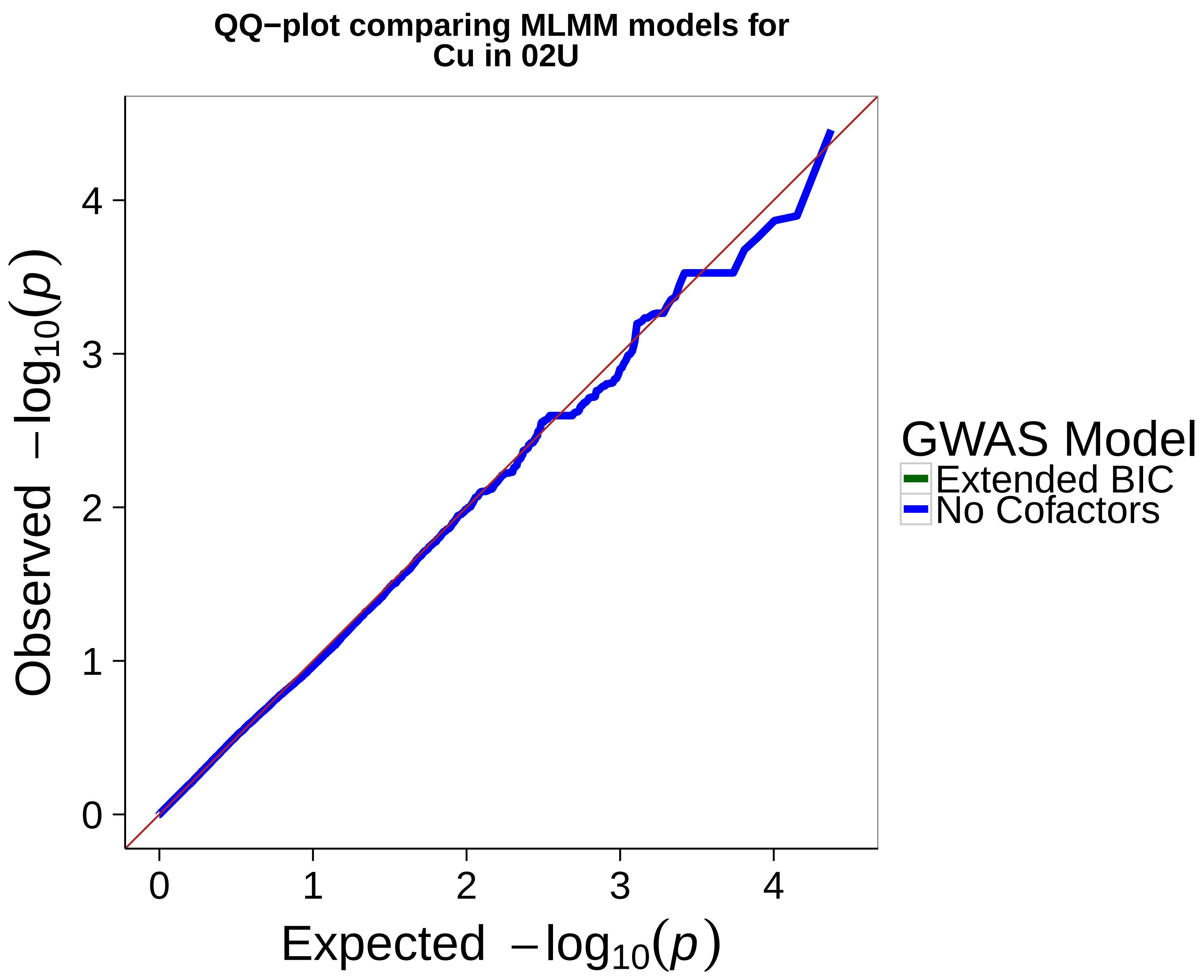
QQ−plot comparing MLMM models for
Cu in 02U
Observed −log10(p)
Expected −log10(p)
GWAS Model
Extended BIC
No Cofactors

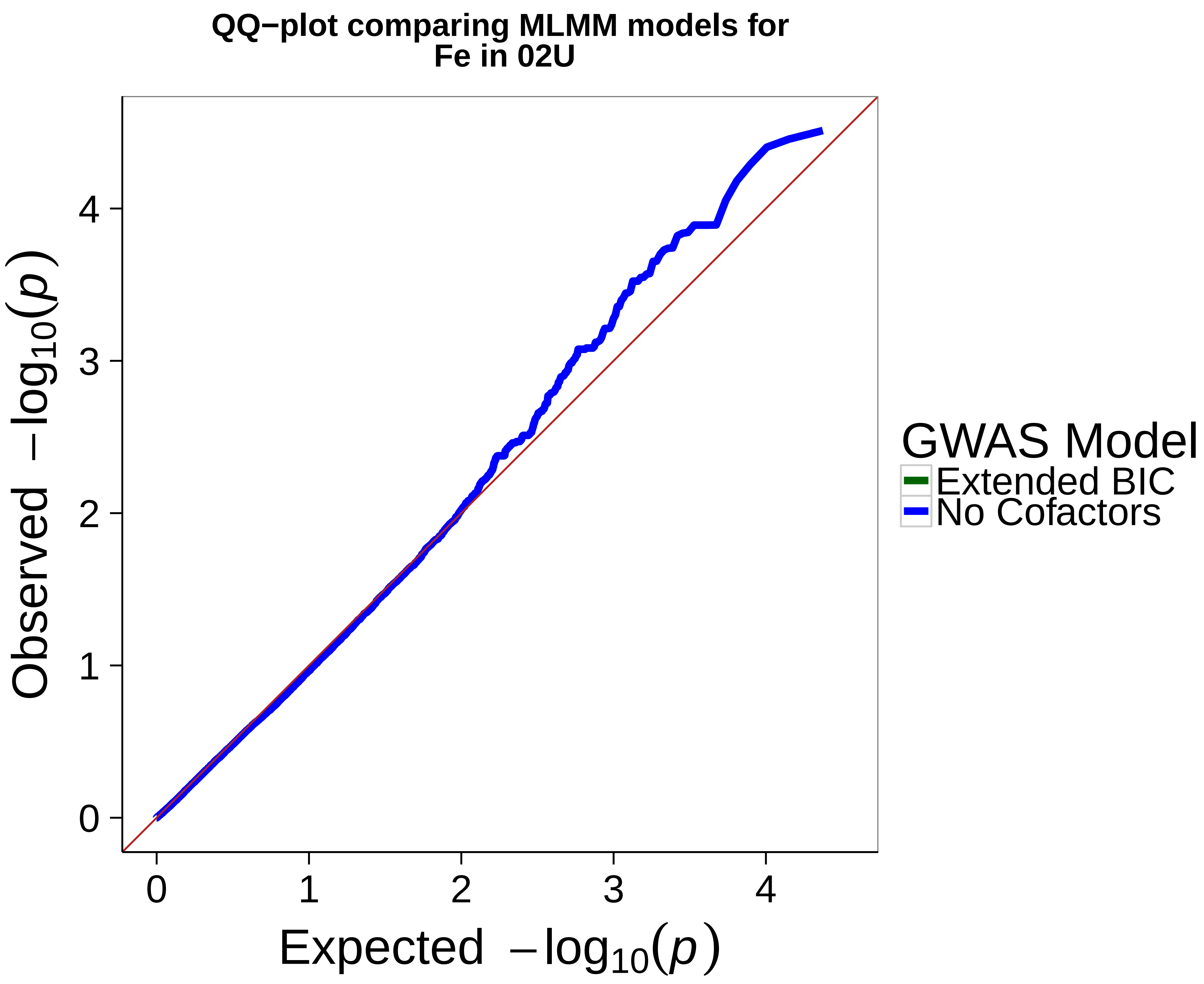
QQ−plot comparing MLMM models for
Fe in 02U
Observed −log10(p)
Expected −log10(p)
GWAS Model
Extended BIC
No Cofactors

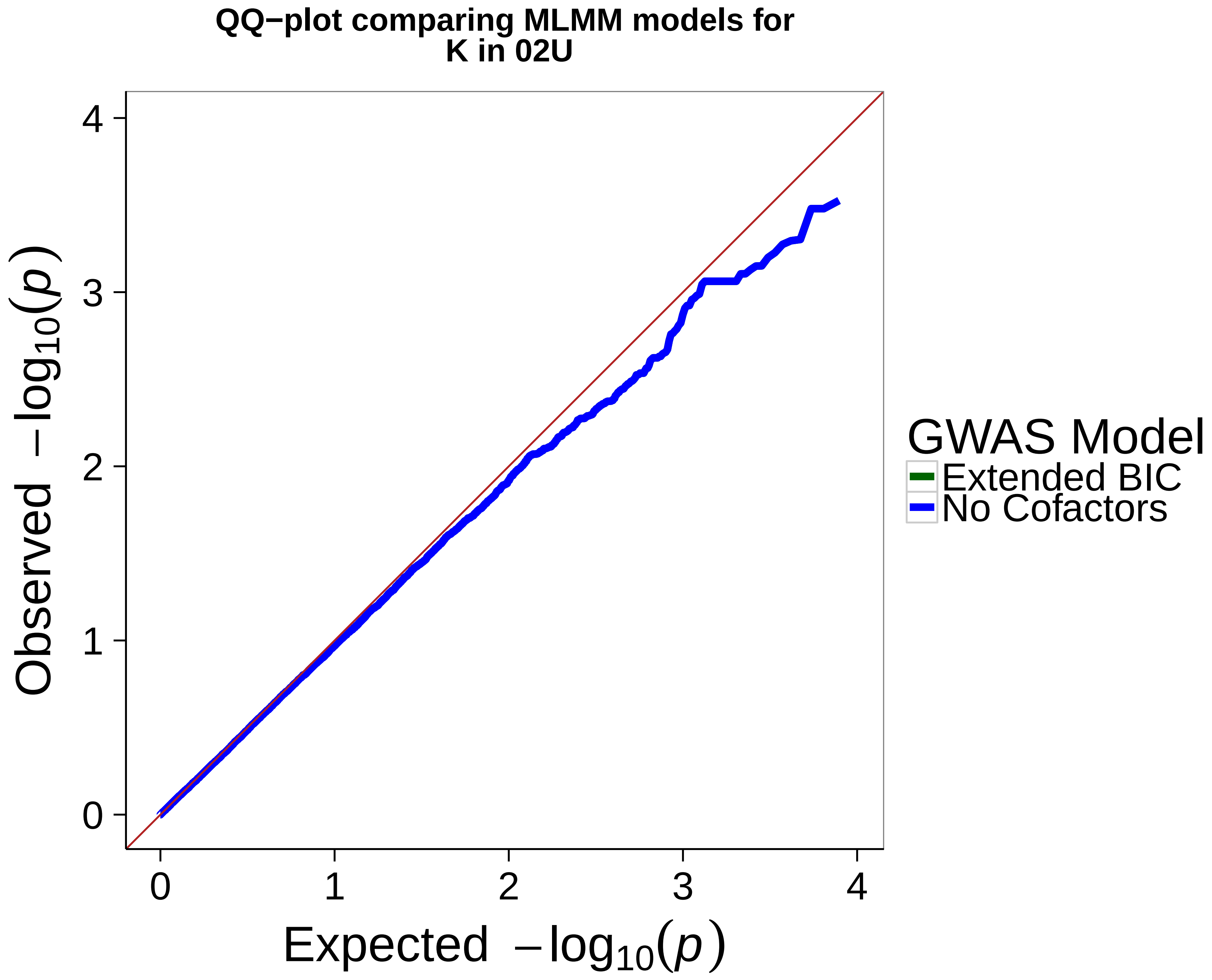
QQ−plot comparing MLMM models for
K in 02U
Observed −log10(p)
Expected −log10(p)
GWAS Model
Extended BIC
No Cofactors

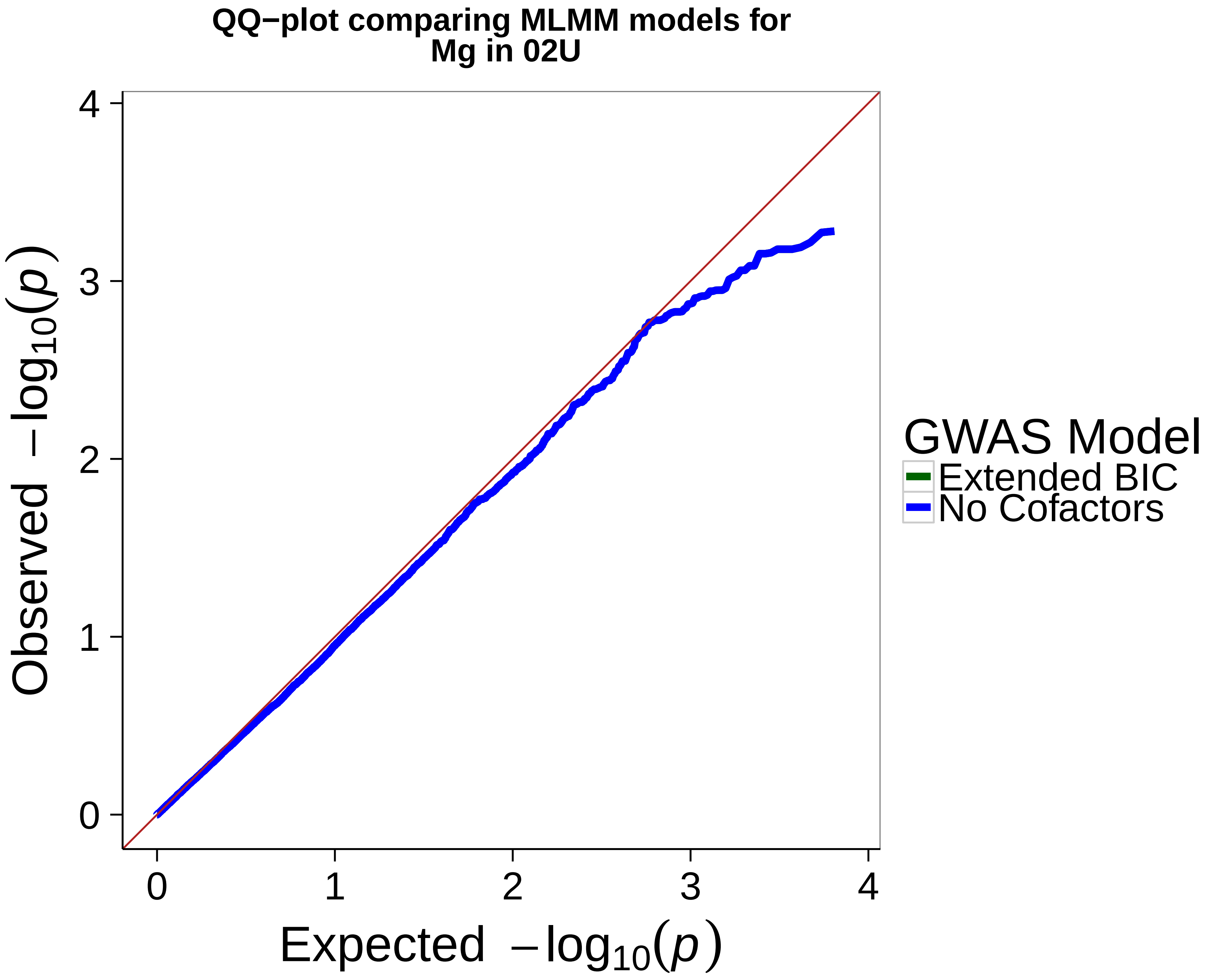
QQ−plot comparing MLMM models for
Mg in 02U
Observed −log10(p)
Expected −log10(p)
GWAS Model
Extended BIC
No Cofactors

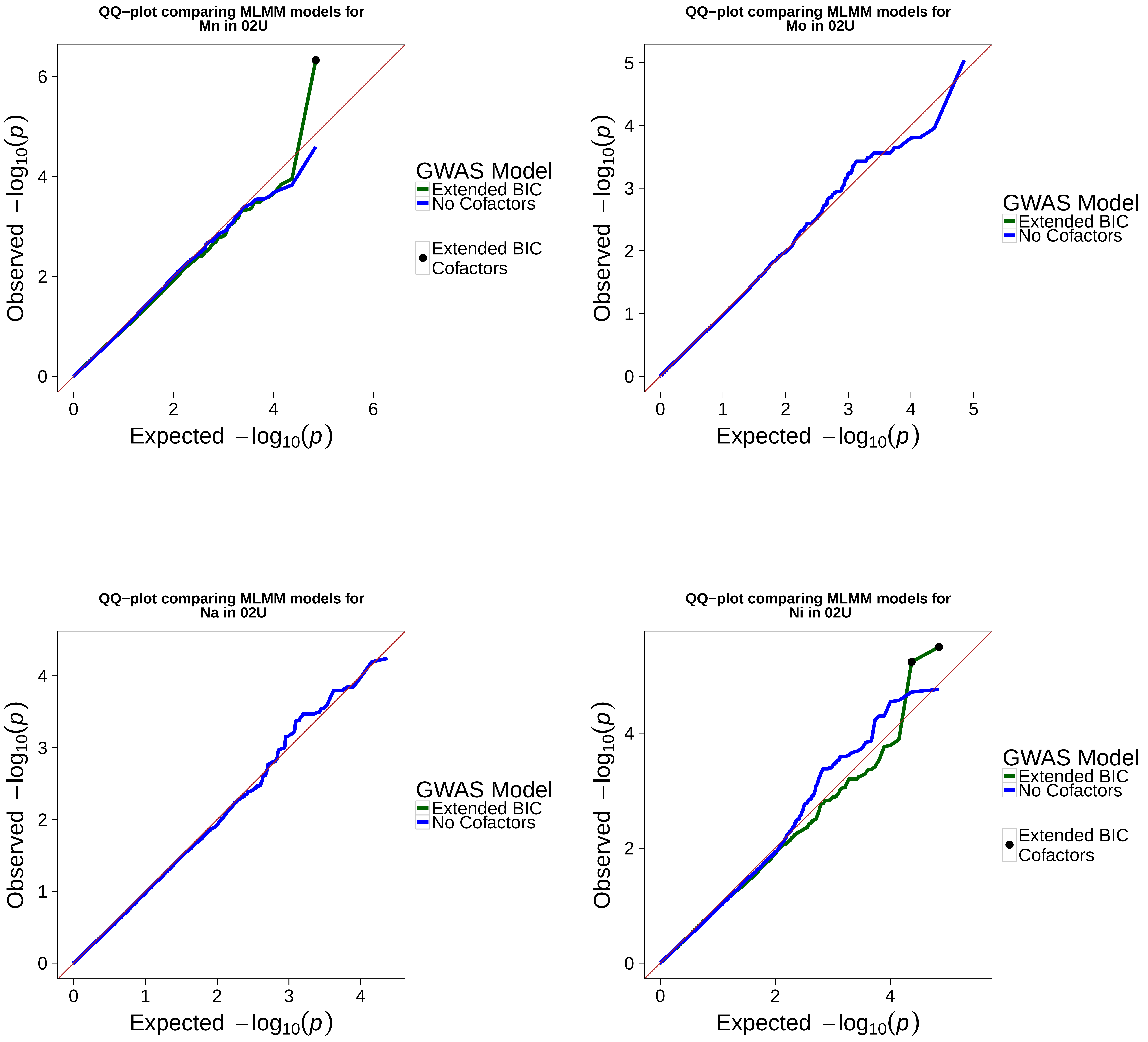
QQ−plot comparing MLMM models for
Mn in 02U
Observed −log10(p)
Expected −log10(p)
GWAS Model
Extended BIC
No Cofactors
Extended BIC
Cofactors
QQ−plot comparing MLMM models for
Mo in 02U
Observed −log10(p)
Expected −log10(p)
GWAS Model
Extended BIC
No Cofactors
QQ−plot comparing MLMM models for
Na in 02U
Observed −log10(p)
Expected −log10(p)
GWAS Model
Extended BIC
No Cofactors
QQ−plot comparing MLMM models for
Ni in 02U
Observed −log10(p)
Expected −log10(p)
GWAS Model
Extended BIC
No Cofactors
Extended BIC
Cofactors

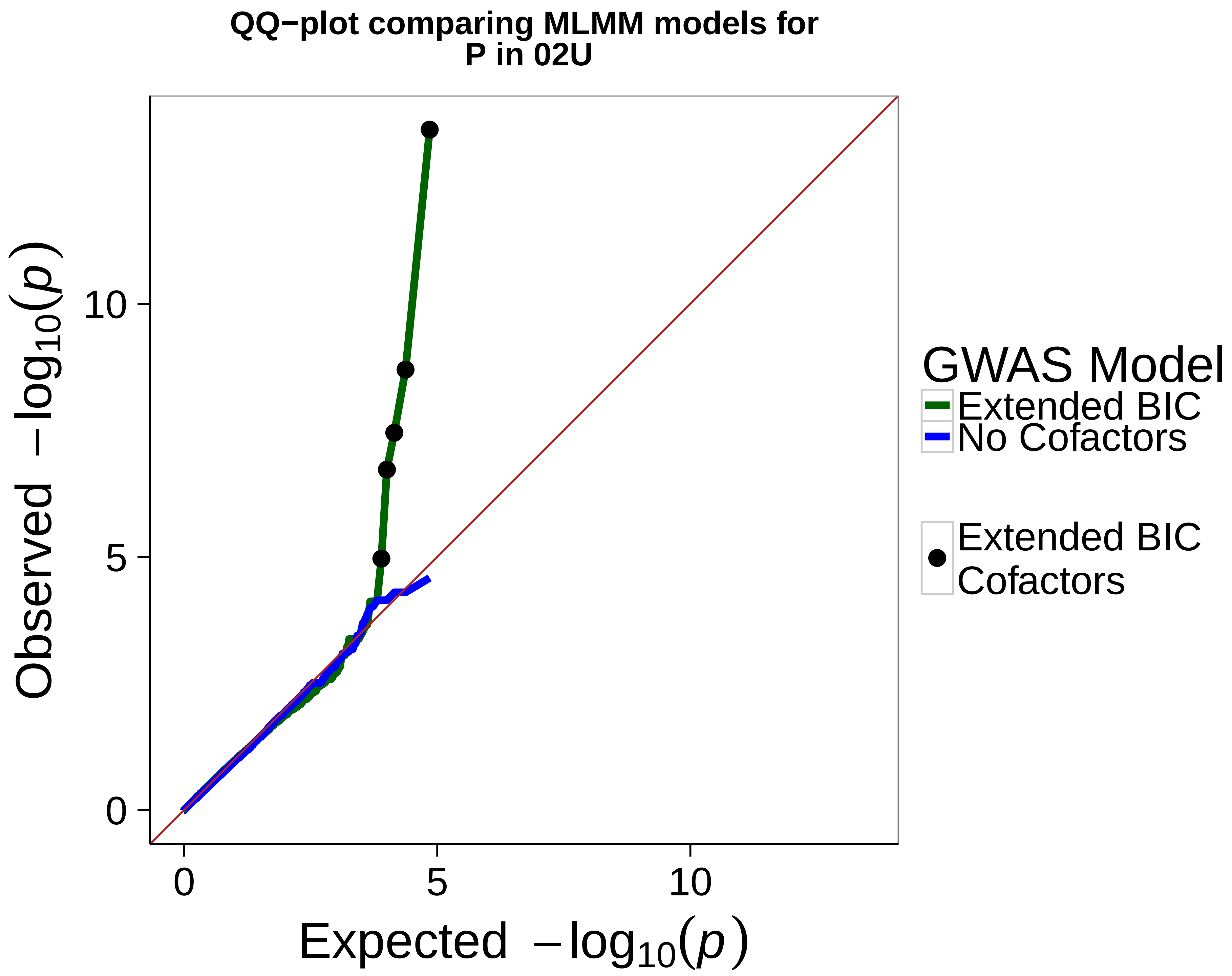
QQ−plot comparing MLMM models for
P in 02U
Observed −log10(p)
Expected −log10(p)
0
5
10
GWAS Model
Extended BIC
No Cofactors
Extended BIC
Cofactors

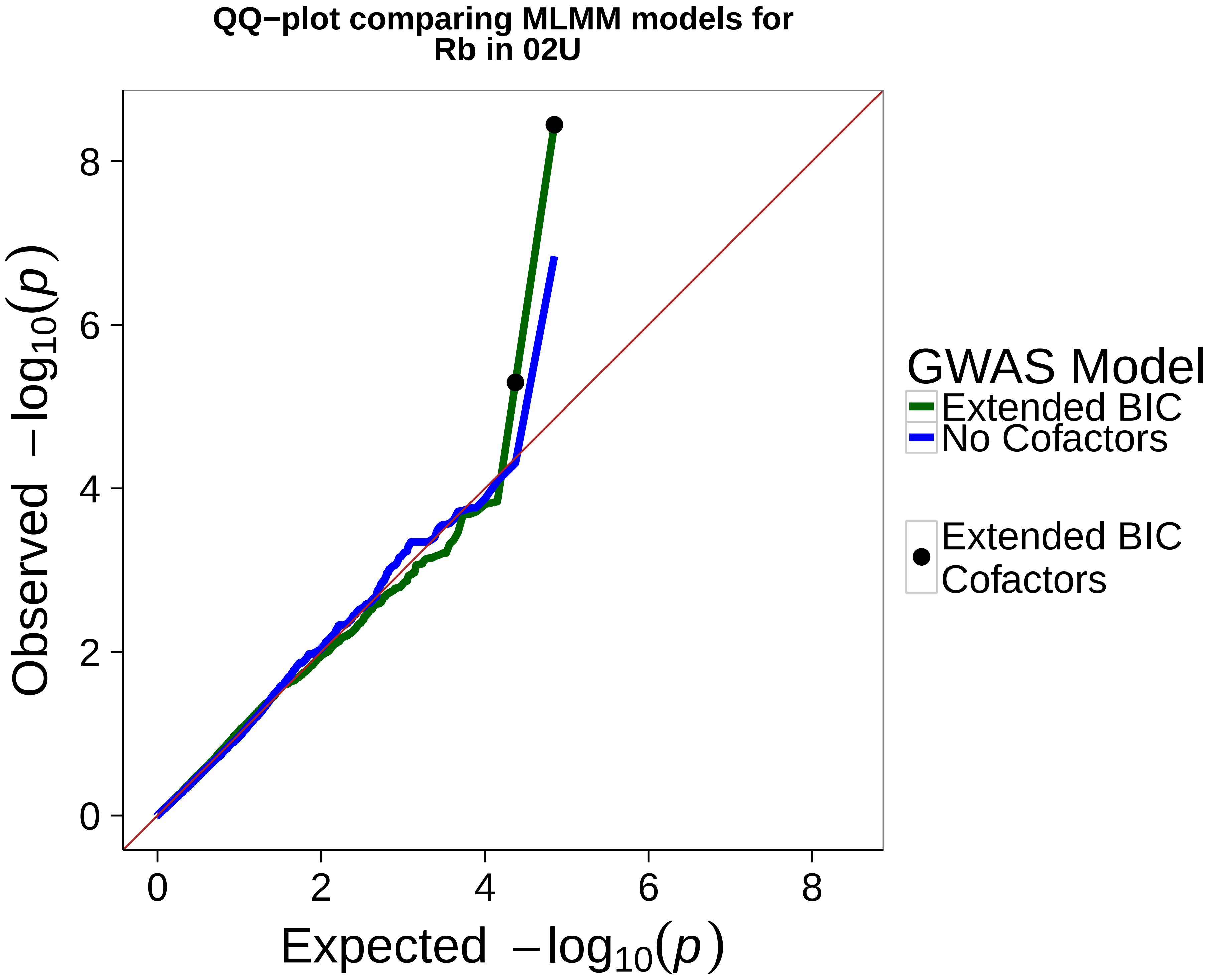
QQ−plot comparing MLMM models for
Rb in 02U
Observed −log10(p)
Expected −log10(p)
0
2
4
6
8
GWAS Model
Extended BIC
No Cofactors
Extended BIC
Cofactors

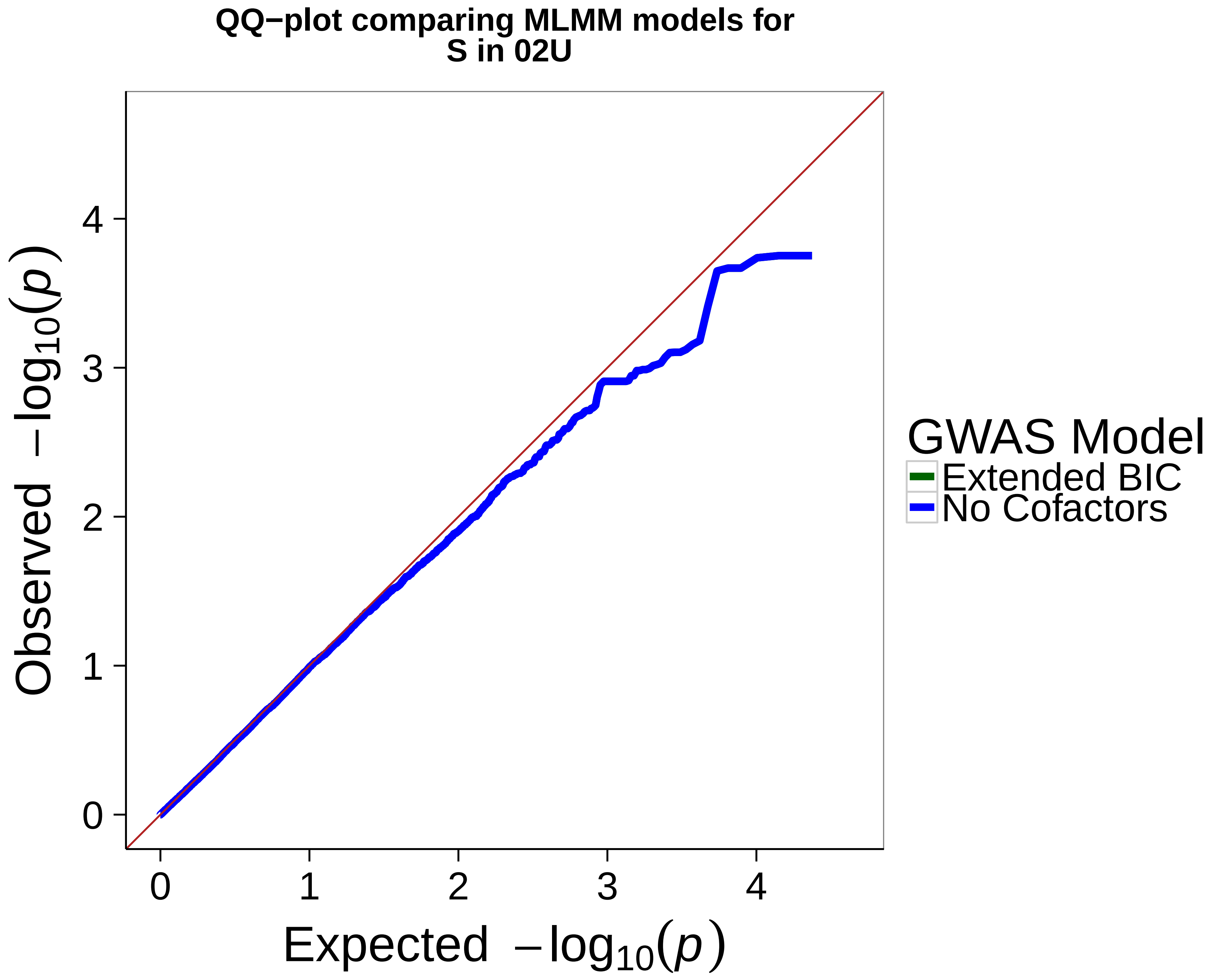
QQ−plot comparing MLMM models for
S in 02U
Observed −log10(p)
Expected −log10(p)
0
1
2
3
4
GWAS Model
Extended BIC
No Cofactors

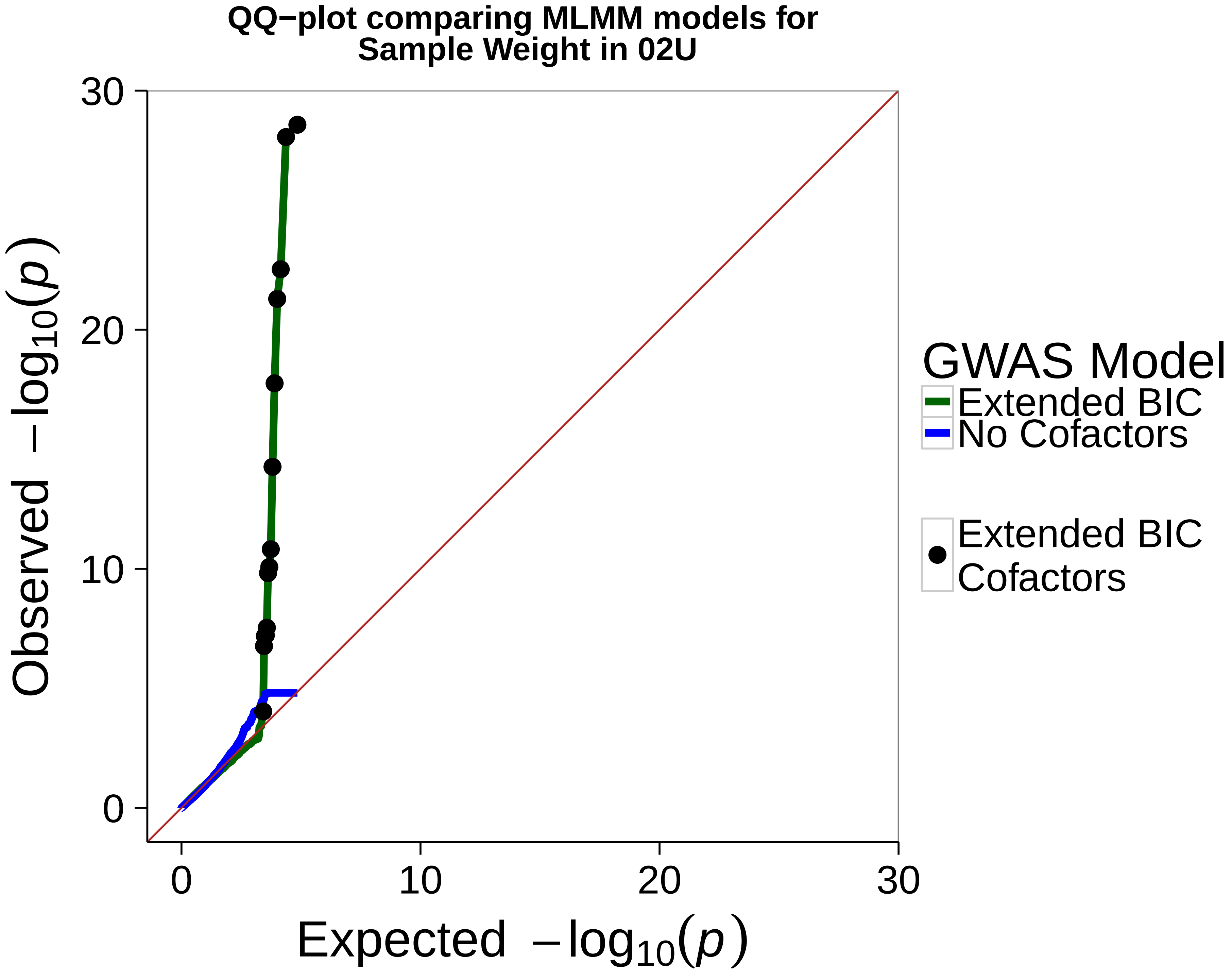
QQ−plot comparing MLMM models for
Sample Weight in 02U
Observed −log10(p)
Expected −log10(p)
0
10
20
30
GWAS Model
Extended BIC
No Cofactors
Extended BIC
Cofactors

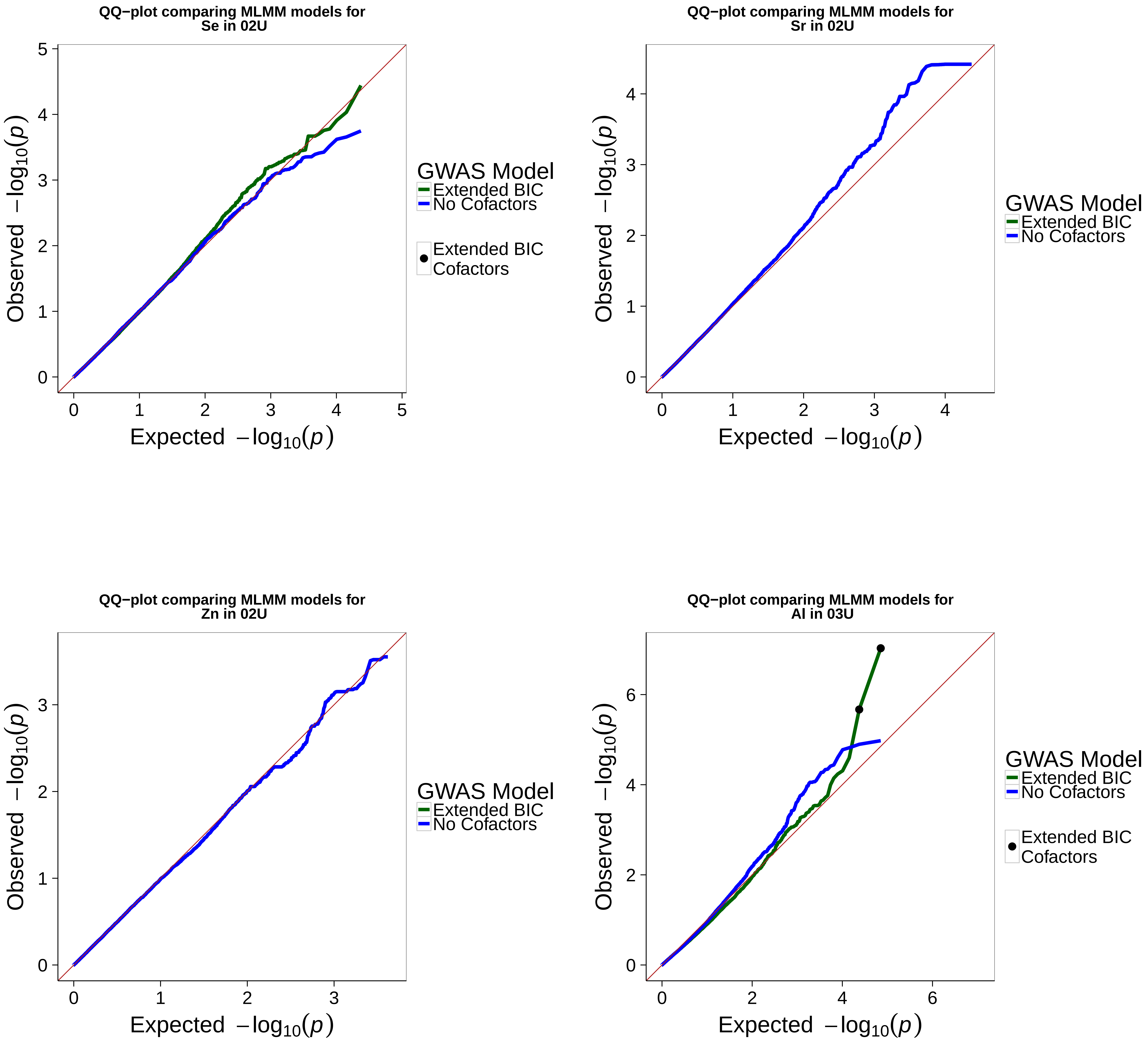
QQ−plot comparing MLMM models for Se in 02U
Observed −log10(p)
Expected −log10(p)
GWAS Model
Extended BIC
No Cofactors
Extended BIC Cofactors
QQ−plot comparing MLMM models for Sr in 02U
Observed −log10(p)
Expected −log10(p)
GWAS Model
Extended BIC
No Cofactors
QQ−plot comparing MLMM models for Zn in 02U
Observed −log10(p)
Expected −log10(p)
GWAS Model
Extended BIC
No Cofactors
QQ−plot comparing MLMM models for Al in 03U
Observed −log10(p)
Expected −log10(p)
GWAS Model
Extended BIC
No Cofactors
Extended BIC Cofactors

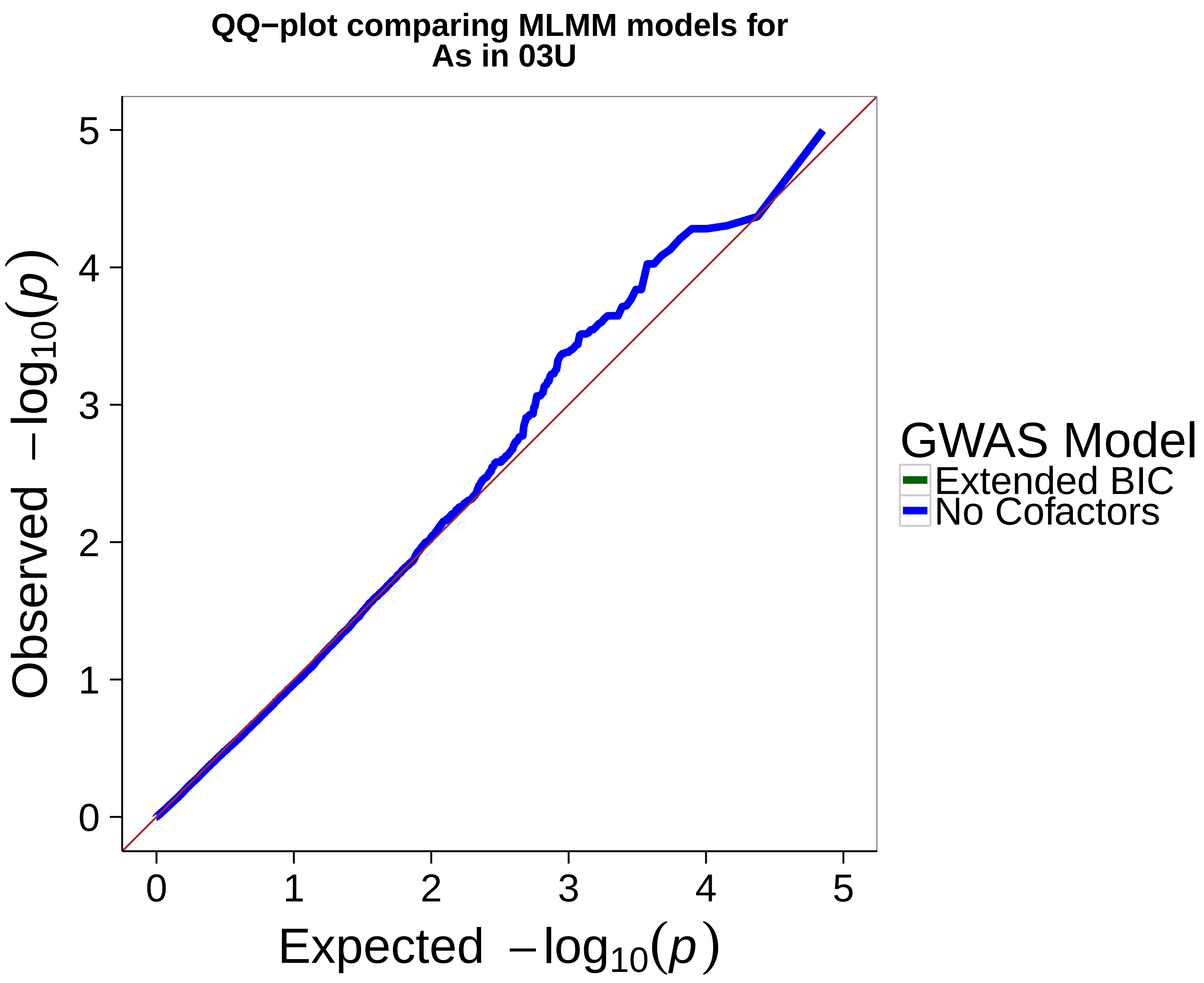
QQ−plot comparing MLMM models for
As in 03U
Observed −log10(p)
Expected −log10(p)
0
1
2
3
4
5
GWAS Model
Extended BIC
No Cofactors

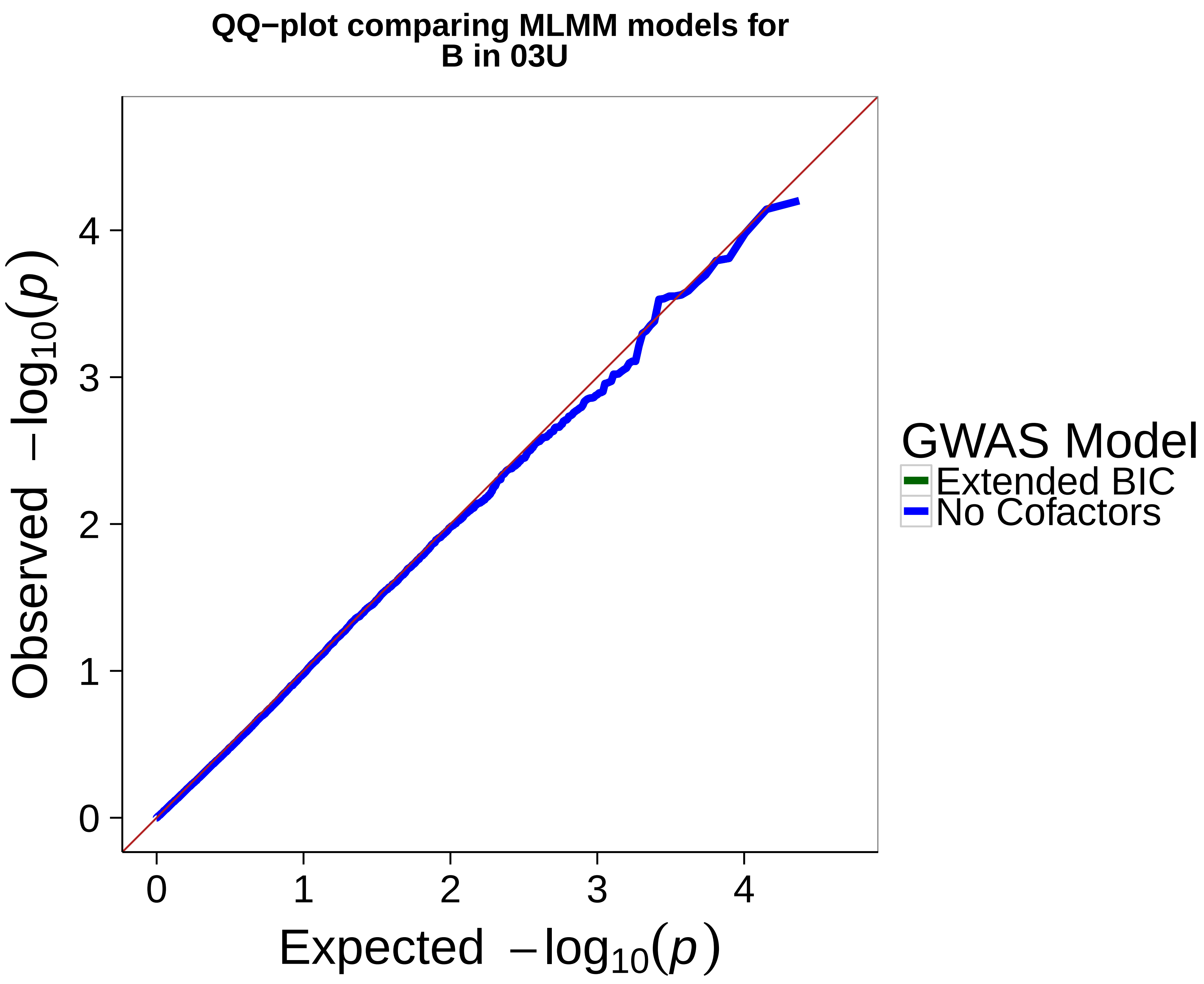
QQ−plot comparing MLMM models for
B in 03U
Observed −log10(p)
Expected −log10(p)
0
1
2
3
4
GWAS Model
Extended BIC
No Cofactors

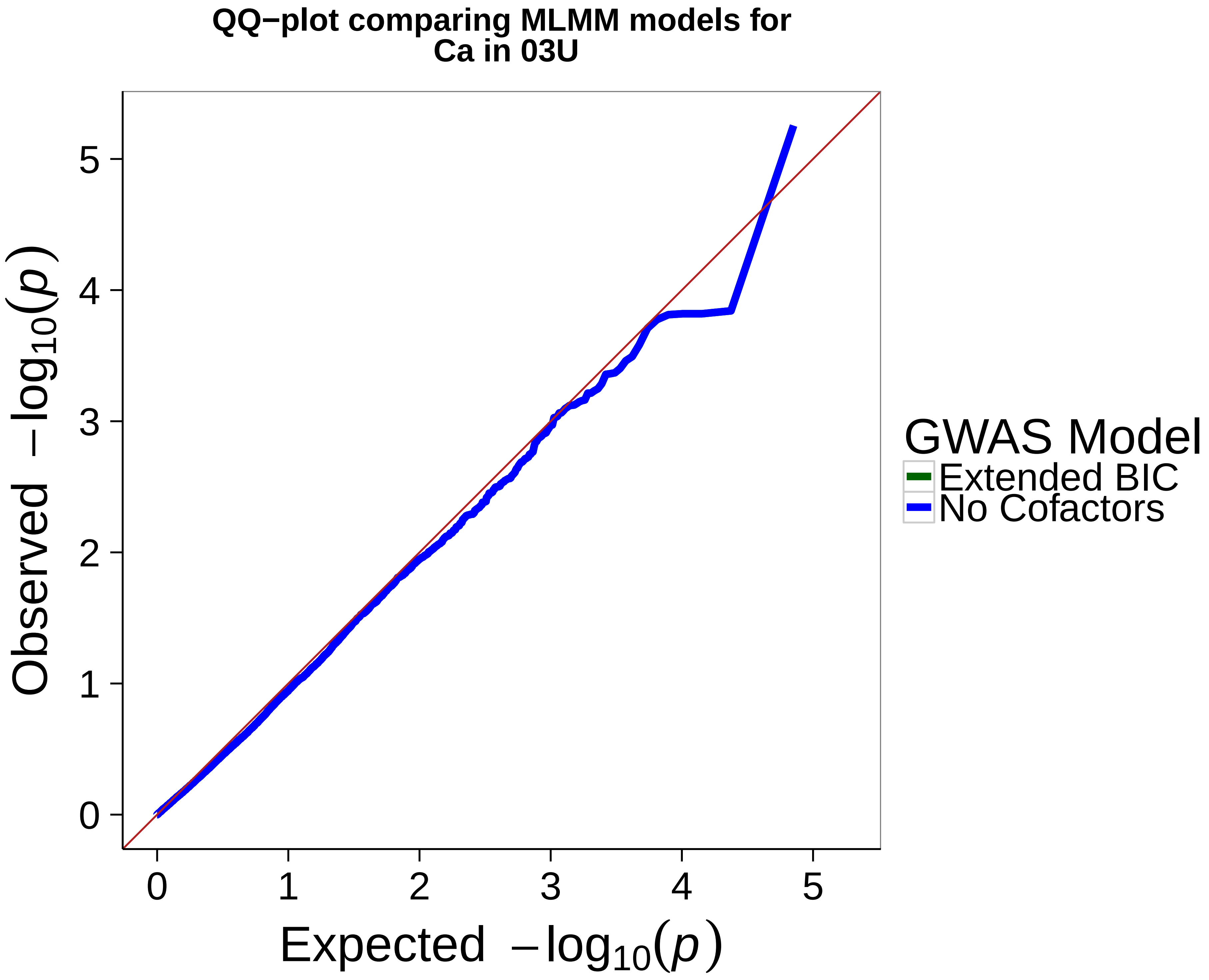
QQ−plot comparing MLMM models for
Ca in 03U
Observed −log10(p)
Expected −log10(p)
0
1
2
3
4
5
GWAS Model
Extended BIC
No Cofactors

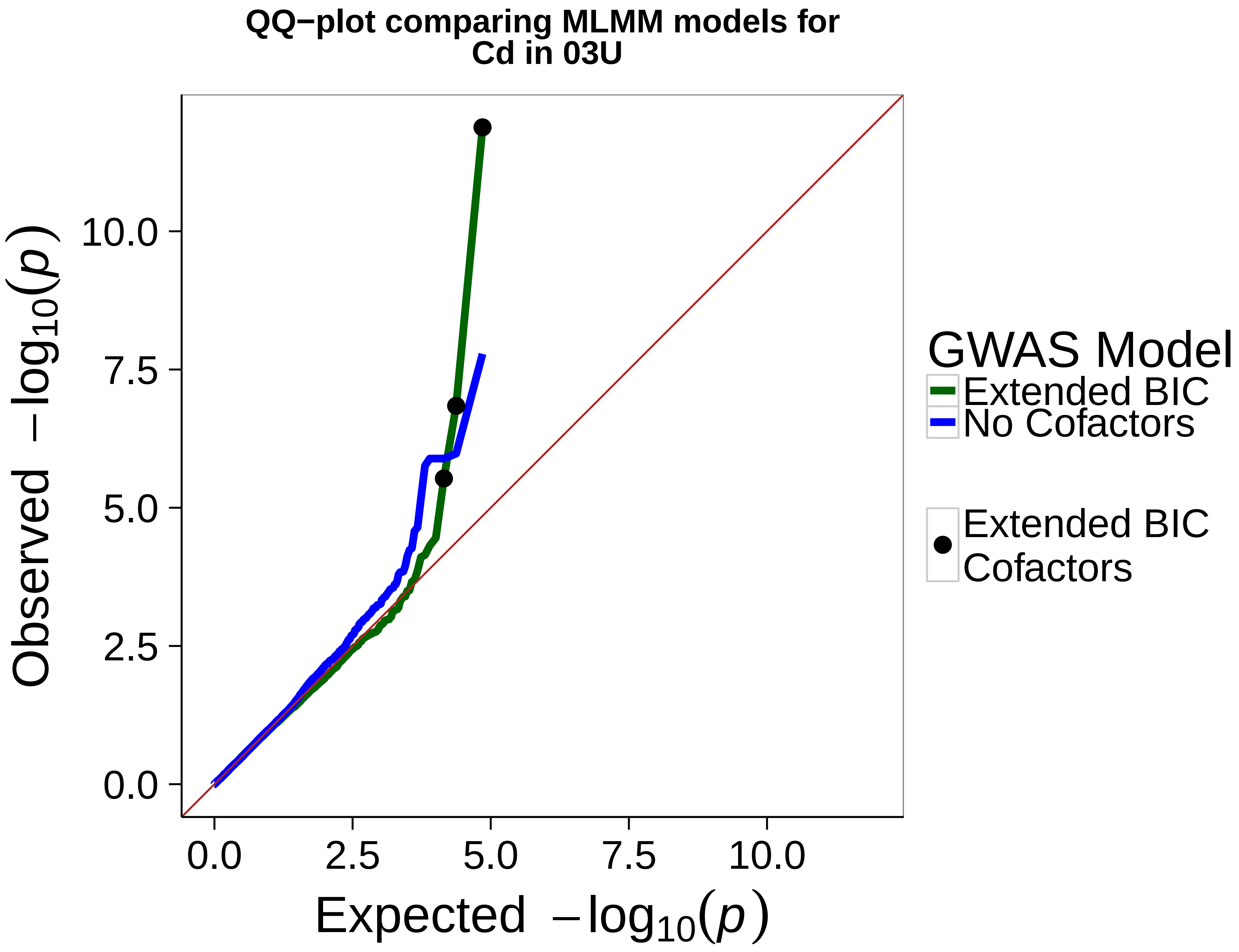
QQ−plot comparing MLMM models for
Cd in 03U
Observed −log10(p)
Expected −log10(p)
0.0
2.5
5.0
7.5
10.0
GWAS Model
Extended BIC
No Cofactors
Extended BIC
Cofactors

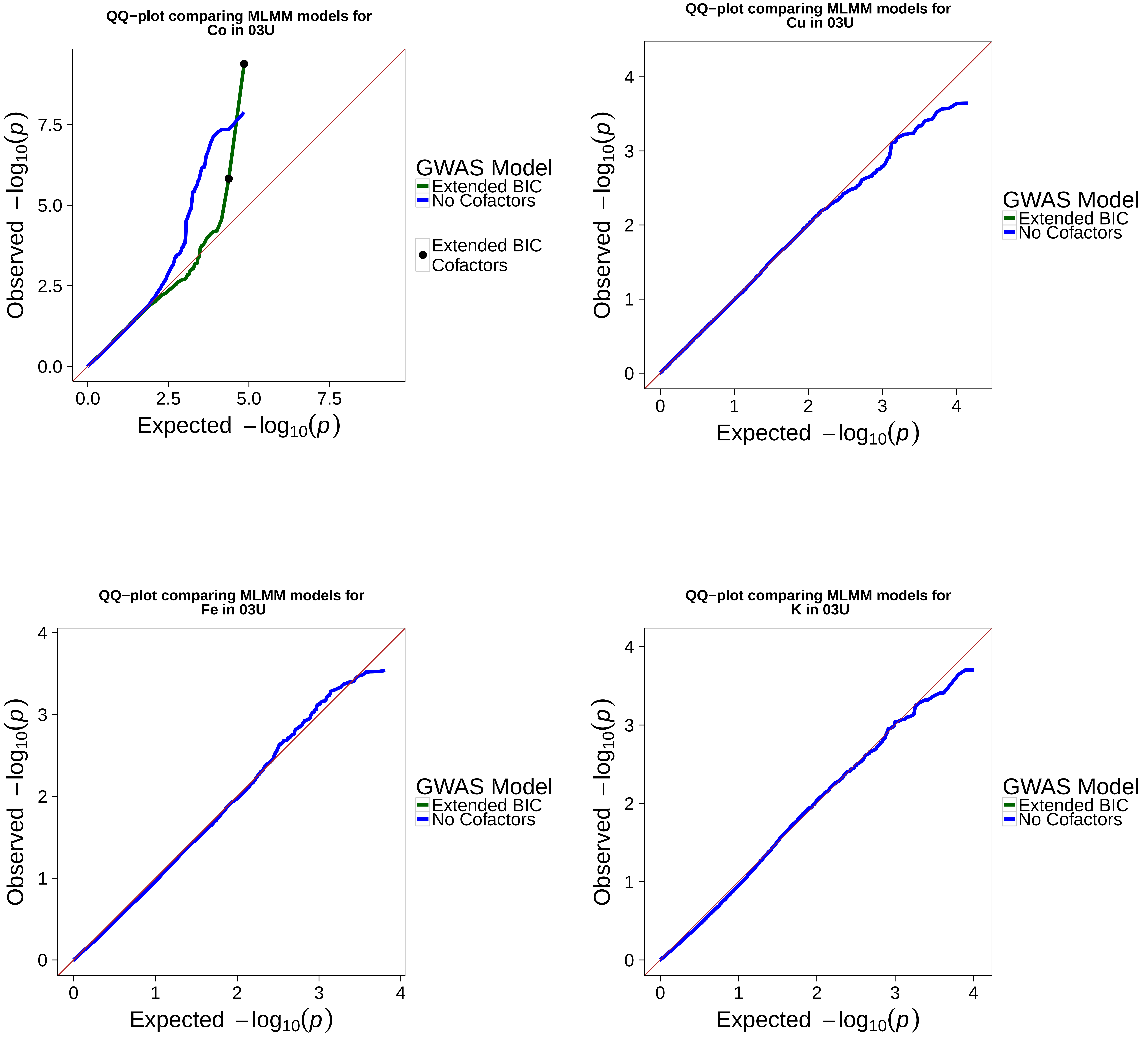
QQ−plot comparing MLMM models for
Co in 03U
Observed −log10(p)
Expected −log10(p)
0.0
2.5
5.0
7.5
GWAS Model
Extended BIC
No Cofactors
Extended BIC
Cofactors
QQ−plot comparing MLMM models for
Cu in 03U
Observed −log10(p)
Expected −log10(p)
0
1
2
3
4
GWAS Model
Extended BIC
No Cofactors
QQ−plot comparing MLMM models for
Fe in 03U
Observed −log10(p)
Expected −log10(p)
GWAS Model
Extended BIC
No Cofactors
QQ−plot comparing MLMM models for
K in 03U
Observed −log10(p)
Expected −log10(p)
GWAS Model
Extended BIC
No Cofactors

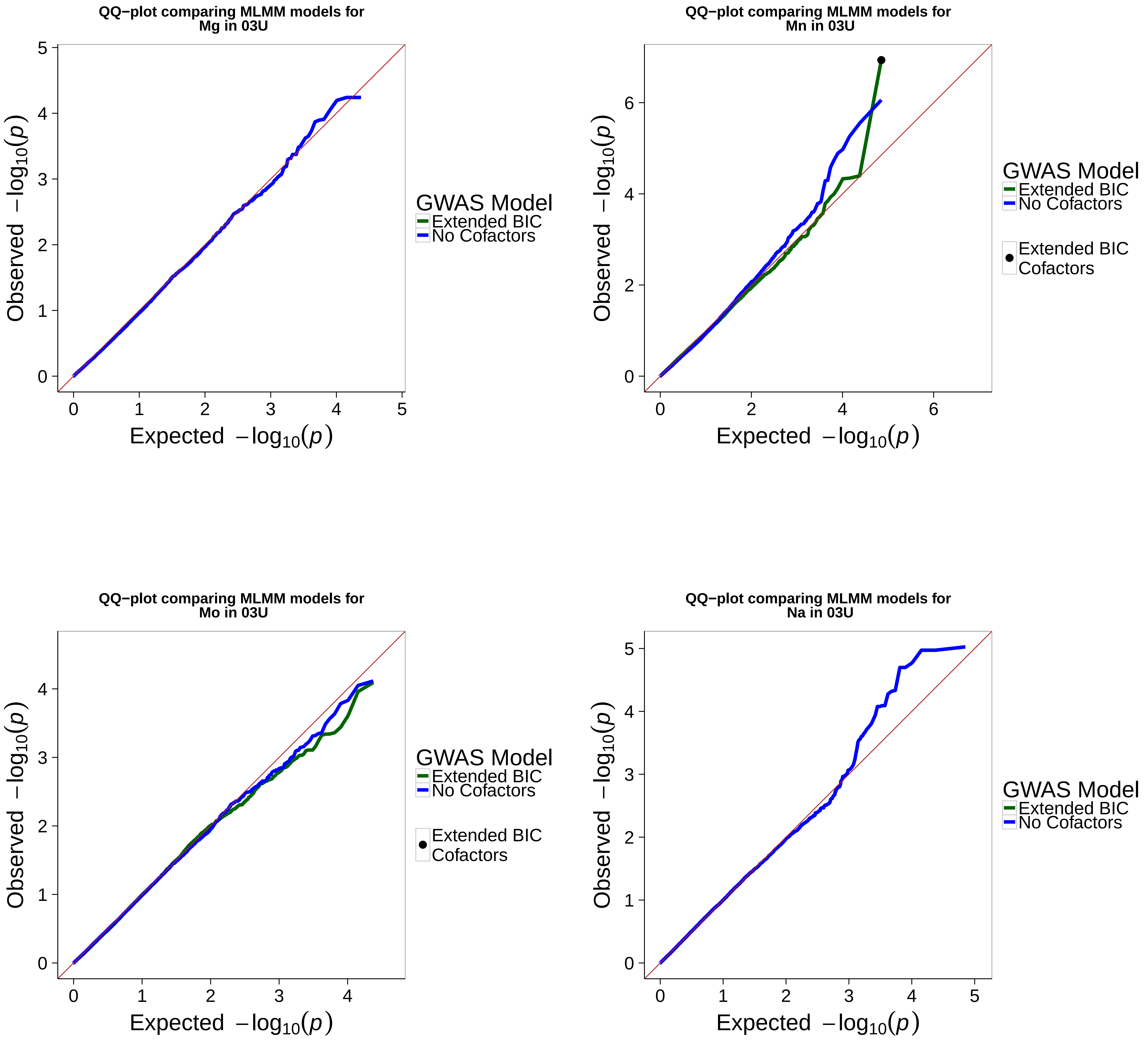

QQ−plot comparing MLMM models for
Mg in 03U
Observed −log10(p)
Expected −log10(p)
GWAS Model
Extended BIC
No Cofactors
QQ−plot comparing MLMM models for
Mn in 03U
Observed −log10(p)
Expected −log10(p)
GWAS Model
Extended BIC
No Cofactors
Extended BIC
Cofactors
QQ−plot comparing MLMM models for
Mo in 03U
Observed −log10(p)
Expected −log10(p)
GWAS Model
Extended BIC
No Cofactors
Extended BIC
Cofactors
QQ−plot comparing MLMM models for
Na in 03U
Observed −log10(p)
Expected −log10(p)
GWAS Model
Extended BIC
No Cofactors

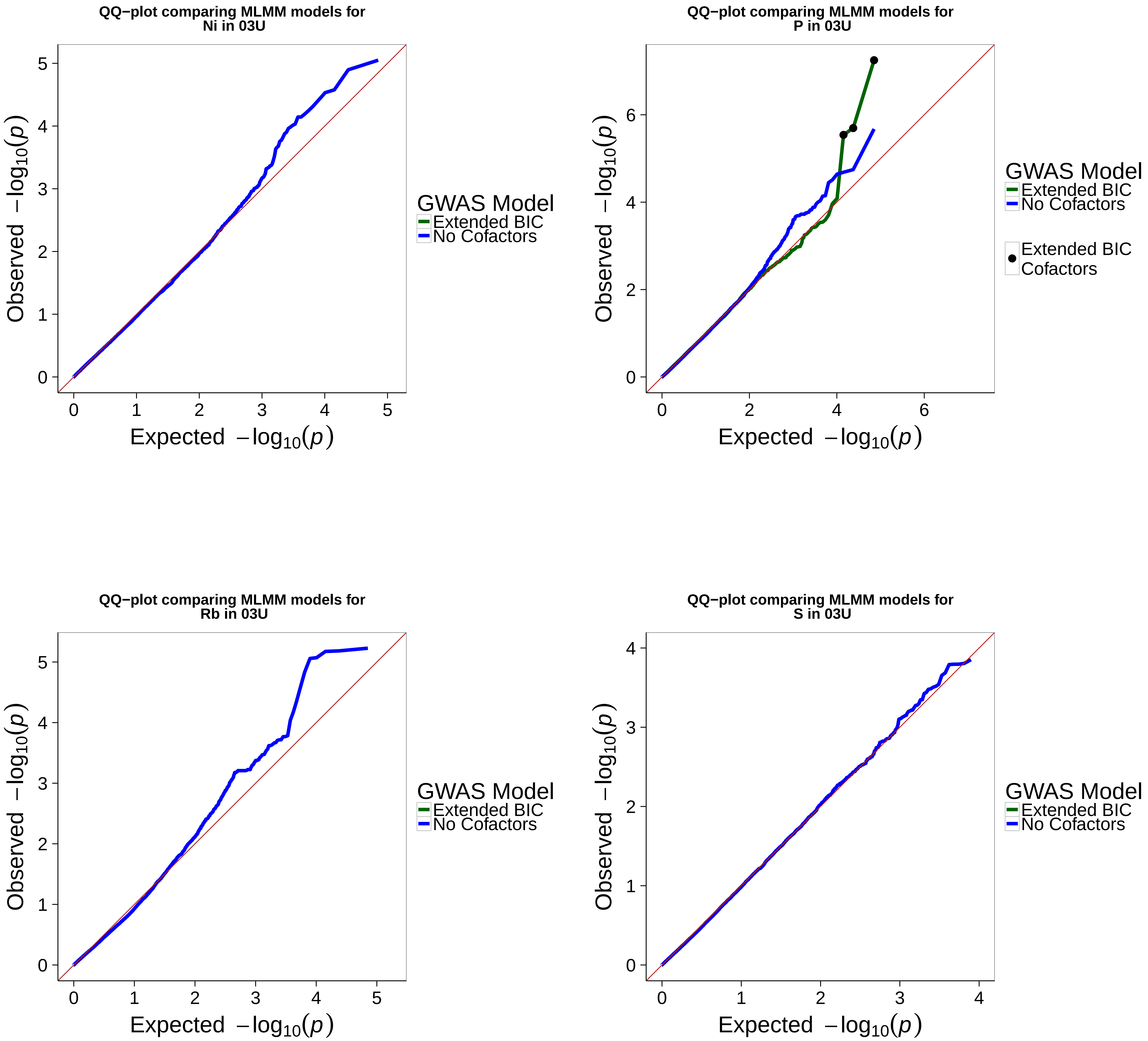
QQ−plot comparing MLMM models for
Ni in 03U
Observed −log$_{10}$(p)
Expected −log$_{10}$(p)
0
1
2
3
4
5
GWAS Model
Extended BIC
No Cofactors
QQ−plot comparing MLMM models for
P in 03U
Observed −log$_{10}$(p)
Expected −log$_{10}$(p)
0
2
4
6
GWAS Model
Extended BIC
No Cofactors
Extended BIC
Cofactors
QQ−plot comparing MLMM models for
Rb in 03U
Observed −log$_{10}$(p)
Expected −log$_{10}$(p)
0
1
2
3
4
5
GWAS Model
Extended BIC
No Cofactors
QQ−plot comparing MLMM models for
S in 03U
Observed −log$_{10}$(p)
Expected −log$_{10}$(p)
0
1
2
3
4
GWAS Model
Extended BIC
No Cofactors

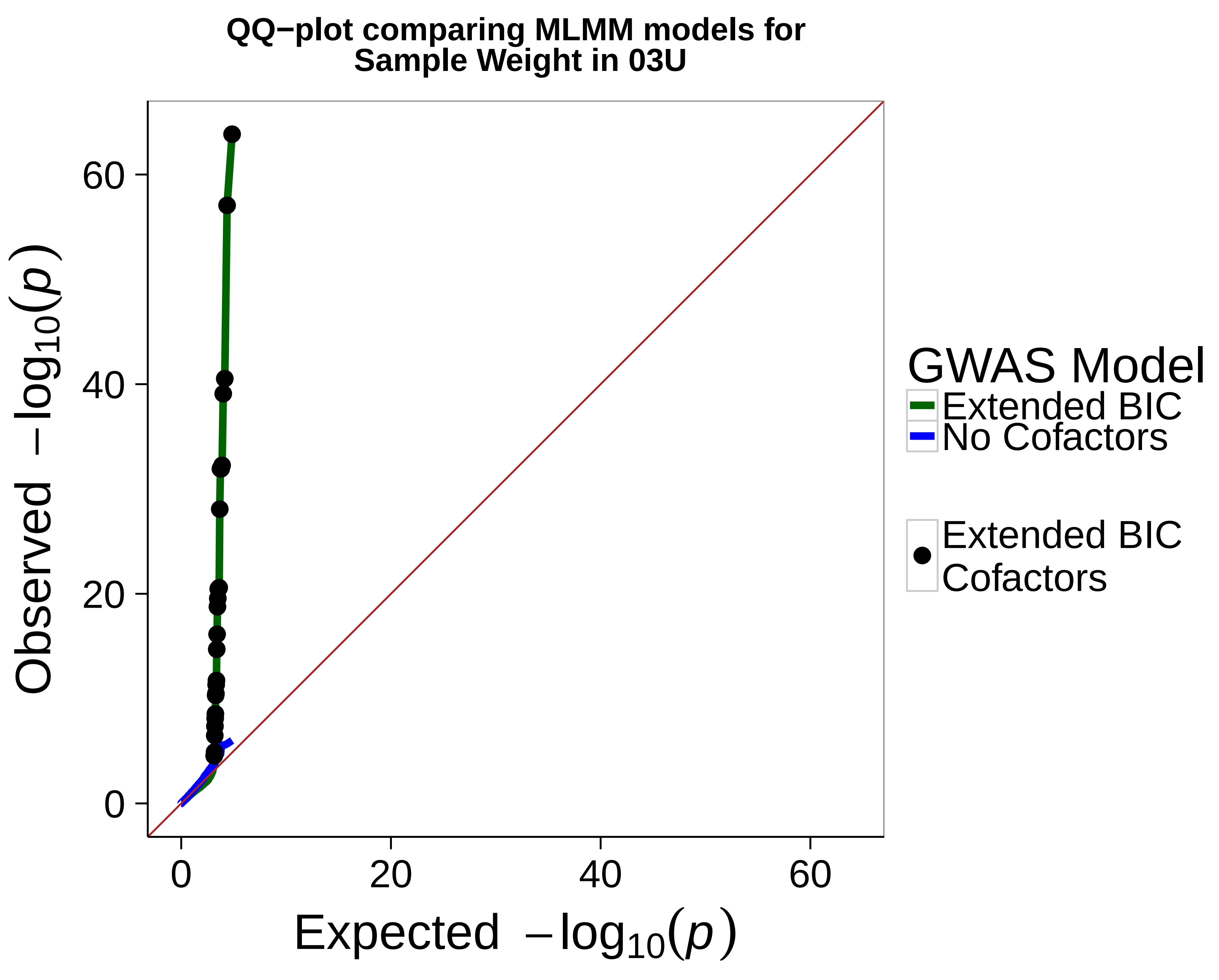
QQ−plot comparing MLMM models for
Sample Weight in 03U
Observed −log10(p)
Expected −log10(p)
0
20
40
60
GWAS Model
Extended BIC
No Cofactors
Extended BIC
Cofactors

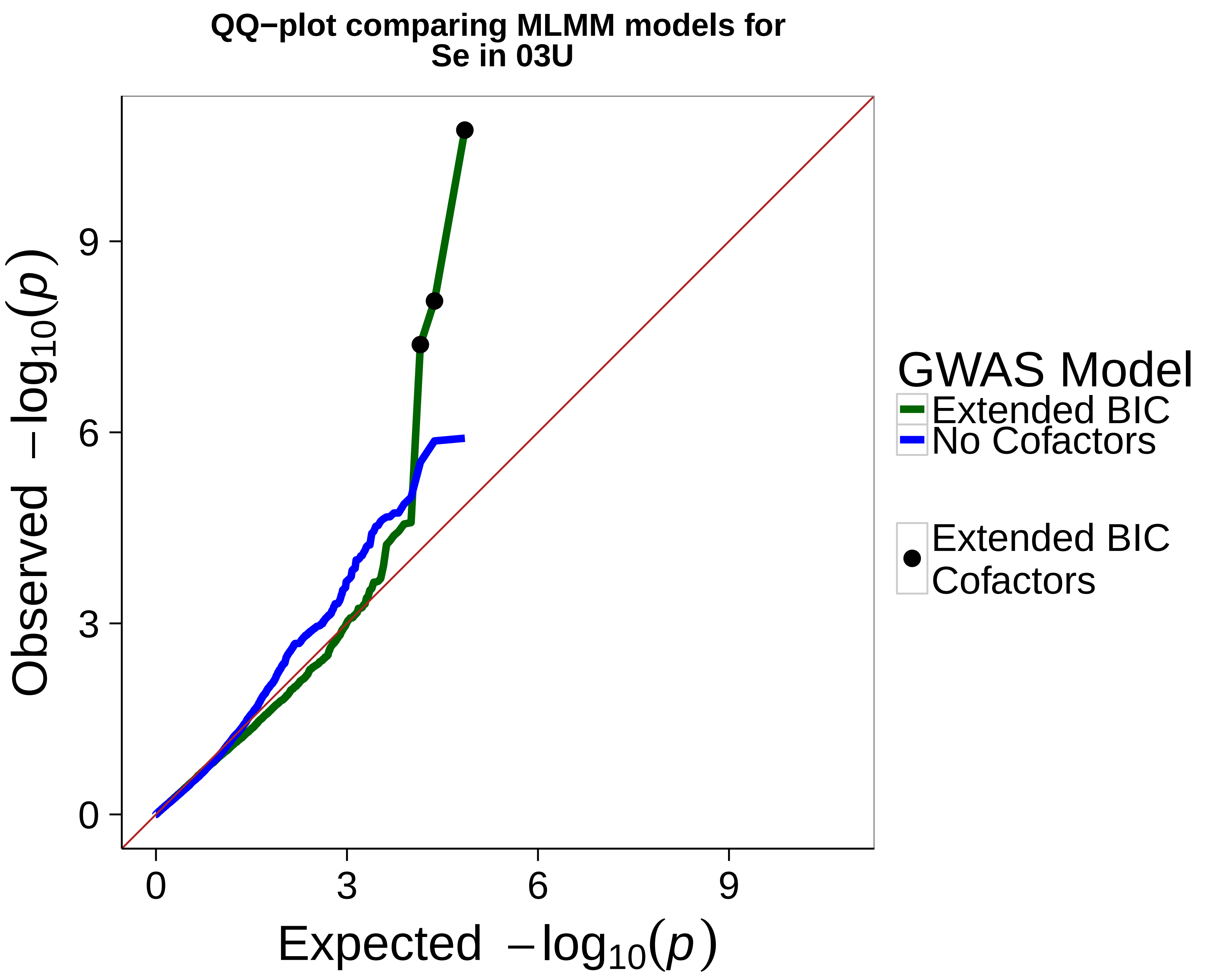
QQ−plot comparing MLMM models for
Se in 03U
Observed −log10(p)
Expected −log10(p)
0
3
6
9
GWAS Model
Extended BIC
No Cofactors
Extended BIC
Cofactors

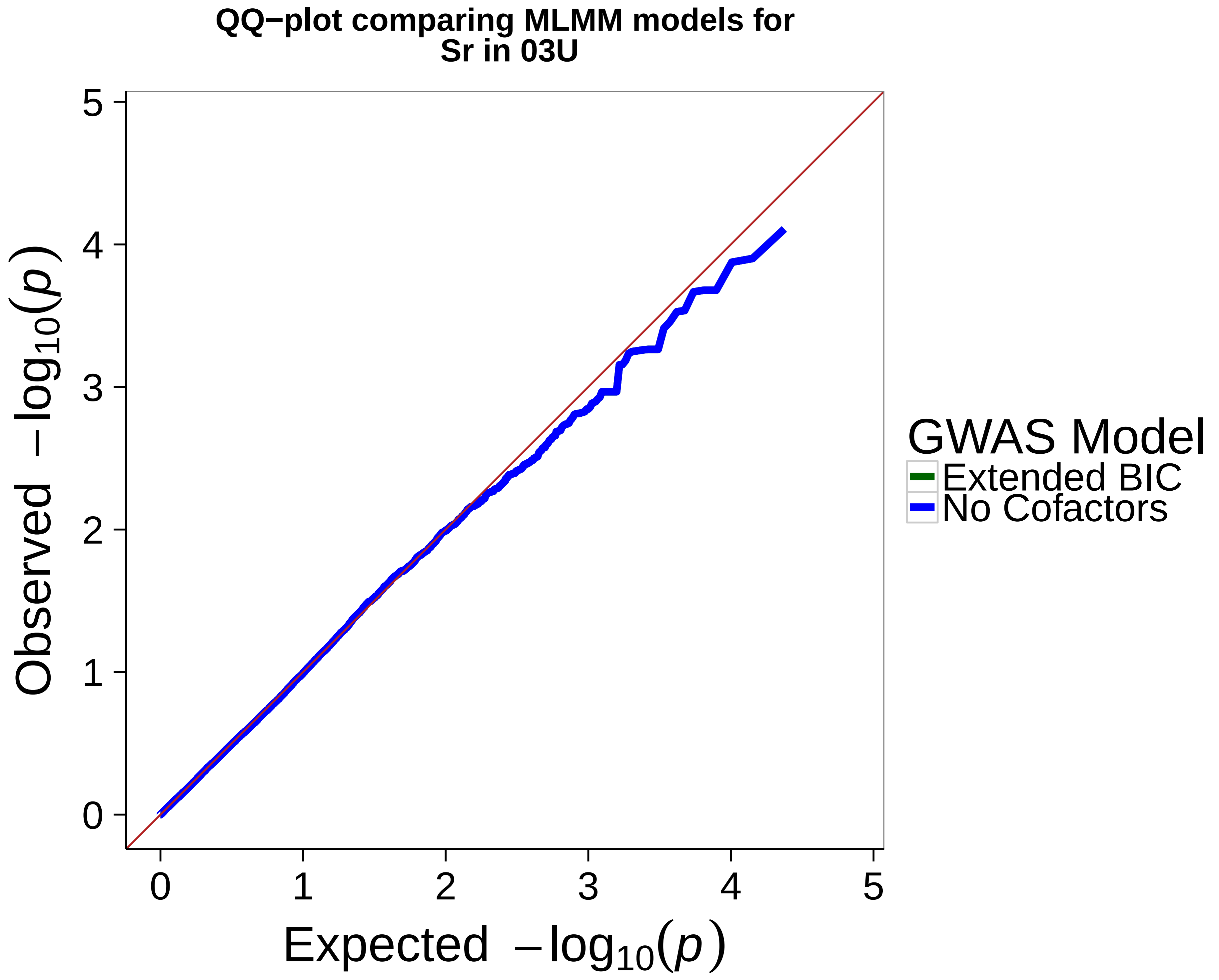
QQ−plot comparing MLMM models for
Sr in 03U
Observed −log10(p)
Expected −log10(p)
0
1
2
3
4
5
GWAS Model
Extended BIC
No Cofactors

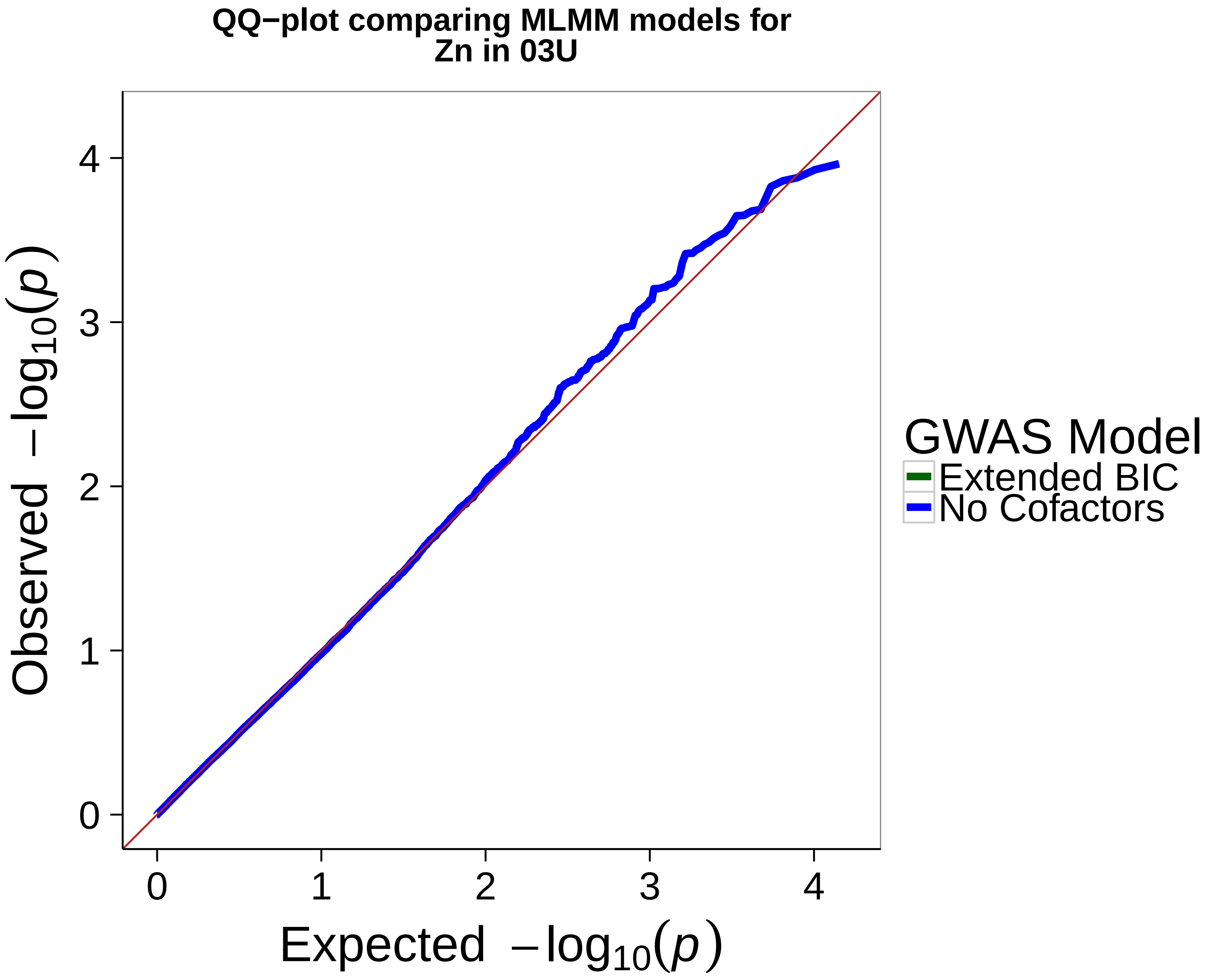
QQ−plot comparing MLMM models for
Zn in 03U
Observed −log10(p)
Expected −log10(p)
0
1
2
3
4
GWAS Model
Extended BIC
No Cofactors

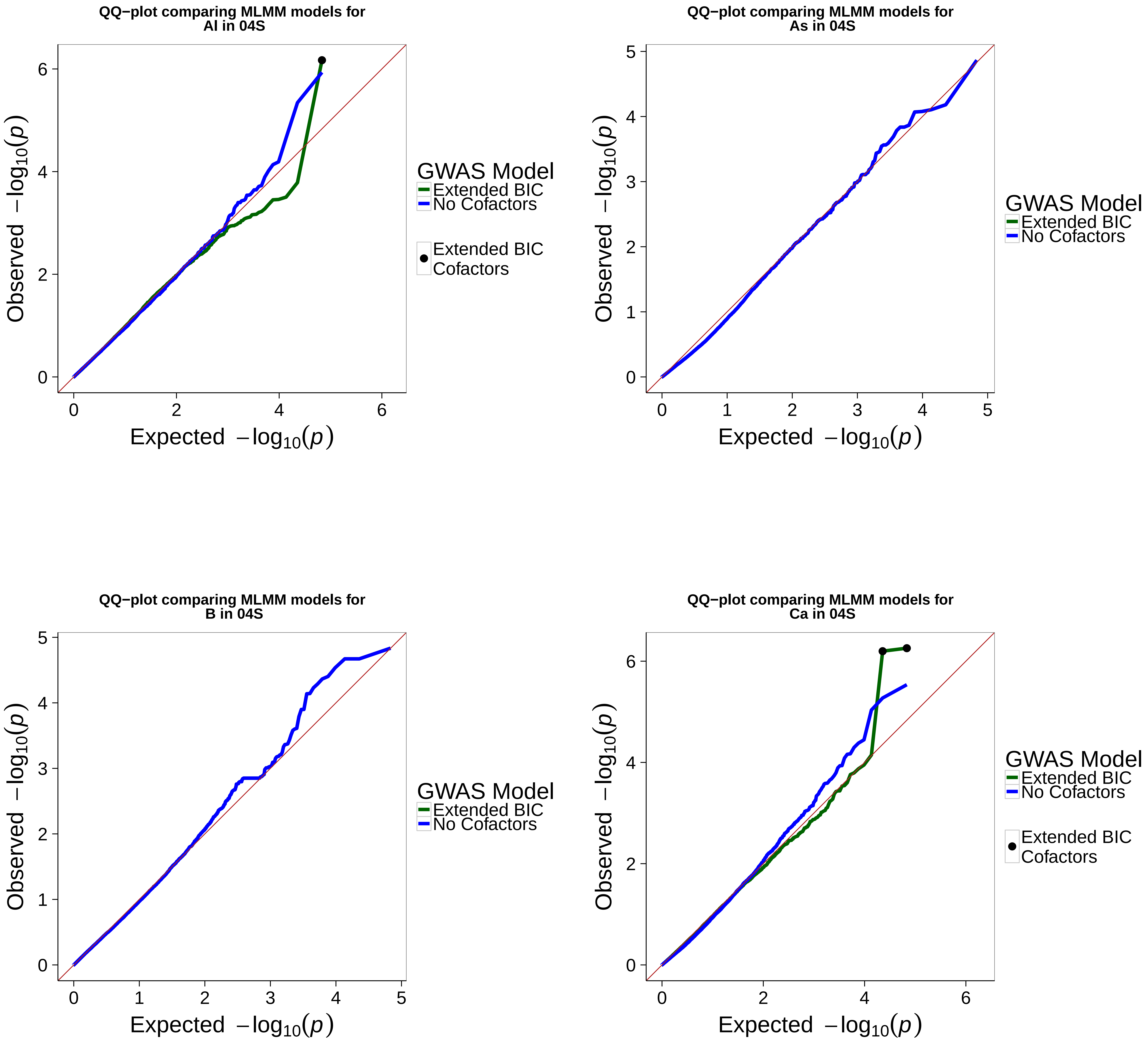

QQ−plot comparing MLMM models for
Al in 04S
Observed −log10(p)
Expected −log10(p)
GWAS Model
Extended BIC
No Cofactors
Extended BIC
Cofactors
QQ−plot comparing MLMM models for
As in 04S
Observed −log10(p)
Expected −log10(p)
GWAS Model
Extended BIC
No Cofactors
QQ−plot comparing MLMM models for
B in 04S
Observed −log10(p)
Expected −log10(p)
GWAS Model
Extended BIC
No Cofactors
QQ−plot comparing MLMM models for
Ca in 04S
Observed −log10(p)
Expected −log10(p)
GWAS Model
Extended BIC
No Cofactors
Extended BIC
Cofactors

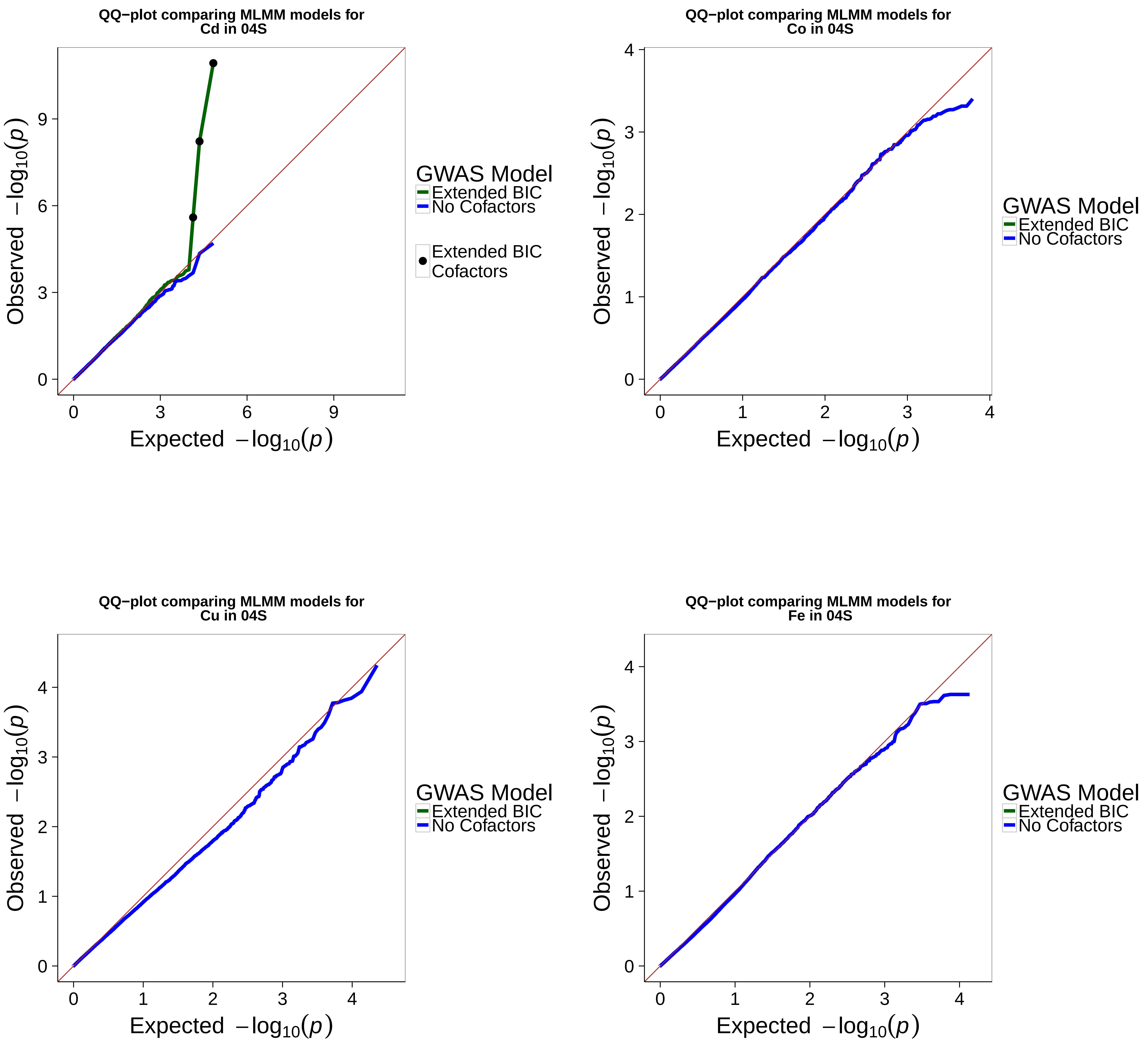
QQ−plot comparing MLMM models for
Cd in 04S
Observed −log10(p)
Expected −log10(p)
GWAS Model
Extended BIC
No Cofactors
Extended BIC
Cofactors
QQ−plot comparing MLMM models for
Co in 04S
Observed −log10(p)
Expected −log10(p)
GWAS Model
Extended BIC
No Cofactors
QQ−plot comparing MLMM models for
Cu in 04S
Observed −log10(p)
Expected −log10(p)
GWAS Model
Extended BIC
No Cofactors
QQ−plot comparing MLMM models for
Fe in 04S
Observed −log10(p)
Expected −log10(p)
GWAS Model
Extended BIC
No Cofactors

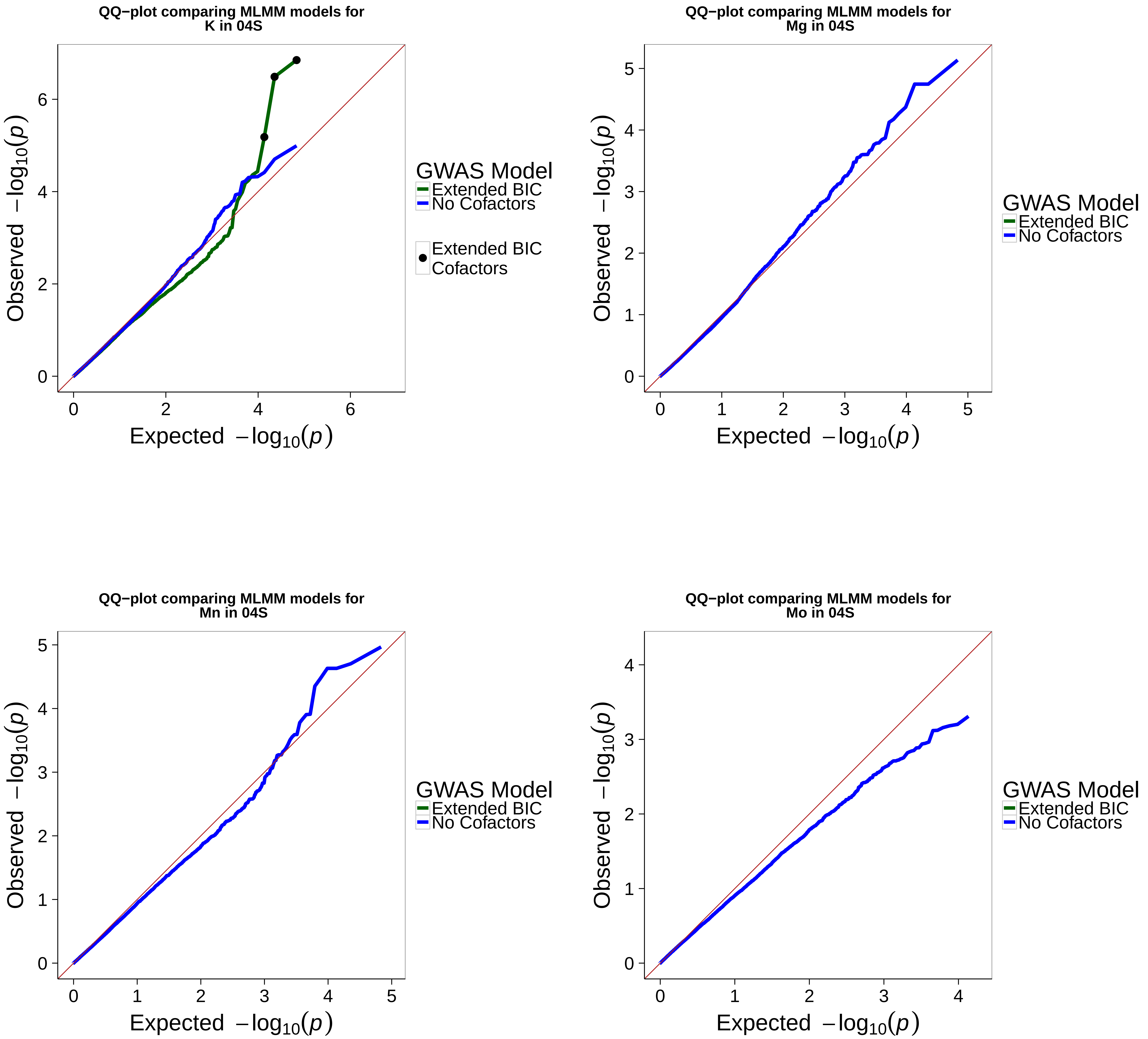
QQ−plot comparing MLMM models for
K in 04S
Observed −log10(p)
Expected −log10(p)
GWAS Model
Extended BIC
No Cofactors
Extended BIC
Cofactors
QQ−plot comparing MLMM models for
Mg in 04S
Observed −log10(p)
Expected −log10(p)
GWAS Model
Extended BIC
No Cofactors
QQ−plot comparing MLMM models for
Mn in 04S
Observed −log10(p)
Expected −log10(p)
GWAS Model
Extended BIC
No Cofactors
QQ−plot comparing MLMM models for
Mo in 04S
Observed −log10(p)
Expected −log10(p)
GWAS Model
Extended BIC
No Cofactors

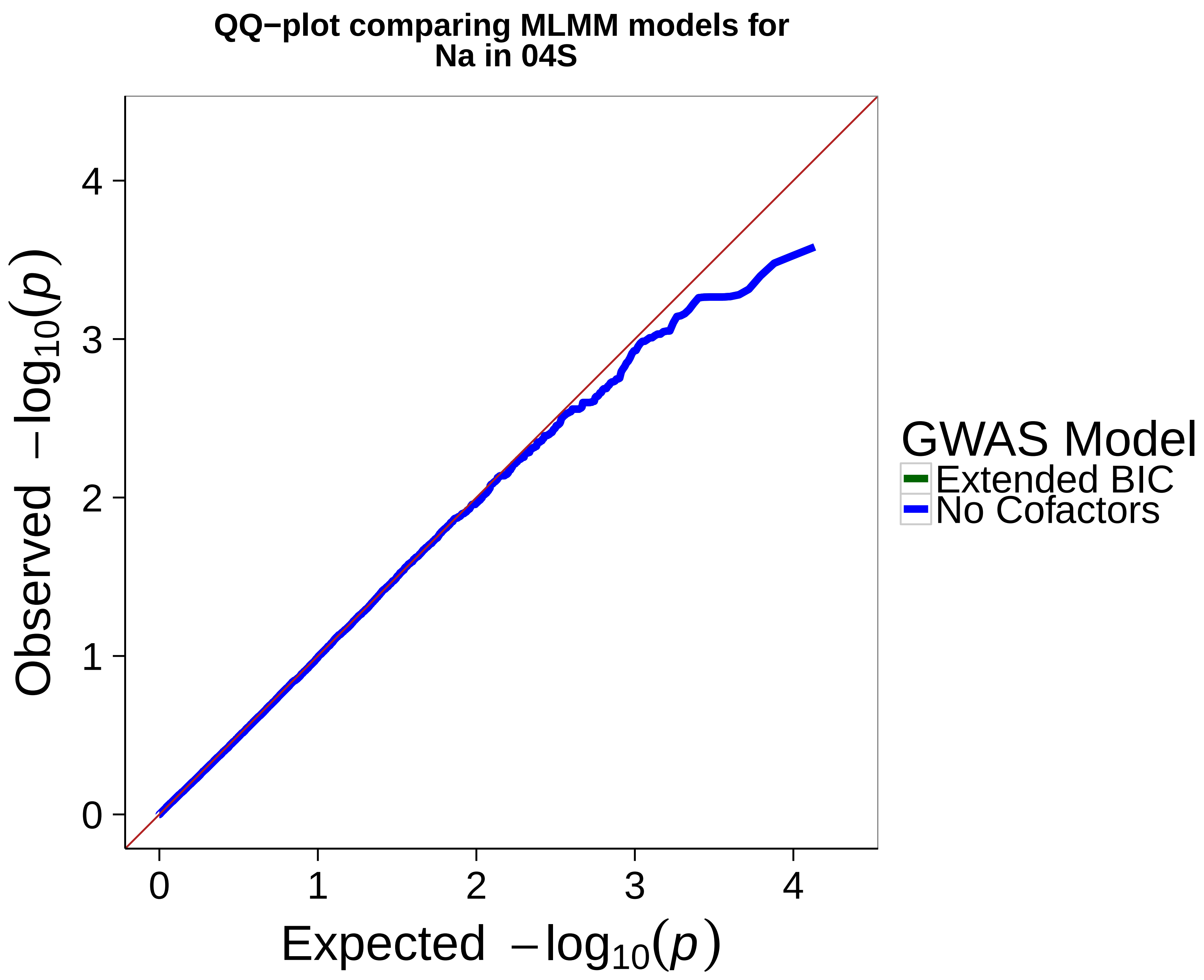
QQ−plot comparing MLMM models for
Na in 04S
Observed −log10(p)
Expected −log10(p)
GWAS Model
Extended BIC
No Cofactors

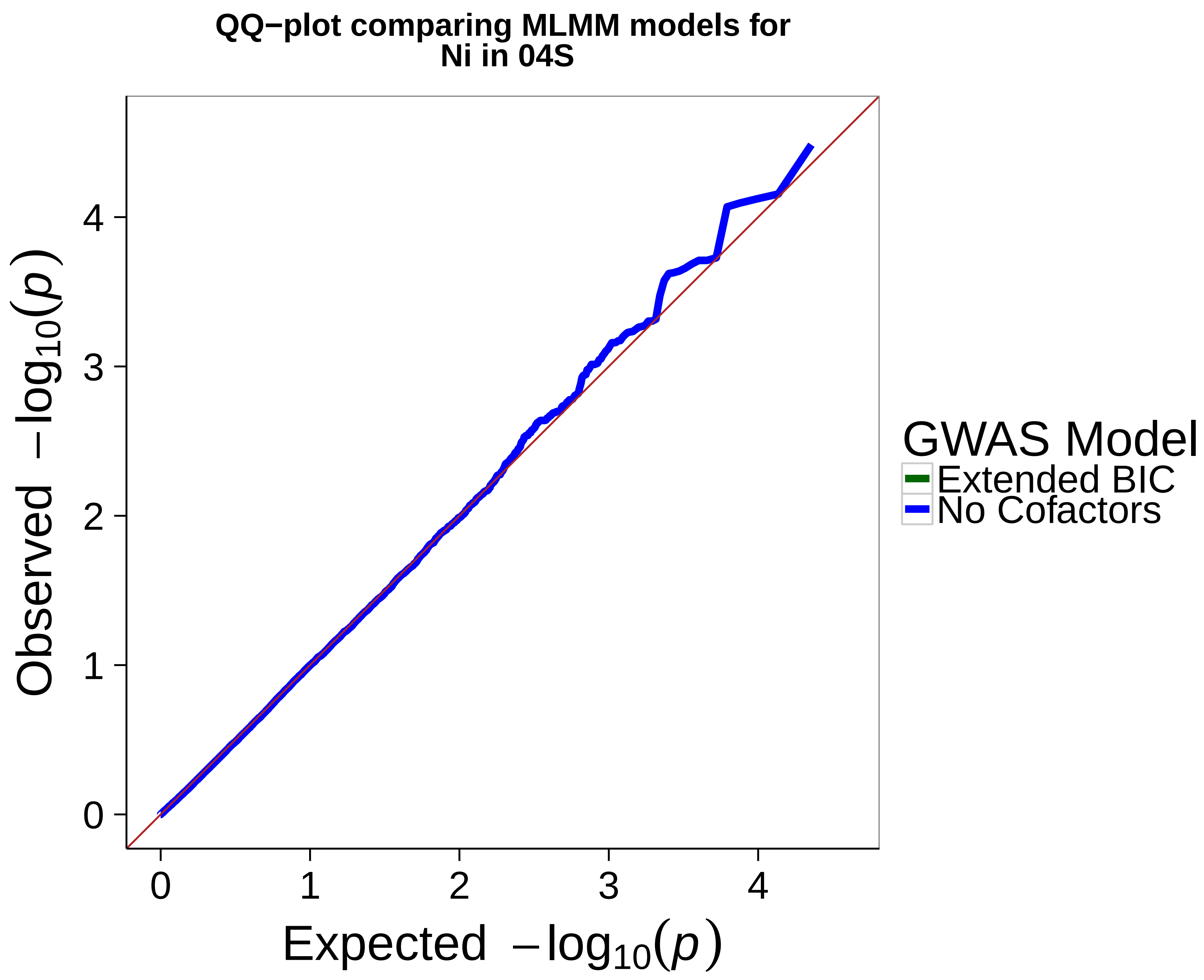
QQ−plot comparing MLMM models for
Ni in 04S
Observed −log10(p)
Expected −log10(p)
GWAS Model
Extended BIC
No Cofactors

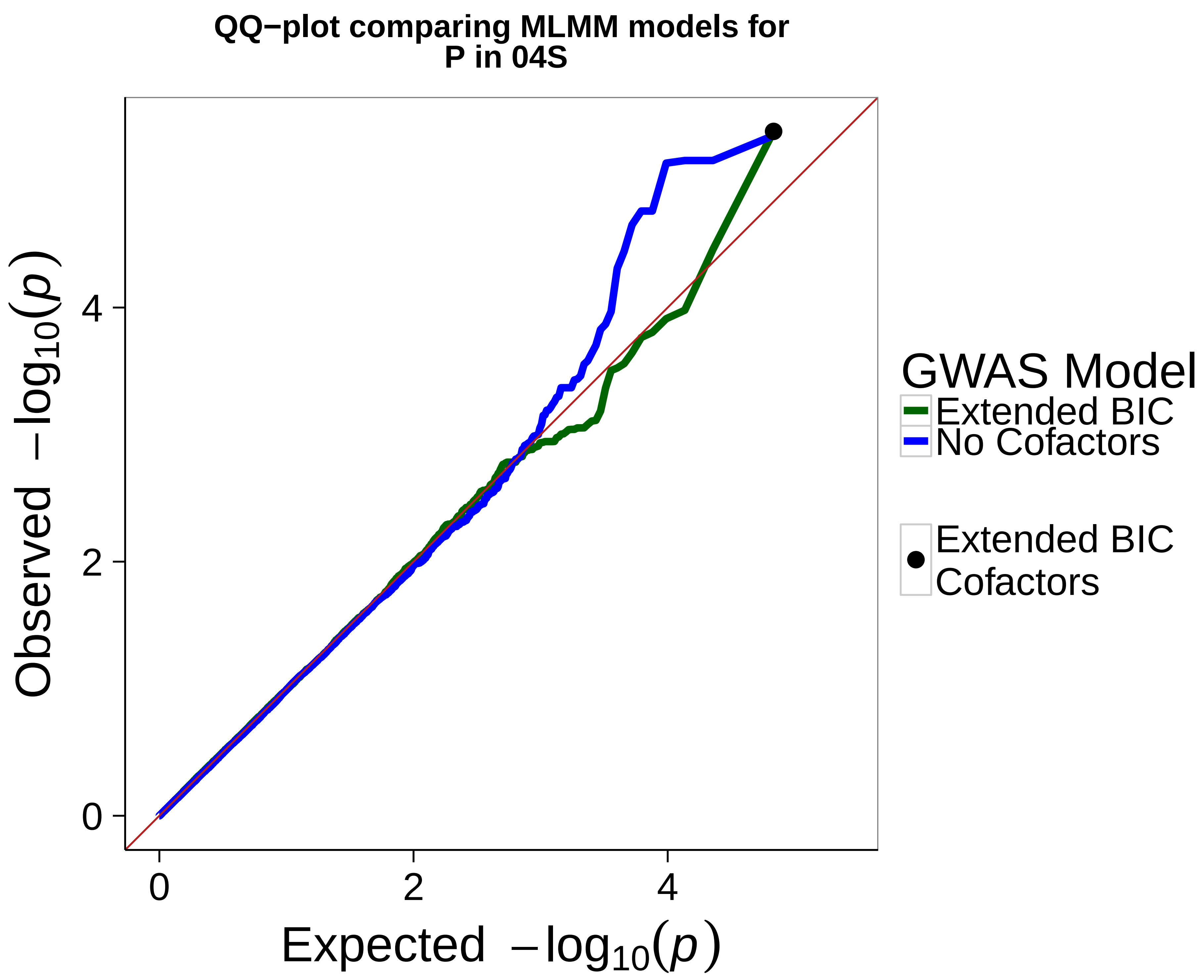
QQ−plot comparing MLMM models for
P in 04S
Observed −log10(p)
Expected −log10(p)
GWAS Model
Extended BIC
No Cofactors
Extended BIC
Cofactors

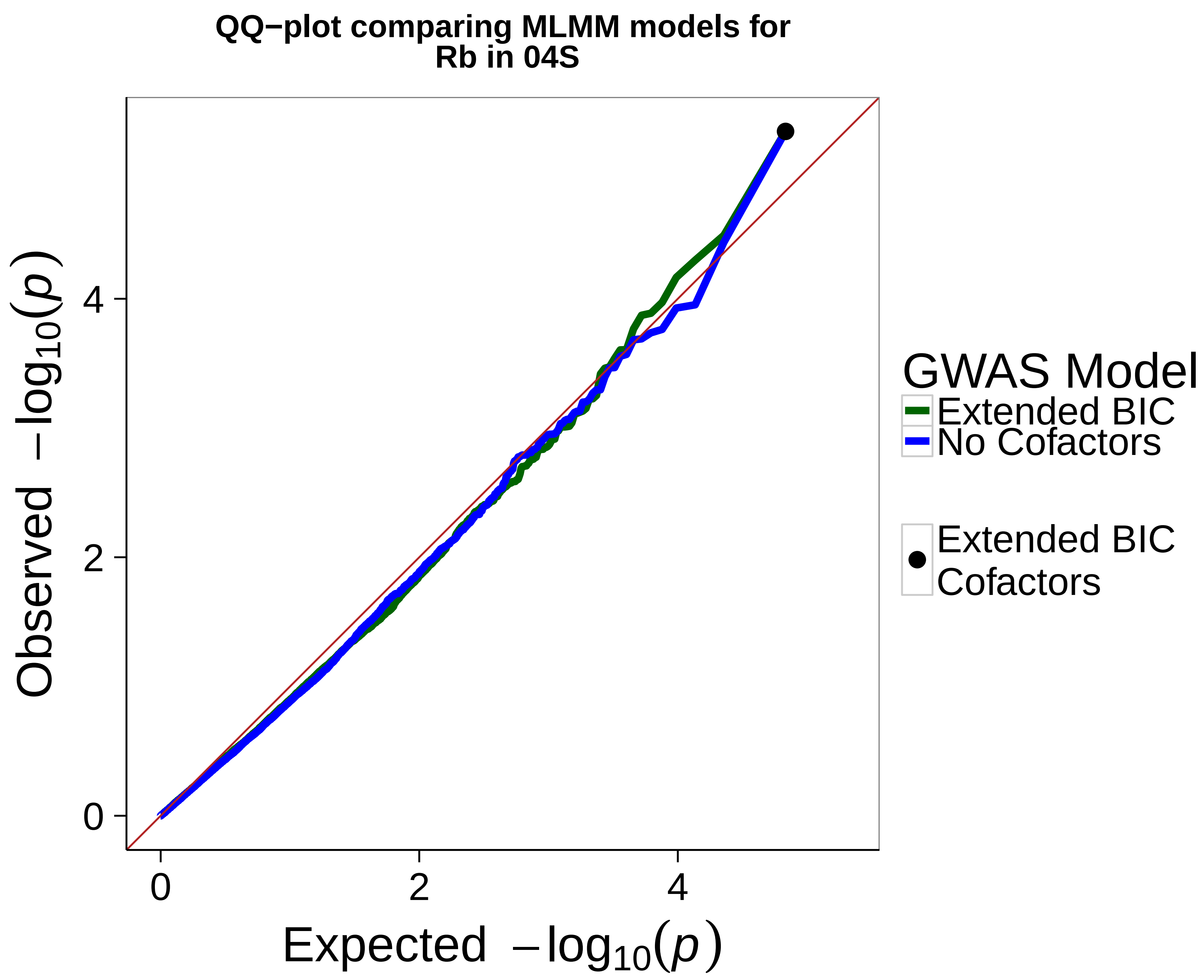
QQ−plot comparing MLMM models for
Rb in 04S
Observed −log10(p)
Expected −log10(p)
GWAS Model
Extended BIC
No Cofactors
Extended BIC
Cofactors

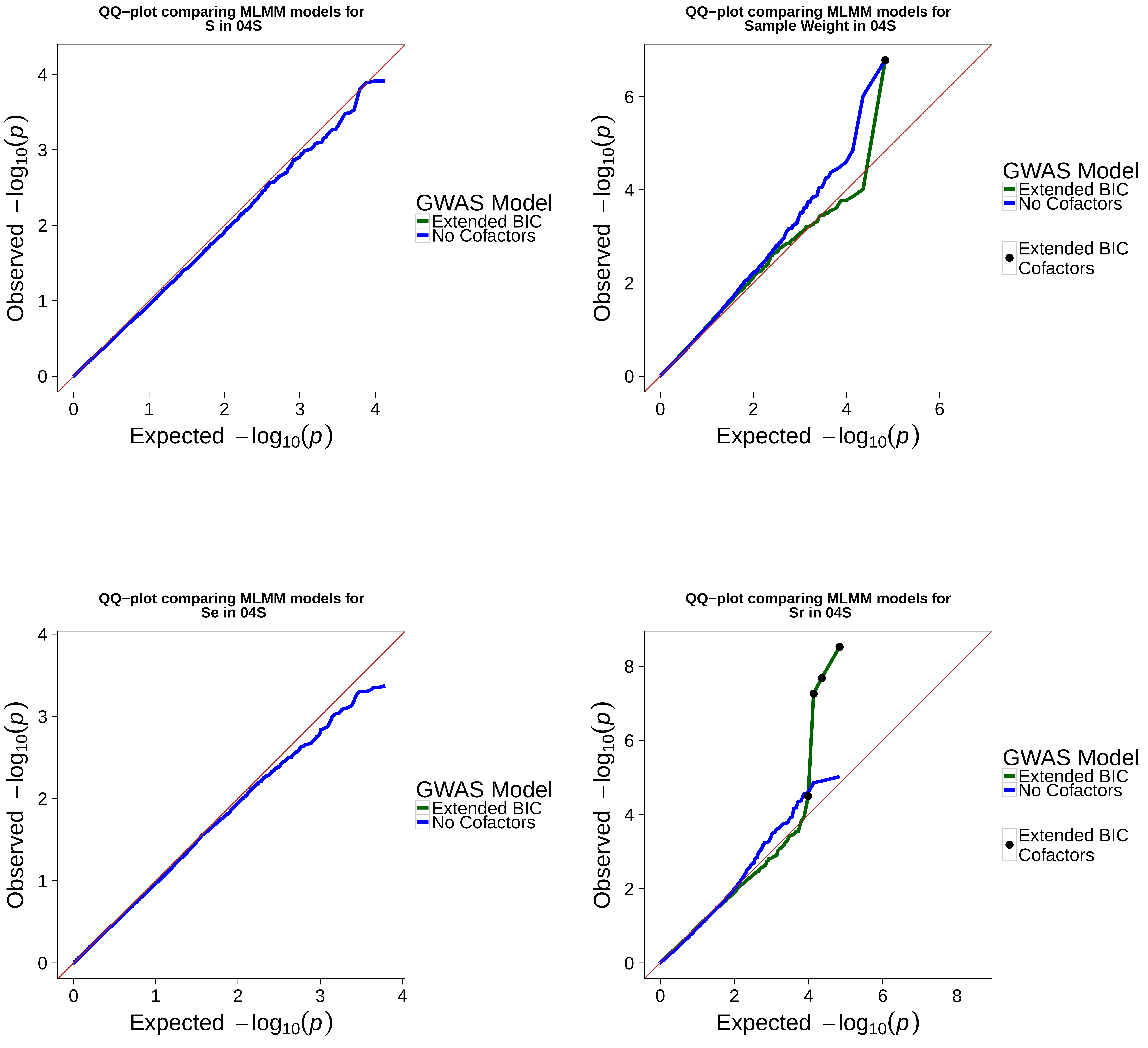
QQ−plot comparing MLMM models for
S in 04S
Observed −log10(p)
Expected −log10(p)
GWAS Model
Extended BIC
No Cofactors
QQ−plot comparing MLMM models for
Sample Weight in 04S
Observed −log10(p)
Expected −log10(p)
GWAS Model
Extended BIC
No Cofactors
Extended BIC
Cofactors
QQ−plot comparing MLMM models for
Se in 04S
Observed −log10(p)
Expected −log10(p)
GWAS Model
Extended BIC
No Cofactors
QQ−plot comparing MLMM models for
Sr in 04S
Observed −log10(p)
Expected −log10(p)
GWAS Model
Extended BIC
No Cofactors
Extended BIC
Cofactors

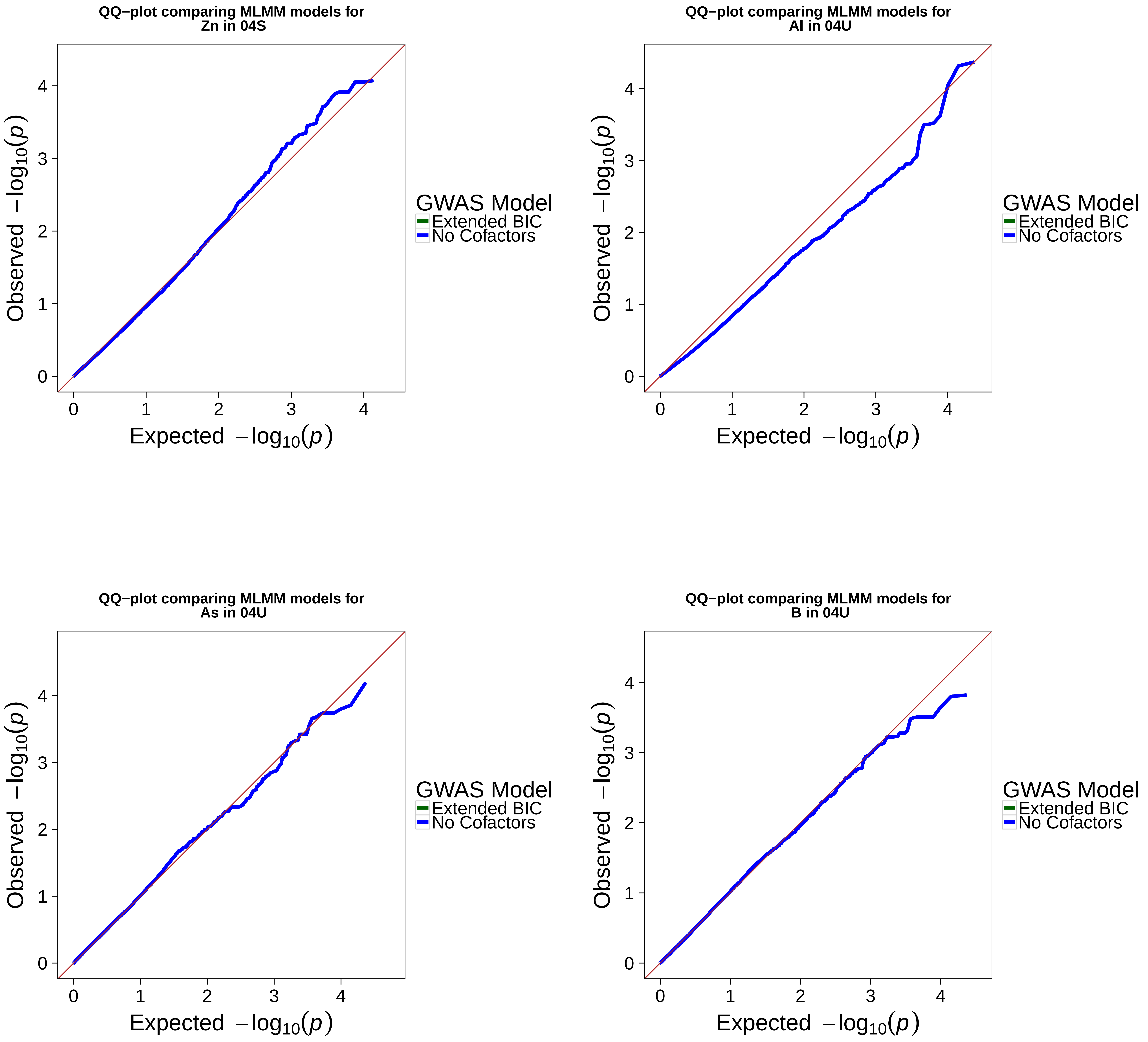
QQ−plot comparing MLMM models for
Zn in 04S
QQ−plot comparing MLMM models for
Al in 04U
QQ−plot comparing MLMM models for
As in 04U
QQ−plot comparing MLMM models for
B in 04U
Observed $-\log_{10}(p)$
Expected $-\log_{10}(p)$
GWAS Model
Extended BIC
No Cofactors
0
1
2
3
4

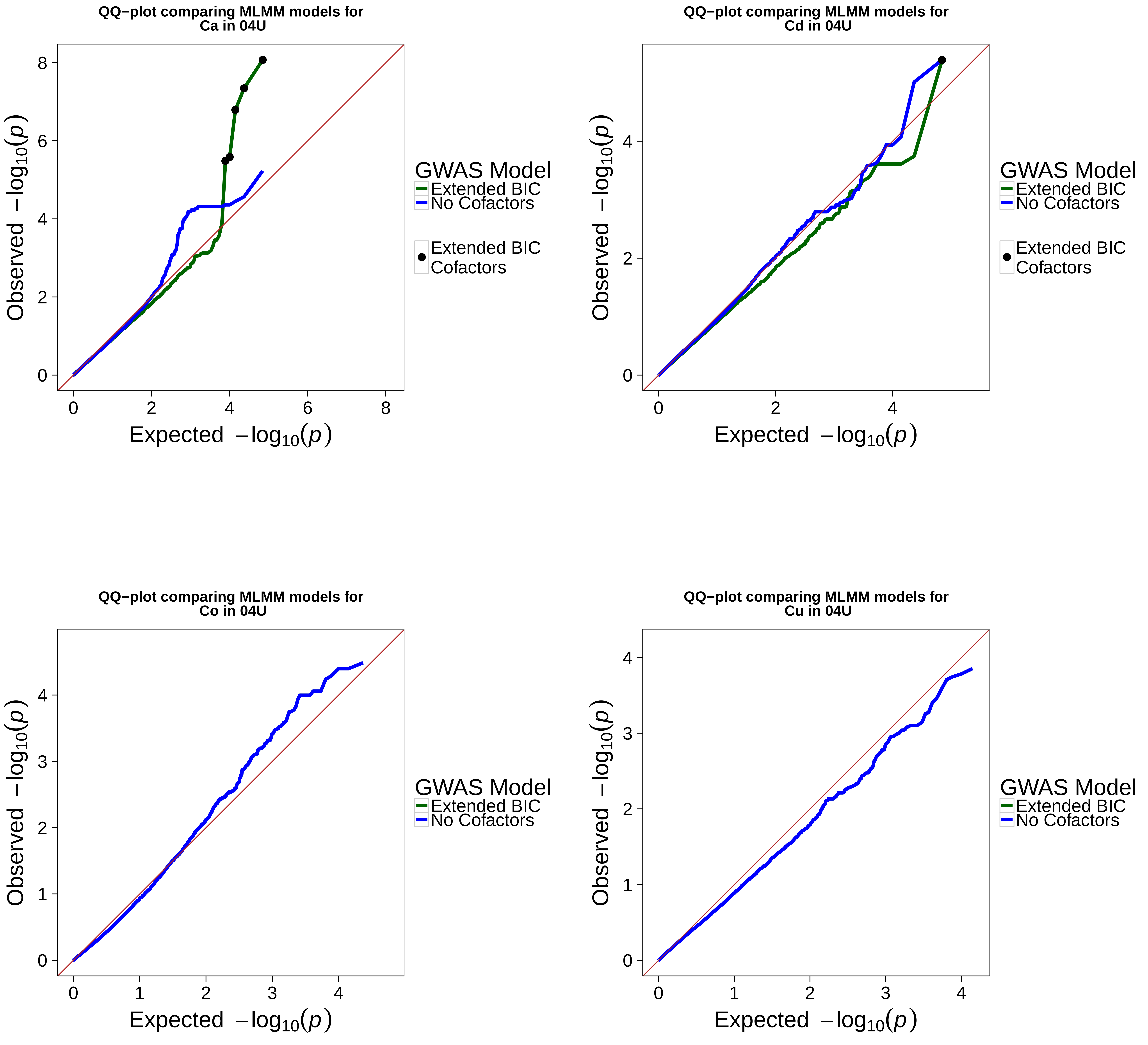
QQ−plot comparing MLMM models for
Ca in 04U
Observed −log10(p)
Expected −log10(p)
GWAS Model
Extended BIC
No Cofactors
Extended BIC
Cofactors
QQ−plot comparing MLMM models for
Cd in 04U
Observed −log10(p)
Expected −log10(p)
GWAS Model
Extended BIC
No Cofactors
Extended BIC
Cofactors
QQ−plot comparing MLMM models for
Co in 04U
Observed −log10(p)
Expected −log10(p)
GWAS Model
Extended BIC
No Cofactors
QQ−plot comparing MLMM models for
Cu in 04U
Observed −log10(p)
Expected −log10(p)
GWAS Model
Extended BIC
No Cofactors

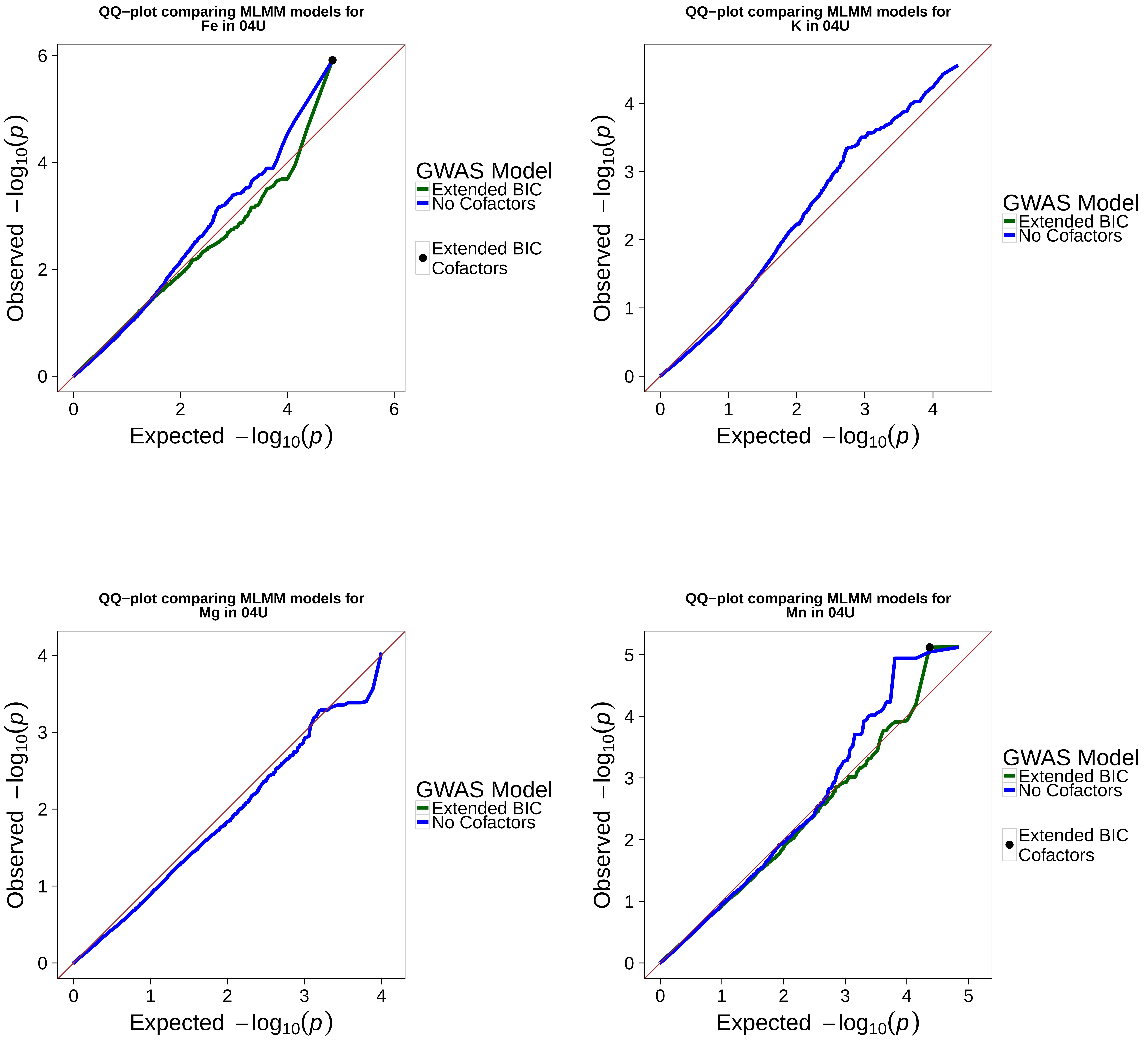
QQ−plot comparing MLMM models for
Fe in 04U
Observed −log10(p)
Expected −log10(p)
GWAS Model
Extended BIC
No Cofactors
Extended BIC
Cofactors
QQ−plot comparing MLMM models for
K in 04U
Observed −log10(p)
Expected −log10(p)
GWAS Model
Extended BIC
No Cofactors
QQ−plot comparing MLMM models for
Mg in 04U
Observed −log10(p)
Expected −log10(p)
GWAS Model
Extended BIC
No Cofactors
QQ−plot comparing MLMM models for
Mn in 04U
Observed −log10(p)
Expected −log10(p)
GWAS Model
Extended BIC
No Cofactors
Extended BIC
Cofactors

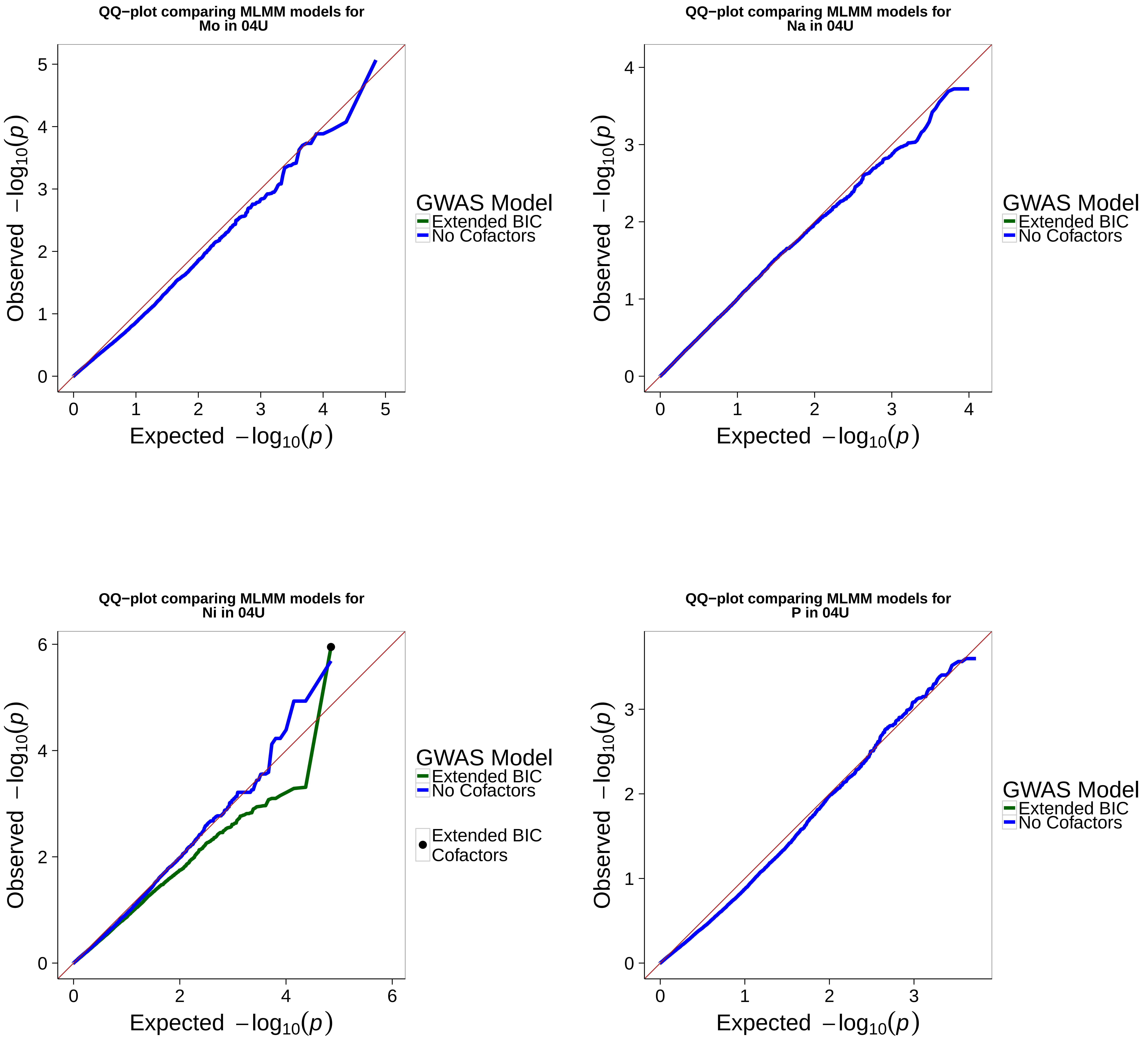

QQ−plot comparing MLMM models for
Mo in 04U
QQ−plot comparing MLMM models for
Na in 04U
QQ−plot comparing MLMM models for
Ni in 04U
QQ−plot comparing MLMM models for
P in 04U
Observed $-\log_{10}(p)$
Expected $-\log_{10}(p)$
GWAS Model
Extended BIC
No Cofactors
Extended BIC
Cofactors

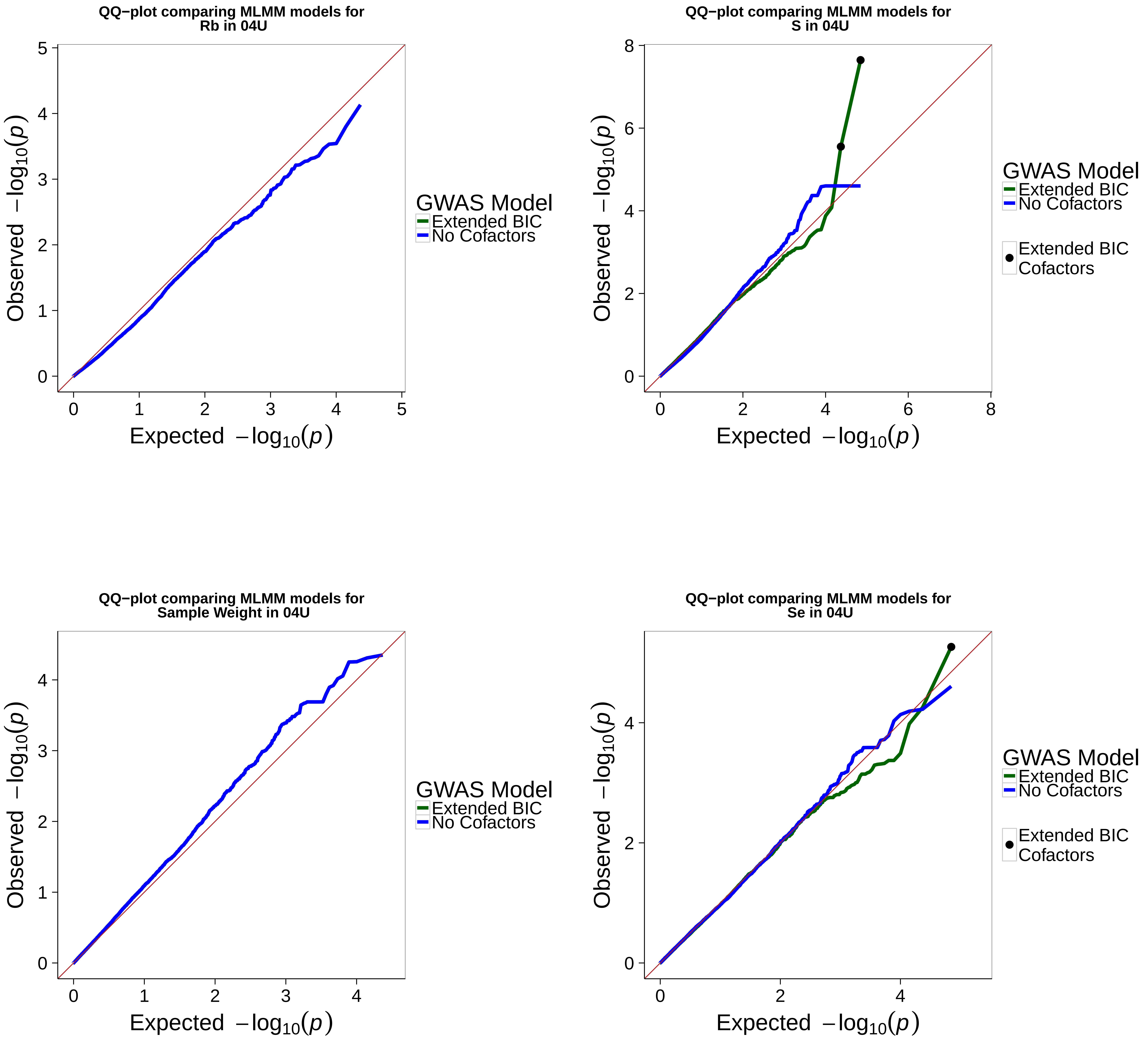
QQ−plot comparing MLMM models for
Rb in 04U
Observed −log10(p)
Expected −log10(p)
GWAS Model
Extended BIC
No Cofactors
QQ−plot comparing MLMM models for
S in 04U
Observed −log10(p)
Expected −log10(p)
GWAS Model
Extended BIC
No Cofactors
Extended BIC
Cofactors
QQ−plot comparing MLMM models for
Sample Weight in 04U
Observed −log10(p)
Expected −log10(p)
GWAS Model
Extended BIC
No Cofactors
QQ−plot comparing MLMM models for
Se in 04U
Observed −log10(p)
Expected −log10(p)
GWAS Model
Extended BIC
No Cofactors
Extended BIC
Cofactors

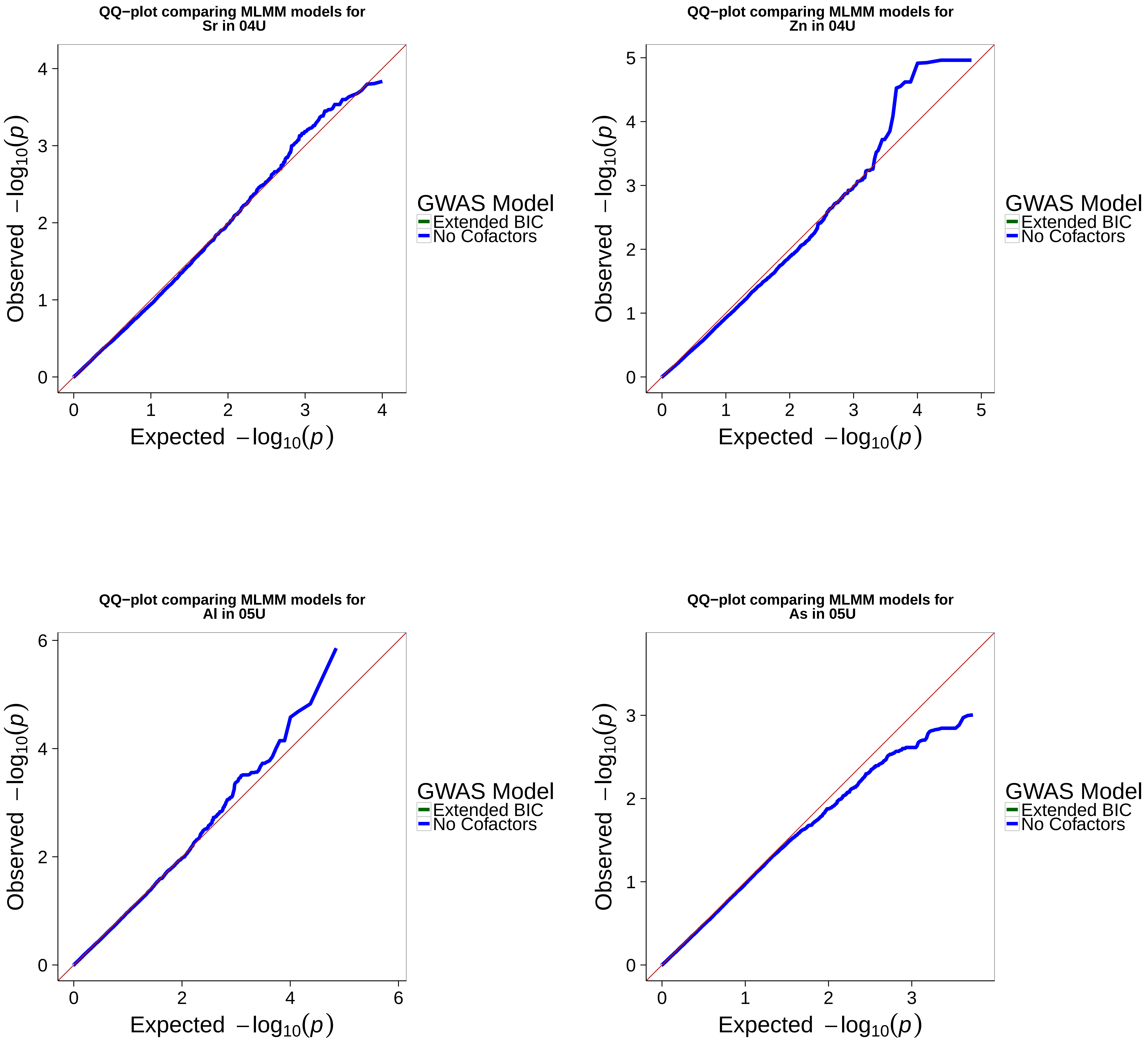
QQ−plot comparing MLMM models for
Sr in 04U
Observed −log10(p)
Expected −log10(p)
GWAS Model
Extended BIC
No Cofactors
QQ−plot comparing MLMM models for
Zn in 04U
Observed −log10(p)
Expected −log10(p)
GWAS Model
Extended BIC
No Cofactors
QQ−plot comparing MLMM models for
Al in 05U
Observed −log10(p)
Expected −log10(p)
GWAS Model
Extended BIC
No Cofactors
QQ−plot comparing MLMM models for
As in 05U
Observed −log10(p)
Expected −log10(p)
GWAS Model
Extended BIC
No Cofactors

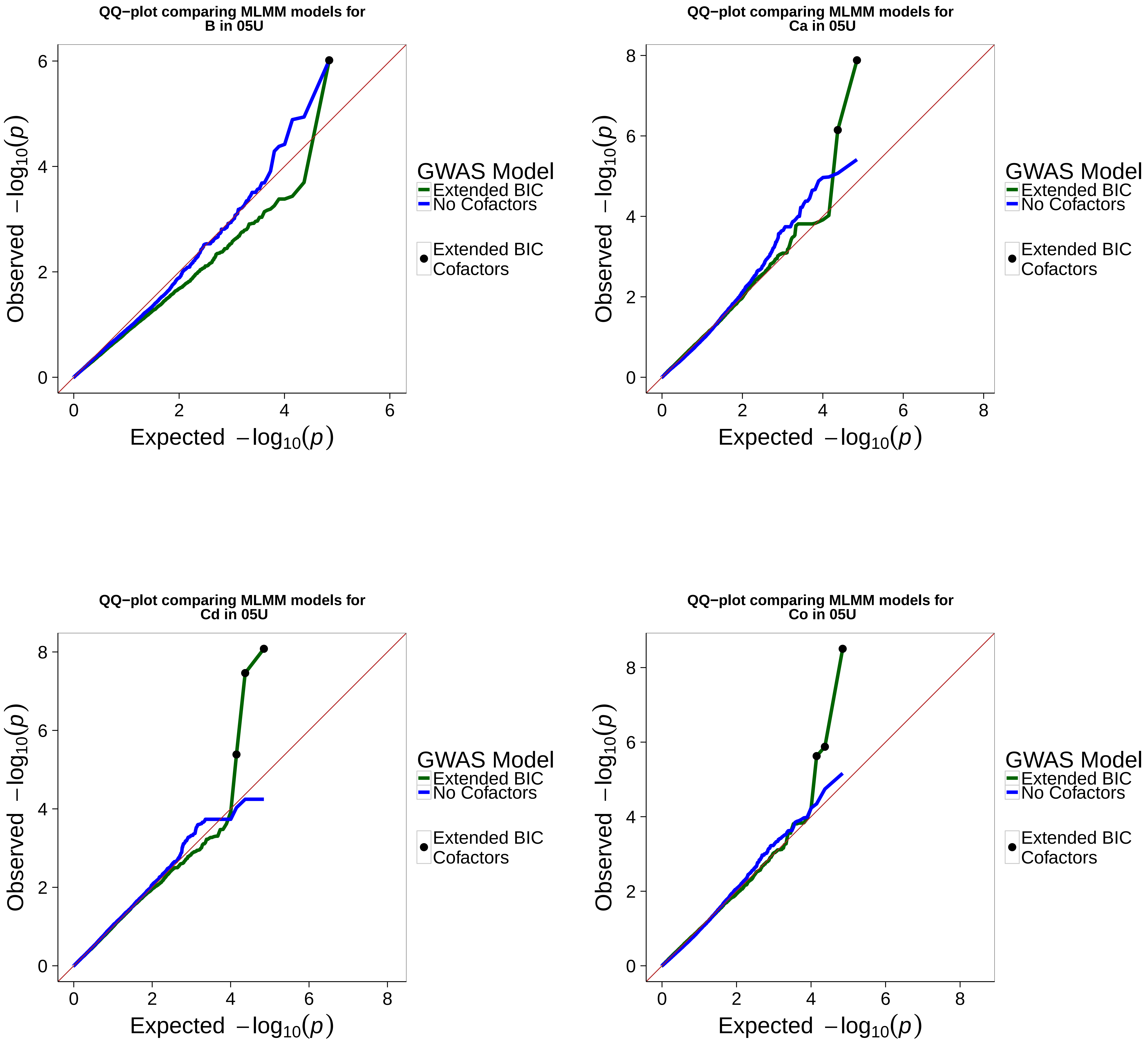
QQ−plot comparing MLMM models for B in 05U
Observed −log10(p)
Expected −log10(p)
GWAS Model
Extended BIC
No Cofactors
Extended BIC Cofactors
QQ−plot comparing MLMM models for Ca in 05U
Observed −log10(p)
Expected −log10(p)
GWAS Model
Extended BIC
No Cofactors
Extended BIC Cofactors
QQ−plot comparing MLMM models for Cd in 05U
Observed −log10(p)
Expected −log10(p)
GWAS Model
Extended BIC
No Cofactors
Extended BIC Cofactors
QQ−plot comparing MLMM models for Co in 05U
Observed −log10(p)
Expected −log10(p)
GWAS Model
Extended BIC
No Cofactors
Extended BIC Cofactors

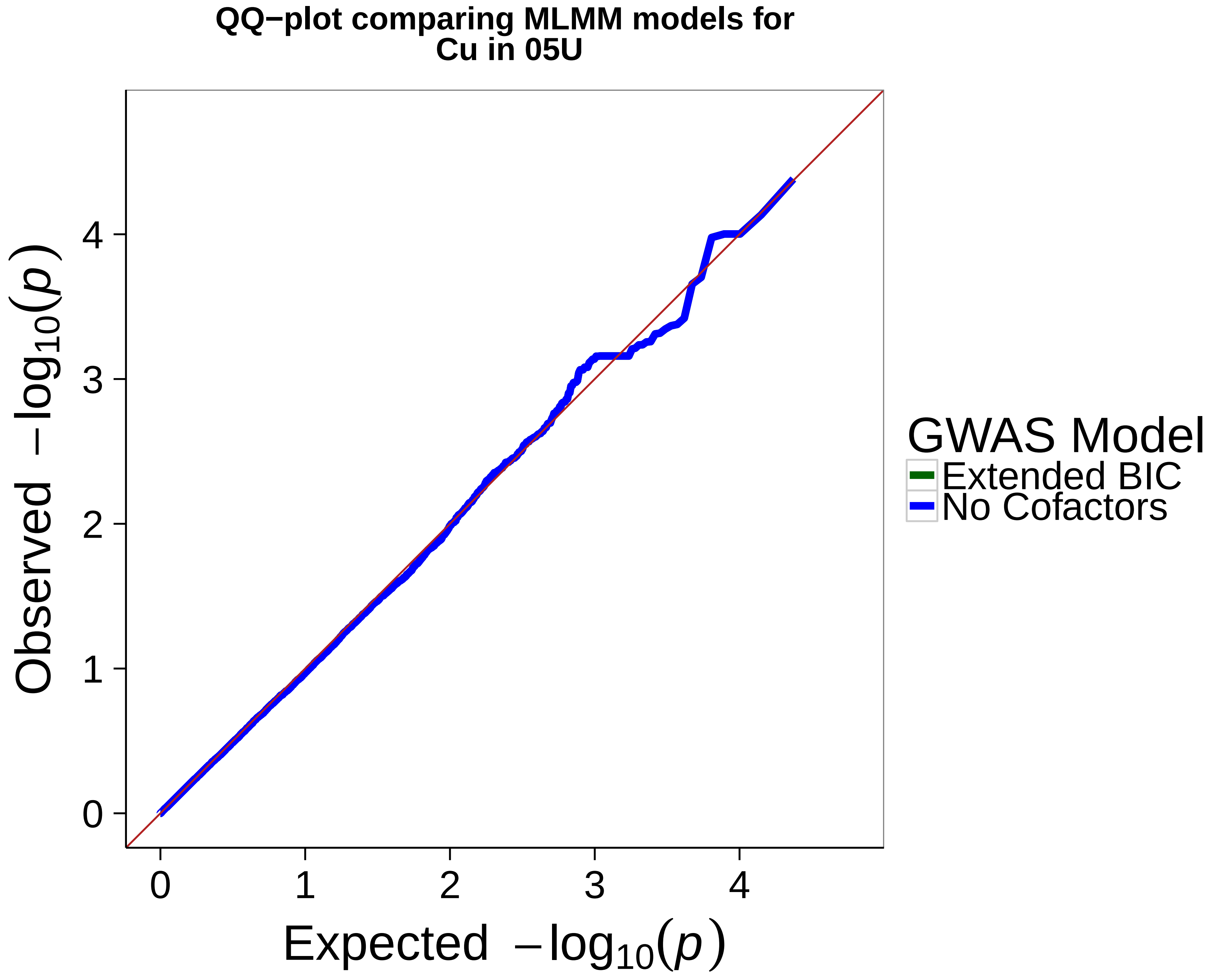
QQ−plot comparing MLMM models for
Cu in 05U
Observed −log10(p)
Expected −log10(p)
GWAS Model
Extended BIC
No Cofactors

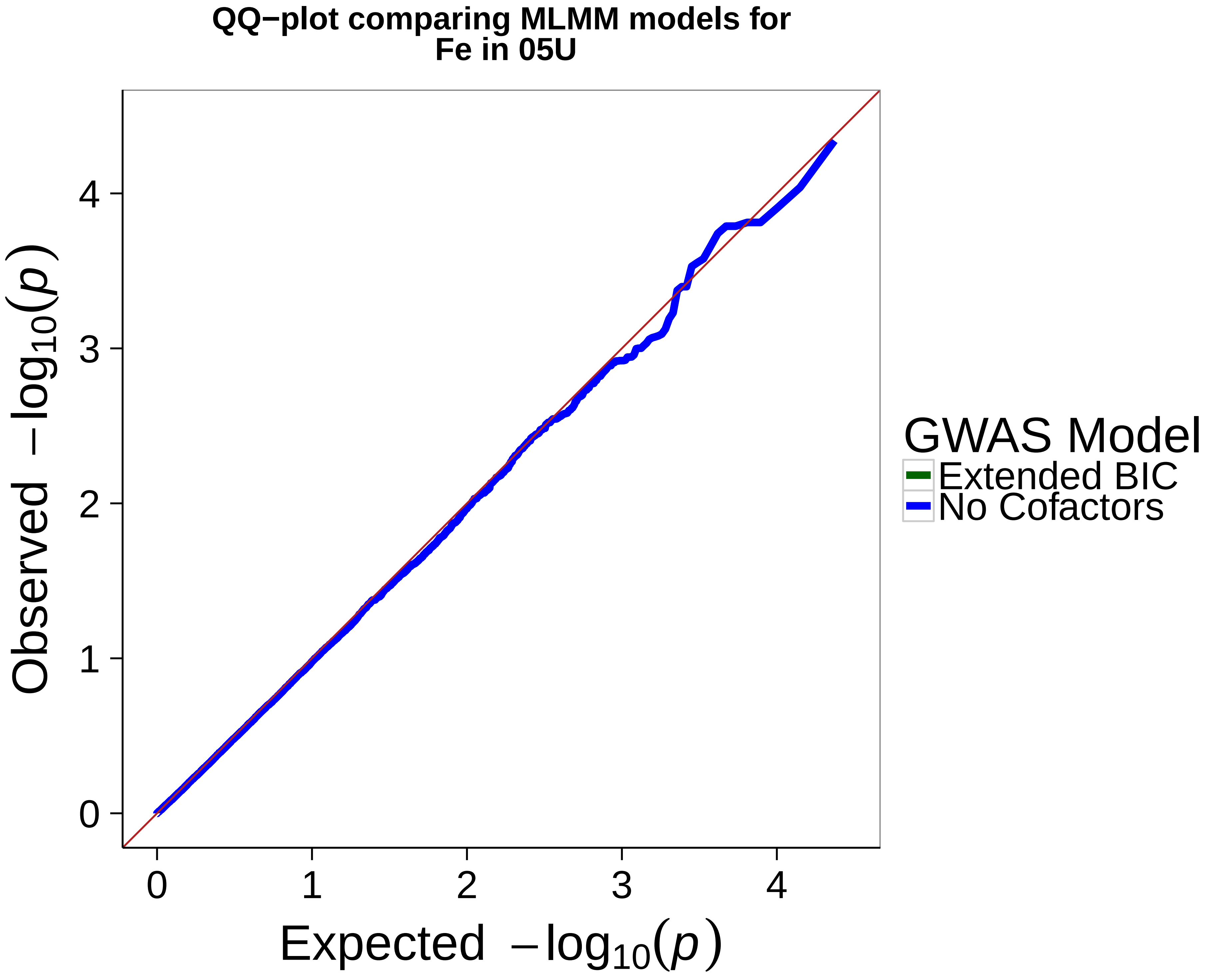
QQ−plot comparing MLMM models for
Fe in 05U
Observed −log10(p)
Expected −log10(p)
GWAS Model
Extended BIC
No Cofactors

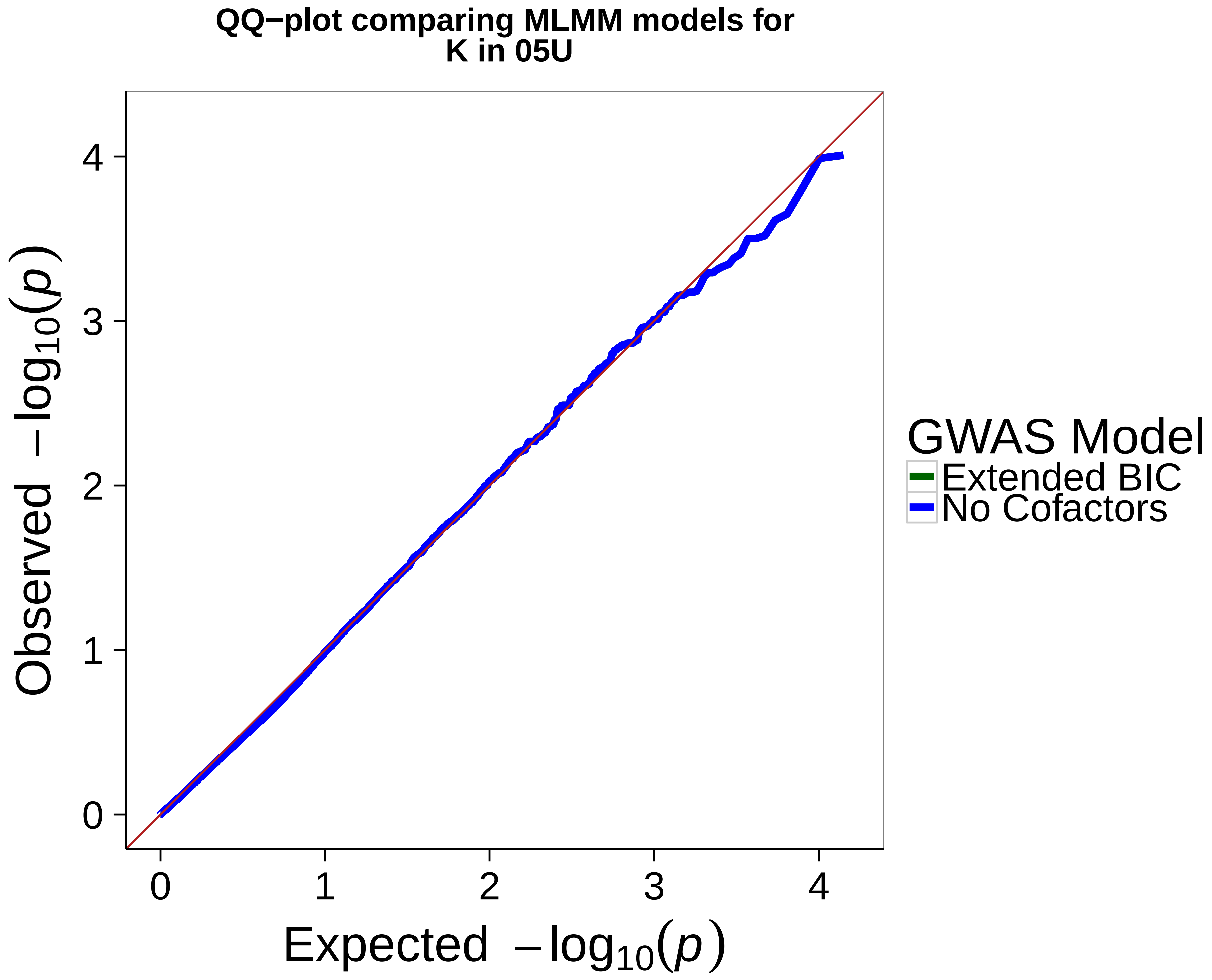
QQ−plot comparing MLMM models for
K in 05U
Observed −log10(p)
Expected −log10(p)
GWAS Model
Extended BIC
No Cofactors

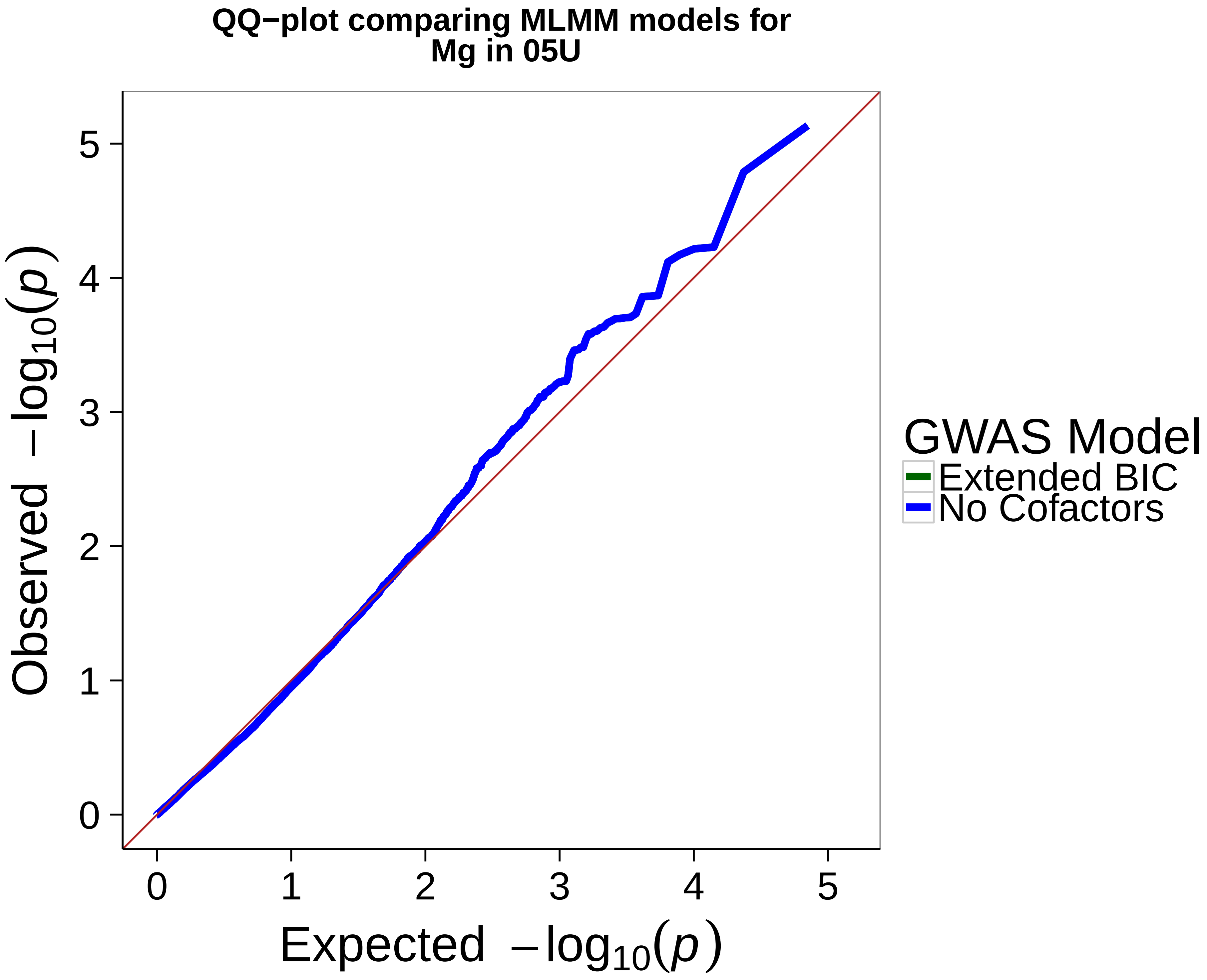
QQ−plot comparing MLMM models for
Mg in 05U
Observed −log10(p)
Expected −log10(p)
GWAS Model
Extended BIC
No Cofactors

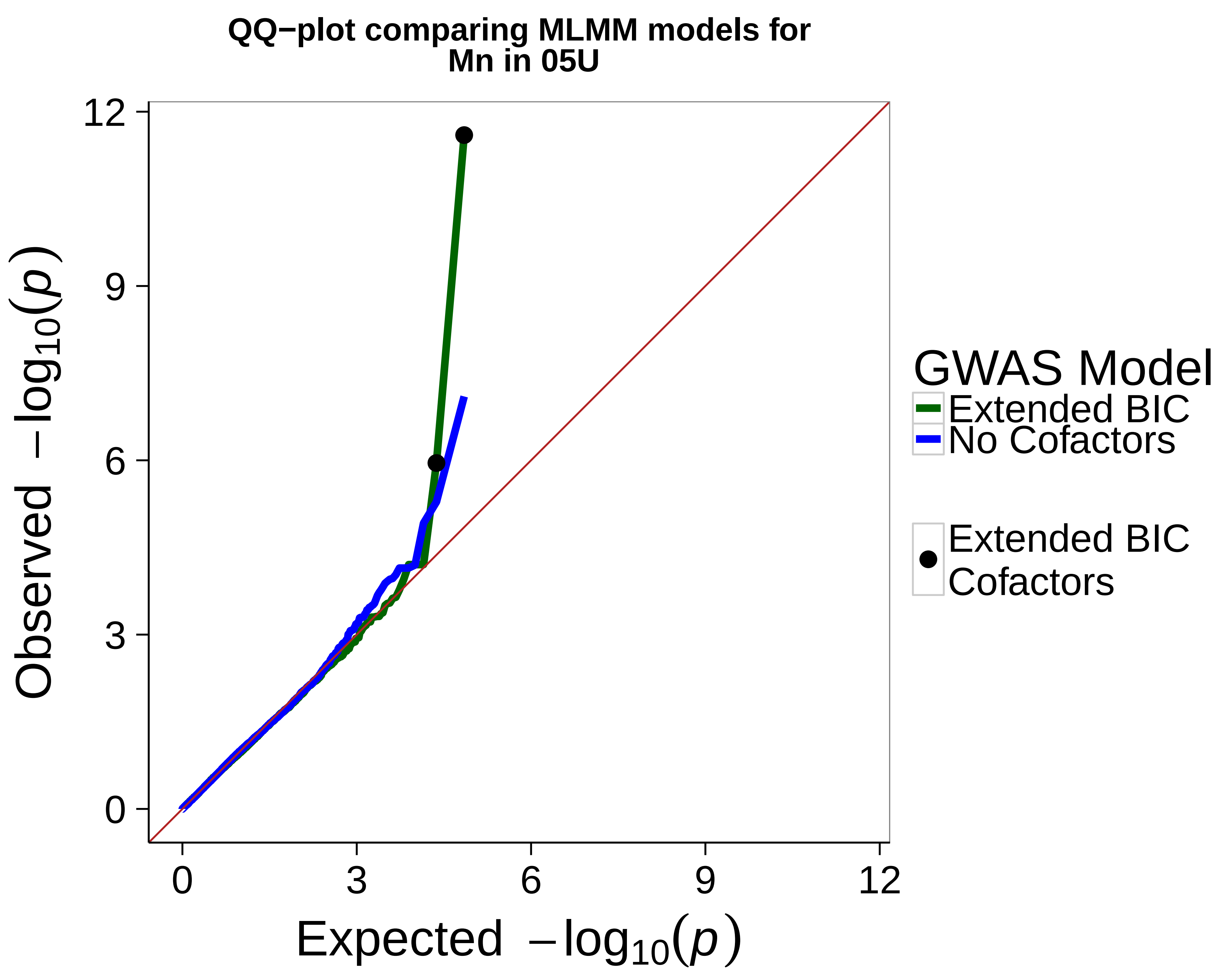
QQ−plot comparing MLMM models for
Mn in 05U
Observed −log10(p)
Expected −log10(p)
GWAS Model
Extended BIC
No Cofactors
Extended BIC
Cofactors
0
3
6
9
12

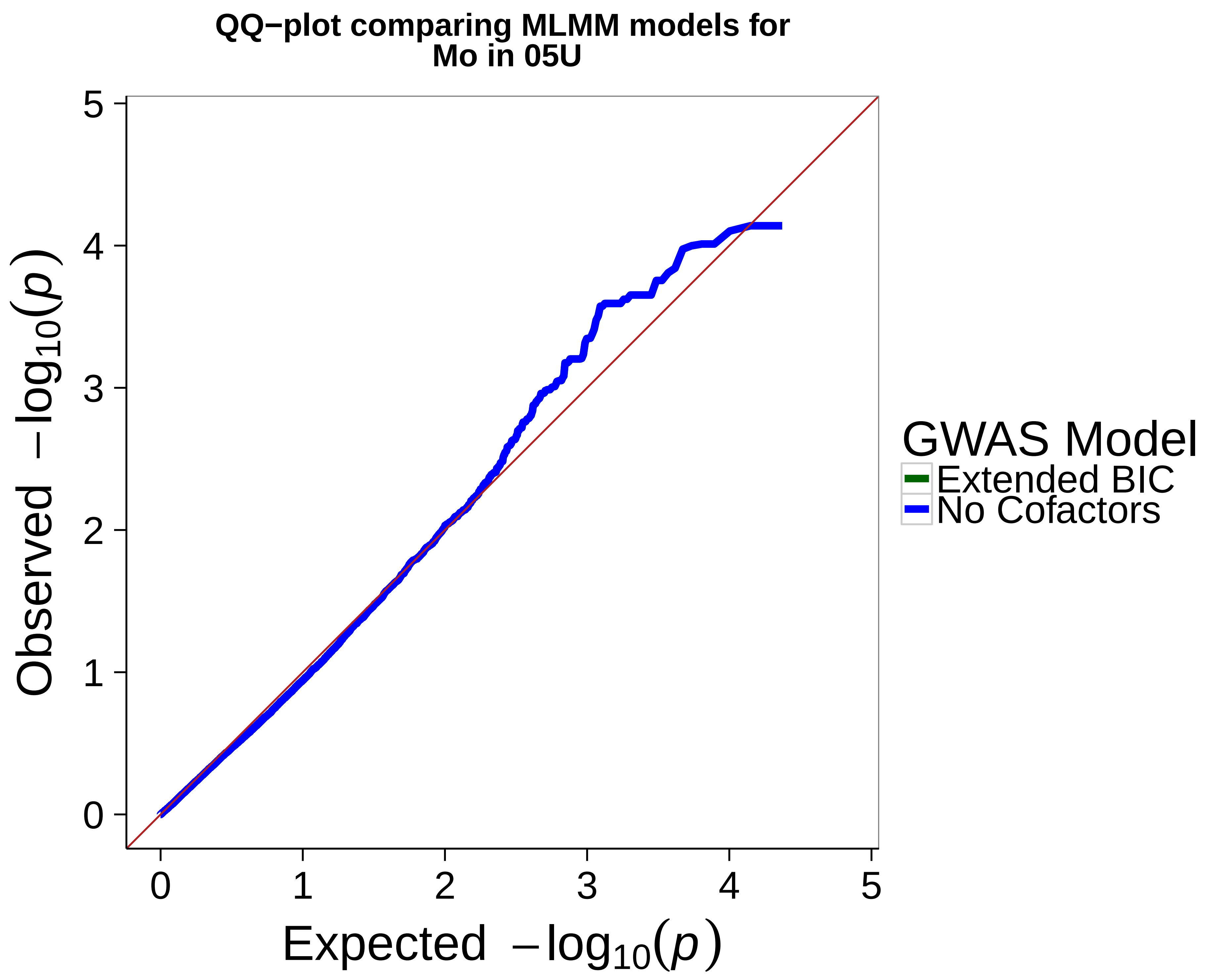
QQ−plot comparing MLMM models for
Mo in 05U
Observed −log10(p)
Expected −log10(p)
GWAS Model
Extended BIC
No Cofactors
0
1
2
3
4
5

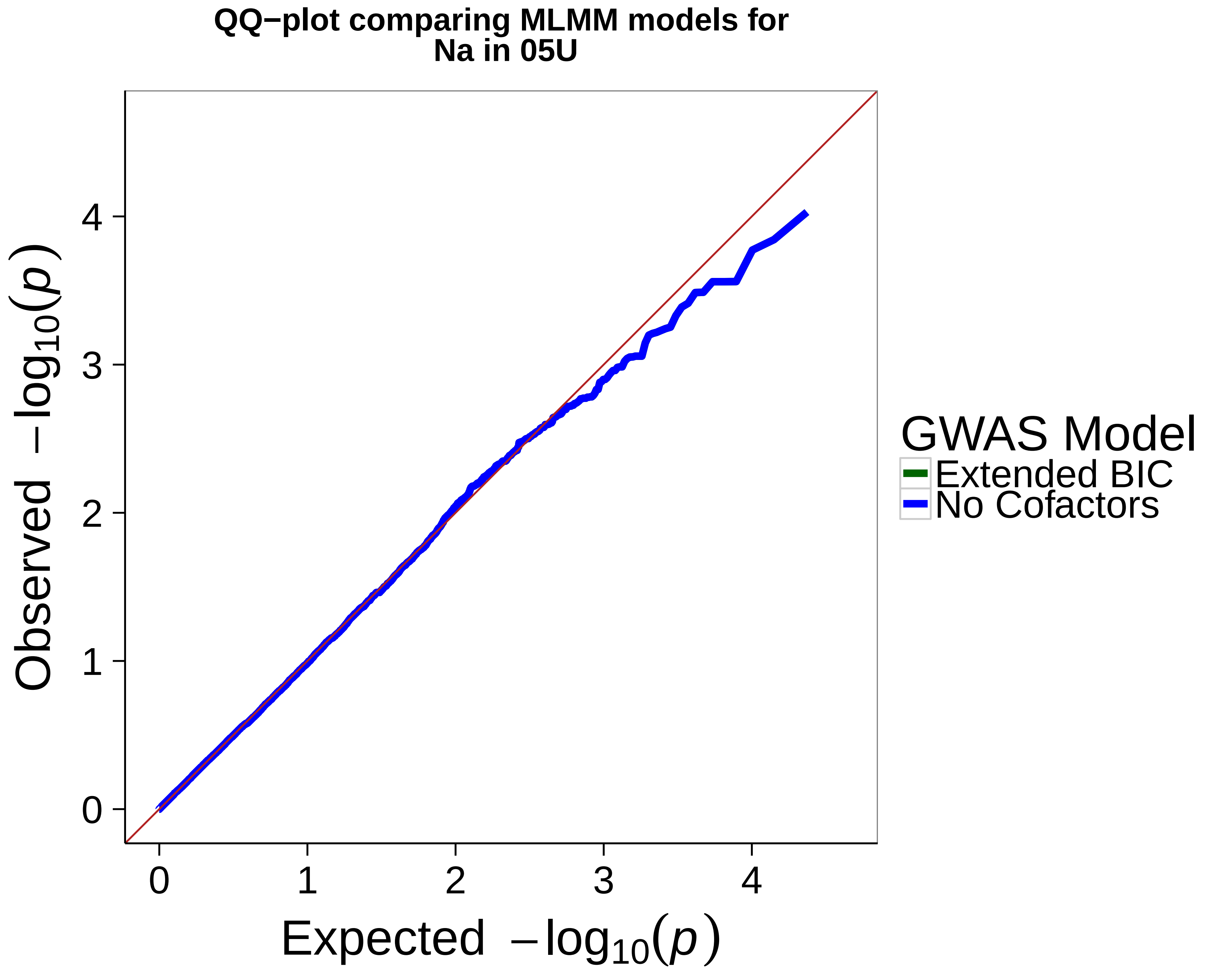
QQ−plot comparing MLMM models for
Na in 05U
Observed −log10(p)
Expected −log10(p)
GWAS Model
Extended BIC
No Cofactors
0
1
2
3
4

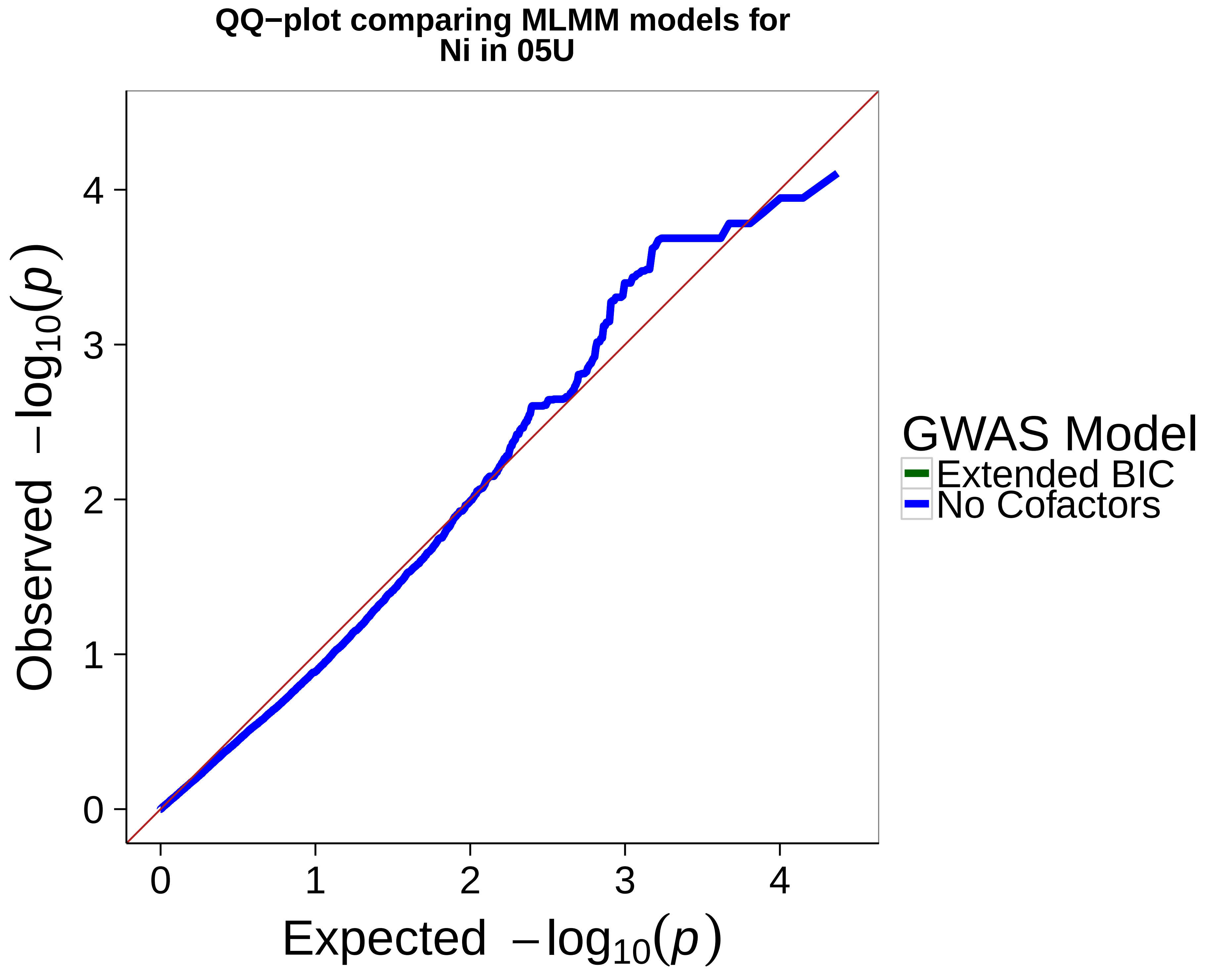
QQ−plot comparing MLMM models for
Ni in 05U
Observed −log10(p)
Expected −log10(p)
GWAS Model
Extended BIC
No Cofactors
0
1
2
3
4

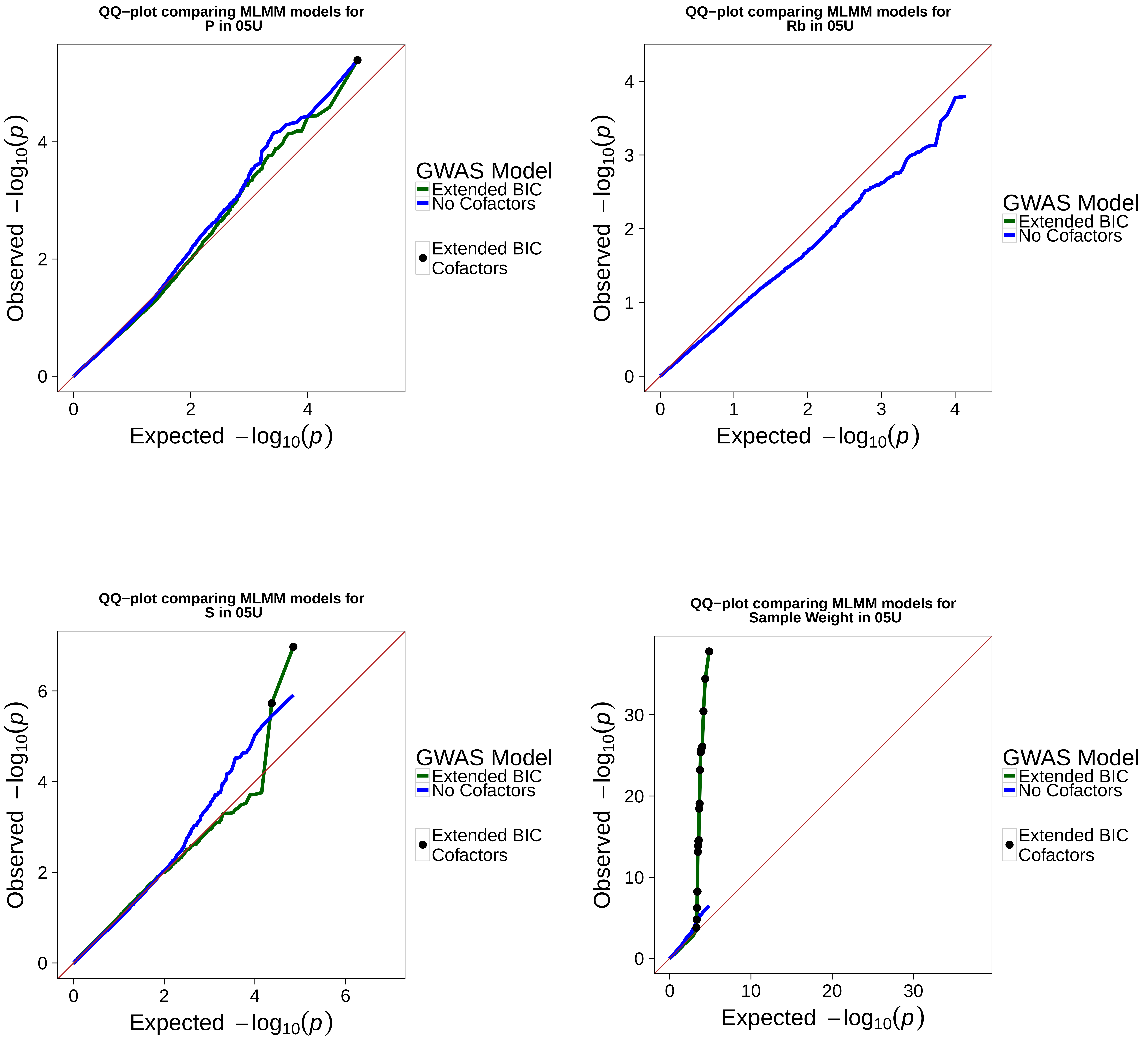
QQ−plot comparing MLMM models for
P in 05U
Observed −log10(p)
Expected −log10(p)
0
2
4
GWAS Model
Extended BIC
No Cofactors
Extended BIC
Cofactors
QQ−plot comparing MLMM models for
Rb in 05U
Observed −log10(p)
Expected −log10(p)
0
1
2
3
4
GWAS Model
Extended BIC
No Cofactors
QQ−plot comparing MLMM models for
S in 05U
Observed −log10(p)
Expected −log10(p)
0
2
4
6
GWAS Model
Extended BIC
No Cofactors
Extended BIC
Cofactors
QQ−plot comparing MLMM models for
Sample Weight in 05U
Observed −log10(p)
Expected −log10(p)
0
10
20
30
GWAS Model
Extended BIC
No Cofactors
Extended BIC
Cofactors

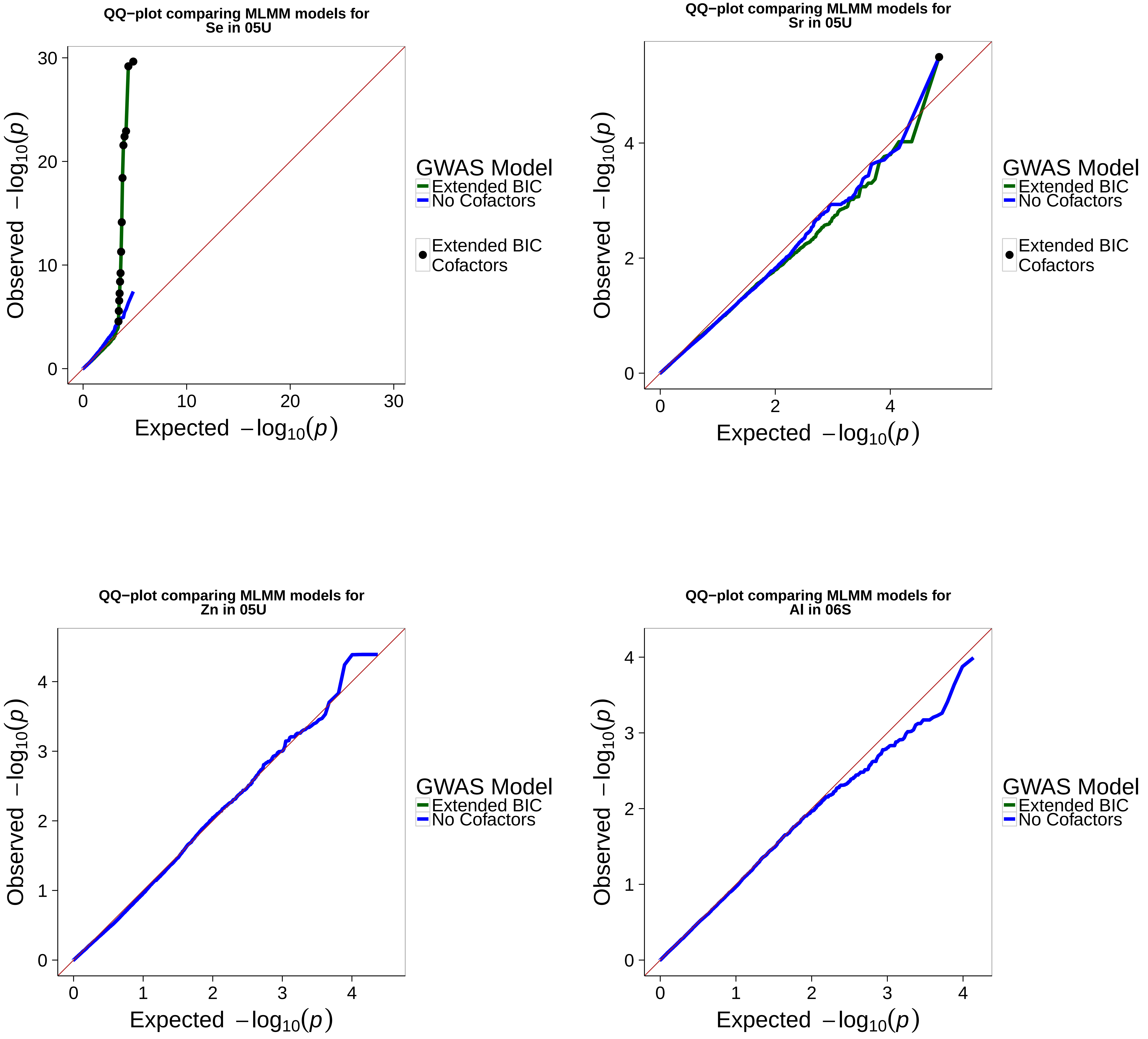
QQ−plot comparing MLMM models for Se in 05U
QQ−plot comparing MLMM models for Sr in 05U
QQ−plot comparing MLMM models for Zn in 05U
QQ−plot comparing MLMM models for Al in 06S
Observed $-\log_{10}(p)$
Expected $-\log_{10}(p)$
GWAS Model
Extended BIC
No Cofactors
Extended BIC Cofactors

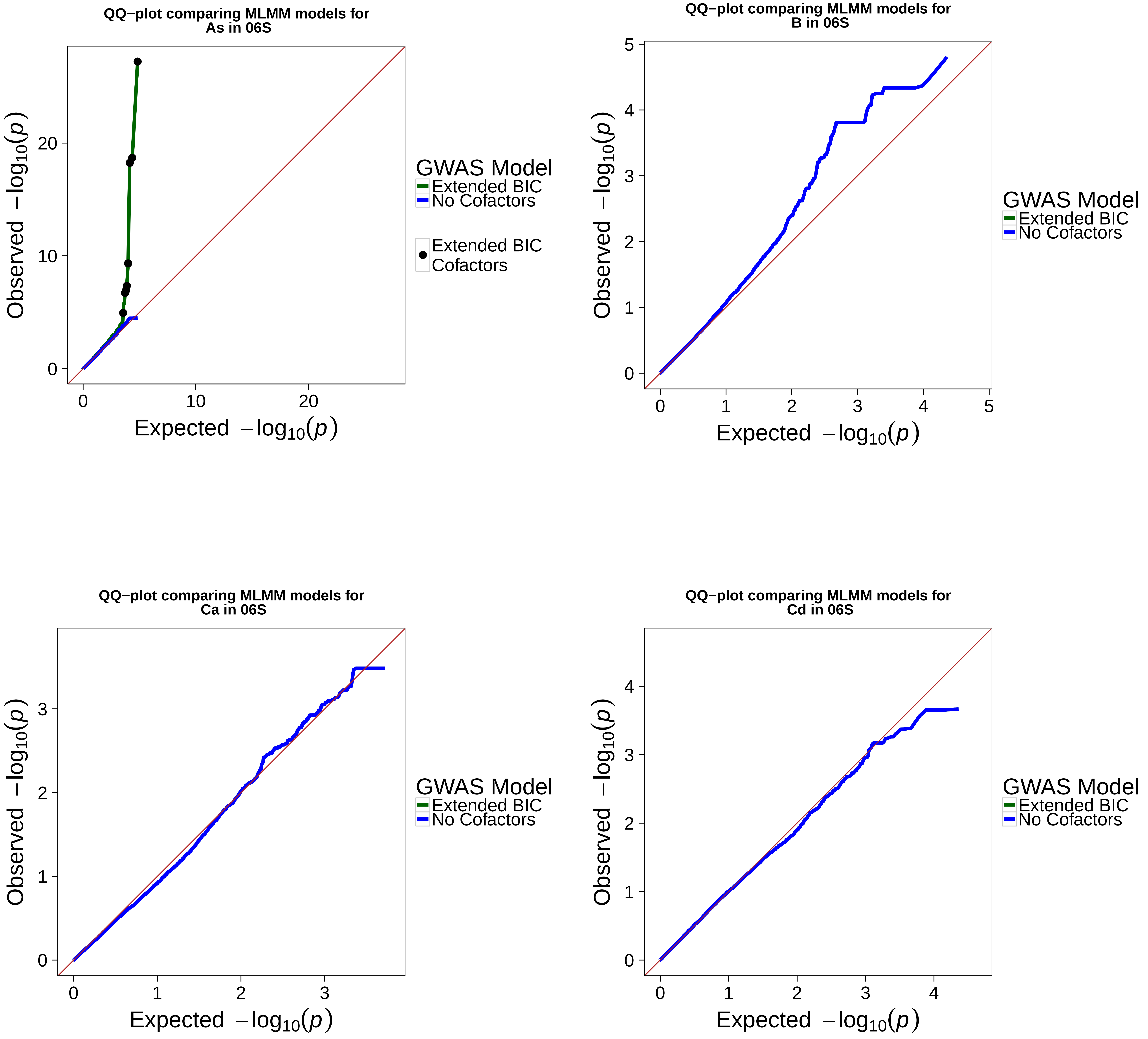

QQ−plot comparing MLMM models for
As in 06S
Observed −log10(p)
Expected −log10(p)
GWAS Model
Extended BIC
No Cofactors
Extended BIC
Cofactors
QQ−plot comparing MLMM models for
B in 06S
Observed −log10(p)
Expected −log10(p)
GWAS Model
Extended BIC
No Cofactors
QQ−plot comparing MLMM models for
Ca in 06S
Observed −log10(p)
Expected −log10(p)
GWAS Model
Extended BIC
No Cofactors
QQ−plot comparing MLMM models for
Cd in 06S
Observed −log10(p)
Expected −log10(p)
GWAS Model
Extended BIC
No Cofactors

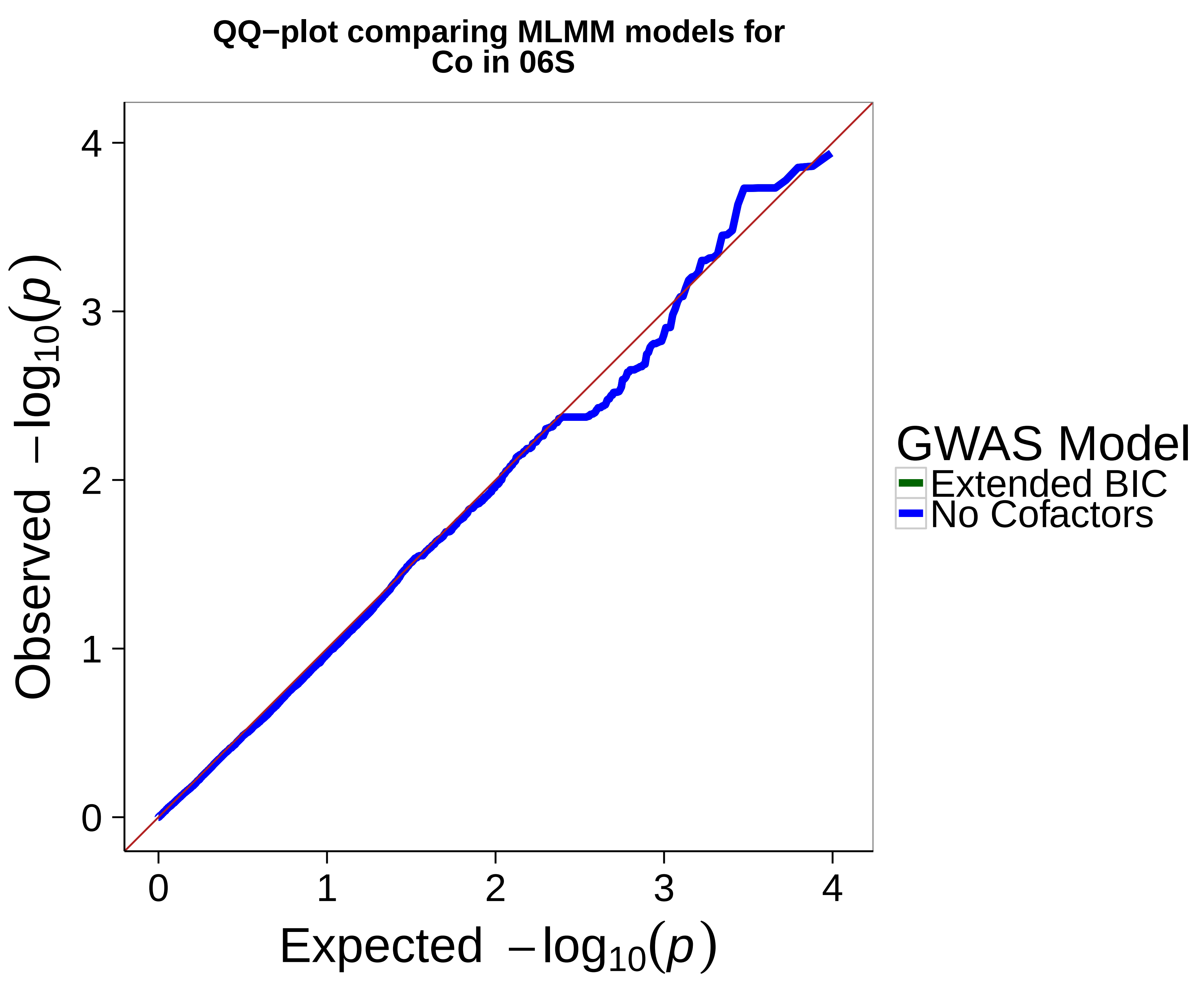
QQ−plot comparing MLMM models for
Co in 06S
Observed −log10(p)
Expected −log10(p)
0
1
2
3
4
GWAS Model
Extended BIC
No Cofactors

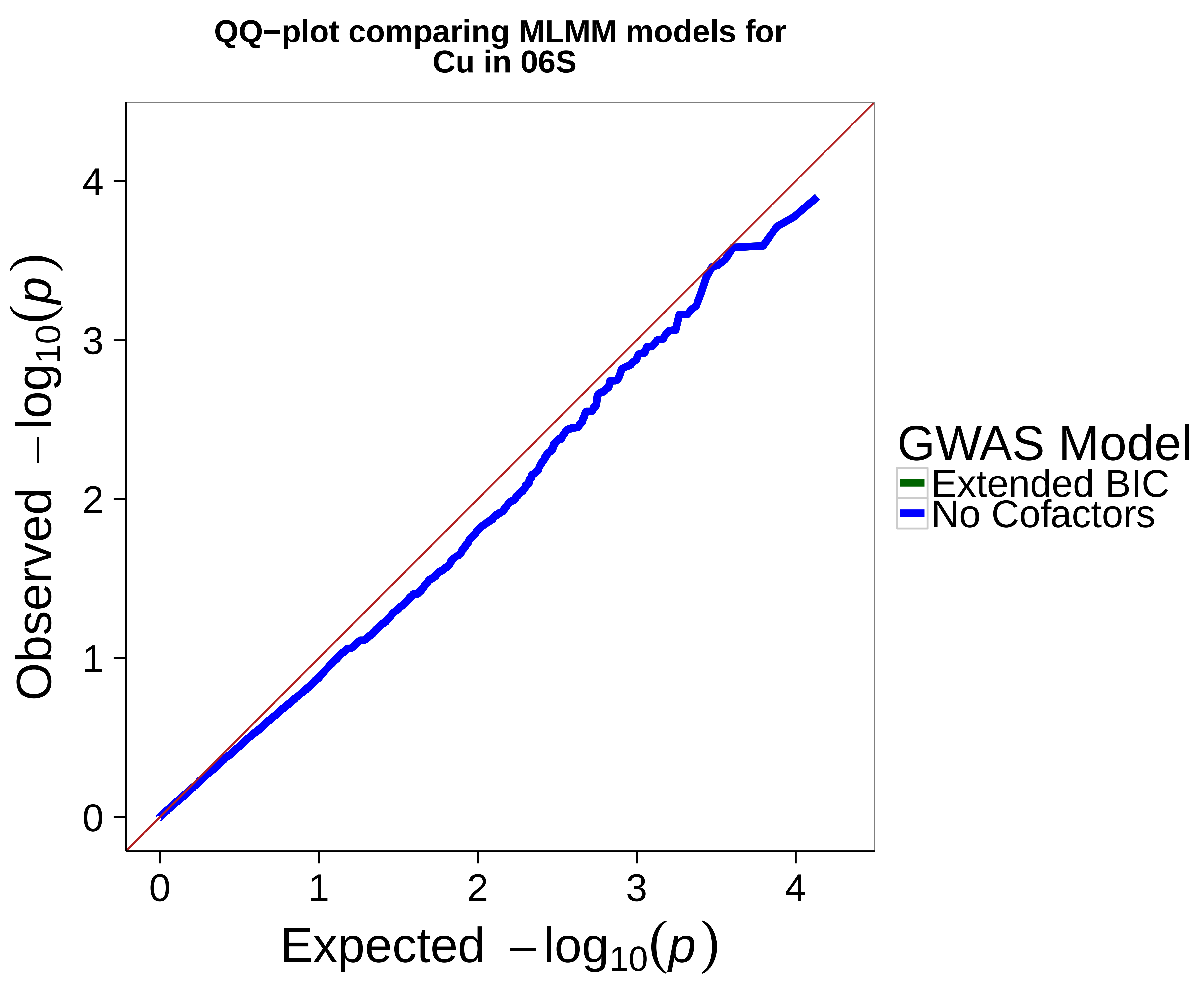
QQ−plot comparing MLMM models for
Cu in 06S
Observed −log10(p)
Expected −log10(p)
0
1
2
3
4
GWAS Model
Extended BIC
No Cofactors

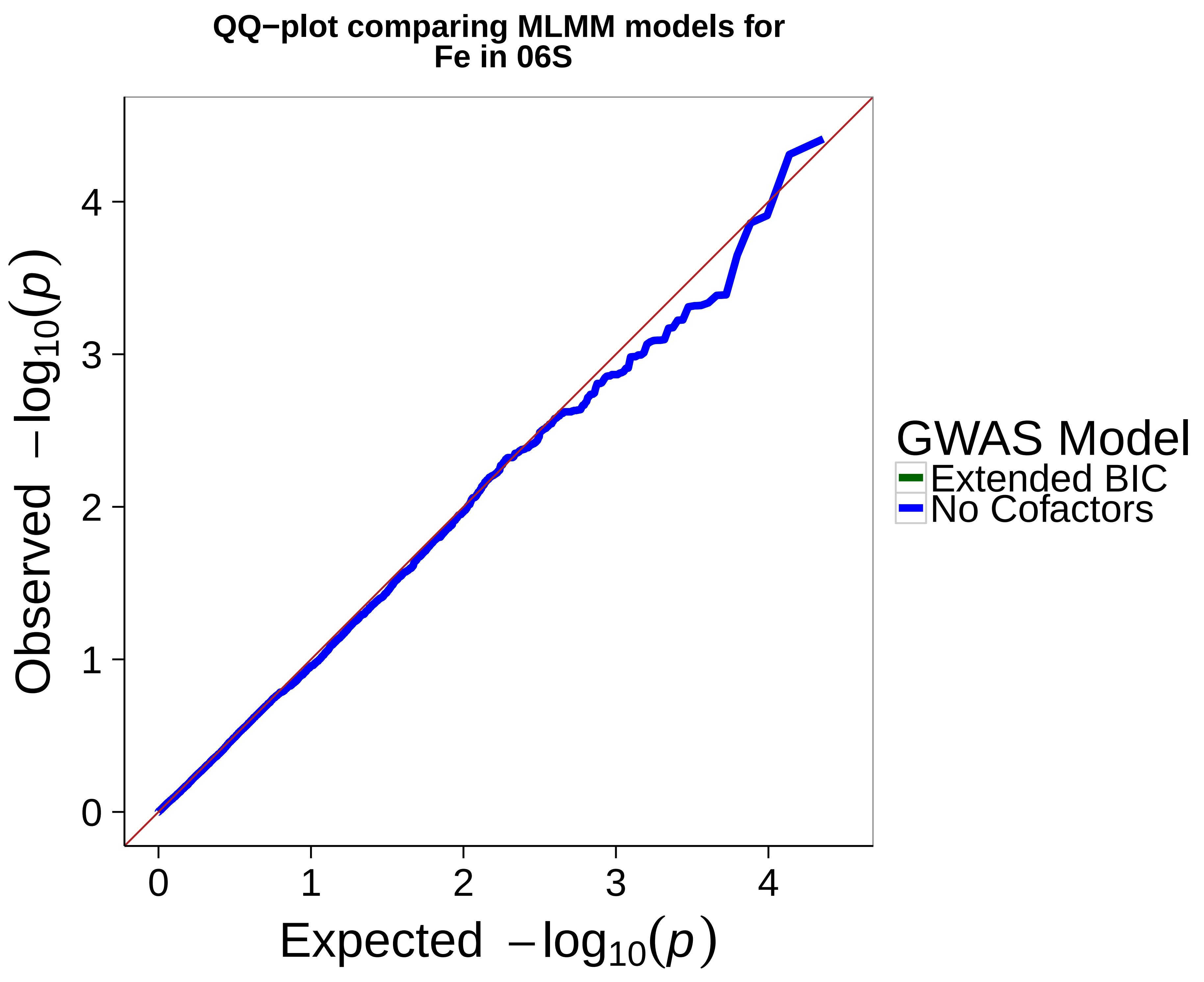
QQ−plot comparing MLMM models for
Fe in 06S
Observed −log10(p)
Expected −log10(p)
0
1
2
3
4
GWAS Model
Extended BIC
No Cofactors

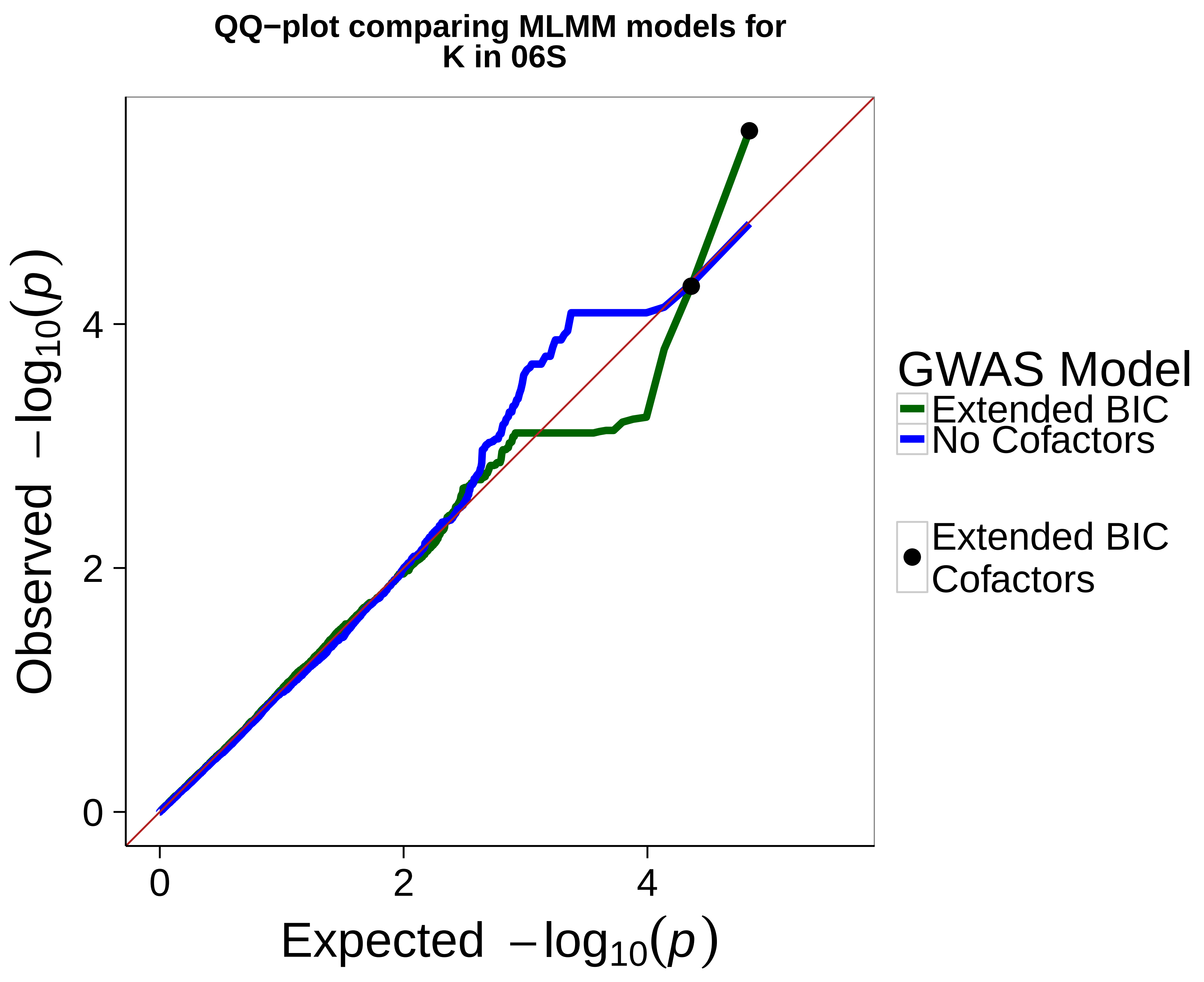
QQ−plot comparing MLMM models for
K in 06S
Observed −log10(p)
Expected −log10(p)
0
2
4
GWAS Model
Extended BIC
No Cofactors
Extended BIC
Cofactors

QQ−plot comparing MLMM models for Mg in 06S

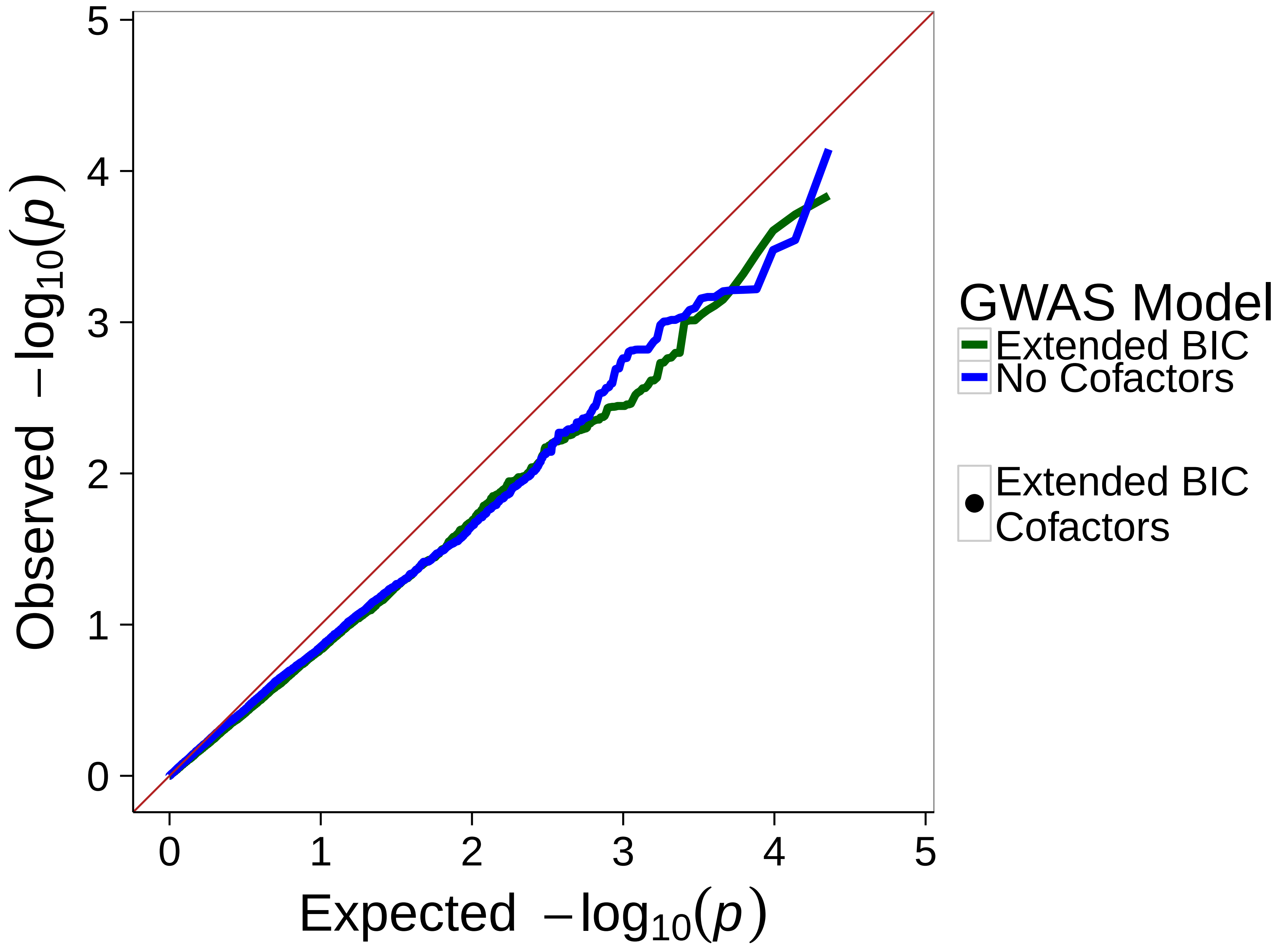

QQ−plot comparing MLMM models for Mn in 06S

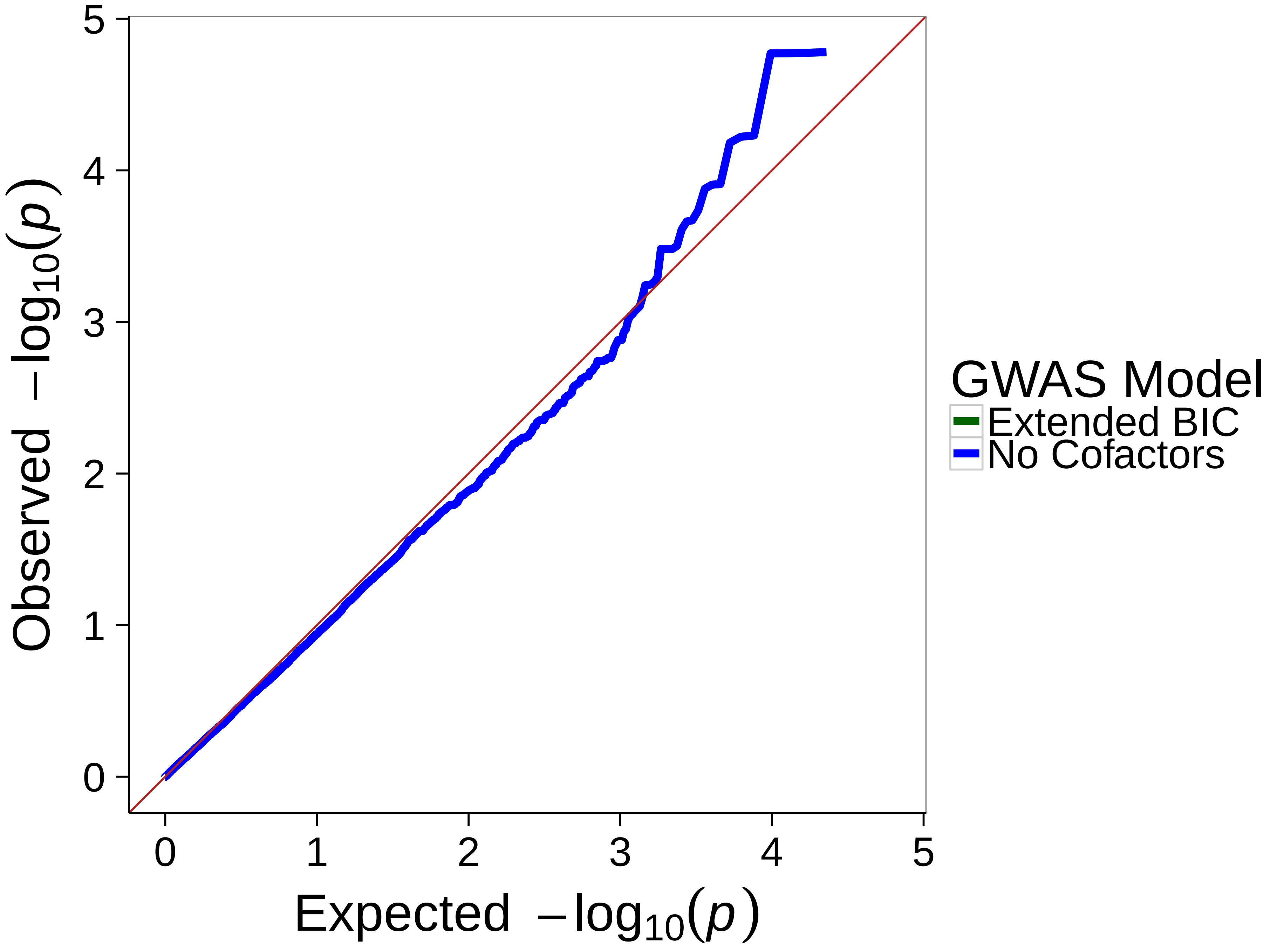

QQ−plot comparing MLMM models for Mo in 06S

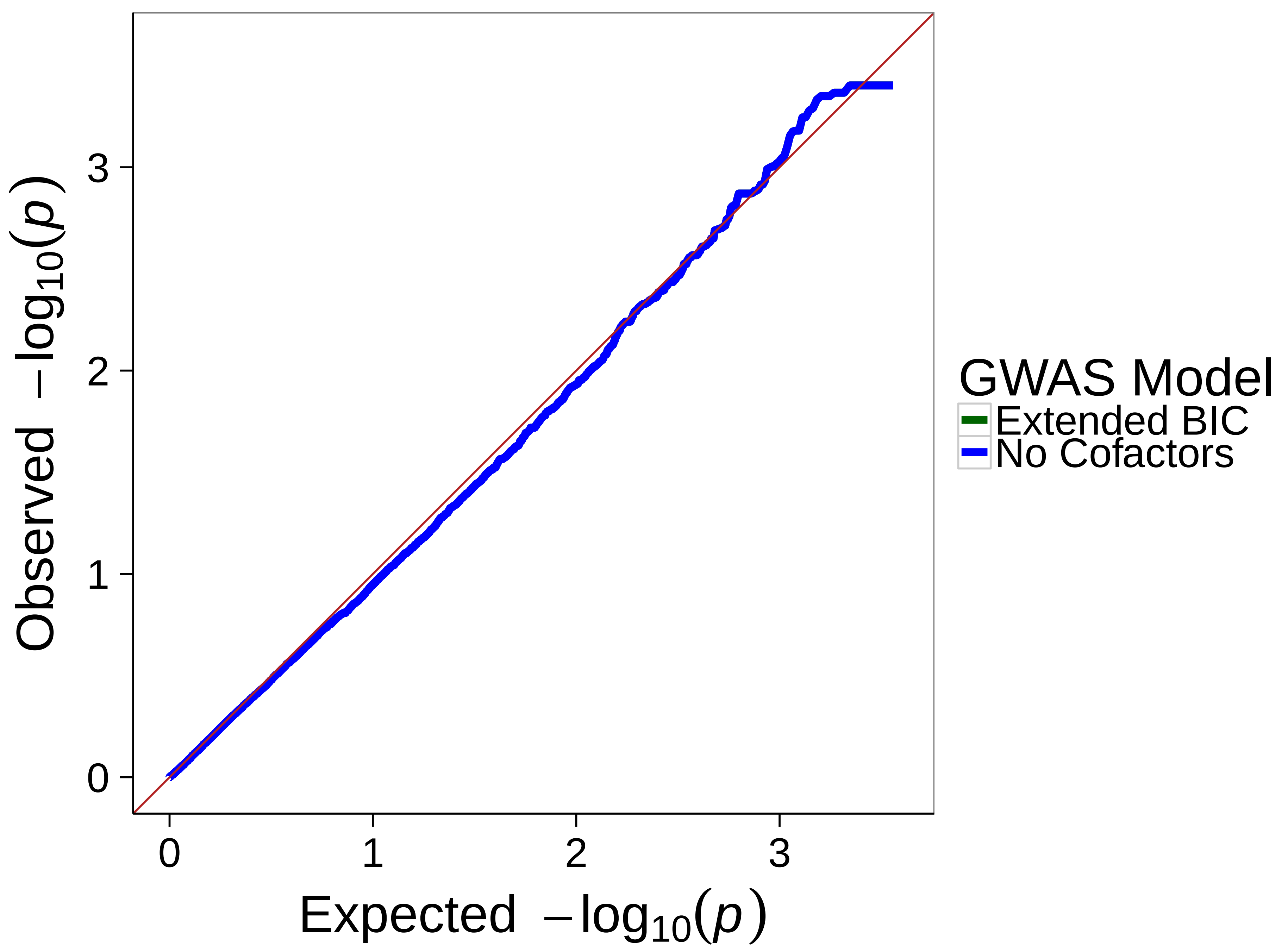

QQ−plot comparing MLMM models for Na in 06S

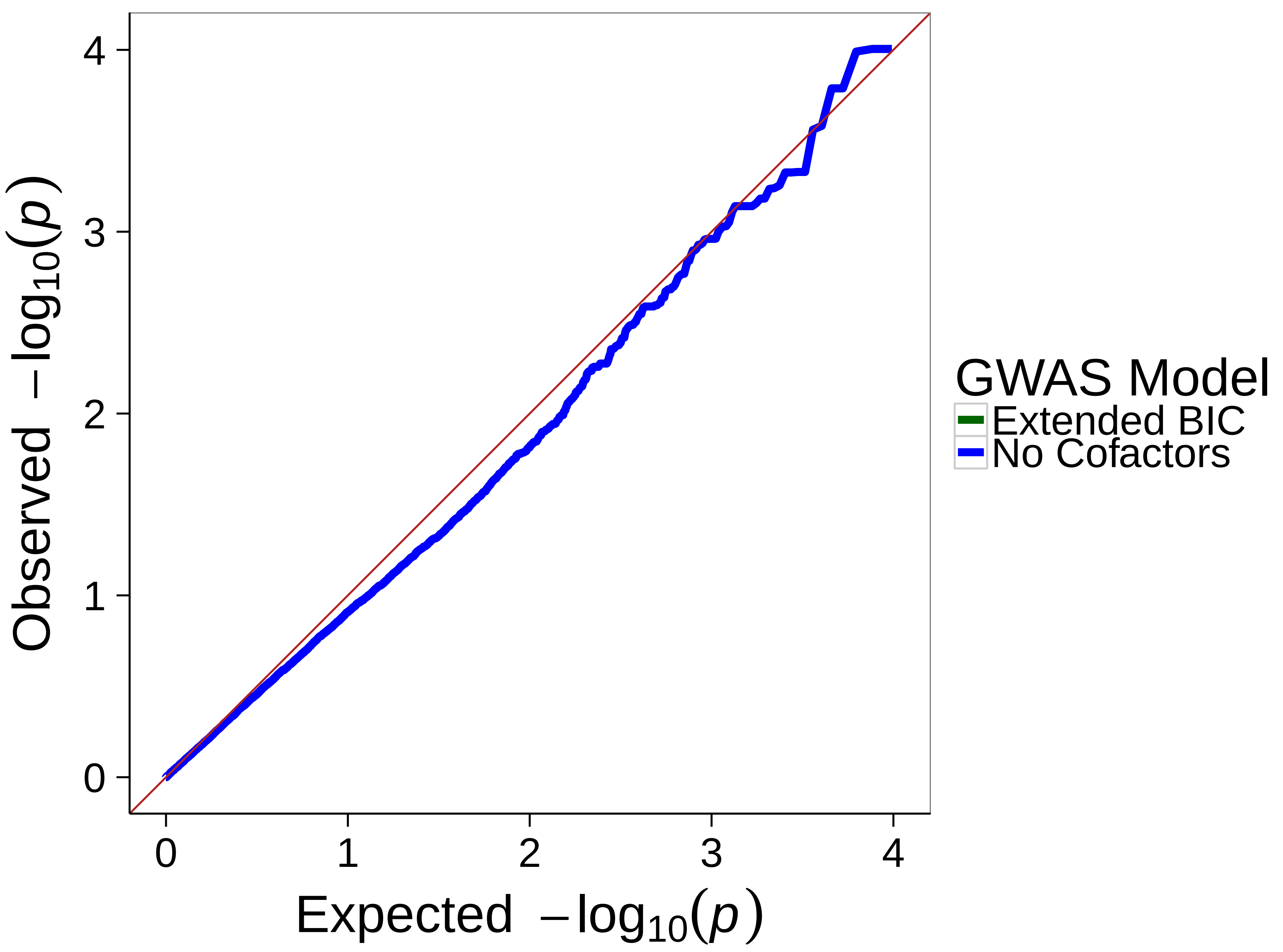

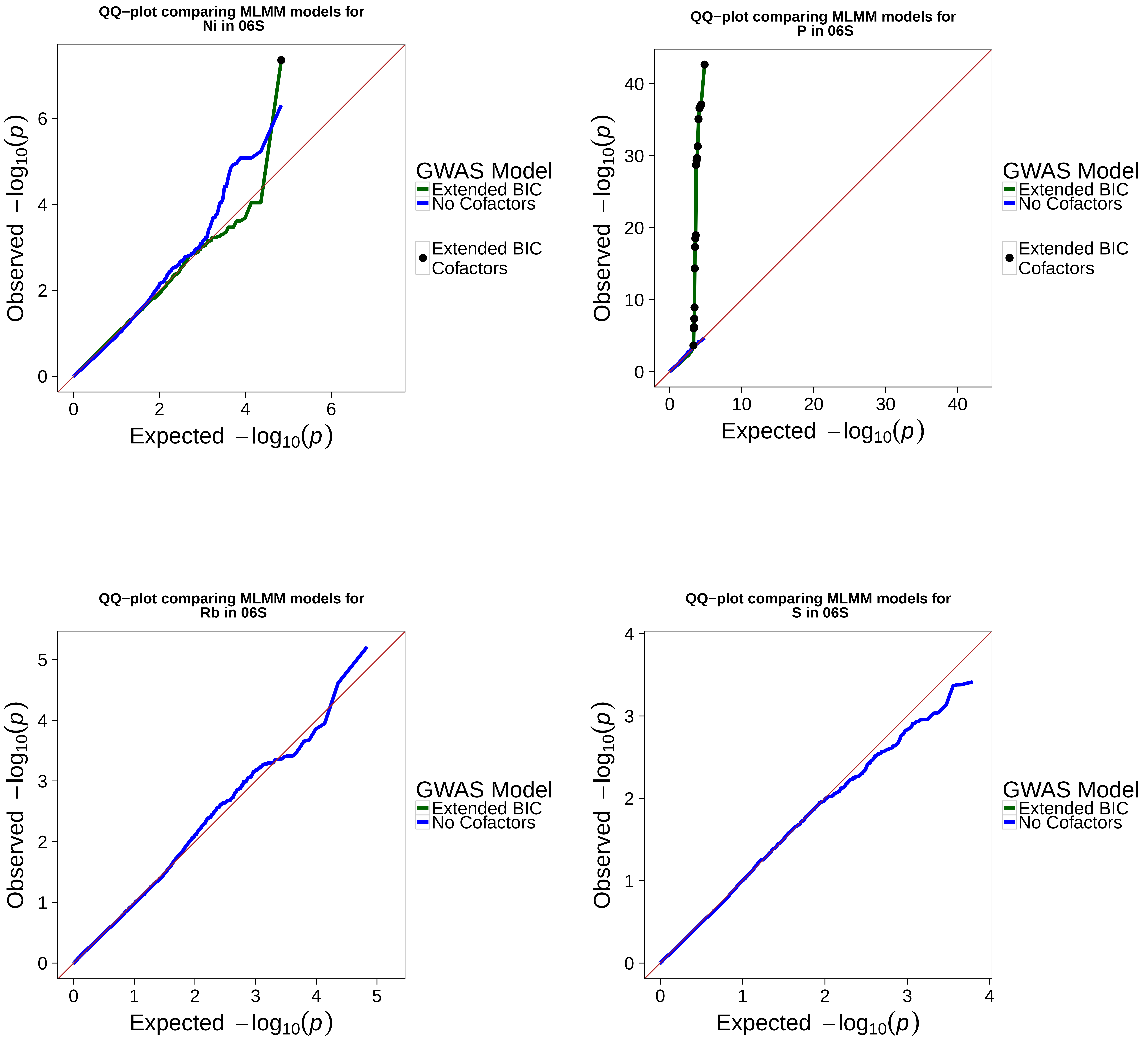

QQ−plot comparing MLMM models for
Ni in 06S
Observed −log10(p)
Expected −log10(p)
GWAS Model
Extended BIC
No Cofactors
Extended BIC
Cofactors
QQ−plot comparing MLMM models for
P in 06S
Observed −log10(p)
Expected −log10(p)
GWAS Model
Extended BIC
No Cofactors
Extended BIC
Cofactors
QQ−plot comparing MLMM models for
Rb in 06S
Observed −log10(p)
Expected −log10(p)
GWAS Model
Extended BIC
No Cofactors
QQ−plot comparing MLMM models for
S in 06S
Observed −log10(p)
Expected −log10(p)
GWAS Model
Extended BIC
No Cofactors

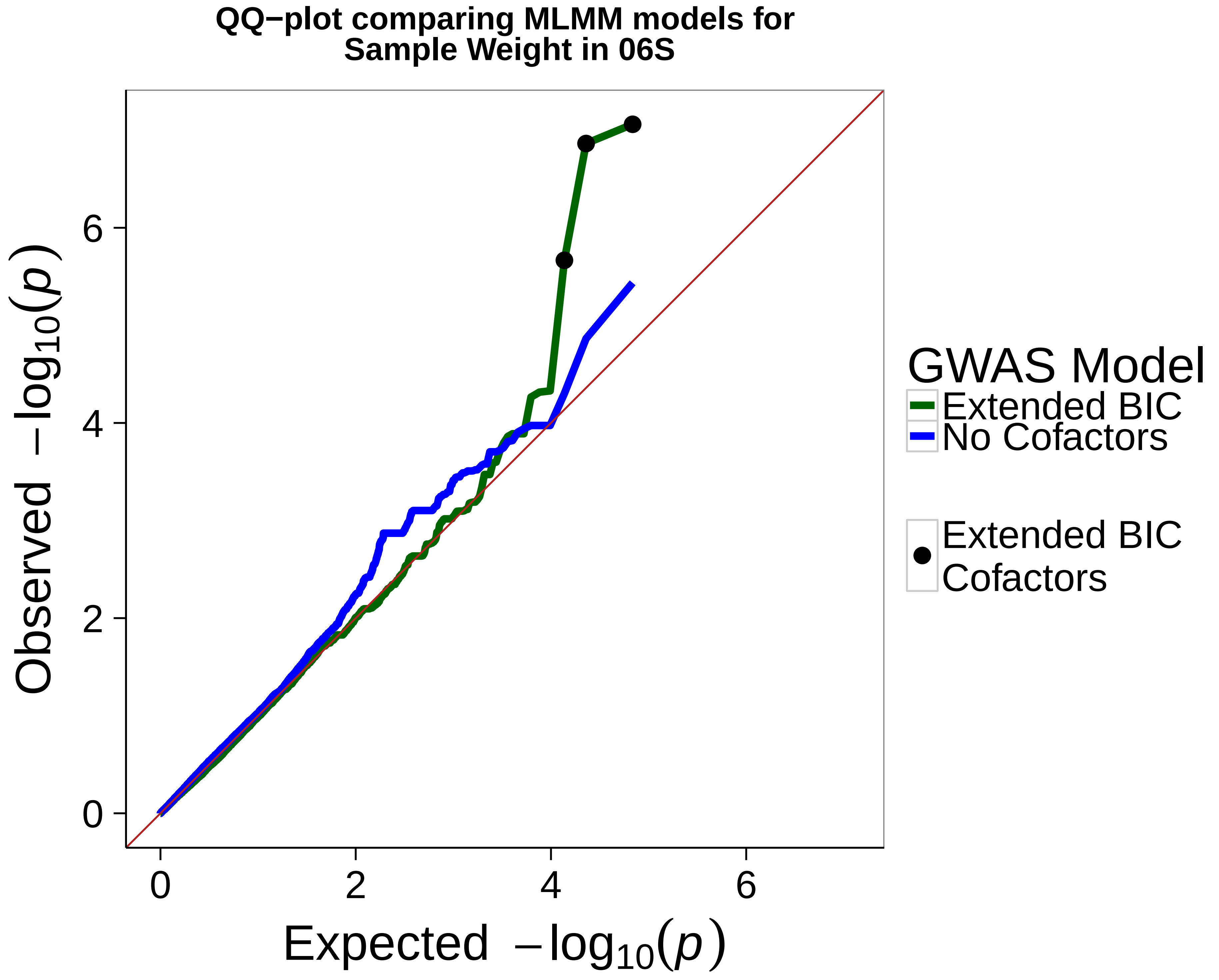
QQ−plot comparing MLMM models for
Sample Weight in 06S
Observed $-\log_{10}(p)$
Expected $-\log_{10}(p)$
0
2
4
6
GWAS Model
Extended BIC
No Cofactors
Extended BIC
Cofactors

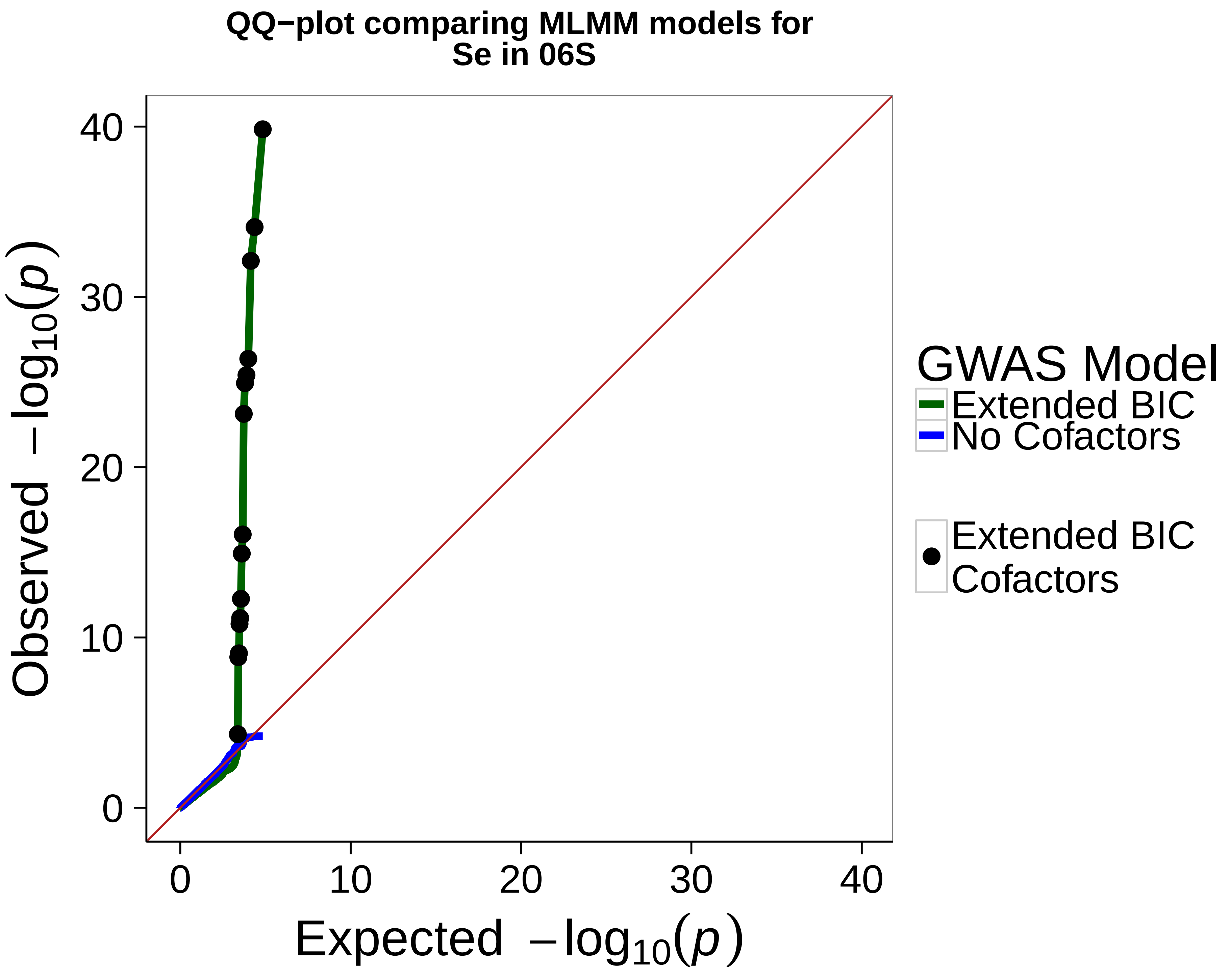
QQ−plot comparing MLMM models for
Se in 06S
Observed $-\log_{10}(p)$
Expected $-\log_{10}(p)$
0
10
20
30
40
GWAS Model
Extended BIC
No Cofactors
Extended BIC
Cofactors

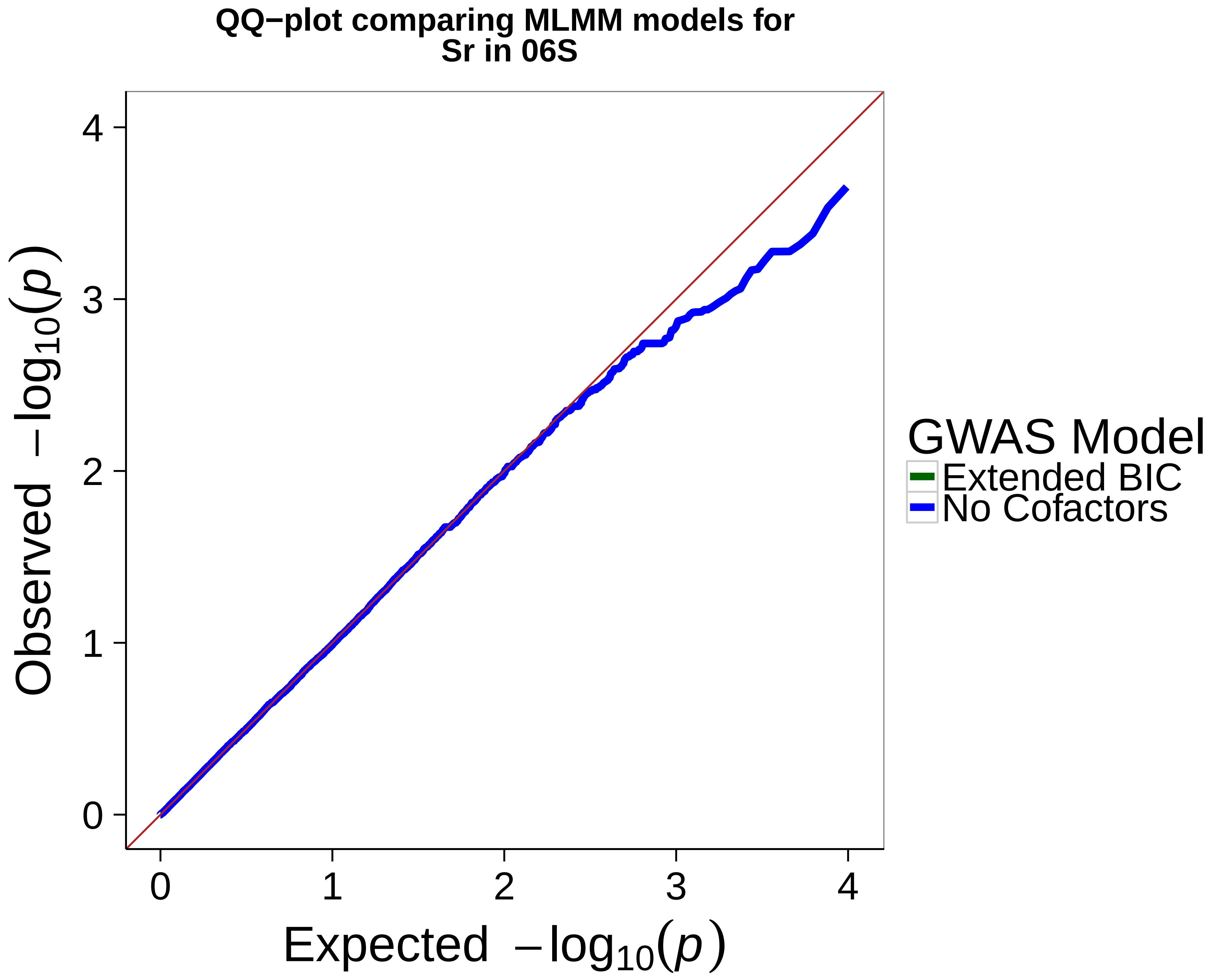
QQ−plot comparing MLMM models for
Sr in 06S
Observed $-\log_{10}(p)$
Expected $-\log_{10}(p)$
0
1
2
3
4
GWAS Model
Extended BIC
No Cofactors

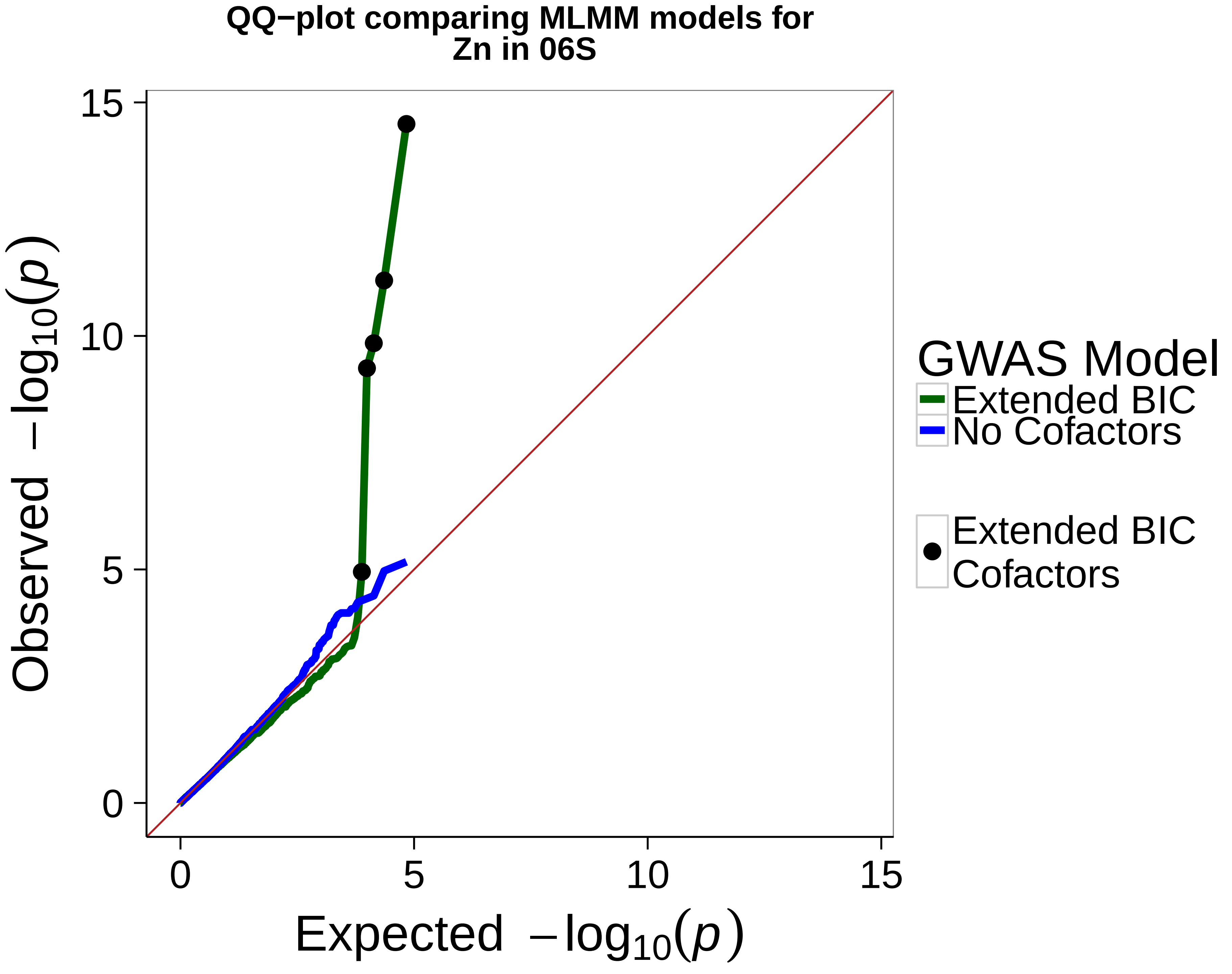
QQ−plot comparing MLMM models for
Zn in 06S
Observed $-\log_{10}(p)$
Expected $-\log_{10}(p)$
0
5
10
15
GWAS Model
Extended BIC
No Cofactors
Extended BIC
Cofactors

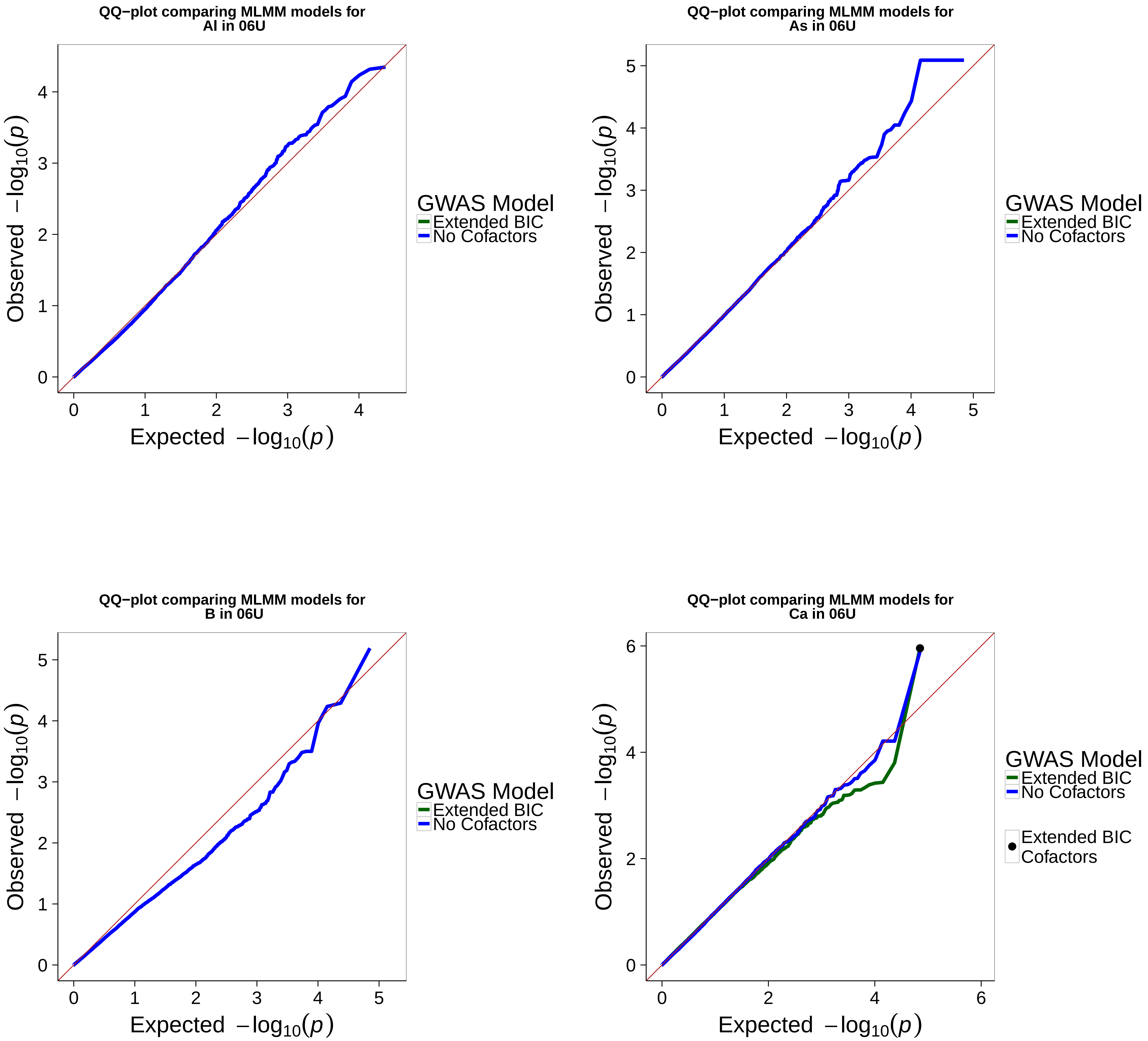
QQ−plot comparing MLMM models for Al in 06U
QQ−plot comparing MLMM models for As in 06U
QQ−plot comparing MLMM models for B in 06U
QQ−plot comparing MLMM models for Ca in 06U
Observed −log10(p)
Expected −log10(p)
GWAS Model
Extended BIC
No Cofactors
Extended BIC Cofactors
0
1
2
3
4
5
6

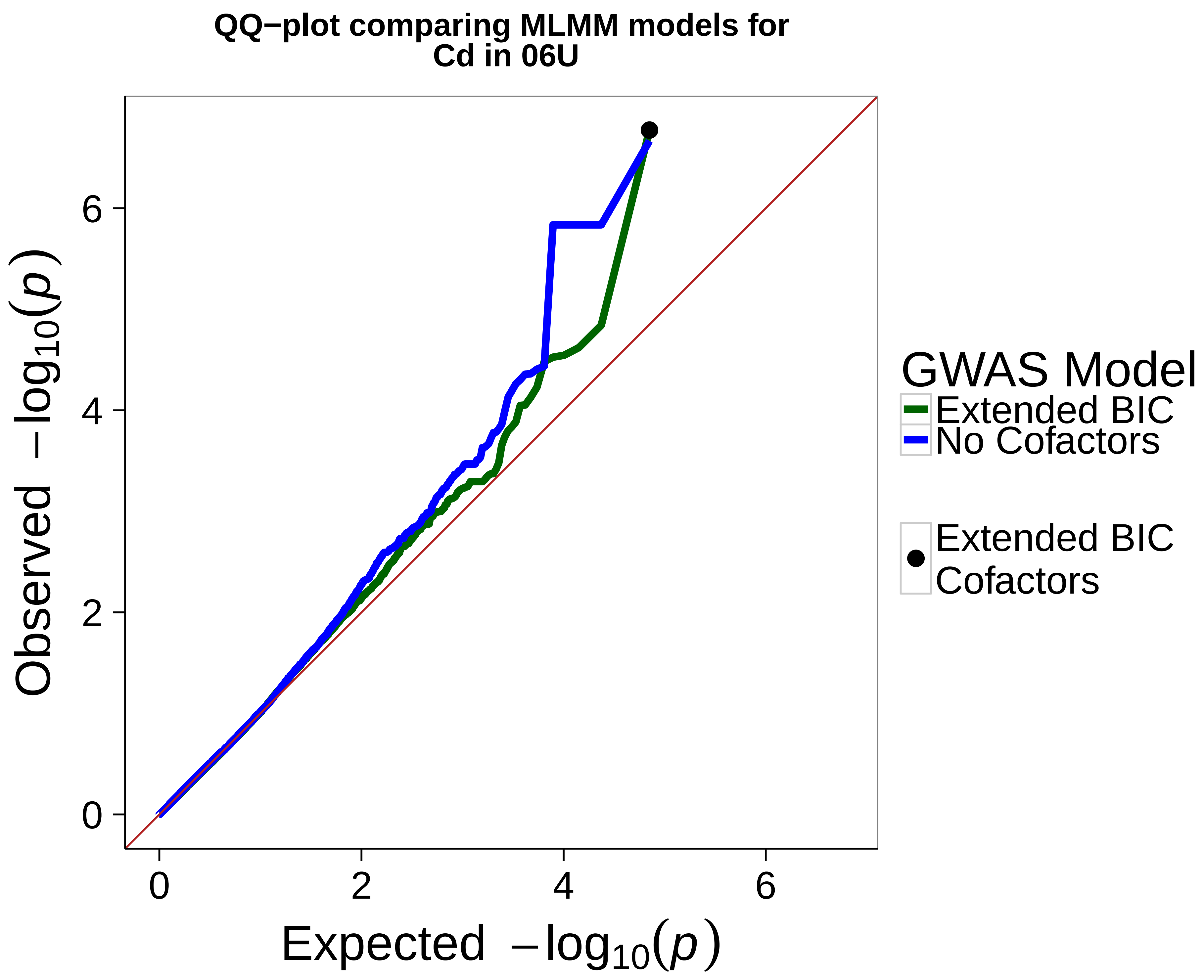
QQ−plot comparing MLMM models for
Cd in 06U
Observed −log10(p)
Expected −log10(p)
GWAS Model
Extended BIC
No Cofactors
Extended BIC
Cofactors

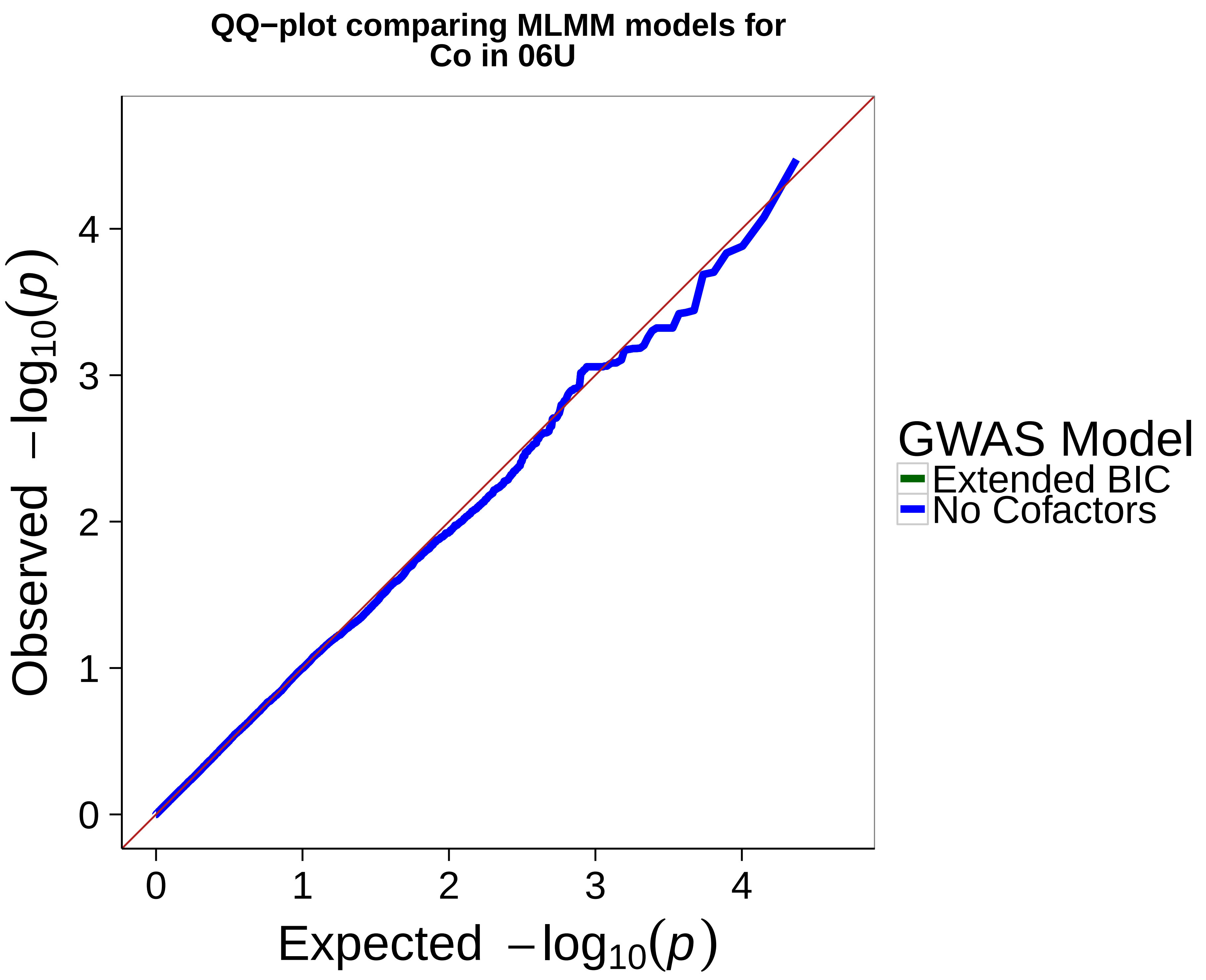
QQ−plot comparing MLMM models for
Co in 06U
Observed −log10(p)
Expected −log10(p)
GWAS Model
Extended BIC
No Cofactors

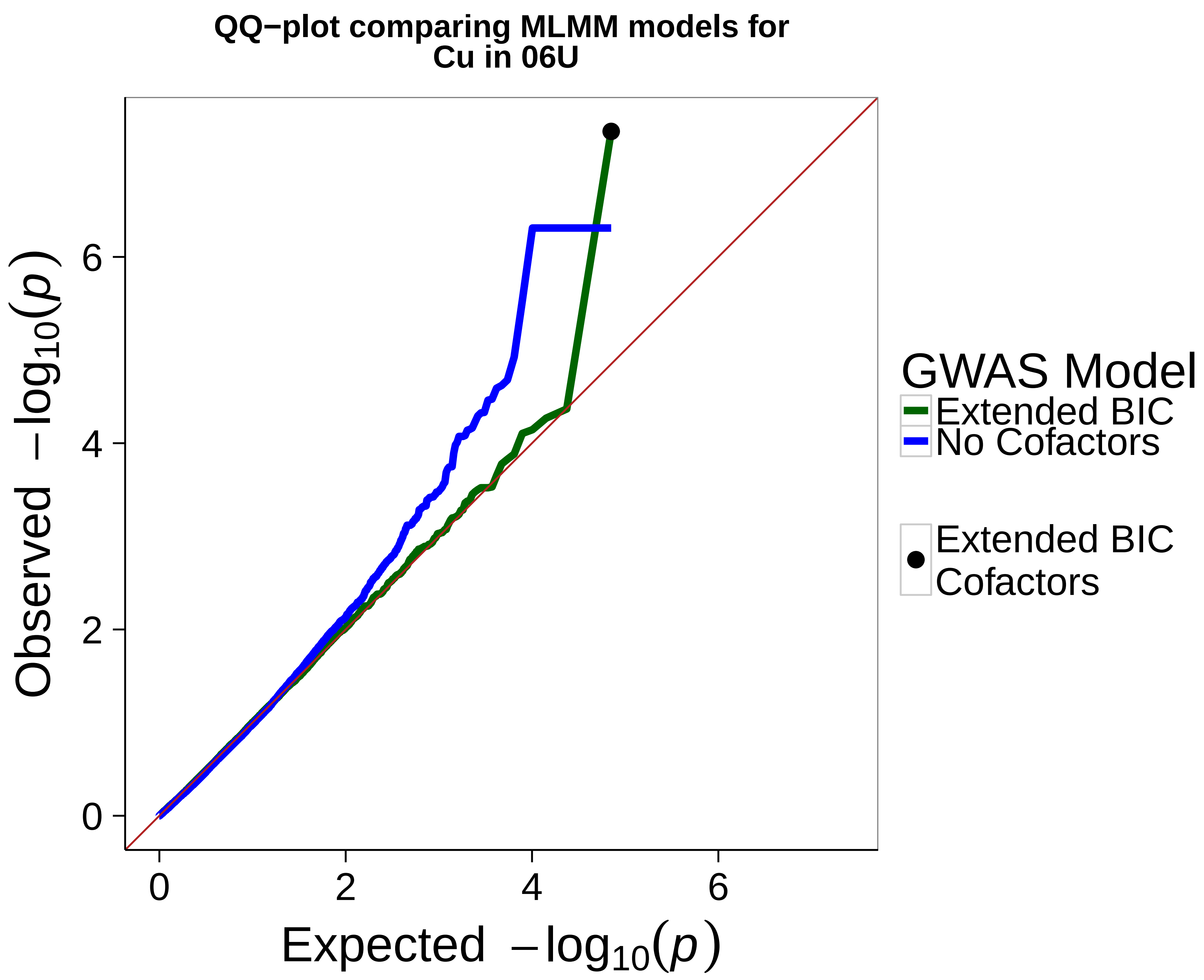
QQ−plot comparing MLMM models for
Cu in 06U
Observed −log10(p)
Expected −log10(p)
GWAS Model
Extended BIC
No Cofactors
Extended BIC
Cofactors

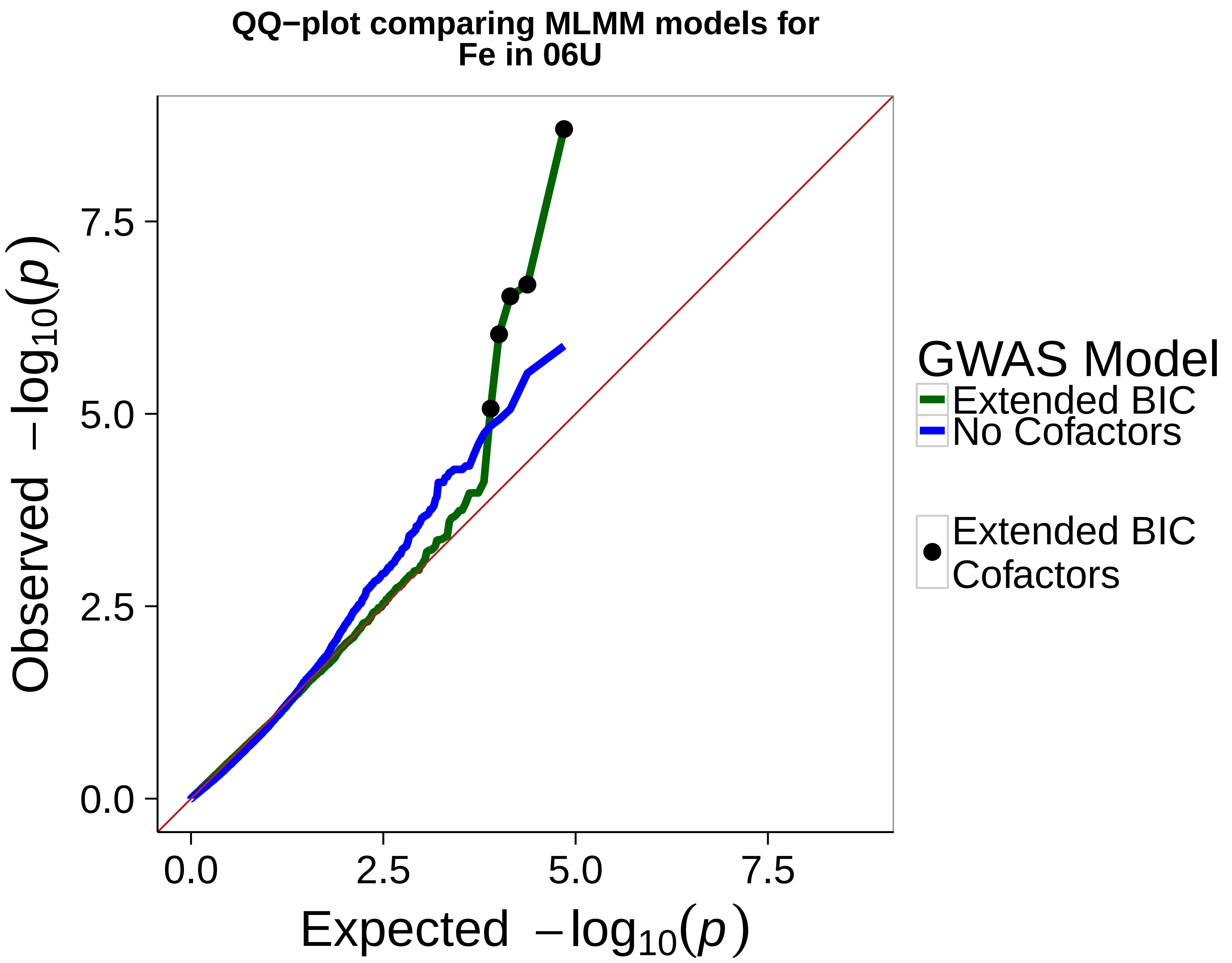
QQ−plot comparing MLMM models for
Fe in 06U
Observed −log10(p)
Expected −log10(p)
GWAS Model
Extended BIC
No Cofactors
Extended BIC
Cofactors

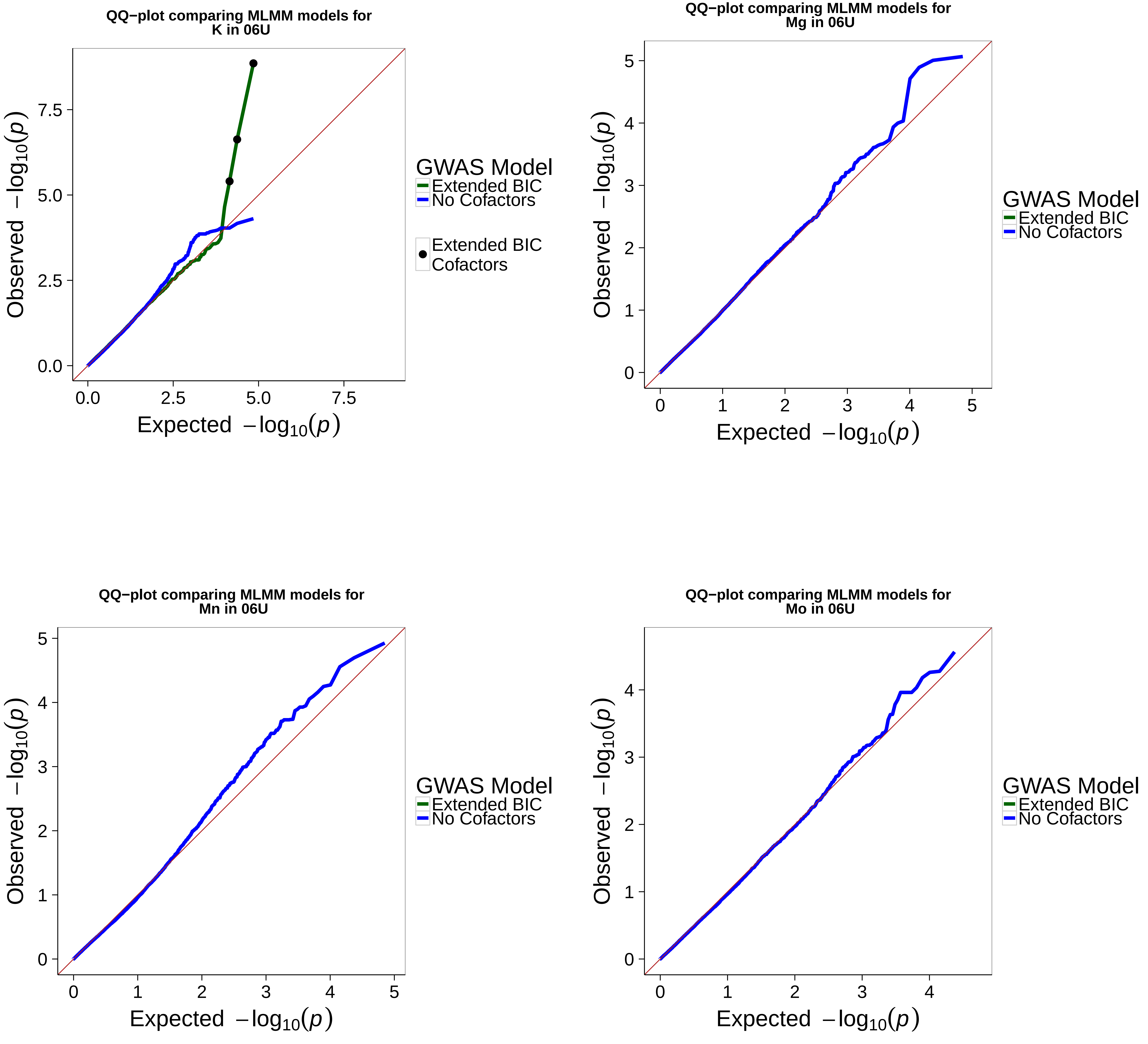
QQ−plot comparing MLMM models for
K in 06U
Observed −log10(p)
Expected −log10(p)
0.0
2.5
5.0
7.5
GWAS Model
Extended BIC
No Cofactors
Extended BIC
Cofactors
QQ−plot comparing MLMM models for
Mg in 06U
Observed −log10(p)
Expected −log10(p)
0
1
2
3
4
5
GWAS Model
Extended BIC
No Cofactors
QQ−plot comparing MLMM models for
Mn in 06U
Observed −log10(p)
Expected −log10(p)
0
1
2
3
4
5
GWAS Model
Extended BIC
No Cofactors
QQ−plot comparing MLMM models for
Mo in 06U
Observed −log10(p)
Expected −log10(p)
0
1
2
3
4
GWAS Model
Extended BIC
No Cofactors

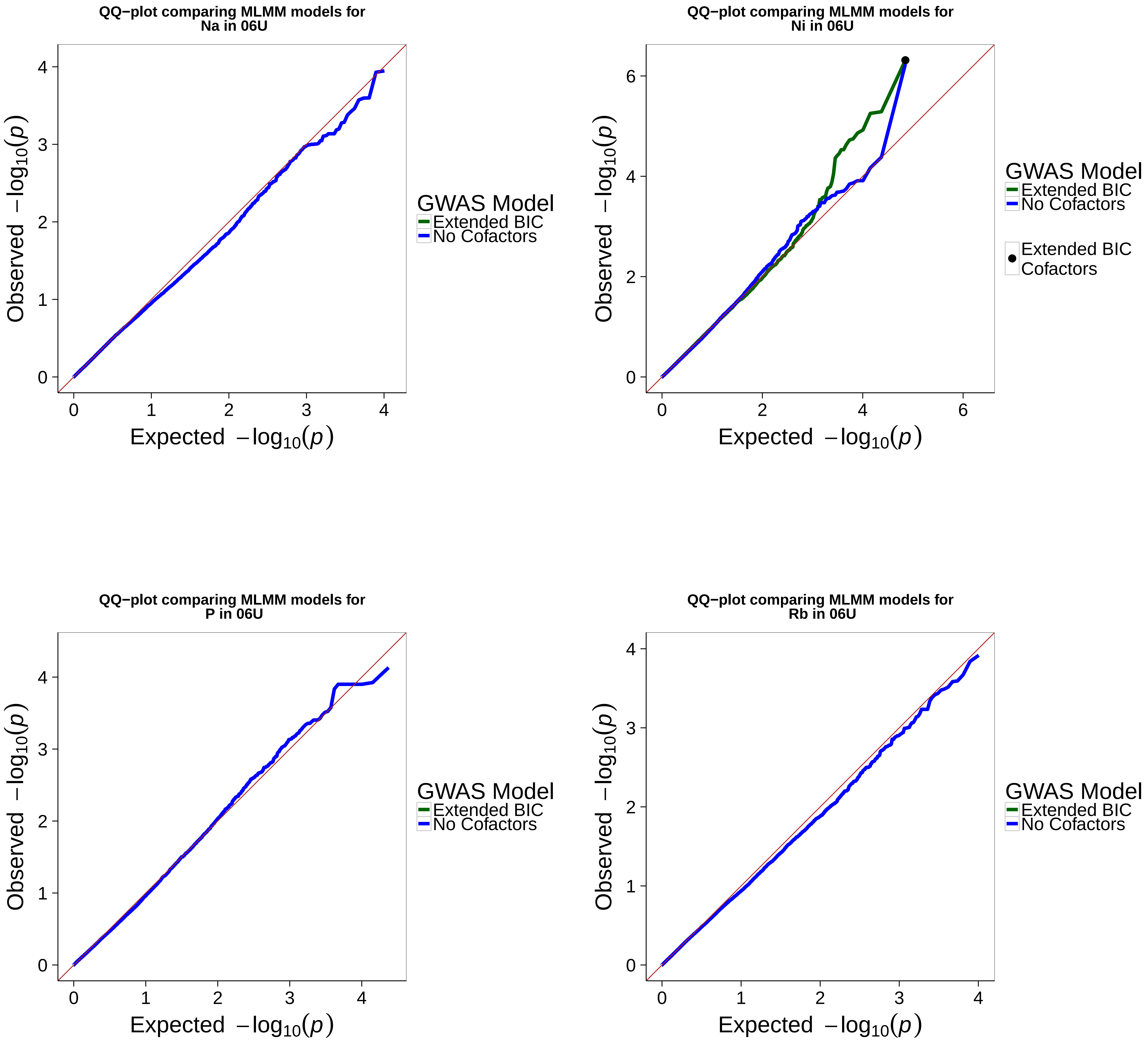
QQ−plot comparing MLMM models for
Na in 06U
Observed −log10(p)
Expected −log10(p)
GWAS Model
Extended BIC
No Cofactors
QQ−plot comparing MLMM models for
Ni in 06U
Observed −log10(p)
Expected −log10(p)
GWAS Model
Extended BIC
No Cofactors
Extended BIC
Cofactors
QQ−plot comparing MLMM models for
P in 06U
Observed −log10(p)
Expected −log10(p)
GWAS Model
Extended BIC
No Cofactors
QQ−plot comparing MLMM models for
Rb in 06U
Observed −log10(p)
Expected −log10(p)
GWAS Model
Extended BIC
No Cofactors

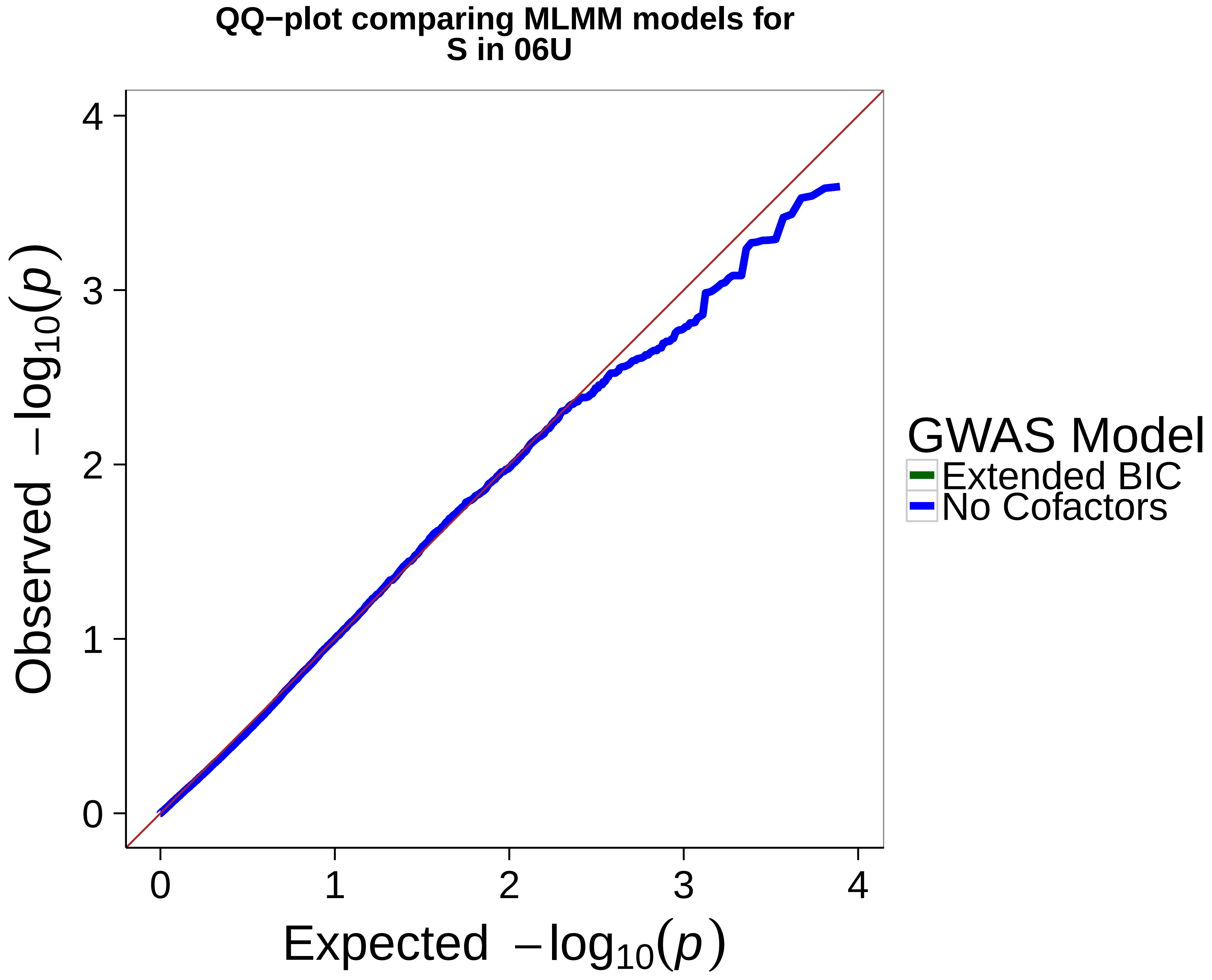
QQ−plot comparing MLMM models for
S in 06U
Observed −log10(p)
Expected −log10(p)
GWAS Model
Extended BIC
No Cofactors

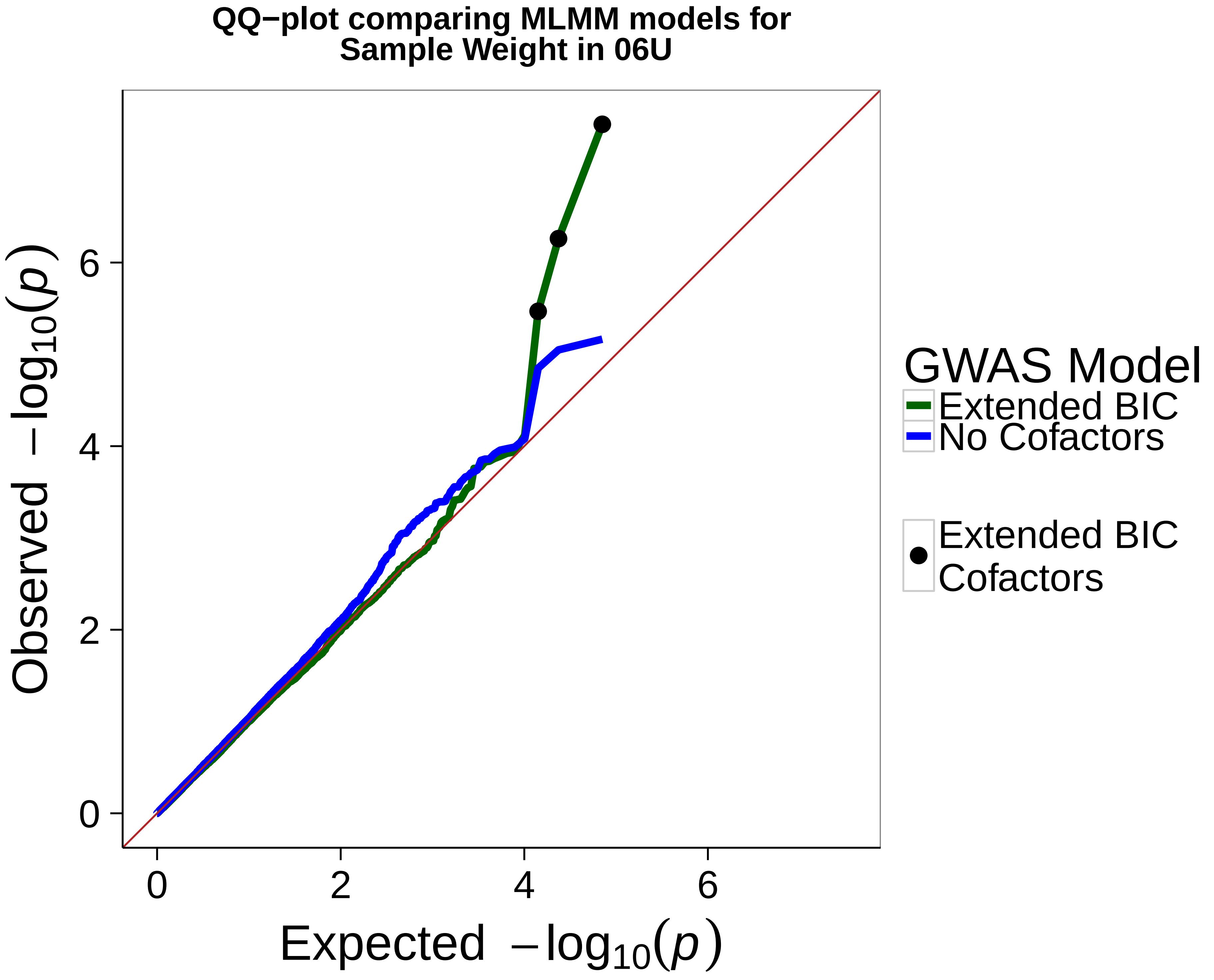
QQ−plot comparing MLMM models for
Sample Weight in 06U
Observed −log10(p)
Expected −log10(p)
GWAS Model
Extended BIC
No Cofactors
Extended BIC
Cofactors

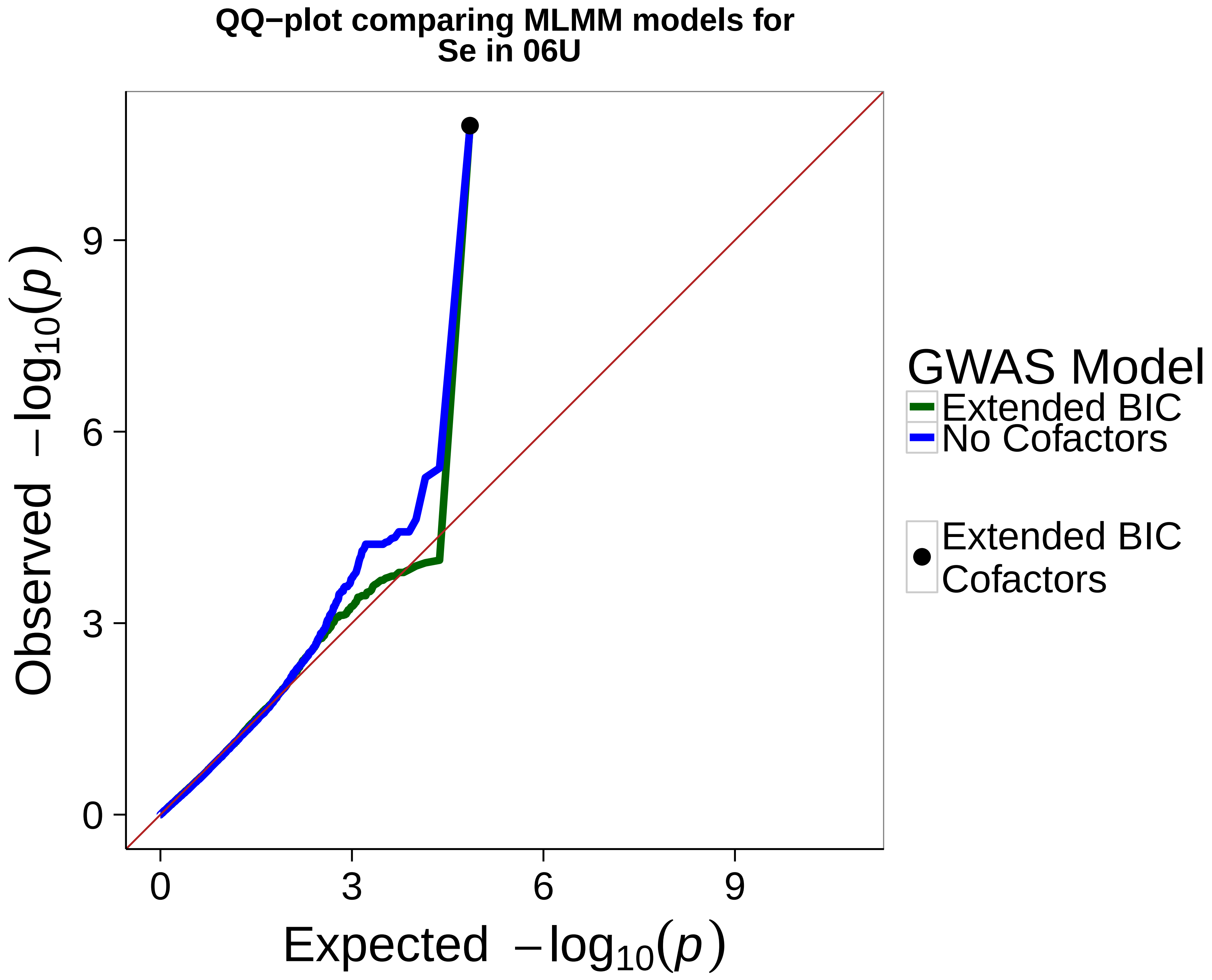
QQ−plot comparing MLMM models for
Se in 06U
Observed −log10(p)
Expected −log10(p)
GWAS Model
Extended BIC
No Cofactors
Extended BIC
Cofactors

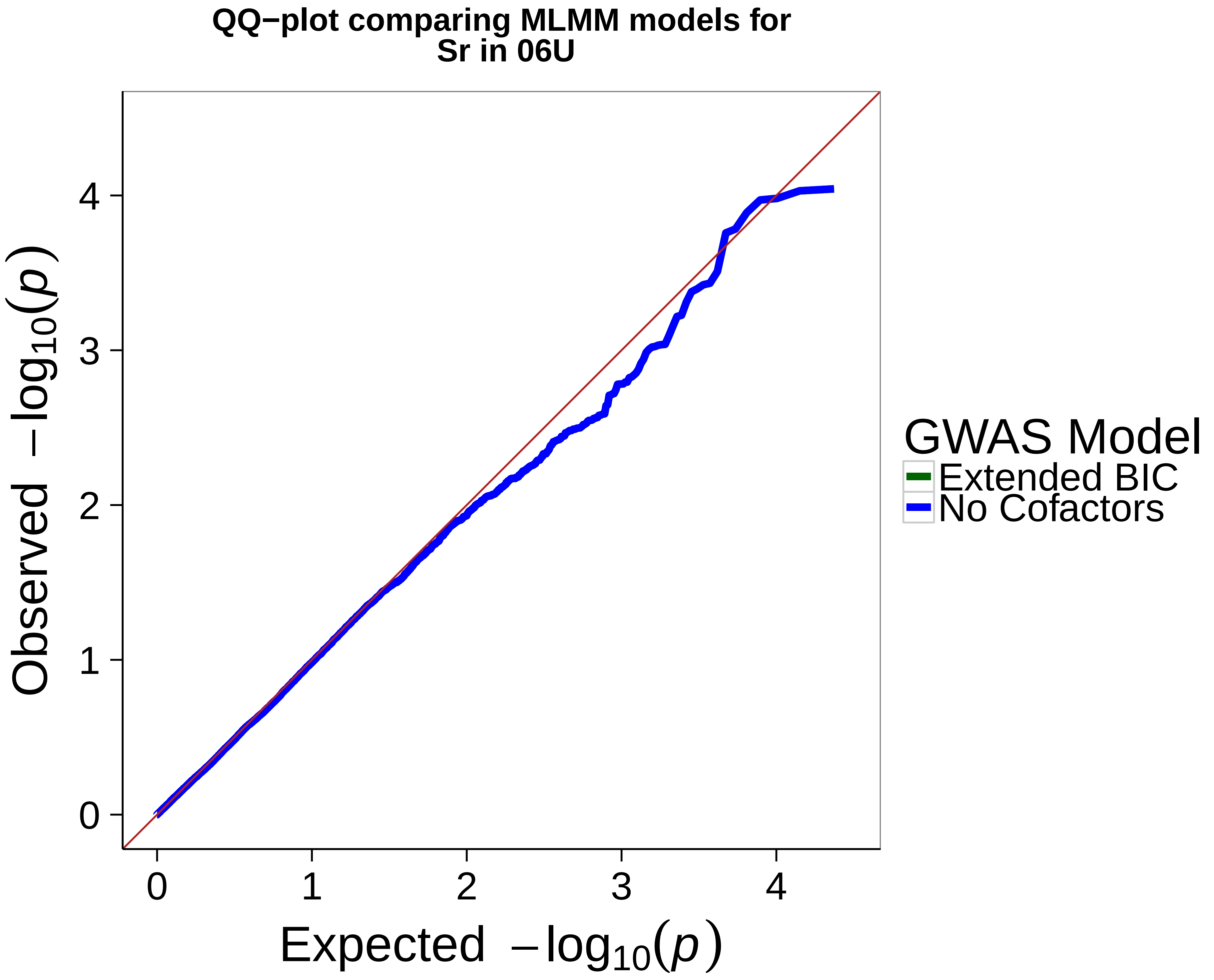
QQ−plot comparing MLMM models for
Sr in 06U
Observed −log10(p)
Expected −log10(p)
GWAS Model
Extended BIC
No Cofactors

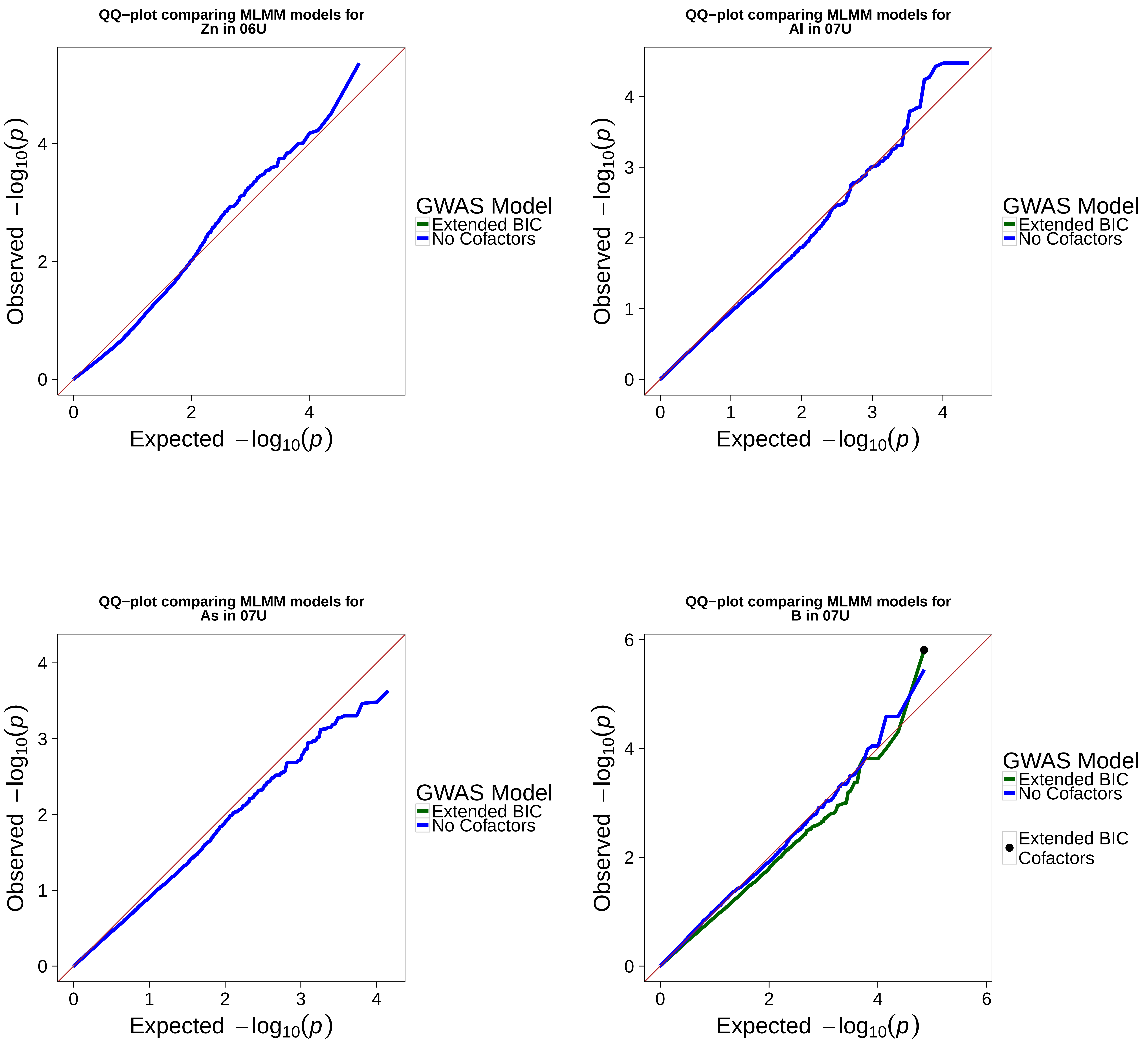
QQ−plot comparing MLMM models for
Zn in 06U
Observed −log10(p)
Expected −log10(p)
GWAS Model
Extended BIC
No Cofactors
QQ−plot comparing MLMM models for
Al in 07U
Observed −log10(p)
Expected −log10(p)
GWAS Model
Extended BIC
No Cofactors
QQ−plot comparing MLMM models for
As in 07U
Observed −log10(p)
Expected −log10(p)
GWAS Model
Extended BIC
No Cofactors
QQ−plot comparing MLMM models for
B in 07U
Observed −log10(p)
Expected −log10(p)
GWAS Model
Extended BIC
No Cofactors
Extended BIC
Cofactors

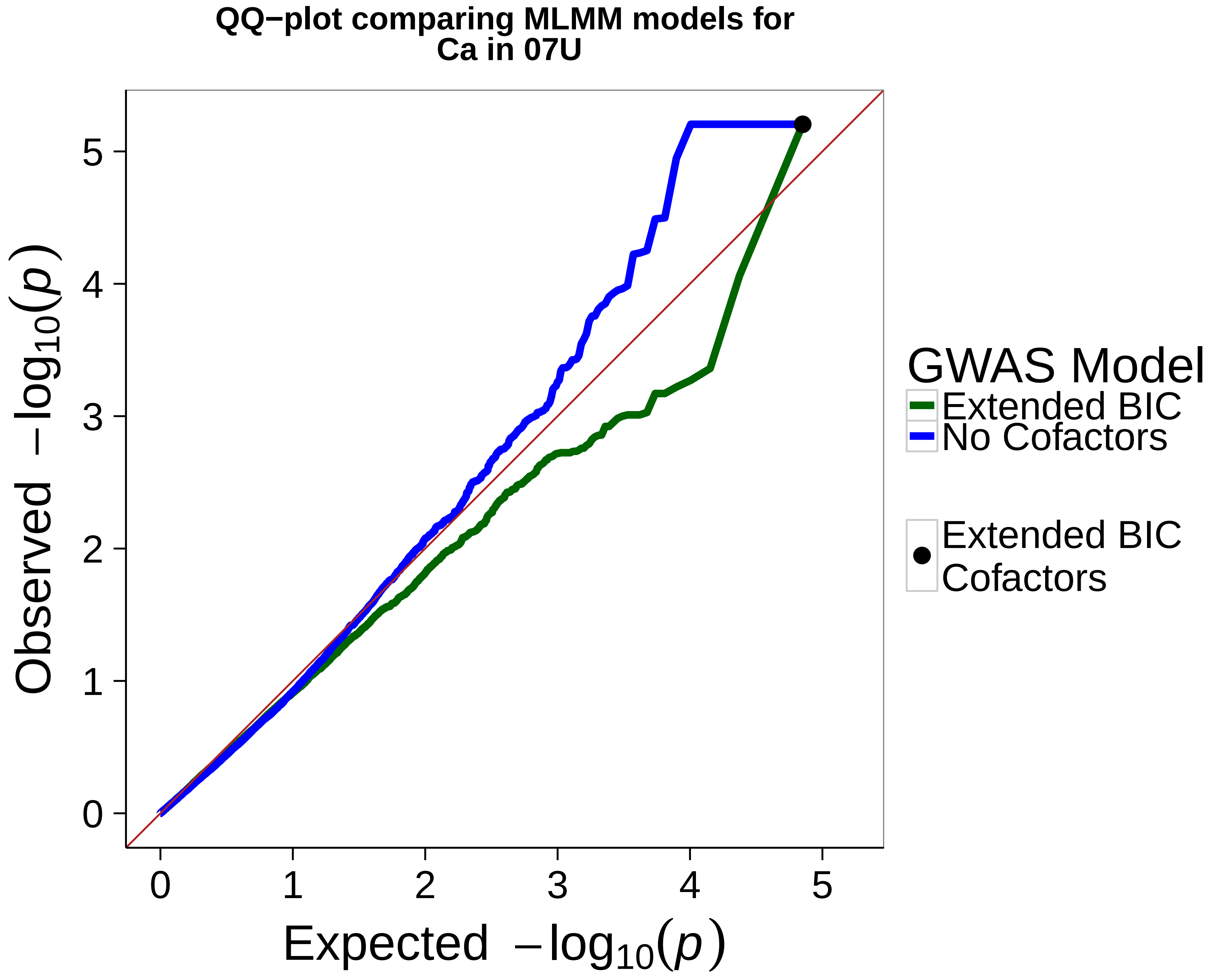
QQ−plot comparing MLMM models for
Ca in 07U
Observed −log10(p)
Expected −log10(p)
GWAS Model
Extended BIC
No Cofactors
Extended BIC
Cofactors

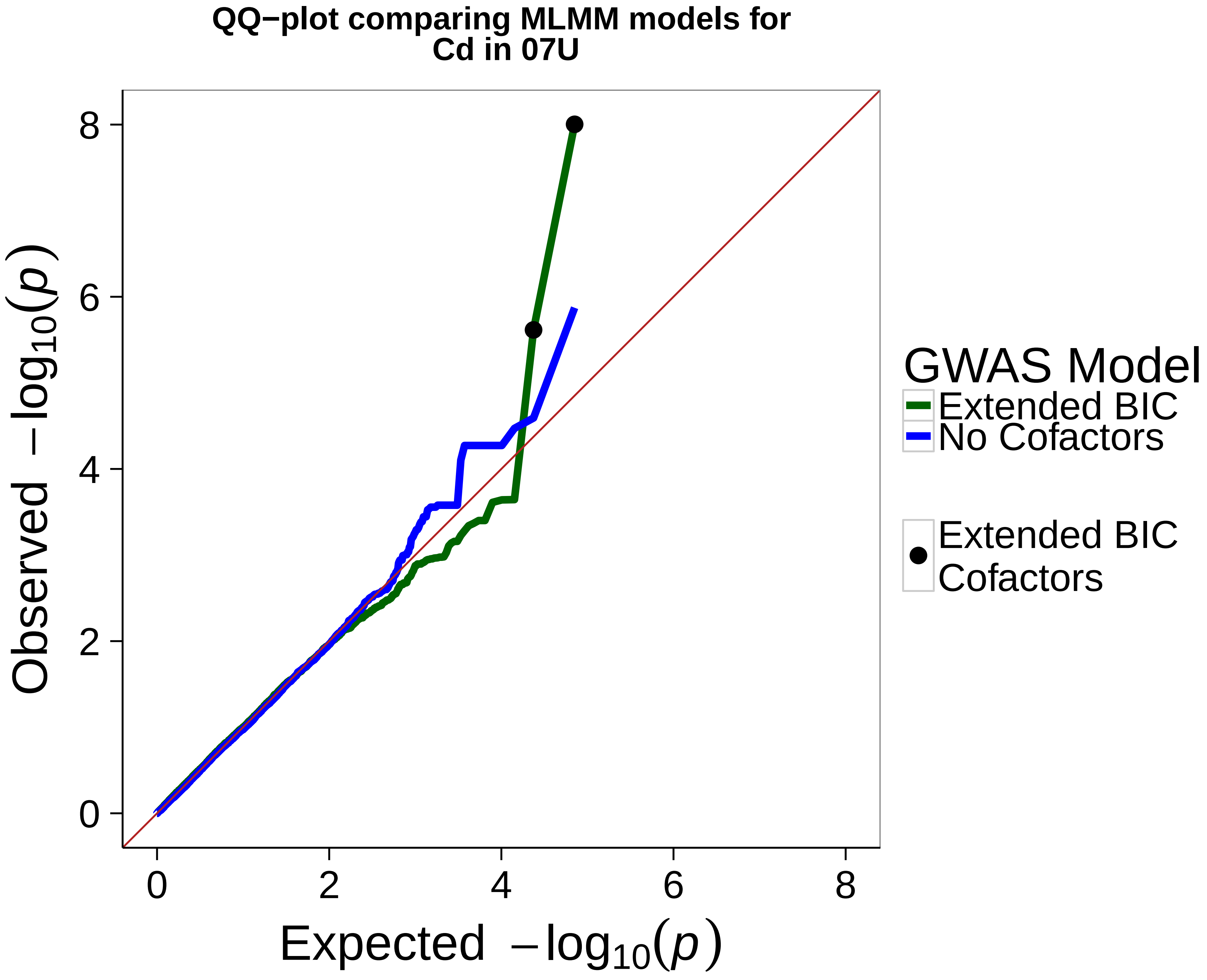
QQ−plot comparing MLMM models for
Cd in 07U
Observed −log10(p)
Expected −log10(p)
GWAS Model
Extended BIC
No Cofactors
Extended BIC
Cofactors

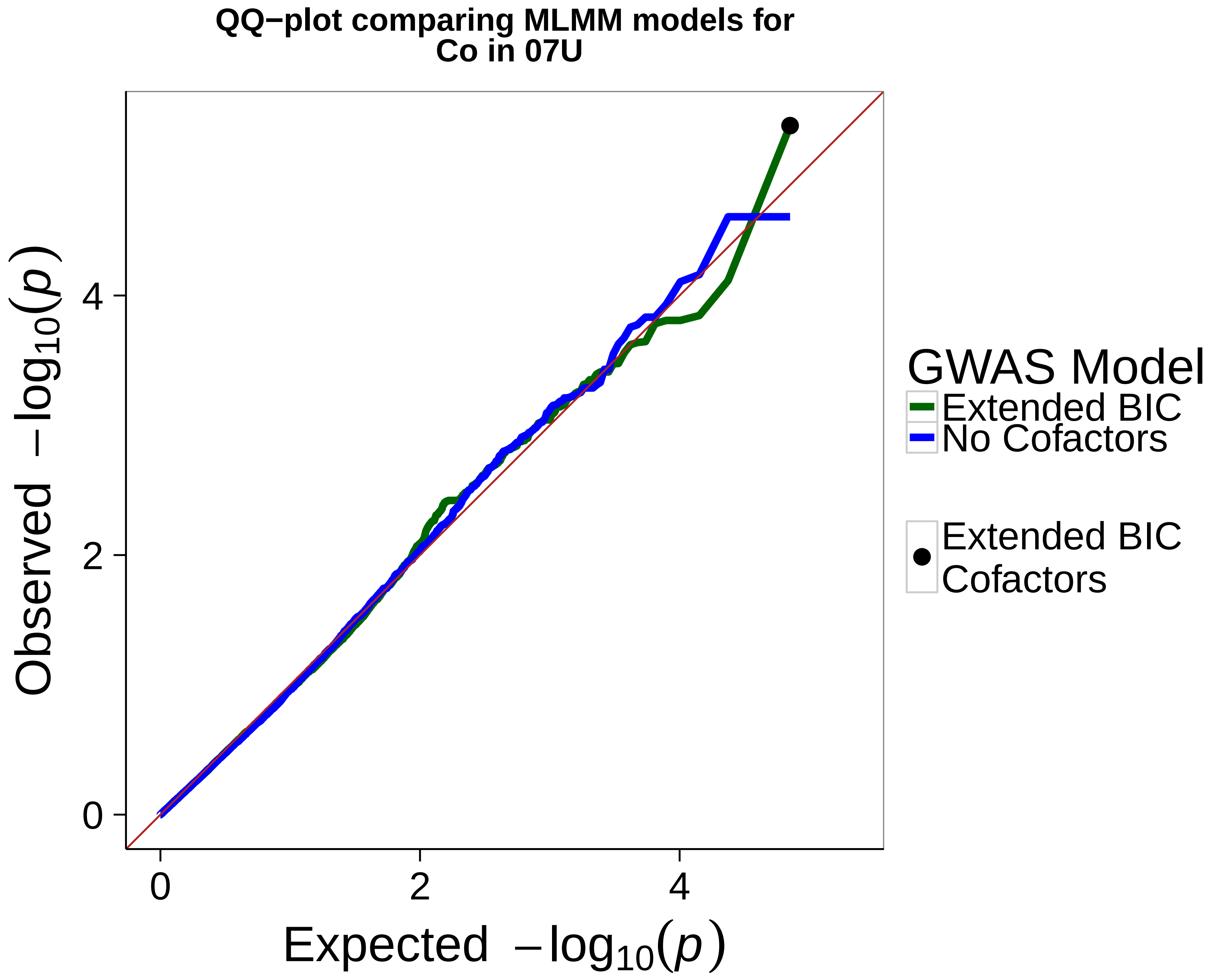
QQ−plot comparing MLMM models for
Co in 07U
Observed −log10(p)
Expected −log10(p)
GWAS Model
Extended BIC
No Cofactors
Extended BIC
Cofactors

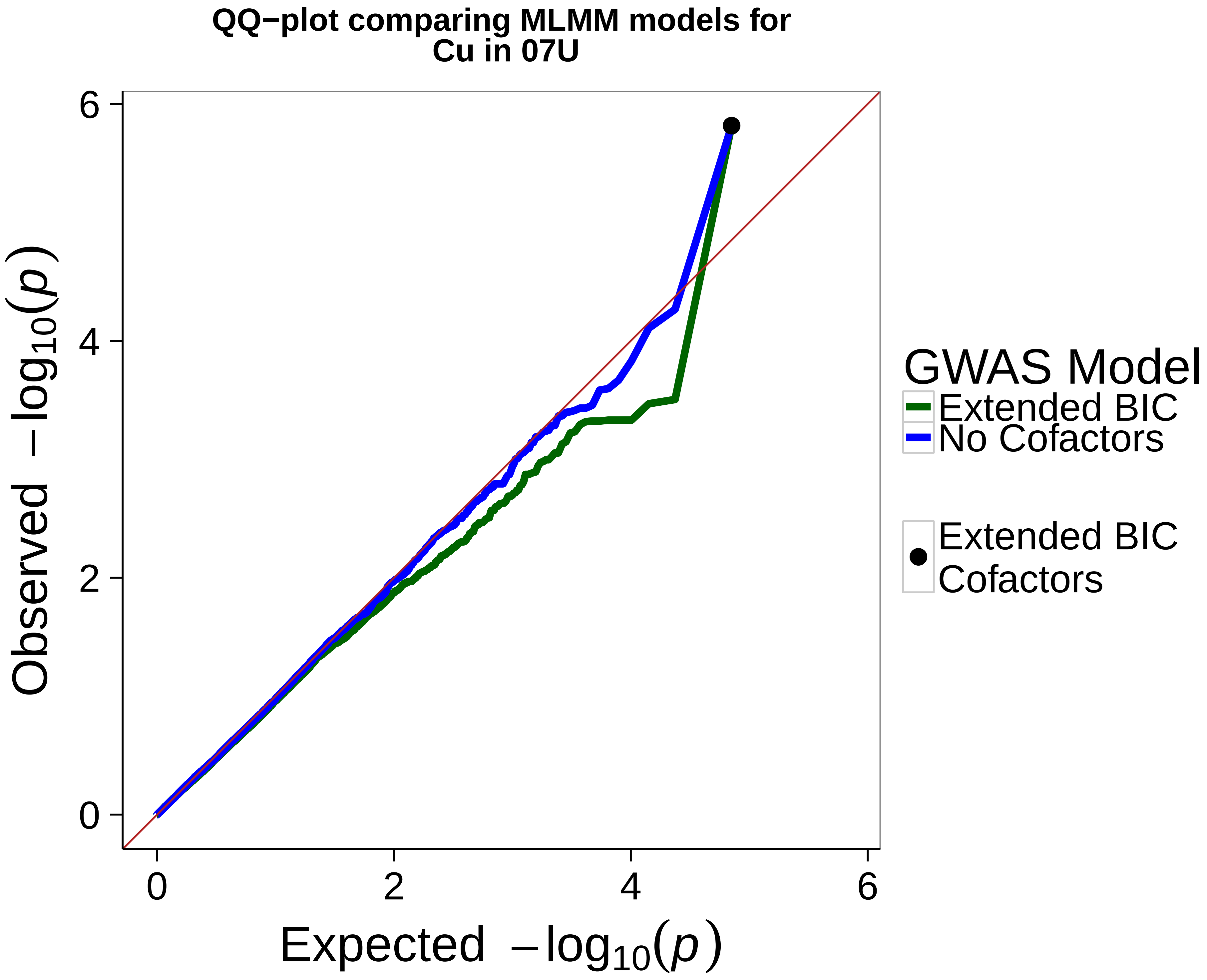
QQ−plot comparing MLMM models for
Cu in 07U
Observed −log10(p)
Expected −log10(p)
GWAS Model
Extended BIC
No Cofactors
Extended BIC
Cofactors

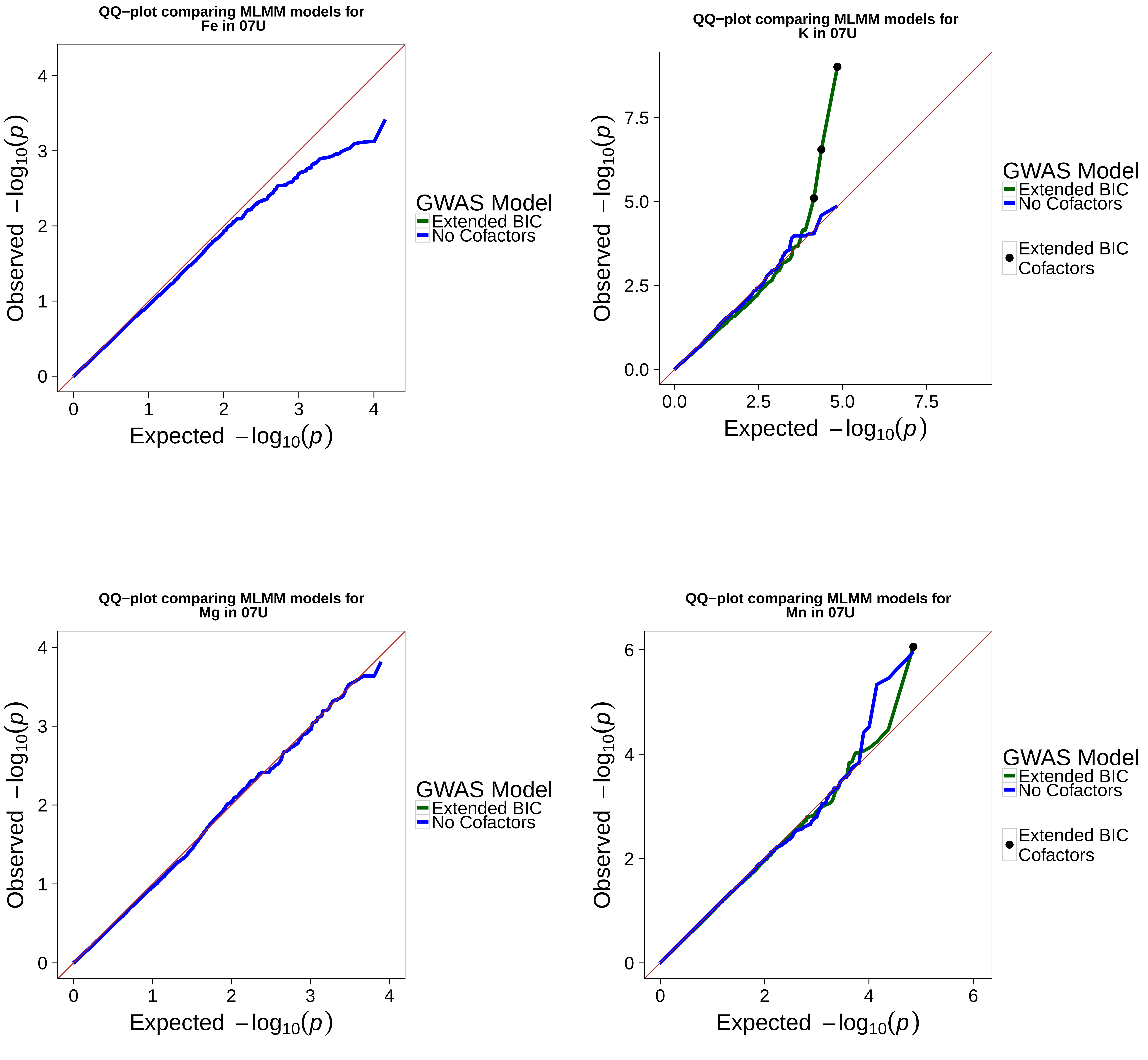
QQ−plot comparing MLMM models for
Fe in 07U
Observed −log10(p)
Expected −log10(p)
GWAS Model
Extended BIC
No Cofactors
QQ−plot comparing MLMM models for
K in 07U
Observed −log10(p)
Expected −log10(p)
GWAS Model
Extended BIC
No Cofactors
Extended BIC
Cofactors
QQ−plot comparing MLMM models for
Mg in 07U
Observed −log10(p)
Expected −log10(p)
GWAS Model
Extended BIC
No Cofactors
QQ−plot comparing MLMM models for
Mn in 07U
Observed −log10(p)
Expected −log10(p)
GWAS Model
Extended BIC
No Cofactors
Extended BIC
Cofactors

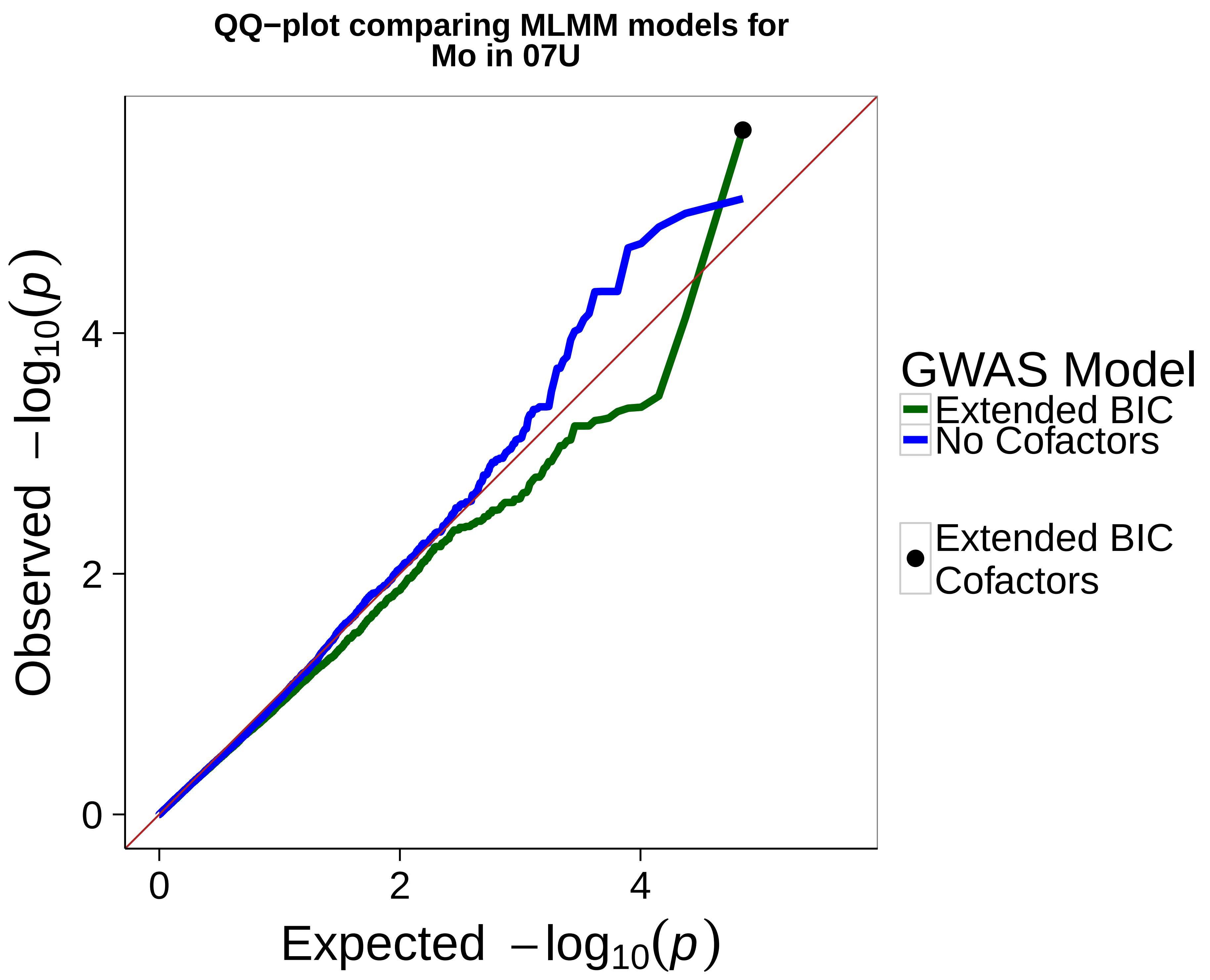
QQ−plot comparing MLMM models for
Mo in 07U
Observed −log10(p)
Expected −log10(p)
GWAS Model
Extended BIC
No Cofactors
Extended BIC
Cofactors
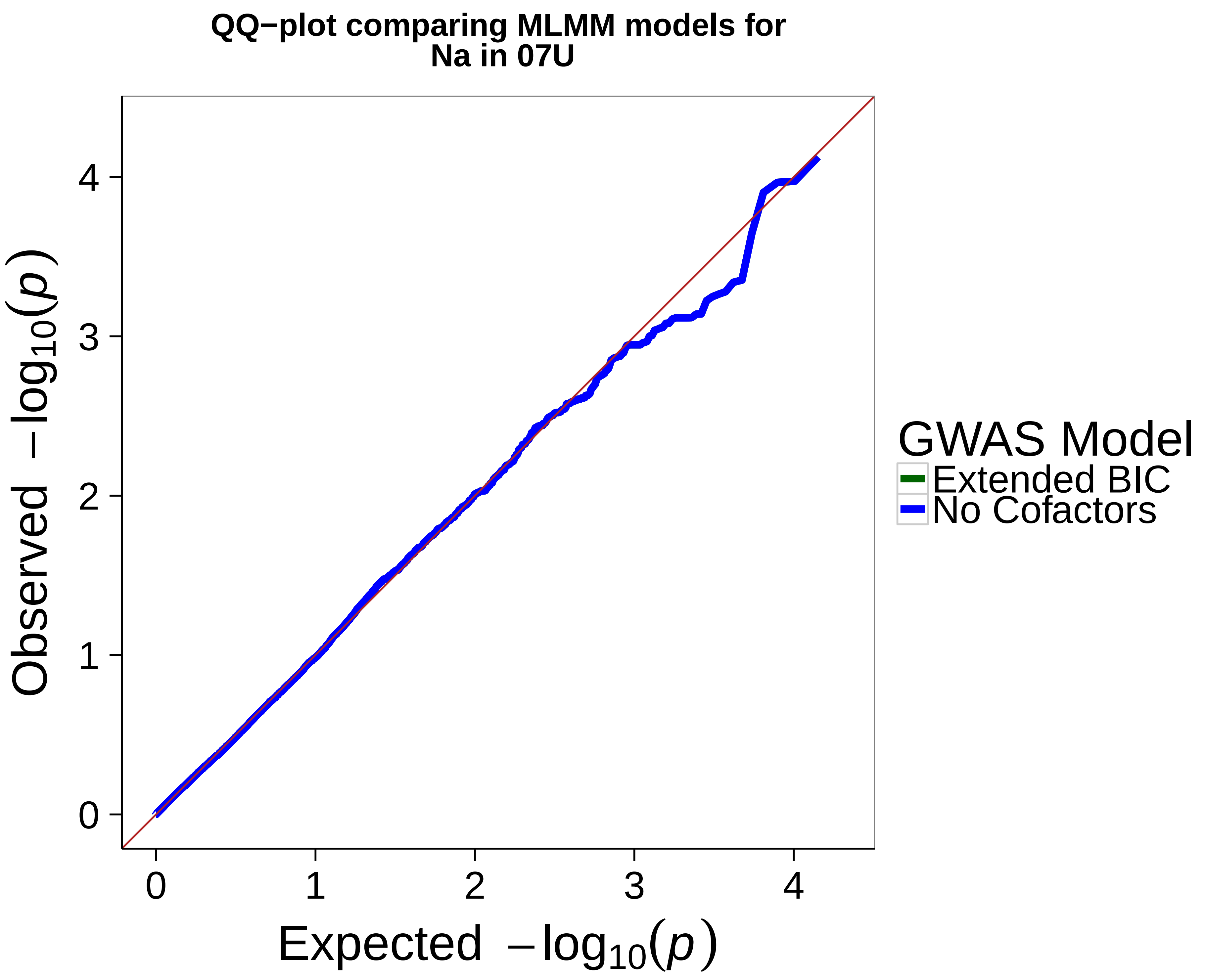
QQ−plot comparing MLMM models for
Na in 07U
Observed −log10(p)
Expected −log10(p)
GWAS Model
Extended BIC
No Cofactors
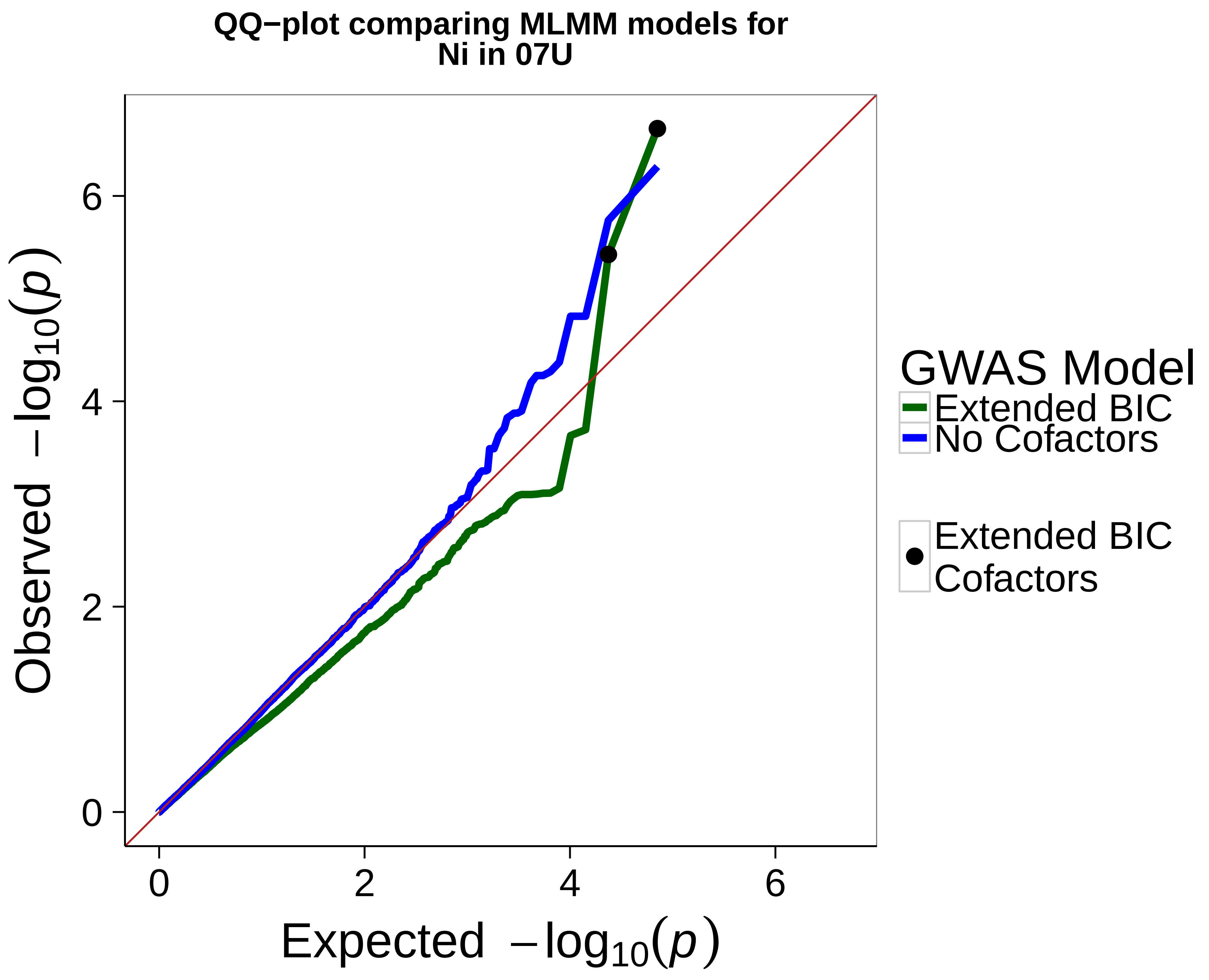
QQ−plot comparing MLMM models for
Ni in 07U
Observed −log10(p)
Expected −log10(p)
GWAS Model
Extended BIC
No Cofactors
Extended BIC
Cofactors
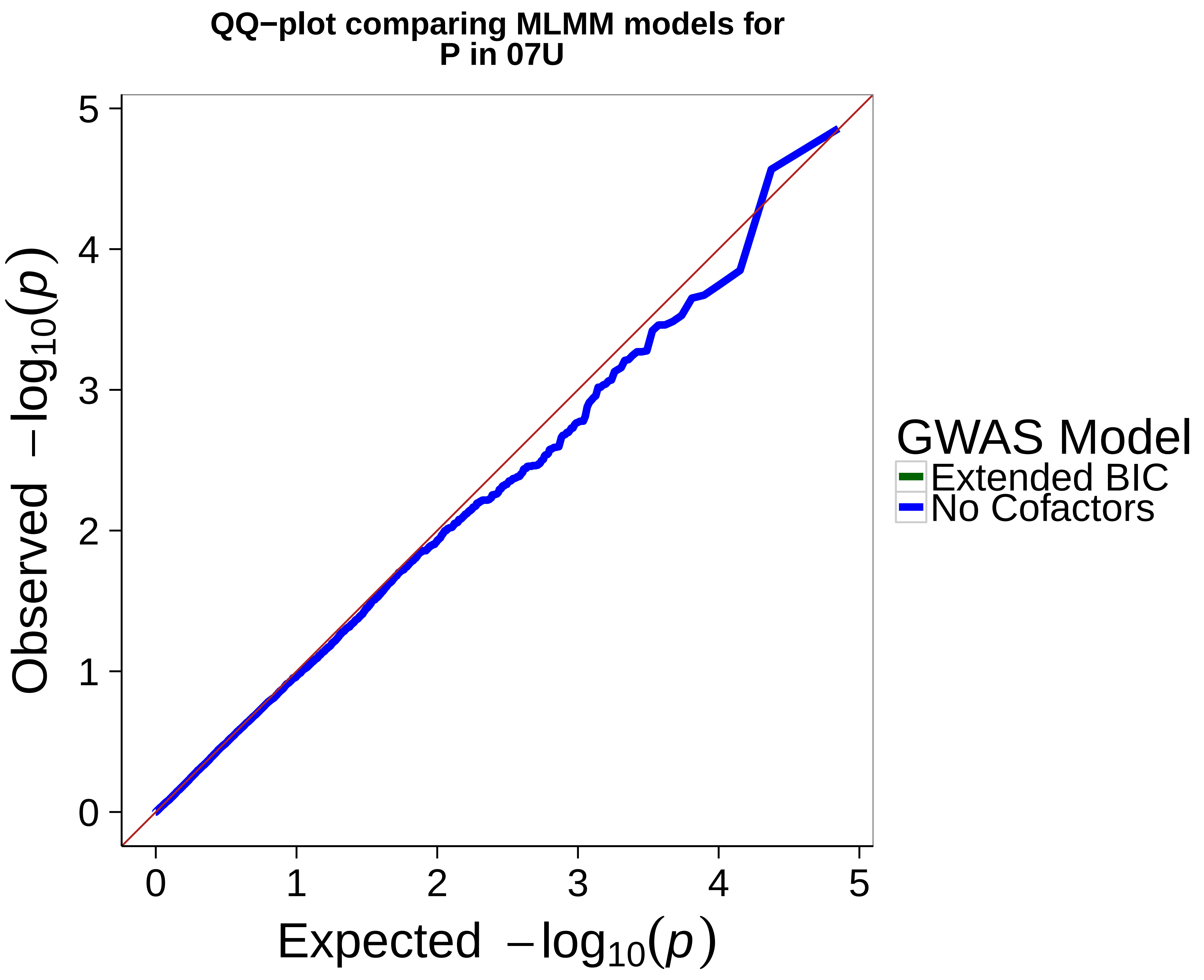
QQ−plot comparing MLMM models for
P in 07U
Observed −log10(p)
Expected −log10(p)
GWAS Model
Extended BIC
No Cofactors

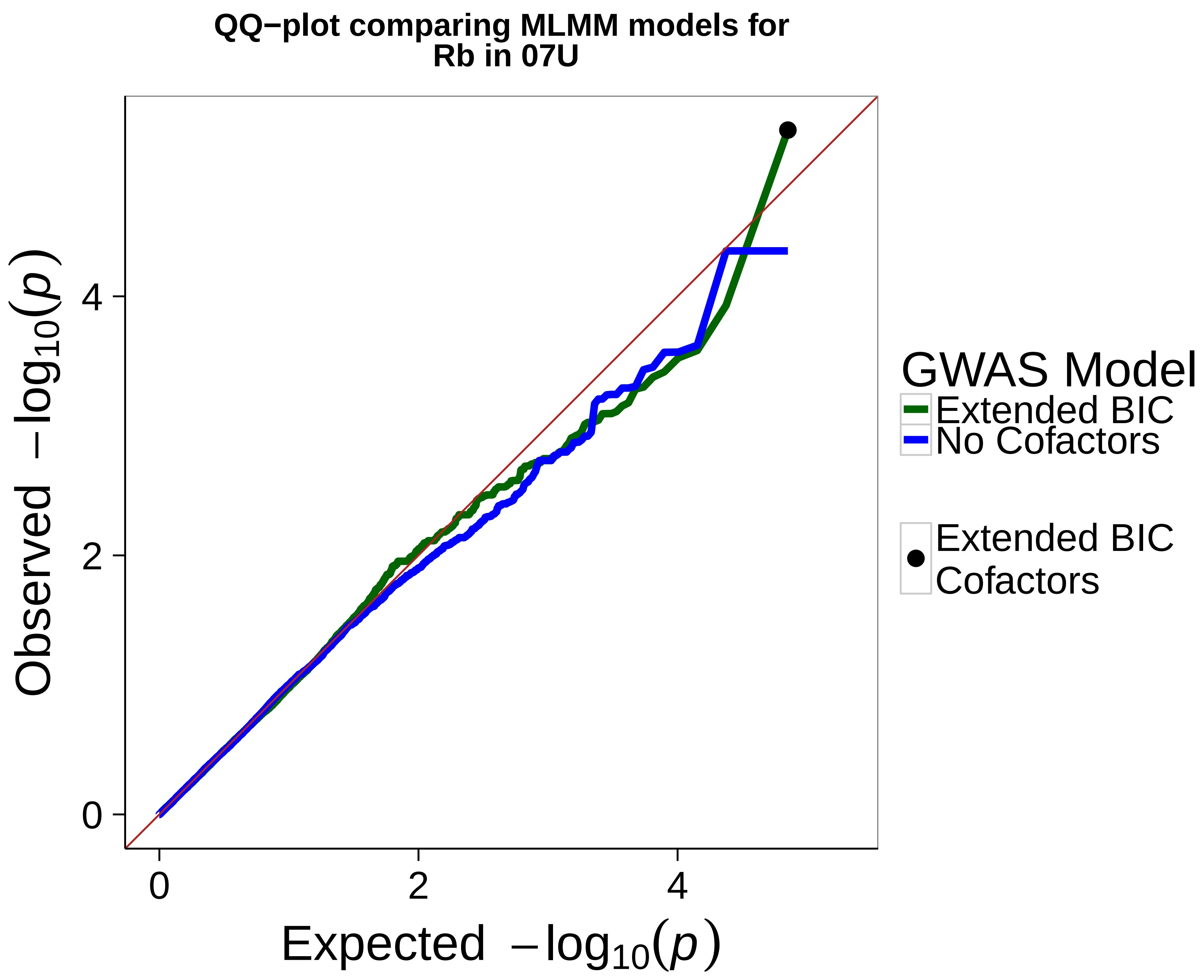
QQ−plot comparing MLMM models for
Rb in 07U
Observed −log10(p)
Expected −log10(p)
0
2
4
GWAS Model
Extended BIC
No Cofactors
Extended BIC
Cofactors

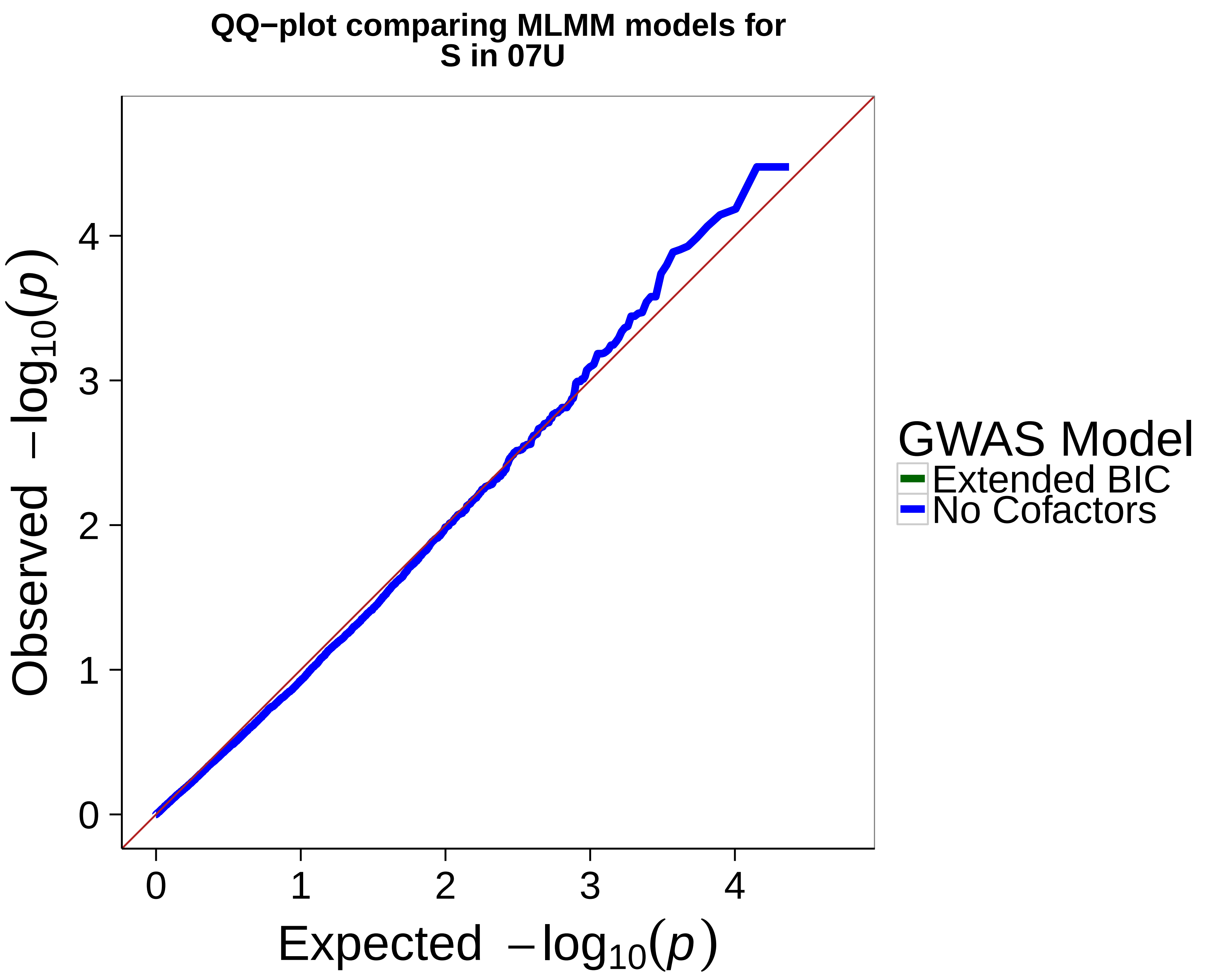
QQ−plot comparing MLMM models for
S in 07U
Observed −log10(p)
Expected −log10(p)
0
1
2
3
4
GWAS Model
Extended BIC
No Cofactors

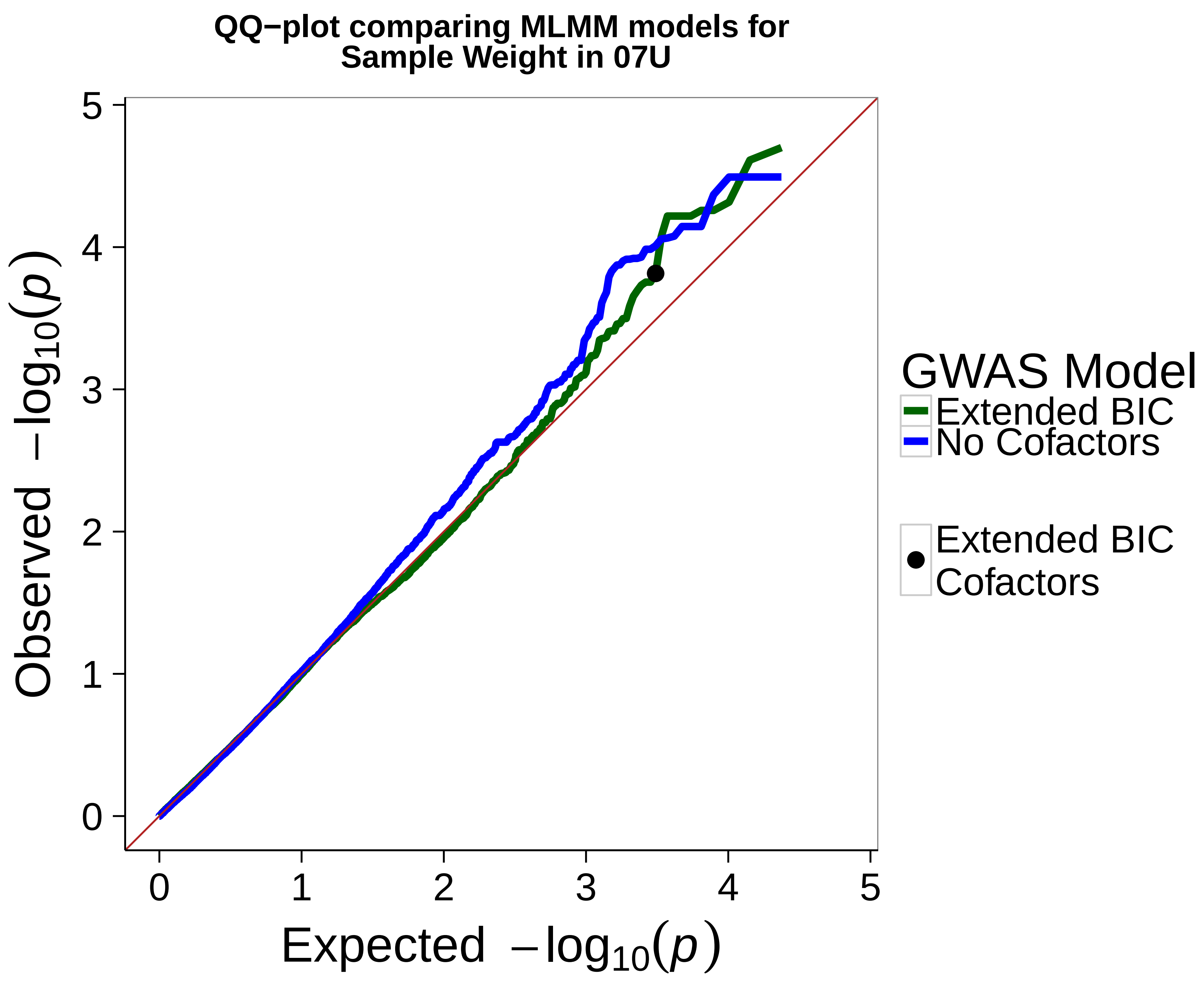
QQ−plot comparing MLMM models for
Sample Weight in 07U
Observed −log10(p)
Expected −log10(p)
0
1
2
3
4
5
GWAS Model
Extended BIC
No Cofactors
Extended BIC
Cofactors

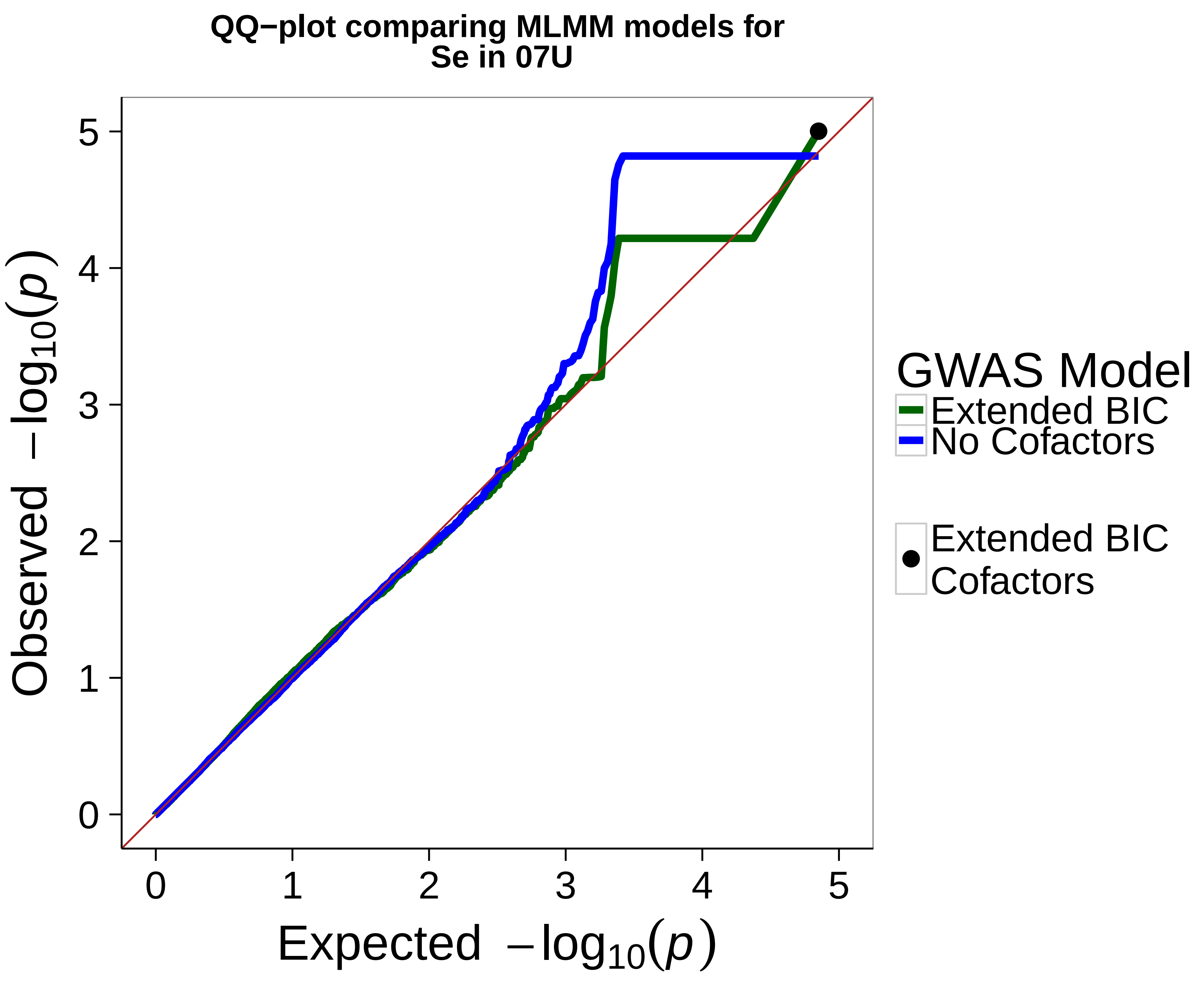
QQ−plot comparing MLMM models for
Se in 07U
Observed −log10(p)
Expected −log10(p)
0
1
2
3
4
5
GWAS Model
Extended BIC
No Cofactors
Extended BIC
Cofactors

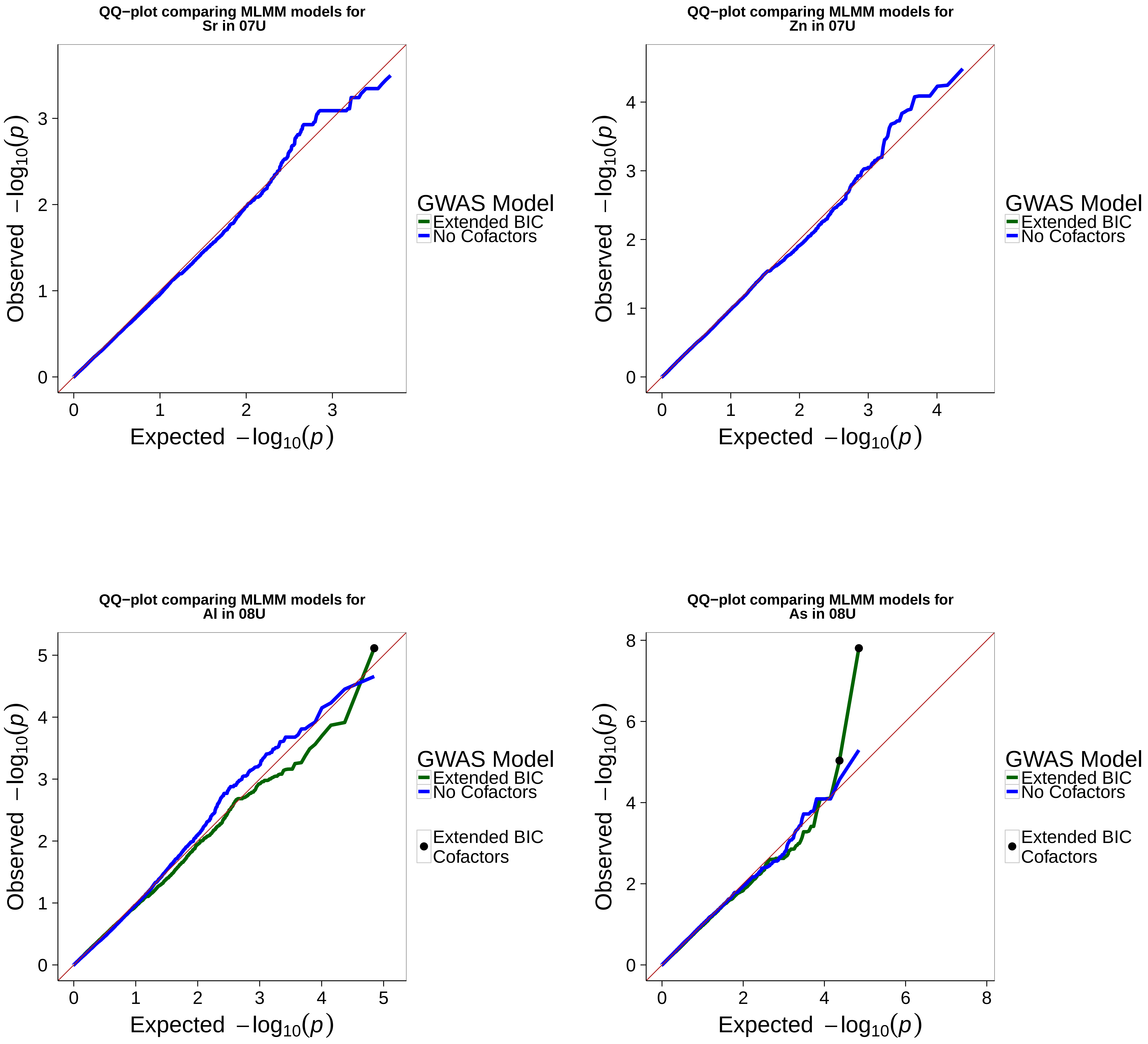
QQ−plot comparing MLMM models for Sr in 07U
QQ−plot comparing MLMM models for Zn in 07U
QQ−plot comparing MLMM models for Al in 08U
QQ−plot comparing MLMM models for As in 08U
Observed $-\log_{10}(p)$
Expected $-\log_{10}(p)$
GWAS Model
Extended BIC
No Cofactors
Extended BIC Cofactors

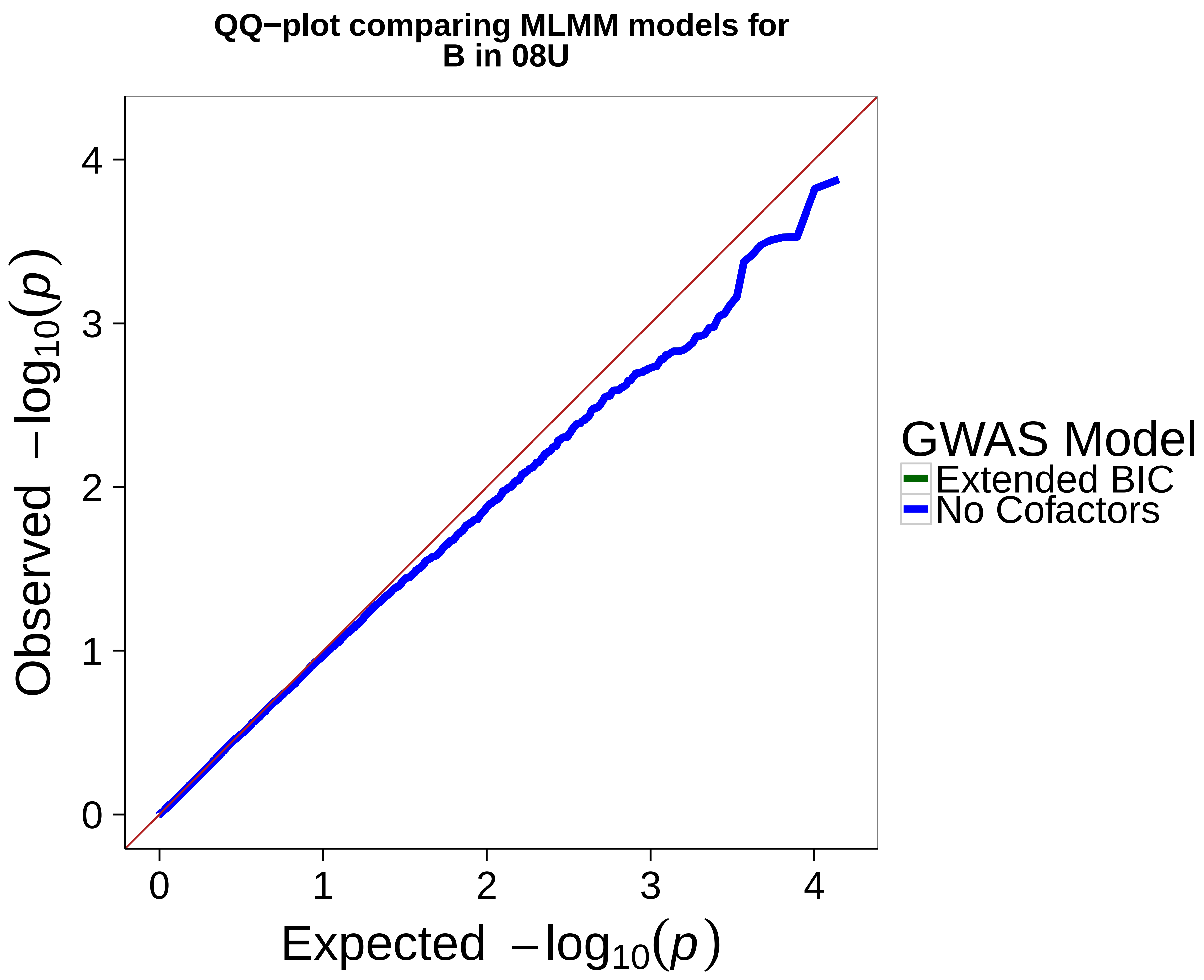
QQ−plot comparing MLMM models for
B in 08U
Observed −log10(p)
Expected −log10(p)
GWAS Model
Extended BIC
No Cofactors

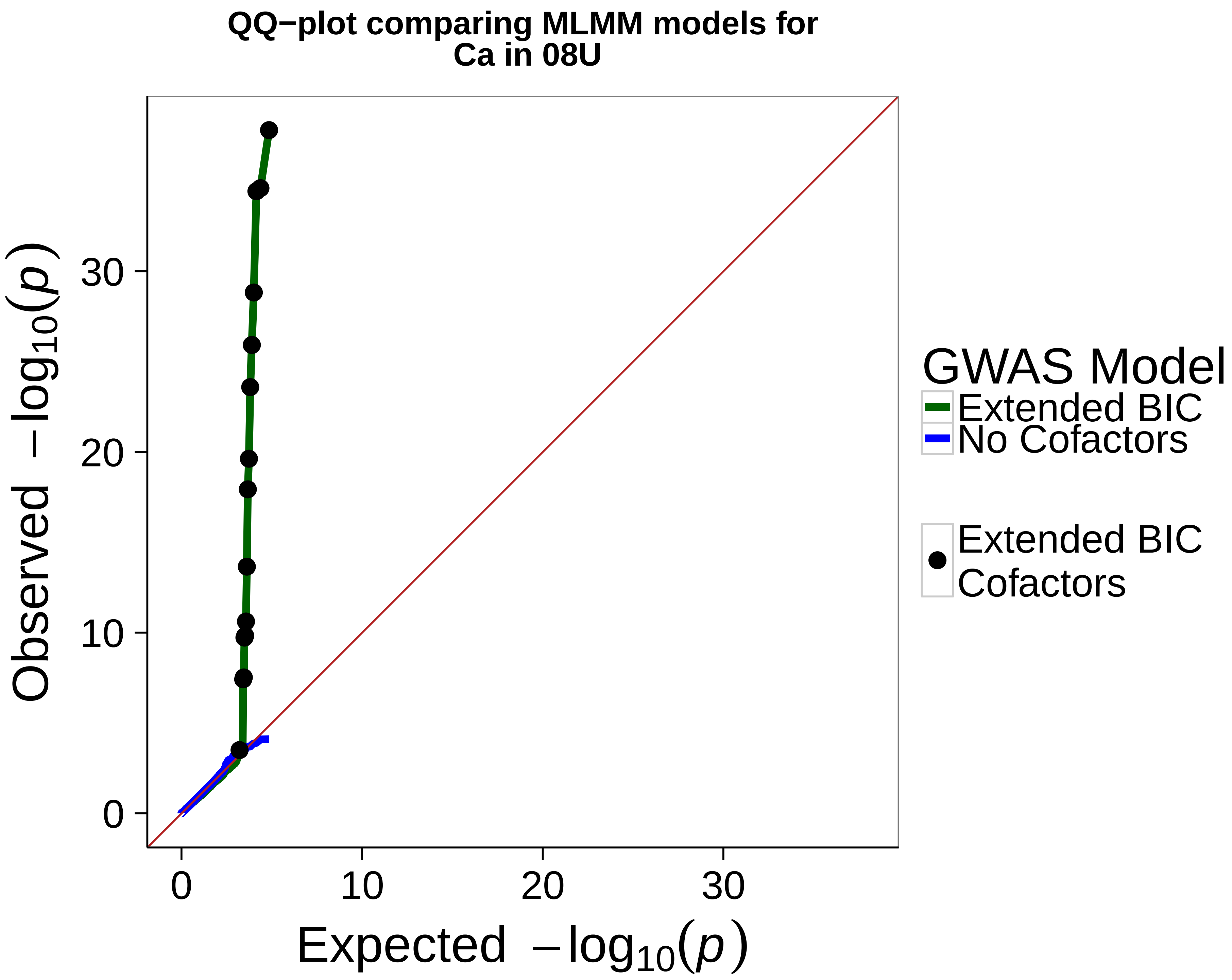
QQ−plot comparing MLMM models for
Ca in 08U
Observed −log10(p)
Expected −log10(p)
GWAS Model
Extended BIC
No Cofactors
Extended BIC
Cofactors

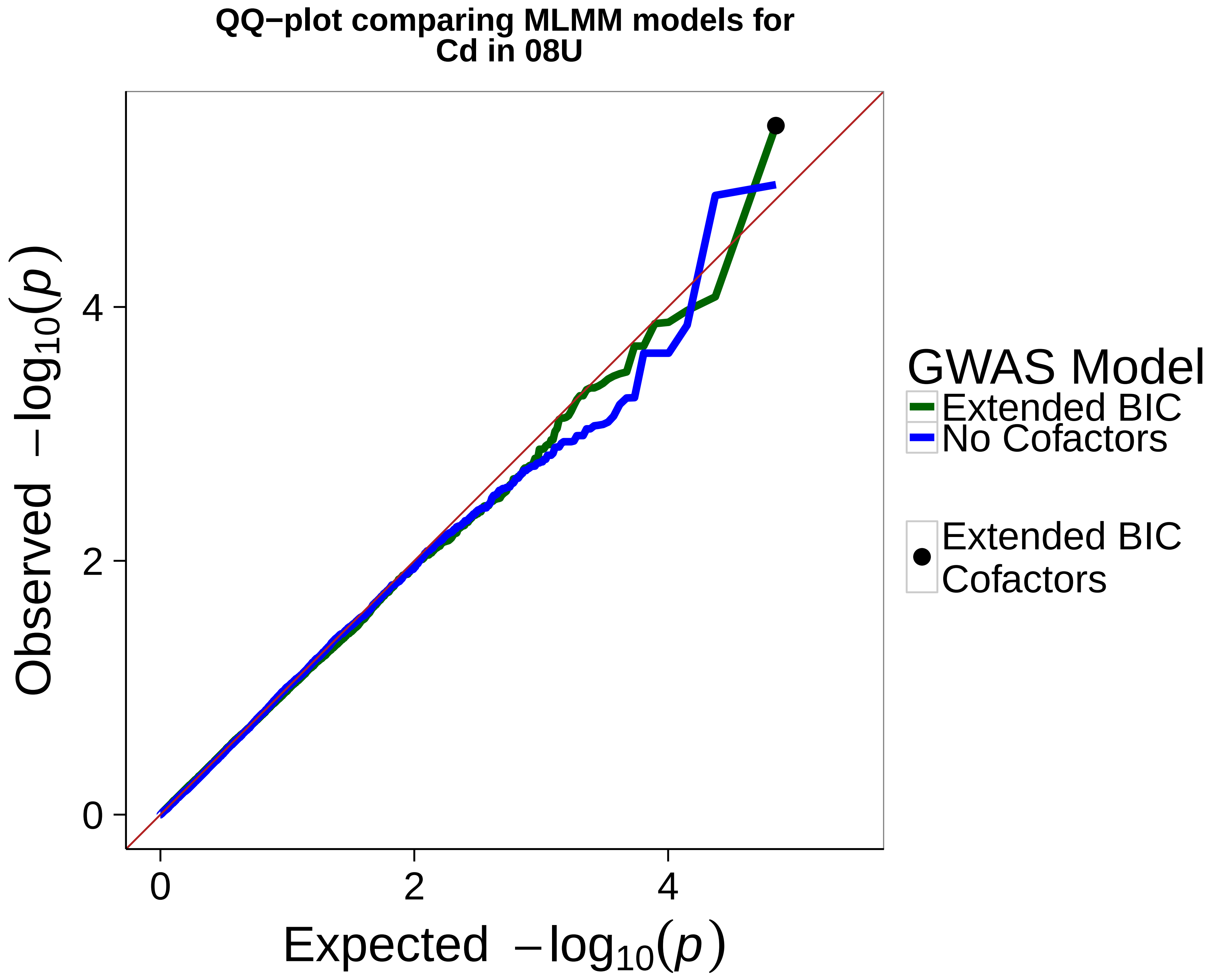
QQ−plot comparing MLMM models for
Cd in 08U
Observed −log10(p)
Expected −log10(p)
GWAS Model
Extended BIC
No Cofactors
Extended BIC
Cofactors

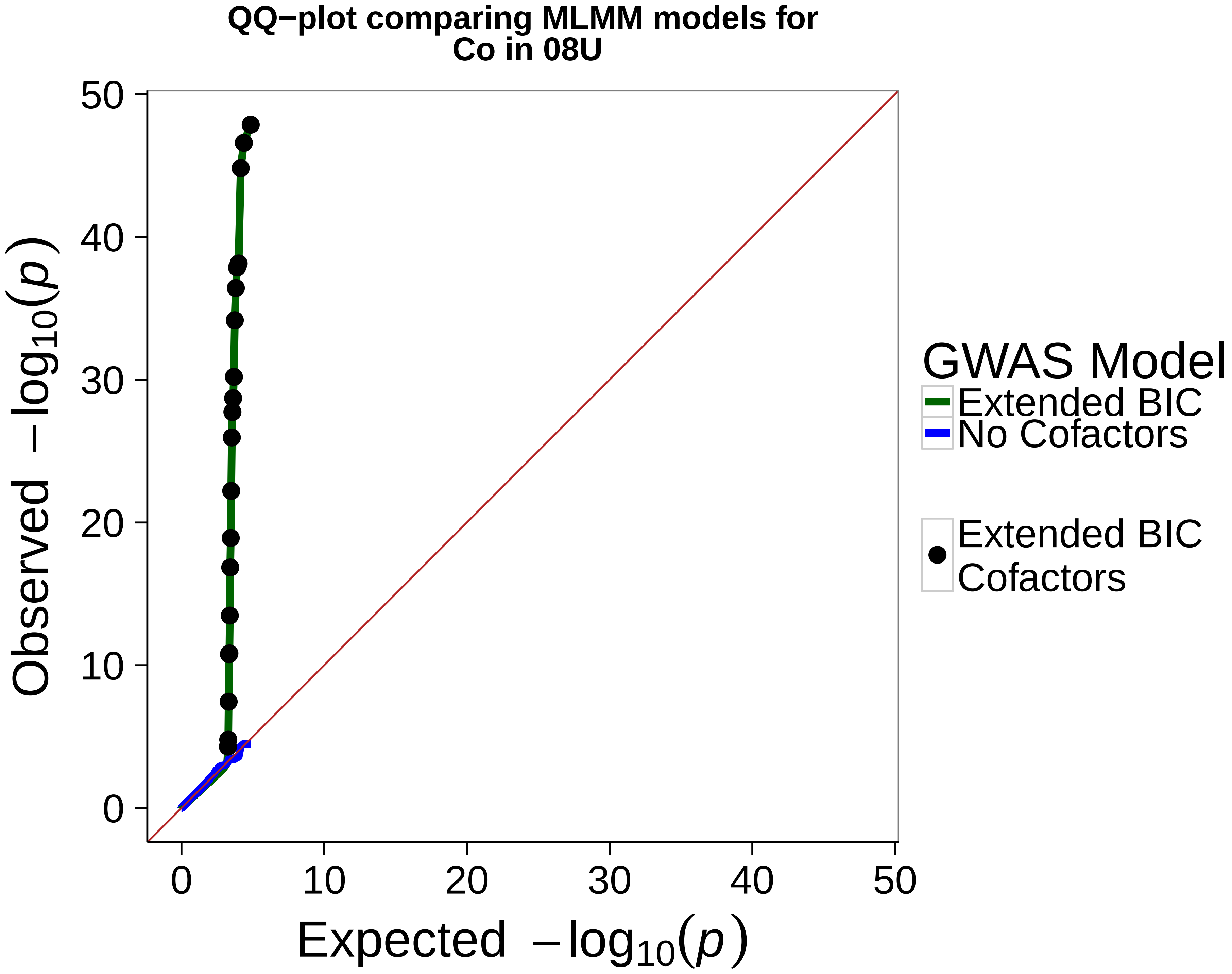
QQ−plot comparing MLMM models for
Co in 08U
Observed −log10(p)
Expected −log10(p)
GWAS Model
Extended BIC
No Cofactors
Extended BIC
Cofactors

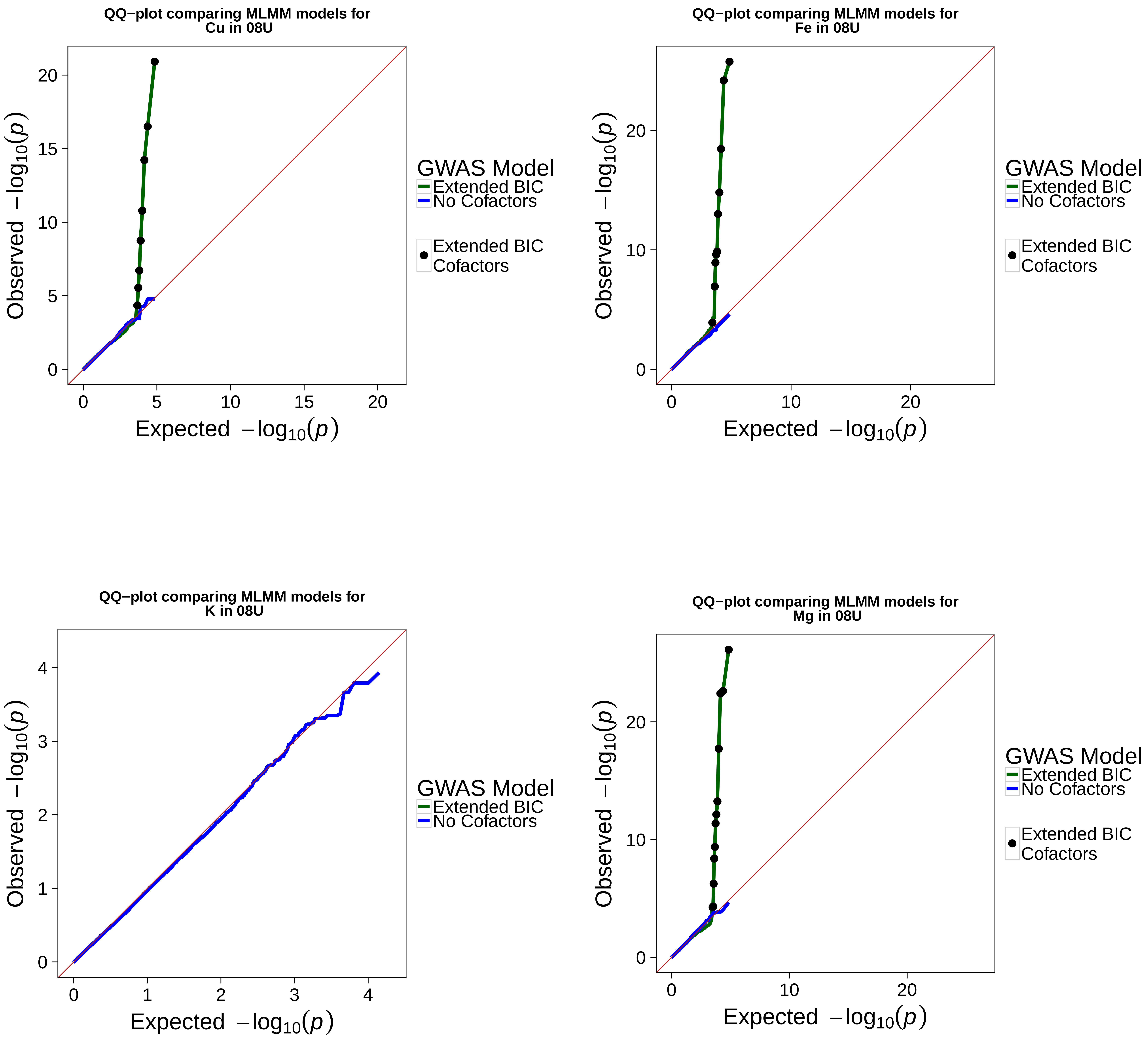

QQ−plot comparing MLMM models for
Cu in 08U
Observed −log10(p)
Expected −log10(p)
GWAS Model
Extended BIC
No Cofactors
Extended BIC
Cofactors
QQ−plot comparing MLMM models for
Fe in 08U
Observed −log10(p)
Expected −log10(p)
GWAS Model
Extended BIC
No Cofactors
Extended BIC
Cofactors
QQ−plot comparing MLMM models for
K in 08U
Observed −log10(p)
Expected −log10(p)
GWAS Model
Extended BIC
No Cofactors
QQ−plot comparing MLMM models for
Mg in 08U
Observed −log10(p)
Expected −log10(p)
GWAS Model
Extended BIC
No Cofactors
Extended BIC
Cofactors

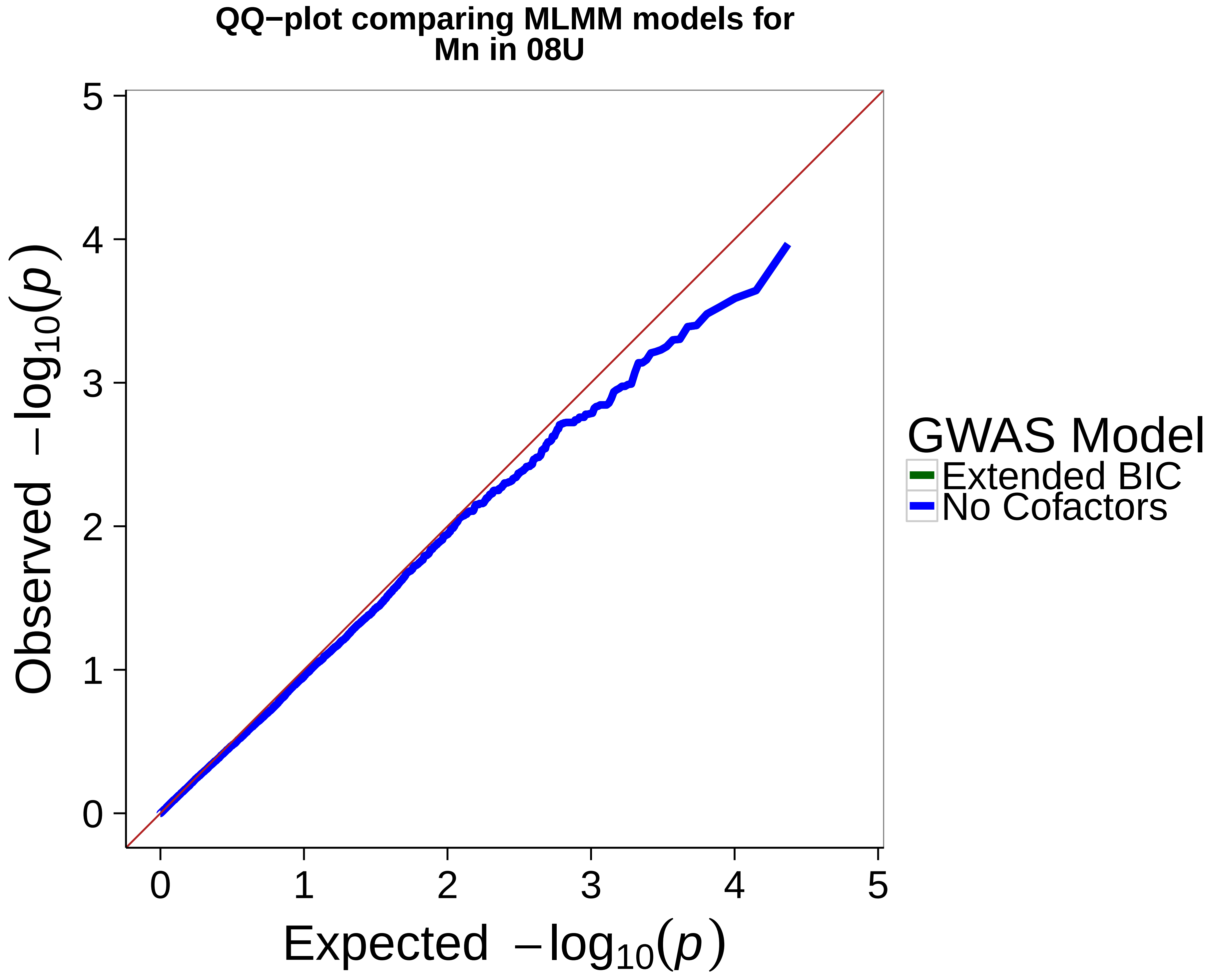
QQ−plot comparing MLMM models for
Mn in 08U
Observed −log10(p)
Expected −log10(p)
GWAS Model
Extended BIC
No Cofactors

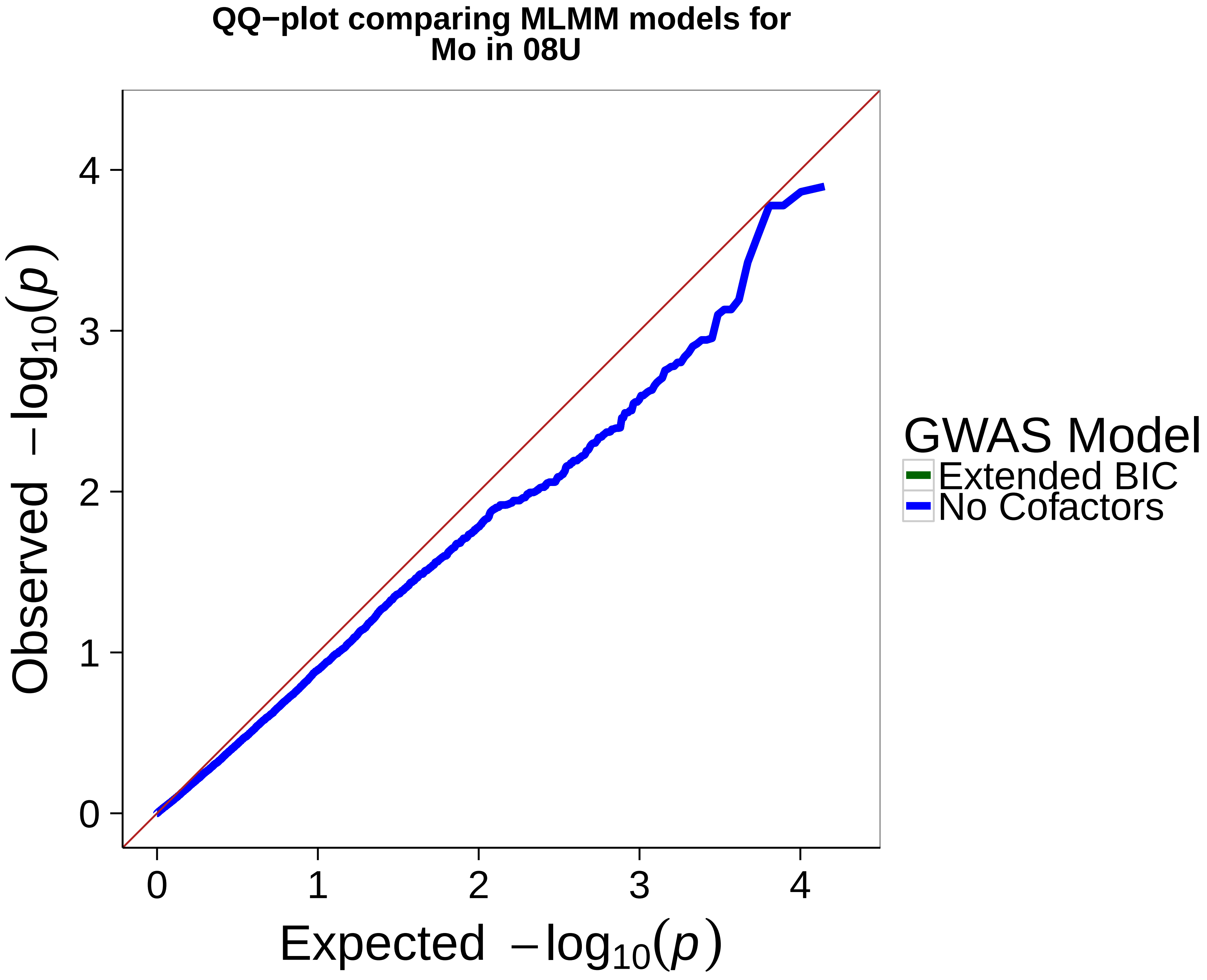
QQ−plot comparing MLMM models for
Mo in 08U
Observed −log10(p)
Expected −log10(p)
GWAS Model
Extended BIC
No Cofactors

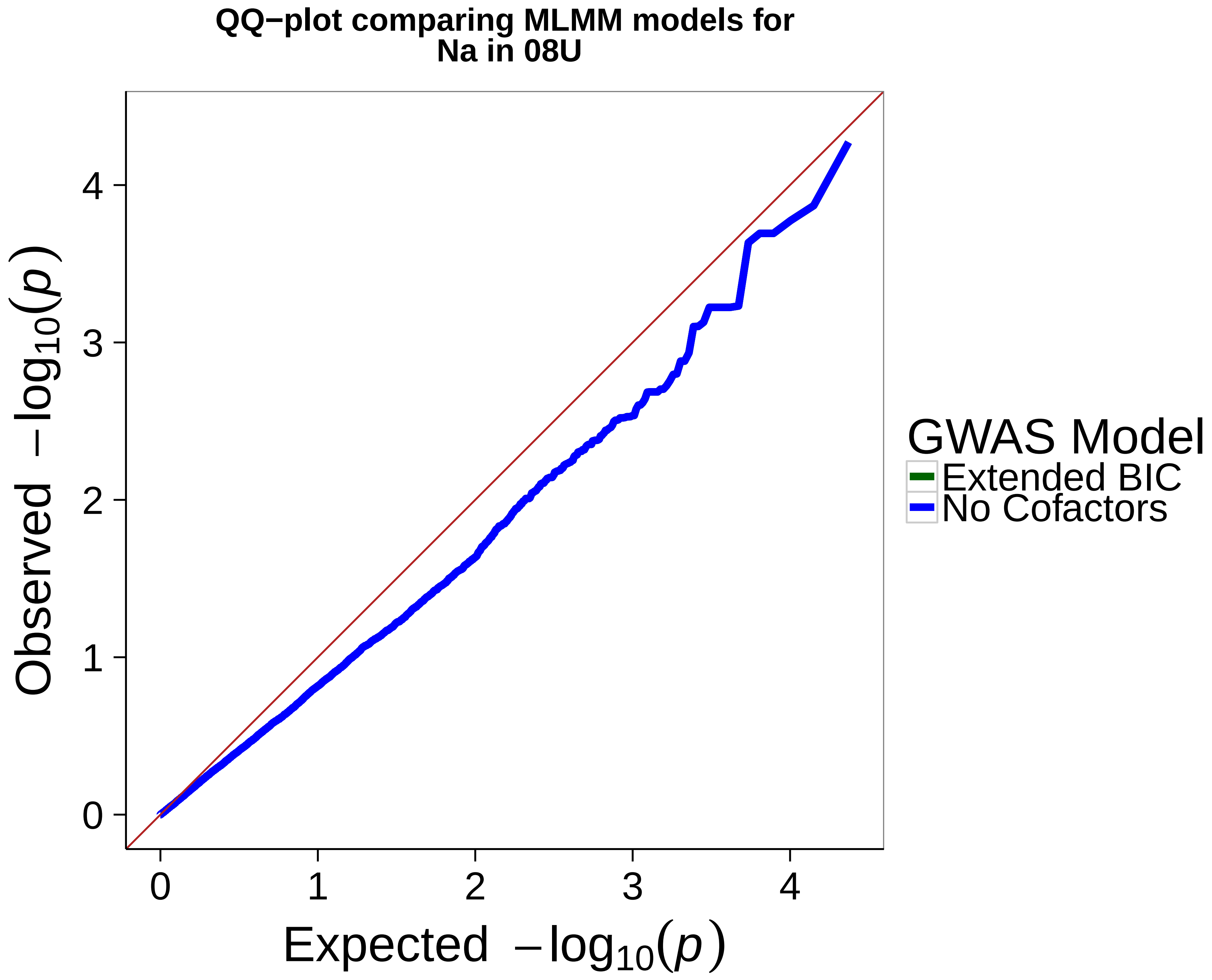
QQ−plot comparing MLMM models for
Na in 08U
Observed −log10(p)
Expected −log10(p)
GWAS Model
Extended BIC
No Cofactors

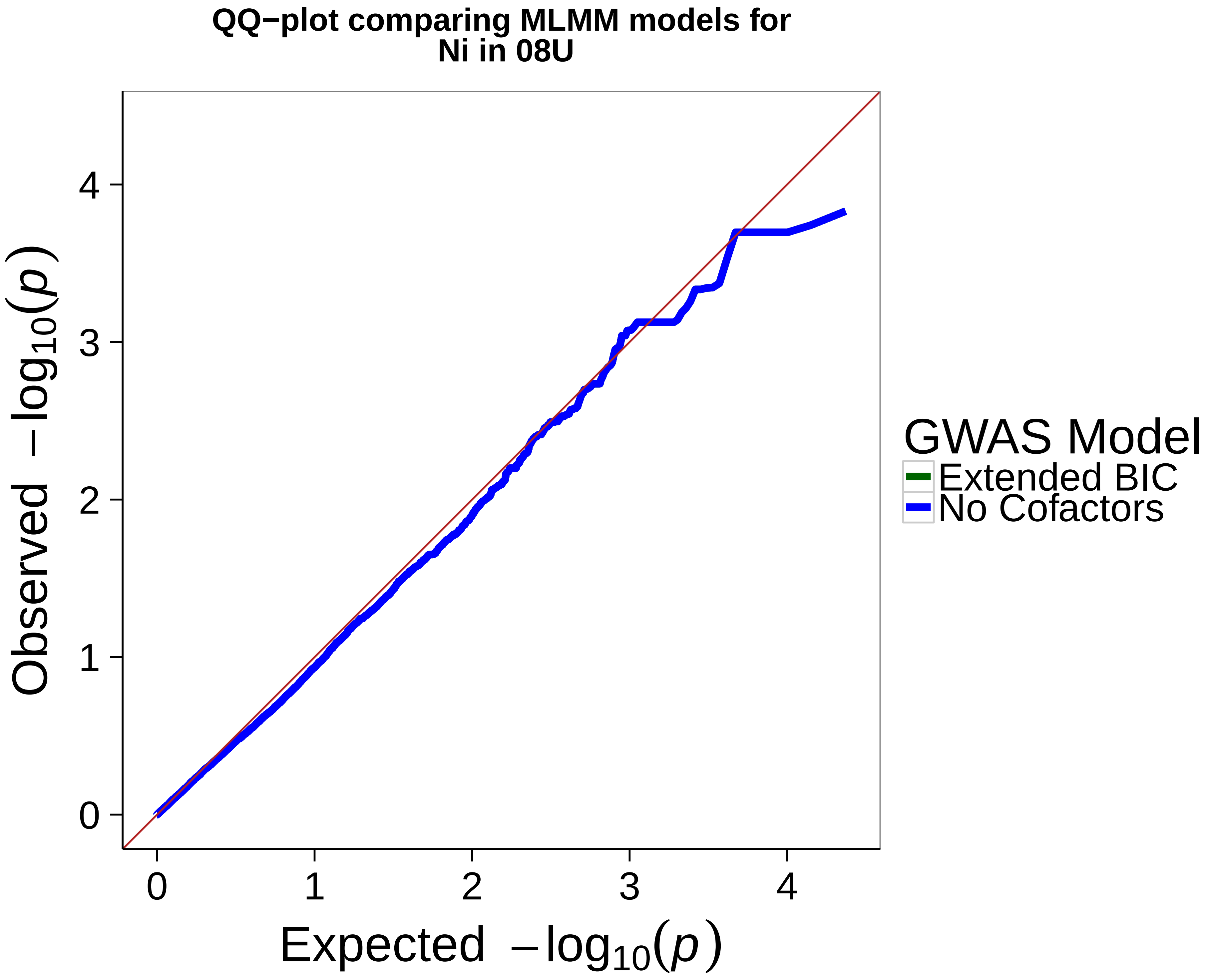
QQ−plot comparing MLMM models for
Ni in 08U
Observed −log10(p)
Expected −log10(p)
GWAS Model
Extended BIC
No Cofactors

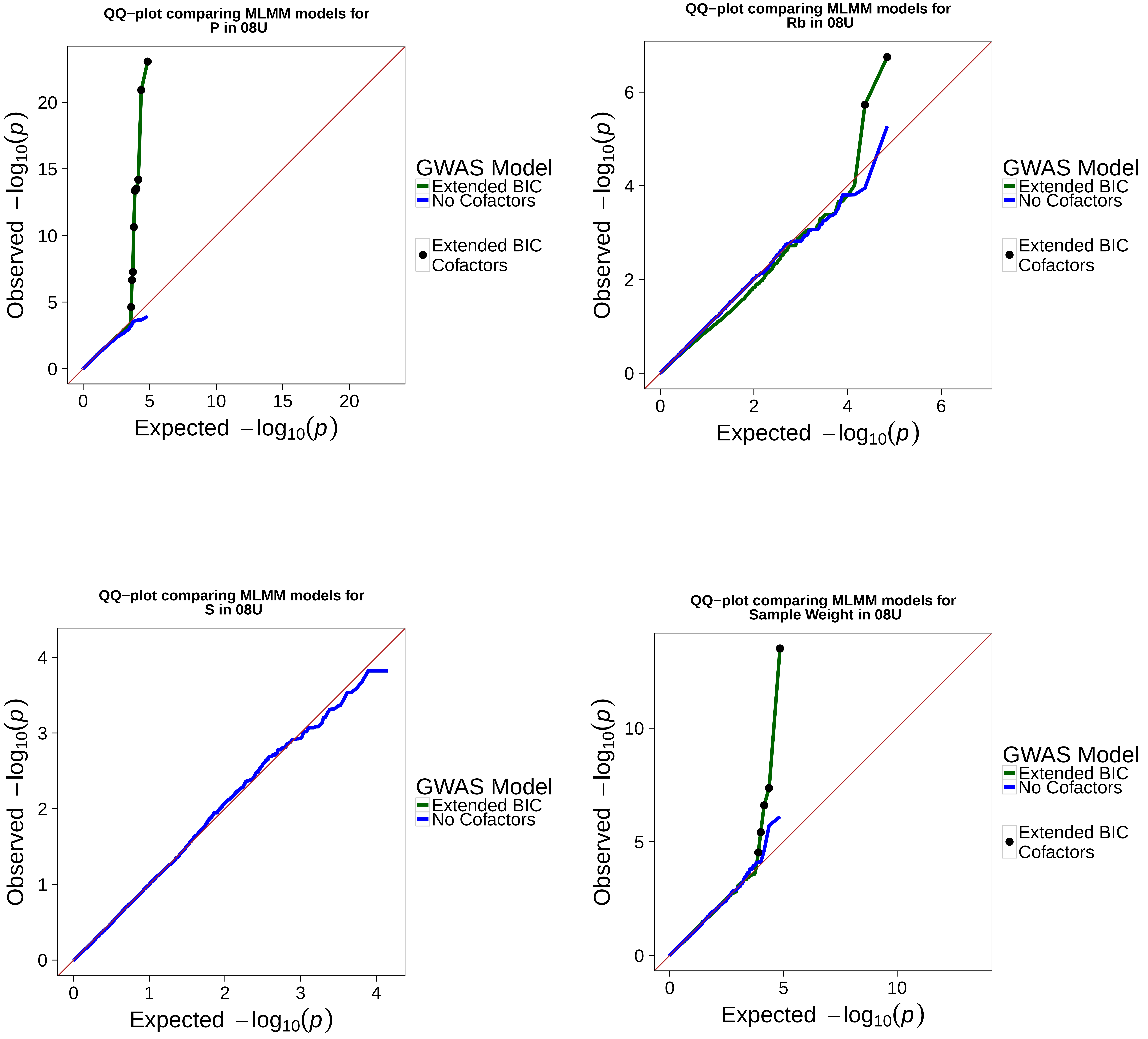
QQ−plot comparing MLMM models for P in 08U
QQ−plot comparing MLMM models for Rb in 08U
QQ−plot comparing MLMM models for S in 08U
QQ−plot comparing MLMM models for Sample Weight in 08U
Observed −log10(p)
Expected −log10(p)
GWAS Model
Extended BIC
No Cofactors
Extended BIC Cofactors

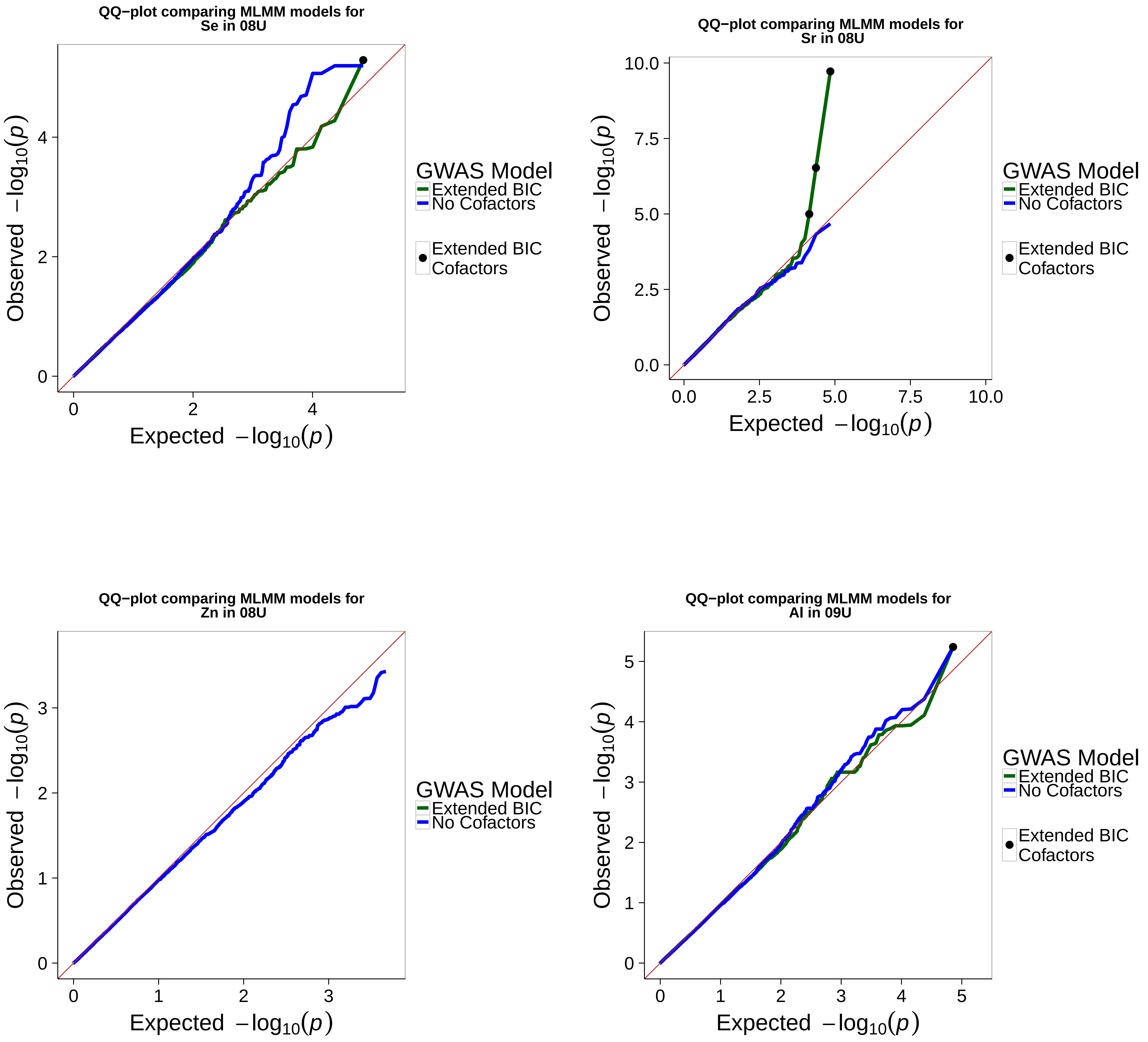
QQ−plot comparing MLMM models for
Se in 08U
Observed −log10(p)
Expected −log10(p)
GWAS Model
Extended BIC
No Cofactors
Extended BIC
Cofactors
QQ−plot comparing MLMM models for
Sr in 08U
Observed −log10(p)
Expected −log10(p)
GWAS Model
Extended BIC
No Cofactors
Extended BIC
Cofactors
QQ−plot comparing MLMM models for
Zn in 08U
Observed −log10(p)
Expected −log10(p)
GWAS Model
Extended BIC
No Cofactors
QQ−plot comparing MLMM models for
Al in 09U
Observed −log10(p)
Expected −log10(p)
GWAS Model
Extended BIC
No Cofactors
Extended BIC
Cofactors

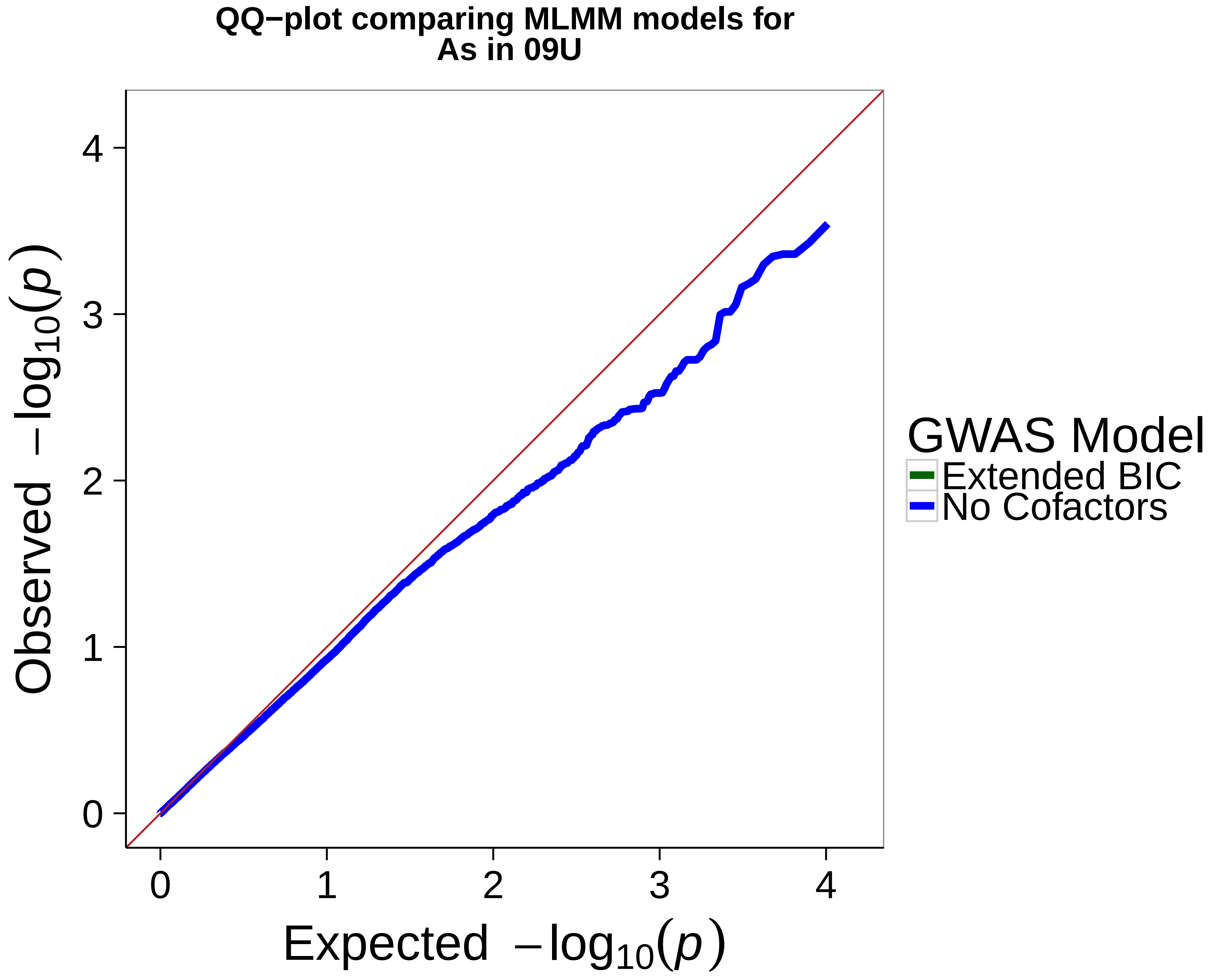
QQ−plot comparing MLMM models for
As in 09U
Observed −log10(p)
Expected −log10(p)
GWAS Model
Extended BIC
No Cofactors

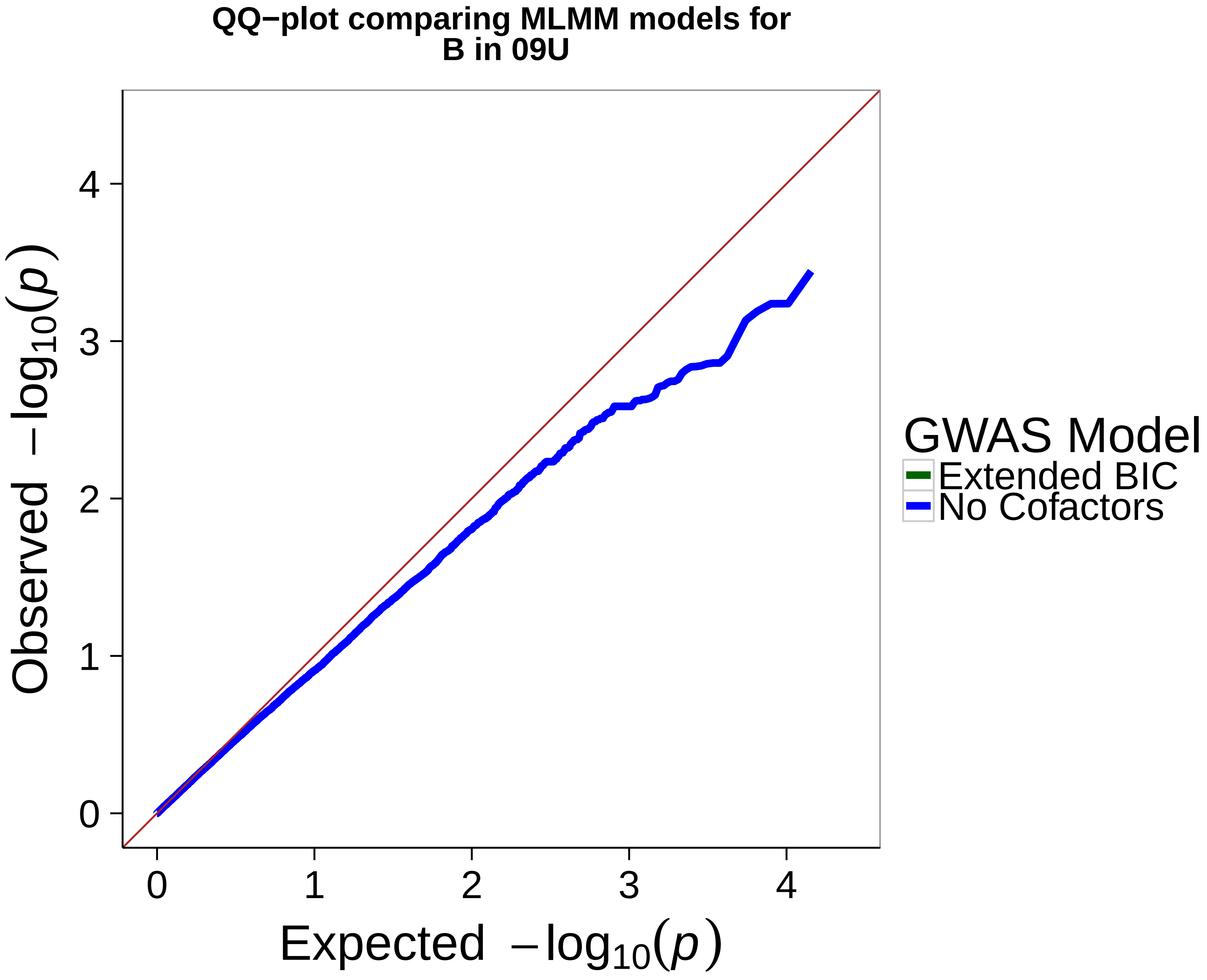
QQ−plot comparing MLMM models for
B in 09U
Observed −log10(p)
Expected −log10(p)
GWAS Model
Extended BIC
No Cofactors

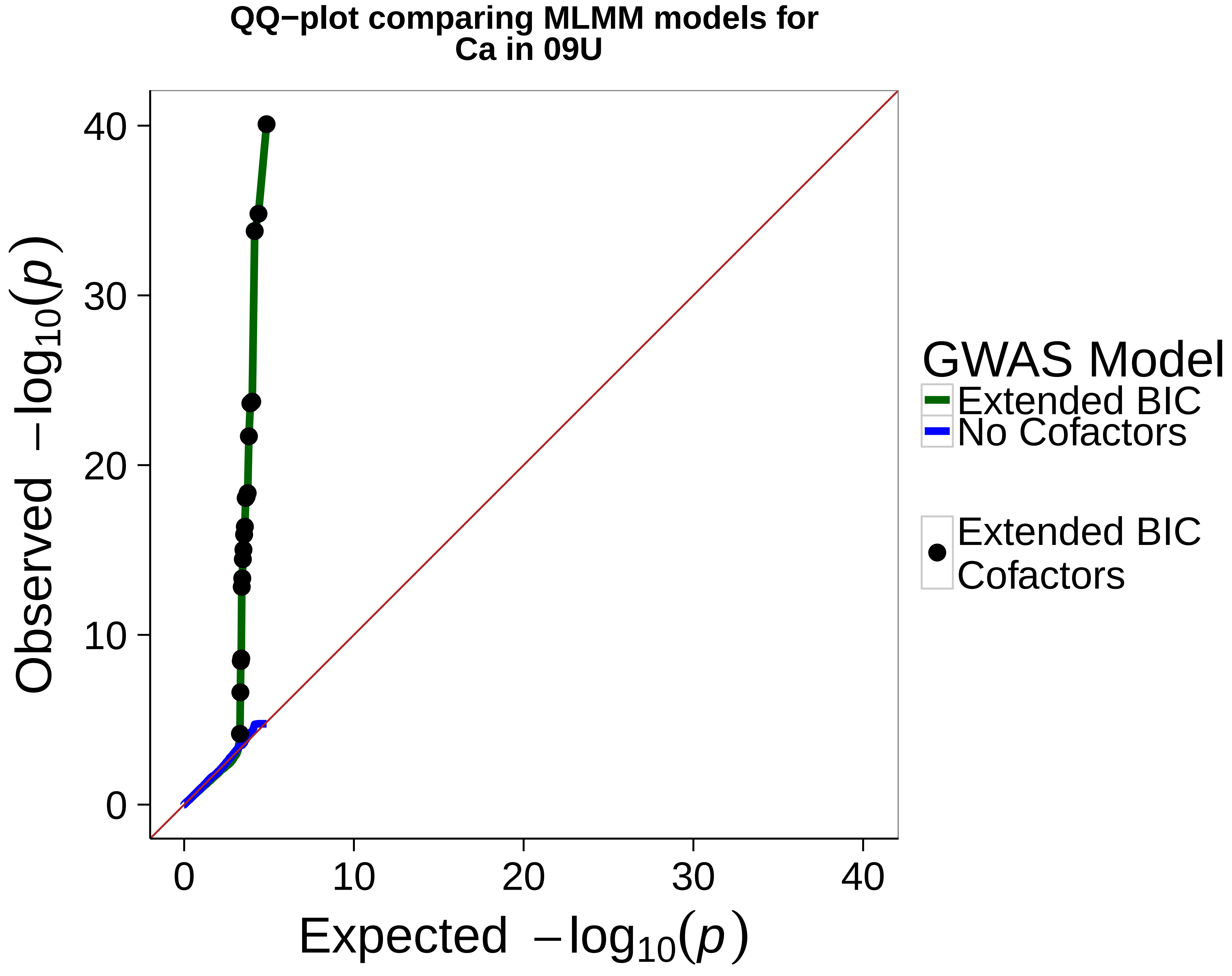
QQ−plot comparing MLMM models for
Ca in 09U
Observed −log10(p)
Expected −log10(p)
GWAS Model
Extended BIC
No Cofactors
Extended BIC
Cofactors

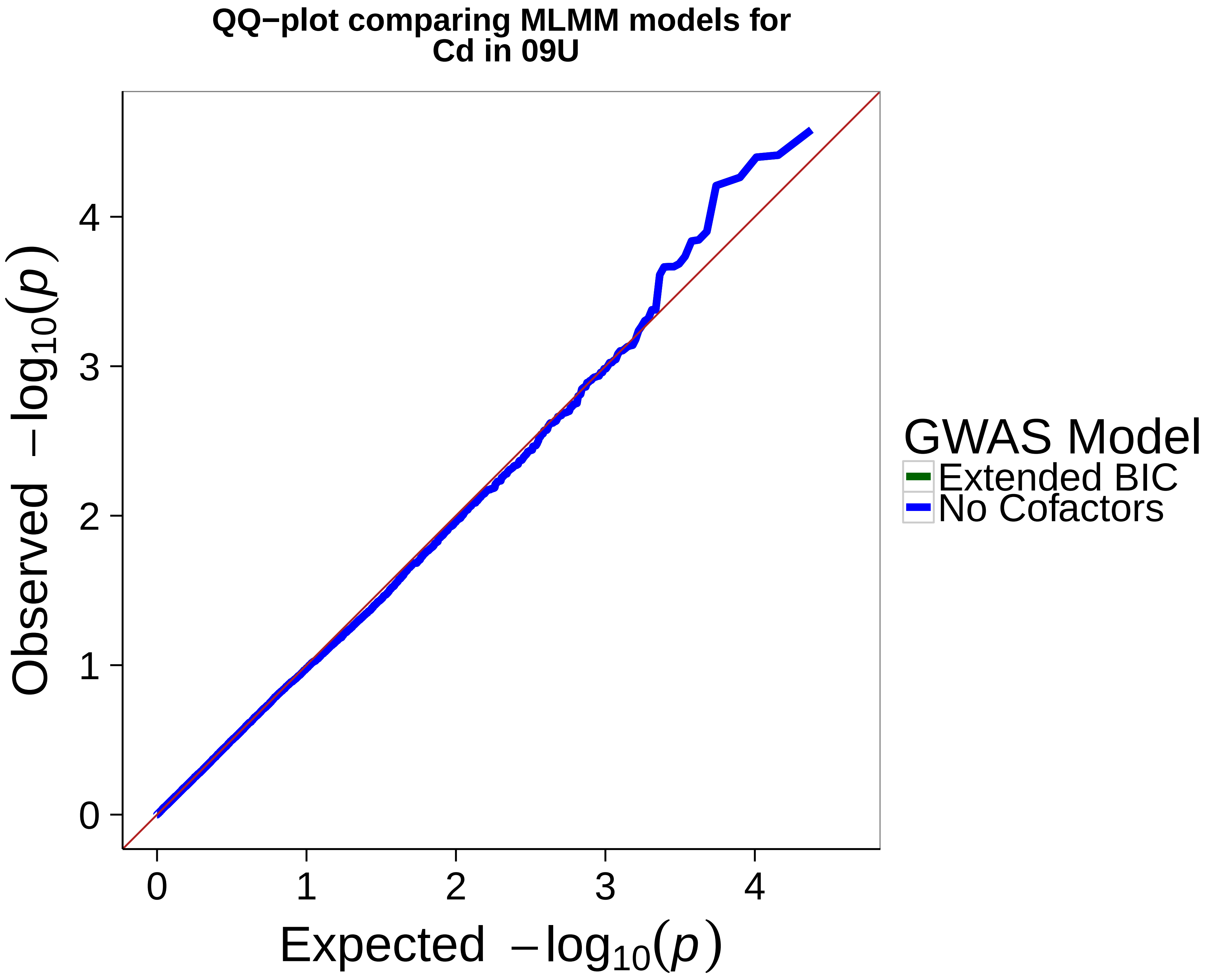
QQ−plot comparing MLMM models for
Cd in 09U
Observed −log10(p)
Expected −log10(p)
GWAS Model
Extended BIC
No Cofactors

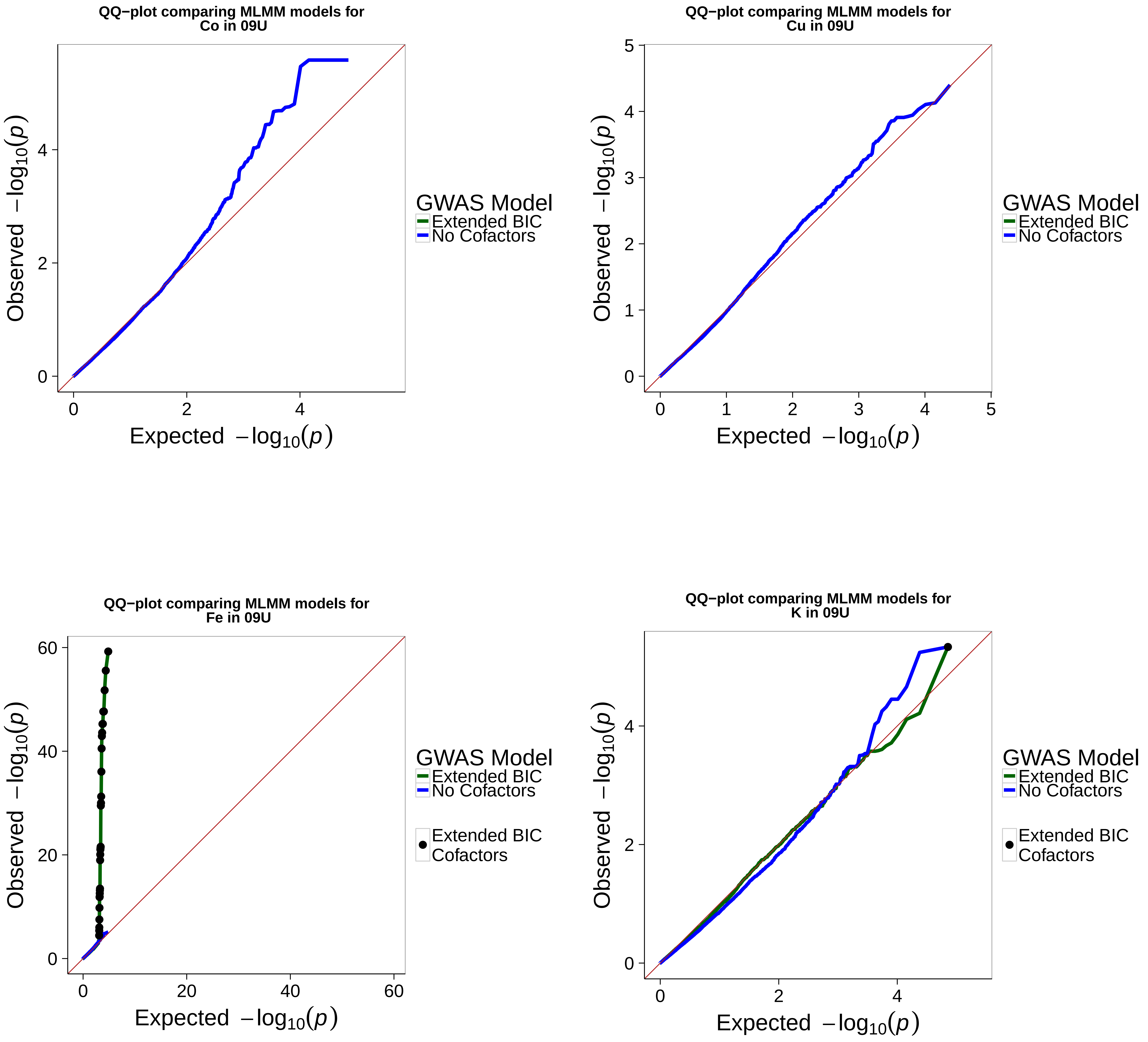

QQ−plot comparing MLMM models for
Co in 09U
Observed −log10(p)
Expected −log10(p)
0
2
4
GWAS Model
Extended BIC
No Cofactors
QQ−plot comparing MLMM models for
Cu in 09U
0
1
2
3
4
5
QQ−plot comparing MLMM models for
Fe in 09U
0
20
40
60
Extended BIC
Cofactors
QQ−plot comparing MLMM models for
K in 09U

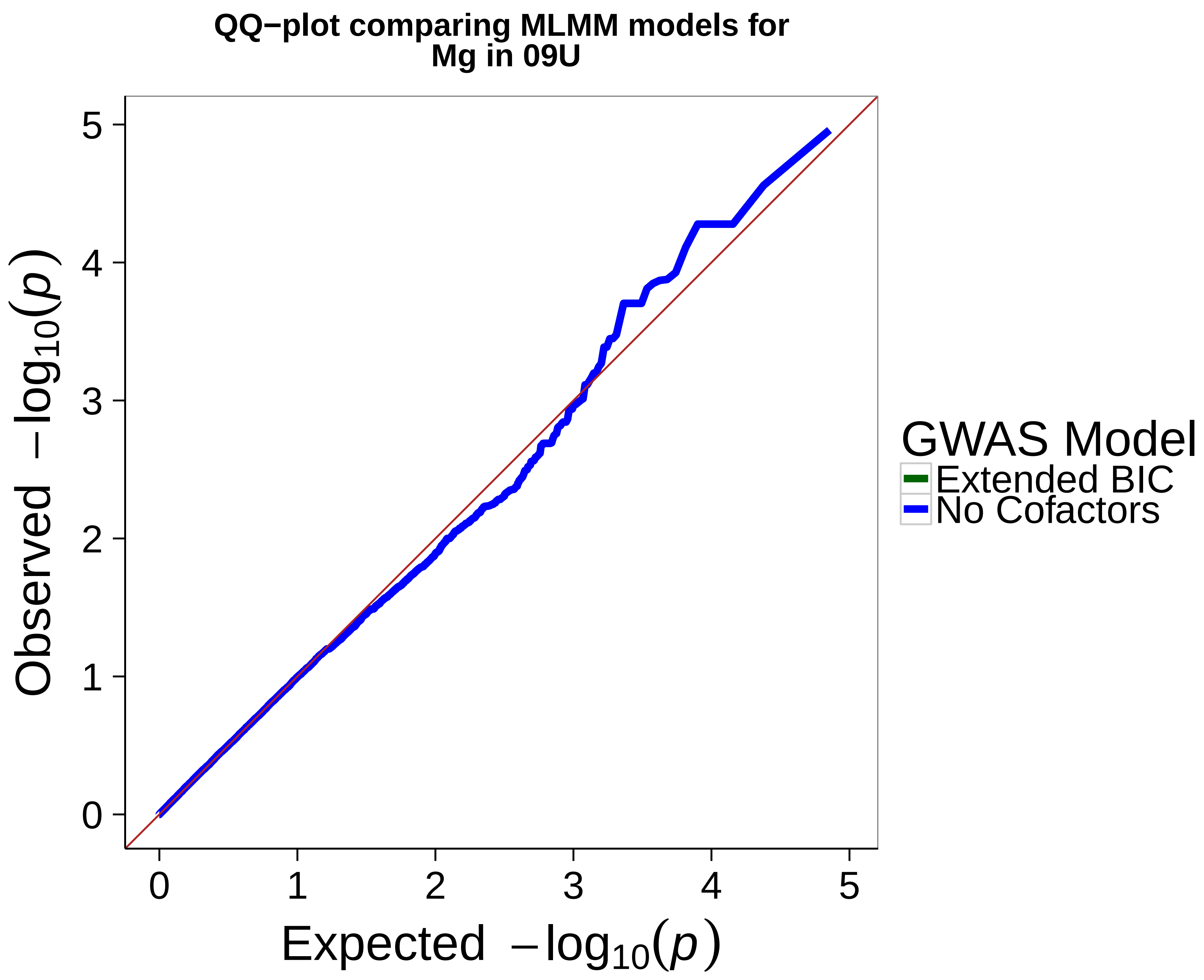
QQ−plot comparing MLMM models for
Mg in 09U
Observed −log10(p)
Expected −log10(p)
GWAS Model
Extended BIC
No Cofactors

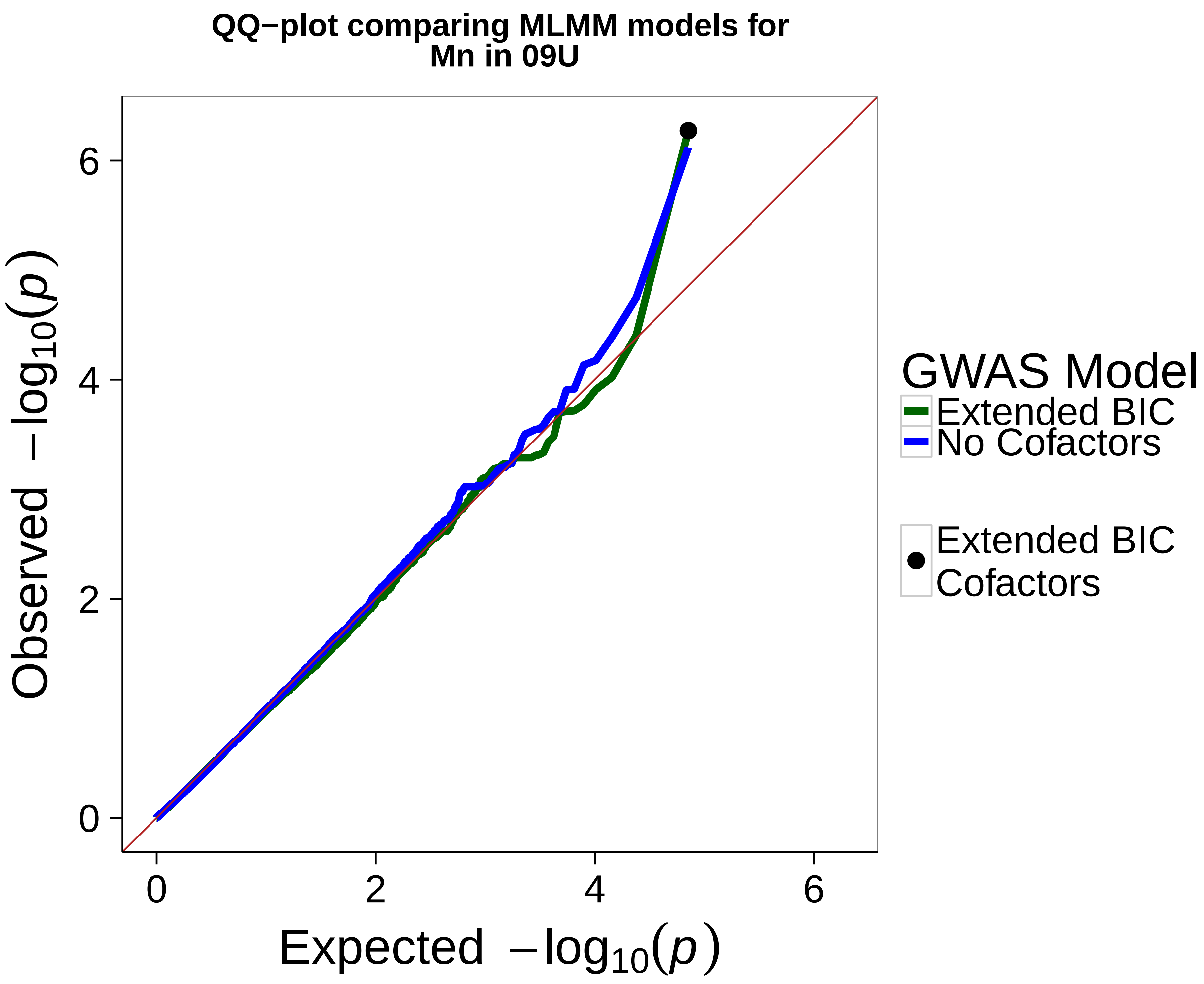
QQ−plot comparing MLMM models for
Mn in 09U
Observed −log10(p)
Expected −log10(p)
GWAS Model
Extended BIC
No Cofactors
Extended BIC
Cofactors

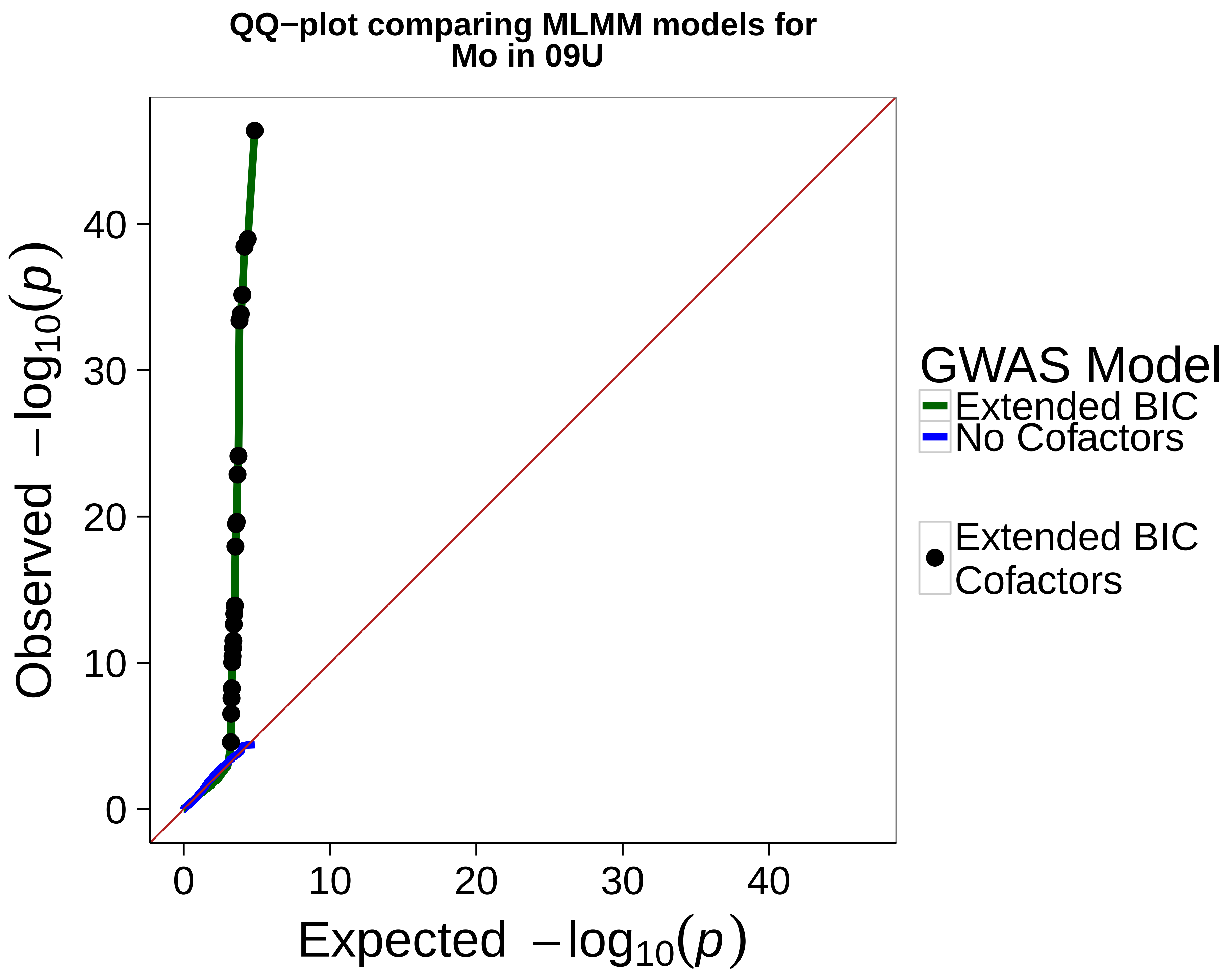
QQ−plot comparing MLMM models for
Mo in 09U
Observed −log10(p)
Expected −log10(p)
GWAS Model
Extended BIC
No Cofactors
Extended BIC
Cofactors

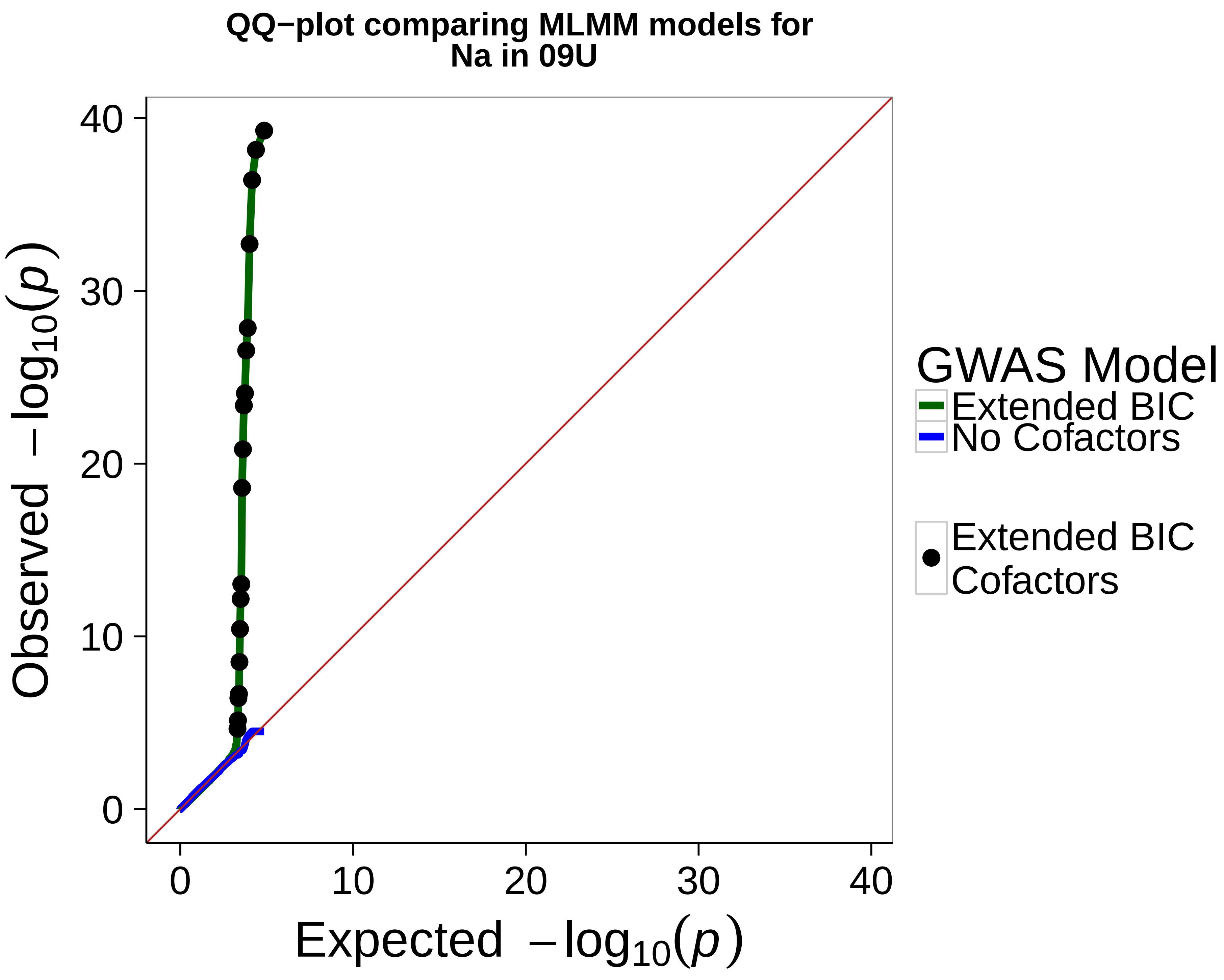
QQ−plot comparing MLMM models for
Na in 09U
Observed −log10(p)
Expected −log10(p)
GWAS Model
Extended BIC
No Cofactors
Extended BIC
Cofactors

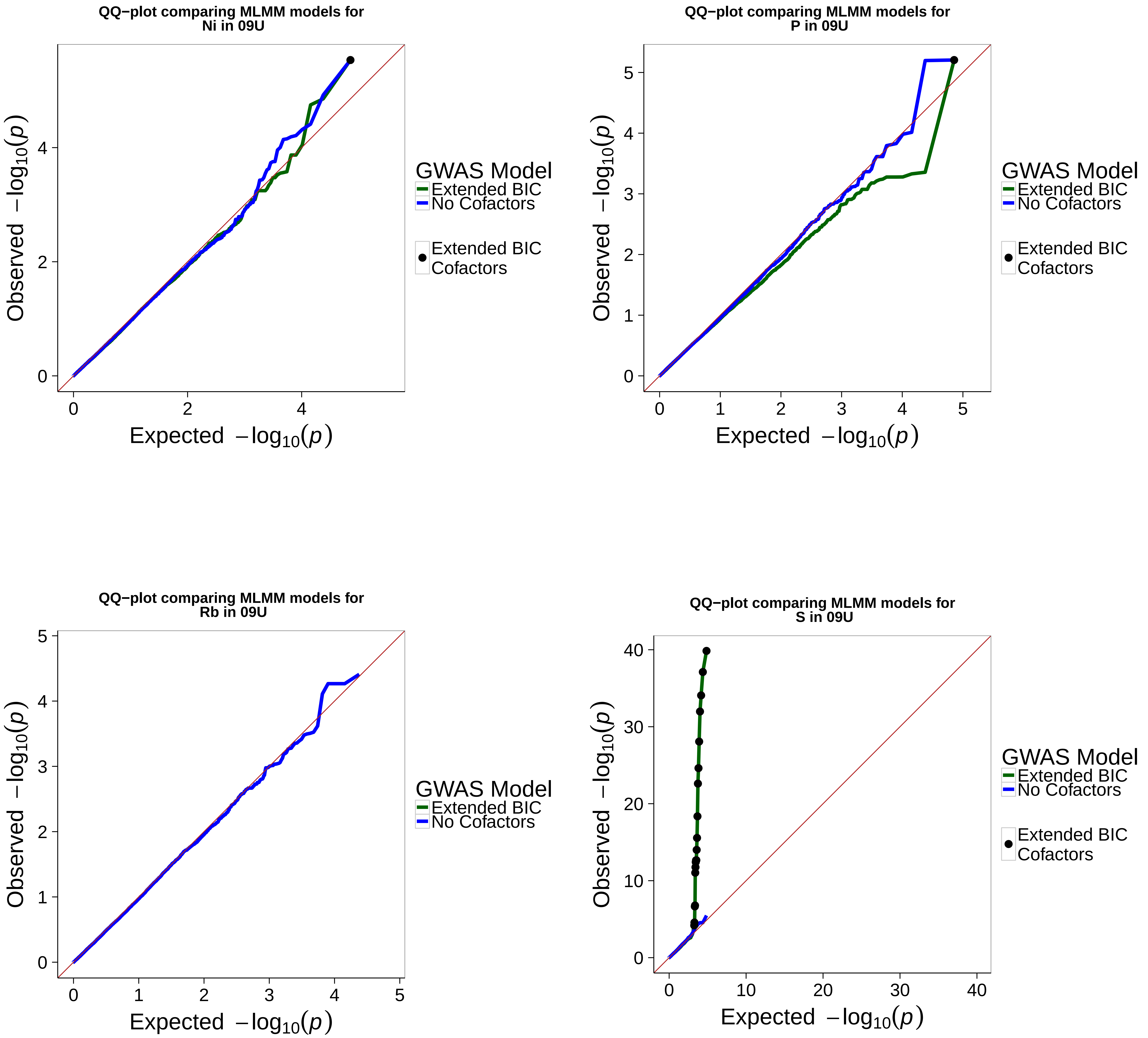
QQ−plot comparing MLMM models for
Ni in 09U
Observed −log10(p)
Expected −log10(p)
GWAS Model
Extended BIC
No Cofactors
Extended BIC
Cofactors
QQ−plot comparing MLMM models for
P in 09U
Observed −log10(p)
Expected −log10(p)
GWAS Model
Extended BIC
No Cofactors
Extended BIC
Cofactors
QQ−plot comparing MLMM models for
Rb in 09U
Observed −log10(p)
Expected −log10(p)
GWAS Model
Extended BIC
No Cofactors
QQ−plot comparing MLMM models for
S in 09U
Observed −log10(p)
Expected −log10(p)
GWAS Model
Extended BIC
No Cofactors
Extended BIC
Cofactors

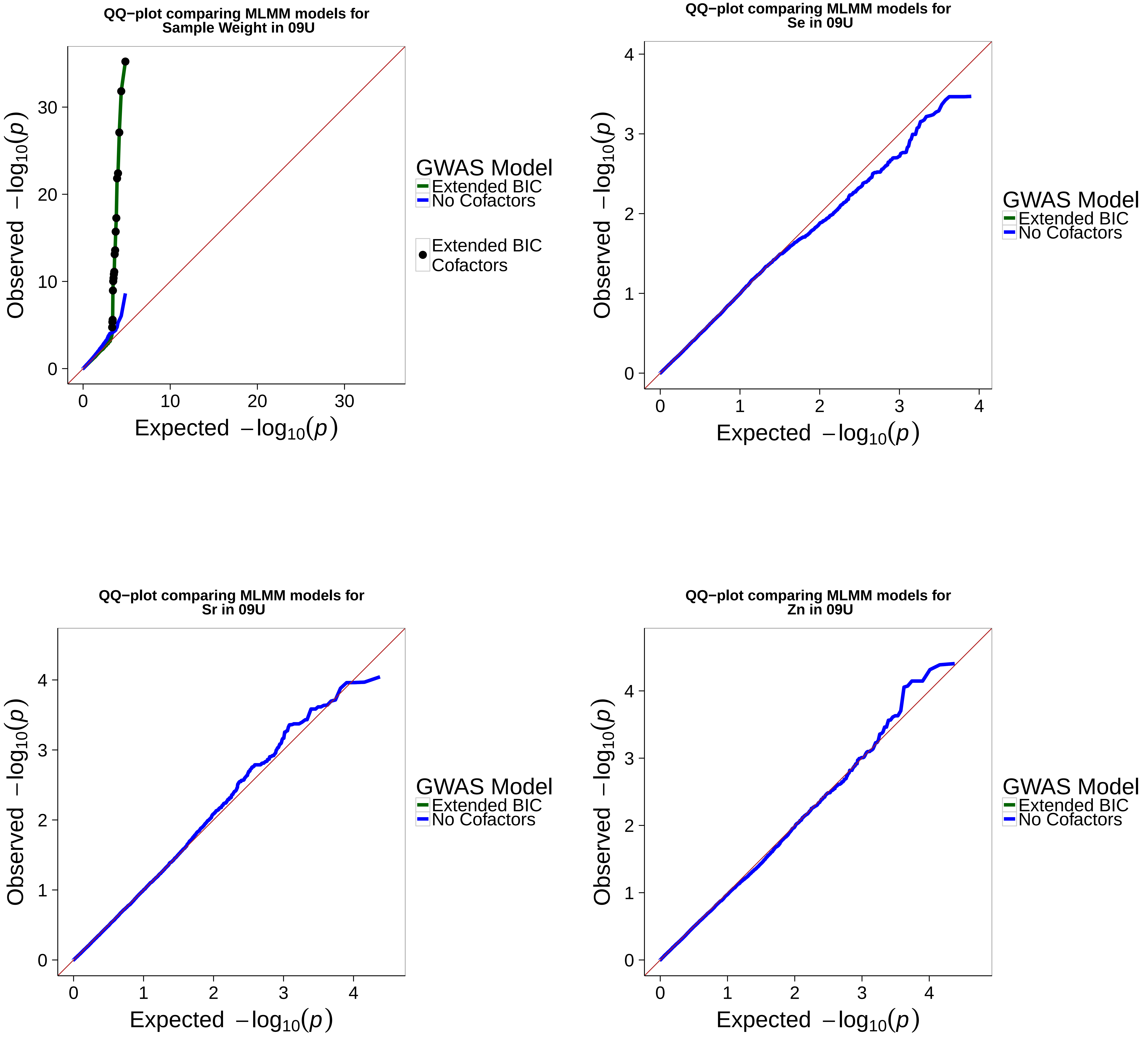
QQ−plot comparing MLMM models for
Sample Weight in 09U
Observed −log10(p)
Expected −log10(p)
GWAS Model
Extended BIC
No Cofactors
Extended BIC
Cofactors
QQ−plot comparing MLMM models for
Se in 09U
Observed −log10(p)
Expected −log10(p)
GWAS Model
Extended BIC
No Cofactors
QQ−plot comparing MLMM models for
Sr in 09U
Observed −log10(p)
Expected −log10(p)
GWAS Model
Extended BIC
No Cofactors
QQ−plot comparing MLMM models for
Zn in 09U
Observed −log10(p)
Expected −log10(p)
GWAS Model
Extended BIC
No Cofactors

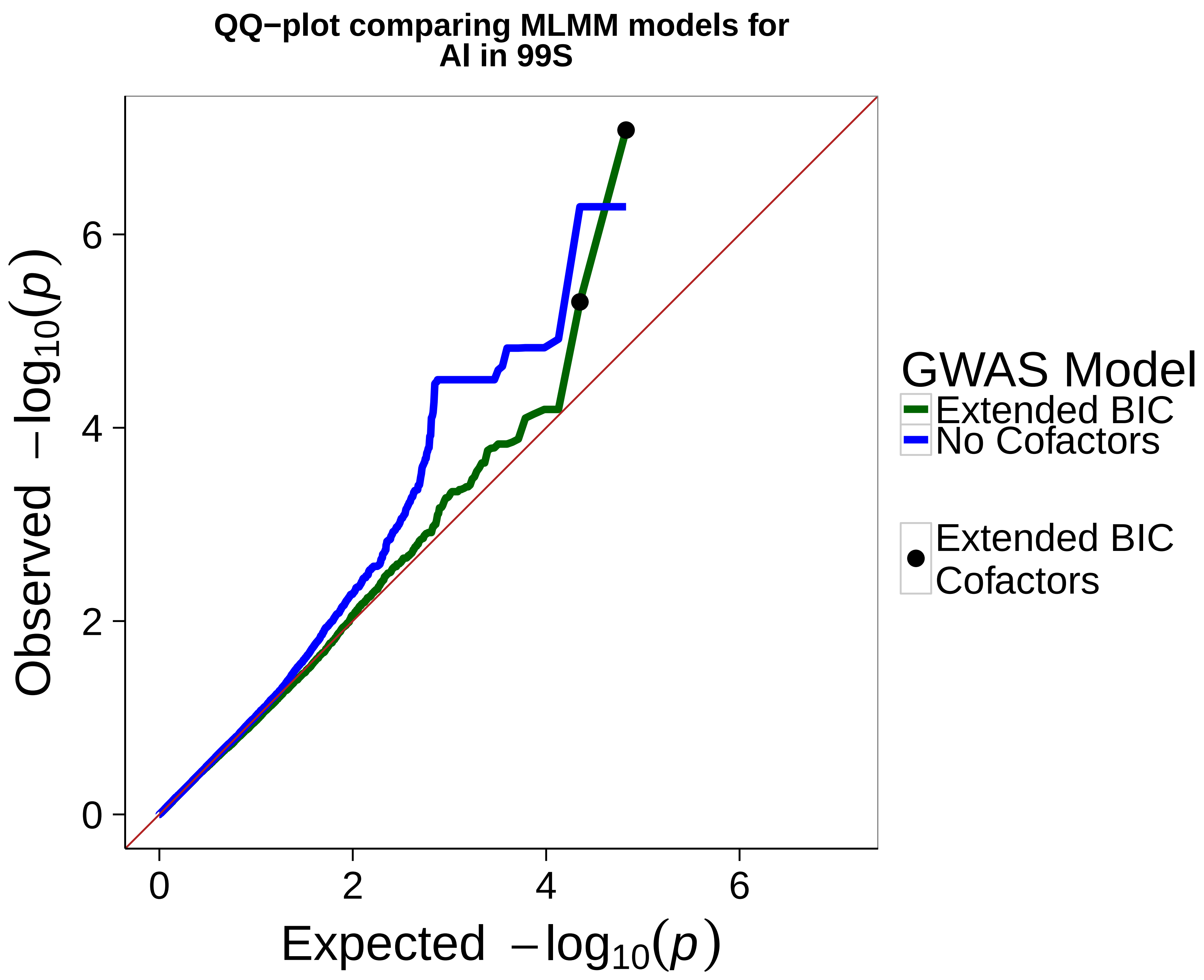
QQ−plot comparing MLMM models for
Al in 99S
Observed −log10(p)
Expected −log10(p)
0
2
4
6
GWAS Model
Extended BIC
No Cofactors
Extended BIC
Cofactors

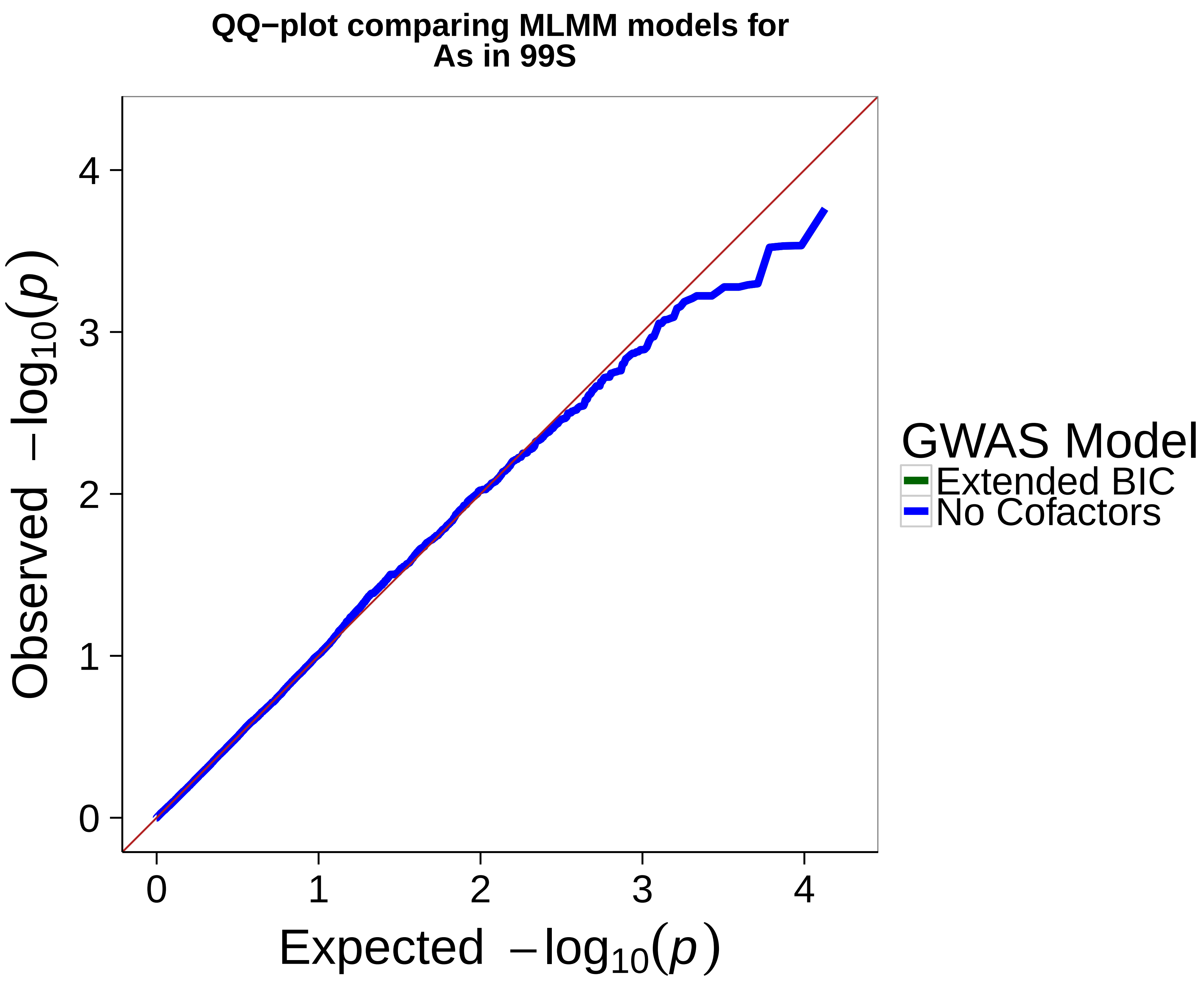
QQ−plot comparing MLMM models for
As in 99S
Observed −log10(p)
Expected −log10(p)
0
1
2
3
4
GWAS Model
Extended BIC
No Cofactors

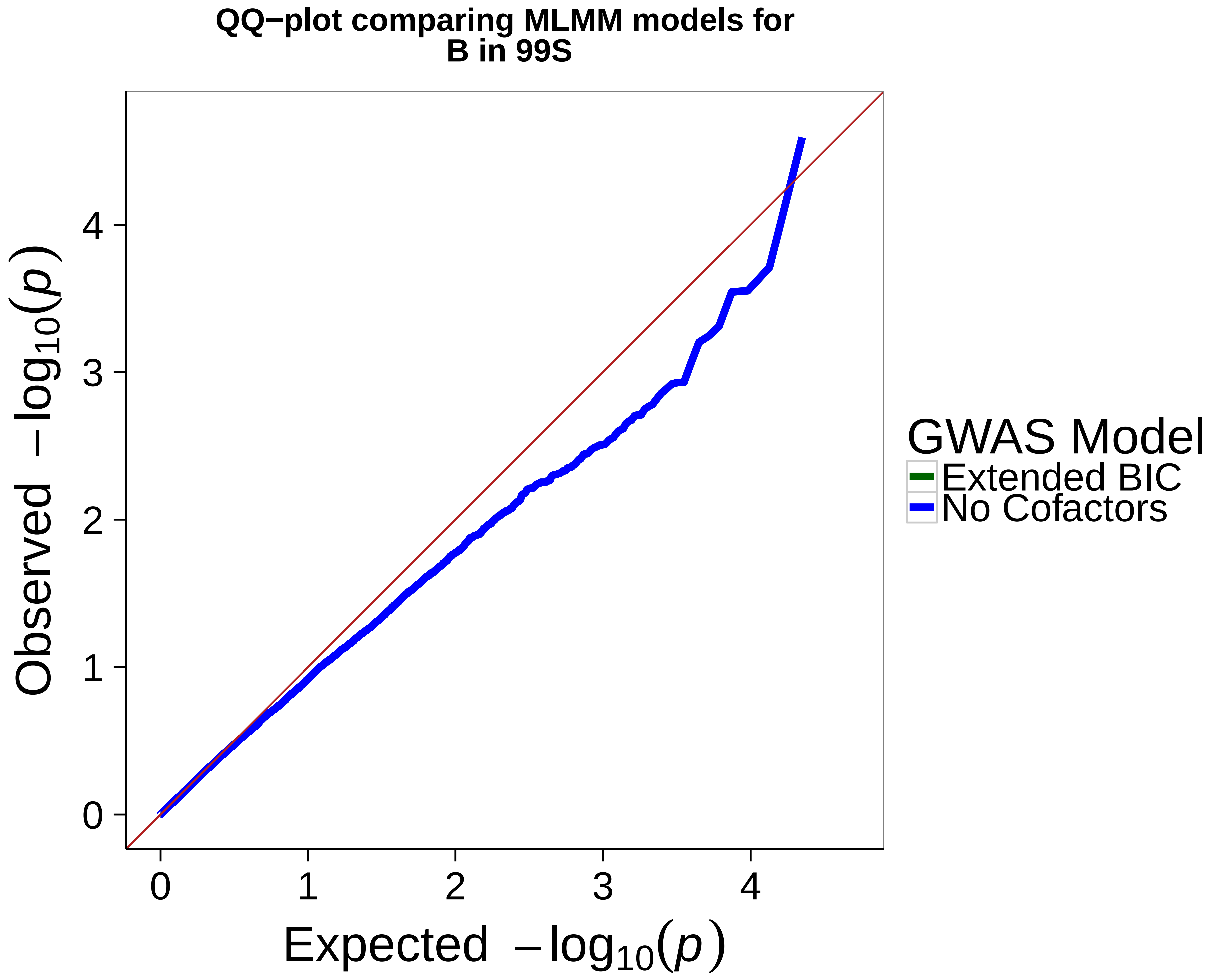
QQ−plot comparing MLMM models for
B in 99S
Observed −log10(p)
Expected −log10(p)
0
1
2
3
4
GWAS Model
Extended BIC
No Cofactors

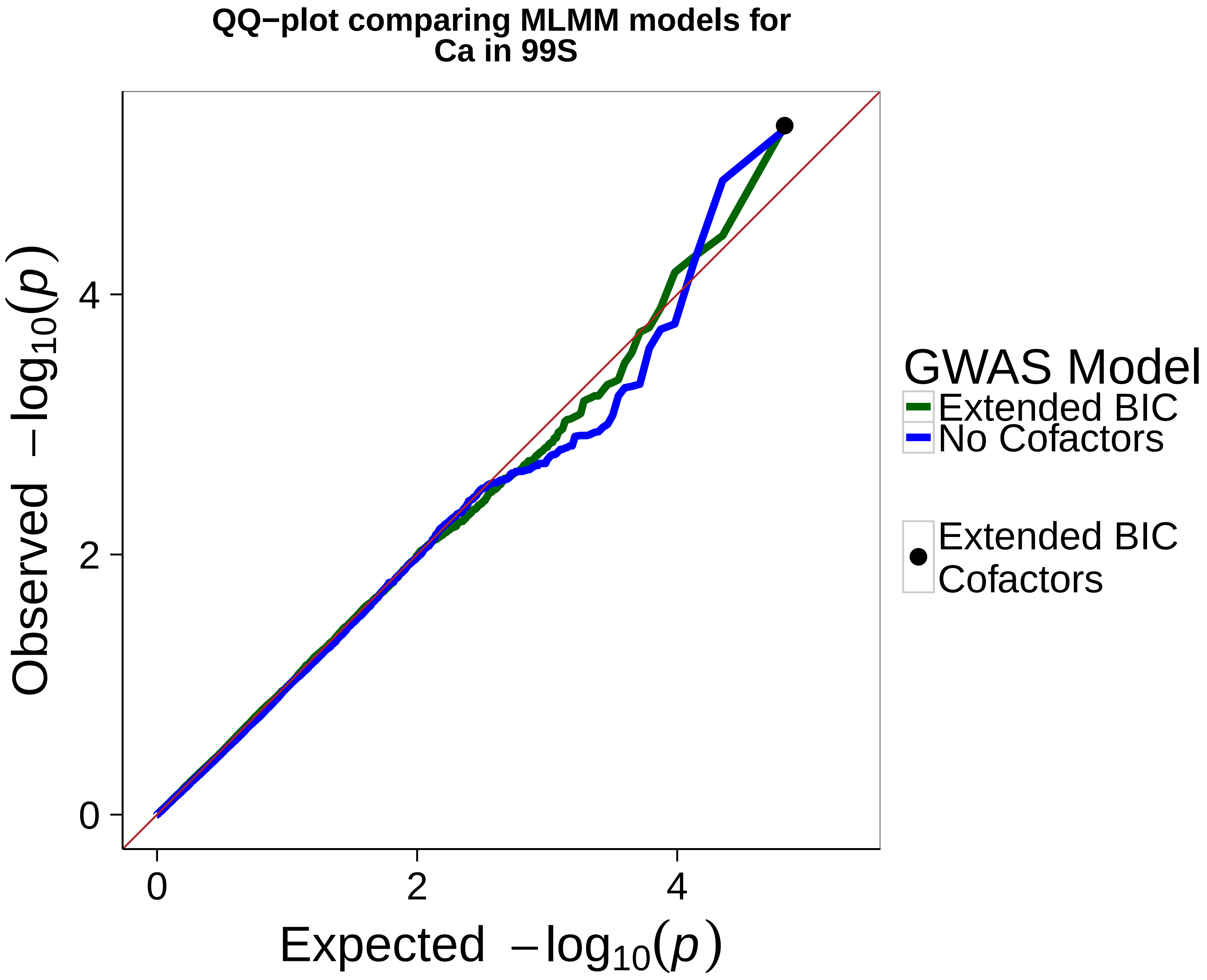
QQ−plot comparing MLMM models for
Ca in 99S
Observed −log10(p)
Expected −log10(p)
0
2
4
GWAS Model
Extended BIC
No Cofactors
Extended BIC
Cofactors

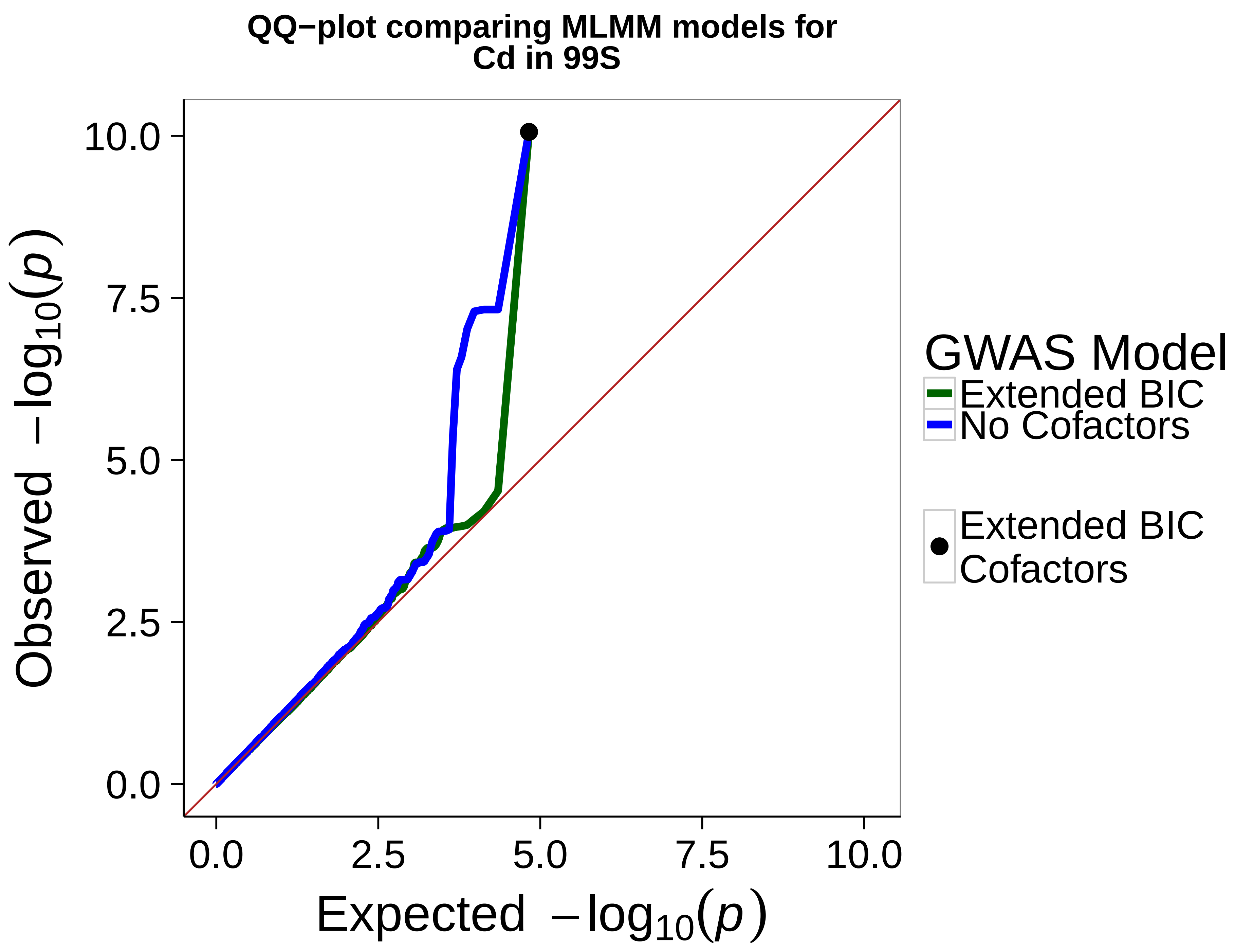
QQ−plot comparing MLMM models for
Cd in 99S
Observed −log10(p)
Expected −log10(p)
0.0
2.5
5.0
7.5
10.0
GWAS Model
Extended BIC
No Cofactors
Extended BIC
Cofactors

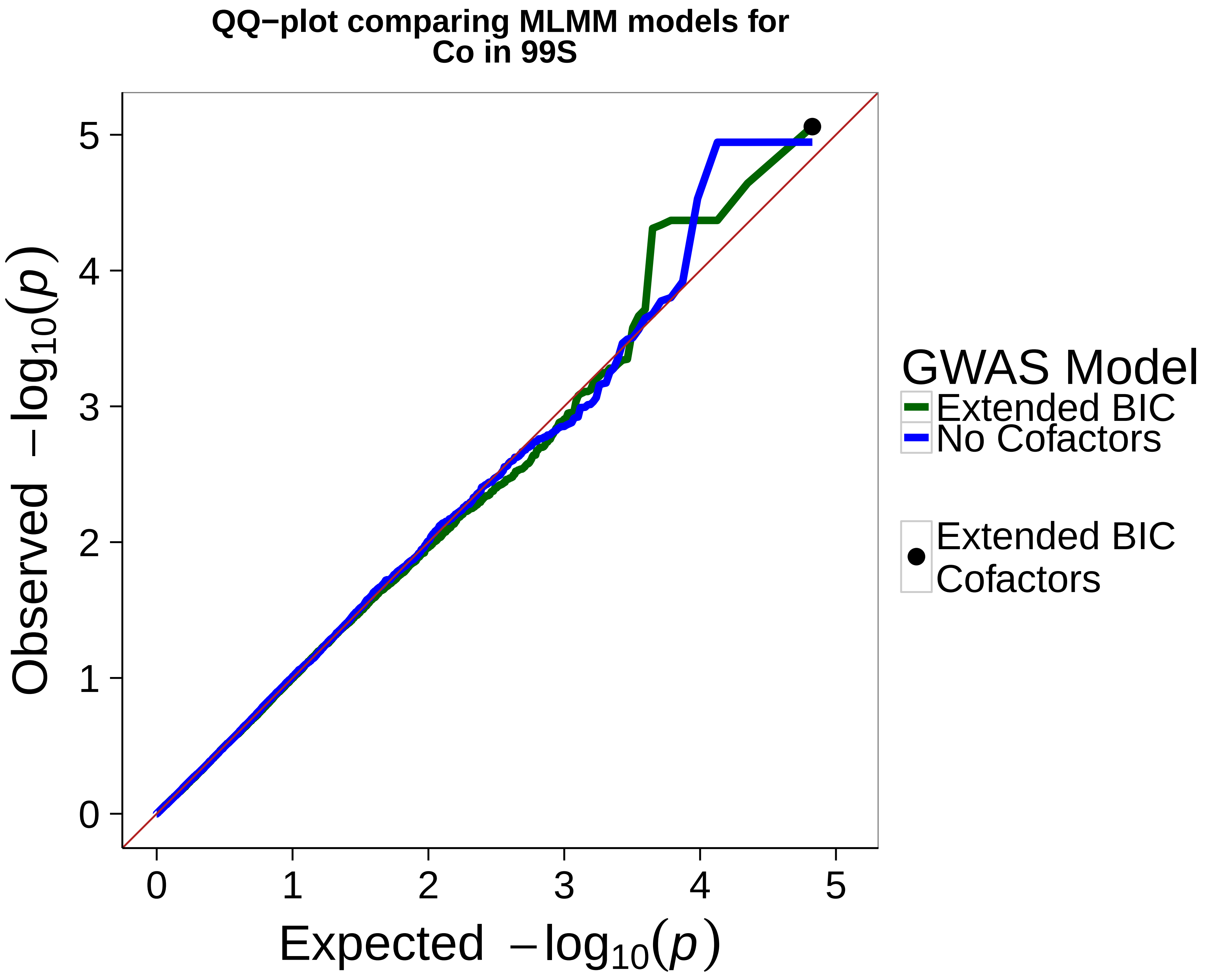
QQ−plot comparing MLMM models for
Co in 99S
Observed −log10(p)
Expected −log10(p)
0
1
2
3
4
5
GWAS Model
Extended BIC
No Cofactors
Extended BIC
Cofactors

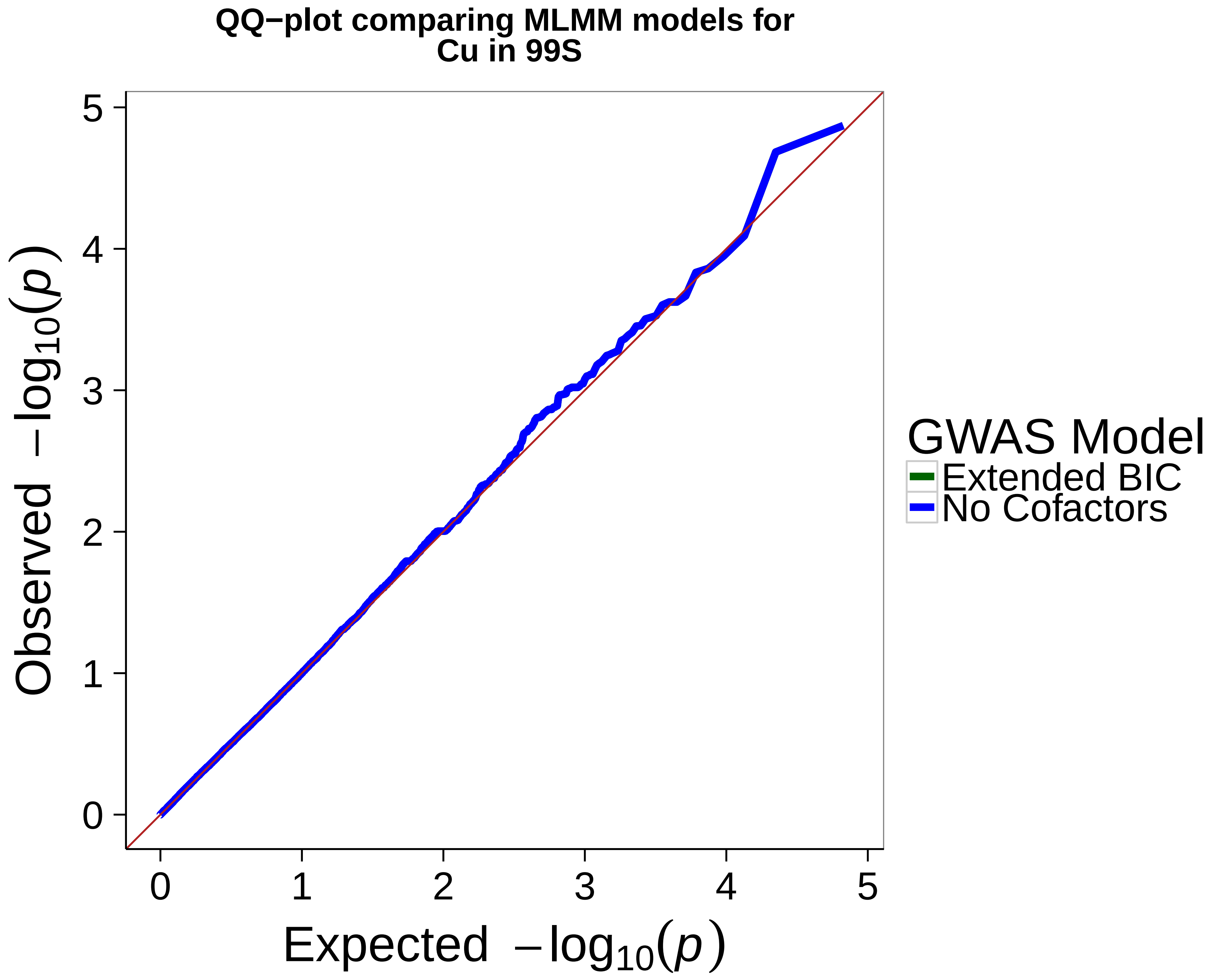
QQ−plot comparing MLMM models for
Cu in 99S
Observed −log10(p)
Expected −log10(p)
0
1
2
3
4
5
GWAS Model
Extended BIC
No Cofactors

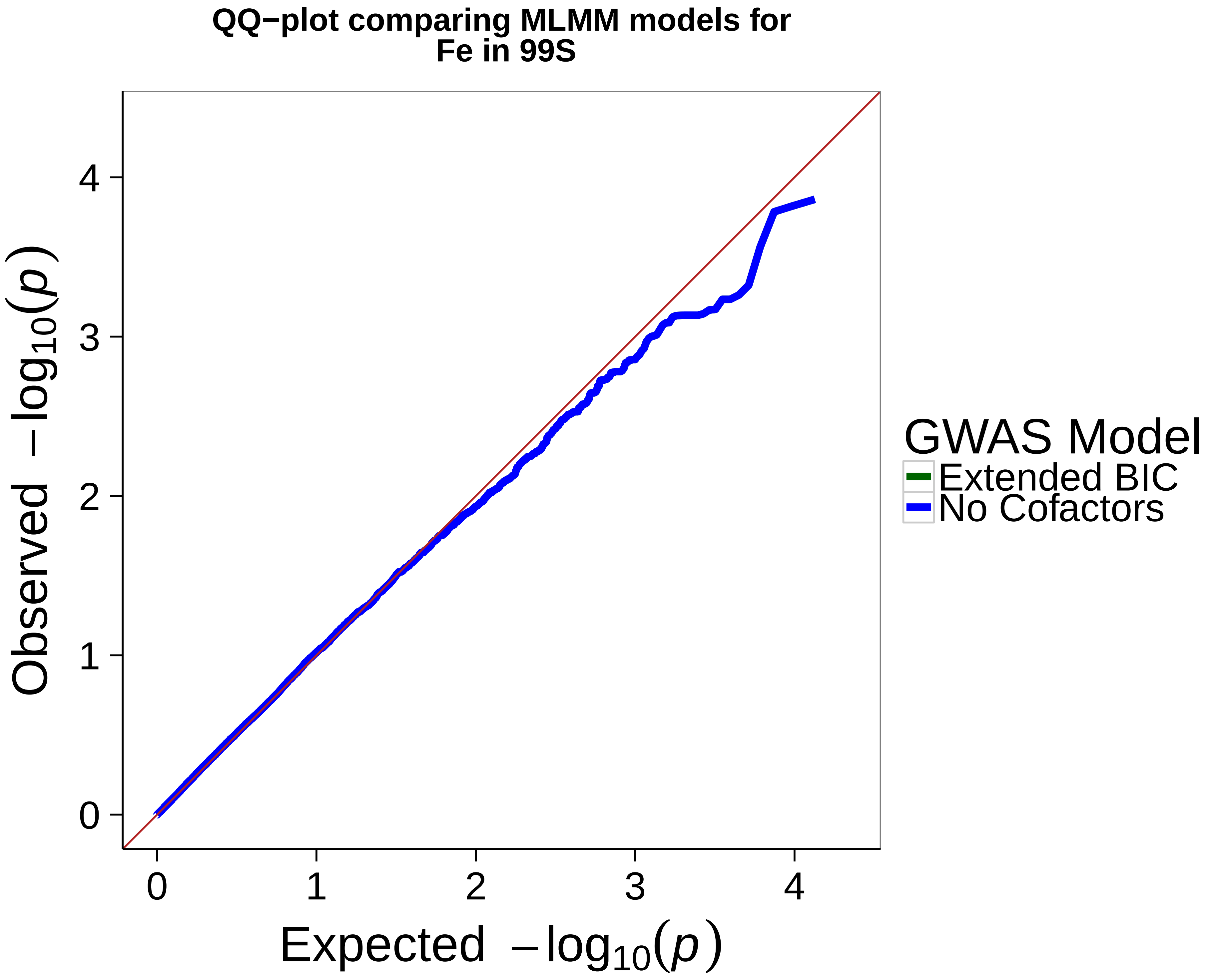
QQ−plot comparing MLMM models for
Fe in 99S
Observed −log10(p)
Expected −log10(p)
0
1
2
3
4
GWAS Model
Extended BIC
No Cofactors

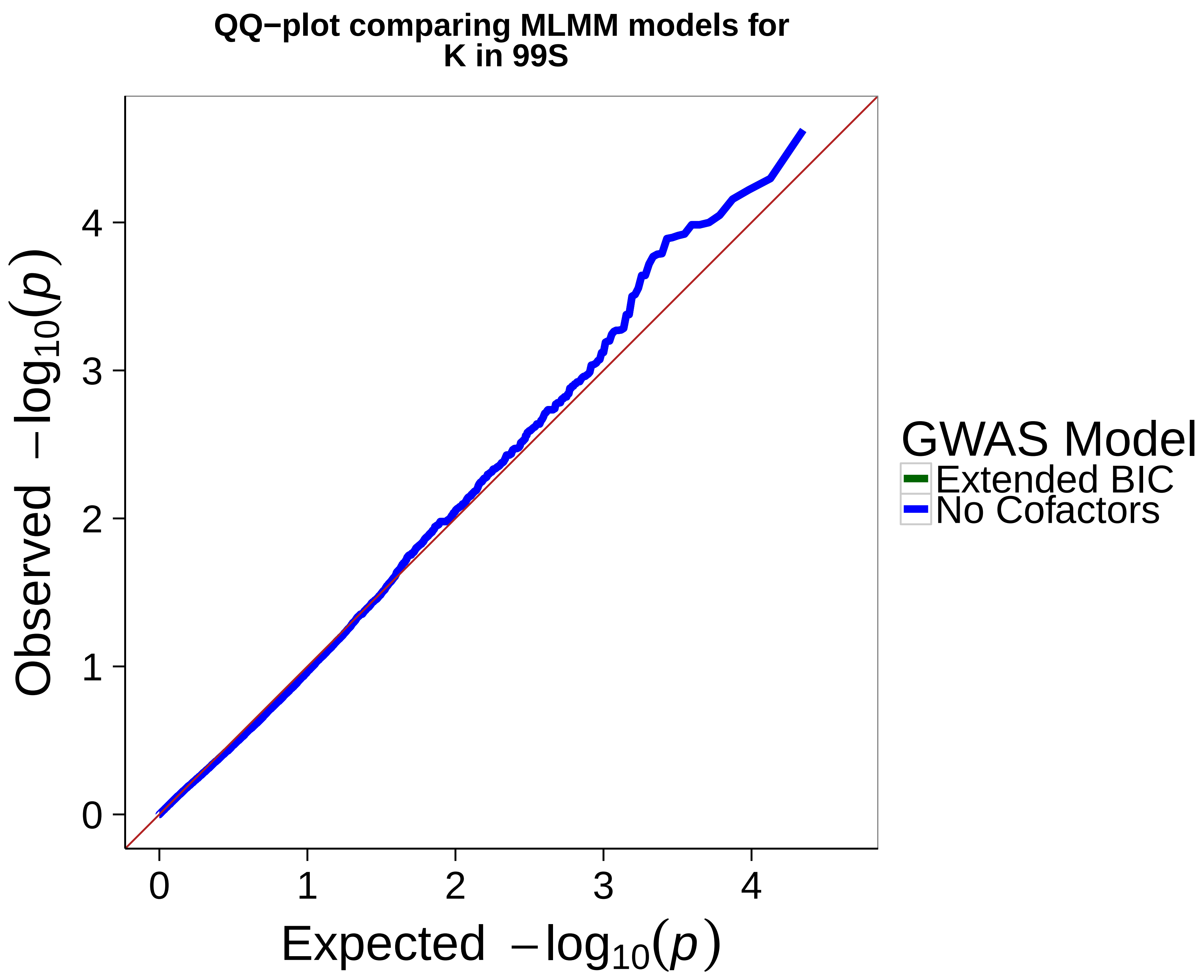
QQ−plot comparing MLMM models for
K in 99S
Observed −log10(p)
Expected −log10(p)
0
1
2
3
4
GWAS Model
Extended BIC
No Cofactors

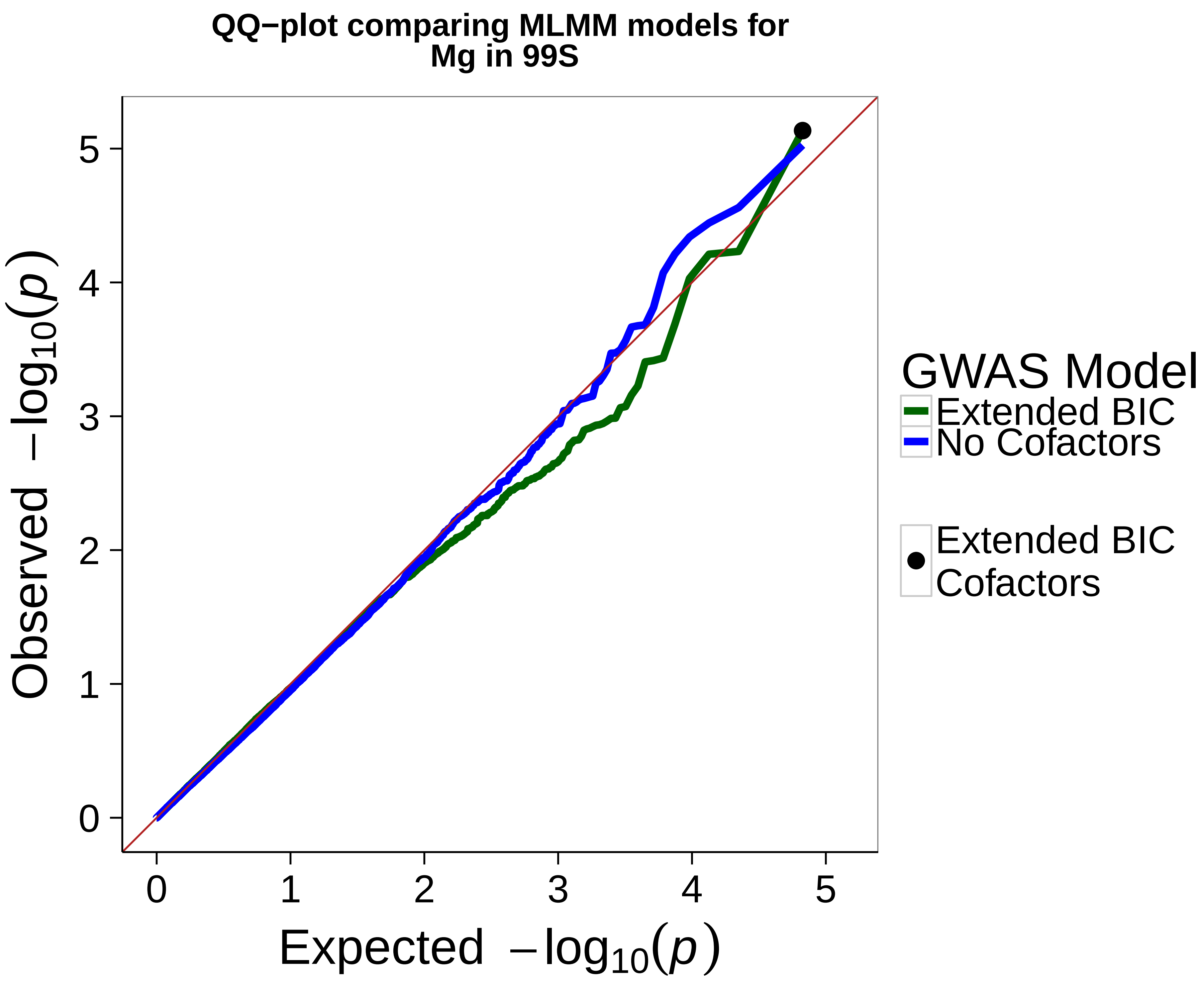
QQ−plot comparing MLMM models for
Mg in 99S
Observed −log10(p)
Expected −log10(p)
0
1
2
3
4
5
GWAS Model
Extended BIC
No Cofactors
Extended BIC
Cofactors

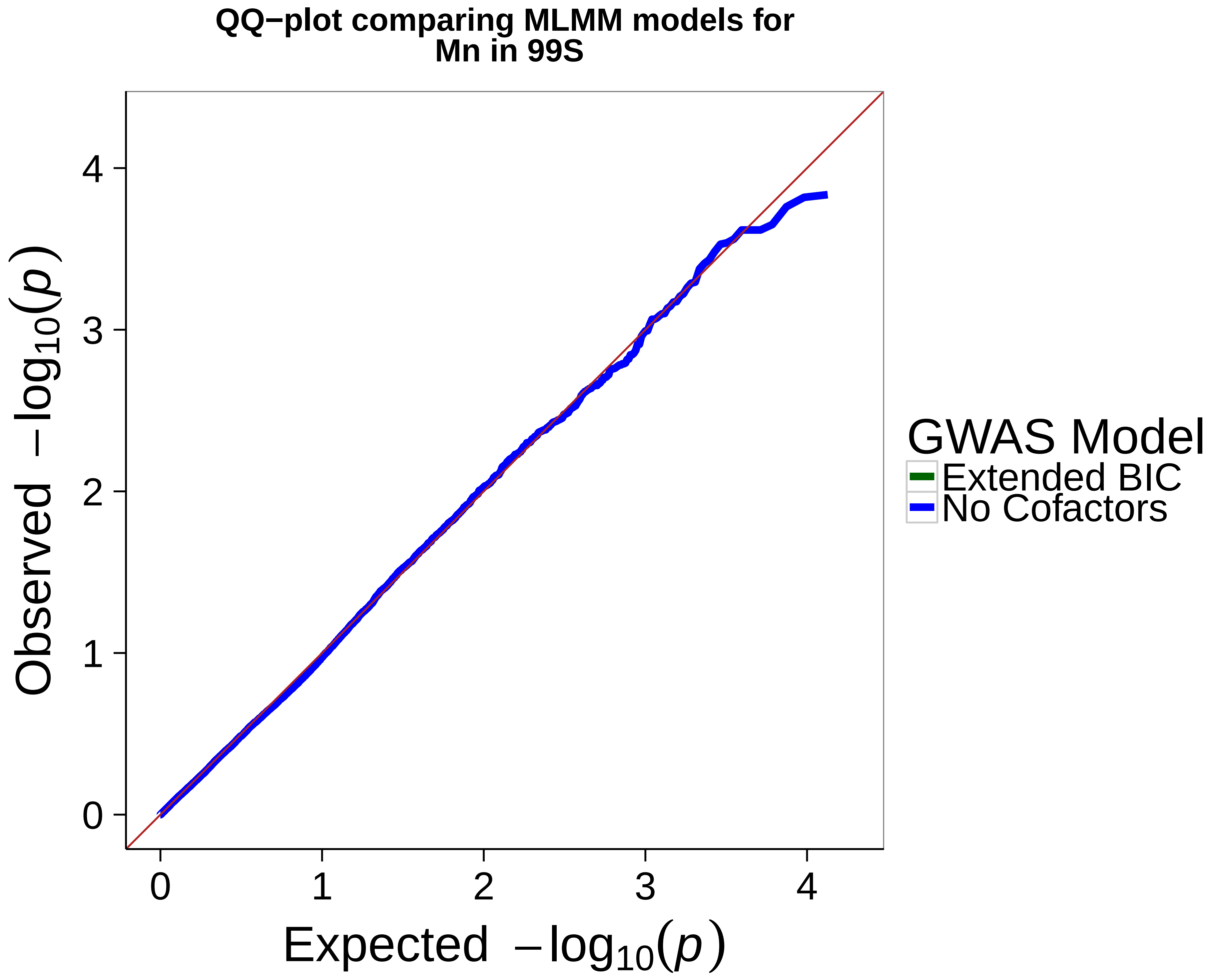
QQ−plot comparing MLMM models for
Mn in 99S
Observed −log10(p)
Expected −log10(p)
0
1
2
3
4
GWAS Model
Extended BIC
No Cofactors

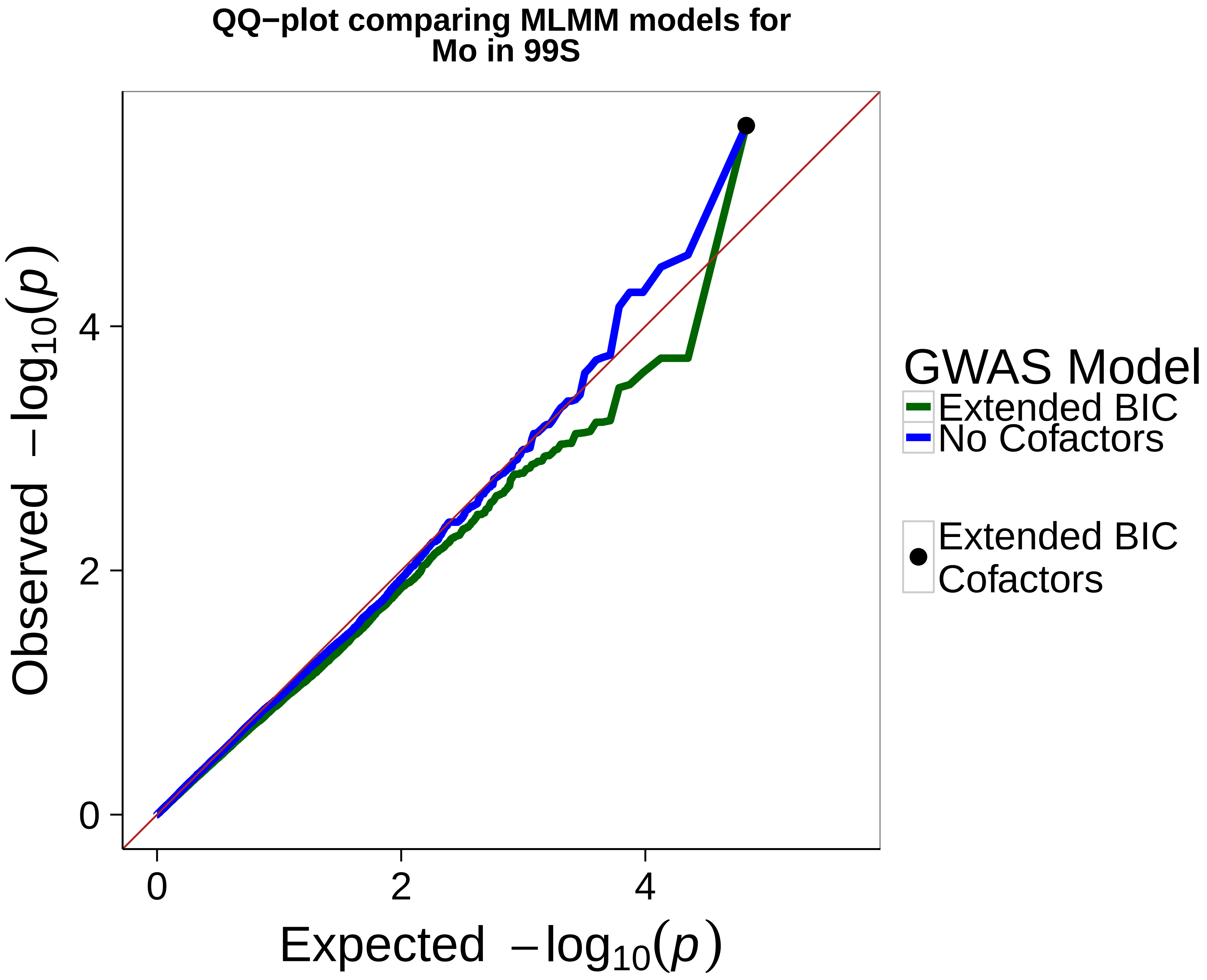
QQ−plot comparing MLMM models for
Mo in 99S
Observed −log10(p)
Expected −log10(p)
0
2
4
GWAS Model
Extended BIC
No Cofactors
Extended BIC
Cofactors

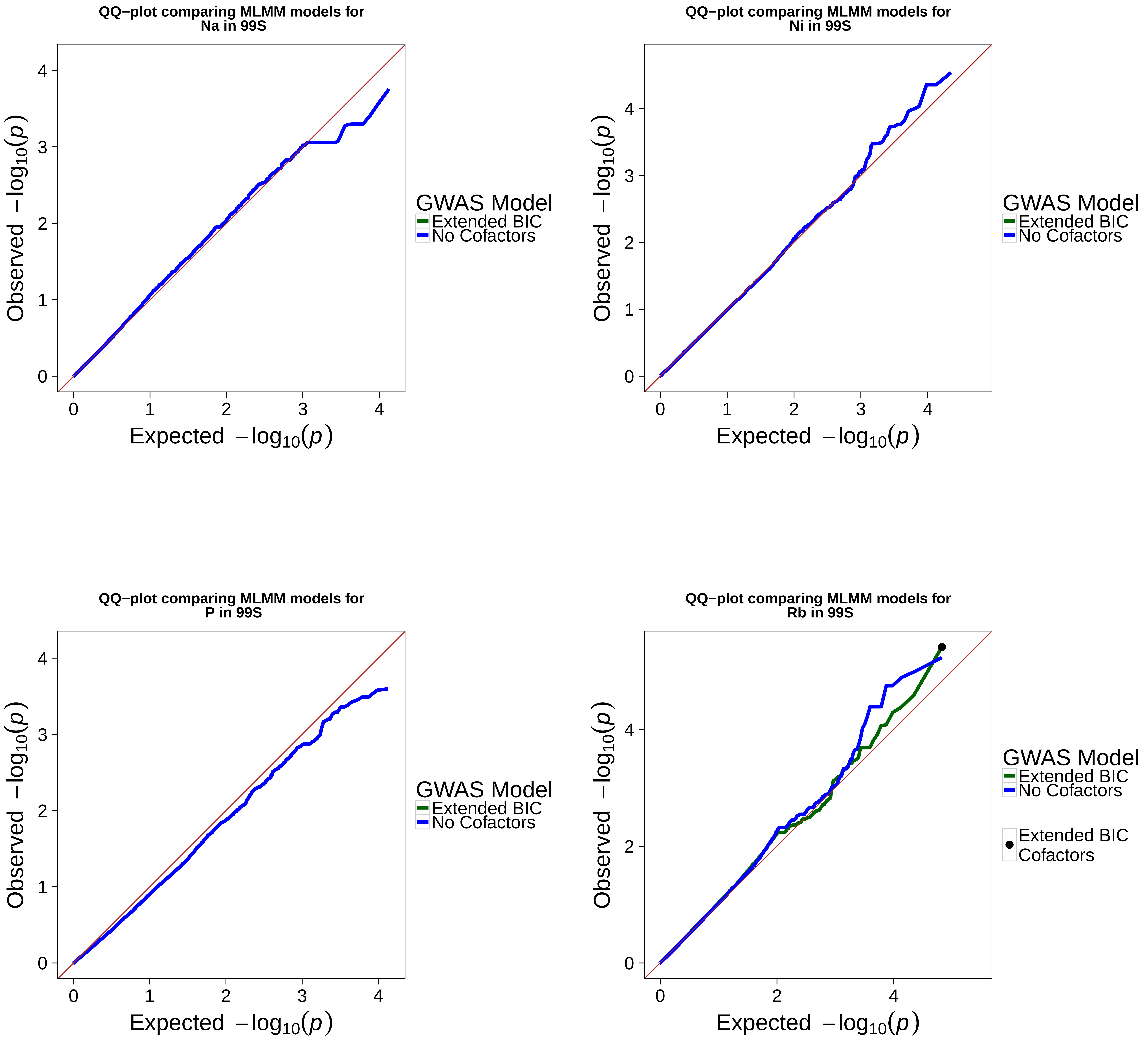
QQ−plot comparing MLMM models for
Na in 99S
Observed −log10(p)
Expected −log10(p)
GWAS Model
Extended BIC
No Cofactors
QQ−plot comparing MLMM models for
Ni in 99S
Observed −log10(p)
Expected −log10(p)
GWAS Model
Extended BIC
No Cofactors
QQ−plot comparing MLMM models for
P in 99S
Observed −log10(p)
Expected −log10(p)
GWAS Model
Extended BIC
No Cofactors
QQ−plot comparing MLMM models for
Rb in 99S
Observed −log10(p)
Expected −log10(p)
GWAS Model
Extended BIC
No Cofactors
Extended BIC
Cofactors

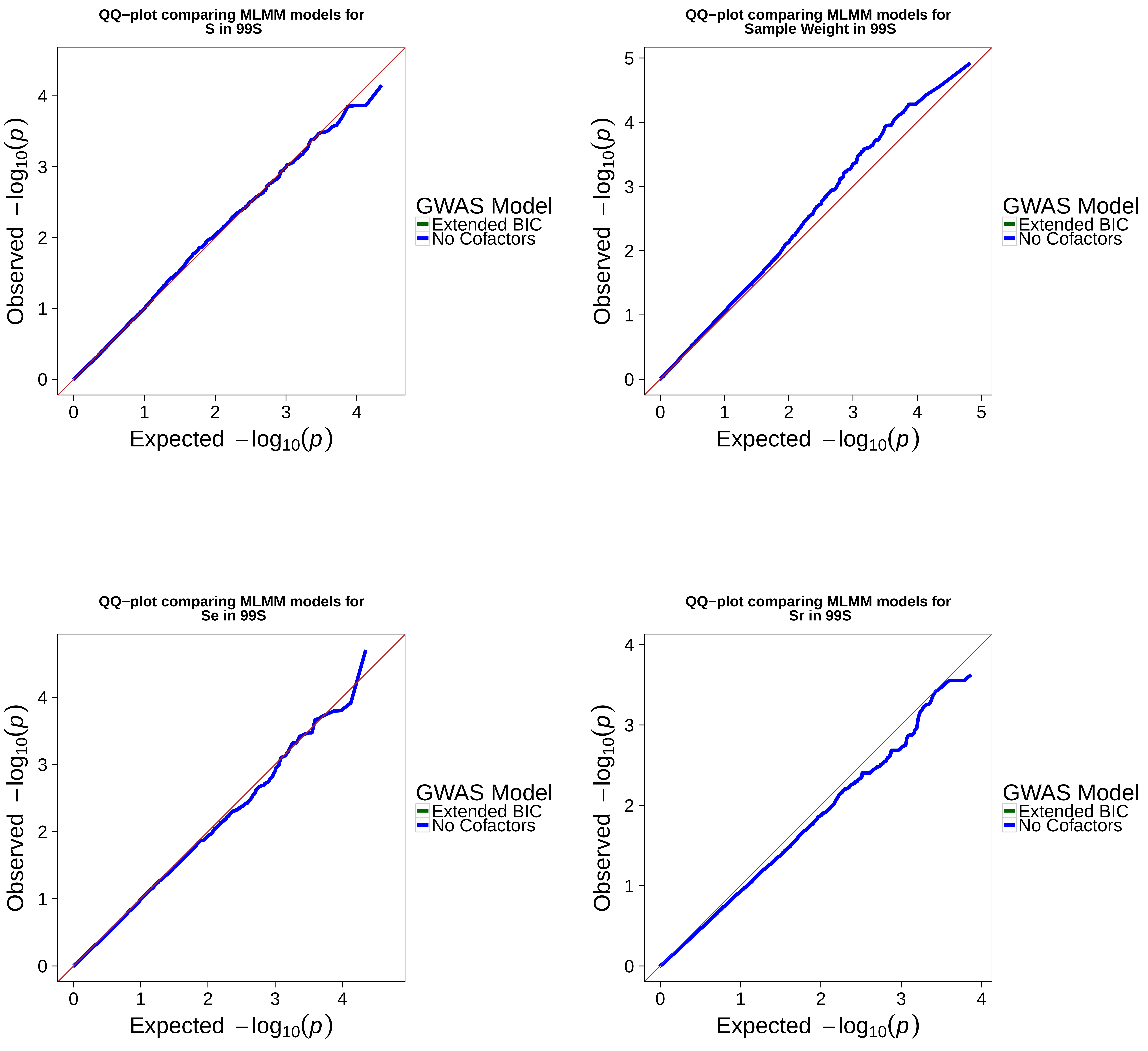
QQ−plot comparing MLMM models for
S in 99S
Observed −log10(p)
Expected −log10(p)
GWAS Model
Extended BIC
No Cofactors
QQ−plot comparing MLMM models for
Sample Weight in 99S
Observed −log10(p)
Expected −log10(p)
GWAS Model
Extended BIC
No Cofactors
QQ−plot comparing MLMM models for
Se in 99S
Observed −log10(p)
Expected −log10(p)
GWAS Model
Extended BIC
No Cofactors
QQ−plot comparing MLMM models for
Sr in 99S
Observed −log10(p)
Expected −log10(p)
GWAS Model
Extended BIC
No Cofactors

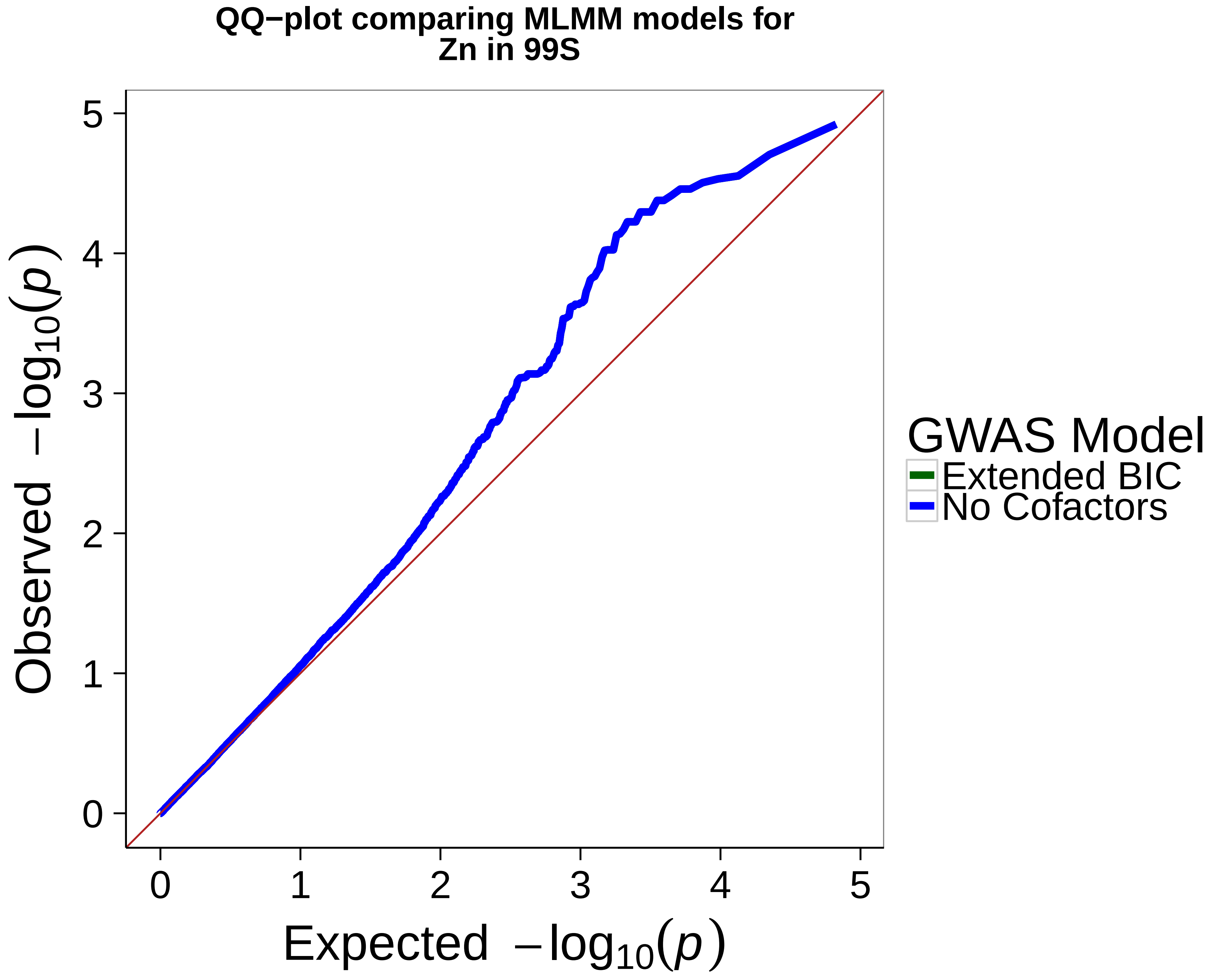
QQ−plot comparing MLMM models for
Zn in 99S
Observed $-\log_{10}(p)$
Expected $-\log_{10}(p)$
GWAS Model
Extended BIC
No Cofactors

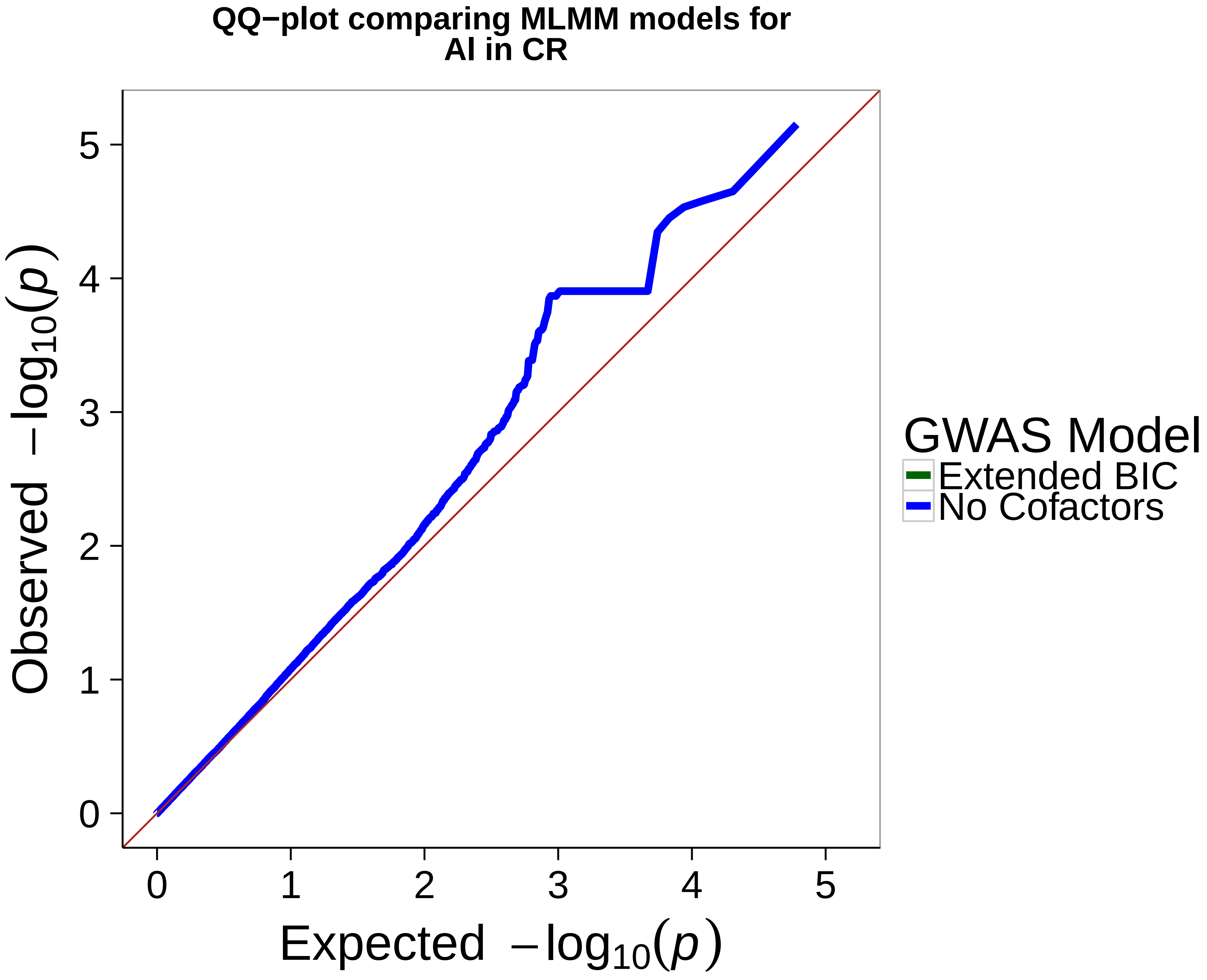
QQ−plot comparing MLMM models for
Al in CR
Observed $-\log_{10}(p)$
Expected $-\log_{10}(p)$
GWAS Model
Extended BIC
No Cofactors

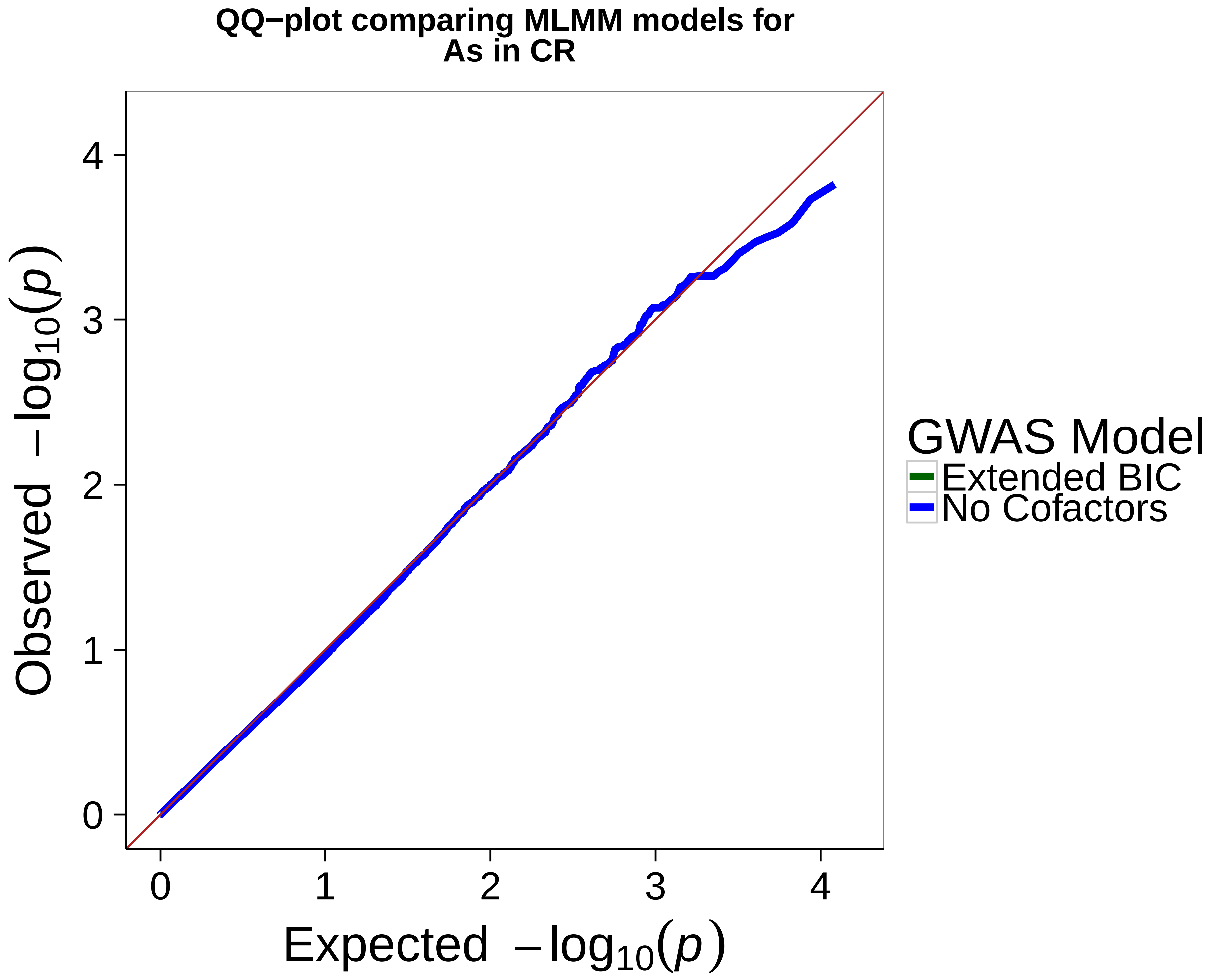
QQ−plot comparing MLMM models for
As in CR
Observed $-\log_{10}(p)$
Expected $-\log_{10}(p)$
GWAS Model
Extended BIC
No Cofactors

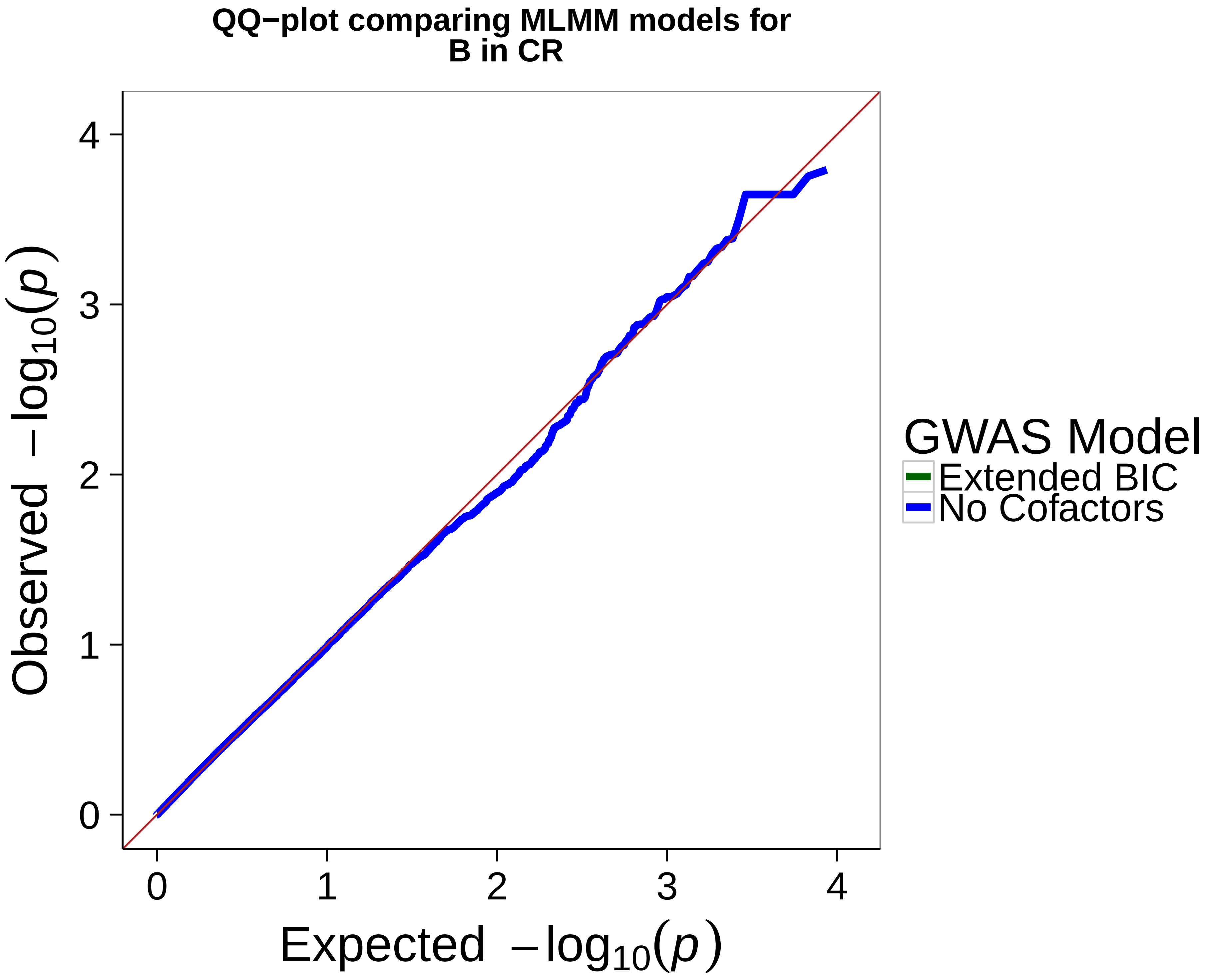
QQ−plot comparing MLMM models for
B in CR
Observed $-\log_{10}(p)$
Expected $-\log_{10}(p)$
GWAS Model
Extended BIC
No Cofactors

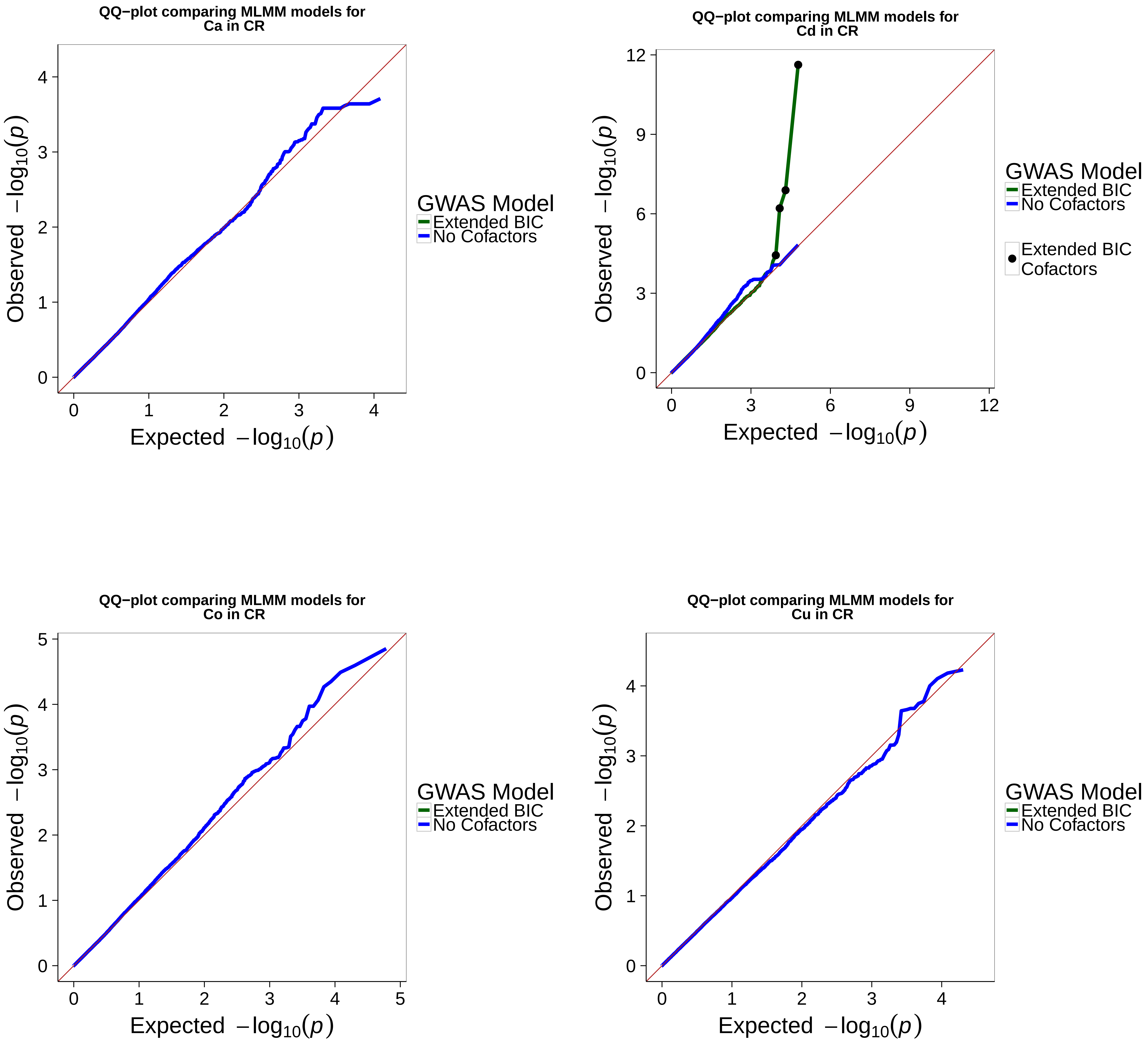
QQ−plot comparing MLMM models for Ca in CR
Observed −log10(p)
Expected −log10(p)
GWAS Model
Extended BIC
No Cofactors
QQ−plot comparing MLMM models for Cd in CR
Observed −log10(p)
Expected −log10(p)
GWAS Model
Extended BIC
No Cofactors
Extended BIC Cofactors
QQ−plot comparing MLMM models for Co in CR
Observed −log10(p)
Expected −log10(p)
GWAS Model
Extended BIC
No Cofactors
QQ−plot comparing MLMM models for Cu in CR
Observed −log10(p)
Expected −log10(p)
GWAS Model
Extended BIC
No Cofactors

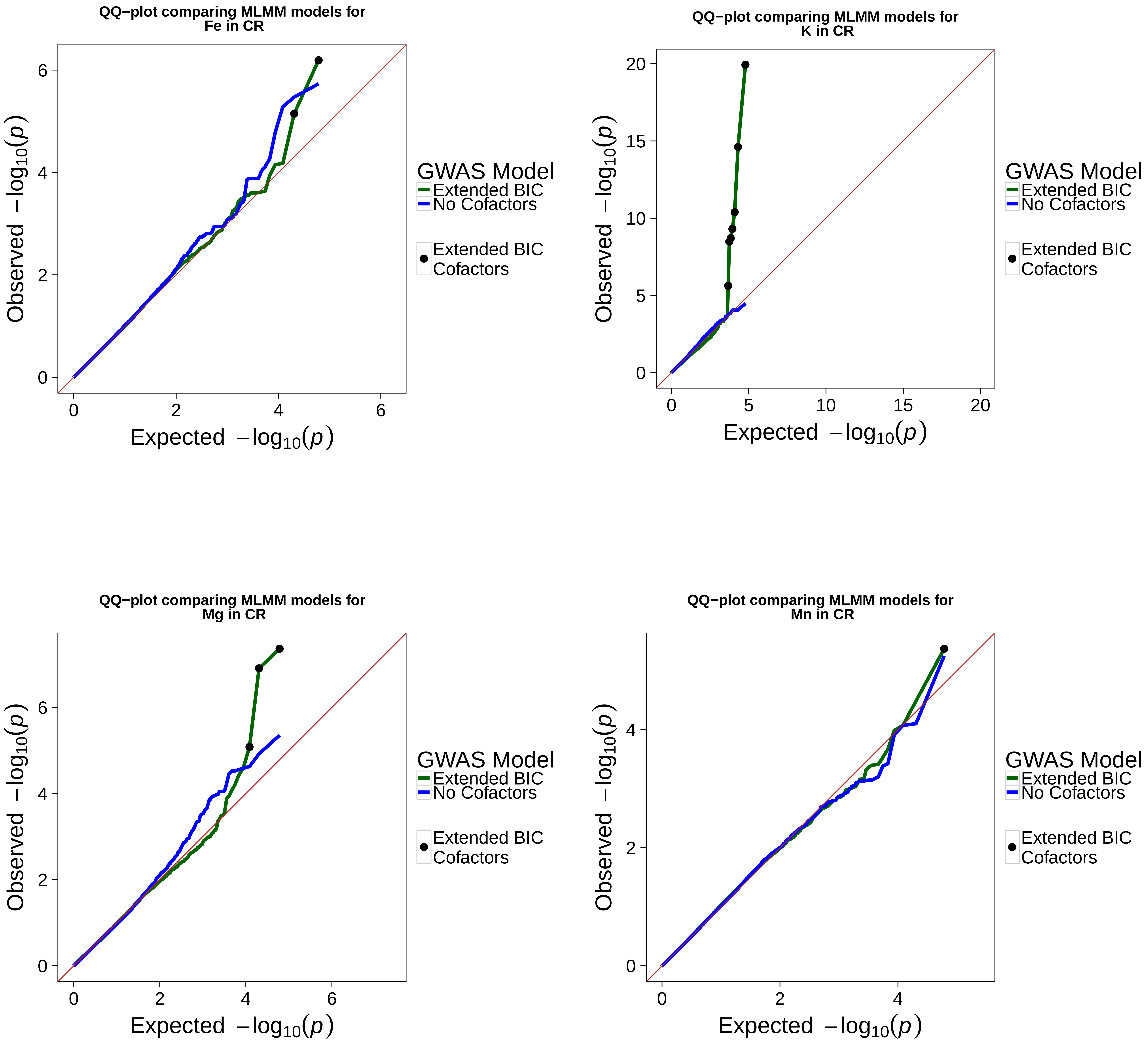

QQ−plot comparing MLMM models for Fe in CR
QQ−plot comparing MLMM models for K in CR
QQ−plot comparing MLMM models for Mg in CR
QQ−plot comparing MLMM models for Mn in CR
Observed $-\log_{10}(p)$
Expected $-\log_{10}(p)$
GWAS Model
Extended BIC
No Cofactors
Extended BIC Cofactors

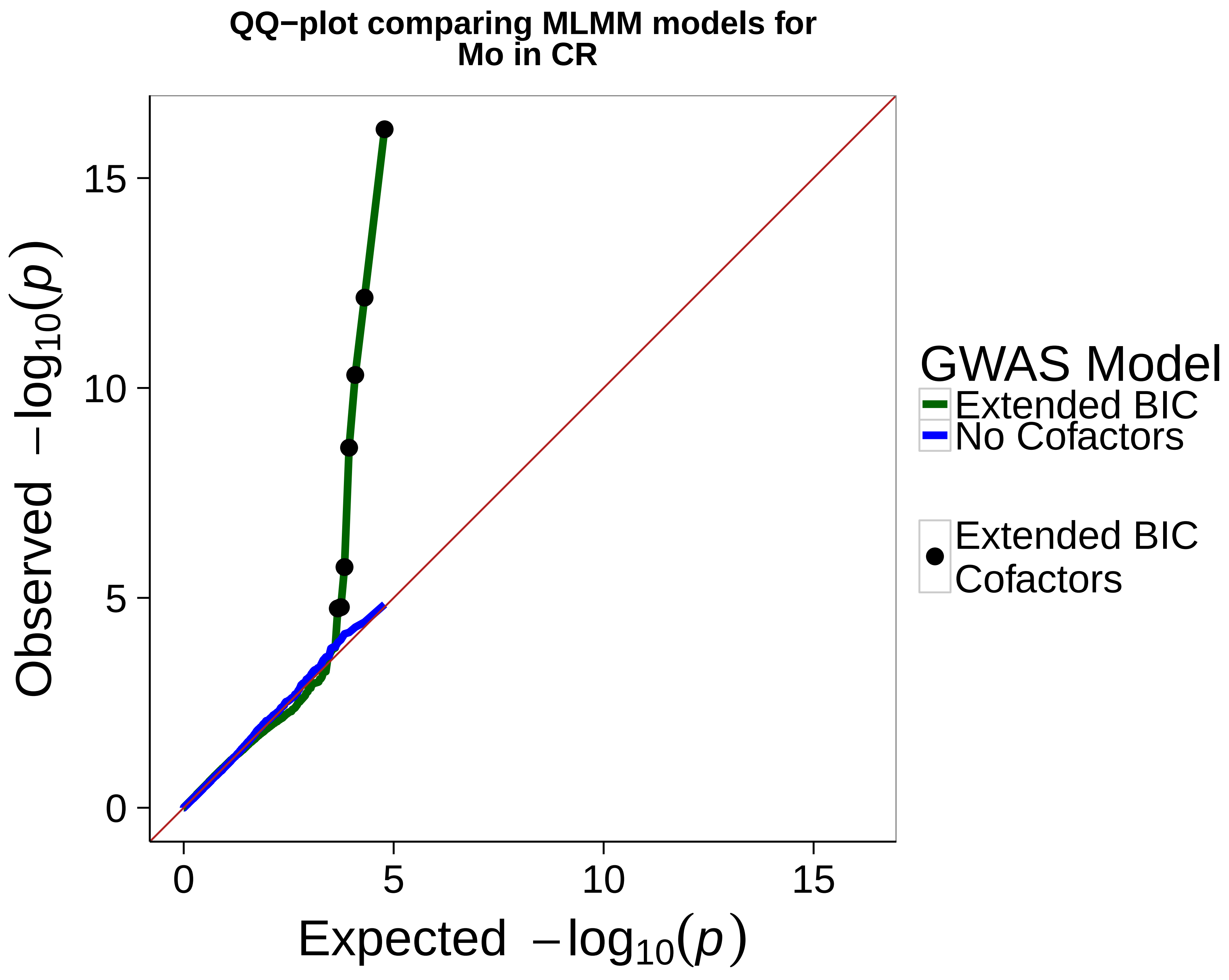
QQ−plot comparing MLMM models for
Mo in CR
Observed −log10(p)
Expected −log10(p)
0
5
10
15
GWAS Model
Extended BIC
No Cofactors
Extended BIC
Cofactors

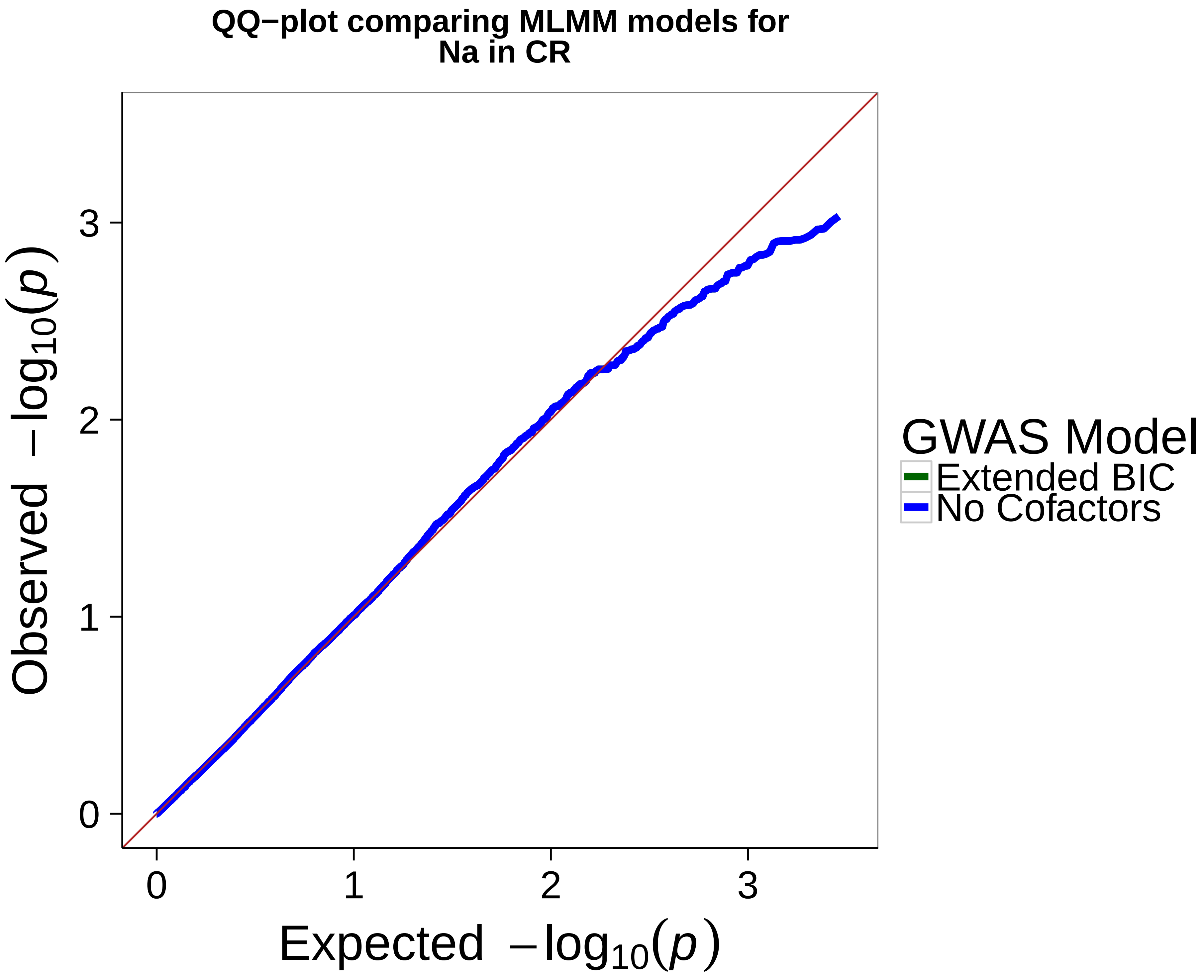
QQ−plot comparing MLMM models for
Na in CR
Observed −log10(p)
Expected −log10(p)
0
1
2
3
GWAS Model
Extended BIC
No Cofactors

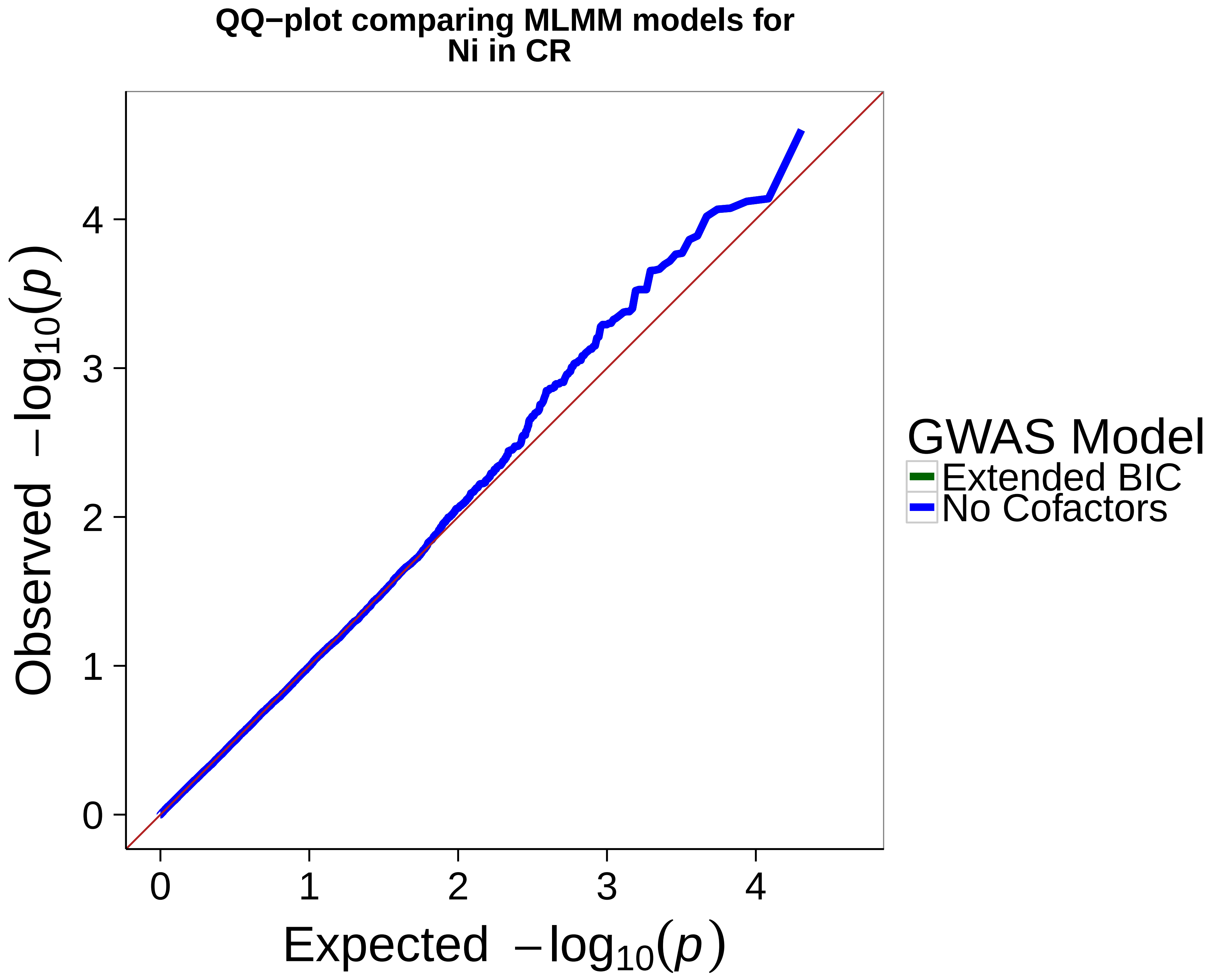
QQ−plot comparing MLMM models for
Ni in CR
Observed −log10(p)
Expected −log10(p)
0
1
2
3
4
GWAS Model
Extended BIC
No Cofactors

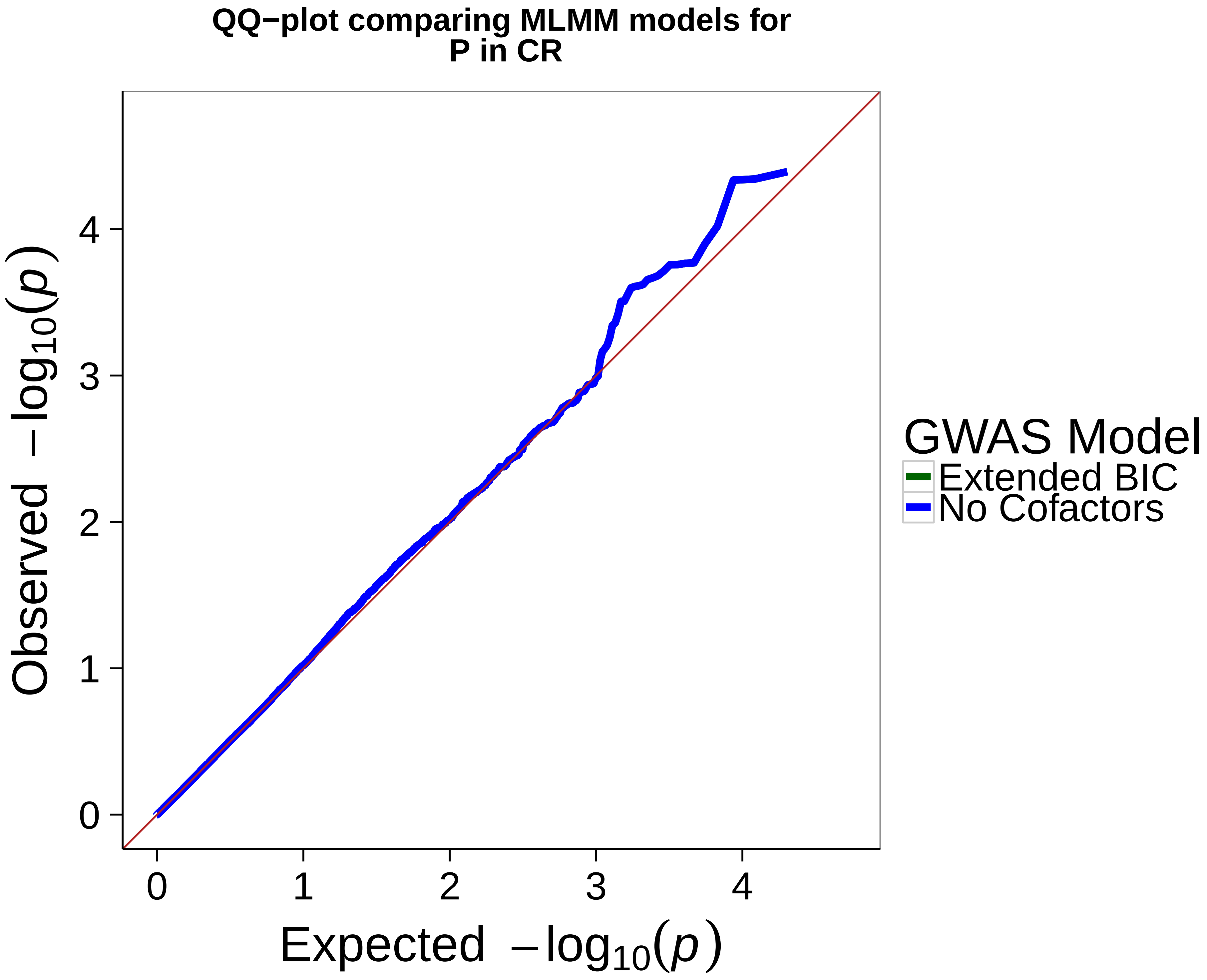
QQ−plot comparing MLMM models for
P in CR
Observed −log10(p)
Expected −log10(p)
0
1
2
3
4
GWAS Model
Extended BIC
No Cofactors

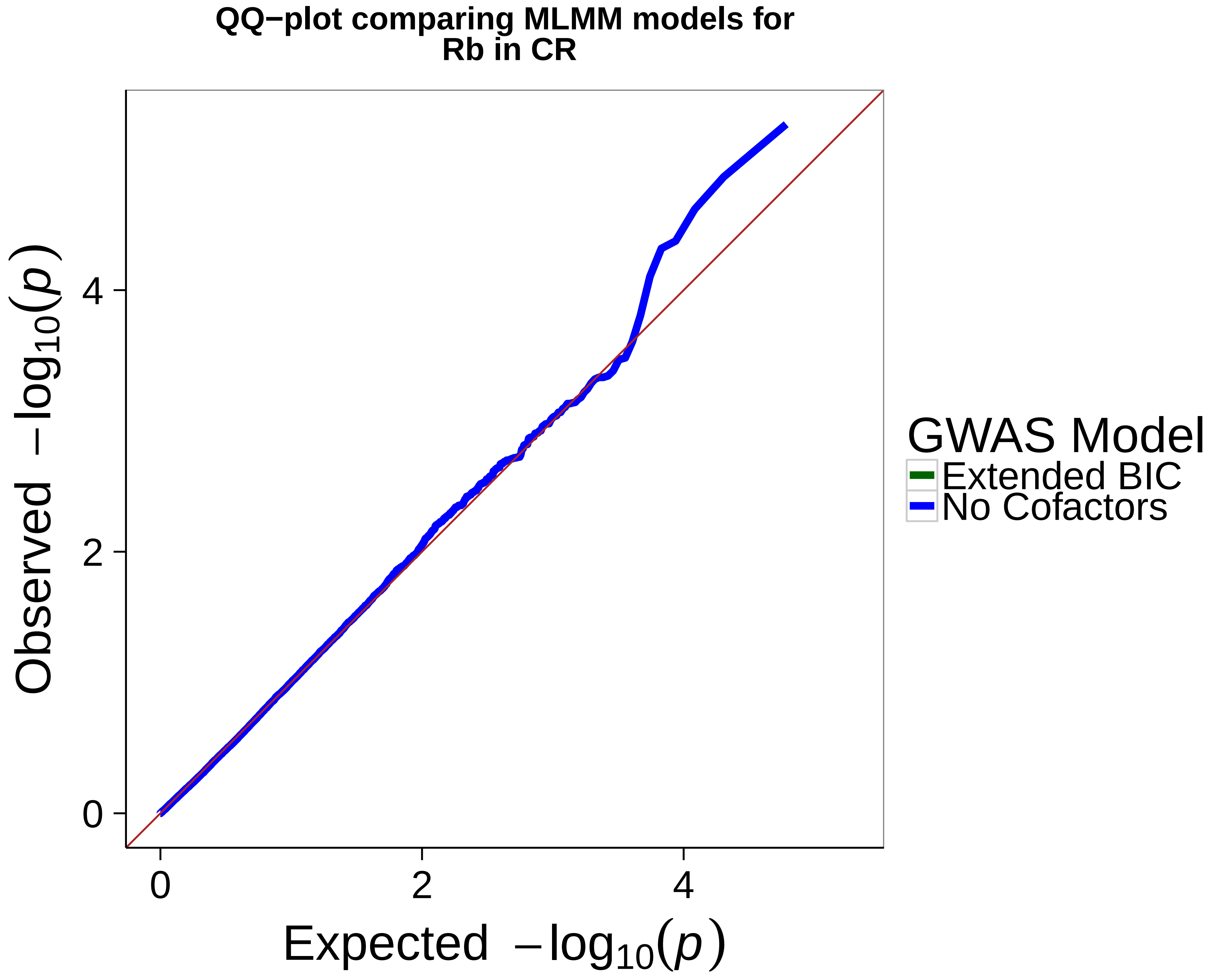
QQ−plot comparing MLMM models for
Rb in CR
Observed −log10(p)
Expected −log10(p)
0
2
4
GWAS Model
Extended BIC
No Cofactors

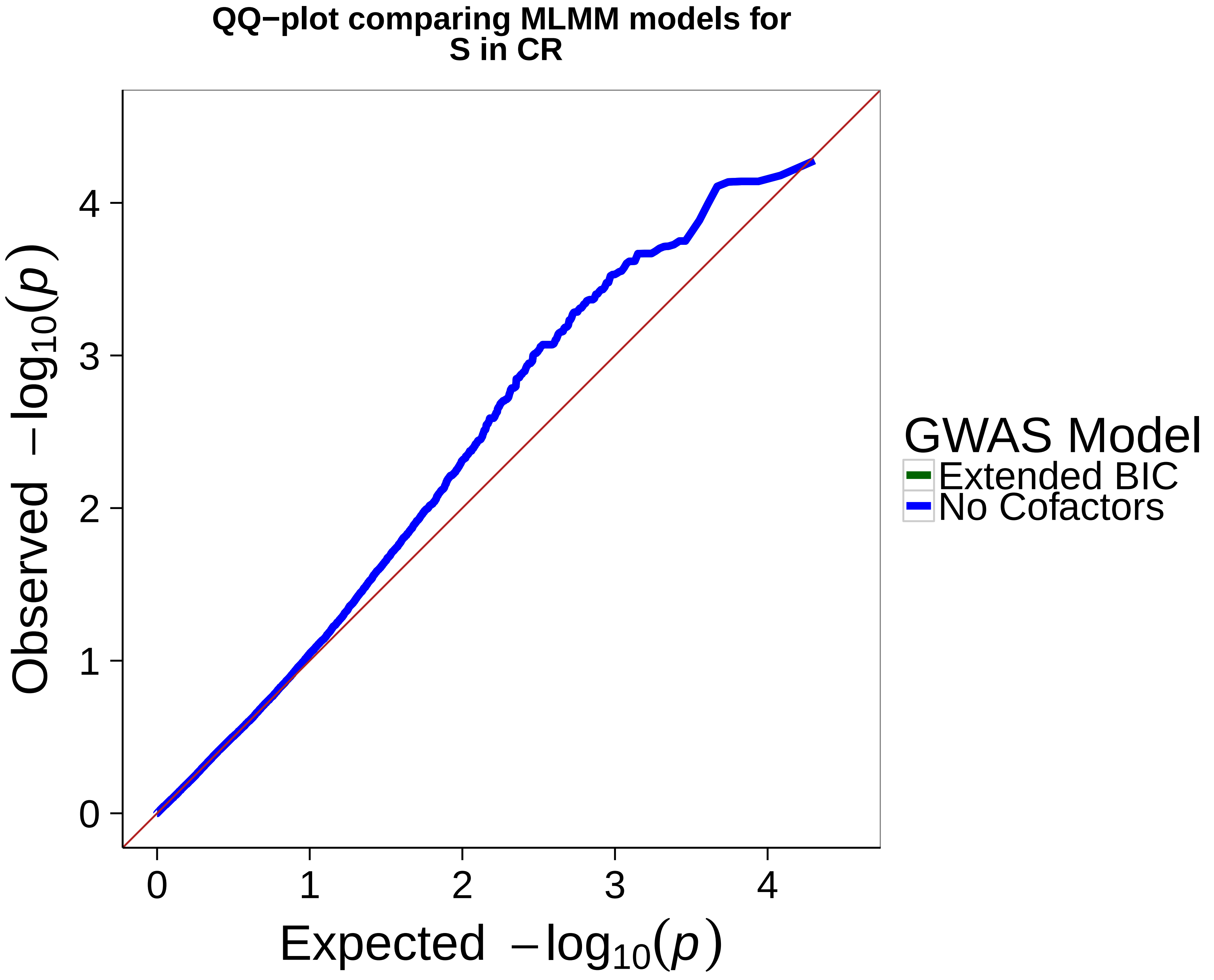
QQ−plot comparing MLMM models for
S in CR
Observed −log10(p)
Expected −log10(p)
0
1
2
3
4
GWAS Model
Extended BIC
No Cofactors

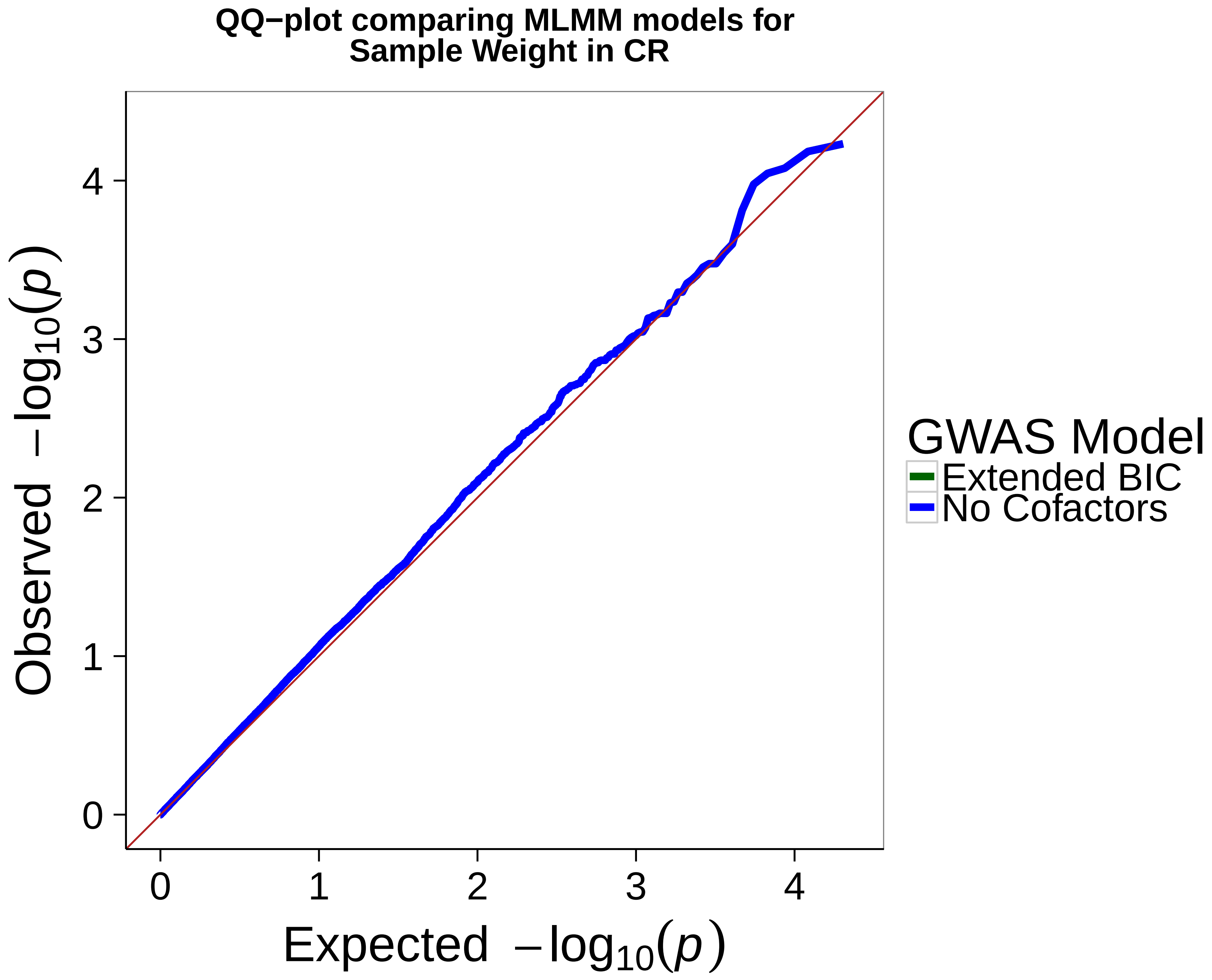
QQ−plot comparing MLMM models for
Sample Weight in CR
Observed −log10(p)
Expected −log10(p)
0
1
2
3
4
GWAS Model
Extended BIC
No Cofactors

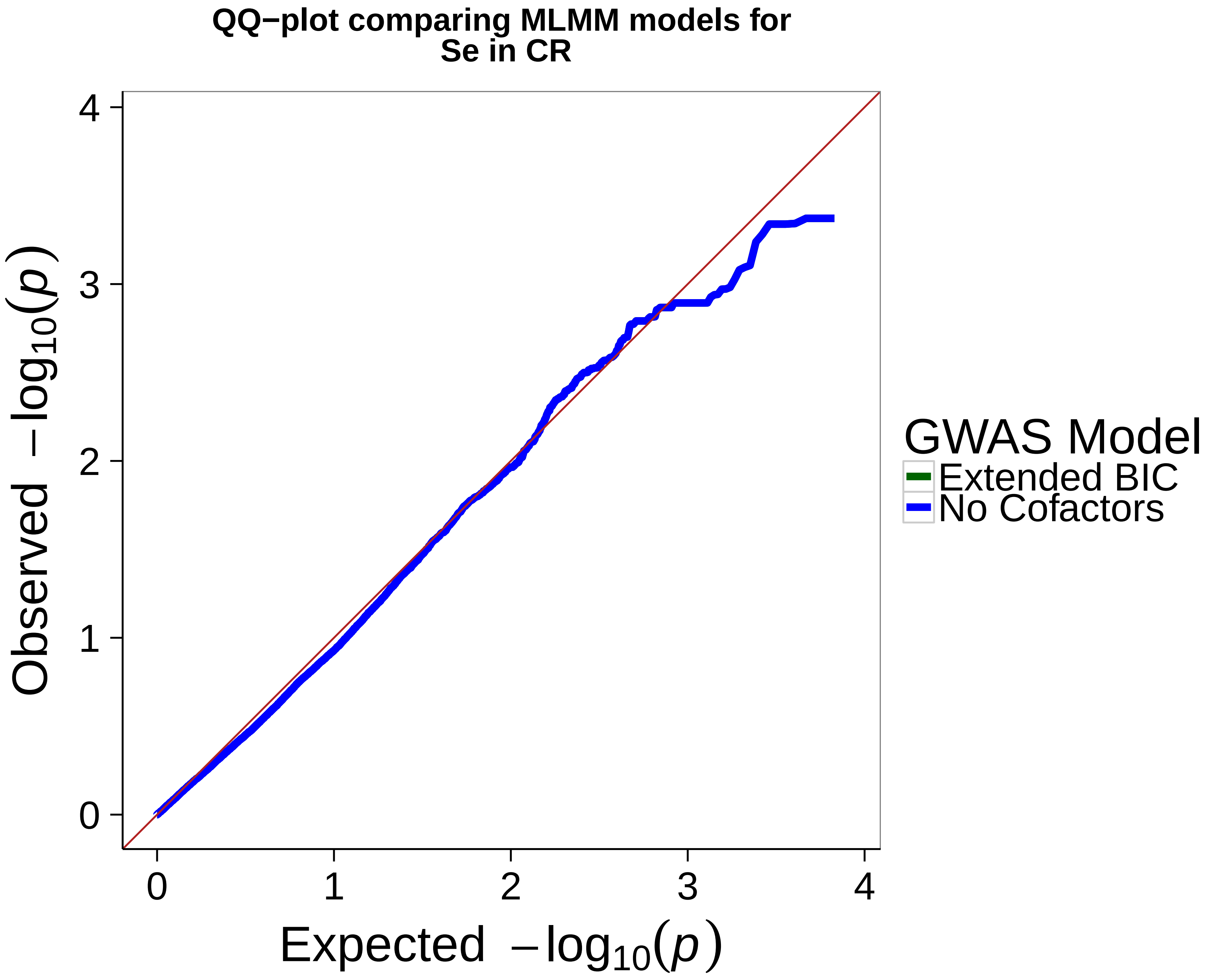
QQ−plot comparing MLMM models for
Se in CR
Observed −log10(p)
Expected −log10(p)
0
1
2
3
4
GWAS Model
Extended BIC
No Cofactors

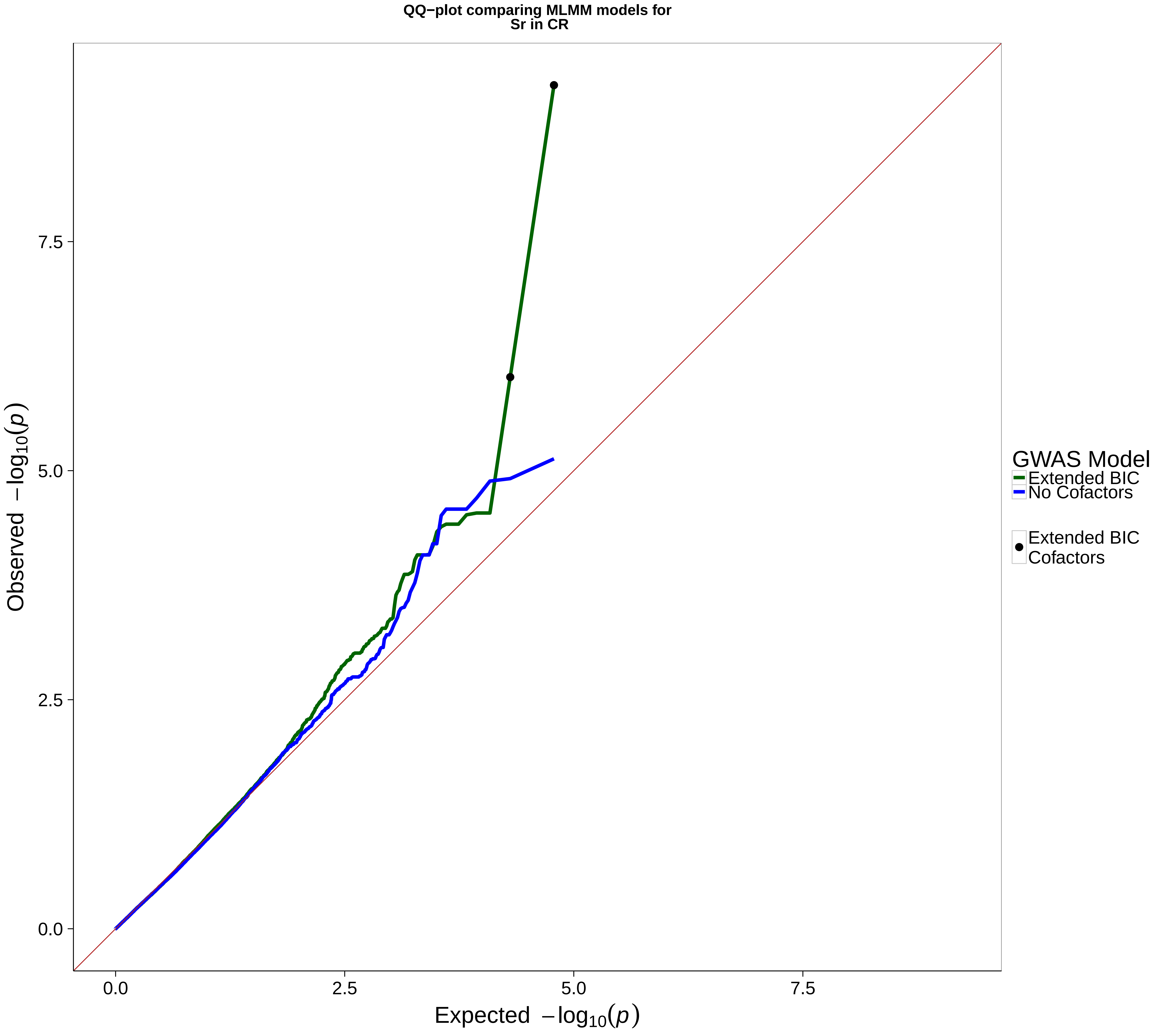
QQ−plot comparing MLMM models for
Sr in CR
Observed −log10(p)
Expected −log10(p)
0.0
2.5
5.0
7.5
GWAS Model
Extended BIC
No Cofactors
Extended BIC
Cofactors
