# Supplementary material for: Genomewide association study of ionomic traits on diverse soybean populations from germplasm collections
Source: Plant Direct. 2018 Jan 15;2(1):e00033. doi: 10.1002/pld3.33 (PMC6508489; doi:10.1002/pld3.33)
Supplement: Supplementary file 5 [file PLD3-2-e00033-s005.pdf]

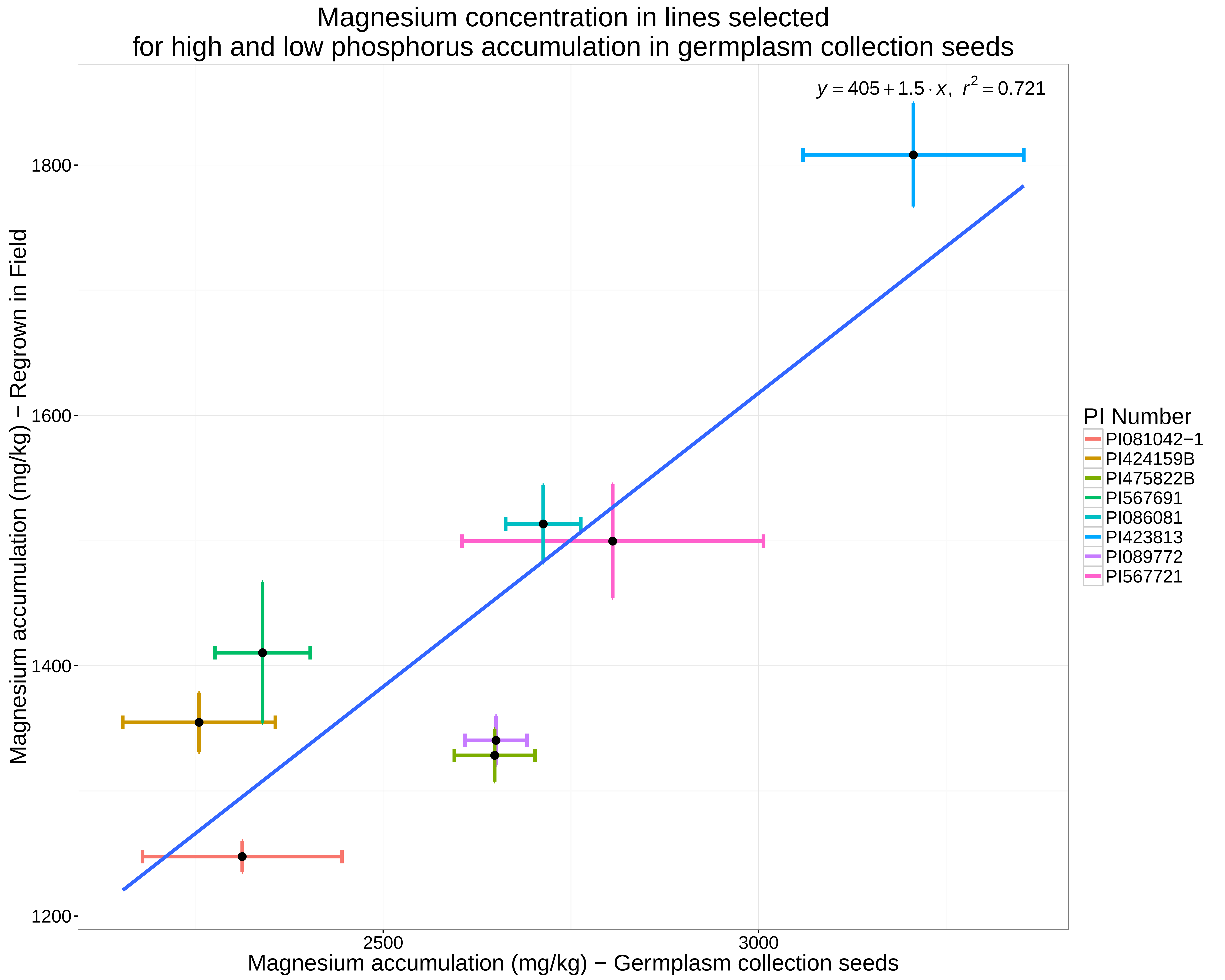

Magnesium concentration in lines selected
for high and low phosphorus accumulation in germplasm collection seeds
$y = 405 + 1.5 \cdot x,\ r^2 = 0.721$
1800
1600
1400
1200
2500
3000
Magnesium accumulation (mg/kg) − Regrown in Field
Magnesium accumulation (mg/kg) − Germplasm collection seeds
PI Number
PI081042−1
PI424159B
PI475822B
PI567691
PI086081
PI423813
PI089772
PI567721

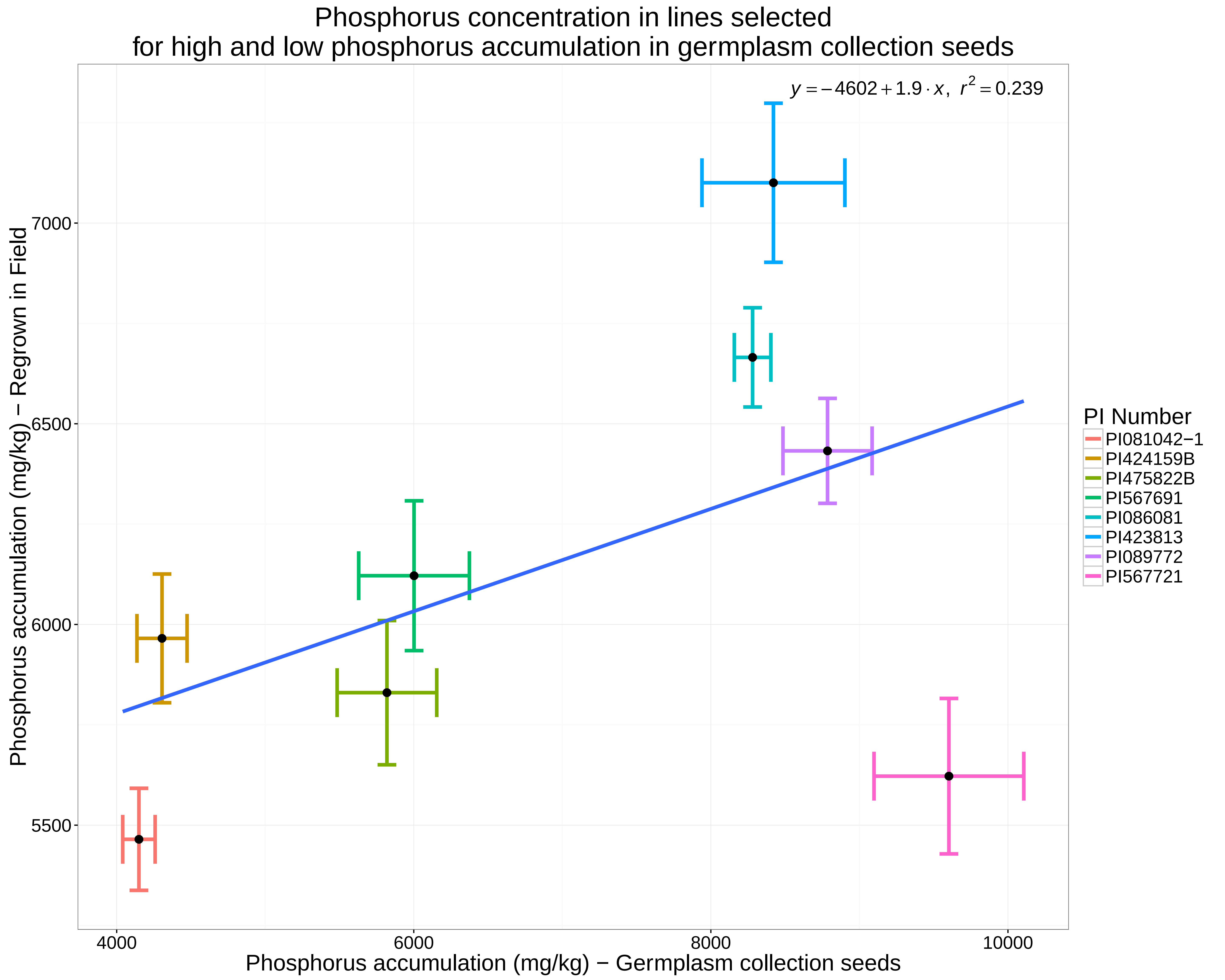
Phosphorus concentration in lines selected
for high and low phosphorus accumulation in germplasm collection seeds
y = −4602 + 1.9 · x, r2 = 0.239
Phosphorus accumulation (mg/kg) − Regrown in Field
7000
6500
6000
5500
4000
6000
8000
10000
Phosphorus accumulation (mg/kg) − Germplasm collection seeds
PI Number
PI081042−1
PI424159B
PI475822B
PI567691
PI086081
PI423813
PI089772
PI567721

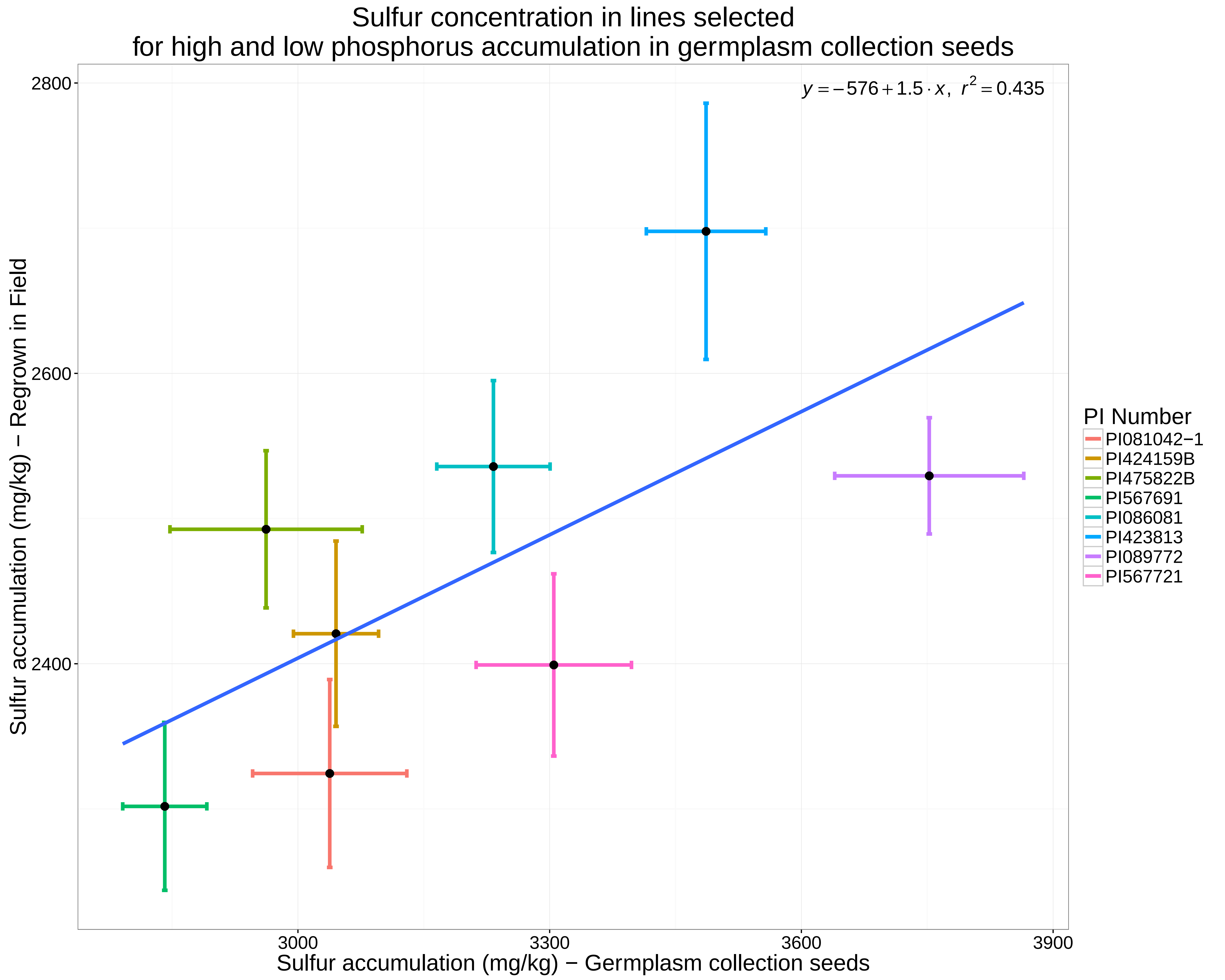

Sulfur concentration in lines selected
for high and low phosphorus accumulation in germplasm collection seeds
$y = -576 + 1.5 \cdot x,\ r^2 = 0.435$
2800
2600
2400
3000
3300
3600
3900
Sulfur accumulation (mg/kg) − Germplasm collection seeds
Sulfur accumulation (mg/kg) − Regrown in Field
PI Number
PI081042−1
PI424159B
PI475822B
PI567691
PI086081
PI423813
PI089772
PI567721

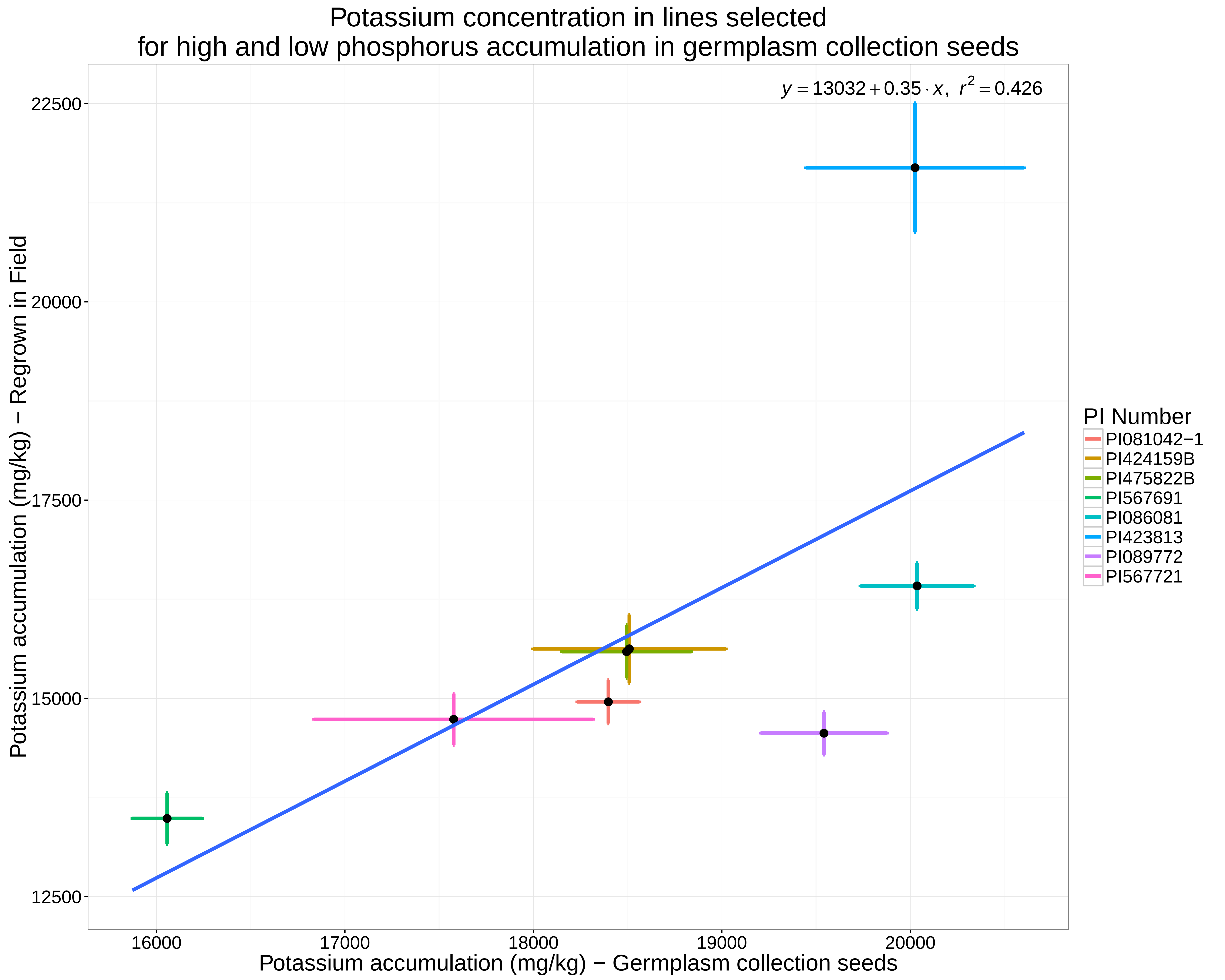
Potassium concentration in lines selected
for high and low phosphorus accumulation in germplasm collection seeds
$y = 13032 + 0.35 \cdot x,\ r^2 = 0.426$
Potassium accumulation (mg/kg) − Regrown in Field
Potassium accumulation (mg/kg) − Germplasm collection seeds
22500
20000
17500
15000
12500
16000
17000
18000
19000
20000
PI Number
PI081042−1
PI424159B
PI475822B
PI567691
PI086081
PI423813
PI089772
PI567721

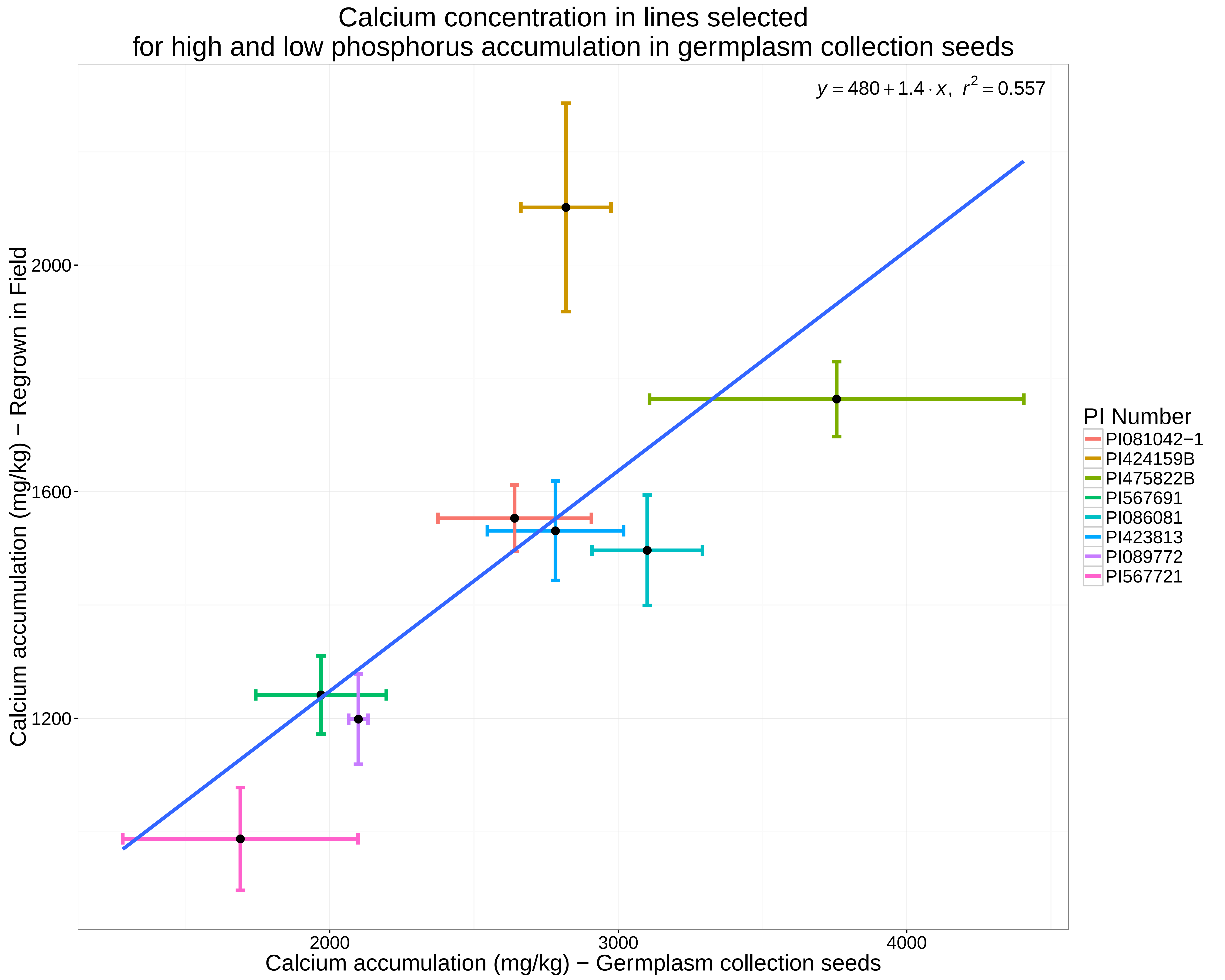

Calcium concentration in lines selected
for high and low phosphorus accumulation in germplasm collection seeds
y = 480 + 1.4 · x, r^2 = 0.557
Calcium accumulation (mg/kg) − Regrown in Field
2000
1600
1200
Calcium accumulation (mg/kg) − Germplasm collection seeds
2000
3000
4000
PI Number
PI081042−1
PI424159B
PI475822B
PI567691
PI086081
PI423813
PI089772
PI567721

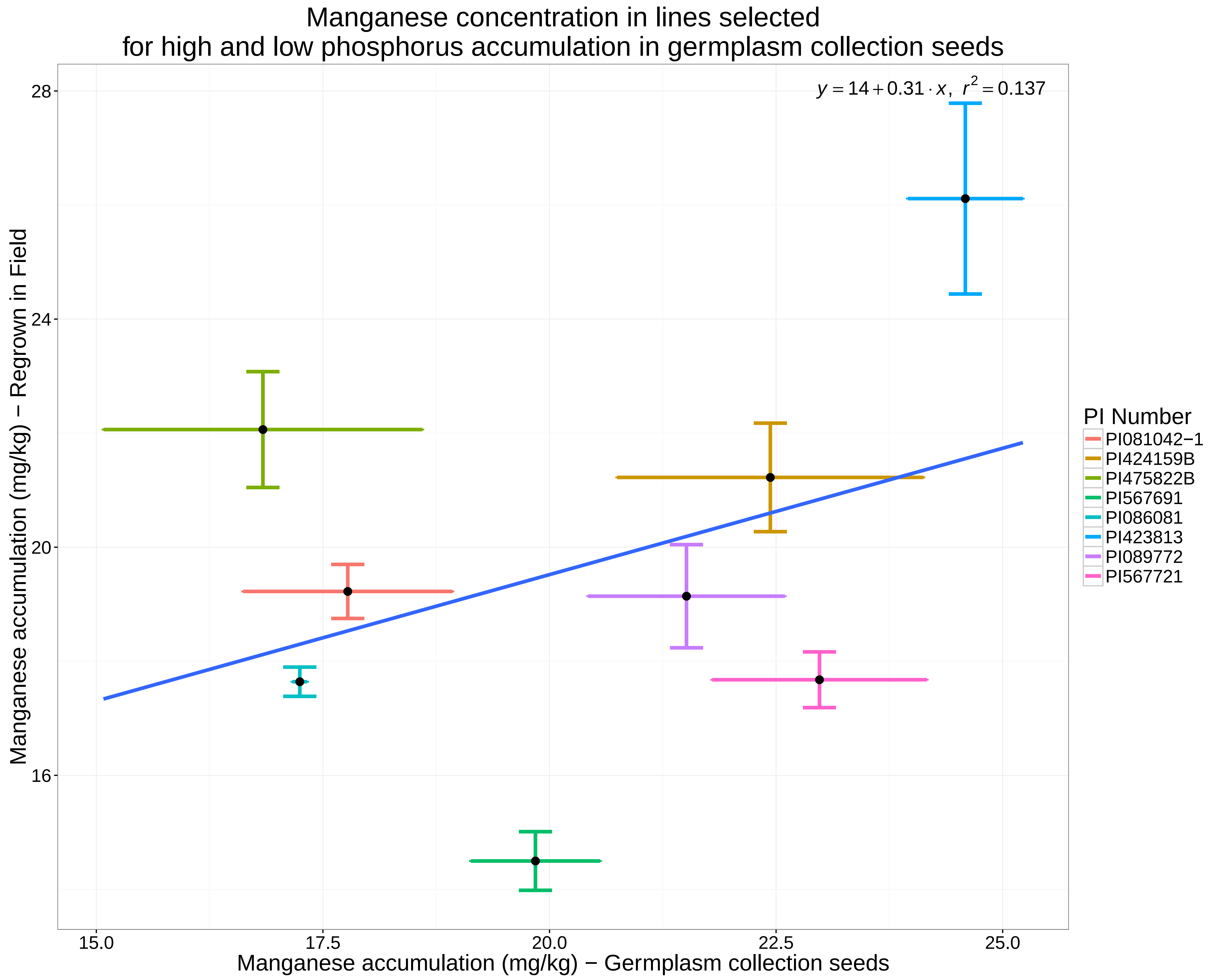
Manganese concentration in lines selected
for high and low phosphorus accumulation in germplasm collection seeds
y = 14 + 0.31 · x, r^2 = 0.137
28
24
20
16
Manganese accumulation (mg/kg) − Regrown in Field
15.0
17.5
20.0
22.5
25.0
Manganese accumulation (mg/kg) − Germplasm collection seeds
PI Number
PI081042−1
PI424159B
PI475822B
PI567691
PI086081
PI423813
PI089772
PI567721

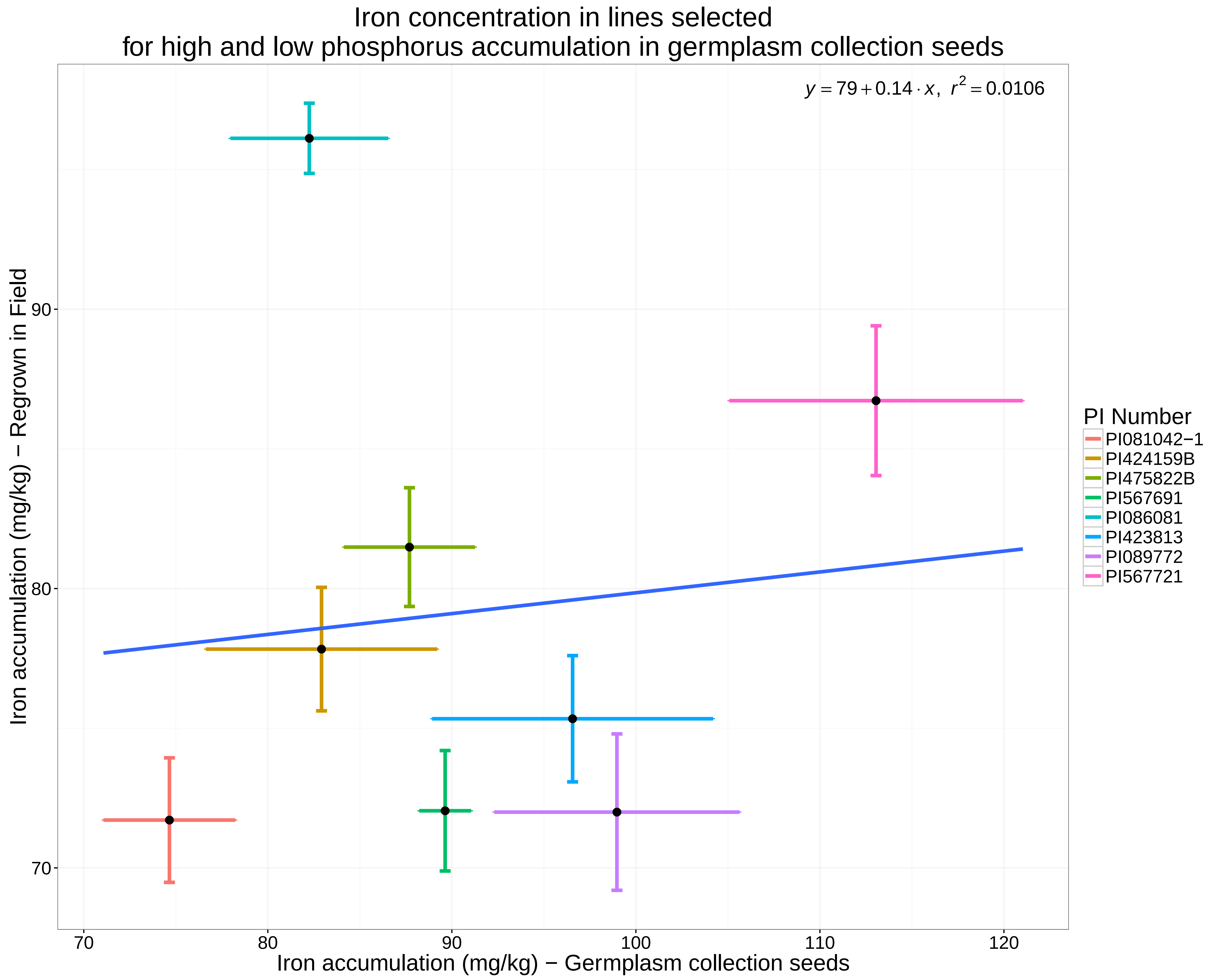
Iron concentration in lines selected
for high and low phosphorus accumulation in germplasm collection seeds
y = 79 + 0.14 · x, r^2 = 0.0106
Iron accumulation (mg/kg) − Regrown in Field
90
80
70
70
80
90
100
110
120
Iron accumulation (mg/kg) − Germplasm collection seeds
PI Number
PI081042−1
PI424159B
PI475822B
PI567691
PI086081
PI423813
PI089772
PI567721

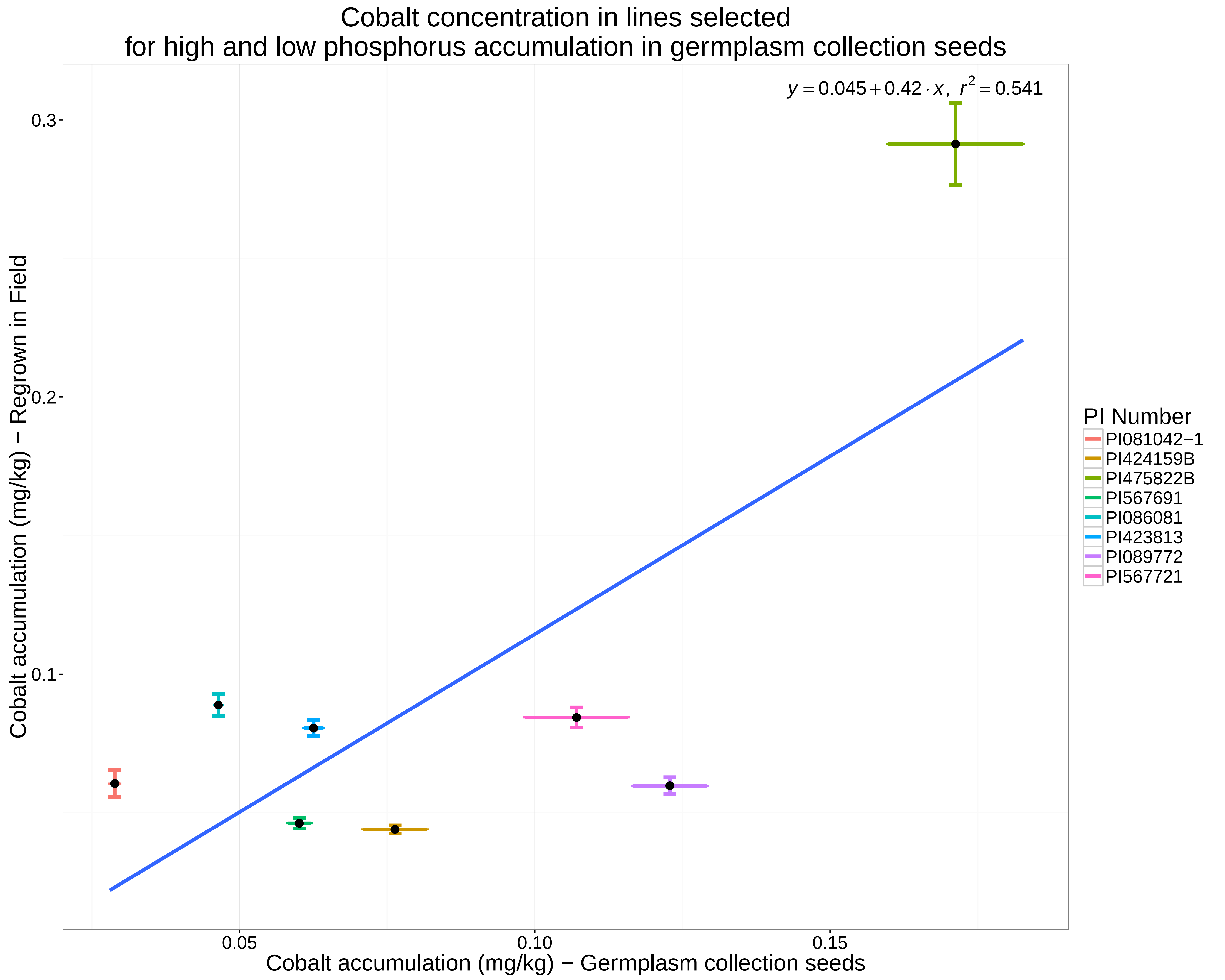

Cobalt concentration in lines selected
for high and low phosphorus accumulation in germplasm collection seeds
y = 0.045 + 0.42 · x, r^2 = 0.541
Cobalt accumulation (mg/kg) − Regrown in Field
0.3
0.2
0.1
Cobalt accumulation (mg/kg) − Germplasm collection seeds
0.05
0.10
0.15
PI Number
PI081042−1
PI424159B
PI475822B
PI567691
PI086081
PI423813
PI089772
PI567721

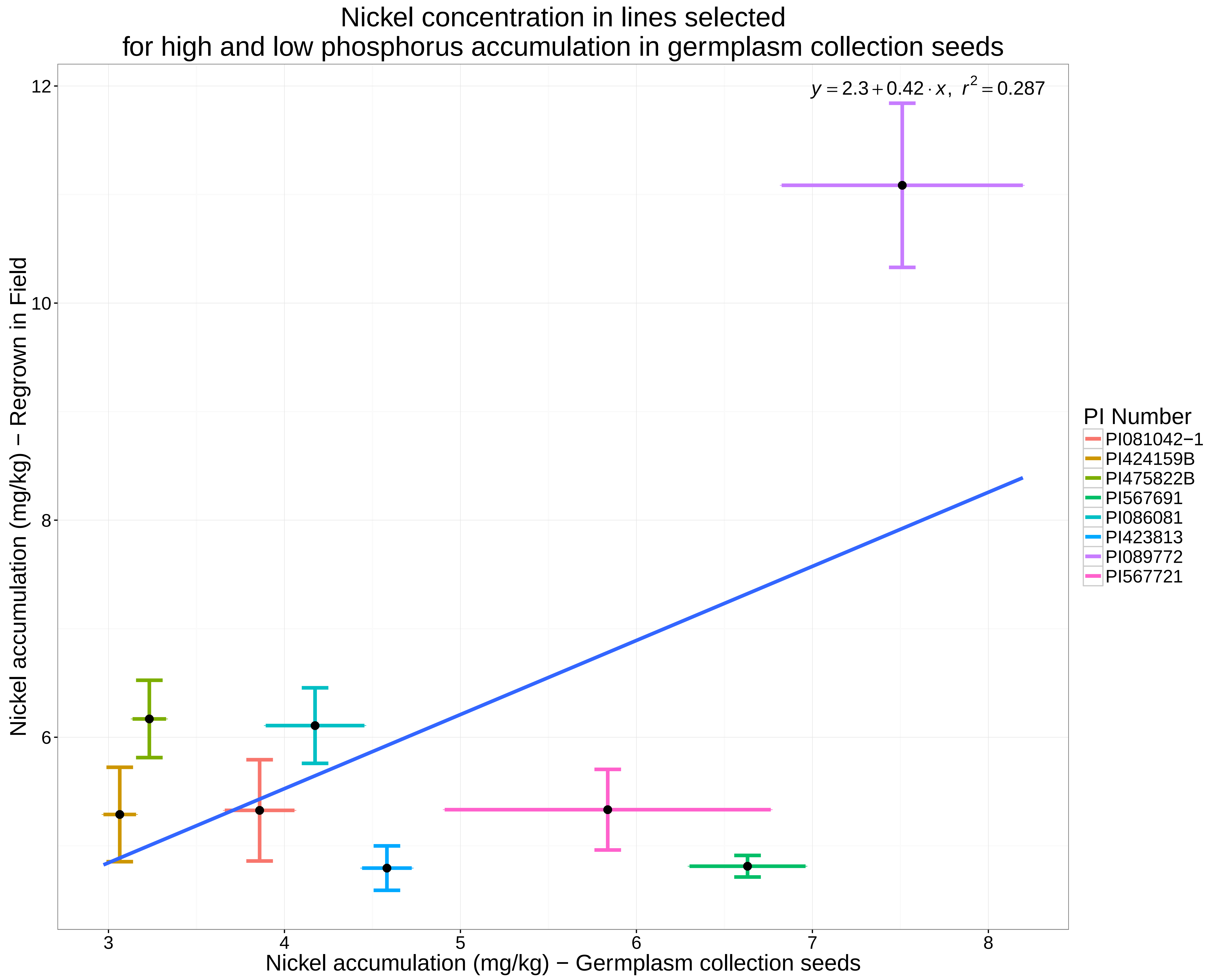

Nickel concentration in lines selected
for high and low phosphorus accumulation in germplasm collection seeds
$y = 2.3 + 0.42 \cdot x,\ r^2 = 0.287$
Nickel accumulation (mg/kg) − Regrown in Field
Nickel accumulation (mg/kg) − Germplasm collection seeds
12
10
8
6
3
4
5
6
7
8
PI Number
PI081042−1
PI424159B
PI475822B
PI567691
PI086081
PI423813
PI089772
PI567721

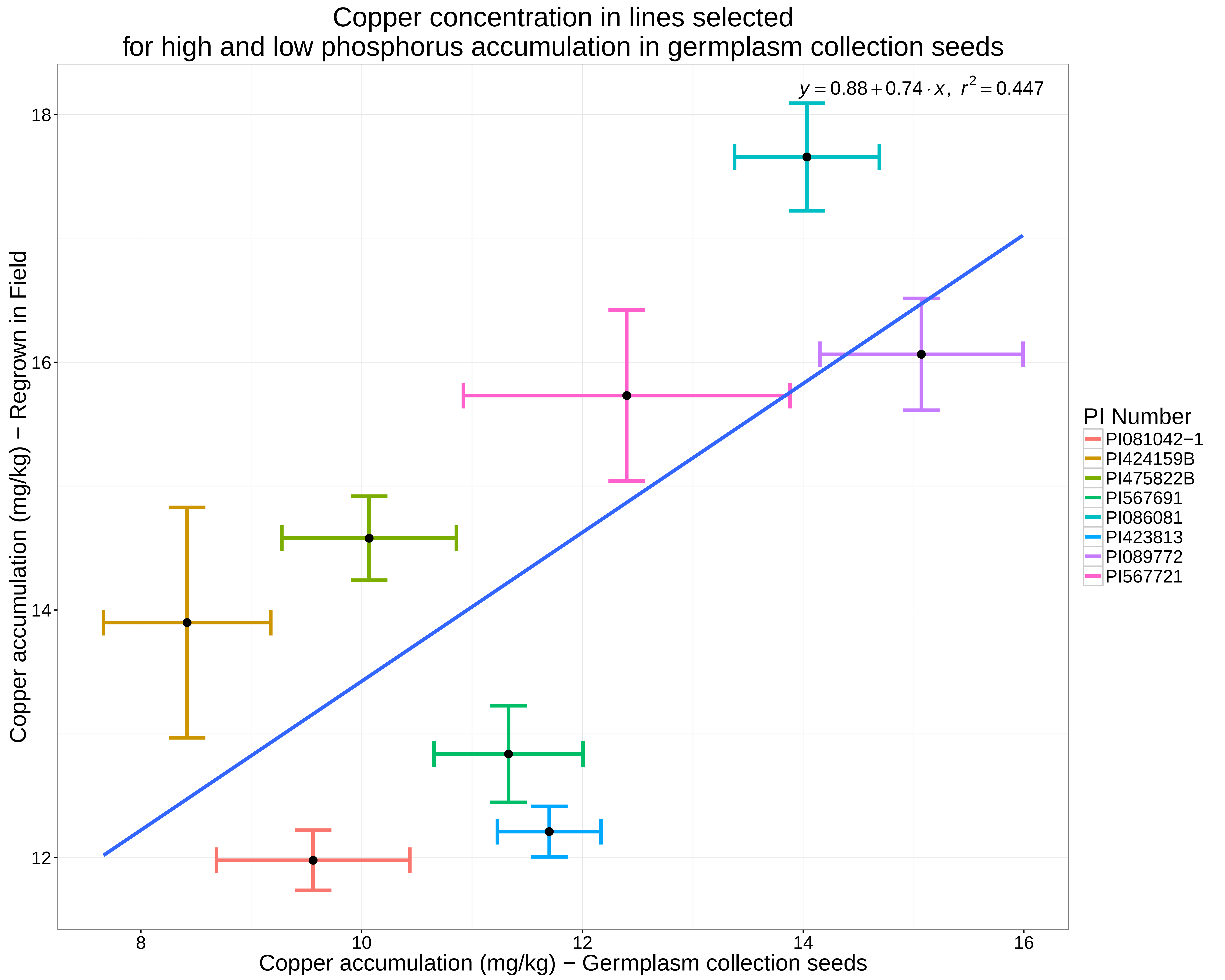

Copper concentration in lines selected
for high and low phosphorus accumulation in germplasm collection seeds
y = 0.88 + 0.74 · x, r² = 0.447
18
16
14
12
8
10
12
14
16
Copper accumulation (mg/kg) − Regrown in Field
Copper accumulation (mg/kg) − Germplasm collection seeds
PI Number
PI081042−1
PI424159B
PI475822B
PI567691
PI086081
PI423813
PI089772
PI567721

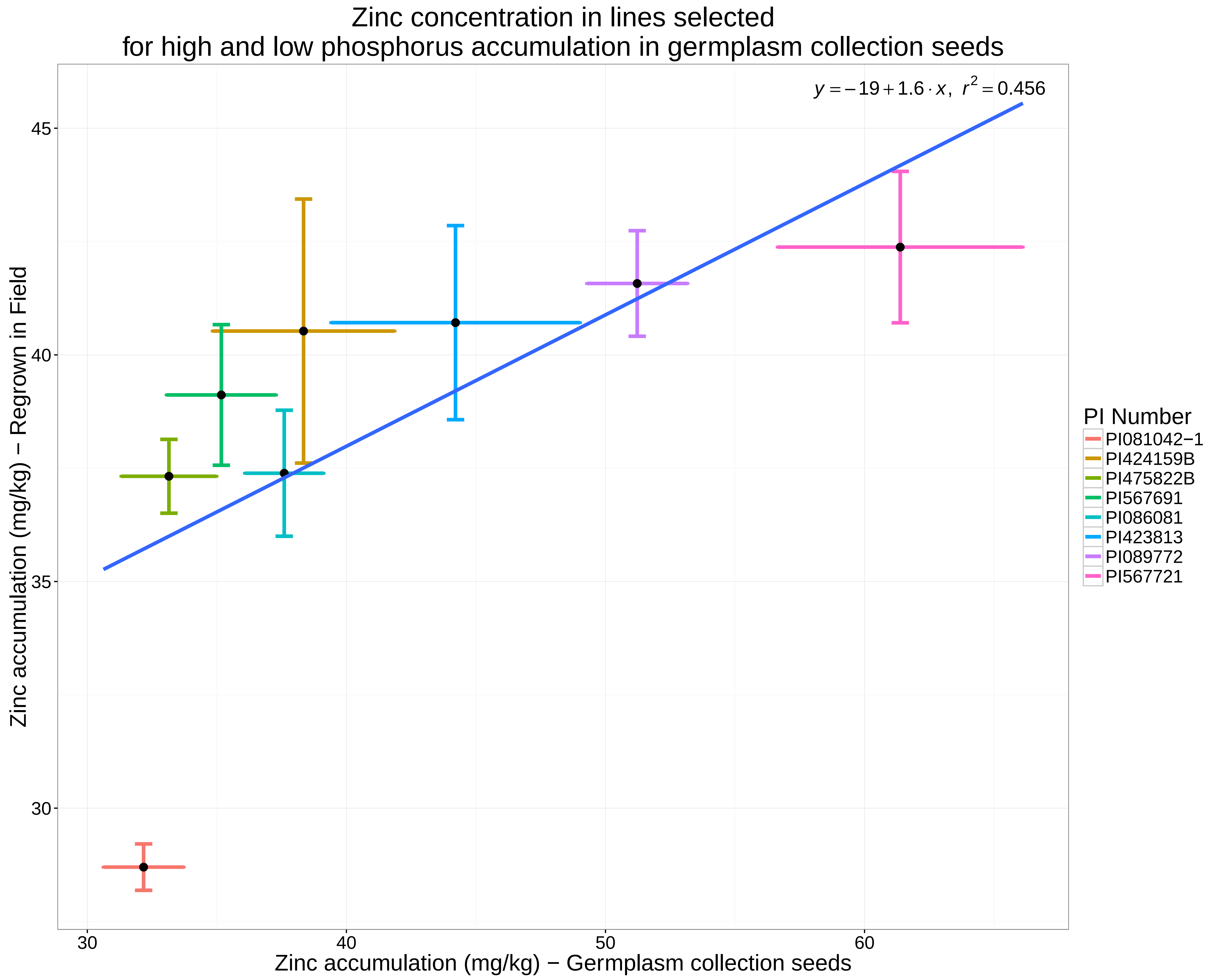

Zinc concentration in lines selected
for high and low phosphorus accumulation in germplasm collection seeds
$y = -19 + 1.6 \cdot x,\ r^2 = 0.456$
Zinc accumulation (mg/kg) − Regrown in Field
Zinc accumulation (mg/kg) − Germplasm collection seeds
45
40
35
30
30
40
50
60
PI Number
PI081042−1
PI424159B
PI475822B
PI567691
PI086081
PI423813
PI089772
PI567721

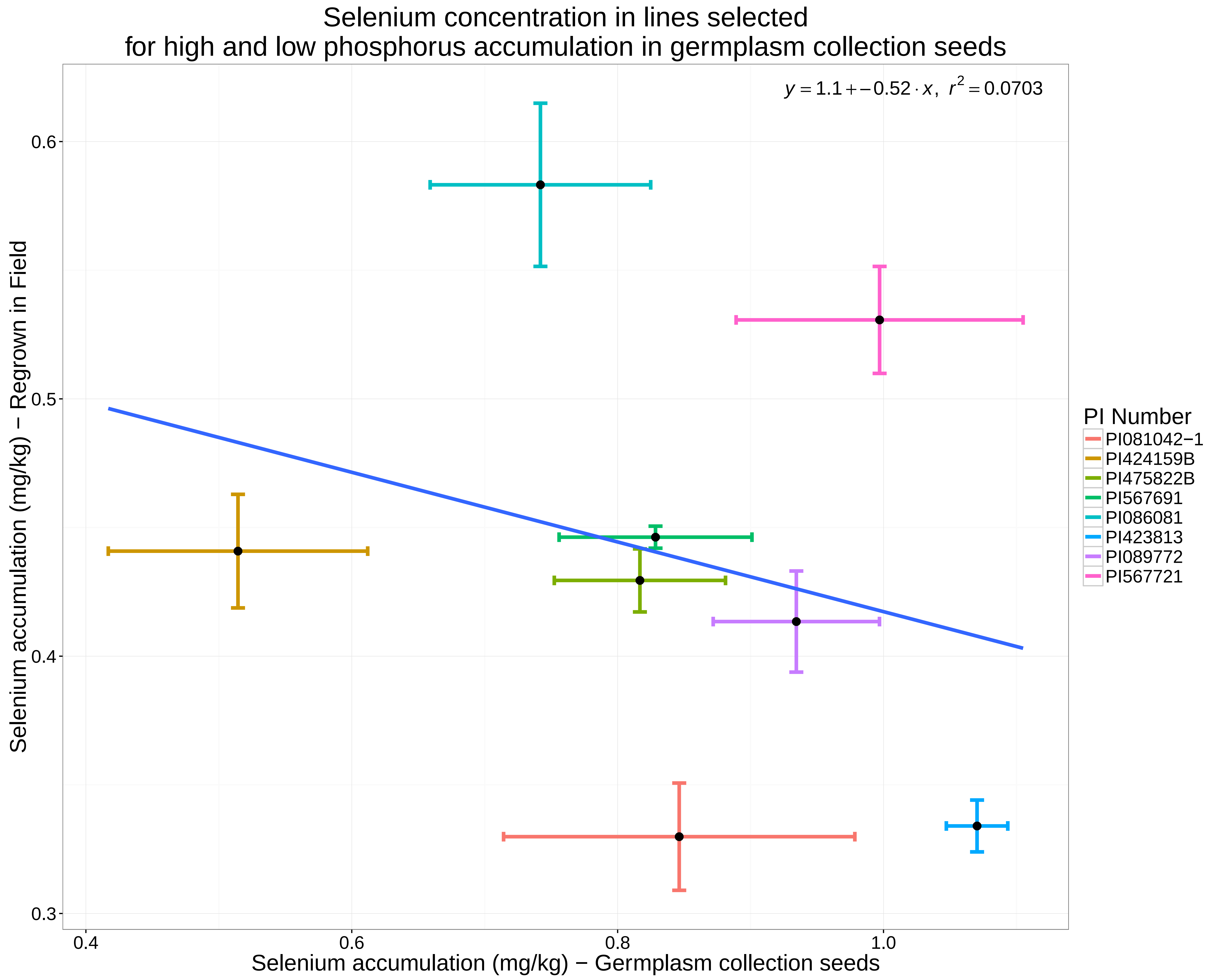

Selenium concentration in lines selected
for high and low phosphorus accumulation in germplasm collection seeds
y = 1.1 + −0.52 · x, r^2 = 0.0703
0.6
0.5
0.4
0.3
0.4
0.6
0.8
1.0
Selenium accumulation (mg/kg) − Regrown in Field
Selenium accumulation (mg/kg) − Germplasm collection seeds
PI Number
PI081042−1
PI424159B
PI475822B
PI567691
PI086081
PI423813
PI089772
PI567721

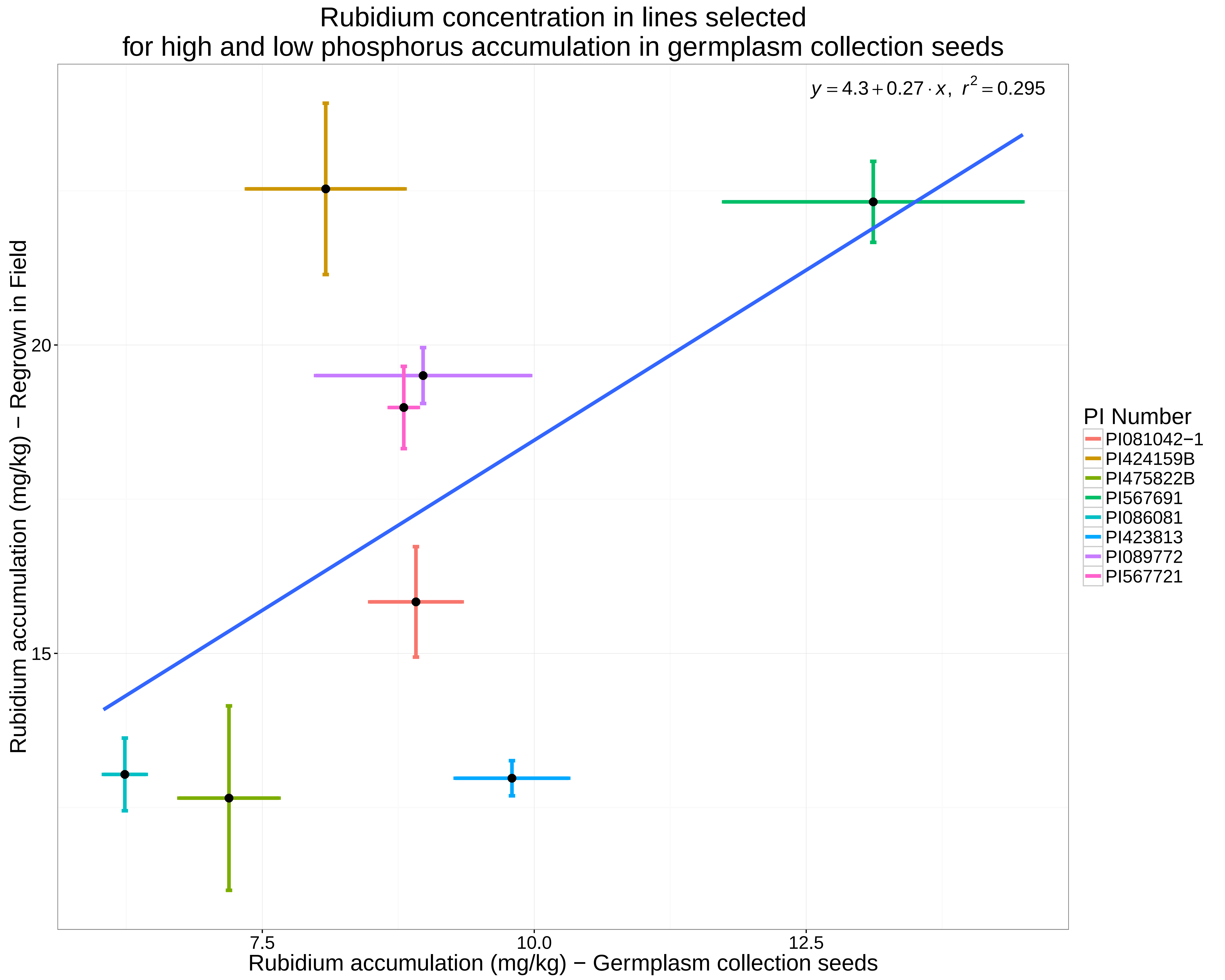
Rubidium concentration in lines selected
for high and low phosphorus accumulation in germplasm collection seeds
$y = 4.3 + 0.27 \cdot x,\ r^2 = 0.295$
20
15
7.5
10.0
12.5
Rubidium accumulation (mg/kg) − Germplasm collection seeds
Rubidium accumulation (mg/kg) − Regrown in Field
PI Number
PI081042−1
PI424159B
PI475822B
PI567691
PI086081
PI423813
PI089772
PI567721

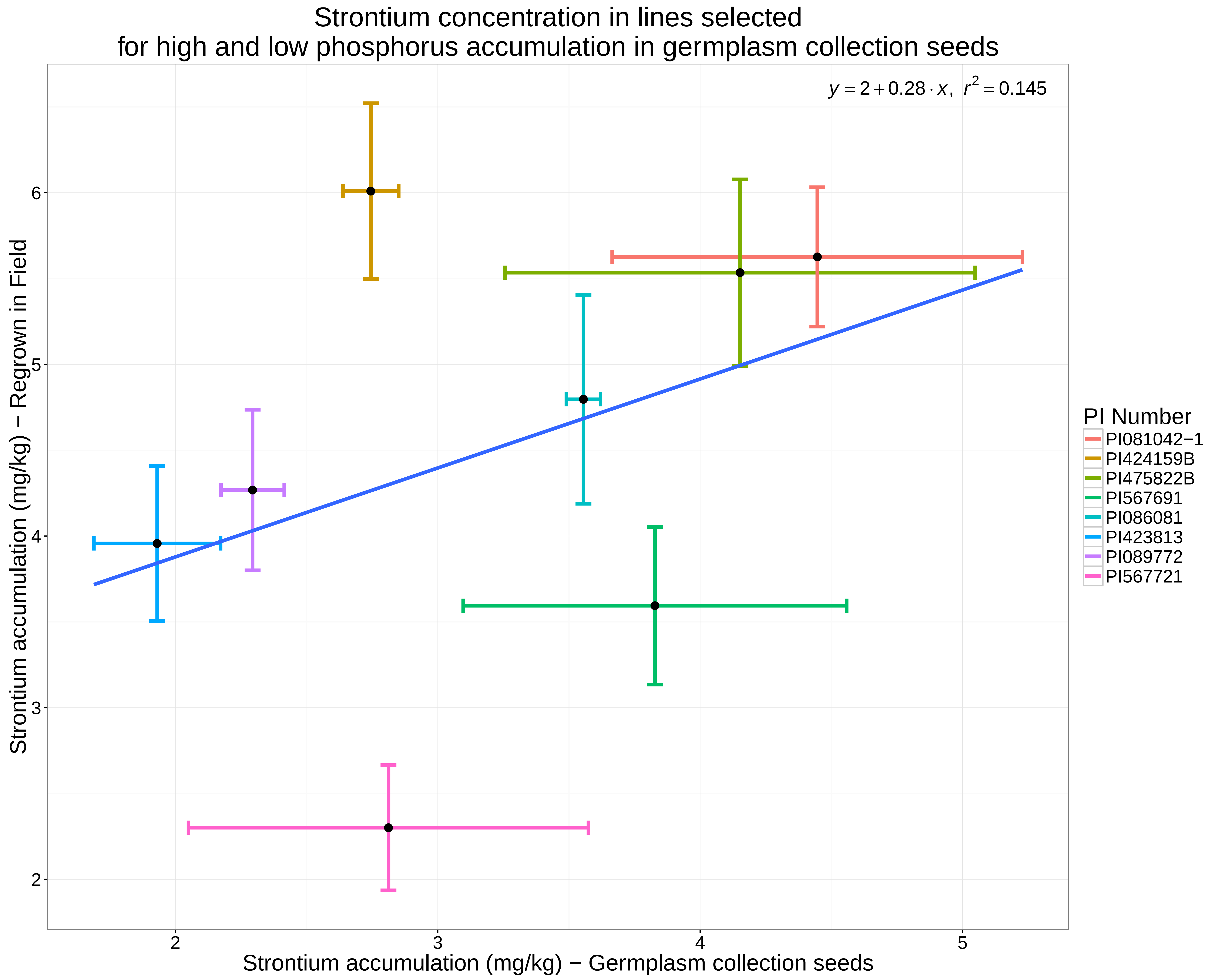

Strontium concentration in lines selected
for high and low phosphorus accumulation in germplasm collection seeds
$y = 2 + 0.28 \cdot x,\ r^2 = 0.145$
Strontium accumulation (mg/kg) − Regrown in Field
Strontium accumulation (mg/kg) − Germplasm collection seeds
6
5
4
3
2
2
3
4
5
PI Number
PI081042−1
PI424159B
PI475822B
PI567691
PI086081
PI423813
PI089772
PI567721

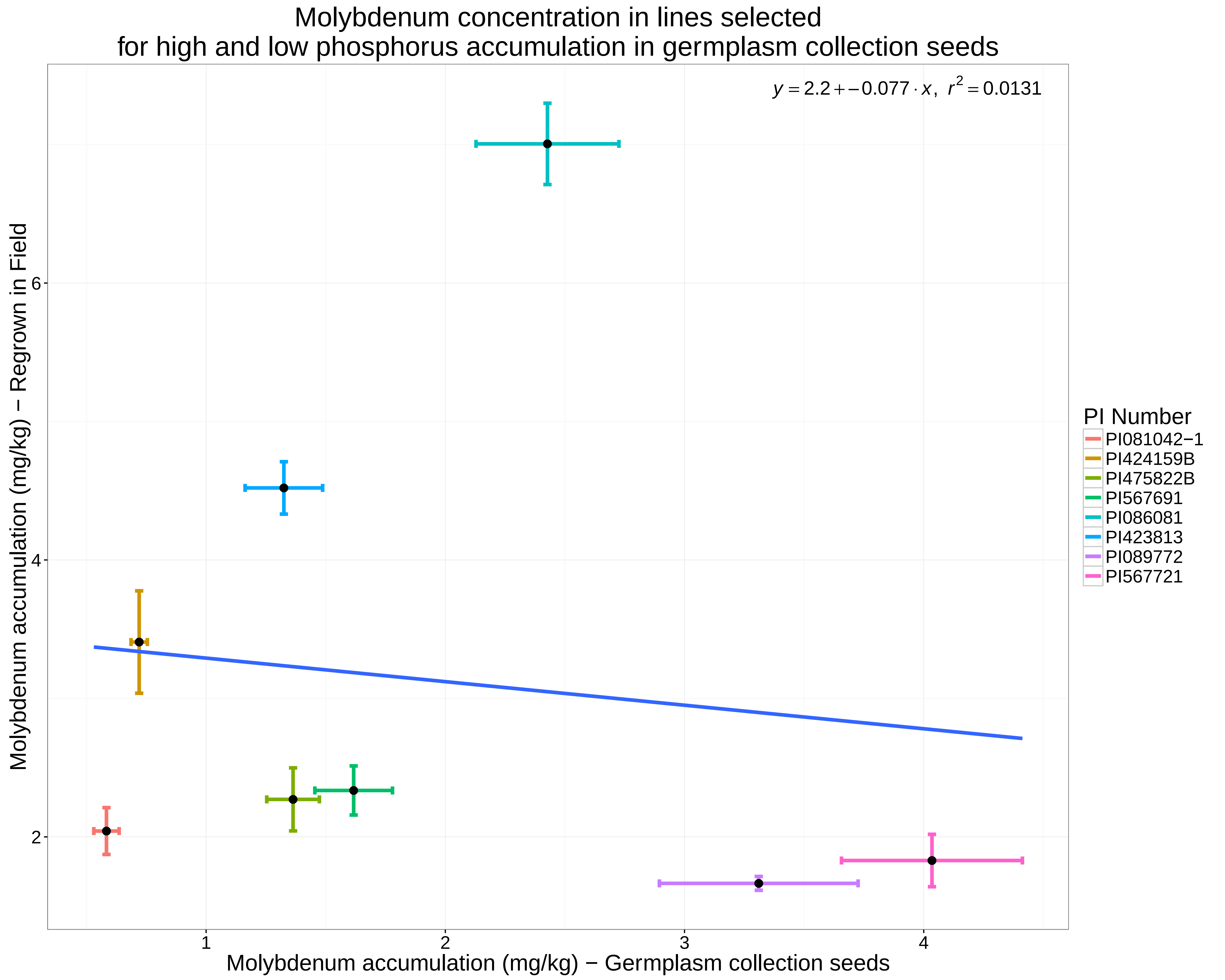

Molybdenum concentration in lines selected
for high and low phosphorus accumulation in germplasm collection seeds
$y = 2.2 + -0.077 \cdot x,\ r^2 = 0.0131$
Molybdenum accumulation (mg/kg) − Regrown in Field
Molybdenum accumulation (mg/kg) − Germplasm collection seeds
2
4
6
1
2
3
4
PI Number
PI081042−1
PI424159B
PI475822B
PI567691
PI086081
PI423813
PI089772
PI567721

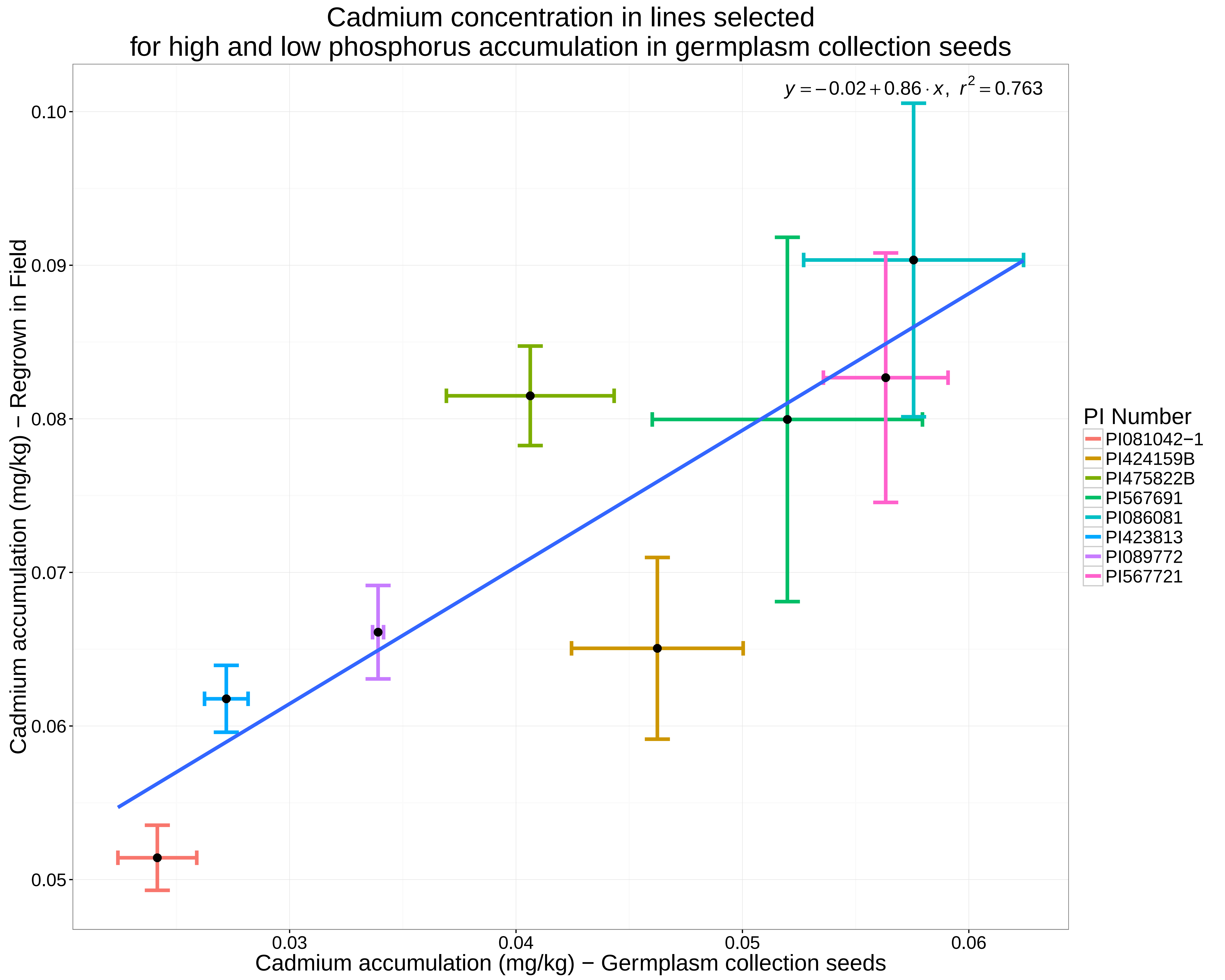

Cadmium concentration in lines selected
for high and low phosphorus accumulation in germplasm collection seeds
$y = -0.02 + 0.86 \cdot x,\ r^2 = 0.763$
Cadmium accumulation (mg/kg) − Regrown in Field
0.10
0.09
0.08
0.07
0.06
0.05
Cadmium accumulation (mg/kg) − Germplasm collection seeds
0.03
0.04
0.05
0.06
PI Number
PI081042−1
PI424159B
PI475822B
PI567691
PI086081
PI423813
PI089772
PI567721
