# Supplementary material for: Genomewide association study of ionomic traits on diverse soybean populations from germplasm collections
Source: Plant Direct. 2018 Jan 15;2(1):e00033. doi: 10.1002/pld3.33 (PMC6508489; doi:10.1002/pld3.33)
Supplement: Supplementary file 6 [file PLD3-2-e00033-s006.pdf]

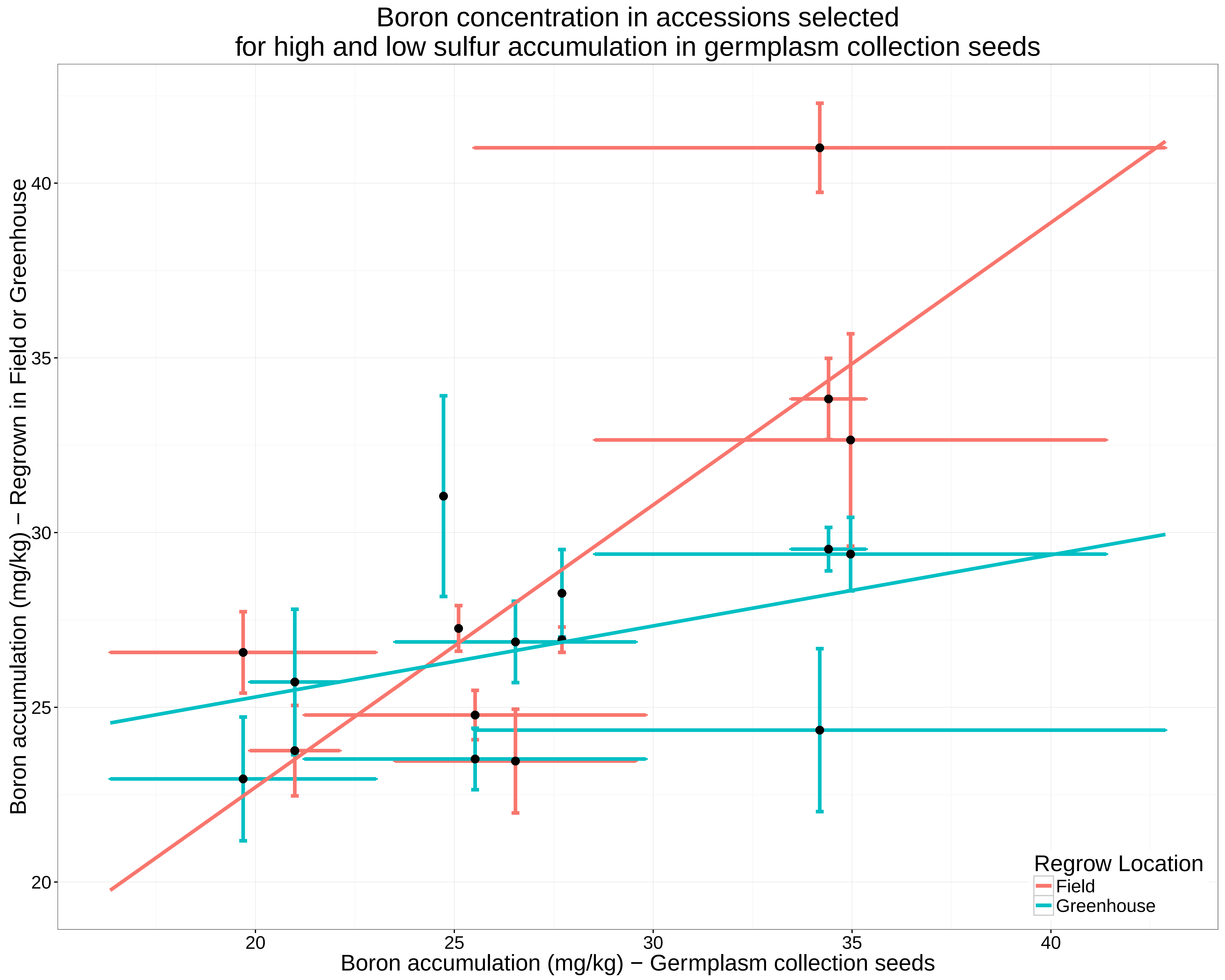

Boron concentration in accessions selected
for high and low sulfur accumulation in germplasm collection seeds
Boron accumulation (mg/kg) − Regrown in Field or Greenhouse
Boron accumulation (mg/kg) − Germplasm collection seeds
20
25
30
35
40
Regrow Location
Field
Greenhouse

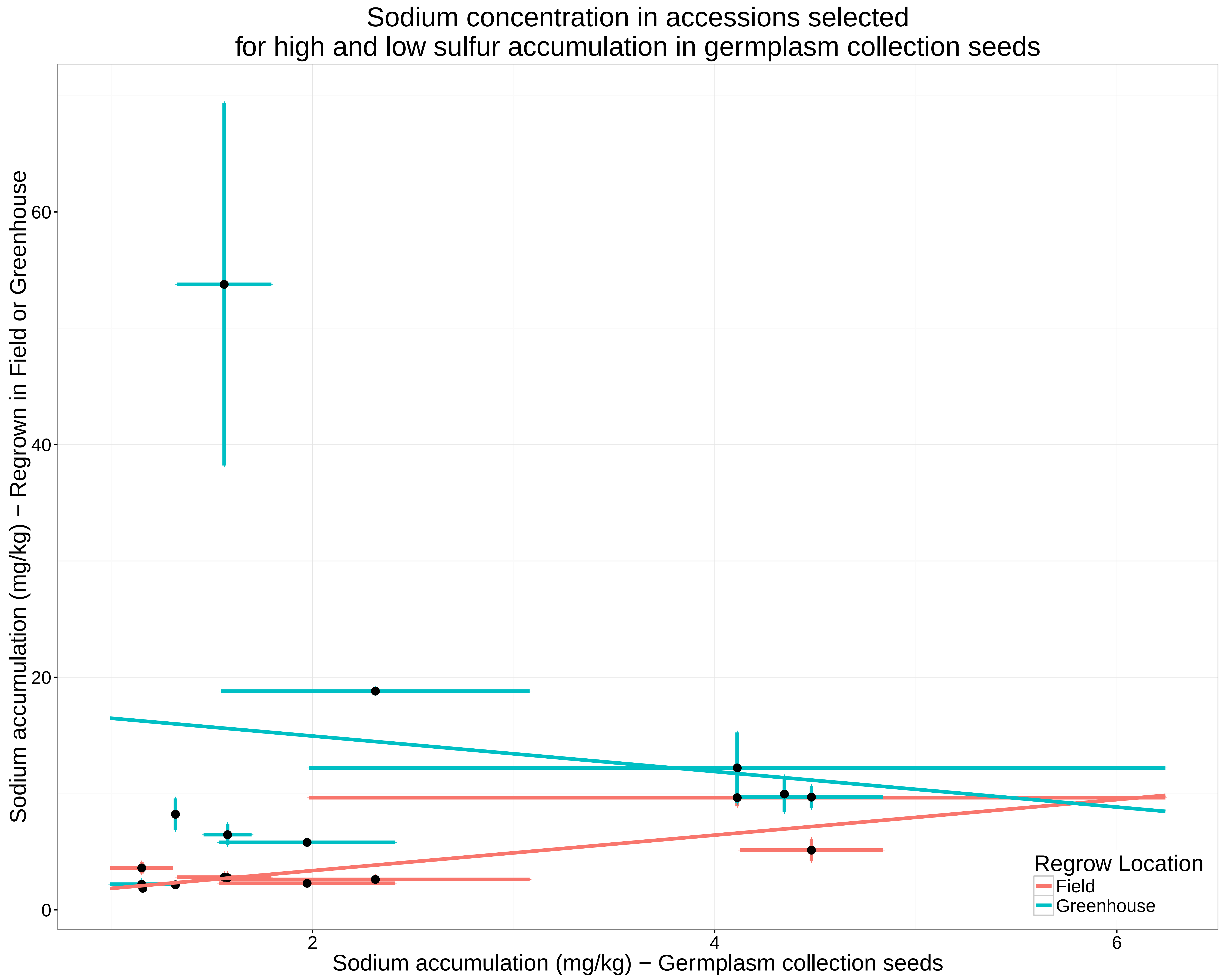
Sodium concentration in accessions selected
for high and low sulfur accumulation in germplasm collection seeds
Sodium accumulation (mg/kg) − Regrown in Field or Greenhouse
Sodium accumulation (mg/kg) − Germplasm collection seeds
0
20
40
60
2
4
6
Regrow Location
Field
Greenhouse

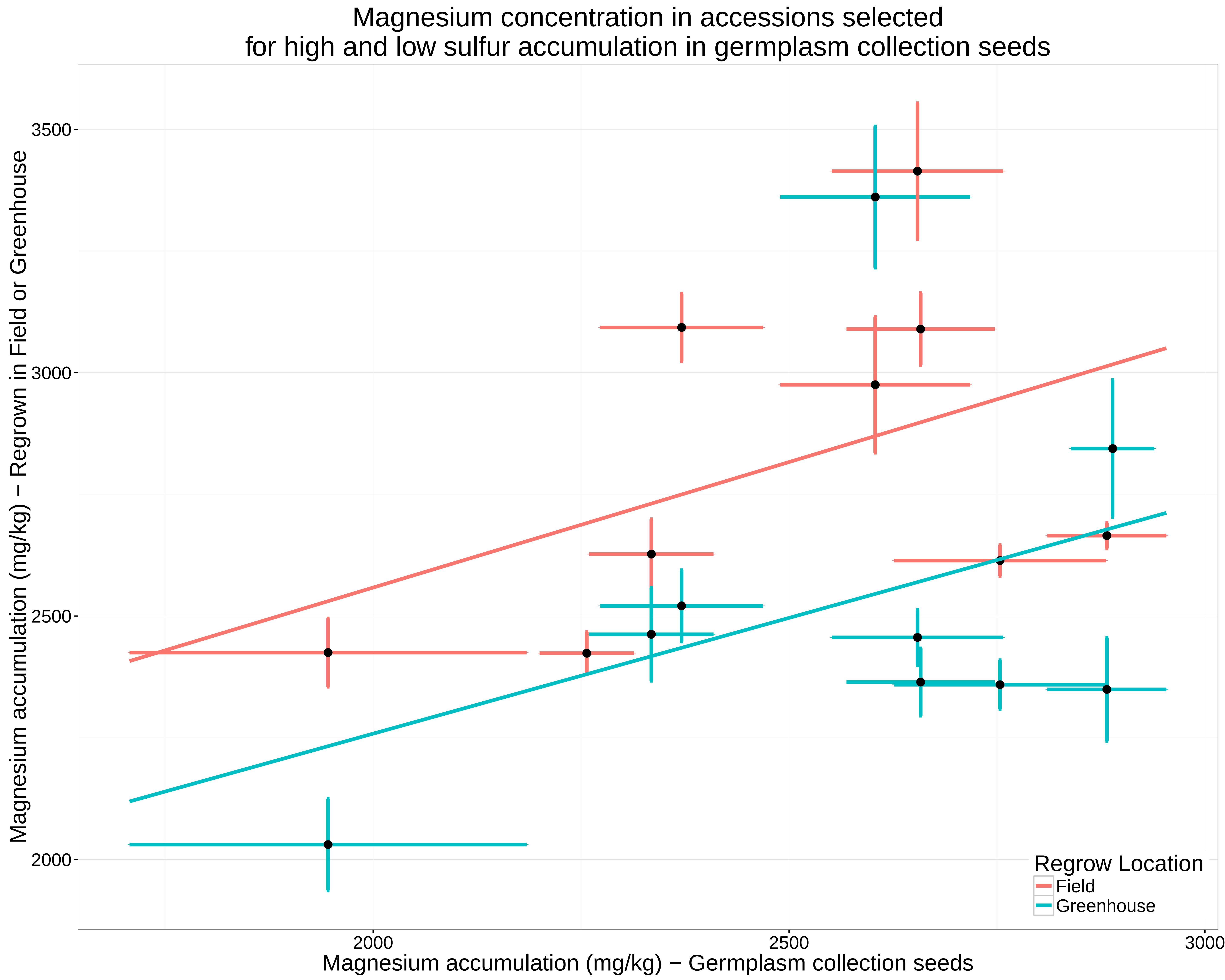

Magnesium concentration in accessions selected
for high and low sulfur accumulation in germplasm collection seeds
Magnesium accumulation (mg/kg) − Regrown in Field or Greenhouse
Magnesium accumulation (mg/kg) − Germplasm collection seeds
3500
3000
2500
2000
2000
2500
3000
Regrow Location
Field
Greenhouse

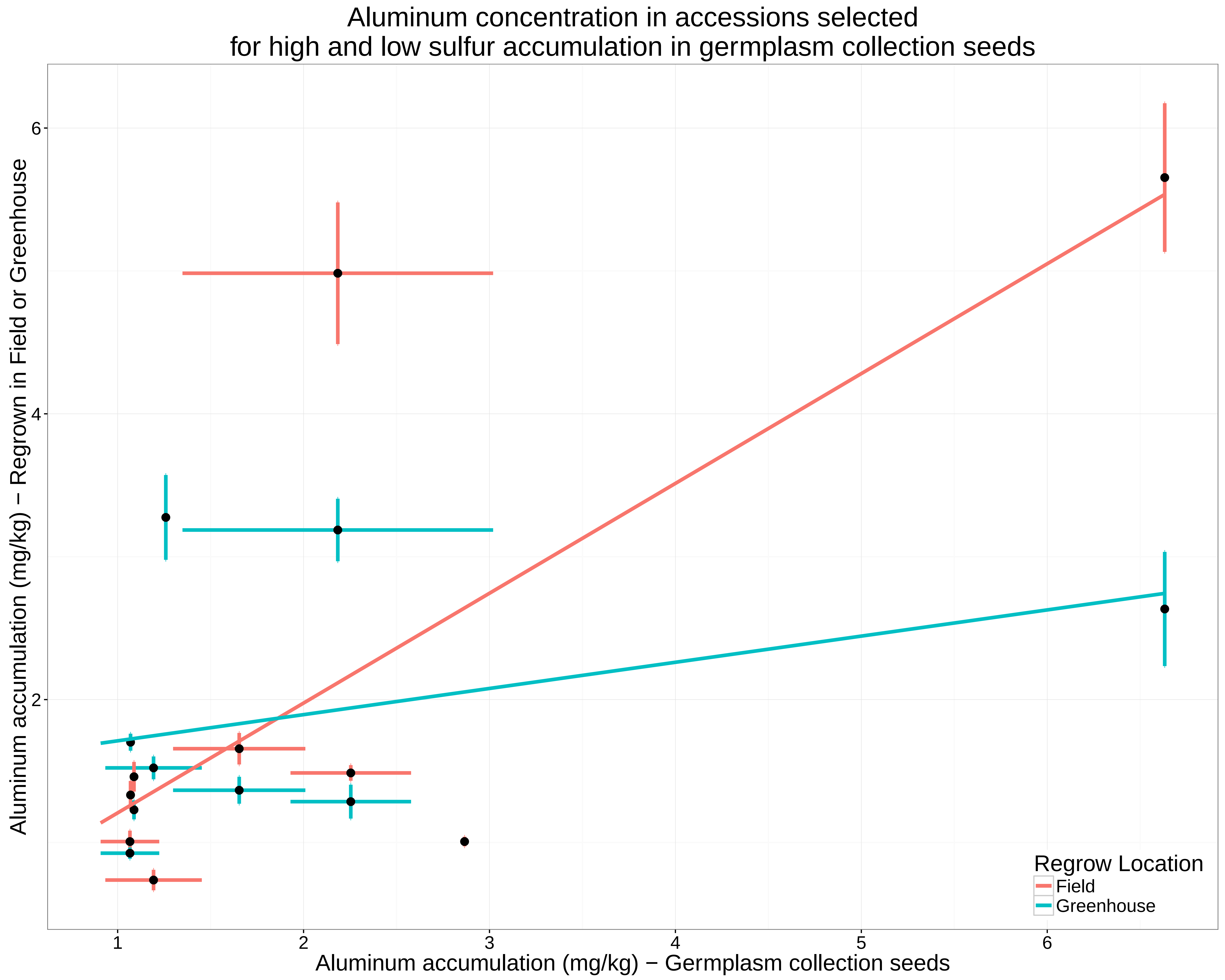
Aluminum concentration in accessions selected
for high and low sulfur accumulation in germplasm collection seeds
Aluminum accumulation (mg/kg) − Regrown in Field or Greenhouse
Aluminum accumulation (mg/kg) − Germplasm collection seeds
6
4
2
1
2
3
4
5
6
Regrow Location
Field
Greenhouse

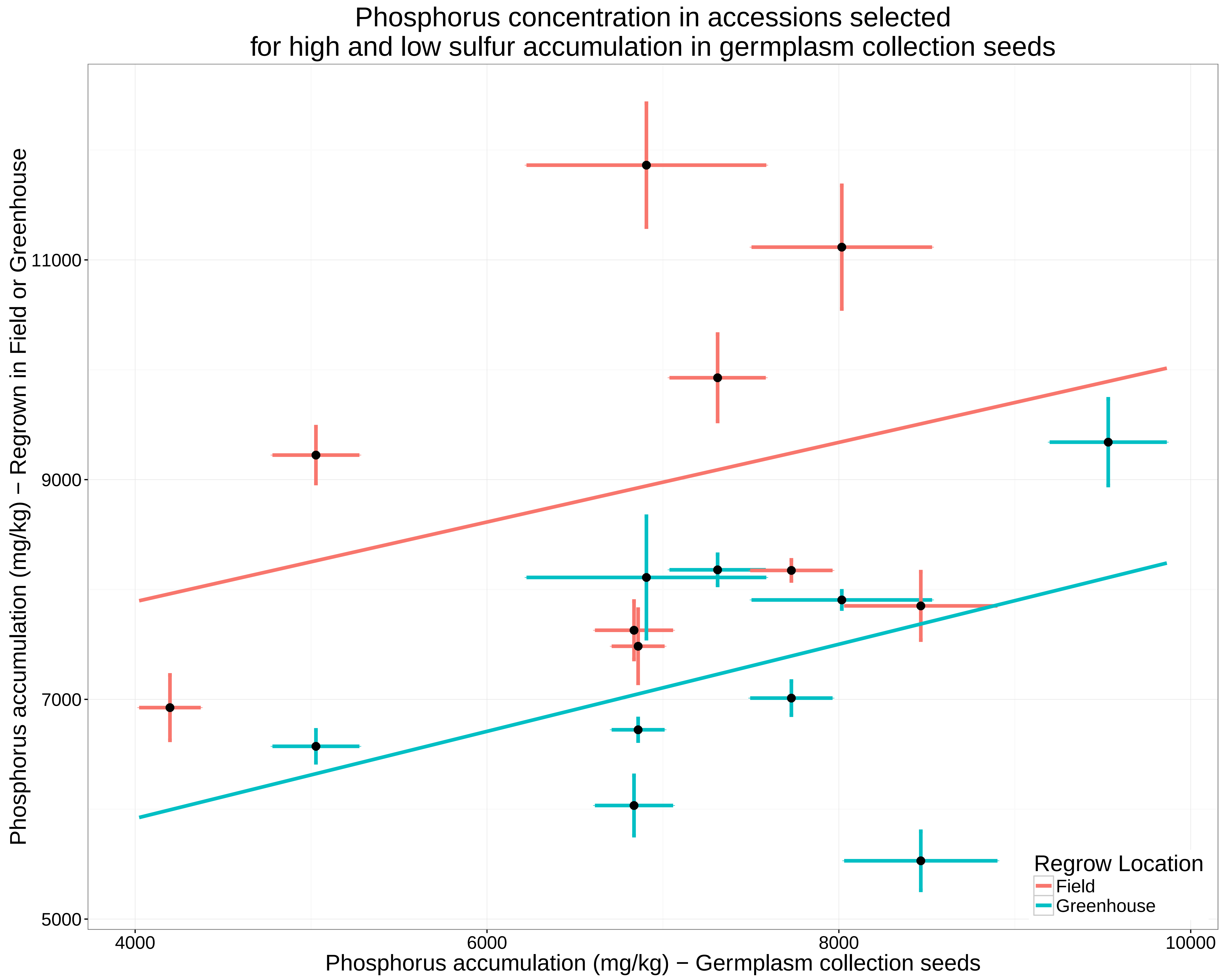

Phosphorus concentration in accessions selected
for high and low sulfur accumulation in germplasm collection seeds
Phosphorus accumulation (mg/kg) − Regrown in Field or Greenhouse
11000
9000
7000
5000
4000
6000
8000
10000
Phosphorus accumulation (mg/kg) − Germplasm collection seeds
Regrow Location
Field
Greenhouse

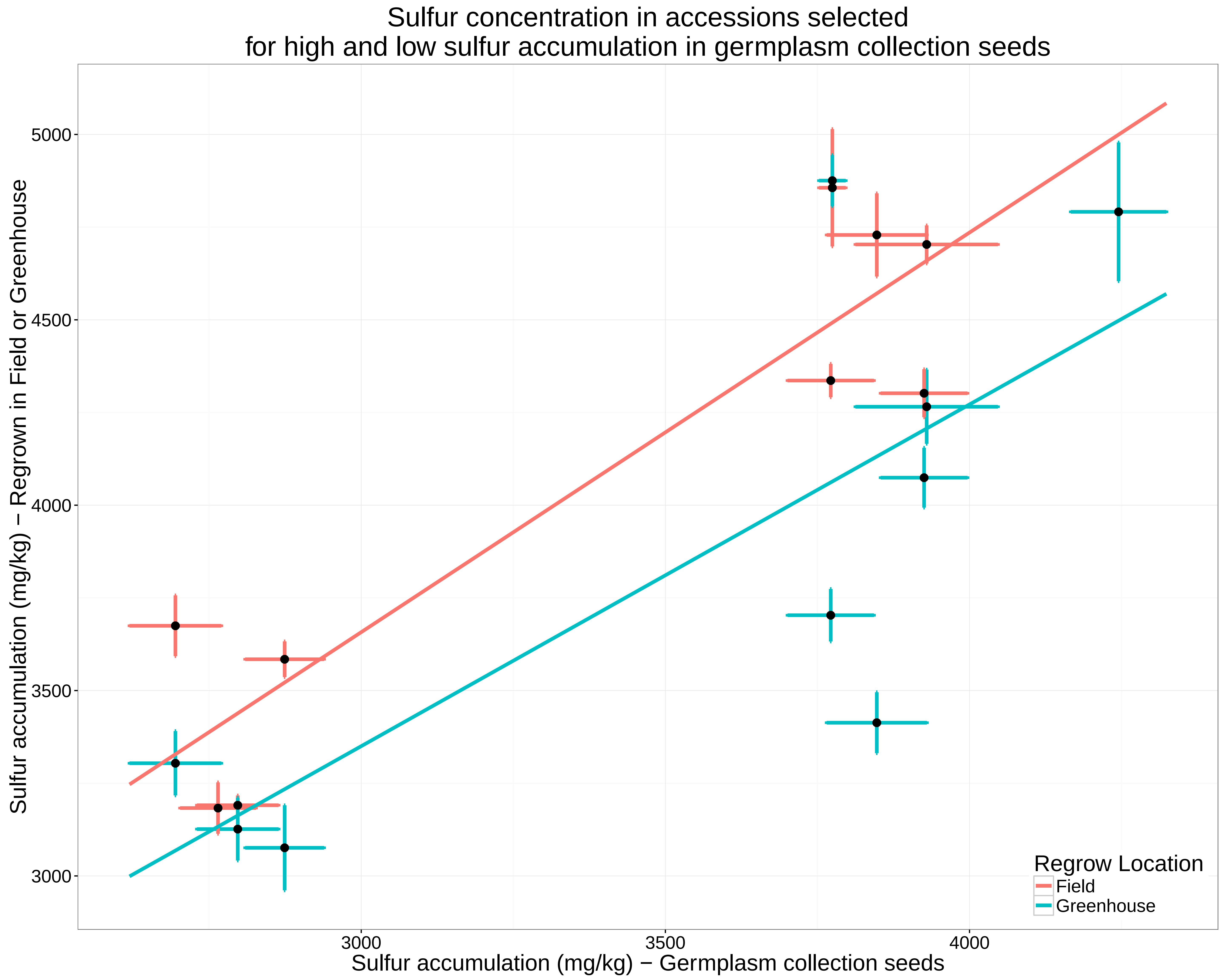

Sulfur concentration in accessions selected
for high and low sulfur accumulation in germplasm collection seeds
Sulfur accumulation (mg/kg) − Regrown in Field or Greenhouse
5000
4500
4000
3500
3000
Sulfur accumulation (mg/kg) − Germplasm collection seeds
3000
3500
4000
Regrow Location
Field
Greenhouse

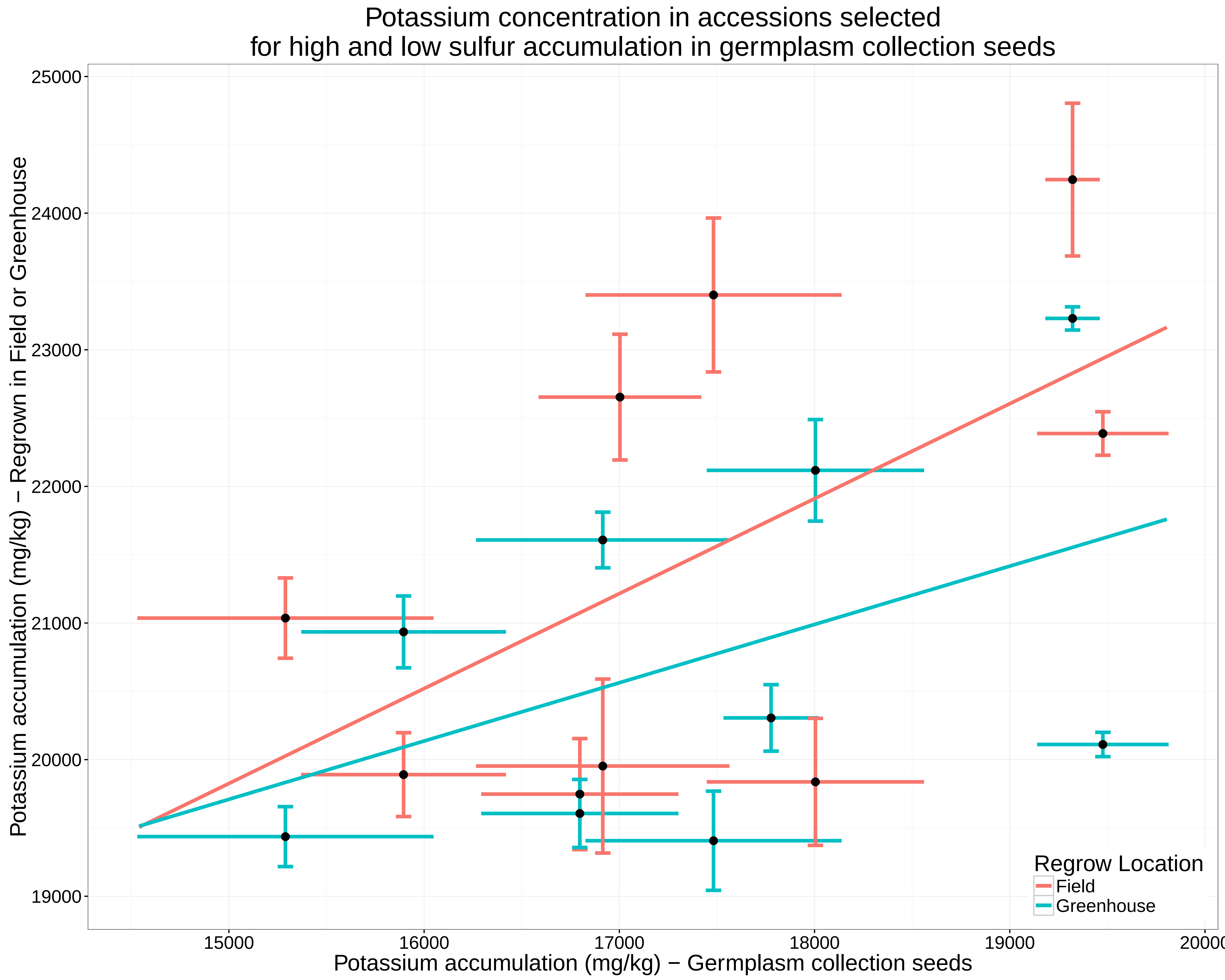

Potassium concentration in accessions selected
for high and low sulfur accumulation in germplasm collection seeds
Potassium accumulation (mg/kg) − Regrown in Field or Greenhouse
Potassium accumulation (mg/kg) − Germplasm collection seeds
25000
24000
23000
22000
21000
20000
19000
15000
16000
17000
18000
19000
20000
Regrow Location
Field
Greenhouse

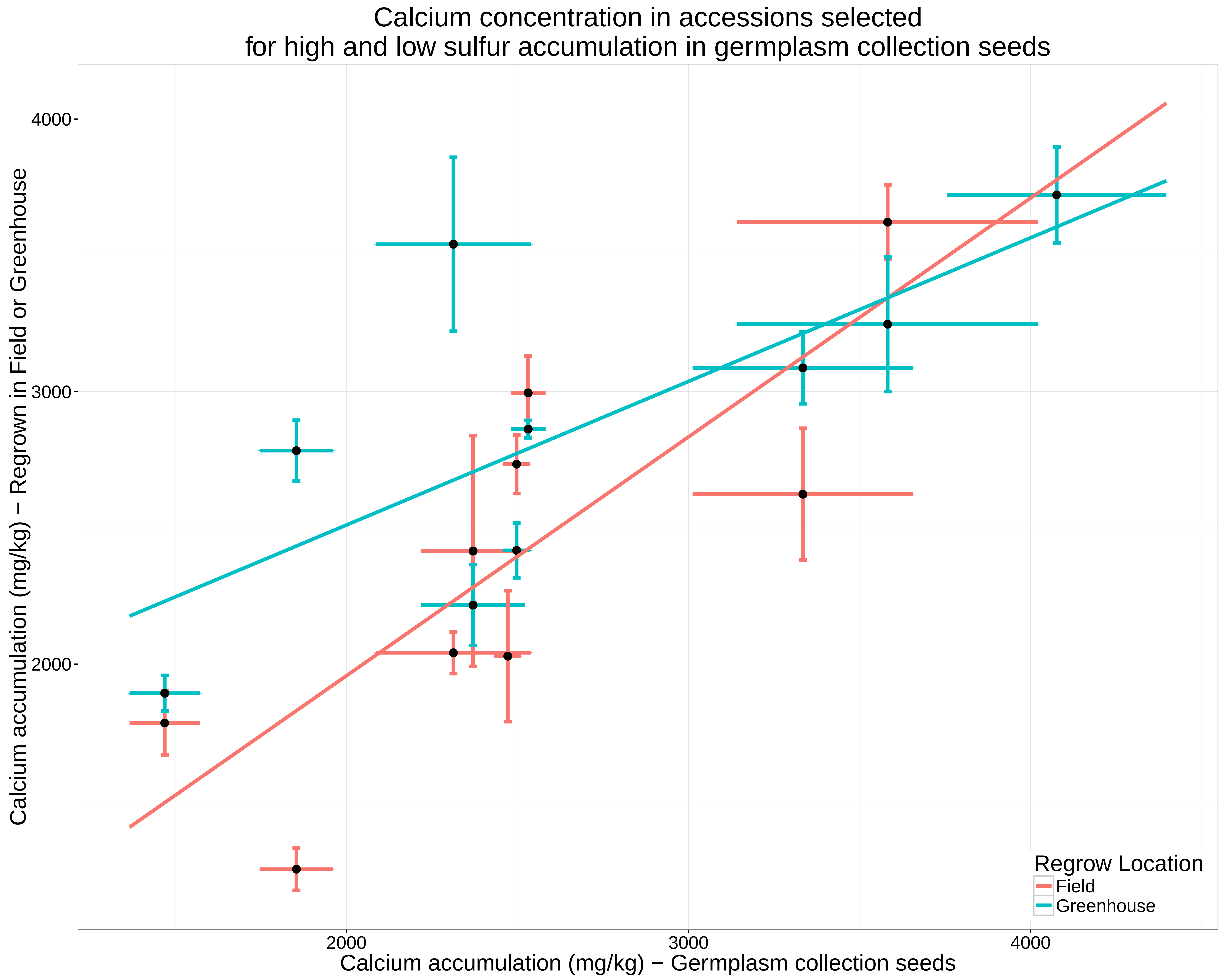
Calcium concentration in accessions selected
for high and low sulfur accumulation in germplasm collection seeds
Calcium accumulation (mg/kg) − Regrown in Field or Greenhouse
4000
3000
2000
2000
3000
4000
Calcium accumulation (mg/kg) − Germplasm collection seeds
Regrow Location
Field
Greenhouse

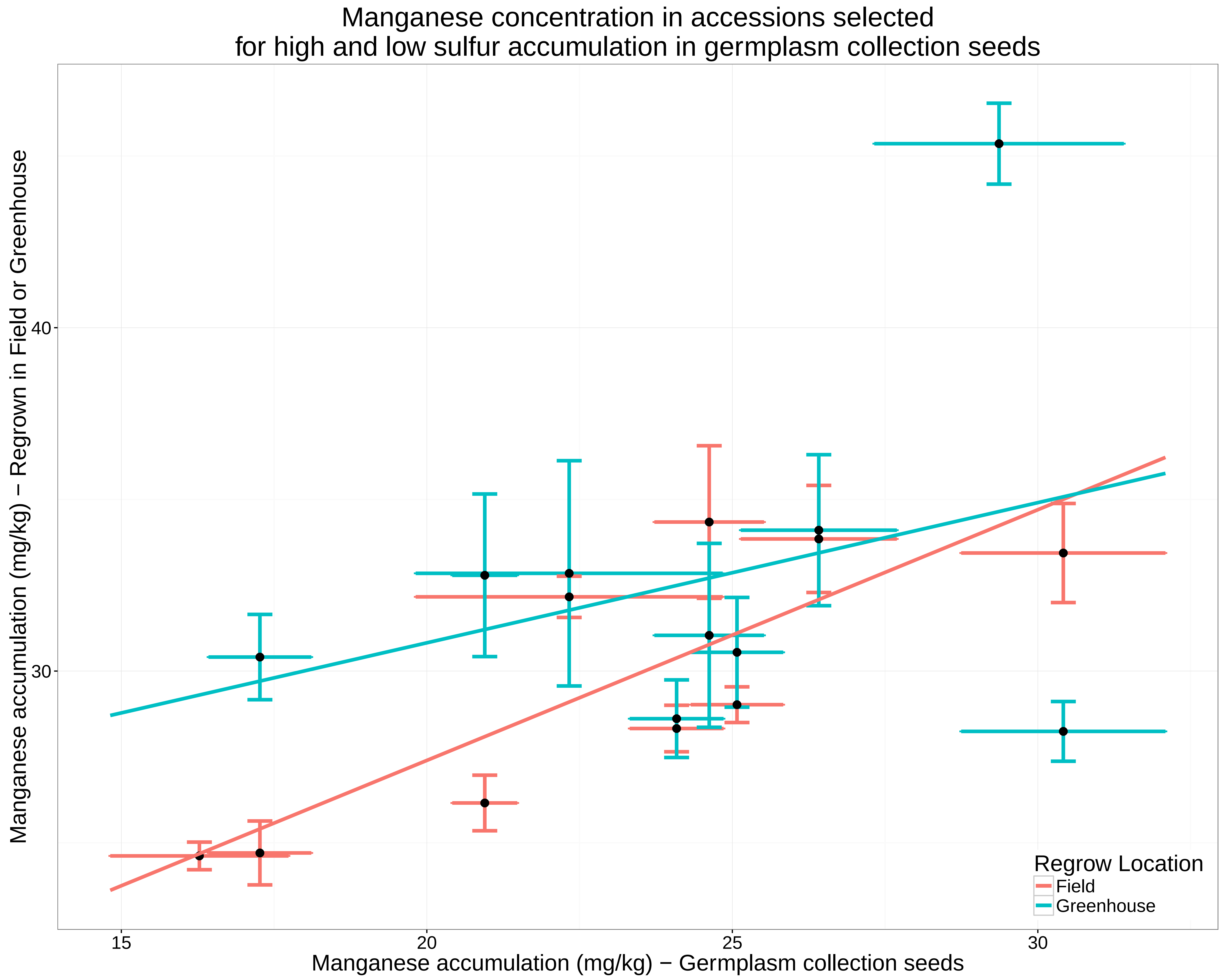
Manganese concentration in accessions selected
for high and low sulfur accumulation in germplasm collection seeds
Manganese accumulation (mg/kg) − Regrown in Field or Greenhouse
Manganese accumulation (mg/kg) − Germplasm collection seeds
40
30
15
20
25
30
Regrow Location
Field
Greenhouse

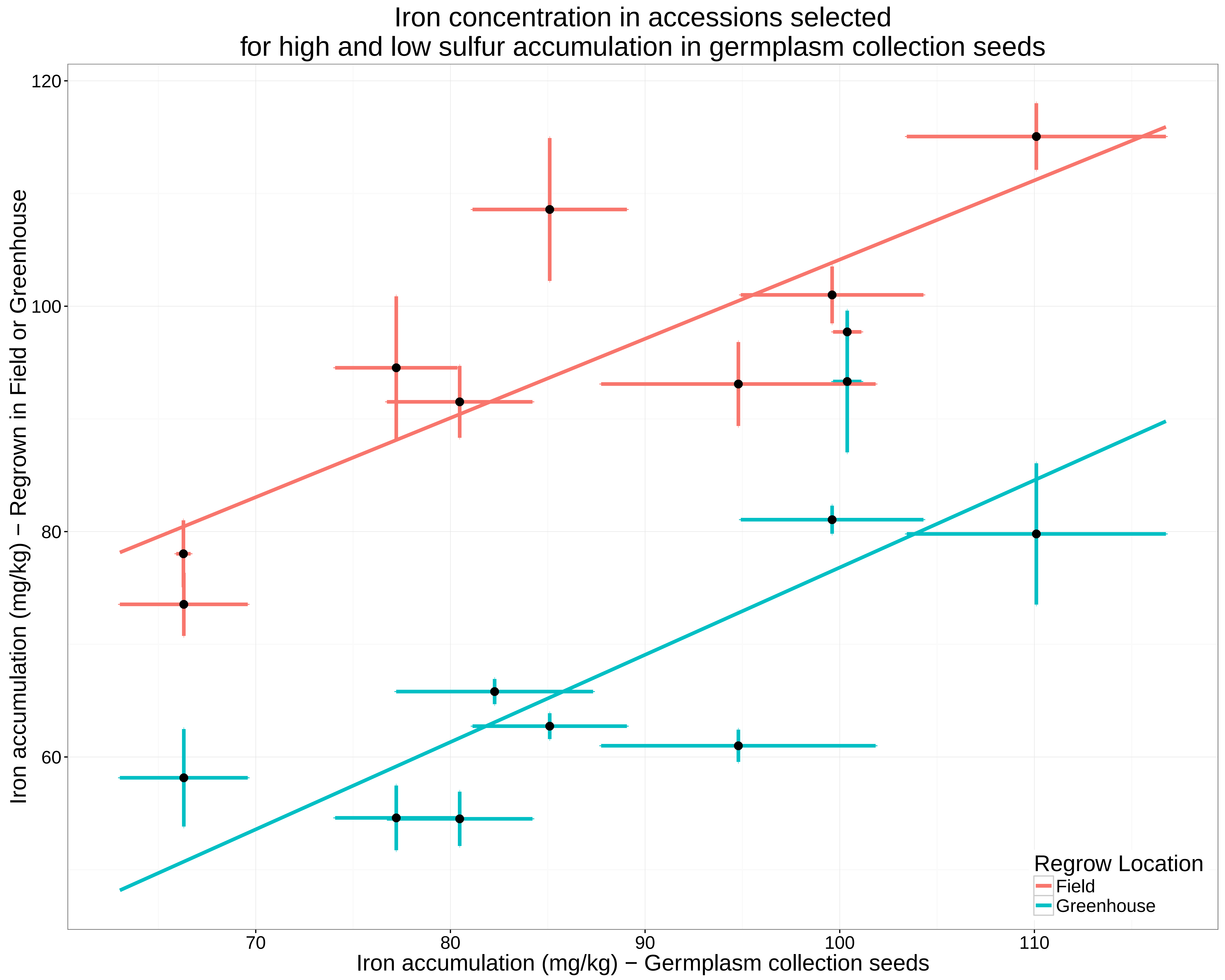

Iron concentration in accessions selected
for high and low sulfur accumulation in germplasm collection seeds
Iron accumulation (mg/kg) − Regrown in Field or Greenhouse
120
100
80
60
70
80
90
100
110
Iron accumulation (mg/kg) − Germplasm collection seeds
Regrow Location
Field
Greenhouse

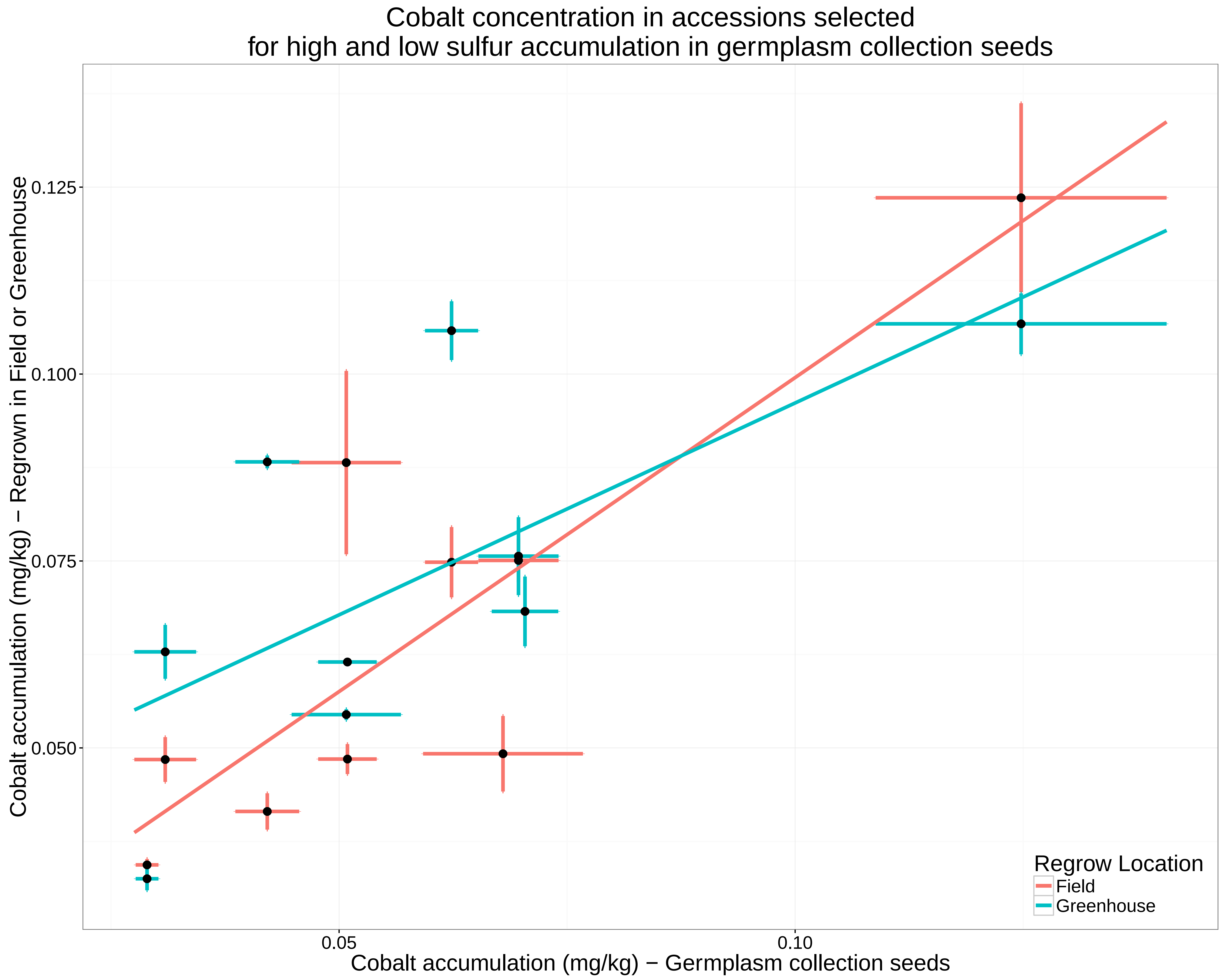
Cobalt concentration in accessions selected
for high and low sulfur accumulation in germplasm collection seeds
Cobalt accumulation (mg/kg) − Regrown in Field or Greenhouse
0.125
0.100
0.075
0.050
0.05
0.10
Cobalt accumulation (mg/kg) − Germplasm collection seeds
Regrow Location
Field
Greenhouse

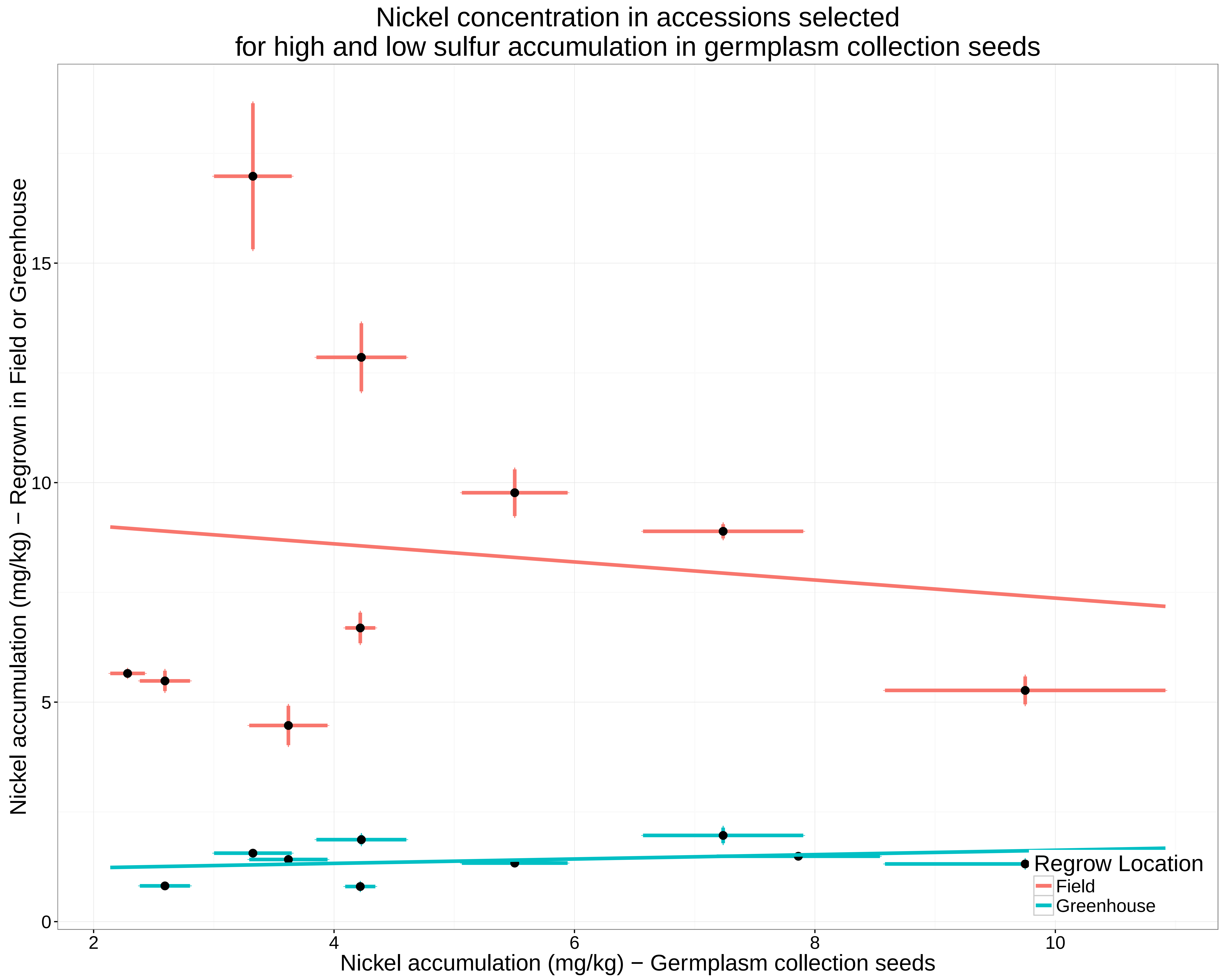

Nickel concentration in accessions selected
for high and low sulfur accumulation in germplasm collection seeds
Nickel accumulation (mg/kg) − Regrown in Field or Greenhouse
Nickel accumulation (mg/kg) − Germplasm collection seeds
0
5
10
15
2
4
6
8
10
Regrow Location
Field
Greenhouse

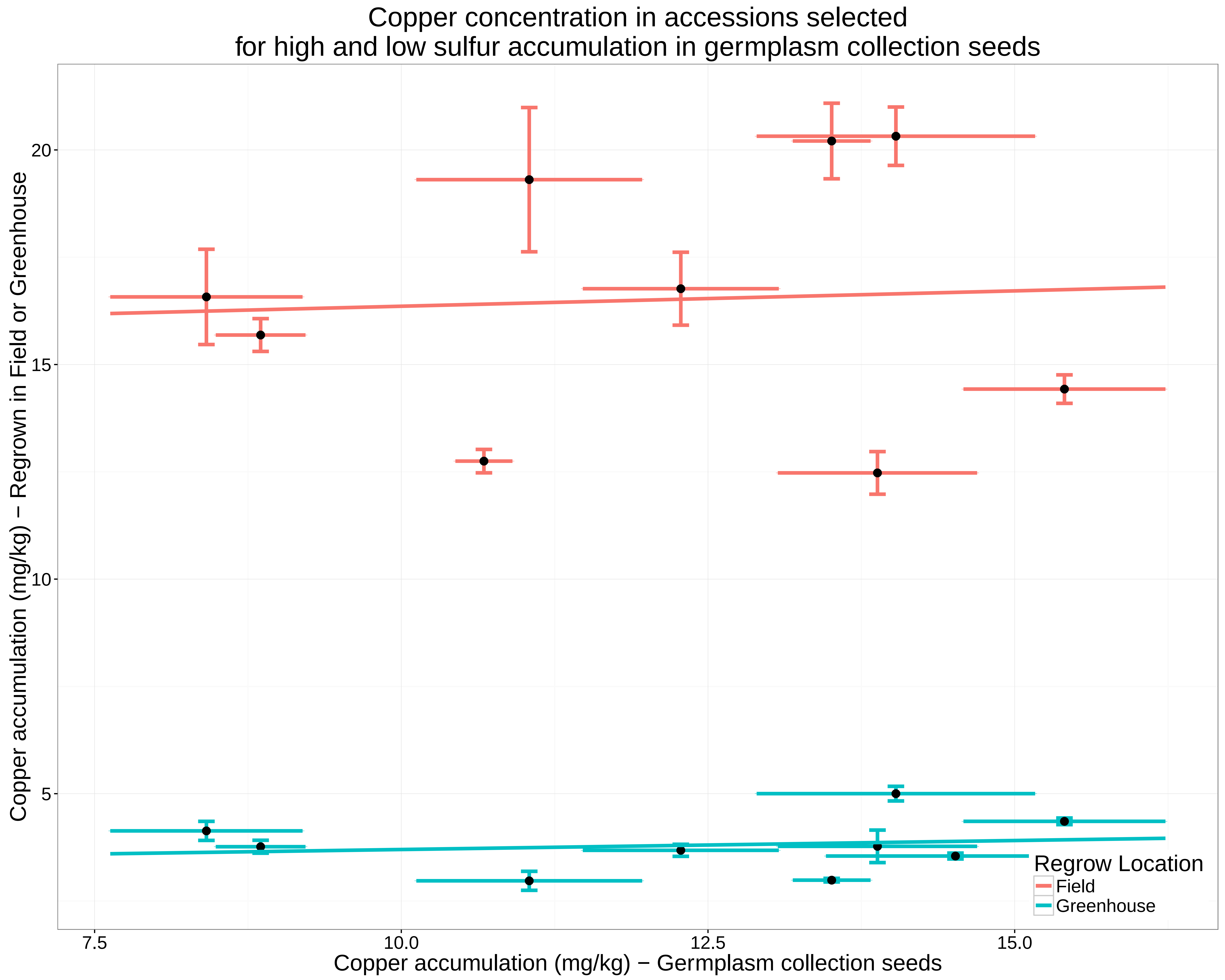

Copper concentration in accessions selected
for high and low sulfur accumulation in germplasm collection seeds
Copper accumulation (mg/kg) − Regrown in Field or Greenhouse
Copper accumulation (mg/kg) − Germplasm collection seeds
20
15
10
5
7.5
10.0
12.5
15.0
Regrow Location
Field
Greenhouse

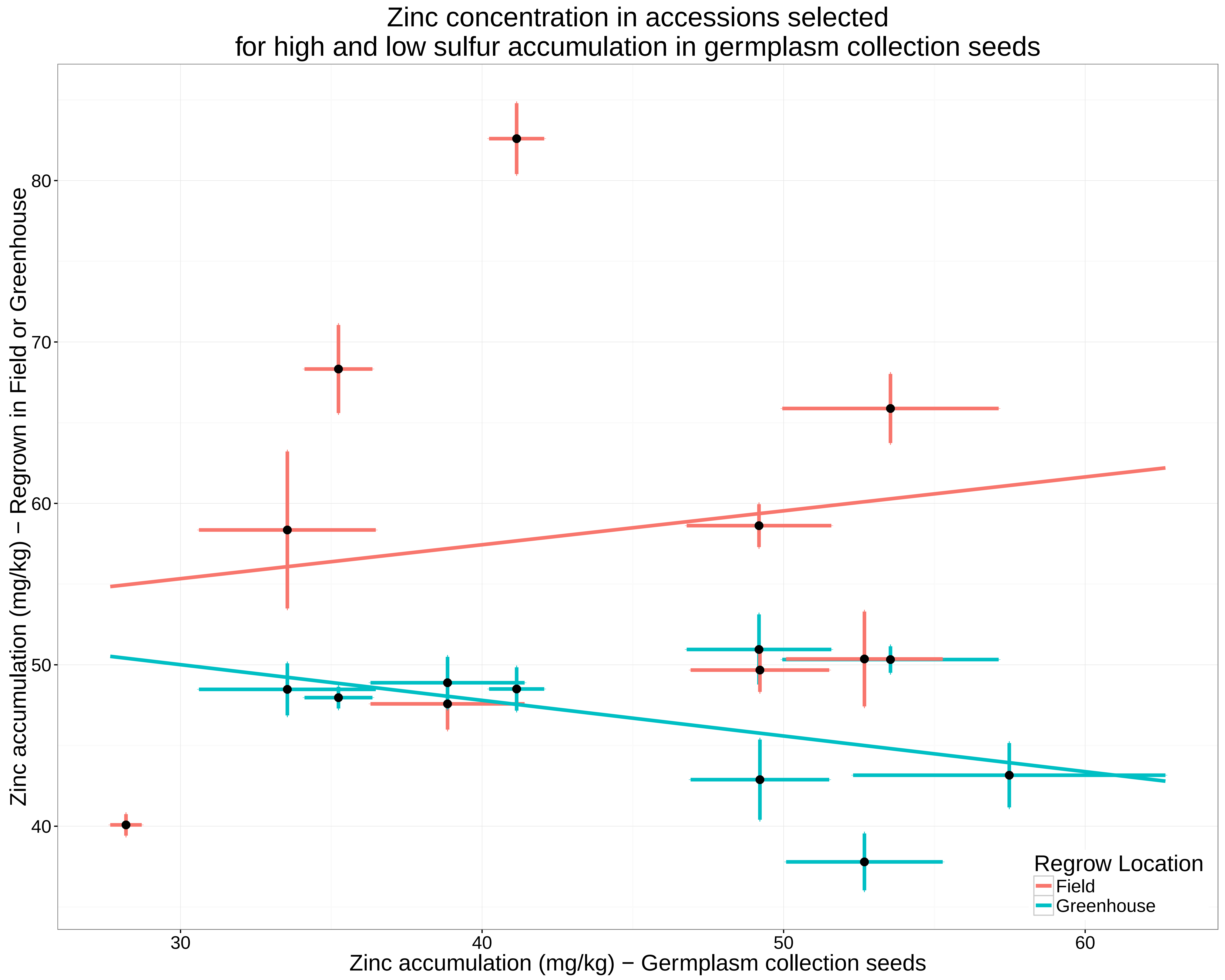

Zinc concentration in accessions selected
for high and low sulfur accumulation in germplasm collection seeds
Zinc accumulation (mg/kg) − Regrown in Field or Greenhouse
Zinc accumulation (mg/kg) − Germplasm collection seeds
80
70
60
50
40
30
40
50
60
Regrow Location
Field
Greenhouse

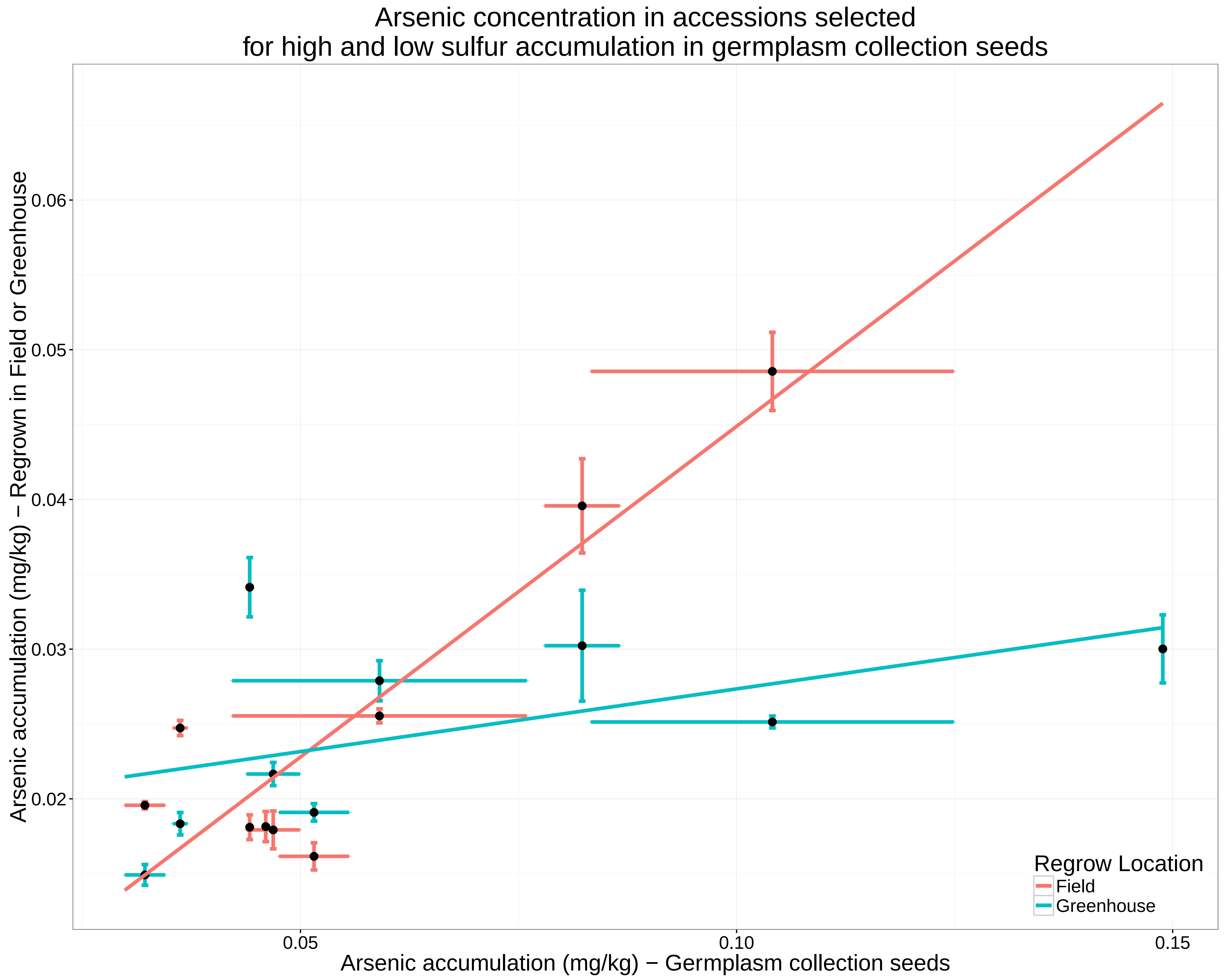

Arsenic concentration in accessions selected
for high and low sulfur accumulation in germplasm collection seeds
Arsenic accumulation (mg/kg) − Regrown in Field or Greenhouse
0.06
0.05
0.04
0.03
0.02
Arsenic accumulation (mg/kg) − Germplasm collection seeds
0.05
0.10
0.15
Regrow Location
Field
Greenhouse

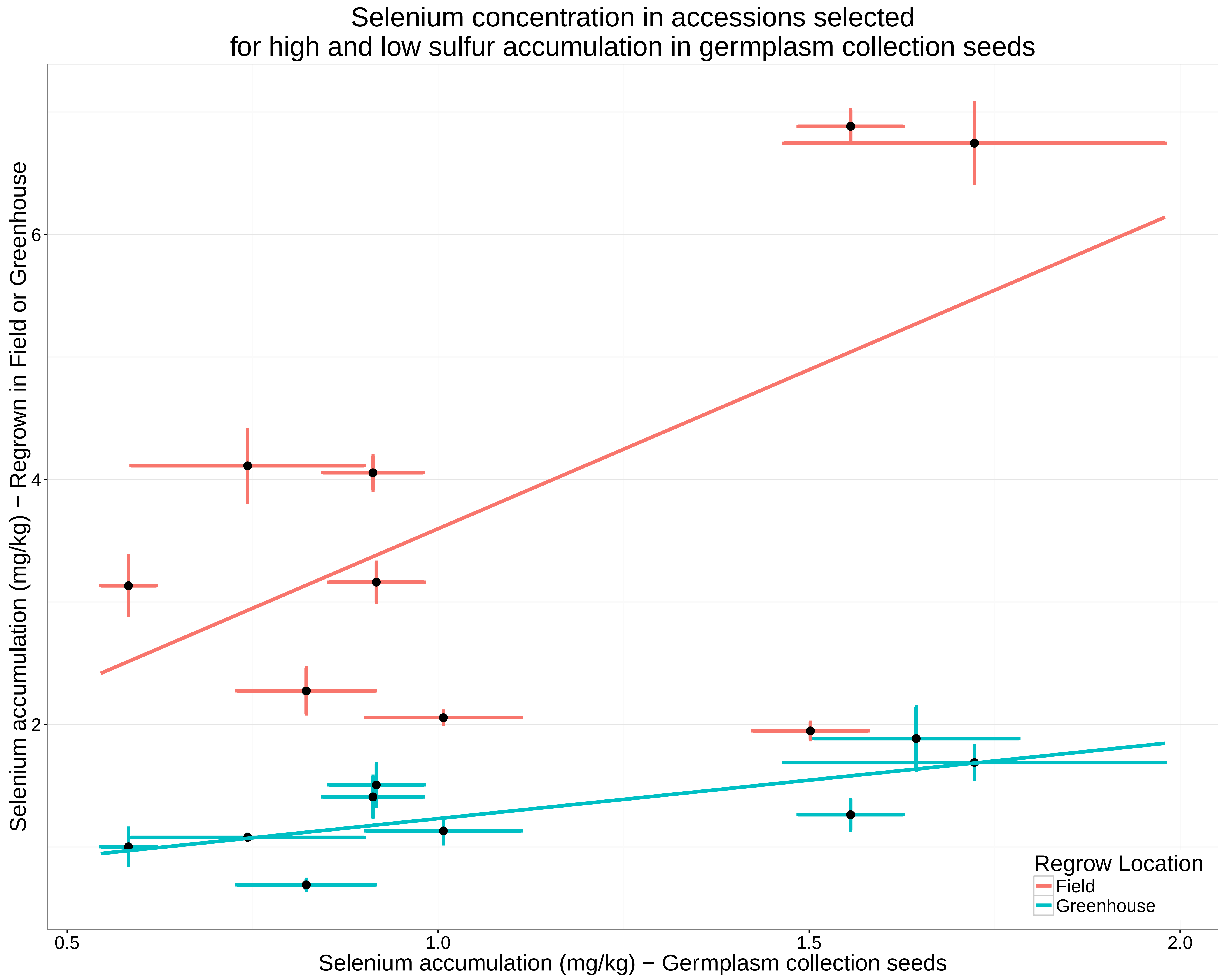
Selenium concentration in accessions selected
for high and low sulfur accumulation in germplasm collection seeds
Selenium accumulation (mg/kg) − Regrown in Field or Greenhouse
Selenium accumulation (mg/kg) − Germplasm collection seeds
0.5
1.0
1.5
2.0
2
4
6
Regrow Location
Field
Greenhouse

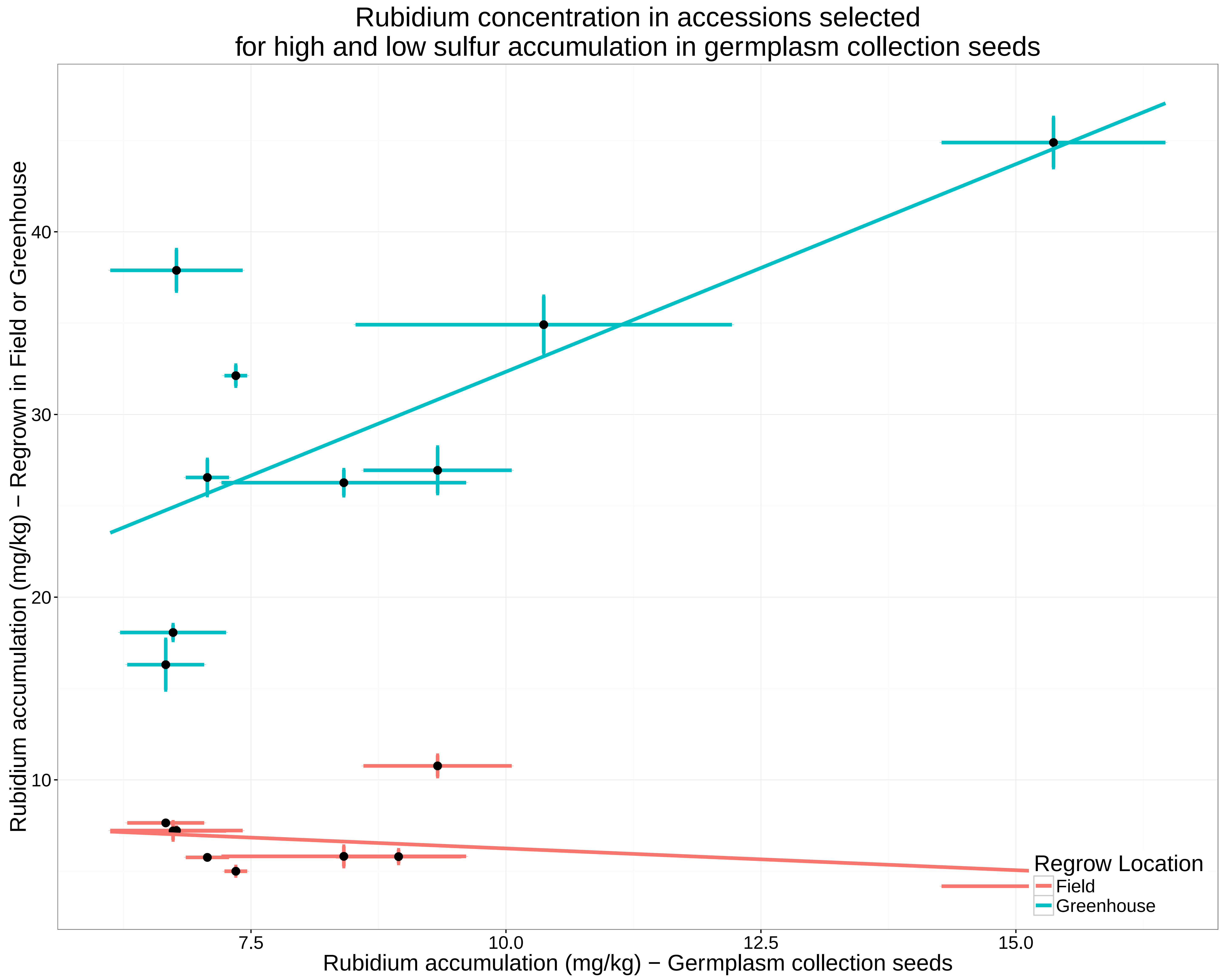

Rubidium concentration in accessions selected
for high and low sulfur accumulation in germplasm collection seeds
Rubidium accumulation (mg/kg) − Regrown in Field or Greenhouse
Rubidium accumulation (mg/kg) − Germplasm collection seeds
40
30
20
10
7.5
10.0
12.5
15.0
Regrow Location
Field
Greenhouse

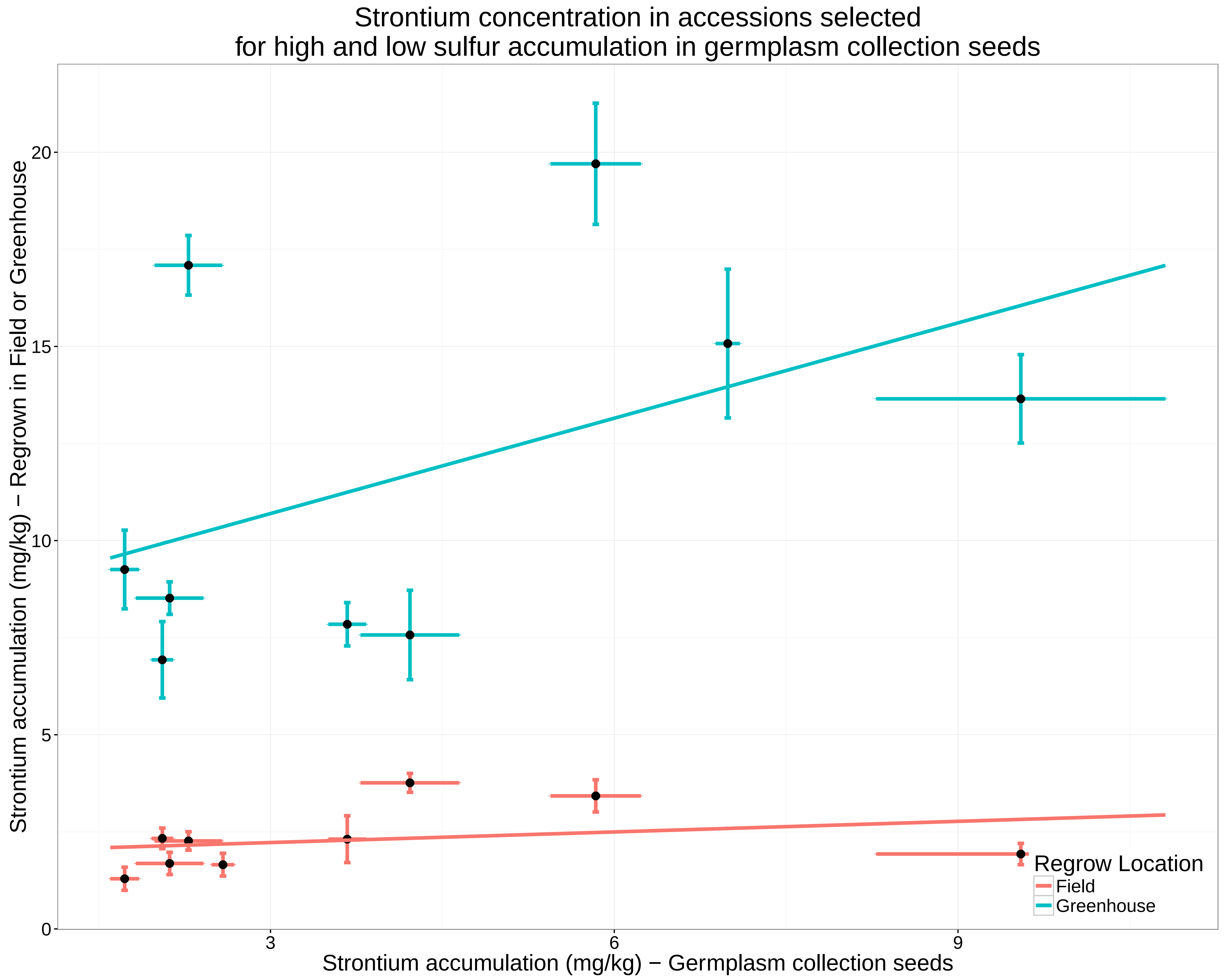

Strontium concentration in accessions selected
for high and low sulfur accumulation in germplasm collection seeds
Strontium accumulation (mg/kg) − Regrown in Field or Greenhouse
Strontium accumulation (mg/kg) − Germplasm collection seeds
0
5
10
15
20
3
6
9
Regrow Location
Field
Greenhouse

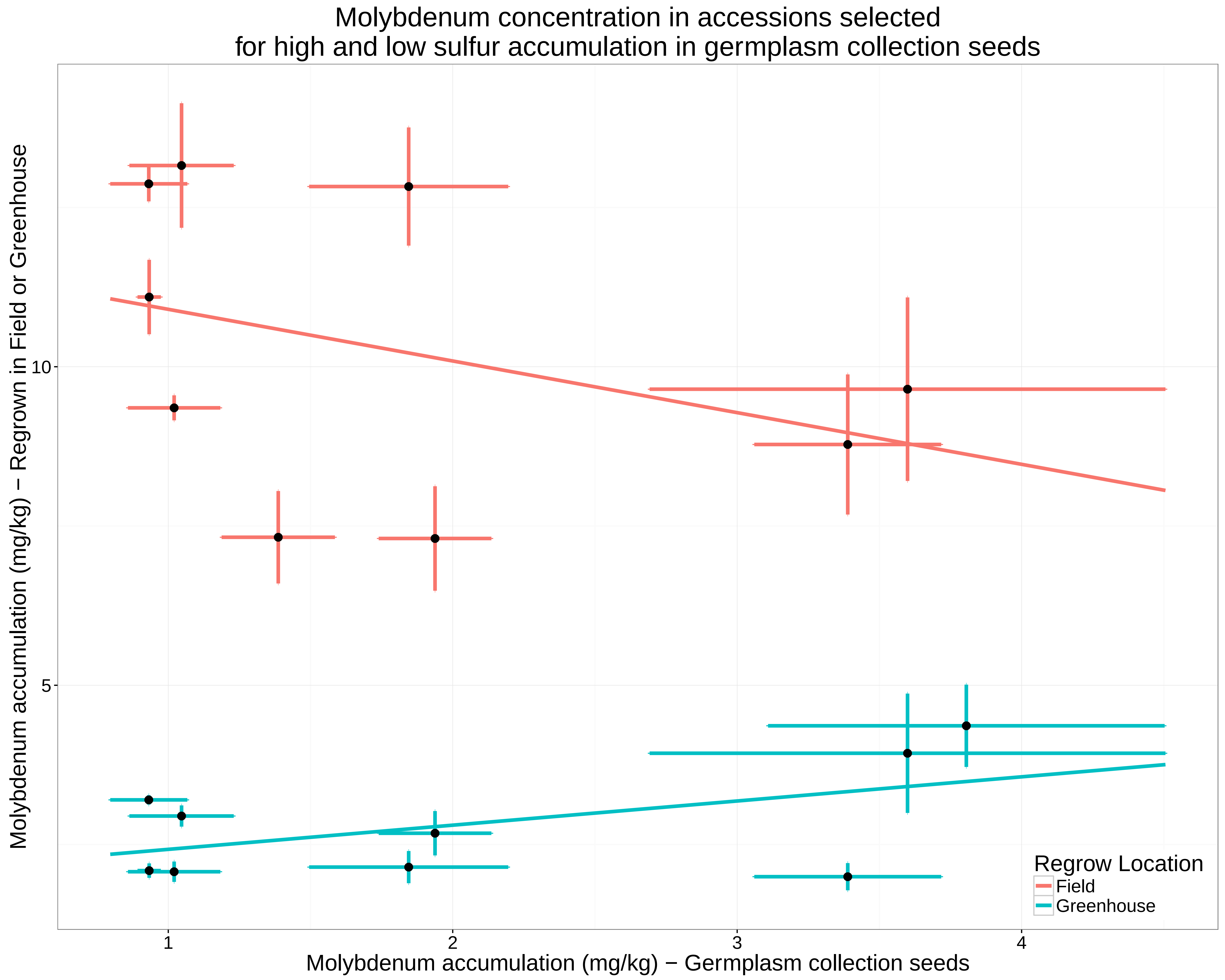

Molybdenum concentration in accessions selected
for high and low sulfur accumulation in germplasm collection seeds
Molybdenum accumulation (mg/kg) − Regrown in Field or Greenhouse
10
5
1
2
3
4
Molybdenum accumulation (mg/kg) − Germplasm collection seeds
Regrow Location
Field
Greenhouse

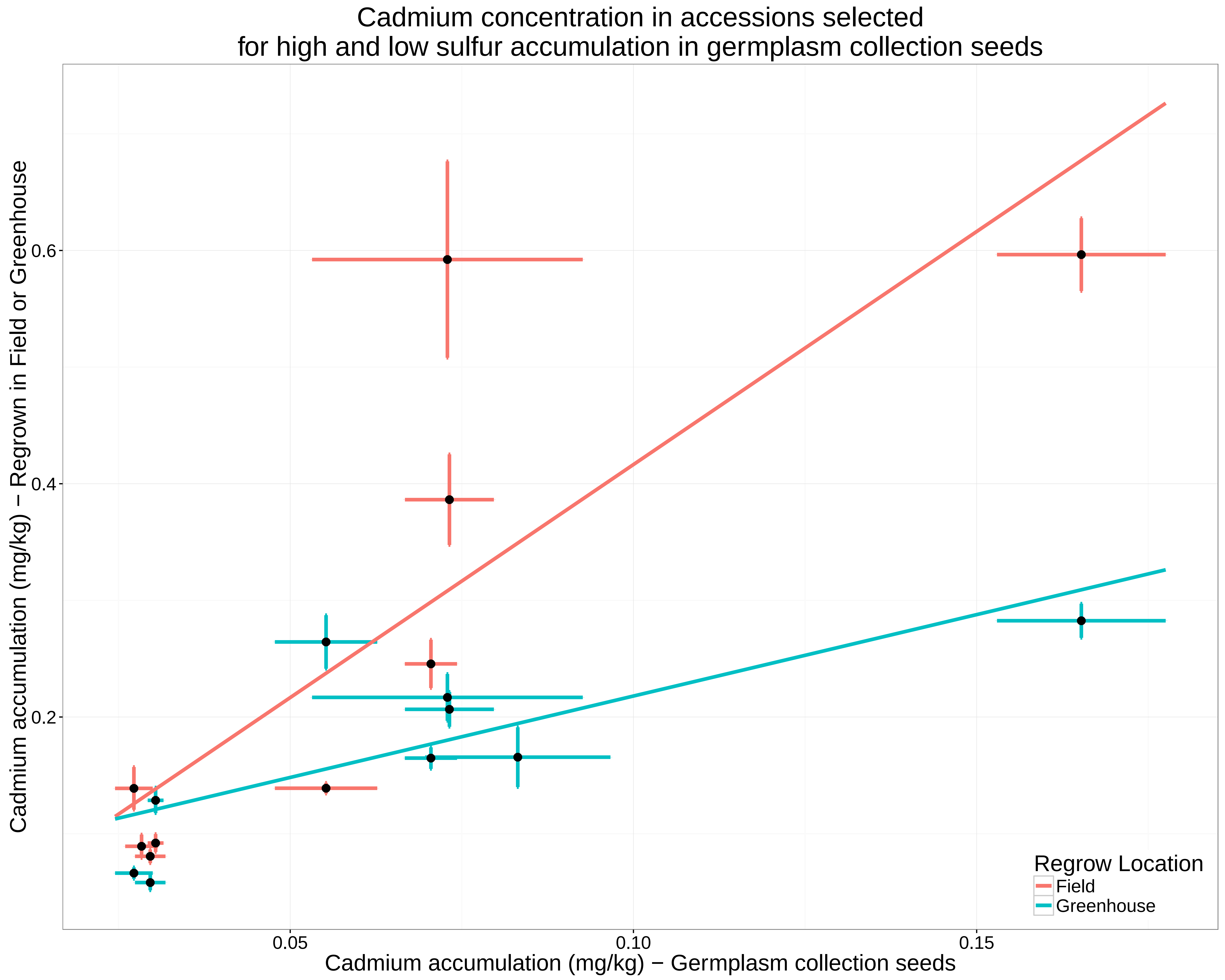

Cadmium concentration in accessions selected
for high and low sulfur accumulation in germplasm collection seeds
Cadmium accumulation (mg/kg) − Regrown in Field or Greenhouse
0.6
0.4
0.2
0.05
0.10
0.15
Cadmium accumulation (mg/kg) − Germplasm collection seeds
Regrow Location
Field
Greenhouse
