# Supplementary material for: Genomewide association study of ionomic traits on diverse soybean populations from germplasm collections
Source: Plant Direct. 2018 Jan 15;2(1):e00033. doi: 10.1002/pld3.33 (PMC6508489; doi:10.1002/pld3.33)
Supplement: Supplementary file 12 [file PLD3-2-e00033-s012.docx]

Response to reviewers

---------------------------------------------------------------------------- Reviewer comments: 
Reviewer #1 : 

In this study, the authors have analyzed the elemental content of soybean seed from germplasm banks, to test whether this resource can be used for gene discovery purposes, despite the many challenges such a resource poses. These challenges include, for example, non-uniform growth conditions and different lines in each experiment. According to the authors, this study is relevant since it was previously demonstrated that the elements are completely stable under different conditions of storage and handling. Although this is not always the case in many other metabolites, demonstrating such a capability is highly valuable. 
The authors performed their study on soybean seeds from 1653 lines of the USDA Soybean and used the information of the grow-outs to define their experiments. In spite of no overlap, between the 14 grow-out they successfully identified several significant associations across at least two separate environments. For this purpose they have used a couple of approaches to define the overlap using a couple of stringency levels, including LD group affiliation. In addition, they have provided a validation to the approach by 1) comparing their finding to characterized genes and likely candidate genes 2) re-growing high and low lines in a common garden environment and testing whether they maintain comparative levels to the collection. 
Comments: 
The authors have chosen an r2>0.2 for defining their LD group with no explanation or citation. I would suggest inserting both.

*We have added and explanation with citations.*

The authors have defined several overlap types, one of which is "different element- different location or same location. However, I did not find a proper explanation as to its biological relevance. I do believe it is important but I suggest a better explanation for this group type at least in the discussion. 
Line 240 it is not clear ;it reads as if the SNPS re-occurred 18-44 times. Please correct.

*We are confused. Those are the three categories of overlap and they are listed individually.*

One of the challenges in these type of studies is to filter the false positives; the authors should address this in the context of their work at least in the discussion.

*We have added to the results and discussion to address this.*


Reviewer #2 : 

The authors set out to test the efficacy of using seed samples from germplasm banks for ionomics profiling and genome-wide association. Using seeds from the USDA Soybean Germplasm Collection, they analyze the elemental profile of 1653 lines. In most instances, they found that the location and year of the grow-outs had significant effects on elemental profiles, such that combining the data for one large GWAS (which would provide the most power) would not be feasible. Therefore, the authors performed GWAS on data from each grow-out separately, except for those from Costa Rica (which were combined). They generated (I believe) 14 x 21 = 280 GWAS datasets (14 locations, 21 phenotypes: 20 elements + seed weight). Then they looked for overlap between the results, which would serve as independent evidence that the association is real, and not a false positive. 

1. Are the experiments and analysis performed to an acceptable technical standard? 

I have a few questions about the methodology. 
a) the box-cox transformation is appropriate; however, I question whether transforming some of the datasets but not others is appropriate. It would make more sense to treat all phenotypic data the same way, especially since results will be compared/combined later. 

*The elements are somewhat independent traits. They have different distributions. The point of transformation is to put them into a close approximation of the normal distribution that the GWAS approach assumes. Treating all of the data the same way would increase the artifacts as some traits would not have normal distributions.*

b) the GWAS approach chosen is not a standard approach. Since this is a proof-of-concept paper focused on testing the phenotyping of seeds from seedbanks, a bit more attention to the GWAS methodology (and better descriptions) are needed. If I understand correctly, they start with a random-effects model only (just the population correction), and keep adding "significant" SNPs as covariates until some criterion tells them that adding more does not add explanatory power. They need to be more explicit about the source of these "significant" SNPs. Are they running regular single-marker GWAS with a mixed model correction? Not clear from the Methods section, either. Including straightforward results from a standard EMMAX would be recommended, or else the authors could include a justification for the choice of approach in their response. 

*We have included extra description in the results and methods. However, we think it erroneous to state that MLMM is not a standard approach. The paper describing MLMM has been cited between 178 (Web of Science) and 270 (Google Scholar) times and is implemented as an option in the web platform GWAPP. We think that an approach that includes other SNPs when testing for association is much more appropriate for our study. As shown by Figure 4 this approach does a much better job of accounting for spurious correlation of SNPs across the genome with phenotypes.*

c) The SNP cofactors in Table 3 - what was the cut-off criteria?

*We have clarified in the methods and added to the legend to direct readers to the explanation.*

d) SNPs in the same LD block (same significant region, ie one GWAS peak) were defined based on r2 > 0.2. A plot of LD decay should be provided to justify the criteria. The size of each region of interest should be included in the candidate genes table, and in a new table summarizing novel regions of interest.

*No*

e) I agree that finding overlap between significant regions identified for the same element in different locations is a great indicator that the association is real. However, I can't see the biological relevance of an overlap between different elements in the same location, or different elements in different locations. In this case where a large number of independent tests are being performed, a few overlaps at random are bound to occur. Without a biological hypothesis for why this overlap might be meaningful (for instance, related elements such as Cd and Zn and others), these overlaps don't serve as evidence that the association is real. 

*We have added text to explain why the overlap between elements is relevant.*

f) Candidate genes matching significant SNPs, in Table 5. The matches need to be supported by a biological hypothesis. There are many candidates and many significant regions, some of these overlaps are bound to occur by random chance. In some cases, the connection is obvious, but in others the connection is lacking. These should be justified, or removed. For instance: I don't see why an Nramp (or ALS3) would be good candidates in Se accumulation. Similarly, the malate transporter ALMT, how would it play a role in Rb, Mo, Co or K content?

*We have clarified our conclusions and rationale to emphasize the overlap of SNPs is the main proof and the candidate genes is further evidence.*

g) Line 402 page 19, ICP methods, 2 or 8 seeds were measures from each line. Two measurements are not enough for outlier detection and removal, please clarify. 
*Done*

2. Are the experiments and analysis described in adequate detail in standard English? 

Although the text is written in perfect English, its structure is inadequate and makes the manuscript really hard to follow. I had to go back and forth a few times until I understood how the data was partitioned and how GWAS was run. The Results section has text that belongs in the introduction (for instance first paragraph, lines 66-79), and text that belongs in the Methods. This lack of structure requires the reader to flip back and forth to understand how things were done. The text is written in dissertation style, and is a bit too wordy. I would suggest that the text be restructured and shortened to improve clarity. 

*We have altered the text*

3. Do the results support the conclusion? 

In the current version of the manuscript, I don't believe they do. In addition to the questions listed above-- 
Since this is a proof-of-concept, the conclusion that the method is working needs to be supported by either of these two lines of evidence: 
-method is capable of detecting significant association previously reported by other methods (QTL mapping, candidate gene) 
-method is capable of detecting novel association; gene is then functionally characterized to prove association was real. 
The second choice is labor-intensive and it's not what this paper is about, understandably. Therefore, the authors need to spend a lot more time describing the associations discovered. Are there novel candidate genes that were not previously characterized, but make sense from a biology point of view, and are worthy of further investigation? List those clearly. And in the case of known genes, for which HMA3 is the strongest candidate: is HMA3 within the same LD block with significant SNPs? Clearly indicate that in a figure. Is there SNP variation within the HMA3 coding region or promoter that co-segregates with the phenotypic variance? From the SNP data or from sanger sequencing of lines with contrasting [Cd]? A little more evidence would go a long way in making the case that the method is working indeed. 

*We have rewritten the conclusions to clarify the goals of the study and how we believe our results support them. The reviewer makes no mention of the fact that we confirmed extreme phenotypes as one method of confirmation. Fundamentally- we believe that finding the same SNPs with completely different populations and environments shows that this method can (and did) work.*

Additional comments: 

- just a suggestion, but in cases where there was no significant difference between years in a same location (table 2 showing ANOVA results), why not combine for greater power? Especially datasets from Urbana, because there are many. Results from different years could be combined for B, Na and As, for instance. 

- also a suggestion, Fig 1 and Suppl Fig 2 show the same data for different elements, and the plots are unnecessarily large; these could be combined in single figure with multiple panels showing a few more examples. 

- figure 2, would be ideal to show the same distribution before and after correction. 

- Line 222 page 10: "Table 5 contains a list of SNPs found on or near a candidate..." how was "near" defined? Significant SNP needs to be shown to be in same LD block as candidate gene.
